# Stress and high fat diet reconfigure the active translatome of CeA-NPY neurons

**DOI:** 10.1016/j.molmet.2025.102176

**Published:** 2025-06-04

**Authors:** Chi Kin Ip, Lei Zhang, Ramon Tasan, Herbert Herzog

**Affiliations:** 1 Brain Tumour Group, Children's Cancer Institute, Australia; 2 St Vincent's Centre for Applied Medical Research, Darlinghurst, Sydney, Australia; 3 Faculty of Medicine, University of New South Wales, Sydney, NSW 2052, Australia; 4 Department of Pharmacology, Medical University Innsbruck, 6020 Innsbruck, Austria

**Keywords:** NPY, Stress, Central Amygdala, TRAPseq, High fat diet, Obesity, Hedonic eating

## Abstract

**Objective:**

The interplay between calorie-dense food and chronic stress significantly accelerates obesity development, with neural circuits expressing Neuropeptide Y (NPY) in the central amygdala (CeA) emerging as the key mediator of this process. While these circuits are known to enhance hedonic feeding behavior and promote weight gain, the precise molecular mechanisms regulating NPY neuron activity at the translational level under the combined influence of high fat diet and stress conditions have remained poorly understood.

**Methods:**

We employed translational ribosome affinity purification coupled with Next-Generation Sequencing (TRAPseq), allowing us to specifically identify RNA transcripts actively undergoing protein translation in NPY neurons under high fat diet (HFD) or high fat diet combined with stress conditions (HFDS).

**Results:**

Our molecular profiling demonstrates that NPY neurons specifically co-express with genes marking the orexigenic (appetite-stimulating) population, while showing minimal overlap with anorexigenic (appetite-suppressing) markers. Gene ontology analysis identified distinct clusters involved in fatty acid metabolic processes, stress response pathways, and the production of feeding-related neuropeptides specifically under HFDS. Immunohistochemical investigations revealed in addition to local CeA (CeA^m^) NPY connection pathways, long-range projections, to the lateral habenula (LHb), the periaqueductal gray (PAG) and parvicellular reticular formation (PCRt). These projections suggest a specific role for CeA NPY neurons in coordinating feeding and emotional responses.

**Conclusion:**

Collectively, our findings identify specific lipid-sensing mechanisms and synaptic modulating pathways as principal targets of stress within the CeA-NPY circuit, revealing novel molecular mechanisms through which NPY neurons integrate and process both dietary and stress signals.

## Highlights

• Central amygdala NPY neurons are of orexigenic nature.
• High fat diet downregulates NPY expression in the CeA.
• Chronic stress in combination with HFD accelerates NPY expression and action.
• CeA NPY neurons coordinate feeding and emotional responses.
• HFD combined with stress modulates lipid-sensing mechanisms and synaptic pathways in CeA-NPY neurons.

## 1 Introduction

Energy homeostasis is intricately regulated by the interaction between dietary choices and stress levels. While acute stress serves adaptive functions, chronic stress can significantly disrupt energy homeostatic control, particularly when combined with calorie-dense food consumption. This disruption manifests in complex behavioral changes, where chronic stress combined with access to highly palatable foods leads to increased “comfort eating,” ultimately accelerating obesity development through both increased food intake and reduced energy expenditure [1]. The CeA has emerged as a critical integration hub for both stress responses and feeding behavior regulation. Within the CeA, neurons expressing NPY play a pivotal role in orchestrating responses to both dietary challenges and stress exposure. NPY, known as one of the most potent orexigenic and anxiolytic neuropeptides, demonstrates remarkable plasticity in response to both dietary composition and stress levels [2,3]. However, the molecular mechanisms by which NPY neurons integrate these signals, particularly at the translational level, remain poorly understood.

Recent evidence suggests that NPY neurons in the CeA exhibit complex patterns of connectivity and signaling that extend beyond local circuits [[4], [5], [6]]. These neurons project to multiple brain regions, including the LHb and lateral hypothalamus (LH), suggesting a broader role in coordinating feeding behavior and stress responses. The combination of chronic stress and HFD creates a particularly potent driver of obesity development, yet how this combination influences gene translation and neuropeptide signaling specifically within CeA NPY neurons remains unclear.

Understanding the molecular identity and translational regulation of CeA NPY neurons is crucial for being able to find potential intervention points that may be utilised as anti-obesity targets. Furthermore, the mechanisms by which these neurons process and respond to lipid signals while simultaneously managing stress responses could provide crucial insights into stress-induced feeding behaviors. To address these fundamental questions, we employed Translating Ribosome Affinity Purification combined with Next-Generation Sequencing (TRAP-seq) to examine the actively translating transcriptome of CeA NPY neurons. Since our previous study [6] as well as confirmed here (Supplementary Figure 1) does not show any significant influence of stress on body weight, fat depots or CeA NPY levels or activation when fed a chow diet we specifically focused our analysis on the comparison between HFD condition and how chronic stress on top of HFD alters this.

## 2 Results

TRAP-seq, a method that allows the identification of all actively translated mRNA species in NPY neurons, rather than just measuring total mRNA levels as in conventional bulk RNA-sequencing [[7], [8], [9]], was employed to investigate changes in translation profiles of CeA NPY neurons under HFD and HFDS conditions.

**Figure 1:**
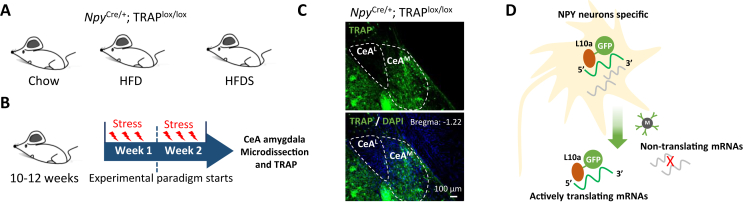
Active translatome approach in CeA Npy neurons. (A,B) Schematic illustration of the experimental model and protocol to perform TRAP on NPY neurons in the CeA^M^ under Chow, HFD and HFDS conditions. For HFDS, mice were stressed three times each week for two weeks followed by brain collection, microdissection and TRAP-sequencing. (C) Fluorescence micrograph of GFP-positive CeA^M^ neurons in *Npy*^*Cre/+*^;TRAP^lox/lox^ mice. (D) Schematic illustration of the TRAP method.

For this we crossed our *Npy*^*cre/+*^ mice with TRAP^lox/lox^ mice [10], hereafter referred to as *Npy*^*cre/+*^;TRAP^lox/lox^ mice, followed by different treatments (Figure 1A and B). Fluorescence microscopy confirmed the successful expression of the L10-GFP fusion protein only in amygdala areas that are known to express NPY, for instance, the medial nucleus of the CeA (CeA^M^), but not in the lateral nucleus of the CeA^L^ (Figure 1C). Different cohorts of 10–12 week old *Npy*^*cre/+*^;TRAP^lox/lox^ male mice were exposed to either a chow, HFD or a HFD combined with repeated Stress (HFDS) paradigm as outlined in the schematic in Figure 1A using previously described protocols [11,12]. Following 2 weeks of treatment the body weight of mice fed a HFD increased significantly, with the mice on HFDS further exceeding this weight gain [6,11]. At the 2-week timepoint mice were culled and brain tissue containing the CeA was isolated and the actively translated mRNA pool bound by the EGFP-tagged ribosome subunit L10a was purified, followed by Next-Generation Sequencing and bioinformatic analysis (Figure 1D).

**Figure 2:**
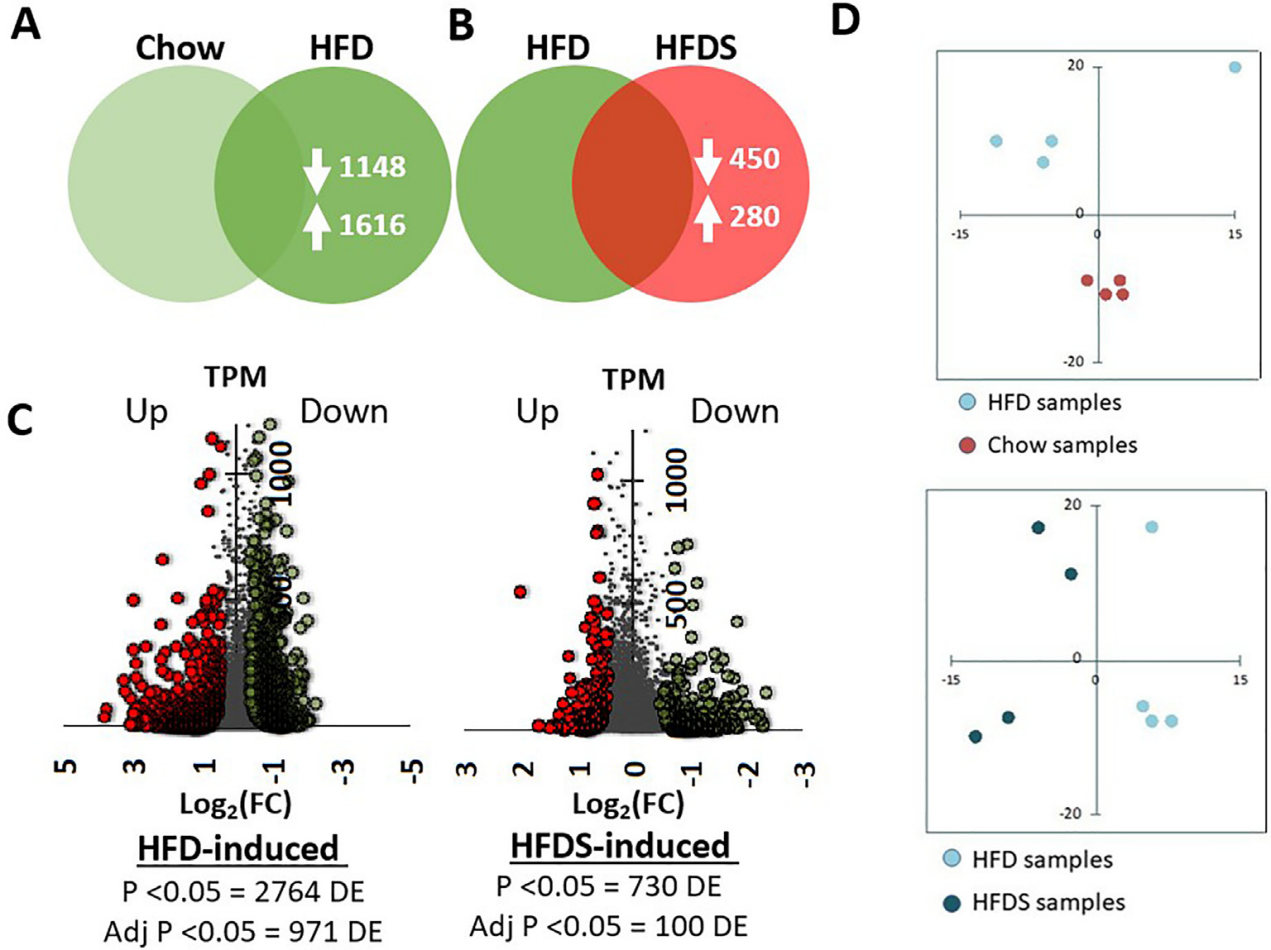
TRAP-seq characterization of CeA-Npy neurons under HFD and HFDS. (A–C) Venn diagram and Volcano plot showing the number of up- and downregulated genes, as well as the magnitude of fold changes in the IP RNA samples between HFD compared to chow samples and HFDS compared to HFD samples. p < 0.05. TPM, transcripts per million. (D) Principal component analysis (PCA) plot in the chow, HFD and HFDS group.

Sequencing data were analysed with a focus on examining changes in actively translating genes between chow versus HFD, and HFD versus HFDS conditions. Our analysis focused on comparing these patterns between chow versus HFD, and HFD versus HFDS, to determine which genes showed the most significant changes in functional activity under these different diet/stress conditions. Comparison between the chow and HFD groups revealed significant changes in gene translation, with 1,148 genes showing downregulation and 1,616 genes showing upregulation in the HFD group (Figure 2A; Supplementary Table 1). When comparing HFD and HFDS conditions, we identified that the translation of 450 genes was downregulated, and that of 280 genes were upregulated in the HFDS group (Figure 2B; Supplementary Table 1).

Volcano plot analysis revealed that HFD feeding induced extensive alterations in gene expression/translation [Log2(−2.9) to Log2(+4)]. Furthermore, when stress was combined with this HFD feeding paradigm, again there was another significant magnitude of changes in the translational gene program in the CeA [Log2(−2.8) to Log2(+2)]. This changes indicate that CeA-NPY neurons exhibit dynamic translational plasticity in response to conditions such as HFD and HFDS (Figure 2C). Principal component analysis (PCA) revealed distinct clustering patterns across dietary conditions. The upper plot shows clear separation between HFD samples (blue dots) and chow samples (red dots), with HFD samples clustering in the upper quadrants and chow samples grouping in the lower quadrants. The lower plot compares HFD samples (light blue dots) with HFDS samples (dark blue dots), showing spatial separation between these two conditions. This distinct clustering in the PCA plots suggests that both the high fat diet and the addition of stress induced significant changes in the functional gene regulatory networks, resulting in distinct translational profiles for each dietary condition (Figure 2D).

**Figure 3:**
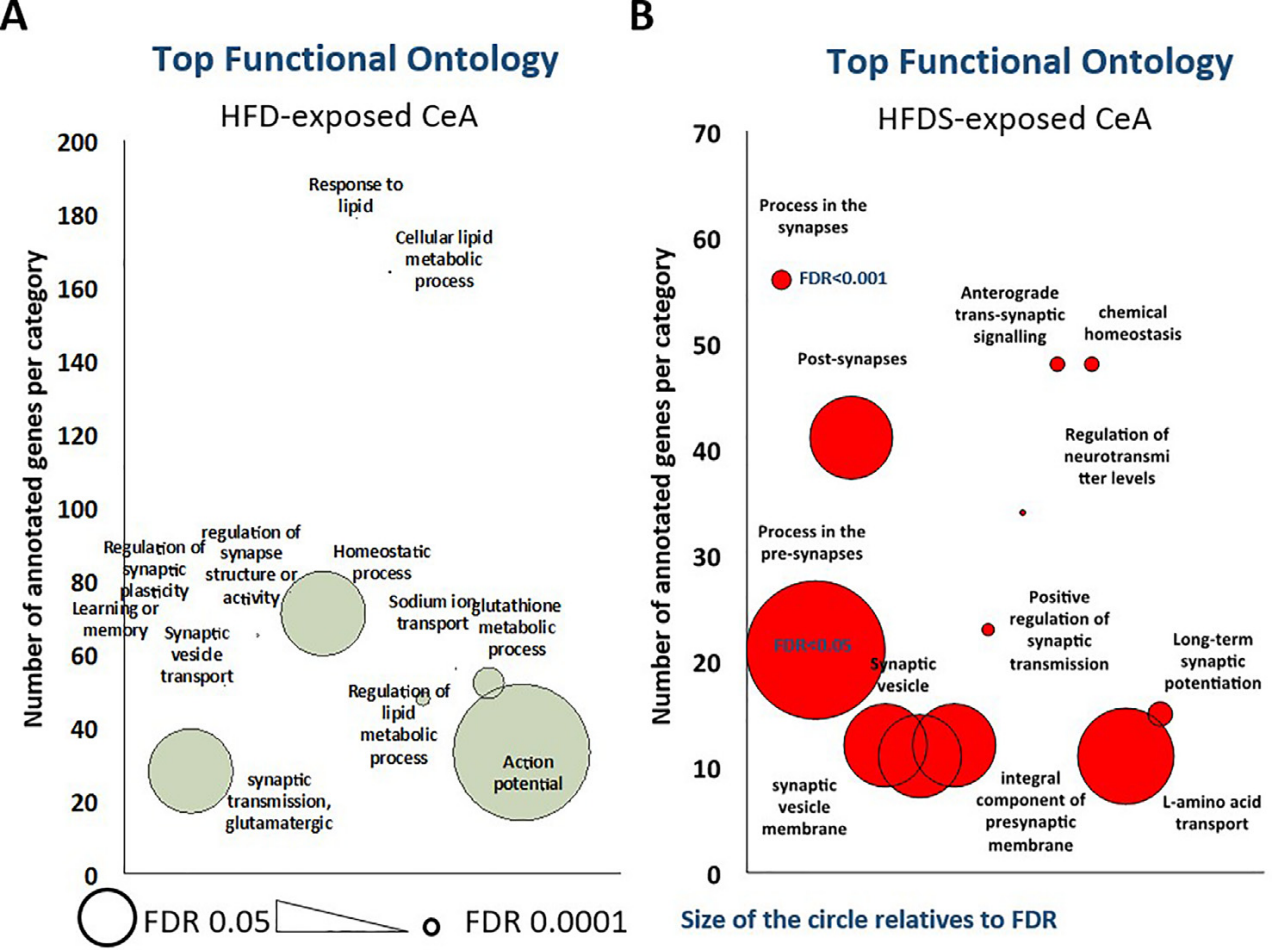
Functional ontology analysis of gene changes altered by HFD and HFDS. (A) Top significant functional ontology categories on genes altered by HFD. (B) Top significant functional ontology categories on genes altered by HFDS. The smaller the circle the lower the false discovery rate (FDR).

Combined functional ontology analysis of the differentially translated (DET) genes triggered by HFD (Figure 3A) showed enrichment of key functions in homeostatic processes (FDR<0.001; 48 genes), cellular lipid metabolism (FDR<10^−9^; 179 genes), response to lipid (FDR<0.0001; 164 genes), regulation of synaptic plasticity (FDR<10^−10^; 64 genes) and synaptic vesicle transport (FDR<10^−5^; 51 genes). Together this suggests that active gene programs that are crucial for metabolic shift and synapse plasticity remodeling are being altered in the CeA-NPY neurons to adapt to long term HFD feeding (Supplementary Table 2). Interestingly, under HFDS, the DET genes showed a completely different result (Figure 3A), with enrichment of genes involved in the regulation of neurotransmitter levels (FDR <10^−3^; 34 genes), anterograde transsynaptic signaling (FDR<0.001; 48 genes), physiological process in synapses such as formation and recycling (FDR<0.001; 56 genes) and synaptic vesicles transport (FDR<0.05; 12 genes), suggesting that specific neuronal properties and neurotransmitter outputs are triggered in the CeA NPY neurons under HFDS condition (Supplementary Table 3).

**Figure 4:**
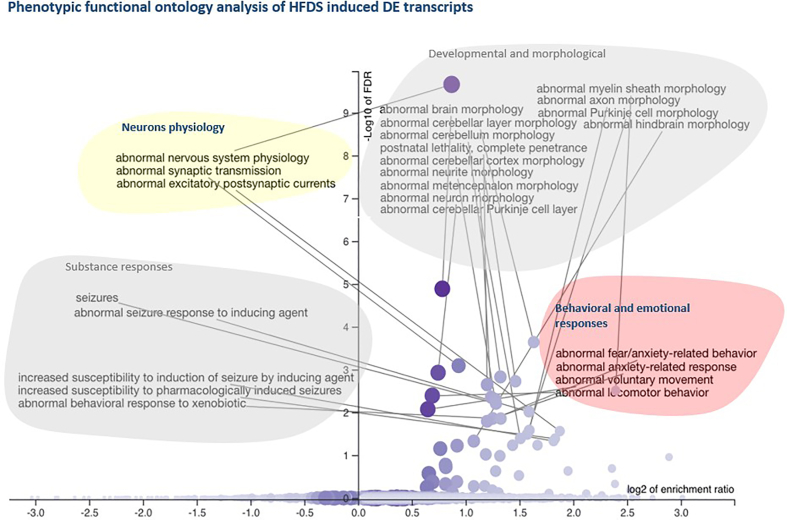
Phenotypic functional ontology analysis of genes altered under HFDS condition. The right panel depicts the categories with significant enrichment indicated by FDR less than 0.05.

In addition, using an alternative gene set enrichment analysis approach exploring the gene and its associated loss of function phenotype showed that the identified DET genes are predominantly involved in four key phenotypic categories, including neuron physiology, behavioral and emotional responses, substance responses and developmental and morphological related phenotypes (Figure 4; Supplementary Table 4). This suggests that HFDS-triggered active translatome changes alter signaling pathways which are important for reward behaviours that can affect various emotional and stress-induced responses.

**Figure 5:**
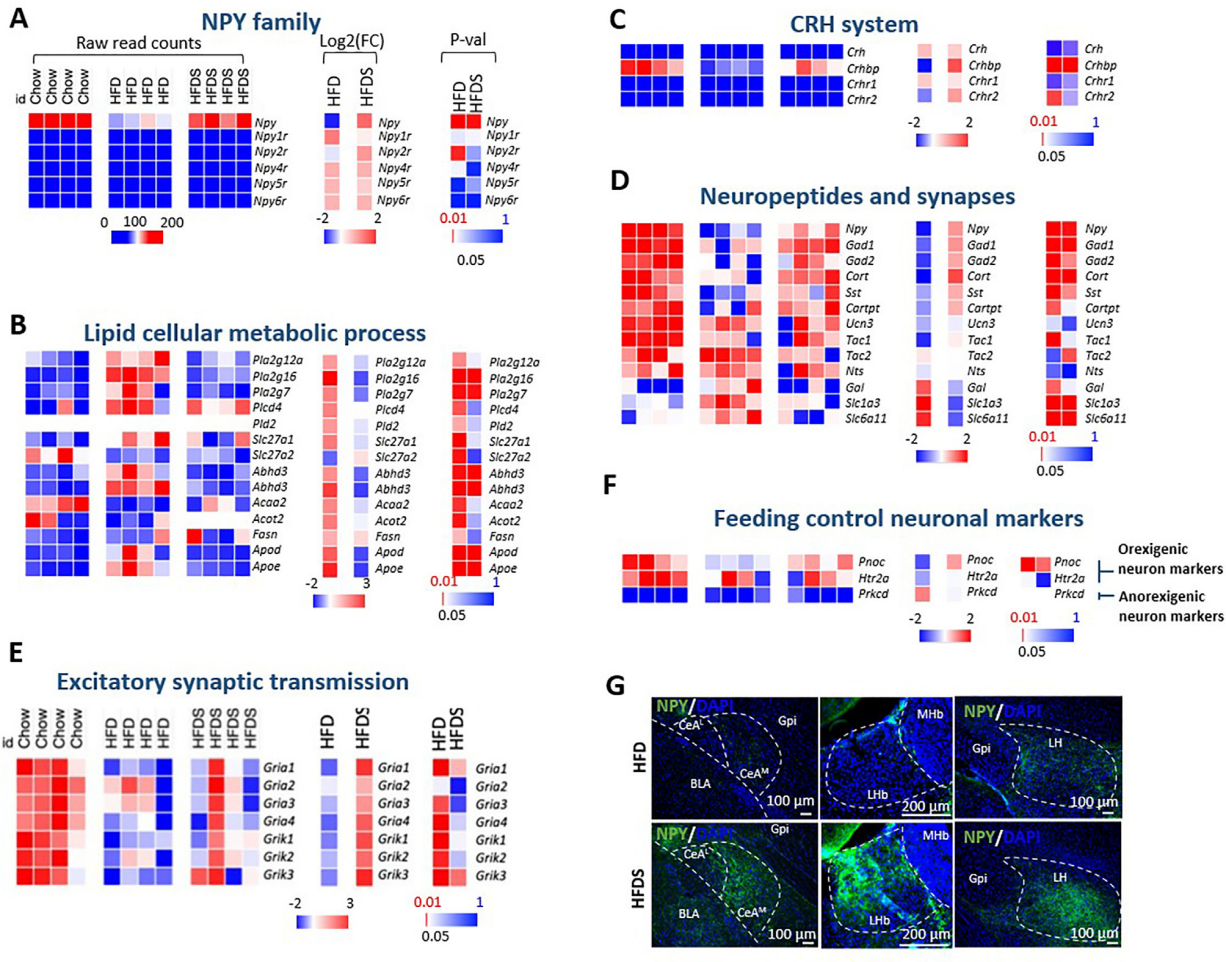
Key actively translating gene changes under HFD and HFDS. Color coded counts for each biological replicate of the Chow, HFD and HFDS group under different categories: (A) NPY family; (B) Lipid cellular metabolic process; (C) Corticoid releasing hormone (CRH) system; (D) Neuropeptides and synapses; (E) Excitatory synaptic transmission and (F) Feeding control neuronal markers. Heat maps show the relative raw read counts of genes in each biological sample, fold changes and p-value. (G) Fluorescence micrograph of NPY expression in the CeA^M^, LHb and LH under HFD and HFDS condition. LHb, lateral habenula; MHb medial habenula; Gpi, globus pallidus; LH, lateral hypothalamus; CeA^M^, medial nuclei of central amygdala; CeA^L^, lateral nuclei of central amygdala; BLA, basolateral amygdala.

Further analysis of the gene sets that were significantly affected by HFD or HFDS identified through GO analysis, we found significant expression changes of *Npy* and its receptors, genes controlling lipid cellular metabolic processes, neuropeptides, synapses, the CRH system and feeding control markers (Figure 5A–E). Consistent with the known involvement of the NPY system in both maintaining energy homeostasis and controlling stress responses [13], we found a significant downregulation of actively translated *Npy* level in the CeA under HFD condition (Figure 5A), suggesting that there is a diet-induced protective mechanism to reduce feeding in response to energy overload, similar to what is observed in Arc NPY neurons under these conditions [11,14]. Importantly, stress is overriding such homeostatic induced protective mechanism and significantly upregulates *Npy* expression (Figure 5A), thereby promoting NPY's orexigenic actions. Interestingly, out of all NPY receptors, only *Npy2r* translation was upregulated in response to HFDS feeding. Since *Npy2r* typically functions as an auto-receptor that negatively regulates *Npy* expression, its desensitization under HFDS conditions suggests it could be one of the main contributors to the observed excessive *Npy* upregulation in the CeA. (Figure 5A).

In response to a HFD diet, we observed significant upregulation in the translation of genes involved in various aspects of lipid metabolism (Figure 5B). A major group of affected genes encoded lipases and phospholipases (*Pla2g12a, Pla2g16, Pla2g7, Plcd4, Pld2*), which catalyze phospholipid hydrolysis and are crucial for membrane maintenance and lipid signaling. The Pla2 family members break down phospholipids to release fatty acids from cell membranes, while *Plcd4* generates important second messengers (IP3 and DAG), and *Pld2* gene encode protein produces phosphatidic acid for membrane trafficking. Another significant group upregulated included fatty acid transport and processing proteins, such as the long-chain fatty acid transporter *Slc27a1*, metabolic enzymes *Abhd3,4* (involved in lipid breakdown), *Acaa2* (essential for fatty acid β-oxidation), *Acot2* (regulating fatty acid oxidation), and *Fasn* (synthesizing long-chain fatty acids). Additionally, lipid transport proteins *ApoD* and *ApoE*, which mediate lipid trafficking and cholesterol homeostasis particularly in the brain, showed upregulated translation. These coordinated changes suggest a comprehensive remodelling of lipid metabolism pathways in response to high fat diet feeding.

Interestingly, under HFDS conditions where further increased feeding was observed [6,11], the translation of genes important for lipid metabolism (eg *Pla2g16, Pla2g7, Abhd3,4, Apod and Apoe*) was significantly downregulated (Figure 5B), suggesting that the addition of stress on top of a HFD may directly interfere with lipid metabolism in CeA NPY neurons, potentially creating a metabolic environment favouring energy storage over utilization. Regarding stress-related pathways, while the translation level of major stress mediators such as corticotropin-releasing hormone (*Crh*) and its receptors (*Crhr1, Crhr2*) remained unchanged, the *Crh* regulator, corticotropin-releasing hormone binding protein (*Crhbp*), showed a dynamic translation pattern (Figure 5C): being significantly suppressed under HFD conditions, but significantly increased under HFDS conditions (Figure 5C). This suggests that the regulation of the CRH system under HFD and HFDS conditions is mostly dependent on *Crhbp* levels.

Importantly, in response to HFD feeding, we also identified significant translational downregulation of several key neuropeptides and enzymes that play crucial roles in various physiological processes (Figure 5D). These include cortistatin (*Cort*), a neuropeptide involved in slow-wave sleep regulation and metabolic control; somatostatin (*Sst*), which regulates numerous physiological processes including neurotransmission and cell proliferation; cocaine- and amphetamine-regulated transcript peptide (*Cartpt*), known for its role in feeding behaviour and energy homeostasis; and glutamate decarboxylase 1 and 2 (*Gad1* and *Gad2*) (Figure 5D). Surprisingly, when chronic stress was combined with HFD (HFDS condition), many genes showed significant increases in translation, with *Gad1*, and *Gad2* showing the most pronounced increases. This finding is particularly significant because *Gad1* and *Gad2* encode glutamate decarboxylase 1 and 2, respectively, which are the rate-limiting enzymes responsible for synthesizing gamma-aminobutyric acid (GABA), the primary inhibitory neurotransmitter in the central nervous system. The concurrent upregulation of NPY, which signals through inhibitory Gi-coupled receptors, and the GABA-synthesizing enzymes suggests that HFDS conditions trigger a robust enhancement of inhibitory signaling capacity in CeA-NPY neurons.

Analysis of glutamatergic signaling components revealed that the translation of multiple α-amino-3-hydroxy-5-methyl-4-isoxazolepropionic acid (AMPA) and kainate glutamate receptors (*Gria 1, 2, 4* and *Grik 1, 2, 3*), which mediate fast excitatory synaptic transmission, were significantly downregulated under HFD conditions. However, *Gria1* (AMPA receptor subunit 1) and *Grik3* (kainate receptor subunit 3) showed selective reactivation under HFDS conditions (Figure 5E), indicating that excitatory synaptic transmission is also modulated by stress. Specifically, this selective reactivation of glutamate receptor subunits might contribute to altered neuronal excitability and synaptic plasticity in NPY neurons under combined stress and HFD conditions.

To further define the molecular identity of CeA-NPY neurons which represent appetite-regulating neuronal populations we investigated the translational changes of some key genes that had been linked to regulate feeding behavior, including the serotonin receptor 2A (*Htr2a*) and prepronociceptin (*Pnoc*) which have been shown to promote food intake (Douglass et al., 2017; Hardaway et al., 2019), while presence of protein kinase C delta (*Prkcd*) is associated with suppression of feeding (Cai et al., 2014). Our analysis revealed significant enrichment of the orexigenic markers *Htr2a* and *Pnoc* in CeA NPY neurons, while the anorexigenic marker *Prkcd* was undetectable (Figure 5F). This molecular profile clearly positions CeA-NPY neurons within the orexigenic circuit, distinct from the anorexigenic *Prkcd*-expressing population.

To validate our translational profiling findings at the protein level and confirm NPY's role as a key mediator of HFDS effects, we performed NPY immunostaining on brain sections from HFD and HFDS-treated mice. Consistent with our TRAP-seq data, we observed reduced NPY protein levels in the CeA of HFD mice, with a striking upregulation of NPY in the CeA under HFDS conditions (Figure 5G). This is consistent to previously reported NPY promoter activity changes under HFD and HFDS conditions [11]. Furthermore, aligned with recent circuit tracing studies showing CeA-NPY neuron projections to both LHb and LH [6], we found that NPY immunoreactivity followed a similar pattern in both target regions: decreased under HFD conditions but robustly increased under HFDS conditions (Figure 5G). Moreover, using our Npy-GFP mouse model, where the expression of GFP is under the control of the Npy promoter, demonstrated that Npy is not expressed in the LHb (Figure 5G), and the level of NPY we detected there originated from upstream protectory neurons, including the predominant CeA-NPY circuit [6]. These findings provide compelling evidence that CeA-NPY neurons serve as the primary source of NPY signaling to the LHb and LH, establishing a dual inhibitory mechanism for modulating energy homeostasis under conditions combining chronic stress with energy oversupply.

**Figure 6:**
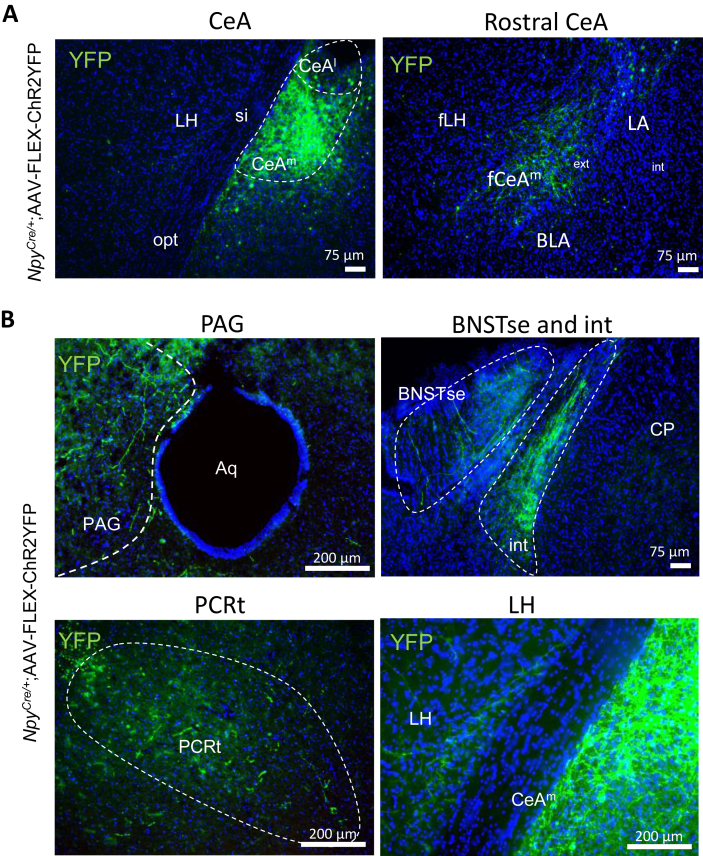
Circuit tracing of the CeA-NPY neuronal network. (A) Representative images of YFP expression in the CeA; (B) in the periaqueductal gray (PAG), the bed nucleus of the stria terminalis (BNSTse), the parvicellular reticular formation (PCRt) and the lateral hypothalamus (LH). Opt, optic tract; si, substantia innominate; CP, caudal putamen; LA, lateral amygdala; BLA, basolateral amygdala; ext, external capsule.

To identify and characterize downstream neural circuits directly connected to NPY neurons originating from the CeA, we employed an anterograde tracing approach. Direct visualization of Npy neuron projections and their target regions was achieved through this technique. A Cre-dependent adeno-associated virus expressing yellow fluorescent protein (YFP) was utilized as the tracing tool. Stereotaxic injection of the virus into the CeA of *Npy*^*Cre/+*^ mice was performed, and high expression of YFP was confirmed specifically in the medial nuclei of the CeA, as well as in the rostral side of the CeA (Figure 6A).

Further downstream targets of CeA NPY neurons revealed projections to established target regions, including the LHb, LH and PAG [6]. In addition, three previously unreported projection targets were identified: the extended amygdala division of the bed nucleus of the stria terminalis (BNSTse), the internal capsule (int), and the PCRt (Figure 6B). These findings significantly broaden our understanding of the CeA Npy neural circuitry, revealing its influence on regions not previously linked to alterations in CeA NPY neuronal activity. This extensive connectivity may explain how stress-induced changes in CeA NPY networks contribute to stress-induced obesity.

## 3 Discussion

In this study, we provide a comprehensive molecular and circuit-level understanding of how Npy neurons in the CeA integrate and process dietary and stress signals to regulate feeding behavior through tightly regulated Npy circuits. Using TRAP-seq analysis, we revealed the molecular identity of these neurons and discovered how their translational profile dynamically changes under high fat diet (HFD) or a combined HFD with stress (HFDS) conditions, providing crucial insights into how Npy neurons originating from the CeA^m^ undergo functional gene remodelling and subsequently influence stress combined with high fat diet induced feeding behavior and obesity development.

Firstly, our molecular profiling definitively established the identity of CeA NPY neurons as part of the orexigenic circuit, characterized by the enrichment of markers that mark the appetite-promoting (Orexigenic) population of neurons such as *Htr2a* and *Pnoc* [5,15], while lacking the marker that characterises appetite-suppressing (Anorexigenic) neurons, *Prkcd* [4]. This molecular signature helps explain CeA Npy neurons’ robust ability to promote feeding behavior when activated [1,6,12]. Importantly, under non-stress, short-term HFD feeding condition, these neurons participate in a homeostatic protective mechanism, evidenced by the downregulation of *Npy* translation in response to HFD, similar to NPY neurons in the arcuate nucleus [8,11,16], a common physiological response that prevents overloading of calories. Importantly, this protective response is overridden by chronic stress, which results in the significant upregulation of *Npy* translation and protein output, providing the core molecular basis for stress-induced over-consumption of HFD.

The complex interaction between stress and metabolic signals is further revealed by our analysis of lipid pathway related gene translation. HFD feeding triggered significant upregulation of multiple gene families involved in lipid metabolism, including lipases (*Pla2g12a, Pla2g16, Pla2g7*), fatty acid transport proteins (*Slc27a1*), and metabolic enzymes (*Abhd3,4, Acaa2, Acot2*). Strikingly, many of these genes modulated by HFD showed reversed expression patterns under additional stress, suggesting that chronic stress on top of HFD fundamentally alters how these neurons process and respond to metabolic signals. This molecular rewiring may create conditions that favour energy storage over utilization, potentially explaining the accelerated weight gain observed under HFDS conditions. Of particular interest is the significant alteration in fatty acid-related enzymes in these neurons under energy-rich conditions. While neurons primarily utilize glucose as their energy source [17,18], the widespread changes in fatty acid metabolic enzymes suggest additional, non-energetic functions for these pathways in neuronal signaling. These could include membrane remodeling, lipid-based signal transduction, or the generation of lipid-derived signaling molecules. However, the precise roles of these fatty acid-associated pathways in CeA NPY neuronal function and their contribution to stress-induced feeding behavior require further investigation.

Another key finding of our study is the coordinated enhancement of inhibitory signaling under HFDS conditions. The concurrent upregulation of *Npy* translation and GABA-synthesizing enzymes (*Gad1* and *Gad2*) suggests that stress on top of HFD triggers a robust enhancement of inhibitory output from these CeA neurons [19]. This enhanced inhibitory capacity manifests through both local and long-range projections, particularly to the lateral habenula (LHb) and lateral hypothalamus (LH) [6]. The significance of this dual inhibitory mechanism is underscored by our immunohistochemistry findings, which showed increased NPY immunoreactivity in both the LHb and LH under HFDS conditions, despite the absence of local NPY expression in the LHb. This enhanced inhibitory output is likely to be the core mechanism that influences downstream targets of the CeA NPY neuronal projections such as the LH and LHb, triggering the behavioral adaptations observed under combined stress and HFD conditions [1,6,11]. The simultaneous activation of multiple inhibitory signaling pathways may represent a coordinated response mechanism through which CeA-NPY neurons modulate feeding behavior and stress responses. These molecular changes align with and explain our previous circuit analysis findings, where we identified LHb *Npy1r*-expressing neurons as a crucial integration node for homeostatic and hedonic feeding circuits [6]. The stress-induced enhancement of CeA originating NPY signaling to the LHb provides a direct mechanism for overriding the anti-reward function of this nucleus, promoting hedonic feeding despite energy surplus. This is further modulated by complex changes in glutamatergic signaling components, particularly the selective reactivation of specific AMPA and kainate receptor subunits under combined stress and HFD conditions, suggesting a tide regulation of synaptic plasticity in these circuits.

Our findings reveal multiple levels of regulation through which stress in combination with HFD can override homeostatic feeding controls. At the molecular level, HFDS alters the translation of genes involved in metabolic processing, neurotransmitter production, and synaptic function. These changes manifest at the circuit level through enhanced inhibitory output to key feeding-related nuclei, ultimately promoting hedonic feeding behavior despite energy surplus [6,20]. This multilevel understanding provides several potential therapeutic targets for treating stress-related eating disorders and obesity. Future treatments might target the stress-induced changes in lipid metabolism, modulate the enhanced inhibitory signaling, or regulate specific aspects of synaptic plasticity. Understanding how these molecular changes relate to circuit-level alterations could guide the development of more effective and targeted treatments for stress-induced obesity.

In conclusion, our work provides a comprehensive framework for understanding how CeA NPY neurons integrate dietary and stress signals to influence feeding behavior. By revealing both molecular and circuit-level mechanisms, we have identified multiple pathways through which chronic stress in combination with excessive calory supply may override homeostatic feeding controls, ultimately promoting accelerated obesity development.

## 4 Material and methods

### 4.1 Mice and animal care

All research and animal care procedures were approved by St. Vincent's Hospital Animal Ethics Committee and the University of New South Wales Animal Care and Ethics Committee in accordance with the Australian Code of Practice for the Care and Use of Animals for Scientific Purpose. Mice were housed under conditions of controlled temperature (22 °C for standard laboratory temperature) and illumination (12 h light cycle, lights on at 07:00 h). Mice had free access to water and were fed either a normal chow diet (8 % calories from fat, 21 % calories from protein, 71 % calories from carbohydrate, 2.6 kcal/g; Gordon's Speciality Stock Feeds, Yanderra, NSW, Australia) or high fat diet (HFD) (43 % calories from fat, 17 % calories from protein and 20 MJ/kg; Specialty Feeds, Glen Forrest, WA, Australia). All mouse models used were on a C57Bl6 background. Male and female mice were used for all experiments if not stated otherwise. Mice were pair housed two weeks before any experiment began. Body weight was monitored weekly. Health status checks in the animal breeding facility were performed on a regular 6 monthly cycle.

#### 4.1.1 Body weight, fat mass, insulin measurements

To determine effects of stress on top of a standard chow diet male and female mice underwent our standard 14-day stress paradigm described before [6,11,16]. For comparing Chow and ChowS mice, body weight, different fat depots were measured the same way as previous described [6,11,16], and a total of 12 male and 9 female mice were used, respectively. For insulin detection, serum from mice of the Chow and ChowS groups was collected at the end of the study and measured using an insulin RIA kits (Millipore). A total of 12 male animal were used.

### 4.2 Translating Ribosome Affinity purification and RNA-sequencing (TRAPseq)

TRAP was performed based on the previously published protocol [7,10,11,21]. RNA from the amygdala of *Npy*^*Cre/+*^*;*TRAP^*lox/lox*^ male mice that either underwent the 2 weeks of chow (n = 4), HFD(n = 4) or HFDS(n = 4) protocol was immunoprecipitated by using the TRAP protocol described above. To obtain enough RNA for purification and sequencing, CeA tissue from both hemispheres was collected from each animal for each immunoprecipitated protocol. To obtain enough RNA for sequencing, immunoprecipitated (IP) RNA from three male mice was pooled together for library preparation and Next-Generation sequencing. Using this method, four biological replicates were included for each treatment group (Chow, HFD and HFDS). Quality control of RNA was first confirmed by Bioanalyzer Picokit (Agilent) to ensure the RNA integrity number is above 8. A total of at least 50 ng of IP RNA was used for RNA-sequencing. Illumina HiSeq HT chemistry sequencing with 100 base pair single-end read was employed. FASTQ raw reads were checked using the FastQC tool [22] and to identify over-represented sequences, adapter sequences were removed if required by using the trimmomatic tool [23]. Reads were then mapped against the reference musculus mm10 genome using Bowtie2 [24] and reads count was determined using htseq-count [25]. Differential gene expression was determined using Deseq2 by independently compared dataset for HFD against dataset of Chow to generate HFD-induced translatome differences, and then datasets for HFDS were compared against HFD to identify additional chronic stress-induced translatome difference under HFD condition. To determine the putative changing genes a p-value of <0.05 was used in this study to be statistically significant. Functional ontology analysis was performed by using SYNGO [26] and Webgestait [27] with a significant enrichment value of q-value at less than 0.05.

### 4.3 Stereotaxic brain injection

Stereotaxic surgery was performed on anesthetized male mice (10–12 weeks old) following established protocols [11,16]. For circuit tracing experiments of the CeA Npy neurons, injections where targeted to the medial nuclei of central amygdala (CeA^M^) using coordinates relative to Bregma: anterioposterior −1.06 mm, mediolateral −2.6 mm, and dorsoventral −4.5 mm [28]. To specifically label and express YFP in Npy neurons from the CeA^M^, AAV-FLEX-ChR2-EYFP-WPRE [29] was used to inject into the CeA^M^ unilaterally into *Npy*^*Cre/+*^ mice. 0.5 μL of viral vector delivered at 0.1 μL/min via Hamilton Syringe and infusion pump (World Precision Instruments). For the circuit tracing experiment, a total of 4 animals with confirmed correctly injected CeA animals were included to proceed for future analysis of their downstream projection targets.

### 4.4 Immunohistochemistry and Fos positive cell counting

For immunohistochemistry on brain sections, mice were sacrificed by cardiac perfusion with 60 mL saline and 60 mL 4 % formaldehyde in PBS. Brains were isolated, post-fixed at 4 °C overnight in 4 % formaldehyde in PBS and dehydrated in 30 % glucose at 4 °C until they sank. Subsequently the brains were stored at −80 °C until sectioned coronally on a cryostat at 30 μm thickness. The exact protocol used here has previously been described [6]. For primary antibody incubation, polyclonal GFP antibody (1:1000; ThermoFisher Scientific; Cat #A11122) and a polyclonal Fos antibody (1:1000; Abcam; Cat #Ab190289) were used. For GFP detection, goat anti-Rabbit Alexa Fluor 488 antibody (1:1000; ThermoFisher Scientific; Cat #A32731) was used to amplify the YFP signal. To determine Fos positive cells in the CeA, mice that underwent Chow or ChowS for two weeks were sacrificed for brain collection. Brain sections and examination were performed using the previous published protocol [11,16]. Brain tissue from a total of 5 male mice were included for counting for these two conditions. The same procedure and number of male mice was used to determine for the number of GFP positive cells in the CeA of Npy-GFP reporter mice that underwent either the chow or the chowS treatment [11,16]. A total of 5 mice was included for counting.

## CRediT authorship contribution statement

**Chi Kin Ip:** Writing – review & editing, Writing – original draft, Visualization, Validation, Methodology, Investigation, Data curation, Conceptualization. **Lei Zhang:** Writing – review & editing, Validation, Supervision, Methodology. **Ramon Tasan:** Writing – review & editing, Resources, Methodology. **Herbert Herzog:** Writing – review & editing, Writing – original draft, Validation, Supervision, Resources, Conceptualization.

## Declaration of competing interest

The authors declare no competing interests.

## Data Availability

Data will be made available on request.

## Appendix A Supplementary data

Supplementary Table 1 Differential gene expression in HFD and HFDS conditions. Supplementary Table 1

Supplementary Table 2 Functional ontology analysis of the HFD-induced differentially expressed genes. Supplementary Table 2

Supplementary Table 3 Functional ontology analysis of the HFDS-induced differentially expressed genes. Supplementary Table 3

Supplementary Table 4 Phenotypic ontology analysis of the HFDS-induced differentially expressed genes. Supplementary Table 4

Supplementary Figure 1 (A) Bodyweight curve of female WT mice on a chow or a chow combined with stress paradigm for 25 days. Data means ± SEM, 7–9 mice per group. (B) Absolute Weight of dissected white adipose depots between male mice on a 14-day Chow or ChowS paradigm: inguinal fat (i), epididymal (e), mesenteric (m), perirenal fat (r) and summed total fat mass and BAT. Data are means ± SEM, 12 mice per group. (C) Fat mass weight of white adipose depots normalized to body weight of male mice on a 14-days Chow or ChowS paradigm: inguinal fat (i), epididymal (e), mesenteric (m), and perirenal fat (r), BAT and sum of total fat mass (Total). Data are means ± SEM, 12 mice per group. (D) Serum insulin levels of male mice on a 14-day Chow and ChowS paradigm at the end of treatment. Data are means ± SEM, 8 mice per group. (E) Quantification of Fos expression in the CeA of male mice 14 days on either a Chow or a ChowS paradigm. Data are means ± SEM, n = 5 mice per group. (F) Quantification of GFP positive neurons in the CeA in Npy GFP reporter mice under a 14day Chow or ChowS paradigm. Data are means ± SEM, 5 mice per group. Supplementary Figure 1
