# Supplementary material for: Stress and high fat diet reconfigure the active translatome of CeA-NPY neurons
Source: Mol Metab. 2025 Jun 4;98:102176. doi: 10.1016/j.molmet.2025.102176 (PMC12214123; doi:10.1016/j.molmet.2025.102176)
Supplement: Supplementary Table 1 — Differential gene expression in HFD and HFDS conditions. [file mmc1.pdf]

GeneID	Base mean	log2(FC)	StdErr	Wald-Stats	P-value	P-adj		GeneID	Normalised expression for Chow#1	Normalised expression for Chow#2	Normalised expression for Chow#3	Normalised expression for Chow#4	Normalised expression for HFD#1	Normalised expression for HFD#2	Normalised expression for HFD#3	Normalised expression for HFD#4
S100b	63.26637	-2.2470014	0.31873814	-7.0496785	1.79E-12	1.55E-08		S100b	131.72108	119.918786	86.5174974	105.475141	19.9777406	12.9365998	25.0188611	4.56525506
S100a1	39.3837827	-2.2765473	0.32603147	-6.9825998	2.90E-12	1.55E-08		S100a1	90.054208	78.7391271	53.1191988	53.7716403	6.65924688	9.40843619	9.62263889	13.6957652
Sparc	150.733511	-2.369971	0.34661563	-6.8374614	8.06E-12	2.87E-08		Sparc	120.968339	345.275598	199.117476	413.628002	48.2795399	28.2253086	29.8301805	20.5436478
Ndrg2	438.485089	-1.8423818	0.2737718	-6.729626	1.70E-11	4.55E-08		Ndrg2	632.395595	755.262087	526.420802	932.731145	214.760712	105.844907	194.377305	146.088162
Malat1	555.253423	1.99383739	0.31342915	6.36136552	2.00E-10	4.27E-07		Malat1	132.393127	183.272106	142.499407	250.244941	915.646446	1060.80118	1321.18832	435.981858
Kcnj10	71.0487344	-2.0838305	0.33145595	-6.2869004	3.24E-10	5.77E-07		Kcnj10	90.054208	168.338823	89.3802087	140.633521	34.9610461	18.8168724	12.5094306	13.6957652
Fxyd1	21.114247	-2.3203423	0.37668077	-6.1599701	7.28E-10	1.11E-06		Fxyd1	29.5700384	50.2301328	28.9451921	45.4990802	8.3240586	1.17605452	2.88679167	2.28262753
Cmtm5	17.2022906	-2.3152195	0.37845114	-6.1176179	9.50E-10	1.27E-06		Cmtm5	27.5538995	32.129184	25.4463228	41.3628002	4.99443516	0	3.84905555	2.28262753
Ntsr2	43.8723261	-2.1842452	0.35951913	-6.0754632	1.24E-09	1.47E-06		Ntsr2	57.1239379	121.276357	62.0254117	70.3167603	18.3129289	3.52816357	11.5471667	6.84788259
Cst3	217.230412	-1.7045948	0.28607454	-5.9585687	2.54E-09	2.72E-06		Cst3	234.544169	535.33556	348.614622	266.790061	104.883138	79.9717076	92.3773333	75.3267085
Rab31	41.6003299	-1.6676561	0.29512348	-5.6507064	1.60E-08	1.55E-05		Rab31	71.2369108	68.3310816	48.3480132	76.5211804	18.3129289	14.1126543	15.3962222	20.5436478
S1pr1	94.5880823	-2.0251524	0.36272045	-5.5832321	2.36E-08	1.98E-05		S1pr1	151.88247	273.324326	157.449122	76.5211804	38.2906696	23.5210905	28.8679167	6.84788259
Mmd2	94.9158122	-1.6675205	0.29885997	-5.5796048	2.41E-08	1.98E-05		Mmd2	112.903783	222.189146	131.048562	138.565381	53.2739751	27.0492541	42.3396111	31.9567854
Gfap	119.138429	-1.6741275	0.30077434	-5.5660583	2.61E-08	1.99E-05		Gfap	186.156833	223.546717	166.673414	184.064461	84.9053977	30.5774176	45.2264028	31.9567854
Mt2	60.0436752	-1.9552974	0.35222271	-5.5513099	2.84E-08	2.02E-05		Mt2	81.9896521	113.583453	85.8813393	128.224681	26.6369875	3.52816357	15.3962222	25.1089028
Prdx6	78.8974552	-1.5629012	0.29276123	-5.3384843	9.37E-08	6.26E-05		Prdx6	133.065173	137.114687	120.870033	101.338861	53.2739751	19.9929269	40.4150833	25.1089028
Mt1	160.195569	-1.9366765	0.3688323	-5.2508321	1.51E-07	9.52E-05		Mt1	268.146485	308.168653	230.289221	301.948441	58.2684102	9.40843619	77.943375	27.3915304
Slc9a3r1	29.3634839	-1.7773228	0.34929984	-5.0882441	3.61E-07	0.00021459		Slc9a3r1	58.4680306	56.1129411	43.8949068	39.2946602	11.653682	5.88027262	17.32075	2.28262753
Gpr37l1	68.1526046	-1.7708803	0.34916303	-5.0717864	3.94E-07	0.00022169		Gpr37l1	57.7959842	177.389298	94.7875523	122.020261	33.2962344	12.9365998	17.32075	29.6741579
Hepacam	53.814597	-1.6329135	0.32404697	-5.039126	4.68E-07	0.00023317		Hepacam	74.5971425	135.304592	79.2016796	55.8397803	29.966611	18.8168724	23.0943333	13.6957652
Csrp1	134.680153	-1.7819054	0.35393384	-5.034572	4.79E-07	0.00023317		Csrp1	202.285945	197.752865	155.540648	343.311242	71.586904	16.4647633	28.8679167	61.6309433
Mobp	43.2859566	-1.7607189	0.34975449	-5.0341566	4.80E-07	0.00023317		Mobp	50.4034746	83.2643643	49.3022503	103.407001	19.9777406	9.40843619	7.69811111	22.8262753
Abhd4	38.4939297	-1.5793518	0.31779664	-4.9696932	6.71E-07	0.00031162		Abhd4	61.8282622	63.3533207	45.4853019	72.3849004	24.9721758	15.2887088	8.660375	15.9783927
Slc25a18	35.9767474	-1.7828746	0.35978878	-4.9553369	7.22E-07	0.00032155		Slc25a18	59.8121232	54.3028463	36.579089	90.9981605	18.3129289	3.52816357	10.5849028	13.6957652
Apoe	280.043433	-1.600293	0.32553438	-4.9158956	8.84E-07	0.00037783		Apoe	420.028955	703.674383	424.317432	237.836101	166.481172	121.133616	102.962236	63.9135709
Ttyh1	252.438358	-1.485451	0.30542214	-4.8635997	1.15E-06	0.00047385		Ttyh1	301.748801	585.565692	357.520835	314.357282	176.470042	95.2604165	72.1697916	116.414004
Cldn11	25.3850964	-1.7410579	0.35916505	-4.8475149	1.25E-06	0.00049488		Cldn11	25.5377605	57.923036	35.9429309	49.6353602	11.653682	9.40843619	3.84905555	9.13051012
Slc39a12	17.2938702	-1.8699538	0.38990756	-4.79589	1.62E-06	0.0006182		Slc39a12	21.5054825	56.1129411	27.0367179	16.5451201	4.99443516	4.7042181	2.88679167	4.56525506
Sepp1	107.75587	-1.8556542	0.39465623	-4.7019508	2.58E-06	0.00089764		Sepp1	141.129729	372.427021	194.028211	51.7035003	16.6481172	31.7534722	45.2264028	9.13051012
Gstm1	125.772335	-1.6811063	0.35762658	-4.7007307	2.59E-06	0.00089764		Gstm1	211.694593	172.411537	148.542909	295.744021	63.2628454	11.7605452	36.5660278	66.1961984
Cldn10	11.9933482	-1.8965712	0.4035828	-4.699336	2.61E-06	0.00089764		Cldn10	10.0806949	33.9392789	21.3112953	22.7495401	1.66481172	2.35210905	3.84905555	0
Ptgds	210.506554	-1.7928326	0.38199352	-4.6933585	2.69E-06	0.00089764		Ptgds	224.463474	746.211613	348.296543	126.156541	49.9443516	61.1548353	98.1509166	29.6741579
Cryab	56.7622942	-1.5459732	0.33172568	-4.6603964	3.16E-06	0.00102216		Cryab	88.038069	81.4542694	64.570044	126.156541	36.6258578	9.40843619	25.0188611	22.8262753
Zcchc24	51.2863835	-1.3751954	0.30526384	-4.5049406	6.64E-06	0.00208704		Zcchc24	75.9412351	87.7896015	74.7485731	72.3849004	43.2851047	16.4647633	25.981125	13.6957652
Tmbim1	10.992412	-1.707096	0.3824643	-4.4634126	8.07E-06	0.00246326		Tmbim1	16.8011582	22.6261859	18.130505	16.5451201	4.99443516	2.35210905	1.92452778	4.56525506
Gatm	35.298787	-1.4537428	0.32620311	-4.4565571	8.33E-06	0.00247268		Gatm	50.4034746	68.3310816	50.2564875	51.7035003	23.3073641	9.40843619	22.1320694	6.84788259
Slc15a2	6.71518834	-1.8230433	0.41121674	-4.4332905	9.28E-06	0.00267587		Slc15a2	14.1129729	17.1959013	12.4050823	6.20442003	1.66481172	1.17605452	0.96226389	0
Fads2	73.8523852	-1.5061999	0.34015821	-4.4279393	9.51E-06	0.00267587		Fads2	81.3176057	202.730626	98.2864217	82.7256004	46.6147282	24.697145	20.2075417	34.239413
Scn4b	28.106048	-1.6216005	0.37636295	-4.3086082	1.64E-05	0.00450225		Scn4b	65.1884939	45.2523719	58.2084633	16.5451201	14.9833055	9.40843619	3.84905555	11.4131377
Miat	16.770979	1.6586845	0.38652014	4.29132752	1.78E-05	0.00471272		Miat	2.01613899	4.52523719	4.13502745	12.4088401	44.9499164	15.2887088	21.1698055	29.6741579
Clu	302.965464	-1.4504973	0.33831699	-4.2873913	1.81E-05	0.00471272		Clu	429.437604	784.676129	493.976741	184.064461	161.486737	164.647633	98.1509166	107.283494
Fads1	104.27688	-1.4391432	0.34055459	-4.2258811	2.38E-05	0.00605673		Fads1	124.328571	271.514231	149.815225	105.475141	54.9387868	70.5632714	27.9056528	29.6741579
Slc4a4	105.837751	-1.3559737	0.32170004	-4.215025	2.50E-05	0.00620775		Slc4a4	123.656524	222.189146	159.039517	138.565381	78.2461509	65.8590533	31.7547083	27.3915304
Prdx6b	24.0086782	-1.4430148	0.34546327	-4.1770425	2.95E-05	0.00717368		Prdx6b	31.5861774	48.8725616	27.990955	43.4309402	3.32962344	12.9365998	12.5094306	11.4131377
C1qb	8.16788097	-1.7077248	0.41016652	-4.1634915	3.13E-05	0.00744402		C1qb	10.7527413	26.6988994	13.6773985	8.27256004	1.66481172	2.35210905	1.92452778	0
Pla2g16	15.0702562	-1.462045	0.35416105	-4.1281926	3.66E-05	0.00849524		Pla2g16	24.8657142	28.5089943	23.8559276	18.6132601	3.32962344	5.88027262	8.660375	6.84788259
1700003M07Rik	12.5093128	-1.5222334	0.37588278	-4.0497555	5.13E-05	0.01143419		1700003M07R	17.4732045	25.793852	17.4943469	22.7495401	1.66481172	5.88027262	6.73584722	2.28262753
Kcnc2	205.616141	1.11982747	0.27670401	4.04702294	5.19E-05	0.01143419		Kcnc2	94.086486	118.108691	154.90449	111.679561	193.11816	426.907792	279.056528	267.067421
Avp	17.7481702	-1.6701844	0.41294566	-4.0445622	5.24E-05	0.01143419		Avp	8.06455594	9.95552182	12.4050823	107.543281	1.66481172	2.35210905	0	0
Pon2	16.2677231	-1.6535521	0.41152258	-4.0181323	5.87E-05	0.01253943		Pon2	34.2743627	52.4927514	27.354797	2.06814001	3.32962344	3.52816357	4.81131944	2.28262753
Aldh1a1	15.9832367	-1.6278816	0.40777342	-3.9921228	6.55E-05	0.01372349		Aldh1a1	40.3227797	40.274611	25.1282437	6.20442003	0	2.35210905	6.73584722	6.84788259
2900052N01Rik	39.2480298	-1.5774834	0.39660652	-3.9774521	6.97E-05	0.01401589		2900052N01R	90.054208	85.9795066	78.2474425	12.4088401	11.653682	14.1126543	19.2452778	2.28262753
S100a16	24.0169062	-1.5192388	0.3823375	-3.9735542	7.08E-05	0.01401589		S100a16	34.2743627	27.1514231	27.0367179	70.3167603	18.3129289	4.7042181	5.77358333	4.56525506
Il33	23.8809056	-1.2795701	0.32228648	-3.9702878	7.18E-05	0.01401589		Il33	33.6023164	43.8948007	28.6271131	37.2265202	16.6481172	9.40843619	12.5094306	9.13051012
Heyl	12.9687423	-1.6521517	0.41624676	-3.9691641	7.21E-05	0.01401589		Heyl	7.39250961	16.2908539	8.90621297	64.1123403	1.66481172	1.17605452	1.92452778	2.28262753
Paqr8	77.4833523	-1.2270036	0.31046184	-3.9521882	7.74E-05	0.01477994		Paqr8	65.8605402	179.651916	107.510714	101.338861	46.6147282	37.6337448	35.6037639	45.6525506
Mlc1	46.8638044	-1.4011332	0.35548995	-3.9414143	8.10E-05	0.01508923		Mlc1	55.1077989	99.1026944	71.5677828	66.1804803	39.9554813	7.05632714	15.3962222	20.5436478
Ppap2b	103.395475	-1.3534503	0.34361836	-3.9388183	8.19E-05	0.01508923		Ppap2b	132.393127	281.469753	146.634435	74.4530404	66.5924688	32.9295267	53.8867778	38.804668
Slc7a10	11.6916064	-1.49441	0.3816044	-3.9161235	9.00E-05	0.01624729		Slc7a10	14.1129729	24.4362808	17.4943469	20.6814001	8.3240586	2.35210905	3.84905555	2.28262753
Adhfe1	9.68370232	-1.5439232	0.39483118	-3.9103375	9.22E-05	0.01624729		Adhfe1	12.7688802	15.8383302	12.0870033	24.8176801	3.32962344	1.17605452	2.88679167	4.56525506
Fgf1	37.6657942	-1.2442376	0.31855229	-3.9059134	9.39E-05	0.01624729		Fgf1	67.8766792	66.068463	48.3480132	41.3628002	28.3017992	12.9365998	25.0188611	11.4131377
Ednrb	21.5803915	-1.5353197	0.39317374	-3.9049398	9.42E-05	0.01624729		Ednrb	24.8657142	63.3533207	44.5310648	12.4088401	4.99443516	10.5844907	9.62263889	2.28262753
Tpcn1	28.4048286	-1.2980989	0.33302248	-3.8979319	9.70E-05	0.01645913		Tpcn1	33.6023164	62.4482732	36.897168	37.2265202	18.3129289	14.1126543	8.660375	15.9783927
Tceal5	62.7386715	1.31750287	0.34053393	3.86893268	0.00010931	0.01815917		Tceal5	20.8334362	17.1959013	30.8536663	53.7716403	73.2517157	65.8590533	89.4905416	150.653417
Glul	594.269033	-1.1472684	0.29672491	-3.8664379	0.00011044	0.01815917		Glul	896.509802	965.685616	700.410034	850.005544	567.700797	192.872942	281.943319	299.024207
Agt	22.9423055	-1.488736	0.3860146	-3.8566832	0.00011494	0.01861266		Agt	13.4409266	46.1574193	25.7644018	66.1804803	8.3240586	5.88027262	8.660375	9.13051012
Gm2a	33.5347767	-1.4011547	0.36454597	-3.843561	0.00012126	0.01926014		Gm2a	46.3711967	91.4097912	52.4830407	20.6814001	14.9833055	9.40843619	19.2452778	13.6957652
Plekhb1	88.1564781	-1.3846981	0.36050537	-3.8409916	0.00012254	0.01926014		Plekhb1	104.167181	140.734877	93.5152361	213.018421	48.2795399	10.5844907	35.6037639	59.3483158
Tsc22d4	61.1120425	-1.4518117	0.38050873	-3.8154491	0.00013594	0.02105621		Tsc22d4	87.3660227	80.0966983	53.1191988	173.723761	33.2962344	10.5844907	9.62263889	41.0872956
Apod	27.8124846	-1.5049479	0.39504327	-3.8095773	0.0001392	0.02125455		Apod	32.9302701	94.5774573	47.3937761	10.3407001	9.98887032	11.7605452	8.660375	6.84788259
Ppp1r3c	26.4261742	-1.1819481	0.31086032	-3.8021839	0.00014343	0.0213881		Ppp1r3c	48.3873356	42.5372296	35.6248519	26.8858201	14.9833055	12.9365998	16.3584861	13.6957652
Necap2	11.6265155	-1.4625841	0.38478376	-3.8010547	0.00014408	0.0213881		Necap2	15.4570656	17.1959013	13.9954775	31.0221002	3.32962344	5.88027262	3.84905555	2.28262753
Cort	14.0600077	1.42613246	0.37859933	3.76686469	0.00016531	0.0240486		Cort	5.37637063	4.52523719	9.54237104	0	18.3129289	23.5210905	19.2452778	31.9567854
Dlx1	65.6651005	1.14104646	0.30306136	3.76506739	0.0001665	0.0240486		Dlx1	36.9625481	26.6988994	33.7163777	51.7035003	93.2294563	77.6195986	61.5848889	143.805534
Trim2	362.701027	0.70720171	0.1885507	3.7507244	0.00017632	0.02512741		Trim2	263.442161	252.055711	299.94853	266.790061	367.92339	491.590791	459.962139	499.895429
Aldoc	290.068614	-1.2053068	0.3219735	-3.7434967	0.00018148	0.02552139		Aldoc	548.389804	407.723871	339.072251	411.559862	281.353181	84.6759257	140.490528	107.283494
Carhsp1	26.6132798	-1.3135873	0.35351483	-3.7157913	0.00020257	0.02811759		Carhsp1	32.9302701	34.3918026	35.9429309	59.9760603	21.6425524	4.7042181	9.62263889	13.6957652
Nfia	59.6412224	-1.0731315	0.28966444	-3.7047403	0.00021161	0.02899564		Nfia	75.2691888	101.817837	80.7920748	78.5893204	44.9499164	21.1689814	47.1509305	27.3915304
Sox9	34.8203089	-1.2875493	0.34929026	-3.6861873	0.00022764	0.03079752		Sox9	64.5164475	43.442277	40.0779583	64.1123403	33.2962344	8.23238167	13.4716944	11.4131377
Dock1	19.8358765	-1.3406138	0.36450005	-3.6779522	0.00023511	0.03141123		Dock1	33.6023164	42.9897533	23.5378486	24.8176801	13.3184938	11.7605452	8.660375	0
Rftn2	19.6073735	-1.3308444	0.3636327	-3.6598589	0.00025235	0.03329829		Rftn2	40.3227797	34.3918026	31.8079035	14.4769801	11.653682	3.52816357	11.5471667	9.13051012
Mal	54.8050278	-1.2699578	0.34731145	-3.6565388	0.00025564	0.03330465		Mal	65.1884939	122.633928	68.3869924	72.3849004	28.3017992	18.8168724	12.5094306	50.2178057
Mog	7.81689289	-1.4756286	0.40396368	-3.6528746	0.00025932	0.03330465		Mog	9.4086486	19.9110436	15.2677937	8.27256004	3.32962344	1.17605452	2.88679167	2.28262753
Cxcl14	32.5099493	-1.2803379	0.35083809	-3.6493698	0.00026288	0.03330465		Cxcl14	49.7314283	71.4987476	54.7095939	22.7495401	14.9833055	16.4647633	23.0943333	6.84788259
Vwc2	14.9541556	1.36261828	0.37358215	3.64743948	0.00026487	0.03330465		Vwc2	4.7043243	6.33533207	7.95197586	6.20442003	14.9833055	43.5140174	15.3962222	20.5436478
Slc1a3	187.406329	-1.3692695	0.37672127	-3.6347018	0.0002783	0.03458716		Slc1a3	237.9044	554.794079	287.543447	107.543281	104.883138	107.020962	72.1697916	27.3915304
Igfbp4	105.223923	1.23298953	0.34196472	3.60560453	0.00031143	0.038259		Igfbp4	32.2582238	35.7493738	48.0299342	99.2707205	114.872009	137.598379	118.358458	255.654283
Pdk4	4.99756759	-1.4945338	0.41517733	-3.599748	0.00031853	0.0386864		Pdk4	6.72046328	14.0282353	9.224292	6.20442003	1.66481172	1.17605452	0.96226389	0
Atp1b2	499.934255	-1.0632919	0.29709737	-3.5789342	0.000345	0.04098711		Atp1b2	459.679689	1008.67537	641.565413	703.167603	382.906696	234.03485	190.52825	378.91617
Inha	8.11766687	1.47023132	0.41097844	3.57739281	0.00034704	0.04098711		Inha	2.68818531	3.16766603	0.9542371	2.06814001	24.9721758	7.05632714	5.77358333	18.2610202
Papss2	16.5429566	-1.310017	0.36634213	-3.5759387	0.00034897	0.04098711		Papss2	27.5538995	34.8443264	27.0367179	12.4088401	13.3184938	5.88027262	6.73584722	4.56525506
Tmem229a	64.4831897	-1.0994472	0.30986712	-3.5481247	0.00038798	0.0447252		Tmem229a	59.8121232	100.912789	69.9773876	138.565381	43.2851047	24.697145	37.5282917	41.0872956
Lpar1	6.75865601	-1.4594306	0.41201768	-3.5421553	0.00039687	0.0447252		Lpar1	6.72046328	20.3635674	11.4508452	8.27256004	1.66481172	2.35210905	0.96226389	2.28262753
Crhbp	55.7413548	1.14971979	0.3246611	3.54129214	0.00039817	0.0447252		Crhbp	24.1936678	33.0342315	35.9429309	28.9539601	48.2795399	136.422325	86.60375	52.5004332
Fam107a	174.099487	-1.2631038	0.35676649	-3.5404216	0.00039949	0.0447252		Fam107a	276.211041	424.919772	253.82707	105.475141	88.2350212	57.6266717	138.566	47.9351782
Abhd3	17.3641365	-1.3158289	0.37181345	-3.538949	0.00040172	0.0447252		Abhd3	24.1936678	42.0847059	25.4463228	14.4769801	8.3240586	5.88027262	4.81131944	13.6957652
Kcnj16	19.8438741	-1.3259174	0.37621514	-3.5243594	0.00042451	0.04677466		Kcnj16	27.5538995	48.8725616	36.2610099	12.4088401	9.98887032	11.7605452	9.62263889	2.28262753
Slc38a3	19.8856152	-1.3213573	0.37722378	-3.5028472	0.00046031	0.0496153		Slc38a3	26.2098068	53.3977988	31.1717454	12.4088401	11.653682	7.05632714	5.77358333	11.4131377
Drd2	19.9364936	-1.2675444	0.36191664	-3.5023103	0.00046124	0.0496153		Drd2	38.978687	24.4362808	38.1694841	18.6132601	9.98887032	11.7605452	3.84905555	13.6957652
Msn	8.43441905	-1.3799315	0.39419876	-3.5005983	0.00046421	0.0496153		Msn	16.8011582	13.1231878	11.4508452	14.4769801	4.99443516	4.7042181	1.92452778	0
Armcx4	57.1274363	1.0332988	0.29553529	3.49636349	0.00047165	0.04991039		Armcx4	26.2098068	36.6544212	33.3982986	43.4309402	83.240586	117.605452	54.8490416	61.6309433
Gja1	68.1526136	-1.4084972	0.40493641	-3.4783169	0.00050457	0.05287134		Gja1	75.2691888	233.049715	117.689243	28.9539601	31.6314227	29.4013631	26.9433889	2.28262753
Plp1	241.862034	-1.050657	0.30330415	-3.4640378	0.00053213	0.05521772		Plp1	239.248493	487.368045	365.79089	264.721921	161.486737	105.844907	98.1509166	212.28436
Rnf144b	28.8892742	0.96369887	0.27885078	3.45596617	0.00054832	0.05635085		Rnf144b	16.8011582	16.2908539	21.6293743	18.6132601	38.2906696	43.5140174	39.4528194	36.5220405
Dlx1as	84.3608037	1.15978218	0.33789931	3.43233072	0.00059842	0.06083535		Dlx1as	37.6345944	32.5817078	33.3982986	78.5893204	133.184938	75.2674895	85.6414861	198.588595
Luc7l3	47.034537	1.05302708	0.30699546	3.43010641	0.00060334	0.06083535		Luc7l3	26.8818531	22.1736622	25.7644018	37.2265202	46.6147282	72.9153805	44.2641389	100.435611
Timp4	13.6902629	-1.2817821	0.37756483	-3.3948663	0.00068662	0.06858512		Timp4	12.7688802	24.4362808	19.7209001	28.9539601	9.98887032	2.35210905	6.73584722	4.56525506
Fmo1	5.1607463	-1.400734	0.41443324	-3.3798784	0.00072518	0.07141036		Fmo1	12.7688802	8.59795066	9.224292	6.20442003	0	3.52816357	0.96226389	0
Zc3h13	64.830892	0.92450774	0.27362744	3.37870992	0.00072827	0.07141036		Zc3h13	36.2905017	35.7493738	36.2610099	64.1123403	81.5757743	98.78858	65.4339444	100.435611
Sipa1l2	63.3215073	1.01118923	0.30014855	3.36896261	0.00075452	0.07297314		Sipa1l2	28.2259458	35.2968501	36.2610099	55.8397803	104.883138	79.9717076	51.96225	114.131377
Scd1	103.946099	-1.1694472	0.34724951	-3.3677434	0.00075786	0.07297314		Scd1	112.903783	237.122429	141.227091	124.088401	94.8942681	63.5069443	32.7169722	25.1089028
Olfr287	5.05418929	-1.4004736	0.41621657	-3.3647715	0.00076607	0.07310505		Olfr287	6.04841696	13.5757116	4.45310648	12.4088401	1.66481172	0	0	2.28262753

GeneID	Base mean	log2(FC)	StdErr	Wald-Stats	P-value	P-adj	GeneID	Normalised expression for Chow#1	Normalised expression for Chow#2	Normalised expression for Chow#3	Normalised expression for Chow#4	Normalised expression for HFD#1	Normalised expression for HFD#2	Normalised expression for HFD#3	Normalised expression for HFD#4
Slc6a11	150.947955	-1.2888522	0.38405838	-3.3558756	0.00079114	0.07482937	Slc6a11	96.7746713	243.457761	132.320878	475.672202	111.542385	38.8097993	33.6792361	75.3267085
Gltp	21.234279	-1.1570524	0.34684589	-3.3359265	0.00085016	0.07970589	Gltp	26.8818531	32.5817078	26.7186389	39.2946602	19.9777406	4.7042181	10.5849028	9.13051012
Tmem198b	5.17425114	-1.3706149	0.41320307	-3.3170491	0.00090974	0.08455009	Tmem198b	7.39250961	13.1231878	8.58813393	6.20442003	1.66481172	1.17605452	0.96226389	2.28262753
Slco1c1	12.7639448	-1.3000827	0.39293499	-3.3086457	0.00093748	0.08637782	Slco1c1	11.4247876	34.3918026	20.9932163	14.4769801	9.98887032	4.7042181	3.84905555	2.28262753
Apln	10.6131631	-1.3458529	0.40721801	-3.3049935	0.00094979	0.08676337	Apln	15.4570656	18.1009488	11.7689243	26.8858201	4.99443516	0	7.69811111	0
Sox8	33.2761698	-1.183579	0.36112521	-3.2774753	0.0010474	0.0948695	Sox8	36.2905017	43.442277	34.0344567	86.8618804	29.966611	9.40843619	12.5094306	13.6957652
Gad1	414.684631	0.83748316	0.25586748	3.27311295	0.0010637	0.09553631	Gad1	308.469265	173.316584	351.159254	306.084722	434.515859	576.266717	533.094194	634.570454
Zrsr2	56.2551419	0.79480988	0.24300664	3.27073317	0.00107269	0.09554099	Zrsr2	40.3227797	37.5594687	32.7621406	51.7035003	69.9220923	72.9153805	74.0943194	70.7614535
Hnrnpm	39.3114294	0.92618355	0.28372059	3.26442134	0.00109688	0.09615272	Hnrnpm	31.5861774	18.5534725	25.4463228	26.8858201	51.6091633	68.2111624	44.2641389	47.9351782
Gjb6	14.8061373	-1.3504758	0.41393038	-3.2625674	0.00110408	0.09615272	Gjb6	10.0806949	55.2078937	26.4005599	10.3407001	1.66481172	7.05632714	7.69811111	0
Baalc	146.353901	-0.7368777	0.22590207	-3.2619343	0.00110655	0.09615272	Baalc	186.156833	193.680152	150.133304	217.154701	116.53682	91.7322529	124.132042	91.3051012
St3gal4	9.38067739	-1.2877296	0.39506036	-3.2595768	0.00111579	0.09617354	St3gal4	15.4570656	23.5312334	13.3593194	8.27256004	1.66481172	4.7042181	5.77358333	2.28262753
Chpt1	17.8250027	-1.2554084	0.38627131	-3.2500691	0.00115377	0.09825352	Chpt1	22.8495752	50.6826565	26.0824808	12.4088401	6.65924688	5.88027262	13.4716944	4.56525506
Rreb1	8.34035923	1.34051451	0.41265961	3.24847519	0.00116025	0.09825352	Rreb1	2.68818531	4.07271347	2.54463228	0	29.966611	3.52816357	12.5094306	11.4131377
Tlcd1	5.99608614	-1.3467786	0.41494288	-3.2456965	0.00117164	0.09825352	Tlcd1	7.39250961	13.5757116	11.4508452	8.27256004	4.99443516	0	0	2.28262753
Gpam	24.7084411	-1.0041348	0.30949105	-3.2444713	0.00117669	0.09825352	Gpam	34.2743627	30.7716129	31.4898244	43.4309402	18.3129289	12.9365998	17.32075	9.13051012
Mbp	267.831143	-1.0396865	0.32142561	-3.2346101	0.00121809	0.10057343	Mbp	276.883087	501.848804	263.68752	471.535922	249.721758	114.077289	91.4150694	173.479692
Sox6	64.2685764	0.79454886	0.24573224	3.23339288	0.00122329	0.10057343	Sox6	46.3711967	43.8948007	48.9841713	41.3628002	79.9109626	109.373071	71.2075278	73.044081
Acsf2	9.32469678	-1.2379289	0.38344381	-3.2284493	0.00124463	0.10154687	Acsf2	13.4409266	17.648425	11.1327662	16.5451201	4.99443516	4.7042181	3.84905555	2.28262753
Tmem132c	23.7191265	1.136026	0.35213225	3.22613449	0.00125474	0.10159623	Tmem132c	12.7688802	9.5029981	6.67965972	24.8176801	29.966611	43.5140174	25.981125	36.5220405
Laptm4a	76.327033	-1.0324851	0.32047186	-3.2217652	0.00127403	0.10195287	Laptm4a	61.8282622	159.288349	129.776246	80.6574604	41.620293	52.9224536	54.8490416	29.6741579
Nde1	6.57008333	-1.3321494	0.41360516	-3.2208239	0.00127823	0.10195287	Nde1	7.39250961	20.3635674	9.224292	8.27256004	0	1.17605452	3.84905555	2.28262753
Sod3	6.45144124	-1.3365986	0.41529129	-3.2184606	0.00128881	0.10203531	Sod3	10.0806949	15.3858064	16.5401098	2.06814001	3.32962344	0	1.92452778	2.28262753
Olig1	67.5135166	-1.2518838	0.38941996	-3.2147396	0.00130563	0.10260707	Olig1	67.8766792	87.7896015	54.7095939	213.018421	46.6147282	9.40843619	17.32075	43.3699231
Rxrg	18.8136148	-1.161938	0.36184534	-3.2111453	0.00132207	0.10314082	Rxrg	27.5538995	16.7433776	26.4005599	43.4309402	13.3184938	11.7605452	6.73584722	4.56525506
Nxph4	5.8429524	-1.3324221	0.41613446	-3.2019028	0.00136523	0.1050038	Nxph4	4.03227797	18.5534725	4.77118552	14.4769801	1.66481172	0	0.96226389	2.28262753
Asrgl1	61.7936547	-0.9052918	0.28274243	-3.2018249	0.0013656	0.1050038	Asrgl1	100.806949	86.884554	66.7965972	80.6574604	43.2851047	31.7534722	56.7735694	27.3915304
Scd2	748.843208	-0.982353	0.30771231	-3.1924397	0.00141076	0.10736165	Scd2	729.170266	1622.29753	981.5919	816.915304	655.935818	528.048481	291.565958	365.220405
Lxn	26.2975113	-1.1072899	0.34712939	-3.1898477	0.00142348	0.10736165	Lxn	52.4196136	42.0847059	32.7621406	26.8858201	19.9777406	8.23238167	21.1698055	6.84788259
Htra1	35.3257874	-1.155413	0.36228304	-3.1892552	0.0014264	0.10736165	Htra1	34.9464091	89.1471726	48.9841713	37.2265202	31.6314227	19.9929269	11.5471667	9.13051012
Hmgcs2	4.08470877	-1.3244608	0.41561328	-3.1867625	0.00143875	NA	Hmgcs2	7.39250961	8.59795066	6.67965972	6.20442003	1.66481172	1.17605452	0.96226389	0
Adrbk2	109.107929	0.80555805	0.25300221	3.1839961	0.00145257	0.10781188	Adrbk2	77.9573741	53.8503226	69.0231505	109.611421	126.525691	141.126543	157.811278	136.957652
Bhlhe40	64.8131001	-0.9313281	0.29260675	-3.182866	0.00145825	0.10781188	Bhlhe40	131.049034	79.1916508	86.5174974	55.8397803	43.2851047	43.5140174	47.1509305	31.9567854
Sh3bgrl3	85.9583362	-0.9249864	0.29069383	-3.1819952	0.00146264	0.10781188	Sh3bgrl3	152.554517	130.779355	94.4694732	86.8618804	48.2795399	35.2816357	66.3962083	73.044081
Otud7a	32.2876536	0.90522491	0.28559621	3.16959705	0.0015265	0.11174851	Otud7a	24.1936678	21.7211385	18.766663	18.6132601	59.9332219	36.4576902	37.5282917	41.0872956
Bmp7	8.18311432	-1.2723653	0.40307926	-3.1566132	0.00159613	0.11605053	Bmp7	12.0968339	18.5534725	12.0870033	10.3407001	8.3240586	1.17605452	2.88679167	0
Wwtr1	8.56396927	-1.2342492	0.39169751	-3.1510264	0.00162698	0.1174942	Wwtr1	16.8011582	14.0282353	11.4508452	12.4088401	6.65924688	2.35210905	4.81131944	0
Sertm1	51.4243192	0.77054696	0.24504901	3.14446064	0.00166393	0.1192969	Sertm1	30.2420848	38.9170398	39.4418003	37.2265202	58.2684102	62.3308898	67.3584722	77.6093361
Chd3	450.889361	0.75716447	0.24102968	3.1413744	0.00168157	0.1192969	Chd3	312.501543	303.643415	284.998815	397.082882	764.14858	563.330117	399.339514	582.07002
Ppp1r9a	106.986568	0.85418026	0.27226436	3.13731942	0.001705	0.1192969	Ppp1r9a	66.5325865	60.6381783	76.3389683	86.8618804	216.425524	124.66178	112.584875	111.848749
Scara3	11.1538011	-1.3054954	0.41612513	-3.1372665	0.00170531	0.1192969	Scara3	19.4893435	39.3695635	18.130505	0	3.32962344	4.7042181	1.92452778	2.28262753
Reep3	13.8810706	-1.2374553	0.39449012	-3.1368475	0.00170775	0.1192969	Reep3	14.1129729	35.7493738	29.2632712	8.27256004	8.3240586	8.23238167	4.81131944	2.28262753
Calb1	296.536926	1.12350836	0.35887143	3.13067091	0.00174407	0.12066739	Calb1	168.683628	136.209639	201.02595	115.815841	141.508996	676.231351	654.339444	278.480559
Hist1h2bc	14.3954564	-1.2134213	0.38771377	-3.1296834	0.00174995	0.12066739	Hist1h2bc	32.2582238	28.0564706	18.766663	10.3407001	4.99443516	2.35210905	11.5471667	6.84788259
Rgma	36.6007632	-1.1308317	0.36155782	-3.127665	0.00176201	0.12072019	Rgma	49.7314283	71.9512713	40.0779583	55.8397803	39.9554813	15.2887088	15.3962222	4.56525506
Rbm25	96.5431142	0.97692474	0.31257161	3.12544295	0.00177538	0.1208612	Rbm25	65.8605402	44.3473245	59.4807795	66.1804803	114.872009	114.077289	76.9811111	230.545381
Jam2	21.1256303	-1.241829	0.39761817	-3.1231697	0.00178915	0.12102776	Jam2	26.8818531	60.6381783	34.3525357	12.4088401	9.98887032	17.6408179	4.81131944	2.28262753
Pllp	17.8208891	-1.1703226	0.37501238	-3.1207572	0.00180387	0.12125616	Pllp	16.1291119	32.129184	20.6751372	39.2946602	9.98887032	2.35210905	10.5849028	11.4131377
Car2	56.7498258	-1.045612	0.33555586	-3.1160594	0.00183285	0.12243461	Car2	97.4467176	96.3875521	88.4259716	41.3628002	36.6258578	17.6408179	50.9999861	25.1089028
Nkain4	7.63301637	-1.280811	0.41232183	-3.1063381	0.0018942	0.12574666	Nkain4	7.39250961	12.2181404	9.86045007	22.7495401	4.99443516	0	3.84905555	0
Fzd2	7.19056061	-1.2885484	0.41521481	-3.1033296	0.00191356	0.12624799	Fzd2	4.03227797	22.1736622	10.8146872	12.4088401	4.99443516	1.17605452	1.92452778	0
Smox	15.9540839	-1.1234049	0.36612735	-3.0683445	0.00215248	0.1411395	Smox	20.1613899	23.0787097	13.9954775	39.2946602	8.3240586	8.23238167	7.69811111	6.84788259
Serinc5	21.1957417	-1.1523054	0.37585027	-3.0658627	0.00217043	0.14144846	Serinc5	16.8011582	56.5654649	28.6271131	24.8176801	14.9833055	10.5844907	5.77358333	11.4131377
Sardh	5.68593079	-1.2710483	0.41508082	-3.0621706	0.00219738	0.14233705	Sardh	8.06455594	7.2403795	10.4966081	12.4088401	4.99443516	0	0	2.28262753
BC026585	3.55186095	-1.2573898	0.41158247	-3.055013	0.00225051	NA	BC026585	6.72046328	3.16766603	4.45310648	12.4088401	1.66481172	0	0	0
Ctss	8.1387295	-1.2675276	0.41500736	-3.0542292	0.0022564	0.14527928	Ctss	8.73660227	25.793852	18.766663	2.06814001	3.32962344	3.52816357	2.88679167	0
Btbd11	62.7480374	0.91629121	0.30042684	3.04996451	0.00228868	0.14647578	Btbd11	26.8818531	32.5817078	34.0344567	72.3849004	83.240586	72.9153805	81.7924305	98.1529838
Pttg1ip	15.8210201	-1.2081602	0.39694452	-3.0436499	0.00233727	0.14682131	Pttg1ip	14.7850192	47.5149905	20.6751372	16.5451201	6.65924688	12.9365998	2.88679167	4.56525506
Tril	32.1015182	-1.1818098	0.38834255	-3.0432147	0.00234065	0.14682131	Tril	29.5700384	68.3310816	41.6683535	57.9079203	36.6258578	5.88027262	7.69811111	9.13051012
Tprkb	42.386813	-0.9196111	0.30249639	-3.040073	0.00236521	0.14682131	Tprkb	65.1884939	64.7108918	51.5288036	51.7035003	18.3129289	27.0492541	42.3396111	18.2610202
Socs5	105.923195	0.67463371	0.22199123	3.03901062	0.00237356	0.14682131	Socs5	68.5487255	67.4260341	90.6525248	93.0663005	124.860879	129.365998	125.094306	148.37079
Cd151	8.49667004	-1.2382557	0.40748606	-3.0387684	0.00237547	0.14682131	Cd151	5.37637063	15.3858064	14.6316356	20.6814001	6.65924688	2.35210905	2.88679167	0
Nrarp	23.3641569	-1.1224685	0.36946111	-3.0381236	0.00238056	0.14682131	Nrarp	31.5861774	28.5089943	27.0367179	53.7716403	18.3129289	3.52816357	17.32075	6.84788259
Mafb	54.2661714	1.08705405	0.3579485	3.03690067	0.00239024	0.14682131	Mafb	21.5054825	16.2908539	25.7644018	55.8397803	79.9109626	55.2745626	47.1509305	132.392397
Rorb	47.0771856	-0.9688176	0.31984047	-3.029065	0.00245312	0.14982249	Rorb	79.9735131	69.236129	48.9841713	66.1804803	33.2962344	29.4013631	40.4150833	9.13051012
Btn2a2	2.61679251	1.22008962	0.40279662	3.02904629	0.00245327	NA	Btn2a2	0	0.45252372	0.63615807	0	1.66481172	9.40843619	1.92452778	6.84788259
Ramp1	24.2371654	-1.0976952	0.36302721	-3.023727	0.00249682	0.15155508	Ramp1	24.8657142	38.9170398	23.2197695	55.8397803	21.6425524	7.05632714	8.660375	13.6957652
Prkcdbp	7.58180433	-1.2243591	0.40512827	-3.0221517	0.00250985	0.15155508	Prkcdbp	15.4570656	13.1231878	6.04350166	14.4769801	4.99443516	2.35210905	1.92452778	2.28262753
Pcdh8	50.8660269	1.0953636	0.36332085	3.01486579	0.00257093	0.15437134	Pcdh8	17.4732045	34.3918026	38.8056422	14.4769801	103.218327	97.6125255	27.9056528	73.044081
Maf	31.4543983	0.97157956	0.32245137	3.01310415	0.0025859	0.15440288	Maf	20.1613899	14.9332827	16.2220308	26.8858201	23.3073641	43.5140174	40.4150833	66.1961984
Atp1a2	373.055419	-1.0436796	0.34713896	-3.006518	0.00264258	0.15663241	Atp1a2	415.996677	819.067931	507.654139	397.082882	406.21406	143.478652	107.773556	187.175458
Efhd1	11.0004339	-1.2413288	0.41303649	-3.0053733	0.00265255	0.15663241	Efhd1	10.0806949	15.3858064	10.4966081	37.2265202	8.3240586	0	1.92452778	4.56525506
Hist1h4d	79.6273989	-0.981766	0.32719409	-3.0005615	0.00269482	0.15825426	Hist1h4d	117.608107	95.9350284	88.1078926	142.701661	38.2906696	19.9929269	56.7735694	77.6093361
Shisa4	56.8809066	-1.0124782	0.33775864	-2.9976383	0.0027208	0.15890684	Shisa4	58.4680306	114.035977	78.2474425	68.2486203	44.9499164	16.4647633	22.1320694	52.5004332
Tmem179b	3.08292246	-1.2254643	0.40976721	-2.9906353	0.00278398	NA	Tmem179b	2.01613899	5.88280835	7.31581779	8.27256004	0	1.17605452	0	0
Egr1	181.251812	-0.8972824	0.30033368	-2.9876184	0.0028116	0.16259166	Egr1	344.08772	190.512486	255.099386	186.132601	114.872009	89.3801438	82.7546944	187.175458
5031439G07Rik	68.7672525	-0.9480457	0.31743432	-2.9865884	0.00282109	0.16259166	5031439G07R	77.9573741	90.9572675	63.6158069	146.837941	49.9443516	25.8731995	35.6037639	59.3483158
Myrf	19.4012656	-1.1652136	0.39026809	-2.9856749	0.00282953	0.16259166	Myrf	10.7527413	51.1351802	26.0824808	31.0221002	11.653682	3.52816357	9.62263889	11.4131377
C530008M17Rik	156.106961	0.95947925	0.32163399	2.98314008	0.00285307	0.16266272	C530008M17R	76.6132814	76.0239848	82.3824699	150.974221	154.82749	255.203832	124.132042	328.698364
Slc17a6	28.3357106	-1.1368076	0.38118888	-2.9822687	0.00286121	0.16266272	Slc17a6	28.8979921	74.2138899	41.9864326	26.8858201	18.3129289	23.5210905	10.5849028	2.28262753
B2m	43.6490359	-1.1211317	0.37731509	-2.9713409	0.00296502	0.16767295	B2m	45.027104	117.203643	65.2062021	35.1583802	8.3240586	37.6337448	26.9433889	13.6957652
Pla2g7	45.139373	-1.1877718	0.40075543	-2.9638321	0.00303834	0.17091463	Pla2g7	64.5164475	127.159165	82.3824699	10.3407001	14.9833055	35.2816357	17.32075	9.13051012
Slc13a3	10.2005775	-1.2243871	0.41433452	-2.955069	0.00312599	0.17492456	Slc13a3	8.06455594	24.4362808	18.766663	16.5451201	11.653682	1.17605452	0.96226389	0
Smim3	3.59206881	-1.2186953	0.41327632	-2.948863	0.00318945	NA	Smim3	3.36023164	10.4080455	4.77118552	8.27256004	0	0	1.92452778	0
Gramd3	10.1220455	-1.1043376	0.37455238	-2.9484195	0.00319403	0.17780116	Gramd3	15.4570656	17.1959013	16.5401098	10.3407001	8.3240586	4.7042181	3.84905555	4.56525506
Plekho2	12.6692725	-1.1253955	0.38214984	-2.9449064	0.00323052	0.17890069	Plekho2	17.4732045	16.2908539	11.1327662	33.0902402	8.3240586	4.7042181	5.77358333	4.56525506
Ebf1	3.34198491	-1.223289	0.41582839	-2.9418122	0.00326298	NA	Ebf1	8.06455594	5.88280835	5.40734359	4.13628002	0	0	0.96226389	2.28262753
Htatip2	3.25707752	1.22363376	0.4159522	2.94176534	0.00326347	NA	Htatip2	1.34409266	1.35757116	0.63615807	0	3.32962344	9.40843619	7.69811111	2.28262753
Satb1	106.180495	0.78689745	0.26754588	2.9411682	0.00326977	0.1792221	Satb1	93.4144396	59.2806072	84.6090232	59.9760603	99.8887032	162.295524	171.282972	118.696632
Npy	185.838726	0.81521087	0.27717332	2.9411592	0.00326986	0.1792221	Npy	114.247876	124.896546	143.771724	126.156541	171.475607	297.541795	161.660333	346.959385
Rin2	14.3013673	-1.0312251	0.351259	-2.935797	0.00332692	0.18141894	Rin2	14.7850192	24.4362808	20.9932163	22.7495401	9.98887032	8.23238167	8.660375	4.56525506
Tmem100	7.07917817	-1.2042077	0.41044562	-2.9339032	0.00334729	0.18160299	Tmem100	13.4409266	19.4585199	10.8146872	2.06814001	3.32962344	2.35210905	2.88679167	2.28262753
Acsbg1	42.6025982	-1.0269041	0.35116441	-2.9242831	0.00345251	0.18636559	Acsbg1	73.2530498	51.587704	45.803381	74.4530404	48.2795399	12.9365998	23.0943333	11.4131377
Cmtm3	4.30630625	-1.211876	0.41614568	-2.9121436	0.00358958	NA	Cmtm3	4.7043243	11.313093	7.95197586	6.20442003	0	2.35210905	1.92452778	0
Mtus1	26.3963322	-0.9787257	0.3364291	-2.9091589	0.00362403	0.19406061	Mtus1	38.3066407	48.4200379	43.2587487	16.5451201	14.9833055	21.1689814	12.5094306	15.9783927
Ucp2	33.2199289	-1.1954848	0.41118776	-2.907394	0.00364454	0.19406061	Ucp2	14.7850192	41.1796584	21.9474534	138.565381	14.9833055	3.52816357	12.5094306	18.2610202
4930413G21Rik	8.76176707	1.20775695	0.41546995	2.90696584	0.00364953	0.19406061	4930413G21R	2.01613899	0.90504744	0.9542371	6.20442003	21.6425524	3.52816357	2.88679167	31.9567854
Spop	69.472764	0.92588935	0.31885451	2.90379884	0.00368665	0.19506391	Spop	38.978687	29.8665654	33.0802196	76.5211804	119.866444	59.9787807	85.6414861	111.848749
Lcat	9.31745548	-1.2019877	0.41556135	-2.8924433	0.00382258	0.20125985	Lcat	16.1291119	27.1514231	14.9497146	4.13628002	8.3240586	0	3.84905555	0
F3	13.1358544	-1.1819107	0.40934686	-2.8873086	0.00388553	0.20320025	F3	11.4247876	39.8220873	27.672876	6.20442003	4.99443516	8.23238167	6.73584722	0
Haus8	4.74914269	1.20149434	0.41629043	2.88619254	0.00389934	0.20320025	Haus8	0	1.81009488	0.9542371	4.13628002	4.99443516	4.7042181	7.69811111	13.6957652
Bicd2	57.6683867	0.70860582	0.24563326	2.88481222	0.00391647	0.20320025	Bicd2	47.043243	34.8443264	37.8514051	51.7035003	76.5813391	67.0351079	66.3962083	79.8919636
Serpine2	70.1732517	-1.0452124	0.36300397	-2.8793415	0.00398507	0.20576027	Serpine2	73.2530498	180.10444	108.78303	45.4990802	48.2795399	52.9224536	36.5660278	15.9783927
Dlgap1	397.039017	0.68097052	0.23695367	2.8738551	0.00405495	0.20836202	Dlgap1	372.985712	297.760607	322.214062	188.200741	512.76201	560.978008	458.037611	463.373389
Prex2	46.4488068	-1.0179276	0.35470268	-2.8698051	0.00410725	0.20955798	Prex2	72.5810035	98.197647	66.1604392	28.9539601	43.2851047	21.1689814	29.8301805	11.4131377
Stx4a	11.9207729	1.0484797	0.36544853	2.86902151	0.00411744	0.20955798	Stx4a	8.06455594	4.52523719	4.77118552	10.3407001	21.6425524	11.7605452	18.2830139	15.9783927
Ptch1	49.0250404	-0.9276962	0.32369853	-2.8659266	0.00415791	0.21061473	Ptch1	42.3389187	80.549222	46.7576181	101.338861	31.6314227	36.4576902	21.1698055	31.9567854
Elovl1	3.17099484	-1.1561632	0.40348208	-2.8654634	0.00416399	NA	Elovl1	2.68818531	14.0282353	5.40734359	2.06814001	0	1.17605452	0	0
Rpp25	38.7280219	0.87329223	0.30542598	2.85925981	0.00424631	0.21407804	Rpp25	31.5861774	23.9837571	23.2197695	20.6814001	48.2795399	30.5774176	53.8867778	77.6093361
Wdr83os	24.9896084	-0.9011679	0.31604096	-2.8514275	0.00435234	0.21839353	Wdr83os	29.5700384	30.3190892	28.9451921	47.5672202	13.3184938	12.9365998	14.4339583	22.8262753
Dazap2	87.3093066	-0.6571832	0.23061804	-2.849661	0.00437658	0.21858383	Dazap2	99.4628566	116.751119	113.554215	103.407001	49.9443516	62.3308898	73.1320555	79.8919636
Gng5	3.16458411	-1.1821598	0.41526723	-2.8467449	0.00441687	NA	Gng5	4.7043243	5.43028463	4.77118552	8.27256004	0	1.17605452	0.96226389	0

GeneID	Base mean	log2(FC)	StdErr	Wald-Stats	P-value	P-adj	GeneID	Normalised expression for Chow#1	Normalised expression for Chow#2	Normalised expression for Chow#3	Normalised expression for Chow#4	Normalised expression for HFD#1	Normalised expression for HFD#2	Normalised expression for HFD#3	Normalised expression for HFD#4
Pmp22	6.2380643	-1.1576077	0.40667334	-2.8465296	0.00441986	0.21971851	Pmp22	8.73660227	13.5757116	10.1785291	6.20442003	3.32962344	2.35210905	0.96226389	4.56525506
0610031J06Rik	18.2236699	-1.0443788	0.3672287	-2.8439466	0.00445585	0.22048217	0610031J06Ri	15.4570656	32.129184	25.7644018	33.0902402	6.65924688	16.4647633	4.81131944	11.4131377
Fmod	9.39217817	-1.1808126	0.41636175	-2.8360256	0.00456788	0.22477916	Fmod	13.4409266	36.6544212	13.3593194	0	1.66481172	3.52816357	1.92452778	4.56525506
Dgkg	133.507741	0.70114072	0.24732924	2.83484764	0.00458475	0.22477916	Dgkg	106.855366	103.627932	110.691504	70.3167603	123.196067	199.929269	198.226361	155.218672
Zfp503	5.00998882	-1.163058	0.41062828	-2.8323865	0.0046202	0.22548248	Zfp503	4.7043243	4.52523719	4.77118552	22.7495401	3.32962344	0	0	0
Alk	15.4808126	1.06630153	0.37668518	2.83074989	0.0046439	0.2256092	Alk	5.37637063	5.88280835	7.31581779	16.5451201	21.6425524	37.6337448	13.4716944	15.9783927
Tmem44	42.2330669	0.71366818	0.25269491	2.82422857	0.00473946	0.22920959	Tmem44	33.6023164	26.2463757	32.7621406	31.0221002	46.6147282	57.6266717	52.9245139	57.0656883
E130114P18Rik	2.00526339	-1.1113077	0.393843	-2.8217024	0.00477695	NA	E130114P18Ri	6.72046328	4.07271347	3.18079035	2.06814001	0	0	0	0
Slc14a1	8.21993727	-1.137157	0.40363259	-2.8173072	0.00484282	0.23229929	Slc14a1	7.39250961	17.1959013	16.2220308	10.3407001	6.65924688	4.7042181	0.96226389	2.28262753
Rxfp3	11.0891058	0.98466346	0.34956783	2.81680228	0.00485044	0.23229929	Rxfp3	6.72046328	5.88280835	6.99773876	6.20442003	18.3129289	16.4647633	14.4339583	13.6957652
Csrnp2	30.2914019	0.873806	0.31034395	2.81560503	0.00486855	0.23229929	Csrnp2	14.1129729	19.9110436	26.0824808	16.5451201	53.2739751	31.7534722	32.7169722	47.9351782
Mt3	99.1292764	-0.9734023	0.34663162	-2.8081751	0.00498231	0.23610139	Mt3	135.081312	111.320835	98.2864217	213.018421	74.9165274	23.5210905	43.301875	93.5877288
Vamp3	6.25716252	-1.1554725	0.41156299	-2.8075229	0.00499241	0.23610139	Vamp3	8.06455594	15.8383302	9.54237104	8.27256004	0	3.52816357	4.81131944	0
Rbms3	51.1737504	0.87142998	0.31099486	2.802072	0.00507755	0.23907003	Rbms3	22.1775288	27.6039469	42.6225906	43.4309402	43.2851047	84.6759257	88.5282777	57.0656883
Lhx6	132.296551	0.79903582	0.28533391	2.8003535	0.00510467	0.23929247	Lhx6	71.2369108	61.0907021	100.831054	134.429101	163.151549	148.18287	144.339583	235.110636
Sptbn1	169.107584	0.91336912	0.32659044	2.79668047	0.00516306	0.23972124	Sptbn1	122.984478	110.868311	115.14461	74.4530404	404.549248	197.57716	222.282958	105.000866
Tmed8	75.1528917	0.63855297	0.22839987	2.7957677	0.00517766	0.23972124	Tmed8	45.6991503	60.6381783	62.3434908	59.9760603	99.8887032	84.6759257	87.5660139	100.435611
S100a10	11.499912	-1.0526319	0.37653799	-2.795553	0.0051811	0.23972124	S100a10	20.8334362	13.5757116	12.4050823	22.7495401	3.32962344	5.88027262	8.660375	4.56525506
Lpcat3	11.5086694	-1.0634949	0.38062777	-2.794055	0.00520516	0.23979648	Lpcat3	8.73660227	23.0787097	20.3570582	16.5451201	4.99443516	7.05632714	6.73584722	4.56525506
Smo	10.6647454	-1.0852681	0.38913594	-2.7889176	0.00528845	0.2412141	Smo	11.4247876	24.4362808	12.0870033	16.5451201	9.98887032	4.7042181	3.84905555	2.28262753
Afap1	31.0113277	0.8199414	0.29404743	2.78846645	0.00529582	0.2412141	Afap1	20.1613899	18.5534725	19.4028211	26.8858201	29.966611	35.2816357	38.4905555	59.3483158
Adora1	91.8148794	-0.8233394	0.29531662	-2.7879886	0.00530364	0.2412141	Adora1	110.887644	159.288349	109.737267	103.407001	98.2238915	39.9858538	49.0754583	63.9135709
Sfxn5	120.58059	-0.9225235	0.33143929	-2.7833861	0.00537947	0.24230534	Sfxn5	147.178146	157.02573	120.551954	239.904241	129.855314	41.1619083	49.0754583	79.8919636
Dbi	25.7700018	-0.9959062	0.3579884	-2.7819511	0.00540332	0.24230534	Dbi	36.2905017	25.3413283	34.0344567	53.7716403	9.98887032	7.05632714	25.981125	13.6957652
Ttr	30.4233253	1.02861193	0.36978149	2.78167498	0.00540792	0.24230534	Ttr	0.67204633	0.45252372	0.9542371	0	1.66481172	3.52816357	135.679208	100.435611
Epb4.1l4a	10.0086162	1.08481361	0.39007321	2.7810513	0.00541832	0.24230534	Epb4.1l4a	2.01613899	4.97776091	3.18079035	12.4088401	13.3184938	14.1126543	16.3584861	13.6957652
Nhsl1	21.9591983	-0.8765434	0.31549857	-2.7782802	0.00546475	0.2433634	Nhsl1	30.2420848	37.5594687	26.4005599	24.8176801	14.9833055	12.9365998	17.32075	11.4131377
Moxd1	37.887419	1.06007436	0.38286726	2.76877779	0.0056267	0.24861755	Moxd1	19.4893435	24.4362808	26.0824808	6.20442003	33.2962344	116.429398	26.9433889	50.2178057
Stxbp6	143.394765	0.70868358	0.25596904	2.76863001	0.00562925	0.24861755	Stxbp6	100.806949	93.6724098	114.826531	111.679561	111.542385	238.739068	181.867875	194.02334
S100a13	6.29183364	-1.1164308	0.40551642	-2.7531088	0.00590323	0.2596448	S100a13	10.0806949	14.0282353	7.31581779	8.27256004	3.32962344	1.17605452	3.84905555	2.28262753
Panx1	10.6602936	-1.0044497	0.3654699	-2.7483786	0.00598908	0.26234139	Panx1	14.7850192	15.3858064	16.2220308	14.4769801	6.65924688	7.05632714	3.84905555	6.84788259
Luzp2	93.9257096	-0.9933311	0.36217006	-2.7427201	0.00609326	0.26554113	Luzp2	144.489961	217.211385	131.048562	45.4990802	38.2906696	78.7956531	66.3962083	29.6741579
Tnfrsf1a	4.72441589	-1.14154	0.41635887	-2.7417212	0.00611182	0.26554113	Tnfrsf1a	4.03227797	14.9332827	5.08926455	8.27256004	3.32962344	1.17605452	0.96226389	0
Gm14204	22.1237503	0.862693	0.31489167	2.73965016	0.00615046	0.26613818	Gm14204	12.0968339	14.0282353	17.4943469	12.4088401	33.2962344	19.9929269	28.8679167	38.804668
Rims4	20.1949448	1.08473342	0.3967826	2.73382305	0.00626037	0.26980166	Rims4	6.04841696	6.78785578	6.67965972	20.6814001	44.9499164	11.7605452	14.4339583	50.2178057
Meg3	151.483382	1.08908426	0.39881716	2.73078586	0.00631835	0.27120698	Meg3	18.8172972	49.3250854	30.5355873	190.268881	327.967909	197.57716	164.547125	232.828008
Ddah1	110.374374	-0.6061882	0.22213753	-2.7288868	0.00635485	0.2716826	Ddah1	149.194285	145.712637	134.229353	109.611421	89.8998329	71.739326	98.1509166	84.4572186
Hist1h4c	36.870246	-0.8263014	0.30347698	-2.7227813	0.00647349	0.27333998	Hist1h4c	49.7314283	43.442277	43.8949068	59.9760603	18.3129289	15.2887088	34.6415	29.6741579
Gng12	36.2747506	-0.9446967	0.34733712	-2.7198265	0.00653162	0.27333998	Gng12	43.6830113	78.2866034	49.9384084	31.0221002	21.6425524	11.7605452	35.6037639	18.2610202
Kif5a	907.786533	0.67499315	0.24820336	2.71951656	0.00653774	0.27333998	Kif5a	814.52015	511.804326	602.75977	783.825064	1450.05101	985.533691	1269.22607	844.572186
Rbp4	14.6504359	1.00955388	0.37130803	2.71891202	0.0065497	0.27333998	Rbp4	9.4086486	9.05047438	8.2700549	4.13628002	6.65924688	22.345036	23.0943333	34.239413
Cit	100.656937	0.59333306	0.21823464	2.71878496	0.00655222	0.27333998	Cit	76.6132814	79.6441745	74.4304941	86.8618804	113.207197	150.534979	121.24525	102.718239
Pdyn	9.46956283	-1.0216313	0.37599003	-2.7171765	0.00658415	0.27333998	Pdyn	12.7688802	16.7433776	14.9497146	10.3407001	6.65924688	5.88027262	3.84905555	4.56525506
Klhl32	14.0691723	1.0212335	0.37584805	2.71714459	0.00658478	0.27333998	Klhl32	5.37637063	6.33533207	6.99773876	12.4088401	21.6425524	8.23238167	17.32075	34.239413
Akap2	52.0852611	0.83260624	0.30650299	2.71647016	0.00659821	0.27333998	Akap2	31.5861774	25.3413283	33.0802196	51.7035003	98.2238915	68.2111624	42.3396111	66.1961984
Zfyve21	5.75100725	-1.129292	0.41598817	-2.7147216	0.00663315	0.27351243	Zfyve21	7.39250961	5.88280835	5.40734359	20.6814001	3.32962344	2.35210905	0.96226389	0
Hnrnpul2	158.406561	0.63331796	0.23337768	2.71370402	0.00665356	0.27351243	Hnrnpul2	126.34471	96.3875521	119.279638	146.837941	163.151549	244.619341	203.999944	166.63181
Pou3f2	12.6361183	-0.969926	0.35802873	-2.7090731	0.00674715	0.27629695	Pou3f2	16.8011582	18.1009488	16.2220308	20.6814001	3.32962344	5.88027262	8.660375	11.4131377
Traf3ip1	42.9351288	1.12288761	0.41477589	2.70721522	0.00678503	0.27678761	Traf3ip1	10.0806949	6.33533207	8.58813393	41.3628002	74.9165274	17.6408179	20.2075417	164.349182
Pde8a	3.38565782	-1.1260652	0.41622892	-2.7053988	0.00682224	NA	Pde8a	8.06455594	6.78785578	6.36158069	2.06814001	1.66481172	1.17605452	0.96226389	0
Cdh6	3.27935818	-1.1089091	0.41026963	-2.7028788	0.00687418	NA	Cdh6	6.04841696	11.313093	4.45310648	2.06814001	0	2.35210905	0	0
Vtn	10.6207847	-1.1172905	0.41590257	-2.6864237	0.00722214	0.29349917	Vtn	4.7043243	34.8443264	21.6293743	10.3407001	9.98887032	1.17605452	0	2.28262753
Aldoart1	111.161034	0.56889462	0.21193067	2.68434299	0.00726725	0.29421355	Aldoart1	106.18332	76.9290322	87.1536555	80.6574604	138.179373	124.66178	138.566	136.957652
D10Bwg1379e	115.559968	0.64844621	0.24191071	2.68051884	0.00735081	0.29647353	D10Bwg1379e	86.6939764	75.5714611	82.700549	107.543281	194.782971	131.718107	122.207514	123.261887
Gstm5	67.1440264	-0.6757118	0.25254906	-2.6755666	0.00746031	0.29975845	Gstm5	94.7585323	96.3875521	81.7463119	64.1123403	51.6091633	45.8661264	61.5848889	41.0872956
Fam213a	49.9684557	-1.0004643	0.37514805	-2.6668519	0.00765654	0.306491	Fam213a	76.6132814	120.823833	69.9773876	22.7495401	19.9777406	47.042181	28.8679167	13.6957652
Rian	82.3673	0.80529341	0.30250247	2.66210526	0.00776536	0.30776179	Rian	46.3711967	38.0119924	47.3937761	97.2025805	123.196067	76.4435441	111.622611	118.696632
Lhfp	9.48871998	-1.0885787	0.4089967	-2.6615831	0.00777741	0.30776179	Lhfp	4.7043243	22.1736622	10.1785291	22.7495401	1.66481172	4.7042181	2.88679167	6.84788259
Rhoc	5.09177411	-1.105889	0.41553928	-2.6613343	0.00778316	0.30776179	Rhoc	11.4247876	6.33533207	4.13502745	12.4088401	3.32962344	1.17605452	1.92452778	0
Folh1	4.00769959	-1.1070381	0.41602483	-2.6609904	0.00779112	NA	Folh1	6.04841696	12.6706641	6.99773876	2.06814001	0	2.35210905	1.92452778	0
Als2	38.297175	0.70917052	0.26655965	2.66045713	0.00780347	0.30776179	Als2	27.5538995	25.3413283	30.2175083	28.9539601	44.9499164	62.3308898	41.3773472	45.6525506
Psat1	34.0961586	-0.7383795	0.2781387	-2.654717	0.0079375	0.31132332	Psat1	49.7314283	38.0119924	43.2587487	43.4309402	19.9777406	22.345036	24.0565972	31.9567854
Wipf1	10.3064283	-1.0295798	0.38792051	-2.6540999	0.00795203	0.31132332	Wipf1	11.4247876	23.9837571	13.3593194	12.4088401	6.65924688	2.35210905	7.69811111	4.56525506
Sart3	32.0044964	1.00271622	0.37818481	2.6513921	0.00801607	0.31268533	Sart3	16.8011582	9.5029981	13.6773985	31.0221002	46.6147282	28.2253086	21.1698055	89.0224737
Myo16	48.8067361	0.726861	0.27460181	2.64696366	0.00812181	0.31565776	Myo16	38.978687	34.3918026	39.1237212	24.8176801	61.5980337	72.9153805	43.301875	75.3267085
Safb2	14.0581612	0.92161088	0.34851681	2.64438001	0.00818407	0.31692522	Safb2	8.73660227	8.14542694	7.31581779	10.3407001	28.3017992	11.7605452	17.32075	20.5436478
Ppp1r26	38.5108286	0.79397429	0.3007153	2.64028565	0.00828362	0.31962204	Ppp1r26	23.5216215	20.8160911	20.0389792	45.4990802	54.9387868	49.39429	41.3773472	52.5004332
Cd81	196.669341	-0.830258	0.31485737	-2.6369337	0.00836592	0.32034515	Cd81	156.586795	395.958254	228.062668	266.790061	166.481172	109.373071	78.9056389	171.197065
Paqr6	9.60646041	-1.047543	0.39729701	-2.6366748	0.00837231	0.32034515	Paqr6	2.01613899	5.43028463	3.81694841	59.9760603	3.32962344	0	0	2.28262753
Bcl2l2	123.168893	0.68741793	0.26085784	2.63522056	0.00840826	0.32034515	Bcl2l2	88.038069	82.8118406	96.0598684	95.1344405	179.799666	98.78858	134.716944	210.001733
Cyp2j9	12.362335	-1.0386026	0.39425459	-2.6343451	0.00842998	0.32034515	Cyp2j9	12.0968339	36.6544212	16.8581888	8.27256004	6.65924688	7.05632714	6.73584722	4.56525506
Pcp4	125.009777	-1.0768941	0.40892897	-2.6334503	0.00845222	0.32034515	Pcp4	334.007025	185.082201	233.151932	28.9539601	11.653682	94.0843619	78.9056389	34.239413
Sdc4	28.8136464	-1.0203717	0.3881685	-2.6286824	0.00857164	0.32279968	Sdc4	18.8172972	68.7836053	36.579089	45.4990802	31.6314227	5.88027262	9.62263889	13.6957652
2900097C17Rik	610.877508	0.5941192	0.22603368	2.62845435	0.00857739	0.32279968	2900097C17Ri	511.427256	440.305579	500.6564	454.990802	739.176404	680.935569	1009.41482	550.113235
Ctso	11.8657259	-1.0351453	0.39446746	-2.624159	0.00868632	0.32575234	Ctso	14.7850192	28.0564706	14.9497146	14.4769801	1.66481172	12.9365998	5.77358333	2.28262753
Akap8l	19.3242214	0.9139085	0.34870205	2.62088654	0.00877014	0.32623416	Akap8l	11.4247876	7.69290322	11.1327662	18.6132601	39.9554813	15.2887088	23.0943333	27.3915304
F2rl1	1.65785473	0.94088316	0.35900514	2.62080694	0.00877219	NA	F2rl1	0	0	0	0	0	3.52816357	2.88679167	6.84788259
Sorbs3	10.4616293	-1.0229654	0.3903575	-2.6205861	0.00877788	0.32623416	Sorbs3	18.1452509	14.9332827	13.3593194	14.4769801	11.653682	2.35210905	1.92452778	6.84788259
Ppapdc1b	8.11255062	-1.0521726	0.4015793	-2.6200867	0.00879074	0.32623416	Ppapdc1b	8.73660227	11.7656167	12.4050823	18.6132601	3.32962344	2.35210905	7.69811111	0
Gpc5	12.8761691	-0.9273494	0.35458822	-2.6152855	0.00891529	0.32917582	Gpc5	18.1452509	22.6261859	19.0847421	12.4088401	8.3240586	8.23238167	9.62263889	4.56525506
2310022B05Rik	72.8123081	-0.9773335	0.37378973	-2.614661	0.0089316	0.32917582	2310022B05Ri	66.5325865	84.1694117	56.6180681	213.018421	63.2628454	17.6408179	35.6037639	45.6525506
Trpc6	6.4023655	1.082382	0.41481956	2.60928391	0.00907319	0.33324498	Trpc6	2.01613899	4.97776091	2.54463228	0	3.32962344	16.4647633	17.32075	4.56525506
Ltbr	2.33703888	-1.0509258	0.40298101	-2.6078793	0.00911051	NA	Ltbr	3.36023164	7.69290322	2.54463228	4.13628002	0	0	0.96226389	0
Mcf2l	44.8654283	0.68051599	0.26097283	2.60761239	0.00911761	0.33372962	Mcf2l	34.2743627	36.2018975	32.7621406	26.8858201	66.5924688	51.7463991	44.2641389	66.1961984
Elfn1	98.6506482	0.8457889	0.32466217	2.60513538	0.0091838	0.33500484	Elfn1	51.7475673	68.7836053	65.2062021	72.3849004	151.497867	189.344778	50.9999861	139.240279
Kcne1l	3.41030772	-1.0454864	0.40142533	-2.6044355	0.00920258	NA	Kcne1l	3.36023164	3.16766603	2.54463228	16.5451201	1.66481172	0	0	0
Gpr17	23.0429927	-1.0540113	0.40510171	-2.6018437	0.00927241	0.33708683	Gpr17	20.8334362	75.1189373	35.6248519	8.27256004	16.6481172	7.05632714	4.81131944	15.9783927
Gfpt1	97.61457	0.5916561	0.22765955	2.5988635	0.0093533	0.33714583	Gfpt1	70.5648645	63.3533207	83.336707	88.9300204	121.531256	134.070216	98.1509166	120.979259
Tcf4	338.951865	0.65991496	0.25400704	2.59801837	0.00937635	0.33714583	Tcf4	297.044477	271.061708	290.406159	155.110501	342.951214	425.731738	532.13193	397.17719
Kcnq1ot1	6.72711021	1.05805726	0.40743647	2.59686441	0.00940791	0.33714583	Kcnq1ot1	2.68818531	4.97776091	1.90847421	2.06814001	16.6481172	3.52816357	10.5849028	11.4131377
9530036O11Rik	1.73060061	1.00455066	0.38685997	2.5966777	0.00941302	NA	9530036O11R	0	0.45252372	0.31807903	0	6.65924688	3.52816357	2.88679167	0
Grip1	25.1273864	0.75593306	0.29113227	2.59652786	0.00941713	0.33714583	Grip1	16.1291119	18.5534725	20.6751372	14.4769801	38.2906696	34.1055812	33.6792361	25.1089028
Scrn1	128.013193	0.54324621	0.20926323	2.59599457	0.00943176	0.33714583	Scrn1	106.855366	91.8623149	108.78303	103.407001	156.492302	183.464506	138.566	134.675024
Gprc5b	62.8762775	-0.8213868	0.31675308	-2.5931455	0.00951025	0.33762138	Gprc5b	68.5487255	133.947021	81.1101538	49.6353602	53.2739751	48.2182355	31.7547083	36.5220405
Limch1	48.3213476	0.72170781	0.2784358	2.59200794	0.00954176	0.33762138	Limch1	30.9141311	37.5594687	36.579089	35.1583802	81.5757743	67.0351079	63.5094166	34.239413
Slc1a2	1597.99307	-0.9987364	0.38532265	-2.5919483	0.00954341	0.33762138	Slc1a2	1760.08933	4409.84364	2615.5639	577.011063	1080.46281	1090.20254	908.377111	342.39413
Rpl27	7.1918098	-1.0469859	0.40409499	-2.5909401	0.00957141	0.33762138	Rpl27	18.1452509	11.7656167	6.36158069	8.27256004	3.32962344	3.52816357	3.84905555	2.28262753
Ttc9b	57.4333735	-0.7666398	0.29605308	-2.589535	0.00961056	0.33774392	Ttc9b	102.823088	72.403795	57.5723052	64.1123403	51.6091633	27.0492541	33.6792361	50.2178057
Ly6e	105.118982	-0.7746454	0.29925841	-2.5885502	0.00963809	0.33774392	Ly6e	105.511274	198.205389	138.682459	103.407001	89.8998329	45.8661264	63.5094166	95.8703563
Efcab14	34.2707748	-0.9076927	0.35092736	-2.5865545	0.00969408	0.33859601	Efcab14	28.8979921	75.1189373	52.1649617	35.1583802	18.3129289	29.4013631	25.981125	9.13051012
Sft2d2	18.1102392	-0.9760603	0.37770208	-2.5842068	0.00976032	0.33979916	Sft2d2	14.7850192	31.6766603	18.130505	39.2946602	6.65924688	7.05632714	6.73584722	20.5436478
Cd4	4.71031486	-1.0749651	0.41633328	-2.5819822	0.00982347	0.34088701	Cd4	11.4247876	2.71514231	9.54237104	8.27256004	1.66481172	1.17605452	2.88679167	0
Dap	4.4948104	-1.0720443	0.41586033	-2.5778949	0.00994042	NA	Dap	9.4086486	6.78785578	2.86271131	12.4088401	0	3.52816357	0.96226389	0
Zic1	13.0073696	-1.066813	0.41520777	-2.5693475	0.01018902	0.35242807	Zic1	8.73660227	8.14542694	10.1785291	57.9079203	8.3240586	2.35210905	3.84905555	4.56525506
Prelp	16.6263485	-1.0364859	0.40457196	-2.5619322	0.01040916	0.35888114	Prelp	20.1613899	54.3028463	21.6293743	4.13628002	8.3240586	8.23238167	4.81131944	11.4131377
Slc10a4	3.55650012	-1.0641322	0.41620041	-2.5567783	0.01056465	NA	Slc10a4	6.04841696	7.2403795	4.45310648	6.20442003	3.32962344	1.17605452	0	0
Pip5k1b	31.8580209	0.74336716	0.29132173	2.55170517	0.01071972	0.36663458	Pip5k1b	18.1452509	24.4362808	25.7644018	22.7495401	31.6314227	48.2182355	51.96225	31.9567854
Tbc1d9	153.894225	0.64067113	0.25110905	2.5513661	0.01073015	0.36663458	Tbc1d9	120.968339	94.5774573	101.785291	150.974221	193.11816	184.64056	138.566	246.523773
Gjc3	13.9018826	-0.9109892	0.35709028	-2.5511452	0.01073696	0.36663458	Gjc3	15.4570656	17.648425	19.4028211	24.8176801	13.3184938	4.7042181	6.73584722	9.13051012
Rgs17	191.344459	0.64862856	0.25458701	2.54776771	0.01084146	0.36902408	Rgs17	142.473822	137.567211	157.449122	140.633521	143.173808	306.950231	274.245208	228.262753
Zfp536	33.0851456	0.73060628	0.28724294	2.54351341	0.01097438	0.37059142	Zfp536	18.1452509	22.1736622	27.672876	26.8858201	31.6314227	51.7463991	38.4905555	47.9351782
Ttc39b	84.3741519	0.68572502	0.26964319	2.54308303	0.01098791	0.37059142	Ttc39b	61.8282622	57.923036	79.5197586	47.5672202	83.240586	150.534979	96.2263889	98.1529838

GeneID	Base mean	log2(FC)	StdErr	Wald-Stats	P-value	P-adj		GeneID	Normalised expression for Chow#1	Normalised expression for Chow#2	Normalised expression for Chow#3	Normalised expression for Chow#4	Normalised expression for HFD#1	Normalised expression for HFD#2	Normalised expression for HFD#3	Normalised expression for HFD#4
Trp53bp2	12.5711267	-0.9790316	0.38511343	-2.5421902	0.01101602	0.37059142		Trp53bp2	20.1613899	26.2463757	17.1762679	10.3407001	8.3240586	3.52816357	12.5094306	2.28262753
Vcam1	7.68812541	-1.0519543	0.41386793	-2.5417632	0.01102949	0.37059142		Vcam1	8.06455594	21.2686148	14.3135566	6.20442003	0	5.88027262	5.77358333	0
Arhgdib	4.87172322	-1.0520916	0.41428895	-2.5395116	0.01110074	0.37059142		Arhgdib	8.06455594	13.1231878	6.04350166	4.13628002	3.32962344	2.35210905	1.92452778	0
Cables1	20.6258254	-0.8624345	0.3397371	-2.5385351	0.01113176	0.37059142		Cables1	24.8657142	25.793852	19.4028211	43.4309402	16.6481172	10.5844907	10.5849028	13.6957652
Dhrs3	7.70204016	-1.0558903	0.41611395	-2.5375027	0.01116465	0.37059142		Dhrs3	17.4732045	16.2908539	14.3135566	2.06814001	6.65924688	0	4.81131944	0
Csf1r	13.4668991	-1.0219273	0.40273341	-2.5374784	0.01116542	0.37059142		Csf1r	12.7688802	44.7998482	17.4943469	6.20442003	8.3240586	5.88027262	7.69811111	4.56525506
Ost4	16.7353803	-0.855033	0.33712431	-2.5362544	0.01120453	0.37059142		Ost4	26.2098068	20.3635674	17.4943469	26.8858201	9.98887032	10.5844907	8.660375	13.6957652
Mob1a	8.38542313	-1.0070194	0.39719509	-2.5353268	0.01123425	0.37059142		Mob1a	12.7688802	15.8383302	8.58813393	14.4769801	3.32962344	8.23238167	3.84905555	0
Slc7a2	12.9784114	-0.9925702	0.39198645	-2.5321544	0.0113364	0.37281074		Slc7a2	18.1452509	35.2968501	15.9039517	6.20442003	9.98887032	4.7042181	6.73584722	6.84788259
Iqgap2	16.449332	1.02232308	0.40392334	2.53098291	0.01137434	0.37291085		Iqgap2	4.7043243	14.480759	11.1327662	0	19.9777406	31.7534722	40.4150833	9.13051012
Atp6v1a	283.793922	0.50829601	0.20114914	2.52696088	0.01150543	0.37605521		Atp6v1a	248.657142	205.898292	268.140626	200.609581	342.951214	329.295267	384.905555	289.893696
Sspn	3.69004148	-1.0508743	0.4163631	-2.5239371	0.01160487	NA		Sspn	8.73660227	7.69290322	6.04350166	2.06814001	1.66481172	2.35210905	0.96226389	0
Hadh	10.0642336	-0.9556735	0.37908043	-2.521031	0.01170115	0.38029186		Hadh	14.1129729	15.8383302	12.0870033	14.4769801	4.99443516	4.7042181	2.88679167	11.4131377
Itgb5	13.3366094	-1.0166751	0.40330186	-2.5208789	0.01170621	0.38029186		Itgb5	9.4086486	40.7271347	18.130505	12.4088401	11.653682	8.23238167	3.84905555	2.28262753
Olfr288	1.94804523	-0.965393	0.38338186	-2.5180976	0.01179906	NA		Olfr288	2.68818531	2.71514231	1.90847421	8.27256004	0	0	0	0
Vill	2.17386569	1.02187567	0.40603276	2.51673207	0.01184489	NA		Vill	0.67204633	0.45252372	0.63615807	0	3.32962344	3.52816357	1.92452778	6.84788259
Dnah5	6.54799561	1.02877274	0.40910157	2.51471228	0.01191296	0.38583537		Dnah5	2.01613899	4.97776091	3.81694841	0	14.9833055	15.2887088	6.73584722	4.56525506
Safb	37.5522334	0.79202584	0.31536223	2.51147969	0.01202262	0.38693898		Safb	35.6184554	15.8383302	19.4028211	33.0902402	43.2851047	57.6266717	38.4905555	57.0656883
Unc13a	172.600571	0.84929948	0.33820116	2.51122584	0.01203127	0.38693898		Unc13a	138.441544	105.89055	113.872294	84.7937404	429.521424	212.865869	99.1131805	196.305968
Rrs1	13.1743485	0.85819521	0.34184078	2.51051149	0.01205564	0.38693898		Rrs1	8.73660227	6.78785578	7.31581779	12.4088401	18.3129289	15.2887088	18.2830139	18.2610202
4833439L19Rik	109.353107	-0.6346514	0.25311209	-2.5073927	0.01216255	0.38920164		4833439L19Ri	137.769497	158.383302	118.64348	126.156541	86.5702095	52.9224536	96.2263889	98.1529838
E030003E18Rik	4.35788866	-1.0162449	0.40547173	-2.5063274	0.01219926	NA		E030003E18Ri	14.7850192	8.59795066	7.95197586	0	0	3.52816357	0	0
Fmn1	18.8281952	0.89482797	0.35787508	2.50039196	0.0124056	0.3954855		Fmn1	8.06455594	12.2181404	13.3593194	12.4088401	8.3240586	34.1055812	27.9056528	34.239413
Pbx3	18.6567409	-0.822389	0.32900663	-2.4996123	0.01243293	0.3954855		Pbx3	24.1936678	25.3413283	27.0367179	24.8176801	6.65924688	17.6408179	14.4339583	9.13051012
Chchd10	207.208002	-0.8151037	0.32701385	-2.4925662	0.01268237	0.40222298		Chchd10	273.522856	244.362808	198.799397	388.810322	131.520126	65.8590533	117.396194	237.393263
Grm3	42.5137806	-0.9756918	0.39168096	-2.4910372	0.01273708	0.40276299		Grm3	61.8282622	108.605693	67.1146763	10.3407001	28.3017992	28.2253086	10.5849028	25.1089028
Kctd1	55.6910421	-0.7339773	0.29478018	-2.489914	0.0127774	0.40284618		Kctd1	96.7746713	81.4542694	69.6593086	37.2265202	44.9499164	35.2816357	41.3773472	38.804668
Isoc1	20.5605927	-0.7866864	0.31609221	-2.4887876	0.01281795	0.40293614		Isoc1	24.1936678	26.6988994	29.5813502	28.9539601	8.3240586	17.6408179	15.3962222	13.6957652
Rras	5.9826466	-1.0059204	0.40446142	-2.4870614	0.01288032	0.40370915		Rras	8.73660227	9.5029981	6.36158069	12.4088401	3.32962344	2.35210905	2.88679167	2.28262753
Tmem109	13.1950893	-0.9236019	0.37181667	-2.4840249	0.01299067	0.40520689		Tmem109	12.0968339	24.4362808	18.130505	18.6132601	8.3240586	3.52816357	6.73584722	13.6957652
Paqr7	23.7746873	-0.8020183	0.32294895	-2.4834214	0.01301271	0.40520689		Paqr7	22.8495752	39.3695635	30.2175083	33.0902402	21.6425524	16.4647633	10.5849028	15.9783927
Npc2	22.9760276	-0.8557967	0.34471446	-2.4826248	0.01304184	0.40520689		Npc2	23.5216215	38.9170398	22.5836114	39.2946602	18.3129289	12.9365998	7.69811111	20.5436478
Mdn1	32.0165332	0.75979313	0.3069432	2.47535415	0.01331041	0.41235269		Mdn1	24.1936678	20.8160911	26.4005599	16.5451201	61.5980337	45.8661264	35.6037639	25.1089028
Ptrh1	3.19505344	-0.9157778	0.3702883	-2.4731481	0.01339286	NA		Ptrh1	1.34409266	2.26261859	1.27231614	20.6814001	0	0	0	0
Ebf3	4.38682957	-0.9007266	0.36465132	-2.4701038	0.01350739	NA		Ebf3	0.67204633	1.81009488	1.59039517	31.0221002	0	0	0	0
Npr3	9.58170172	0.921044	0.37295926	2.46955661	0.01352806	0.41788415		Npr3	6.04841696	6.33533207	6.67965972	2.06814001	8.3240586	12.9365998	18.2830139	15.9783927
Colec12	1.50122864	-0.9444778	0.38260559	-2.4685417	0.01356648	NA		Colec12	2.68818531	4.07271347	3.18079035	2.06814001	0	0	0	0
Aldh6a1	30.4218058	-0.7284677	0.29515714	-2.4680675	0.01358447	0.4184173		Aldh6a1	46.3711967	42.9897533	36.579089	31.0221002	21.6425524	17.6408179	28.8679167	18.2610202
Ankrd50	64.5558794	0.73094586	0.29652966	2.46500084	0.01370129	0.42029611		Ankrd50	42.3389187	41.6321821	55.9819101	43.4309402	74.9165274	132.894161	56.7735694	68.4788259
Entpd2	3.88394579	-1.023315	0.41519515	-2.4646603	0.01371432	NA		Entpd2	4.7043243	7.69290322	5.40734359	8.27256004	4.99443516	0	0	0
AI464131	16.0944687	-1.009038	0.40944498	-2.4644044	0.01372412	0.42029611		AI464131	20.8334362	47.0624668	25.4463228	4.13628002	14.9833055	2.35210905	4.81131944	9.13051012
Scnn1a	2.66388035	-0.9913107	0.40274651	-2.4613762	0.01384052	NA		Scnn1a	2.68818531	4.07271347	2.54463228	10.3407001	1.66481172	0	0	0
Myh10	176.772599	0.69841656	0.28386141	2.46041393	0.01387769	0.42173874		Myh10	156.586795	96.3875521	145.362119	115.815841	296.336486	288.133358	162.622597	152.936045
Qk	235.728523	-0.854275	0.34726066	-2.4600396	0.01389217	0.42173874		Qk	336.023164	432.160152	305.355873	215.086561	89.8998329	151.711034	284.830111	70.7614535
Ifit3	3.37088698	-1.0201737	0.41476725	-2.4596292	0.01390806	NA		Ifit3	2.68818531	5.43028463	5.40734359	10.3407001	0	1.17605452	1.92452778	0
Cntnap3	21.8348202	0.963722	0.39188435	2.45920005	0.0139247	0.42173874		Cntnap3	6.04841696	13.5757116	20.0389792	6.20442003	23.3073641	67.0351079	20.2075417	18.2610202
Lonrf1	30.011827	0.77415067	0.31481214	2.45908771	0.01392906	0.42173874		Lonrf1	18.1452509	20.3635674	19.0847421	24.8176801	49.9443516	19.9929269	37.5282917	50.2178057
Slc12a4	5.64157335	-1.0063131	0.40979093	-2.4556745	0.01406205	0.42456257		Slc12a4	5.37637063	12.2181404	8.2700549	8.27256004	3.32962344	1.17605452	1.92452778	4.56525506
Ppm1h	107.083003	0.54965324	0.22419477	2.45167731	0.01421921	0.42809839		Ppm1h	70.5648645	82.8118406	86.8355764	101.338861	136.514561	121.133616	106.811292	150.653417
Evi2a	3.4918677	-1.0018772	0.40870047	-2.4513728	0.01423125	NA		Evi2a	7.39250961	12.6706641	4.77118552	0	0	1.17605452	1.92452778	0
Rassf3	18.8883907	0.77645763	0.31713611	2.44834189	0.01435154	0.43078645		Rassf3	12.7688802	11.313093	11.7689243	16.5451201	28.3017992	17.6408179	23.0943333	29.6741579
Tmbim4	18.4158567	-0.8914208	0.36436161	-2.4465278	0.01442397	0.43078645		Tmbim4	12.0968339	37.106945	23.8559276	31.0221002	9.98887032	12.9365998	13.4716944	6.84788259
Fam43a	27.227075	0.97715378	0.3995579	2.44558743	0.01446164	0.43078645		Fam43a	14.1129729	9.05047438	12.0870033	20.6814001	38.2906696	8.23238167	24.0565972	91.3051012
Tmem63c	22.9405011	-0.7861559	0.32148538	-2.445386	0.01446972	0.43078645		Tmem63c	26.2098068	30.7716129	30.2175083	33.0902402	23.3073641	15.2887088	8.660375	15.9783927
Gpr4	3.9545075	-1.0180289	0.41637012	-2.4450094	0.01448484	NA		Gpr4	5.37637063	8.59795066	4.13502745	8.27256004	3.32962344	0	1.92452778	0
Cers2	13.833106	-0.9497251	0.38881613	-2.4426074	0.01458159	0.43291123		Cers2	18.8172972	33.9392789	22.2655324	4.13628002	6.65924688	7.05632714	8.660375	9.13051012
Elp3	54.0093438	0.60521969	0.24800578	2.44034514	0.01467324	0.43358048		Elp3	49.059382	37.106945	37.8514051	43.4309402	81.5757743	61.1548353	62.5471528	59.3483158
Tmem119	2.27451956	-0.9615756	0.39405899	-2.4401818	0.01467987	NA		Tmem119	1.34409266	9.05047438	4.77118552	2.06814001	0	0	0.96226389	0
St18	10.0343512	0.94355203	0.38669388	2.44004907	0.01468527	0.43358048		St18	7.39250961	4.97776091	4.77118552	6.20442003	4.99443516	16.4647633	24.0565972	11.4131377
Gm9833	18.4987897	0.93490088	0.38336405	2.43867644	0.01474116	0.43403174		Gm9833	14.7850192	11.7656167	12.0870033	2.06814001	11.653682	41.1619083	38.4905555	15.9783927
Nxph2	13.6321324	0.94426523	0.38755598	2.43646151	0.01483175	0.43506039		Nxph2	6.04841696	8.59795066	9.224292	6.20442003	6.65924688	39.9858538	16.3584861	15.9783927
Clic4	32.0416794	-0.7532746	0.30924713	-2.4358338	0.01485751	0.43506039		Clic4	35.6184554	39.3695635	33.0802196	59.9760603	29.966611	15.2887088	20.2075417	22.8262753
Krt2	1.67715295	0.92124458	0.37872409	2.43249534	0.01499518	NA		Krt2	0	0.45252372	0.31807903	0	6.65924688	1.17605452	4.81131944	0
Tomm6	92.1790031	-0.7190229	0.29603479	-2.428846	0.01514697	0.44232452		Tomm6	114.919922	98.197647	96.6960265	165.451201	49.9443516	41.1619083	86.60375	84.4572186
Znrf2	14.6069834	0.85839001	0.35412473	2.42397644	0.0153516	0.44707878		Znrf2	7.39250961	10.4080455	10.4966081	8.27256004	9.98887032	17.6408179	29.8301805	22.8262753
Gna13	57.8587765	-0.6989466	0.28909764	-2.4176836	0.01561965	0.45354595		Gna13	57.1239379	84.6219354	59.4807795	97.2025805	38.2906696	36.4576902	57.7358333	31.9567854
Olig2	15.683847	-0.8931286	0.36959342	-2.4165166	0.01566981	0.45354595		Olig2	17.4732045	26.2463757	16.5401098	26.8858201	16.6481172	3.52816357	6.73584722	11.4131377
Tac2	4.9324004	-0.9896512	0.4096589	-2.4157933	0.01570097	0.45354595		Tac2	9.4086486	8.59795066	6.67965972	6.20442003	3.32962344	2.35210905	2.88679167	0
Rab3c	800.33894	0.61120239	0.25332673	2.41270389	0.01583468	0.45617532		Rab3c	618.282622	600.498975	719.176697	521.171283	685.902429	1283.07549	1207.64118	766.96285
Cpeb3	128.410291	0.53939906	0.22372001	2.41104523	0.01590688	0.45677216		Cpeb3	94.086486	97.7451233	95.7417894	126.156541	169.810795	176.408179	121.24525	146.088162
Fcer1g	3.45801395	-0.9482559	0.39352341	-2.4096557	0.01596758	NA		Fcer1g	6.72046328	15.8383302	3.18079035	0	0	0	1.92452778	0
Arl4c	88.4688219	0.69450299	0.28825442	2.40934027	0.01598139	0.45677216		Arl4c	51.7475673	50.6826565	69.0231505	88.9300204	69.9220923	98.78858	146.264111	132.392397
Crot	10.7298464	-0.9142093	0.3794727	-2.409157	0.01598942	0.45677216		Crot	12.7688802	15.3858064	22.2655324	10.3407001	8.3240586	3.52816357	8.660375	4.56525506
Birc6	150.589639	0.65077132	0.27022719	2.40823774	0.01602974	0.45677216		Birc6	124.328571	109.058216	136.137827	78.5893204	269.699499	192.872942	143.377319	150.653417
Fxyd7	51.9268939	-0.7927464	0.3293035	-2.4073428	0.01606908	0.45677216		Fxyd7	88.038069	62.4482732	54.7095939	68.2486203	33.2962344	15.2887088	31.7547083	61.6309433
Mcur1	13.9555459	-0.8936545	0.371443	-2.4058995	0.0161327	0.4573642		Mcur1	21.5054825	22.6261859	22.2655324	12.4088401	14.9833055	8.23238167	9.62263889	0
Rhobtb3	18.2308739	-0.8447998	0.35172091	-2.4019038	0.01631	0.45951092		Rhobtb3	34.2743627	22.1736622	22.2655324	22.7495401	9.98887032	10.5844907	19.2452778	4.56525506
Mical3	43.229773	0.81277669	0.33853496	2.40086485	0.01635638	0.45951092		Mical3	26.2098068	25.3413283	27.990955	35.1583802	99.8887032	65.8590533	28.8679167	36.5220405
Gpr56	62.5350647	-0.8437112	0.35145087	-2.4006519	0.0163659	0.45951092		Gpr56	54.4357526	135.304592	75.0666521	74.4530404	51.6091633	47.042181	14.4339583	47.9351782
Map1a	1024.04386	0.6338292	0.26405943	2.40032782	0.01638039	0.45951092		Map1a	788.98239	746.211613	718.858618	847.937404	1741.39306	1480.65265	736.131875	1132.18326
Taf15	20.742403	0.79752874	0.33245337	2.39891913	0.01644355	0.45957617		Taf15	14.7850192	14.480759	13.9954775	12.4088401	24.9721758	45.8661264	21.1698055	18.2610202
Gpr137b	12.6632242	-0.8606885	0.35886561	-2.3983589	0.01646872	0.45957617		Gpr137b	13.4409266	19.9110436	16.8581888	18.6132601	4.99443516	7.05632714	6.73584722	13.6957652
Hexb	18.2800741	-0.9828127	0.41077036	-2.3926087	0.01672907	0.46561779		Hexb	16.8011582	53.8503226	38.8056422	2.06814001	11.653682	11.7605452	6.73584722	4.56525506
Dync2h1	32.2440304	0.67607077	0.2826784	2.39166053	0.01677235	0.46561779		Dync2h1	20.1613899	24.8888045	27.354797	22.7495401	33.2962344	52.9224536	42.3396111	34.239413
Hlf	112.62068	-0.6018177	0.25249716	-2.3834631	0.0171506	0.47388098		Hlf	149.866331	153.858064	141.54517	107.543281	91.5646446	63.5069443	115.471667	77.6093361
Cyp2d22	8.84136811	-0.9779114	0.41031995	-2.3832899	0.01715868	0.47388098		Cyp2d22	9.4086486	21.2686148	14.9497146	8.27256004	9.98887032	5.88027262	0.96226389	0
Fam135b	26.5715519	0.78216121	0.3283669	2.38197337	0.01722014	0.47394702		Fam135b	17.4732045	18.1009488	21.9474534	14.4769801	39.9554813	49.39429	37.5282917	13.6957652
Fa2h	9.63252459	-0.9877132	0.4147719	-2.3813406	0.01724976	0.47394702		Fa2h	12.0968339	24.4362808	16.8581888	6.20442003	11.653682	3.52816357	0	2.28262753
Ccdc136	51.03054	0.64660144	0.27173609	2.37951994	0.01733521	0.4746295		Ccdc136	40.3227797	27.1514231	36.2610099	53.7716403	61.5980337	63.5069443	73.1320555	52.5004332
Pdlim5	15.349816	-0.8387888	0.35259217	-2.3789207	0.01736341	0.4746295		Pdlim5	24.1936678	27.6039469	17.4943469	14.4769801	11.653682	7.05632714	13.4716944	6.84788259
Tlr3	7.63922132	-0.9878382	0.41572939	-2.3761567	0.01749403	0.47624275		Tlr3	8.06455594	25.3413283	11.1327662	6.20442003	0	9.40843619	0.96226389	0
Lgi3	35.7307252	-0.7070021	0.29772985	-2.3746429	0.01756593	0.47624275		Lgi3	37.6345944	58.3755597	42.9406697	43.4309402	36.6258578	27.0492541	19.2452778	20.5436478
Ccdc177	18.2466716	0.8549135	0.36017055	2.37363524	0.01761394	0.47624275		Ccdc177	8.73660227	5.88280835	13.3593194	18.6132601	29.966611	18.8168724	16.3584861	34.239413
Hook3	65.4885207	0.64207265	0.27053855	2.37331293	0.01762932	0.47624275		Hook3	52.4196136	54.75537	54.0734359	35.1583802	68.2572805	101.140689	101.037708	57.0656883
Tmem189	17.8867378	-0.7783598	0.32800943	-2.37298	0.01764522	0.47624275		Tmem189	20.1613899	28.0564706	24.4920857	20.6814001	9.98887032	14.1126543	9.62263889	15.9783927
Sash1	55.3389693	-0.6396109	0.26998437	-2.3690665	0.01783305	0.47833862		Sash1	60.4841696	78.2866034	58.2084633	80.6574604	56.6035985	36.4576902	42.3396111	29.6741579
Lyar	8.20816706	0.95974636	0.40519945	2.36857765	0.01785663	0.47833862		Lyar	4.03227797	3.16766603	3.18079035	6.20442003	8.3240586	4.7042181	8.660375	27.3915304
Tpmt	5.67086819	-0.9745174	0.41146834	-2.3683898	0.0178657	0.47833862		Tpmt	9.4086486	9.95552182	10.4966081	6.20442003	0	3.52816357	5.77358333	0
Fam163a	9.07034175	-0.935097	0.39511709	-2.3666328	0.01795073	0.47833862		Fam163a	22.8495752	12.6706641	8.58813393	8.27256004	4.99443516	3.52816357	4.81131944	6.84788259
LOC101669761	4.38036426	-0.9845752	0.41627248	-2.365218	0.01801946	NA		LOC10166976	8.73660227	9.5029981	6.67965972	4.13628002	0	1.17605452	4.81131944	0
Rgs19	16.085196	-0.8473527	0.3583188	-2.3648011	0.01803975	0.47833862		Rgs19	20.8334362	14.9332827	23.8559276	31.0221002	4.99443516	11.7605452	14.4339583	6.84788259
Maf1	28.0616112	-0.701019	0.29651494	-2.3641947	0.01806932	0.47833862		Maf1	43.6830113	33.0342315	35.3067728	31.0221002	18.3129289	15.2887088	25.0188611	22.8262753
Timp3	49.3368948	-0.8687112	0.36745011	-2.3641609	0.01807096	0.47833862		Timp3	30.9141311	127.159165	66.4785182	49.6353602	18.3129289	48.2182355	28.8679167	25.1089028
1810037I17Rik	14.6876555	-0.8444096	0.35720176	-2.3639569	0.01808091	0.47833862		1810037I17Ri	22.1775288	18.5534725	18.130505	22.7495401	6.65924688	4.7042181	15.3962222	9.13051012
Mro	7.03986821	-0.9386405	0.39722217	-2.3630114	0.01812711	0.47837673		Mro	10.0806949	13.5757116	10.1785291	8.27256004	1.66481172	3.52816357	6.73584722	2.28262753
Catsperd	1.16633114	0.82171539	0.34789548	2.36196054	0.01817858	NA		Catsperd	0	0	0	0	1.66481172	1.17605452	1.92452778	4.56525506
Sdha	244.009204	0.54007994	0.22885028	2.35997063	0.01827638	0.47941207		Sdha	231.855983	144.80759	184.48584	221.290981	316.314227	338.703703	252.113139	262.502166
Smpdl3a	8.08156183	-0.9759323	0.41369942	-2.3590372	0.01832242	0.47941207		Smpdl3a	8.73660227	26.2463757	11.4508452	4.13628002	4.99443516	2.35210905	6.73584722	0
Sptbn2	154.214544	0.8264036	0.35032952	2.35893224	0.0183276	0.47941207		Sptbn2	104.167181	101.365313	93.8333152	97.2025805	434.515859	142.302597	91.4150694	168.914437
Mdc1	12.1313857	-0.9259814	0.39260378	-2.3585646	0.01834577	0.47941207		Mdc1	18.1452509	17.648425	19.0847421	16.5451201	0	15.2887088	5.77358333	4.56525506
Cgnl1	3.58809292	0.98104186	0.41633705	2.35636455	0.0184548	NA		Cgnl1	1.34409266	1.81009488	1.90847421	0	9.98887032	2.35210905	6.73584722	4.56525506
Rgcc	4.70605997	-0.9804803	0.41621169	-2.355725	0.0184866	0.48191408		Rgcc	10.7527413	4.97776091	9.54237104	4.13628002	4.99443516	0	0.96226389	2.28262753

GeneID	Base mean	log2(FC)	StdErr	Wald-Stats	P-value	P-adj	GeneID	Normalised expression for Chow#1	Normalised expression for Chow#2	Normalised expression for Chow#3	Normalised expression for Chow#4	Normalised expression for HFD#1	Normalised expression for HFD#2	Normalised expression for HFD#3	Normalised expression for HFD#4
Fgd6	12.4188836	-0.9202466	0.39083404	-2.3545712	0.01854409	0.48223663	Fgd6	14.7850192	32.129184	16.2220308	8.27256004	11.653682	8.23238167	5.77358333	2.28262753
Ppp6r2	90.2529784	0.59155058	0.25139762	2.35304765	0.01862025	0.48304177	Ppp6r2	79.9735131	64.2583681	79.8378377	51.7035003	129.855314	85.8519803	98.1509166	132.392397
Rhou	25.5745361	-0.6925	0.29454996	-2.3510443	0.01872081	0.48306817	Rhou	26.2098068	34.3918026	35.3067728	35.1583802	18.3129289	19.9929269	19.2452778	15.9783927
Rassf2	27.9422502	-0.8172559	0.34767972	-2.3505998	0.01874318	0.48306817	Rassf2	36.2905017	36.2018975	26.0824808	53.7716403	16.6481172	7.05632714	26.9433889	20.5436478
Gstm7	9.8216954	-0.9749317	0.41491024	-2.3497413	0.01878646	0.48306817	Gstm7	8.06455594	5.43028463	8.58813393	41.3628002	3.32962344	4.7042181	4.81131944	2.28262753
Ctdsp1	12.1829106	-0.9580819	0.4077929	-2.3494325	0.01880205	0.48306817	Ctdsp1	10.7527413	15.3858064	8.58813393	39.2946602	8.3240586	1.17605452	4.81131944	9.13051012
Lrrc24	17.1458245	0.84368074	0.35931069	2.34805356	0.01887181	0.48369755	Lrrc24	8.73660227	13.5757116	11.1327662	8.27256004	19.9777406	32.9295267	10.5849028	31.9567854
Gstk1	2.27308296	-0.9386046	0.39987199	-2.3472627	0.01891192	NA	Gstk1	6.72046328	4.07271347	1.59039517	4.13628002	1.66481172	0	0	0
Chsy3	13.0406571	0.8199129	0.34976412	2.34418811	0.01906855	0.487571	Chsy3	7.39250961	9.95552182	9.86045007	6.20442003	19.9777406	23.5210905	18.2830139	9.13051012
Dock5	4.30964917	-0.9743903	0.41576478	-2.3436094	0.01909816	NA	Dock5	5.37637063	4.97776091	4.45310648	14.4769801	1.66481172	3.52816357	0	0
Mar-02	14.8373221	-0.8286978	0.35366311	-2.3431843	0.01911994	0.4877181	Mar-02	13.4409266	19.4585199	23.2197695	24.8176801	14.9833055	8.23238167	7.69811111	6.84788259
Ppapdc1a	3.18565646	-0.9436551	0.40300317	-2.3415575	0.01920347	NA	Ppapdc1a	8.06455594	11.313093	2.86271131	0	0	0	0.96226389	2.28262753
Frmpd3	6.2001471	0.93878553	0.40135134	2.33906169	0.01933224	0.48970972	Frmpd3	2.68818531	3.62018975	2.86271131	4.13628002	4.99443516	14.1126543	5.77358333	11.4131377
Cited2	42.6369999	0.62629228	0.26779073	2.33873767	0.01934901	0.48970972	Cited2	34.2743627	25.3413283	32.4440615	39.2946602	46.6147282	43.5140174	62.5471528	57.0656883
Slc35b2	8.58124263	-0.8943855	0.38244213	-2.3386165	0.01935529	0.48970972	Slc35b2	10.7527413	13.1231878	11.4508452	14.4769801	1.66481172	5.88027262	6.73584722	4.56525506
Lrrfip1	16.7146193	0.83118749	0.35560854	2.33736654	0.01942013	0.48970972	Lrrfip1	14.7850192	11.7656167	11.1327662	4.13628002	21.6425524	19.9929269	36.5660278	13.6957652
Ampd3	15.4017358	-0.8029652	0.34355388	-2.3372323	0.01942711	0.48970972	Ampd3	18.1452509	19.9110436	19.4028211	24.8176801	14.9833055	5.88027262	8.660375	11.4131377
Dbx2	12.0594822	-0.9090958	0.39020594	-2.3297848	0.01981753	0.49784143	Dbx2	18.8172972	15.8383302	15.5858727	20.6814001	11.653682	2.35210905	11.5471667	0
Epb4.1l1	484.861495	0.62487963	0.26826858	2.32930608	0.01984286	0.49784143	Epb4.1l1	320.566099	321.29184	343.843436	488.081042	677.57837	531.576645	381.0565	814.898029
Trpc4	20.6087872	0.83604983	0.35939198	2.32628965	0.0200031	0.50068659	Trpc4	8.06455594	16.7433776	18.448584	8.27256004	23.3073641	38.8097993	37.5282917	13.6957652
Id4	35.7487613	-0.6902198	0.29701022	-2.3238925	0.02013125	0.50271693	Id4	60.4841696	39.3695635	42.6225906	41.3628002	18.3129289	30.5774176	32.7169722	20.5436478
Prg4	5.90707585	-0.9538074	0.41095411	-2.3209584	0.02028909	0.50547736	Prg4	4.03227797	26.2463757	9.86045007	0	1.66481172	3.52816357	1.92452778	0
Hexa	21.7519394	-0.8096064	0.3489723	-2.3199732	0.02034233	0.50562516	Hexa	22.1775288	45.2523719	28.9451921	20.6814001	11.653682	22.345036	11.5471667	11.4131377
Adcy1	288.042733	0.62097135	0.26822393	2.31512288	0.02060621	0.51019799	Adcy1	200.269806	255.675901	222.973403	196.473301	484.460211	438.668337	231.905597	273.915304
Hist1h2ak	3.20260428	-0.9477904	0.40944333	-2.3148269	0.02062241	NA	Hist1h2ak	8.06455594	6.78785578	4.13502745	2.06814001	0	0	0	4.56525506
Eea1	44.1419914	0.85761738	0.37096759	2.31183912	0.02078655	0.51019799	Eea1	29.5700384	22.1736622	23.5378486	35.1583802	21.6425524	110.549125	80.8301666	29.6741579
Pnoc	20.2626157	0.75385554	0.32612787	2.31153364	0.0208034	0.51019799	Pnoc	13.4409266	14.480759	12.7231614	14.4769801	23.3073641	31.7534722	15.3962222	36.5220405
Eci2	11.0225619	-0.8389021	0.36294204	-2.3113941	0.0208111	0.51019799	Eci2	11.4247876	16.7433776	14.3135566	18.6132601	6.65924688	5.88027262	7.69811111	6.84788259
Macf1	84.3723573	0.77387245	0.33489508	2.31079076	0.02084442	0.51019799	Macf1	58.4680306	64.7108918	59.1627004	45.4990802	176.470042	123.485725	110.660347	36.5220405
Dab2	5.38548306	-0.9474406	0.41005296	-2.3105321	0.02085871	0.51019799	Dab2	6.04841696	9.95552182	6.36158069	10.3407001	4.99443516	1.17605452	1.92452778	2.28262753
Tgfbr1	6.46037901	-0.9580651	0.41465691	-2.3105006	0.02086045	0.51019799	Tgfbr1	6.72046328	18.1009488	13.0412404	2.06814001	1.66481172	5.88027262	1.92452778	2.28262753
Sowahb	12.8138181	0.83377677	0.36156755	2.30600556	0.02111032	0.51495663	Sowahb	12.0968339	7.69290322	6.99773876	4.13628002	16.6481172	10.5844907	19.2452778	25.1089028
Lix1	25.7353233	-0.9455979	0.41029396	-2.304684	0.02118428	0.51495663	Lix1	55.1077989	48.4200379	41.0321955	12.4088401	3.32962344	10.5844907	32.7169722	2.28262753
Tspan7	652.906118	-0.7481682	0.3246678	-2.3044114	0.02119956	0.51495663	Tspan7	641.804244	1432.69009	921.156884	419.832422	487.789834	591.555426	317.547083	410.872956
Ccnd3	14.4506706	-0.8880679	0.38590111	-2.3012835	0.02137561	0.51555461	Ccnd3	19.4893435	15.8383302	12.0870033	35.1583802	8.3240586	2.35210905	8.660375	13.6957652
Rbm3	6.46333669	0.9371833	0.40729096	2.30101667	0.02139069	0.51555461	Rbm3	2.68818531	2.26261859	3.49886938	6.20442003	8.3240586	5.88027262	18.2830139	4.56525506
Sp9	22.1962648	0.8288691	0.36033994	2.3002421	0.02143451	0.51555461	Sp9	13.4409266	11.7656167	15.2677937	14.4769801	21.6425524	15.2887088	24.0565972	61.6309433
Zfp326	13.7268025	0.77654417	0.33769771	2.29952452	0.02147517	0.51555461	Zfp326	9.4086486	9.95552182	10.8146872	6.20442003	16.6481172	19.9929269	23.0943333	13.6957652
Ik	63.4645331	0.6724746	0.29252662	2.29884919	0.0215135	0.51555461	Ik	47.043243	30.3190892	38.4875632	74.4530404	68.2572805	71.739326	76.9811111	100.435611
Car4	6.78960704	-0.9387111	0.40833989	-2.2988475	0.0215136	0.51555461	Car4	12.7688802	15.8383302	10.1785291	2.06814001	1.66481172	4.7042181	4.81131944	2.28262753
Tor1aip1	23.0193546	-0.8260285	0.35959712	-2.2970944	0.02161338	0.51678711	Tor1aip1	28.8979921	40.274611	33.0802196	24.8176801	13.3184938	27.0492541	14.4339583	2.28262753
Tmem9b	53.0502701	-0.7917778	0.34521892	-2.2935526	0.02181621	0.52005051	Tmem9b	45.027104	106.795598	79.5197586	53.7716403	18.3129289	52.9224536	45.2264028	22.8262753
Nrn1	88.8679983	-0.7535506	0.32862884	-2.2930143	0.02184718	0.52005051	Nrn1	119.624246	162.003491	113.554215	72.3849004	73.2517157	49.39429	93.3395972	27.3915304
Hist1h4k	12.9228416	-0.8455857	0.36901936	-2.2914399	0.02193798	0.52035375	Hist1h4k	19.4893435	15.8383302	12.7231614	22.7495401	11.653682	4.7042181	4.81131944	11.4131377
Hnrnpa0	120.301928	0.82928209	0.36197357	2.29100178	0.02196331	0.52035375	Hnrnpa0	53.7637063	38.4645161	68.0689134	146.837941	196.447783	94.0843619	106.811292	257.936911
Grik3	128.737399	0.77742018	0.3394467	2.29025704	0.02200642	0.52035375	Grik3	63.8444012	108.605693	88.1078926	84.7937404	224.749582	264.612268	67.3584722	127.827142
Rrp1	130.743395	0.54652375	0.23871659	2.28942512	0.02205466	0.52035375	Rrp1	126.34471	78.2866034	87.7898135	126.156541	156.492302	166.999742	139.528264	164.349182
Serping1	1.90226693	-0.9138697	0.39928238	-2.2887805	0.02209211	NA	Serping1	2.01613899	4.07271347	3.81694841	4.13628002	0	1.17605452	0	0
Clic1	2.1975261	-0.911382	0.39819834	-2.2887639	0.02209307	NA	Clic1	2.68818531	2.26261859	3.18079035	8.27256004	0	1.17605452	0	0
Cdr2	11.3175245	0.83309905	0.36464137	2.28470801	0.02232995	0.52361266	Cdr2	6.72046328	5.43028463	5.72542262	12.4088401	9.98887032	15.2887088	14.4339583	20.5436478
Gab1	19.6046503	-0.7506286	0.32855275	-2.2846517	0.02233326	0.52361266	Gab1	25.5377605	30.7716129	21.6293743	24.8176801	21.6425524	11.7605452	11.5471667	9.13051012
Fancm	9.57310702	0.84067915	0.36798604	2.2845409	0.02233976	0.52361266	Fancm	6.04841696	4.52523719	7.31581779	6.20442003	8.3240586	14.1126543	16.3584861	13.6957652
Smarcd3	52.7372487	-0.5996349	0.26285653	-2.2812252	0.02253512	0.52626855	Smarcd3	77.9573741	59.7331309	62.0254117	59.9760603	26.6369875	44.6900719	45.2264028	45.6525506
Zcchc12	98.8104404	0.55969689	0.24537912	2.28094752	0.02255155	0.52626855	Zcchc12	78.6294204	64.2583681	64.251965	109.611421	109.877574	136.422325	108.735819	118.696632
Tmem60	11.7117937	-0.8799802	0.38604106	-2.279499	0.02263742	0.5271214	Tmem60	11.4247876	16.7433776	17.8124259	22.7495401	3.32962344	5.88027262	13.4716944	2.28262753
Fbxo32	5.47821764	-0.9298727	0.40830461	-2.2773995	0.02276238	0.52792605	Fbxo32	10.0806949	7.2403795	10.8146872	4.13628002	4.99443516	2.35210905	1.92452778	2.28262753
Plod2	1.68424847	0.88576122	0.38900519	2.27699074	0.02278678	NA	Plod2	0	0.45252372	0.63615807	0	3.32962344	3.52816357	0.96226389	4.56525506
Thumpd1	35.1697184	0.65131566	0.28613496	2.27625334	0.02283085	0.52792605	Thumpd1	21.5054825	19.4585199	27.354797	39.2946602	39.9554813	43.5140174	42.3396111	47.9351782
Dpysl4	26.5497666	0.86434222	0.37983325	2.27558335	0.02287096	0.52792605	Dpysl4	16.8011582	9.5029981	14.6316356	22.7495401	49.9443516	11.7605452	23.0943333	63.9135709
Ak3	55.5949496	-0.7811096	0.34343454	-2.2744061	0.02294158	0.52792605	Ak3	77.2853278	96.3875521	87.4717345	35.1583802	18.3129289	35.2816357	60.622625	34.239413
Thsd7a	72.8342454	0.84088596	0.3700037	2.27264201	0.02304776	0.52792605	Thsd7a	30.9141311	59.2806072	60.4350166	28.9539601	58.2684102	214.041923	57.7358333	73.044081
Bcas1	11.0833847	-0.8414593	0.37026603	-2.2725804	0.02305148	0.52792605	Bcas1	17.4732045	16.7433776	13.9954775	12.4088401	1.66481172	8.23238167	6.73584722	11.4131377
Bag3	5.91247946	-0.9380126	0.4127572	-2.272553	0.02305313	0.52792605	Bag3	5.37637063	9.05047438	7.31581779	14.4769801	6.65924688	1.17605452	0.96226389	2.28262753
Pxmp4	8.05072525	-0.9236741	0.40658456	-2.2717885	0.02309929	0.52792605	Pxmp4	15.4570656	13.5757116	5.08926455	14.4769801	3.32962344	1.17605452	6.73584722	4.56525506
Hsd17b10	10.5152732	-0.8403697	0.36996189	-2.2715034	0.02311652	0.52792605	Hsd17b10	14.7850192	19.4585199	11.1327662	12.4088401	6.65924688	7.05632714	5.77358333	6.84788259
Fam69c	3.49995531	-0.9394065	0.4136227	-2.2711676	0.02313683	NA	Fam69c	3.36023164	5.88280835	2.54463228	12.4088401	1.66481172	1.17605452	0.96226389	0
Tns3	36.2705132	-0.7288585	0.3213525	-2.2680964	0.02332333	0.53040556	Tns3	43.010965	62.9007969	36.579089	45.4990802	43.2851047	22.345036	18.2830139	18.2610202
Ece2	22.6953371	0.76455245	0.3370924	2.26807975	0.02332435	0.53040556	Ece2	8.73660227	19.4585199	20.9932163	10.3407001	31.6314227	37.6337448	23.0943333	29.6741579
Pde6d	31.825039	-0.6408123	0.28284341	-2.265608	0.02347539	0.53270701	Pde6d	43.010965	37.106945	41.9864326	37.2265202	24.9721758	17.6408179	29.8301805	22.8262753
Cdkn1c	3.42121917	0.93428411	0.41256346	2.26458277	0.0235383	NA	Cdkn1c	2.68818531	0	0.63615807	2.06814001	8.3240586	2.35210905	6.73584722	4.56525506
Arpp21	222.077498	-0.8257203	0.36474959	-2.2638004	0.02358639	0.53310711	Arpp21	396.507334	358.398785	368.017443	93.0663005	124.860879	150.534979	221.320694	63.9135709
Etl4	166.41544	0.7260275	0.32072651	2.26369658	0.02359278	0.53310711	Etl4	130.376988	130.326831	153.950253	49.6353602	143.173808	317.534722	214.584847	191.740713
Dlg2	381.89875	0.43431856	0.19210209	2.26087364	0.02376708	0.53543158	Dlg2	323.92633	315.861556	364.200495	279.198901	359.599332	475.126028	464.773458	472.503899
Btf3	10.8872072	-0.8321832	0.36815595	-2.2604095	0.02379585	0.53543158	Btf3	14.1129729	17.648425	13.0412404	16.5451201	3.32962344	8.23238167	9.62263889	4.56525506
Golgb1	40.6498976	0.85602299	0.37909799	2.25805202	0.02394242	0.53695534	Golgb1	28.2259458	27.1514231	31.4898244	10.3407001	46.6147282	110.549125	54.8490416	15.9783927
Appl2	18.2353508	-0.7888279	0.34939367	-2.2577052	0.02396404	0.53695534	Appl2	31.5861774	23.9837571	25.4463228	16.5451201	8.3240586	7.05632714	19.2452778	13.6957652
Zswim7	3.14167149	-0.9009064	0.39916125	-2.2569986	0.02400816	NA	Zswim7	2.01613899	2.26261859	4.45310648	14.4769801	0	0	1.92452778	0
Hnrnpa2b1	74.4236684	0.68408949	0.30325554	2.25581863	0.02408199	0.53846931	Hnrnpa2b1	62.5003085	36.2018975	47.0756971	74.4530404	68.2572805	136.422325	101.999972	68.4788259
Rab7l1	2.09609195	-0.8719858	0.38660184	-2.2555137	0.0241011	NA	Rab7l1	8.73660227	4.52523719	2.54463228	0	0	0	0.96226389	0
Hist1h2ao	2.72992802	-0.8914407	0.39593667	-2.251473	0.02435559	NA	Hist1h2ao	10.0806949	6.33533207	3.49886938	0	0	0	1.92452778	0
Litaf	7.82968983	-0.9215995	0.409756	-2.2491422	0.02450345	0.54505143	Litaf	10.0806949	10.8605693	8.2700549	16.5451201	3.32962344	1.17605452	0.96226389	11.4131377
Sptbn4	64.8445106	0.89830955	0.39953978	2.24836072	0.0245532	0.54505143	Sptbn4	22.8495752	18.5534725	16.5401098	86.8618804	151.497867	43.5140174	44.2641389	134.675024
Ogfr	31.2716161	0.90768697	0.40374365	2.24817648	0.02456494	0.54505143	Ogfr	12.7688802	11.7656167	13.3593194	28.9539601	53.2739751	19.9929269	9.62263889	100.435611
Nr1d1	166.285859	-0.5705154	0.25384497	-2.2474954	0.02460838	0.54505143	Nr1d1	239.920539	165.171157	176.215785	227.495401	166.481172	90.5561984	118.358458	146.088162
Col25a1	95.0498475	0.78221519	0.34819999	2.2464538	0.02467496	0.54505143	Col25a1	41.6668724	74.2138899	67.1146763	70.3167603	93.2294563	256.379886	79.8679027	77.6093361
Ppp1ca	118.784281	-0.5867282	0.26119317	-2.2463383	0.02468234	0.54505143	Ppp1ca	175.404092	121.276357	111.645741	173.723761	94.8942681	65.8590533	93.3395972	114.131377
Trf	31.8803944	-0.830251	0.3697487	-2.2454468	0.02473946	0.54518638	Trf	38.978687	74.6664136	40.3960374	20.6814001	31.6314227	27.0492541	12.5094306	9.13051012
1110002L01Rik	72.2820648	0.5494727	0.24486888	2.24394668	0.02483583	0.5461839	1110002L01Ri	47.043243	47.9675142	62.9796488	74.4530404	78.2461509	98.78858	86.60375	82.1745911
Chodl	3.7660279	0.8922034	0.3976077	2.24392886	0.02483698	NA	Chodl	1.34409266	0.45252372	1.90847421	0	3.32962344	21.1689814	1.92452778	0
Ephb1	12.7428547	-0.8526974	0.38048025	-2.241108	0.02501908	0.5472219	Ephb1	23.5216215	15.3858064	14.3135566	16.5451201	8.3240586	8.23238167	1.92452778	13.6957652
Chst8	5.96361426	-0.909783	0.40597085	-2.2410057	0.0250257	0.5472219	Chst8	7.39250961	13.1231878	6.67965972	8.27256004	3.32962344	4.7042181	1.92452778	2.28262753
1700052N19Rik	15.1171933	0.75771231	0.33813807	2.24083707	0.02503663	0.5472219	1700052N19R	12.7688802	7.69290322	11.4508452	8.27256004	26.6369875	15.2887088	18.2830139	20.5436478
Fezf1	2.48223724	-0.8040841	0.35887417	-2.2405739	0.02505369	NA	Fezf1	0.67204633	4.07271347	0.63615807	14.4769801	0	0	0	0
Fgfr3	37.9847267	-0.7984604	0.3566399	-2.2388419	0.02516621	0.54811839	Fgfr3	38.3066407	72.8563187	45.4853019	47.5672202	51.6091633	17.6408179	14.4339583	15.9783927
Efna3	15.5408682	-0.7600773	0.33952837	-2.2386267	0.02518021	0.54811839	Efna3	23.5216215	23.9837571	16.8581888	16.5451201	11.653682	9.40843619	8.660375	13.6957652
Usp13	29.2510357	0.64797233	0.2896976	2.23671972	0.02530466	0.54915945	Usp13	24.8657142	18.5534725	22.9016905	20.6814001	44.9499164	43.5140174	28.8679167	29.6741579
Gart	17.2108299	0.72558644	0.32467707	2.23479419	0.02543086	0.54915945	Gart	11.4247876	10.4080455	11.7689243	14.4769801	24.9721758	17.6408179	17.32075	29.6741579
Id3	10.1181657	-0.8909611	0.39878439	-2.2341924	0.02547042	0.54915945	Id3	12.0968339	11.7656167	11.7689243	22.7495401	11.653682	1.17605452	2.88679167	6.84788259
Dnah9	5.16515138	0.92889412	0.41577173	2.23414451	0.02547357	0.54915945	Dnah9	1.34409266	4.52523719	2.22655324	0	16.6481172	5.88027262	3.84905555	6.84788259
Snx3	156.759842	-0.5423344	0.24276693	-2.2339716	0.02548494	0.54915945	Snx3	171.371814	184.177154	166.991493	233.699821	106.54795	91.7322529	144.339583	155.218672
Prdm5	1.8438833	0.86080894	0.38534787	2.23384898	0.02549301	NA	Prdm5	0	1.35757116	0	0	1.66481172	2.35210905	4.81131944	4.56525506
Orai3	12.8495944	-0.8385875	0.37574385	-2.2318063	0.02562777	0.55082763	Orai3	10.7527413	18.5534725	13.3593194	28.9539601	8.3240586	4.7042181	6.73584722	11.4131377
Idh3b	133.525672	0.4531494	0.20309335	2.23123701	0.02566543	0.55082763	Idh3b	120.296293	107.248121	117.371164	99.2707205	133.184938	151.711034	177.056555	162.066555
Mpped1	228.586019	0.64808382	0.29082211	2.22845446	0.02585023	0.55368177	Mpped1	172.04386	150.237875	148.860988	208.882141	344.616026	164.647633	219.396167	420.003466
Gng10	20.406512	-0.8243139	0.37031853	-2.2259591	0.02601692	0.55533809	Gng10	37.6345944	31.6766603	29.5813502	14.4769801	8.3240586	12.9365998	24.0565972	4.56525506
Ppm1e	173.660369	0.53869837	0.2421717	2.22444806	0.02611831	0.55533809	Ppm1e	150.538378	146.165161	162.538387	90.9981605	164.81636	230.506687	240.565972	203.15385
Ep400	82.0466269	0.66838885	0.30053825	2.22397263	0.02615029	0.55533809	Ep400	66.5325865	42.5372296	53.1191988	80.6574604	148.168243	116.429398	64.4716805	84.4572186
1700001L05Rik	6.06568203	0.89008722	0.40029331	2.22358755	0.02617621	0.55533809	1700001L05Ri	3.36023164	5.43028463	2.22655324	2.06814001	9.98887032	7.05632714	11.5471667	6.84788259
Pnpla2	14.8395238	-0.8085361	0.36364488	-2.2234221	0.02618735	0.55533809	Pnpla2	20.8334362	27.1514231	13.6773985	18.6132601	14.9833055	4.7042181	9.62263889	9.13051012
Ccbl2	2.00594481	-0.8870463	0.39898361	-2.2232651	0.02619793	NA	Ccbl2	3.36023164	4.97776091	1.90847421	4.13628002	1.66481172	0	0	0
Cd302	4.41899977	-0.9238998	0.41633349	-2.2191341	0.02647761	NA	Cd302	8.73660227	9.95552182	9.54237104	0	1.66481172	3.52816357	1.92452778	0
Gad2	518.62161	0.68570358	0.30948775	2.21560814	0.02671835	0.5654767	Gad2	398.523473	316.31408	476.482394	308.152862	377.912261	1041.98431	789.056389	440.547113

GeneID	Base mean	log2(FC)	StdErr	Wald-Stats	P-value	P-adj		GeneID	Normalised expression for Chow#1	Normalised expression for Chow#2	Normalised expression for Chow#3	Normalised expression for Chow#4	Normalised expression for HFD#1	Normalised expression for HFD#2	Normalised expression for HFD#3	Normalised expression for HFD#4
Rhog	13.2637443	-0.8616959	0.38919419	-2.2140512	0.02682525	0.56661723		Rhog	16.8011582	17.648425	12.4050823	28.9539601	16.6481172	2.35210905	6.73584722	4.56525506
Fhod3	27.9239609	0.64067542	0.28975855	2.2110665	0.02703123	0.56984183		Fhod3	18.1452509	19.4585199	22.5836114	24.8176801	43.2851047	37.6337448	34.6415	22.8262753
Nadk	42.96111	-0.6808015	0.30815451	-2.2092861	0.02715475	0.57131881		Nadk	51.7475673	57.923036	47.0756971	59.9760603	26.6369875	17.6408179	27.9056528	54.7830607
Sst	786.859189	0.63587053	0.28793969	2.20834623	0.02722015	0.5715696		Sst	492.609959	566.559696	549.004414	750.734824	925.635317	900.857765	568.697958	1540.77358
Elavl2	134.829674	0.63653363	0.28857637	2.20577186	0.02739998	0.57382183		Elavl2	112.903783	91.4097912	115.46269	84.7937404	98.2238915	212.865869	232.867861	130.109769
Pus1	10.7201091	0.83840126	0.38017986	2.2052753	0.02743478	0.57382183		Pus1	7.39250961	3.62018975	5.72542262	10.3407001	16.6481172	7.05632714	14.4339583	20.5436478
Lgi4	12.7213486	-0.8448874	0.38328751	-2.2043175	0.02750203	0.5741048		Lgi4	14.1129729	18.5534725	15.2677937	22.7495401	16.6481172	4.7042181	2.88679167	6.84788259
Klhl21	17.3356802	0.89763331	0.40760775	2.20219884	0.02765127	0.57413116		Klhl21	6.72046328	4.52523719	5.72542262	20.6814001	29.966611	14.1126543	6.73584722	50.2178057
Atp6v0a2	40.1374632	-0.674775	0.30649275	-2.2016019	0.02769344	0.57413116		Atp6v0a2	43.010965	70.1411764	45.803381	45.4990802	31.6314227	42.3379629	22.1320694	20.5436478
Nfe2l2	7.3974997	-0.8646253	0.3928059	-2.2011516	0.02772529	0.57413116		Nfe2l2	10.0806949	14.480759	11.1327662	6.20442003	6.65924688	3.52816357	4.81131944	2.28262753
St8sia1	93.8790082	0.72831958	0.33090688	2.20098046	0.02773741	0.57413116		St8sia1	30.9141311	60.1856546	65.5242811	107.543281	124.860879	164.647633	74.0943194	123.261887
Ctsc	1.85635834	-0.8718358	0.39617865	-2.2006129	0.02776344	NA		Ctsc	2.01613899	4.97776091	2.54463228	4.13628002	0	1.17605452	0	0
Epas1	107.421499	-0.7486256	0.34035998	-2.1995111	0.0278416	0.57413116		Epas1	133.737219	150.690398	110.055346	171.655621	141.508996	31.7534722	60.622625	59.3483158
Tspan12	11.3812919	-0.8594377	0.39086073	-2.1988337	0.02788975	0.57413116		Tspan12	11.4247876	21.7211385	19.4028211	12.4088401	0	10.5844907	8.660375	6.84788259
Exoc8	36.3940445	0.62630568	0.28487237	2.19854834	0.02791005	0.57413116		Exoc8	30.9141311	27.6039469	33.0802196	14.4769801	51.6091633	47.042181	38.4905555	47.9351782
Lrig1	24.6356787	-0.8319315	0.3784559	-2.1982257	0.02793303	0.57413116		Lrig1	24.8657142	57.923036	31.8079035	20.6814001	23.3073641	19.9929269	4.81131944	13.6957652
Hist1h4h	27.8591293	-0.7521947	0.34255006	-2.195868	0.0281014	0.57574272		Hist1h4h	43.010965	38.0119924	26.0824808	39.2946602	16.6481172	7.05632714	23.0943333	29.6741579
Klhl18	33.3257478	0.62420399	0.28429512	2.19561976	0.02811917	0.57574272		Klhl18	24.8657142	22.1736622	24.1740066	31.0221002	49.9443516	49.39429	30.7924444	34.239413
Cpt1a	9.08140968	-0.8299154	0.37819531	-2.1944096	0.02820597	0.57641577		Cpt1a	14.1129729	16.7433776	11.1327662	8.27256004	4.99443516	7.05632714	5.77358333	4.56525506
Ppih	1.90038339	0.87458204	0.39856518	2.19432628	0.02821196	NA		Ppih	0	0.45252372	1.27231614	0	4.99443516	2.35210905	3.84905555	2.28262753
Zfp112	4.59650877	0.89716053	0.40895812	2.19377119	0.02825186	NA		Zfp112	1.34409266	3.62018975	2.22655324	2.06814001	6.65924688	8.23238167	5.77358333	6.84788259
Obscn	3.13534747	0.86994171	0.39679662	2.1924121	0.02834976	NA		Obscn	0.67204633	0	0.9542371	2.06814001	11.653682	0	2.88679167	6.84788259
Tubb2a-ps2	12.4529301	0.78642598	0.35881753	2.19171559	0.02840005	0.57757687		Tubb2a-ps2	9.4086486	9.5029981	7.63389683	6.20442003	21.6425524	15.2887088	23.0943333	6.84788259
Cntfr	68.5775875	-0.7199096	0.32851968	-2.1913744	0.02842471	0.57757687		Cntfr	65.1884939	128.064212	67.1146763	95.1344405	76.5813391	45.8661264	25.0188611	45.6525506
Bgn	4.89726898	-0.8851489	0.40406102	-2.1906317	0.02847846	0.57757687		Bgn	1.34409266	19.4585199	10.4966081	2.06814001	0	3.52816357	0	2.28262753
Lrrc16a	14.5449849	-0.779134	0.35566746	-2.1906249	0.02847895	0.57757687		Lrrc16a	20.8334362	24.4362808	16.8581888	16.5451201	14.9833055	11.7605452	8.660375	2.28262753
Hnrnpd	105.058408	0.59979371	0.27405269	2.18860728	0.0286254	0.57786921		Hnrnpd	86.02193	66.5209867	103.693765	66.1804803	98.2238915	162.295524	161.660333	95.8703563
Anapc7	22.5936086	0.81156231	0.3708411	2.1884368	0.0286378	0.57786921		Anapc7	12.7688802	12.2181404	11.4508452	20.6814001	41.620293	14.1126543	15.3962222	52.5004332
Zfp407	18.359168	0.81207758	0.37111794	2.18819275	0.02865557	0.57786921		Zfp407	14.1129729	8.59795066	16.8581888	6.20442003	19.9777406	44.6900719	25.0188611	11.4131377
Tap2	3.01891065	-0.9060013	0.41410325	-2.1878633	0.02867956	NA		Tap2	3.36023164	5.43028463	3.49886938	8.27256004	1.66481172	0	1.92452778	0
Ptprz1	108.19166	-0.7625432	0.34866668	-2.1870262	0.02874061	0.57849281		Ptprz1	125.672663	243.005237	151.40562	53.7716403	91.5646446	94.0843619	55.8113055	50.2178057
Edaradd	1.52301579	0.82172934	0.37573513	2.18699098	0.02874318	NA		Edaradd	0.67204633	0	0.31807903	0	0	4.7042181	1.92452778	4.56525506
Tgfa	14.2663207	-0.8285933	0.37904956	-2.1859763	0.02881734	0.5789469		Tgfa	17.4732045	27.1514231	22.9016905	10.3407001	9.98887032	2.35210905	12.5094306	11.4131377
Hcfc1r1	98.9193887	-0.6536149	0.29923876	-2.1842588	0.02894323	0.58038513		Hcfc1r1	121.640385	111.773359	97.9683426	167.519341	59.9332219	41.1619083	81.7924305	109.566121
Aqp4	51.4361306	-0.8614477	0.39464745	-2.1828286	0.02904844	0.58140389		Aqp4	63.8444012	125.801594	89.3802087	14.4769801	26.6369875	44.6900719	37.5282917	9.13051012
Nsun3	4.59121794	-0.8980677	0.41170042	-2.1813622	0.02915664	NA		Nsun3	6.04841696	8.14542694	7.63389683	6.20442003	0	3.52816357	2.88679167	2.28262753
Slc25a34	8.42047406	-0.8876982	0.4072481	-2.1797479	0.02927616	0.58486645		Slc25a34	10.0806949	11.313093	7.63389683	20.6814001	9.98887032	1.17605452	1.92452778	4.56525506
Degs1	46.8964933	-0.7281258	0.33427352	-2.1782336	0.02938865	0.58601849		Degs1	45.6991503	96.3875521	70.9316247	31.0221002	34.9610461	43.5140174	29.8301805	22.8262753
Nos1ap	73.2365453	0.68269717	0.31376369	2.17583229	0.0295678	0.58799947		Nos1ap	54.4357526	38.4645161	46.7576181	72.3849004	104.883138	76.4435441	50.9999861	141.522907
Rpl41	97.0658271	-0.6935626	0.31887973	-2.1749974	0.02963031	0.58799947		Rpl41	161.963165	86.884554	110.691504	138.565381	68.2572805	30.5774176	83.7169583	95.8703563
1810058I24Rik	13.2024496	-0.7859928	0.36149974	-2.1742556	0.02968595	0.58799947		1810058I24Ri	17.4732045	13.5757116	16.2220308	24.8176801	13.3184938	4.7042181	8.660375	6.84788259
Pigx	5.89376985	-0.8839134	0.40659118	-2.1739611	0.02970806	0.58799947		Pigx	8.73660227	12.6706641	6.67965972	6.20442003	1.66481172	4.7042181	1.92452778	4.56525506
Herc2	170.409987	0.69815003	0.32134966	2.17255565	0.02981378	0.5890012		Herc2	120.968339	113.13093	102.10337	150.974221	349.610461	271.668595	108.735819	146.088162
Ccdc80	3.15309104	-0.9008739	0.4149638	-2.1709698	0.02993345	NA		Ccdc80	5.37637063	9.05047438	4.45310648	2.06814001	0	2.35210905	1.92452778	0
Tnfsf12	5.55760259	-0.888268	0.40948908	-2.1692104	0.03006671	0.59290229		Tnfsf12	6.72046328	10.4080455	7.31581779	8.27256004	3.32962344	0	3.84905555	4.56525506
Gpr50	1.71283345	-0.788431	0.3634753	-2.1691459	0.03007161	NA		Gpr50	0.67204633	3.16766603	1.59039517	8.27256004	0	0	0	0
Eif4ebp1	1.2290582	-0.8034412	0.37048345	-2.1686291	0.03011086	NA		Eif4ebp1	2.68818531	3.16766603	1.90847421	2.06814001	0	0	0	0
C030037D09Rik	3.13328827	-0.9028916	0.41634916	-2.1685924	0.03011364	NA		C030037D09Ri	6.04841696	4.07271347	6.04350166	4.13628002	1.66481172	1.17605452	1.92452778	0
Camta1	298.789901	0.49084079	0.22640168	2.16800857	0.03015803	0.59309134		Camta1	223.119381	212.233624	239.831592	306.084722	344.616026	457.48521	307.924444	299.024207
Htr1a	18.3372801	0.79677655	0.36758056	2.16762429	0.03018728	0.59309134		Htr1a	4.03227797	18.1009488	10.4966081	14.4769801	34.9610461	23.5210905	18.2830139	22.8262753
Cdk11b	36.3911166	0.63770455	0.2943544	2.16645152	0.0302767	0.59375665		Cdk11b	34.9464091	22.6261859	19.7209001	33.0902402	38.2906696	37.6337448	50.0377222	54.7830607
Fgfr2	14.4767979	-0.8582284	0.39644588	-2.1648058	0.03040256	0.59513286		Fgfr2	14.7850192	31.2241366	17.8124259	18.6132601	19.9777406	8.23238167	2.88679167	2.28262753
Pcdhgc3	253.762782	-0.6651805	0.30744804	-2.1635542	0.03049858	0.59592103		Pcdhgc3	317.877913	473.33981	318.079035	175.791901	254.716193	206.985596	121.24525	162.066555
Dpp7	3.92523395	-0.9000598	0.41608366	-2.1631703	0.03052809	NA		Dpp7	2.68818531	8.14542694	7.31581779	6.20442003	1.66481172	1.17605452	1.92452778	2.28262753
Tecr	173.40869	-0.6254877	0.28920857	-2.1627566	0.03055991	0.59602973		Tecr	153.226563	285.089943	198.799397	225.427261	134.849749	142.302597	74.0943194	173.479692
Rnasek	127.852772	-0.6412808	0.29673366	-2.1611326	0.0306851	0.59738124		Rnasek	108.871505	199.56296	139.000538	192.337021	93.2294563	74.091435	67.3584722	148.37079
Cfh	3.23258409	-0.8716507	0.40343269	-2.1605853	0.03072739	NA		Cfh	2.68818531	11.313093	8.2700549	0	1.66481172	0	1.92452778	0
Cpe	730.654231	-0.8170887	0.3785244	-2.1586157	0.03087999	0.60008242		Cpe	939.520767	1656.23681	1109.77775	328.834262	359.599332	812.653676	462.84893	175.76232
Rasgef1c	15.0790038	-0.7729189	0.35867513	-2.1549274	0.03116751	0.60243913		Rasgef1c	26.2098068	19.9110436	21.6293743	14.4769801	8.3240586	15.2887088	12.5094306	2.28262753
Herc1	190.955382	0.52802591	0.24508913	2.15442399	0.03120694	0.60243913		Herc1	169.355675	142.997495	154.268332	144.769801	291.342051	279.900977	176.094292	168.914437
Snhg11	81.4916435	0.84397121	0.39174995	2.15436199	0.03121179	0.60243913		Snhg11	21.5054825	26.6988994	23.2197695	124.088401	131.520126	64.6829988	98.1509166	162.066555
Ift27	11.5204077	-0.8313339	0.38591819	-2.1541715	0.03122673	0.60243913		Ift27	16.8011582	10.8605693	11.1327662	26.8858201	11.653682	3.52816357	6.73584722	4.56525506
Hist1h2ap	2.54959688	-0.8474297	0.39375786	-2.1521596	0.03138479	NA		Hist1h2ap	9.4086486	5.88280835	3.18079035	0	0	0	1.92452778	0
Tmem159	7.86003073	-0.8654759	0.40223067	-2.1516903	0.03142175	0.60510933		Tmem159	9.4086486	10.4080455	6.36158069	20.6814001	3.32962344	2.35210905	5.77358333	4.56525506
Acadl	13.0482366	-0.7832313	0.36416762	-2.150744	0.03149641	0.60540932		Acadl	20.1613899	20.3635674	16.5401098	14.4769801	3.32962344	8.23238167	14.4339583	6.84788259
Nars	128.710303	0.56757001	0.26397892	2.15005813	0.03155062	0.60540932		Nars	125.000617	80.549222	86.5174974	113.747701	108.212762	188.168724	190.52825	136.957652
Snord15b	2.57736251	0.73489207	0.3419551	2.14908933	0.03162732	NA		Snord15b	0	0.45252372	0	0	13.3184938	0	0	6.84788259
Aars	103.619173	0.51840425	0.24128067	2.14855274	0.03166987	0.60660858		Aars	96.7746713	60.1856546	85.5632603	93.0663005	138.179373	137.598379	112.584875	105.000866
Rab27a	11.3176471	0.78006143	0.36338138	2.14667417	0.03181923	0.60806972		Rab27a	9.4086486	6.33533207	5.40734359	8.27256004	14.9833055	11.7605452	11.5471667	22.8262753
Kdelr1	46.5944542	-0.5964897	0.27804116	-2.1453288	0.03192657	0.60806972		Kdelr1	46.3711967	78.2866034	56.6180681	47.5672202	38.2906696	39.9858538	33.6792361	31.9567854
Barx2	3.25492727	0.89286764	0.41626074	2.144972	0.03195509	NA		Barx2	2.01613899	0.90504744	1.27231614	2.06814001	6.65924688	4.7042181	3.84905555	4.56525506
Tnks1bp1	44.3147079	0.83457778	0.38923491	2.14414938	0.03202092	0.60806972		Tnks1bp1	25.5377605	17.648425	15.2677937	49.6353602	139.844185	39.9858538	34.6415	31.9567854
Ddx23	51.5757963	0.66065929	0.30818139	2.14373519	0.03205411	0.60806972		Ddx23	31.5861774	28.961518	39.7598793	51.7035003	89.8998329	58.8027262	36.5660278	75.3267085
Rab3b	164.919634	0.5731302	0.26744652	2.14297124	0.03211541	0.60806972		Rab3b	133.065173	111.320835	130.730483	138.565381	129.855314	156.415252	238.641444	280.763186
Golga7	38.2214742	-0.6320609	0.29507704	-2.1420197	0.03219189	0.60806972		Golga7	34.9464091	46.1574193	44.2129858	68.2486203	29.966611	21.1689814	33.6792361	27.3915304
Rpph1	136.191538	0.88673771	0.41402549	2.14174666	0.03221387	0.60806972		Rpph1	56.4518916	24.8888045	27.354797	161.314921	366.258578	22.345036	284.830111	146.088162
Hist1h4m	14.7924528	-0.798196	0.37268977	-2.1417169	0.03221627	0.60806972		Hist1h4m	20.8334362	18.5534725	18.130505	22.7495401	11.653682	1.17605452	11.5471667	13.6957652
Cdk19	35.4711134	-0.6691741	0.31262361	-2.1405104	0.03231354	0.60806972		Cdk19	48.3873356	46.1574193	55.345752	31.0221002	29.966611	36.4576902	25.0188611	11.4131377
Fgf11	37.7195888	0.62344821	0.29126407	2.14049131	0.03231508	0.60806972		Fgf11	25.5377605	25.3413283	28.9451921	33.0902402	44.9499164	31.7534722	41.3773472	70.7614535
Atp10a	3.67447688	-0.8895381	0.41622381	-2.137163	0.03258474	NA		Atp10a	3.36023164	9.5029981	5.08926455	6.20442003	0	2.35210905	2.88679167	0
Cacng3	80.2360809	-0.6854646	0.32093239	-2.1358536	0.03269135	0.61406884		Cacng3	109.543552	151.142922	108.146872	41.3628002	51.6091633	70.5632714	61.5848889	47.9351782
Scrg1	10.5328299	-0.8862753	0.41526003	-2.1342659	0.03282102	0.61439057		Scrg1	11.4247876	33.9392789	22.2655324	0	1.66481172	8.23238167	6.73584722	0
Tprgl	100.401485	-0.5971132	0.27984365	-2.1337388	0.03286417	0.61439057		Tprgl	140.457683	104.080455	108.146872	142.701661	84.9053977	41.1619083	90.4528055	91.3051012
P2ry12	6.72283228	-0.8870002	0.41581712	-2.1331498	0.03291244	0.61439057		P2ry12	7.39250961	23.9837571	9.86045007	2.06814001	0	4.7042181	5.77358333	0
Rnf114	46.0895914	-0.6930381	0.32493778	-2.1328331	0.03293842	0.61439057		Rnf114	65.8605402	44.7998482	35.6248519	93.0663005	44.9499164	25.8731995	28.8679167	29.6741579
Fez1	75.707117	0.58962634	0.27654777	2.1320958	0.03299897	0.61439219		Fez1	72.5810035	51.587704	69.9773876	37.2265202	84.9053977	81.1477622	126.056569	82.1745911
Brd8	27.7970138	0.60572352	0.28423924	2.13103414	0.03308633	0.61439219		Brd8	25.5377605	19.4585199	20.0389792	20.6814001	34.9610461	32.9295267	41.3773472	27.3915304
Il12rb2	4.65091582	0.88226597	0.41406644	2.13073529	0.03311096	0.61439219		Il12rb2	0.67204633	3.16766603	1.90847421	4.13628002	8.3240586	10.5844907	3.84905555	4.56525506
Kcns3	5.84876451	0.84219956	0.39567359	2.12852102	0.03329391	0.61671628		Kcns3	2.68818531	3.16766603	3.81694841	4.13628002	9.98887032	9.40843619	6.73584722	6.84788259
Tmem123	4.64248179	-0.88443	0.41589757	-2.1265571	0.0334569	NA		Tmem123	4.03227797	13.5757116	7.31581779	4.13628002	1.66481172	3.52816357	2.88679167	0
Mpdu1	21.419438	-0.6703808	0.31528314	-2.1262819	0.0334798	0.61908665		Mpdu1	24.8657142	33.9392789	24.8101647	24.8176801	21.6425524	14.1126543	13.4716944	13.6957652
Rasgef1b	20.5628132	-0.7022442	0.33070005	-2.1235081	0.0337113	0.61980762		Rasgef1b	31.5861774	22.6261859	33.7163777	18.6132601	16.6481172	12.9365998	19.2452778	9.13051012
Fkbp9	12.9866835	-0.7946971	0.37424448	-2.1234704	0.03371446	0.61980762		Fkbp9	19.4893435	26.2463757	13.0412404	12.4088401	13.3184938	9.40843619	7.69811111	2.28262753
Ank1	73.8369233	0.69483877	0.32723698	2.12335041	0.0337245	0.61980762		Ank1	34.9464091	35.2968501	48.6660923	95.1344405	89.8998329	127.013889	61.5848889	98.1529838
Igsf11	29.8686917	-0.7623935	0.35910513	-2.123037	0.03375075	0.61980762		Igsf11	36.9625481	70.1411764	36.579089	14.4769801	14.9833055	24.697145	18.2830139	22.8262753
Gm14405	1.44455576	0.79243643	0.37355914	2.1213145	0.03389535	NA		Gm14405	0.67204633	0.45252372	0	0	1.66481172	5.88027262	2.88679167	0
Laptm5	10.3091869	-0.880132	0.41492803	-2.121168	0.03390767	0.62112775		Laptm5	6.04841696	36.6544212	17.1762679	2.06814001	6.65924688	3.52816357	5.77358333	4.56525506
Praf2	63.3998917	-0.7332959	0.34584905	-2.1202774	0.03398266	0.62112775		Praf2	88.038069	128.064212	89.6982877	26.8858201	34.9610461	55.2745626	50.0377222	34.239413
Kdelr2	23.3347281	-0.6879131	0.32447087	-2.1201075	0.03399698	0.62112775		Kdelr2	21.5054825	41.6321821	26.0824808	31.0221002	18.3129289	19.9929269	14.4339583	13.6957652
Cuta	43.7143779	-0.7660451	0.36203109	-2.1159649	0.0343478	0.62646629		Cuta	45.027104	42.0847059	45.4853019	101.338861	31.6314227	15.2887088	16.3584861	52.5004332
Galns	2.91016615	-0.8754473	0.41423025	-2.1134317	0.03456384	NA		Galns	3.36023164	5.88280835	3.81694841	6.20442003	1.66481172	2.35210905	0	0
Pusl1	10.4281884	-0.800903	0.37953058	-2.1102462	0.03483715	0.6343092		Pusl1	20.1613899	11.313093	9.54237104	16.5451201	8.3240586	5.88027262	4.81131944	6.84788259
Nrsn1	107.009324	0.6290389	0.29845705	2.10763625	0.03506247	0.6367093		Nrsn1	66.5325865	91.8623149	88.4259716	74.4530404	121.531256	96.436471	223.245222	93.5877288
Aup1	32.1665553	-0.7031085	0.3336943	-2.1070437	0.0351138	0.6367093		Aup1	34.2743627	53.8503226	40.0779583	35.1583802	18.3129289	18.8168724	13.4716944	43.3699231
AI593442	187.08843	0.52906541	0.25115097	2.10656323	0.03515546	0.6367093		AI593442	136.425405	153.858064	157.449122	146.837941	196.447783	204.633487	165.509389	335.546247
Rmnd5b	21.7893577	-0.7057323	0.33528502	-2.1048729	0.03530238	0.6367093		Rmnd5b	33.6023164	23.5312334	20.9932163	37.2265202	21.6425524	14.1126543	16.3584861	6.84788259
Mfge8	53.6112709	-0.8266267	0.39275601	-2.1046824	0.03531897	0.6367093		Mfge8	60.4841696	127.159165	78.8836006	33.0902402	71.586904	16.4647633	11.5471667	29.6741579
Pde1c	16.5174376	0.72741338	0.346	2.10235083	0.03552255	0.6367093		Pde1c	10.7527413	11.313093	13.6773985	10.3407001	14.9833055	31.7534722	27.9056528	11.4131377
Mertk	12.1413989	-0.8753228	0.41635692	-2.1023376	0.03552372	0.6367093		Mertk	16.1291119	40.274611	19.0847421	0	9.98887032	5.88027262	5.77358333	0
9630033F20Rik	23.2398278	0.72187284	0.34349608	2.10154608	0.03559306	0.6367093		9630033F20Ri	20.8334362	15.3858064	18.448584	6.20442003	41.620293	21.1689814	21.1698055	41.0872956
Gria1	435.778171	0.69130271	0.32898696	2.10130729	0.035614	0.6367093		Gria1	275.538995	399.12592	347.342306	217.154701	394.560378	1058.44907	449.377236	344.676757
Sipa1l1	270.4343	0.56586021	0.26932888	2.10100084	0.03564089	0.6367093		Sipa1l1	191.533204	249.793093	213.112953	188.200741	497.778704	308.126285	213.622583	301.306834

GeneID	Base mean	log2(FC)	StdErr	Wald-Stats	P-value	P-adj		GeneID	Normalised expression for Chow#1	Normalised expression for Chow#2	Normalised expression for Chow#3	Normalised expression for Chow#4	Normalised expression for HFD#1	Normalised expression for HFD#2	Normalised expression for HFD#3	Normalised expression for HFD#4
Fermt2	31.3011931	-0.7722884	0.36775236	-2.1000231	0.03572681	0.6367093		Fermt2	62.5003085	49.7776091	42.6225906	14.4769801	19.9777406	28.2253086	25.981125	6.84788259
Phf14	23.4343064	0.66730775	0.317782	2.09989159	0.03573838	0.6367093		Phf14	20.8334362	15.8383302	16.2220308	16.5451201	19.9777406	39.9858538	37.5282917	20.5436478
Gpm6b	281.698774	-0.7706801	0.36701947	-2.0998344	0.03574341	0.6367093		Gpm6b	295.700384	662.494725	432.587487	128.224681	134.849749	269.316486	229.981069	100.435611
Cmtm6	8.89044917	-0.8726449	0.41588791	-2.0982695	0.03588135	0.63810293		Cmtm6	5.37637063	17.1959013	11.4508452	20.6814001	13.3184938	1.17605452	1.92452778	0
Abcc4	3.63613858	-0.8564266	0.40836342	-2.0972168	0.03597439	NA		Abcc4	4.03227797	15.8383302	4.45310648	0	1.66481172	1.17605452	1.92452778	0
Mrpl9	20.0385298	0.65718651	0.31344227	2.0966748	0.03602237	0.63859698		Mrpl9	18.8172972	13.1231878	11.1327662	16.5451201	24.9721758	22.345036	25.981125	27.3915304
Itpkb	31.3809721	-0.8237624	0.39290316	-2.0966042	0.03602863	0.63859698		Itpkb	29.5700384	32.5817078	18.766663	97.2025805	34.9610461	11.7605452	12.5094306	13.6957652
Sh3rf2	4.73309301	-0.8719922	0.4163145	-2.0945516	0.03621087	0.64076462		Sh3rf2	11.4247876	7.69290322	10.1785291	0	3.32962344	2.35210905	2.88679167	0
Mob1b	21.6396192	0.62826707	0.30015959	2.09311012	0.03633933	0.64197472		Mob1b	19.4893435	15.8383302	16.2220308	12.4088401	21.6425524	27.0492541	30.7924444	29.6741579
Slc6a9	37.2704529	-0.7507507	0.35894957	-2.0915214	0.03648135	0.64342022		Slc6a9	30.2420848	52.4927514	31.4898244	84.7937404	33.2962344	22.345036	11.5471667	31.9567854
Ifitm2	3.24166917	-0.8685638	0.41535085	-2.0911568	0.03651401	NA		Ifitm2	5.37637063	8.14542694	5.08926455	2.06814001	3.32962344	0	1.92452778	0
Chil1	3.21919288	-0.8441227	0.40371965	-2.0908636	0.03654029	NA		Chil1	10.7527413	4.07271347	2.22655324	4.13628002	0	0	0	4.56525506
Gapdh	110.400102	-0.6812184	0.32606132	-2.089234	0.03668666	0.64593459		Gapdh	232.52803	136.209639	136.455906	59.9760603	69.9220923	65.8590533	100.075444	82.1745911
Zxdc	21.7043248	0.67302411	0.32230491	2.08815964	0.03678344	0.64593459		Zxdc	14.1129729	12.6706641	15.5858727	20.6814001	28.3017992	29.4013631	16.3584861	36.5220405
Sptan1	278.79288	0.70772382	0.33898413	2.08777863	0.03681781	0.64593459		Sptan1	244.624864	188.702391	181.941208	163.383061	740.841216	231.682741	246.339555	232.828008
Slc48a1	73.4803501	-0.6530222	0.31286266	-2.0872488	0.03686565	0.64593459		Slc48a1	88.038069	111.320835	62.0254117	109.611421	74.9165274	28.2253086	45.2264028	68.4788259
Pofut2	17.2868865	-0.715973	0.34329367	-2.0855991	0.03701496	0.64602306		Pofut2	16.1291119	30.3190892	20.3570582	22.7495401	9.98887032	14.1126543	8.660375	15.9783927
Arl2bp	102.361506	-0.4850487	0.23258757	-2.0854456	0.03702887	0.64602306		Arl2bp	121.640385	104.080455	114.826531	144.769801	76.5813391	65.8590533	95.264125	95.8703563
Zbed3	5.27207972	-0.8483514	0.40684603	-2.0851903	0.03705203	0.64602306		Zbed3	10.7527413	7.69290322	8.2700549	4.13628002	1.66481172	3.52816357	3.84905555	2.28262753
Tmem163	9.7148024	-0.8238495	0.39534431	-2.0838785	0.03717122	0.64622146		Tmem163	10.0806949	22.6261859	11.7689243	10.3407001	8.3240586	9.40843619	2.88679167	2.28262753
Itfg3	5.97725317	-0.85771	0.41162158	-2.0837344	0.03718434	0.64622146		Itfg3	5.37637063	12.2181404	8.58813393	8.27256004	6.65924688	1.17605452	0.96226389	4.56525506
Olfml3	4.36936592	-0.8643858	0.41506939	-2.0825091	0.03729599	NA		Olfml3	7.39250961	14.9332827	5.72542262	0	1.66481172	2.35210905	2.88679167	0
Cyp4f13	1.26566543	-0.7435068	0.35715137	-2.0817693	0.03736355	NA		Cyp4f13	2.68818531	3.62018975	3.81694841	0	0	0	0	0
Gsto2	1.08410157	0.72415597	0.34787555	2.0816524	0.03737423	NA		Gsto2	0	0	0.31807903	0	3.32962344	1.17605452	3.84905555	0
Mtfr1l	54.6176773	-0.5857121	0.28148697	-2.0807789	0.03745415	0.6495387		Mtfr1l	66.5325865	62.9007969	64.888123	74.4530404	26.6369875	30.5774176	53.8867778	57.0656883
Mad2l1bp	4.18314062	-0.8598072	0.41323864	-2.0806554	0.03746546	NA		Mad2l1bp	8.06455594	5.88280835	5.08926455	6.20442003	1.66481172	2.35210905	1.92452778	2.28262753
Htr1b	7.80456557	-0.827063	0.39756645	-2.0803138	0.03749676	0.6495387		Htr1b	10.7527413	13.1231878	10.4966081	8.27256004	4.99443516	4.7042181	0.96226389	9.13051012
Slc18a3	1.25334588	-0.7438085	0.35765618	-2.0796746	0.03755539	NA		Slc18a3	3.36023164	3.16766603	3.49886938	0	0	0	0	0
A730043L09Rik	1.32184482	0.76875695	0.36974227	2.07916978	0.03760175	NA		A730043L09Ri	0	0.45252372	0.31807903	0	0	2.35210905	2.88679167	4.56525506
Sh3pxd2b	11.4405781	-0.7770371	0.37377201	-2.0789065	0.03762595	0.65072185		Sh3pxd2b	16.1291119	20.8160911	14.6316356	10.3407001	13.3184938	8.23238167	5.77358333	2.28262753
Kcnip3	62.2626886	-0.623462	0.30017944	-2.0769643	0.03780486	0.65091719		Kcnip3	88.7101153	101.817837	82.3824699	35.1583802	51.6091633	45.8661264	42.3396111	50.2178057
Arx	41.0089425	0.71334549	0.34346301	2.07692083	0.03780887	0.65091719		Arx	31.5861774	14.9332827	26.7186389	41.3628002	41.620293	38.8097993	39.4528194	93.5877288
Prlr	8.82804769	0.85966639	0.41393781	2.07680083	0.03781995	0.65091719		Prlr	1.34409266	7.69290322	6.36158069	2.06814001	3.32962344	24.697145	18.2830139	6.84788259
Gmppb	9.81548396	0.80480529	0.38789597	2.07479674	0.03800538	0.65294702		Gmppb	8.06455594	2.26261859	6.04350166	8.27256004	19.9777406	10.5844907	9.62263889	13.6957652
Cacng8	53.9856307	0.66405094	0.3202985	2.07322525	0.03815132	0.65294702		Cacng8	34.9464091	37.106945	34.9886938	49.6353602	103.218327	43.5140174	39.4528194	89.0224737
Arrdc3	20.4013376	-0.7195863	0.34718665	-2.0726209	0.03820758	0.65294702		Arrdc3	26.8818531	29.4140417	29.8994292	22.7495401	8.3240586	21.1689814	20.2075417	4.56525506
Tmem63a	6.71999024	-0.8594855	0.41479138	-2.072091	0.03825695	0.65294702		Tmem63a	5.37637063	14.9332827	7.95197586	12.4088401	9.98887032	1.17605452	1.92452778	0
Tspan15	5.62546273	-0.8609772	0.41553729	-2.0719613	0.03826905	0.65294702		Tspan15	6.04841696	10.8605693	8.2700549	8.27256004	0	4.7042181	0	6.84788259
Cyth3	37.6788458	0.53935057	0.26035684	2.07158207	0.03830443	0.65294702		Cyth3	33.6023164	27.1514231	29.2632712	31.0221002	41.620293	47.042181	52.9245139	38.804668
Adk	28.0441817	-0.6840747	0.33048728	-2.0698971	0.03846198	0.65300122		Adk	43.6830113	38.9170398	34.6706148	28.9539601	11.653682	22.345036	32.7169722	11.4131377
Pafah1b2	218.462591	0.4127042	0.199494	2.06875498	0.03856908	0.65300122		Pafah1b2	186.828879	156.573207	192.437816	208.882141	213.0959	245.795396	283.867847	260.219539
Apex1	30.5392658	0.64319872	0.31095375	2.06847067	0.03859579	0.65300122		Apex1	22.1775288	15.8383302	21.9474534	31.0221002	39.9554813	25.8731995	32.7169722	54.7830607
Lgals1	12.4476452	0.83385342	0.40320931	2.06804109	0.03863616	0.65300122		Lgals1	9.4086486	8.14542694	9.54237104	0	8.3240586	12.9365998	37.5282917	13.6957652
Smg6	38.6432777	0.64214651	0.31070748	2.06672365	0.0387602	0.65300122		Smg6	22.1775288	21.7211385	23.2197695	51.7035003	49.9443516	55.2745626	39.4528194	45.6525506
Tom1l1	9.57811898	-0.7935772	0.38404284	-2.0663767	0.03879292	0.65300122		Tom1l1	12.0968339	9.05047438	16.2220308	16.5451201	4.99443516	3.52816357	9.62263889	4.56525506
Ccdc28a	12.4661819	-0.8334943	0.40370965	-2.0645884	0.03896196	0.65300122		Ccdc28a	12.0968339	18.1009488	11.1327662	33.0902402	4.99443516	5.88027262	14.4339583	0
Plec	98.3574559	0.85284444	0.41332792	2.06336036	0.0390784	0.65300122		Plec	34.2743627	27.6039469	27.990955	113.747701	389.565943	45.8661264	33.6792361	114.131377
Rlbp1	7.55092581	-0.8374183	0.40587346	-2.0632498	0.0390889	0.65300122		Rlbp1	17.4732045	11.7656167	10.1785291	4.13628002	6.65924688	1.17605452	6.73584722	2.28262753
Fmo5	2.27645227	-0.8413016	0.40775998	-2.0632275	0.03909102	NA		Fmo5	6.04841696	2.71514231	4.13502745	2.06814001	0	0	0.96226389	2.28262753
Ifngr1	4.51221599	-0.8586417	0.41620808	-2.0630107	0.03911162	NA		Ifngr1	5.37637063	13.5757116	6.99773876	2.06814001	1.66481172	3.52816357	2.88679167	0
Kctd8	18.5866144	0.6750622	0.32722766	2.06297414	0.03911509	0.65300122		Kctd8	18.8172972	10.4080455	11.7689243	12.4088401	16.6481172	25.8731995	23.0943333	29.6741579
Snap23	1.16017318	-0.7531107	0.36507012	-2.0629207	0.03912016	NA		Snap23	3.36023164	2.26261859	1.59039517	2.06814001	0	0	0	0
Prokr2	10.1713665	0.84596195	0.41018912	2.06237051	0.03917247	0.65300122		Prokr2	2.68818531	7.2403795	7.63389683	4.13628002	9.98887032	36.4576902	8.660375	4.56525506
Tll1	4.44986601	0.85854562	0.41631807	2.0622348	0.03918539	NA		Tll1	0.67204633	1.35757116	1.27231614	6.20442003	11.653682	4.7042181	2.88679167	6.84788259
Smarca2	217.668057	0.43494272	0.21091698	2.06215129	0.03919333	0.65300122		Smarca2	209.006408	173.316584	188.938946	159.246781	244.727323	315.182612	238.641444	212.28436
Med28	56.6620764	-0.6582225	0.31921268	-2.0620186	0.03920597	0.65300122		Med28	55.1077989	61.0907021	67.7508343	103.407001	36.6258578	22.345036	38.4905555	68.4788259
Stom	4.1426818	-0.8582776	0.41626159	-2.0618708	0.03922004	NA		Stom	3.36023164	13.1231878	4.77118552	4.13628002	3.32962344	1.17605452	0.96226389	2.28262753
Slc39a7	40.6436244	0.72289071	0.35069612	2.06130226	0.03927421	0.65300122		Slc39a7	26.8818531	24.8888045	20.9932163	39.2946602	88.2350212	38.8097993	22.1320694	63.9135709
Alox8	5.94994907	0.82586977	0.40068208	2.06115976	0.0392878	0.65300122		Alox8	3.36023164	2.71514231	3.49886938	4.13628002	13.3184938	4.7042181	6.73584722	9.13051012
Suclg2	4.95058185	-0.847347	0.41110629	-2.0611385	0.03928983	0.65300122		Suclg2	8.73660227	6.78785578	6.36158069	8.27256004	0	2.35210905	4.81131944	2.28262753
Sergef	16.5677404	0.72996633	0.35425848	2.06054722	0.03934626	0.65300122		Sergef	11.4247876	10.4080455	7.95197586	16.5451201	31.6314227	10.5844907	21.1698055	22.8262753
Tardbp	62.7599076	0.51782844	0.25155567	2.05850432	0.03954175	0.65437138		Tardbp	49.059382	46.6099431	55.9819101	51.7035003	59.9332219	79.9717076	97.1886527	61.6309433
2310047M10Rik	21.9002803	0.77830982	0.37817304	2.05807857	0.03958259	0.65437138		2310047M10R	16.1291119	7.2403795	12.0870033	20.6814001	41.620293	14.1126543	15.3962222	47.9351782
Ppig	47.408439	0.62177664	0.30216083	2.05776717	0.03961249	0.65437138		Ppig	36.2905017	28.961518	42.9406697	35.1583802	36.6258578	88.2040893	65.4339444	45.6525506
Ssr2	30.1090949	-0.6855896	0.33342866	-2.0561808	0.03976509	0.65531392		Ssr2	34.2743627	58.8280835	33.3982986	26.8858201	21.6425524	28.2253086	12.5094306	25.1089028
Ybx3	6.05286519	-0.831339	0.40436747	-2.0558998	0.03979217	0.65531392		Ybx3	8.73660227	10.4080455	8.58813393	8.27256004	3.32962344	2.35210905	6.73584722	0
Emp2	9.05643082	-0.8367552	0.40732801	-2.0542541	0.0399511	0.65691908		Emp2	8.73660227	22.1736622	16.2220308	4.13628002	9.98887032	4.7042181	1.92452778	4.56525506
Hist1h4f	12.8469871	-0.751412	0.36593566	-2.0533991	0.04003389	0.65726911		Hist1h4f	18.8172972	18.5534725	17.1762679	14.4769801	6.65924688	3.52816357	14.4339583	9.13051012
Gpr88	64.3334795	-0.7501337	0.36551391	-2.0522714	0.0401433	0.65805455		Gpr88	114.919922	79.1916508	117.053085	31.0221002	31.6314227	65.8590533	20.2075417	54.7830607
Malt1	1.78237039	-0.8013836	0.39051175	-2.0521369	0.04015636	NA		Malt1	2.01613899	3.16766603	1.90847421	6.20442003	0	0	0.96226389	0
Ccdc57	1.28886375	0.7759173	0.37824569	2.05135796	0.0402321	NA		Ccdc57	0.67204633	0.45252372	0	0	1.66481172	2.35210905	2.88679167	2.28262753
Enpp1	1.63516457	-0.7840457	0.38235675	-2.0505607	0.04030974	NA		Enpp1	2.68818531	4.97776091	4.45310648	0	0	0	0.96226389	0
Hist1h3e	20.5171211	-0.7196871	0.35110297	-2.0497893	0.040385	0.65989267		Hist1h3e	28.2259458	26.6988994	23.5378486	28.9539601	16.6481172	3.52816357	18.2830139	18.2610202
Lamp1	145.074979	-0.7572836	0.36949836	-2.049491	0.04041412	0.65989267		Lamp1	168.683628	319.481746	215.975664	76.5211804	64.9276571	177.584233	89.4905416	47.9351782
Lhx2	37.9438522	-0.7985937	0.38970626	-2.0492195	0.04044065	0.65989267		Lhx2	85.3498837	66.5209867	48.0299342	10.3407001	29.966611	11.7605452	35.6037639	15.9783927
Rab4b	42.4861392	-0.5717989	0.27928647	-2.0473563	0.04062311	0.6618595		Rab4b	48.3873356	46.1574193	44.2129858	70.3167603	41.620293	27.0492541	27.9056528	34.239413
Mars2	9.73914766	0.76187633	0.37244088	2.04563027	0.04079276	0.66326697		Mars2	9.4086486	4.07271347	7.31581779	4.13628002	9.98887032	12.9365998	16.3584861	13.6957652
Rbm22	13.9553734	-0.7607267	0.3719543	-2.0452155	0.04083361	0.66326697		Rbm22	24.1936678	22.1736622	15.5858727	14.4769801	11.653682	5.88027262	15.3962222	2.28262753
Cd248	2.3211802	-0.8242467	0.40344137	-2.0430395	0.04104853	NA		Cd248	2.01613899	8.14542694	3.49886938	2.06814001	1.66481172	1.17605452	0	0
Serpinf1	2.09692507	-0.8164284	0.39979485	-2.0421184	0.04113979	NA		Serpinf1	4.03227797	6.78785578	3.81694841	0	0	1.17605452	0.96226389	0
Tmem125	1.94456255	-0.7909239	0.38746595	-2.0412733	0.04122367	NA		Tmem125	2.01613899	2.71514231	1.59039517	8.27256004	0	0	0.96226389	0
Hist1h3f	12.7038969	-0.732958	0.35908567	-2.0411785	0.04123308	0.6686917		Hist1h3f	20.1613899	13.5757116	13.9954775	20.6814001	6.65924688	5.88027262	11.5471667	9.13051012
Plagl2	7.36761409	-0.8167614	0.40033246	-2.0402077	0.04132964	0.6686917		Plagl2	9.4086486	12.6706641	9.224292	10.3407001	9.98887032	1.17605452	3.84905555	2.28262753
Myo5a	468.515235	0.46153222	0.22625519	2.03987456	0.04136282	0.6686917		Myo5a	423.389187	372.879544	406.505006	349.515662	532.739751	726.801696	543.679097	392.611935
Cux2	69.173743	0.66470533	0.32600992	2.03891134	0.04145888	0.6686917		Cux2	53.0916599	33.9392789	39.7598793	74.4530404	93.2294563	63.5069443	53.8867778	141.522907
Pwp2	15.6176691	0.75575592	0.37072565	2.03858548	0.04149142	0.6686917		Pwp2	10.0806949	4.97776091	11.4508452	14.4769801	23.3073641	12.9365998	13.4716944	34.239413
Zmat4	49.7007572	0.64260723	0.3153019	2.03806961	0.04154297	0.6686917		Zmat4	38.3066407	38.0119924	46.439539	22.7495401	36.6258578	90.5561984	76.9811111	47.9351782
Rsf1	44.5871934	0.64227042	0.31523378	2.03744162	0.04160581	0.66869603		Rsf1	34.2743627	38.4645161	29.2632712	31.0221002	43.2851047	84.6759257	68.3207361	27.3915304
Usp49	9.57903976	0.75933183	0.37306559	2.03538428	0.04181222	0.67060626		Usp49	7.39250961	5.43028463	5.72542262	6.20442003	14.9833055	16.4647633	6.73584722	13.6957652
Slc35a5	27.3311259	-0.7248114	0.35636557	-2.0338985	0.04196183	0.67060626		Slc35a5	24.1936678	63.8058444	37.215247	18.6132601	16.6481172	22.345036	22.1320694	13.6957652
Nom1	16.597236	0.67979836	0.33426693	2.0336991	0.04198194	0.67060626		Nom1	16.8011582	8.59795066	11.4508452	10.3407001	28.3017992	18.8168724	20.2075417	18.2610202
Phgdh	7.61208129	-0.8441183	0.41530126	-2.0325446	0.04209855	0.67060626		Phgdh	10.7527413	3.62018975	10.8146872	20.6814001	8.3240586	1.17605452	0.96226389	4.56525506
Homer2	44.7971842	0.57396085	0.28245751	2.03202547	0.04215108	0.67060626		Homer2	33.6023164	35.2968501	44.2129858	24.8176801	53.2739751	62.3308898	68.3207361	36.5220405
Chrd	3.25378953	0.84598446	0.41636713	2.03182334	0.04217154	NA		Chrd	0.67204633	2.26261859	2.22655324	0	3.32962344	5.88027262	4.81131944	6.84788259
Map1lc3b	98.5258198	-0.55519	0.27325721	-2.0317486	0.04217912	0.67060626		Map1lc3b	108.871505	111.773359	116.098848	142.701661	104.883138	43.5140174	82.7546944	77.6093361
Nudt7	1.78602651	-0.7913312	0.38948867	-2.0317181	0.04218221	NA		Nudt7	1.34409266	3.16766603	6.04350166	2.06814001	1.66481172	0	0	0
Arhgef26	18.3364121	-0.6954746	0.34236091	-2.0314078	0.04221365	0.67060626		Arhgef26	22.1775288	25.793852	22.2655324	26.8858201	13.3184938	8.23238167	21.1698055	6.84788259
Tmem231	10.3305279	-0.7793169	0.38372554	-2.0309226	0.04226285	0.67060626		Tmem231	7.39250961	19.0059962	11.7689243	18.6132601	8.3240586	5.88027262	4.81131944	6.84788259
Zfp36l1	25.6782784	-0.8256652	0.40666047	-2.0303552	0.04232045	0.67060626		Zfp36l1	28.8979921	28.0564706	27.0367179	64.1123403	23.3073641	0	13.4716944	20.5436478
Nabp2	54.4083478	-0.6825599	0.33622916	-2.0300437	0.0423521	0.67060626		Nabp2	95.4305786	75.5714611	51.5288036	57.9079203	21.6425524	22.345036	60.622625	50.2178057
Chd6	80.0796643	0.53136263	0.26212912	2.02710261	0.04265192	0.67435461		Chd6	63.1723549	59.7331309	59.7988585	74.4530404	106.54795	131.718107	72.1697916	73.044081
Ryr2	128.499369	0.66970045	0.33050631	2.02628644	0.04273544	0.67467709		Ryr2	106.855366	100.912789	92.560999	68.2486203	278.023557	197.57716	103.9245	79.8919636
Dus2	5.48575814	0.81387065	0.40196842	2.02471292	0.04289685	0.67583052		Dus2	5.37637063	2.26261859	2.86271131	2.06814001	8.3240586	9.40843619	6.73584722	6.84788259
Ptn	46.7699828	-0.8077344	0.39901082	-2.0243421	0.04293497	0.67583052		Ptn	63.8444012	107.700645	82.0643909	10.3407001	16.6481172	52.9224536	26.9433889	13.6957652
Sall1	12.2155295	-0.8066799	0.39861364	-2.0237138	0.04299961	0.67583677		Sall1	25.5377605	22.1736622	10.8146872	10.3407001	8.3240586	1.17605452	12.5094306	6.84788259
Slc7a11	25.2489034	-0.7321862	0.3619115	-2.0231085	0.04306196	0.67583677		Slc7a11	24.8657142	51.587704	40.7141164	16.5451201	24.9721758	15.2887088	21.1698055	6.84788259
Leo1	14.7900813	0.81908573	0.40498742	2.02249671	0.04312506	0.67583677		Leo1	7.39250961	4.97776091	8.58813393	14.4769801	3.32962344	44.6900719	21.1698055	13.6957652
Gpld1	37.9005569	-0.6247879	0.30919144	-2.0207153	0.04330924	0.6777294		Gpld1	32.9302701	54.3028463	40.0779583	66.1804803	28.3017992	31.7534722	33.6792361	15.9783927
Trp53bp1	87.455941	0.58168941	0.28797288	2.01994512	0.04338908	0.6779861		Trp53bp1	62.5003085	75.1189373	71.5677828	59.9760603	74.9165274	174.05607	85.6414861	95.8703563
Kctd18	5.04950475	0.82464065	0.40923187	2.01509392	0.04389481	0.68488719		Kctd18	2.01613899	1.81009488	3.18079035	4.13628002	8.3240586	4.7042181	4.81131944	11.4131377
Pdgfra	23.2489372	-0.8254206	0.40989341	-2.0137446	0.04403636	0.68503986		Pdgfra	15.4570656	79.1916508	34.0344567	6.20442003	18.3129289	17.6408179	10.5849028	4.56525506
Dcaf5	89.0711264	0.47789326	0.23733019	2.01362185	0.04404925	0.68503986		Dcaf5	72.5810035	75.5714611	61.3892537	82.7256004	106.54795	107.020962	78.9056389	127.827142

GeneID	Base mean	log2(FC)	StdErr	Wald-Stats	P-value	P-adj	GeneID	Normalised expression for Chow#1	Normalised expression for Chow#2	Normalised expression for Chow#3	Normalised expression for Chow#4	Normalised expression for HFD#1	Normalised expression for HFD#2	Normalised expression for HFD#3	Normalised expression for HFD#4
Klc1	383.741867	0.5006717	0.24869832	2.01316877	0.04409688	0.68503986	Klc1	328.630655	240.290095	278.319155	397.082882	479.465775	356.344521	384.905555	604.896296
Etnppl	7.67134434	-0.8106221	0.40280352	-2.0124505	0.04417247	0.68521828	Etnppl	6.04841696	11.313093	8.58813393	18.6132601	8.3240586	2.35210905	3.84905555	2.28262753
Vmac	8.23487422	0.76932222	0.38251094	2.01124239	0.04429986	0.68562868	Vmac	5.37637063	5.43028463	5.08926455	4.13628002	16.6481172	5.88027262	9.62263889	13.6957652
Tmem186	8.77374367	0.77334684	0.38456156	2.01098322	0.04432723	0.68562868	Tmem186	6.72046328	2.71514231	6.99773876	6.20442003	16.6481172	10.5844907	13.4716944	6.84788259
Ryr1	4.06100472	0.8331597	0.41443719	2.0103401	0.04439521	NA	Ryr1	2.68818531	2.71514231	1.90847421	0	9.98887032	3.52816357	4.81131944	6.84788259
Gid4	40.2711256	0.528211	0.26290152	2.00915919	0.04452025	0.68704331	Gid4	38.978687	31.6766603	30.8536663	24.8176801	46.6147282	41.1619083	50.9999861	57.0656883
Pde4b	68.9717747	-0.549836	0.27369942	-2.0089046	0.04454725	0.68704331	Pde4b	97.4467176	95.029981	86.8355764	55.8397803	54.9387868	65.8590533	61.5848889	34.239413
Aldh9a1	24.8789272	-0.7752117	0.3863669	-2.0064132	0.04481218	0.69013339	Aldh9a1	24.1936678	18.5534725	20.6751372	74.4530404	19.9777406	12.9365998	7.69811111	20.5436478
Zfp362	24.4447802	-0.6292802	0.31376895	-2.0055527	0.04490399	0.69055233	Zfp362	26.8818531	37.106945	26.0824808	31.0221002	18.3129289	15.2887088	13.4716944	27.3915304
Mgst1	2.48138672	-0.8152872	0.40667154	-2.0047805	0.04498652	NA	Mgst1	1.34409266	4.07271347	4.77118552	6.20442003	0	1.17605452	0	2.28262753
Heatr3	16.2067186	0.66191348	0.3302165	2.00448339	0.0450183	0.69131558	Heatr3	12.7688802	10.4080455	14.6316356	8.27256004	24.9721758	24.697145	20.2075417	13.6957652
Rsph4a	3.76870142	-0.8344486	0.41636657	-2.0041201	0.0450572	NA	Rsph4a	8.06455594	7.2403795	6.36158069	2.06814001	0	3.52816357	2.88679167	0
Nme5	3.99930542	-0.8323839	0.41539482	-2.003838	0.04508742	NA	Nme5	9.4086486	4.97776091	7.31581779	2.06814001	1.66481172	2.35210905	1.92452778	2.28262753
Serpinb8	2.26103244	0.7967646	0.39812756	2.00127968	0.04536226	NA	Serpinb8	0.67204633	0.45252372	1.27231614	0	4.99443516	0	3.84905555	6.84788259
Tulp4	213.378789	0.421776	0.21076672	2.00115089	0.04537613	0.69581075	Tulp4	157.258841	167.433776	172.080758	227.495401	281.353181	255.203832	208.811264	237.393263
Pde9a	15.1342626	0.66753444	0.33381444	1.99971709	0.04553082	0.69618721	Pde9a	12.0968339	7.2403795	10.8146872	14.4769801	19.9777406	17.6408179	18.2830139	20.5436478
Atp6v1e1	199.50576	0.45610621	0.22808551	1.99971584	0.04553096	0.69618721	Atp6v1e1	177.420231	129.421784	201.662108	153.042361	189.788536	234.03485	273.282944	237.393263
Pxmp2	2.94118948	-0.8249766	0.41259523	-1.9994817	0.04555626	NA	Pxmp2	5.37637063	5.43028463	2.22655324	6.20442003	3.32962344	0	0.96226389	0
Igfbp7	3.603673	-0.8324891	0.4163673	-1.9994103	0.04556397	NA	Igfbp7	4.03227797	9.95552182	5.72542262	2.06814001	1.66481172	1.17605452	1.92452778	2.28262753
Gdpd2	3.23928851	-0.8313094	0.41581225	-1.9992423	0.04558214	NA	Gdpd2	3.36023164	6.33533207	3.18079035	8.27256004	1.66481172	1.17605452	1.92452778	0
Tmub1	14.4451935	-0.7047354	0.35293862	-1.9967647	0.04585075	0.70007548	Tmub1	19.4893435	21.7211385	14.3135566	18.6132601	11.653682	7.05632714	6.73584722	15.9783927
Klhl40	2.83865969	0.82911966	0.41534632	1.99621285	0.04591076	NA	Klhl40	2.01613899	0.90504744	1.59039517	0	8.3240586	4.7042181	2.88679167	2.28262753
Slc8b1	2.8174106	-0.827658	0.4148856	-1.9949065	0.04605308	NA	Slc8b1	4.03227797	5.43028463	3.81694841	4.13628002	1.66481172	1.17605452	0	2.28262753
Mcm3ap	27.341875	0.5746633	0.2882238	1.99380931	0.0461729	0.70369503	Mcm3ap	23.5216215	18.1009488	20.6751372	22.7495401	43.2851047	27.0492541	33.6792361	29.6741579
Mapk8ip3	335.282154	0.62608122	0.31413271	1.99304689	0.04625631	0.70369503	Mapk8ip3	223.119381	165.623681	210.568321	401.219162	625.969207	317.534722	277.132	461.090761
Cbx5	344.629886	0.4443914	0.22300051	1.99278194	0.04628533	0.70369503	Cbx5	250.67328	270.15666	312.98977	320.561702	337.956779	483.358409	468.622514	312.719972
S100a6	4.14620993	-0.8233119	0.41347706	-1.9911912	0.04645987	NA	S100a6	3.36023164	4.07271347	5.08926455	14.4769801	4.99443516	1.17605452	0	0
Arfgef2	66.3312393	0.55232782	0.27740701	1.99103776	0.04647674	0.70418322	Arfgef2	48.3873356	60.1856546	43.2587487	57.9079203	96.5590798	107.020962	62.5471528	54.7830607
Nop10	32.6948609	-0.6882705	0.34571014	-1.9908889	0.0464931	0.70418322	Nop10	36.2905017	42.5372296	43.8949068	49.6353602	8.3240586	23.5210905	41.3773472	15.9783927
Abhd12	133.383995	-0.5466305	0.27459362	-1.9906889	0.0465151	0.70418322	Abhd12	141.801775	223.094193	176.215785	103.407001	91.5646446	132.894161	88.5282777	109.566121
Mif4gd	7.45347178	-0.8274007	0.41579809	-1.9899099	0.04660086	0.70444249	Mif4gd	9.4086486	5.88280835	5.40734359	24.8176801	6.65924688	0	2.88679167	4.56525506
Nek6	18.5461322	-0.6765255	0.3400759	-1.9893368	0.04666404	0.70444249	Nek6	18.1452509	25.3413283	23.8559276	31.0221002	11.653682	16.4647633	17.32075	4.56525506
Hnrnpu	139.943573	0.5709772	0.28712756	1.98858375	0.04674717	0.704702	Hnrnpu	144.489961	105.438027	114.190373	68.2486203	143.173808	243.443286	181.867875	118.696632
Barhl2	3.23467337	-0.8273149	0.41609611	-1.9882784	0.0467809	NA	Barhl2	7.39250961	4.97776091	3.49886938	4.13628002	1.66481172	0	1.92452778	2.28262753
Cntnap4	36.6937342	0.73554266	0.37007488	1.9875509	0.04686138	0.70542879	Cntnap4	27.5538995	29.8665654	25.1282437	14.4769801	29.966611	105.844907	35.6037639	25.1089028
Abcc9	2.68086049	-0.7860611	0.39636386	-1.9831805	0.04734727	NA	Abcc9	0.67204633	9.5029981	2.22655324	6.20442003	1.66481172	1.17605452	0	0
Pigs	49.1009233	-0.6336995	0.31966483	-1.9823873	0.04743591	0.71284974	Pigs	40.3227797	76.4765085	54.7095939	74.4530404	44.9499164	28.2253086	21.1698055	52.5004332
Mir5133	2.04204421	0.77546762	0.3911983	1.98228782	0.04744704	NA	Mir5133	0.67204633	0	0.31807903	2.06814001	3.32962344	1.17605452	1.92452778	6.84788259
Gm14305	17.6188278	0.80165789	0.40457697	1.98147188	0.04753838	0.71284974	Gm14305	10.7527413	12.6706641	14.6316356	2.06814001	11.653682	29.4013631	52.9245139	6.84788259
Eml6	21.9734325	0.6355858	0.32082006	1.98112864	0.04757685	0.71284974	Eml6	21.5054825	14.9332827	15.5858727	12.4088401	39.9554813	22.345036	30.7924444	18.2610202
Acbd3	36.2934202	0.56559895	0.28560692	1.98034049	0.04766528	0.71284974	Acbd3	27.5538995	19.9110436	33.7163777	33.0902402	36.6258578	54.0985081	44.2641389	41.0872956
Tdrd3	11.3128744	0.73506216	0.37121732	1.9801397	0.04768783	0.71284974	Tdrd3	5.37637063	8.59795066	11.1327662	4.13628002	13.3184938	17.6408179	21.1698055	9.13051012
Fosl1	1.59766304	0.72909755	0.36824521	1.97992407	0.04771206	NA	Fosl1	0	0	0.9542371	0	1.66481172	2.35210905	0.96226389	6.84788259
Inpp4a	217.456821	0.44118413	0.22300664	1.97834525	0.04788977	0.71401232	Inpp4a	159.947026	161.550968	175.897706	231.631681	288.012428	235.210905	202.075417	285.328441
Twsg1	16.8884837	-0.7408255	0.3744831	-1.9782615	0.04789922	0.71401232	Twsg1	29.5700384	33.0342315	20.0389792	8.27256004	6.65924688	15.2887088	15.3962222	6.84788259
Lrig3	1.09297297	-0.7126564	0.36030118	-1.9779465	0.04793475	NA	Lrig3	2.68818531	2.71514231	1.27231614	2.06814001	0	0	0	0
Ccl21c	4.39959727	-0.8188879	0.4140113	-1.9779362	0.04793591	NA	Ccl21c	9.4086486	5.88280835	5.40734359	6.20442003	1.66481172	4.7042181	1.92452778	0
Gm10591	4.39959727	-0.8188879	0.4140113	-1.9779362	0.04793591	NA	Gm10591	9.4086486	5.88280835	5.40734359	6.20442003	1.66481172	4.7042181	1.92452778	0
Gm13304	4.39959727	-0.8188879	0.4140113	-1.9779362	0.04793591	NA	Gm13304	9.4086486	5.88280835	5.40734359	6.20442003	1.66481172	4.7042181	1.92452778	0
Gm21541	4.39959727	-0.8188879	0.4140113	-1.9779362	0.04793591	NA	Gm21541	9.4086486	5.88280835	5.40734359	6.20442003	1.66481172	4.7042181	1.92452778	0
Ahr	7.78069611	0.7743135	0.39155102	1.97755456	0.04797898	0.71420519	Ahr	4.03227797	4.52523719	7.31581779	2.06814001	6.65924688	15.2887088	8.660375	13.6957652
Atxn7l3b	231.599544	0.45340255	0.22959502	1.97479265	0.04829168	0.71740435	Atxn7l3b	165.323397	170.148918	176.533864	260.585641	284.682804	222.274305	255.962194	317.285227
4930506M07Rik	28.0901819	0.63995551	0.32411481	1.97447165	0.04832814	0.71740435	4930506M07R	29.5700384	18.5534725	16.8581888	18.6132601	21.6425524	38.8097993	50.9999861	29.6741579
Ginm1	5.41635934	-0.8214395	0.41632083	-1.9730926	0.04848502	0.71781533	Ginm1	6.04841696	15.8383302	9.86045007	2.06814001	0	4.7042181	4.81131944	0
Crebrf	63.358454	0.5663566	0.28704661	1.97304755	0.04849014	0.71781533	Crebrf	49.7314283	44.3473245	58.2084633	45.4990802	49.9443516	105.844907	96.2263889	57.0656883
Arl6ip4	13.1928648	0.70271527	0.35663332	1.97041397	0.04879095	0.7212692	Arl6ip4	10.0806949	7.2403795	7.31581779	14.4769801	13.3184938	17.6408179	24.0565972	11.4131377
Magt1	3.75395497	-0.8084505	0.4103009	-1.9703844	0.04879434	NA	Magt1	6.04841696	13.1231878	5.40734359	0	0	3.52816357	1.92452778	0
Pdcd2	8.50025271	0.77039963	0.39154275	1.96760031	0.04911404	0.72504269	Pdcd2	5.37637063	4.97776091	5.40734359	4.13628002	9.98887032	4.7042181	10.5849028	22.8262753
Timm21	1.62210639	-0.7488697	0.38062397	-1.9674792	0.04912799	NA	Timm21	3.36023164	2.71514231	5.72542262	0	0	1.17605452	0	0
Gcsh	25.8608305	-0.6891005	0.35033807	-1.9669587	0.04918797	0.72513247	Gcsh	31.5861774	22.6261859	27.672876	55.8397803	9.98887032	15.2887088	27.9056528	15.9783927
Itm2a	6.35989764	-0.816234	0.41518923	-1.9659324	0.04930642	0.72587741	Itm2a	4.7043243	17.648425	13.0412404	2.06814001	3.32962344	5.88027262	1.92452778	2.28262753
Th	14.5864944	-0.7976247	0.40596227	-1.9647754	0.04944024	0.72684634	Th	2.68818531	4.07271347	4.45310648	84.7937404	4.99443516	2.35210905	1.92452778	11.4131377
2010111I01Rik	6.25747227	0.80247629	0.40865673	1.96369284	0.04956572	0.72769019	2010111I01Ri	4.03227797	2.71514231	4.45310648	2.06814001	16.6481172	2.35210905	8.660375	9.13051012
Eif3d	31.0572141	0.57739823	0.29430717	1.96188978	0.04977532	0.72976488	Eif3d	32.9302701	19.9110436	23.5378486	18.6132601	38.2906696	48.2182355	32.7169722	34.239413
Vegfb	56.9062945	-0.5995779	0.30570727	-1.961281	0.04984625	0.72980378	Vegfb	76.6132814	63.8058444	60.7530956	80.6574604	53.2739751	18.8168724	44.2641389	57.0656883
Sfrp5	1.97752179	-0.6630732	0.33868307	-1.9577986	0.05025365	NA	Sfrp5	0	4.52523719	0.9542371	10.3407001	0	0	0	0
Dcxr	2.499374	-0.8007537	0.40902341	-1.957721	0.05026276	NA	Dcxr	3.36023164	3.62018975	2.86271131	6.20442003	1.66481172	0	0	2.28262753
Ttc23	1.3395002	-0.6984812	0.35689125	-1.9571261	0.05033265	NA	Ttc23	2.01613899	0.90504744	1.59039517	6.20442003	0	0	0	0
Tfap4	3.21321284	0.8126221	0.41526769	1.95686332	0.05036354	NA	Tfap4	1.34409266	1.35757116	0.9542371	2.06814001	3.32962344	2.35210905	2.88679167	11.4131377
Rnf40	32.1432128	0.62474906	0.31929516	1.95665059	0.05038856	0.73631149	Rnf40	20.8334362	20.3635674	23.2197695	31.0221002	56.6035985	24.697145	27.9056528	52.5004332
Ogfrl1	123.439487	0.43264192	0.22117596	1.9560983	0.05045358	0.73631149	Ogfrl1	104.167181	113.13093	100.512975	97.2025805	109.877574	150.534979	175.132028	136.957652
1700071M16Rik	3.68212484	-0.8135388	0.41592422	-1.9559785	0.05046769	NA	1700071M16R	5.37637063	9.5029981	7.31581779	0	1.66481172	2.35210905	0.96226389	2.28262753
Trio	141.876773	0.4971417	0.254198	1.95572626	0.05049741	0.73631149	Trio	116.936061	114.488501	131.68472	95.1344405	219.755147	202.281378	133.754681	120.979259
Rpl23a	3.44043831	-0.8107344	0.41488492	-1.9541188	0.05068717	NA	Rpl23a	8.73660227	4.52523719	6.67965972	2.06814001	1.66481172	0	3.84905555	0
Ppp1r1b	93.7719897	-0.7338161	0.37565432	-1.9534344	0.05076814	0.7364447	Ppp1r1b	198.925713	100.007742	134.547432	66.1804803	71.586904	32.9295267	25.0188611	120.979259
Tle2	9.95297083	0.78806203	0.4034742	1.95319063	0.05079701	0.7364447	Tle2	2.01613899	5.88280835	5.40734359	10.3407001	16.6481172	9.40843619	4.81131944	25.1089028
Adora2a	21.449774	-0.7369166	0.37732256	-1.9530149	0.05081783	0.7364447	Adora2a	35.6184554	26.2463757	34.0344567	16.5451201	21.6425524	4.7042181	7.69811111	25.1089028
Tchh	8.05434731	0.78860185	0.40387973	1.95256605	0.05087104	0.7364447	Tchh	4.7043243	3.62018975	1.90847421	10.3407001	14.9833055	9.40843619	5.77358333	13.6957652
Pdlim4	4.60195126	-0.8126574	0.41621836	-1.9524786	0.05088141	NA	Pdlim4	4.03227797	4.97776091	5.08926455	14.4769801	4.99443516	0	0.96226389	2.28262753
Hibadh	31.5285159	-0.5793738	0.29695681	-1.9510372	0.05105262	0.7364447	Hibadh	45.027104	33.4867552	40.7141164	37.2265202	18.3129289	19.9929269	34.6415	22.8262753
Erbb4	97.2232388	0.73150216	0.37494314	1.95096824	0.05106082	0.7364447	Erbb4	49.059382	81.4542694	86.1994184	39.2946602	63.2628454	291.661522	84.6792222	82.1745911
Ptpn1	21.9231339	-0.6135686	0.31449677	-1.9509535	0.05106257	0.7364447	Ptpn1	30.2420848	29.8665654	19.7209001	31.0221002	14.9833055	15.2887088	18.2830139	15.9783927
Cd9	4.84306655	-0.8097878	0.41522937	-1.950218	0.05115014	0.7364447	Cd9	5.37637063	12.6706641	7.63389683	2.06814001	3.32962344	1.17605452	1.92452778	4.56525506
Tpp1	34.5265624	-0.6752245	0.34629677	-1.9498434	0.05119479	0.7364447	Tpp1	30.2420848	70.5937002	54.7095939	20.6814001	18.3129289	24.697145	25.0188611	31.9567854
Cspg5	128.192715	-0.6161413	0.31601512	-1.949721	0.05120938	0.7364447	Cspg5	146.5061	235.764858	163.174545	93.0663005	126.525691	84.6759257	54.8490416	120.979259
Xpnpep1	22.2589894	0.65199843	0.3345121	1.94910268	0.05128316	0.7364447	Xpnpep1	16.1291119	15.8383302	17.4943469	12.4088401	31.6314227	23.5210905	15.3962222	45.6525506
Lrrn4	1.81202668	0.72554509	0.37228953	1.94887321	0.05131057	NA	Lrrn4	0	0.45252372	0.9542371	0	9.98887032	1.17605452	1.92452778	0
Fam222b	64.8986894	-0.5160051	0.26479696	-1.9486822	0.05133339	0.7364447	Fam222b	85.3498837	71.9512713	62.6615698	90.9981605	66.5924688	47.042181	37.5282917	57.0656883
Cpq	1.91146887	-0.7796472	0.4002879	-1.9477161	0.05144895	NA	Cpq	3.36023164	3.16766603	4.77118552	2.06814001	0	0	1.92452778	0
Tulp3	7.15579268	-0.7811805	0.40129343	-1.9466566	0.05157592	0.73808569	Tulp3	11.4247876	14.480759	6.67965972	8.27256004	6.65924688	5.88027262	3.84905555	0
Pcdhgb2	222.431318	-0.5861783	0.30113341	-1.9465735	0.05158589	0.73808569	Pcdhgb2	273.522856	387.812827	278.319155	157.178641	223.084771	201.105324	107.773556	150.653417
Rpl18a	30.4552707	-0.6357693	0.32683496	-1.9452303	0.05174727	0.7394048	Rpl18a	41.6668724	36.6544212	36.2610099	41.3628002	26.6369875	11.7605452	35.6037639	13.6957652
Fkbp4	67.9059645	0.61660235	0.31738715	1.94274515	0.05204696	0.74243278	Fkbp4	40.994826	41.6321821	37.8514051	84.7937404	66.5924688	76.4435441	62.5471528	132.392397
Serpinb9	6.41115522	-0.7996594	0.41170275	-1.9423222	0.05209811	0.74243278	Serpinb9	8.73660227	7.2403795	6.67965972	14.4769801	0	1.17605452	3.84905555	9.13051012
Mapk8ip2	110.562666	0.8047402	0.41452388	1.94136029	0.05221459	0.74310193	Mapk8ip2	37.6345944	30.3190892	32.1259825	134.429101	387.901131	37.6337448	32.7169722	191.740713
Gpr37	16.9235455	-0.7000037	0.36075485	-1.9403862	0.05233277	0.74335205	Gpr37	14.1129729	34.3918026	22.9016905	16.5451201	18.3129289	9.40843619	10.5849028	9.13051012
Ppm1g	40.3255618	0.60464127	0.31166633	1.9400276	0.05237634	0.74335205	Ppm1g	22.1775288	21.2686148	29.8994292	51.7035003	56.6035985	56.4506172	36.5660278	47.9351782
Traf4	5.71123661	-0.7957885	0.41030656	-1.9394973	0.05244082	0.74335205	Traf4	10.0806949	8.59795066	4.45310648	10.3407001	1.66481172	1.17605452	4.81131944	4.56525506
Cct7	59.5097863	0.47039594	0.24279605	1.93741183	0.05269502	0.74596608	Cct7	55.7798453	45.7048956	39.7598793	55.8397803	76.5813391	65.8590533	63.5094166	73.044081
Rgs20	25.7569894	-0.6752859	0.3487495	-1.9363063	0.05283019	0.74625695	Rgs20	41.6668724	32.5817078	44.2129858	14.4769801	9.98887032	21.1689814	25.981125	15.9783927
Ptpre	22.4827472	0.66031504	0.34105385	1.93610198	0.05285521	0.74625695	Ptpre	13.4409266	21.7211385	19.7209001	8.27256004	21.6425524	44.6900719	29.8301805	20.5436478
Asb1	54.074357	0.56784774	0.29339675	1.93542616	0.05293802	0.74644007	Asb1	35.6184554	32.5817078	42.3045116	57.9079203	81.5757743	42.3379629	55.8113055	84.4572186
Ccdc183	1.82376	0.72573785	0.37505788	1.93500226	0.05299002	NA	Ccdc183	0.67204633	0.45252372	0.31807903	0	1.66481172	2.35210905	0	9.13051012
A330102I10Rik	1.17899286	-0.6881829	0.35586633	-1.933824	0.05313477	NA	A330102I10Ri	0.67204633	2.71514231	1.90847421	4.13628002	0	0	0	0
Ccdc149	25.6758797	0.58626404	0.30327814	1.93309033	0.05322507	0.74943586	Ccdc149	25.5377605	15.3858064	20.9932163	16.5451201	26.6369875	41.1619083	31.7547083	27.3915304
Lef1	2.39007811	-0.7958264	0.4118523	-1.9323102	0.05332123	NA	Lef1	4.03227797	4.97776091	4.45310648	2.06814001	1.66481172	0	1.92452778	0
Ly6a	3.90265317	-0.7668964	0.39691391	-1.9321479	0.05334126	NA	Ly6a	0.67204633	12.6706641	6.67965972	6.20442003	4.99443516	0	0	0
Snn	117.245004	-0.4767618	0.24675618	-1.9321169	0.05334507	0.74943586	Snn	138.441544	151.595446	116.416927	144.769801	94.8942681	70.5632714	86.60375	134.675024
Jakmip3	19.6917935	0.64604749	0.33439498	1.93198917	0.05336084	0.74943586	Jakmip3	13.4409266	13.1231878	20.3570582	8.27256004	28.3017992	32.9295267	18.2830139	22.8262753
Mien1	31.5151572	-0.6531929	0.33838528	-1.9303231	0.05356681	0.74975053	Mien1	36.9625481	27.6039469	41.0321955	55.8397803	13.3184938	12.9365998	27.9056528	36.5220405
Zfp64	7.20192989	-0.7520236	0.38961274	-1.9301823	0.05358425	0.74975053	Zfp64	11.4247876	7.2403795	10.4966081	10.3407001	4.99443516	4.7042181	3.84905555	4.56525506
2810468N07Rik	10.8131606	-0.748581	0.38784451	-1.9301061	0.05359369	0.74975053	2810468N07R	10.7527413	11.313093	12.0870033	26.8858201	3.32962344	4.7042181	10.5849028	6.84788259

GeneID	Base mean	log2(FC)	StdErr	Wald-Stats	P-value	P-adj		GeneID	Normalised expression for Chow#1	Normalised expression for Chow#2	Normalised expression for Chow#3	Normalised expression for Chow#4	Normalised expression for HFD#1	Normalised expression for HFD#2	Normalised expression for HFD#3	Normalised expression for HFD#4
Copz2	2.50636168	-0.7957126	0.41227704	-1.9300434	0.05360145	NA		Copz2	4.03227797	5.43028463	2.86271131	4.13628002	1.66481172	0	1.92452778	0
Gripap1	39.3551153	0.57207438	0.29649604	1.92945031	0.05367498	0.74990616		Gripap1	38.3066407	27.6039469	34.3525357	20.6814001	46.6147282	64.6829988	52.9245139	29.6741579
Aldh1a2	3.68437445	-0.8026098	0.41632851	-1.9278281	0.05387651	NA		Aldh1a2	6.04841696	9.05047438	4.77118552	2.06814001	3.32962344	0	1.92452778	2.28262753
Hrh2	2.15563907	-0.7757732	0.40243154	-1.9277146	0.05389064	NA		Hrh2	2.01613899	4.97776091	1.90847421	6.20442003	0	1.17605452	0.96226389	0
Bcan	66.9107673	-0.7356199	0.38170178	-1.9272111	0.05395333	0.75190443		Bcan	66.5325865	136.662163	68.3869924	88.9300204	101.553515	31.7534722	16.3584861	25.1089028
Arnt2	273.557494	0.38849714	0.20164479	1.92664114	0.05402437	0.75190443		Arnt2	223.791427	202.278102	235.696565	279.198901	374.582637	295.189686	280.981055	296.741579
Vps16	21.2937985	0.62628515	0.32507215	1.92660351	0.05402906	0.75190443		Vps16	15.4570656	15.3858064	14.9497146	16.5451201	39.9554813	15.2887088	23.0943333	29.6741579
Anln	12.0188812	-0.7132406	0.37041194	-1.9255336	0.05416264	0.75219414		Anln	14.7850192	21.2686148	14.6316356	14.4769801	3.32962344	10.5844907	12.5094306	4.56525506
Nxph1	82.2335111	0.69955174	0.36334506	1.92530963	0.05419063	0.75219414		Nxph1	51.7475673	56.1129411	72.2039408	45.4990802	41.620293	223.45036	101.037708	66.1961984
Dip2c	90.9493039	0.41946364	0.21816462	1.92269321	0.05451859	0.75324299		Dip2c	86.6939764	72.8563187	73.794336	74.4530404	119.866444	114.077289	99.1131805	86.7398462
Ddo	3.87560167	-0.7758362	0.40355209	-1.9225182	0.05454059	NA		Ddo	1.34409266	3.16766603	3.81694841	18.6132601	0	1.17605452	2.88679167	0
Crebzf	26.261138	0.65168771	0.33901325	1.92230747	0.05456708	0.75324299		Crebzf	22.8495752	11.7656167	15.2677937	26.8858201	24.9721758	24.697145	28.8679167	54.7830607
Vps39	57.11997	0.46832194	0.24363796	1.92220431	0.05458005	0.75324299		Vps39	41.6668724	46.1574193	53.4372778	45.4990802	79.9109626	70.5632714	55.8113055	63.9135709
Dbp	81.2743002	-0.6922708	0.36019555	-1.9219304	0.05461452	0.75324299		Dbp	122.312432	64.7108918	74.4304941	163.383061	51.6091633	23.5210905	45.2264028	105.000866
Tnc	2.08839607	-0.7708401	0.40107608	-1.92193	0.05461457	NA		Tnc	4.7043243	4.07271347	5.08926455	0	1.66481172	1.17605452	0	0
Slc29a3	9.55719957	-0.7861842	0.40906654	-1.9218982	0.05461857	0.75324299		Slc29a3	6.04841696	27.1514231	13.3593194	8.27256004	1.66481172	10.5844907	4.81131944	4.56525506
Exosc3	7.41267345	0.73155579	0.3807865	1.92117052	0.05471021	0.75353449		Exosc3	4.7043243	5.43028463	4.45310648	4.13628002	9.98887032	8.23238167	8.660375	13.6957652
Apcdd1	4.90542993	-0.7835308	0.40852103	-1.9179692	0.05511491	0.75688675		Apcdd1	3.36023164	19.4585199	4.45310648	4.13628002	6.65924688	1.17605452	0	0
Ercc2	18.7230806	0.73422789	0.382883	1.91762989	0.05515796	0.75688675		Ercc2	12.7688802	9.5029981	9.86045007	16.5451201	44.9499164	9.40843619	12.5094306	34.239413
Pcdhgb8	221.19176	-0.5751813	0.29995383	-1.9175661	0.05516605	0.75688675		Pcdhgb8	272.850809	384.192637	272.275654	157.178641	224.749582	197.57716	107.773556	152.936045
Tro	152.202373	0.50802278	0.26501568	1.91695363	0.05524382	0.75698202		Tro	113.575829	118.108691	120.551954	138.565381	193.11816	261.084104	117.396194	155.218672
Col1a1	2.99797162	-0.796575	0.41574173	-1.9160334	0.05536085	NA		Col1a1	4.7043243	4.97776091	6.36158069	2.06814001	1.66481172	0	1.92452778	2.28262753
Pcdhga11	232.044288	-0.5708995	0.29838815	-1.9132781	0.05571247	0.76127051		Pcdhga11	276.883087	405.008728	286.271131	169.587481	238.068076	202.281378	111.622611	166.63181
Slc44a1	24.0020553	-0.7318563	0.38253405	-1.9131796	0.05572507	0.76127051		Slc44a1	27.5538995	49.3250854	40.0779583	12.4088401	18.3129289	28.2253086	11.5471667	4.56525506
Sart1	20.898927	0.6843707	0.35785274	1.91243666	0.05582021	0.76127051		Sart1	20.1613899	7.69290322	15.2677937	14.4769801	38.2906696	25.8731995	13.4716944	31.9567854
Asxl3	15.4445372	0.66649686	0.34874056	1.91115383	0.05598481	0.76127051		Asxl3	16.1291119	9.95552182	12.4050823	4.13628002	19.9777406	27.0492541	20.2075417	13.6957652
Capn3	1.03640751	-0.6828279	0.35731972	-1.9109719	0.0560082	NA		Capn3	2.68818531	2.26261859	1.27231614	2.06814001	0	0	0	0
Rhof	40.9497242	-0.5218874	0.27313706	-1.9107161	0.05604107	0.76127051		Rhof	49.059382	60.6381783	39.4418003	47.5672202	34.9610461	27.0492541	34.6415	34.239413
Pcdhga7	229.088431	-0.5840656	0.30571664	-1.9104802	0.05607142	0.76127051		Pcdhga7	281.587412	402.74611	286.907289	159.246781	234.738453	205.809542	108.735819	152.936045
Eif3a	92.0780538	0.55386547	0.28994883	1.91021799	0.05610515	0.76127051		Eif3a	84.6778374	62.4482732	71.8858618	70.3167603	84.9053977	171.703961	122.207514	68.4788259
A330050F15Rik	10.6471121	0.74435513	0.38970426	1.91005132	0.05612661	0.76127051		A330050F15Ri	9.4086486	4.52523719	6.99773876	6.20442003	4.99443516	9.40843619	23.0943333	20.5436478
Pcdhgb5	222.577071	-0.5747777	0.30110174	-1.9089152	0.05627303	0.76140766		Pcdhgb5	276.211041	387.360303	274.820286	155.110501	228.079206	196.401106	109.698083	152.936045
Pcdhgb4	222.601215	-0.5722014	0.29975963	-1.9088674	0.0562792	0.76140766		Pcdhgb4	274.194902	386.90778	271.957575	159.246781	223.084771	201.105324	106.811292	157.5013
Bckdhb	15.1800556	0.66655948	0.34939452	1.90775599	0.05642276	0.76161397		Bckdhb	12.0968339	12.2181404	12.0870033	4.13628002	14.9833055	14.1126543	22.1320694	29.6741579
Tmtc4	16.1057393	0.73809752	0.38699645	1.90724624	0.05648871	0.76161397		Tmtc4	6.72046328	16.2908539	13.0412404	4.13628002	9.98887032	41.1619083	19.2452778	18.2610202
Ppp1r18	4.48682837	-0.7935827	0.41609332	-1.907223	0.05649172	NA		Ppp1r18	6.04841696	6.33533207	2.86271131	12.4088401	4.99443516	0	0.96226389	2.28262753
Pcdhga9	226.843351	-0.5773164	0.30278998	-1.9066563	0.05656511	0.76161397		Pcdhga9	284.947643	395.958254	278.001076	157.178641	228.079206	206.985596	110.660347	152.936045
Axl	17.5079215	-0.7467758	0.39179384	-1.9060428	0.05664465	0.76161397		Axl	30.9141311	39.3695635	17.1762679	8.27256004	18.3129289	14.1126543	9.62263889	2.28262753
Prkd3	10.6240813	-0.6931347	0.3636601	-1.9059959	0.05665074	0.76161397		Prkd3	12.0968339	13.1231878	15.9039517	14.4769801	8.3240586	9.40843619	4.81131944	6.84788259
Ccnyl1	2.41540326	-0.7838008	0.41195661	-1.9026295	0.05708891	NA		Ccnyl1	4.7043243	2.71514231	4.45310648	4.13628002	0	2.35210905	0.96226389	0
Tcf7l2	13.8475541	-0.7276901	0.38278393	-1.9010466	0.05729591	0.76470545		Tcf7l2	14.7850192	19.0059962	14.3135566	26.8858201	11.653682	1.17605452	11.5471667	11.4131377
Tst	9.14343843	-0.766975	0.40346453	-1.9009724	0.05730563	0.76470545		Tst	10.0806949	7.2403795	8.2700549	26.8858201	6.65924688	2.35210905	4.81131944	6.84788259
Cox6a1	114.144238	-0.4525437	0.23820919	-1.8997741	0.05746276	0.76470545		Cox6a1	119.624246	139.377305	142.817486	132.360961	89.8998329	67.0351079	119.320722	102.718239
Slc18b1	19.5280684	-0.6519064	0.34323242	-1.8993147	0.05752311	0.76470545		Slc18b1	16.8011582	34.3918026	31.4898244	16.5451201	11.653682	15.2887088	16.3584861	13.6957652
Golga4	48.2315334	0.62815378	0.33078692	1.89896802	0.05756868	0.76470545		Golga4	38.978687	32.5817078	41.6683535	28.9539601	38.2906696	111.72518	54.8490416	38.804668
Gpr176	19.6173278	0.69853722	0.36789908	1.89871969	0.05760134	0.76470545		Gpr176	7.39250961	15.3858064	22.9016905	6.20442003	26.6369875	36.4576902	25.981125	15.9783927
Plagl1	192.027495	0.78204424	0.41204264	1.89796919	0.05770014	0.76470545		Plagl1	49.7314283	41.1796584	55.027673	283.335181	188.123724	123.485725	117.396194	677.940377
Eif2ak4	15.5305137	0.63625716	0.33527784	1.89770119	0.05773546	0.76470545		Eif2ak4	13.4409266	11.313093	11.7689243	8.27256004	23.3073641	27.0492541	15.3962222	13.6957652
Selplg	4.7274432	-0.7843004	0.41337298	-1.8973191	0.05778584	0.76470545		Selplg	2.01613899	18.5534725	7.31581779	2.06814001	1.66481172	2.35210905	3.84905555	0
Pcdh18	16.8362401	0.72152135	0.38031098	1.89718777	0.05780316	0.76470545		Pcdh18	10.0806949	14.9332827	11.1327662	8.27256004	13.3184938	48.2182355	17.32075	11.4131377
Mapkapk3	1.29974032	-0.6698276	0.35308923	-1.8970491	0.05782146	NA		Mapkapk3	2.01613899	0.90504744	1.27231614	6.20442003	0	0	0	0
Foxf2	2.96697377	-0.7528067	0.39695236	-1.8964661	0.05789844	NA		Foxf2	2.01613899	4.07271347	1.90847421	12.4088401	3.32962344	0	0	0
Lancl3	24.2758916	0.65206002	0.34383252	1.89644661	0.05790101	0.76470545		Lancl3	12.7688802	9.5029981	17.8124259	33.0902402	26.6369875	36.4576902	25.981125	31.9567854
Thg1l	5.83050658	-0.7805203	0.41169669	-1.8958624	0.05797825	0.76470545		Thg1l	9.4086486	5.43028463	6.36158069	14.4769801	1.66481172	3.52816357	5.77358333	0
St8sia4	18.9456334	0.78296312	0.41301897	1.89570739	0.05799875	0.76470545		St8sia4	7.39250961	14.0282353	13.9954775	6.20442003	6.65924688	75.2674895	21.1698055	6.84788259
1110008P14Rik	75.5881082	-0.6601591	0.34825357	-1.8956278	0.05800928	0.76470545		1110008P14Ri	114.247876	77.8340797	82.0643909	113.747701	59.9332219	15.2887088	54.8490416	86.7398462
Zfp867	10.3618048	0.69975172	0.36928127	1.89490176	0.05810542	0.76470545		Zfp867	6.04841696	6.78785578	6.04350166	10.3407001	13.3184938	7.05632714	17.32075	15.9783927
Dlx2	38.3559629	0.74699007	0.39428811	1.89452853	0.05815489	0.76470545		Dlx2	21.5054825	9.95552182	16.5401098	49.6353602	46.6147282	27.0492541	25.981125	109.566121
Masp1	4.76044315	0.78002294	0.41173936	1.89445802	0.05816424	0.76470545		Masp1	3.36023164	3.62018975	2.54463228	0	4.99443516	10.5844907	3.84905555	9.13051012
Rarb	12.0553418	-0.7155367	0.3778401	-1.8937554	0.05825748	0.76470545		Rarb	24.8657142	12.6706641	19.7209001	6.20442003	9.98887032	9.40843619	6.73584722	6.84788259
Ldlrad3	4.40591447	-0.7884235	0.41636171	-1.8936023	0.05827782	NA		Ldlrad3	2.01613899	11.313093	7.63389683	6.20442003	1.66481172	3.52816357	2.88679167	0
Mettl25	5.95616304	0.76878319	0.40604105	1.8933632	0.05830959	0.76470545		Mettl25	2.68818531	3.62018975	2.22655324	6.20442003	4.99443516	9.40843619	4.81131944	13.6957652
Serpind1	1.00448115	-0.673775	0.3559691	-1.8927907	0.05838571	NA		Serpind1	1.34409266	2.71514231	1.90847421	2.06814001	0	0	0	0
Nhsl2	108.380894	0.48664366	0.25711977	1.89267301	0.05840137	0.76470545		Nhsl2	85.3498837	95.9350284	104.648002	64.1123403	114.872009	177.584233	105.849028	118.696632
Bak1	9.52191587	-0.7385662	0.39023763	-1.8926063	0.05841025	0.76470545		Bak1	10.7527413	15.8383302	11.4508452	12.4088401	8.3240586	1.17605452	4.81131944	11.4131377
1700094D03Rik	2.00660338	-0.7266504	0.38400134	-1.8923121	0.05844941	NA		1700094D03Ri	0.67204633	2.26261859	3.18079035	8.27256004	1.66481172	0	0	0
Pros1	1.9357658	-0.6912399	0.36534413	-1.892024	0.05848779	NA		Pros1	0.67204633	9.5029981	4.13502745	0	0	1.17605452	0	0
Hist1h2ad	1.6737255	-0.7258965	0.38369845	-1.8918412	0.05851214	NA		Hist1h2ad	2.68818531	2.26261859	1.27231614	6.20442003	0	0	0.96226389	0
Igfbp2	4.57252148	-0.7873593	0.41623339	-1.8916293	0.05854039	NA		Igfbp2	10.7527413	11.7656167	5.08926455	0	1.66481172	1.17605452	3.84905555	2.28262753
Mavs	8.44062523	-0.7839808	0.41454987	-1.8911617	0.05860276	0.76470545		Mavs	7.39250961	5.43028463	10.1785291	26.8858201	1.66481172	5.88027262	0.96226389	9.13051012
Cplx2	1071.03891	0.43081303	0.22785797	1.89070861	0.05866325	0.76470545		Cplx2	883.068876	943.05943	938.651231	825.187864	1618.19699	937.315456	1000.75444	1422.07695
Gm12338	66.9156554	-0.5629719	0.29776788	-1.89064	0.05867242	0.76470545		Gm12338	71.2369108	89.1471726	67.7508343	103.407001	39.9554813	39.9858538	82.7546944	41.0872956
Pid1	35.9787721	-0.5518754	0.29197354	-1.8901556	0.05873716	0.76470545		Pid1	46.3711967	54.3028463	36.897168	37.2265202	39.9554813	22.345036	27.9056528	22.8262753
Klhdc2	109.499405	0.50308645	0.26616539	1.89012727	0.05874094	0.76470545		Klhdc2	88.038069	79.6441745	99.8768168	86.8618804	83.240586	151.711034	177.056555	109.566121
Gm20604	35.4852547	0.60163464	0.31844398	1.88929505	0.0588523	0.76522316		Gm20604	26.8818531	22.1736622	28.6271131	31.0221002	31.6314227	32.9295267	74.0943194	36.5220405
Mecp2	234.62366	0.36562622	0.19376238	1.88698243	0.05916269	0.7683242		Mecp2	180.108416	188.249867	197.845159	250.244941	256.381005	266.964377	247.301819	289.893696
Mapk3	164.760447	-0.5362953	0.28428983	-1.8864386	0.05923588	0.76834112		Mapk3	203.630038	189.607438	168.263809	235.767961	173.140419	68.2111624	117.396194	162.066555
Ifitm3	1.93042447	-0.7638716	0.40497439	-1.886222	0.05926505	NA		Ifitm3	3.36023164	4.52523719	2.86271131	2.06814001	1.66481172	0	0.96226389	0
Synm	17.8939513	0.64870151	0.34402319	1.88563311	0.05934441	0.76870215		Synm	16.8011582	11.313093	11.7689243	12.4088401	33.2962344	28.2253086	20.2075417	9.13051012
C920025E04Rik	1.2110936	0.59423957	0.31514085	1.88563167	0.05934461	NA		C920025E04Ri	0	0	0	0	1.66481172	1.17605452	0	6.84788259
Parp6	73.5944771	-0.5227291	0.2772856	-1.8851651	0.05940756	0.76870215		Parp6	106.855366	100.912789	84.9271022	62.0442003	61.5980337	48.2182355	80.8301666	43.3699231
Pcdhga3	228.101966	-0.5641592	0.29940318	-1.8842792	0.05952722	0.76931916		Pcdhga3	276.211041	395.053207	284.044578	161.314921	231.408829	209.337705	114.509403	152.936045
Hpca	268.169333	-0.5254483	0.27913978	-1.8823841	0.05978389	0.7700093		Hpca	450.943086	251.603188	346.388069	241.972381	218.090335	136.422325	232.867861	267.067421
Penk	109.679454	-0.7347918	0.39058721	-1.8812489	0.05993807	0.7700093		Penk	184.140694	189.154915	186.394314	37.2265202	39.9554813	150.534979	37.5282917	52.5004332
Kctd12	90.4478826	0.45725809	0.24316942	1.88040953	0.06005229	0.7700093		Kctd12	70.5648645	87.7896015	74.4304941	66.1804803	118.201632	88.2040893	133.754681	84.4572186
Zcchc18	155.474195	0.46932566	0.2496539	1.87990518	0.060121	0.7700093		Zcchc18	151.88247	95.4825047	146.316356	117.883981	153.162678	234.03485	194.377305	150.653417
Myh9	28.3459145	0.66422945	0.35344263	1.87931333	0.06020172	0.7700093		Myh9	22.1775288	17.1959013	24.4920857	14.4769801	69.9220923	21.1689814	23.0943333	34.239413
Gnai2	187.669984	-0.5776723	0.30742975	-1.8790385	0.06023924	0.7700093		Gnai2	208.334362	234.407286	150.769462	332.970542	183.129289	90.5561984	104.886764	196.305968
Fbxo38	31.2997212	0.55609028	0.29603902	1.87843576	0.06032158	0.7700093		Fbxo38	24.1936678	19.0059962	26.0824808	31.0221002	34.9610461	39.9858538	50.0377222	25.1089028
Mag	21.5522304	-0.7805587	0.41554878	-1.8783804	0.06032914	0.7700093		Mag	16.8011582	35.7493738	28.6271131	45.4990802	33.2962344	2.35210905	0.96226389	9.13051012
Sgk3	5.23757498	-0.781817	0.41625509	-1.8782161	0.06035161	0.7700093		Sgk3	10.0806949	13.1231878	8.90621297	0	1.66481172	2.35210905	5.77358333	0
Rpl29	23.6184895	-0.7271095	0.38713586	-1.8781766	0.06035701	0.7700093		Rpl29	53.7637063	33.0342315	30.5355873	10.3407001	11.653682	7.05632714	28.8679167	13.6957652
Tead1	16.5605929	-0.6427756	0.3422553	-1.8780589	0.06037311	0.7700093		Tead1	15.4570656	23.0787097	17.8124259	28.9539601	8.3240586	9.40843619	13.4716944	15.9783927
Nxph3	7.05616461	-0.7545912	0.40206006	-1.8768122	0.06054384	0.77063261		Nxph3	6.04841696	11.313093	7.31581779	14.4769801	4.99443516	3.52816357	1.92452778	6.84788259
Msmo1	30.4244218	-0.730025	0.38909972	-1.8761901	0.06062918	0.77063261		Msmo1	32.2582238	75.1189373	48.9841713	8.27256004	13.3184938	30.5774176	21.1698055	13.6957652
Pcdhga6	229.545346	-0.5612197	0.29916198	-1.8759725	0.06065905	0.77063261		Pcdhga6	282.259458	396.863302	282.136104	161.314921	231.408829	211.689814	115.471667	155.218672
Hipk2	68.1213987	-0.5587639	0.29791228	-1.8755988	0.06071039	0.77063261		Hipk2	75.9412351	112.225882	75.3847312	70.3167603	76.5813391	59.9787807	47.1509305	27.3915304
Elovl2	4.73254501	-0.7764084	0.41433557	-1.8738638	0.06094919	0.77274613		Elovl2	3.36023164	14.480759	11.4508452	0	3.32962344	2.35210905	2.88679167	0
Sfmbt1	40.3144279	0.54165291	0.28932558	1.87212241	0.06118967	0.77487583		Sfmbt1	28.2259458	33.9392789	36.2610099	28.9539601	53.2739751	59.9787807	56.7735694	25.1089028
Cep192	3.08515269	0.77886597	0.41618546	1.87143963	0.06128417	NA		Cep192	0.67204633	1.81009488	1.59039517	2.06814001	4.99443516	7.05632714	1.92452778	4.56525506
Rasgrp4	4.77472059	0.76674641	0.40978182	1.87110887	0.06132999	0.77573368		Rasgrp4	2.68818531	1.35757116	2.86271131	0	8.3240586	4.7042181	0	18.2610202
Sparcl1	472.958036	-0.7491687	0.40072053	-1.869554	0.06154578	0.77607367		Sparcl1	567.207101	1147.60015	725.538278	186.132601	183.129289	624.484952	274.245208	75.3267085
Ptges2	18.7840443	0.60097519	0.3214601	1.86951718	0.0615509	0.77607367		Ptges2	14.7850192	11.7656167	11.1327662	20.6814001	21.6425524	18.8168724	24.0565972	27.3915304
Dync1h1	289.537122	0.6322627	0.3384035	1.86836927	0.06171062	0.77607367		Dync1h1	204.97413	174.221632	183.531603	283.335181	690.896864	238.739068	179.943347	360.65515
Nrl	0.94209734	0.59076514	0.31621508	1.86823837	0.06172885	NA		Nrl	0	0	0	0	3.32962344	0	1.92452778	2.28262753
Naa60	129.805388	-0.475155	0.25441057	-1.8676701	0.06180807	0.77607367		Naa60	158.602934	123.086452	154.586411	175.791901	106.54795	71.739326	101.999972	146.088162
Dusp1	15.5008765	-0.6257911	0.33506601	-1.8676652	0.06180876	0.77607367		Dusp1	20.1613899	19.9110436	17.1762679	22.7495401	13.3184938	9.40843619	14.4339583	6.84788259
A730008H23Rik	10.761089	0.71985316	0.3855414	1.86712286	0.06188443	0.77607367		A730008H23R	4.03227797	7.69290322	4.77118552	12.4088401	11.653682	11.7605452	8.660375	25.1089028
Alms1	9.83839598	0.72296173	0.38720799	1.86711469	0.06188557	0.77607367		Alms1	8.73660227	5.43028463	8.58813393	2.06814001	18.3129289	11.7605452	19.2452778	4.56525506
Pcdhga10	225.4066	-0.561149	0.30060358	-1.866741	0.06193777	0.77607367		Pcdhga10	281.587412	390.527969	275.456444	155.110501	224.749582	209.337705	113.547139	152.936045
H2-DMa	6.36464508	-0.7659776	0.41085083	-1.8643691	0.0622699	0.77637456		H2-DMa	10.0806949	13.1231878	4.77118552	8.27256004	8.3240586	1.17605452	2.88679167	2.28262753
Hist1h4n	27.777776	-0.6972496	0.37403726	-1.8641181	0.06230512	0.77637456		Hist1h4n	32.9302701	32.5817078	41.9864326	39.2946602	23.3073641	2.35210905	26.9433889	22.8262753

GeneID	Base mean	log2(FC)	StdErr	Wald-Stats	P-value	P-adj	GeneID	Normalised expression for Chow#1	Normalised expression for Chow#2	Normalised expression for Chow#3	Normalised expression for Chow#4	Normalised expression for HFD#1	Normalised expression for HFD#2	Normalised expression for HFD#3	Normalised expression for HFD#4
Dynlt1b	1.44085121	-0.6990057	0.37498957	-1.864067	0.0623123	NA	Dynlt1b	3.36023164	4.97776091	2.22655324	0	0	0	0.96226389	0
Rps15a	72.0838847	0.4406737	0.23643895	1.86379484	0.06235052	0.77637456	Rps15a	58.4680306	55.6604174	52.1649617	78.5893204	91.5646446	79.9717076	89.4905416	70.7614535
Psmc5	60.093302	0.54534588	0.29264047	1.86353544	0.06238697	0.77637456	Psmc5	73.9250961	36.2018975	37.215247	41.3628002	74.9165274	64.6829988	88.5282777	63.9135709
Cdc42ep1	12.7903871	-0.7745909	0.41577943	-1.8629851	0.06246437	0.77637456	Cdc42ep1	11.4247876	13.1231878	9.54237104	41.3628002	13.3184938	1.17605452	0.96226389	11.4131377
1700084C01Rik	5.90904532	-0.775682	0.41636999	-1.8629633	0.06246743	0.77637456	1700084C01Ri	10.7527413	6.78785578	6.99773876	12.4088401	1.66481172	0	8.660375	0
Trpm3	37.1751363	-0.6502474	0.34911723	-1.8625473	0.06252598	0.77637456	Trpm3	39.6507334	77.3815559	41.9864326	33.0902402	16.6481172	36.4576902	38.4905555	13.6957652
Dusp4	13.1647213	0.686903	0.36882139	1.8624272	0.0625429	0.77637456	Dusp4	10.7527413	7.69290322	9.54237104	6.20442003	11.653682	11.7605452	13.4716944	34.239413
Pcdhga5	228.399986	-0.5667053	0.30447542	-1.8612513	0.06270869	0.77652481	Pcdhga5	280.915365	401.388539	281.181867	157.178641	228.079206	215.217978	112.584875	150.653417
P2rx7	2.55664252	-0.7500274	0.40297924	-1.861206	0.0627151	NA	P2rx7	1.34409266	5.88280835	4.13502745	6.20442003	0	0	2.88679167	0
Esrrg	66.3989786	0.54853223	0.29473403	1.86110924	0.06272875	0.77652481	Esrrg	44.3550577	30.3190892	55.663831	80.6574604	66.5924688	77.6195986	84.6792222	91.3051012
Mtmr10	9.22747793	-0.7567919	0.40670325	-1.8607962	0.06277296	0.77652481	Mtmr10	9.4086486	20.8160911	7.31581779	14.4769801	13.3184938	2.35210905	3.84905555	2.28262753
Slc6a13	2.17253253	-0.7404966	0.39808961	-1.8601253	0.0628678	NA	Slc6a13	4.03227797	7.2403795	2.86271131	0	0	0	0.96226389	2.28262753
Iscu	33.4904551	-0.5698832	0.30653531	-1.8591112	0.06301137	0.77857292	Iscu	42.3389187	29.8665654	43.8949068	51.7035003	18.3129289	24.697145	36.5660278	20.5436478
E2f5	5.48877497	-0.7737496	0.41632145	-1.8585389	0.06309252	0.77867533	E2f5	10.7527413	10.8605693	10.1785291	2.06814001	0	2.35210905	7.69811111	0
S100a11	1.48409301	-0.7146525	0.38461521	-1.8580973	0.0631552	NA	S100a11	2.01613899	3.16766603	1.59039517	4.13628002	0	0	0.96226389	0
Aifm3	20.6095787	-0.6757907	0.36381526	-1.8575106	0.06323854	0.77957731	Aifm3	19.4893435	22.6261859	17.1762679	49.6353602	19.9777406	9.40843619	10.5849028	15.9783927
Itgb8	25.6232333	-0.7135973	0.38447455	-1.8560327	0.0634489	0.781195	Itgb8	27.5538995	56.5654649	42.6225906	10.3407001	16.6481172	30.5774176	11.5471667	9.13051012
Zkscan4	2.8123877	0.77124121	0.41595388	1.85415078	0.06371759	NA	Zkscan4	2.01613899	2.26261859	0.9542371	0	3.32962344	5.88027262	5.77358333	2.28262753
Rgs6	12.2339967	-0.6913291	0.37291331	-1.8538601	0.06375918	0.781195	Rgs6	22.8495752	16.2908539	13.6773985	12.4088401	6.65924688	4.7042181	14.4339583	6.84788259
Vgll3	4.30441411	0.76954607	0.41532004	1.85289896	0.06389684	NA	Vgll3	2.01613899	1.81009488	2.22655324	4.13628002	9.98887032	2.35210905	9.62263889	2.28262753
Alg3	8.5004811	-0.7502524	0.40502964	-1.8523395	0.06397709	0.781195	Alg3	6.04841696	18.5534725	15.2677937	6.20442003	4.99443516	5.88027262	1.92452778	9.13051012
Pcdhga2	227.497405	-0.5631164	0.30402743	-1.8521895	0.06399861	0.781195	Pcdhga2	278.899226	397.315825	279.273392	159.246781	229.744017	212.865869	109.698083	152.936045
Naaa	9.5744101	-0.7153685	0.38625971	-1.8520401	0.06402007	0.781195	Naaa	15.4570656	17.648425	13.3593194	4.13628002	9.98887032	4.7042181	6.73584722	4.56525506
Pvrl4	3.08317673	0.75998432	0.41036525	1.85197049	0.06403006	NA	Pvrl4	0.67204633	1.35757116	2.22655324	0	3.32962344	4.7042181	0.96226389	11.4131377
Slc20a2	25.7943163	-0.6146229	0.33199008	-1.8513292	0.06412221	0.781195	Slc20a2	30.9141311	50.6826565	21.3112953	26.8858201	18.3129289	18.8168724	21.1698055	18.2610202
Kcnn1	6.05946575	-0.7501774	0.40528643	-1.8509809	0.06417231	0.781195	Kcnn1	8.73660227	8.59795066	6.36158069	10.3407001	0	4.7042181	2.88679167	6.84788259
Sox1	50.3252836	0.58900059	0.31824977	1.85074948	0.0642056	0.781195	Sox1	32.2582238	29.8665654	40.0779583	49.6353602	58.2684102	39.9858538	45.2264028	107.283494
Pcdhgb1	225.509941	-0.5604917	0.30285488	-1.8506941	0.06421357	0.781195	Pcdhgb1	276.211041	390.527969	277.682997	159.246781	233.073641	206.985596	109.698083	150.653417
Zdhhc14	19.8573592	-0.6993603	0.3779301	-1.8505017	0.06424127	0.781195	Zdhhc14	22.8495752	43.8948007	28.6271131	8.27256004	6.65924688	16.4647633	11.5471667	20.5436478
Creb3l1	5.43516634	-0.7700508	0.41630123	-1.8497443	0.06435042	0.781195	Creb3l1	2.68818531	17.1959013	7.63389683	4.13628002	1.66481172	2.35210905	0.96226389	6.84788259
Gata2	1.0168007	-0.6459158	0.34935802	-1.8488648	0.06447733	NA	Gata2	0.67204633	3.16766603	2.22655324	2.06814001	0	0	0	0
Snrpf	2.20278193	0.7275724	0.39359014	1.84855342	0.06452232	NA	Snrpf	0	0.90504744	0.63615807	2.06814001	4.99443516	0	6.73584722	2.28262753
Pcdhgb7	223.65855	-0.5616763	0.30391014	-1.8481657	0.06457837	0.781195	Pcdhgb7	273.522856	389.622922	277.046839	155.110501	226.414394	208.161651	108.735819	150.653417
Atp6ap1l	3.3662733	-0.7663738	0.41470206	-1.8480105	0.06460082	NA	Atp6ap1l	6.72046328	7.69290322	6.36158069	0	1.66481172	3.52816357	0.96226389	0
Dlx6os1	60.4169141	0.64101258	0.34693035	1.84766935	0.06465019	0.781195	Dlx6os1	46.3711967	42.0847059	63.6158069	20.6814001	33.2962344	105.844907	102.962236	68.4788259
Higd2a	76.7635931	-0.5816711	0.31492266	-1.8470285	0.06474301	0.781195	Higd2a	76.6132814	91.4097912	70.2954666	142.701661	71.586904	29.4013631	56.7735694	75.3267085
Pcnt	28.3500052	0.58149946	0.31485868	1.84685861	0.06476763	0.781195	Pcnt	23.5216215	20.8160911	15.5858727	26.8858201	39.9554813	36.4576902	20.2075417	43.3699231
Stard4	12.8484033	-0.6491053	0.35148472	-1.8467524	0.06478303	0.781195	Stard4	18.1452509	17.648425	13.3593194	18.6132601	8.3240586	10.5844907	11.5471667	4.56525506
Hbb-b1	9.58580471	0.71139808	0.38526392	1.84652141	0.06481653	0.781195	Hbb-b1	8.06455594	4.07271347	6.99773876	6.20442003	19.9777406	5.88027262	16.3584861	9.13051012
Hbb-bs	9.58580471	0.71139808	0.38526392	1.84652141	0.06481653	0.781195	Hbb-bs	8.06455594	4.07271347	6.99773876	6.20442003	19.9777406	5.88027262	16.3584861	9.13051012
Pcdhga8	229.423251	-0.5531284	0.2996076	-1.8461761	0.06486664	0.781195	Pcdhga8	276.211041	396.863302	282.772262	163.383061	234.738453	211.689814	114.509403	155.218672
Loh12cr1	27.8973052	0.55634728	0.30142589	1.84571834	0.06493311	0.781195	Loh12cr1	22.1775288	17.1959013	21.3112953	26.8858201	28.3017992	23.5210905	40.4150833	43.3699231
Dnajc21	8.94024525	0.72281104	0.39166533	1.8454813	0.06496756	0.781195	Dnajc21	8.73660227	5.43028463	7.63389683	0	11.653682	12.9365998	18.2830139	6.84788259
Rph3a	447.23719	0.4672706	0.25320678	1.84541106	0.06497777	0.781195	Rph3a	348.119998	310.431271	328.575643	483.944762	591.008161	519.8161	345.452736	650.548846
Nkd1	7.08848344	-0.7490862	0.40610674	-1.844555	0.0651023	0.78138251	Nkd1	13.4409266	9.5029981	6.04350166	10.3407001	8.3240586	3.52816357	0.96226389	4.56525506
Sim1	3.26052368	-0.7642432	0.41436005	-1.8443939	0.06512575	NA	Sim1	2.68818531	7.69290322	3.81694841	6.20442003	3.32962344	2.35210905	0	0
Purg	17.5403929	0.65155474	0.35346183	1.84335246	0.06527757	0.78138251	Purg	14.1129729	12.2181404	12.4050823	12.4088401	11.653682	38.8097993	25.0188611	13.6957652
Hspa14	20.6467748	0.56341256	0.30566251	1.84325048	0.06529245	0.78138251	Hspa14	18.8172972	14.480759	14.3135566	16.5451201	18.3129289	27.0492541	25.981125	29.6741579
Pcdhga12	233.574879	-0.5540927	0.30068523	-1.8427667	0.06536308	0.78138251	Pcdhga12	286.291736	400.483491	285.953052	167.519341	239.732888	217.570087	113.547139	157.5013
Ugt8a	10.3489944	-0.76072	0.41282516	-1.8427172	0.06537031	0.78138251	Ugt8a	12.7688802	25.3413283	19.7209001	0	4.99443516	4.7042181	3.84905555	11.4131377
Ogn	3.2534158	-0.7588918	0.41183526	-1.8427071	0.06537179	NA	Ogn	5.37637063	9.05047438	6.36158069	0	0	2.35210905	2.88679167	0
Kif5c	509.978293	0.5893061	0.32000185	1.84157094	0.06553794	0.78138251	Kif5c	397.17938	471.077191	424.95359	248.176801	432.851047	1067.85751	679.358305	358.372522
Gm5607	15.0127468	0.65802622	0.35732482	1.84153515	0.06554317	0.78138251	Gm5607	11.4247876	6.78785578	13.9954775	10.3407001	13.3184938	30.5774176	15.3962222	18.2610202
Nos1	134.746603	0.62652438	0.34026276	1.84129576	0.06557823	0.78138251	Nos1	91.3983007	73.7613662	85.2451812	144.769801	231.408829	169.351851	60.622625	221.414871
Spag5	1.54446381	-0.6807538	0.36988827	-1.840431	0.06570498	NA	Spag5	6.04841696	4.07271347	1.27231614	0	0	0	0.96226389	0
D7Ertd715e	5.08039892	0.76373723	0.41500092	1.84032658	0.06572031	0.78220337	D7Ertd715e	4.03227797	3.62018975	1.90847421	0	4.99443516	5.88027262	20.2075417	0
Rcan2	310.170413	0.45967189	0.24986656	1.83966951	0.06581677	0.78248016	Rcan2	258.737836	208.160911	256.371702	301.948441	228.079206	368.105066	407.999889	451.960251
Frg1	17.9828763	0.64961656	0.35348102	1.83776927	0.06609641	0.78493161	Frg1	14.7850192	15.8383302	10.4966081	8.27256004	23.3073641	10.5844907	24.0565972	36.5220405
Tgfbrap1	49.3799351	0.52503548	0.28606454	1.8353742	0.06645026	0.78825796	Tgfbrap1	46.3711967	33.9392789	40.3960374	33.0902402	73.2517157	39.9858538	48.1131944	79.8919636
Tmem254a	23.4223772	-0.6260085	0.3413091	-1.8341396	0.06663328	0.78955261	Tmem254a	24.1936678	41.1796584	33.3982986	20.6814001	13.3184938	15.2887088	27.9056528	11.4131377
Chd5	318.224564	0.65306191	0.35650348	1.83185284	0.06697335	0.79158244	Chd5	162.635211	143.450019	162.220308	438.445682	596.002596	323.414994	199.188625	520.439077
Ppfia3	116.333439	0.682379	0.37257757	1.83150851	0.06702468	0.79158244	Ppfia3	65.8605402	48.8725616	52.8011197	153.042361	258.045817	85.8519803	72.1697916	194.02334
Rimbp2	76.3146661	0.50037502	0.27320586	1.83149449	0.06702677	0.79158244	Rimbp2	55.7798453	52.4927514	52.8011197	86.8618804	101.553515	94.0843619	59.6603611	107.283494
Cnp	74.6869128	-0.6322388	0.34545854	-1.8301437	0.06722845	0.79289113	Cnp	77.9573741	64.7108918	64.570044	175.791901	88.2350212	37.6337448	45.2264028	43.3699231
Sytl5	21.2793539	0.71280346	0.38956134	1.82975921	0.06728595	0.79289113	Sytl5	16.1291119	13.5757116	17.4943469	8.27256004	11.653682	56.4506172	37.5282917	9.13051012
Asb4	3.39733548	-0.6433046	0.35178775	-1.8286724	0.06744869	NA	Asb4	0	1.81009488	0.9542371	22.7495401	1.66481172	0	0	0
Ghr	5.0556655	-0.7600698	0.41610843	-1.8266148	0.0677577	0.79624306	Ghr	6.72046328	7.2403795	12.4050823	4.13628002	0	7.05632714	2.88679167	0
Ino80	23.5704782	0.54493628	0.29837643	1.82633819	0.06779934	0.79624306	Ino80	20.1613899	19.9110436	17.8124259	16.5451201	28.3017992	32.9295267	34.6415	18.2610202
1110051M20Rik	56.4072132	-0.5015701	0.27469288	-1.8259304	0.06786074	0.79624306	1110051M20R	69.2207718	71.0462239	56.2999891	74.4530404	49.9443516	28.2253086	58.6980972	43.3699231
Cryl1	5.79251652	0.75742127	0.41482542	1.82587962	0.0678684	0.79624306	Cryl1	3.36023164	3.62018975	4.13502745	0	16.6481172	2.35210905	4.81131944	11.4131377
Adra1d	7.31238691	0.7391755	0.40505453	1.82487896	0.0680193	0.7969816	Adra1d	3.36023164	4.52523719	3.49886938	6.20442003	3.32962344	7.05632714	7.69811111	22.8262753
A930011O12Rik	11.4359482	0.75961601	0.41634801	1.82447374	0.06808048	0.7969816	A930011O12R	1.34409266	2.71514231	3.18079035	18.6132601	38.2906696	8.23238167	7.69811111	11.4131377
6430584L05Rik	2.87827482	-0.7587861	0.41590562	-1.824419	0.06808876	NA	6430584L05Ri	4.03227797	4.52523719	3.49886938	6.20442003	1.66481172	1.17605452	1.92452778	0
D1Pas1	2.81992623	0.75081512	0.4115551	1.82433682	0.06810117	NA	D1Pas1	2.01613899	1.35757116	1.59039517	0	1.66481172	8.23238167	7.69811111	0
Nnmt	1.52736665	0.67341529	0.36915562	1.82420433	0.06812119	NA	Nnmt	0.67204633	0	0.63615807	0	0	1.17605452	2.88679167	6.84788259
Ap3s1	12.4162988	-0.6886946	0.377697	-1.8234049	0.06824208	0.79767869	Ap3s1	19.4893435	18.5534725	16.8581888	12.4088401	3.32962344	12.9365998	13.4716944	2.28262753
Sox12	17.7246451	-0.6168188	0.33833642	-1.8230931	0.0682893	0.79767869	Sox12	25.5377605	17.648425	17.4943469	31.0221002	14.9833055	10.5844907	15.3962222	9.13051012
Bcl7a	84.6795751	0.42660951	0.23416317	1.82184721	0.06847817	0.79806026	Bcl7a	74.5971425	72.403795	65.5242811	70.3167603	118.201632	74.091435	90.4528055	111.848749
Rqcd1	28.3716898	-0.5738986	0.31501703	-1.8218018	0.06848507	0.79806026	Rqcd1	32.9302701	36.6544212	40.0779583	31.0221002	13.3184938	16.4647633	33.6792361	22.8262753
Blmh	27.0980334	0.54045279	0.29672372	1.8214007	0.06854597	0.79806026	Blmh	18.8172972	19.4585199	18.448584	31.0221002	39.9554813	27.0492541	34.6415	27.3915304
1700016K19Rik	1.43367626	-0.69985	0.38428934	-1.8211539	0.06858347	NA	1700016K19Ri	2.01613899	1.81009488	2.54463228	4.13628002	0	0	0.96226389	0
Ndufb4	11.2081364	-0.6704655	0.36829914	-1.8204373	0.06869243	0.79846093	Ndufb4	16.8011582	14.9332827	11.4508452	16.5451201	6.65924688	12.9365998	5.77358333	4.56525506
Pcdhga1	223.18814	-0.5545445	0.30475183	-1.8196595	0.06881088	0.79846093	Pcdhga1	272.178763	388.265351	275.138365	155.110501	228.079206	206.985596	106.811292	152.936045
Dnmt1	42.3246681	0.49032581	0.26946758	1.81960964	0.06881847	0.79846093	Dnmt1	40.3227797	31.6766603	30.8536663	33.0902402	63.2628454	39.9858538	42.3396111	57.0656883
Hif3a	3.13600668	-0.7506777	0.41255934	-1.8195629	0.0688256	NA	Hif3a	1.34409266	9.05047438	5.08926455	4.13628002	3.32962344	1.17605452	0.96226389	0
Asl	4.70384685	0.75517223	0.4151968	1.81882959	0.06893743	0.79846093	Asl	2.01613899	3.16766603	1.59039517	4.13628002	13.3184938	8.23238167	2.88679167	2.28262753
Frzb	6.99597384	-0.7242948	0.3982439	-1.8187216	0.06895391	0.79846093	Frzb	6.04841696	13.1231878	10.8146872	8.27256004	6.65924688	5.88027262	2.88679167	2.28262753
Rnh1	14.0608213	0.66197227	0.36408238	1.81819362	0.06903454	0.79852945	Rnh1	8.06455594	8.14542694	9.54237104	14.4769801	6.65924688	17.6408179	18.2830139	29.6741579
Pbxip1	18.3674178	-0.6929271	0.38138953	-1.8168488	0.06924027	0.79925727	Pbxip1	27.5538995	36.2018975	20.0389792	12.4088401	26.6369875	8.23238167	6.73584722	9.13051012
Sirt2	84.1293793	-0.4795247	0.2639385	-1.8168047	0.06924703	0.79925727	Sirt2	115.591968	103.627932	87.1536555	90.9981605	79.9109626	41.1619083	76.9811111	77.6093361
Gm11744	1.30241636	0.68040657	0.37475559	1.81560086	0.06943162	NA	Gm11744	0	0.45252372	0.63615807	0	1.66481172	1.17605452	1.92452778	4.56525506
Isca2	20.0100731	-0.6042066	0.33329543	-1.8128258	0.06985869	0.80429217	Isca2	28.2259458	21.2686148	27.990955	24.8176801	11.653682	12.9365998	24.0565972	9.13051012
6430548M08Rik	282.185473	0.46924551	0.25886108	1.81273105	0.06987331	0.80429217	6430548M08R	209.006408	217.211385	211.840637	287.471461	389.565943	328.119212	201.113153	413.155583
Bnip1	10.2786732	0.66308068	0.36584456	1.81246559	0.06991429	0.80429217	Bnip1	10.0806949	6.33533207	7.31581779	4.13628002	11.653682	9.40843619	17.32075	15.9783927
Mdk	2.68512539	-0.7269188	0.40112061	-1.81222	0.06995221	NA	Mdk	2.68818531	9.05047438	5.72542262	0	1.66481172	2.35210905	0	0
BC021891	3.28638843	0.75360169	0.41586092	1.81214836	0.06996328	NA	BC021891	2.01613899	0.90504744	2.22655324	2.06814001	4.99443516	4.7042181	4.81131944	4.56525506
Rbm19	6.60988475	0.71327831	0.39363872	1.81201256	0.06998426	0.80429217	Rbm19	6.72046328	4.52523719	3.49886938	2.06814001	6.65924688	12.9365998	9.62263889	6.84788259
Ccl21b	4.19631184	-0.75147	0.41488134	-1.8112888	0.07009615	NA	Ccl21b	8.73660227	5.88280835	4.45310648	6.20442003	1.66481172	4.7042181	1.92452778	0
Lonrf3	16.8718062	-0.6416929	0.35436824	-1.8108082	0.07017055	0.80472968	Lonrf3	24.1936678	25.3413283	23.2197695	12.4088401	14.9833055	17.6408179	5.77358333	11.4131377
Adamts17	3.15683893	0.75389653	0.41635194	1.81071937	0.0701843	NA	Adamts17	2.01613899	1.35757116	1.27231614	2.06814001	4.99443516	7.05632714	1.92452778	4.56525506
Sstr2	11.2104639	-0.7322638	0.40446997	-1.810428	0.07022944	0.80472968	Sstr2	10.0806949	25.793852	16.2220308	10.3407001	3.32962344	16.4647633	2.88679167	4.56525506
Ddost	46.602901	-0.5798882	0.32039314	-1.8099271	0.07030709	0.80472968	Ddost	43.010965	85.9795066	48.6660923	51.7035003	26.6369875	43.5140174	23.0943333	50.2178057
Bloc1s1	7.60060988	-0.7134264	0.39419716	-1.8098214	0.0703235	0.80472968	Bloc1s1	10.0806949	7.69290322	10.8146872	14.4769801	1.66481172	7.05632714	6.73584722	2.28262753
Slc32a1	218.832905	0.64493983	0.35653027	1.80893429	0.07046121	0.80544324	Slc32a1	102.823088	125.34907	117.053085	281.267041	306.325357	276.372813	107.773556	433.699231
Cp	2.8572441	-0.7209524	0.39881532	-1.8077351	0.07064774	NA	Cp	2.01613899	8.59795066	7.95197586	0	3.32962344	0	0.96226389	0
Srgap3	524.349414	0.4434956	0.24535241	1.80758603	0.07067095	0.80697772	Srgap3	386.426639	409.533966	434.495961	512.898723	710.874605	519.8161	419.547055	801.202263
Ogfod2	11.4893744	0.73085019	0.40469021	1.80594976	0.07092619	0.80701448	Ogfod2	6.72046328	4.52523719	7.63389683	8.27256004	14.9833055	9.40843619	3.84905555	36.5220405
Fcgr3	2.89181547	-0.7380204	0.40875857	-1.8055166	0.07099388	NA	Fcgr3	2.68818531	9.95552182	5.72542262	0	1.66481172	1.17605452	1.92452778	0
Sptb	56.0633296	0.71110051	0.39386706	1.8054328	0.07100698	0.80701448	Sptb	30.9141311	17.1959013	29.5813502	68.2486203	161.486737	38.8097993	26.9433889	75.3267085
Sgpl1	9.15644358	-0.6934113	0.3840958	-1.8053082	0.07102646	0.80701448	Sgpl1	13.4409266	16.2908539	13.3593194	6.20442003	4.99443516	7.05632714	9.62263889	2.28262753
Dnaic1	1.1413535	-0.6064353	0.33610265	-1.8043158	0.07118181	NA	Dnaic1	6.04841696	1.81009488	1.27231614	0	0	0	0	0
Tbp	12.9321404	-0.6933118	0.3842658	-1.8042506	0.07119203	0.80701448	Tbp	19.4893435	22.1736622	21.6293743	6.20442003	6.65924688	10.5844907	14.4339583	2.28262753

GeneID	Base mean	log2(FC)	StdErr	Wald-Stats	P-value	P-adj	GeneID	Normalised expression for Chow#1	Normalised expression for Chow#2	Normalised expression for Chow#3	Normalised expression for Chow#4	Normalised expression for HFD#1	Normalised expression for HFD#2	Normalised expression for HFD#3	Normalised expression for HFD#4
Zfp358	27.029787	-0.5907594	0.32762439	-1.8031606	0.07136299	0.80701448	Zfp358	32.2582238	28.961518	28.3090341	45.4990802	33.2962344	12.9365998	14.4339583	20.5436478
Srsf11	31.9585115	0.49553825	0.27486449	1.80284564	0.07141246	0.80701448	Srsf11	30.9141311	22.1736622	26.0824808	24.8176801	38.2906696	42.3379629	41.3773472	29.6741579
Atxn7l1	38.4110289	-0.51217	0.28411984	-1.8026549	0.07144244	0.80701448	Atxn7l1	45.027104	59.2806072	42.9406697	35.1583802	38.2906696	30.5774176	24.0565972	31.9567854
Cml3	1.06855085	-0.6072349	0.33688941	-1.8024754	0.07147064	NA	Cml3	1.34409266	4.97776091	2.22655324	0	0	0	0	0
Nelfcd	22.4454303	0.5439275	0.3018074	1.80223384	0.07150862	0.80701448	Nelfcd	19.4893435	15.8383302	18.448584	14.4769801	31.6314227	22.345036	23.0943333	34.239413
Tmem98	2.58263738	-0.7191153	0.3990218	-1.8021956	0.07151464	NA	Tmem98	2.01613899	7.2403795	4.77118552	2.06814001	0	0	0	4.56525506
Btbd9	66.1940712	0.52691038	0.29248835	1.80147475	0.07162808	0.80701448	Btbd9	53.7637063	41.1796584	43.5768277	72.3849004	114.872009	63.5069443	55.8113055	84.4572186
Kalrn	197.746376	0.47751834	0.26512085	1.80113463	0.07168166	0.80701448	Kalrn	215.054825	145.712637	167.94573	113.747701	324.638285	223.45036	208.811264	182.610202
Aldh5a1	97.5242217	0.43357652	0.24073419	1.80105916	0.07169356	0.80701448	Aldh5a1	67.8766792	74.2138899	94.4694732	90.9981605	96.5590798	145.830761	102.962236	107.283494
Rhoj	1.43465823	-0.6647836	0.36920801	-1.8005666	0.07177121	NA	Rhoj	4.7043243	1.35757116	4.45310648	0	0	0	0.96226389	0
Slc4a2	11.5186061	-0.6957689	0.38645775	-1.800375	0.07180145	0.80701448	Slc4a2	10.7527413	16.7433776	11.4508452	22.7495401	9.98887032	9.40843619	1.92452778	9.13051012
Fam214a	16.8506619	0.61149874	0.33970197	1.80010363	0.07184428	0.80701448	Fam214a	8.73660227	10.8605693	18.448584	10.3407001	26.6369875	19.9929269	19.2452778	20.5436478
Crtc3	16.3786894	-0.61101	0.33951471	-1.7996568	0.07191484	0.80701448	Crtc3	18.1452509	15.8383302	20.0389792	28.9539601	11.653682	11.7605452	8.660375	15.9783927
B230209E15Rik	73.007697	0.55135071	0.30650057	1.79885703	0.0720413	0.80701448	B230209E15Ri	44.3550577	55.2078937	53.4372778	74.4530404	44.9499164	92.9083074	81.7924305	136.957652
Cyp4v3	1.83714655	-0.6765783	0.37614974	-1.7986941	0.07206709	NA	Cyp4v3	1.34409266	4.07271347	6.99773876	0	0	0	0	2.28262753
Itga5	0.9692073	-0.6300254	0.35031837	-1.7984368	0.07210782	NA	Itga5	2.01613899	2.71514231	0.9542371	2.06814001	0	0	0	0
Pacsin2	37.0431459	0.49000013	0.27247248	1.79834724	0.072122	0.80701448	Pacsin2	32.2582238	23.9837571	34.9886938	28.9539601	39.9554813	54.0985081	43.301875	38.804668
Ptpn11	135.806082	0.45762479	0.25458672	1.79752026	0.07225306	0.80701448	Ptpn11	92.070347	92.7673624	108.464951	117.178641	181.464478	139.950488	125.094306	189.458085
Mllt4	69.768963	0.48167771	0.26798182	1.7974268	0.07226789	0.80701448	Mllt4	62.5003085	55.6604174	64.888123	43.4309402	84.9053977	110.549125	83.7169583	52.5004332
Nwd1	40.9929344	-0.6182424	0.34419769	-1.7961841	0.07246525	0.80701448	Nwd1	59.8121232	63.8058444	48.0299342	35.1583802	59.9332219	21.1689814	24.0565972	15.9783927
Pwwp2b	9.65717531	-0.6888136	0.38350666	-1.796093	0.07247972	0.80701448	Pwwp2b	8.73660227	14.0282353	11.4508452	16.5451201	9.98887032	3.52816357	3.84905555	9.13051012
Otx1	3.24502942	-0.7463622	0.41556055	-1.7960371	0.07248862	NA	Otx1	5.37637063	4.07271347	3.81694841	6.20442003	0	0	1.92452778	4.56525506
Cplx1	902.995848	0.59643503	0.33208654	1.7960229	0.07249088	0.80701448	Cplx1	604.169649	483.295332	626.933777	986.502785	1107.09979	635.069443	744.79225	2036.10376
Mad2l2	4.91230528	-0.7455165	0.41521792	-1.7954825	0.07257685	0.80701448	Mad2l2	2.68818531	5.88280835	6.36158069	14.4769801	3.32962344	2.35210905	1.92452778	2.28262753
Slc26a2	2.74969384	-0.7460204	0.41550462	-1.7954562	0.07258104	NA	Slc26a2	4.03227797	5.88280835	3.18079035	4.13628002	1.66481172	1.17605452	1.92452778	0
Rad54l2	29.7095685	0.55577561	0.30958827	1.79520886	0.07262043	0.80701448	Rad54l2	18.8172972	28.5089943	17.8124259	26.8858201	51.6091633	30.5774176	26.9433889	36.5220405
Rfx4	17.9188202	-0.7091901	0.39516926	-1.7946488	0.07270967	0.80701448	Rfx4	15.4570656	17.1959013	16.2220308	49.6353602	23.3073641	4.7042181	7.69811111	9.13051012
Col19a1	9.97697392	0.74197586	0.41349419	1.79440457	0.07274862	0.80701448	Col19a1	4.03227797	5.43028463	9.224292	4.13628002	4.99443516	35.2816357	14.4339583	2.28262753
Zfp658	4.76828916	0.73016453	0.40699368	1.79404391	0.07280616	0.80701448	Zfp658	2.68818531	2.71514231	2.54463228	4.13628002	4.99443516	9.40843619	4.81131944	6.84788259
Gstt3	2.76621015	-0.6622756	0.36915273	-1.7940424	0.07280641	NA	Gstt3	0.67204633	1.35757116	1.27231614	16.5451201	0	0	0	2.28262753
As3mt	4.52556274	-0.7464351	0.41635039	-1.7928052	0.07300408	NA	As3mt	4.7043243	4.52523719	6.04350166	12.4088401	4.99443516	3.52816357	0	0
Arc	30.0931051	-0.6385587	0.35626418	-1.7923742	0.07307304	0.80701448	Arc	59.8121232	24.8888045	33.0802196	35.1583802	28.3071992	21.1689814	8.660375	29.6741579
Tram1	20.5437861	-0.6400728	0.35713356	-1.7922506	0.07309283	0.80701448	Tram1	21.5054825	39.8220873	27.990955	16.5451201	6.65924688	21.1689814	19.2452778	11.4131377
Dgka	5.87176713	0.73436631	0.40975432	1.79221126	0.07309913	0.80701448	Dgka	4.7043243	1.81009488	4.13502745	4.13628002	4.99443516	15.2887088	9.62263889	2.28262753
Fmnl2	34.4445953	-0.6930676	0.38681582	-1.791725	0.07317702	0.80701448	Fmnl2	53.0916599	70.5937002	41.0321955	20.6814001	8.3240586	29.4013631	43.301875	9.13051012
Trank1	100.005954	0.625959	0.34951132	1.79095488	0.07330054	0.80701448	Trank1	46.3711967	42.5372296	51.8468826	155.110501	156.492302	139.950488	116.433931	91.3051012
Spry4	37.0594109	0.6095843	0.34037271	1.79093178	0.07330424	0.80701448	Spry4	25.5377605	19.9110436	20.0389792	45.4990802	51.6091633	32.9295267	27.9056528	73.044081
Ppargc1a	92.2474729	0.52397916	0.29263026	1.79058433	0.07336002	0.80701448	Ppargc1a	70.5648645	72.403795	89.3820087	59.9760603	84.9053977	164.647633	129.905625	66.1961984
B230219D22Rik	122.313861	0.37754206	0.21086298	1.79046153	0.07337975	0.80701448	B230219D22R	99.4628566	104.532979	116.098848	101.338861	119.866444	136.422325	168.39618	132.392397
Rufy2	36.8350235	0.48326946	0.26996753	1.79010218	0.07343749	0.80701448	Rufy2	33.6023164	26.6988994	29.8994292	31.0221002	33.2962344	49.39429	51.96225	38.804668
Mir684-1	9.6004603	-0.7277194	0.40653074	-1.7900724	0.07344228	0.80701448	Mir684-1	14.7850192	19.9110436	9.224292	10.3407001	3.32962344	1.17605452	13.4716944	4.56525506
Slc26a11	3.11736082	0.70714804	0.39513023	1.78965816	0.07350888	NA	Slc26a11	0	1.81009488	0.63615807	2.06814001	6.65924688	2.35210905	0	11.4131377
C1qa	6.26604449	-0.732082	0.40906679	-1.7896392	0.07351193	0.80701448	C1qa	6.72046328	21.2686148	8.90621297	2.06814001	9.98887032	1.17605452	0	0
Fam227a	0.97003267	0.6022299	0.33654489	1.78944892	0.07354255	NA	Fam227a	0	0.45252372	0	0	0	1.17605452	3.84905555	2.28262753
Gm9079	1.83370485	0.708208	0.39585088	1.78907776	0.07360229	NA	Gm9079	2.01613899	0.45252372	0.31807903	0	3.32962344	4.7042181	3.84905555	0
Myoc	5.21375934	-0.7320551	0.40935864	-1.7882976	0.07372801	0.80701448	Myoc	6.04841696	9.05047438	7.95197586	6.20442003	3.32962344	5.88027262	0.96226389	2.28262753
Susd2	7.37467547	-0.7171541	0.40109214	-1.7880035	0.07377545	0.80701448	Susd2	7.39250961	15.3858064	10.4966081	6.20442003	8.3240586	4.7042181	1.92452778	4.56525506
Chd2	46.3158739	0.47378834	0.26501092	1.78780686	0.07380717	0.80701448	Chd2	42.3389187	31.6766603	40.7141164	37.2265202	41.620293	70.5632714	53.8867778	52.5004332
Bsn	453.497768	0.60451897	0.33822994	1.78730177	0.07388873	0.80701448	Bsn	253.361466	450.713624	308.854743	330.902402	1010.54071	580.970935	233.830125	458.808134
Psmd1	96.8657311	0.43569096	0.24382044	1.7869337	0.07394821	0.80701448	Psmd1	86.6939764	73.3088425	88.7440506	74.4530404	88.2350212	152.887088	101.037708	109.566121
Impact	396.255352	0.46888093	0.26240712	1.78684527	0.0739625	0.80701448	Impact	293.684246	264.273852	349.568859	395.014742	319.64385	585.675153	594.679083	367.503033
Necab2	78.5832873	-0.5253324	0.29407948	-1.7863621	0.07404064	0.80701448	Necab2	104.839227	93.2198861	93.1971571	88.9300204	59.9332219	30.5774176	89.4905416	68.4788259
Cbx2	1.01065199	-0.6130953	0.34328507	-1.7859655	0.07410484	NA	Cbx2	0.67204633	4.07271347	1.27231614	2.06814001	0	0	0	0
Hsdl2	20.9935422	-0.6947884	0.38915806	-1.7853629	0.07420248	0.80701448	Hsdl2	36.2950017	25.793852	31.4898244	20.6814001	3.32962344	11.7605452	31.7547083	6.84788259
P2ry13	1.56964918	-0.6446046	0.36105224	-1.7853499	0.07420458	NA	P2ry13	0.67204633	6.78785578	4.13502745	0	0	0	0.96226389	0
Cdk9	25.909257	0.55269924	0.30962185	1.78507829	0.07424862	0.80701448	Cdk9	19.4893435	20.8160911	16.5401098	22.7495401	31.6314227	21.1689814	26.9433889	47.9351782
Coq10b	15.8286566	-0.6503261	0.36439382	-1.7846794	0.07431334	0.80701448	Coq10b	28.2259458	11.313093	20.3570582	22.7495401	6.65924688	8.23238167	15.3962222	13.6957652
Prr14l	79.7289256	0.42116916	0.23607228	1.78406864	0.07441251	0.80701448	Prr14l	58.4680306	71.0462239	71.2497037	66.1804803	99.8887032	105.844907	69.283	95.8703563
Ubash3b	32.5977795	0.5344334	0.29957761	1.78395641	0.07443075	0.80701448	Ubash3b	24.8657142	27.1514231	28.6271131	20.6814001	21.6425524	51.7463991	40.4150833	45.6525506
C1ql1	11.1775953	-0.6997242	0.39229207	-1.7836816	0.07447541	0.80701448	C1ql1	14.1129729	18.1009488	13.0412404	14.4769801	6.65924688	12.9365998	0.96226389	9.13051012
Zkscan2	6.75009873	0.7068202	0.39633814	1.78337667	0.07452501	0.80701448	Zkscan2	4.03227797	3.62018975	6.67965972	2.06814001	6.65924688	14.1126543	7.69811111	9.13051012
Eif3b	61.6671718	0.52929461	0.2969077	1.78269072	0.07463666	0.80709269	Eif3b	45.027104	44.3473245	44.2129858	59.9760603	81.5757743	51.7463991	50.0377222	116.414004
Cep131	9.64957352	0.71832845	0.4030353	1.78229661	0.07470088	0.80709269	Cep131	7.39250961	1.81009488	5.08926455	10.3407001	14.9833055	7.05632714	7.69811111	22.8262753
Gm15800	414.351099	0.61764199	0.34661182	1.78194149	0.07475877	0.80709269	Gm15800	290.99606	248.888045	243.012382	430.173122	1022.1944	349.288194	246.339555	483.917037
Pcdhgb6	230.574593	-0.5393904	0.3027775	-1.7814744	0.07483499	0.80710023	Pcdhgb6	280.915365	393.695635	280.863787	165.451201	241.397699	216.394032	110.660347	155.218672
Usp24	58.5776243	0.45800058	0.25720343	1.7806939	0.07496247	0.80766016	Usp24	43.010965	54.75537	50.5745665	45.4990802	79.9109626	76.4435441	75.0565833	43.3699231
Phf5a	7.80234868	-0.7174952	0.4032719	-1.7791848	0.07520946	0.80869548	Phf5a	8.73660227	6.78785578	10.4966081	16.5451201	6.65924688	1.17605452	2.88679167	9.13051012
Entpd1	5.45375116	-0.7349666	0.41313917	-1.7789806	0.07524294	0.80869548	Entpd1	3.36023164	10.4080455	6.04350166	12.4088401	4.99443516	3.52816357	2.88679167	0
Apoa1bp	36.3519892	-0.6526206	0.36690447	-1.7787208	0.07528555	0.80869548	Apoa1bp	45.027104	37.5594687	29.8994292	76.5211804	29.966611	7.05632714	25.981125	38.804668
Zc3h18	37.0815393	0.73514236	0.41341492	1.77821922	0.07536786	0.80876672	Zc3h18	16.1291119	9.5029981	8.90621297	51.7035003	71.586904	19.9929269	11.5471667	107.283494
Tmbim6	156.55388	-0.5005095	0.28172934	-1.7765614	0.07564043	0.810277	Tmbim6	116.264015	249.340569	202.298266	179.928181	134.849745	145.830761	84.6792222	139.240279
Arhgef19	8.504306	-0.7267709	0.40911619	-1.7764412	0.07566022	0.810277	Arhgef19	10.7527413	8.14542694	6.99773876	22.7495401	11.653682	3.52816357	1.92452778	2.28262753
Ramp2	9.03282462	-0.7159315	0.4034603	-1.774478	0.07598411	0.8129311	Ramp2	6.04841696	21.7211385	10.4966081	10.3407001	8.3240586	2.35210905	3.84905555	9.13051012
9130221H12Rik	104.987583	-0.5496272	0.30987997	-1.7736776	0.07611649	0.81353304	9130221H12Ri	100.806949	127.159165	118.961559	165.451201	91.5646446	42.3379629	63.5094166	130.109769
Nmi	0.91879054	-0.6221347	0.35125409	-1.7711813	0.07653056	NA	Nmi	2.01613899	1.35757116	1.90847421	2.06814001	0	0	0	0
Scrt1	213.836966	0.72264934	0.40814626	1.77056462	0.07663313	0.81823665	Scrt1	79.9735131	70.5937002	79.2016796	287.471461	336.291968	105.844907	77.943375	673.375122
Il13ra1	3.37440239	-0.7193175	0.40633094	-1.770275	0.07668134	NA	Il13ra1	3.36023164	12.6706641	5.72542262	0	0	2.35210905	2.88679167	0
Zfp775	6.86610836	0.73187875	0.41361212	1.7694809	0.07681366	0.81861732	Zfp775	2.01613899	4.07271347	1.27231614	10.3407001	14.9833055	10.5844907	4.81131944	6.84788259
Fam155a	106.060765	0.39793403	0.22489377	1.76943106	0.07682197	0.81861732	Fam155a	76.6132814	90.0522201	89.6982877	105.475141	121.531256	132.894161	95.264125	136.957652
Unc93b1	2.14817946	-0.7068753	0.39956292	-1.7691214	0.07687362	NA	Unc93b1	3.36023164	5.88280835	2.54463228	2.06814001	3.32962344	0	0	0
Aif1	1.18042691	0.64628586	0.36575383	1.76699846	0.07722876	NA	Aif1	0	0.45252372	0.63615807	0	3.32962344	1.17605452	3.84905555	0
Serpinh1	5.00421136	-0.7353248	0.41615653	-1.7669429	0.07723779	0.82168157	Serpinh1	2.68818531	11.313093	6.67965972	8.27256004	6.65924688	1.17605452	0.96226389	2.28262753
Ntn4	4.83252833	0.73235435	0.41451109	1.76679072	0.07726328	0.82168157	Ntn4	3.36023164	2.26261859	2.22655324	4.13628002	3.32962344	15.2887088	5.77358333	2.28262753
Anp32a	94.0942823	0.44982322	0.25490027	1.76470282	0.07761373	0.82271141	Anp32a	75.2691888	59.7331309	67.7508343	113.747701	101.553515	96.436471	124.132042	114.131377
Suv39h2	4.06035314	0.73458805	0.41630593	1.76453898	0.0776428	NA	Suv39h2	3.36023164	2.26261859	2.22655324	0	0	5.88027262	9.62263889	9.13051012
Scn1a	119.977252	0.65796821	0.37306365	1.76368888	0.07778437	0.82271141	Scn1a	67.2046328	100.007742	111.645741	53.7716403	76.5813391	351.640303	114.509403	84.4572186
Psmd13	20.494954	0.58744512	0.33308467	1.76365105	0.07779075	0.82271141	Psmd13	12.7688802	11.7656167	19.0847421	18.6132601	13.3184938	24.697145	31.7547083	31.9567854
Serinc4	10.8584872	-0.6676582	0.37861351	-1.763429	0.07782809	0.82271141	Serinc4	12.7688802	20.8160911	14.3135566	8.27256004	4.99443516	11.7605452	4.81131944	9.13051012
Tmem14a	12.8490831	0.60585816	0.34363509	1.76308583	0.07788601	0.82271141	Tmem14a	8.73660227	12.2181404	9.54237104	6.20442003	16.6481172	19.9929269	13.4716944	15.9783927
Azi2	65.1927468	0.51104243	0.28989306	1.76286535	0.0779232	0.82271141	Azi2	57.1239379	56.1129411	62.0254117	31.0221002	54.9387868	90.5561984	105.849028	63.9135709
Nap1l5	518.3246	0.61086868	0.34657271	1.76259889	0.07796816	0.82271141	Nap1l5	377.690037	348.443264	453.580703	343.311242	231.408829	823.238167	1130.66007	438.264486
Pja1	112.769472	0.45661906	0.25906687	1.76255293	0.07797592	0.82271141	Pja1	118.9522	61.5432258	105.28416	84.7937404	151.497867	130.542052	112.584875	136.957652
Pcdhb2	3.5407431	0.69777639	0.39593654	1.76234399	0.0780112	NA	Pcdhb2	0	4.07271347	0.9542371	0	1.66481172	16.4647633	2.88679167	2.28262753
Sep-04	35.0259478	-0.5550585	0.31517435	-1.7611155	0.07821885	0.82317556	Sep-04	33.6023164	32.129184	40.0779583	68.2486203	29.966611	17.6408179	28.8679167	29.6741579
Adap2	1.36842634	-0.6563507	0.37276229	-1.7607756	0.07827639	NA	Adap2	2.01613899	3.62018975	4.13502745	0	0	1.17605452	0	0
Pea15a	371.618649	-0.398147	0.22613154	-1.7606877	0.07829128	0.82317556	Pea15a	464.384013	440.758102	364.518574	438.445682	317.979039	219.922196	386.830083	340.111502
Nrgn	697.92665	-0.5841431	0.33189573	-1.76002	0.07840441	0.82317556	Nrgn	1054.44069	913.645389	889.985139	620.442003	667.5895	194.048996	458.037611	785.223871
Klhl11	69.3122154	0.39381662	0.22386521	1.75916849	0.0785489	0.82317556	Klhl11	51.7475673	55.6604174	62.3434908	68.2486203	86.5702095	77.6195986	76.9811111	75.3267085
Supt6	116.145955	0.52019293	0.29583578	1.75838408	0.07868218	0.82317556	Supt6	128.360849	71.9512713	80.1559167	86.8618804	213.0959	139.950488	92.3773333	116.414004
Pcdhga4	231.452999	-0.528275	0.30046739	-1.7581775	0.0787173	0.82317556	Pcdhga4	280.915365	397.315825	280.545708	161.314921	239.732888	209.337705	113.547139	168.914437
Tcn2	5.6617197	-0.7318877	0.41629867	-1.7580832	0.07873335	0.82317556	Tcn2	6.72046328	15.3858064	8.58813393	2.06814001	8.3240586	0	1.92452778	2.28262753
Serbp1	137.666057	0.51082357	0.29058966	1.75788629	0.07876686	0.82317556	Serbp1	129.704941	117.656167	114.508452	76.5211804	114.872009	239.915123	196.301833	111.848749
Ndrg1	31.2680897	-0.5632016	0.3204012	-1.757801	0.07878137	0.82317556	Ndrg1	32.2582238	36.2018975	27.0367179	59.9760603	29.966611	19.9929269	17.32075	27.3915304
Trim59	4.85739659	-0.7231938	0.4114928	-1.7574883	0.07883461	0.82317556	Trim59	6.72046328	8.59795066	8.2700549	4.13628002	3.32962344	5.88027262	1.92452778	0
Recql5	7.69866705	0.6660669	0.37903174	1.75728528	0.0788692	0.82317556	Recql5	7.39250961	5.43028463	5.08926455	2.06814001	9.98887032	10.5844907	9.62263889	11.4131377
Vav3	3.15209983	-0.7294841	0.41530851	-1.7564872	0.07900525	NA	Vav3	6.04841696	3.16766603	4.77118552	6.20442003	0	1.17605452	3.84905555	0
Plekhf2	4.65765359	-0.7311571	0.41630924	-1.7562836	0.07903999	0.82317556	Plekhf2	5.37637063	8.14542694	8.90621297	4.13628002	0	0	3.84905555	6.84788259
Rph3al	3.74669236	-0.7284615	0.41484635	-1.755979	0.07909199	NA	Rph3al	4.7043243	4.97776091	2.54463228	10.3407001	1.66481172	1.17605452	0	4.56525506
Tbck	31.9924624	0.54195883	0.30872429	1.7554784	0.07917751	0.82317556	Tbck	32.2582238	27.1514231	31.1717454	12.4088401	36.6258578	43.5140174	48.1131944	29.6741579
Rab40b	18.0930784	-0.6178228	0.35197285	-1.7553139	0.07920564	0.82317556	Rab40b	27.5538995	27.1514231	24.4920857	10.3407001	8.3240586	10.5844907	13.4716944	22.8262753
Ddx51	14.8411477	0.64245813	0.36613736	1.75469154	0.07931208	0.82317556	Ddx51	10.0806949	7.2403795	11.4508452	12.4088401	21.6425524	15.2887088	8.660375	31.9567854
Ate1	90.1549366	0.44731836	0.2549445	1.75457149	0.07933263	0.82317556	Ate1	79.3014668	55.2078937	76.6570473	90.9981605	76.5813391	124.66178	117.396194	100.435611

GeneID	Base mean	log2(FC)	StdErr	Wald-Stats	P-value	P-adj
Jagn1	16.947069	-0.6670525	0.38033273	-1.7538657	0.07945352	0.82317556
Ankrd34b	47.7133961	0.54695146	0.31196321	1.75325628	0.07955803	0.82317556
Rnase4	1.92073591	-0.7004776	0.39955192	-1.7531579	0.07957491	NA
Ltv1	7.69464464	-0.6764247	0.3858344	-1.7531477	0.07957666	0.82317556
Zrsr1	87.0329487	0.4329934	0.24701588	1.75289702	0.07961968	0.82317556
Ints1	68.5120891	0.65388625	0.3730536	1.75279439	0.07963731	0.82317556
Hps6	7.9518326	0.7181847	0.41010848	1.75120668	0.07991032	0.82519947
Snx29	4.63464938	0.72883313	0.41635443	1.75051127	0.08003013	NA
Slc41a1	38.9382759	-0.5768738	0.32954773	-1.7505014	0.08003183	0.82565656
Ccdc55	13.5290766	0.67675943	0.3869161	1.74911157	0.08027174	0.82579009
Celsr3	39.8517544	0.55855787	0.31939395	1.74880538	0.08032467	0.82579009
9530052E02Rik	1.21787242	0.59344589	0.33936545	1.74869271	0.08034415	NA
Phf20	52.7672274	0.49103212	0.2808201	1.74856475	0.08036628	0.82579009
Znf512b	30.6784572	0.47960092	0.27433176	1.74825154	0.08042048	0.82579009
Mafg	24.7963892	0.61897308	0.35406506	1.74819025	0.08043109	0.82579009
Eva1a	3.18590563	-0.7264786	0.4156106	-1.7479791	0.08046765	NA
Cobl	65.1964544	-0.5773018	0.33042478	-1.7471505	0.08061123	0.82612206
Srcin1	300.121181	0.63397454	0.36288424	1.74704344	0.0806298	0.82612206
Exosc7	8.55371262	0.67948903	0.3890512	1.74652857	0.08071915	0.82612206
Atrip	11.7791795	0.67501097	0.38655535	1.74622075	0.0807726	0.82612206
Isoc2b	2.35711793	-0.7061566	0.40445982	-1.7459253	0.08082394	NA
Rps6kc1	38.1008732	0.49244689	0.28209059	1.74570481	0.08086226	0.8262484
Basp1	1103.74837	0.72434968	0.41514019	1.7448315	0.0810142	0.82701032
Smim11	3.36174411	-0.7138643	0.40937795	-1.743778	0.08119781	NA
Pradc1	4.6601639	-0.722529	0.41438516	-1.7436169	0.08122591	0.82807853
Rtkn2	1.56017439	0.66613099	0.3820433	1.74360073	0.08122874	NA
Vwa5a	8.17791751	-0.6723839	0.3857242	-1.7431727	0.08130345	0.82807853
Plch2	43.4977376	0.55385566	0.31782352	1.74265159	0.08139449	0.82807853
Amer3	26.4494194	0.5542666	0.31809511	1.74245559	0.08142876	0.82807853
Tcf7l1	4.99827529	-0.7195503	0.41330082	-1.7409844	0.08168632	0.82990817
Rnft1	5.66545323	-0.7240593	0.41636829	-1.7389876	0.08203695	0.83118107
Huwe1	371.434278	0.48639975	0.27973782	1.73877007	0.08207522	0.83118107
Tbc1d9b	133.386074	0.46760992	0.26894177	1.73870325	0.08208697	0.83118107
Etv4	3.41642909	-0.7146797	0.41112431	-1.7383543	0.0821484	NA
Gm20594	3.44246674	0.69657181	0.40076298	1.73811415	0.0821907	NA
Polr1b	11.4929192	0.63102028	0.36312248	1.73776152	0.08225284	0.83118107
Reep6	11.4822408	-0.6765327	0.3893314	-1.7376781	0.08226754	0.83118107
Fam195a	5.10959974	-0.7115442	0.4094941	-1.7376176	0.08227822	0.83118107
Crocc	13.9937634	0.71944941	0.41416462	1.73710978	0.0823678	0.83130029
Cuedc2	43.444032	0.45615014	0.26272967	1.73619578	0.08252922	0.83213308
Gm2506	2.80019752	0.72014617	0.41493578	1.73556056	0.08264157	NA
Tjp2	14.2332494	-0.634445	0.36568533	-1.734948	0.08275001	0.83213308
Zkscan14	4.5253405	-0.7222193	0.41633612	-1.7347025	0.08279351	NA
Prkd1	5.216044	-0.7054518	0.40670908	-1.7345366	0.08282291	0.83213308
Mtor	88.5524567	0.55190203	0.31827298	1.73405244	0.08290878	0.83213308
BC020402	2.38048083	-0.7111545	0.41017698	-1.7337749	0.08295803	NA
Pcdhb22	6.81438131	0.69990185	0.40394022	1.73268672	0.08315137	0.83213308
Derl3	1.66779377	-0.621892	0.35901327	-1.7322257	0.08323338	NA
Rbfox2	286.395957	0.42495609	0.24536905	1.73190583	0.08329033	0.83213308
Set	96.3230967	0.43965146	0.25388937	1.73166552	0.08333313	0.83213308
Lonp1	35.1355203	0.57508499	0.33212279	1.7315433	0.08335491	0.83213308
Tssc1	21.16352	0.56032642	0.32360788	1.73149809	0.08336297	0.83213308
Nt5m	28.0080424	0.54650049	0.31562302	1.73149755	0.08336306	0.83213308
Pkig	23.1152407	-0.6369157	0.36791446	-1.7311517	0.08342471	0.83213308
Znrf3	13.1601058	-0.6838648	0.39506904	-1.7310008	0.08345162	0.83213308
Usp54	42.666443	-0.4795101	0.27702296	-1.7309401	0.08346245	0.83213308
Rasl10a	17.495763	-0.6208326	0.35887094	-1.7299607	0.08363729	0.83309912
Gan	5.45962118	0.71046627	0.41100165	1.72862145	0.08387687	0.8347076
Plcl2	55.0350617	0.4470999	0.25877248	1.72777225	0.08402906	0.83532818
Arntl	15.5522623	-0.6039443	0.34962585	-1.7274017	0.08409554	0.83532818
Cdc34	9.81270042	-0.6596821	0.38200847	-1.7268782	0.08418955	0.8354792
Vstm2a	133.498578	0.64355412	0.37276223	1.72644669	0.08426708	0.8354792
Hs3st3b1	0.89583625	-0.6025618	0.34909419	-1.7260723	0.08433441	NA
Ubr4	190.916186	0.58050863	0.33633205	1.72599855	0.08434767	0.83550318
0610007P14Rik	23.7006427	-0.6047214	0.35053081	-1.7251592	0.08449879	0.83581334
Elovl5	26.9134347	-0.693784	0.40220386	-1.7249561	0.08453539	0.83581334
Bmpr1b	8.49666079	-0.7177178	0.41634398	-1.7238578	0.08473352	0.83609956
Ssr4	14.8962606	-0.6195743	0.35945583	-1.723645	0.08477194	0.83609956
Gsg1l	36.1811336	-0.5234104	0.30373672	-1.723237	0.08484568	0.83609956
Phtf2	9.13314256	0.66092898	0.38367251	1.72263835	0.08495394	0.83609956
Ift172	21.2524422	0.62759871	0.36432591	1.7226299	0.08495548	0.83609956
Gpt2	25.465523	-0.5459395	0.31710761	-1.7216221	0.085138	0.83712508
Fam76b	5.56864761	0.69073135	0.40147639	1.72047813	0.08534557	0.83839468
Sox10	25.8201097	-0.6930296	0.40311111	-1.7192023	0.08557753	0.83916908
Ankrd46	136.387017	0.36453625	0.21206315	1.71899853	0.08561464	0.83916908
Thrb	75.2208323	0.4630891	0.26947611	1.71847925	0.08570924	0.83916908
Rgs9	28.3558422	-0.5871642	0.3417085	-1.7183189	0.08573846	0.83916908
Hspa1a	11.2226191	0.70305735	0.40938431	1.71735294	0.0859147	0.83953126
Pgam1	140.985616	0.42106201	0.24519478	1.71725516	0.08593256	0.83953126
Lhx8	3.2413119	-0.7078909	0.41222592	-1.7172402	0.0859353	NA
H2-Q1	0.95857256	-0.5846065	0.34074392	-1.7156771	0.08622116	NA
Trp53i11	133.189159	-0.5492259	0.32025935	-1.714941	0.08635605	0.84239987
Map1b	1500.69597	0.48968562	0.28568263	1.71408957	0.08651228	0.84239987
Sat1	7.24989766	-0.6702521	0.39105615	-1.7139535	0.08653726	0.84239987
Gal3st3	58.184641	-0.5351442	0.31223209	-1.7139307	0.08654145	0.84239987
Prpf38b	33.9812127	0.67041536	0.39151052	1.71238146	0.08682639	0.843645
Trub1	15.7303761	0.59505455	0.34750209	1.71237688	0.08682724	0.843645
Atp6v0e	11.3852556	-0.6692508	0.39126479	-1.7104806	0.08717704	0.84627452
Kiss1r	4.13221236	0.71104522	0.41580917	1.71002774	0.08726074	NA
Cldn1	1.31798889	0.62826554	0.36757761	1.70920515	0.08741296	NA
Ptbp1	9.29808705	-0.6566419	0.38442362	-1.7081207	0.08761395	0.84974404
Lats2	32.5838388	0.49783678	0.29157836	1.70738591	0.08775035	0.85007785
Fus	49.4560715	0.59976477	0.35133987	1.70707861	0.08780744	0.85007785
Syndig1l	20.020484	-0.6485659	0.38003628	-1.7065895	0.08789837	0.85018803
Brpf1	18.1923849	0.58135504	0.3410636	1.70453557	0.08828108	0.85097693
Gtf2f1	24.5420224	0.50591519	0.29683085	1.70438885	0.08830847	0.85097693
Dio2	21.8267202	-0.7028011	0.41235859	-1.7043446	0.08831672	0.85097693
Tceal3	173.261204	0.69025564	0.40504172	1.70415937	0.08835132	0.85097693
Xpo4	5.76571905	0.69627531	0.40860835	1.70401636	0.08837803	0.85097693
Pgam2	2.52967705	0.70185264	0.41190683	1.70391117	0.08839768	NA
Sema3g	1.89492951	0.57894726	0.33978878	1.70384453	0.08841014	NA
2410004N09Rik	1.57267229	0.6774075	0.39764899	1.7035313	0.08846869	NA
Ralgds	63.0821253	-0.4989884	0.29302645	-1.7028784	0.08859083	0.85225813
Diablo	12.6205925	-0.6497387	0.38168848	-1.7022749	0.08870385	0.85233511
Dhx8	31.2069755	0.47373575	0.27834861	1.70195119	0.08876452	0.85233511
Emc3	25.5066935	-0.6394262	0.37590267	-1.701042	0.0889351	0.85233511
Pxn	8.76893419	-0.697297	0.40995361	-1.700917	0.08895858	0.85233511
Npffr1	3.7199574	0.69820114	0.41052793	1.70073968	0.08899188	NA
Ano4	12.3087641	0.65497613	0.38511937	1.70070942	0.08899757	0.85233511
Cwc22	3.549601	0.70787845	0.41630371	1.70038947	0.08905769	NA
Pold2	12.6175965	-0.6724599	0.39553785	-1.7001152	0.08910926	0.85264079
Prrx1	6.08368782	-0.6889705	0.40541785	-1.6994084	0.08924225	0.85314955

GeneID	Normalised expression for Chow#1	Normalised expression for Chow#2	Normalised expression for Chow#3	Normalised expression for Chow#4	Normalised expression for HFD#1	Normalised expression for HFD#2	Normalised expression for HFD#3	Normalised expression for HFD#4
Jagn1	10.0806949	23.9837571	16.5401098	39.2946602	9.98887032	5.88027262	11.5471667	18.2610202
Ankrd34b	43.6830113	33.9392789	39.1237212	31.0221002	24.9721758	63.5069443	76.9811111	68.4788259
Rnase4	1.34409266	4.97776091	4.13502745	2.06814001	1.66481172	1.17605452	0	0
Ltv1	12.0968339	12.6706641	9.86045007	6.20442003	3.32962344	7.05632714	5.77358333	4.56525506
Zrsr1	77.9573741	65.1634155	83.6547861	62.0442003	69.9220923	109.373071	104.886764	123.261887
Ints1	45.6991503	28.0564706	34.0344567	84.7937404	166.481172	44.6900719	46.1886666	98.1529838
Hps6	1.34409266	4.07271347	4.13502745	10.3407001	14.9833055	4.7042181	5.77358333	18.2610202
Snx29	5.37637063	1.35757116	1.90847421	0	8.3240586	3.52816357	2.88679167	13.6957652
Slc41a1	31.5861774	74.6664136	52.8011197	33.0902402	36.6258578	36.4576902	21.1698055	25.1089028
Ccdc55	16.1291119	6.78785578	7.31581779	6.20442003	4.99443516	23.5210905	25.0188611	18.2610202
Celsr3	23.5216215	28.961518	26.7186389	45.4990802	64.9276571	61.1548353	26.9433889	41.0872956
9530052E02Ri	0.67204633	0	0	0	3.32962344	1.17605452	0	4.56525506
Phf20	51.075521	25.3413283	48.6660923	45.4990802	58.2684102	59.9787807	62.5471528	70.7614535
Znf512b	24.1936678	21.2686148	27.990955	26.8858201	38.2906696	41.1619083	33.6792361	31.9567854
Mafg	17.4732045	9.95552182	13.9954775	33.0902402	43.2851047	19.9929269	24.0565972	36.5220405
Eva1a	2.01613899	4.97776091	4.13502745	8.27256004	1.66481172	1.17605452	0.96226389	2.28262753
Cobl	110.887644	86.884554	90.9706039	35.1583802	58.2684102	62.3308898	51.96225	25.1089028
Srcin1	167.339536	162.456015	170.172283	357.788222	694.226487	211.689814	169.358444	467.938644
Exosc7	4.03227797	5.88280835	3.81694841	10.3407001	9.98887032	5.88027262	12.5094306	15.9783927
Atrip	4.03227797	7.69290322	9.224292	10.3407001	24.9721758	5.88027262	11.5471667	20.5436478
Isoc2b	4.03227797	1.81009488	2.86271131	6.20442003	1.66481172	0	0	2.28262753
Rps6kc1	32.9302701	31.6766603	26.0824808	33.0902402	64.9276571	44.6900719	39.4528194	31.9567854
Basp1	377.01799	306.358558	365.79089	1433.22103	1265.25691	250.499614	499.414958	4332.42705
Smim11	6.72046328	8.14542694	6.36158069	0	0	4.7042181	0.96226389	0
Pradc1	8.73660227	4.07271347	5.08926455	10.3407001	1.66481172	3.52816357	3.84905555	0
Rtkn2	0	0.90504744	0.9542371	0	0	3.52816357	4.81131944	2.28262753
Vwa5a	13.4409266	12.6706641	9.54237104	8.27256004	4.99443516	9.40843619	4.81131944	2.28262753
Plch2	32.9302701	33.0342315	29.8994292	37.2265202	79.9109626	37.6337448	28.8679167	68.4788259
Amer3	20.8334362	14.0282353	16.5401098	33.0902402	36.6258578	23.5210905	32.7169722	34.239413
Tcf7l1	4.7043243	8.59795066	5.40734359	10.3407001	6.65924688	2.35210905	1.92452778	0
Rnft1	6.04841696	12.2181404	12.4050823	2.06814001	0	7.05632714	0.96226389	4.56525506
Huwe1	266.130346	344.823074	309.490901	281.267041	542.728621	633.893388	314.660292	278.480559
Tbc1d9b	108.199459	106.795598	93.5152361	128.224681	228.079206	131.718107	103.9245	166.63181
Etv4	8.73660227	4.52523719	1.90847421	6.20442003	4.99443516	0	0.96226389	0
Gm20594	1.34409266	0.45252372	0.63615807	4.13628002	14.9833055	1.17605452	4.81131944	0
Polr1b	10.7527413	7.2403795	7.31581779	6.20442003	16.6481172	9.40843619	11.5471667	22.8262753
Reep6	15.4570656	23.9837571	15.9039517	4.13628002	9.98887032	9.40843619	3.84905555	9.13051012
Fam195a	7.39250961	5.88280835	5.08926455	10.3407001	3.32962344	2.35210905	1.92452778	4.56525506
Crocc	1.34409266	5.88280835	4.13502745	22.7495401	26.6369875	10.5844907	8.660375	31.9567854
Cuedc2	36.9625481	31.2241366	36.579089	37.2265202	49.9443516	39.9858538	47.1509305	68.4788259
Gm2506	2.01613899	0.45252372	1.27231614	2.06814001	3.32962344	3.52816357	2.88679167	6.84788259
Tjp2	18.8172972	22.6261859	13.3593194	18.6132601	18.3129289	4.7042181	10.5849028	6.84788259
Zkscan14	3.36023164	3.62018975	4.45310648	16.5451201	1.66481172	2.35210905	1.92452778	2.28262753
Prkd1	8.73660227	7.69290322	6.99773876	6.20442003	3.32962344	5.88027262	2.88679167	0
Mtor	72.5810035	56.5654649	72.2039408	70.3167603	151.497867	95.2604165	46.1886666	143.805534
BC020402	3.36023164	3.62018975	2.54463228	6.20442003	0	2.35210905	0.96226389	0
Pcdhb22	4.7043243	5.43028463	1.59039517	6.20442003	14.9833055	7.05632714	7.69811111	6.84788259
Derl3	0	6.33533207	1.90847421	4.13628002	0	0	0.96226389	0
Rbfox2	200.941852	228.977002	208.341768	324.697982	397.890001	299.893904	249.226347	381.198798
Set	96.7746713	71.4987476	92.24292	57.9079203	88.2350212	107.020962	140.490528	116.414004
Lonp1	20.1613899	19.9110436	19.7209001	49.6353602	44.9499164	31.7534722	35.6037639	59.3483158
Tssc1	22.1775288	11.313093	15.5858727	16.5451201	34.9610461	23.5210905	26.9433889	18.2610202
Nt5m	20.8334362	14.0282353	24.8101647	26.8858201	33.2962344	27.0492541	26.9433889	50.2178057
Pkig	49.7314283	26.6988994	34.0344567	8.27256004	18.3129289	15.2887088	21.1698055	11.4131377
Znrf3	19.4893435	26.6988994	13.6773985	12.4088401	4.99443516	16.4647633	11.5471667	0
Usp54	51.7475673	65.1634155	46.12146	39.2946602	41.620293	37.6337448	34.6415	25.1089028
Rasl10a	26.2098068	26.6988994	20.9932163	12.4088401	11.653682	8.23238167	8.660375	25.1089028
Gan	5.37637063	4.07271347	1.59039517	2.06814001	13.3184938	8.23238167	6.73584722	2.28262753
Plcl2	49.7314283	36.6544212	47.0756971	51.7035003	61.5980337	77.6195986	70.2452639	45.6525506
Arntl	22.1775288	21.2686148	23.5378486	12.4088401	9.98887032	14.1126543	16.3584861	4.56525506
Cdc34	9.4086486	8.14542694	14.6316356	20.6814001	4.99443516	7.05632714	6.73584722	6.84788259
Vstm2a	80.6455594	124.444023	124.686982	43.4309402	79.9109626	344.583976	178.981083	91.3051012
Hs3st3b1	2.01613899	1.81009488	1.27231614	2.06814001	0	0	0	0
Ubr4	122.984478	123.538975	118.961559	210.950281	452.828788	198.753215	139.528264	159.783927
0610007P14Ri	21.5054825	37.106945	38.1694841	20.6814001	8.3240586	16.4647633	15.3962222	31.9567854
Elovl5	22.1775288	72.8563187	43.5768277	8.27256004	18.3129289	28.2253086	17.32075	4.56525506
Bmpr1b	9.4086486	22.6261859	14.3135566	4.13628002	1.66481172	2.35210905	13.4716944	0
Ssr4	16.1291119	21.2686148	13.0412404	26.8858201	6.65924688	7.05632714	14.4339583	13.6957652
Gsg1l	32.9302701	59.2806072	52.8011197	28.9539601	29.966611	30.5774176	29.8301805	25.1089028
Phtf2	4.03227797	9.05047438	7.95197586	2.06814001	8.3240586	14.1126543	11.5471667	15.9783927
Ift172	20.8334362	9.95552182	16.8581888	12.4088401	51.6091633	24.697145	15.3962222	18.2610202
Gpt2	37.6345944	37.5594687	28.9451921	18.6132601	14.9833055	16.4647633	22.1320694	27.3915304
Fam76b	4.03227797	4.52523719	3.18079035	2.06814001	3.32962344	10.5844907	7.69811111	9.13051012
Sox10	34.9464091	35.2968501	26.0824808	43.4309402	29.966611	2.35210905	4.81131944	29.6741579
Ankrd46	106.18332	108.153169	114.508452	144.769801	149.833055	149.358925	135.679208	182.610202
Thrb	61.8282622	74.6664136	65.8423601	43.4309402	71.586904	115.253343	102.962236	66.1961984
Rgs9	51.075521	21.7211385	33.0802196	35.1583802	16.6481172	24.697145	12.5094306	31.9567854
Hspa1a	2.68818531	7.69290322	6.67965972	10.3407001	34.9610461	4.7042181	6.73584722	15.9783927
Pgam1	122.984478	83.2643643	129.140088	138.565381	154.82749	139.950488	149.150903	210.001733
Lhx8	2.01613899	6.78785578	8.2700549	2.06814001	3.32962344	1.17605452	0	2.28262753
H2-Q1	1.34409266	3.62018975	0.63615807	2.06814001	0	0	0	0
Trp53i11	125.000617	214.043719	137.092064	175.791901	151.497867	56.4506172	66.3962083	139.240279
Map1b	1219.09204	1324.0844	1331.16076	963.753245	1704.7672	2873.1012	1521.33921	1068.26968
Sat1	8.73660227	11.7656167	7.95197586	10.3407001	1.66481172	5.88027262	4.81131944	6.84788259
Gal3st3	63.1723549	97.7451233	69.3412295	49.6353602	58.2684102	36.4576902	26.9433889	63.9135709
Prpf38b	10.0806949	15.3858064	15.5858727	51.7035003	43.2851047	37.6337448	18.2830139	79.8919636
Trub1	12.7688802	12.6706641	9.86045007	12.4088401	11.653682	16.4647633	31.7547083	18.2610202
Atp6v0e	7.39250961	18.1009488	15.5858727	18.6132601	3.32962344	8.23238167	3.84905555	15.9783927
Kiss1r	2.01613899	2.26261859	1.59039517	2.06814001	8.3240586	1.17605452	1.92452778	13.6957652
Cldn1	0	1.35757116	0	0	1.66481172	2.35210905	2.88679167	2.28262753
Ptbp1	14.7850192	12.6706641	7.63389683	14.4769801	9.98887032	3.52816357	6.73584722	4.56525506
Lats2	24.1936678	22.6261859	25.4463228	35.1583802	38.2906696	52.9224536	34.6415	27.3915304
Fus	36.9625481	17.1959013	26.0824808	68.2486203	68.2572805	41.1619083	50.9999861	86.7398462
Syndig1l	39.6507334	16.2908539	24.1740066	22.7495401	21.6425524	7.05632714	5.77358333	22.8262753
Brpf1	13.4409266	16.2908539	14.6316356	8.27256004	38.2906696	19.9929269	16.3584861	18.2610202
Gtf2f1	25.5377605	18.5534725	16.8581888	16.5451201	34.9610461	28.2253086	25.981125	29.6741579
Dio2	36.9625481	45.2523719	36.897168	4.13628002	9.98887032	21.1689814	20.2075417	0
Tceal3	60.4841696	47.9675142	62.6615698	266.790061	186.458913	104.668853	118.358458	538.700097
Xpo4	3.36023164	4.07271347	5.72542262	0	11.653682	9.40843619	9.62263889	2.28262753
Pgam2	0.67204633	1.81009488	1.59039517	0	3.32962344	1.17605452	4.81131944	6.84788259
Sema3g	0	0.90504744	0.31807903	0	11.653682	0	0	2.28262753
2410004N09R	1.34409266	0.45252372	0.63615807	0	1.66481172	2.35210905	3.84905555	2.28262753
Ralgds	65.8605402	76.0239848	63.2977279	97.2025805	73.2517157	30.5774176	41.3773472	57.0656883
Diablo	13.4409266	12.2181404	11.7689243	31.0221002	4.99443516	5.88027262	12.5094306	9.13051012
Dhx8	24.1936678	25.3413283	26.7186389	24.8176801	38.2906696	45.8661264	27.9056528	36.5220405
Emc3	30.9141311	47.0624668	40.3960374	14.4769801	18.3129289	32.9295267	15.3962222	4.56525506
Pxn	8.06455594	14.480759	5.40734359	20.6814001	8.3240586	1.17605452	2.88679167	9.13051012
Npffr1	2.68818531	1.81009488	1.59039517	0	11.653682	0	2.88679167	9.13051012
Ano4	9.4086486	12.2181404	10.1785291	0	9.98887032	24.697145	18.2830139	13.6957652
Cwc22	2.68818531	2.26261859	2.54463228	0	3.32962344	4.7042181	10.5849028	2.28262753
Pold2	15.4570656	10.8605693	8.90621297	33.0902402	4.99443516	5.88027262	5.77358333	15.9783927
Prrx1	7.39250961	8.59795066	6.04350166	12.4088401	4.99443516	1.17605452	5.77358333	2.28262753

GeneID	Base mean	log2(FC)	StdErr	Wald-Stats	P-value	P-adj	GeneID	Normalised expression for Chow#1	Normalised expression for Chow#2	Normalised expression for Chow#3	Normalised expression for Chow#4	Normalised expression for HFD#1	Normalised expression for HFD#2	Normalised expression for HFD#3	Normalised expression for HFD#4
S1pr5	3.23363495	-0.7056247	0.41530964	-1.6990328	0.089313	NA	S1pr5	3.36023164	6.33533207	3.18079035	6.20442003	3.32962344	1.17605452	0	2.28262753
Prex1	108.721751	-0.5629434	0.33145101	-1.6984212	0.08942829	0.85341438	Prex1	112.231737	139.829829	94.7875523	192.337021	148.168243	45.8661264	63.5094166	73.044081
4933412E12Rik	1.5537951	-0.6438937	0.37915733	-1.698223	0.08946568	NA	4933412E12Ri	4.7043243	2.26261859	3.18079035	0	0	0	0	2.28262753
Anp32e	74.1698831	0.4217433	0.2483629	1.69809301	0.08949021	0.85341438	Anp32e	57.7959842	45.7048956	65.5242811	82.7256004	79.9109626	81.1477622	84.6792222	95.8703563
Baz2b	19.6514407	0.58264199	0.34320043	1.69767267	0.08956956	0.85341438	Baz2b	13.4409266	22.6261859	14.9497146	6.20442003	21.6425524	34.1055812	25.981125	18.2610202
Tmem254b	22.2002064	-0.5982921	0.35252493	-1.6971626	0.08966592	0.85341438	Tmem254b	23.5216215	39.3695635	33.0802196	16.5451201	11.653682	14.1126543	27.9056528	11.4131377
Tbc1d8	17.9529158	-0.5768416	0.33988932	-1.6971453	0.08966919	0.85341438	Tbc1d8	22.1775288	14.9332827	25.1282437	26.8858201	18.3129289	10.5844907	9.62263889	15.9783927
Trim25	4.04370257	-0.7063983	0.4163037	-1.696834	0.08972804	NA	Trim25	4.7043243	7.69290322	3.18079035	8.27256004	3.32962344	0	2.88679167	2.28262753
Zfp871	122.303658	0.3602937	0.21236506	1.6965771	0.08977664	0.85367683	Zfp871	88.7101153	103.627932	121.188112	111.679561	136.514561	155.239197	140.490528	120.979259
Zfp523	28.8576508	0.54007527	0.31884866	1.6938295	0.09029769	0.85784904	Zfp523	26.2098068	15.3858064	18.130505	31.0221002	43.2851047	29.4013631	24.0565972	43.3699231
Camkk1	43.5291706	0.54008766	0.31893511	1.69340921	0.0903776	0.85784904	Camkk1	34.2743627	23.0787097	29.5813502	49.6353602	76.5813391	38.8097993	34.6415	61.6309433
Whrn	73.4668745	0.6471958	0.38255344	1.6917788	0.09068816	0.85784904	Whrn	36.9625481	33.0342315	34.6706148	99.2707205	119.866444	52.9224536	37.5282917	173.479692
Tubb2a	308.500833	0.48909881	0.28915316	1.69148697	0.09074383	0.85784904	Tubb2a	213.710732	178.294345	239.831592	363.992642	484.460211	263.436213	272.32068	451.960251
Syt5	70.6344422	-0.479206	0.28335602	-1.69118	0.09080242	0.85784904	Syt5	83.3337447	116.298596	69.3412295	64.1123403	58.2684102	63.5069443	39.4528194	70.7614535
4933421O10Rik	7.18718455	0.65383486	0.38665424	1.69100654	0.09083555	0.85784904	4933421O10R	6.72046328	3.16766603	5.40734359	4.13628002	6.65924688	9.40843619	10.5849028	11.4131377
Tcf20	141.425243	0.51437741	0.30420138	1.69091083	0.09085383	0.85784904	Tcf20	137.097451	127.159165	118.325401	64.1123403	143.173808	231.682741	211.698055	98.1529838
Sars	91.393271	0.47403388	0.28038219	1.69067045	0.09089976	0.85784904	Sars	88.038069	44.7998482	62.9796488	105.475141	93.2294563	107.020962	115.471667	114.131377
Ascc2	5.06685158	-0.6967814	0.41218167	-1.6904715	0.09093778	0.85784904	Ascc2	6.72046328	5.88280835	8.90621297	8.27256004	1.66481172	2.35210905	6.73584722	0
Mfhas1	59.8576823	0.44518344	0.26349642	1.68952364	0.09111912	0.85850438	Mfhas1	43.010965	48.8725616	49.9384084	55.8397803	94.8942681	59.9787807	50.9999861	75.3267085
Myo6	38.6926711	-0.6194376	0.366854	-1.6885125	0.09131289	0.85850438	Myo6	57.7959842	56.5654649	50.8926455	33.0902402	58.2684102	25.8731995	20.2075417	6.84788259
BC048546	14.0542902	-0.6305927	0.37364353	-1.6876854	0.09147163	0.85850438	BC048546	18.1452509	23.5312334	24.4920857	6.20442003	11.653682	9.40843619	14.4339583	4.56525506
Rgl1	94.1975425	-0.4687677	0.27777141	-1.6876024	0.09148757	0.85850438	Rgl1	110.215598	150.237875	119.279638	64.1123403	89.8998329	67.0351079	86.60375	66.1961984
Crmp1	143.53785	0.41372233	0.24521647	1.68717188	0.09157031	0.85850438	Crmp1	124.328571	106.795598	123.096586	128.224681	218.090335	139.950488	118.358458	189.458085
Tmem205	5.37027482	-0.6892041	0.40850189	-1.6871503	0.09157446	0.85850438	Tmem205	4.7043243	6.33533207	6.99773876	12.4088401	4.99443516	2.35210905	2.88679167	2.28262753
Cartpt	22.413146	0.59801874	0.35452236	1.6868294	0.09163616	0.85850438	Cartpt	11.4247876	15.8383302	11.1327662	28.9539601	26.6369875	22.345036	17.32075	45.6525506
Pdgfa	19.8477222	0.51824164	0.3072412	1.68675828	0.09164984	0.85850438	Pdgfa	14.1129729	13.5757116	16.5401098	18.6132601	26.6369875	18.8168724	23.0943333	27.3915304
Rn4.5s	4.38989594	0.70082703	0.41551668	1.68663994	0.09167261	NA	Rn4.5s	2.68818531	0.90504744	1.90847421	6.20442003	6.65924688	3.52816357	8.660375	4.56525506
Morn2	5.64259491	-0.6836455	0.40545184	-1.6861324	0.09177031	0.85888007	Morn2	11.4247876	5.88280835	7.95197586	6.20442003	1.66481172	5.88027262	3.84905555	2.28262753
AW011738	2.54271614	-0.692303	0.41061985	-1.685995	0.09179677	NA	AW011738	2.68818531	2.71514231	2.86271131	8.27256004	1.66481172	1.17605452	0.96226389	0
Med27	9.82173894	-0.6244985	0.37062278	-1.6849975	0.09198905	0.85944135	Med27	13.4409266	9.05047438	15.2677937	12.4088401	6.65924688	5.88027262	6.73584722	9.13051012
Id2	59.6889368	-0.6289082	0.3732422	-1.6849869	0.0919911	0.85944135	Id2	90.054208	113.583453	78.2474425	26.8858201	23.3073641	29.4013631	81.7924305	34.239413
Mfsd8	4.53953402	-0.7003929	0.41568911	-1.6848959	0.09200866	NA	Mfsd8	6.04841696	9.5029981	8.90621297	2.06814001	1.66481172	2.35210905	5.77358333	0
Psmb8	0.93256557	0.58923496	0.34996663	1.68368896	0.0922418	NA	Psmb8	0	0.45252372	0.31807903	0	1.66481172	1.17605452	3.84905555	0
Gpatch2l	29.052592	0.54410315	0.32316805	1.68365391	0.09224857	0.86103908	Gpatch2l	23.5216215	23.9837571	21.6293743	18.6132601	54.9387868	29.4013631	19.2452778	41.0872956
Hes7	2.20175771	0.65422153	0.38860852	1.68349763	0.0922788	NA	Hes7	0.67204633	1.81009488	0.31807903	0	8.3240586	0	1.92452778	4.56525506
Tspan13	96.1428078	-0.5575803	0.33124868	-1.6832679	0.09232324	0.86103908	Tspan13	102.151042	187.34482	131.048562	53.7716403	83.240586	97.6125255	68.3207361	45.6525506
Gab2	26.3444748	0.53198924	0.31619722	1.68246024	0.09247963	0.86174566	Gab2	23.5216215	23.9837571	20.9932163	12.4088401	34.9610461	47.042181	25.0188611	22.8262753
Asb3	15.6122908	0.56899814	0.33833505	1.68175936	0.09261551	0.8622601	Asb3	14.1129729	10.8605693	12.7231614	10.3407001	14.9833055	23.5210905	26.9433889	11.4131377
Dtnbp1	16.8898148	-0.5838245	0.3474184	-1.6804652	0.09286685	0.8626417	Dtnbp1	27.5538995	22.6261859	24.1740066	10.3407001	13.3184938	12.9365998	17.32075	6.84788259
BC064078	0.8865349	-0.559247	0.33279721	-1.6804439	0.09287098	NA	BC064078	2.01613899	3.16766603	1.90847421	0	0	0	0	0
Shank3	169.578878	0.65476693	0.38978391	1.67982032	0.09299228	0.8626417	Shank3	88.7101153	86.884554	77.2932054	206.814001	419.532554	116.429398	59.6603611	301.306834
Grik1	27.4010447	0.55874845	0.33271237	1.67937387	0.0930792	0.8626417	Grik1	12.7688802	23.0787097	23.8559276	24.8176801	19.9777406	52.9224536	29.8301805	31.9567854
Slc7a3	10.0156168	0.67042794	0.39923099	1.67929833	0.09309392	0.8626417	Slc7a3	2.68818531	8.14542694	5.72542262	10.3407001	21.6425524	17.6408179	4.81131944	9.13051012
Pmepa1	26.5086749	-0.5460489	0.32527182	-1.6787462	0.09320151	0.8626417	Pmepa1	35.6184554	42.5372296	30.8536663	18.6132601	23.3073641	23.5210905	12.5094306	25.1089028
Usp4	50.6279415	0.45421461	0.27058742	1.67862426	0.0932253	0.8626417	Usp4	54.4357526	35.2968501	42.9406697	33.0902402	73.2517157	49.39429	66.3962083	50.2178057
Olfml1	2.56522073	-0.6873041	0.40946833	-1.6785281	0.09324406	NA	Olfml1	5.37637063	6.78785578	2.22655324	2.06814001	0	1.17605452	2.88679167	0
Metrn	13.9126074	-0.6969656	0.41530926	-1.6781847	0.09331105	0.8626417	Metrn	14.7850192	14.0282353	7.31581779	43.4309402	9.98887032	0	5.77358333	15.9783927
Fat4	28.3695278	0.60115756	0.35823647	1.67810259	0.09332707	0.8626417	Fat4	20.1613899	28.961518	21.6293743	10.3407001	61.5980337	43.5140174	20.2075417	20.5436478
Pgap2	7.50083276	-0.6663005	0.39734132	-1.676897	0.09356263	0.8626417	Pgap2	6.72046328	12.2181404	9.224292	12.4088401	9.98887032	2.35210905	4.81131944	2.28262753
Pgs1	41.6128717	0.51532256	0.30731009	1.67688136	0.09356568	0.8626417	Pgs1	29.5700384	29.4140417	30.8536663	41.3628002	63.2628454	28.2253086	39.4528194	70.7614535
Rnf122	2.16383839	-0.6844003	0.40815141	-1.6768295	0.09357582	NA	Rnf122	3.36023164	5.43028463	2.86271131	2.06814001	1.66481172	0	1.92452778	0
Psenen	15.9877148	-0.586087	0.34962407	-1.6763347	0.09367264	0.8626417	Psenen	20.8334362	21.2686148	16.8581888	20.6814001	18.3129289	4.7042181	11.5471667	13.6957652
Maob	8.55380088	-0.6492153	0.38729741	-1.6762706	0.09368521	0.8626417	Maob	16.8011582	12.6706641	7.95197586	8.27256004	6.65924688	7.05632714	6.73584722	2.28262753
Tspyl4	520.30516	0.34764317	0.20740382	1.67616567	0.09370575	0.8626417	Tspyl4	433.469882	337.582694	484.43437	562.534083	576.024855	570.386444	590.830028	607.178923
Celf4	485.226599	0.43672253	0.2607452	1.67490149	0.09395356	0.86375687	Celf4	353.496369	383.28759	344.479594	533.580123	627.634019	408.09092	455.150819	776.093361
Ulk3	17.8958224	0.64009799	0.38223263	1.67462937	0.09400697	0.86375687	Ulk3	6.72046328	9.5029981	11.4508452	22.7495401	26.6369875	10.5844907	14.4339583	41.0872956
Gm10069	2.24101328	-0.6735098	0.40219657	-1.6745787	0.09401693	NA	Gm10069	5.37637063	3.62018975	1.90847421	4.13628002	0	0	2.88679167	0
Ntm	117.621028	0.57359461	0.34264037	1.67404271	0.09412221	0.86375687	Ntm	72.5810035	114.035977	76.6570473	88.9300204	124.860879	184.64056	50.9999861	228.262753
Madd	160.232976	0.47133568	0.28157926	1.67390055	0.09415015	0.86375687	Madd	131.72108	104.532979	112.918057	173.723761	259.710628	148.18287	125.094306	225.980126
Klhl5	25.2520187	-0.5170812	0.30907686	-1.672986	0.09433006	0.86466521	Klhl5	35.6184554	32.129184	29.5813502	24.8176801	29.966611	21.1689814	17.32075	11.4131377
Gls	194.100494	0.35354053	0.2115215	1.67141654	0.09463943	0.86571936	Gls	199.59776	141.639924	190.529342	140.633521	204.771842	236.386959	215.547111	223.697498
Casp7	1.80773205	-0.6556969	0.3923466	-1.6712186	0.09467851	NA	Casp7	6.72046328	2.26261859	1.27231614	2.06814001	0	1.17605452	0.96226389	0
Gng3	280.284785	-0.4420266	0.26450731	-1.6711321	0.0946956	0.86571936	Gng3	350.808183	304.095939	285.316894	372.265202	194.782971	143.478652	274.245208	317.285227
Cat	30.6062824	-0.493083	0.29508626	-1.6709792	0.09472578	0.86571936	Cat	36.9625481	39.8220873	38.1694841	31.0221002	29.966611	15.2887088	30.7924444	22.8262753
Ilf3	45.4219913	0.45482507	0.27243495	1.66948139	0.09502202	0.86571936	Ilf3	40.994826	30.7716129	33.3982986	45.4990802	61.5980337	58.8027262	37.5282917	54.7830607
Gna12	29.9516039	-0.5334685	0.31955309	-1.6694206	0.09503406	0.86571936	Gna12	36.2905017	43.8948007	26.7186389	39.2946602	36.6258578	14.1126543	22.1320694	20.5436478
Akap5	130.097247	0.54240054	0.32492134	1.66932876	0.09505224	0.86571936	Akap5	133.065173	96.8400759	117.371164	53.7716403	98.2238915	248.147505	183.792403	109.566121
Cnn3	18.7076675	-0.5933859	0.35551358	-1.6690949	0.09509858	0.86571936	Cnn3	31.5861774	20.3635674	24.4920857	16.5451201	18.3129289	4.7042181	15.3962222	18.2610202
Bambi	2.07448658	-0.6672088	0.39975289	-1.6690532	0.09510684	NA	Bambi	1.34409266	5.43028463	2.22655324	4.13628002	0	1.17605452	0	2.28262753
Chrdl1	3.52011029	-0.6803868	0.40766884	-1.6689694	0.09512345	NA	Chrdl1	5.37637063	8.14542694	8.2700549	0	1.66481172	4.7042181	0	0
Lck	2.24147378	0.67943584	0.40710745	1.66893491	0.09513028	NA	Lck	0.67204633	0.90504744	0.9542371	2.06814001	0	5.88027262	2.88679167	4.56525506
Ddx17	131.697945	0.37469877	0.22456418	1.66855984	0.09520464	0.86571936	Ddx17	97.4467176	93.6724098	113.236136	153.042361	154.82749	143.478652	147.226375	150.653417
Setd6	4.87758813	-0.694223	0.41618613	-1.6680589	0.09530403	0.86571936	Setd6	8.06455594	3.62018975	3.81694841	14.4769801	1.66481172	3.52816357	3.84905555	0
Lfng	9.381509	-0.6834315	0.40974234	-1.6679543	0.0953248	0.86571936	Lfng	4.03227797	20.3635674	11.1327662	16.5451201	13.3184938	3.52816357	3.84905555	2.28262753
Sun2	17.6624878	-0.5437213	0.32599201	-1.6678976	0.09533605	0.86571936	Sun2	20.8334362	23.9837571	20.6751372	20.6814001	19.9777406	14.1126543	9.62263889	11.4131377
Gnpat	39.7155846	-0.4448015	0.26699226	-1.6659716	0.09571907	0.86640791	Gnpat	43.010965	43.8948007	53.4372778	45.4990802	24.9721758	30.5774176	37.5282917	38.804668
Mapkapk2	17.2531276	-0.5936975	0.35637255	-1.6659463	0.09572412	0.86640791	Mapkapk2	20.8334362	21.7211385	20.0389792	24.8176801	23.3073641	4.7042181	13.4716944	9.13051012
Ankrd24	18.8604888	0.63112765	0.37895265	1.66545253	0.09582251	0.86640791	Ankrd24	14.7850192	5.88280835	9.224292	24.8176801	29.966611	11.7605452	20.2075417	34.239413
Cav1	4.33427745	-0.6915126	0.41535118	-1.6648865	0.09593539	NA	Cav1	5.37637063	9.95552182	7.31581779	2.06814001	3.32962344	4.7042181	1.92452778	0
Ubr3	220.557104	0.45905758	0.27575526	1.66472827	0.09596698	0.86640791	Ubr3	194.893435	210.876053	209.932163	105.475141	201.442218	338.703703	295.415014	207.719105
Taok2	115.51151	0.49260867	0.29594761	1.66451307	0.09600994	0.86640791	Taok2	95.4305786	69.6886527	72.8400989	134.429101	159.821925	122.309671	84.6792222	184.89283
Actr3	72.2980247	0.40304016	0.24217882	1.66422549	0.09606738	0.86640791	Actr3	61.1562159	56.1129411	62.9796488	64.1123403	79.9109626	59.9787807	91.4150694	102.718239
Sec61a1	28.5842919	-0.5667167	0.34054179	-1.6641621	0.09608005	0.86640791	Sec61a1	28.2259458	45.2523719	40.7141164	26.8858201	31.6314227	29.4013631	10.5849028	15.9783927
N4bp3	13.1375506	0.61981155	0.37245302	1.66413349	0.09608576	0.86640791	N4bp3	12.7688802	7.69290322	9.54237104	6.20442003	19.9777406	5.88027262	20.2075417	22.8262753
Mapkbp1	38.1992785	0.512879	0.30824745	1.66385481	0.09614145	0.86640791	Mapkbp1	32.2582238	32.129184	37.8514051	14.4769801	59.9332219	44.6900719	31.7547083	52.5004332
Nfic	68.9234454	-0.5465617	0.32862677	-1.6631686	0.0962787	0.86643717	Nfic	69.8928182	71.9512713	52.1649617	146.837941	68.2572805	34.1055812	44.2641389	63.9135709
Zmym1	17.3176224	0.54405784	0.32714893	1.66302802	0.09630683	0.86643717	Zmym1	16.1291119	14.0282353	11.7689243	12.4088401	14.9833055	28.2253086	25.0188611	15.9783927
Col1a2	5.19671004	-0.685776	0.41270006	-1.6616813	0.09657669	0.86813429	Col1a2	6.04841696	6.78785578	7.31581779	8.27256004	6.65924688	0	1.92452778	4.56525506
Ptpn20	0.86222508	-0.571393	0.34402245	-1.6609177	0.09672997	NA	Ptpn20	2.01613899	0.90504744	1.90847421	2.06814001	0	0	0	0
Dst	137.573276	0.54946228	0.33095171	1.66024912	0.09686435	0.8695285	Dst	118.280154	113.583453	105.920318	84.7937404	179.799666	297.541795	129.905625	70.7614535
Baz1b	64.2359104	0.41157041	0.24791917	1.66009917	0.0968945	0.8695285	Baz1b	61.1562159	50.2301328	54.0734359	51.7035003	83.240586	92.9083074	63.5094166	57.0656883
Wdr60	8.41731465	0.66591645	0.40136844	1.65911512	0.0970926	0.87046375	Wdr60	1.34409266	7.2403795	6.36158069	6.20442003	8.3240586	10.5844907	6.73584722	20.5436478
Tmem218	5.45815941	-0.6757568	0.40755846	-1.658061	0.09730515	0.87046375	Tmem218	4.7043243	9.95552182	7.31581779	8.27256004	3.32962344	5.88027262	1.92452778	2.28262753
Cmbl	5.46617074	-0.6729528	0.40587506	-1.6580296	0.0973115	0.87046375	Cmbl	5.37637063	7.69290322	9.54237104	8.27256004	3.32962344	4.7042181	4.81131944	0
Ndp	5.9715038	-0.6658914	0.40171102	-1.6576378	0.09739059	0.87046375	Ndp	6.04841696	9.5029981	9.224292	8.27256004	4.99443516	5.88027262	3.84905555	0
Tmem51	1.44884911	-0.6354178	0.38347966	-1.6569792	0.09752368	NA	Tmem51	1.34409266	2.71514231	3.18079035	2.06814001	0	0	0	2.28262753
Hnrnpdl	43.0401497	0.5486913	0.33120012	1.65667603	0.09758499	0.87046375	Hnrnpdl	41.6668724	20.3635674	34.6706148	37.2265202	26.6369875	51.7463991	81.7924305	50.2178057
Igip	24.6789377	0.63396589	0.382721	1.65647006	0.09762666	0.87046375	Igip	16.8011582	18.5534725	21.3112953	12.4088401	14.9833055	59.9787807	44.2641389	9.13051012
Lrp10	14.6107674	-0.6369971	0.38459236	-1.6562916	0.09766279	0.87046375	Lrp10	11.4247876	28.0564706	20.0389792	14.4769801	11.653682	5.88027262	4.81131944	20.5436478
Isl1	2.37423178	-0.6574317	0.39698511	-1.6560612	0.09770942	NA	Isl1	2.68818531	3.62018975	0.9542371	8.27256004	0	1.17605452	0	2.28262753
Brinp1	85.603704	-0.5786322	0.34941829	-1.6559871	0.09772443	0.87046375	Brinp1	103.495135	159.288349	120.551954	45.4990802	56.6035985	110.549125	50.0377222	38.804668
Ntmt1	9.04993795	0.67342057	0.4067457	1.65563044	0.09779668	0.87046375	Ntmt1	2.01613899	4.97776091	5.08926455	12.4088401	6.65924688	4.7042181	18.2830139	18.2610202
Stx1b	253.359172	0.50657331	0.30611126	1.6548666	0.09795156	0.87046375	Stx1b	201.613899	146.617685	169.536125	287.471461	319.64385	234.03485	188.603722	479.351782
Tmed3	12.1761697	-0.6553697	0.39603836	-1.6548137	0.0979623	0.87046375	Tmed3	9.4086486	21.2686148	9.224292	26.8858201	4.99443516	15.2887088	5.77358333	4.56525506
Tmc6	1.64841304	-0.6274796	0.37925939	-1.6544866	0.09802869	NA	Tmc6	2.68818531	1.35757116	1.27231614	6.20442003	1.66481172	0	0	0
Gnai1	130.898126	0.56589649	0.34206962	1.65433134	0.09806021	0.87046375	Gnai1	119.624246	102.722884	128.50393	43.4309402	104.883138	195.225051	256.924458	95.8703563
Galntl6	27.5483776	0.64575325	0.39044003	1.65391147	0.0981455	0.87046375	Galntl6	18.8172972	21.2686148	22.5836114	12.4088401	11.653682	77.6195986	42.3396111	13.6957652
Gcnt2	10.5546976	-0.6458001	0.39057015	-1.6534803	0.09823315	0.87046375	Gcnt2	10.7527413	19.0059962	18.448584	8.27256004	3.32962344	11.7605452	10.5849028	2.28262753
Atcay	165.206949	0.38803221	0.23473972	1.65303173	0.09832441	0.87046375	Atcay	117.608107	143.450019	150.451383	150.974221	198.112595	196.401106	131.830153	232.828008
Hist1h3i	6.42264984	-0.6595492	0.39916516	-1.6523216	0.098469	0.87046375	Hist1h3i	6.72046328	7.2403795	8.90621297	12.4088401	6.65924688	2.35210905	4.81131944	2.28262753
Tmem254c	22.4083079	-0.5774331	0.34949862	-1.6521756	0.09849875	0.87046375	Tmem254c	23.5216215	39.3695635	33.0802196	16.5451201	13.3184938	14.1126543	27.9056528	11.4131377
Lmf2	5.24917661	-0.6878262	0.41636286	-1.6519875	0.0985371	0.87046375	Lmf2	6.04841696	9.5029981	10.1785291	4.13628002	9.98887032	1.17605452	0.96226389	0

GeneID	Base mean	log2(FC)	StdErr	Wald-Stats	P-value	P-adj	GeneID	Normalised expression for Chow#1	Normalised expression for Chow#2	Normalised expression for Chow#3	Normalised expression for Chow#4	Normalised expression for HFD#1	Normalised expression for HFD#2	Normalised expression for HFD#3	Normalised expression for HFD#4
Kdm8	1.30991059	-0.6232412	0.37732222	-1.6517481	0.09858592	NA	Kdm8	1.34409266	1.81009488	2.22655324	4.13628002	0	0	0.96226389	0
Srrm3	23.1782793	0.51921156	0.31438865	1.65149586	0.09863736	0.87046375	Srrm3	20.8334362	15.3858064	16.8581888	18.6132601	26.6369875	23.5210905	20.2075417	43.3699231
Ptprk	21.7746603	-0.6225959	0.37711658	-1.6509374	0.09875136	0.87046375	Ptprk	17.4732045	49.7776091	30.2175083	14.4769801	19.9777406	23.5210905	9.62263889	9.13051012
Frmd5	30.7243707	0.48940231	0.29648198	1.65069835	0.09880018	0.87046375	Frmd5	24.1936678	21.2686148	25.1282437	31.0221002	28.3017992	49.39429	41.3773472	25.1089028
Gm13375	11.7901091	-0.6510003	0.39457127	-1.6498927	0.09896487	0.87046375	Gm13375	21.5054825	11.313093	11.1327662	18.6132601	1.66481172	3.52816357	10.5849028	15.9783927
Csrnp3	143.171534	0.47056872	0.28525752	1.64962775	0.0990191	0.87046375	Csrnp3	104.839227	104.985503	122.460428	136.497241	94.8942681	245.795396	194.377305	141.522907
Gpsm1	32.1781161	0.57439312	0.34825794	1.64933246	0.09907954	0.87046375	Gpsm1	18.8172972	15.8383302	17.1762679	47.5672202	46.6147282	32.9295267	25.981125	52.5004332
Dguok	7.26373839	-0.6820635	0.41356427	-1.6492321	0.09910009	0.87046375	Dguok	6.04841696	4.52523719	7.31581779	24.8176801	4.99443516	2.35210905	5.77358333	2.28262753
Cd53	1.19946858	-0.6031711	0.36578141	-1.6489934	0.09914899	NA	Cd53	2.01613899	4.07271347	2.54463228	0	0	0	0.96226389	0
Kcnc1	263.225305	0.53510026	0.32450424	1.64897773	0.0991522	0.87046375	Kcnc1	128.360849	204.540721	191.1655	293.675881	382.906696	341.055812	139.528264	424.568721
Rel	2.03740521	-0.6385067	0.38723541	-1.6488851	0.09917118	NA	Rel	1.34409266	3.16766603	1.59039517	8.27256004	0	0	1.92452778	0
Grpel2	10.8411305	0.58138196	0.3526495	1.64861134	0.09922728	0.87046375	Grpel2	7.39250961	7.69290322	8.2700549	8.27256004	13.3184938	12.9365998	10.5849028	18.2610202
2310011J03Rik	13.5519364	-0.6497025	0.39415122	-1.6483585	0.09927913	0.87046375	2310011J03Ri	17.4732045	5.88280835	16.8581888	33.0902402	11.653682	4.7042181	9.62263889	9.13051012
Lhx5	2.72736651	-0.5520939	0.33493603	-1.6483562	0.0992796	NA	Lhx5	0.67204633	1.35757116	0	18.6132601	0	1.17605452	0	0
Fam228a	0.80047959	0.552155	0.33519143	1.64728254	0.09949999	NA	Fam228a	0	0	0.31807903	0	1.66481172	1.17605452	0.96226389	2.28262753
Akap8	17.9738617	0.58243288	0.35366861	1.64683229	0.09959252	0.87219738	Akap8	11.4247876	6.78785578	15.5858727	20.6814001	26.6369875	14.1126543	21.1698055	27.3915304
AI504432	37.1646375	0.66245047	0.40253081	1.64571371	0.09982272	0.87219738	AI504432	26.2098068	23.5312334	34.3525357	12.4088401	19.9777406	108.197016	63.5094166	9.13051012
Cdk18	47.8075447	-0.5737862	0.34870598	-1.6454729	0.09987233	0.87219738	Cdk18	51.075521	40.274611	37.8514051	111.679561	48.2795399	25.5731995	24.0565972	43.3699231
Polr1d	26.8350196	-0.5289138	0.32147798	-1.6452567	0.09991689	0.87219738	Polr1d	25.5377605	30.3190892	33.7163777	43.4309402	9.98887032	19.9929269	28.8679167	22.8262753
Fam196b	5.98399973	-0.6832828	0.41531619	-1.6452094	0.09992664	0.87219738	Fam196b	7.39250961	2.26261859	10.1785291	14.4769801	3.32962344	4.7042181	0.96226389	4.56525506
Egfl6	1.25811877	-0.607231	0.36916952	-1.6448568	0.09999934	NA	Egfl6	3.36023164	3.62018975	1.90847421	0	0	1.17605452	0	0
LOC102308570	3.83842488	-0.6839383	0.41583178	-1.6447475	0.1000219	NA	LOC10230857	8.06455594	6.78785578	5.08926455	2.06814001	0	3.52816357	2.88679167	2.28262753
Nrros	1.89962344	-0.65254	0.39695318	-1.6438714	0.10020278	NA	Nrros	4.03227797	4.52523719	3.18079035	0	0	1.17605452	0	2.28262753
Fech	36.7715817	-0.5284575	0.32149021	-1.6437749	0.10022271	0.87219738	Fech	57.1239379	40.7271347	46.439539	37.2265202	29.966611	34.1055812	39.4528194	9.13051012
1110032A03Rik	26.7596291	-0.6237999	0.37962325	-1.6432079	0.10033992	0.87219738	1110032A03Ri	47.043243	42.9897533	36.897168	12.4088401	6.65924688	25.8731995	30.7924444	11.4131377
Stk40	29.3385441	-0.5011188	0.30499995	-1.6430126	0.10038032	0.87219738	Stk40	36.9625481	28.961518	26.4005599	51.7035003	23.3073641	24.697145	22.1320694	20.5436478
Stard3	13.4631817	-0.6588708	0.401118	-1.6425859	0.10046864	0.87219738	Stard3	19.4893435	26.2463757	14.3135566	10.3407001	13.3184938	7.05632714	0.96226389	15.9783927
Cyp4f14	2.41008841	-0.6715561	0.40884953	-1.6425507	0.10047592	NA	Cyp4f14	2.01613899	6.78785578	3.49886938	2.06814001	1.66481172	0	0.96226389	2.28262753
Gsap	1.18266299	-0.6012823	0.366098	-1.6424081	0.10050546	NA	Gsap	2.01613899	3.62018975	2.86271131	0	0	0	0.96226389	0
B4galnt1	87.0964917	-0.5089881	0.31003654	-1.6417036	0.10065146	0.87219738	B4galnt1	88.038069	140.734877	91.924841	97.2025805	93.2294563	56.4506172	35.6037639	93.5877288
Npy1r	10.5138061	-0.6539674	0.39845222	-1.6412694	0.10074151	0.87219738	Npy1r	11.4247876	23.9837571	14.6316356	6.20442003	6.65924688	14.1126543	4.81131944	2.28262753
Ndrg4	1423.44544	0.4677862	0.28513348	1.64058672	0.10088323	0.87219738	Ndrg4	1135.75829	894.639392	1110.41391	1497.33337	1989.45001	1143.125	1165.30157	2451.54197
Etnk1	257.475672	0.33598257	0.2048143	1.64042533	0.10091676	0.87219738	Etnk1	210.350501	217.211385	243.64854	233.699821	253.051381	358.69663	301.188597	241.958518
Grin2c	13.2733061	-0.668813	0.40773497	-1.640313	0.10094011	0.87219738	Grin2c	17.4732045	31.2241366	20.6751372	2.06814001	18.3129289	7.05632714	4.81131944	4.56525506
Pde5a	12.0105765	0.63999429	0.3901987	1.64017534	0.10096871	0.87219738	Pde5a	8.73660227	9.5029981	8.90621297	6.20442003	4.99443516	25.8731995	25.0188611	6.84788259
Klf12	83.4937254	0.5650296	0.34449598	1.64016311	0.10097126	0.87219738	Klf12	51.7475673	84.1694117	81.4282328	33.0902402	49.9443516	170.527906	112.584875	84.4572186
C130030K03Rik	4.96134858	0.68264282	0.41634429	1.63961133	0.10108601	0.87219738	C130030K03Ri	0.67204633	4.52523719	3.49886938	2.06814001	8.3240586	14.1126543	1.92452778	4.56525506
Wdr59	26.1423133	0.47503394	0.28978799	1.63924646	0.10116194	0.87219738	Wdr59	19.4893435	16.7433776	22.9016905	26.8858201	34.9610461	30.5774176	27.9056528	29.6741579
Zfp821	13.5694683	0.55440088	0.33822115	1.6391668	0.10117853	0.87219738	Zfp821	10.0806949	10.8605693	8.58813393	12.4088401	18.3129289	11.7605452	18.2830139	18.2610202
Ech1	13.0171478	-0.5859338	0.35746587	-1.6391321	0.10118576	0.87219738	Ech1	14.7850192	18.5534725	14.9497146	18.6132601	13.3184938	5.88027262	13.4716944	4.56525506
Atm	19.9371676	0.49974757	0.30493898	1.63884451	0.10124565	0.87219738	Atm	18.8172972	17.1959013	15.9039517	10.3407001	23.3073641	27.0492541	24.0565972	22.8262753
Col4a3bp	68.9756746	0.39645661	0.24196013	1.63852039	0.10131318	0.87219738	Col4a3bp	55.1077989	47.9675142	57.5723052	76.5211804	66.5924688	78.7956531	77.943375	91.3051012
Nckap1l	3.10206591	-0.6804756	0.41531531	-1.6384555	0.10132672	NA	Nckap1l	4.7043243	5.43028463	7.63389683	0	1.66481172	1.17605452	1.92452778	2.28262753
Ubiad1	6.53286041	-0.672196	0.41029451	-1.6383257	0.10135378	0.87219738	Ubiad1	6.04841696	16.7433776	8.58813393	4.13628002	6.65924688	5.88027262	1.92452778	2.28262753
Mrps24	11.0103466	-0.6471063	0.39532218	-1.6369087	0.10164955	0.87300004	Mrps24	16.8011582	9.95552182	12.4050823	20.6814001	9.98887032	1.17605452	12.5094306	4.56525506
Tmem88b	27.0776776	-0.5852787	0.3575808	-1.6367732	0.10167786	0.87300004	Tmem88b	28.2259458	43.442277	25.4463228	37.2265202	18.3129289	9.40843619	13.4716944	41.0872956
Aqp11	5.65224382	-0.6600729	0.40329371	-1.6367052	0.10169209	0.87300004	Aqp11	6.04841696	11.313093	6.99773876	6.20442003	4.99443516	3.52816357	3.84905555	2.28262753
Gm15706	3.24904279	-0.6812849	0.41629897	-1.6365279	0.10172914	NA	Gm15706	6.04841696	5.88280835	5.08926455	2.06814001	1.66481172	2.35210905	2.88679167	0
Slc26a10	1.59995583	0.60472708	0.36969678	1.63573805	0.10189442	NA	Slc26a10	0	0	1.59039517	0	3.32962344	2.35210905	0.96226389	4.56525506
Whsc1	61.594717	0.58660599	0.35869077	1.63540867	0.10196341	0.87452011	Whsc1	50.4034746	48.8725616	55.345752	26.8858201	36.6258578	118.781507	119.320722	36.5220405
Wwox	30.2437822	0.46545172	0.28473317	1.63469439	0.10211313	0.87452011	Wwox	26.8818531	23.0787097	21.3112953	26.8858201	36.6258578	29.4013631	29.8301805	47.9351782
Trim44	278.223959	0.37308455	0.22822992	1.63468727	0.10211462	0.87452011	Trim44	215.726871	284.184895	254.145149	202.677721	254.716193	392.802211	352.188583	269.350049
Tmem132b	114.095864	0.5126773	0.31405305	1.63245446	0.10258378	0.87742646	Tmem132b	66.5325865	119.918786	97.6502636	74.4530404	138.179373	216.394032	74.0943194	125.544514
Cdkl1	6.69104178	-0.6752526	0.41368394	-1.6322911	0.10261818	0.87742646	Cdkl1	8.73660227	4.52523719	6.67965972	18.6132601	0	2.35210905	5.77358333	6.84788259
Lepre1	1.61200065	-0.6144006	0.37644983	-1.6320915	0.10266021	NA	Lepre1	0.67204633	1.81009488	2.54463228	6.20442003	1.66481172	0	0	0
Vsig2	3.60769963	0.67933816	0.41628879	1.63189154	0.10270234	NA	Vsig2	2.68818531	2.26261859	2.54463228	0	3.32962344	10.5844907	2.88679167	4.56525506
Aplp1	614.875516	-0.5121313	0.31392796	-1.6313658	0.10281315	0.87839086	Aplp1	659.277448	1145.79006	734.126412	436.377542	592.672972	592.73148	262.698042	495.330174
Endou	1.43402916	-0.6011347	0.36860682	-1.630829	0.10292641	NA	Endou	2.01613899	5.88280835	1.90847421	0	1.66481172	0	0	0
Hmox1	1.71061959	-0.6506397	0.39916135	-1.6300168	0.10309795	NA	Hmox1	2.01613899	3.16766603	2.22655324	4.13628002	0	1.17605452	0.96226389	0
Chtf18	1.54922291	0.57269035	0.35144106	1.62954879	0.1031969	NA	Chtf18	0.67204633	0.45252372	0.31807903	0	9.98887032	0	0.96226389	0
Aak1	402.019274	0.38886035	0.23874728	1.62875299	0.10336532	0.88225041	Aak1	363.577064	365.639165	409.367717	229.563541	499.443516	548.041408	433.01875	367.503033
Tmem248	42.7173936	-0.4972104	0.30532783	-1.6284477	0.10342999	0.88225041	Tmem248	33.6023164	72.403795	48.3480132	51.7035003	31.6314227	45.8661264	30.7924444	27.3915304
Zfp94	4.89109536	0.66072157	0.40584055	1.62803243	0.10351801	0.88229703	Zfp94	2.68818531	3.62018975	3.49886938	2.06814001	8.3240586	8.23238167	3.84905555	6.84788259
3110082J24Rik	0.88966541	-0.5487983	0.33727192	-1.6271686	0.1037013	NA	3110082J24Ri	2.68818531	0.45252372	1.90847421	2.06814001	0	0	0	0
Npdc1	93.7709988	-0.4731035	0.29095329	-1.6260461	0.10393984	0.88494941	Npdc1	78.6294204	145.712637	97.3321846	122.020261	74.9165274	72.9153805	49.0754583	109.566121
Adnp2	24.8566773	0.51135995	0.31469013	1.62496342	0.10417035	0.88494941	Adnp2	20.8334362	15.3858064	24.8101647	16.5451201	38.2906696	19.9929269	35.6037639	27.3915304
Tbc1d14	67.0260865	-0.4515748	0.27800942	-1.6243146	0.10430868	0.88494941	Tbc1d14	71.2369108	65.6159392	65.8423601	115.815841	51.6091633	39.9858538	64.4716805	61.6309433
Ncl	102.717654	0.56341773	0.34691308	1.62408906	0.1043568	0.88494941	Ncl	91.3983007	77.8340797	90.6525248	49.6353602	58.2684102	228.154578	148.188639	77.6093361
Fabp7	8.80301723	-0.6445862	0.3970438	-1.6234638	0.10449029	0.88494941	Fabp7	18.8172972	13.1231878	9.224292	6.20442003	6.65924688	3.52816357	10.5849028	2.28262753
Syt10	8.87508845	-0.6603964	0.40679491	-1.6234135	0.10450103	0.88494941	Syt10	10.0806949	19.9110436	16.2220308	2.06814001	3.32962344	9.40843619	7.69811111	2.28262753
Snrnp40	14.5705682	0.61028927	0.37596708	1.62325189	0.10453557	0.88494941	Snrnp40	13.4409266	11.313093	6.36158069	10.3407001	23.3073641	5.88027262	23.0943333	22.8262753
Mitf	8.73752381	0.64300806	0.39615449	1.62312452	0.10456279	0.88494941	Mitf	9.4086486	3.62018975	6.67965972	4.13628002	4.99443516	18.8168724	15.3962222	6.84788259
Moap1	31.9605941	0.54502408	0.33579821	1.62307023	0.10457439	0.88494941	Moap1	26.2098068	21.7211385	26.7186389	24.8176801	24.9721758	29.4013631	72.1697916	29.6741579
Pip4k2a	48.8810216	-0.5537529	0.34140437	-1.6219855	0.10480646	0.88499699	Pip4k2a	73.9250961	78.2866034	54.0734359	37.2265202	21.6425524	37.6337448	65.4339444	22.8262753
Tnfrsf11a	4.67092144	0.6443118	0.39732	1.62164453	0.10487948	0.88499699	Tnfrsf11a	2.01613899	1.81009488	1.59039517	2.06814001	0	7.05632714	0	22.8262753
Dad1	84.4476486	-0.5588862	0.34467764	-1.6214751	0.10491578	0.88499699	Dad1	60.4841696	111.773359	79.5197586	167.519341	64.9276571	42.3379629	39.4528194	109.566121
Rasd2	106.458727	-0.5695617	0.35126894	-1.6214407	0.10492317	0.88499699	Rasd2	155.242702	88.2421252	123.732744	163.383061	88.2350212	51.7463991	32.7169722	148.37079
Zfp410	9.51582763	-0.6155598	0.37976992	-1.6208756	0.10504433	0.88499699	Zfp410	13.4409266	10.8605693	12.0870033	14.4769801	1.66481172	9.40843619	9.62263889	4.56525506
Fabp5	7.79285325	-0.6366698	0.3928305	-1.6207241	0.10507683	0.88499699	Fabp5	10.0806949	10.8605693	9.54237104	12.4088401	1.66481172	5.88027262	9.62263889	2.28262753
Rec8	4.16085505	0.67107464	0.41415475	1.62034758	0.10515763	NA	Rec8	2.01613899	0.90504744	0.63615807	6.20442003	6.65924688	3.52816357	1.92452778	11.4131377
Sep-02	23.636229	-0.5370156	0.33163428	-1.6193006	0.10538261	0.88637504	Sep-02	27.5538995	35.7493738	31.4898244	22.7495401	14.9833055	24.697145	25.0188611	6.84788259
Kansl2	16.43243	0.59684581	0.36865554	1.61897962	0.10545165	0.88637504	Kansl2	10.7527413	5.88280835	13.0412404	18.6132601	23.3073641	10.5844907	17.32075	31.9567854
Ankrd9	9.76972605	0.67178075	0.4150771	1.6184481	0.10556606	0.88637504	Ankrd9	5.37637063	2.26261859	2.54463228	14.4769801	24.9721758	3.52816357	6.73584722	18.2610202
Syt6	22.6992021	-0.5351762	0.33074031	-1.6181159	0.10563762	0.88637504	Syt6	27.5538995	24.4362808	27.672876	33.0902402	9.98887032	29.4013631	13.4716944	15.9783927
Hist1h2bm	1.44914845	-0.6026146	0.37243224	-1.6180518	0.10565144	NA	Hist1h2bm	1.34409266	1.81009488	1.27231614	6.20442003	0	0	0.96226389	0
Fra10ac1	7.05269201	0.6396766	0.39535972	1.61796099	0.105671	0.88637504	Fra10ac1	3.36023164	4.07271347	5.40734359	6.20442003	3.32962344	9.40843619	8.660375	15.9783927
Masp2	6.94035701	0.63403291	0.3919469	1.61765003	0.10573804	0.88637504	Masp2	5.37637063	5.88280835	4.77118552	2.06814001	6.65924688	5.88027262	13.4716944	11.4131377
Snrnp200	98.5377347	0.42379352	0.26223385	1.61609008	0.10607484	0.88774066	Snrnp200	98.1187639	68.7836053	94.1513942	66.1804803	156.492302	115.253343	86.60375	102.718239
Dync1li1	49.7603731	0.47798923	0.29581807	1.61582163	0.10613288	0.88774066	Dync1li1	57.7959842	36.2018975	44.8491439	18.6132601	61.5980337	63.5069443	53.8867778	61.6309433
Vps13c	54.59955	0.517801	0.32052994	1.61545284	0.10621266	0.88774066	Vps13c	43.010965	52.2664553	45.803381	28.9539601	86.5702095	97.6125255	48.1131944	34.239413
Ddit4l	7.16098924	-0.6354161	0.39347435	-1.6148857	0.10633546	0.88774066	Ddit4l	11.4247876	8.59795066	11.4508452	6.20442003	4.99443516	2.35210905	7.69811111	4.56525506
Adipor1	54.7920302	-0.4623091	0.28630062	-1.6147681	0.10636092	0.88774066	Adipor1	44.3550577	84.1694117	72.2039408	55.8397803	51.6091633	38.8097993	36.5660278	54.7830607
Zmpste24	16.2607772	-0.5994712	0.37128367	-1.614591	0.10639931	0.88774066	Zmpste24	18.8172972	30.7716129	24.8101647	8.27256004	9.98887032	19.9929269	10.5849028	6.84788259
Zbtb46	4.25546985	0.67109665	0.41566099	1.61452882	0.10641277	NA	Zbtb46	2.01613899	2.71514231	3.81694841	0	4.99443516	2.35210905	6.73584722	11.4131377
Ttpa	2.8191655	-0.669737	0.41485559	-1.6143858	0.10644378	NA	Ttpa	6.72046328	4.07271347	4.45310648	2.06814001	0	2.35210905	2.88679167	0
Mynn	11.0851691	-0.5899985	0.36574573	-1.6131384	0.10671444	0.88967547	Mynn	12.7688802	16.7433776	11.1327662	16.5451201	3.32962344	9.40843619	9.62263889	9.13051012
Ccdc28b	2.47568201	-0.6626029	0.41091029	-1.6125244	0.1068487	NA	Ccdc28b	5.37637063	3.62018975	6.04350166	0	1.66481172	1.17605452	1.92452778	0
Ube2d1	47.4641003	-0.5510623	0.34188507	-1.6118349	0.10699788	0.89090819	Ube2d1	71.9089571	69.6886527	62.9796488	31.0221002	16.6481172	42.3379629	57.7358333	27.3915304
Pdzrn4	6.41268325	0.64903654	0.40270511	1.61169183	0.10702902	0.89090819	Pdzrn4	7.39250961	4.97776091	2.54463228	2.06814001	6.65924688	10.5844907	12.5094306	4.56525506
1700003D09Rik	3.03608173	-0.6552732	0.40661629	-1.6115271	0.10706488	NA	1700003D09Ri	8.06455594	2.71514231	8.2700549	0	0	2.35210905	2.88679167	0
Gpx3	40.4648043	-0.6113501	0.37969195	-1.6101214	0.10737135	0.89326229	Gpx3	23.5216215	42.0847059	29.2632712	115.815841	24.9721758	23.5210905	21.1698055	43.3699231
6330409D20Rik	2.39289998	-0.6430299	0.39939741	-1.6100002	0.10739781	NA	6330409D20Ri	3.36023164	2.71514231	1.90847421	8.27256004	0	0	2.88679167	0
Slc35f2	1.03675893	-0.5301404	0.32931283	-1.6098383	0.10743317	NA	Slc35f2	0.67204633	3.16766603	0.31807903	4.13628002	0	0	0	0
Hopx	12.1719121	-0.5999298	0.37270609	-1.6096593	0.10747225	0.89320639	Hopx	23.5216215	13.5757116	11.1327662	14.4769801	8.3240586	4.7042181	12.5094306	9.13051012
Chat	0.91117401	-0.5168704	0.32123397	-1.6090155	0.10761296	NA	Chat	0.67204633	4.07271347	2.54463228	0	0	0	0	0
Med25	51.0813556	-0.5551221	0.34503314	-1.6088951	0.10763928	0.89384531	Med25	59.8121232	53.3977988	45.1672229	95.1344405	58.2684102	16.4647633	27.9056528	52.5004332
Cmtr1	55.1987476	0.38550247	0.23965947	1.60854262	0.10771639	0.89384531	Cmtr1	41.6686724	52.9452751	41.3502745	53.7716403	66.5924688	58.8027262	62.5471528	63.9135709
Rdh5	1.18380817	-0.5174978	0.32188452	-1.6077125	0.10789817	NA	Rdh5	0	1.35757116	1.90847421	6.20442003	0	0	0	0
Unc80	329.091315	0.42305764	0.26321104	1.60729446	0.10798979	0.89447138	Unc80	292.340153	274.229374	264.323678	270.926341	431.186236	557.449844	275.207472	267.067421
Steap3	1.56656641	-0.6273434	0.39034323	-1.6071584	0.10801963	NA	Steap3	2.68818531	4.52523719	3.18079035	0	0	1.17605452	0.96226389	0
Slc44a2	13.6418364	-0.6449955	0.40135961	-1.6070265	0.10804854	0.89447138	Slc44a2	12.0968339	33.9392789	16.8581888	10.3407001	9.98887032	18.8168724	4.81131944	2.28262753
5730409E04Rik	85.3182536	0.4966605	0.30909725	1.60680983	0.10809609	0.89447138	5730409E04Ri	51.075521	47.5149905	62.6615698	113.747701	101.553515	67.0351079	101.999972	136.957652

GeneID	Base mean	log2(FC)	StdErr	Wald-Stats	P-value	P-adj	GeneID	Normalised expression for Chow#1	Normalised expression for Chow#2	Normalised expression for Chow#3	Normalised expression for Chow#4	Normalised expression for HFD#1	Normalised expression for HFD#2	Normalised expression for HFD#3	Normalised expression for HFD#4
Coro1a	34.7647658	0.56947083	0.35444151	1.60667085	0.10812659	0.89447138	Coro1a	19.4893435	17.1959013	21.9474534	47.5672202	64.9276571	21.1689814	35.6037639	50.2178057
A730056A06Rik	2.20226487	-0.6551027	0.40779742	-1.6064415	0.10817693	NA	A730056A06R	2.68818531	4.97776091	2.22655324	4.13628002	1.66481172	0	1.92452778	0
Shisa2	5.05176354	0.66859708	0.41632354	1.6059555	0.10828369	0.89486422	Shisa2	3.36023164	2.26261859	4.13502745	0	6.65924688	7.05632714	0.96226389	15.9783927
Anxa5	17.1962671	-0.5526312	0.34417007	-1.6056923	0.10834153	0.89486422	Anxa5	22.8495752	17.648425	15.9039517	28.9539601	13.3184938	12.9365998	7.69811111	18.2610202
Spsb4	10.3695553	0.66052206	0.41186658	1.60372821	0.10877398	0.89774228	Spsb4	4.03227797	4.97776091	6.04350166	10.3407001	14.9833055	3.52816357	4.81131944	34.239413
Mri1	4.10860567	0.66742482	0.41632116	1.60314893	0.10890178	NA	Mri1	1.34409266	1.35757116	2.54463228	4.13628002	1.66481172	2.35210905	5.77358333	13.6957652
Stxbp3a	4.75205601	-0.6514226	0.40683286	-1.6012044	0.10933166	0.89997302	Stxbp3a	6.04841696	7.69290322	6.04350166	6.20442003	3.32962344	3.52816357	2.88679167	2.28262753
Tspan31	32.7890753	-0.5570512	0.34798683	-1.6007824	0.10942511	0.89997302	Tspan31	32.2582238	69.236129	37.215247	24.8176801	19.9777406	35.2816357	29.8301805	13.6957652
Acy1	3.79237043	-0.6645255	0.41516586	-1.6006265	0.10945968	NA	Acy1	3.36023164	3.62018975	3.81694841	12.4088401	4.99443516	1.17605452	0.96226389	0
Map4k5	18.8420092	-0.6242276	0.38998962	-1.6006262	0.10945974	0.89997302	Map4k5	26.2098068	37.5594687	29.5813502	4.13628002	23.3073641	10.5844907	12.5094306	6.84788259
1700008O03Rik	1.91441443	0.64361563	0.40221521	1.60017725	0.10955927	NA	1700008O03R	1.34409266	0.90504744	0.9542371	0	6.65924688	3.52816357	1.92452778	0
Mpdz	48.3824136	0.41375989	0.25864488	1.59972192	0.10966029	0.89997302	Mpdz	49.7314283	42.5372296	37.8514051	31.0221002	58.2684102	67.0351079	48.1131944	52.5004332
Tysnd1	11.1467207	0.63610089	0.39774874	1.59925305	0.10976439	0.89997302	Tysnd1	7.39250961	6.33533207	6.99773876	8.27256004	18.3129289	3.52816357	8.660375	29.6741579
Xkr4	49.1939616	0.50518941	0.31591238	1.59914406	0.1097886	0.89997302	Xkr4	30.2420848	52.9452751	42.9406697	28.9539601	54.9387868	94.0843619	52.9245139	36.5220405
Ccdc91	24.5391708	0.51521257	0.32221321	1.59898029	0.10982498	0.89997302	Ccdc91	23.5216215	12.2181404	21.3112953	20.6814001	19.9777406	25.8731995	38.4905555	34.239413
Htra3	1.44814582	-0.6054161	0.37865331	-1.5988665	0.10985027	NA	Htra3	1.34409266	3.16766603	1.27231614	4.13628002	1.66481172	0	0	0
Pitpna	368.545734	0.34089945	0.2132321	1.59872484	0.10988176	0.89997302	Pitpna	359.544786	291.877799	344.479594	289.539601	321.308662	381.041666	456.113083	504.460684
Galk2	13.4562854	0.54995642	0.34399828	1.59871852	0.10988316	0.89997302	Galk2	10.7527413	9.05047438	11.4508452	10.3407001	21.6425524	14.1126543	21.1698055	9.13051012
Fau	13.6766344	-0.6127289	0.38333949	-1.5983976	0.10995452	0.89997302	Fau	21.5054825	10.8605693	8.90621297	31.0221002	9.98887032	7.05632714	8.660375	11.4131377
Spred3	78.2316678	0.57913356	0.36239395	1.59807735	0.11002576	0.89997302	Spred3	46.3711967	38.9170398	47.3937761	99.2707205	143.173808	52.9224536	47.1509305	150.653417
Arhgap18	2.85374498	-0.6624778	0.41457853	-1.5979549	0.11005301	NA	Arhgap18	4.7043243	6.78785578	3.81694841	2.06814001	0	3.52816357	1.92452778	0
Hunk	12.7615151	0.56266595	0.35211797	1.59794728	0.11005471	0.89997302	Hunk	7.39250961	10.4080455	9.86045007	10.3407001	23.3073641	9.40843619	15.3962222	15.9783927
Ttyh2	11.9008031	-0.655357	0.41044556	-1.5966966	0.11033335	0.90060124	Ttyh2	7.39250961	26.2463757	15.5858727	14.4769801	13.3184938	3.52816357	0.96226389	13.6957652
Grn	26.858471	-0.6132385	0.38412362	-1.5964612	0.11038586	0.90060124	Grn	18.8172972	64.7108918	25.7644018	28.9539601	31.6314227	19.9929269	6.73584722	18.2610202
Grb2	103.899543	0.40538444	0.25409017	1.59543535	0.11061492	0.90060124	Grb2	87.3660227	68.7836053	77.2932054	119.952121	129.855314	94.0843619	107.773556	146.088162
Gal	2.15535146	-0.602745	0.37784333	-1.5952246	0.11066202	NA	Gal	0.67204633	2.71514231	1.59039517	10.3407001	0	0	1.92452778	0
Nfrkb	11.1980203	0.58448392	0.36642856	1.59508288	0.1106937	0.90060124	Nfrkb	12.0968339	5.43028463	8.58813393	6.20442003	19.9777406	15.2887088	10.5849028	11.4131377
Wnt7a	12.9501284	0.62166896	0.38986904	1.59455844	0.11081101	0.90060124	Wnt7a	8.73660227	8.59795066	5.08926455	14.4769801	16.6481172	24.697145	4.81131944	20.5436478
Kctd11	1.27015071	-0.5972153	0.37514937	-1.5919401	0.11139817	NA	Kctd11	1.34409266	1.81009488	1.90847421	4.13628002	0	0	0.96226389	0
Slc6a1	699.824051	0.48442285	0.30432777	1.59177998	0.11143415	0.90060124	Slc6a1	360.888878	576.515218	556.320231	754.871104	812.42812	999.646345	426.282903	1111.63961
Smarca4	115.516145	0.38093171	0.23936334	1.59143714	0.11151123	0.90060124	Smarca4	98.1187639	106.343074	97.9683426	90.9981605	183.129289	122.309671	101.999972	123.261887
Gsn	8.3753278	-0.6195989	0.38943523	-1.5910192	0.11160525	0.90060124	Gsn	13.4409266	13.1231878	7.95197586	8.27256004	9.98887032	3.52816357	3.84905555	6.84788259
Pvrl2	5.62203047	0.66024661	0.41510196	1.5905649	0.11170753	0.90060124	Pvrl2	3.36023164	4.97776091	3.81694841	0	18.3129289	7.05632714	2.88679167	4.56525506
Mettl13	6.79151277	0.6377026	0.40095461	1.59046082	0.11173097	0.90060124	Mettl13	6.04841696	2.71514231	2.54463228	8.27256004	6.65924688	7.05632714	9.62263889	11.4131377
Atp5a1	583.213443	0.40651676	0.25564598	1.59015511	0.11179985	0.90060124	Atp5a1	514.115441	363.82907	424.95359	668.009223	785.791132	522.168209	569.660222	817.180656
Srek1	26.2711438	0.50801513	0.31948116	1.5901255	0.11180652	0.90060124	Srek1	18.8172972	17.648425	22.9016905	24.8176801	31.6314227	49.39429	22.1320694	22.8262753
Slc3a2	47.6100915	-0.5069282	0.31882458	-1.589991	0.11183683	0.90060124	Slc3a2	32.9302701	74.2138899	55.345752	68.2486203	39.9554813	50.5703445	23.0943333	36.5220405
2610002M06Rik	76.5864935	0.51602956	0.32458259	1.58982514	0.11187423	0.90060124	2610002M06R	61.1562159	60.1856546	84.6090232	33.0902402	63.2628454	110.549125	140.490528	59.3483158
Ankrd26	12.7720044	0.63420943	0.3989766	1.58959053	0.11192713	0.90060124	Ankrd26	9.4086486	9.05047438	13.0412404	2.06814001	11.653682	34.1055812	18.2830139	4.56525506
Plekha2	13.0367563	-0.6216518	0.39110094	-1.589492	0.11194935	0.90060124	Plekha2	20.8334362	21.7211385	20.0389792	6.20442003	11.653682	9.40843619	14.4339583	0
Ap3d1	139.167931	0.47143271	0.29660225	1.58944415	0.11196015	0.90060124	Ap3d1	116.936061	80.0966983	100.194896	155.110501	231.408829	129.365998	103.9245	196.305968
Meis2	78.4682057	-0.5468292	0.34408659	-1.5892198	0.11201077	0.90060124	Meis2	133.737219	105.89055	116.735006	31.0221002	49.9443516	87.0280348	57.7358333	45.6525506
C030018K13Rik	0.82379859	-0.5194764	0.32693192	-1.5889437	0.1120731	NA	C030018K13Ri	2.68818531	1.35757116	2.54463228	0	0	0	0	0
Iffo1	28.236373	0.62294094	0.39212346	1.58863472	0.11214289	0.90060124	Iffo1	22.1775288	10.4080455	12.7231614	33.0902402	56.6035985	19.9929269	11.5471667	59.3483158
Mief2	15.3845231	0.65874401	0.41466713	1.58860918	0.11214865	0.90060124	Mief2	3.36023164	6.33533207	5.72542262	22.7495401	26.6369875	5.88027262	6.73584722	45.6525506
Trmu	5.6475713	0.66127791	0.41634243	1.58830296	0.11221785	0.90060124	Trmu	4.03227797	0.45252372	1.59039517	8.27256004	6.65924688	4.7042181	5.77358333	13.6957652
Adssl1	10.7749954	0.5905019	0.37180786	1.58819099	0.11224316	0.90060124	Adssl1	12.0968339	6.33533207	5.40734359	8.27256004	14.9833055	9.40843619	18.2830139	11.4131377
Smad1	26.1465009	-0.4660417	0.29346885	-1.5880448	0.11227621	0.90060124	Smad1	27.5538995	29.8665654	27.672876	41.3628002	19.9777406	22.345036	22.1320694	18.2610202
Dlc1	92.2523874	0.34408087	0.2166717	1.58802867	0.11227986	0.90060124	Dlc1	86.6939764	79.6441745	71.2497037	84.7937404	119.866444	105.844907	89.4905416	100.435611
Pla2g12a	7.80479904	-0.6121616	0.38548758	-1.5880189	0.11228206	0.90060124	Pla2g12a	10.0806949	8.14542694	9.224292	14.4769801	3.32962344	5.88027262	6.73584722	4.56525506
Gne	34.1545076	0.47100894	0.29663488	1.58784069	0.11232237	0.90060124	Gne	35.6184554	25.3413283	27.354797	20.6814001	59.9332219	34.1055812	33.6792361	36.5220405
Park2	3.52839867	-0.6562663	0.41350903	-1.5870665	0.11249759	NA	Park2	6.04841696	1.35757116	4.13502745	10.3407001	0	1.17605452	2.88679167	2.28262753
Ctsd	118.929226	-0.517397	0.32601401	-1.5870393	0.11250376	0.90137941	Ctsd	105.511274	237.574952	130.412404	103.407001	116.53682	111.72518	48.1131944	98.1529838
Otp	1.09196886	-0.5281956	0.33294418	-1.586439	0.11263976	NA	Otp	0.67204633	0.90504744	0.9542371	6.20442003	0	0	0	0
Inpp5d	2.66504901	-0.6550582	0.41291254	-1.5864334	0.11264104	NA	Inpp5d	4.03227797	2.71514231	3.81694841	6.20442003	1.66481172	0	2.88679167	0
Gps2	9.66206512	0.63189959	0.39840952	1.58605544	0.11272673	0.90154924	Gps2	8.73660227	8.59795066	5.08926455	2.06814001	26.6369875	7.05632714	7.69811111	11.4131377
Mtus2	42.4206714	0.44194809	0.27873808	1.58553177	0.11284556	0.90154924	Mtus2	43.010965	26.2463757	30.8536663	41.3628002	59.9332219	50.5703445	39.4528194	47.9351782
Mrpl4	22.3926417	-0.4943427	0.31178749	-1.5855118	0.1128501	0.90154924	Mrpl4	24.1936678	22.1736622	25.1282437	37.2265202	14.9833055	15.2887088	17.32075	22.8262753
Tor1aip2	33.6157617	-0.4463483	0.28152647	-1.5854578	0.11286236	0.90154924	Tor1aip2	40.3227797	39.3695635	47.3937761	31.0221002	23.3073641	32.9295267	31.7547083	22.8262753
Sncaip	2.15798947	-0.6155036	0.38837591	-1.584814	0.11300859	NA	Sncaip	7.39250961	3.16766603	3.81694841	0	0	0	2.88679167	0
Car11	49.8942583	-0.5370575	0.33904534	-1.5840287	0.11318719	0.90316035	Car11	69.8928182	84.6219354	63.6158069	24.8176801	56.6035985	39.9858538	23.0943333	36.5220405
Fam133b	13.0755602	0.56253826	0.35517654	1.58382718	0.11323305	0.90316035	Fam133b	9.4086486	9.5029981	10.1785291	10.3407001	9.98887032	10.5844907	24.0565972	20.5436478
P4ha3	3.83773736	-0.6588646	0.41604872	-1.5836237	0.11327938	NA	P4ha3	4.03227797	6.78785578	5.40734359	6.20442003	0	1.17605452	4.81131944	2.28262753
Jam3	5.74291612	-0.6562675	0.41458439	-1.5829528	0.11343221	0.90360992	Jam3	6.04841696	15.8383302	7.95197586	2.06814001	6.65924688	3.52816357	3.84905555	0
Ctnnal1	4.41011844	-0.6589602	0.41632723	-1.5827939	0.11346845	NA	Ctnnal1	8.73660227	3.16766603	4.45310648	8.27256004	1.66481172	1.17605452	0.96226389	6.84788259
Spin1	239.747121	0.31878885	0.20144099	1.5825421	0.11352587	0.90360992	Spin1	181.452509	194.132675	214.703348	260.585641	253.051381	245.795396	301.188597	267.067421
Pcdhgc4	241.016883	-0.4593942	0.29030261	-1.5824668	0.11354305	0.90360992	Pcdhgc4	282.931504	402.293586	280.863787	171.655621	244.727323	230.506687	127.981097	187.175458
Mpeg1	4.11003008	-0.6546827	0.41378252	-1.5821904	0.11360611	NA	Mpeg1	2.01613899	11.7656167	5.08926455	6.20442003	0	5.88027262	1.92452778	0
Synpo2l	0.69962506	0.47335434	0.29918046	1.58216994	0.11361078	NA	Synpo2l	0	0	0	0	0	2.35210905	0.96226389	2.28262753
Arsb	83.7731011	-0.5366529	0.33932347	-1.5815378	0.11375512	0.90433371	Arsb	84.0057911	162.908539	121.506191	41.3628002	84.9053977	70.5632714	43.301875	61.6309433
Usp48	25.4324348	0.44992715	0.284525	1.58132729	0.11380322	0.90433371	Usp48	24.8657142	21.7211385	19.7209001	16.5451201	31.6314227	31.7534722	29.8301805	27.3915304
Clptm1	116.177977	-0.4596998	0.29087239	-1.5804173	0.11401132	0.90476664	Clptm1	110.215598	185.082201	123.414665	130.292821	108.212762	127.013889	53.8867778	91.3051012
Nkain3	23.6250888	0.62788425	0.39730738	1.58034881	0.11402701	0.90476664	Nkain3	12.7688802	26.6988994	17.4943469	6.20442003	19.9777406	72.9153805	19.2452778	13.6957652
Mblac2	24.7957211	0.54242671	0.34343781	1.579403	0.11424365	0.9053235	Mblac2	18.8172972	14.0282353	15.9039517	31.0221002	21.6425524	30.5774176	48.1131944	18.2610202
Tppp3	27.8627115	-0.6498696	0.41161409	-1.5788323	0.11437452	0.9053235	Tppp3	16.1291119	16.7433776	12.4050823	109.611421	18.3129289	8.23238167	16.3584861	25.1089028
Mzt2	9.82157221	0.58134539	0.36829316	1.57848543	0.11445413	0.9053235	Mzt2	5.37637063	9.5029981	6.67965972	6.20442003	9.98887032	12.9365998	9.62263889	18.2610202
1190005I06Rik	1.76105736	-0.629656	0.39890299	-1.578469	0.1144579	NA	1190005I06Ri	2.01613899	1.81009488	3.49886938	4.13628002	1.66481172	0	0.96226389	0
Gm4285	4.01157798	0.65599518	0.41560665	1.57840397	0.11447283	NA	Gm4285	3.36023164	2.71514231	2.54463228	0	3.32962344	2.35210905	8.660375	9.13051012
Epm2aip1	188.801593	0.3594815	0.22780412	1.57802892	0.11455897	0.9053235	Epm2aip1	146.5061	143.902543	161.902229	206.814001	186.458913	243.443286	250.188611	171.197065
Thop1	21.0864979	0.52681157	0.33390109	1.57774738	0.11462366	0.9053235	Thop1	20.8334362	16.7433776	12.4050823	14.4769801	39.9554813	17.6408179	19.2452778	27.3915304
Srek1ip1	13.5933706	0.55584682	0.35236016	1.57749623	0.11468139	0.9053235	Srek1ip1	11.4247876	12.2181404	11.7689243	4.13628002	9.98887032	18.8168724	22.1320694	18.2610202
Tceb2	48.198217	-0.5171486	0.32783663	-1.5774583	0.11469012	0.9053235	Tceb2	74.5971425	64.2583681	68.7050715	26.8858201	33.2962344	31.7534722	58.6980972	27.3915304
Tmem176b	19.9505995	-0.5384482	0.34143566	-1.5770123	0.11479271	0.90546453	Tmem176b	18.1452509	34.3918026	22.9016905	20.6814001	18.3129289	11.7605452	10.5849028	22.8262753
Surf1	31.9345476	-0.5294477	0.33585495	-1.5764179	0.11492954	0.90587528	Surf1	28.8979921	31.6766603	30.2175083	68.2486203	21.6425524	15.2887088	29.8301805	29.6741579
Lad1	0.82828463	-0.5137202	0.32595504	-1.5760462	0.11501517	NA	Lad1	3.36023164	1.35757116	1.90847421	0	0	0	0	0
Hipk4	13.9289372	0.65114545	0.41322646	1.57575931	0.11508128	0.90597922	Hipk4	7.39250961	10.4080455	10.8146872	4.13628002	39.9554813	9.40843619	1.92452778	27.3915304
Ccdc94	2.92995591	0.63941868	0.40581366	1.57564602	0.1151074	NA	Ccdc94	2.01613899	0.45252372	2.22655324	0	1.66481172	4.7042181	0.96226389	11.4131377
Snx12	60.3113879	0.49223288	0.3124989	1.57515077	0.11522165	0.90597922	Snx12	48.3873356	31.6766603	45.1672229	68.2486203	79.9109626	39.9858538	66.3962083	102.718239
Hmgb3	11.4939039	0.61687464	0.391649	1.57507014	0.11524026	0.90597922	Hmgb3	10.7527413	2.26261859	6.04350166	14.4769801	13.3184938	15.2887088	11.5471667	18.2610202
Tpgs2	62.4262531	-0.4178174	0.26529938	-1.5748902	0.11528179	0.90597922	Tpgs2	89.3821617	66.9735104	76.0208892	57.9079203	41.620293	47.042181	70.2452639	50.2178057
Ednra	0.80867782	-0.5115202	0.32490441	-1.5743714	0.11540161	NA	Ednra	1.34409266	2.26261859	2.86271131	0	0	0	0	0
Pou3f3os	14.0285403	-0.6473244	0.41122496	-1.574137	0.11545577	0.90609445	Pou3f3os	22.1775288	28.0564706	17.4943469	10.3407001	3.32962344	3.52816357	25.0188611	2.28262753
Gsr	23.0741663	-0.5024712	0.31921319	-1.5740928	0.115466	0.90609445	Gsr	36.2905017	22.6261859	25.7644018	28.9539601	18.3129289	22.345036	21.1698055	9.13051012
Vwa1	6.32623488	-0.6513796	0.41402818	-1.5732736	0.11565547	0.90691538	Vwa1	4.7043243	15.3858064	6.04350166	8.27256004	8.3240586	2.35210905	0.96226389	4.56525506
Mtmr9	74.9250148	0.34290945	0.21810073	1.57225269	0.11589196	0.90755297	Mtmr9	71.2369108	58.8280835	66.7965972	64.1123403	86.5702095	75.2674895	87.5660139	89.0224737
Jade2	91.3108515	0.52663201	0.33500414	1.57201643	0.11594674	0.90755297	Jade2	65.1884939	51.587704	61.3892537	105.475141	136.514561	85.8519803	50.9999861	173.479692
Grb7	0.96170306	-0.5292472	0.33668795	-1.5719222	0.11596861	NA	Grb7	2.01613899	0.90504744	0.63615807	4.13628002	0	0	0	0
Ahsa1	62.7621654	0.36080754	0.22956963	1.57166934	0.11602726	0.90755297	Ahsa1	57.1239379	49.3250854	52.8011197	57.9079203	61.5980337	70.5632714	68.3207361	84.4572186
Bet1	17.0161596	-0.5888884	0.3747403	-1.5714574	0.11607643	0.90755297	Bet1	17.4732045	18.5534725	14.3135566	37.2265202	4.99443516	8.23238167	12.5094306	22.8262753
Arhgef12	380.024188	0.32224051	0.20524803	1.57000535	0.11641387	0.90949762	Arhgef12	267.474439	327.627173	341.616883	405.355442	436.180671	471.597864	358.92443	431.416603
Gm5089	1.27941038	-0.5656269	0.36031455	-1.5698141	0.11645836	NA	Gm5089	4.03227797	4.07271347	0.9542371	0	0	1.17605452	0	0
Kctd5	17.428244	-0.5336422	0.34003013	-1.5693968	0.11655551	0.90949762	Kctd5	22.8495752	21.7211385	18.448584	22.7495401	4.99443516	11.7605452	16.3584861	20.5436478
St13	71.5778561	0.40028305	0.25514993	1.56881503	0.11669104	0.90949762	St13	64.5164475	50.2301328	64.251965	64.1123403	53.2739751	87.0280348	93.3395972	95.8703563
Cdc42bpb	167.736487	0.46391779	0.29575369	1.56859509	0.11674232	0.90949762	Cdc42bpb	125.000617	99.5552182	118.64348	204.745861	264.705064	156.415252	130.867889	241.958518
Rae1	11.7993501	0.6083609	0.38793913	1.5681865	0.11683761	0.90949762	Rae1	5.37637063	6.33533207	5.08926455	18.6132601	19.9777406	8.23238167	12.5094306	18.2610202
Stra13	4.30464831	-0.6511456	0.41531507	-1.5678351	0.11691961	NA	Stra13	8.73660227	6.33533207	3.18079035	8.27256004	0	1.17605452	6.73584722	0
Kif1a	880.768877	0.43355418	0.27655269	1.56770911	0.11694904	0.90949762	Kif1a	674.734514	814.09017	656.515127	777.620644	1465.03431	1181.9348	567.735694	908.485757
L3mbtl2	36.8872102	0.46401478	0.29599447	1.56764677	0.11696359	0.90949762	L3mbtl2	31.5861774	24.8888045	25.4463228	39.2946602	58.2684102	28.2253086	39.4528194	47.9351782
Thada	14.9866962	0.5774812	0.36851553	1.56704713	0.11710368	0.90949762	Thada	6.72046328	12.6706641	10.8146872	14.4769801	28.3017992	25.8731995	9.62263889	11.4131377
Ptprm	43.4065089	0.56729401	0.36208098	1.56676003	0.11717079	0.90949762	Ptprm	32.2582238	43.8948007	31.8079035	20.6814001	43.2851047	111.72518	38.4905555	25.1089028
Kxd1	16.1723153	-0.5714849	0.36478078	-1.5666529	0.11719585	0.90949762	Kxd1	26.2098068	19.9110436	18.130505	18.6132601	11.653682	5.88027262	22.1320694	6.84788259
Tnfsf12Tnfsf13	1.78169041	-0.6054411	0.38648737	-1.5665222	0.11722641	NA	Tnfsf12Tnfsf1	5.37637063	4.97776091	1.27231614	0	1.66481172	0	0.96226389	0

GeneID	Base mean	log2(FC)	StdErr	Wald-Stats	P-value	P-adj	GeneID	Normalised expression for Chow#1	Normalised expression for Chow#2	Normalised expression for Chow#3	Normalised expression for Chow#4	Normalised expression for HFD#1	Normalised expression for HFD#2	Normalised expression for HFD#3	Normalised expression for HFD#4
Tomm7	19.5804182	-0.5003246	0.31941592	-1.5663735	0.1172612	0.90949762	Tomm7	25.5377605	21.2686148	18.130505	31.0221002	14.9833055	15.2887088	14.4339583	15.9783927
Cep63	8.8786935	0.61608852	0.39354683	1.56547704	0.1174711	0.91046489	Cep63	7.39250961	5.88280835	6.67965972	4.13628002	1.66481172	12.9365998	16.3584861	15.9783927
Agrn	56.0378386	0.53870039	0.34422964	1.56494484	0.11759584	0.9107713	Agrn	29.5700384	54.3028463	36.2610099	51.7035003	131.520126	55.2745626	39.4528194	50.2178057
Kcns1	3.62842637	0.65130517	0.41636769	1.56425485	0.11775773	NA	Kcns1	2.01613899	3.62018975	1.59039517	0	8.3240586	4.7042181	1.92452778	6.84788259
Rtn4rl2	28.6390148	-0.5914637	0.37815521	-1.5640767	0.11779956	0.91168838	Rtn4rl2	35.6184554	55.6604174	38.1694841	14.4769801	36.6258578	10.5844907	10.5849028	27.3915304
Otud6b	48.5300067	-0.4527399	0.28959572	-1.5633514	0.11796996	0.91185305	Otud6b	55.1077989	63.8058444	49.6203294	64.1123403	38.2906696	39.9858538	56.7735694	20.5436478
Ermn	11.5280796	-0.6272094	0.40121891	-1.56326	0.11799146	0.91185305	Ermn	14.1129729	25.793852	16.8581888	4.13628002	1.66481172	7.05632714	13.4716944	9.13051012
Nupr1	1.54361211	-0.6065414	0.38812603	-1.5627435	0.11811293	NA	Nupr1	2.68818531	4.97776091	2.54463228	0	0	1.17605452	0.96226389	0
2810459M11Rik	5.6510029	-0.6414725	0.41054814	-1.5624782	0.11817539	0.91198241	2810459M11R	12.7688802	7.69290322	5.40734359	4.13628002	3.32962344	1.17605452	3.84905555	6.84788259
Myl12b	206.191978	-0.3831867	0.24524521	-1.5624634	0.11817886	0.91198241	Myl12b	264.114207	210.876053	225.199956	244.040521	131.520126	134.070216	206.886736	232.828008
Wnt2b	2.4267212	0.62349066	0.39916854	1.56197343	0.11829425	NA	Wnt2b	0	2.71514231	1.27231614	0	6.65924688	5.88027262	2.88679167	0
Upf3b	16.9061999	0.5659276	0.36233686	1.5618825	0.11831567	0.91235667	Upf3b	19.4893435	6.33533207	13.9954775	10.3407001	11.653682	22.345036	25.981125	25.1089028
BC023829	28.512731	-0.5226502	0.33470329	-1.5615328	0.11839808	0.91235667	BC023829	30.2420848	54.3028463	30.8536663	22.7495401	19.9777406	31.7534722	15.3962222	22.8262753
Wisp2	1.1177343	0.44274596	0.28372906	1.56045334	0.11865279	NA	Wisp2	0	0	0	0	6.65924688	0	0	2.28262753
Dync1li2	163.709821	0.30487681	0.19543535	1.55998807	0.1187627	0.91450703	Dync1li2	161.291119	136.209639	143.135566	140.633521	154.82749	176.408179	196.301833	200.871223
Tsc22d3	44.1501758	-0.4988487	0.31985744	-1.5595971	0.11885512	0.91455977	Tsc22d3	37.6345944	77.8340797	48.9841713	47.5672202	26.6369875	22.345036	44.2641389	47.9351782
D10Jhu81e	37.1056155	0.50336848	0.32292525	1.5587771	0.11904915	0.91463363	D10Jhu81e	31.5861774	22.6261859	25.4463228	37.2265202	46.6147282	24.697145	35.6037639	73.044081
Ppp1cc	55.3638477	0.45114153	0.28946176	1.55855309	0.11910219	0.91463363	Ppp1cc	51.7475673	35.2968501	53.4372778	39.2946602	49.9443516	45.8661264	76.0188472	91.3051012
Hspb1	0.85460854	-0.4963627	0.31850285	-1.5584246	0.11913262	NA	Hspb1	0.67204633	3.62018975	2.54463228	0	0	0	0	0
Daam1	60.6166925	0.38331644	0.24596565	1.55841454	0.11913501	0.91463363	Daam1	53.0916599	48.4200379	58.2084633	47.5672202	56.6035985	88.2040893	71.2075278	61.6309433
Klhl28	18.5493726	0.56322564	0.36153054	1.55789227	0.11925879	0.91463363	Klhl28	14.1129729	16.7433776	15.9039517	8.27256004	9.98887032	24.697145	40.4150833	18.2610202
Kif3a	137.861493	0.37467861	0.24057552	1.55742612	0.11936935	0.91463363	Kif3a	141.801775	94.1249335	119.915796	119.952121	148.168243	185.816615	178.981083	114.131377
Slc4a1ap	21.2857582	0.52285689	0.3357266	1.55738893	0.11937817	0.91463363	Slc4a1ap	15.4570656	23.0787097	16.5401098	10.3407001	16.6481172	38.8097993	28.8679167	20.5436478
Efna2	4.15403251	-0.6466509	0.41531177	-1.5570253	0.11946448	NA	Efna2	4.7043243	7.69290322	4.45310648	6.20442003	3.32962344	0	0	6.84788259
Ddn	479.207632	0.51463366	0.33056923	1.55681053	0.11951548	0.91488937	Ddn	485.889495	365.186641	359.429309	285.403321	1033.84808	297.541795	369.509333	636.853081
Islr2	28.0051541	-0.5282531	0.33937928	-1.5565273	0.11958275	0.91488937	Islr2	22.8495752	49.3250854	34.9886938	28.9539601	36.6258578	17.6408179	15.3962222	18.2610202
Rps26	10.2400065	-0.5849946	0.37593714	-1.5560968	0.11968507	0.91501717	Rps26	18.8172972	11.7656167	14.3135566	6.20442003	8.3240586	4.7042181	8.660375	9.13051012
Igsf1	4.59351123	-0.647144	0.41621543	-1.5548295	0.11998669	NA	Igsf1	4.03227797	9.5029981	4.13502745	8.27256004	4.99443516	3.52816357	0	2.28262753
Cystm1	15.6738523	-0.5746628	0.36961358	-1.5547666	0.12000167	0.91673673	Cystm1	21.5054825	23.0787097	16.8581888	16.5451201	13.3184938	2.35210905	13.4716944	18.2610202
Mgat4c	8.79313165	0.64488502	0.41499005	1.55397708	0.12018988	0.91673673	Mgat4c	3.36023164	12.2181404	3.18079035	0	3.32962344	30.5774176	15.3962222	2.28262753
Utp14b	7.63458315	-0.6306245	0.40586775	-1.5537685	0.12023964	0.91673673	Utp14b	10.0806949	13.5757116	14.3135566	2.06814001	9.98887032	5.88027262	2.88679167	2.28262753
Abcf1	42.218961	0.49417178	0.31809968	1.55351236	0.12030078	0.91673673	Abcf1	41.6668724	19.4585199	25.7644018	49.6353602	63.2628454	44.6900719	38.4905555	54.7830607
Sdc2	25.2773973	-0.5713854	0.36785562	-1.5532871	0.12035455	0.91673673	Sdc2	24.8657142	53.8503226	28.6271131	20.6814001	8.3240586	32.9295267	19.2452778	13.6957652
Tbc1d30	62.6754133	0.42786343	0.27556388	1.55268331	0.12049881	0.91673673	Tbc1d30	60.4841696	44.3473245	68.7050715	33.0902402	69.9220923	84.6759257	80.8301666	59.3483158
Tgfbr3	7.30093135	-0.62898	0.40516339	-1.5524109	0.12056394	0.91673673	Tgfbr3	8.06455594	14.9332827	11.7689243	4.13628002	9.98887032	4.7042181	4.81131944	0
Irak1bp1	9.12152903	-0.5969357	0.38455512	-1.5522761	0.12059617	0.91673673	Irak1bp1	12.7688802	13.1231878	12.7231614	8.27256004	1.66481172	4.7042181	10.5849028	9.13051012
Gm20743	0.82695123	-0.5227013	0.33676768	-1.5521124	0.12063534	NA	Gm20743	2.68818531	0.90504744	0.9542371	2.06814001	0	0	0	0
Zkscan16	43.0156272	0.54588816	0.35177666	1.55180325	0.12070931	0.91694463	Zkscan16	39.6507334	25.793852	39.1237212	26.8858201	34.9610461	91.7322529	65.4339444	20.5436478
Serpinb1a	1.96935939	-0.6145462	0.39603551	-1.5517452	0.1207232	NA	Serpinb1a	4.7043243	2.71514231	0.9542371	4.13628002	0	0	0.96226389	2.28262753
Tmco6	2.89626211	-0.6376354	0.41101008	-1.5513862	0.12080916	NA	Tmco6	6.04841696	4.07271347	1.59039517	6.20442003	3.32962344	0	1.92452778	0
Rnf13	47.0474322	-0.6292266	0.40573892	-1.5508165	0.12094566	0.91808752	Rnf13	50.4034746	104.080455	70.2954666	28.9539601	9.98887032	59.9787807	48.1131944	4.56525506
Zufsp	4.63574399	0.64239381	0.41435365	1.5503515	0.12105717	NA	Zufsp	4.7043243	3.16766603	3.18079035	0	3.32962344	11.7605452	8.660375	2.28262753
Rita1	14.001099	0.54810544	0.35358117	1.55015451	0.12110444	0.91823128	Rita1	10.7527413	6.78785578	9.54237104	16.5451201	21.6425524	11.7605452	14.4339583	20.5436478
Adamts8	9.46759208	0.63020309	0.4066842	1.54961292	0.12123445	0.91823128	Adamts8	5.37637063	2.71514231	4.13502745	14.4769801	8.3240586	17.6408179	4.81131944	18.2610202
Mplkip	8.59431937	0.59705329	0.38535838	1.54934554	0.12129868	0.91823128	Mplkip	5.37637063	5.43028463	6.99773876	6.20442003	4.99443516	7.05632714	14.4339583	18.2610202
Dlgap2	67.3562638	0.45081449	0.2909784	1.54930571	0.12130825	0.91823128	Dlgap2	68.5487255	61.5432258	52.8011197	35.1583802	116.53682	79.9717076	55.8113055	68.4788259
Actrt3	0.93880121	0.49868601	0.3220009	1.54871001	0.12145145	NA	Actrt3	0	0	0.31807903	0	1.66481172	0	0.96226389	4.56525506
Syngr2	3.5366593	-0.642586	0.41509694	-1.5480385	0.12161303	NA	Syngr2	2.01613899	9.95552182	3.81694841	4.13628002	1.66481172	1.17605452	0.96226389	4.56525506
Myl4	1.72615751	-0.6100178	0.39406241	-1.5480233	0.12161668	NA	Myl4	4.03227797	2.71514231	3.81694841	0	0	0	0.96226389	2.28262753
Slc29a4	17.146071	-0.5878164	0.37972088	-1.5480223	0.12161693	0.91985107	Slc29a4	10.0806949	30.7716129	15.9039517	31.0221002	19.9777406	7.05632714	8.660375	13.6957652
Snhg4	3.50544131	0.64406858	0.41612585	1.54777353	0.12167683	NA	Snhg4	2.01613899	1.35757116	2.86271131	2.06814001	1.66481172	3.52816357	7.69811111	6.84788259
Adcy2	64.2431986	-0.5573909	0.36014173	-1.5476988	0.12169482	0.91985107	Adcy2	59.1400769	138.019734	94.1513942	28.9539601	53.2739751	70.5632714	35.6037639	34.239413
Chmp7	71.3258699	0.41391778	0.26750219	1.54734352	0.12178043	0.91985107	Chmp7	57.1239379	42.9897533	55.663831	86.8618804	73.2517157	70.5632714	83.7169583	100.435611
Samd3	1.94055158	-0.6166212	0.39862914	-1.5468542	0.12189841	NA	Samd3	3.36023164	5.88280835	3.18079035	0	0	1.17605452	1.92452778	0
Gm5065	1.22693104	-0.5451884	0.35246444	-1.5467898	0.12191394	NA	Gm5065	2.01613899	5.88280835	0.9542371	0	0	0	0.96226389	0
Ccdc53	5.70691566	-0.6419134	0.41502437	-1.5466884	0.12193841	0.92033638	Ccdc53	8.73660227	9.5029981	6.67965972	8.27256004	1.66481172	1.17605452	9.62263889	0
Mmp14	5.33550123	-0.6320993	0.40882087	-1.5461521	0.12206783	0.92033638	Mmp14	8.73660227	4.97776091	6.04350166	10.3407001	4.99443516	4.7042181	2.88679167	0
Psmd7	83.7462625	0.56996959	0.36887423	1.54515971	0.12230764	0.92033638	Psmd7	66.5325865	29.4140417	46.439539	105.475141	83.240586	56.4506172	76.9811111	205.436478
Acot11	26.4527845	-0.5132853	0.33219654	-1.5451254	0.12231594	0.92033638	Acot11	32.2582238	35.2968501	23.5378486	37.2265202	34.9610461	15.2887088	12.5094306	20.5436478
Tmem173	0.78572353	-0.4997037	0.32354608	-1.5444591	0.12247716	NA	Tmem173	1.34409266	2.71514231	2.22655324	0	0	0	0	0
Plekhd1	2.48662104	0.63020261	0.40811384	1.54418337	0.12254392	NA	Plekhd1	0.67204633	1.81009488	1.59039517	0	1.66481172	1.17605452	3.84905555	9.13051012
Hnrnph3	14.4576241	0.56643261	0.36683418	1.54411078	0.1225615	0.92033638	Hnrnph3	14.1129729	10.8605693	9.224292	8.27256004	4.99443516	19.9929269	23.0943333	25.1089028
Sepw1	299.412434	-0.5511698	0.35716501	-1.5431799	0.12278714	0.92033638	Sepw1	444.222623	289.162656	270.685258	488.081042	266.369875	74.091435	165.509389	397.17719
Cdh4	49.9715494	0.52412877	0.33964923	1.54314722	0.12279506	0.92033638	Cdh4	34.9464091	54.75537	39.7598793	22.7495401	58.2684102	92.9083074	27.9056528	68.4788259
Slc4a8	111.958603	0.49309838	0.31958574	1.54292984	0.1228478	0.92033638	Slc4a8	55.1077989	121.72888	101.149133	76.5211804	144.83862	199.929269	73.1320555	123.261887
Ccdc22	8.11549764	0.62523676	0.40525027	1.54284108	0.12286934	0.92033638	Ccdc22	3.36023164	5.43028463	8.58813393	2.06814001	8.3240586	4.7042181	9.62263889	22.8262753
Usp2	51.5374141	-0.4045933	0.26230197	-1.5424713	0.12295911	0.92033638	Usp2	65.8605402	65.6159392	54.7095939	49.6353602	53.2739751	43.5140174	31.7547083	47.9351782
Mxd3	1.10795855	-0.5742739	0.37234639	-1.5423108	0.12299808	NA	Mxd3	1.34409266	2.26261859	2.22655324	2.06814001	0	0	0.96226389	0
Tab2	42.152529	-0.5797508	0.37608954	-1.5415233	0.12318947	0.92033638	Tab2	72.5810035	68.3310816	55.9819101	18.6132601	23.3073641	41.1619083	48.1131944	9.13051012
Trpm2	23.7670569	0.53121009	0.34463139	1.54138624	0.12322281	0.92033638	Trpm2	12.0968339	20.8160911	14.3135566	26.8858201	46.6147282	24.697145	17.32075	27.3915304
Snord64	1.10371658	0.55716957	0.36150088	1.54126756	0.12325168	NA	Snord64	0.67204633	0	0.63615807	0	0	2.35210905	2.88679167	2.28262753
Slc17a7	555.824642	-0.5599304	0.36329889	-1.5412389	0.12325866	0.92033638	Slc17a7	633.739688	1144.43249	705.499299	304.016582	780.796697	257.555941	234.792389	385.764053
Ptpn14	19.5940278	0.61214605	0.397226	1.54105232	0.12330405	0.92033638	Ptpn14	14.7850192	15.3858064	13.6773985	8.27256004	61.5980337	10.5844907	9.62263889	22.8262753
Zfyve20	53.8529129	0.37230771	0.24160217	1.54099492	0.12331802	0.92033638	Zfyve20	39.6507334	47.0624668	51.5288036	45.4990802	66.5924688	51.7463991	62.5471528	66.1961984
Itgb4	2.02804741	-0.5899284	0.38289336	-1.5407119	0.12338691	NA	Itgb4	0	5.43028463	3.81694841	4.13628002	1.66481172	1.17605452	0	0
Ndufa4l2	1.35417863	-0.5530962	0.35906343	-1.5403858	0.12346635	NA	Ndufa4l2	1.34409266	4.07271347	0.31807903	4.13628002	0	0	0.96226389	0
Kcnj12	17.7480331	0.54905737	0.35668507	1.53933377	0.12372283	0.92033638	Kcnj12	11.4247876	16.7433776	8.58813393	16.5451201	24.9721758	21.1689814	10.5849028	31.9567854
Pam	227.877625	0.53481061	0.34743034	1.5393319	0.12372329	0.92033638	Pam	133.737219	217.663909	219.474534	126.156541	149.833055	562.154063	203.999944	210.001733
Nfe2l1	254.223077	0.38162472	0.24798616	1.53889524	0.12382987	0.92033638	Nfe2l1	190.189111	233.954763	198.163238	246.108661	334.627156	317.534722	179.943347	333.26362
Nrsn2	131.642941	-0.4207564	0.27344987	-1.5386967	0.12387837	0.92033638	Nrsn2	104.839227	175.579203	134.547432	198.541441	109.877574	122.309671	75.0565833	132.392397
Irak1	27.7847415	-0.4289785	0.27880561	-1.5386293	0.12389481	0.92033638	Irak1	32.9302701	31.6766603	30.5355873	35.1583802	21.6425524	21.1689814	24.0565972	25.1089028
Pld6	2.25602967	0.61406657	0.39913777	1.53848276	0.12393062	NA	Pld6	0.67204633	0.90504744	0.63615807	2.06814001	4.99443516	0	1.92452778	6.84788259
Sox13	9.31233111	-0.6405436	0.41636292	-1.5384263	0.12394443	0.92033638	Sox13	8.06455594	8.59795066	6.04350166	31.0221002	13.3184938	0	2.88679167	4.56525506
Smad5	34.9528647	-0.4397798	0.28589448	-1.538259	0.1239853	0.92033638	Smad5	38.978687	38.0119924	44.2129858	45.4990802	28.3017992	38.8097993	29.8301805	15.9783927
Mex3a	6.74913333	0.6396152	0.41581145	1.53823373	0.12399148	0.92033638	Mex3a	3.36023164	1.35757116	2.54463228	8.27256004	8.3240586	1.17605452	3.84905555	25.1089028
Spidr	2.04007272	-0.6236266	0.40541845	-1.5382295	0.12399251	NA	Spidr	2.68818531	5.43028463	2.54463228	2.06814001	1.66481172	0	1.92452778	0
Ptar1	12.5875851	0.5921393	0.38495232	1.53821466	0.12399614	0.92033638	Ptar1	6.04841696	10.4080455	14.3135566	4.13628002	6.65924688	22.345036	23.0943333	13.6957652
Socs7	104.906139	0.46207474	0.30039781	1.53820943	0.12399742	0.92033638	Socs7	81.3176057	77.8340797	70.6135457	111.679561	176.470042	78.7956531	82.7546944	159.783927
BC049635	6.04647518	0.61130317	0.3976276	1.5373761	0.12420124	0.92069233	BC049635	6.04841696	4.52523719	4.77118552	0	8.3240586	8.23238167	9.62263889	6.84788259
Zeb2os	5.96882588	0.62907116	0.40920281	1.537309	0.12421766	0.92069233	Zeb2os	2.68818531	4.52523719	3.81694841	4.13628002	16.6481172	2.35210905	6.73584722	6.84788259
Hhex	0.78403871	-0.4979678	0.32407635	-1.5365755	0.12439729	NA	Hhex	2.68818531	1.35757116	2.22655324	0	0	0	0	0
Prkce	518.818481	0.31268668	0.20350022	1.53654223	0.12440545	0.92144521	Prkce	528.90046	487.368045	476.800473	337.106822	617.645148	537.456918	539.830041	625.439944
Psmd8	55.6913636	-0.4902475	0.31913534	-1.5361742	0.12449566	0.92147483	Psmd8	67.2046328	73.7613662	69.9773876	57.9079203	31.6314227	24.697145	76.9811111	43.3699231
Athl1	5.40707269	-0.629456	0.41011868	-1.5348143	0.12482946	0.9227856	Athl1	5.37637063	4.52523719	4.13502745	18.6132601	8.3240586	0	0	2.28262753
Jmjd6	12.73437	0.53464832	0.348362	1.53474925	0.12484543	0.9227856	Jmjd6	7.39250961	11.313093	9.54237104	10.3407001	9.98887032	14.1126543	16.3584861	22.8262753
Cers5	14.3588545	-0.5527962	0.36045815	-1.5335932	0.12512976	0.92313091	Cers5	12.0968339	23.5312334	24.8101647	10.3407001	11.653682	11.7605452	11.5471667	9.13051012
Kat6b	42.5894404	0.48286061	0.31487192	1.53351436	0.12514917	0.92313091	Kat6b	38.3066407	35.2968501	40.0779583	22.7495401	43.2851047	64.6829988	71.2075278	25.1089028
Hcn2	84.9458997	0.47832273	0.31214526	1.53237226	0.1254306	0.92313091	Hcn2	69.8928182	59.2806072	55.027673	88.9300204	119.866444	101.140689	46.1886666	139.240279
Mrpl32	7.2299226	-0.628907	0.41063825	-1.5315354	0.12563712	0.92313091	Mrpl32	14.7850192	8.14542694	11.1327662	6.20442003	0	4.7042181	10.5849028	2.28262753
P2rx6	1.84618848	-0.6081126	0.39735105	-1.5304165	0.12591366	NA	P2rx6	4.7043243	1.35757116	3.18079035	2.06814001	0	1.17605452	0	2.28262753
Bcat1	137.003051	0.46593911	0.30446422	1.53035753	0.12592826	0.92313091	Bcat1	73.9250961	81.0017457	108.146872	184.064461	141.508996	166.999742	121.24525	219.132243
Wdfy3	263.465623	0.42580347	0.27826406	1.53021368	0.12596385	0.92313091	Wdfy3	220.431196	230.334573	217.56606	208.882141	446.169541	376.337448	211.698055	196.305968
Atox1	14.6078944	-0.5265352	0.34412445	-1.5300722	0.12599886	0.92313091	Atox1	21.5054825	19.0059962	14.6316356	16.5451201	9.98887032	7.05632714	14.4339583	13.6957652
Ebf4	4.64483498	-0.6333145	0.41396385	-1.5298788	0.12604673	NA	Ebf4	7.39250961	4.52523719	4.45310648	10.3407001	4.99443516	3.52816357	1.92452778	0
Ndufs8	50.0580623	-0.4038488	0.26398887	-1.5297948	0.12606753	0.92313091	Ndufs8	61.1562159	54.3028463	50.8926455	66.1804803	34.9610461	31.7534722	50.9999861	50.2178057
Ubl7	58.7491034	-0.4724514	0.3089439	-1.5292466	0.12620332	0.92313091	Ubl7	59.8121232	59.2806072	64.570044	97.2025805	49.9443516	22.345036	52.9245139	63.9135709
Atp5b	570.556148	0.41296861	0.27006385	1.52915175	0.12622683	0.92313091	Atp5b	474.464708	333.962505	436.086356	672.145503	695.891299	478.654191	589.867764	883.376855
Fastkd3	9.07265713	-0.5952321	0.38925663	-1.5291507	0.12622708	0.92313091	Fastkd3	11.4247876	9.5029981	11.4508452	16.5451201	1.66481172	5.88027262	11.5471667	4.56525506
Pde10a	142.147098	-0.4363028	0.28532615	-1.5291372	0.12623043	0.92313091	Pde10a	225.13552	115.393548	218.838376	105.475141	111.542385	128.189943	111.622611	120.979259
Lrrc75a	6.308731	-0.6275814	0.4104506	-1.5290059	0.12626298	0.92313091	Lrrc75a	12.0968339	9.95552182	6.67965972	6.20442003	6.65924688	1.17605452	7.69811111	0
Tmem91	31.5633214	-0.5766477	0.37717502	-1.5288596	0.12629924	0.92313091	Tmem91	27.5538995	22.1736622	24.4920857	88.9300204	24.9721758	12.9365998	24.0565972	27.3915304
Lingo2	24.0534801	-0.5414124	0.35447973	-1.5273437	0.12667556	0.92313091	Lingo2	26.8818531	43.8948007	30.5355873	18.6132601	21.6425524	27.0492541	19.2452778	4.56525506

GeneID	Base mean	log2(FC)	StdErr	Wald-Stats	P-value	P-adj		GeneID	Normalised expression for Chow#1	Normalised expression for Chow#2	Normalised expression for Chow#3	Normalised expression for Chow#4	Normalised expression for HFD#1	Normalised expression for HFD#2	Normalised expression for HFD#3	Normalised expression for HFD#4
Itih3	6.07054684	-0.6355114	0.41619175	-1.5269679	0.126769	0.92313091		Itih3	1.34409266	13.5757116	9.54237104	10.3407001	4.99443516	5.88027262	2.88679167	0
Adi1	14.7700495	-0.5927516	0.38826968	-1.5266491	0.12684829	0.92313091		Adi1	17.4732045	18.5534725	21.9474534	20.6814001	3.32962344	11.7605452	22.1320694	2.28262753
Ebf2	2.27187908	-0.6252853	0.40964401	-1.5264114	0.12690744	NA		Ebf2	3.36023164	2.26261859	2.54463228	6.20442003	1.66481172	1.17605452	0.96226389	0
Nfkbil1	5.38597976	0.61744254	0.4045272	1.52633135	0.12692737	0.92313091		Nfkbil1	4.7043243	3.16766603	2.54463228	4.13628002	3.32962344	7.05632714	6.73584722	11.4131377
Fam57a	3.03948618	-0.6355062	0.41636647	-1.5263145	0.12693156	NA		Fam57a	5.37637063	3.62018975	4.13502745	4.13628002	1.66481172	1.17605452	1.92452778	2.28262753
Cdh20	6.68734121	-0.6189281	0.40551893	-1.526262	0.12694463	0.92313091		Cdh20	9.4086486	14.9332827	8.90621297	2.06814001	4.99443516	7.05632714	3.84905555	2.28262753
Gcc2	48.4527421	0.61842506	0.40521669	1.5261589	0.1269703	0.92313091		Gcc2	35.6184554	47.5149905	36.897168	8.27256004	23.3073641	119.957561	100.075444	15.9783927
Cckbr	20.7559031	-0.5505436	0.36093017	-1.5253464	0.12717271	0.92313091		Cckbr	14.7850192	40.274611	30.5355873	16.5451201	18.3129289	19.9929269	9.62263889	15.9783927
Sec11c	19.5936136	-0.5331528	0.34982491	-1.524056	0.12749471	0.92313091		Sec11c	25.5377605	32.129184	19.7209001	20.6814001	14.9833055	24.697145	14.4339583	4.56525506
Cadps	258.883302	0.32786071	0.21513185	1.52399895	0.12750897	0.92313091		Cadps	232.52803	198.205389	248.737805	231.631681	226.414394	364.576902	297.339542	271.632676
Ptma	102.133041	-0.4877701	0.32006445	-1.5239747	0.12751502	0.92313091		Ptma	118.280154	100.912789	115.46269	157.178641	68.2572805	34.1055812	108.735819	114.131377
Prodh	5.06963118	-0.6244552	0.40997349	-1.5231599	0.12771871	0.92313091		Prodh	7.39250961	4.52523719	8.2700549	8.27256004	3.32962344	5.88027262	2.88679167	0
Jakmip1	41.0810148	0.41299773	0.27118642	1.52292924	0.12777641	0.92313091		Jakmip1	43.6830113	28.961518	37.8514051	26.8858201	46.6147282	52.9224536	52.9245139	38.804668
Gm6277	1.49801067	-0.6013954	0.39491875	-1.5228332	0.12780044	NA		Gm6277	2.01613899	2.26261859	3.49886938	2.06814001	0	1.17605452	0.96226389	0
Keap1	50.9111392	0.59411694	0.39019323	1.52262239	0.12785321	0.92313091		Keap1	34.9464091	17.648425	20.0389792	72.3849004	83.240586	23.5210905	41.3773472	114.131377
Pvalb	39.0397288	0.54249533	0.35631503	1.52251597	0.12787985	0.92313091		Pvalb	30.9141311	14.9332827	24.8101647	49.6353602	26.6369875	32.9295267	54.8490416	77.6093361
Fyco1	17.5162062	0.50533003	0.33193035	1.52239779	0.12790944	0.92313091		Fyco1	19.4893435	14.480759	12.4050823	8.27256004	21.6425524	27.0492541	23.0943333	13.6957652
Tbc1d24	88.9752814	0.40438347	0.26564083	1.52229413	0.1279354	0.92313091		Tbc1d24	67.2046328	68.3310816	71.2497037	93.0663005	109.877574	96.436471	66.3962083	139.240279
Pcdhgc5	277.083746	-0.4853198	0.31893285	-1.5216991	0.1280845	0.92313091		Pcdhgc5	329.974747	482.842808	338.754172	177.860041	317.979039	250.499614	127.018833	191.740713
Gm14164	12.7274629	-0.5358944	0.35219661	-1.5215775	0.12811498	0.92313091		Gm14164	11.4247876	15.8383302	17.4943469	18.6132601	13.3184938	10.5844907	7.69811111	6.84788259
Klf9	104.301811	-0.3923207	0.25789978	-1.5212139	0.12820617	0.92313091		Klf9	133.737219	141.639924	122.778507	78.5893204	104.883138	65.8590533	93.3395972	93.5877288
Pex1	17.3941584	0.50467445	0.3317613	1.52119746	0.12821029	0.92313091		Pex1	12.0968339	16.7433776	16.8581888	6.20442003	24.9721758	21.1689814	18.2830139	22.8262753
Pola1	3.10712603	0.629727	0.41408388	1.52077158	0.12831717	NA		Pola1	1.34409266	2.71514231	2.22655324	0	0	8.23238167	5.77358333	4.56525506
Map6d1	26.0231029	-0.5290883	0.34807859	-1.5200254	0.12850458	0.92313091		Map6d1	36.2905017	35.2968501	35.3067728	22.7495401	8.3240586	27.0492541	31.7547083	11.4131377
Ndufaf6	3.83475112	0.63269009	0.41627751	1.51987575	0.12854221	NA		Ndufaf6	2.68818531	2.71514231	2.86271131	0	1.66481172	2.35210905	11.5471667	6.84788259
Strip2	17.3869624	-0.5920215	0.38952984	-1.519836	0.1285522	0.92313091		Strip2	33.6023164	21.2686148	30.5355873	4.13628002	9.98887032	21.1689814	11.5471667	6.84788259
Ankrd13a	23.6575017	-0.4958825	0.32628716	-1.5197732	0.12856798	0.92313091		Ankrd13a	26.2098068	28.961518	25.7644018	35.1583802	29.966611	19.9929269	16.3584861	6.84788259
Gorasp2	127.798893	-0.4099667	0.2699423	-1.5187197	0.12883307	0.92313091		Gorasp2	142.473822	131.231878	125.32314	194.405161	98.2238915	68.2111624	116.433931	146.088162
Syndig1	19.4831219	-0.4817686	0.31723509	-1.5186486	0.12885097	0.92313091		Syndig1	27.5538995	24.8888045	23.8559276	16.5451201	11.653682	19.9929269	15.3962222	15.9783927
Rps16	7.62358445	-0.6278352	0.4134792	-1.5184201	0.12890852	0.92313091		Rps16	10.0806949	2.26261859	12.0870033	18.6132601	3.32962344	2.35210905	7.69811111	4.56525506
Disp1	6.78302666	-0.6135997	0.40430804	-1.5176539	0.12910166	0.92313091		Disp1	9.4086486	9.5029981	4.77118552	12.4088401	1.66481172	3.52816357	3.84905555	9.13051012
Entpd5	14.901869	-0.545833	0.35966926	-1.5175971	0.12911599	0.92313091		Entpd5	14.1129729	28.5089943	18.130505	12.4088401	11.653682	15.2887088	7.69811111	11.4131377
Gm10532	10.3766517	0.54445435	0.35879987	1.51743187	0.12915768	0.92313091		Gm10532	6.72046328	7.2403795	6.67965972	12.4088401	13.3184938	11.7605452	13.4716944	11.4131377
Bin2	1.34941181	-0.5326957	0.3510812	-1.5173007	0.12919079	NA		Bin2	6.72046328	2.26261859	0.63615807	0	0	1.17605452	0	0
Psmd2	64.9380304	0.42244343	0.27844182	1.51716948	0.1292239	0.92313091		Psmd2	59.8121232	34.8443264	52.8011197	70.3167603	84.9053977	65.8590533	59.6603611	91.3051012
Rasl10b	131.163589	0.58095418	0.38293368	1.51711434	0.12923782	0.92313091		Rasl10b	79.9735131	61.5432258	61.0711746	175.791901	276.358746	79.9717076	63.5094166	251.089028
Dcaf7	357.048583	0.49755086	0.32798321	1.51700099	0.12926643	0.92313091		Dcaf7	212.36664	200.468007	235.696565	481.876622	604.326654	297.541795	269.433889	554.67849
Anxa3	0.83165425	-0.4781922	0.31525029	-1.5168651	0.12930074	NA		Anxa3	0.67204633	4.07271347	1.90847421	0	0	0	0	0
Gpatch4	9.34855581	0.61478897	0.40551883	1.51605532	0.12950537	0.92313091		Gpatch4	7.39250961	1.81009488	6.67965972	10.3407001	3.32962344	19.9929269	11.5471667	13.6957652
Brd3	75.9294117	0.40376162	0.2664373	1.5154095	0.12966873	0.92313091		Brd3	63.8444012	54.75537	55.345752	82.7256004	94.8942681	71.739326	65.4339444	118.696632
P4ha1	13.0007223	-0.6171793	0.40730149	-1.5152887	0.12969932	0.92313091		P4ha1	16.8011582	24.8888045	16.2220308	12.4088401	4.99443516	23.5210905	2.88679167	2.28262753
Plac9b	1.33468561	-0.5621199	0.37102998	-1.5150255	0.12976596	NA		Plac9b	2.68818531	3.62018975	0.63615807	2.06814001	1.66481172	0	0	0
Tfpt	9.7075866	0.56379065	0.37216081	1.51491138	0.12979485	0.92313091		Tfpt	10.0806949	7.2403795	5.08926455	6.20442003	6.65924688	12.9365998	13.4716944	15.9783927
Tmem181b-ps	13.6654643	0.52827458	0.34876718	1.51469121	0.12985062	0.92313091		Tmem181b-p	7.39250961	14.480759	11.4508452	8.27256004	16.6481172	22.345036	17.32075	11.4131377
Zswim8	97.0443501	0.57310021	0.37842173	1.5144485	0.12991213	0.92313091		Zswim8	65.1884939	48.4200379	49.6203294	119.952121	223.084771	57.6266717	48.1131944	164.349182
Abca1	21.3688017	-0.5314813	0.35094324	-1.5144367	0.12991513	0.92313091		Abca1	21.5054825	39.8220873	24.8101647	18.6132601	21.6425524	23.5210905	9.62263889	11.4131377
Traf3	39.6671831	0.50457066	0.33320523	1.51429394	0.12995131	0.92313091		Traf3	21.5054825	29.4140417	25.7644018	49.6353602	76.5813391	37.6337448	28.8679167	47.9351782
Snord22	0.98134067	0.48053522	0.31742355	1.51386127	0.13006103	NA		Snord22	0.67204633	0	0	0	3.32962344	0	3.84905555	0
Cadm4	113.527577	0.57974467	0.38297644	1.51378678	0.13007993	0.92313091		Cadm4	51.075521	73.3088425	56.2999891	146.837941	214.760712	91.7322529	41.3773472	232.828008
Trrap	114.562118	0.51749301	0.34192259	1.51348001	0.13015778	0.92313091		Trrap	78.6294204	82.8118406	82.700549	111.679561	251.38657	139.950488	52.9245139	116.414004
Pde7b	40.1827631	-0.4934039	0.32601036	-1.5134607	0.13016268	0.92313091		Pde7b	53.7637063	49.3250854	61.3892537	26.8858201	53.2739751	21.1689814	25.981125	29.6741579
Rmrp	54.3212984	0.62796844	0.41493284	1.51342186	0.13017254	0.92313091		Rmrp	22.8495752	16.2908539	18.130505	78.5893204	166.481172	5.88027262	69.283	57.0656883
Iqsec2	157.064853	0.51552931	0.34065662	1.51334008	0.1301933	0.92313091		Iqsec2	129.704941	105.89055	111.327662	140.633521	359.599332	103.492798	98.1509166	207.719105
Ddc	6.12506889	-0.6151551	0.40654947	-1.5131124	0.1302511	0.92313091		Ddc	3.36023164	2.26261859	1.90847421	31.0221002	4.99443516	3.52816357	1.92452778	0
Pcsk6	7.59255772	-0.6266195	0.41413307	-1.5130873	0.13025749	0.92313091		Pcsk6	3.36023164	10.8605693	12.7231614	14.4769801	11.653682	1.17605452	1.92452778	4.56525506
Slc16a9	1.75659346	-0.5941313	0.39296263	-1.5119284	0.13055209	NA		Slc16a9	0.67204633	4.07271347	2.54463228	4.13628002	1.66481172	0	0.96226389	0
Mprip	113.929938	0.43061459	0.28483467	1.51180543	0.13058337	0.92313091		Mprip	92.070347	80.549222	84.2909441	124.088401	203.10703	115.253343	129.905625	82.1745911
Uvssa	5.97364201	0.59927936	0.39644529	1.51163193	0.13062752	0.92313091		Uvssa	3.36023164	4.97776091	5.08926455	2.06814001	11.653682	7.05632714	6.73584722	6.84788259
Aldh1l1	20.6876869	-0.5843515	0.3866274	-1.5114074	0.13068469	0.92313091		Aldh1l1	34.2743627	19.4585199	16.5401098	35.1583802	23.3073641	5.88027262	5.77358333	25.1089028
Dclk2	77.5522593	0.41386766	0.27384956	1.51129567	0.13071314	0.92313091		Dclk2	59.1400769	56.1129411	60.1169375	84.7937404	99.8887032	69.3872169	65.4339444	125.544514
Ssu72	39.5928086	0.42413927	0.28067811	1.51112345	0.130757	0.92313091		Ssu72	39.6507334	32.129184	32.1259825	24.8176801	44.9499164	36.4576902	40.4150833	66.1961984
Ets1	7.30505402	0.60169587	0.39823093	1.51092199	0.13080833	0.92313091		Ets1	4.7043243	9.05047438	3.81694841	2.06814001	8.3240586	8.23238167	15.3962222	6.84788259
Zfp111	8.98108926	0.59768824	0.39560411	1.51082414	0.13083326	0.92313091		Zfp111	2.68818531	8.59795066	8.90621297	4.13628002	4.99443516	17.6408179	13.4716944	11.4131377
Lats1	41.7334974	-0.4817702	0.31889434	-1.5107517	0.13085173	0.92313091		Lats1	53.0916599	52.9452751	51.2107246	45.4990802	34.9610461	44.6900719	42.3396111	9.13051012
Kcnmb4	13.4397506	-0.5433495	0.35977054	-1.5102667	0.13097537	0.92331226		Kcnmb4	23.5216215	20.3635674	12.0870033	10.3407001	11.653682	11.7605452	8.660375	9.13051012
Lyrm9	35.1874968	0.44011141	0.29146965	1.50997339	0.13105021	0.92331226		Lyrm9	21.5054825	27.6039469	29.8994292	39.2946602	54.9387868	35.2816357	43.301875	29.6741579
Zfp330	6.36564304	0.61687883	0.40865192	1.50954591	0.13115933	0.9234723		Zfp330	3.36023164	3.16766603	5.08926455	6.20442003	1.66481172	8.23238167	16.3584861	6.84788259
Fgd5	3.49994427	0.62779399	0.41600951	1.50908568	0.13127689	NA		Fgd5	2.68818531	2.26261859	1.27231614	2.06814001	6.65924688	2.35210905	3.84905555	6.84788259
Kcnip1	54.6738411	0.49992197	0.33137282	1.50863903	0.13139105	0.92371194		Kcnip1	39.6507334	42.5372296	53.4372778	35.1583802	24.9721758	58.8027262	84.6792222	98.1529838
Zfand3	50.8887247	-0.3972187	0.26329616	-1.5086383	0.13139125	0.92371194		Zfand3	62.5003085	63.8058444	59.1627004	47.5672202	49.9443516	29.4013631	49.0754583	45.6525506
Add2	244.252749	0.35412923	0.23481961	1.50809054	0.13153136	0.92371194		Add2	188.845018	214.496243	199.435555	244.040521	374.582637	250.499614	205.924472	276.197931
Shank1	787.295197	0.58664323	0.38908559	1.50774854	0.1316189	0.92371194		Shank1	305.109033	491.893282	378.83213	1056.81955	1794.66703	505.703445	320.433875	1444.90323
Gars	91.2228057	0.37799646	0.25078642	1.50724452	0.13174799	0.92371194		Gars	87.3660227	72.403795	91.6067619	59.9760603	81.5757743	124.66178	123.169778	89.0224737
Gigyf2	38.8400719	0.4278109	0.28388552	1.50698387	0.13181479	0.92371194		Gigyf2	36.9625481	33.9392789	33.3982986	24.8176801	51.6091633	52.9224536	51.96225	25.1089028
Padi2	21.1113817	-0.5107938	0.33904329	-1.5065739	0.13191992	0.92371194		Padi2	30.2420848	25.3413283	21.9474534	24.8176801	29.966611	9.40843619	13.4716944	13.6957652
Chadl	6.74611872	-0.5963135	0.39588449	-1.5062816	0.1319949	0.92371194		Chadl	8.06455594	7.2403795	7.95197586	12.4088401	3.32962344	2.35210905	5.77358333	6.84788259
Rspo2	7.33974579	0.58086129	0.38566279	1.50613778	0.13203181	0.92371194		Rspo2	4.7043243	6.78785578	4.77118552	4.13628002	11.653682	11.7605452	5.77358333	9.13051012
Tnfaip8	3.60513262	-0.6258749	0.4156894	-1.505631	0.13216192	NA		Tnfaip8	6.72046328	7.2403795	2.86271131	4.13628002	4.99443516	0	2.88679167	0
Pafah1b1	504.053913	0.33749771	0.22417985	1.5054775	0.13220136	0.92371194		Pafah1b1	442.87853	412.249108	496.839452	411.559862	482.795399	663.294752	700.528111	422.286093
Clptm1l	51.0418559	-0.5088998	0.33803911	-1.5054464	0.13220936	0.92371194		Clptm1l	47.7152893	93.6724098	66.4785182	41.3628002	34.9610461	67.0351079	36.5660278	20.5436478
Wnk2	162.243761	0.4886642	0.32461527	1.50536419	0.13223047	0.92371194		Wnk2	127.016756	119.918786	95.1056313	173.723761	264.705064	131.718107	98.1509166	287.611069
Gpn2	5.79686655	0.6203151	0.41220234	1.50488012	0.1323549	0.92397725		Gpn2	2.68818531	2.71514231	4.45310648	4.13628002	9.98887032	3.52816357	2.88679167	15.9783927
Slc4a5	0.93150584	0.42706643	0.28381697	1.5047248	0.13239485	NA		Slc4a5	0	0	0	0	0	0	2.88679167	4.56525506
Gatsl3	2.0412179	-0.5848896	0.38874775	-1.5045479	0.13244035	NA		Gatsl3	0.67204633	3.16766603	1.59039517	8.27256004	1.66481172	0	0.96226389	0
Tor3a	3.26754137	-0.6235451	0.41446568	-1.5044552	0.1324642	NA		Tor3a	3.36023164	7.69290322	3.49886938	4.13628002	0	0	2.88679167	4.56525506
1700019G17Rik	5.6758789	-0.6164697	0.40982951	-1.5042102	0.13252726	0.92403688		1700019G17R	4.03227797	9.95552182	6.99773876	10.3407001	0	4.7042181	4.81131944	4.56525506
Dlg3	149.804464	0.34027054	0.22621741	1.50417485	0.13253635	0.92403688		Dlg3	159.947026	109.51074	129.458167	122.020261	201.442218	155.239197	140.490528	180.327575
Hacl1	1.56941078	-0.5884752	0.39126904	-1.5040168	0.13257705	NA		Hacl1	4.03227797	2.71514231	3.18079035	0	1.66481172	0	0.96226389	0
Fam124a	26.7819504	0.46257842	0.30765239	1.50357492	0.13269085	0.92423596		Fam124a	26.8818531	24.8888045	20.0389792	14.4769801	28.3017992	27.0492541	45.2264028	27.3915304
Zscan18	12.4908474	0.53073241	0.35302318	1.50339252	0.13273785	0.92423596		Zscan18	9.4086486	9.5029981	10.1785291	10.3407001	9.98887032	21.1689814	20.2075417	9.13051012
D330023K18Rik	1.47047001	-0.5648954	0.37594729	-1.502592	0.13294428	NA		D330023K18Ri	0.67204633	1.81009488	2.86271131	4.13628002	0	0	0	2.28262753
Nsdhl	12.0653399	-0.5682134	0.37816072	-1.5025713	0.13294963	0.92452758		Nsdhl	23.5216215	17.648425	11.7689243	8.27256004	13.3184938	5.88027262	11.5471667	4.56525506
Rab3gap2	69.3587981	0.42679551	0.28408709	1.50234036	0.13300923	0.92452758		Rab3gap2	63.8444012	47.0624668	68.0689134	51.7035003	68.2572805	124.66178	67.3584722	63.9135709
Pgk1	30.9021554	0.48868548	0.32531622	1.50218601	0.13304908	0.92452758		Pgk1	26.2098068	15.8383302	26.7186389	28.9539601	48.2795399	21.1689814	29.8301805	50.2178057
Tac1	27.7286973	0.62126126	0.41365321	1.50188913	0.13312574	0.92452758		Tac1	19.4893435	22.1736622	23.5378486	4.13628002	3.32962344	88.2040893	40.4150833	20.5436478
Tmem204	2.11281366	-0.6084814	0.40525005	-1.5014961	0.13322728	NA		Tmem204	2.01613899	5.43028463	3.18079035	2.06814001	0	0	1.92452778	2.28262753
Ctsa	37.6230801	-0.534572	0.35606361	-1.5013385	0.13326804	0.9247007		Ctsa	25.5377605	79.6441745	52.8011197	26.8858201	31.6314227	38.8097993	18.2830139	27.3915304
Hist1h1c	14.2304509	0.55208764	0.36778304	1.50112317	0.13332371	0.9247007		Hist1h1c	7.39250961	5.43028463	13.9954775	16.5451201	16.6481172	11.7605452	19.2452778	22.8262753
Ddx42	48.9235236	0.4213154	0.28075491	1.50065196	0.13344561	0.92494593		Ddx42	45.027104	31.6766603	43.5768277	45.4990802	43.2851047	72.9153805	68.3207361	41.0872956
Car14	1.90362169	-0.5651677	0.37663045	-1.5005894	0.1334618	NA		Car14	0	4.97776091	1.90847421	6.20442003	0	1.17605452	0.96226389	0
Tmem59	76.3870879	-0.4887262	0.32591455	-1.4995532	0.13373019	0.92617189		Tmem59	65.8605402	123.086452	126.277377	51.7035003	51.6091633	92.9083074	47.1509305	52.5004332
Ing1	17.354095	0.54901546	0.36623583	1.49907629	0.13385384	0.92617189		Ing1	19.4893435	11.313093	14.3135566	6.20442003	16.6481172	14.1126543	38.4905555	18.2610202
Nktr	24.2317941	0.49481956	0.33020352	1.49852904	0.13399585	0.92617189		Nktr	25.5377605	19.0059962	23.5378486	6.20442003	26.6369875	27.0492541	38.4905555	27.3915304
Nkap	16.3452484	0.50060203	0.33408635	1.49842107	0.13402389	0.92617189		Nkap	15.4570656	11.7656167	13.9954775	10.3407001	11.653682	22.345036	26.9433889	18.2610202
Rasal1	68.2394847	0.58902959	0.39314124	1.49826455	0.13406453	0.92617189		Rasal1	51.075521	28.0564706	36.579089	76.5211804	181.464478	27.0492541	35.6037639	109.566121
Gtf3c1	100.620875	0.45012899	0.30053778	1.49774511	0.13419949	0.92617189		Gtf3c1	79.3014668	64.2583681	76.9751264	109.611421	171.475607	115.253343	62.5471528	125.544514
Arhgef18	36.549593	0.44708553	0.29852843	1.49763132	0.13422907	0.92617189		Arhgef18	26.2098068	25.793852	26.4005599	43.4309402	58.2684102	42.3379629	28.8679167	41.0872956
Hs3st2	15.5994533	-0.5103647	0.3409908	-1.4967112	0.13446842	0.92722481		Hs3st2	23.5216215	20.8160911	15.9039517	16.5451201	6.65924688	16.4647633	13.4716944	11.4131377
Nek7	53.6893562	0.5824553	0.38949337	1.49541776	0.13480545	0.92825793		Nek7	43.6830113	41.1796584	47.0756971	20.6814001	18.3129289	99.9646345	128.943361	29.6741579
Myrip	39.3024503	0.40622456	0.27171924	1.49501579	0.13491032	0.92825793		Myrip	35.6184554	30.3190892	34.6706148	31.0221002	63.2628454	36.4576902	44.2641389	38.804668
Col6a3	1.21873132	-0.5614237	0.37556991	-1.494858	0.13495151	NA		Col6a3	1.34409266	1.81009488	2.86271131	2.06814001	1.66481172	0	0	0
Npepl1	5.00746885	-0.6200299	0.41483946	-1.4946262	0.13501202	0.92825793		Npepl1	7.39250961	7.2403795	4.13502745	10.3407001	9.98887032	0	0.96226389	0

GeneID	Base mean	log2(FC)	StdErr	Wald-Stats	P-value	P-adj		GeneID	Normalised expression for Chow#1	Normalised expression for Chow#2	Normalised expression for Chow#3	Normalised expression for Chow#4	Normalised expression for HFD#1	Normalised expression for HFD#2	Normalised expression for HFD#3	Normalised expression for HFD#4
Furin	35.4942585	0.43689251	0.29231524	1.49459369	0.13502051	0.92825793		Furin	23.5216215	36.6544212	25.1282437	31.0221002	54.9387868	35.2816357	31.7547083	45.6525506
Sec14l5	1.04333834	0.50926639	0.34092299	1.4937872	0.13523125	NA		Sec14l5	0	0	0.9542371	0	3.32962344	1.17605452	2.88679167	0
Bcl2l12	1.28160135	-0.4559818	0.3052775	-1.4936634	0.13526361	NA		Bcl2l12	1.34409266	0	0.63615807	8.27256004	0	0	0	0
5031426D15Rik	0.88674337	0.41790401	0.27982118	1.4934681	0.1353147	NA		5031426D15Ri	0	0	0	0	0	0	4.81131944	2.28262753
Slc39a1	19.3155042	-0.5631761	0.37728325	-1.4927142	0.13551201	0.92825793		Slc39a1	14.7850192	39.3695635	25.4463228	16.5451201	24.9721758	5.88027262	11.5471667	15.9783927
Trappc5	25.7410299	0.5230607	0.35046632	1.49247067	0.1355758	0.92825793		Trappc5	14.1129729	18.5534725	16.8581888	31.0221002	28.3017992	12.9365998	38.4905555	45.6525506
Ift22	17.8462965	-0.5091167	0.34119581	-1.492154	0.13565879	0.92825793		Ift22	26.2098068	16.7433776	17.8124259	28.9539601	14.9833055	12.9365998	18.2830139	6.84788259
Nipal4	0.81150118	-0.4720226	0.31634521	-1.4921122	0.13566973	NA		Nipal4	2.68818531	3.16766603	0.63615807	0	0	0	0	0
Pigk	61.4674837	0.54078847	0.36244793	1.49204456	0.13568746	0.92825793		Pigk	40.994826	61.9957495	48.9841713	33.0902402	34.9610461	161.11947	55.8113055	54.7830607
Emc6	34.3152873	-0.5439006	0.36462194	-1.4916837	0.13578209	0.92825793		Emc6	42.3389187	36.2018975	37.215247	53.7716403	18.3129289	9.40843619	20.2075417	57.0656883
Bach2	9.91983233	0.5843024	0.39170788	1.49167895	0.13578333	0.92825793		Bach2	2.01613899	6.33533207	9.224292	10.3407001	11.653682	10.5844907	8.660375	20.5436478
Pja2	470.634921	0.39637653	0.26575806	1.49149392	0.13583187	0.92825793		Pja2	466.400152	372.427021	457.715731	295.744021	446.169541	758.555168	593.716819	374.350915
Mxi1	35.5560172	-0.4732384	0.31750681	-1.4904828	0.13609733	0.92825793		Mxi1	53.7637063	46.6099431	39.7598793	31.0221002	14.9833055	34.1055812	41.3773472	22.8262753
Rbm33	42.4048311	0.41610228	0.27918212	1.49043311	0.13611039	0.92825793		Rbm33	34.2743627	39.3695635	38.8056422	28.9539601	49.9443516	69.3872169	44.2641389	34.239413
Sfrp1	4.41664207	0.61420304	0.41214715	1.49025181	0.13615804	NA		Sfrp1	2.68818531	2.26261859	3.81694841	2.06814001	9.98887032	7.05632714	2.88679167	4.56525506
Eml4	21.8651569	0.47459156	0.31847736	1.49018934	0.13617446	0.92825793		Eml4	20.1613899	22.1736622	18.130505	8.27256004	23.3073641	30.5774176	31.7547083	20.5436478
Sep-03	480.799645	0.38896973	0.26102473	1.49016431	0.13618104	0.92825793		Sep-03	385.754593	351.158406	344.161515	554.261523	567.700797	416.323302	453.226291	773.810733
Akr7a5	16.4931749	-0.5842881	0.39214688	-1.4899726	0.13623145	0.92825793		Akr7a5	18.1452509	18.1009488	14.3135566	35.1583802	13.3184938	1.17605452	13.4716944	18.2610202
Ppat	13.6015593	0.50645536	0.3399103	1.48996768	0.13623273	0.92825793		Ppat	10.7527413	9.05047438	13.9954775	8.27256004	14.9833055	18.8168724	19.2452778	13.6957652
Dnm3	330.951526	0.39440221	0.26484364	1.48918891	0.13643763	0.92825793		Dnm3	313.845635	227.619431	329.847959	250.244941	271.36431	563.330117	369.509333	321.850482
Sh3bp5	27.1491966	0.47528285	0.31916564	1.48914165	0.13645007	0.92825793		Sh3bp5	32.9302701	18.5534725	23.5378486	10.3407001	26.6369875	37.6337448	35.6037639	31.9567854
Adra1a	25.5903453	0.48077724	0.32287592	1.48904645	0.13647514	0.92825793		Adra1a	18.1452509	25.3413283	21.9474534	14.4769801	26.6369875	44.6900719	19.2452778	34.239413
Uri1	29.2481598	0.51807345	0.3479861	1.48877629	0.13654629	0.92825793		Uri1	23.5216215	21.2686148	35.6248519	8.27256004	26.6369875	43.5140174	50.0377222	25.1089028
Flywch1	158.469542	0.45397603	0.30496403	1.48862153	0.13658706	0.92825793		Flywch1	139.785636	76.4765085	98.6045007	204.745861	213.0959	144.654706	152.999958	237.393263
Gm6793	5.84609039	0.59605517	0.40053382	1.48815191	0.13671084	0.92825793		Gm6793	6.04841696	3.62018975	4.13502745	2.06814001	6.65924688	12.9365998	6.73584722	4.56525506
Nnat	188.262199	-0.4492788	0.30194343	-1.4879569	0.13676226	0.92825793		Nnat	165.323397	202.278102	173.353074	351.583802	173.140419	88.2040893	174.169764	178.044947
Fut10	5.87178472	-0.6194807	0.416368	-1.4878202	0.13679832	0.92825793		Fut10	7.39250961	16.7433776	8.58813393	0	6.65924688	4.7042181	2.88679167	0
Cd274	1.59748866	-0.5890109	0.39596438	-1.487535	0.13687358	NA		Cd274	2.01613899	2.26261859	2.22655324	4.13628002	0	1.17605452	0.96226389	0
Gm4477	1.04657777	-0.5352956	0.359881	-1.487424	0.13690287	NA		Gm4477	2.01613899	3.16766603	2.22655324	0	0	0	0.96226389	0
Clip1	74.621127	0.39250732	0.2638865	1.48740965	0.13690665	0.92825793		Clip1	77.2853278	58.3755597	61.0711746	57.9079203	63.2628454	107.020962	105.849028	66.1961984
Grin3a	84.494196	0.49570302	0.33341347	1.48675164	0.13708042	0.92825793		Grin3a	42.3389187	85.5269829	78.5655215	59.9760603	91.5646446	183.464506	68.3207361	66.1961984
Aarsd1	29.2216262	0.56768144	0.38191646	1.48640214	0.13717278	0.92825793		Aarsd1	17.4732045	7.69290322	16.5401098	45.4990802	33.2962344	24.697145	26.9433889	61.6309433
Mageh1	10.7267331	0.55838373	0.3756775	1.48633795	0.13718975	0.92825793		Mageh1	8.06455594	12.2181404	6.36158069	4.13628002	6.65924688	14.1126543	18.2830139	15.9783927
Hivep2	184.451541	0.45277439	0.30471439	1.4858976	0.13730621	0.92825793		Hivep2	207.662315	148.42778	172.716916	70.3167603	258.045817	258.731995	213.622583	146.088162
Npm1	25.797858	0.44370268	0.29862388	1.48582453	0.13732554	0.92825793		Npm1	20.8334362	19.9110436	25.1282437	18.6132601	24.9721758	25.8731995	41.3773472	29.6741579
Ankrd16	4.65528757	0.6120391	0.41199419	1.48555275	0.13739746	0.92825793		Ankrd16	2.01613899	1.81009488	3.18079035	6.20442003	4.99443516	3.52816357	8.660375	6.84788259
Mycn	6.27755215	0.58546785	0.39431658	1.48476602	0.13760582	0.92907832		Mycn	3.36023164	5.88280835	4.13502745	4.13628002	8.3240586	11.7605452	5.77358333	6.84788259
Spcs1	23.727714	-0.4812939	0.32427617	-1.48421	0.13775323	0.92948643		Spcs1	17.4732045	35.7493738	35.6248519	24.8176801	19.9777406	19.9929269	20.2075417	15.9783927
Tmem63b	72.1897206	-0.46798	0.31543435	-1.4836049	0.13791376	0.92966419		Tmem63b	79.9735131	132.136926	75.3847312	55.8397803	74.9165274	76.4435441	39.4528194	43.3699231
Cdk5rap3	10.5736524	0.60195773	0.40586227	1.48315766	0.13803253	0.92966419		Cdk5rap3	3.36023164	4.52523719	4.13502745	18.6132601	19.9777406	7.05632714	8.660375	18.2610202
Ankrd42	11.3810483	0.55521396	0.37435347	1.48312757	0.13804052	0.92966419		Ankrd42	13.4409266	6.33533207	10.4966081	2.06814001	11.653682	14.1126543	19.2452778	13.6957652
Plekhf1	1.11861543	-0.540883	0.3648584	-1.4824463	0.13822159	NA		Plekhf1	1.34409266	3.62018975	0.9542371	2.06814001	0	0	0.96226389	0
Lamp3	3.81047689	0.61681279	0.4163626	1.48143178	0.13849155	NA		Lamp3	4.7043243	1.35757116	2.54463228	0	1.66481172	4.7042181	8.660375	6.84788259
Tnrc18	143.213057	0.58427293	0.39442518	1.48132768	0.13851928	0.93110888		Tnrc18	62.5003085	82.8118406	67.7508343	190.268881	317.979039	83.4998712	50.9999861	289.893696
Fign	14.4687213	0.52985362	0.357699	1.48128349	0.13853105	0.93110888		Fign	6.72046328	9.5029981	14.3135566	14.4769801	19.9777406	25.8731995	13.4716944	11.4131377
Ppp1r16b	111.34384	0.32029019	0.21626088	1.48103622	0.13859692	0.93110888		Ppp1r16b	91.3983007	108.153169	92.24292	99.2707205	144.83862	124.66178	100.075444	130.109769
Chgb	119.190034	0.55388961	0.37406993	1.4807114	0.13868349	0.93110888		Chgb	97.4467176	112.225882	96.6960265	45.4990802	73.2517157	333.999485	114.509403	79.8919636
Gm5424	5.53817562	0.61650454	0.4163645	1.48068468	0.13869062	0.93110888		Gm5424	4.03227797	2.71514231	0.63615807	6.20442003	8.3240586	3.52816357	2.88679167	15.9783927
Syne3	1.7009239	0.58206553	0.39326787	1.48007397	0.13885351	NA		Syne3	1.34409266	0.90504744	0.63615807	0	6.65924688	1.17605452	2.88679167	0
Ccdc77	0.74427883	-0.4748173	0.32081201	-1.4800483	0.13886036	NA		Ccdc77	2.68818531	1.35757116	1.90847421	0	0	0	0	0
Slc4a3	103.488269	0.54440416	0.36784416	1.47998585	0.13887702	0.93172588		Slc4a3	49.059382	73.3088425	58.2084633	130.292821	176.470042	109.373071	39.4528194	191.740713
Pappa2	5.45791022	-0.6119965	0.41366183	-1.4794608	0.13901721	0.93172588		Pappa2	8.73660227	16.7433776	6.36158069	0	1.66481172	8.23238167	1.92452778	0
1110057K04Rik	21.7964886	-0.4815713	0.32552671	-1.4793603	0.13904405	0.93172588		1110057K04Ri	30.2420848	23.9837571	29.8994292	20.6814001	8.3240586	24.697145	18.2830139	18.2610202
Kcnh4	4.51776901	-0.6096005	0.41222393	-1.4788091	0.13919135	NA		Kcnh4	5.37637063	6.33533207	7.63389683	4.13628002	4.99443516	1.17605452	1.92452778	4.56525506
Pkp4	212.981615	0.34274293	0.23177704	1.47876137	0.1392041	0.93221394		Pkp4	218.415057	194.132675	194.34629	132.360961	208.101465	303.422067	227.094278	225.980126
Srd5a3	9.26080121	-0.5716649	0.38695325	-1.4773486	0.13958221	0.93374842		Srd5a3	7.39250961	15.8383302	9.224292	16.5451201	6.65924688	9.40843619	6.73584722	2.28262753
Zbtb21	13.557483	-0.5136338	0.34775474	-1.4770001	0.1396756	0.93374842		Zbtb21	19.4893435	19.0059962	16.5401098	10.3407001	13.3184938	12.9365998	7.69811111	9.13051012
Dag1	60.3695825	-0.523705	0.35459111	-1.4769265	0.13969533	0.93374842		Dag1	68.5487255	122.181404	70.9316247	35.1583802	56.6035985	72.9153805	26.9433889	29.6741579
Paqr5	1.47949468	-0.4849566	0.32844862	-1.4765068	0.13980789	NA		Paqr5	0	7.69290322	3.18079035	0	0	0	0.96226389	0
Nolc1	30.8912362	0.44902461	0.304203	1.47606896	0.13992538	0.93433511		Nolc1	25.5377605	21.7211385	26.4005599	28.9539601	26.6369875	56.4506172	31.7547083	29.6741579
St6gal1	13.3595389	-0.536505	0.36391073	-1.4742763	0.14040722	0.93433511		St6gal1	16.1291119	23.9837571	16.8581888	8.27256004	11.653682	14.1126543	6.73584722	9.13051012
Cxxc4	39.3677718	0.44513583	0.30197176	1.47409754	0.14045533	0.93433511		Cxxc4	26.2098068	29.4140417	35.3067728	41.3628002	38.2906696	50.5703445	66.3962083	27.3915304
Magee1	155.051453	0.43711968	0.29656403	1.47394705	0.14049585	0.93433511		Magee1	114.919922	91.4097912	107.192635	200.609581	256.381005	150.534979	129.905625	189.458085
Elof1	37.5257287	-0.4841061	0.32848623	-1.4737485	0.14054932	0.93433511		Elof1	51.075521	41.1796584	31.4898244	55.8397803	26.6369875	16.4647633	25.0188611	52.5004332
Mars	24.6875668	0.535688	0.36349503	1.47371479	0.1405584	0.93433511		Mars	20.8334362	24.8888045	21.3112953	4.13628002	46.6147282	25.8731995	17.32075	36.5220405
Ano8	15.698446	0.58899136	0.3996666	1.47370672	0.14056057	0.93433511		Ano8	8.73660227	10.8605693	7.95197586	16.5451201	33.2962344	27.0492541	2.88679167	18.2610202
Cacng4	15.5715718	-0.5393321	0.36597194	-1.4736979	0.14056296	0.93433511		Cacng4	12.7688802	25.3413283	12.7231614	26.8858201	9.98887032	7.05632714	11.5471667	18.2610202
Slc35a4	40.9494145	-0.5032023	0.34146146	-1.4736722	0.14056988	0.93433511		Slc35a4	47.7152893	60.6381783	40.3960374	47.5672202	33.2962344	19.9929269	16.3584861	61.6309433
Cdc26	4.97414811	-0.6069669	0.41203	-1.4731134	0.14072045	0.93450448		Cdc26	5.37637063	7.2403795	9.86045007	4.13628002	1.66481172	1.17605452	5.77358333	4.56525506
Crk	115.854207	0.36340143	0.24672029	1.47292882	0.14077023	0.93450448		Crk	112.231737	105.89055	111.009583	68.2486203	106.54795	152.887088	155.88675	114.131377
Heatr2	2.84697826	-0.6104021	0.41457853	-1.4723437	0.14092809	NA		Heatr2	5.37637063	1.81009488	5.72542262	4.13628002	1.66481172	1.17605452	2.88679167	0
Atp6v1f	75.7447939	-0.4723998	0.32088335	-1.4721855	0.1409708	0.93479314		Atp6v1f	86.6939764	64.2583681	69.6593086	142.701661	66.5924688	34.1055812	52.9245139	89.0224737
Nap1l2	82.809192	0.42570298	0.28920998	1.47195121	0.14103405	0.93479314		Nap1l2	84.0057911	56.5654649	64.251965	72.3849004	54.9387868	131.718107	116.433931	82.1745911
Abi1	83.3861379	-0.3874967	0.2633155	-1.4716062	0.14112725	0.93479314		Abi1	102.823088	106.343074	104.329923	70.3167603	56.6035985	68.2111624	99.1131805	59.3483158
Kmt2d	246.636583	0.52638473	0.35772668	1.47147183	0.14116356	0.93479314		Kmt2d	120.296293	164.718634	130.730483	339.174962	477.800964	210.51376	139.528264	390.329308
Hnrnpk	60.4219389	0.41955751	0.28522478	1.47097148	0.14129883	0.93510953		Hnrnpk	43.010965	41.6321821	45.803381	72.3849004	76.5813391	49.39429	58.6980972	95.8703563
Gm12070	79.6809514	0.43619729	0.29665063	1.4704074	0.14145145	0.93554027		Gm12070	65.8605402	54.3028463	61.3892537	80.6574604	126.525691	63.5069443	59.6603611	125.544514
Nek11	1.2029505	-0.5389965	0.36657661	-1.4703515	0.14146658	NA		Nek11	0.67204633	2.26261859	1.59039517	4.13628002	0	0	0.96226389	0
Prkacb	809.554436	0.2656845	0.18076844	1.46975041	0.14162936	0.93613769		Prkacb	717.073432	656.159392	776.112844	777.620644	724.193098	971.421037	899.716736	954.138308
Tnfrsf19	20.6542929	-0.5403295	0.36784081	-1.4689221	0.14185392	0.93704244		Tnfrsf19	30.9141311	36.6544212	25.1282437	8.27256004	18.3129289	19.9929269	7.69811111	18.2610202
Clip3	864.811774	-0.3018568	0.20554926	-1.4685377	0.14195821	0.93715217		Clip3	1115.59691	931.293814	978.41111	814.847164	792.450379	599.787807	770.773375	915.33364
Cfdp1	58.9378793	0.50714193	0.34543044	1.46814485	0.14206488	0.93727743		Cfdp1	50.4034746	45.7048956	51.2107246	37.2265202	34.9610461	82.3238167	130.867889	38.804668
Prok2	2.12203738	0.5684709	0.38746469	1.46715537	0.1423338	NA		Prok2	0.67204633	0.45252372	0.63615807	2.06814001	1.66481172	2.35210905	0	9.13051012
Msrb1	9.42001497	-0.5534182	0.37737441	-1.4664964	0.14251311	0.93885898		Msrb1	9.4086486	15.3858064	10.1785291	12.4088401	6.65924688	3.52816357	8.660375	9.13051012
Cflar	9.08416197	-0.5491701	0.3745656	-1.4661521	0.14260685	0.93885898		Cflar	10.0806949	14.480759	10.8146872	10.3407001	9.98887032	4.7042181	7.69811111	4.56525506
Rnf185	26.1165964	0.45800485	0.31240772	1.46604846	0.14263509	0.93885898		Rnf185	15.4570656	19.9110436	22.2655324	26.8858201	39.9554813	23.5210905	22.1320694	38.804668
Dnajc5	357.07092	0.31309304	0.21357371	1.46597181	0.14265597	0.93885898		Dnajc5	281.587412	282.827324	295.177344	405.355442	446.169541	351.640303	346.415	447.394996
Tmem50b	67.2322707	-0.4450389	0.30372516	-1.4652685	0.14284768	0.93916383		Tmem50b	52.4196136	113.583453	84.6090232	66.1804803	36.6258578	74.091435	50.9999861	59.3483158
Npas4	6.57422888	-0.5904698	0.40306455	-1.4649509	0.14293432	0.93916383		Npas4	5.37637063	6.78785578	8.58813393	14.4769801	4.99443516	5.88027262	1.92452778	4.56525506
Ppp1r3f	34.7474336	0.58995149	0.40283963	1.46448227	0.14306222	0.93916383		Ppp1r3f	16.8011582	9.95552182	14.3135566	55.8397803	53.2739751	24.697145	16.3584861	86.7398462
C3ar1	0.79189437	-0.455838	0.31159489	-1.4629189	0.14348958	NA		C3ar1	0.67204633	4.07271347	1.59039517	0	0	0	0	0
Rnf112	66.1209085	0.52861963	0.36135485	1.46288233	0.14349958	0.93916383		Rnf112	57.7959842	32.129184	33.7163777	78.5893204	134.849749	35.2816357	53.8867778	102.718239
Rer1	27.4612307	-0.4747735	0.32455327	-1.4628524	0.14350776	0.93916383		Rer1	24.1936678	47.9675142	37.5333261	20.6814001	24.9721758	25.8731995	20.2075417	18.2610202
Cnih1	44.3660459	-0.4634921	0.31690674	-1.4625506	0.14359038	0.93916383		Cnih1	41.6668724	85.0744592	50.5745665	33.0902402	26.6369875	39.9858538	41.3773472	36.5220405
Zc3h7a	10.161851	0.54690981	0.37414113	1.4617741	0.14380312	0.93916383		Zc3h7a	7.39250961	9.95552182	6.99773876	6.20442003	13.3184938	9.40843619	21.1698055	6.84788259
Tnrc6c	61.6654803	0.38879671	0.26601302	1.46157021	0.14385902	0.93916383		Tnrc6c	59.1400769	52.9452751	49.3022503	47.5672202	98.2238915	78.7956531	54.8490416	52.5004332
Dennd2d	0.83588861	0.43991216	0.30099448	1.46152898	0.14387033	NA		Dennd2d	0	0	0.31807903	0	1.66481172	4.7042181	0	0
Tmem126b	14.9178427	0.58824458	0.40259509	1.46113202	0.14397921	0.93916383		Tmem126b	9.4086486	12.2181404	16.5401098	2.06814001	8.3240586	34.1055812	29.8301805	6.84788259
Fam221b	1.05139308	-0.5392735	0.36909286	-1.4610782	0.14399399	NA		Fam221b	1.34409266	1.81009488	2.22655324	2.06814001	0	0	0.96226389	0
Lamp2	32.9982683	-0.5726411	0.39197348	-1.460918	0.14403796	0.93916383		Lamp2	28.2259458	70.5937002	55.027673	16.5451201	11.653682	45.8661264	26.9433889	9.13051012
Dcaf15	9.8053951	0.56786912	0.38881444	1.46051446	0.14414874	0.93916383		Dcaf15	3.36023164	7.2403795	6.04350166	12.4088401	19.9777406	7.05632714	8.660375	13.6957652
Gpx8	0.80869996	-0.4523499	0.3098082	-1.4600966	0.14426352	NA		Gpx8	0.67204633	4.52523719	1.27231614	0	0	0	0	0
Thy1	318.770385	-0.4226319	0.28946934	-1.4600231	0.14428372	0.93916383		Thy1	385.754593	532.167893	372.470549	196.473301	248.056946	324.591049	203.03768	287.611069
Oasl2	1.09010718	-0.5263702	0.36053404	-1.4599738	0.14429728	NA		Oasl2	2.01613899	3.62018975	1.90847421	0	0	1.17605452	0	0
9530068E07Rik	41.8263919	-0.5052767	0.34618275	-1.4595664	0.14440927	0.93916383		9530068E07Ri	36.9625481	76.0239848	63.6158069	26.8858201	24.9721758	54.0985081	26.9433889	25.1089028
Cds1	47.8659155	0.47588444	0.32607671	1.45942484	0.14444821	0.93916383		Cds1	32.9302701	50.2301328	50.5745665	16.5451201	58.2684102	83.4998712	45.2264028	45.6525506
Mok	4.89522683	-0.6066489	0.41571368	-1.4592951	0.1444839	0.93916383		Mok	3.36023164	6.33533207	4.77118552	14.4769801	1.66481172	4.7042181	3.84905555	0
Kcnab3	39.8256328	0.55347354	0.3793191	1.45912386	0.14453102	0.93916383		Kcnab3	22.8495752	13.1231878	24.8101647	57.9079203	38.2906696	38.8097993	26.9433889	95.8703563
Plcg1	36.4084618	0.42071265	0.28836594	1.45895401	0.14457777	0.93916383		Plcg1	30.9141311	22.1736622	30.2175083	39.2946602	53.2739751	43.5140174	30.7924444	41.0872956
Dnm1	976.432434	0.4132928	0.28328821	1.45891285	0.1445891	0.93916383		Dnm1	864.923625	636.700873	783.428662	980.298365	1539.95084	738.562241	786.169597	1481.42527
Rcl1	12.7405281	0.5626554	0.38569652	1.45880341	0.14461922	0.93916383		Rcl1	10.0806949	8.14542694	12.0870033	4.13628002	8.3240586	15.2887088	9.62263889	34.239413
R3hcc1l	10.2573351	0.54537395	0.37385179	1.45879723	0.14462093	0.93916383		R3hcc1l	11.4247876	7.2403795	7.63389683	4.13628002	14.9833055	17.6408179	14.4339583	4.56525506

GeneID	Base mean	log2(FC)	StdErr	Wald-Stats	P-value	P-adj	GeneID	Normalised expression for Chow#1	Normalised expression for Chow#2	Normalised expression for Chow#3	Normalised expression for Chow#4	Normalised expression for HFD#1	Normalised expression for HFD#2	Normalised expression for HFD#3	Normalised expression for HFD#4
4932438H23Rik	1.22669192	-0.516215	0.35387174	-1.4587631	0.14463032	NA	4932438H23Ri	5.37637063	0.45252372	0.9542371	2.06814001	0	0	0.96226389	0
Jmjd7-pla2g4b	3.16273111	0.59881803	0.41056281	1.45852965	0.14469461	NA	Jmjd7-pla2g4	0.67204633	2.26261859	0.31807903	4.13628002	3.32962344	3.52816357	1.92452778	9.13051012
Ttc1	20.6916236	0.46100274	0.31610408	1.4583891	0.14473332	0.93916383	Ttc1	17.4732045	17.1959013	18.130505	14.4769801	19.9777406	21.1689814	36.5660278	20.5436478
4930402H24Rik	42.3428125	-0.3948397	0.27075477	-1.4582927	0.14475987	0.93916383	4930402H24Ri	40.994826	53.3977988	55.663831	43.4309402	46.6147282	29.4013631	32.7169722	36.5220405
Dgkd	92.001072	0.35131177	0.24090901	1.45827577	0.14476455	0.93916383	Dgkd	79.9735131	77.8340797	70.6135457	90.9981605	128.190502	107.020962	74.0943194	107.283494
Ift20	17.685039	-0.5267537	0.36127089	-1.4580575	0.1448247	0.93916383	Ift20	28.8979921	20.3635674	21.3112953	18.6132601	4.99443516	14.1126543	24.0565972	9.13051012
1190007I07Rik	3.73197095	-0.6061059	0.41573481	-1.4579148	0.14486404	NA	1190007I07Ri	6.04841696	1.81009488	4.13502745	10.3407001	0	2.35210905	2.88679167	2.28262753
Srrt	47.3863043	0.38359363	0.26317614	1.45755471	0.14496332	0.93916383	Srrt	40.994826	39.3695635	33.7163777	47.5672202	53.2739751	55.2745626	40.4150833	68.4788259
Gon4l	45.585714	0.41532898	0.28496589	1.45746911	0.14498693	0.93916383	Gon4l	38.978687	40.7271347	34.3525357	37.2265202	76.5813391	52.9224536	33.6792361	50.2178057
Trib1	9.02366597	-0.5853549	0.40175828	-1.4569827	0.14512116	0.93946391	Trib1	10.7527413	11.313093	6.99773876	18.6132601	13.3184938	4.7042181	1.92452778	4.56525506
Ccdc85b	57.1910811	0.596844	0.40976276	1.45655987	0.14523791	0.93963253	Ccdc85b	20.1613899	16.2908539	29.2632712	86.8618804	91.5646446	17.6408179	33.6792361	162.066555
AF357359	4.3976091	0.60630193	0.41636948	1.45616324	0.1453475	NA	AF357359	2.01613899	4.52523719	2.22655324	2.06814001	0	8.23238167	11.5471667	4.56525506
Taf9b	28.5355724	-0.5165526	0.35481323	-1.4558436	0.14543587	0.93963253	Taf9b	30.2420848	40.7271347	40.0779583	31.0221002	6.65924688	34.1055812	31.7547083	13.6957652
Pdpn	3.63872248	-0.6007996	0.4127168	-1.4557187	0.14547039	NA	Pdpn	8.73660227	7.69290322	5.08926455	0	0	4.7042181	2.88679167	0
Smug1	9.89797631	-0.5796879	0.39821785	-1.4557055	0.14547404	0.93963253	Smug1	14.1129729	9.95552182	9.86045007	16.5451201	9.98887032	1.17605452	3.84905555	13.6957652
Ndufb11	43.9948853	-0.5200372	0.35726268	-1.4556158	0.14549886	0.93963253	Ndufb11	36.2905017	53.3977988	36.579089	90.9981605	28.3017992	15.2887088	31.7547083	59.3483158
Cep164	5.47922129	0.59102061	0.40620096	1.45499562	0.14567047	0.94017272	Cep164	3.36023164	2.71514231	2.54463228	8.27256004	6.65924688	7.05632714	8.660375	4.56525506
Lepr	1.01745263	-0.5230248	0.35953428	-1.4547286	0.1457444	NA	Lepr	2.68818531	2.26261859	2.22655324	0	0	0	0.96226389	0
Itga2b	2.92357169	0.56166066	0.38615204	1.45450651	0.14580593	NA	Itga2b	0.67204633	0.45252372	1.59039517	2.06814001	1.66481172	0	0.96226389	15.9783927
Ovol2	1.20126569	-0.534855	0.36775267	-1.4543879	0.14583879	NA	Ovol2	2.01613899	0.90504744	1.59039517	4.13628002	0	0	0.96226389	0
Pcbd2	4.05807659	-0.6054287	0.41631102	-1.4542703	0.14587138	NA	Pcbd2	8.73660227	3.62018975	2.86271131	8.27256004	1.66481172	1.17605452	3.84905555	2.28262753
Ipo13	65.5951952	-0.4336604	0.29843234	-1.4531281	0.14618819	0.94253237	Ipo13	68.5487255	70.5937002	62.9796488	105.475141	64.9276571	39.9858538	34.6415	77.6093361
Ddrgk1	13.6019668	0.54391545	0.37432912	1.45304071	0.14621245	0.94253237	Ddrgk1	8.06455594	8.14542694	7.31581779	18.6132601	14.9833055	21.1689814	7.69811111	22.8262753
Hpgd	1.58851659	-0.565235	0.38928085	-1.4519981	0.14650215	NA	Hpgd	0.67204633	2.26261859	3.49886938	4.13628002	0	1.17605452	0.96226389	0
Aph1c	8.23820071	0.56636992	0.390065	1.45198857	0.14650478	0.94286416	Aph1c	3.36023164	9.5029981	6.99773876	2.06814001	13.3184938	10.5844907	8.660375	11.4131377
Lrrc8b	39.5784005	-0.5145909	0.35440991	-1.4519653	0.14651127	0.94286416	Lrrc8b	45.6991503	75.5714611	50.2564875	22.7495401	24.9721758	54.0985081	25.0188611	18.2610202
Dus1l	12.5402849	0.55308807	0.38103819	1.45152924	0.14663255	0.94286416	Dus1l	7.39250961	5.88280835	9.224292	14.4769801	18.3129289	7.05632714	10.5849028	27.3915304
Eci1	6.50039855	-0.5982843	0.41232715	-1.4509941	0.1467815	0.94286416	Eci1	10.7527413	5.43028463	5.40734359	14.4769801	0	2.35210905	6.73584722	6.84788259
Baz1a	0.74876487	-0.4607503	0.31755324	-1.4509387	0.14679693	NA	Baz1a	3.36023164	1.35757116	1.27231614	0	0	0	0	0
Cttn	53.7302075	0.53787867	0.37073448	1.45084608	0.14682272	0.94286416	Cttn	34.2743627	24.8888045	28.9451921	74.4530404	79.9109626	27.0492541	46.1886666	114.131377
Inpp5b	22.215512	0.46378401	0.31969919	1.45068873	0.14686655	0.94286416	Inpp5b	22.1775288	14.480759	21.9474534	12.4088401	24.9721758	22.345036	36.5660278	22.8262753
Rsu1	10.6097357	-0.5577325	0.38447467	-1.4506353	0.14688144	0.94286416	Rsu1	14.1129729	18.5534725	13.9954775	8.27256004	4.99443516	8.23238167	14.4339583	2.28262753
Tmem25	26.8235646	0.44592431	0.30750806	1.45012233	0.14702441	0.94321542	Tmem25	24.1936678	25.793852	15.2677937	22.7495401	28.3017992	39.9858538	24.0565972	34.239413
Calcrl	3.70233957	-0.6029593	0.41587545	-1.4498554	0.14709884	NA	Calcrl	6.72046328	6.78785578	4.77118552	2.06814001	4.99443516	2.35210905	1.92452778	0
Kcnj6	29.5321247	0.53465601	0.36883866	1.44956608	0.14717956	0.94360338	Kcnj6	18.8172972	35.7493738	22.9016905	10.3407001	51.6091633	51.7463991	33.6792361	11.4131377
St5	10.6093714	-0.5609454	0.38705305	-1.4492727	0.14726146	0.94360338	St5	15.4570656	18.1009488	8.90621297	10.3407001	8.3240586	2.35210905	7.69811111	13.6957652
Scrn2	2.23824305	0.56753989	0.39161435	1.44923161	0.14727291	NA	Scrn2	0	2.26261859	1.27231614	0	4.99443516	0	4.81131944	4.56525506
Gm2011	1.29234635	0.52128599	0.35991563	1.44835607	0.14751749	NA	Gm2011	0	0.90504744	0.63615807	0	6.65924688	1.17605452	0.96226389	0
Bcap31	17.0952609	-0.4827245	0.33330812	-1.4482829	0.14753794	0.94480858	Bcap31	20.1613899	22.6261859	24.1740066	14.4769801	11.653682	9.40843619	18.2830139	15.9783927
Rorc	1.09457108	-0.5202824	0.35934735	-1.4478537	0.14765797	NA	Rorc	3.36023164	1.35757116	2.86271131	0	0	1.17605452	0	0
Wdr37	82.0973459	0.3671382	0.25363335	1.44751549	0.1477526	0.94495957	Wdr37	56.4518916	64.7108918	91.2886829	70.3167603	88.2350212	91.7322529	116.433931	77.6093361
Klhl8	30.0639562	0.41834479	0.28917425	1.44668756	0.14798445	0.94495957	Klhl8	19.4893435	20.8160911	26.7186389	35.1583802	36.6258578	37.6337448	29.8301805	34.239413
Myo10	21.7889292	-0.4942351	0.34165606	-1.4465868	0.14801269	0.94495957	Myo10	34.2743627	20.8160911	18.130505	33.0902402	24.9721758	16.4647633	10.5849028	15.9783927
Myo18a	90.7263823	0.56400765	0.38991471	1.44648979	0.14803987	0.94495957	Myo18a	43.6830113	47.5149905	45.1672229	126.156541	253.051381	69.3872169	40.4150833	100.435611
Sil1	6.64351378	-0.5836478	0.40364205	-1.4459538	0.14819015	0.94495957	Sil1	7.39250961	6.78785578	8.2700549	14.4769801	4.99443516	3.52816357	7.69811111	0
Pnma2	83.4375798	0.3463418	0.23956477	1.44571254	0.14825785	0.94495957	Pnma2	77.2853278	72.8563187	82.700549	55.8397803	78.2461509	96.436471	117.396194	86.7398462
Gmpr2	10.7646325	0.52766149	0.36504531	1.44546846	0.14832635	0.94495957	Gmpr2	7.39250961	6.33533207	7.63389683	12.4088401	19.9777406	9.40843619	11.5471667	11.4131377
Tecpr2	49.5363058	0.40359197	0.27932682	1.44487368	0.14849337	0.94495957	Tecpr2	39.6507334	34.8443264	41.6683535	51.7035003	84.9053977	49.39429	46.1886666	47.9351782
Fezf2	11.5677705	-0.571823	0.39576516	-1.4448544	0.14849878	0.94495957	Fezf2	20.8334362	17.648425	12.7231614	8.27256004	8.3240586	1.17605452	14.4339583	9.13051012
Olfml2b	1.27749004	-0.5456071	0.37771512	-1.4444937	0.14860014	NA	Olfml2b	2.01613899	2.26261859	1.59039517	2.06814001	0	0	0	2.28262753
Hist2h4	22.0052282	-0.5760624	0.39884454	-1.444328	0.14864672	0.94495957	Hist2h4	33.6023164	28.5089943	16.2220308	35.1583802	14.9833055	1.17605452	14.4339583	31.9567854
Ppp2r5d	94.3991239	-0.3776815	0.261525	-1.4441508	0.14869656	0.94495957	Ppp2r5d	127.016756	100.460266	98.6045007	105.475141	108.212762	85.8519803	51.96225	77.6093361
Wasf2	14.7401746	-0.5218847	0.36143737	-1.4439145	0.14876301	0.94495957	Wasf2	14.1129729	22.1736622	13.3593194	22.7495401	16.6481172	9.40843619	5.77358333	13.6957652
Fbl	2.09489724	0.58665658	0.40642732	1.4434477	0.14889439	NA	Fbl	0.67204633	1.81009488	1.27231614	0	6.65924688	1.17605452	2.88679167	2.28262753
Cachd1	9.18673535	-0.5743882	0.3980041	-1.4431716	0.14897214	0.94495957	Cachd1	7.39250961	14.9332827	13.3593194	12.4088401	13.3184938	8.23238167	3.84905555	0
Cdk16	157.830018	-0.3472775	0.24069122	-1.442834	0.14906724	0.94495957	Cdk16	200.269806	185.082201	176.851943	148.906081	156.492302	90.5561984	142.415056	162.066555
Ssrp1	46.3601519	0.37925398	0.2628895	1.44263647	0.1491229	0.94495957	Ssrp1	51.7475673	33.0342315	36.897168	37.2265202	58.2684102	56.4506172	53.8867778	43.3699231
Anpep	1.97086653	-0.5449211	0.37782423	-1.4422607	0.14922883	NA	Anpep	2.01613899	7.2403795	3.18079035	0	3.32962344	0	0	0
Ndufb3	13.6410134	0.52906535	0.36686031	1.44214389	0.14926179	0.94495957	Ndufb3	14.1129729	14.0282353	6.04350166	6.20442003	9.98887032	17.6408179	18.2830139	22.8262753
Znhit6	6.36087582	0.58785679	0.40763778	1.44210574	0.14927255	0.94495957	Znhit6	4.7043243	3.16766603	4.13502745	6.20442003	1.66481172	16.4647633	7.69811111	6.84788259
Zbtb16	33.6037323	0.47204968	0.32735518	1.44201071	0.14929935	0.94495957	Zbtb16	18.1452509	23.0787097	28.9451921	37.2265202	33.2962344	31.7534722	27.9056528	68.4788259
Rbmxl1	14.4107109	0.53882733	0.37367129	1.44198217	0.1493074	0.94495957	Rbmxl1	10.7527413	9.95552182	17.1762679	4.13628002	19.9777406	15.2887088	28.8679167	9.13051012
Prkar1a	627.524613	0.39093905	0.27112718	1.44190285	0.14932978	0.94495957	Prkar1a	465.728106	378.762353	442.766016	843.801124	760.818956	613.900462	662.999819	851.420069
Gm3558	0.87270397	0.4721057	0.32774747	1.44045566	0.14973853	NA	Gm3558	0	0	0.63615807	0	0	1.17605452	2.88679167	2.28262753
Klf13	170.666233	0.38302965	0.26596937	1.44012692	0.14983149	0.94747518	Klf13	147.850192	163.813586	139.954775	126.156541	281.353181	155.239197	131.830153	219.132243
Il11ra1	3.54836667	-0.59838	0.41557201	-1.4398948	0.14989716	NA	Il11ra1	5.37637063	5.43028463	5.08926455	4.13628002	3.32962344	1.17605452	3.84905555	0
Arl8a	189.170889	-0.3350959	0.23272675	-1.4398685	0.14990462	0.94747518	Arl8a	207.662315	190.965009	197.52708	256.449361	178.134854	114.077289	172.245236	196.305968
Zfp316	25.9044593	0.59081842	0.41044076	1.43947305	0.15001654	0.94762221	Zfp316	9.4086486	8.59795066	10.8146872	41.3628002	44.9499164	24.697145	5.77358333	61.6309433
Zfp385c	4.02787819	-0.5980386	0.41568917	-1.4386678	0.15024467	NA	Zfp385c	5.37637063	4.97776091	4.13502745	8.27256004	3.32962344	0	3.84905555	2.28262753
Dmxl2	252.63749	0.38848135	0.27005727	1.43851465	0.15028808	0.94839686	Dmxl2	239.920539	220.831575	246.511252	148.906081	327.967909	364.576902	301.188597	171.197065
Fkbp10	1.85156476	-0.5510488	0.38309347	-1.4384186	0.15031531	NA	Fkbp10	2.68818531	4.97776091	3.81694841	0	3.32962344	0	0	0
Ppp1r9b	535.132877	0.49843541	0.34651737	1.43841393	0.15031664	0.94839686	Ppp1r9b	311.15745	371.521973	292.314633	699.031323	1003.88147	412.795138	325.245194	865.115834
Chd3os	141.233938	0.33235212	0.23116339	1.4377368	0.15050875	0.94874232	Chd3os	124.328571	115.393548	122.142349	134.429101	109.877574	155.239197	197.264097	171.197065
Cldn25	36.0089368	-0.4334103	0.30150467	-1.4374912	0.15057848	0.94874232	Cldn25	30.9141311	59.2806072	43.2587487	35.1583802	21.6425524	34.1055812	31.7547083	31.9567854
Ehmt2	123.112705	0.51918336	0.36132507	1.4368872	0.15075005	0.94874232	Ehmt2	84.6778374	61.9957495	62.0254117	169.587481	238.068076	105.844907	66.3962083	196.305968
Acbd6	29.9219205	0.45872937	0.31926101	1.43684747	0.15076134	0.94874232	Acbd6	18.1452509	18.5534725	25.4463228	35.1583802	33.2962344	27.0492541	26.9433889	54.7830607
Tmem47	96.3772965	-0.5191615	0.36145156	-1.436324	0.15091018	0.94874232	Tmem47	85.3498837	189.607438	119.597717	82.7256004	49.9443516	147.006816	62.5471528	34.239413
Fam210b	45.6751142	0.48130417	0.33519714	1.43588388	0.15103539	0.94874232	Fam210b	43.010965	30.3190892	34.6706148	35.1583802	36.6258578	30.5774176	50.0377222	105.000866
Zc3h6	20.0744757	0.4950679	0.34481474	1.43575039	0.15107338	0.94874232	Zc3h6	15.4570656	14.0282353	20.0389792	14.4769801	16.6481172	32.9295267	35.6037639	11.4131377
Plcd3	5.22586716	0.5972682	0.41604399	1.43558906	0.15111931	0.94874232	Plcd3	3.36023164	3.16766603	3.18079035	2.06814001	9.98887032	1.17605452	2.88679167	15.9783927
Cyp2s1	1.88611399	-0.5802975	0.40434048	-1.4351703	0.15123857	NA	Cyp2s1	4.03227797	4.07271347	3.18079035	0	1.66481172	1.17605452	0.96226389	0
Cep350	47.6348166	0.51539623	0.35912484	1.43514505	0.15124577	0.94874232	Cep350	30.9141311	40.7271347	36.897168	39.2946602	49.9443516	110.549125	56.7735694	15.9783927
Gm527	1.82846643	-0.5707569	0.397779	-1.4348592	0.15132722	NA	Gm527	2.68818531	3.16766603	3.81694841	2.06814001	0	0	2.88679167	0
Syna	2.09837999	0.58713188	0.40919915	1.4348316	0.15133509	NA	Syna	0.67204633	1.81009488	1.59039517	0	1.66481172	5.88027262	2.88679167	2.28262753
Abhd11os	0.85704505	0.50074729	0.34902925	1.43468576	0.15137666	NA	Abhd11os	0	0.45252372	0.31807903	0	1.66481172	1.17605452	0.96226389	2.28262753
Cenpb	62.3893758	0.46494598	0.32410322	1.43456145	0.15141211	0.94874232	Cenpb	53.0916599	45.7048956	32.7621406	70.3167603	108.212762	39.9858538	57.7358333	91.3051012
Dynll2	772.515373	0.50996643	0.35550996	1.43446453	0.15143974	0.94874232	Dynll2	404.57189	342.560455	477.436631	1162.29469	942.283434	539.809027	754.414889	1556.75198
Tceal6	91.4783542	0.55746783	0.38868704	1.43423312	0.15150575	0.94874232	Tceal6	49.7314283	28.5089943	47.3937761	140.633521	103.218327	64.6829988	62.5471528	235.110636
Slc22a6	4.11951962	-0.5969728	0.41633163	-1.4338876	0.15160433	NA	Slc22a6	4.7043243	12.2181404	3.81694841	2.06814001	1.66481172	2.35210905	3.84905555	2.28262753
Itpkc	8.58606919	0.56021667	0.39071609	1.43382032	0.15162355	0.94874232	Itpkc	4.03227797	7.69290322	6.36158069	6.20442003	19.9777406	4.7042181	10.5849028	9.13051012
Aass	1.09940853	-0.5311998	0.37065641	-1.4331326	0.15181995	NA	Aass	2.01613899	2.26261859	1.27231614	2.06814001	0	1.17605452	0	0
Cox20	3.8801475	-0.5944432	0.41489019	-1.4327724	0.15192289	NA	Cox20	7.39250961	6.33533207	4.13502745	4.13628002	1.66481172	3.52816357	3.84905555	0
Ucp3	2.59983319	-0.5813663	0.40578633	-1.4326907	0.15194625	NA	Ucp3	4.03227797	1.35757116	2.22655324	8.27256004	1.66481172	0	0.96226389	2.28262753
Adarb2	21.6228883	0.49315642	0.34423968	1.43259606	0.15197331	0.94874232	Adarb2	21.5054825	14.9332827	21.6293743	8.27256004	34.9610461	36.4576902	19.2452778	15.9783927
Nrip3	128.21732	0.43973798	0.30699153	1.43241081	0.15202629	0.94874232	Nrip3	104.167181	106.795598	129.458167	80.6574604	91.5646446	181.112397	233.830125	98.1529838
Pak3	183.824557	0.46941331	0.32771638	1.43237669	0.15203604	0.94874232	Pak3	155.914748	163.361063	178.442338	90.9981605	111.542385	349.288194	270.396153	150.653417
Tigd2	12.1252697	0.54083424	0.3776107	1.43225348	0.15207129	0.94874232	Tigd2	8.06455594	9.5029981	12.4050823	6.20442003	8.3240586	23.5210905	22.1320694	6.84788259
Aggf1	40.5619782	0.43405481	0.30306785	1.43220338	0.15208562	0.94874232	Aggf1	43.010965	28.5089943	35.6248519	26.8858201	31.6314227	69.3872169	52.9245139	36.5220405
Blvra	6.9164128	0.56514174	0.39467029	1.43193381	0.15216276	0.94874232	Blvra	6.04841696	5.43028463	6.67965972	0	6.65924688	11.7605452	9.62263889	9.13051012
Nup107	10.6887309	0.56926611	0.39761947	1.43168568	0.1522338	0.94874232	Nup107	8.06455594	6.78785578	8.2700549	8.27256004	14.9833055	14.1126543	25.0188611	0
Pitpnm3	111.971073	0.50798149	0.35486344	1.43148443	0.15229143	0.94874232	Pitpnm3	75.2691888	81.4542694	65.5242811	124.088401	223.084771	78.7956531	55.8113055	191.740713
Hs3st3a1	0.76143584	-0.466197	0.32569017	-1.4314127	0.15231196	NA	Hs3st3a1	0.67204633	2.71514231	0.63615807	2.06814001	0	0	0	0
Rtn4r	32.7821675	-0.5229176	0.36542979	-1.4309659	0.15243999	0.94874232	Rtn4r	35.6184554	69.6886527	33.0802196	22.7495401	46.6147282	14.1126543	22.1320694	18.2610202
Ppfia1	17.8656337	0.48090508	0.33613465	1.43069178	0.15251857	0.94874232	Ppfia1	15.4570656	18.5534725	11.4508452	10.3407001	33.2962344	17.6408179	20.2075417	15.9783927
D130043K22Rik	14.2328716	-0.5551662	0.38804257	-1.4306838	0.15252085	0.94874232	D130043K22Ri	16.8011582	23.9837571	23.5378486	8.27256004	6.65924688	22.345036	7.69811111	4.56525506
Sstr1	32.8330496	0.52755058	0.36880283	1.4304407	0.15259057	0.94874232	Sstr1	15.4570656	21.7211385	20.6751372	43.4309402	33.2962344	67.0351079	15.3962222	45.6525506
Trmt2b	18.3746639	0.5103131	0.35693321	1.42971594	0.15279856	0.9488917	Trmt2b	15.4570656	10.4080455	18.448584	12.4088401	8.3240586	34.1055812	25.0188611	22.8262753
Csnk2b	81.2659834	-0.3585679	0.25081354	-1.4296194	0.15282629	0.9488917	Csnk2b	100.134903	93.2198861	80.7920748	95.1344405	66.5924688	47.042181	82.7546944	84.4572186
Grid2	7.89198025	-0.5616004	0.39293613	-1.4292409	0.152935	0.9488917	Grid2	8.73660227	12.6706641	7.95197586	12.4088401	8.3240586	8.23238167	4.81131944	0
Ascl1	7.24890411	0.5638599	0.3945504	1.42912008	0.15296972	0.9488917	Ascl1	5.37637063	4.52523719	4.13502745	6.20442003	6.65924688	7.05632714	5.77358333	18.2610202
Pglyrp1	1.2385341	-0.4899101	0.34288294	-1.428797	0.1530626	NA	Pglyrp1	0.67204633	6.78785578	1.27231614	0	0	1.17605452	0	0
Cnppd1	15.2301791	-0.5728752	0.40106033	-1.4284014	0.15317635	0.94961652	Cnppd1	16.8011582	11.313093	18.766663	33.0902402	24.9721758	2.35210905	7.69811111	6.84788259

GeneID	Base mean	log2(FC)	StdErr	Wald-Stats	P-value	P-adj		GeneID	Normalised expression for Chow#1	Normalised expression for Chow#2	Normalised expression for Chow#3	Normalised expression for Chow#4	Normalised expression for HFD#1	Normalised expression for HFD#2	Normalised expression for HFD#3	Normalised expression for HFD#4
Neurl4	84.1409844	0.5403919	0.3784003	1.42809587	0.15326427	0.94961652		Neurl4	57.1239379	41.6321821	48.0299342	103.407001	159.821925	41.1619083	46.1886666	175.76232
Vstm4	0.70620377	-0.4517259	0.31636729	-1.4278527	0.15333426	NA		Vstm4	1.34409266	2.71514231	1.59039517	0	0	0	0	0
Etv3	34.4460729	0.39601173	0.2774418	1.42736864	0.15347367	0.94963169		Etv3	30.9141311	26.2463757	25.7644018	33.0902402	41.620293	31.7534722	33.6792361	52.5004332
Gareml	12.1505898	-0.5931136	0.41560184	-1.4271198	0.15354537	0.94963169		Gareml	9.4086486	13.1231878	9.54237104	35.1583802	18.3129289	0	4.81131944	6.84788259
Pick1	15.3726368	0.47591448	0.33351561	1.42696311	0.15359053	0.94963169		Pick1	15.4570656	10.8605693	14.6316356	6.20442003	21.6425524	17.6408179	18.2830139	18.2610202
Ifngr2	26.3697251	-0.5211268	0.36522798	-1.4268535	0.15362212	0.94963169		Ifngr2	33.6023164	52.4927514	32.1259825	12.4088401	13.3184938	30.5774176	25.0188611	11.4131377
Klhdc4	7.88205849	0.56988617	0.39955004	1.42631989	0.15377603	0.95003362		Klhdc4	4.03227797	6.33533207	3.81694841	8.27256004	6.65924688	14.1126543	3.84905555	15.9783927
Atad2b	12.6670151	0.50387756	0.35344879	1.42560273	0.15398305	0.95063251		Atad2b	11.4247876	10.8605693	10.8146872	6.20442003	14.9833055	19.9929269	20.2075417	6.84788259
C1qtnf1	9.19076739	0.56088271	0.39357314	1.42510412	0.15412711	0.95063251		C1qtnf1	4.03227797	9.95552182	3.81694841	8.27256004	11.653682	11.7605452	5.77358333	18.2610202
Mar-09	25.6149374	0.51279492	0.35984088	1.42506022	0.1541398	0.95063251		Mar-09	15.4570656	14.9332827	14.6316356	35.1583802	39.9554813	24.697145	14.4339583	45.6525506
Lamb3	2.40472139	0.56943664	0.39959338	1.4250402	0.15414558	NA		Lamb3	0.67204633	1.81009488	0.31807903	2.06814001	0	2.35210905	2.88679167	9.13051012
Jkamp	27.7039255	-0.4818098	0.33822218	-1.4245363	0.15429129	0.95063736		Jkamp	30.9141311	47.0624668	37.8514051	16.5451201	11.653682	29.4013631	23.0943333	25.1089028
Taf3	28.1314845	0.49263334	0.34584295	1.42444232	0.15431847	0.95063736		Taf3	20.1613899	23.5312334	23.2197695	18.6132601	36.6258578	25.8731995	15.3962222	61.6309433
Maff	1.50230604	0.47108039	0.33084614	1.42386549	0.15448541	NA		Maff	0	0.90504744	0.31807903	0	1.66481172	0	0	9.13051012
Rhpn2	1.9896781	-0.5694032	0.39989988	-1.4238644	0.15448572	NA		Rhpn2	2.01613899	6.33533207	1.90847421	2.06814001	1.66481172	0	1.92452778	0
Arih2	23.3667717	0.48016065	0.33741604	1.42305222	0.15472102	0.95240479		Arih2	17.4732045	11.7656167	18.448584	26.8858201	29.966611	18.8168724	20.2075417	43.3699231
Rpl7a	11.5584805	-0.5368342	0.37731089	-1.4227902	0.15479698	0.95240479		Rpl7a	18.1452509	13.5757116	16.8581888	10.3407001	9.98887032	5.88027262	15.3962222	2.28262753
Mdh1	461.148205	0.41368424	0.29080898	1.4225291	0.15487271	0.95240479		Mdh1	409.276214	334.415028	517.832668	277.130761	346.280838	600.963862	783.282805	420.003466
Kif22	1.54809766	0.51135081	0.35955341	1.42218319	0.15497308	NA		Kif22	0	0.45252372	0.31807903	2.06814001	6.65924688	0	2.88679167	0
Fam117b	96.3039267	0.30298417	0.21305021	1.42212565	0.15498978	0.95257663		Fam117b	82.6616984	87.7896015	90.3344458	80.6574604	109.877574	127.013889	96.2263889	95.8703563
Lactb2	6.78812235	0.57862725	0.40701683	1.42162978	0.15513375	0.95291354		Lactb2	8.06455594	3.16766603	5.08926455	2.06814001	13.3184938	5.88027262	14.4339583	2.28262753
Fgf9	19.9892828	0.54034161	0.3803912	1.42048925	0.1554653	0.95440154		Fgf9	14.1129729	19.4585199	16.8581888	8.27256004	8.3240586	50.5703445	24.0565972	18.2610202
Ly86	2.16990963	-0.5570399	0.39219131	-1.4203271	0.15551248	NA		Ly86	2.01613899	8.14542694	3.18079035	0	1.66481172	2.35210905	0	0
Ing5	11.0428262	0.51174016	0.36054366	1.41935697	0.15579497	0.95492361		Ing5	4.7043243	9.95552182	10.1785291	8.27256004	14.9833055	11.7605452	12.5094306	15.9783927
Fmo2	1.38749046	-0.5117376	0.36054496	-1.4193448	0.15579852	NA		Fmo2	1.34409266	5.88280835	1.59039517	0	0	0	0	2.28262753
Gpr165	29.0364307	-0.5340236	0.37644629	-1.4185919	0.15601804	0.95492361		Gpr165	11.4247876	40.274611	29.5813502	66.1804803	18.3129289	30.5774176	15.3962222	20.5436478
Ptplb	9.14158804	-0.5527252	0.38969087	-1.4183684	0.15608323	0.95492361		Ptplb	10.0806949	17.648425	12.7231614	6.20442003	6.65924688	11.7605452	5.77358333	2.28262753
Sh3glb1	49.9326628	-0.5527511	0.38974188	-1.4182491	0.15611805	0.95492361		Sh3glb1	77.2853278	86.4320303	73.1581779	18.6132601	14.9833055	44.6900719	68.3207361	15.9783927
Mcmbp	18.0092336	-0.4613268	0.32528732	-1.4182132	0.15612854	0.95492361		Mcmbp	24.8657142	25.3413283	19.7209001	16.5451201	11.653682	15.2887088	19.2452778	11.4131377
Mtss1	66.5537283	0.36999836	0.26094051	1.41794143	0.15620786	0.95492361		Mtss1	43.010965	49.3250854	58.2084633	80.6574604	61.5980337	78.7956531	74.0943194	86.7398462
Ciapin1	37.8549445	0.46528772	0.32822506	1.41758745	0.15631125	0.95492361		Ciapin1	32.9302701	21.2686148	29.5813502	37.2265202	59.9332219	25.8731995	29.8301805	66.1961984
Flnb	9.06429488	0.56622368	0.39944354	1.41753121	0.15632768	0.95492361		Flnb	4.03227797	3.62018975	5.40734359	14.4769801	18.3129289	11.7605452	5.77358333	9.13051012
Strbp	212.81605	0.45732414	0.32275254	1.41694978	0.15649761	0.95492361		Strbp	185.484787	191.870057	198.799397	111.679561	196.447783	402.210647	297.339542	118.696632
Spock2	503.363571	0.41821799	0.29516222	1.41690893	0.15650955	0.95492361		Spock2	345.431813	520.402277	389.964896	417.764282	757.489333	755.027004	283.867847	556.961118
Ephx1	8.5112662	-0.5878227	0.41488635	-1.4168283	0.15653314	0.95492361		Ephx1	8.06455594	24.4362808	11.4508452	2.06814001	9.98887032	8.23238167	3.84905555	0
Ip6k2	31.7832287	0.4384626	0.30955369	1.41643475	0.15664826	0.95508076		Ip6k2	26.2098068	24.4362808	18.766663	37.2265202	21.6425524	37.6337448	40.4150833	47.9351782
E130102H24Rik	1.37784977	0.53682643	0.37917839	1.41576218	0.15684515	NA		E130102H24Ri	0	0.45252372	0.63615807	2.06814001	1.66481172	2.35210905	3.84905555	0
Accs	1.5378137	-0.5528411	0.39050334	-1.415714	0.15685925	NA		Accs	1.34409266	1.81009488	4.45310648	2.06814001	1.66481172	0	0.96226389	0
Nkd2	2.90083557	0.58851179	0.41573681	1.4155874	0.15689634	NA		Nkd2	2.68818531	1.81009488	0.9542371	2.06814001	4.99443516	5.88027262	4.81131944	0
Rtca	28.4281933	0.44912193	0.31735091	1.41522184	0.15700346	0.95648841		Rtca	23.5216215	19.4585199	25.7644018	24.8176801	26.6369875	22.345036	52.9245139	31.9567854
Prpf4	11.30583	0.52260177	0.36938615	1.41478441	0.15713172	0.95648841		Prpf4	8.06455594	7.2403795	11.1327662	6.20442003	19.9777406	7.05632714	12.5094306	18.2610202
Arpp19	136.295372	-0.4701269	0.33230853	-1.4147302	0.15714761	0.95648841		Arpp19	186.156833	196.847818	204.842898	66.1804803	78.2461509	111.72518	166.471653	79.8919636
Lrrc75b	20.8021567	-0.5123346	0.36235857	-1.4138884	0.15739468	0.95690815		Lrrc75b	19.4893435	21.7211385	14.3135566	49.6353602	19.9777406	14.1126543	13.4716944	13.6957652
Chga	69.1945537	0.45587497	0.32242716	1.41388513	0.15739563	0.95690815		Chga	40.994826	61.0907021	61.3892537	59.9760603	66.5924688	128.189943	39.4528194	95.8703563
Fnbp4	16.6353414	-0.488681	0.34573903	-1.413439	0.15752669	0.95716046		Fnbp4	21.5054825	15.3858064	17.4943469	28.9539601	8.3240586	18.8168724	13.4716944	9.13051012
Lrfn2	16.0273059	-0.5241486	0.37098011	-1.4128753	0.15769241	0.95743756		Lrfn2	14.7850192	28.5089943	22.9016905	12.4088401	23.3073641	11.7605452	7.69811111	6.84788259
Cars2	5.3919558	0.57260173	0.40539793	1.41244364	0.15781937	0.95743756		Cars2	5.37637063	4.52523719	2.54463228	2.06814001	11.653682	4.7042181	7.69811111	4.56525506
Srsf1	73.6347936	0.32718934	0.23165979	1.41237004	0.15784103	0.95743756		Srsf1	59.8121232	75.1189373	56.6180681	68.2486203	71.586904	83.4998712	94.3018611	79.8919636
Grin2d	29.4302334	0.54683339	0.3872815	1.41197912	0.15795611	0.95759211		Grin2d	18.1452509	14.9332827	14.3135566	39.2946602	38.2906696	25.8731995	11.5471667	73.044081
Trmt13	2.45405309	0.57741704	0.40902063	1.4117064	0.15803643	NA		Trmt13	2.68818531	0.90504744	1.59039517	0	3.32962344	8.23238167	2.88679167	0
Lap3	16.6761505	-0.4867598	0.34494265	-1.4111326	0.15820553	0.95801886		Lap3	23.5216215	22.6261859	14.9497146	20.6814001	19.9777406	9.40843619	15.3962222	6.84788259
Hsd17b11	15.0913201	-0.5478816	0.38825688	-1.4111317	0.15820577	0.95801886		Hsd17b11	12.7688802	37.106945	20.0389792	6.20442003	8.3240586	11.7605452	15.3962222	9.13051012
1700007K13Rik	2.01948928	-0.5681389	0.40263434	-1.4110542	0.15822862	NA		1700007K13Ri	3.36023164	4.07271347	1.27231614	4.13628002	0	2.35210905	0.96226389	0
Slitrk6	1.40857267	-0.5449538	0.38630045	-1.4106994	0.15833328	NA		Slitrk6	2.01613899	2.71514231	2.54463228	2.06814001	0	0	1.92452778	0
Cyp2j6	5.5359826	-0.5870547	0.41628242	-1.4102317	0.15847128	0.9588131		Cyp2j6	7.39250961	13.1231878	10.1785291	0	3.32962344	3.52816357	6.73584722	0
Ppp1r1a	38.4288482	-0.4381847	0.31078515	-1.4099281	0.15856091	0.9588131		Ppp1r1a	58.4680306	52.0402277	38.1694841	33.0902402	26.6369875	23.5210905	48.1131944	27.3915304
Ccdc37	0.85269419	0.42654987	0.30255225	1.40983866	0.15858733	NA		Ccdc37	0	0.45252372	0	0	1.66481172	4.7042181	0	0
Pnmal1	69.108742	0.39257314	0.27854834	1.40935369	0.15873061	0.9588131		Pnmal1	69.8928182	52.9452751	44.5310648	68.2486203	68.2572805	111.72518	59.6603611	77.6093361
Zfp57	1.71878428	0.55958791	0.39714836	1.40901479	0.15883079	NA		Zfp57	0.67204633	1.35757116	0.9542371	0	0	2.35210905	3.84905555	4.56525506
Ppp1r3g	3.38855957	-0.578115	0.41043522	-1.4085415	0.15897079	NA		Ppp1r3g	10.7527413	3.62018975	4.77118552	0	3.32962344	2.35210905	0	2.28262753
Cdc42ep2	8.11381028	-0.5848853	0.4152963	-1.4083565	0.15902552	0.9588131		Cdc42ep2	8.06455594	6.78785578	5.08926455	24.8176801	9.98887032	2.35210905	0.96226389	6.84788259
Tyrobp	4.95970522	-0.582227	0.4136192	-1.4076401	0.15923766	0.9588131		Tyrobp	4.03227797	9.05047438	5.08926455	10.3407001	9.98887032	1.17605452	0	0
Htatsf1	35.0097727	0.52925661	0.37600227	1.40758886	0.15925285	0.9588131		Htatsf1	29.5700384	24.8888045	35.9429309	14.4769801	24.9721758	78.7956531	57.7358333	13.6957652
Sft2d1	2.9375925	-0.5744538	0.40813401	-1.4075128	0.1592754	NA		Sft2d1	1.34409266	3.62018975	4.45310648	8.27256004	0	3.52816357	0	2.28262753
Cenpl	1.57799563	-0.5568614	0.39568649	-1.4073299	0.15932959	NA		Cenpl	3.36023164	1.81009488	2.54463228	2.06814001	1.66481172	1.17605452	0	0
Fmnl3	4.34177106	-0.5857449	0.41632688	-1.406935	0.15944667	NA		Fmnl3	2.68818531	6.33533207	3.81694841	12.4088401	4.99443516	3.52816357	0.96226389	0
Trib2	37.5568999	-0.4479394	0.31839697	-1.4068581	0.15946946	0.9588131		Trib2	57.7959842	35.2968501	39.4418003	45.4990802	33.2962344	14.1126543	38.4905555	36.5220405
Shroom4	2.78253205	0.56619433	0.40246322	1.40682254	0.15948002	NA		Shroom4	0	0.90504744	4.13502745	0	4.99443516	7.05632714	2.88679167	2.28262753
Ppp2r2c	442.580466	0.29026007	0.20635963	1.40657386	0.15955379	0.9588131		Ppp2r2c	401.211658	375.142163	343.843436	461.195222	566.035985	452.780992	392.603667	547.830607
Spg20	19.2699664	-0.4561317	0.32432315	-1.4064112	0.15960206	0.9588131		Spg20	19.4893435	19.4585199	20.6751372	33.0902402	13.3184938	14.1126543	13.4716944	20.5436478
Gtf3c5	11.3325243	0.51268467	0.36464889	1.40596801	0.15973362	0.9588131		Gtf3c5	10.0806949	7.69290322	11.7689243	4.13628002	8.3240586	17.6408179	17.32075	13.6957652
Slc30a5	25.8527105	-0.4899936	0.34860118	-1.4055995	0.15984309	0.9588131		Slc30a5	22.1775288	43.442277	41.0321955	18.6132601	16.6481172	32.9295267	18.2830139	13.6957652
Smim14	88.8858729	-0.3881928	0.27618421	-1.4055575	0.15985556	0.9588131		Smim14	75.2691888	84.6219354	101.785291	150.974221	79.9109626	55.2745626	85.6414861	77.6093361
Cct5	81.310063	0.36885903	0.26244521	1.40547061	0.15988138	0.9588131		Cct5	73.9250961	58.3755597	72.2039408	76.5211804	59.9332219	94.0843619	124.132042	91.3051012
Znrd1	10.3136718	-0.5056277	0.35981764	-1.4052332	0.15995194	0.9588131		Znrd1	13.4409266	12.6706641	13.3593194	10.3407001	9.98887032	5.88027262	7.69811111	9.13051012
Cdk5rap2	3.53108756	-0.5849212	0.41629618	-1.4050603	0.16000336	NA		Cdk5rap2	4.7043243	4.97776091	4.77118552	6.20442003	0	4.7042181	2.88679167	0
3110040N11Rik	3.42144134	0.58222348	0.41441262	1.40493665	0.16004012	NA		3110040N11R	4.7043243	2.26261859	0.9542371	0	1.66481172	5.88027262	9.62263889	2.28262753
Pdlim2	1.64626375	-0.5556496	0.39560585	-1.4045535	0.16015409	NA		Pdlim2	2.68818531	1.81009488	1.90847421	4.13628002	1.66481172	0	0.96226389	0
Zdhhc13	9.69928582	-0.5191245	0.36962039	-1.4044803	0.16017589	0.9588131		Zdhhc13	13.4409266	11.7656167	14.6316356	8.27256004	4.99443516	7.05632714	10.5849028	6.84788259
Cox7a2l	44.1020851	-0.4358465	0.31048234	-1.4037724	0.16038663	0.9588131		Cox7a2l	47.7152893	33.9392789	45.1672229	84.7937404	33.2962344	29.4013631	44.2641389	34.239413
Ddx6	145.564044	0.31403642	0.22378034	1.40332442	0.16052012	0.9588131		Ddx6	139.11359	119.918786	142.817486	111.679561	188.123724	183.464506	160.698069	118.696632
Sgol2	1.13284172	0.51340576	0.36586702	1.40325783	0.16053997	NA		Sgol2	0	0.90504744	0.63615807	0	0	2.35210905	2.88679167	2.28262753
Rps15	34.4959541	-0.4792696	0.34155866	-1.4031838	0.16056204	0.9588131		Rps15	46.3711967	28.961518	29.5813502	62.0442003	29.966611	12.9365998	25.0188611	41.0872956
Tmem104	14.5725144	-0.5292711	0.37728719	-1.4028336	0.16066647	0.9588131		Tmem104	12.7688802	22.6261859	12.0870033	24.8176801	16.6481172	5.88027262	5.77358333	15.9783927
Ostf1	5.3519516	-0.5785611	0.41247545	-1.4026558	0.16071951	0.9588131		Ostf1	8.73660227	9.05047438	4.45310648	6.20442003	4.99443516	0	4.81131944	4.56525506
Pik3c3	27.735772	0.47273702	0.33734097	1.40136261	0.16110567	0.9588131		Pik3c3	28.2259458	17.648425	26.4005599	16.5451201	26.6369875	52.9224536	37.5282917	15.9783927
Slc45a4	28.6717584	0.49845843	0.35572361	1.40125204	0.16113872	0.9588131		Slc45a4	13.4409266	17.1959013	20.9932163	39.2946602	46.6147282	37.6337448	15.3962222	38.804668
Zfp839	5.72828167	0.55906296	0.39907763	1.40088775	0.16124764	0.9588131		Zfp839	4.7043243	4.07271347	3.49886938	4.13628002	4.99443516	4.7042181	10.5849028	9.13051012
Hprt	94.1932208	0.39593411	0.28268624	1.40061328	0.16132975	0.9588131		Hprt	81.3176057	69.6886527	88.1078926	80.6574604	66.5924688	118.781507	161.660333	86.7398462
Tmem107	5.1511432	-0.5812322	0.41516012	-1.4000195	0.16150749	0.9588131		Tmem107	3.36023164	5.43028463	6.04350166	14.4769801	6.65924688	2.35210905	2.88679167	0
Hist3h2a	27.5794476	-0.4922556	0.35165787	-1.3998139	0.16156906	0.9588131		Hist3h2a	33.6023164	28.0564706	23.5378486	49.6353602	18.3129289	8.23238167	25.0188611	34.239413
Mgp	4.42523086	-0.5729446	0.4095189	-1.3990676	0.16179272	NA		Mgp	2.68818531	13.5757116	8.90621297	0	0	4.7042181	0.96226389	4.56525506
Epm2a	14.857084	0.48474788	0.34656204	1.39873334	0.16189296	0.9588131		Epm2a	9.4086486	9.95552182	11.4508452	16.5451201	24.9721758	10.5844907	15.3962222	20.5436478
B3galt6	8.41427997	0.53318067	0.38120994	1.39865361	0.16191688	0.9588131		B3galt6	5.37637063	6.78785578	7.95197586	4.13628002	16.6481172	7.05632714	12.5094306	6.84788259
Aspn	0.67707863	-0.4427846	0.316581	-1.3986455	0.16191932	NA		Aspn	2.01613899	1.81009488	1.59039517	0	0	0	0	0
Rbm15b	44.7000876	0.38814605	0.27755098	1.39846761	0.16197269	0.9588131		Rbm15b	38.978687	33.4867552	39.4418003	37.2265202	53.2739751	42.3379629	37.5282917	75.3267085
Nr4a1	37.9882585	-0.4978066	0.35620192	-1.3975406	0.16225108	0.9588131		Nr4a1	65.1884939	32.5817078	44.5310648	41.3628002	46.6147282	12.9365998	17.32075	43.3699231
Lrrcc1	8.5068981	0.56094973	0.40143182	1.39737234	0.16230163	0.9588131		Lrrcc1	7.39250961	7.2403795	5.72542262	4.13628002	9.98887032	15.2887088	18.2830139	0
Snx18	16.498575	0.47094948	0.33709927	1.39706465	0.16239413	0.9588131		Snx18	8.73660227	10.8605693	12.7231614	22.7495401	18.3129289	18.8168724	19.2452778	20.5436478
Bop1	27.5851353	0.47526091	0.34021145	1.39695743	0.16242637	0.9588131		Bop1	19.4893435	17.648425	15.2677937	37.2265202	44.9499164	19.9929269	25.0188611	41.0872956
Adarb1	166.912996	0.31867136	0.22816322	1.39668155	0.16250936	0.9588131		Adarb1	135.753358	132.58945	131.68472	190.268881	203.10703	194.048996	142.415056	205.436478
Eid2	26.2397416	0.48973358	0.35067864	1.39653097	0.16255466	0.9588131		Eid2	23.5216215	14.480759	18.766663	24.8176801	19.9777406	18.8168724	27.9056528	61.6309433
Slc24a4	13.6754092	-0.5651448	0.4047278	-1.3963578	0.16260678	0.9588131		Slc24a4	18.1452509	28.5089943	15.9039517	8.27256004	21.6425524	11.7605452	2.88679167	2.28262753
Plod1	4.74355778	-0.5802524	0.41560414	-1.3961661	0.16266448	0.9588131		Plod1	3.36023164	11.7656167	5.40734359	6.20442003	8.3240586	0	2.88679167	0
Ddr1	28.5258918	-0.5393936	0.38640945	-1.3959121	0.16274098	0.9588131		Ddr1	26.2098068	39.8220873	25.1282437	53.7716403	49.9443516	9.40843619	12.5094306	11.4131377
Xkrx	1.0979407	-0.487856	0.34949428	-1.3958911	0.16274729	NA		Xkrx	0.67204633	4.07271347	2.86271131	0	0	1.17605452	0	0
Dact1	9.46911293	0.51183791	0.36676427	1.39555011	0.16285001	0.9588131		Dact1	8.73660227	5.88280835	7.95197586	6.20442003	6.65924688	14.1126543	12.5094306	13.6957652
Sharpin	28.3456143	-0.4490522	0.32179225	-1.3954725	0.16287341	0.9588131		Sharpin	36.2905017	29.8665654	26.4005599	41.3628002	26.6369875	11.7605452	20.2075417	34.239413
Golga2	62.569568	0.38975948	0.27937972	1.39508867	0.1629891	0.9588131		Golga2	64.5164475	40.274611	43.2587487	64.1123403	74.9165274	64.6829988	52.9245139	95.8703563
Elavl1	109.406575	0.31459205	0.22550181	1.39507547	0.16299308	0.9588131		Elavl1	88.038069	93.6724098	96.0598684	107.543281	104.883138	122.309671	102.962236	159.783927
Lrrc28	20.0849179	-0.4888012	0.35042774	-1.3948702	0.163055	0.9588131		Lrrc28	32.2582238	19.0059962	22.9016905	24.8176801	23.3073641	9.40843619	22.1320694	6.84788259
Mettl2	7.12263145	-0.5544978	0.39760908	-1.3945803	0.16314245	0.9588131		Mettl2	10.0806949	7.2403795	6.67965972	14.4769801	4.99443516	3.52816357	7.69811111	2.28262753

GeneID	Base mean	log2(FC)	StdErr	Wald-Stats	P-value	P-adj		GeneID	Normalised expression for Chow#1	Normalised expression for Chow#2	Normalised expression for Chow#3	Normalised expression for Chow#4	Normalised expression for HFD#1	Normalised expression for HFD#2	Normalised expression for HFD#3	Normalised expression for HFD#4
Atp1a4	1.55471978	0.52045411	0.37321441	1.39451771	0.16316132	NA		Atp1a4	0	0	0.9542371	2.06814001	4.99443516	1.17605452	0.96226389	2.28262753
Ubr5	103.037838	0.37060354	0.26581558	1.39421299	0.1632533	0.9588131		Ubr5	83.3337447	88.6946489	87.7898135	95.1344405	138.179373	158.767361	103.9245	68.4788259
Stoml2	20.2740131	0.46080927	0.33056728	1.39399538	0.163319	0.9588131		Stoml2	15.4570656	16.7433776	16.5401098	14.4769801	21.6425524	14.1126543	22.1320694	41.0872956
Mdga2	66.9847425	0.40712331	0.29206586	1.39394353	0.16333466	0.9588131		Mdga2	36.9625481	62.4482732	47.7118552	78.5893204	69.9220923	88.2040893	53.8867778	98.1529838
Tspyl5	73.3162553	0.31253566	0.22421265	1.39392516	0.16334021	0.9588131		Tspyl5	60.4841696	61.9957495	63.9338859	74.4530404	96.5590798	82.3238167	76.0188472	70.7614535
Tmem169	10.8461951	0.52349195	0.37557028	1.39385882	0.16336024	0.9588131		Tmem169	12.0968339	6.33533207	9.86045007	4.13628002	8.3240586	17.6408179	19.2452778	9.13051012
Hspa12a	153.995549	0.35198713	0.25262639	1.39331098	0.16352577	0.9588131		Hspa12a	160.619072	129.874307	150.769462	88.9300204	218.090335	182.288451	171.282972	130.109769
Rims2	94.8824384	0.42374726	0.30415624	1.39318941	0.16356252	0.9588131		Rims2	90.054208	85.0744592	83.336707	55.8397803	74.9165274	156.415252	147.226375	66.1961984
Chpf2	33.2659301	0.5379089	0.3861137	1.39313601	0.16357866	0.9588131		Chpf2	15.4570656	18.1009488	14.6316356	51.7035003	68.2572805	25.8731995	17.32075	54.7830607
1700020I14Rik	46.8293742	0.46306009	0.33244799	1.3928798	0.16365614	0.9588131		1700020I14Ri	49.059382	40.7271347	39.4418003	20.6814001	41.620293	49.39429	97.1886527	36.5220405
Ccdc110	0.65486259	0.38119696	0.2738179	1.39215499	0.16387547	NA		Ccdc110	0	0	0	0	0	2.35210905	2.88679167	0
Galnt10	6.08923372	-0.5749739	0.41306782	-1.3919602	0.16393446	0.9588131		Galnt10	8.73660227	7.2403795	6.67965972	10.3407001	11.653682	1.17605452	2.88679167	0
Stra6	1.41607691	-0.5174868	0.37191693	-1.3914042	0.16410288	NA		Stra6	2.01613899	4.52523719	2.86271131	0	0	0	1.92452778	0
Tctn2	15.477479	-0.503552	0.36193056	-1.3912946	0.16413612	0.9588131		Tctn2	14.1129729	22.1736622	13.6773985	26.8858201	13.3184938	16.4647633	5.77358333	11.4131377
Igsf10	12.1343203	0.521401	0.37491058	1.39073427	0.16430602	0.9588131		Igsf10	8.73660227	10.8605693	10.8146872	6.20442003	9.98887032	28.2253086	15.3962222	6.84788259
Hip1r	34.1454327	0.46889949	0.33718019	1.3906496	0.16433171	0.9588131		Hip1r	29.5700384	19.9110436	26.7186389	33.0902402	73.2517157	22.345036	31.7547083	36.5220405
Fam117a	4.70042679	-0.5789072	0.41635484	-1.3904179	0.16440202	0.9588131		Fam117a	4.03227797	5.88280835	4.77118552	12.4088401	6.65924688	0	3.84905555	0
Hmbox1	36.5249125	0.38991058	0.28046533	1.39022738	0.16445984	0.9588131		Hmbox1	29.5700384	32.5817078	30.2175083	33.0902402	34.9610461	58.8027262	43.301875	29.6741579
Islr	4.38921402	-0.5734691	0.41260116	-1.3898874	0.16456309	NA		Islr	8.06455594	4.07271347	5.08926455	6.20442003	1.66481172	3.52816357	1.92452778	4.56525506
Pigw	3.82134163	0.57662417	0.41494802	1.38962988	0.1646413	NA		Pigw	1.34409266	4.52523719	1.90847421	2.06814001	3.32962344	7.05632714	5.77358333	4.56525506
Tbkbp1	20.6443891	0.49479849	0.35608771	1.38954107	0.16466828	0.9588131		Tbkbp1	10.0806949	17.648425	13.9954775	22.7495401	38.2906696	21.1689814	11.5471667	29.6741579
Armcx1	47.8577894	0.45682715	0.32880629	1.38935039	0.16472623	0.9588131		Armcx1	44.3550577	38.0119924	45.4853019	26.8858201	33.2962344	68.2111624	92.3773333	34.239413
Msl3l2	12.5155609	0.53755111	0.38698531	1.38907366	0.16481035	0.9588131		Msl3l2	9.4086486	10.8605693	9.224292	6.20442003	26.6369875	3.52816357	18.2830139	15.9783927
Slc12a5	375.4034	0.47638036	0.3429754	1.38896366	0.1648438	0.9588131		Slc12a5	202.285945	349.800835	257.00786	380.537762	664.259876	450.428883	151.07543	547.830607
Mphosph6	3.85645457	-0.5759332	0.41472102	-1.3887244	0.16491659	NA		Mphosph6	7.39250961	4.97776091	5.08926455	4.13628002	1.66481172	4.7042181	2.88679167	0
Zeb2	134.988498	0.48753922	0.35114923	1.38841031	0.16501214	0.9588131		Zeb2	117.608107	140.282353	126.595456	37.2265202	113.207197	206.985596	244.415028	93.5877288
Lingo1	187.625269	-0.4884891	0.35187492	-1.3882464	0.16506202	0.9588131		Lingo1	237.232354	354.326072	229.971142	88.9300204	243.062511	119.957561	83.7169583	143.805534
Mllt3	33.525119	0.49953	0.35986119	1.38811857	0.16510094	0.9588131		Mllt3	29.5700384	30.7716129	34.0344567	8.27256004	29.966611	50.5703445	64.4716805	20.5436478
Slc27a1	31.4552592	-0.5248013	0.37809349	-1.3880199	0.165131	0.9588131		Slc27a1	26.2098068	44.7998482	29.2632712	55.8397803	31.6314227	7.05632714	13.4716944	43.3699231
Xlr3b	1.2178069	0.51240144	0.36916431	1.38800374	0.16513591	NA		Xlr3b	0	0.45252372	0.31807903	2.06814001	1.66481172	2.35210905	2.88679167	0
A330048O09Rik	0.90233638	-0.4327409	0.3119564	-1.3871838	0.16538573	NA		A330048O09R	0	1.81009488	1.27231614	4.13628002	0	0	0	0
Slc46a3	3.24308871	-0.5751395	0.41469179	-1.3869084	0.16546969	NA		Slc46a3	2.01613899	7.69290322	4.13502745	4.13628002	3.32962344	2.35210905	0	2.28262753
Fxr2	109.782929	0.48351491	0.34863379	1.38688484	0.16547688	0.9588131		Fxr2	79.3014668	59.7331309	51.5288036	157.178641	158.157113	96.436471	75.0565833	200.871223
Eef1a2	570.837345	0.31304165	0.22572729	1.38681354	0.16549863	0.9588131		Eef1a2	591.400769	378.309829	469.802734	577.011063	750.830086	612.724407	510.962125	675.657749
Bend3	5.23532603	0.56713448	0.40899389	1.38665757	0.1655462	0.9588131		Bend3	5.37637063	4.07271347	3.18079035	0	8.3240586	4.7042181	4.81131944	11.4131377
Mroh1	51.7217159	0.53261976	0.3841173	1.38660706	0.16556161	0.9588131		Mroh1	29.5700384	25.3413283	21.6293743	78.5893204	101.553515	48.2182355	22.1320694	86.7398462
0610010K14Rik	19.7643508	-0.5073358	0.36591318	-1.3864923	0.16559664	0.9588131		0610010K14Ri	16.8011582	24.4362808	17.1762679	39.2946602	14.9833055	5.88027262	14.4339583	25.1089028
Prkch	4.017045	-0.5762601	0.41567517	-1.3863232	0.16564823	NA		Prkch	5.37637063	9.5029981	4.45310648	2.06814001	4.99443516	1.17605452	0	4.56525506
Unc79	57.6679799	0.35404466	0.25538656	1.38630889	0.1656526	0.9588131		Unc79	57.1239379	47.9675142	52.8011197	41.3628002	74.9165274	76.4435441	67.3584722	43.3699231
Itm2b	249.457309	-0.5086924	0.36695714	-1.3862448	0.16567217	0.9588131		Itm2b	263.442161	524.927514	323.804457	119.952121	106.54795	342.231867	191.490514	123.261887
Lgr4	21.9053734	-0.5161274	0.37232496	-1.3862283	0.1656772	0.9588131		Lgr4	20.8334362	40.274611	31.8079035	16.5451201	13.3184938	30.5774176	17.32075	4.56525506
Klf3	16.9301212	-0.4617244	0.33318674	-1.3857825	0.16581334	0.9588131		Klf3	24.8657142	20.3635674	22.2655324	12.4088401	11.653682	10.5844907	17.32075	15.9783927
Cox17	20.4895799	-0.4853488	0.35035255	-1.3853155	0.165956	0.9588131		Cox17	30.9141311	28.961518	22.2655324	18.6132601	16.6481172	11.7605452	27.9056528	6.84788259
Foxg1	126.405687	0.46130073	0.33301785	1.38521324	0.16598727	0.9588131		Foxg1	110.887644	111.320835	109.419188	70.3167603	153.162678	74.091435	105.849028	276.197931
Hspa2	29.5423447	0.49784545	0.35940454	1.38519521	0.16599278	0.9588131		Hspa2	30.2420848	21.7211385	24.4920857	12.4088401	63.2628454	23.5210905	17.32075	43.3699231
Ypel2	58.7363452	-0.4159216	0.30029859	-1.3850268	0.16604428	0.9588131		Ypel2	73.9250961	86.884554	79.8378377	31.0221002	58.2684102	42.3379629	51.96225	45.6525506
Vps13d	111.734467	0.37141851	0.26819034	1.38490637	0.1660811	0.9588131		Vps13d	101.478996	93.2198861	88.1078926	101.338861	156.492302	171.703961	103.9245	77.6093361
Dcakd	41.1426706	-0.5260274	0.37985061	-1.3848269	0.16610539	0.9588131		Dcakd	43.010965	44.3473245	39.4418003	78.5893204	44.9499164	7.05632714	19.2452778	52.5004332
Nln	22.1936587	0.44542626	0.32169404	1.38462702	0.16616655	0.9588131		Nln	25.5377605	16.7433776	18.766663	10.3407001	26.6369875	35.2816357	25.981125	18.2610202
Ttc28	25.4164912	0.50687837	0.36613355	1.38440842	0.16623343	0.9588131		Ttc28	18.8172972	17.1959013	16.5401098	24.8176801	49.9443516	18.8168724	11.5471667	45.6525506
Kcng4	2.75222958	-0.5544503	0.40050011	-1.3843948	0.16623761	NA		Kcng4	4.03227797	4.52523719	0.63615807	8.27256004	1.66481172	0	2.88679167	0
Trim32	146.546548	0.37028454	0.26747163	1.38438806	0.16623966	0.9588131		Trim32	109.543552	98.197647	116.416927	179.928181	211.431088	142.302597	125.094306	189.458085
Supt4a	7.24109017	-0.5480302	0.39599221	-1.383942	0.16637622	0.9588131		Supt4a	8.73660227	8.14542694	9.86045007	10.3407001	1.66481172	2.35210905	7.69811111	9.13051012
Desi1	86.6927181	-0.385016	0.27821531	-1.3838779	0.16639585	0.9588131		Desi1	99.4628566	104.985503	94.1513942	99.2707205	63.2628454	41.1619083	88.5282777	102.718239
Pmpca	55.5672797	0.38465858	0.2779875	1.38372618	0.16644232	0.9588131		Pmpca	43.010965	46.1574193	56.2999891	41.3628002	44.9499164	49.39429	78.9056389	84.4572186
Noc4l	17.4939344	0.51624146	0.37313691	1.38351757	0.16650622	0.9588131		Noc4l	14.1129729	8.59795066	14.9497146	14.4769801	38.2906696	10.5844907	11.5471667	27.3915304
Ccnl1	8.50779529	0.52091377	0.37652956	1.3834605	0.16652371	0.9588131		Ccnl1	6.04841696	4.52523719	8.2700549	6.20442003	13.3184938	10.5844907	7.69811111	11.4131377
Efr3a	117.496493	0.32268399	0.23331176	1.38305923	0.1666467	0.9588131		Efr3a	95.4305786	87.7896015	118.64348	113.747701	111.542385	136.422325	164.547125	111.848749
Prickle2	192.705705	0.35440841	0.25636028	1.38246225	0.16682981	0.9588131		Prickle2	187.500926	192.322581	180.986971	101.338861	264.705064	243.443286	200.150889	171.197065
Nkx6-2	10.498739	-0.57203	0.4139027	-1.3820397	0.16695951	0.9588131		Nkx6-2	4.7043243	5.43028463	5.08926455	45.4990802	13.3184938	1.17605452	1.92452778	6.84788259
Exoc4	34.9398277	0.4071727	0.29477377	1.38130574	0.16718498	0.9588131		Exoc4	37.6345944	26.6988994	32.4440615	18.6132601	48.2795399	50.5703445	35.6037639	29.6741579
Abca9	2.51205483	-0.5543547	0.4013296	-1.3812953	0.16718817	NA		Abca9	1.34409266	8.59795066	4.77118552	0	0	1.17605452	1.92452778	2.28262753
Atxn1	188.025917	0.27535249	0.19934583	1.3812804	0.16719276	0.9588131		Atxn1	158.602934	185.082201	171.4446	157.178641	234.738453	190.520833	176.094292	230.545381
Tsr3	7.26796697	0.55373995	0.40090919	1.38121042	0.16721427	0.9588131		Tsr3	4.7043243	3.62018975	4.45310648	8.27256004	3.32962344	5.88027262	9.62263889	18.2610202
BC030336	40.929037	0.40597309	0.29393984	1.38114348	0.16723485	0.9588131		BC030336	34.9464091	45.2523719	32.7621406	22.7495401	31.6314227	50.5703445	61.5848889	47.9351782
Ccdc42	1.12651957	-0.4830306	0.34975331	-1.3810608	0.16726027	NA		Ccdc42	4.03227797	3.16766603	0.63615807	0	0	1.17605452	0	0
Ndufc1	17.4252629	-0.5263831	0.38114439	-1.3810596	0.16726064	0.9588131		Ndufc1	26.2098068	9.95552182	14.9497146	37.2265202	9.98887032	7.05632714	13.4716944	20.5436478
Ift81	14.048226	0.53853682	0.38999841	1.38086927	0.16731916	0.9588131		Ift81	11.4247876	9.95552182	15.2677937	4.13628002	11.653682	29.4013631	25.981125	4.56525506
E2f3	31.3783552	0.37041853	0.26828194	1.38070617	0.16736932	0.9588131		E2f3	23.5216215	26.6988994	28.6271131	28.9539601	39.9554813	37.6337448	33.6792361	31.9567854
Cep170b	357.272185	0.5097008	0.36924116	1.3804008	0.16746327	0.9588131		Cep170b	220.431196	189.154915	161.58415	521.171283	674.248747	283.42914	189.565986	618.592061
Nr2f1	56.808635	0.36935281	0.26766363	1.37991406	0.16761311	0.9588131		Nr2f1	51.7475673	50.2301328	54.0734359	35.1583802	63.2628454	44.6900719	73.1320555	82.1745911
Scai	59.4687259	0.4992451	0.36181089	1.37985093	0.16763255	0.9588131		Scai	42.3389187	54.75537	59.7988585	26.8858201	46.6147282	110.549125	109.698083	25.1089028
Fam151b	1.73275798	-0.5461007	0.39579423	-1.3797591	0.16766084	NA		Fam151b	2.68818531	3.62018975	4.45310648	0	0	1.17605452	1.92452778	0
4930577N17Rik	2.58962486	0.56062556	0.40634587	1.37967581	0.16768648	NA		4930577N17R	0.67204633	1.35757116	1.27231614	2.06814001	3.32962344	0	2.88679167	9.13051012
Rab3gap1	61.5889154	0.34547498	0.25042865	1.37953457	0.16773	0.9588131		Rab3gap1	58.4680306	51.587704	52.4830407	51.7035003	56.6035985	94.0843619	61.5848889	66.1961984
8030443G20Rik	1.17020029	0.49988131	0.36237653	1.37945277	0.1677552	NA		8030443G20R	0.67204633	0	0.9542371	0	0	3.52816357	1.92452778	2.28262753
Prpsap2	8.65238728	0.53315505	0.38649861	1.37944884	0.16775641	0.9588131		Prpsap2	4.7043243	6.33533207	5.72542262	10.3407001	3.32962344	12.9365998	14.4339583	11.4131377
Smg1	61.2510835	0.47616487	0.34533752	1.37883909	0.16794437	0.95937437		Smg1	61.8282622	49.3250854	60.7530956	20.6814001	88.2350212	103.492798	76.0188472	29.6741579
Grid2ip	1.62847547	-0.4534043	0.32912315	-1.377613	0.16832281	NA		Grid2ip	0	0.45252372	1.27231614	10.3407001	0	0	0.96226389	0
Cpsf1	35.3542407	0.50672189	0.3678683	1.37745464	0.16837173	0.95959688		Cpsf1	27.5538995	17.648425	18.766663	45.4990802	68.2572805	18.8168724	26.9433889	59.3483158
Mid1ip1	55.9882847	-0.4771557	0.34642722	-1.377362	0.16840035	0.95959688		Mid1ip1	61.8282622	46.1574193	45.1672229	119.952121	69.9220923	24.697145	41.3773472	38.804668
Zgpat	11.1974659	0.55210181	0.40092904	1.37705617	0.16849488	0.95959688		Zgpat	5.37637063	5.43028463	6.67965972	14.4769801	11.653682	8.23238167	5.77358333	31.9567854
Neat1	3.12652374	0.54615554	0.39668888	1.37678559	0.16857855	NA		Neat1	1.34409266	0.90504744	1.90847421	2.06814001	16.6481172	1.17605452	0.96226389	0
Net1	12.1793774	-0.5097284	0.37024139	-1.376746	0.16859079	0.95959688		Net1	14.1129729	23.9837571	13.6773985	8.27256004	11.653682	10.5844907	10.5849028	4.56525506
Rnf5	58.8289	-0.5161097	0.37495767	-1.376448	0.16868297	0.95959688		Rnf5	71.2369108	52.0402277	52.8011197	115.815841	28.3017992	17.6408179	34.6415	98.1529838
Sh3gl3	17.8233665	-0.5171264	0.3757604	-1.3762131	0.16875568	0.95959688		Sh3gl3	16.8011582	23.5312334	18.448584	33.0902402	11.653682	11.7605452	25.0188611	2.28262753
Prdm13	2.97359303	0.46588587	0.33855386	1.3761056	0.16878894	NA		Prdm13	0	0	0	4.13628002	4.99443516	0	0.96226389	13.6957652
Nefh	51.4124623	0.51939864	0.37744705	1.37608346	0.16879579	0.95959688		Nefh	29.5700384	21.2686148	23.8559276	82.7256004	38.2906696	71.739326	36.5660278	107.283494
1700025G04Rik	103.877414	0.30982536	0.22524508	1.37550333	0.16897545	0.95959688		1700025G04R	96.102625	96.8400759	96.0598684	78.5893204	118.201632	127.013889	133.754681	84.4572186
Tagln2	4.72028542	-0.5723033	0.41613276	-1.3752903	0.16904146	0.95959688		Tagln2	3.36023164	7.2403795	3.81694841	12.4088401	6.65924688	2.35210905	1.92452778	0
Ppdpf	25.7937202	-0.4536803	0.32992644	-1.3750952	0.16910194	0.95959688		Ppdpf	36.2905017	36.2018975	30.5355873	18.6132601	26.6369875	10.5844907	26.9433889	20.5436478
Cpne1	26.5153374	0.47234132	0.34355649	1.3748578	0.16917553	0.95959688		Cpne1	34.9464091	11.313093	18.766663	18.6132601	33.2962344	22.345036	31.7547083	41.0872956
Galr1	3.66127516	-0.5708148	0.41534035	-1.3743302	0.16933918	NA		Galr1	2.01613899	6.33533207	2.86271131	10.3407001	0	3.52816357	1.92452778	2.28262753
Car5b	1.77702603	-0.5404906	0.3933086	-1.3742151	0.16937491	NA		Car5b	2.68818531	5.88280835	2.54463228	0	0	1.17605452	1.92452778	0
Dnajb11	15.4393926	-0.5051529	0.36763059	-1.3740773	0.16941768	0.95959688		Dnajb11	16.1291119	27.1514231	22.2655324	10.3407001	9.98887032	21.1689814	9.62263889	6.84788259
Bbs9	16.7569062	0.47157752	0.34321594	1.37399657	0.16944275	0.95959688		Bbs9	11.4247876	9.5029981	12.4050823	22.7495401	14.9833055	16.4647633	25.981125	20.5436478
Ankrd35	7.47392825	0.54725588	0.39832534	1.37389171	0.1694753	0.95959688		Ankrd35	4.7043243	4.07271347	4.45310648	10.3407001	9.98887032	15.2887088	8.660375	2.28262753
Tmem120a	6.38874117	-0.5523579	0.40218638	-1.3733878	0.16963183	0.95959688		Tmem120a	6.04841696	13.5757116	6.67965972	6.20442003	6.65924688	3.52816357	3.84905555	4.56525506
Fam180a	1.10078508	-0.4982988	0.36284048	-1.3733274	0.1696506	NA		Fam180a	2.01613899	2.26261859	2.86271131	0	1.66481172	0	0	0
Phactr4	8.67460775	-0.5448946	0.39677918	-1.3732944	0.16966085	0.95959688		Phactr4	11.4247876	13.5757116	8.58813393	12.4088401	8.3240586	3.52816357	11.5471667	0
Zfhx2os	12.4209713	-0.5083387	0.37018511	-1.3732013	0.16968979	0.95959688		Zfhx2os	16.8011582	16.2908539	13.6773985	14.4769801	19.9777406	5.88027262	7.69811111	4.56525506
Fam179a	3.718744	-0.5709227	0.41587158	-1.3728342	0.16980388	NA		Fam179a	4.03227797	4.07271347	5.40734359	6.20442003	3.32962344	1.17605452	0.96226389	4.56525506
Tiam2	30.3559878	-0.5237754	0.38153338	-1.3728168	0.16980932	0.95959688		Tiam2	59.1400769	44.3473245	40.3960374	8.27256004	19.9777406	31.7534722	29.8301805	9.13051012
B230216N24Rik	3.6858164	0.57049105	0.41559681	1.37270317	0.16984464	NA		B230216N24R	3.36023164	2.26261859	2.54463228	0	0	3.52816357	8.660375	9.13051012
Dach1	19.0035952	-0.4615071	0.33622927	-1.3725966	0.1698778	0.95959688		Dach1	26.8818531	18.5534725	29.2632712	14.4769801	21.6425524	11.7605452	13.4716944	15.9783927
Xiap	68.79553	0.43648713	0.31802361	1.37249916	0.1699081	0.95959688		Xiap	60.4841696	52.9452751	70.2954666	41.3628002	43.2851047	78.7956531	134.716944	68.4788259
Zhx2	18.8687806	-0.5266603	0.38374369	-1.3724272	0.1699305	0.95959688		Zhx2	18.8172972	23.5312334	14.9497146	39.2946602	28.3017992	7.05632714	14.4339583	4.56525506
Ctsh	0.96705801	-0.4853904	0.35371459	-1.3722656	0.16998078	NA		Ctsh	2.01613899	3.16766603	1.59039517	0	0	0	0.96226389	0
Mboat2	20.8722406	-0.5253658	0.38288848	-1.3721117	0.17002868	0.95959688		Mboat2	18.1452509	42.9897533	26.0824808	18.6132601	13.3184938	28.2253086	17.32075	2.28262753
Ctxn1	434.646459	-0.464757	0.33873218	-1.3720485	0.17004832	0.95959688		Ctxn1	588.712584	515.87704	488.887476	492.217322	486.125022	107.020962	309.848972	488.482292
Fbn1	3.59941742	0.57004821	0.4155745	1.37171124	0.17015335	NA		Fbn1	0.67204633	2.71514231	4.13502745	0	1.66481172	4.7042181	5.77358333	9.13051012
Cpsf7	68.3071493	0.34108648	0.24887086	1.37053602	0.17051964	0.96010402		Cpsf7	61.8282622	52.4927514	50.8926455	74.4530404	94.8942681	61.1548353	73.1320555	77.6093361

GeneID	Base mean	log2(FC)	StdErr	Wald-Stats	P-value	P-adj		GeneID	Normalised expression for Chow#1	Normalised expression for Chow#2	Normalised expression for Chow#3	Normalised expression for Chow#4	Normalised expression for HFD#1	Normalised expression for HFD#2	Normalised expression for HFD#3	Normalised expression for HFD#4
Cbx4	55.4352959	0.40575272	0.2960705	1.37045982	0.17054341	0.96010402		Cbx4	37.6345944	38.9170398	46.7576181	62.0442003	64.9276571	51.7463991	43.301875	98.1529838
Rnf214	83.3394661	0.30842889	0.22505551	1.37045692	0.17054432	0.96010402		Rnf214	67.2046328	73.3088425	71.5677828	84.7937404	73.2517157	107.020962	91.4150694	98.1529838
Zfp14	6.87797631	0.5440148	0.39703721	1.37018595	0.17062886	0.96010402		Zfp14	5.37637063	4.07271347	3.81694841	8.27256004	3.32962344	11.7605452	11.5471667	6.84788259
Bicc1	1.56250242	-0.5122151	0.37391735	-1.3698619	0.17073	NA		Bicc1	4.03227797	4.52523719	1.59039517	0	0	2.35210905	0	0
0610009O20Rik	20.0419153	-0.4843155	0.35360709	-1.3696432	0.1707983	0.96010402		0610009O20R	16.8011582	24.8888045	15.9039517	41.3628002	18.3129289	9.40843619	15.3962222	18.2610202
Sppl2a	20.8590592	-0.5457192	0.3985292	-1.3693329	0.17089523	0.96010402		Sppl2a	16.8011582	42.0847059	41.6683535	6.20442003	8.3240586	28.2253086	14.4339583	9.13051012
Fam115a	143.932572	0.42448743	0.3100564	1.3690652	0.17097889	0.96010402		Fam115a	102.823088	80.0966983	109.101109	186.132601	248.056946	169.351851	102.962236	152.936045
Jag2	17.5677776	0.54158872	0.39564771	1.36886605	0.17104115	0.96010402		Jag2	6.72046328	10.8605693	9.54237104	24.8176801	43.2851047	10.5844907	9.62263889	25.1089028
Il18	5.10570063	-0.5652919	0.41299492	-1.3687623	0.17107358	0.96010402		Il18	6.04841696	7.69290322	4.45310648	10.3407001	3.32962344	7.05632714	1.92452778	0
Kcnn2	13.2730709	-0.5289384	0.38651257	-1.3684895	0.1711589	0.96010402		Kcnn2	12.7688802	29.4140417	19.7209001	4.13628002	9.98887032	11.7605452	11.5471667	6.84788259
Tatdn2	31.2541903	0.44103794	0.32231661	1.36833759	0.17120643	0.96010402		Tatdn2	16.8011582	18.1009488	28.9451921	39.2946602	31.6314227	32.9295267	29.8301805	52.5004332
Slc25a39	91.9171731	-0.5217335	0.3812987	-1.3683065	0.17121616	0.96010402		Slc25a39	77.2853278	77.8340797	66.1604392	239.904241	86.5702095	28.2253086	45.2264028	114.131377
Gm973	2.78814215	-0.5689361	0.41585399	-1.368115	0.17127607	NA		Gm973	4.7043243	3.62018975	3.49886938	4.13628002	0	1.17605452	2.88679167	2.28262753
Rcn3	0.92809189	-0.4204905	0.30749754	-1.3674599	0.17148121	NA		Rcn3	2.01613899	0	1.27231614	4.13628002	0	0	0	0
Helb	2.74110952	0.56888553	0.41607684	1.36726074	0.17154359	NA		Helb	2.68818531	1.35757116	1.90847421	0	4.99443516	3.52816357	2.88679167	4.56525506
Plac9a	0.8790528	-0.4094631	0.29960589	-1.3666725	0.17172797	NA		Plac9a	1.34409266	3.62018975	0	2.06814001	0	0	0	0
Itih5	13.1154078	-0.5666239	0.41460243	-1.366668	0.17172938	0.96185675		Itih5	9.4086486	35.7493738	18.448584	6.20442003	16.6481172	12.9365998	0.96226389	4.56525506
Ccnt2	19.38441	-0.441812	0.32344497	-1.3659571	0.17195242	0.96185675		Ccnt2	18.8172972	24.8888045	26.7186389	22.7495401	13.3184938	21.1689814	18.2830139	9.13051012
Rspo3	3.81593066	-0.5653223	0.41388579	-1.3658896	0.17197362	NA		Rspo3	6.04841696	9.5029981	6.36158069	0	1.66481172	1.17605452	5.77358333	0
Ppp1r16a	30.8441075	0.47698253	0.34934518	1.36536171	0.17213939	0.96185675		Ppp1r16a	28.2259458	21.2686148	23.5378486	22.7495401	63.2628454	17.6408179	22.1320694	47.9351782
Slc9a9	5.59762809	-0.5621685	0.41196585	-1.3645997	0.17237888	0.96185675		Slc9a9	6.72046328	10.4080455	10.8146872	2.06814001	3.32962344	4.7042181	6.73584722	0
Optn	11.6697126	0.54042822	0.39607285	1.36446671	0.17242072	0.96185675		Optn	14.1129729	7.2403795	6.99773876	6.20442003	6.65924688	23.5210905	24.0565972	4.56525506
Ppme1	154.985356	0.40057923	0.29364214	1.36417488	0.17251253	0.96185675		Ppme1	122.312432	86.884554	112.918057	200.609581	219.755147	129.365998	144.339583	223.697498
Rsrp1	28.4179576	0.45525215	0.33373282	1.36412161	0.17252929	0.96185675		Rsrp1	29.5700384	17.1959013	23.2197695	22.7495401	36.6258578	54.0985081	27.9056528	15.9783927
Gfpt2	6.09656378	0.56373819	0.41366344	1.36279434	0.17294734	0.96185675		Gfpt2	2.68818531	1.81009488	6.67965972	4.13628002	3.32962344	7.05632714	4.81131944	18.2610202
Ppp1r14a	1.75607714	-0.5409691	0.39707024	-1.3624015	0.1730712	NA		Ppp1r14a	3.36023164	1.81009488	2.86271131	2.06814001	1.66481172	0	0	2.28262753
Xlr3a	1.37031141	0.48018216	0.35245804	1.36238109	0.17307765	NA		Xlr3a	0	0.90504744	0.9542371	0	3.32962344	0	5.77358333	0
Tomt	3.8662819	0.56182804	0.41249136	1.3620359	0.17318656	NA		Tomt	3.36023164	1.81009488	3.18079035	2.06814001	3.32962344	5.88027262	6.73584722	4.56525506
Sbk1	80.993652	0.4587582	0.33684209	1.36193846	0.17321731	0.96185675		Sbk1	57.7959842	52.9452751	59.7988585	88.9300204	99.8887032	74.091435	43.301875	171.197065
Wipi1	9.14940149	-0.5437287	0.39924689	-1.3618858	0.17323394	0.96185675		Wipi1	12.7688802	20.8160911	10.8146872	2.06814001	6.65924688	5.88027262	9.62263889	4.56525506
Bace2	2.02186951	0.55710004	0.40918569	1.36148467	0.17336057	NA		Bace2	1.34409266	1.35757116	1.59039517	0	3.32962344	4.7042181	3.84905555	0
Inpp5j	49.7836398	0.48437222	0.35586434	1.36111478	0.17347742	0.96185675		Inpp5j	35.6184554	28.961518	44.5310648	45.4990802	93.2294563	34.1055812	25.0188611	91.3051012
Cds2	595.861602	0.4172903	0.3065799	1.36111435	0.17347755	0.96185675		Cds2	331.31884	645.751347	468.848497	529.443843	792.450379	872.632457	325.245194	801.202263
Yap1	17.7448047	-0.4443732	0.32658069	-1.3606843	0.17361349	0.96185675		Yap1	22.1775288	24.4362808	20.9932163	16.5451201	13.3184938	10.5844907	20.2075417	13.6957652
Tmem151b	145.239614	0.50541128	0.37161242	1.36004948	0.17381427	0.96185675		Tmem151b	63.1723549	85.5269829	83.336707	213.018421	239.732888	129.365998	69.283	278.480559
Dpysl2	967.530997	0.40280581	0.29620665	1.35988106	0.17386757	0.96185675		Dpysl2	665.997911	602.30907	699.455797	1276.04239	1451.71582	829.118439	814.07525	1401.5333
Rapgef4	155.223673	0.47646841	0.3504293	1.35967058	0.17393419	0.96185675		Rapgef4	168.011582	123.991499	135.501669	62.0442003	129.855314	252.851723	289.64143	79.8919636
Telo2	11.6616338	0.56594811	0.41626575	1.35958366	0.17396171	0.96185675		Telo2	9.4086486	4.97776091	3.81694841	10.3407001	28.3017992	3.52816357	0.96226389	31.9567854
Tanc2	248.125654	0.34479665	0.25362073	1.35949713	0.17398911	0.96185675		Tanc2	191.533204	206.80334	201.980187	262.653781	391.230754	301.069958	199.188625	230.545381
Fbxl12	5.58260243	0.56515047	0.41571862	1.35945432	0.17400267	0.96185675		Fbxl12	2.01613899	1.35757116	3.81694841	6.20442003	6.65924688	1.17605452	2.88679167	20.5436478
Nr4a3	13.707512	-0.5395932	0.39693871	-1.3593867	0.17402409	0.96185675		Nr4a3	25.5377605	16.2908539	20.0389792	8.27256004	18.3129289	3.52816357	15.3962222	2.28262753
Gm3317	0.85466533	0.41945328	0.30865651	1.35896462	0.1741578	NA		Gm3317	0	0	0.63615807	0	0	2.35210905	3.84905555	0
Gm3488	0.85466533	0.41945328	0.30865651	1.35896462	0.1741578	NA		Gm3488	0	0	0.63615807	0	0	2.35210905	3.84905555	0
Dcaf17	8.50630364	0.52938227	0.38960581	1.35876377	0.17422146	0.96185675		Dcaf17	10.7527413	5.43028463	6.04350166	2.06814001	8.3240586	17.6408179	8.660375	9.13051012
Zfhx3	27.1880844	-0.5235273	0.38531958	-1.3586835	0.17424692	0.96185675		Zfhx3	16.1291119	29.8665654	20.6751372	72.3849004	34.9610461	17.6408179	14.4339583	11.4131377
Crat	24.9834628	-0.4647818	0.3421076	-1.3585838	0.17427851	0.96185675		Crat	40.3227797	34.8443264	25.1282437	20.6814001	9.98887032	21.1689814	31.7547083	15.9783927
Gpx1	48.4001837	-0.5287873	0.38924775	-1.3584852	0.17430979	0.96185675		Gpx1	45.6991503	43.442277	41.9864326	113.747701	23.3073641	9.40843619	36.5660278	73.044081
Herc3	145.650508	0.34442409	0.25360439	1.35811563	0.17442699	0.96185675		Herc3	143.817914	88.6946489	123.096586	150.974221	194.782971	121.133616	157.811278	184.89283
Pde8b	53.6831371	-0.4019413	0.29604899	-1.3576851	0.17456361	0.96185675		Pde8b	83.3337447	59.7331309	70.2954666	35.1583802	54.9387868	48.2182355	48.1131944	29.6741579
Gm13152	0.77791176	0.44390795	0.32718226	1.35676046	0.17485733	NA		Gm13152	0	0.45252372	0.31807903	0	0	3.52816357	1.92452778	0
Ndufs1	65.6684392	0.4408915	0.32505194	1.35637248	0.17498069	0.96185675		Ndufs1	69.8928182	49.3250854	62.9796488	31.0221002	41.620293	108.197016	102.962236	59.3483158
Slc5a7	1.89020011	-0.5324724	0.39278423	-1.355636	0.17521502	NA		Slc5a7	2.01613899	6.33533207	3.18079035	0	1.66481172	0	1.92452778	0
Gjb2	3.49845438	-0.5630538	0.41550004	-1.3551233	0.17537828	NA		Gjb2	3.36023164	9.05047438	4.45310648	2.06814001	0	3.52816357	0.96226389	4.56525506
Txndc17	11.4906791	-0.4912596	0.3625555	-1.3549914	0.17542029	0.96185675		Txndc17	12.7688802	10.8605693	14.3135566	18.6132601	4.99443516	7.05632714	9.62263889	13.6957652
Atxn7	15.2363857	0.47069363	0.34741052	1.35486291	0.17546124	0.96185675		Atxn7	10.7527413	11.7656167	16.8581888	8.27256004	23.3073641	23.5210905	18.2830139	9.13051012
Purb	397.74657	0.48415309	0.3574392	1.35450476	0.17557539	0.96185675		Purb	350.136137	379.214876	367.063206	146.837941	298.001298	636.245498	796.7545	207.719105
Zfp608	28.2802509	0.42729002	0.31547576	1.35443061	0.17559904	0.96185675		Zfp608	23.5216215	22.1736622	25.7644018	22.7495401	21.6425524	52.9224536	34.6415	22.8262753
BC005624	27.0339965	0.45865909	0.33872781	1.3540639	0.17571599	0.96185675		BC005624	16.1291119	17.1959013	17.8124259	37.2265202	34.9610461	19.9929269	25.0188611	47.9351782
Uqcrfs1	84.4110801	0.42889244	0.31675663	1.35401249	0.17573239	0.96185675		Uqcrfs1	46.3711967	47.0624668	70.6135457	115.815841	96.5590798	68.2111624	91.4150694	139.240279
Cisd3	10.1203342	-0.5014071	0.37031529	-1.3540005	0.17573621	0.96185675		Cisd3	10.7527413	13.1231878	10.8146872	14.4769801	6.65924688	4.7042181	6.73584722	13.6957652
Prrg3	36.5045009	0.42826368	0.31633631	1.35382398	0.17579254	0.96185675		Prrg3	26.2098068	41.6321821	24.1740066	26.8858201	51.6091633	54.0985081	24.0565972	43.3699231
Cenpw	1.97703728	0.53332486	0.39423389	1.35281332	0.17611528	NA		Cenpw	0.67204633	1.35757116	1.27231614	0	0	4.7042181	0.96226389	6.84788259
Bcl2l11	8.83485538	0.50607787	0.37419839	1.35243199	0.17623716	0.96185675		Bcl2l11	7.39250961	5.43028463	6.99773876	6.20442003	8.3240586	9.40843619	8.660375	18.2610202
Arhgef1	8.16687878	0.55098519	0.40752813	1.35201756	0.1763697	0.96185675		Arhgef1	5.37637063	4.97776091	6.67965972	4.13628002	18.3129289	4.7042181	2.88679167	18.2610202
Pik3r3	36.2464164	0.4568426	0.33793315	1.35187271	0.17641604	0.96185675		Pik3r3	33.6023164	28.0564706	39.4418003	14.4769801	29.966611	63.5069443	55.8113055	25.1089028
Tspan14	12.3285707	-0.5460288	0.40397029	-1.3516559	0.17648541	0.96185675		Tspan14	9.4086486	24.4362808	20.9932163	8.27256004	19.9777406	9.40843619	3.84905555	2.28262753
Ext1	23.1806211	-0.4393294	0.32508567	-1.3514266	0.17655882	0.96185675		Ext1	20.8334362	34.8443264	31.8079035	22.7495401	11.653682	23.5210905	24.0565972	15.9783927
Eif1	200.786465	-0.4127722	0.30553644	-1.3509754	0.1767033	0.96185675		Eif1	182.124555	221.736622	200.071713	335.038682	133.184938	94.0843619	186.679194	253.371656
Cep152	1.23369625	0.51051238	0.37792322	1.35083623	0.1767479	NA		Cep152	0	0.90504744	0.9542371	0	1.66481172	1.17605452	2.88679167	2.28262753
Pfkl	101.018664	0.5107956	0.37820261	1.35058721	0.1768277	0.96185675		Pfkl	79.3014668	46.6099431	52.1649617	128.224681	221.419959	47.042181	64.4716805	168.914437
Kdm6a	14.1304488	-0.5021038	0.37177078	-1.3505735	0.17683209	0.96185675		Kdm6a	18.8172972	19.0059962	24.8101647	6.20442003	4.99443516	15.2887088	12.5094306	11.4131377
Rnf165	102.242552	0.37475917	0.27749653	1.35050039	0.17685553	0.96185675		Rnf165	76.6132814	95.9350284	73.476257	101.338861	136.514561	84.6759257	82.7546944	166.63181
Tfam	24.1667258	0.43639155	0.32314981	1.35043112	0.17687773	0.96185675		Tfam	16.8011582	15.8383302	23.5378486	24.8176801	24.9721758	23.5210905	43.301875	20.5436478
Ripk1	1.3660822	-0.5154287	0.38173576	-1.3502238	0.1769442	NA		Ripk1	4.03227797	3.16766603	1.59039517	0	0	1.17605452	0.96226389	0
Wnt10a	1.3660822	-0.5154287	0.38173576	-1.3502238	0.1769442	NA		Wnt10a	4.03227797	3.16766603	1.59039517	0	0	1.17605452	0.96226389	0
Ankrd40	73.2183349	-0.4046409	0.29968684	-1.3502124	0.17694787	0.96185675		Ankrd40	110.215598	106.343074	79.5197586	43.4309402	46.6147282	62.3308898	77.943375	59.3483158
Nkx2-2	4.7034138	-0.5604497	0.41508382	-1.3502086	0.17694908	0.96185675		Nkx2-2	4.7043243	2.71514231	3.49886938	16.5451201	4.99443516	0	2.88679167	2.28262753
Rnmtl1	9.29907199	0.5269359	0.39031347	1.35003258	0.17700553	0.96185675		Rnmtl1	4.7043243	4.97776091	5.08926455	14.4769801	14.9833055	5.88027262	10.5849028	13.6957652
Mrpl37	26.8114073	-0.398229	0.29499406	-1.3499561	0.17703007	0.96185675		Mrpl37	28.2259458	29.4140417	35.9429309	31.0221002	24.9721758	16.4647633	27.9056528	20.5436478
Szt2	25.5279409	0.52110978	0.38626766	1.34908984	0.17730811	0.96185675		Szt2	17.4732045	12.6706641	17.1762679	28.9539601	61.5980337	21.1689814	8.660375	36.5220405
Mier2	23.0190761	0.52372882	0.38821864	1.34905636	0.17731886	0.96185675		Mier2	12.7688802	12.6706641	13.9954775	28.9539601	39.9554813	11.7605452	11.5471667	52.5004332
Klf8	3.65924797	-0.5608958	0.41586027	-1.3487603	0.17741398	NA		Klf8	4.7043243	5.88280835	5.08926455	4.13628002	3.32962344	0	3.84905555	2.28262753
Lrfn1	43.8757182	0.49915204	0.37008414	1.34875284	0.17741637	0.96185675		Lrfn1	22.8495752	33.0342315	28.9451921	49.6353602	74.9165274	35.2816357	17.32075	89.0224737
Cask	76.9327522	0.42157242	0.31265401	1.34836724	0.1775403	0.96185675		Cask	68.5487255	71.0462239	76.0208892	37.2265202	66.5924688	149.358925	82.7546944	63.9135709
Hdac4	47.0995177	0.42878539	0.31800363	1.34836632	0.17754059	0.96185675		Hdac4	36.2905017	38.9170398	30.5355873	49.6353602	83.240586	56.4506172	26.9433889	54.7830607
Acaa2	6.44703939	-0.5328884	0.39521409	-1.3483538	0.1775446	0.96185675		Acaa2	9.4086486	10.4080455	8.90621297	4.13628002	4.99443516	4.7042181	6.73584722	2.28262753
Snapin	32.0267504	-0.4929998	0.36567488	-1.3481917	0.17759673	0.96185675		Snapin	44.3550577	44.3473245	39.1237212	31.0221002	13.3184938	16.4647633	53.8867778	13.6957652
Prss53	3.31096271	0.55390172	0.41096199	1.34781739	0.17771712	NA		Prss53	0.67204633	2.26261859	2.22655324	2.06814001	9.98887032	4.7042181	0	4.56525506
Gli2	4.24685792	-0.5600491	0.41569725	-1.3472525	0.17789893	NA		Gli2	6.04841696	5.43028463	1.90847421	10.3407001	3.32962344	2.35210905	0	4.56525506
Psmb1	47.344523	0.36510322	0.27105693	1.34696138	0.17799267	0.96185675		Psmb1	53.7637063	36.6544212	36.2610099	35.1583802	44.9499164	45.8661264	64.4716805	61.6309433
Mfsd2a	12.7157688	-0.5518435	0.40974574	-1.3467951	0.17804624	0.96185675		Mfsd2a	6.04841696	28.5089943	17.8124259	12.4088401	9.98887032	3.52816357	2.88679167	20.5436478
Sla2	14.4908773	-0.4799181	0.35635735	-1.3467326	0.17806635	0.96185675		Sla2	13.4409266	12.2181404	19.0847421	26.8858201	13.3184938	7.05632714	12.5094306	11.4131377
Mrps6	19.2259414	0.43656046	0.3242227	1.34648334	0.17814669	0.96185675		Mrps6	13.4409266	17.1959013	12.0870033	20.6814001	23.3073641	15.2887088	22.1320694	29.6741579
Syn2	639.261061	0.34878608	0.25909201	1.34618615	0.17824249	0.96185675		Syn2	566.535055	504.111423	511.789167	628.714563	912.316823	496.295009	537.905514	956.420936
Rpl23	9.72937524	-0.4995698	0.37131388	-1.3454111	0.17849253	0.96185675		Rpl23	13.4409266	11.7656167	10.1785291	14.4769801	6.65924688	9.40843619	9.62263889	2.28262753
Ccdc85a	55.5305546	0.3104189	0.23077347	1.34512383	0.17858525	0.96185675		Ccdc85a	51.075521	50.6826565	43.5768277	51.7035003	63.2628454	58.8027262	63.5094166	61.6309433
Gpatch8	50.8240011	0.33145112	0.24644528	1.34492786	0.17864853	0.96185675		Gpatch8	43.010965	39.3695635	47.0756971	49.6353602	66.5924688	62.3308898	52.9245139	45.6525506
Il17ra	2.75154451	-0.5478285	0.40749299	-1.3443875	0.17882313	NA		Il17ra	4.03227797	7.69290322	3.49886938	0	3.32962344	1.17605452	0	2.28262753
Mrpl10	37.2379171	-0.3992438	0.29713084	-1.3436632	0.17905731	0.96185675		Mrpl10	39.6507334	38.0119924	52.1649617	43.4309402	24.9721758	21.1689814	44.2641389	34.239413
Lekr1	2.05247086	0.52025685	0.38721374	1.34359086	0.17908073	NA		Lekr1	0.67204633	0.90504744	2.22655324	0	0	5.88027262	6.73584722	0
Setbp1	38.125087	0.43109206	0.32103921	1.34280188	0.17933613	0.96185675		Setbp1	32.9302701	30.3190892	37.215247	24.8176801	49.9443516	68.2111624	43.301875	18.2610202
Pfkm	215.33805	0.45818893	0.34124923	1.3426812	0.17937522	0.96185675		Pfkm	181.452509	92.3148387	124.686982	293.675881	359.599332	155.239197	184.754667	330.980992
Mapre1	62.709102	0.31576725	0.23521783	1.34244609	0.1794514	0.96185675		Mapre1	56.4518916	58.8280835	53.4372778	53.7716403	66.5924688	74.091435	83.7169583	54.7830607
Lmf1	7.34194473	-0.5517488	0.41100719	-1.342431	0.17945627	0.96185675		Lmf1	3.36023164	13.5757116	7.31581779	14.4769801	9.98887032	3.52816357	1.92452778	4.56525506
Rfx2	2.56473309	-0.5317239	0.39611674	-1.3423413	0.17948535	NA		Rfx2	1.34409266	5.43028463	2.54463228	6.20442003	4.99443516	0	0	0
Fem1b	177.534776	0.30678637	0.22859562	1.34204831	0.17958033	0.96185675		Fem1b	142.473822	143.450019	179.078496	165.451201	149.833055	245.795396	218.433903	175.76232
Zxdb	22.1688572	0.47468918	0.35375894	1.34184362	0.17964671	0.96185675		Zxdb	10.7527413	13.5757116	17.8124259	28.9539601	41.620293	17.6408179	17.32075	29.6741579
Rasa3	50.2828509	-0.3797745	0.2830549	-1.341699	0.17969362	0.96185675		Rasa3	71.2369108	50.6826565	48.0299342	59.9760603	54.9387868	34.1055812	30.7924444	52.5004332
Pex5	32.6882132	0.51700346	0.38533763	1.34168954	0.17969668	0.96185675		Pex5	24.1936678	12.6706641	13.0412404	49.6353602	61.5980337	21.1689814	22.1320694	57.0656883
C030023E24Rik	0.78434726	0.39682725	0.29577391	1.34165737	0.17970712	NA		C030023E24Ri	0	0	0.31807903	0	4.99443516	0	0.96226389	0
Slco5a1	1.42269082	-0.5186619	0.386685	-1.3413034	0.17982197	NA		Slco5a1	3.36023164	3.16766603	2.22655324	0	1.66481172	0	0.96226389	0
Peak1	42.5293753	0.39209253	0.29234198	1.34121184	0.17985168	0.96185675		Peak1	38.3066407	39.8220873	36.2610099	28.9539601	63.2628454	62.3308898	46.1886666	25.1089028

GeneID	Base mean	log2(FC)	StdErr	Wald-Stats	P-value	P-adj	GeneID	Normalised expression for Chow#1	Normalised expression for Chow#2	Normalised expression for Chow#3	Normalised expression for Chow#4	Normalised expression for HFD#1	Normalised expression for HFD#2	Normalised expression for HFD#3	Normalised expression for HFD#4
Bri3bp	155.611922	0.38399739	0.28635118	1.34100158	0.17991994	0.96185675	Bri3bp	95.4305786	164.26611	125.32314	140.633521	216.425524	182.288451	99.1131805	221.414871
Atraid	19.7241284	-0.4495249	0.33528861	-1.3407103	0.18001454	0.96185675	Atraid	16.8011582	30.3190892	24.8101647	22.7495401	8.3240586	17.6408179	21.1698055	15.9783927
Ube2cbp	2.21021962	-0.5448827	0.40654736	-1.3402686	0.18015803	NA	Ube2cbp	1.34409266	3.16766603	2.54463228	6.20442003	0	1.17605452	0.96226389	2.28262753
Rp9	18.58698	0.54348138	0.40553521	1.34015832	0.18019388	0.96185675	Rp9	14.7850192	4.07271347	6.04350166	28.9539601	16.6481172	12.9365998	17.32075	47.9351782
A230103J11Rik	32.3138433	0.46523933	0.34720158	1.33996892	0.18025545	0.96185675	A230103J11Ri	29.5700384	16.7433776	23.2197695	33.0902402	58.2684102	25.8731995	19.2452778	52.5004332
Stk39	49.9035317	0.37266408	0.27817391	1.33968019	0.18034934	0.96185675	Stk39	44.3550577	43.8948007	47.3937761	35.1583802	43.2851047	59.9787807	81.7924305	43.3699231
Zkscan3	5.62607609	-0.542236	0.4047518	-1.3396754	0.18035091	0.96185675	Zkscan3	9.4086486	6.33533207	7.63389683	6.20442003	6.65924688	5.88027262	2.88679167	0
Mthfr	7.78637919	0.54895004	0.40978361	1.33960954	0.18037232	0.96185675	Mthfr	3.36023164	4.07271347	5.40734359	8.27256004	11.653682	7.05632714	1.92452778	20.5436478
Urgcp	67.4717256	0.48006887	0.35846821	1.33922301	0.18049808	0.96185675	Urgcp	45.6991503	33.0342315	43.2587487	90.9981605	138.179373	42.3379629	48.1131944	98.1529838
Adap1	98.8983128	0.33693076	0.25161323	1.33908203	0.18054397	0.96185675	Adap1	87.3660227	79.1916508	83.336707	93.0663005	139.844185	83.4998712	85.6414861	139.240279
Ppp2r5e	102.42282	0.32826596	0.24515152	1.33903293	0.18055995	0.96185675	Ppp2r5e	94.086486	80.549222	100.194896	84.7937404	89.8998329	154.063143	122.207514	93.5877288
Ranbp6	37.4190928	0.44599424	0.33308807	1.3389679	0.18058112	0.96185675	Ranbp6	40.994826	25.793852	35.6248519	18.6132601	28.3017992	65.8590533	56.7735694	27.3915304
Alx3	0.76388564	-0.4020416	0.30035395	-1.3385594	0.18071414	NA	Alx3	4.7043243	0.45252372	0.9542371	0	0	0	0	0
Slc22a8	7.97116257	-0.5566274	0.41591438	-1.3383221	0.18079145	0.96185675	Slc22a8	7.39250961	22.6261859	13.0412404	0	11.653682	3.52816357	0.96226389	4.56525506
Capn15	29.3144612	0.4919712	0.36761611	1.33827433	0.18080703	0.96185675	Capn15	14.7850192	16.7433776	19.0847421	41.3628002	54.9387868	22.345036	17.32075	47.9351782
Kirrel3	41.3145656	0.42760346	0.31952142	1.33826227	0.18081096	0.96185675	Kirrel3	27.5538995	50.6826565	33.0802196	22.7495401	49.9443516	71.739326	33.6792361	41.0872956
Wibg	13.7332	-0.5469741	0.4087708	-1.3380949	0.1808655	0.96185675	Wibg	12.7688802	6.33533207	10.4966081	43.4309402	4.99443516	5.88027262	7.69811111	18.2610202
Ift52	21.7033778	0.40649643	0.30385227	1.33780939	0.18095858	0.96185675	Ift52	16.8011582	17.648425	18.766663	20.6814001	18.3129289	25.8731995	32.7169722	22.8262753
Zfp862-ps	10.7672033	0.53916719	0.40312903	1.33745565	0.18107395	0.96185675	Zfp862-ps	4.7043243	9.5029981	5.40734359	10.3407001	18.3129289	4.7042181	5.77358333	27.3915304
Nefm	80.2388489	0.37792432	0.28259107	1.3373541	0.18110708	0.96185675	Nefm	69.8928182	44.3473245	66.1604392	95.1344405	108.212762	104.668853	64.4716805	89.0224737
Klhl12	33.8211591	0.40969355	0.30639541	1.33713997	0.18117695	0.96185675	Klhl12	30.9141311	16.7433776	24.8101647	43.4309402	39.9554813	36.4576902	39.4528194	38.804668
Rrp12	28.1060065	0.48334436	0.36154363	1.33689083	0.18125827	0.96185675	Rrp12	22.1775288	16.7433776	21.3112953	26.8858201	59.9332219	19.9929269	14.4339583	43.3699231
Prdm15	8.26659011	-0.5057928	0.37835199	-1.3368313	0.18127772	0.96185675	Prdm15	8.73660227	11.313093	10.4966081	10.3407001	9.98887032	5.88027262	4.81131944	4.56525506
Tchp	4.8345862	0.55646979	0.416303	1.33669416	0.18132249	0.96185675	Tchp	3.36023164	2.71514231	2.22655324	4.13628002	16.6481172	1.17605452	3.84905555	4.56525506
Pisd-ps1	5.28907097	-0.5443809	0.40728563	-1.3366072	0.18135089	0.96185675	Pisd-ps1	6.04841696	6.33533207	5.40734359	10.3407001	6.65924688	2.35210905	2.88679167	2.28262753
Boc	8.59317785	-0.5198973	0.38897197	-1.3365931	0.18135548	0.96185675	Boc	8.06455594	18.1009488	8.58813393	8.27256004	8.3240586	7.05632714	5.77358333	4.56525506
Slc25a25	53.4621738	0.41638114	0.31158444	1.33633484	0.18143985	0.96185675	Slc25a25	45.6991503	32.129184	36.2610099	64.1123403	76.5813391	39.9858538	46.1886666	86.7398462
Agmo	1.65683755	-0.4508716	0.33746601	-1.3360503	0.18153284	NA	Agmo	0	5.88280835	5.08926455	0	0	0	0	2.28262753
Vps52	59.4481035	0.4262601	0.31910166	1.33581285	0.18161045	0.96185675	Vps52	55.7798453	39.3695635	36.579089	64.1123403	101.553515	45.8661264	43.301875	89.0224737
Arid3b	0.70318556	-0.4346722	0.32540764	-1.3357776	0.18162199	NA	Arid3b	2.01613899	0.90504744	0.63615807	2.06814001	0	0	0	0
Zfp777	18.6550854	0.43768991	0.32769057	1.33568052	0.18165372	0.96185675	Zfp777	16.8011582	13.1231878	18.130505	10.3407001	26.6369875	15.2887088	19.2452778	29.6741579
Vcl	13.6928737	-0.478223	0.35807046	-1.3355555	0.18169459	0.96185675	Vcl	21.5054825	17.648425	17.8124259	8.27256004	6.65924688	15.2887088	8.660375	13.6957652
C130036L24Rik	0.90133412	0.42965411	0.32173523	1.33542762	0.18173643	NA	C130036L24Ri	0	0	0.63615807	0	3.32962344	0	0.96226389	2.28262753
Amd1	53.9788279	-0.4263466	0.31933235	-1.3351188	0.18183748	0.96185675	Amd1	71.9089571	81.9067931	65.8423601	35.1583802	38.2906696	55.2745626	60.622625	22.8262753
Frmd8	11.1902617	-0.5526215	0.41398083	-1.3348964	0.18191026	0.96185675	Frmd8	4.7043243	9.5029981	9.224292	37.2265202	13.3184938	3.52816357	2.88679167	9.13051012
Hspb8	10.9814225	-0.5028659	0.37671601	-1.3348674	0.18191973	0.96185675	Hspb8	14.1129729	13.1231878	8.90621297	20.6814001	8.3240586	11.7605452	8.660375	2.28262753
Nell1	24.7661319	0.49282188	0.36927178	1.3345777	0.1820146	0.96185675	Nell1	17.4732045	29.4140417	22.9016905	4.13628002	31.6314227	48.2182355	19.2452778	25.1089028
Igf2r	31.5618	0.44220136	0.33144037	1.33418073	0.18214463	0.96185675	Igf2r	28.8979921	29.8665654	28.3090341	12.4088401	43.2851047	49.39429	19.2452778	41.0872956
Frmpd1	7.93892148	-0.5114595	0.38337035	-1.3341134	0.18216671	0.96185675	Frmpd1	8.73660227	8.59795066	8.90621297	14.4769801	3.32962344	5.88027262	6.73584722	6.84788259
Tut1	16.6695359	0.48970548	0.3670934	1.33400787	0.18220128	0.96185675	Tut1	11.4247876	10.8605693	10.4966081	18.6132601	23.3073641	10.5844907	11.5471667	36.5220405
Ltbp4	30.2544715	0.53104908	0.39812714	1.33386806	0.1822471	0.96185675	Ltbp4	18.1452509	26.6988994	17.4943469	24.8176801	91.5646446	28.2253086	7.69811111	27.3915304
Ascc1	15.4770585	-0.5094346	0.38196586	-1.3337175	0.18229645	0.96185675	Ascc1	13.4409266	12.2181404	13.3593194	41.3628002	8.3240586	10.5844907	15.3962222	9.13051012
Samd1	23.4688513	-0.4528794	0.33968359	-1.3332391	0.18245334	0.96185675	Samd1	32.9302701	25.3413283	19.4028211	35.1583802	24.9721758	8.23238167	21.1698055	20.5436478
Ctgf	13.5008965	-0.5012398	0.37605096	-1.3329039	0.18256334	0.96185675	Ctgf	18.1452509	22.6261859	17.1762679	8.27256004	14.9833055	16.4647633	5.77358333	4.56525506
Kdm4a	29.7046855	0.41958175	0.31488564	1.3324893	0.18269945	0.96185675	Kdm4a	28.2259458	19.0059962	20.6751372	31.0221002	39.9554813	18.8168724	36.5660278	43.3699231
Ndc1	7.58092215	-0.534139	0.40088388	-1.3324032	0.18272773	0.96185675	Ndc1	5.37637063	15.3858064	13.3593194	4.13628002	4.99443516	7.05632714	5.77358333	4.56525506
P2rx3	1.67924482	-0.5270445	0.39557048	-1.3323657	0.18274004	NA	P2rx3	2.01613899	1.81009488	2.22655324	4.13628002	0	0	0.96226389	2.28262753
Bptf	97.7173105	0.42638074	0.32002418	1.33233914	0.18274877	0.96185675	Bptf	101.478996	79.6441745	82.3824699	57.9079203	86.5702095	201.105324	108.735819	63.9135709
Crb2	2.65334732	0.52959326	0.39754212	1.33216894	0.18280468	NA	Crb2	1.34409266	1.35757116	0.9542371	2.06814001	11.653682	0	3.84905555	0
Mtfp1	71.473668	0.46027901	0.34567661	1.33153066	0.18301446	0.96185675	Mtfp1	54.4357526	49.3250854	45.4853019	78.5893204	83.240586	36.4576902	64.4716805	159.783927
B3gnt7	1.26542267	0.4786512	0.35959198	1.33109532	0.18315765	NA	B3gnt7	0	0.90504744	0.63615807	0	1.66481172	2.35210905	0	4.56525506
Brwd1	71.0706347	0.32025497	0.24062849	1.33091044	0.18321848	0.96185675	Brwd1	63.1723549	56.1129411	62.0254117	70.3167603	56.6035985	94.0843619	81.7924305	84.4572186
Jade1	26.7933417	0.42960674	0.32282238	1.33078363	0.18326022	0.96185675	Jade1	29.5700384	21.7211385	23.2197695	12.4088401	23.3073641	48.2182355	30.7924444	25.1089028
Nudt14	4.72847496	-0.5528636	0.41548275	-1.3306536	0.18330303	0.96185675	Nudt14	5.37637063	3.62018975	1.90847421	16.5451201	4.99443516	1.17605452	1.92452778	2.28262753
Mxra8	1.41841271	-0.4828266	0.36292432	-1.3303781	0.18339373	NA	Mxra8	0.67204633	3.62018975	0.63615807	4.13628002	0	0	0	2.28262753
Crls1	19.2864591	-0.4457574	0.33507074	-1.3303381	0.18340689	0.96185675	Crls1	24.8657142	29.4140417	21.9474534	16.5451201	11.653682	17.6408179	23.0943333	9.13051012
Braf	158.413295	0.31007227	0.23310104	1.33020543	0.1834506	0.96185675	Braf	123.656524	147.070209	165.083019	124.088401	164.81636	215.217978	197.264097	130.109769
Gm266	0.63731875	-0.4147651	0.31185264	-1.3300036	0.18351709	NA	Gm266	2.01613899	1.81009488	1.27231614	0	0	0	0	0
Ss18l1	65.3326748	0.33368146	0.25089793	1.32994907	0.18353505	0.96185675	Ss18l1	61.1562159	52.0402277	52.1649617	62.0442003	88.2350212	52.9224536	67.3584722	86.7398462
Rab7	46.2549406	-0.4172534	0.31384123	-1.3295048	0.1836815	0.96185675	Rab7	81.3176057	47.0624668	50.2564875	39.2946602	26.6369875	39.9858538	55.8113055	29.6741579
St3gal2	72.9084309	0.36987036	0.27821305	1.32945007	0.18369953	0.96185675	St3gal2	58.4680306	64.7108918	62.0254117	62.0442003	124.860879	61.1548353	58.6980972	91.3051012
Actn4	31.1079599	0.50084227	0.37684156	1.32905266	0.1838306	0.96185675	Actn4	28.8979921	22.1736622	16.5401098	26.8858201	83.240586	17.6408179	19.2452778	34.239413
Cdc42se1	40.9653289	-0.3928149	0.29556866	-1.329014	0.18384336	0.96185675	Cdc42se1	61.1562159	47.5149905	38.1694841	41.3628002	46.6147282	22.345036	31.7547083	38.804668
Nsun5	9.02352737	0.53527375	0.40283499	1.32876677	0.18392493	0.96185675	Nsun5	4.03227797	4.52523175	5.40734359	12.4088401	16.6481172	7.05632714	3.84905555	18.2610202
Prkag1	37.3629589	0.40397345	0.30403336	1.32871423	0.18394227	0.96185675	Prkag1	38.978687	28.5089943	27.0367179	28.9539601	29.96611	31.7534722	45.2264028	68.4788259
Manba	6.37040833	0.54379949	0.40938275	1.32834004	0.1840658	0.96185675	Manba	4.7043243	4.97776091	5.40734359	4.13628002	3.32962344	18.8168724	4.81131944	6.84788259
Ndn	124.031389	0.32666317	0.24596061	1.32811171	0.1841412	0.96185675	Ndn	104.167181	90.0522201	92.24292	153.043261	128.190502	169.351851	125.094306	130.109769
Pomc	1.15783568	-0.3828866	0.28837505	-1.3277384	0.18426453	NA	Pomc	0.67204633	0	0.31807903	8.27256004	0	0	0	0
Cbfa2t2	63.8817286	0.33694265	0.25379632	1.32761044	0.18430684	0.96185675	Cbfa2t2	51.075521	50.6826565	56.9361472	64.1123403	79.9109626	48.2182355	77.943375	82.1745911
Ikbkg	32.0426613	0.38998897	0.29375594	1.32759518	0.18431188	0.96185675	Ikbkg	25.5377605	23.9837571	32.4440615	24.8176801	26.6369875	29.4013631	43.301875	50.2178057
Pp2d1	2.15478052	0.53187741	0.40069681	1.32738121	0.18438261	NA	Pp2d1	1.34409266	2.26261859	0.9542371	0	1.66481172	2.35210905	8.660375	0
Nt5e	2.54475356	-0.514753	0.38786537	-1.3271436	0.18446119	NA	Nt5e	3.36023164	4.97776091	7.31581779	0	0	4.7042181	0	0
Snrnp70	48.8510906	0.47503988	0.35795334	1.32710002	0.1844756	0.96185675	Snrnp70	31.5861774	25.793852	31.1717454	66.1804803	79.9109626	41.1619083	25.981125	89.0224737
Tgs1	15.5633074	0.43746398	0.32972201	1.3267661	0.18458607	0.96185675	Tgs1	15.4570656	11.7656167	13.3593194	10.3407001	14.9833055	24.697145	20.2075417	13.6957652
Rnf219	9.86491044	0.50885091	0.38356337	1.32664102	0.18462746	0.96185675	Rnf219	6.04841696	7.69290322	8.58813393	8.27256004	13.3184938	21.1689814	11.5471667	2.28262753
Spef1	2.66657785	-0.547939	0.41304783	-1.3265751	0.18464928	NA	Spef1	2.01613899	4.97776091	3.49886938	4.13628002	0	1.17605452	0.96226389	4.56525506
Fndc3b	17.507037	-0.4401381	0.33181762	-1.3264457	0.1846921	0.96185675	Fndc3b	20.1613899	15.3858064	19.7209001	28.9539601	9.98887032	14.1126543	13.4716944	18.2610202
Zmynd8	59.033645	0.31005043	0.23379393	1.32616968	0.1847835	0.96185675	Zmynd8	48.3873356	52.0402277	54.0734359	53.7716403	63.2628454	76.4435441	55.8113055	68.4788259
Heph	2.07313198	-0.5323306	0.40145917	-1.3259894	0.1848432	NA	Heph	3.36023164	4.07271347	5.08926455	0	0	1.17605452	2.88679167	0
Eif2b1	10.7914474	-0.5323936	0.40154433	-1.325865	0.18488441	0.96185675	Eif2b1	20.1613899	12.2181404	15.9039517	8.27256004	3.32962344	5.88027262	18.2830139	2.28262753
Dbndd2	40.4588218	-0.3401036	0.25653261	-1.3257715	0.18491541	0.96185675	Dbndd2	52.4196136	45.7048956	41.3502745	43.4309402	34.9610461	31.7534722	37.5282917	36.5220405
Mycbp2	226.82856	0.44729663	0.3374557	1.32549733	0.18500625	0.96185675	Mycbp2	184.81274	221.284099	207.70561	117.883981	259.710628	484.534464	222.282958	116.414004
Dtx2	5.15097838	-0.5483152	0.413745	-1.325249	0.18508856	0.96185675	Dtx2	5.37637063	3.62018975	5.08926455	14.4769801	1.66481172	3.52816357	2.88679167	4.56525506
Drd1a	17.7981606	-0.5288607	0.3991093	-1.3251025	0.18513716	0.96185675	Drd1a	18.1452509	30.7716129	29.2632712	12.4088401	4.99443516	30.5774176	4.81131944	11.4131377
Nfkbie	2.16395068	-0.526216	0.397294	-1.3245002	0.18533698	NA	Nfkbie	4.7043243	0.90504744	1.90847421	6.20442003	1.66481172	0	1.92452778	0
Josd2	11.0795562	-0.523638	0.39569214	-1.3233469	0.18572003	0.96185675	Josd2	8.73660227	11.7656167	9.224292	26.8858201	9.98887032	3.52816357	4.81131944	13.6957652
Leprot	7.47257051	-0.5349242	0.40423262	-1.3233079	0.18573299	0.96185675	Leprot	10.7527413	17.1959013	7.63389683	2.06814001	6.65924688	7.05632714	3.84905555	4.56525506
6430706D22Rik	21.4965218	0.45162486	0.34128613	1.32330116	0.18573525	0.96185675	6430706D22Ri	16.8011582	11.7656167	13.3593194	28.9539601	29.966611	17.6408179	19.2452778	34.239413
Ilvbl	8.5348048	0.53671882	0.40563088	1.3231705	0.18577868	0.96185675	Ilvbl	1.34409266	5.88280835	4.45310648	14.4769801	13.3184938	12.9365998	6.73584722	9.13051012
Gopc	41.4685775	0.38042462	0.28752745	1.32308977	0.18580553	0.96185675	Gopc	44.3550577	8.14542694	37.215247	28.9539601	29.966611	47.042181	57.7358333	57.0656883
B3glct	7.91766764	-0.538823	0.40729564	-1.3229284	0.1858592	0.96185675	B3glct	12.0968339	15.8383302	11.4508452	2.06814001	4.99443516	8.23238167	8.660375	0
Tmppe	17.7784999	0.53215184	0.40227709	1.32284899	0.1858856	0.96185675	Tmppe	6.72046328	18.1009488	11.1327662	14.4769801	11.653682	38.8097993	4.81131944	36.5220405
Clasrp	6.61255812	0.54414223	0.41136739	1.32276464	0.18591366	0.96185675	Clasrp	6.04841696	2.26261859	3.81694841	6.20442003	13.3184938	1.17605452	8.660375	11.4131377
Zfp882	6.57840816	-0.5299651	0.40072934	-1.3225014	0.18600125	0.96185675	Zfp882	8.73660227	9.95552182	9.86045007	6.20442003	3.32962344	5.88027262	8.660375	0
2810410L24Rik	3.23668505	-0.4716579	0.35664533	-1.3224844	0.18600689	NA	2810410L24Ri	0.67204633	0.45252372	1.59039517	18.6132601	0	0	0	4.56525506
Rpusd2	12.4742083	0.50173878	0.3794936	1.32212709	0.18612583	0.96185675	Rpusd2	4.7043243	5.88280835	10.1785291	18.6132601	13.3184938	11.7605452	12.5094306	22.8262753
Plxnc1	103.446474	0.48201392	0.36461001	1.32199861	0.18616861	0.96185675	Plxnc1	59.8121232	122.180114	101.467212	37.2265202	113.207197	239.915123	87.5660139	66.1961984
Tbc1d10b	115.245691	0.5260491	0.39793579	1.32194468	0.18618657	0.96185675	Tbc1d10b	52.4196136	52.4927514	50.8926455	179.928181	179.799666	48.2182355	68.3207361	289.893696
Apex2	3.61077128	-0.5417982	0.40988288	-1.3218367	0.18622255	NA	Apex2	2.01613899	4.07271347	1.27231614	14.4769801	1.66481172	1.17605452	1.92452778	2.28262753
Slc41a3	3.27124405	-0.5479677	0.41456424	-1.3217919	0.18623746	NA	Slc41a3	1.34409266	3.62018975	3.81694841	10.3407001	1.66481172	1.17605452	1.92452778	2.28262753
Ints9	9.00450471	-0.5141478	0.38900406	-1.321703	0.18626707	0.96185675	Ints9	14.1129729	14.0282353	10.4966081	6.20442003	11.653682	9.40843619	3.84905555	2.28262753
Cbs	6.24484755	-0.5502676	0.41633459	-1.3216957	0.18626951	0.96185675	Cbs	9.4086486	8.14542694	4.13502745	12.4088401	11.653682	0	1.92452778	2.28262753
Tmem191c	42.2126373	0.55022276	0.41637067	1.32147339	0.18634357	0.96185675	Tmem191c	21.5054825	11.7656167	10.4966081	64.1123403	71.586904	5.88027262	15.3962222	136.957652
Ifit2	7.78481395	0.54195161	0.41012105	1.32144307	0.18635367	0.96185675	Ifit2	6.72046328	6.78785578	6.04350166	2.06814001	6.65924688	17.6408179	16.3584861	0
Snd1	43.9088767	0.38631644	0.29235866	1.3213785	0.18637519	0.96185675	Snd1	30.2420848	31.2241366	34.6706148	35.1583802	64.9276571	37.6337448	39.4528194	59.3483158
Arhgap21	69.8944706	0.33965167	0.25705456	1.32132131	0.18639425	0.96185675	Arhgap21	53.0916599	67.8785578	60.1169375	64.1123403	84.9053977	96.436471	84.6792222	47.9351782
Ifi35	0.74458738	0.35431425	0.26816957	1.32123213	0.18642398	NA	Ifi35	0	0	0	0	4.99443516	0	0.96226389	0
Trim7	4.29380894	-0.549241	0.41574673	-1.3210952	0.18646961	NA	Trim7	5.37637063	6.33533207	8.2700549	2.06814001	0	3.52816357	1.92452778	6.84788259
A430033K04Rik	18.4834436	0.49362068	0.37373317	1.3207837	0.18657349	0.96185675	A430033K04Ri	17.4732045	11.313093	15.2677937	10.3407001	13.3184938	16.4647633	13.4716944	50.2178057
Faxc	128.196581	0.35162352	0.26624985	1.32065246	0.18661727	0.96185675	Faxc	110.215598	132.58945	129.140088	68.2486203	144.83862	186.992669	146.264111	107.283494
Smarca1	32.254513	0.47884346	0.36268889	1.32025952	0.18674839	0.96185675	Smarca1	34.9464091	28.0564706	27.0367179	10.3407001	21.6425524	52.9224536	62.5471528	20.5436478
Blnk	3.4084205	-0.54964	0.41637066	-1.3200738	0.18681038	NA	Blnk	3.36023164	5.88280835	4.77118552	4.13628002	1.66481172	0	2.88679167	4.56525506

GeneID	Base mean	log2(FC)	StdErr	Wald-Stats	P-value	P-adj
Tenm2	59.5847418	0.41913964	0.31751618	1.32005757	0.1868158	0.96185675
Amd2	53.8091315	-0.4204759	0.31855139	-1.3199626	0.18684751	0.96185675
Supv3l1	20.8023379	0.4730454	0.35838925	1.31992074	0.18686148	0.96185675
Tcte2	0.99209703	-0.4682986	0.3547988	-1.3198991	0.18686872	NA
Slc6a17	465.582195	0.40658838	0.3080578	1.31984446	0.18688696	0.96185675
H2-K1	3.39665246	-0.5495099	0.41636274	-1.3197865	0.18690632	NA
Esyt3	2.0233023	0.53546897	0.40578843	1.31957673	0.18697638	NA
Fgf13	179.050785	0.30313558	0.22974903	1.31942049	0.18702857	0.96185675
Nat10	12.8919051	-0.4737634	0.35909732	-1.3193175	0.18706298	0.96185675
Ppa1	41.4357514	0.39070134	0.2962324	1.31890145	0.18720206	0.96185675
Sep-05	517.097282	-0.3156108	0.23929891	-1.3188977	0.18720331	0.96185675
D030028A08Rik	3.93238747	0.53044104	0.40223625	1.31873007	0.18725937	NA
Gm10052	9.88875513	0.52836136	0.40067858	1.31866634	0.18728068	0.96185675
Smim1	25.4892808	-0.5048315	0.38294007	-1.3183043	0.18740182	0.96185675
C2cd4b	1.90893332	0.52117906	0.39537579	1.31818657	0.1874412	NA
Spire2	12.4745271	0.54024571	0.40986933	1.31809255	0.18747267	0.96185675
Serf2	65.2280593	-0.5224951	0.3964553	-1.3179168	0.1875315	0.96185675
Nop16	10.2424894	0.50226833	0.38111243	1.31790068	0.1875369	0.96185675
Csnk1g3	62.8779812	0.40327314	0.30601046	1.31784103	0.18755687	0.96185675
Pop5	20.3254113	-0.4478811	0.34028242	-1.3162041	0.18810556	0.96185675
Rps28	0.83793739	-0.4214377	0.32025339	-1.3159508	0.18819057	NA
Ap3s2	90.9838408	0.39278881	0.29850684	1.31584527	0.18822598	0.96185675
Ubac1	18.5897119	-0.421224	0.32015388	-1.3156923	0.18827734	0.96185675
Sar1a	83.7913436	0.27318949	0.20764564	1.31565244	0.18829072	0.96185675
Atp6v1b2	662.140022	0.40432775	0.30732706	1.31562692	0.18829929	0.96185675
Erich3	3.69477133	0.53742209	0.40867351	1.31504019	0.1884964	NA
Drap1	57.9541276	-0.4044821	0.30758663	-1.3150184	0.18850372	0.96185675
Elac2	16.1658125	0.43780853	0.33296711	1.31487021	0.18855353	0.96185675
Wdr13	122.728563	0.323194	0.24592471	1.31419893	0.18877927	0.96185675
Cx3cr1	11.2001697	-0.5353592	0.40742039	-1.3140217	0.18883889	0.96185675
4932416H05Rik	11.0866855	0.50215215	0.38224717	1.31368442	0.18895243	0.96185675
Ass1	6.10111993	0.54664126	0.41623908	1.31328673	0.18908635	0.96185675
Gabra5	30.7229366	-0.516434	0.39329506	-1.3130957	0.18915072	0.96185675
Tspan33	10.6157691	-0.5346553	0.4071786	-1.3130733	0.18915825	0.96185675
Hyou1	82.6203726	0.40409174	0.30776074	1.31300613	0.18918088	0.96185675
Pde4dip	145.75009	0.38415181	0.29258873	1.31294121	0.18920276	0.96185675
Srsf5	35.1394667	0.48646818	0.37055549	1.31280792	0.18924768	0.96185675
Rpl34-ps1	1.61713861	-0.511341	0.38957172	-1.3125723	0.18932712	NA
2900079G21Rik	9.08724504	-0.5139973	0.3917514	-1.3120498	0.18950334	0.96185675
Zfp697	24.9559741	0.38972747	0.29703979	1.31203793	0.18950734	0.96185675
Naf1	12.803595	-0.526681	0.4014789	-1.3118522	0.18957	0.96185675
Lrrk1	2.06835076	-0.5306974	0.40457909	-1.3117271	0.18961222	NA
Syvn1	38.1502136	-0.4696049	0.3580424	-1.3115902	0.18965844	0.96185675
Gla	8.01987768	0.53482566	0.40777371	1.31157467	0.18966369	0.96185675
Wipf3	151.454981	-0.3975124	0.30318621	-1.3111165	0.18981841	0.96185675
LOC100505025	0.67574524	-0.4264959	0.32535212	-1.3108748	0.18990008	NA
Tmem222	57.8847726	-0.4284988	0.32700559	-1.3103714	0.19007022	0.96185675
Perp	1.60157478	-0.4714791	0.35985321	-1.3101985	0.1901287	NA
Napb	522.234755	0.30749435	0.23470956	1.31010578	0.19016005	0.96185675
Mtch2	25.8531916	-0.4490897	0.3429059	-1.3096586	0.19031136	0.96185675
Pms1	5.46544739	0.53961571	0.41228195	1.30885116	0.19058477	0.96185675
Gck	1.74990512	-0.4410001	0.33697424	-1.3087056	0.1906341	NA
Cep162	4.64548364	0.53347336	0.40764645	1.30866675	0.19064726	NA
Ccdc171	1.59320345	0.51314967	0.39222312	1.30831061	0.19076798	NA
Tln2	100.280392	0.3470983	0.26531731	1.30823845	0.19079244	0.96185675
Tmem128	8.02618952	-0.5317634	0.40650504	-1.3081348	0.1908276	0.96185675
Kmt2a	123.093795	0.34902747	0.26684703	1.30796836	0.19088404	0.96185675
Rbl1	0.63898143	-0.3994589	0.30545593	-1.3077464	0.19095934	NA
C1qc	6.93488063	-0.5323672	0.40720054	-1.3073833	0.19108257	0.96185675
Nbea	222.129706	0.39989343	0.30590248	1.30725788	0.19112515	0.96185675
Efemp1	4.73480808	-0.5388796	0.4123506	-1.3068481	0.19126432	0.96185675
Kcnj9	48.5771129	0.4441944	0.33997723	1.30654163	0.19136843	0.96185675
Bmp4	0.92729813	-0.4565981	0.34948119	-1.3065026	0.1913817	NA
Gulp1	1.79595949	-0.5228491	0.40036948	-1.3059165	0.19158097	NA
Anks6	3.54892814	0.534354	0.40922663	1.30576544	0.19163233	NA
Cpne2	42.2384489	0.327206	0.25062827	1.30554308	0.19170799	0.96185675
Nagk	32.8739062	0.43018405	0.32956158	1.3053222	0.19178316	0.96185675
Chst12	13.7669672	-0.5098023	0.39058349	-1.3052327	0.19181364	0.96185675
Pdgfc	4.91004899	-0.5317188	0.40741199	-1.3051132	0.1918543	0.96185675
Mpst	13.9249052	-0.5069785	0.38846472	-1.3050826	0.19186474	0.96185675
Fam69b	21.9748953	-0.4374378	0.33522407	-1.3049115	0.19192297	0.96185675
Fam20a	6.1109748	-0.5324005	0.40800608	-1.3048838	0.19193243	0.96185675
Gm10653	1.47190389	0.46230228	0.35452094	1.30401968	0.19222687	NA
Utrn	50.7885119	0.36039536	0.2763753	1.30400712	0.19223116	0.96185675
Med18	2.39999559	-0.5375093	0.41228747	-1.3037247	0.19232748	NA
Greb1	1.70358975	0.49033099	0.37612639	1.30363357	0.19235855	NA
Echdc2	1.47892552	-0.4974573	0.38162847	-1.3035121	0.1924	NA
Lrfn5	42.0100236	-0.4896933	0.37571336	-1.3033693	0.19244871	0.96185675
Sec23a	117.435734	0.32823827	0.25185431	1.30328629	0.19247704	0.96185675
Sall3	5.63593295	-0.5425912	0.41634515	-1.3032245	0.19249814	0.96185675
Arap1	7.24283517	-0.5419226	0.41596093	-1.3028208	0.19263594	0.96185675
Arsj	1.42717685	-0.4996839	0.38354713	-1.3027965	0.19264423	NA
Snf8	9.4764074	-0.5150074	0.39538286	-1.3025536	0.1927272	0.96185675
Usmg5	4.87892911	-0.5401118	0.41467789	-1.3024852	0.19275057	0.96185675
Rab27b	66.6674367	0.51909588	0.39855206	1.30245437	0.19276111	0.96185675
Dennd1b	12.0948409	0.47708224	0.3663125	1.30239136	0.19278263	0.96185675
Camk2a	1590.66376	0.43047049	0.33054325	1.30231215	0.1928097	0.96185675
Rhoq	31.0902073	-0.4028534	0.30934339	-1.3022853	0.19281886	0.96185675
Rapgef2	159.401229	0.36877415	0.28322388	1.30205883	0.19289628	0.96185675
Cstf1	15.4365091	0.45333238	0.34816583	1.30205877	0.1928963	0.96185675
Nr2c1	4.8617342	-0.5325347	0.4090486	-1.3018861	0.19295534	0.96185675
Tek	0.95568464	-0.4584338	0.35219091	-1.3016629	0.19303166	NA
Neto2	65.6281371	0.52180669	0.40094682	1.30143618	0.1931092	0.96185675
Foxp2	19.6757163	-0.4928192	0.37868683	-1.3013898	0.19312507	0.96185675
Itga3	14.2586324	0.51000877	0.39191496	1.30132508	0.1931472	0.96185675
Dcaf4	13.0502863	-0.46177	0.35489102	-1.30116	0.19320368	0.96185675
Vkorc1l1	38.1013243	-0.4469326	0.34349981	-1.3011145	0.19321927	0.96185675
Pip4k2b	179.192832	0.35787001	0.27512935	1.30073367	0.19334963	0.96185675
Exoc3l	6.93348047	-0.5249918	0.40369693	-1.3004603	0.19344325	0.96185675
Prpf19	71.8627193	0.42060736	0.32345824	1.3003452	0.19348268	0.96185675
5031425E22Rik	6.13847031	0.53628985	0.4124533	1.30024379	0.19351743	0.96185675
Plrg1	16.8604242	-0.4273548	0.32870201	-1.3001284	0.19355696	0.96185675
Tube1	1.43458	-0.498673	0.38360286	-1.2999721	0.19361052	NA
Arntl2	1.77925385	0.51930402	0.3994878	1.2999246	0.19362681	NA
Taf1c	6.68919041	0.53950035	0.41503217	1.29990008	0.19363522	0.96185675
Shc3	45.8587693	0.3897517	0.29983976	1.29986664	0.19364668	0.96185675
Me1	12.1102398	-0.5208027	0.40065894	-1.2998654	0.19364711	0.96185675
Dgcr8	18.6404936	0.40348825	0.31044353	1.29971547	0.19369851	0.96185675
Mtmr14	7.8724851	-0.5224222	0.40203169	-1.2994553	0.19378772	0.96185675
Sv2b	221.10546	-0.5036842	0.38768182	-1.2992205	0.19386827	0.96185675
Abr	551.512079	0.39321687	0.30270283	1.29901947	0.19393725	0.96185675
Dact3	140.434741	0.45739508	0.35221092	1.29863967	0.19406762	0.9620569

GeneID	Normalised expression for Chow#1	Normalised expression for Chow#2	Normalised expression for Chow#3	Normalised expression for Chow#4	Normalised expression for HFD#1	Normalised expression for HFD#2	Normalised expression for HFD#3	Normalised expression for HFD#4
Tenm2	43.6830113	64.7108918	48.9841713	39.2946602	69.9220923	116.429398	54.8490416	38.804668
Amd2	71.9089571	80.549222	65.8423601	35.1583802	38.2906696	55.2745626	60.622625	22.8262753
Supv3l1	12.7688802	16.7433776	16.5401098	18.6132601	16.6481172	10.5844907	28.8679167	45.6525506
Tcte2	3.36023164	1.81009488	1.59039517	0	0	1.17605452	0	0
Slc6a17	269.490578	467.909525	390.919133	421.900562	705.880169	577.442771	240.565972	650.548846
H2-K1	4.03227797	4.97776091	6.67965972	2.06814001	4.99443516	1.17605452	0.96226389	2.28262753
Esyt3	0.67204633	1.35757116	0.9542371	2.06814001	3.32962344	5.88027262	1.92452778	0
Fgf13	156.586795	136.209639	156.494885	186.132601	149.833055	185.816615	201.113153	260.219539
Nat10	16.1291119	11.7656167	15.9039517	18.6132601	14.9833055	4.7042181	9.62263889	11.4131377
Ppa1	40.994826	30.3190892	39.7598793	26.8858201	29.966611	39.9858538	59.6603611	63.9135709
Sep-05	762.772583	577.420265	553.775599	417.764282	509.432386	359.872684	433.01875	522.721705
D030028A08Ri	0.67204633	1.35757116	5.40734359	0	6.65924688	8.23238167	0	9.13051012
Gm10052	10.7527413	3.62018975	11.4508452	2.06814001	11.653682	10.5844907	22.1320694	6.84788259
Smim1	23.5216215	22.6261859	20.9932163	59.9760603	11.653682	10.5844907	13.4716944	41.0872956
C2cd4b	0.67204633	0.45252372	0.9542371	2.06814001	0	2.35210905	1.92452778	6.84788259
Spire2	6.72046328	8.59795066	4.77118552	14.4769801	19.9777406	2.35210905	8.660375	34.239413
Serf2	45.027104	54.3028463	46.439539	186.132601	29.966611	27.0492541	27.9056528	105.000866
Nop16	6.04841696	6.78785578	6.67965972	12.4088401	11.653682	4.7042181	15.3962222	18.2610202
Csnk1g3	60.4841696	58.3755597	62.3434908	26.8858201	44.9499164	95.2604165	88.5282777	66.1961984
Pop5	16.1291119	27.1514231	20.3570582	35.1583802	11.653682	11.7605452	22.1320694	18.2610202
Rps28	1.34409266	0.90504744	0.31807903	4.13628002	0	0	0	0
Ap3s2	63.1723549	67.4260341	66.4785182	109.611421	101.553515	74.091435	78.9056389	166.63181
Ubac1	24.8657142	19.4585199	18.448584	24.8176801	18.3129289	15.2887088	11.5471667	15.9783927
Sar1a	73.2530498	74.2138899	79.2016796	74.4530404	88.2350212	101.140689	88.5282777	91.3051012
Atp6v1b2	473.792662	369.259355	503.837191	864.482524	880.6854	484.534464	622.584736	1097.94384
Erich3	5.37637063	0.45252372	2.54463228	0	3.32962344	8.23238167	9.62263889	0
Drap1	71.9089571	42.5372296	60.4350166	97.2025805	38.2906696	32.9295267	58.6980972	61.6309433
Elac2	16.8011582	11.7656167	13.9954775	8.27256004	23.3073641	21.1689814	13.4716944	20.5436478
Wdr13	129.032895	97.7451233	101.467212	99.2707205	163.151549	107.020962	110.660347	173.479692
Cx3cr1	9.4086486	30.7716129	14.6316356	2.06814001	11.653682	9.40843619	4.81131944	6.84788259
4932416H05Ri	6.72046328	9.05047438	5.40734359	12.4088401	13.3184938	7.05632714	9.62263889	25.1089028
Ass1	4.03227797	3.16766603	0.9542371	8.27256004	9.98887032	3.52816357	2.88679167	15.9783927
Gabra5	45.6991503	59.7331309	43.5768277	6.20442003	16.6481172	28.2253086	36.5660278	9.13051012
Tspan33	4.03227797	19.0059962	9.86045007	22.7495401	14.9833055	5.88027262	3.84905555	4.56525506
Hyou1	53.0916599	94.5774573	76.0208892	49.6353602	121.531256	125.837834	55.8113055	84.4572186
Pde4dip	135.081312	125.34907	139.318617	93.0663005	189.788536	244.619341	152.037694	86.7398462
Srsf5	22.8495752	14.480759	17.8124259	55.8397803	49.9443516	27.0492541	26.9433889	66.1961984
Rpl34-ps1	3.36023164	0.90504744	1.90847421	4.13628002	1.66481172	0	0.96226389	0
2900079G21Ri	11.4247876	13.1231878	9.54237104	10.3407001	6.65924688	1.17605452	6.73584722	13.6957652
Zfp697	22.1775288	20.8160911	23.2197695	18.6132601	26.6369875	35.2816357	34.6415	18.2610202
Naf1	14.7850192	10.8605693	15.5858727	26.8858201	1.66481172	18.8168724	11.5471667	2.28262753
Lrrk1	4.03227797	2.26261859	1.90847421	4.13628002	0	0	1.92452778	2.28262753
Syvn1	22.8495752	67.8785578	41.6683535	51.7035003	49.9443516	27.0492541	14.4339583	29.6741579
Gla	3.36023164	9.5029981	6.99773876	2.06814001	6.65924688	22.345036	8.660375	4.56525506
Wipf3	176.748184	238.932524	210.8864	74.4530404	138.179373	139.950488	118.358458	114.131377
LOC10050502	1.34409266	1.35757116	0.63615807	2.06814001	0	0	0	0
Tmem222	59.8121232	53.3977988	47.3937761	113.747701	46.6147282	30.5774176	38.4905555	73.044081
Perp	0	4.52523719	2.22655324	4.13628002	0	0	1.92452778	0
Napb	519.491812	457.954004	528.329276	339.174962	481.130587	670.351079	704.377166	477.069154
Mtch2	36.9625481	21.2686148	28.6271131	39.2946602	26.6369875	14.1126543	30.7924444	9.13051012
Pms1	4.7043243	3.16766603	2.54463228	6.20442003	3.32962344	7.05632714	14.4339583	2.28262753
Gck	0	1.35757116	0.63615807	10.3407001	1.66481172	0	0	0
Cep162	3.36023164	4.07271347	3.18079035	2.06814001	6.65924688	9.40843619	3.84905555	4.56525506
Ccdc171	0.67204633	0.90504744	0.31807903	2.06814001	3.32962344	3.52816357	1.92452778	0
Tln2	88.7101153	85.5269829	96.0598684	74.4530404	158.157113	123.485725	73.1320555	102.718239
Tmem128	11.4247876	11.7656167	14.9497146	4.13628002	3.32962344	7.05632714	11.5471667	0
Kmt2a	112.231737	97.7451233	96.0598684	122.020261	176.470042	179.936342	113.547139	86.7398462
Rbl1	1.34409266	0.90504744	2.86271131	0	0	0	0	0
C1qc	8.73660227	17.1959013	13.6773985	0	9.98887032	5.88027262	0	0
Nbea	196.237528	200.015484	214.385269	132.360961	273.029122	375.161393	269.433889	116.414004
Efemp1	4.03227797	11.313093	5.40734359	4.13628002	3.32962344	3.52816357	3.84905555	2.28262753
Kcnj9	32.9302701	41.1796584	32.4440615	49.6353602	81.5757743	42.3379629	24.0565972	84.4572186
Bmp4	2.01613899	3.16766603	1.27231614	0	0	0	0.96226389	0
Gulp1	3.36023164	4.97776091	2.22655324	0	1.66481172	1.17605452	0.96226389	0
Anks6	2.01613899	4.07271347	1.27231614	0	11.653682	0	4.81131944	4.56525506
Cpne2	35.6184554	34.8443264	38.8056422	37.2265202	48.2795399	44.6900719	41.3773472	57.0656883
Nagk	24.1936678	17.1959013	26.0824808	41.3628002	54.9387868	24.697145	28.8679167	45.6525506
Chst12	5.37637063	29.8665654	18.448584	14.4769801	9.98887032	10.5844907	7.69811111	13.6957652
Pdgfc	4.7043243	7.69290322	5.40734359	8.27256004	3.32962344	4.7042181	2.88679167	2.28262753
Mpst	11.4247876	10.4080455	12.7231614	37.2265202	14.9833055	5.88027262	9.62263889	9.13051012
Fam69b	20.1613899	33.0342315	20.0389792	31.0221002	23.3073641	9.40843619	18.2830139	20.5436478
Fam20a	8.73660227	19.0059962	7.31581779	0	0	10.5844907	0.96226389	2.28262753
Gm10653	0	0	0.63615807	2.06814001	3.32962344	1.17605452	0	4.56525506
Utrn	45.6991503	40.7271347	47.3937761	41.3628002	78.2461509	56.4506172	64.4716805	31.9567854
Med18	2.01613899	2.71514231	3.49886938	6.20442003	1.66481172	1.17605452	1.92452778	0
Greb1	0.67204633	0.45252372	0.31807903	2.06814001	6.65924688	1.17605452	0	2.28262753
Echdc2	2.68818531	1.81009488	1.27231614	4.13628002	0	0	1.92452778	0
Lrfn5	39.6507334	76.4765085	61.0711746	31.0221002	14.9833055	55.2745626	46.1886666	11.4131377
Sec23a	120.296293	109.963264	114.826531	62.0442003	106.54795	145.830761	145.301847	134.675024
Sall3	4.03227797	11.7656167	4.13502745	10.3407001	8.3240586	0	1.92452778	4.56525506
Arap1	6.04841696	8.59795066	5.08926455	20.6814001	13.3184938	0	1.92452778	2.28262753
Arsj	4.03227797	3.16766603	1.59039517	0	1.66481172	0	0.96226389	0
Snf8	12.7688802	15.3858064	10.4966081	10.3407001	3.32962344	3.52816357	15.3962222	4.56525506
Usmg5	6.04841696	1.35757116	5.72542262	14.4769801	3.32962344	3.52816357	0	4.56525506
Rab27b	66.5325865	49.7776091	62.9796488	14.4769801	36.6258578	132.894161	147.226375	22.8262753
Dennd1b	10.7527413	7.69290322	9.86045007	10.3407001	9.98887032	9.40843619	25.0188611	13.6957652
Camk2a	1366.94223	1301.00569	1191.84214	1333.95031	3278.01428	1020.81533	947.82993	2284.91016
Rhoq	33.6023164	43.8948007	29.8994292	39.2946602	31.6314227	18.8168724	35.6037639	15.9783927
Rapgef2	166.667489	145.260114	142.499407	88.9300204	191.453348	249.323559	183.792403	107.283494
Cstf1	10.7527413	12.2181404	15.5858727	8.27256004	14.9833055	15.2887088	14.4339583	31.9567854
Nr2c1	4.03227797	8.14542694	8.2700549	4.13628002	3.32962344	3.52816357	2.88679167	4.56525506
Tek	1.34409266	2.26261859	2.86271131	0	0	1.17605452	0	0
Neto2	44.3550577	64.2583681	62.0254117	18.6132601	23.3073641	184.64056	98.1509166	29.6741579
Foxp2	27.5538995	28.0564706	30.8536663	10.3407001	4.99443516	29.4013631	12.5094306	13.6957652
Itga3	8.73660227	12.6706641	9.224292	10.3407001	26.6369875	12.9365998	3.84905555	29.6741579
Dcaf4	20.1613899	13.5757116	16.5401098	12.4088401	9.98887032	5.88027262	14.4339583	11.4131377
Vkorc1l1	36.2905017	64.7108918	54.7095939	26.8858201	18.3129289	42.3379629	43.301875	18.2610202
Pip4k2b	139.11359	132.58945	149.497146	194.405161	244.727323	172.880015	124.132042	276.197931
Exoc3l	6.72046328	9.05047438	5.72542262	14.4769801	6.65924688	1.17605452	4.81131944	6.84788259
Prpf19	55.7798453	40.274611	52.4830407	88.9300204	96.5590798	48.2182355	62.5471528	130.109769
5031425E22Ri	6.04841696	2.26261859	2.54463228	6.20442003	11.653682	7.05632714	1.92452778	11.4131377
Plrg1	23.5216215	19.9110436	20.0389792	16.5451201	9.98887032	19.9929269	13.4716944	11.4131377
Tube1	1.34409266	3.16766603	2.54463228	2.06814001	0	2.35210905	0	0
Arntl2	2.01613899	0.45252372	1.27231614	0	3.32962344	2.35210905	4.81131944	0
Taf1c	4.7043243	3.16766603	5.08926455	4.13628002	19.9777406	1.17605452	3.84905555	11.4131377
Shc3	43.010965	45.2523719	33.0802196	33.0902402	63.2628454	76.4435441	38.4905555	34.239413
Me1	17.4732045	20.8160911	14.3135566	10.3407001	9.98887032	4.7042181	19.2452778	0
Dgcr8	14.1129729	14.480759	16.8581888	16.5451201	16.6481172	19.9929269	23.0943333	27.3915304
Mtmr14	9.4086486	6.33533207	6.04350166	20.6814001	3.32962344	5.88027262	6.73584722	4.56525506
Sv2b	252.017373	483.295332	328.257564	41.3628002	181.464478	211.689814	170.320708	100.435611
Abr	411.292353	400.483491	380.422525	661.804803	972.250045	456.309155	394.528194	735.006065
Dact3	100.806949	100.460266	90.6525248	155.110501	249.721758	67.0351079	97.1886527	262.502166

GeneID	Base mean	log2(FC)	StdErr	Wald-Stats	P-value	P-adj		GeneID	Normalised expression for Chow#1	Normalised expression for Chow#2	Normalised expression for Chow#3	Normalised expression for Chow#4	Normalised expression for HFD#1	Normalised expression for HFD#2	Normalised expression for HFD#3	Normalised expression for HFD#4
Rnmt	42.2600621	0.41516521	0.3197665	1.29833865	0.19417099	0.96212312		Rnmt	32.9302701	32.5817078	49.3022503	24.8176801	36.6258578	59.9787807	72.1697916	29.6741579
Kctd14	2.47941682	0.53016486	0.4084587	1.29796441	0.19429956	NA		Kctd14	1.34409266	1.35757116	0.9542371	2.06814001	6.65924688	0	2.88679167	4.56525506
Gp5	1.52671974	0.49316548	0.38007884	1.2975347	0.19444728	NA		Gp5	0.67204633	0.90504744	0	2.06814001	3.32962344	2.35210905	2.88679167	0
Zfhx4	15.0192181	-0.5342303	0.41180579	-1.297287	0.19453247	0.96307102		Zfhx4	14.7850192	18.5534725	16.5401098	31.0221002	3.32962344	28.2253086	7.69811111	0
Prkx	9.1059762	-0.4947674	0.38139486	-1.2972578	0.19454251	0.96307102		Prkx	15.4570656	13.5757116	8.90621297	6.20442003	9.98887032	7.05632714	4.81131944	6.84788259
Ccl27b	3.02028853	0.53994217	0.41630312	1.29699284	0.19463365	NA		Ccl27b	2.68818531	0.90504744	1.90847421	2.06814001	3.32962344	3.52816357	2.88679167	6.84788259
Gm13306	3.02028853	0.53994217	0.41630312	1.29699284	0.19463365	NA		Gm13306	2.68818531	0.90504744	1.90847421	2.06814001	3.32962344	3.52816357	2.88679167	6.84788259
LOC100861978	3.02028853	0.53994217	0.41630312	1.29699284	0.19463365	NA		LOC10086197	2.68818531	0.90504744	1.90847421	2.06814001	3.32962344	3.52816357	2.88679167	6.84788259
Arrdc2	3.72032133	0.53979962	0.4162357	1.29686047	0.1946792	NA		Arrdc2	1.34409266	2.26261859	2.22655324	4.13628002	9.98887032	2.35210905	2.88679167	4.56525506
Sltm	24.2754759	0.52584172	0.40555168	1.29660842	0.19476595	0.96373079		Sltm	19.4893435	16.7433776	20.9932163	12.4088401	4.99443516	62.3308898	48.1131944	9.13051012
Sgk1	39.6126063	-0.441227	0.34036347	-1.2963406	0.19485816	0.96374087		Sgk1	38.978687	69.236129	56.6180681	22.7495401	18.3129289	30.5774176	46.1886666	34.239413
Slc46a1	11.8028452	-0.504039	0.38897985	-1.2957972	0.19504536	0.96422053		Slc46a1	12.7688802	14.480759	10.4966081	20.6814001	13.3184938	7.05632714	1.92452778	13.6957652
A430105I19Rik	3.82712469	0.51868743	0.40037691	1.29549786	0.19514854	NA		A430105I19Ri	1.34409266	0.45252372	0.9542371	6.20442003	8.3240586	0	1.92452778	11.4131377
Rtl1	4.74492593	0.51171826	0.39512161	1.29509054	0.195289	0.96497864		Rtl1	0	2.71514231	0	8.27256004	13.3184938	2.35210905	6.73584722	4.56525506
Ipcef1	42.7922265	0.41306813	0.31917879	1.29415907	0.19561048	0.96555186		Ipcef1	30.9141311	50.6826565	41.3502745	16.5451201	58.2684102	45.8661264	64.4716805	34.239413
Rps10	4.33381681	-0.5316843	0.41085474	-1.2940932	0.19563324	NA		Rps10	7.39250961	4.97776091	4.77118552	6.20442003	1.66481172	3.52816357	3.84905555	2.28262753
Rbm7	12.611542	-0.474546	0.36673167	-1.2939869	0.19566996	0.96555186		Rbm7	20.8334362	12.6706641	12.7231614	16.5451201	3.32962344	14.1126543	11.5471667	9.13051012
Emx2	6.69050089	-0.5341596	0.412807	-1.2939693	0.19567602	0.96555186		Emx2	8.73660227	10.8605693	9.86045007	6.20442003	4.99443516	0	10.5849028	2.28262753
Ttc32	0.94336508	-0.457968	0.35397068	-1.2938021	0.19573379	NA		Ttc32	2.01613899	1.81009488	2.54463228	0	0	1.17605452	0	0
Atxn2	138.030916	-0.3032915	0.23446776	-1.2935318	0.1958272	0.96563328		Atxn2	191.533204	163.813586	153.950253	103.407001	128.190502	127.013889	122.207514	114.131377
Scyl3	14.8062895	0.45363887	0.35073399	1.29339865	0.19587322	0.96563328		Scyl3	10.7527413	8.59795066	16.8581888	10.3407001	11.653682	15.2887088	22.1320694	22.8262753
2700097O09Rik	2.26800183	-0.5281807	0.40854647	-1.292829	0.19607022	NA		2700097O09R	5.37637063	1.81009488	2.54463228	4.13628002	0	2.35210905	1.92452778	0
Spata13	13.8209895	-0.4595513	0.35547682	-1.2927742	0.19608915	0.96625212		Spata13	16.1291119	19.9110436	11.4508452	18.6132601	9.98887032	11.7605452	6.73584722	15.9783927
Tmem209	21.1522526	-0.448771	0.347327	-1.2920707	0.19633266	0.96673754		Tmem209	16.1291119	38.0119924	28.3090341	18.6132601	16.6481172	24.697145	15.3962222	11.4131377
4931406P16Rik	15.9036143	-0.4198215	0.32494752	-1.291967	0.19636856	0.96673754		4931406P16Ri	22.1775288	17.648425	17.1762679	18.6132601	11.653682	14.1126543	14.4339583	11.4131377
Oaf	3.69355424	-0.5378716	0.41634915	-1.2918763	0.19639997	NA		Oaf	6.04841696	6.33533207	7.63389683	0	3.32962344	2.35210905	3.84905555	0
Gamt	6.46433772	-0.5286493	0.40949202	-1.2909879	0.19670786	0.96715288		Gamt	7.39250961	4.97776091	6.67965972	14.4769801	4.99443516	1.17605452	2.88679167	9.13051012
Agap1	165.991373	0.34278713	0.26552364	1.29098536	0.19670875	0.96715288		Agap1	120.968339	134.399545	116.098848	206.814001	224.749582	190.520833	128.943361	205.436478
Tgfbi	1.16696016	-0.4595597	0.35605441	-1.2907008	0.19680746	NA		Tgfbi	0.67204633	2.71514231	0.63615807	4.13628002	0	1.17605452	0	0
A630075F10Rik	0.65893965	-0.4200388	0.32551167	-1.2903957	0.19691331	NA		A630075F10Ri	1.34409266	0.90504744	0.9542371	2.06814001	0	0	0	0
Dnajc30	50.320928	-0.4519436	0.35024662	-1.2903581	0.19692635	0.96715288		Dnajc30	47.043243	49.3250854	42.3045116	103.407001	36.6258578	18.8168724	36.5660278	68.4788259
Arhgap26	131.184729	0.31865682	0.24702797	1.28996254	0.19706366	0.96715288		Arhgap26	110.215598	117.203643	128.822009	105.475141	128.190502	206.985596	145.301847	107.283494
Mar-05	47.6716077	0.36173393	0.28043093	1.28992165	0.19707786	0.96715288		Mar-05	46.3711967	42.5372296	41.9864326	31.0221002	54.9387868	35.2816357	72.1697916	57.0656883
Nkain1	41.6990103	-0.3939654	0.30545854	-1.2897508	0.19713721	0.96715288		Nkain1	34.2743627	60.1856546	38.4875632	59.9760603	34.9610461	24.697145	30.7924444	50.2178057
Hrsp12	9.9190354	-0.5369615	0.41635646	-1.2896677	0.19716605	0.96715288		Hrsp12	14.7850192	21.7211385	17.8124259	0	3.32962344	8.23238167	13.4716944	0
Slc35a1	5.97488916	0.52679291	0.40851273	1.28953855	0.19721092	0.96715288		Slc35a1	4.7043243	6.33533207	4.77118552	0	3.32962344	14.1126543	7.69811111	6.84788259
Bola1	5.06247511	0.53130047	0.41214122	1.2891224	0.19735554	0.96715288		Bola1	1.34409266	2.71514231	2.86271131	8.27256004	6.65924688	4.7042181	4.81131944	9.13051012
Ly6c1	7.68196917	-0.4064864	0.31532186	-1.2891158	0.19735783	0.96715288		Ly6c1	1.34409266	38.9170398	13.3593194	0	6.65924688	1.17605452	0	0
Zfp647	1.36151758	0.48387218	0.37551727	1.28854839	0.19755513	NA		Zfp647	0	0.45252372	0.63615807	2.06814001	0	3.52816357	1.92452778	2.28262753
Eif3c	94.9852957	0.30620783	0.23764246	1.28852323	0.19756389	0.96771899		Eif3c	87.3660227	78.2866034	96.0598684	74.4530404	101.553515	136.422325	105.849028	79.8919636
Fam84a	98.2446804	0.32298383	0.25074441	1.28809984	0.19771121	0.96796828		Fam84a	71.2369108	71.9512713	83.6547861	119.952121	106.54795	91.7322529	103.9245	136.957652
Sirt3	33.233054	0.35547852	0.27602339	1.28785652	0.19779591	0.96796828		Sirt3	25.5377605	26.6988994	30.8536663	31.0221002	39.9554813	27.0492541	41.3773472	43.3699231
Trp53i13	3.33755541	-0.5347342	0.4154455	-1.2871343	0.19804747	NA		Trp53i13	5.37637063	3.16766603	3.49886938	6.20442003	4.99443516	1.17605452	0	2.28262753
Slc27a4	49.20431	0.44235711	0.34372886	1.28693621	0.19811652	0.96892031		Slc27a4	30.2420848	48.4200379	38.8056422	39.2946602	83.240586	47.042181	22.1320694	84.4572186
Igfn1	7.66537486	0.53570134	0.41631225	1.28677776	0.19817176	0.96892031		Igfn1	8.73660227	6.33533207	2.22655324	2.06814001	21.6425524	16.4647633	3.84905555	0
Xrra1	1.27676461	-0.4657183	0.3620531	-1.2863259	0.19832935	NA		Xrra1	2.68818531	0.45252372	1.27231614	4.13628002	1.66481172	0	0	0
Lrch4	16.1742767	0.51960281	0.40404856	1.28599099	0.19844621	0.9698185		Lrch4	6.72046328	7.69290322	5.08926455	28.9539601	21.6425524	14.1126543	8.660375	36.5220405
Tmem143	12.3663325	-0.4702348	0.36574392	-1.2856941	0.19854985	0.96988155		Tmem143	17.4732045	10.4080455	13.9954775	18.6132601	14.9833055	4.7042181	9.62263889	9.13051012
Mafk	7.34365819	0.50481922	0.39280805	1.28515497	0.19873814	0.96997443		Mafk	8.06455594	4.52523719	3.18079035	6.20442003	8.3240586	7.05632714	7.69811111	13.6957652
Hepacam2	0.86778832	0.38859924	0.30239768	1.28506026	0.19877123	NA		Hepacam2	0	0.45252372	0	0	0	0	1.92452778	4.56525506
Urb2	13.6932355	0.46292832	0.36029149	1.28487165	0.19883714	0.96997443		Urb2	10.7527413	14.0282353	12.0870033	4.13628002	18.3129289	22.345036	9.62263889	18.2610202
Foxr2	0.60987842	-0.3951511	0.30755241	-1.2848252	0.19885336	NA		Foxr2	1.34409266	2.26261859	1.27231614	0	0	0	0	0
Got1	231.473129	0.33077256	0.2574473	1.28481657	0.19885639	0.96997443		Got1	215.054825	150.237875	198.163238	244.040521	271.36431	192.872942	219.396167	360.65515
Dleu7	8.88182299	0.50086004	0.38989553	1.28460062	0.19893188	0.96997443		Dleu7	6.72046328	4.07271347	6.99773876	10.3407001	4.99443516	8.23238167	18.2830139	11.4131377
E430018J23Rik	2.49849377	0.51438798	0.40062808	1.28395389	0.19915809	NA		E430018J23Ri	2.01613899	0.90504744	1.90847421	0	9.98887032	0	2.88679167	2.28262753
Ndufv3	42.6662637	-0.4231482	0.32959235	-1.2838532	0.19919334	0.97080638		Ndufv3	49.059382	33.9392789	45.4853019	74.4530404	16.6481172	28.2253086	43.301875	50.2178057
Lacc1	3.43639422	-0.5193879	0.40471134	-1.2833538	0.19936814	NA		Lacc1	5.37637063	1.81009488	1.90847421	12.4088401	0	1.17605452	4.81131944	0
Myh8	1.25027141	0.45611886	0.35543522	1.28326861	0.19939799	NA		Myh8	0	0.45252372	0.31807903	2.06814001	0	2.35210905	4.81131944	0
Fen1	6.29563334	0.5132109	0.39993097	1.28324871	0.19940496	0.97105192		Fen1	3.36023164	3.62018975	6.67965972	4.13628002	13.3184938	4.7042181	7.69811111	6.84788259
Fam162a	9.28937334	-0.5122374	0.39930007	-1.2828383	0.19954874	0.97105192		Fam162a	19.4893435	12.6706641	11.4508452	4.13628002	3.32962344	9.40843619	11.5471667	2.28262753
Sep-15	68.1746255	-0.4521709	0.35256981	-1.2825002	0.19966722	0.97105192		Sep-15	76.6132814	109.963264	78.8836006	64.1123403	26.6369875	89.3801438	76.9811111	22.8262753
Zfp438	4.3673626	-0.5278326	0.41169467	-1.2820973	0.19980851	NA		Zfp438	7.39250961	4.52523719	5.40734359	6.20442003	4.99443516	3.52816357	2.88679167	0
Tspyl1	184.831391	0.28772497	0.2245205	1.2815087	0.20001505	0.97105192		Tspyl1	141.801775	128.516736	174.307311	219.222841	206.436653	206.985596	198.226361	203.15385
Rsbn1	36.9710294	0.45617079	0.35598442	1.28143471	0.20004102	0.97105192		Rsbn1	34.9464091	30.3190892	33.7163777	18.6132601	18.3129289	77.6195986	54.8490416	27.3915304
Nudt16	6.43968729	0.52127371	0.40679052	1.28143033	0.20004256	0.97105192		Nudt16	4.03227797	4.52523719	4.45310648	6.20442003	9.98887032	2.35210905	15.3962222	4.56525506
Phtf1os	10.0897472	0.48972802	0.38217804	1.28141329	0.20004854	0.97105192		Phtf1os	6.72046328	7.2403795	9.54237104	8.27256004	4.99443516	18.8168724	18.2830139	6.84788259
Herpud1	22.7247378	-0.4737917	0.36988501	-1.2809162	0.2002231	0.97105192		Herpud1	28.8979921	44.7998482	25.7644018	10.3407001	18.3129289	29.4013631	10.5849028	13.6957652
Kcna3	31.2885638	0.46922181	0.36632236	1.28089861	0.20022928	0.97105192		Kcna3	14.7850192	20.8160911	19.0847421	45.4990802	38.2906696	51.7463991	14.4339583	45.6525506
Zfp646	19.0761123	0.42682698	0.33326497	1.28074359	0.20028374	0.97105192		Zfp646	11.4247876	17.648425	20.6751372	10.3407001	31.6314227	18.8168724	19.2452778	22.8262753
Fahd1	7.89444172	0.51581075	0.40274646	1.28073317	0.2002874	0.97105192		Fahd1	4.03227797	2.71514231	8.58813393	8.27256004	3.32962344	9.40843619	15.3962222	11.4131377
Phf23	30.2645832	-0.4058155	0.31693848	-1.2804236	0.20039621	0.97105192		Phf23	38.3066407	30.3190892	30.2175083	45.4990802	11.653682	30.5774176	32.7169722	22.8262753
Ndufb9	53.1412752	-0.4180156	0.32648738	-1.2803422	0.20042483	0.97105192		Ndufb9	69.8928182	61.9957495	46.7576181	74.4530404	21.6425524	38.8097993	75.0565833	36.5220405
Kcnmb2	7.26992088	0.52482248	0.41006646	1.27984737	0.20059882	0.97145455		Kcnmb2	2.01613899	3.16766603	9.54237104	6.20442003	8.3240586	14.1126543	12.5094306	2.28262753
Ccdc96	1.32649576	0.4670647	0.36500141	1.27962438	0.20067727	NA		Ccdc96	0	1.81009488	0.31807903	0	0	2.35210905	3.84905555	2.28262753
Il15ra	1.1817948	-0.458664	0.35847815	-1.2794756	0.20072963	NA		Il15ra	2.68818531	2.71514231	0.31807903	2.06814001	1.66481172	0	0	0
Sema6a	34.8471514	-0.4619835	0.36108156	-1.2794436	0.2007409	0.97159019		Sema6a	29.5700384	70.5937002	45.1672229	22.7495401	24.9721758	48.2182355	19.2452778	18.2610202
Matr3	203.500591	0.43257292	0.33814543	1.27925112	0.20080864	0.97159019		Matr3	207.662315	175.579203	191.1655	86.8618804	154.82749	352.816357	335.830097	123.261887
Trip11	21.2784697	0.52721645	0.41230098	1.27871743	0.20099658	0.97173079		Trip11	18.1452509	16.2908539	16.2220308	8.27256004	6.65924688	74.091435	25.981125	4.56525506
Rcor1	9.63416521	-0.4962889	0.38813434	-1.2786523	0.20101953	0.97173079		Rcor1	10.7527413	14.0282353	17.1762679	6.20442003	4.99443516	5.88027262	13.4716944	4.56525506
Akap6	174.273389	0.38960561	0.30491368	1.27775709	0.20133509	0.9728162		Akap6	173.387953	155.215636	163.492624	93.0663005	184.794101	328.119212	175.132028	120.979259
Emp1	0.65580915	-0.3824892	0.29935164	-1.2777255	0.20134623	NA		Emp1	0.67204633	3.62018975	0.9542371	0	0	0	0	0
Ptger3	0.65580915	-0.3824892	0.29935164	-1.2777255	0.20134623	NA		Ptger3	0.67204633	3.62018975	0.9542371	0	0	0	0	0
Smyd1	2.15895702	-0.5230541	0.40954098	-1.2771716	0.20154167	NA		Smyd1	3.36023164	3.16766603	2.54463228	4.13628002	0	1.17605452	2.88679167	0
Cenpc1	9.31878814	0.47321136	0.37060325	1.27686783	0.20164891	0.97389227		Cenpc1	5.37637063	8.14542694	7.63389683	8.27256004	6.65924688	16.4647633	10.5849028	11.4131377
Cd72	1.6425955	0.48004366	0.37614389	1.27622347	0.20187653	NA		Cd72	0.67204633	1.35757116	0.9542371	0	0	8.23238167	1.92452778	0
Kif17	14.240317	0.48622069	0.38110641	1.27581344	0.20202147	0.97498392		Kif17	20.1613899	5.43028463	8.90621297	8.27256004	23.3073641	10.5844907	14.4339583	22.8262753
Mfng	1.76966435	-0.4909819	0.38484067	-1.2758056	0.20202424	NA		Mfng	0.67204633	3.16766603	1.27231614	6.20442003	1.66481172	1.17605452	0	0
Zfp618	4.44627469	0.52418122	0.41091713	1.27563731	0.20208376	NA		Zfp618	2.01613899	2.71514231	2.86271131	6.20442003	6.65924688	7.05632714	5.77358333	2.28262753
H2-Ab1	0.47539127	0.35538502	0.27860849	1.27557141	0.20210707	NA		H2-Ab1	0	0	0	0	1.66481172	1.17605452	0.96226389	0
Insig1	31.1378885	-0.3792402	0.29731856	-1.2755347	0.20212004	0.97498392		Insig1	30.9141311	45.2523719	35.6248519	31.0221002	34.9610461	30.5774176	20.2075417	20.5436478
Ift140	16.3368482	0.42576788	0.33382128	1.27543662	0.20215474	0.97498392		Ift140	12.7688802	9.5029981	14.6316356	16.5451201	23.3073641	17.6408179	13.4716944	22.8262753
Cps1	0.85776155	0.43347575	0.33990736	1.27527615	0.20221152	NA		Cps1	0.67204633	0	0.31807903	0	1.66481172	0	1.92452778	2.28262753
Sez6	112.833288	-0.4538605	0.35591431	-1.2751961	0.20223983	0.97498392		Sez6	121.640385	214.043719	133.275115	72.3849004	134.849749	112.901234	33.6792361	79.8919636
Mtm1	1.76956494	-0.5131226	0.40241494	-1.2751082	0.20227094	NA		Mtm1	2.01613899	3.62018975	2.86271131	2.06814001	1.66481172	0	1.92452778	0
Rimkla	12.7948596	0.45078589	0.35358247	1.27491018	0.20234103	0.975032		Rimkla	6.72046328	8.59795066	12.4050823	14.4769801	13.3184938	12.9365998	20.2075417	13.6957652
Spock3	78.5916196	0.51981474	0.40789657	1.27437881	0.2025292	0.97549891		Spock3	58.4680306	75.5714611	71.5677828	16.5451201	29.966611	251.675668	95.264125	29.6741579
Epor	3.14263336	-0.5230299	0.41059208	-1.2738431	0.20271902	NA		Epor	4.7043243	3.16766603	1.59039517	8.27256004	1.66481172	1.17605452	0	4.56525506
Rplp2	4.36631528	-0.5286061	0.41499552	-1.2737633	0.20274732	NA		Rplp2	6.72046328	3.16766603	4.45310648	10.3407001	8.3240586	0	1.92452778	0
Xkr7	3.16965333	0.52068371	0.40884521	1.27354728	0.20282391	NA		Xkr7	0	3.16766603	1.90847421	2.06814001	11.653682	2.35210905	1.92452778	2.28262753
Pigq	80.407816	-0.3693537	0.29013634	-1.273035	0.20300563	0.97690671		Pigq	69.8928182	95.9350284	71.8858618	132.360961	88.2350212	61.1548353	46.1886666	77.6093361
Zdhhc21	34.370288	0.44276216	0.34787192	1.27277349	0.20309843	0.97690671		Zdhhc21	27.5538995	37.5594687	32.1259825	12.4088401	28.3017992	67.0351079	47.1509305	22.8262753
Atg4a	0.93721637	-0.4490732	0.35289085	-1.2725555	0.20317581	NA		Atg4a	2.01613899	2.71514231	1.59039517	0	0	1.17605452	0	0
Zbtb34	20.6282075	0.45208882	0.35531533	1.27235946	0.20324543	0.97690671		Zbtb34	11.4247876	14.0282353	17.4943469	22.7495401	38.2906696	11.7605452	17.32075	31.9567854
Pam16	1.7354381	0.5075054	0.39887339	1.27234711	0.20324982	NA		Pam16	0.67204633	1.81009488	0.9542371	0	4.99443516	3.52816357	1.92452778	0
Med13l	122.912044	0.31621647	0.24855511	1.27221871	0.20329542	0.97690671		Med13l	104.839227	110.415787	109.737267	105.475141	196.447783	125.837834	98.1509166	132.392397
Sfi1	2.86550536	0.52772013	0.4148873	1.27196018	0.20338727	NA		Sfi1	3.36023164	2.26261859	1.27231614	0	1.66481172	8.23238167	3.84905555	2.28262753
Tdp1	7.04671789	-0.5117375	0.40235114	-1.2718679	0.20342005	0.97690671		Tdp1	14.1129729	9.05047438	4.77118552	8.27256004	1.66481172	5.88027262	5.77358333	6.84788259
Lrrc14	27.4638904	0.49173782	0.38668557	1.27167355	0.20348913	0.97690671		Lrrc14	12.0968339	15.3858064	20.6751372	35.1583802	41.620293	23.5210905	9.62263889	61.6309433
E030024N20Rik	12.7028086	0.44829371	0.35253934	1.27161329	0.20351055	0.97690671		E030024N20R	9.4086486	10.4080455	11.1327662	8.27256004	14.9833055	8.23238167	16.3584861	22.8262753
Mlx	15.8470937	-0.4416943	0.34738195	-1.2714947	0.2035527	0.97690671		Mlx	25.5377605	19.9110436	21.3112953	8.27256004	13.3184938	12.9365998	16.3584861	9.13051012
Tas1r1	1.10494034	-0.4512696	0.35497189	-1.2712826	0.20362815	NA		Tas1r1	2.01613899	0.45252372	1.27231614	4.13628002	0	0	0.96226389	0
Zfp652os	1.83094681	0.50506926	0.39756467	1.27040779	0.20393941	NA		Zfp652os	1.34409266	0.90504744	1.27231614	0	4.99443516	0	3.84905555	2.28262753

GeneID	Base mean	log2(FC)	StdErr	Wald-Stats	P-value	P-adj		GeneID	Normalised expression for Chow#1	Normalised expression for Chow#2	Normalised expression for Chow#3	Normalised expression for Chow#4	Normalised expression for HFD#1	Normalised expression for HFD#2	Normalised expression for HFD#3	Normalised expression for HFD#4
Brca2	6.37883584	0.51459986	0.40516495	1.27009965	0.20404914	0.97884972		Brca2	4.03227797	3.16766603	7.31581779	4.13628002	3.32962344	12.9365998	11.5471667	4.56525506
Wdr4	14.5165314	0.51984764	0.4095596	1.26928448	0.20433962	0.97980343		Wdr4	5.37637063	8.14542694	5.72542262	22.7495401	31.6314227	7.05632714	5.77358333	29.6741579
Angpt1	4.41177423	-0.5249392	0.41368468	-1.2689356	0.20446402	NA		Angpt1	8.73660227	6.78785578	4.45310648	4.13628002	1.66481172	4.7042181	4.81131944	0
Ptgs1	13.2569176	0.46667691	0.36794661	1.2683278	0.20468091	0.98006925		Ptgs1	10.0806949	12.6706641	12.0870033	4.13628002	13.3184938	19.9929269	8.660375	25.1089028
Prdm16	13.2733235	-0.4679162	0.36893593	-1.2682857	0.20469594	0.98006925		Prdm16	10.0806949	16.2908539	13.3593194	24.8176801	11.653682	8.23238167	5.77358333	15.9783927
Lmtk3	113.1623	0.47298111	0.37295861	1.26818661	0.20473132	0.98006925		Lmtk3	66.5325865	100.007742	69.3412295	115.815841	246.392135	129.365998	31.7547083	146.088162
Atad2	4.83025775	0.52584583	0.41467185	1.2681011	0.20476185	0.98006925		Atad2	4.03227797	4.97776091	3.49886938	0	9.98887032	9.40843619	6.73584722	0
BC017643	4.48195729	0.52515432	0.41412953	1.26809195	0.20476512	NA		BC017643	1.34409266	3.16766603	3.81694841	4.13628002	9.98887032	8.23238167	2.88679167	2.28262753
F8	3.28689737	0.52627056	0.41520255	1.26750319	0.20497542	NA		F8	2.01613899	4.07271347	1.90847421	0	3.32962344	8.23238167	6.73584722	0
Sdc3	195.784297	-0.3895815	0.30741544	-1.2672801	0.20505516	0.98103382		Sdc3	155.242702	334.415028	181.941208	235.767961	214.760712	189.344778	90.4528055	164.349182
Mex3d	18.085638	0.43475392	0.34323865	1.26662286	0.20529017	0.98129613		Mex3d	11.4247876	11.313093	13.9954775	22.7495401	28.3017992	14.1126543	15.3962222	27.3915304
Hmcn1	2.00413922	0.46598233	0.36790926	1.26656863	0.20530958	NA		Hmcn1	1.34409266	0.90504744	0.9542371	0	11.653682	1.17605452	0	0
Armcx6	3.39948513	0.52669191	0.41601999	1.26602547	0.20550396	NA		Armcx6	1.34409266	4.52523719	1.90847421	0	1.66481172	7.05632714	3.84905555	6.84788259
Atp13a4	2.29162413	-0.5031992	0.3975217	-1.2658409	0.20557005	NA		Atp13a4	1.34409266	4.07271347	2.86271131	6.20442003	0	0	3.84905555	0
9930014A18Rik	1.99959594	0.49477651	0.39088432	1.26578757	0.20558915	NA		9930014A18Ri	0	0.90504744	0.31807903	4.13628002	3.32962344	1.17605452	3.84905555	2.28262753
Opalin	2.94888884	-0.5183622	0.4096619	-1.2653416	0.20574891	NA		Opalin	4.7043243	4.97776091	3.81694841	2.06814001	0	1.17605452	0	6.84788259
Mrpl11	16.4566255	0.43332451	0.3425292	1.26507319	0.20584509	0.98129613		Mrpl11	17.4732045	13.1231878	16.2220308	4.13628002	18.3129289	16.4647633	23.0943333	22.8262753
Bhlhe22	24.2303221	0.45775337	0.36204038	1.26437105	0.20609688	0.98129613		Bhlhe22	25.5377605	20.3635674	18.448584	12.4088401	34.9610461	16.4647633	51.96225	13.6957652
Cdipt	83.7686374	-0.3772414	0.29849744	-1.2638011	0.20630144	0.98129613		Cdipt	72.5810035	122.633928	102.421449	84.7937404	81.5757743	58.8027262	42.3396111	105.000866
Fjx1	29.6780028	-0.4586703	0.36294012	-1.263763	0.20631511	0.98129613		Fjx1	36.9625481	52.9452751	40.3960374	10.3407001	34.9610461	19.9929269	14.4339583	27.3915304
Slc35a2	13.073304	-0.5017238	0.39714597	-1.2633234	0.20647299	0.98129613		Slc35a2	9.4086486	18.5534725	11.4508452	26.8858201	16.6481172	10.5844907	1.92452778	9.13051012
Arhgef11	94.8912709	0.31748518	0.25131661	1.26328769	0.20648581	0.98129613		Arhgef11	90.7262543	73.7613662	68.7050715	101.338861	136.514561	83.4998712	90.4528055	114.131377
Rab3ip	24.3618374	-0.4145538	0.32819504	-1.2631323	0.20654162	0.98129613		Rab3ip	39.6507334	28.961518	27.990955	18.6132601	14.9833055	21.1689814	29.8301805	13.6957652
Tm7sf3	24.5768656	-0.450153	0.35638316	-1.2631151	0.20654781	0.98129613		Tm7sf3	16.1291119	41.6321821	22.2655324	39.2946602	14.9833055	30.5774176	13.4716944	18.2610202
Tmem41b	10.9773711	-0.4739599	0.37531244	-1.262841	0.20664633	0.98129613		Tmem41b	12.0968339	15.3858064	17.4943469	8.27256004	6.65924688	15.2887088	5.77358333	6.84788259
Mib2	72.1022674	0.51696044	0.40937876	1.26279254	0.20666375	0.98129613		Mib2	35.6184554	21.7211385	23.8559276	124.088401	163.151549	37.6337448	26.9433889	143.805534
Sox18	3.28842356	-0.5219524	0.41336116	-1.2627031	0.2066959	NA		Sox18	1.34409266	5.43028463	5.08926455	6.20442003	4.99443516	0	0.96226389	2.28262753
Pts	11.3019218	-0.4690286	0.37154341	-1.2623791	0.2068124	0.98129613		Pts	14.7850192	18.1009488	12.4050823	10.3407001	6.65924688	5.88027262	15.3962222	6.84788259
Gm14322	5.78086059	0.52079906	0.41273494	1.26182451	0.20701194	0.98129613		Gm14322	2.68818531	3.16766603	3.18079035	6.20442003	0	11.7605452	19.2452778	0
Ier3ip1	25.614541	0.39600354	0.31384937	1.26176305	0.20703406	0.98129613		Ier3ip1	21.5054825	25.793852	20.6751372	16.5451201	14.9833055	32.9295267	33.6792361	38.804668
Phyh	61.2206033	-0.4120712	0.32660863	-1.2616664	0.20706883	0.98129613		Phyh	86.02193	62.9007969	62.3434908	78.5893204	21.6425524	44.6900719	85.6414861	47.9351782
Incenp	8.36927938	0.49667065	0.39368486	1.26159449	0.20709474	0.98129613		Incenp	5.37637063	8.14542694	6.04350166	6.20442003	8.3240586	19.9929269	10.5849028	2.28262753
Esrra	29.062779	0.49735011	0.39423181	1.2615677	0.20710438	0.98129613		Esrra	20.1613899	14.480759	14.9497146	37.2265202	39.9554813	11.7605452	16.3584861	77.6093361
BC004004	23.1814418	-0.4719746	0.37413788	-1.2614992	0.20712905	0.98129613		BC004004	25.5377605	47.9675142	30.8536663	8.27256004	11.653682	28.2253086	19.2452778	13.6957652
Tnrc6b	147.691566	0.32386441	0.25677981	1.26125341	0.20721756	0.98129613		Tnrc6b	89.3821617	126.706641	132.320878	171.655621	189.788536	196.401106	133.754681	141.522907
Shkbp1	1.87745471	-0.4785182	0.37949424	-1.2609367	0.20733165	NA		Shkbp1	5.37637063	0.90504744	1.27231614	4.13628002	3.32962344	0	0	0
Acot7	153.840099	0.3433788	0.27233917	1.26084981	0.20736296	0.98129613		Acot7	154.570656	95.9350284	113.236136	169.587481	189.788536	124.66178	150.113167	232.828008
4933431E20Rik	89.727889	-0.2772826	0.21992567	-1.2608015	0.20738037	0.98129613		4933431E20Ri	100.134903	109.058216	92.8790781	90.9981605	79.9109626	76.4435441	70.2452639	98.1529838
Dhcr7	18.7081047	-0.4453238	0.35326737	-1.2605856	0.2074582	0.98129613		Dhcr7	12.7688802	32.129184	22.2655324	20.6814001	16.6481172	11.7605452	10.5849028	22.8262753
Gpr39	71.6512128	-0.3923857	0.31127898	-1.2605594	0.20746762	0.98129613		Gpr39	81.3176057	118.561214	83.9728651	45.4990802	86.5702095	45.8661264	45.2264028	66.1961984
Klhdc10	118.284765	0.32516491	0.25796294	1.26051017	0.20748538	0.98129613		Klhdc10	81.3176057	84.6219354	94.7875523	157.178641	141.508996	117.605452	123.169778	146.088162
Xrcc1	14.1516663	0.46259375	0.36703696	1.26034649	0.20754439	0.98129613		Xrcc1	10.7527413	7.2403795	7.95197586	20.6814001	23.3073641	10.5844907	14.4339583	18.2610202
Grwd1	10.1389034	0.48116061	0.38177091	1.26033859	0.20754724	0.98129613		Grwd1	5.37637063	6.78785578	7.31581779	12.4088401	18.3129289	4.7042181	12.5094306	13.6957652
2700049A03Rik	4.0896996	0.52461155	0.4162822	1.26023057	0.2075862	NA		2700049A03Ri	2.01613899	3.62018975	4.13502745	0	1.66481172	10.5844907	3.84905555	6.84788259
Mocs3	9.39532857	0.50477106	0.40054107	1.26022298	0.20758894	0.98129613		Mocs3	7.39250961	5.43028463	4.45310648	10.3407001	8.3240586	3.52816357	10.5849028	25.1089028
Fbxo9	57.2357807	0.31761112	0.25230447	1.25884062	0.20808791	0.9827714		Fbxo9	56.4518916	44.3473245	47.3937761	55.8397803	53.2739751	68.2111624	79.8679027	52.5004332
Ccdc113	0.87948279	0.43905187	0.34878197	1.25881471	0.20809727	NA		Ccdc113	0.67204633	0	0.63615807	0	1.66481172	1.17605452	2.88679167	0
Cplx3	15.1579572	0.48148941	0.38252144	1.25872528	0.20812958	0.9827714		Cplx3	8.06455594	5.88280835	13.9954775	18.6132601	19.9777406	14.1126543	8.660375	31.9567854
Supt16	66.300217	0.32526406	0.25848797	1.25833347	0.20827118	0.9827714		Supt16	59.8121232	43.8948007	59.4807795	72.3849004	61.5980337	91.7322529	79.8679027	61.6309433
Id1	3.01148103	-0.5229343	0.41569764	-1.257968	0.20840332	NA		Id1	2.68818531	4.97776091	4.13502745	4.13628002	1.66481172	0	1.92452778	4.56525506
Sptlc2	13.9066521	0.48730102	0.38738971	1.25790904	0.20842465	0.9827714		Sptlc2	6.72046328	16.2908539	11.1327662	8.27256004	14.9833055	32.9295267	16.3584861	4.56525506
Clic5	1.47438303	-0.4855827	0.3860273	-1.2578974	0.20842888	NA		Clic5	0.67204633	2.71514231	3.49886938	2.06814001	1.66481172	1.17605452	0	0
Galnt18	10.2561466	-0.4678016	0.37193838	-1.2577394	0.20848601	0.9827714		Galnt18	10.7527413	15.3858064	13.3593194	10.3407001	13.3184938	4.7042181	9.62263889	4.56525506
Aldh4a1	13.4714652	-0.5016191	0.39893472	-1.2573965	0.2086101	0.9827714		Aldh4a1	12.7688802	6.78785578	12.4050823	37.2265202	9.98887032	5.88027262	6.73584722	15.9783927
Actn1	23.286833	0.4225402	0.33611341	1.2571358	0.20870447	0.9827714		Actn1	20.1613899	15.8383302	20.9932163	18.6132601	49.9443516	19.9929269	20.2075417	20.5436478
Brk1	100.73541	-0.3719998	0.29598339	-1.2568265	0.20881646	0.9827714		Brk1	129.032895	91.4097912	107.828793	134.429101	93.2294563	41.1619083	92.3773333	116.414004
Sidt1	22.2573131	-0.4698243	0.37383992	-1.2567525	0.20884325	0.9827714		Sidt1	18.1452509	50.2301328	29.2632712	10.3407001	16.6481172	24.697145	17.32075	11.4131377
Gm13498	13.1174321	0.51211067	0.40754537	1.25657342	0.20890815	0.9827714		Gm13498	12.0968339	6.78785578	17.4943469	0	8.3240586	27.0492541	24.0565972	9.13051012
Tmco1	16.1305451	-0.4819469	0.38354421	-1.2565614	0.20891248	0.9827714		Tmco1	18.8172972	25.3413283	27.0367179	8.27256004	11.653682	24.697145	8.660375	4.56525506
AI413582	14.42742	-0.4676142	0.37225011	-1.2561829	0.20904967	0.9829841		AI413582	22.8495752	17.1959013	13.6773985	16.5451201	8.3240586	3.52816357	17.32075	15.9783927
Gpr182	1.16604392	-0.4589638	0.36551092	-1.2556775	0.20923293	NA		Gpr182	0.67204633	2.71514231	1.59039517	2.06814001	0	0	0	2.28262753
Tubb4a	624.255385	-0.366445	0.29186329	-1.2555363	0.20928415	0.98365389		Tubb4a	841.402003	564.749601	635.521911	821.051584	735.84678	324.591049	372.396125	698.484025
Acat1	47.1946001	0.35374271	0.28190457	1.2548314	0.20953997	0.98415944		Acat1	44.3550577	38.9170398	44.5310648	35.1583802	38.2906696	51.7463991	78.9056389	45.6525506
Zfp609	59.1988225	0.30952563	0.24668654	1.25473254	0.20957587	0.98415944		Zfp609	49.7314283	58.8280835	50.8926455	47.5672202	84.9053977	52.9224536	62.5471528	66.1961984
Ccdc103	1.96902534	0.49993551	0.39850215	1.25453654	0.20964705	NA		Ccdc103	0.67204633	1.81009488	0.9542371	0	3.32962344	1.17605452	0.96226389	6.84788259
Ndufs2	92.3495742	0.35030589	0.27926577	1.25438177	0.20970327	0.98432525		Ndufs2	73.9250961	52.9452751	70.9316247	124.088401	113.207197	89.3801438	93.3395972	120.979259
Zfp830	8.26190504	0.50646758	0.40389882	1.25394668	0.20986139	0.98463499		Zfp830	6.72046328	3.16766603	4.77118552	10.3407001	1.66481172	10.5844907	10.5849028	18.2610202
Stc2	1.4715962	0.48722134	0.3885607	1.25391305	0.20987361	NA		Stc2	0.67204633	1.81009488	0.31807903	0	1.66481172	1.17605452	3.84905555	2.28262753
Usp43	0.59755887	-0.3835472	0.30590189	-1.2538243	0.20990588	NA		Usp43	2.01613899	1.81009488	0.9542371	0	0	0	0	0
Fam46a	12.9649567	0.45641148	0.36406864	1.25364129	0.20997242	0.98472364		Fam46a	8.73660227	8.59795066	13.3593194	10.3407001	8.3240586	25.8731995	12.5094306	15.9783927
Parp4	11.238724	-0.5203935	0.41521668	-1.2533059	0.2100944	0.98486355		Parp4	3.36023164	11.313093	8.2700549	37.2265202	4.99443516	5.88027262	2.88679167	15.9783927
Cetn4	3.62850978	-0.5059214	0.40380383	-1.2528891	0.21024608	NA		Cetn4	10.0806949	8.59795066	2.54463228	0	0	5.88027262	1.92452778	0
Itga6	5.13077971	-0.5187591	0.41408713	-1.2527778	0.2102866	0.98533237		Itga6	5.37637063	13.5757116	6.99773876	2.06814001	8.3240586	4.7042181	0	0
Setd7	310.719862	0.34517349	0.27561606	1.25237076	0.21043479	0.98554627		Setd7	179.43637	269.251613	267.822547	357.788222	321.308662	315.182612	263.660305	511.308567
Itpka	38.8072868	0.46799823	0.37379959	1.25200305	0.21056875	0.98554627		Itpka	44.3550577	25.3413283	30.8536663	18.6132601	94.8942681	16.4647633	36.5660278	43.3699231
Plcxd3	28.6397497	0.42481879	0.33934115	1.25189294	0.21060888	0.98554627		Plcxd3	23.5216215	25.793852	27.354797	16.5451201	13.3184938	50.5703445	42.3396111	29.6741579
Ccpg1os	3.54448941	-0.5210302	0.41622585	-1.2517968	0.2106439	NA		Ccpg1os	7.39250961	4.97776091	5.08926455	2.06814001	1.66481172	2.35210905	4.81131944	0
Utp3	16.9070089	0.45595251	0.36440445	1.25122649	0.21085186	0.98625149		Utp3	15.4570656	14.9332827	9.224292	14.4769801	11.653682	36.4576902	12.5094306	20.5436478
Actr2	171.569093	0.35921138	0.28727113	1.25042629	0.21114386	0.98650119		Actr2	168.683628	138.019734	190.847421	88.9300204	159.821925	201.105324	279.056528	146.088162
A830082K12Rik	7.83563351	0.49487352	0.39584405	1.25017294	0.21123638	0.98650119		A830082K12Ri	4.7043243	9.05047438	5.72542262	4.13628002	4.99443516	14.1126543	15.3962222	4.56525506
Scgn	0.96820319	-0.3728275	0.29823074	-1.2501311	0.21125167	NA		Scgn	0	0.90504744	0.63615807	6.20442003	0	0	0	0
Acyp1	6.52283415	0.4967117	0.39764619	1.24912978	0.21161761	0.98650119		Acyp1	7.39250961	4.07271347	5.08926455	2.06814001	3.32962344	8.23238167	10.5849028	11.4131377
Mrpl3	25.3098833	-0.4862025	0.38925807	-1.2490493	0.21164703	0.98650119		Mrpl3	42.3389187	34.8443264	35.3067728	14.4769801	13.3184938	15.2887088	42.3396111	4.56525506
Hist1h2ac	2.56173693	-0.5090442	0.40754657	-1.2490456	0.21164841	NA		Hist1h2ac	5.37637063	1.81009488	5.72542262	2.06814001	1.66481172	0	3.84905555	0
Tyw1	5.62201494	0.51026439	0.40863185	1.24871419	0.21176963	0.98650119		Tyw1	2.01613899	1.81009488	5.08926455	8.27256004	8.3240586	5.88027262	6.73584722	6.84788259
Mturn	129.72147	0.30254573	0.24230698	1.2486051	0.21180955	0.98650119		Mturn	101.478996	106.795598	91.924841	163.383061	139.844185	123.485725	151.07543	159.783927
Mrgpre	15.4262076	0.48054554	0.38488942	1.24852882	0.21183746	0.98650119		Mrgpre	7.39250961	9.5029981	13.3593194	16.5451201	23.3073641	18.8168724	4.81131944	29.6741579
P2ry1	3.23763382	-0.5195136	0.41620915	-1.2482032	0.21195664	NA		P2ry1	3.36023164	4.07271347	6.67965972	4.13628002	1.66481172	1.17605452	4.81131944	0
Xbp1	42.3709274	-0.3647189	0.29223279	-1.2480425	0.21201548	0.98650119		Xbp1	33.6023164	65.1634155	48.6660923	47.5672202	39.9554813	42.3379629	36.5660278	25.1089028
Cdr1	275.068402	0.49813304	0.39915375	1.24797286	0.212041	0.98650119		Cdr1	171.371814	203.18315	204.524819	18.6132601	79.9109626	519.8161	975.735583	27.3915304
Sin3b	86.9104249	0.41602912	0.3333828	1.24790216	0.21206689	0.98650119		Sin3b	65.8605402	55.2078937	52.4830407	113.747701	156.492302	57.6266717	68.3207361	125.544514
Fbxo8	14.6776658	-0.4947939	0.39657159	-1.2476786	0.21214879	0.98650119		Fbxo8	24.8657142	18.1009488	23.5378486	8.27256004	8.3240586	14.1126543	20.2075417	0
Numb	19.838568	-0.395333	0.31686396	-1.2476426	0.21216198	0.98650119		Numb	20.8334362	28.0564706	20.9932163	22.7495401	13.3184938	22.345036	14.4339583	15.9783927
Hba-a1	25.1591111	0.49630231	0.39782099	1.24755184	0.21219523	0.98650119		Hba-a1	16.1291119	17.648425	13.9954775	26.8858201	69.9220923	7.05632714	15.3962222	34.239413
Bzrap1	72.4037448	0.42205663	0.33835848	1.24736531	0.21226358	0.98650119		Bzrap1	57.1239379	48.4200379	41.6683535	90.9981605	134.849749	74.091435	38.4905555	93.5877288
Snrnp35	2.99529841	0.51263497	0.41104399	1.24715355	0.2123412	NA		Snrnp35	2.68818531	3.16766603	0.9542371	0	9.98887032	2.35210905	4.81131944	0
Ctif	124.6837	0.44005406	0.35289058	1.24699862	0.212398	0.98650119		Ctif	74.5971425	65.6159392	74.4304941	188.200741	191.453348	99.9646345	81.7924305	221.414871
Tmed2	24.991508	-0.4773472	0.3828021	-1.2469817	0.21240419	0.98650119		Tmed2	23.5216215	52.0402277	40.0779583	6.20442003	13.3184938	28.2253086	18.2830139	18.2610202
Dennd5b	99.4522431	0.38445733	0.30835761	1.24679046	0.21247434	0.98650119		Dennd5b	91.3983007	89.5996963	96.0598684	57.9079203	74.9165274	176.408179	138.566	70.7614535
Usp34	167.737299	0.44970304	0.36079583	1.24641973	0.21261034	0.986704		Usp34	145.162007	136.662163	173.034995	78.5893204	138.179373	348.112139	255.962194	66.1961984
Gm13031	1.10360843	-0.4362506	0.35004873	-1.2462567	0.21267016	NA		Gm13031	3.36023164	3.16766603	0.63615807	0	1.66481172	0	0	0
Bmp1	4.86466601	-0.5168635	0.41498903	-1.2454871	0.21295277	0.98702747		Bmp1	3.36023164	11.313093	8.2700549	2.06814001	4.99443516	4.7042181	1.92452778	2.28262753
Cdc42se2	127.360429	-0.2684877	0.21557723	-1.2454364	0.21297138	0.98702747		Cdc42se2	145.834053	143.450019	155.540648	113.747701	118.201632	98.78858	133.754681	109.566121
Rpe65	0.79970576	-0.3438778	0.27613363	-1.245331	0.21301011	NA		Rpe65	0	2.26261859	4.13502745	0	0	0	0	0
Vimp	9.41902195	-0.5183953	0.41628528	-1.2452885	0.21302571	0.98702747		Vimp	8.73660227	24.4362808	17.1762679	0	8.3240586	7.05632714	9.62263889	0
Fv1	3.93516692	0.5118694	0.4111183	1.24506595	0.21310751	NA		Fv1	2.68818531	2.71514231	3.49886938	2.06814001	3.32962344	5.88027262	6.73584722	4.56525506
Rpusd3	3.94291208	-0.5125634	0.41168414	-1.2450405	0.21311687	NA		Rpusd3	3.36023164	4.07271347	2.22655324	12.4088401	1.66481172	0	0.96226389	6.84788259
Psmg3	3.25509852	-0.5141346	0.41294931	-1.2450309	0.2131204	NA		Psmg3	0.67204633	3.62018975	5.72542262	8.27256004	3.32962344	1.17605452	0.96226389	2.28262753
Arrb1	326.879317	0.3271303	0.26275012	1.24502436	0.2131228	0.98702747		Arrb1	268.146485	264.726376	260.506729	347.447522	437.845482	301.069958	237.67918	497.612802
Nrxn3	357.03828	0.3820037	0.30687593	1.24481479	0.21319984	0.98702747		Nrxn3	262.770114	360.20888	296.44966	283.335181	324.638285	599.787807	197.264097	531.852215
Bin3	2.5976839	-0.5126927	0.41188396	-1.2447503	0.21322353	NA		Bin3	4.03227797	1.81009488	2.86271131	6.20442003	1.66481172	0	1.92452778	2.28262753
Pcgf6	4.13636023	0.51558386	0.41429441	1.24448665	0.21332051	NA		Pcgf6	4.7043243	3.62018975	2.54463228	0	3.32962344	4.7042181	9.62263889	4.56525506

GeneID	Base mean	log2(FC)	StdErr	Wald-Stats	P-value	P-adj	GeneID	Normalised expression for Chow#1	Normalised expression for Chow#2	Normalised expression for Chow#3	Normalised expression for Chow#4	Normalised expression for HFD#1	Normalised expression for HFD#2	Normalised expression for HFD#3	Normalised expression for HFD#4
Slc25a36	17.7177671	0.45839828	0.36835036	1.24446268	0.21332933	0.98702747	Slc25a36	18.1452509	15.3858064	15.5858727	6.20442003	8.3240586	29.4013631	32.7169722	15.9783927
Rwdd4a	26.2169818	0.40035624	0.3217122	1.24445462	0.21333229	0.98702747	Rwdd4a	30.2420848	19.0059962	23.2197695	14.4769801	18.3129289	37.6337448	39.4528194	27.3915304
Cry1	17.0356942	-0.4186373	0.33646581	-1.2442194	0.21341883	0.98702747	Cry1	22.1775288	14.0282353	21.3112953	22.7495401	19.9777406	11.7605452	10.5849028	13.6957652
Gm5434	2.46360615	-0.5074257	0.40786429	-1.2441003	0.21346118	NA	Gm5434	6.04841696	4.07271347	4.13502745	0	0	3.52816357	1.92452778	0
Sulf1	8.56690548	-0.4923727	0.39636778	-1.2422117	0.21415847	0.98951308	Sulf1	8.73660227	16.7433776	13.3593194	4.13628002	4.99443516	10.5844907	7.69811111	2.28262753
Bcr	213.11532	0.34864881	0.28067206	1.24219279	0.21416544	0.98951308	Bcr	204.302084	157.930778	182.577366	188.200741	319.64385	234.03485	126.056569	292.176324
Zfp512	46.1765264	0.38763424	0.31210313	1.24200688	0.21423403	0.98951308	Zfp512	31.5861774	32.129184	29.5813502	66.1804803	54.9387868	36.4576902	68.3207361	50.2178057
Sh3rf3	42.3618993	0.36570988	0.2945407	1.24162763	0.21437398	0.98962862	Sh3rf3	30.2420848	30.3190892	34.6706148	49.6353602	53.2739751	37.6337448	34.6415	68.4788259
AI987944	3.90432213	-0.5165469	0.41606167	-1.2415153	0.21441545	NA	AI987944	4.7043243	9.05047438	5.40734359	2.06814001	1.66481172	3.52816357	4.81131944	0
Hist1h2bb	4.27213572	-0.5165574	0.41613215	-1.2413302	0.21448379	NA	Hist1h2bb	6.04841696	6.78785578	4.45310648	6.20442003	1.66481172	0	6.73584722	2.28262753
Dock3	283.047972	0.29832396	0.24036483	1.24112981	0.2145578	0.98962862	Dock3	298.38857	249.793093	248.737805	204.745861	432.851047	317.534722	252.113139	260.219539
Gnl1	133.098583	0.46522407	0.37485057	1.24109208	0.21457174	0.98962862	Gnl1	79.3014668	61.0907021	77.6112844	198.541441	269.699499	77.6195986	81.7924305	219.132243
Ncapg	1.85739706	0.48993863	0.39476527	1.24108849	0.21457306	NA	Ncapg	2.01613899	0.45252372	0.31807903	2.06814001	1.66481172	3.52816357	4.81131944	0
4833422C13Rik	22.9616964	0.38517652	0.31040279	1.24089258	0.21464543	0.98962862	4833422C13Ri	17.4732045	20.8160911	18.448584	22.7495401	19.9777406	29.4013631	36.5660278	18.2610202
Gjb1	2.35212499	-0.5121119	0.41271933	-1.2408237	0.21467087	NA	Gjb1	2.68818531	5.43028463	2.54463228	2.06814001	1.66481172	1.17605452	0.96226389	2.28262753
Lemd2	7.43208447	0.50313232	0.4055784	1.24053039	0.21477928	0.98962862	Lemd2	6.72046328	4.07271347	4.45310648	6.20442003	21.6425524	4.7042181	4.81131944	6.84788259
5330417C22Rik	19.1355297	-0.4629014	0.3731767	-1.2404349	0.21481459	0.98962862	5330417C22Ri	12.0968339	38.4645161	23.5378486	18.6132601	13.3184938	24.697145	8.660375	13.6957652
Thsd4	3.53313817	-0.5157426	0.41585931	-1.2401852	0.21490688	NA	Thsd4	6.04841696	6.33533207	4.77118552	2.06814001	1.66481172	3.52816357	3.84905555	0
Arl5c	1.20465805	0.39891941	0.32169081	1.24007091	0.21494917	NA	Arl5c	0.67204633	0.45252372	0	0	1.66481172	0	0	6.84788259
Tpst2	8.15099514	0.5027203	0.40542034	1.23999772	0.21497624	0.98994659	Tpst2	7.39250961	7.2403795	4.45310648	4.13628002	23.3073641	3.52816357	10.5849028	4.56525506
Tha1	1.24367085	0.38603601	0.31138995	1.23971892	0.21507937	NA	Tha1	0	0	0	2.06814001	4.99443516	0	2.88679167	0
Prelid1	80.5704766	-0.4364399	0.3520704	-1.2396382	0.21510925	0.99013251	Prelid1	95.4305786	64.2583681	60.4350166	165.451201	59.9332219	31.7534722	57.7358333	109.566121
Ctla2a	0.80421392	-0.3413459	0.27555276	-1.2387679	0.21543148	NA	Ctla2a	0	4.52523719	1.90847421	0	0	0	0	0
Parm1	22.7941496	-0.4613941	0.37248688	-1.2386856	0.21546196	0.99018091	Parm1	15.4570656	50.2301328	28.3090341	16.5451201	13.3184938	29.4013631	15.3962222	13.6957652
Rpl37a	4.50779875	-0.5100028	0.41178728	-1.2385103	0.21552691	NA	Rpl37a	6.72046328	4.97776091	6.36158069	6.20442003	0	4.7042181	4.81131944	2.28262753
Zfp109	5.84265989	0.50848808	0.41056744	1.23850073	0.21553045	0.99018091	Zfp109	6.72046328	4.52523719	4.13502745	0	1.66481172	10.5844907	7.69811111	11.4131377
Hba-a2	25.198871	0.49245452	0.39763506	1.23845852	0.2155461	0.99018091	Hba-a2	16.1291119	17.648425	14.3135566	26.8858201	69.9220923	7.05632714	15.3962222	34.239413
Tifa	2.03132221	-0.5053513	0.40810794	-1.2382785	0.2156128	NA	Tifa	2.01613899	4.07271347	3.81694841	2.06814001	0	2.35210905	1.92452778	0
Zbtb43	18.6487584	0.40370859	0.32610903	1.23795589	0.21573242	0.99018091	Zbtb43	15.4570656	13.1231878	15.9039517	18.6132601	26.6369875	29.4013631	16.3584861	13.6957652
Il10rb	1.71716435	-0.4580388	0.37001718	-1.2378853	0.21575858	NA	Il10rb	1.34409266	5.88280835	3.18079035	0	3.32962344	0	0	0
Akt2	19.6171437	-0.4534188	0.36628648	-1.2378802	0.21576048	0.99018091	Akt2	27.5538995	23.0787097	15.5858727	26.8858201	14.9833055	8.23238167	8.660375	31.9567854
Snurf	483.383384	-0.2981938	0.24092188	-1.2377197	0.21582002	0.99018091	Snurf	655.24517	517.234611	551.230967	426.036842	341.286403	336.351594	503.264014	536.41747
Dera	0.58075329	-0.3780861	0.30550064	-1.2375951	0.21586624	NA	Dera	2.01613899	1.35757116	1.27231614	0	0	0	0	0
Kcna5	10.4110213	-0.4518555	0.36520529	-1.2372643	0.21598899	0.99018091	Kcna5	11.4247876	11.313093	14.3135566	12.4088401	11.653682	8.23238167	4.81131944	9.13051012
Apoc1	0.63149932	-0.3972686	0.32117518	-1.2369218	0.21611612	NA	Apoc1	0.67204633	1.35757116	0.9542371	2.06814001	0	0	0	0
Kiz	8.37607886	-0.4926649	0.39839349	-1.2366288	0.21622493	0.99018091	Kiz	15.4570656	9.95552182	11.4508452	6.20442003	1.66481172	9.40843619	10.5849028	2.28262753
Chrna4	21.5048532	-0.4255651	0.34416923	-1.2364996	0.21627293	0.99018091	Chrna4	23.5216215	38.9170398	25.1282437	12.4088401	24.9721758	17.6408179	13.4716944	15.9783927
Greb1l	7.49208887	0.4861203	0.393153	1.23646597	0.21628541	0.99018091	Greb1l	6.72046328	3.62018975	5.08926455	8.27256004	8.3240586	15.2887088	5.77358333	6.84788259
Napepld	17.9892919	0.3938562	0.31860925	1.23617314	0.21639421	0.99018091	Napepld	13.4409266	13.5757116	14.6316356	18.6132601	24.9721758	15.2887088	18.2830139	25.1089028
Hdac5	143.10886	0.44988094	0.36394583	1.23612059	0.21641374	0.99018091	Hdac5	87.3660227	94.5774573	76.9751264	196.473301	288.012428	95.2604165	77.943375	228.262753
Psd3	325.664456	0.41550464	0.33623693	1.23574957	0.21655167	0.99018091	Psd3	270.83467	329.437267	319.66943	148.906081	298.001298	512.759772	559.075319	166.63181
Ahrr	0.76110618	0.37338201	0.30216733	1.23567962	0.21657768	NA	Ahrr	0	0	0.63615807	0	0	3.52816357	1.92452778	0
AI848285	3.33767342	0.48880331	0.39559042	1.2356298	0.2165962	NA	AI848285	3.36023164	0.45252372	0.9542371	2.06814001	4.99443516	1.17605452	0	13.6957652
Tmem50a	26.8029248	-0.4154114	0.33619699	-1.235619	0.21660023	0.99018091	Tmem50a	26.2098068	44.3473245	35.9429309	16.5451201	21.6425524	15.2887088	20.2075417	34.239413
Tmem129	14.3009118	-0.4406655	0.35667763	-1.2354727	0.21665464	0.99018091	Tmem129	11.4247876	18.5534725	19.0847421	18.6132601	6.65924688	14.1126543	7.69811111	18.2610202
Dlk1	34.2959617	-0.4946995	0.40049322	-1.2352257	0.21674651	0.99018091	Dlk1	12.0968339	39.3695635	20.6751372	103.407001	21.6425524	37.6337448	14.4339583	25.1089028
Dusp9	0.95738302	0.43075821	0.34879518	1.23498898	0.21683461	NA	Dusp9	0.67204633	0.45252372	0.31807903	0	3.32962344	0	2.88679167	0
Crtc2	12.8921733	-0.5017848	0.40640868	-1.2346802	0.21694954	0.99018091	Crtc2	14.1129729	10.8605693	8.58813393	33.0902402	19.9777406	3.52816357	3.84905555	9.13051012
Acss1	11.6297152	-0.4547974	0.36838243	-1.2345795	0.21698704	0.99018091	Acss1	15.4570656	14.0282353	12.4050823	14.4769801	18.3129289	7.05632714	6.73584722	4.56525506
Lig1	7.93814197	0.50920439	0.41250329	1.23442505	0.21704456	0.99018091	Lig1	8.06455594	2.71514231	4.13502745	8.27256004	0	14.1126543	12.5094306	13.6957652
Myd88	3.49392908	-0.513588	0.41607958	-1.2343503	0.21707238	NA	Myd88	6.04841696	3.62018975	5.08926455	4.13628002	4.99443516	1.17605452	2.88679167	0
Mylk	8.89120497	0.47721138	0.38680454	1.23372745	0.21730448	0.99018091	Mylk	6.72046328	10.4080455	5.72542262	4.13628002	14.9833055	8.23238167	16.3584861	4.56525506
Lrrfip2	18.6916021	0.41908326	0.33971438	1.23363416	0.21733926	0.99018091	Lrrfip2	16.8011582	14.480759	12.7231614	18.6132601	9.98887032	32.9295267	21.1698055	22.8262753
Gprin1	104.808615	0.30654434	0.24849688	1.23359435	0.2173541	0.99018091	Gprin1	108.199459	83.2643643	80.4739957	97.2025805	149.833055	97.6125255	89.4905416	132.392397
Map3k1	10.2281575	0.48058664	0.38966941	1.23331886	0.21745682	0.99018091	Map3k1	10.7527413	8.59795066	9.54237104	0	6.65924688	16.4647633	11.5471667	18.2610202
Gm3230	1.14538277	0.43885908	0.35587622	1.23317899	0.21750899	NA	Gm3230	0.67204633	0	0.9542371	0	3.32962344	0	1.92452778	2.28262753
4930570G19Rik	4.80119726	0.50164438	0.40687821	1.23291041	0.21760919	0.99018091	4930570G19Ri	4.7043243	4.07271347	3.18079035	2.06814001	4.99443516	9.40843619	7.69811111	2.28262753
Angel1	6.01487847	0.50315593	0.40820657	1.23260124	0.21772457	0.99018091	Angel1	3.36023164	5.88280835	3.18079035	4.13628002	9.98887032	8.23238167	1.92452778	11.4131377
Zswim3	6.10275687	-0.4986455	0.40457797	-1.2325078	0.21775944	0.99018091	Zswim3	10.7527413	5.88280835	8.90621297	6.20442003	4.99443516	8.23238167	3.84905555	0
Fuom	12.1442148	-0.4336551	0.35201343	-1.2319277	0.2179761	0.99018091	Fuom	18.1452509	13.1231878	12.7231614	14.4769801	9.98887032	7.05632714	12.5094306	9.13051012
Fam19a5	111.590193	-0.2824603	0.22932077	-1.2317258	0.21805155	0.99018091	Fam19a5	114.919922	113.583453	113.236136	153.042361	113.207197	77.6195986	95.264125	111.848749
Fhdc1	5.1266825	-0.5112665	0.41508612	-1.2317118	0.21805674	0.99018091	Fhdc1	6.04841696	3.62018975	4.77118552	14.4769801	3.32962344	5.88027262	2.88679167	0
Zfand6	17.9423837	-0.4291797	0.34849965	-1.2315067	0.21813341	0.99018091	Zfand6	22.8495752	23.0787097	22.2655324	18.6132601	13.3184938	10.5844907	25.981125	6.84788259
Epg5	54.4306606	0.41364618	0.33589484	1.23147522	0.21814518	0.99018091	Epg5	45.6991503	40.274611	50.5745665	41.3628002	114.872009	65.8590533	28.8679167	47.9351782
Bean1	9.862513	0.45076476	0.36605812	1.23140215	0.21817249	0.99018091	Bean1	6.04841696	8.59795066	9.224292	6.20442003	13.3184938	14.1126543	7.69811111	13.6957652
5930412G12Rik	1.18733554	-0.4460809	0.3623112	-1.2312091	0.21824466	NA	5930412G12R	1.34409266	3.16766603	0.63615807	2.06814001	0	0	0	2.28262753
Srsf3	35.4385129	0.48705174	0.39565581	1.23099857	0.2183234	0.99018091	Srsf3	36.2905017	24.8888045	36.2610099	8.27256004	14.9833055	64.6829988	79.8679027	18.2610202
Gpank1	7.58762421	-0.5119455	0.41590339	-1.2309241	0.21835126	0.99018091	Gpank1	6.04841696	4.97776091	5.08926455	24.8176801	3.32962344	1.17605452	3.84905555	11.4131377
Eid1	175.954147	0.30368054	0.24671532	1.23089453	0.21836231	0.99018091	Eid1	163.307258	123.538975	142.181328	198.541441	164.81636	197.57716	257.886722	159.783927
Hnrnph2	58.4533136	0.43009229	0.34966498	1.23001248	0.21869243	0.99019711	Hnrnph2	77.2853278	51.587704	45.803381	12.4088401	54.9387868	94.0843619	72.1697916	59.3483158
Sestd1	34.4740608	0.40024351	0.32544157	1.22984751	0.21875421	0.99019711	Sestd1	36.9625481	31.6766603	27.354797	18.6132601	49.9443516	42.3379629	52.9245139	15.9783927
Traf2	6.52859247	-0.5097606	0.41449746	-1.229828	0.21876152	0.99019711	Traf2	4.03227797	4.52523719	7.63389683	18.6132601	1.66481172	4.7042181	1.92452778	9.13051012
2410089E03Rik	24.1140586	0.40443962	0.32891301	1.22962488	0.21883761	0.99019711	2410089E03Ri	18.1452509	20.3635674	23.8559276	16.5451201	38.2906696	38.8097993	16.3584861	20.5436478
Shmt2	6.85302362	0.48439585	0.39404811	1.229281	0.21896647	0.99019711	Shmt2	4.7043243	4.07271347	4.77118552	8.27256004	6.65924688	4.7042181	12.5094306	9.13051012
Rnu12	0.99527974	0.40870463	0.33252271	1.22910291	0.21903322	NA	Rnu12	0	0	1.27231614	0	1.66481172	1.17605452	3.84905555	0
Tnfrsf22	1.27781932	-0.4462736	0.36310052	-1.2290634	0.21904802	NA	Tnfrsf22	0.67204633	0.90504744	2.22655324	4.13628002	0	0	0	2.28262753
Bloc1s6	37.3097183	-0.3830915	0.31177558	-1.2287414	0.2191688	0.99019711	Bloc1s6	47.7152893	34.8443264	34.6706148	57.9079203	28.3017992	18.8168724	44.2641389	31.9567854
Pitpnm2	186.274562	0.4243747	0.34538467	1.22870161	0.2191837	0.99019711	Pitpnm2	140.457683	162.003491	139.000538	163.383061	407.878871	115.253343	101.999972	260.219539
Tnpo2	168.097968	0.36471886	0.29688776	1.22847391	0.21926912	0.99019711	Tnpo2	109.543552	119.466262	140.590933	204.745861	273.029122	134.070216	132.792417	230.545381
H2afy3	3.80364639	0.51077971	0.41578538	1.22846962	0.21927073	NA	H2afy3	3.36023164	3.16766603	2.86271131	0	9.98887032	5.88027262	2.88679167	2.28262753
Cntrob	10.1414405	0.4724934	0.38491343	1.2275316	0.21962285	0.99019711	Cntrob	4.03227797	10.4080455	8.2700549	8.27256004	23.3073641	9.40843619	10.5849028	6.84788259
Tle1	31.6831787	-0.3521041	0.28684574	-1.2275032	0.21963352	0.99019711	Tle1	37.6345944	32.129184	39.1237212	35.1583802	36.6258578	18.8168724	28.8679167	25.1089028
Lrrc45	3.69326756	0.50752897	0.41350286	1.22738928	0.21967632	NA	Lrrc45	2.01613899	2.26261859	2.54463228	4.13628002	3.32962344	5.88027262	4.81131944	4.56525506
Fes	1.04050733	0.29920918	0.24379244	1.22731117	0.21970566	NA	Fes	0	0	0	0	8.3240586	0	0	0
Ptpn13	7.3505356	-0.4968923	0.40493708	-1.2270852	0.21979059	0.99019711	Ptpn13	8.06455594	12.6706641	5.40734359	12.4088401	9.98887032	3.52816357	6.73584722	0
Stx3	7.88124552	-0.485335	0.39551925	-1.2270831	0.21979137	0.99019711	Stx3	10.0806949	9.95552182	8.58813393	10.3407001	13.3184938	2.35210905	3.84905555	4.56525506
Gsx1	1.999959	-0.4027005	0.32826184	-1.2267661	0.21991052	NA	Gsx1	0.67204633	0	0.63615807	12.4088401	0	0	0	2.28262753
Cpped1	24.9923575	-0.4183411	0.34102196	-1.2267278	0.2199249	0.99019711	Cpped1	35.6184554	25.793852	24.8101647	35.1583802	16.6481172	22.345036	32.7169722	6.84788259
Zfp747	3.98756726	0.50906481	0.41501686	1.22661235	0.21996832	NA	Zfp747	2.01613899	1.35757116	2.54463228	6.20442003	6.65924688	4.7042181	3.84905555	4.56525506
Rxra	55.0582193	0.47879737	0.39040922	1.22639873	0.22004867	0.99019711	Rxra	22.8495752	37.1076455	30.5355873	90.9981605	83.240586	44.6900719	25.0188611	118.696632
Sorcs2	28.3509596	-0.4289216	0.34985613	-1.2259941	0.22020088	0.99019711	Sorcs2	28.2259458	51.1351802	33.7163777	20.6814001	31.6314227	32.9295267	12.5094306	15.9783927
Eya4	3.23390098	0.51024306	0.41635611	1.22549674	0.22038812	NA	Eya4	2.68818531	2.26261859	3.18079035	0	1.66481172	7.05632714	6.73584722	2.28262753
Morc4	3.35603622	-0.5029699	0.41049145	-1.2252871	0.22046706	NA	Morc4	4.7043243	1.35757116	3.81694841	10.3407001	0	4.7042181	1.92452778	0
Kansl1	90.4961191	0.28421149	0.23197581	1.22517728	0.22050843	0.99019711	Kansl1	87.3660227	66.5209867	84.6090232	84.7937404	123.196067	101.140689	82.7546944	93.5877288
Ap3b2	111.617399	0.34545769	0.28200702	1.22499675	0.22057644	0.99019711	Ap3b2	96.7746713	76.9290072	97.3321846	113.747701	178.134854	104.668853	76.9811111	148.37079
Vapb	156.773926	0.33476785	0.27328353	1.22498362	0.22058139	0.99019711	Vapb	112.231737	101.817837	112.918057	223.359121	184.794101	157.591306	185.71693	175.76232
Nudcd3	121.492169	0.36229935	0.29583166	1.22468081	0.22069551	0.99019711	Nudcd3	88.038069	87.3370778	96.0598684	144.769801	201.442218	127.013889	78.9056389	148.37079
Dcun1d3	22.926761	0.36023118	0.29415967	1.22461103	0.22072181	0.99019711	Dcun1d3	18.1452509	20.3635674	21.6293743	18.6132601	21.6425524	29.4013631	30.7924444	22.8262753
Cep83	16.7422807	0.49539032	0.40458938	1.22442739	0.22079104	0.99019711	Cep83	20.8334362	9.05047438	16.2220308	2.06814001	4.99443516	28.2253086	36.5660278	15.9783927
Ccdc30	4.98811492	0.49520467	0.40445833	1.22436512	0.22081452	0.99019711	Ccdc30	3.36023164	3.16766603	4.45310648	4.13628002	3.32962344	8.23238167	8.660375	4.56525506
Kcnd1	3.29845189	-0.5003424	0.40873936	-1.2241112	0.2209103	NA	Kcnd1	4.03227797	8.59795066	4.77118552	0	0	1.17605452	0.96226389	6.84788259
Xpc	9.24904815	0.46400301	0.37917547	1.22371577	0.22105947	0.99019711	Xpc	7.39250961	7.2403795	5.40734359	10.3407001	8.3240586	18.8168724	9.62263889	6.84788259
Pld4	1.9574783	-0.4920922	0.4021397	-1.2236847	0.22107118	NA	Pld4	2.01613899	4.52523719	2.54463228	2.06814001	3.32962344	1.17605452	0	0
Entpd7	17.1725453	0.43031001	0.35181493	1.22311469	0.22128638	0.99019711	Entpd7	8.73660227	15.3858064	10.1785291	22.7495401	14.9833055	23.5210905	14.4339583	27.3915304
Zfp941	38.6172687	0.37062034	0.30315621	1.22253918	0.2215038	0.99019711	Zfp941	33.6023164	29.8665654	31.1717454	39.2946602	29.966611	68.2111624	47.1509305	29.6741579
Taf7	5.82412548	0.49934073	0.40847663	1.22244625	0.22153892	0.99019711	Taf7	5.37637063	4.07271347	5.40734359	2.06814001	3.32962344	10.5844907	13.4716944	2.28262753
Lmnb1	4.14835713	0.50703675	0.41477364	1.22244209	0.22154049	NA	Lmnb1	0.67204633	3.62018975	3.49886938	4.13628002	3.32962344	4.7042181	8.660375	4.56525506
Mpv17l2	6.87214973	-0.4882494	0.39944184	-1.2223291	0.22158321	0.99019711	Mpv17l2	13.4409266	9.95552182	6.36158069	4.13628002	3.32962344	7.05632714	3.84905555	6.84788259
Hspa9	81.6330492	0.35215328	0.288175	1.2220119	0.22170313	0.99019711	Hspa9	85.3498837	48.4200379	69.0231505	80.6574604	71.586904	135.24627	94.3018611	68.4788259
Iffo2	22.3148004	0.42657734	0.34917416	1.22167498	0.22183056	0.99019711	Iffo2	14.1129729	11.7656167	19.0847421	28.9539601	43.2851047	21.1689814	17.32075	22.8262753
Pigu	21.8361396	-0.4348787	0.35597279	-1.2216626	0.22183524	0.99019711	Pigu	14.1129729	40.274611	25.7644018	24.8176801	11.653682	27.0492541	17.32075	13.6957652
Aaas	10.7004931	-0.4551349	0.37262393	-1.2214322	0.22192244	0.99019711	Aaas	14.1129729	13.1231878	11.1327662	12.4088401	6.65924688	3.52816357	8.660375	15.9783927

GeneID	Base mean	log2(FC)	StdErr	Wald-Stats	P-value	P-adj	GeneID	Normalised expression for Chow#1	Normalised expression for Chow#2	Normalised expression for Chow#3	Normalised expression for Chow#4	Normalised expression for HFD#1	Normalised expression for HFD#2	Normalised expression for HFD#3	Normalised expression for HFD#4
Thnsl1	19.3438643	0.40814635	0.3341713	1.22136866	0.22194647	0.99019711	Thnsl1	18.1452509	17.648425	19.0847421	8.27256004	23.3073641	27.0492541	29.8301805	11.4131377
Pou6f1	69.4530993	0.3344885	0.27398777	1.22081543	0.22215591	0.99019711	Pou6f1	63.8444012	62.4482732	58.5265423	53.7716403	111.542385	56.4506172	57.7358333	91.3051012
Dynlrb1	139.118613	-0.4345498	0.35595836	-1.2207883	0.2221662	0.99019711	Dynlrb1	186.156833	184.629677	186.712393	109.611421	53.2739751	136.422325	205.924472	50.2178057
Vps28	65.9545988	-0.3482027	0.28522907	-1.2207828	0.22216828	0.99019711	Vps28	79.9735131	57.0179886	66.7965972	99.2707205	39.9554813	44.6900719	76.0188472	63.9135709
Ccnk	33.739605	-0.3600707	0.29495328	-1.2207722	0.2221723	0.99019711	Ccnk	30.9141311	42.9897533	31.1717454	51.7035003	23.3073641	36.4576902	25.981125	27.3915304
Atp6v1h	94.4798306	0.28633951	0.23457559	1.22067052	0.22221079	0.99019711	Atp6v1h	89.3821617	76.0239848	88.1078926	84.7937404	73.2517157	119.957561	119.320722	105.000866
Atp5j2	24.4574532	-0.3930364	0.32199712	-1.2206208	0.22222963	0.99019711	Atp5j2	28.2259458	19.9110436	29.2632712	37.2265202	16.6481172	12.9365998	24.0565972	27.3915304
Snx24	5.50961329	-0.5060702	0.41464839	-1.2204802	0.22228289	0.99019711	Snx24	6.72046328	6.33533207	7.31581779	10.3407001	0	4.7042181	8.660375	0
Dtna	95.9347558	-0.3387001	0.27755505	-1.220299	0.22235156	0.99019711	Dtna	119.624246	125.801594	119.279638	70.3167603	63.2628454	91.7322529	113.547139	63.9135709
Rbm4b	19.583362	0.41716162	0.34193491	1.22000303	0.22246373	0.99019711	Rbm4b	12.0968339	12.6706641	13.3593194	28.9539601	16.6481172	17.6408179	27.9056528	27.3915304
E130309F12Rik	12.5263952	-0.4885063	0.40050045	-1.2197396	0.22256361	0.99019711	E130309F12Ri	15.4570656	23.5312334	17.4943469	6.20442003	1.66481172	19.9929269	6.73584722	9.13051012
Cpeb2	78.4478343	0.29365973	0.24080307	1.21950162	0.22265386	0.99019711	Cpeb2	63.1723549	62.4482732	70.9316247	84.7937404	63.2628454	87.0280348	100.075444	95.8703563
Lysmd2	34.7215341	-0.3861922	0.3166829	-1.2194917	0.22265764	0.99019711	Lysmd2	37.6345944	53.8503226	38.8056422	31.0221002	16.6481172	25.8731995	44.2641389	29.6741579
Fbxo21	141.883006	0.41472495	0.34013393	1.21929897	0.22273074	0.99019711	Fbxo21	107.527413	87.3370778	99.2406588	171.655621	259.710628	87.0280348	94.3018611	228.262753
Ecm1	2.21517991	-0.4958591	0.40678423	-1.2189732	0.22285435	NA	Ecm1	2.68818531	5.43028463	4.13502745	0	3.32962344	1.17605452	0.96226389	0
Gm5136	1.17033546	0.37272816	0.30591469	1.21840555	0.2230699	NA	Gm5136	1.34409266	0	0	0	0	7.05632714	0.96226389	0
Gabpa	12.227357	-0.4425899	0.36328016	-1.2183155	0.2231041	0.99019711	Gabpa	16.1291119	19.0059962	15.2677937	8.27256004	4.99443516	10.5844907	14.4339583	9.13051012
Ndnl2	12.7477678	0.44129123	0.36222858	1.21826729	0.22312242	0.99019711	Ndnl2	14.7850192	7.69290322	7.63389683	10.3407001	11.653682	16.4647633	10.5849028	22.8262753
4930513N10Rik	2.04058141	0.49557606	0.40679362	1.21824934	0.22312923	NA	4930513N10R	1.34409266	1.81009488	1.27231614	0	6.65924688	2.35210905	2.88679167	0
Trex1	4.81453673	0.50118051	0.4114836	1.21798418	0.22322998	0.99019711	Trex1	4.03227797	3.16766603	3.18079035	2.06814001	4.99443516	3.52816357	3.84905555	13.6957652
Gcn1l1	54.0964995	0.46144621	0.37888782	1.21789665	0.22326325	0.99019711	Gcn1l1	34.9464091	28.0564706	30.2175083	76.5211804	123.196067	35.2816357	26.9433889	77.6093361
Myt1	10.7771318	0.43311851	0.35568277	1.21771014	0.22333414	0.99019711	Myt1	9.4086486	10.8605693	6.67965972	8.27256004	13.3184938	14.1126543	14.4339583	9.13051012
Atf1	5.80383278	0.50690885	0.41628393	1.21769976	0.22333809	0.99019711	Atf1	4.03227797	4.97776091	5.08926455	0	11.653682	0	11.5471667	9.13051012
Map3k19	3.18678818	-0.5050011	0.41480031	-1.2174559	0.22343079	NA	Map3k19	4.03227797	5.43028463	7.95197586	0	1.66481172	3.52816357	2.88679167	0
Wdr75	11.0489289	0.45751691	0.37581247	1.21740746	0.22344923	0.99019711	Wdr75	11.4247876	6.78785578	11.4508452	4.13628002	8.3240586	22.345036	12.5094306	11.4131377
Nfat5	117.136455	0.31154359	0.25593255	1.21728785	0.22349472	0.99019711	Nfat5	77.9573741	97.2925996	98.9225797	140.633521	128.190502	154.063143	96.2263889	143.805534
Mrps21	8.03344253	-0.4858699	0.39917264	-1.2171924	0.22353103	0.99019711	Mrps21	13.4409266	12.2181404	10.8146872	4.13628002	1.66481172	5.88027262	11.5471667	4.56525506
Alas1	31.2286848	0.34233874	0.28134143	1.21680885	0.22367695	0.99019711	Alas1	30.9141311	28.5089943	23.8559276	22.7495401	34.9610461	35.2816357	27.9056528	45.6525506
Slc2a1	27.5190472	-0.5044074	0.41454532	-1.2167726	0.22369074	0.99019711	Slc2a1	10.0806949	79.1916508	34.9886938	18.6132601	46.6147282	4.7042181	7.69811111	18.2610202
Tcp11l1	27.8492055	0.36924897	0.30353719	1.21648673	0.22379956	0.99019711	Tcp11l1	23.5216215	23.9837571	26.7186389	20.6814001	39.9554813	41.1619083	30.7924444	15.9783927
1700001K19Rik	1.39916294	0.41616229	0.34212599	1.21640068	0.22383232	NA	1700001K19Ri	0.67204633	0	0	2.06814001	4.99443516	1.17605452	0	2.28262753
Esr2	2.71047246	0.48920004	0.40221568	1.21626299	0.22388476	NA	Esr2	0.67204633	1.35757116	0.63615807	4.13628002	3.32962344	4.7042181	0	6.84788259
Gng7	151.866971	-0.285061	0.23444081	-1.2159189	0.22401583	0.99019711	Gng7	200.941852	149.785351	194.664369	124.088401	139.844185	112.901234	144.339583	148.37079
Ccdc19	1.97438392	0.48634181	0.40003398	1.21575127	0.22407969	NA	Ccdc19	2.01613899	0.45252372	0.63615807	2.06814001	0	3.52816357	4.81131944	2.28262753
Hspb6	8.45048868	-0.4896246	0.40281125	-1.2155187	0.22416832	0.99019711	Hspb6	7.39250961	8.14542694	8.2700549	20.6814001	3.32962344	2.35210905	10.5849028	6.84788259
Pik3r6	1.52173871	0.44766524	0.3686523	1.21432916	0.22462205	NA	Pik3r6	0.67204633	0	0.63615807	2.06814001	6.65924688	1.17605452	0.96226389	0
Ncbp1	30.0254555	-0.3682591	0.3032843	-1.2142372	0.22465715	0.99019711	Ncbp1	39.6507334	31.2241366	38.8056422	28.9539601	14.9833055	25.8731995	35.6037639	25.1089028
Dhx16	12.2254986	0.43262752	0.35641026	1.213847	0.22480616	0.99019711	Dhx16	10.7527413	8.59795066	8.58813393	12.4088401	23.3073641	10.5844907	14.4339583	9.13051012
Dnal1	130.056563	0.28109119	0.23157729	1.21381155	0.22481969	0.99019711	Dnal1	112.903783	142.997495	117.053085	90.9981605	124.860879	152.887088	173.2075	125.544514
9230110C19Rik	9.22619684	-0.4642058	0.38252663	-1.2135255	0.22492898	0.99019711	9230110C19Ri	11.4247876	14.0282353	8.58813393	10.3407001	3.32962344	4.7042181	7.69811111	13.6957652
Gria4	89.1247024	0.46062031	0.37966537	1.2132271	0.22504301	0.99019711	Gria4	63.8444012	83.2643643	96.0598684	33.0902402	39.9554813	235.210905	88.5282777	73.044081
Ppm1b	64.0235088	0.3355468	0.27660606	1.21308548	0.22509714	0.99019711	Ppm1b	51.075521	41.1796584	55.345752	76.5211804	53.2739751	63.5069443	73.1320555	98.1529838
Hk1	222.31356	0.37843805	0.31200629	1.21291803	0.22516116	0.99019711	Hk1	226.479613	155.215636	141.227091	227.495401	424.526989	192.872942	148.188639	262.502166
Gyg	19.5229771	-0.4324747	0.35662991	-1.2126708	0.22525571	0.99019711	Gyg	24.1936678	28.5089943	28.3090341	12.4088401	6.65924688	23.5210905	21.1698055	11.4131377
Ripk2	2.68577543	-0.4962047	0.40920178	-1.2126162	0.2252766	NA	Ripk2	3.36023164	1.35757116	8.2700549	2.06814001	3.32962344	1.17605452	1.92452778	0
Dchs1	23.0248704	0.44924622	0.37049522	1.21255607	0.22529959	0.99019711	Dchs1	13.4409266	16.7433776	15.9039517	26.8858201	39.9554813	27.0492541	7.69811111	36.5220405
Mapk4	70.3645808	-0.3444789	0.28411906	-1.2124457	0.22534181	0.99019711	Mapk4	95.4305786	88.6946489	76.6570473	55.8397803	84.9053977	39.9858538	52.9245139	68.4788259
F2r	11.6645908	0.44228551	0.36496728	1.21184974	0.2255699	0.99019711	F2r	8.06455594	9.5029981	9.54237104	10.3407001	9.98887032	23.5210905	8.660375	13.6957652
Zfp521	9.55300312	-0.48114	0.39710817	-1.2116095	0.22566191	0.99019711	Zfp521	6.04841696	14.0282353	12.7231614	14.4769801	13.3184938	7.05632714	1.92452778	6.84788259
Cdk4	25.8745118	-0.4201537	0.3467964	-1.2115284	0.22569295	0.99019711	Cdk4	29.5700384	21.7211385	27.990955	45.4990802	36.6258578	15.2887088	21.1698055	9.13051012
Add3	64.5567671	-0.3450868	0.28484646	-1.2114836	0.22571011	0.99019711	Add3	79.3014668	72.8563187	63.6158069	80.6574604	74.9165274	49.39429	68.3207361	27.3915304
Pank4	12.4248883	0.44946652	0.37106332	1.2112933	0.22578301	0.99019711	Pank4	9.4086486	8.14542694	8.90621297	12.4088401	23.3073641	7.05632714	9.62263889	20.5436478
Cbln2	9.52224263	-0.4933295	0.40735597	-1.2110525	0.22587529	0.99019711	Cbln2	9.4086486	16.7433776	11.1327662	12.4088401	4.99443516	17.6408179	3.84905555	0
Gng2	284.38426	0.34267657	0.28296678	1.21101344	0.22589026	0.99019711	Gng2	257.393744	273.77685	269.412942	181.996321	198.112595	353.992412	480.16968	260.219539
Dio3	1.61992246	0.42020145	0.34698787	1.21099752	0.22589636	NA	Dio3	0.67204633	0	1.59039517	0	0	0	3.84905555	6.84788259
Six3	46.0720651	-0.4910601	0.40550442	-1.2109858	0.22590084	0.99019711	Six3	38.978687	21.2686148	36.2610099	138.565381	41.620293	29.4013631	7.69811111	54.7830607
Dyrk2	16.3295479	0.43559348	0.35977356	1.21074343	0.22599375	0.99019711	Dyrk2	18.8172972	15.3858064	14.3135566	2.06814001	18.3129289	18.8168724	26.9433889	15.9783927
Trim41	34.0141496	0.43222781	0.35704248	1.21057813	0.22605713	0.99019711	Trim41	22.8495752	13.1231878	24.1740066	51.7035003	48.2795399	23.5210905	33.6792361	54.7830607
Hcfc1	99.4219711	0.30059456	0.24831285	1.21054771	0.22606879	0.99019711	Hcfc1	84.0057911	84.1694117	86.1994184	97.2025805	154.82749	97.6215255	81.7924305	109.566121
C030029H02Rik	1.52866893	-0.4667052	0.3855492	-1.2104946	0.22608917	NA	C030029H02Ri	1.34409266	3.16766603	0.9542371	4.13628002	1.66481172	0	0.96226389	0
Dcp2	81.4999231	0.34860901	0.28801771	1.21037352	0.2261356	0.99019711	Dcp2	59.8121232	71.0462239	79.2016796	72.3849004	63.2628454	142.302597	100.075444	63.9135709
Dph1	15.1700399	0.44636785	0.36883374	1.21021425	0.22619669	0.99019711	Dph1	10.7527413	11.7656167	6.36158069	20.6814001	11.653682	14.1126543	16.3584861	29.6741579
Dab2ip	114.953873	0.42267055	0.34929288	1.2100749	0.22625015	0.99019711	Dab2ip	78.6294204	73.7613662	69.0231505	153.042361	166.481172	81.1477622	69.283	228.262753
Fosb	16.5307441	-0.4322027	0.35726857	-1.2097419	0.22637796	0.99019711	Fosb	16.1291119	20.8160911	20.3570582	20.6814001	21.6425524	14.1126543	4.81131944	13.6957652
Uso1	56.2291281	0.42404606	0.35070849	1.20911259	0.22661959	0.99019711	Uso1	48.3873356	48.8725616	62.9796488	20.6814001	38.2906696	122.309671	55.8113055	52.5004332
Ppt2	14.0035599	-0.4651316	0.38468939	-1.2091095	0.22662079	0.99019711	Ppt2	8.06455594	25.793852	19.4028211	14.4769801	19.9777406	9.40843619	5.77358333	9.13051012
Tpd52l2	23.272237	0.37083249	0.30678942	1.20875252	0.22675794	0.99019711	Tpd52l2	16.8011582	20.3635674	20.3570582	22.7495401	16.6481172	35.2816357	28.8679167	25.1089028
Kdm4b	31.6774348	0.44949212	0.37201254	1.20827144	0.22694287	0.99019711	Kdm4b	23.5216215	15.3858064	25.7644018	35.1583802	66.5924688	22.345036	14.4339583	50.2178057
Gm20605	16.6806555	0.48953468	0.40516924	1.20822271	0.22696161	0.99019711	Gm20605	6.72046328	7.69290322	5.40734359	31.0221002	23.3073641	14.1126543	8.660375	36.5220405
Tceal7	0.91423994	-0.4182433	0.34625574	-1.2079029	0.22708461	NA	Tceal7	2.68818531	0.90504744	2.54463228	0	0	1.17605452	0	0
Tlr4	1.35717542	-0.4537146	0.37563392	-1.2078638	0.22709964	NA	Tlr4	1.34409266	4.97776091	1.90847421	0	1.66481172	0	0.96226389	0
Dpm3	8.97630373	-0.5023646	0.41608586	-1.207358	0.22729429	0.99019711	Dpm3	4.7043243	9.5029981	5.40734359	28.9539601	6.65924688	0	2.88679167	13.6957652
Vps37b	12.0548413	-0.4357523	0.36091651	-1.2073493	0.22729764	0.99019711	Vps37b	15.4570656	13.1231878	12.0870033	16.5451201	14.9833055	7.05632714	5.77358333	11.4131377
Ptk7	4.671442	0.50220334	0.41622872	1.2065562	0.2276031	0.99019711	Ptk7	2.01613899	2.71514231	1.59039517	6.20442003	6.65924688	3.52816357	0.96226389	13.6957652
Scamp2	8.39397797	-0.4828832	0.40042598	-1.2059238	0.22784687	0.99019711	Scamp2	6.04841696	17.1959013	9.54237104	8.27256004	11.653682	4.7042181	2.88679167	6.84788259
Zfp518b	17.9161878	0.40527356	0.33611271	1.20576683	0.22790741	0.99019711	Zfp518b	13.4409266	18.1009488	16.5401098	10.3407001	11.653682	30.5774176	22.1320694	20.5436478
Zc3h7b	145.262392	0.37638129	0.3121891	1.20561957	0.22796421	0.99019711	Zc3h7b	119.624246	118.561214	98.9225797	153.042361	251.38657	102.316744	99.1131805	219.132243
Parg	37.0044747	0.37776356	0.31347366	1.20508869	0.22816906	0.99019711	Parg	30.9141311	27.6039469	33.7163777	35.1583802	26.6369875	48.2182355	66.3962083	27.3915304
Ppp2r1b	21.1375634	0.36913542	0.30632977	1.20502629	0.22819315	0.99019711	Ppp2r1b	18.1452509	15.8383302	16.5401098	22.7495401	26.6369875	29.4013631	19.2452778	20.5436478
Brd4	175.151599	-0.2663087	0.22105723	-1.2047046	0.22831737	0.99019711	Brd4	184.140694	209.065958	203.570582	169.587481	176.470042	165.823688	114.509403	178.044947
6330416G13Rik	10.9309982	0.4654638	0.38638014	1.20467837	0.22832749	0.99019711	6330416G13Ri	10.7527413	4.97776091	7.31581779	10.3407001	14.9833055	4.7042181	11.5471667	22.8262753
Slc16a1	9.48415519	-0.4573166	0.37965562	-1.2045564	0.22837459	0.99019711	Slc16a1	10.0806949	17.648425	9.86045007	8.27256004	11.653682	7.05632714	6.73584722	4.56525506
Nin	18.9675179	0.4242521	0.35221359	1.20453077	0.2283845	0.99019711	Nin	22.8495752	12.2181404	16.2220308	10.3407001	16.6481172	37.6337448	22.1320694	13.6957652
Spi1	0.94203169	-0.4347789	0.36097396	-1.2044604	0.22841167	NA	Spi1	1.34409266	1.35757116	1.59039517	2.06814001	0	1.17605452	0	0
Nostrin	0.58692413	-0.359176	0.29829219	-1.2041078	0.2285479	NA	Nostrin	1.34409266	2.71514231	0.63615807	0	0	0	0	0
Slc8a3	32.4492746	0.42764951	0.35518221	1.20402852	0.22857855	0.99019711	Slc8a3	15.4570656	36.2018975	26.0824808	26.8858201	26.6369875	67.0351079	20.2075417	41.0872956
Ssbp1	3.23823844	0.50003341	0.41532208	1.20396538	0.22860296	NA	Ssbp1	2.01613899	1.81009488	1.90847421	2.06814001	1.66481172	1.17605452	3.84905555	11.4131377
4933407L21Rik	1.49505776	-0.468278	0.38895869	-1.2039274	0.22861763	NA	4933407L21Ri	1.34409266	2.26261859	1.59039517	4.13628002	1.66481172	0	0.96226389	0
Wdsub1	7.19964445	-0.4894905	0.40658436	-1.2039087	0.22862485	0.99019711	Wdsub1	6.72046328	5.88280835	7.63389683	16.5451201	6.65924688	1.17605452	3.84905555	9.13051012
Vcp	69.0350632	0.35315	0.29333876	1.20389819	0.22862893	0.99019711	Vcp	72.5810035	47.0217296	61.0711746	59.9760603	119.866444	67.0351079	53.8867778	75.3267085
Fgf12	296.696311	0.41279797	0.34294528	1.20368466	0.22871148	0.99019711	Fgf12	254.705558	244.362808	308.218584	165.451201	151.497867	492.766846	546.565889	210.001733
Sh3yl1	10.1148169	0.4685629	0.38932995	1.20351107	0.22877861	0.99019711	Sh3yl1	10.7527413	6.33533207	7.95197586	6.20442003	8.3240586	5.88027262	24.0565972	11.4131377
Arhgef10	15.9052745	-0.438317	0.36429406	-1.2031956	0.22890066	0.99019711	Arhgef10	17.4732045	13.1231878	20.0389792	26.8858201	23.3073641	7.05632714	12.5094306	6.84788259
Uchl5	20.2426407	0.43791122	0.36397644	1.2031307	0.22892575	0.99019711	Uchl5	20.8334362	16.2908539	20.6751372	6.20442003	13.3184938	28.2253086	40.4150833	15.9783927
Abi3bp	2.35827371	-0.4627676	0.3847125	-1.202892	0.22901811	NA	Abi3bp	2.68818531	5.43028463	6.04350166	0	0	4.7042181	0	0
Traf3ip2	3.21870646	0.50061173	0.41623962	1.2027008	0.22909212	NA	Traf3ip2	2.68818531	1.35757116	2.54463228	2.06814001	8.3240586	5.88027262	2.88679167	0
Cdc37	70.150401	0.42278451	0.35155014	1.20262933	0.22911979	0.99019711	Cdc37	53.7637063	28.0564706	35.6248519	113.747701	89.8998329	67.0351079	63.5094166	109.566121
Slc52a2	8.99428928	0.48450305	0.4029573	1.2023682	0.2292209	0.99019711	Slc52a2	4.7043243	5.43028463	4.45310648	12.4088401	11.653682	4.7042181	5.77358333	22.8262753
Sec22b	45.8641348	0.31749606	0.26407695	1.20228618	0.22925267	0.99019711	Sec22b	47.043243	33.0342315	41.0321955	39.2946602	39.9554813	45.8661264	56.7735694	63.9135709
Cyb5	23.5267695	-0.4944823	0.41131812	-1.2021894	0.22929016	0.99019711	Cyb5	38.3066407	38.0119924	39.4418003	6.20442003	6.65924688	18.8168724	38.4905555	2.28262753
Iqsec3	158.80454	0.39412041	0.32784656	1.20214901	0.2293058	0.99019711	Iqsec3	93.4144396	99.5552182	114.826531	223.359121	261.37544	156.415252	100.075444	221.414871
Atpif1	77.7224175	-0.3287642	0.27357798	-1.2017203	0.22947191	0.99019711	Atpif1	103.495135	88.2421252	74.7485731	84.7937404	44.9499164	52.9224536	90.4528055	82.1745911
Osmr	1.63405346	-0.4715435	0.39246004	-1.201507	0.22955461	NA	Osmr	2.68818531	4.52523719	2.54463228	0	0	2.35210905	0.96226389	0
Zwint	132.225124	0.38830698	0.32326667	1.20119709	0.22967476	0.99019711	Zwint	144.489961	105.89055	121.506191	70.3167603	76.5813391	209.337705	215.547111	114.131377
Gm10767	1.75796277	0.47534073	0.39578922	1.20099464	0.22975328	NA	Gm10767	1.34409266	1.81009488	0.31807903	0	4.99443516	2.35210905	0.96226389	2.28262753
H2afy2	10.6380454	0.46783871	0.38976001	1.20032507	0.23001312	0.99019711	H2afy2	13.4409266	5.43028463	6.99773876	6.20442003	8.3240586	5.88027262	18.2830139	20.5436478
Nrd1	72.0987379	0.39367482	0.32797837	1.20030727	0.23002003	0.99019711	Nrd1	65.8605402	65.1634155	77.2932054	31.0221002	76.5813391	115.253343	106.811292	38.804668
Tdp2	9.26220219	0.46864701	0.39053508	1.20001256	0.23013446	0.99019711	Tdp2	8.06455594	8.14542694	10.4966081	0	8.3240586	16.4647633	13.4716944	9.13051012
Man1b1	39.6271573	-0.3430844	0.2859505	-1.1998035	0.23021568	0.99019711	Man1b1	36.9625481	53.8503226	48.0299342	41.3628002	33.2962344	48.2182355	27.9056528	27.3915304
Cars	12.1902513	0.42748749	0.35634456	1.19964645	0.23027668	0.99019711	Cars	10.7527413	12.2181404	10.8146872	4.13628002	11.653682	11.7605452	20.2075417	15.9783927
Samm50	40.2770996	0.39940314	0.33300453	1.19939251	0.23037536	0.99019711	Samm50	18.8172972	24.4362808	32.7621406	59.9760603	46.6147282	35.2816357	40.4150833	63.9135709
Unc119b	23.1676252	-0.467154	0.38955935	-1.1991858	0.23045571	0.99019711	Unc119b	18.8172972	25.3413283	15.5858727	55.8397803	34.9610461	8.23238167	10.5849028	15.9783927

GeneID	Base mean	log2(FC)	StdErr	Wald-Stats	P-value	P-adj		GeneID	Normalised expression for Chow#1	Normalised expression for Chow#2	Normalised expression for Chow#3	Normalised expression for Chow#4	Normalised expression for HFD#1	Normalised expression for HFD#2	Normalised expression for HFD#3	Normalised expression for HFD#4
Igdcc4	29.7131542	-0.3961721	0.33053139	-1.1985914	0.23068686	0.99019711		Igdcc4	24.1936678	40.274611	32.4440615	41.3628002	38.2906696	23.5210905	12.5094306	25.1089028
Tapbp	18.7294901	-0.4109988	0.34298535	-1.1982985	0.23080084	0.99019711		Tapbp	16.1291119	20.8160911	21.9474534	28.9539601	23.3073641	11.7605452	8.660375	18.2610202
Zranb2	57.5259374	0.45097759	0.37640531	1.19811698	0.23087148	0.99019711		Zranb2	40.994826	57.0179886	63.2977279	18.6132601	29.966611	103.492798	112.584875	34.239413
Nelfb	42.9013677	0.42764867	0.35695554	1.19804463	0.23089964	0.99019711		Nelfb	28.8979921	26.2463757	24.4920857	59.9760603	58.2684102	25.8731995	32.7169722	86.7398462
Snrpn	486.709232	-0.2881928	0.24055412	-1.1980372	0.23090253	0.99019711		Snrpn	654.573124	519.044706	553.45752	430.173122	341.286403	342.231867	511.924389	540.982725
Lsm12	26.3931251	-0.3657999	0.30533591	-1.1980245	0.2309075	0.99019711		Lsm12	30.2420848	27.6039469	37.5333261	26.8858201	19.9777406	21.1689814	31.7547083	15.9783927
Bahcc1	33.6652305	0.46402543	0.38732937	1.1980125	0.23091215	0.99019711		Bahcc1	20.1613899	28.0564706	18.130505	37.2265202	83.240586	18.8168724	13.4716944	50.2178057
Cpm	3.55044688	-0.4961892	0.41443783	-1.1972585	0.2312058	NA		Cpm	2.68818531	9.05047438	6.04350166	2.06814001	0	4.7042181	3.84905555	0
Srgap2	66.8345887	0.29336036	0.2450377	1.19720497	0.23122667	0.99019711		Srgap2	51.7475673	58.8280835	64.251965	64.1123403	64.9276571	97.6125255	69.283	63.9135709
Hspa4	174.024459	0.35877448	0.29967826	1.19719888	0.23122905	0.99019711		Hspa4	160.619072	147.522732	165.401098	122.020261	136.514561	321.062885	220.35843	118.696632
Ubl4	42.24673	-0.3323027	0.27759846	-1.1970626	0.23128217	0.99019711		Ubl4	43.010965	48.4200379	55.663831	43.4309402	43.2851047	24.697145	45.2264028	34.239413
Rprd1b	33.7474569	0.37831035	0.31605922	1.19696034	0.23132201	0.99019711		Rprd1b	28.8979921	17.1959013	30.5355873	37.2265202	43.2851047	28.2253086	29.8301805	54.7830607
Ndufa9	103.729647	0.35346292	0.29540625	1.19653162	0.23148917	0.99019711		Ndufa9	79.3014668	64.2583681	72.2039408	142.701661	111.542385	94.0843619	99.1131805	166.63181
Nmbr	3.07004595	0.49138548	0.41069351	1.19647735	0.23151033	NA		Nmbr	1.34409266	4.52523719	1.27231614	0	1.66481172	10.5844907	2.88679167	2.28262753
Smim20	10.1229553	-0.4777025	0.39933137	-1.1962558	0.23159673	0.99019711		Smim20	7.39250961	11.7656167	9.86045007	22.7495401	8.3240586	1.17605452	10.5849028	9.13051012
Tmem237	10.0981105	-0.4467483	0.37346576	-1.1962229	0.23160957	0.99019711		Tmem237	10.7527413	16.2908539	13.6773985	8.27256004	11.653682	8.23238167	9.62263889	2.28262753
Ptprt	245.414345	0.43048653	0.35990441	1.19611352	0.23165226	0.99019711		Ptprt	156.586795	288.710133	237.605039	105.475141	198.112595	583.323044	240.565972	152.936045
Notch3	7.87320347	-0.4979926	0.4163707	-1.1960318	0.23168415	0.99019711		Notch3	9.4086486	12.6706641	7.63389683	12.4088401	14.9833055	5.88027262	0	0
Rgs8	143.677602	0.35545553	0.2972242	1.19591719	0.23172887	0.99019711		Rgs8	100.806949	101.817837	129.776246	159.246781	209.766277	132.894161	91.4150694	223.697498
Krt9	3.19496532	0.49791043	0.41636354	1.19585502	0.23175314	NA		Krt9	1.34409266	1.81009488	3.49886938	2.06814001	3.32962344	3.52816357	7.69811111	2.28262753
Ndufaf5	20.4518093	-0.4033794	0.33751114	-1.1951589	0.23202495	0.99019711		Ndufaf5	24.8657142	18.5534725	24.4920857	31.0221002	13.3184938	15.2887088	26.9433889	9.13051012
Parvb	26.897191	-0.4637865	0.38807053	-1.1951088	0.23204453	0.99019711		Parvb	15.4570656	22.6261859	25.4463228	70.3167603	31.6314227	8.23238167	16.3584861	25.1089028
Bcl2l13	54.6475896	0.3142244	0.26295872	1.19495715	0.23210377	0.99019711		Bcl2l13	42.3389187	42.5372296	52.4830407	53.7716403	78.2461509	54.0985081	45.2264028	68.4788259
Rab11fip3	86.8873996	0.34786177	0.29114508	1.19480559	0.23216299	0.99019711		Rab11fip3	72.5810035	66.5209867	59.4807795	101.338861	123.196067	92.9083074	55.8113055	123.261887
Srr	67.2104011	-0.3959028	0.3313645	-1.1947653	0.23217874	0.99019711		Srr	92.7423933	95.029981	89.3802087	37.2265202	34.9610461	72.9153805	78.9056389	36.5220405
Cenpe	0.76875317	0.35394615	0.29627733	1.19464472	0.23222587	NA		Cenpe	0	0	0.63615807	0	1.66481172	0	3.84905555	0
Larp6	27.0848133	0.37720987	0.31581219	1.19441201	0.23231684	0.99019711		Larp6	21.5054825	17.1959013	21.9474534	31.0221002	36.6258578	19.9929269	25.0188611	43.3699231
Nlgn2	288.766816	0.40244618	0.33705856	1.19399481	0.23248	0.99019711		Nlgn2	167.339536	280.564706	189.575105	316.425422	481.130587	285.781249	132.792417	456.525506
Zbtb48	3.03505254	0.49620877	0.41560054	1.19395602	0.23249517	NA		Zbtb48	0.67204633	1.81009488	2.86271131	2.06814001	4.99443516	1.17605452	3.84905555	6.84788259
Nup155	15.1628583	0.38978863	0.32654563	1.19367278	0.23260599	0.99019711		Nup155	11.4247876	13.5757116	14.6316356	10.3407001	16.6481172	22.345036	16.3584861	15.9783927
Pisd	34.5276199	-0.3490954	0.29247976	-1.1935713	0.23264573	0.99019711		Pisd	36.2905017	34.3918026	36.2610099	51.7035003	38.2906696	18.8168724	30.7924444	29.6741579
Atp5c1	48.0796999	-0.3714665	0.31123607	-1.1935201	0.23266574	0.99019711		Atp5c1	52.4196136	51.1351802	42.3045116	80.6574604	33.2962344	42.3379629	59.6603611	22.8262753
Kcnmb4os1	3.29650627	0.47958977	0.40190478	1.19329201	0.23275503	NA		Kcnmb4os1	0.67204633	1.35757116	1.90847421	4.13628002	14.9833055	2.35210905	0.96226389	0
Decr1	9.5942229	-0.4428564	0.37124889	-1.1928827	0.2329153	0.99019711		Decr1	10.0806949	11.313093	13.3593194	12.4088401	9.98887032	10.5844907	6.73584722	2.28262753
Tmem138	4.71211798	-0.493175	0.41361911	-1.192341	0.23312756	0.99019711		Tmem138	6.04841696	7.69290322	6.99773876	4.13628002	1.66481172	1.17605452	7.69811111	2.28262753
Gper1	1.06586265	-0.4264891	0.35777247	-1.1920681	0.23323456	NA		Gper1	0.67204633	3.16766603	0.9542371	2.06814001	1.66481172	0	0	0
Eif5b	49.1097001	0.35947091	0.30158582	1.19193572	0.23328645	0.99019711		Eif5b	51.7475673	44.3473245	44.5310648	26.8858201	41.620293	74.091435	73.1320555	36.5220405
Kcnab1	101.52991	0.34308712	0.28793186	1.19155666	0.23343513	0.99019711		Kcnab1	112.903783	70.5937002	111.645741	53.7716403	89.8998329	139.950488	137.603736	95.8703563
D930016D06Rik	3.37951798	0.4951029	0.41560187	1.19129133	0.23353924	NA		D930016D06R	2.68818531	2.26261859	3.49886938	0	3.32962344	5.88027262	4.81131944	4.56525506
Gyk	13.3859761	0.44699489	0.37523372	1.19124393	0.23355784	0.99019711		Gyk	12.0968339	9.05047438	16.2220308	4.13628002	11.653682	18.8168724	25.981125	9.13051012
Cbr3	2.03613861	0.48846821	0.41006164	1.19120679	0.23357242	NA		Cbr3	1.34409266	0.90504744	2.22655324	0	3.32962344	2.35210905	3.84905555	2.28262753
Pi4ka	219.308619	0.24781862	0.20805132	1.19114175	0.23359794	0.99019711		Pi4ka	211.694593	189.154915	201.02595	194.405161	298.001298	255.203832	197.264097	207.719105
Uck1	14.1554197	-0.4855912	0.40767779	-1.1911153	0.23360833	0.99019711		Uck1	18.1452509	8.59795066	6.67965972	41.3628002	11.653682	5.88027262	16.3584861	4.56525506
H2afx	34.0878417	-0.4629852	0.38878419	-1.1908541	0.23371088	0.99019711		H2afx	38.3066407	23.5312334	31.1717454	74.4530404	29.966611	14.1126543	8.660375	52.5004332
Apol8	1.4265685	0.45841022	0.38496988	1.19076904	0.23374426	NA		Apol8	0.67204633	0.45252372	1.59039517	0	0	3.52816357	2.88679167	2.28262753
1500009L16Rik	7.15665975	-0.4598356	0.38617785	-1.1907354	0.23375747	0.99019711		1500009L16Ri	8.73660227	9.05047438	8.2700549	10.3407001	4.99443516	5.88027262	7.69811111	2.28262753
Uhrf1bp1	28.6894399	0.45363828	0.38109734	1.19034756	0.23390982	0.99019711		Uhrf1bp1	16.1291119	12.6706641	16.8581888	45.4990802	34.9610461	30.5774176	13.4716944	59.3483158
Fhod1	2.89620096	0.48084903	0.40396132	1.19033433	0.23391501	NA		Fhod1	0.67204633	1.35757116	0.9542371	4.13628002	4.99443516	0	1.92452778	9.13051012
Rapgef5	45.5077602	0.42373073	0.35599402	1.19027483	0.23393839	0.99019711		Rapgef5	40.3227797	46.6099431	46.7576181	12.4088401	49.9443516	68.2111624	76.9811111	22.8262753
Pura	57.5563759	0.32839711	0.27593675	1.19011732	0.23400028	0.99019711		Pura	61.8282622	43.442277	47.0756971	45.4990802	66.5924688	55.2745626	47.1509305	93.5877288
Lcp1	2.38696882	0.46874067	0.39390211	1.18999278	0.23404923	NA		Lcp1	0.67204633	0.90504744	0.31807903	4.13628002	3.32962344	0	2.88679167	6.84788259
Spry3	17.4892291	0.39108677	0.32879908	1.18943998	0.23426658	0.99019711		Spry3	16.1291119	17.648425	15.9039517	6.20442003	21.6425524	16.4647633	23.0943333	22.8262753
B430010I23Rik	0.84160753	-0.4104721	0.34511482	-1.1893784	0.2342908	NA		B430010I23Ri	2.68818531	1.81009488	1.27231614	0	0	0	0.96226389	0
Abcb10	18.6588355	0.42221277	0.35499736	1.1893406	0.23430567	0.99019711		Abcb10	10.7527413	14.480759	15.9039517	18.6132601	11.653682	22.345036	14.4339583	41.0872956
Eri2	4.21392933	-0.4927724	0.41436363	-1.1892269	0.2343504	NA		Eri2	6.72046328	6.78785578	6.67965972	2.06814001	3.32962344	2.35210905	5.77358333	0
Map9	121.095335	0.38845565	0.32671786	1.18896363	0.23445398	0.99019711		Map9	118.9522	113.583453	119.279638	51.7035003	79.9109626	211.689814	173.2075	100.435611
Usp19	68.3475541	0.35948743	0.30243057	1.18866103	0.23457308	0.99019711		Usp19	53.7637063	47.0624668	50.8926455	82.7256004	104.883138	70.5632714	43.301875	93.5877288
Smyd3	28.6544712	0.39084169	0.32885367	1.18849725	0.23463756	0.99019711		Smyd3	22.8495752	19.0059962	20.9932163	33.0902402	23.3073641	21.1689814	31.7547083	57.0656883
H2afz	20.6529768	0.4239018	0.3567075	1.18837366	0.23468623	0.99019711		H2afz	12.7688802	14.480759	23.2197695	16.5451201	16.6481172	14.1126543	42.3396111	25.1089028
Trmt5	4.52818734	-0.4892298	0.41172755	-1.1882366	0.23474021	NA		Trmt5	8.06455594	5.43028463	4.45310648	6.20442003	1.66481172	2.35210905	5.77358333	2.28262753
Tm6sf1	15.4994624	0.40013717	0.33688466	1.18775715	0.2349291	0.99019711		Tm6sf1	8.06455594	15.8383302	12.0870033	16.5451201	18.3129289	21.1689814	18.2830139	13.6957652
Scamp3	37.1450083	-0.4493263	0.37835083	-1.1875916	0.23499433	0.99019711		Scamp3	24.8657142	55.2078937	39.1237212	59.9760603	43.2851047	12.9365998	11.5471667	50.2178057
Las1l	21.580545	0.36281701	0.30552128	1.18753432	0.23501693	0.99019711		Las1l	15.4570656	17.1959013	21.9474534	18.6132601	26.6369875	18.8168724	28.8679167	25.1089028
Slc2a8	8.21779317	-0.468937	0.39491036	-1.1874517	0.2350495	0.99019711		Slc2a8	6.72046328	12.6706641	7.31581779	14.4769801	11.653682	3.52816357	4.81131944	4.56525506
Akap13	17.5323813	0.40570819	0.34168284	1.1873824	0.23507682	0.99019711		Akap13	16.8011582	18.1009488	11.4508452	12.4088401	19.9777406	23.5210905	28.8679167	9.13051012
Zfp1	7.79612509	0.45378947	0.38219895	1.18731219	0.2351045	0.99019711		Zfp1	6.72046328	6.78785578	7.63389683	2.06814001	8.3240586	8.23238167	13.4716944	9.13051012
6030458C11Rik	37.4661543	0.31451728	0.26506937	1.18654707	0.23540632	0.99019711		6030458C11Ri	34.2743627	35.7493738	29.2632712	31.0221002	36.6258578	44.6900719	35.6037639	52.5004332
Sv2a	396.467981	0.3742185	0.31540173	1.18648207	0.23543198	0.99019711		Sv2a	239.248493	339.845313	309.80898	450.854522	529.410127	566.858281	192.452778	543.265352
Clstn3	297.189126	0.43886943	0.3699002	1.18645361	0.23544321	0.99019711		Clstn3	150.538378	205.445768	226.790352	361.924502	479.465775	331.647376	87.5660139	534.134842
Zfp563	12.0774058	0.4637948	0.39096701	1.18627604	0.23551331	0.99019711		Zfp563	6.72046328	10.8605693	13.3593194	6.20442003	13.3184938	11.7605452	29.8301805	4.56525506
Man2b2	13.5126593	-0.4460751	0.37603587	-1.1862567	0.23552095	0.99019711		Man2b2	12.7688802	24.4362808	16.5401098	10.3407001	18.3129289	11.7605452	4.81131944	9.13051012
Btbd8	0.91361052	0.41966307	0.35382004	1.18609186	0.23558603	NA		Btbd8	0	0.90504744	0.31807903	0	1.66481172	1.17605452	0.96226389	2.28262753
Tex261	16.3614333	-0.4640882	0.39129062	-1.1860449	0.23560458	0.99019711		Tex261	13.4409266	25.3413283	15.5858727	24.8176801	4.99443516	7.05632714	7.69811111	31.9567854
Pdgfrb	6.65499378	-0.4909195	0.41394838	-1.1859438	0.23564448	0.99019711		Pdgfrb	4.7043243	20.8160911	8.2700549	2.06814001	13.3184938	1.17605452	2.88679167	0
Ryk	10.6649776	-0.4660773	0.39306272	-1.185758	0.23571788	0.99019711		Ryk	12.7688802	21.2686148	15.9039517	2.06814001	8.3240586	11.7605452	8.660375	4.56525506
Fbxo42	51.5744169	0.34025058	0.287049	1.1853397	0.23588316	0.99019711		Fbxo42	40.3227797	33.0342315	42.3045116	64.1123403	68.2572805	48.2182355	43.301875	73.044081
Zmynd19	5.82085671	0.49305143	0.41598593	1.18525988	0.23591471	0.99019711		Zmynd19	6.72046328	4.52523719	4.45310648	0	4.99443516	15.2887088	10.5849028	0
Mpc1	22.4991761	-0.3807076	0.32122457	-1.1851758	0.23594796	0.99019711		Mpc1	28.2259458	30.7716129	25.1282437	20.6814001	9.98887032	19.9929269	26.9433889	18.2610202
Cgrrf1	6.54814426	-0.4692668	0.3960744	-1.1847946	0.23609865	0.99019711		Cgrrf1	4.7043243	9.5029981	8.90621297	10.3407001	4.99443516	5.88027262	5.77358333	2.28262753
Kras	107.034563	0.36725672	0.3100022	1.18469066	0.23613978	0.99019711		Kras	82.6616984	97.2925996	96.3779475	88.9300204	64.9276571	168.175797	178.018819	79.8919636
Mical2	143.989637	0.32071078	0.27076168	1.18447624	0.2362246	0.99019711		Mical2	131.72108	139.829829	145.362119	82.7256004	236.403264	127.013889	140.490528	148.37079
Col20a1	0.63598536	-0.3728836	0.31488232	-1.1841999	0.23633394	NA		Col20a1	1.34409266	1.35757116	0.31807903	2.06814001	0	0	0	0
Gjd3	0.87216574	0.31445817	0.2656312	1.1838149	0.23648634	NA		Gjd3	0	0	0.31807903	0	6.65924688	0	0	0
Myh1	0.50785577	0.3017744	0.25493924	1.18371109	0.23652745	NA		Myh1	0	0	0	0	0	1.17605452	2.88679167	0
Tex264	29.2731007	-0.3989032	0.33716755	-1.1831007	0.23676922	0.99019711		Tex264	18.8172972	42.9897533	35.3067728	39.2946602	13.3184938	23.5210905	22.1320694	38.804668
Bcl11b	153.112984	0.30044233	0.25400426	1.18282397	0.23687892	0.99019711		Bcl11b	149.194285	128.516736	125.32314	136.497241	228.079206	132.894161	121.24525	203.15385
Radil	9.99035689	-0.4878407	0.41252193	-1.1825812	0.23697518	0.99019711		Radil	8.06455594	8.59795066	6.67965972	28.9539601	11.653682	5.88027262	0.96226389	9.13051012
Cpne9	7.58262833	-0.4728137	0.39999561	-1.1820473	0.23718693	0.99019711		Cpne9	10.0806949	6.33533207	6.04350166	16.5451201	8.3240586	5.88027262	2.88679167	4.56525506
Luc7l	55.117242	0.33110897	0.28015224	1.18188944	0.23724957	0.99019711		Luc7l	38.3066407	38.0119924	42.9406697	76.5211804	54.9387868	68.2111624	55.8113055	66.1961984
Fam3a	7.94954875	-0.4478515	0.3789369	-1.1818629	0.2372601	0.99019711		Fam3a	10.7527413	9.05047438	9.54237104	10.3407001	6.65924688	8.23238167	6.73584722	2.28262753
Dpp3	16.9322512	0.47111632	0.39862236	1.18186124	0.23726076	0.99019711		Dpp3	10.7527413	5.88280835	8.90621297	26.8858201	23.3073641	5.88027262	17.32075	36.5220405
A230070E04Rik	34.7139395	-0.3722462	0.31497033	-1.1818453	0.23726709	0.99019711		A230070E04Ri	37.6345944	32.5817078	34.6706148	55.8397803	41.620293	27.0492541	16.3584861	31.9567854
9030025P20Rik	2.93712117	0.49080492	0.41531952	1.18175259	0.23730388	NA		9030025P20Ri	2.68818531	1.81009488	2.86271131	0	1.66481172	3.52816357	8.660375	2.28262753
Clec1a	0.51515115	0.35792171	0.30296212	1.18140746	0.2374409	NA		Clec1a	0	0	0.31807903	0	1.66481172	1.17605452	0.96226389	0
Col8a2	0.51515115	0.35792171	0.30296212	1.18140746	0.2374409	NA		Col8a2	0	0	0.31807903	0	1.66481172	1.17605452	0.96226389	0
Kndc1	61.8854776	0.43203448	0.36573997	1.18126133	0.23749893	0.99019711		Kndc1	39.6507334	38.0119924	43.5768277	78.5893204	158.157113	51.7463991	44.2641389	41.0872956
Ube2z	105.7461	0.38428206	0.32532887	1.18121104	0.2375189	0.99019711		Ube2z	75.9412351	66.9735104	79.5197586	132.360961	151.497867	69.3872169	80.8301666	189.458085
Iws1	20.5966385	0.40290018	0.34110417	1.18116464	0.23753733	0.99019711		Iws1	16.8011582	9.95552182	18.766663	24.8176801	26.6369875	32.9295267	21.1698055	13.6957652
Gpr83	37.0649494	0.42428123	0.35920906	1.18115402	0.23754154	0.99019711		Gpr83	18.1452509	39.3695635	29.8994292	31.0221002	43.2851047	56.4506172	14.4339583	63.9135709
Sync	1.38312602	0.43552971	0.36879899	1.18094063	0.23762631	NA		Sync	0.67204633	0.45252372	0.9542371	0	0	1.17605452	0.96226389	6.84788259
Oxr1	314.825809	0.43038656	0.3645447	1.18061397	0.23775611	0.99019711		Oxr1	328.630655	278.302087	298.358134	103.407001	171.475607	536.280863	589.867764	212.28436
Pla2g4b	1.45745646	0.44419291	0.37637963	1.18017256	0.23793159	NA		Pla2g4b	0	0.90504744	0.31807903	2.06814001	1.66481172	1.17605452	0.96226389	4.56525506
Depdc5	56.2895152	0.35014919	0.2967106	1.18010343	0.23795908	0.99019711		Depdc5	33.6023164	46.1574193	47.0756971	68.2486203	84.9053977	70.5632714	40.4150833	59.3483158
Igtp	0.86384532	-0.41193	0.34910171	-1.1799714	0.23801159	NA		Igtp	2.01613899	1.81009488	1.90847421	0	0	1.17605452	0	0
Ftsj3	11.2100953	0.42463558	0.35993855	1.17974465	0.23810179	0.99019711		Ftsj3	10.7527413	5.43028463	10.4966081	10.3407001	14.9833055	14.1126543	14.4339583	9.13051012
Fam199x	18.6453422	0.42721167	0.36214461	1.17967146	0.23813091	0.99019711		Fam199x	7.39250961	12.2181404	18.130505	24.8176801	13.3184938	29.4013631	27.9056528	15.9783927
Cacng2	62.8420185	0.37475337	0.31771062	1.17954311	0.23818198	0.99019711		Cacng2	30.9141311	57.0179886	51.8468826	72.3849004	53.2739751	74.091435	49.0754583	114.131377
Setd2	54.6505672	0.39427212	0.33427285	1.17949189	0.23820236	0.99019711		Setd2	57.7959842	52.0402277	46.439539	24.8176801	43.2851047	107.020962	69.283	36.5220405
Neurl1b	51.7232575	0.42417293	0.35967492	1.17932306	0.23826956	0.99019711		Neurl1b	43.6830113	57.0179886	38.4875632	24.8176801	104.883138	39.9858538	25.0188611	79.8919636
Pdcd4	61.4524342	0.43975517	0.37291825	1.17922672	0.23830791	0.99019711		Pdcd4	57.7959842	52.9452751	64.888123	18.6132601	43.2851047	96.436471	127.981097	29.6741579
Slc39a9	37.1323115	-0.3567664	0.30255735	-1.1791693	0.23833075	0.99019711		Slc39a9	30.9141311	54.3028463	38.1694841	47.5672202	29.966611	44.6900719	24.0565972	27.3915304
Brms1	7.62674147	-0.4600963	0.39020179	-1.1791241	0.23834876	0.99019711		Brms1	12.0968339	11.313093	9.54237104	4.13628002	9.98887032	5.88027262	5.77358333	2.28262753

GeneID	Base mean	log2(FC)	StdErr	Wald-Stats	P-value	P-adj		GeneID	Normalised expression for Chow#1	Normalised expression for Chow#2	Normalised expression for Chow#3	Normalised expression for Chow#4	Normalised expression for HFD#1	Normalised expression for HFD#2	Normalised expression for HFD#3	Normalised expression for HFD#4
Chn1	561.69709	-0.3762197	0.31911881	-1.178933	0.23842484	0.99019711		Chn1	780.917834	790.106413	735.398728	293.675881	402.884436	445.724665	720.735653	324.133109
Yars	45.4586652	0.3333323	0.28277873	1.17877429	0.23848806	0.99019711		Yars	46.3711967	38.9170398	45.1672229	26.8858201	48.2795399	56.4506172	67.3584722	34.239413
Gnaz	78.8286565	0.32712254	0.27753214	1.17868344	0.23852425	0.99019711		Gnaz	71.9089571	72.403795	65.2062021	62.0442003	118.201632	59.9787807	64.4716805	116.414004
6430571L13Rik	3.06368786	0.4907088	0.41634791	1.17860279	0.23855638	NA		6430571L13Ri	3.36023164	1.35757116	1.90847421	2.06814001	1.66481172	7.05632714	4.81131944	2.28262753
Zfp622	22.5077448	0.45010059	0.38189561	1.17859587	0.23855914	0.99019711		Zfp622	12.0968339	8.14542694	15.2677937	37.2265202	41.620293	12.9365998	23.0943333	29.6741579
Mthfs	1.19378469	0.44702158	0.37929668	1.17855389	0.23857586	NA		Mthfs	1.34409266	0.45252372	0.63615807	0	1.66481172	3.52816357	1.92452778	0
Ier3	5.18694843	-0.4870131	0.41326718	-1.1784461	0.23861881	0.99019711		Ier3	5.37637063	7.2403795	2.86271131	12.4088401	1.66481172	3.52816357	3.84905555	4.56525506
Nkiras1	64.4984246	0.35681864	0.3028855	1.17806446	0.23877091	0.99019711		Nkiras1	61.1562159	53.3977988	62.3434908	43.4309402	38.2906696	71.739326	112.584875	73.044081
Pgf	1.91189016	-0.464961	0.3946891	-1.1780437	0.23877919	NA		Pgf	3.36023164	1.35757116	1.27231614	6.20442003	0	1.17605452	1.92452778	0
Klhl3	12.7644654	0.45075734	0.38263444	1.17803651	0.23878205	0.99019711		Klhl3	13.4409266	9.5029981	7.63389683	8.27256004	29.966611	10.5844907	6.73584722	15.9783927
Gtf2i	130.864755	0.26557964	0.22544698	1.17801375	0.23879112	0.99019711		Gtf2i	119.624246	103.627932	104.966081	144.769801	169.810795	130.542052	118.358458	155.218672
Tubb6	6.47161362	0.48433872	0.41121858	1.17781332	0.23887104	0.99019711		Tubb6	6.04841696	2.71514231	3.81694841	6.20442003	14.9833055	1.17605452	7.69811111	9.13051012
Tshz1	48.5880804	-0.3291064	0.2794471	-1.1777056	0.238914	0.99019711		Tshz1	55.1077989	57.923036	55.027673	51.7035003	31.6314227	63.5069443	32.7169722	41.0872956
Prr18	16.248858	-0.4771849	0.40523098	-1.1775628	0.23897094	0.99019711		Prr18	10.0806949	9.95552182	15.2677937	47.5672202	13.3184938	9.40843619	3.84905555	20.5436478
Ppp2r5a	29.431075	-0.3455982	0.29352694	-1.1773985	0.23903649	0.99019711		Ppp2r5a	27.5538995	38.9170398	29.5813502	37.2265202	28.3017992	18.8168724	23.0943333	31.9567854
Lyz2	1.2148762	-0.4408972	0.37451385	-1.1772521	0.23909489	NA		Lyz2	2.68818531	3.62018975	1.27231614	0	0	1.17605452	0.96226389	0
Slc33a1	16.120852	-0.4157775	0.35318613	-1.1772191	0.23910808	0.99019711		Slc33a1	14.1129729	27.1514231	21.9474534	12.4088401	8.3240586	18.8168724	12.5094306	13.6957652
Amer2	32.2799039	0.34390284	0.2921374	1.17719552	0.23911748	0.99019711		Amer2	31.5861774	26.6988994	23.2197695	28.9539601	48.2795399	28.2253086	27.9056528	43.3699231
Usp47	70.9629202	0.39038286	0.33162183	1.17719291	0.23911852	0.99019711		Usp47	61.1562159	61.9957495	66.7965972	47.5672202	46.6147282	127.013889	115.471667	41.0872956
Nmd3	14.6615947	0.45149319	0.38371296	1.17664307	0.239338	0.99068113		Nmd3	10.7527413	10.8605693	17.8124259	6.20442003	13.3184938	25.8731995	27.9056528	4.56525506
Senp5	61.5491176	0.34200313	0.29079291	1.17610544	0.23955274	0.99068113		Senp5	59.1400769	59.2806072	58.5265423	35.1583802	59.9332219	71.739326	102.962236	45.6525506
Them6	18.5248928	-0.4708265	0.40043911	-1.1757755	0.23968461	0.99068113		Them6	14.7850192	24.4362808	11.7689243	41.3628002	14.9833055	10.5844907	2.88679167	27.3915304
Prpf8	208.390108	0.29587206	0.25169934	1.17549794	0.23979556	0.99068113		Prpf8	194.221389	146.165161	188.620867	210.950281	322.973474	237.563014	170.320708	196.305968
Gpr116	7.34491675	-0.489339	0.41628462	-1.1754914	0.23979818	0.99068113		Gpr116	1.34409266	23.5312334	9.54237104	4.13628002	6.65924688	7.05632714	1.92452778	4.56525506
Pcbp3	48.2965055	-0.3283935	0.27936863	-1.1754844	0.23980096	0.99068113		Pcbp3	67.2046328	55.2078937	45.4853019	47.5672202	44.9499164	32.9295267	33.6792361	59.3483158
Musk	0.53650737	-0.3532119	0.30055983	-1.1751799	0.23992277	NA		Musk	1.34409266	1.35757116	1.59039517	0	0	0	0	0
Nfatc1	4.48737577	-0.4881556	0.41542613	-1.1750719	0.23996595	NA		Nfatc1	4.03227797	6.78785578	5.72542262	6.20442003	6.65924688	0	1.92452778	4.56525506
Iah1	4.21450065	-0.4892398	0.41636433	-1.1750281	0.23998347	NA		Iah1	3.36023164	3.62018975	4.45310648	12.4088401	0	4.7042181	2.88679167	2.28262753
Ehd4	12.2302696	-0.4416806	0.37600842	-1.174656	0.24013236	0.99068113		Ehd4	18.1452509	10.4080455	11.4508452	18.6132601	9.98887032	9.40843619	3.84905555	15.9783927
Sugp1	17.5300448	0.38211823	0.32534558	1.17449955	0.24019499	0.99068113		Sugp1	20.1613899	14.9332827	11.7689243	12.4088401	19.9777406	19.9929269	25.0188611	15.9783927
Hectd1	98.2449931	0.35221749	0.29992776	1.17434106	0.24025844	0.99068113		Hectd1	99.4628566	85.5269829	106.238398	43.4309402	101.553515	161.11947	108.735819	79.8919636
Spag4	1.65461492	0.44454603	0.37855883	1.17431161	0.24027024	NA		Spag4	0.67204633	0.45252372	1.59039517	0	4.99443516	0	0.96226389	4.56525506
Rab2b	34.7468805	0.34943118	0.29758599	1.17421921	0.24030723	0.99068113		Rab2b	25.5377605	29.4140417	32.7621406	33.0902402	38.2906696	28.2253086	58.6980972	31.9567854
Ube2o	107.584195	0.41136938	0.35043237	1.17389093	0.24043872	0.99068113		Ube2o	68.5487255	58.3755597	69.6593086	157.178641	206.436653	87.0280348	67.3584722	146.088162
Fam20b	79.6196848	0.34365268	0.29278155	1.17375118	0.2404947	0.99068113		Fam20b	46.3711967	82.3593168	81.7463119	64.1123403	89.8998329	132.894161	77.943375	61.6309433
Ppp4r1l-ps	4.14606814	-0.4879431	0.41580002	-1.1735042	0.24059367	NA		Ppp4r1l-ps	4.7043243	5.43028463	2.54463228	10.3407001	1.66481172	2.35210905	3.84905555	2.28262753
Gm684	0.54882693	-0.3478439	0.29644252	-1.1733941	0.24063779	NA		Gm684	0.67204633	1.81009488	1.90847421	0	0	0	0	0
Abhd14a	13.1275124	-0.4410271	0.37590472	-1.1732417	0.24069888	0.99068113		Abhd14a	14.1129729	16.2908539	8.58813393	24.8176801	11.653682	5.88027262	7.69811111	15.9783927
Rbm43	9.82222749	0.42515479	0.36239998	1.17316449	0.24072985	0.99068113		Rbm43	7.39250961	8.14542694	7.31581779	10.3407001	9.98887032	14.1126543	14.4339583	6.84788259
Zfp280b	24.1585566	0.33809618	0.28819818	1.17313784	0.24074054	0.99068113		Zfp280b	24.1936678	20.8160911	19.4028211	18.6132601	24.9721758	30.5774176	25.0188611	29.6741579
Fscn2	0.78137193	-0.3643825	0.31063928	-1.1730084	0.24079246	NA		Fscn2	1.34409266	0.45252372	0.31807903	4.13628002	0	0	0	0
Stxbp5	136.066552	0.25641145	0.21862114	1.17285752	0.24085295	0.99068113		Stxbp5	138.441544	108.153169	123.414665	124.088401	168.145984	168.175797	146.264111	111.848749
Fgfr1	51.3013823	-0.4083986	0.3482861	-1.1725952	0.24095817	0.99068113		Fgfr1	36.2905017	103.627932	48.0299342	53.7716403	43.2851047	63.5069443	23.0943333	38.804668
Anks1	34.3855195	0.42535661	0.3627748	1.17250873	0.24099287	0.99068113		Anks1	26.2098068	19.9110436	16.5401098	49.6353602	49.9443516	32.9295267	18.2830139	61.6309433
Ube2h	109.132567	0.27803732	0.23713212	1.1724996	0.24099653	0.99068113		Ube2h	83.3337447	87.7896015	96.3779475	124.088401	108.212762	107.020962	108.735819	157.5013
Dph7	6.86209577	-0.4751487	0.40543135	-1.1719584	0.24121375	0.99082561		Dph7	12.7688802	8.59795066	8.90621297	4.13628002	1.66481172	2.35210905	9.62263889	6.84788259
Itgb3bp	0.98949591	0.42738164	0.36471704	1.17181702	0.24127053	NA		Itgb3bp	0.67204633	0.45252372	0.63615807	0	1.66481172	3.52816357	0.96226389	0
Ndufs3	16.5698354	0.4324577	0.36906243	1.17177382	0.24128788	0.99082561		Ndufs3	13.4409266	7.69290322	14.9497146	16.5451201	11.653682	10.5844907	21.1698055	36.5220405
Ntrk2	540.389295	-0.3717804	0.31729476	-1.1717192	0.24130979	0.99082561		Ntrk2	446.238762	978.35628	589.71853	481.876622	411.208495	740.91435	286.754639	388.04668
Cyfip1	45.1637427	-0.3233486	0.27605112	-1.171336	0.24146374	0.99098405		Cyfip1	45.6991503	55.6604174	52.1649617	51.7035003	51.6091633	43.5140174	40.4150833	20.5436478
Rabggta	20.5584412	0.46295456	0.39529519	1.17116162	0.24153382	0.99098405		Rabggta	13.4409266	10.4080455	17.4943469	20.6814001	48.2795399	7.05632714	10.5849028	36.5220405
Nudt15	1.63790046	-0.4647839	0.39713336	-1.1703472	0.24186127	NA		Nudt15	4.03227797	1.35757116	2.54463228	2.06814001	0	1.17605452	1.92452778	0
Ndor1	8.75043625	-0.4371441	0.37355127	-1.1702387	0.24190494	0.9912847		Ndor1	12.0968339	9.5029981	9.54237104	12.4088401	4.99443516	8.23238167	8.660375	4.56525506
Eny2	44.3001656	0.3880383	0.33168372	1.16990456	0.24203938	0.9912847		Eny2	36.2905017	38.0119924	45.1672229	28.9539601	29.966611	49.39429	92.3773333	34.239413
Ccdc176	3.18635056	0.48684854	0.41616555	1.16984345	0.24206397	NA		Ccdc176	3.36023164	1.81009488	1.59039517	2.06814001	3.32962344	5.88027262	2.88679167	4.56525506
Zfp771	14.5970017	0.47894814	0.40948098	1.16964686	0.24214311	0.9912847		Zfp771	7.39250961	7.69290322	8.58813393	18.6132601	16.6481172	3.52816357	8.660375	45.6525506
H1f0	56.94781	0.33596062	0.28728354	1.16943916	0.24222674	0.9912847		H1f0	55.7798453	40.274611	51.5288036	49.6353602	36.6258578	56.4506172	80.8301666	84.4572186
Ttbk1	116.3704	0.42557071	0.36398117	1.16921079	0.24231872	0.9912847		Ttbk1	70.5648645	83.716888	63.9338859	157.178641	238.068076	71.739326	65.4339444	180.327575
Gorasp1	29.0203258	0.37451026	0.32047048	1.16862642	0.24255418	0.9912847		Gorasp1	26.8818531	23.5312334	21.9474534	24.8176801	53.2739751	17.6408179	29.8301805	34.239413
Hmgn1	19.2476503	-0.4129015	0.35333748	-1.1685756	0.24257467	0.9912847		Hmgn1	22.1775288	18.1009488	23.5378486	28.9539601	4.99443516	12.9365998	25.0188611	18.2610202
Ccdc93	13.5411214	0.43607399	0.37317442	1.16855273	0.24258388	0.9912847		Ccdc93	14.1129729	8.59795066	13.6773985	6.20442003	26.6369875	15.2887088	19.2452778	4.56525506
Dck	19.940588	-0.410098	0.35105622	-1.1681832	0.24273289	0.9912847		Dck	20.1613899	23.0787097	20.6751372	33.0902402	11.653682	10.5844907	28.8679167	11.4131377
Rpsa	49.5953655	-0.3958631	0.33888446	-1.1681358	0.24275198	0.9912847		Rpsa	75.9412351	38.4645161	41.6683535	76.5211804	61.5980337	19.9929269	34.6415	47.9351782
Slc19a1	6.57875878	-0.4689592	0.40155644	-1.1678537	0.24286577	0.9912847		Slc19a1	5.37637063	7.69290322	7.63389683	12.4088401	8.3240586	4.7042181	1.92452778	4.56525506
Lhcgr	0.55779899	-0.3478634	0.29800051	-1.1673248	0.24307925	NA		Lhcgr	2.01613899	1.81009488	0.63615807	0	0	0	0	0
Strap	87.220064	0.32172336	0.27562811	1.1672371	0.24311463	0.9912847		Strap	69.2207718	60.6381783	84.9271022	93.0663005	71.586904	88.2040893	143.377319	86.7398462
Rgs5	7.9031065	-0.4793508	0.41075373	-1.1670029	0.2432092	0.9912847		Rgs5	8.06455594	16.7433776	13.0412404	2.06814001	11.653682	5.88027262	5.77358333	0
Spp1	3.86047267	-0.4729551	0.40532872	-1.1668433	0.24327367	NA		Spp1	2.68818531	16.2908539	2.86271131	0	1.66481172	3.52816357	3.84905555	0
Letmd1	29.1515919	-0.3664749	0.31412453	-1.1666547	0.24334983	0.9912847		Letmd1	39.6507334	40.274611	29.8994292	22.7495401	38.2906696	23.5210905	18.2830139	20.5436478
Casc3	53.5656827	0.33622894	0.28821446	1.16659288	0.24337482	0.9912847		Casc3	39.6507334	44.7998482	48.3480132	51.7035003	66.5924688	69.3872169	32.7169722	75.3267085
Afmid	3.25609463	0.48373634	0.414868	1.16600059	0.24361421	NA		Afmid	1.34409266	1.35757116	0.9542371	6.20442003	4.99443516	4.7042181	1.92452778	4.56525506
Mar-04	46.3325117	0.30804475	0.26442278	1.16497057	0.24403091	0.9912847		Mar-04	39.6507334	34.8443264	36.897168	53.7716403	58.2684102	55.2745626	39.4528194	52.5004332
Ppp1r12c	56.1794821	0.42863808	0.36803859	1.16465526	0.24415857	0.9912847		Ppp1r12c	49.059382	28.961518	24.4920857	78.5893204	76.5813391	45.8661264	31.7547083	114.131377
Nanp	5.35629427	0.48470689	0.4161968	1.16460983	0.24417696	0.9912847		Nanp	3.36023164	2.71514231	6.67965972	2.06814001	3.32962344	14.1126543	10.5849028	0
Ide	27.6769698	-0.4043374	0.3472532	-1.1643878	0.24426689	0.9912847		Ide	42.3389187	37.5594687	32.7621406	18.6132601	16.6481172	36.4576902	27.9056528	9.13051012
1700048O20Rik	6.17261385	0.47592703	0.40884212	1.16408511	0.24438952	0.9912847		1700048O20R	2.68818531	3.62018975	4.13502745	8.27256004	4.99443516	2.35210905	9.62263889	13.6957652
Fzd8	3.54040723	0.48015803	0.41255986	1.1638506	0.24448456	NA		Fzd8	0.67204633	2.71514231	2.86271131	2.06814001	4.99443516	5.88027262	0	9.13051012
Dido1	61.7749169	0.3420903	0.29393533	1.16382843	0.24449355	0.9912847		Dido1	47.043243	57.923036	46.439539	62.0442003	74.9165274	95.2604165	37.5282917	73.044081
Mga	60.2313254	0.41682542	0.35816609	1.1637769	0.24451444	0.9912847		Mga	55.1077989	59.7331309	57.2542262	22.7495401	59.9332219	121.133616	80.8301666	25.1089028
Trim62	39.403067	0.31336916	0.26933339	1.16349909	0.24462707	0.9912847		Trim62	34.2743627	25.793852	35.9429309	43.4309402	44.9499164	41.1619083	39.4528194	50.2178057
Gtf2a1	39.8125858	0.30823343	0.2651058	1.1626808	0.24495903	0.9912847		Gtf2a1	34.9464091	38.0119924	33.3982986	35.1583802	33.2962344	52.9224536	51.96225	38.804668
Rnf7	28.9786972	-0.4411123	0.37940499	-1.1626423	0.24497465	0.9912847		Rnf7	38.3066407	44.7998482	34.9886938	22.7495401	3.32962344	21.1689814	41.3773472	25.1089028
ChkbCpt1b	7.58091379	0.44276649	0.38084613	1.16258628	0.2449974	0.9912847		ChkbCpt1b	4.03227797	5.88280835	6.04350166	8.27256004	8.3240586	7.05632714	9.62263889	11.4131377
Cpne8	9.36630809	0.44083055	0.37927068	1.16231117	0.24510909	0.9912847		Cpne8	8.06455594	5.43028463	9.54237104	6.20442003	18.3129289	7.05632714	13.4716944	6.84788259
Mpp6	37.0586869	0.44879952	0.386152	1.1622354	0.24513986	0.9912847		Mpp6	33.6023164	25.793852	39.4418003	16.5451201	18.3129289	92.9083074	53.8867778	15.9783927
Trmt61a	15.9834238	0.45267257	0.38958577	1.16193301	0.24526268	0.9912847		Trmt61a	9.4086486	7.69290322	7.31581779	26.8858201	24.9721758	9.40843619	12.5094306	29.6741579
2010107G23Rik	19.2773466	0.44468324	0.38278491	1.16170524	0.24535522	0.9912847		2010107G23R	16.8011582	8.14542694	11.1327662	24.8176801	19.9777406	9.40843619	18.2830139	45.6525506
Psph	6.15781069	-0.4769286	0.41059778	-1.1615471	0.2454195	0.9912847		Psph	11.4247876	4.97776091	5.40734359	10.3407001	0	3.52816357	6.73584722	6.84788259
Rad51	0.73624551	0.39115645	0.33680971	1.16135741	0.24549658	NA		Rad51	0.67204633	0.45252372	0	0	1.66481172	1.17605452	1.92452778	0
4930483K19Rik	0.941094	0.37541353	0.32326487	1.16131867	0.24551233	NA		4930483K19Ri	0	0	0.9542371	0	3.32962344	0	0.96226389	2.28262753
Elac1	45.026086	0.39985767	0.34432935	1.16126516	0.24553408	0.9912847		Elac1	28.2259458	21.7211385	37.8514051	64.1123403	26.6369875	48.2182355	74.0943194	59.3483158
Ptpru	25.9439708	0.39517678	0.34031992	1.16119201	0.24556382	0.9912847		Ptpru	18.1452509	21.7211385	18.130505	28.9539601	54.9387868	25.8731995	19.2452778	20.5436478
Fbxo41	119.17004	0.42155734	0.36304793	1.16116165	0.24557617	0.9912847		Fbxo41	81.3176057	70.5937002	80.1559167	153.042361	239.732888	89.3801438	51.96225	187.175458
Bok	14.3283895	-0.4579992	0.39449177	-1.1609854	0.24564782	0.9912847		Bok	12.7688802	12.6706641	11.4508452	35.1583802	14.9833055	2.35210905	11.5471667	13.6957652
Ip6k1	228.713855	0.42472294	0.36587042	1.1608562	0.24570038	0.9912847		Ip6k1	132.393127	123.086452	145.680198	335.038682	362.928955	145.830761	132.792417	451.960251
Npc1	31.4792001	-0.4478666	0.38597202	-1.1603602	0.24590218	0.9912847		Npc1	30.9141311	67.4260341	37.8514051	16.5451201	33.2962344	41.1619083	8.660375	15.9783927
Rsg1	0.9461663	-0.3904216	0.33662585	-1.1598086	0.24612674	NA		Rsg1	4.03227797	0.45252372	1.90847421	0	0	1.17605452	0	0
Bcl6	25.2631989	-0.3624309	0.31261333	-1.1593584	0.24631012	0.9912847		Bcl6	26.2098068	31.2241366	28.9451921	31.0221002	31.6314227	24.697145	19.2452778	9.13051012
Kdm5b	56.563822	0.28167088	0.24300783	1.15910206	0.24641459	0.9912847		Kdm5b	47.7152893	52.9452751	48.9841713	53.7716403	56.6035985	79.9717076	57.7358333	54.7830607
Rbm20	4.4762169	0.4749936	0.40984013	1.15897289	0.24646724	NA		Rbm20	4.7043243	1.81009488	4.13502745	2.06814001	6.65924688	7.05632714	4.81131944	4.56525506
Fgfrl1	5.58662087	-0.4822741	0.41613405	-1.1589394	0.24648089	0.9912847		Fgfrl1	8.73660227	10.8605693	6.67965972	2.06814001	8.3240586	1.17605452	0	6.84788259
Kank2	4.17228396	-0.4811234	0.41521967	-1.1587201	0.24657028	NA		Kank2	6.72046328	3.62018975	5.08926455	6.20442003	3.32962344	0	3.84905555	4.56525506
Bmpr1a	39.8500055	-0.458714	0.39594948	-1.1585164	0.24665334	0.9912847		Bmpr1a	40.3227797	79.6441745	59.1627004	18.6132601	24.9721758	58.8027262	32.7169722	4.56525506
Dcst1	2.53758866	0.4313461	0.37234561	1.15845625	0.24667789	NA		Dcst1	2.01613899	0	0.9542371	2.06814001	0	0	3.84905555	11.4131377
Dnajb1	48.7602517	0.30975649	0.26740147	1.15839484	0.24670294	0.9912847		Dnajb1	45.6991503	32.5817078	40.7141164	53.7716403	61.5980337	41.1619083	52.9245139	61.6309433
Vps11	46.9676888	0.36289609	0.31328836	1.15834526	0.24672317	0.9912847		Vps11	45.027104	25.3413283	39.7598793	49.6353602	76.5813391	34.1055812	41.3773472	63.9135709
Cpox	25.6030122	0.37854135	0.32685285	1.15813996	0.24680692	0.9912847		Cpox	19.4893435	14.0282353	23.5378486	28.9539601	34.9610461	23.5210905	19.2452778	41.0872956
Sult4a1	360.293675	0.38396008	0.33153801	1.15811781	0.24681596	0.9912847		Sult4a1	290.99606	182.367059	234.424248	498.421742	472.806529	239.915123	324.28293	639.135709
Igsf21	25.8024168	-0.4032255	0.3481995	-1.15803	0.24685181	0.9912847		Igsf21	25.5377605	41.1796584	29.5813502	22.7495401	34.9610461	10.5844907	14.4339583	27.3915304
Ulk4	2.15583921	-0.4730815	0.40861093	-1.1577797	0.24695394	NA		Ulk4	3.36023164	2.71514231	2.54463228	4.13628002	0	3.52816357	0.96226389	0
Actr5	2.85311534	0.47245999	0.40819149	1.15744694	0.24708981	NA		Actr5	2.68818531	0.45252372	2.86271131	0	6.65924688	2.35210905	0.96226389	6.84788259
Gm10432	1.47826146	0.4429407	0.38268816	1.15744554	0.24709038	NA		Gm10432	0	0.90504744	1.59039517	0	1.66481172	1.17605452	1.92452778	4.56525506

GeneID	Base mean	log2(FC)	StdErr	Wald-Stats	P-value	P-adj		GeneID	Normalised expression for Chow#1	Normalised expression for Chow#2	Normalised expression for Chow#3	Normalised expression for Chow#4	Normalised expression for HFD#1	Normalised expression for HFD#2	Normalised expression for HFD#3	Normalised expression for HFD#4
Sap30	2.94050331	0.48111962	0.41572532	1.15730171	0.24714911	NA		Sap30	0.67204633	1.81009488	1.90847421	4.13628002	1.66481172	5.88027262	2.88679167	4.56525506
Wbp5	48.285353	0.37374086	0.32302226	1.15701271	0.24726717	0.9912847		Wbp5	39.6507334	28.0564706	41.0321955	55.8397803	33.2962344	36.4576902	78.9056389	73.044081
Zfp704	73.5139903	0.32398617	0.28002753	1.15697973	0.24728064	0.9912847		Zfp704	60.4841696	66.068463	69.0231505	62.0442003	101.553515	107.020962	80.8301666	41.0872956
Gm3414	1.74585134	0.44541704	0.38503077	1.15683492	0.24733981	NA		Gm3414	0	2.26261859	1.27231614	0	1.66481172	5.88027262	2.88679167	0
Pou3f1	9.39986056	-0.4464817	0.38595345	-1.1568278	0.24734272	0.9912847		Pou3f1	10.0806949	9.5029981	9.54237104	16.5451201	9.98887032	2.35210905	5.77358333	11.4131377
Cul9	52.7965754	0.46936723	0.40605629	1.15591666	0.24771525	0.9912847		Cul9	29.5700384	25.793852	23.2197695	78.5893204	126.525691	27.0492541	13.4716944	98.1529838
Errfi1	25.3247583	-0.3492373	0.30220977	-1.1556123	0.24783979	0.9912847		Errfi1	30.9141311	27.1514231	26.0824808	33.0902402	29.966611	17.6408179	24.0565972	13.6957652
Tbccd1	12.1948443	-0.4182273	0.36196037	-1.1554505	0.24790597	0.9912847		Tbccd1	20.8334362	9.95552182	14.9497146	12.4088401	13.3184938	10.5844907	8.660375	6.84788259
Bhlhe41	48.1317251	-0.3374429	0.29212137	-1.1551461	0.24803061	0.9912847		Bhlhe41	51.7475673	41.6321821	44.5310648	82.7256004	33.2962344	38.8097993	37.5282917	54.7830607
Oxtr	12.7713245	0.42972464	0.37208782	1.15490112	0.24813092	0.9912847		Oxtr	4.7043243	12.6706641	11.7689243	12.4088401	13.3184938	24.697145	13.4716944	9.13051012
Ncan	147.509288	-0.4093276	0.35448678	-1.1547048	0.24821132	0.9912847		Ncan	174.732045	267.441518	171.4446	82.7256004	224.749582	105.844907	66.3962083	86.7398462
Trmt112	13.7551283	-0.4525333	0.39190611	-1.1546982	0.24821403	0.9912847		Trmt112	15.4570656	13.5757116	13.9954775	24.8176801	8.3240586	1.17605452	14.4339583	18.2610202
Cldn3	0.77875835	0.30232818	0.26184245	1.15461865	0.24824663	NA		Cldn3	0	0	0	0	1.66481172	0	0	4.56525506
Eppk1	0.77875835	0.30232818	0.26184245	1.15461865	0.24824663	NA		Eppk1	0	0	0	0	1.66481172	0	0	4.56525506
H2-Bl	0.77875835	0.30232818	0.26184245	1.15461865	0.24824663	NA		H2-Bl	0	0	0	0	1.66481172	0	0	4.56525506
Mir1906-1	0.77875835	0.30232818	0.26184245	1.15461865	0.24824663	NA		Mir1906-1	0	0	0	0	1.66481172	0	0	4.56525506
Mir1906-2	0.77875835	0.30232818	0.26184245	1.15461865	0.24824663	NA		Mir1906-2	0	0	0	0	1.66481172	0	0	4.56525506
Slc35g2	15.0231691	0.43445645	0.37628781	1.15458549	0.24826021	0.9912847		Slc35g2	7.39250961	16.2908539	13.6773985	10.3407001	8.3240586	34.1055812	16.3584861	13.6957652
Cdk5r1	313.774129	0.33320643	0.28861367	1.15450674	0.24829248	0.9912847		Cdk5r1	277.555134	257.033472	237.28696	314.357282	451.163976	223.45036	235.754653	513.591194
Exoc1	17.6045084	-0.4147618	0.35931293	-1.1543192	0.24836932	0.9912847		Exoc1	27.5538995	20.8160911	23.5378486	12.4088401	18.3129289	17.6408179	18.2830139	2.28262753
Kcnj5	4.99076734	0.47844091	0.41453759	1.15415566	0.24843636	0.9912847		Kcnj5	0	2.71514231	2.86271131	8.27256004	3.32962344	9.40843619	1.92452778	11.4131377
Dusp23	5.42960578	-0.4803506	0.41632384	-1.1537907	0.24858597	0.9912847		Dusp23	4.03227797	4.07271347	3.18079035	18.6132601	1.66481172	1.17605452	3.84905555	6.84788259
Ehd3	101.764958	0.23474407	0.2034796	1.15364916	0.24864403	0.9912847		Ehd3	94.7585323	101.365313	89.3802087	84.7937404	116.53682	99.9646345	115.471667	111.848749
Pcnxl2	57.9730593	0.34593729	0.29986662	1.15363721	0.24864894	0.9912847		Pcnxl2	45.6991503	67.4260341	49.6203294	33.0902402	76.5813391	75.2674895	38.4905555	77.6093361
Megf8	175.141979	0.46880328	0.40643376	1.15345554	0.24872346	0.9912847		Megf8	51.7475673	111.773359	76.3389683	279.198901	399.554813	150.534979	37.5282917	294.458952
Lin52	18.3006024	-0.3693625	0.32024031	-1.1533916	0.24874968	0.9912847		Lin52	25.5377605	21.7211385	22.2655324	14.4769801	13.3184938	19.9929269	15.3962222	13.6957652
Plekhg5	33.4939871	0.44890347	0.38924878	1.15325594	0.24880535	0.9912847		Plekhg5	26.8818531	15.8383302	20.3570582	41.3628002	86.5702095	15.2887088	18.2830139	43.3699231
Dbpht2	59.8057394	0.39092745	0.33915256	1.15265958	0.24905014	0.9912847		Dbpht2	45.6991503	58.8280835	66.4785182	26.8858201	39.9554813	75.2674895	117.396194	47.9351782
Rasgrp1	331.887066	-0.4360293	0.37832034	-1.15254	0.24909924	0.9912847		Rasgrp1	518.147719	504.111423	513.061483	70.3167603	223.084771	317.534722	344.490472	164.349182
Leng9	4.1497253	0.47548099	0.4125701	1.15248536	0.24912168	NA		Leng9	2.01613899	3.16766603	2.86271131	4.13628002	8.3240586	2.35210905	5.77358333	4.56525506
Zbtb17	13.8064135	0.42490625	0.36871987	1.1523823	0.24916401	0.9912847		Zbtb17	17.4732045	6.78785578	8.90621297	10.3407001	18.3129289	12.9365998	10.5849028	25.1089028
Riok1	10.0856778	0.42074786	0.36520387	1.15209037	0.24928394	0.9912847		Riok1	6.04841696	8.14542694	7.95197586	10.3407001	16.6481172	8.23238167	9.62263889	13.6957652
Serinc3	110.916475	-0.4326412	0.37555819	-1.1519952	0.24932305	0.9912847		Serinc3	90.054208	233.502239	174.62539	35.1583802	86.5702095	131.718107	55.8113055	79.8919636
Nt5dc2	5.36467487	0.47172676	0.40951034	1.15192883	0.24935032	0.9912847		Nt5dc2	4.03227797	2.71514231	5.40734359	2.06814001	6.65924688	3.52816357	4.81131944	13.6957652
Zfp180	28.4188501	0.33864736	0.293995	1.15188139	0.24936982	0.9912847		Zfp180	28.8979921	22.6261859	27.354797	16.5451201	29.966611	25.8731995	32.7169722	43.3699231
Zfp707	6.43501633	0.46826003	0.40652791	1.15185209	0.24938186	0.9912847		Zfp707	8.06455594	5.43028463	4.13502745	0	8.3240586	3.52816357	10.5849028	11.4131377
Dicer1	33.3093862	0.32170796	0.2793279	1.15172152	0.24943553	0.9912847		Dicer1	32.9302701	29.4140417	28.9451921	24.8176801	48.2795399	42.3379629	34.6415	25.1089028
Rmi1	17.3097917	0.36371417	0.31580691	1.15169795	0.24944522	0.9912847		Rmi1	14.1129729	16.2908539	14.6316356	12.4088401	21.6425524	21.1689814	15.3962222	22.8262753
Crip2	71.6779101	-0.4422741	0.38402877	-1.1516692	0.24945703	0.9912847		Crip2	74.5971425	64.7108918	48.6660923	161.314921	91.5646446	21.1689814	26.9433889	84.4572186
Pnisr	20.753009	0.44920869	0.39008933	1.15155339	0.24950464	0.9912847		Pnisr	26.2098068	16.2908539	18.766663	2.06814001	16.6481172	39.9858538	34.6415	11.4131377
Zfp870	6.56128725	0.46674659	0.40543383	1.15122754	0.24963864	0.9912847		Zfp870	8.06455594	4.07271347	4.13502745	4.13628002	4.99443516	9.40843619	15.3962222	2.28262753
Itpk1	32.2932307	-0.3411546	0.2963428	-1.1512159	0.24964341	0.9912847		Itpk1	35.6184554	34.8443264	31.8079035	43.4309402	29.966611	22.345036	19.2452778	41.0872956
Map3k2	27.4882811	0.37503454	0.32579082	1.15115137	0.24966997	0.9912847		Map3k2	28.8979921	28.0564706	20.9932163	14.4769801	19.9777406	42.3379629	42.3396111	22.8262753
Fry	186.780189	0.32891005	0.28574601	1.15105739	0.24970863	0.9912847		Fry	158.602934	181.462011	160.947991	146.837941	321.308662	237.563014	123.169778	164.349182
Drg1	39.221275	0.32896108	0.28592391	1.15051965	0.2499299	0.9912847		Drg1	36.2905017	31.6766603	38.4875632	31.0221002	34.9610461	45.8661264	63.5094166	31.9567854
Sema3d	3.56155513	-0.4770003	0.41460981	-1.15048	0.24994622	NA		Sema3d	4.03227797	8.14542694	6.99773876	0	3.32962344	1.17605452	4.81131944	0
Clip2	128.116747	0.42540314	0.3697775	1.15043002	0.2499668	0.9912847		Clip2	79.3014668	77.8340797	76.3389683	177.860041	236.403264	94.0843619	54.8490416	228.262753
Naga	7.60213933	-0.448567	0.38993281	-1.1503699	0.24999155	0.9912847		Naga	6.72046328	10.8605693	7.31581779	12.4088401	8.3240586	3.52816357	4.81131944	6.84788259
Fam89b	15.225492	-0.4213019	0.36631843	-1.1500974	0.25010374	0.9912847		Fam89b	16.8011582	17.1959013	14.6316356	22.7495401	14.9833055	5.88027262	6.73584722	22.8262753
Lrsam1	21.5087841	0.36664226	0.31883259	1.14995226	0.25016353	0.9912847		Lrsam1	24.8657142	16.2908539	14.3135566	16.5451201	29.966611	21.1689814	19.2452778	29.6741579
Rptor	72.303046	0.28289581	0.24602326	1.14987422	0.25019568	0.9912847		Rptor	58.4680306	70.1411764	54.7095939	74.4530404	91.5646446	65.8590533	67.3584722	95.8703563
Rps15a-ps6	3.0544941	-0.4661108	0.40536893	-1.1498433	0.25020841	NA		Rps15a-ps6	5.37637063	5.43028463	6.67965972	0	0	1.17605452	5.77358333	0
Kcnn3	22.9915442	-0.3986994	0.34677653	-1.14973	0.2502551	0.9912847		Kcnn3	20.8334362	30.3190892	28.9451921	26.8858201	24.9721758	24.697145	6.73584722	20.5436478
Rpl36	3.47445358	0.47643343	0.41444249	1.14957669	0.25031827	NA		Rpl36	4.03227797	1.35757116	1.27231614	2.06814001	1.66481172	1.17605452	4.81131944	11.4131377
Zdbf2	67.0870151	0.40717214	0.35426152	1.14935471	0.25040975	0.9912847		Zdbf2	38.3066407	51.587704	61.3892537	70.3167603	43.2851047	157.591306	73.1320555	41.0872956
Mfap3l	36.6939518	-0.4784674	0.41632752	-1.1492573	0.25044989	0.9912847		Mfap3l	51.7475673	83.716888	59.1627004	0	8.3240586	45.8661264	35.6037639	9.13051012
Stk10	3.29021509	0.47847239	0.41636923	1.14915406	0.25049246	NA		Stk10	4.03227797	2.71514231	1.59039517	0	8.3240586	3.52816357	3.84905555	2.28262753
Fam13b	84.9358376	0.36115573	0.31430877	1.14904756	0.25053637	0.9912847		Fam13b	90.054208	73.3088425	77.9293635	47.5672202	66.5924688	150.534979	116.433931	57.0656883
Rap1gap2	169.507259	0.36649305	0.31897691	1.14896421	0.25057074	0.9912847		Rap1gap2	123.656524	120.371309	124.050823	206.814001	307.990168	125.837834	114.509403	232.828008
Mrps11	10.8811858	0.43636197	0.37979246	1.14894847	0.25057723	0.9912847		Mrps11	10.7527413	7.2403795	8.90621297	6.20442003	4.99443516	9.40843619	14.4339583	25.1089028
Tfdp1	15.2884429	0.38432601	0.33452918	1.14885645	0.25061518	0.9912847		Tfdp1	14.1129729	9.95552182	14.9497146	12.4088401	19.9777406	14.1126543	23.0943333	13.6957652
Apc2	107.734434	0.42949855	0.37390625	1.14867978	0.25068805	0.9912847		Apc2	68.5487255	80.549222	56.6180681	138.565381	251.38657	103.492798	39.4528194	123.261887
Adamts5	5.15963078	0.47619113	0.41462439	1.14848798	0.25076717	0.9912847		Adamts5	2.68818531	2.71514231	5.40734359	4.13628002	1.66481172	15.2887088	4.81131944	4.56525506
Pcdhb10	3.222318	-0.4761855	0.41471602	-1.1482207	0.25087747	NA		Pcdhb10	2.01613899	4.97776091	3.18079035	8.27256004	1.66481172	4.7042181	0.96226389	0
Aldoart2	61.6005878	0.41162493	0.35859718	1.14787552	0.25101995	0.9912847		Aldoart2	47.7152893	46.6099431	42.9406697	62.0442003	106.54795	29.4013631	36.5660278	120.979259
Med30	12.6393705	-0.4422517	0.38556549	-1.1470209	0.25137298	0.9912847		Med30	15.4570656	11.313093	16.2220308	20.6814001	3.32962344	7.05632714	20.2075417	6.84788259
Kif26b	12.6194927	0.41958836	0.3658208	1.14697785	0.25139077	0.9912847		Kif26b	7.39250961	8.14542694	9.86045007	16.5451201	19.9777406	17.6408179	7.69811111	13.6957652
Egfl7	6.2853217	0.47644452	0.41549465	1.14669231	0.25150881	0.9912847		Egfl7	4.7043243	7.2403795	4.13502745	0	19.9777406	3.52816357	3.84905555	6.84788259
Znhit1	37.5743731	-0.3505543	0.30571562	-1.1466678	0.25151895	0.9912847		Znhit1	45.6991503	38.4645161	29.2632712	59.9760603	23.3073641	25.8731995	34.6415	43.3699231
Golm1	8.37873323	0.46301936	0.40382909	1.14657258	0.25155831	0.9912847		Golm1	5.37637063	9.05047438	6.67965972	4.13628002	3.32962344	22.345036	11.5471667	4.56525506
Cdkn1a	10.875744	-0.4530183	0.39511825	-1.1465385	0.25157239	0.9912847		Cdkn1a	13.4409266	16.7433776	7.63389683	14.4769801	8.3240586	2.35210905	5.77358333	18.2610202
2210016F16Rik	8.11904438	-0.4461181	0.38914496	-1.146406	0.25162722	0.9912847		2210016F16Ri	8.06455594	9.05047438	8.58813393	14.4769801	11.653682	4.7042181	3.84905555	4.56525506
Insc	0.93725953	-0.4031617	0.35169193	-1.1463491	0.25165074	NA		Insc	1.34409266	2.26261859	2.22655324	0	1.66481172	0	0	0
Fkbp15	23.1625286	0.35174348	0.30686856	1.14623501	0.25169793	0.9912847		Fkbp15	20.1613899	17.648425	22.2655324	20.6814001	28.3017992	31.7534722	30.7924444	13.6957652
Syt3	29.5591611	0.38163684	0.3329577	1.14620216	0.25171151	0.9912847		Syt3	23.5216215	28.5089943	23.5378486	20.6814001	38.2906696	31.7534722	15.3962222	54.7830607
Raver1	39.8394841	-0.3678868	0.32096905	-1.1461754	0.25172258	0.9912847		Raver1	53.0916599	38.4645161	27.0367179	66.1804803	38.2906696	28.2253086	24.0565972	43.3699231
Eif4b	121.301813	0.26120536	0.22791953	1.14604201	0.25177777	0.9912847		Eif4b	105.511274	85.0744592	110.691504	138.565381	143.173808	115.253343	126.056569	146.088162
Fosl2	33.846151	0.33624355	0.293415	1.14596577	0.25180931	0.9912847		Fosl2	30.2420848	24.8888045	34.3525357	24.8176801	44.9499164	28.2253086	30.7924444	52.5004332
Uba52	25.7682151	-0.4294544	0.3748609	-1.1456368	0.25194548	0.99145555		Uba52	35.6184554	34.3918026	37.8514051	16.5451201	4.99443516	19.9929269	38.4905555	18.2610202
Inhbb	3.97033351	0.47670984	0.41622818	1.14530888	0.25208123	NA		Inhbb	2.68818531	4.97776091	2.22655324	0	8.3240586	7.05632714	1.92452778	4.56525506
5430416N02Rik	0.91188247	0.36591996	0.3195304	1.14518042	0.25213443	NA		5430416N02R	0.67204633	0.45252372	0	0	4.99443516	1.17605452	0	0
Cxcr4	1.50570179	0.44598757	0.38945496	1.14515826	0.25214361	NA		Cxcr4	0.67204633	0.45252372	1.59039517	0	1.66481172	1.17605452	1.92452778	4.56525506
Gnas	1195.70031	-0.2255677	0.19700269	-1.1449981	0.25220995	0.99213099		Gnas	1438.85119	1315.03393	1320.98223	1104.38677	870.69653	1163.11792	1286.54682	1065.98706
Creb1	45.665883	0.38678372	0.33792635	1.14457996	0.2523832	0.99244725		Creb1	39.6507334	47.9675142	44.8491439	18.6132601	29.966611	82.3238167	65.4339444	36.5220405
Tbc1d4	9.62821764	0.4444018	0.3884235	1.14411666	0.25257526	0.9928372		Tbc1d4	4.03227797	9.5029981	11.4508452	4.13628002	11.653682	18.8168724	10.5849028	6.84788259
Igfbp5	96.7993677	-0.3692894	0.32294775	-1.1434959	0.25283277	0.99345555		Igfbp5	90.7262543	184.629677	100.512975	70.3167603	98.2238915	112.901234	57.7358333	59.3483158
B330016D10Rik	1.50054642	-0.4198087	0.36721394	-1.1432264	0.25294462	NA		B330016D10R	2.01613899	0.90504744	0.9542371	6.20442003	0	0	1.92452778	0
Clec14a	0.99999584	-0.3920403	0.34297659	-1.1430527	0.25301671	NA		Clec14a	0.67204633	4.07271347	1.59039517	0	1.66481172	0	0	0
Cyb5d2	9.10692289	-0.4585893	0.40126339	-1.1428635	0.25309525	0.99345555		Cyb5d2	12.0968339	17.648425	11.4508452	4.13628002	9.98887032	11.7605452	5.77358333	0
Mmgt2	8.89670711	-0.4483615	0.3923152	-1.1428604	0.25309654	0.99345555		Mmgt2	5.37637063	10.8605693	9.224292	18.6132601	4.99443516	5.88027262	4.81131944	11.4131377
Hyls1	4.94738802	0.47580676	0.41637078	1.14274771	0.25314335	0.99345555		Hyls1	4.7043243	5.88280835	1.27231614	2.06814001	1.66481172	8.23238167	13.4716944	2.28262753
Klhl15	19.1508659	0.41138152	0.36003423	1.14261779	0.25319732	0.99345555		Klhl15	10.7527413	11.313093	17.1762679	22.7495401	19.9777406	17.6408179	12.5094306	41.0872956
Plag1	1.90281958	0.46609254	0.40802696	1.14230821	0.25332593	NA		Plag1	1.34409266	0.90504744	1.90847421	0	3.32962344	3.52816357	1.92452778	2.28262753
Vprbp	41.8509988	0.3485915	0.30518568	1.14222758	0.25335943	0.99362749		Vprbp	28.8979921	29.4140417	34.0344567	51.7035003	46.6147282	36.4576902	34.6415	73.044081
Med10	6.82221741	-0.4675944	0.40951726	-1.1418185	0.25352947	0.99362749		Med10	11.4247876	4.52523719	6.99773876	12.4088401	9.98887032	1.17605452	5.77358333	2.28262753
Copz1	73.063313	-0.2855877	0.25013919	-1.1417152	0.25357244	0.99362749		Copz1	86.6939764	72.8563187	77.9293635	86.8618804	83.240586	44.6900719	68.3207361	63.9135709
Clip4	67.9327915	0.2756665	0.24148202	1.14156121	0.25363646	0.99362749		Clip4	71.2369108	48.8725616	67.4327553	55.8397803	66.5924688	79.9717076	82.7546944	70.7614535
Rogdi	93.0974561	-0.3816144	0.33434065	-1.1413941	0.25370597	0.99362749		Rogdi	112.231737	85.9795066	85.8813393	148.906081	104.883138	30.5774176	64.4716805	111.848749
Bsx	1.55110501	-0.2721928	0.23850064	-1.1412667	0.25375897	NA		Bsx	0	0	0	12.4088401	0	0	0	0
Hcrt	1.55110501	-0.2721928	0.23850064	-1.1412667	0.25375897	NA		Hcrt	0	0	0	12.4088401	0	0	0	0
Sirpa	232.350718	-0.4034229	0.35359452	-1.1409196	0.25390338	0.9940364		Sirpa	219.087103	475.602429	299.630451	101.338861	169.810795	292.837576	127.018833	173.479692
Pim1	2.73294089	0.46642494	0.40899579	1.14041501	0.25411344	NA		Pim1	2.01613899	1.35757116	2.22655324	0	4.99443516	1.17605452	0.96226389	9.13051012
Fgf10	9.39130604	0.45446134	0.39853997	1.14031559	0.25415485	0.99465653		Fgf10	4.03227797	4.97776091	5.08926455	16.5451201	6.65924688	7.05632714	12.5094306	18.2610202
Fbxl7	2.54590061	0.47049884	0.41270725	1.14003046	0.25427361	NA		Fbxl7	2.68818531	0.90504744	0.9542371	2.06814001	1.66481172	2.35210905	2.88679167	6.84788259
Tmem72	0.56442124	0.32888869	0.28852183	1.13990921	0.25432413	NA		Tmem72	0	0.45252372	0	0	0	1.17605452	2.88679167	0
Mpp4	2.76078907	0.4684481	0.41097534	1.1398448	0.25435097	NA		Mpp4	0	2.26261859	2.54463228	2.06814001	1.66481172	7.05632714	1.92452778	4.56525506
B230217O12Rik	7.17176484	-0.4693644	0.41179901	-1.1397899	0.25437382	0.99514913		B230217O12R	6.04841696	3.62018975	7.31581779	20.6814001	6.65924688	2.35210905	3.84905555	6.84788259
Amigo3	0.89633153	0.34902526	0.30633365	1.13936312	0.25455173	NA		Amigo3	0	0	0.9542371	0	3.32962344	0	2.88679167	0
AU021092	0.70153137	0.29549594	0.25951573	1.13864369	0.2548518	NA		AU021092	0	0	0	0	3.32962344	0	0	2.28262753
Gstm3	0.70153137	0.29549594	0.25951573	1.13864369	0.2548518	NA		Gstm3	0	0	0	0	3.32962344	0	0	2.28262753
Oat	41.3485537	0.29510771	0.25918761	1.13858728	0.25487534	0.9964798		Oat	40.994826	29.8665654	37.8514051	39.2946602	41.620293	52.9224536	47.1509305	41.0872956
Elavl3	251.703896	0.35576877	0.31248159	1.13852715	0.25490043	0.9964798		Elavl3	205.646176	171.959013	163.492624	318.493562	387.901131	209.337705	166.471653	390.329308

GeneID	Base mean	log2(FC)	StdErr	Wald-Stats	P-value	P-adj		GeneID	Normalised expression for Chow#1	Normalised expression for Chow#2	Normalised expression for Chow#3	Normalised expression for Chow#4	Normalised expression for HFD#1	Normalised expression for HFD#2	Normalised expression for HFD#3	Normalised expression for HFD#4
Gm5796	0.63090495	0.3614245	0.31762873	1.13788353	0.25516912	NA		Gm5796	0	0.45252372	0.31807903	0	0	2.35210905	1.92452778	0
AI854703	35.0352069	0.41735808	0.36678892	1.13786993	0.2551748	0.99718767		AI854703	20.8334362	20.3635674	22.9016905	49.6353602	43.2851047	23.5210905	22.1320694	77.6093361
Cecr2	3.801252	-0.473107	0.41579829	-1.1378281	0.25519226	NA		Cecr2	2.68818531	6.78785578	4.77118552	6.20442003	3.32962344	4.7042181	1.92452778	0
Dnah11	0.74250274	0.36028894	0.31673864	1.137496	0.255331	NA		Dnah11	0	0.45252372	0.31807903	0	0	0	2.88679167	2.28262753
Itgb1	18.8221604	-0.4565155	0.40136271	-1.1374138	0.25536533	0.99746363		Itgb1	16.8011582	44.7998482	23.5378486	8.27256004	6.65924688	31.7534722	9.62263889	9.13051012
Rgl2	8.63474378	0.46494096	0.4089462	1.13692452	0.25556984	0.99746363		Rgl2	4.03227797	6.78785578	4.45310648	10.3407001	21.6425524	2.35210905	5.77358333	13.6957652
Tmco3	17.1869008	-0.4261217	0.37482101	-1.136867	0.25559387	0.99746363		Tmco3	14.1129729	28.0564706	29.2632712	10.3407001	9.98887032	24.697145	9.62263889	11.4131377
Hnrnpa1	23.3017417	0.36927496	0.32483506	1.13680761	0.25561872	0.99746363		Hnrnpa1	22.1775288	20.3635674	23.5378486	12.4088401	23.3073641	28.2253086	40.4150833	15.9783927
Atoh8	0.54267822	-0.331699	0.29198716	-1.1360055	0.25595425	NA		Atoh8	0.67204633	2.71514231	0.9542371	0	0	0	0	0
Emp3	0.54267822	-0.331699	0.29198716	-1.1360055	0.25595425	NA		Emp3	0.67204633	2.71514231	0.9542371	0	0	0	0	0
Dcn	5.6262459	-0.4729564	0.41635802	-1.1359369	0.25598297	0.99852045		Dcn	3.36023164	16.7433776	5.72542262	4.13628002	4.99443516	2.35210905	7.69811111	0
Slc13a5	4.16671784	-0.4719827	0.41551591	-1.1358957	0.25600023	NA		Slc13a5	4.7043243	8.59795066	5.08926455	2.06814001	4.99443516	2.35210905	0.96226389	4.56525506
Tex40	0.51970179	-0.3359861	0.29596814	-1.1352104	0.25628718	NA		Tex40	1.34409266	0.90504744	1.90847421	0	0	0	0	0
Ifi203	0.65762878	0.3568499	0.31434934	1.13520168	0.25629082	NA		Ifi203	0	0.45252372	0.31807903	0	0	3.52816357	0.96226389	0
Acadm	9.05613251	-0.4338637	0.3822061	-1.1351565	0.25630975	0.99873346		Acadm	15.4570656	11.313093	8.58813393	8.27256004	3.32962344	10.5844907	5.77358333	9.13051012
Nefl	184.211173	0.35078042	0.30904932	1.13503056	0.25636251	0.99873346		Nefl	129.704941	113.583453	149.179067	239.904241	324.638285	159.943415	137.603736	219.132243
Prkab2	21.9281378	-0.3547624	0.31258073	-1.1349464	0.25639778	0.99873346		Prkab2	18.8172972	24.4362808	23.5378486	35.1583802	21.6425524	15.2887088	18.2830139	18.2610202
Gm16062	1.9320211	-0.4241535	0.37375978	-1.1348291	0.25644694	NA		Gm16062	2.01613899	0	4.13502745	6.20442003	0	1.17605452	1.92452778	0
Bex2	211.707375	0.32187978	0.28383584	1.13403501	0.25677986	0.99873346		Bex2	190.861157	120.371309	154.90449	275.062621	239.732888	171.703961	232.867861	308.154717
Pebp1	116.562036	0.36998244	0.32626756	1.13398474	0.25680095	0.99873346		Pebp1	98.7908103	69.236129	75.7028102	150.974221	129.855314	65.8590533	136.641472	205.436478
1810022K09Rik	1.40215053	-0.4383772	0.38668004	-1.133695	0.2569225	NA		1810022K09Ri	3.36023164	1.35757116	1.59039517	2.06814001	1.66481172	1.17605452	0	0
Cog3	14.7838473	-0.3894601	0.34362167	-1.133398	0.25704714	0.99873346		Cog3	14.7850192	20.3635674	16.8581888	18.6132601	6.65924688	16.4647633	15.3962222	9.13051012
Dhodh	11.3084519	0.42892899	0.37844612	1.13339513	0.25704836	0.99873346		Dhodh	8.73660227	6.33533207	7.31581779	14.4769801	8.3240586	7.05632714	15.3962222	22.8262753
Emr1	0.69423563	-0.3236889	0.28560755	-1.1333344	0.25707383	NA		Emr1	0	3.16766603	0.31807903	2.06814001	0	0	0	0
Myeov2	16.0698595	-0.4191922	0.36997181	-1.1330382	0.25719821	0.99873346		Myeov2	13.4409266	20.3635674	23.8559276	20.6814001	6.65924688	9.40843619	25.0188611	9.13051012
Trpc7	5.68541159	-0.4662756	0.41162256	-1.1327745	0.25730895	0.99873346		Trpc7	6.04841696	9.95552182	6.36158069	6.20442003	3.32962344	0	6.73584722	6.84788259
Aacs	25.0486423	0.44056032	0.38894544	1.13270468	0.25733829	0.99873346		Aacs	20.1613899	14.480759	14.9497146	28.9539601	68.2572805	11.7605452	14.4339583	27.3915304
Kcna2	232.790188	0.36782843	0.32474178	1.13267974	0.25734877	0.99873346		Kcna2	151.210424	262.011233	245.238936	126.156541	203.10703	468.069701	182.830139	223.697498
Lcp2	1.96562563	-0.4465633	0.39426647	-1.1326433	0.25736408	NA		Lcp2	6.72046328	2.71514231	2.22655324	0	0	1.17605452	2.88679167	0
Rell2	49.4188142	0.35534594	0.31381033	1.13235895	0.25748355	0.99873346		Rell2	34.2743627	47.5149905	42.6225906	41.3628002	59.9332219	35.2816357	38.4905555	95.8703563
Frmpd4	101.883589	0.38857083	0.34324357	1.13205566	0.25761103	0.99873346		Frmpd4	100.806949	77.8340797	115.46269	43.4309402	86.5702095	190.520833	143.377319	57.0656883
Dpf2	34.1252465	-0.3518053	0.31086585	-1.131695	0.25776266	0.99873346		Dpf2	43.6830113	34.3918026	32.7621406	45.4990802	46.6147282	17.6408179	25.0188611	27.3915304
L3hypdh	0.7076716	-0.3261444	0.28825057	-1.1314615	0.25786089	NA		L3hypdh	2.68818531	0.90504744	0	2.06814001	0	0	0	0
Plxnb1	48.5042348	-0.4226125	0.37352821	-1.1314071	0.2578838	0.99873346		Plxnb1	44.3550577	82.8118406	58.2084633	45.4990802	81.5757743	22.345036	14.4339583	38.804668
Ssbp2	57.2135139	0.36090111	0.31908691	1.13104327	0.25803688	0.99873346		Ssbp2	53.0916599	48.8725616	62.9796488	28.9539601	54.9387868	69.3872169	102.962236	36.5220405
Zfhx2	54.23758	-0.3545582	0.31359461	-1.130626	0.25821255	0.99873346		Zfhx2	62.5003085	65.1634155	53.1191988	68.2486203	83.240586	41.1619083	30.7924444	29.6741579
Evc	3.47747491	-0.4703585	0.4160627	-1.1304991	0.25826596	NA		Evc	4.03227797	4.07271347	4.45310648	6.20442003	4.99443516	1.17605452	2.88679167	0
Decr2	30.6057233	-0.3454768	0.30563123	-1.1303713	0.25831981	0.99873346		Decr2	40.3227797	35.2968501	33.3982986	31.0221002	16.6481172	19.9929269	38.4905555	29.6741579
Tmem223	22.6712059	-0.4420715	0.39109255	-1.1303501	0.25832875	0.99873346		Tmem223	18.8172972	19.9110436	20.3570582	51.7035003	8.3240586	10.5844907	10.5849028	41.0872956
Plekhm2	48.8353197	0.34720367	0.30722514	1.1301278	0.25842238	0.99873346		Plekhm2	39.6507334	32.5817078	31.8079035	66.1804803	71.586904	35.2816357	51.96225	61.6309433
Shisa5	36.835071	-0.3755433	0.33231821	-1.1300714	0.25844614	0.99873346		Shisa5	29.5700384	57.0179886	42.9406697	39.2946602	48.2795399	24.697145	16.3584861	36.5220405
Psmd9	13.9884376	-0.4221804	0.37360468	-1.1300191	0.25846819	0.99873346		Psmd9	16.8011582	9.05047438	18.130505	24.8176801	14.9833055	5.88027262	15.3962222	6.84788259
Tnfaip6	1.29641006	-0.4128158	0.36534248	-1.1299419	0.25850072	NA		Tnfaip6	0	3.62018975	2.54463228	2.06814001	0	1.17605452	0.96226389	0
Mkln1os	1.24713184	-0.43336	0.38358539	-1.1297614	0.25857676	NA		Mkln1os	2.68818531	1.81009488	1.27231614	2.06814001	0	1.17605452	0.96226389	0
Phax	29.4561696	0.35704742	0.31611094	1.12950039	0.25868681	0.99873346		Phax	29.5700384	21.7211385	27.672876	22.7495401	24.9721758	36.4576902	51.96225	20.5436478
Tcap	1.03227326	0.3816288	0.33791791	1.12935358	0.25874871	NA		Tcap	0	0	0.31807903	2.06814001	1.66481172	0	1.92452778	2.28262753
Spata5	8.16385309	0.43947537	0.38921601	1.12912975	0.2588431	0.99873346		Spata5	7.39250961	8.14542694	7.31581779	2.06814001	4.99443516	14.1126543	14.4339583	6.84788259
Bcl9	121.728596	0.39601024	0.3507664	1.12898568	0.25890388	0.99873346		Bcl9	70.5648645	95.029981	68.7050715	167.519341	146.503431	99.9646345	72.1697916	253.371656
Chic1	41.4602115	0.37581778	0.33294437	1.12877048	0.25899467	0.99873346		Chic1	33.6023164	40.7271347	36.579089	28.9539601	24.9721758	82.3238167	54.8490416	29.6741579
Ikbkap	38.7991171	0.36854613	0.32661338	1.12838653	0.25915672	0.99873346		Ikbkap	26.8818531	25.793852	36.579089	43.4309402	64.9276571	58.8027262	28.8679167	25.1089028
Kcnab2	185.110108	0.3472239	0.3078008	1.1280799	0.25928618	0.99873346		Kcnab2	180.780462	113.13093	136.137827	204.745861	293.006863	127.013889	145.301847	280.763186
Pex6	42.6153722	0.46207242	0.40963075	1.12802182	0.2593107	0.99873346		Pex6	14.1129729	19.9110436	14.3135566	78.5893204	78.2461509	23.5210905	16.3584861	95.8703563
Rai14	4.50233748	-0.4677072	0.4147254	-1.1277515	0.25942488	NA		Rai14	9.4086486	6.33533207	5.40734359	2.06814001	4.99443516	5.88027262	1.92452778	0
Stard8	6.88852483	-0.4528625	0.40162734	-1.1275688	0.25950206	0.99873346		Stard8	10.7527413	11.7656167	6.36158069	6.20442003	6.65924688	4.7042181	8.660375	0
Syce2	1.58609481	0.42832066	0.3798635	1.12756466	0.25950382	NA		Syce2	1.34409266	0.45252372	1.59039517	0	0	3.52816357	5.77358333	0
Urod	20.4120061	-0.3756342	0.33314781	-1.1275302	0.25951837	0.99873346		Urod	24.8657142	17.1959013	21.3112953	33.0902402	23.3073641	10.5844907	19.2452778	13.6957652
Col4a5	0.52587263	-0.3303265	0.2929831	-1.1274593	0.25954832	NA		Col4a5	0.67204633	2.26261859	1.27231614	0	0	0	0	0
Ugt1a6a	0.52587263	-0.3303265	0.2929831	-1.1274593	0.25954832	NA		Ugt1a6a	0.67204633	2.26261859	1.27231614	0	0	0	0	0
Siah2	16.4096397	-0.4005981	0.35535048	-1.1273325	0.25960195	0.99873346		Siah2	18.8172972	14.480759	14.3135566	31.0221002	18.3129289	12.9365998	7.69811111	13.6957652
Rpl24	1.02441796	-0.4048858	0.35918474	-1.1272355	0.25964292	NA		Rpl24	2.01613899	1.81009488	0.63615807	2.06814001	1.66481172	0	0	0
Hist1h2bl	0.59173945	-0.3548654	0.31483055	-1.127163	0.25967356	NA		Hist1h2bl	0.67204633	1.35757116	0.63615807	2.06814001	0	0	0	0
Nfkbid	0.59173945	-0.3548654	0.31483055	-1.127163	0.25967356	NA		Nfkbid	0.67204633	1.35757116	0.63615807	2.06814001	0	0	0	0
Pld2	10.9648282	-0.4539812	0.40281565	-1.1270197	0.25973415	0.99873346		Pld2	10.7527413	11.7656167	10.8146872	20.6814001	14.9833055	1.17605452	3.84905555	13.6957652
A330041J22Rik	0.53195673	0.34380051	0.30506126	1.12698845	0.25974736	NA		A330041J22Ri	0	0.45252372	0	0	1.66481172	1.17605452	0.96226389	0
Kbtbd7	41.3271453	0.28000243	0.24847605	1.12687893	0.25979367	0.99873346		Kbtbd7	38.3066407	35.2968501	39.4418003	33.0902402	49.9443516	42.3379629	44.2641389	47.9351782
Hist1h2bf	2.50772914	-0.4567875	0.40535946	-1.1268703	0.25979731	NA		Hist1h2bf	2.68818531	3.16766603	0.9542371	8.27256004	1.66481172	2.35210905	0.96226389	0
Abcg2	4.69086268	-0.4663585	0.41387067	-1.1268217	0.25981786	0.99873346		Abcg2	7.39250961	9.5029981	6.67965972	0	3.32962344	3.52816357	4.81131944	2.28262753
Zfp641	5.78817731	0.45017074	0.39950652	1.12681701	0.25981986	0.99873346		Zfp641	3.36023164	3.62018975	5.40734359	6.20442003	8.3240586	9.40843619	7.69811111	2.28262753
D430042O09Rik	16.7908555	0.39413947	0.3497897	1.12678981	0.25983136	0.99873346		D430042O09R	17.4732045	14.480759	14.9497146	6.20442003	31.6314227	18.8168724	12.5094306	18.2610202
Rps27a	7.59647542	-0.4546252	0.40356956	-1.1265102	0.25994964	0.99873346		Rps27a	7.39250961	7.2403795	8.90621297	16.5451201	3.32962344	3.52816357	11.5471667	2.28262753
Desi2	15.463019	-0.3895668	0.34586288	-1.1263619	0.26001235	0.99873346		Desi2	16.8011582	20.8160911	17.4943469	18.6132601	4.99443516	15.2887088	18.2830139	11.4131377
Eml2	129.460923	0.45970246	0.40816987	1.12625279	0.26005853	0.99873346		Eml2	51.7475673	31.6766603	45.4853019	256.449361	211.431088	75.2674895	76.0188472	287.611069
Sp2	23.8827932	-0.3796276	0.33712843	-1.1260622	0.26013918	0.99873346		Sp2	20.1613899	28.0564706	20.0389792	43.4309402	19.9777406	15.2887088	14.4339583	29.6741579
Ralbp1	76.3734746	0.27285084	0.24253128	1.12501297	0.26058354	0.99873346		Ralbp1	67.2046328	69.236129	56.6180681	84.7937404	91.5646446	95.2604165	84.6792222	61.6309433
Tada3	13.3803565	0.42571742	0.37846173	1.12486254	0.26064729	0.99873346		Tada3	16.1291119	4.07271347	11.4508452	10.3407001	13.3184938	14.1126543	12.5094306	25.1089028
Aar2	28.6523089	0.36962279	0.32861236	1.12479879	0.26067431	0.99873346		Aar2	18.1452509	23.9837571	24.1740066	28.9539601	36.6258578	22.345036	20.2075417	54.7830607
Pcsk2	124.534924	-0.385101	0.3423832	-1.1247661	0.26068818	0.99873346		Pcsk2	158.602934	194.585199	179.078496	47.5672202	124.860879	137.598379	74.0943194	79.8919636
Kcnq5	35.3340614	-0.4399838	0.3912087	-1.1246781	0.26072548	0.99873346		Kcnq5	38.978687	74.6664136	50.2564875	8.27256004	16.6481172	50.5703445	25.0188611	18.2610202
Coch	11.0791089	-0.4190716	0.37264139	-1.1245976	0.2607596	0.99873346		Coch	11.4247876	21.7211385	11.1327662	8.27256004	9.98887032	10.5844907	8.660375	6.84788259
Ddt	20.8959328	-0.4072615	0.36217732	-1.1244809	0.26080906	0.99873346		Ddt	21.5054825	18.5534725	16.2220308	45.4990802	9.98887032	11.7605452	23.0943333	20.5436478
Psmd14	36.057521	0.32760315	0.29133986	1.12447075	0.26081337	0.99873346		Psmd14	30.9141311	24.8888045	31.1717454	39.2946602	46.6147282	24.697145	45.2264028	45.6525506
Cd109	0.53035867	-0.3303962	0.29382468	-1.1244671	0.26081493	NA		Cd109	1.34409266	2.26261859	0.63615807	0	0	0	0	0
Zmynd10	1.21571312	-0.3842377	0.34173872	-1.1243609	0.26085996	NA		Zmynd10	2.01613899	0	1.90847421	4.13628002	1.66481172	0	0	0
Aox4	0.73018319	0.33393133	0.29701675	1.12428451	0.26089235	NA		Aox4	0.67204633	0	0	0	0	0	2.88679167	2.28262753
Sox5	28.4579708	0.35458414	0.3154221	1.12415759	0.26094618	0.99873346		Sox5	24.8657142	20.8160911	23.5378486	31.0221002	23.3073641	43.5140174	42.3396111	18.2610202
Dis3	7.96297053	-0.4387947	0.39034009	-1.1241343	0.26095605	0.99873346		Dis3	10.7527413	6.78785578	8.90621297	14.4769801	4.99443516	5.88027262	9.62263889	2.28262753
Fam131b	91.194843	0.33680475	0.29963363	1.12405525	0.26098959	0.99873346		Fam131b	83.3337447	72.403795	71.8858618	86.8618804	176.470042	70.5632714	72.1697916	95.8703563
Cyp7b1	5.38620944	-0.4661277	0.41468443	-1.124054	0.26099013	0.99873346		Cyp7b1	4.7043243	12.6706641	6.67965972	4.13628002	0	5.88027262	6.73584722	2.28262753
Mdfic	1.57778245	-0.4157936	0.37000933	-1.1237382	0.26112411	NA		Mdfic	1.34409266	3.62018975	4.77118552	0	0	0	2.88679167	0
C230037L18Rik	0.75848331	0.38278127	0.34064353	1.12370042	0.26114014	NA		C230037L18Ri	0	0.45252372	0.63615807	0	1.66481172	2.35210905	0.96226389	0
Fubp3	16.0476408	0.40866037	0.36384629	1.1231676	0.26136632	0.99932603		Fubp3	10.7527413	8.59795066	11.1327662	24.8176801	21.6425524	11.7605452	25.981125	13.6957652
Nfx1	60.8960857	0.28646042	0.25511288	1.12287713	0.26148968	0.99932603		Nfx1	59.8121232	51.587704	51.8468826	53.7716403	48.2795399	88.2040893	60.622625	73.044081
Tmem88	11.1599098	-0.4278278	0.38103192	-1.1228135	0.26151671	0.99932603		Tmem88	19.4893435	13.1231878	11.1327662	8.27256004	4.99443516	8.23238167	5.77358333	18.2610202
Zfp324	7.14346311	0.43831701	0.3903757	1.12280814	0.26151899	0.99932603		Zfp324	2.68818531	6.33533207	6.67965972	6.20442003	8.3240586	5.88027262	9.62263889	11.4131377
Rpl17	3.31039713	-0.4667518	0.41574549	-1.1226865	0.26157068	NA		Rpl17	4.03227797	4.07271347	6.36158069	2.06814001	0	1.17605452	1.92452778	6.84788259
Odf2	23.3861302	0.36382515	0.32425185	1.12204494	0.26184333	0.99934		Odf2	29.5700384	13.5757116	20.0389792	16.5451201	23.3073641	31.7534722	31.7547083	20.5436478
Cwf19l2	8.53950618	0.46448023	0.41404008	1.12182432	0.26193715	0.99934		Cwf19l2	8.73660227	7.2403795	6.67965972	2.06814001	4.99443516	16.4647633	22.1320694	0
Cep112	2.11049145	0.43258171	0.38577473	1.12133243	0.26214639	NA		Cep112	1.34409266	2.71514231	0.63615807	0	0	3.52816357	8.660375	0
Camsap3	25.590552	0.41185988	0.36731155	1.12128215	0.26216778	0.99934		Camsap3	27.5538995	13.1231878	27.0367179	12.4088401	44.9499164	17.6408179	16.3584861	45.6525506
Bach1	14.6221077	0.36695149	0.32726307	1.12127376	0.26217135	0.99934		Bach1	10.0806949	13.5757116	12.0870033	14.4769801	18.3129289	16.4647633	18.2830139	13.6957652
Ndufv1	84.5303503	0.40513999	0.36134757	1.12119198	0.26220615	0.99934		Ndufv1	59.8121232	38.4645161	45.1672229	134.429101	123.196067	51.7463991	75.0565833	148.37079
Vwa3a	2.28520199	-0.4599743	0.41026161	-1.121173	0.26221422	NA		Vwa3a	2.68818531	2.71514231	1.90847421	6.20442003	1.66481172	1.17605452	1.92452778	0
Med26	9.34703124	0.45177155	0.40301515	1.12097907	0.26229677	0.99934		Med26	8.06455594	3.62018975	6.04350166	10.3407001	16.6481172	4.7042181	4.81131944	20.5436478
Lrrc3	6.96095711	-0.4575769	0.40842098	-1.120356	0.2625621	0.99934		Lrrc3	7.39250961	12.2181404	7.31581779	8.27256004	6.65924688	10.5844907	0.96226389	2.28262753
Tenm1	57.3733144	0.44458082	0.39689454	1.1201485	0.26265048	0.99934		Tenm1	44.3550577	59.7331309	53.1191988	18.6132601	39.9554813	168.175797	56.7735694	18.2610202
Zbtb10	7.53322661	0.43262271	0.3864421	1.11950201	0.26292603	0.99934		Zbtb10	8.06455594	7.69290322	3.81694841	4.13628002	8.3240586	11.7605452	9.62263889	6.84788259
Lrp1	341.389741	0.41235858	0.36837862	1.11938791	0.26297469	0.99934		Lrp1	267.474439	363.376546	243.012382	227.495401	804.104061	410.443029	111.622611	303.589462
Med22	17.4820847	0.39567294	0.35350924	1.11927183	0.26302419	0.99934		Med22	16.8011582	11.7656167	9.54237104	20.6814001	33.2962344	14.1126543	15.3962222	18.2610202
Clp1	2.6485877	-0.4607336	0.41164643	-1.1192459	0.26303524	NA		Clp1	6.72046328	1.81009488	3.81694841	2.06814001	0	3.52816357	0.96226389	2.28262753
Hist1h4a	12.7394033	-0.4410188	0.39403239	-1.119245	0.26303562	0.99934		Hist1h4a	24.8657142	13.1231878	15.2677937	8.27256004	6.65924688	2.35210905	15.3962222	15.9783927
Pdk2	117.68901	-0.3274201	0.29263632	-1.1188637	0.26319831	0.99934		Pdk2	144.489961	138.019734	103.375686	144.769801	126.525691	56.4506172	81.7924305	146.088162
Pnp2	0.98632075	-0.402993	0.3602347	-1.1186956	0.26327003	NA		Pnp2	1.34409266	0.90504744	1.90847421	2.06814001	1.66481172	0	0	0

GeneID	Base mean	log2(FC)	StdErr	Wald-Stats	P-value	P-adj		GeneID	Normalised expression for Chow#1	Normalised expression for Chow#2	Normalised expression for Chow#3	Normalised expression for Chow#4	Normalised expression for HFD#1	Normalised expression for HFD#2	Normalised expression for HFD#3	Normalised expression for HFD#4
Alox5ap	1.84088611	-0.4442574	0.39717493	-1.1185434	0.263335	NA		Alox5ap	0.67204633	4.52523719	1.59039517	4.13628002	1.66481172	1.17605452	0.96226389	0
Dynll1	129.15561	-0.4333658	0.38754927	-1.1182212	0.26347252	0.99934		Dynll1	191.533204	185.534725	186.076235	66.1804803	31.6314227	102.316744	217.471639	52.5004332
St7l	13.1376603	0.4310375	0.38548353	1.11817357	0.26349287	0.99934		St7l	9.4086486	16.7433776	13.0412404	0	11.653682	19.9929269	18.2830139	15.9783927
Zfp334	26.333643	0.34517173	0.30871018	1.11810935	0.26352029	0.99934		Zfp334	20.1613899	16.7433776	22.9016905	33.0902402	23.3073641	38.8097993	25.981125	29.6741579
Wdr7	232.92871	0.26424452	0.2363632	1.11795967	0.26358422	0.99934		Wdr7	233.200076	173.769108	191.483579	239.904241	316.314227	219.922196	194.377305	294.458952
Dut	18.2047756	0.38424316	0.34390092	1.11730773	0.26386277	0.99934		Dut	14.1129729	14.480759	10.1785291	22.7495401	19.9777406	12.9365998	19.2452778	31.9567854
A730046J19Rik	6.33840685	-0.4634197	0.41482183	-1.1171536	0.26392865	0.99934		A730046J19Ri	8.06455594	9.05047438	6.67965972	10.3407001	0	11.7605452	4.81131944	0
Slmo2	21.1995743	-0.3484787	0.31194012	-1.1171333	0.26393734	0.99934		Slmo2	27.5538995	27.1514231	20.9932163	22.7495401	16.6481172	19.9929269	23.0943333	11.4131377
Elp5	47.7213647	-0.3304557	0.29583034	-1.1170447	0.26397522	0.99934		Elp5	53.0916599	47.5149905	44.2129858	74.4530404	36.6258578	27.0492541	57.7358333	41.0872956
Ankrd6	35.4249569	-0.3465806	0.31028929	-1.1169594	0.26401166	0.99934		Ankrd6	35.6184554	44.7998482	43.8949068	39.2946602	18.3129289	48.2182355	32.7169722	20.5436478
Tbx3	2.09161324	-0.4425316	0.39622259	-1.1168763	0.2640472	NA		Tbx3	1.34409266	3.62018975	1.27231614	6.20442003	3.32962344	0	0.96226389	0
Nt5c1a	4.38386736	-0.461247	0.41310927	-1.1165254	0.26419729	NA		Nt5c1a	5.37637063	2.71514231	6.67965972	8.27256004	3.32962344	3.52816357	2.88679167	2.28262753
Lrrc48	4.89810465	0.457104	0.40940075	1.11651968	0.26419975	0.99934		Lrrc48	4.7043243	5.43028463	3.49886938	0	6.65924688	4.7042181	9.62263889	4.56525506
Gm20754	1.07928553	0.39640926	0.35511791	1.11627503	0.26430443	NA		Gm20754	0.67204633	0	1.27231614	0	1.66481172	1.17605452	3.84905555	0
Srebf1	18.6175082	-0.4186916	0.37508163	-1.116268	0.26430742	0.99934		Srebf1	17.4732045	29.8665654	14.3135566	26.8858201	26.6369875	5.88027262	9.62263889	18.2610202
Tmem97	3.69050172	-0.4643393	0.41617142	-1.1157404	0.26453328	NA		Tmem97	4.7043243	7.69290322	6.67965972	0	4.99443516	3.52816357	1.92452778	0
Tnni1	0.62701329	-0.3290471	0.29493803	-1.1156482	0.26457274	NA		Tnni1	0	1.35757116	1.59039517	2.06814001	0	0	0	0
Vars	29.881214	0.4435873	0.39762602	1.11558921	0.26459801	0.99934		Vars	18.1452509	15.3858064	11.7689243	47.5672202	44.9499164	14.1126543	16.3584861	70.7614535
Rnf14	341.029387	0.26546546	0.23796322	1.11557351	0.26460473	0.99934		Rnf14	305.781079	265.178899	368.335522	287.471461	263.040252	359.872684	485.943264	392.611935
Ddx52	12.1115098	0.44951109	0.40300116	1.11540893	0.26467523	0.99934		Ddx52	13.4409266	10.4080455	8.90621297	4.13628002	3.32962344	19.9929269	29.8301805	6.84788259
Trappc13	60.4848169	0.38692003	0.34694499	1.11522013	0.2647561	0.99934		Trappc13	56.4518916	49.3250854	63.9338859	31.0221002	34.9610461	85.8519803	121.24525	41.0872956
Sf3b2	155.699265	0.27962084	0.25075504	1.11511551	0.26480093	0.99934		Sf3b2	144.489961	108.153169	108.78303	198.541441	163.151549	159.943415	161.660333	200.871223
Arid5a	8.13375918	0.43344749	0.38872707	1.11504322	0.2648319	0.99934		Arid5a	8.73660227	5.88280835	3.18079035	8.27256004	11.653682	8.23238167	7.69811111	11.4131377
Uqcc1	27.3799476	0.35911077	0.32215926	1.1146995	0.26497921	0.99934		Uqcc1	25.5377605	13.5757116	20.6751372	35.1583802	28.3017992	32.9295267	24.0565972	38.804668
Sympk	55.598321	0.34150576	0.30640153	1.11456939	0.26503499	0.99934		Sympk	47.7152893	41.1796584	38.4875632	64.1123403	84.9053977	43.5140174	40.4150833	84.4572186
Fam86	6.20449774	0.45831713	0.41121893	1.11453315	0.26505053	0.99934		Fam86	3.36023164	4.97776091	4.77118552	4.13628002	8.3240586	9.40843619	0.96226389	13.6957652
Zcchc14	92.8162759	0.31438557	0.28208904	1.11449052	0.26506881	0.99934		Zcchc14	75.2691888	81.0017457	69.9773876	99.2707205	151.497867	103.492798	61.5848889	100.435611
Nfyb	16.7412026	0.41567131	0.3730499	1.11425122	0.26517143	0.99934		Nfyb	16.8011582	13.5757116	10.8146872	14.4769801	18.3129289	18.8168724	36.5660278	4.56525506
Ebna1bp2	28.1577508	0.33603917	0.3016337	1.11406374	0.26525184	0.99934		Ebna1bp2	22.1775288	23.5312334	21.6293743	31.0221002	18.3129289	30.5774176	34.6415	43.3699231
Txndc5	37.5287564	-0.3791874	0.34043385	-1.1138357	0.26534968	0.99934		Txndc5	39.6507334	67.8785578	47.3937761	18.6132601	26.6369875	47.042181	27.9056528	25.1089028
Hjurp	26.8088132	0.36604008	0.3286681	1.11370736	0.26540475	0.99934		Hjurp	22.1775288	17.648425	15.5858727	37.2265202	36.6258578	22.345036	24.0565972	38.804668
5930430L01Rik	1.93075481	0.4386929	0.39393465	1.11361847	0.2654429	NA		5930430L01Ri	0	1.81009488	2.22655324	0	4.99443516	3.52816357	2.88679167	0
Nedd9	6.11497156	-0.4404649	0.39553895	-1.1135816	0.26545871	0.99934		Nedd9	5.37637063	9.05047438	7.63389683	8.27256004	3.32962344	5.88027262	4.81131944	4.56525506
Zfp592	26.269231	-0.321236	0.28852491	-1.1133734	0.26554808	0.99934		Zfp592	28.2259458	31.2241366	29.5813502	28.9539601	24.9721758	27.0492541	17.32075	22.8262753
Scube1	24.4768	-0.4634272	0.41636705	-1.1130257	0.26569738	0.99934		Scube1	28.8979921	64.7108918	34.0344567	0	24.9721758	30.5774176	5.77358333	6.84788259
Med9	40.7038589	-0.3491102	0.31368265	-1.1129407	0.2657339	0.99934		Med9	42.3389187	36.2018975	36.2610099	74.4530404	46.6147282	23.5210905	36.5660278	29.6741579
Unc5d	76.9850381	0.43706475	0.39280737	1.11266943	0.26585043	0.99934		Unc5d	39.6507334	109.058216	70.6135457	18.6132601	58.2684102	199.929269	74.0943194	45.6525506
Rrbp1	22.7263384	0.42148744	0.37888902	1.1124298	0.2659534	0.99934		Rrbp1	12.7688802	25.3413283	11.1327662	22.7495401	31.6314227	29.4013631	7.69811111	41.0872956
1810009A15Rik	4.7409489	-0.4575315	0.4112903	-1.1124296	0.26595347	0.99934		1810009A15Ri	8.73660227	6.78785578	5.40734359	2.06814001	1.66481172	3.52816357	2.88679167	6.84788259
Tiam1	167.23004	0.3385058	0.30430634	1.112385	0.26597265	0.99934		Tiam1	190.189111	157.930778	156.812964	68.2486203	223.084771	248.147505	140.490528	152.936045
2010204K13Rik	22.4304577	-0.3876416	0.34848763	-1.1123541	0.26598595	0.99934		2010204K13Ri	25.5377605	23.9837571	17.1762679	41.3628002	8.3240586	18.8168724	25.981125	18.2610202
Dpysl5	59.499584	0.40047631	0.36004457	1.11229648	0.26601069	0.99934		Dpysl5	38.978687	25.793852	31.4898244	101.338861	73.2517157	55.2745626	47.1509305	102.718239
Runx1	2.41265993	-0.4568169	0.41079513	-1.112031	0.26612481	NA		Runx1	4.03227797	5.88280835	1.59039517	2.06814001	1.66481172	1.17605452	2.88679167	0
Pex10	6.23615876	0.44314036	0.39851185	1.1119879	0.26614335	0.99938111		Pex10	3.36023164	4.97776091	5.72542262	4.13628002	11.653682	7.05632714	3.84905555	9.13051012
Rps3	53.1777176	0.35046547	0.31531624	1.11147295	0.26636483	0.99938111		Rps3	71.2369108	36.6544212	40.7141164	33.0902402	43.2851047	52.9224536	90.4528055	57.0656883
Amacr	8.41719009	0.43821052	0.39433618	1.11126127	0.26645591	0.99938111		Amacr	6.72046328	8.59795066	3.81694841	6.20442003	16.6481172	5.88027262	5.77358333	13.6957652
Eef2k	16.2829014	-0.3853898	0.34695476	-1.1107784	0.26666377	0.99938111		Eef2k	22.1775288	18.1009488	16.2220308	20.6814001	21.6425524	8.23238167	16.3584861	6.84788259
Ak5	136.615335	0.26762859	0.24097517	1.11060652	0.26673776	0.99938111		Ak5	157.258841	120.823833	121.188112	88.9300204	184.794101	129.365998	155.88675	134.675024
Grk5	7.57997377	0.44009778	0.3962939	1.11053382	0.26676906	0.99938111		Grk5	9.4086486	5.88280835	6.99773876	0	11.653682	10.5844907	11.5471667	4.56525506
Tmem192	13.3212974	-0.3831008	0.34511396	-1.1100703	0.26696873	0.99938111		Tmem192	17.4732045	14.9332827	16.2220308	12.4088401	9.98887032	7.05632714	12.5094306	15.9783927
Bid	8.13757226	0.42796863	0.38559737	1.10988473	0.2670487	0.99938111		Bid	10.0806949	5.43028463	7.31581779	2.06814001	11.653682	8.23238167	13.4716944	6.84788259
Shc1	3.18116931	-0.4597578	0.41442927	-1.1093758	0.2672681	NA		Shc1	5.37637063	4.97776091	2.54463228	4.13628002	0	0	3.84905555	4.56525506
Tnnc1	1.03409331	-0.3897812	0.35149712	-1.1089171	0.26746594	NA		Tnnc1	3.36023164	1.35757116	1.27231614	0	0	0	0	2.28262753
Cdh19	2.03198226	-0.4441023	0.4004939	-1.1088867	0.26747907	NA		Cdh19	1.34409266	4.52523719	5.40734359	0	1.66481172	2.35210905	0.96226389	0
Ccdc120	12.5271833	0.45604914	0.41131442	1.10876039	0.26753356	0.99938111		Ccdc120	6.72046328	4.97776091	10.4966081	14.4769801	31.6314227	2.35210905	6.73584722	22.8262753
Rpn2	66.1721587	-0.3424824	0.30890147	-1.1087108	0.26755498	0.99938111		Rpn2	59.1400769	110.415787	77.6112844	53.7716403	53.2739751	85.8519803	41.3773472	47.9351782
Afg3l2	46.3288158	0.29703251	0.26795361	1.10852213	0.26763639	0.99938111		Afg3l2	34.9464091	39.3695635	40.3960374	51.7035003	39.9554813	68.2111624	48.1131944	47.9351782
Mroh8	1.12974544	0.3831069	0.34575279	1.10803703	0.26784582	NA		Mroh8	0	0.90504744	0.9542371	0	3.32962344	0	3.84905555	0
Tubgcp2	27.9622402	0.34159215	0.30830126	1.10798169	0.26786972	0.99938111		Tubgcp2	28.8979921	20.8160911	20.3570582	24.8176801	33.2962344	23.5210905	24.0565972	47.9351782
Amph	164.589104	0.21887661	0.19759447	1.10770613	0.26798875	0.99938111		Amph	161.963165	148.42778	156.494885	136.497241	214.760712	165.823688	168.39618	164.349182
Usp21	45.034057	-0.3727269	0.33650055	-1.1076562	0.26801031	0.99938111		Usp21	49.059382	40.274611	43.2587487	76.5211804	39.9554813	15.2887088	36.5660278	59.3483158
Pnpla7	3.81870493	-0.4591069	0.41455978	-1.1074563	0.26809669	NA		Pnpla7	1.34409266	10.8605693	6.04350166	2.06814001	4.99443516	2.35210905	2.88679167	0
Ap1b1	133.824993	0.32152949	0.29044972	1.1070057	0.26829147	0.99938111		Ap1b1	99.4628566	91.4097912	103.693765	173.723761	204.771842	128.189943	98.1509166	171.197065
Ntpcr	9.50284084	0.43555928	0.3934883	1.106918	0.26832938	0.99938111		Ntpcr	8.73660227	7.2403795	5.08926455	8.27256004	3.32962344	7.05632714	13.4716944	22.8262753
Bace1	92.8449467	0.31662639	0.28608711	1.10674818	0.26840282	0.99938111		Bace1	71.9089571	109.058216	90.3344458	49.6353602	116.53682	131.718107	73.1320555	100.435611
Mir6236	71067.7675	0.30396375	0.27466309	1.10667856	0.26843293	0.99938111		Mir6236	87021.2629	33372.7192	59580.9743	70267.125	88676.1963	68965.0133	83752.5621	76906.2868
Golga3	32.6912888	0.38324451	0.34637523	1.10644318	0.26853474	0.99938111		Golga3	36.2905017	25.3413283	34.0344567	12.4088401	26.6369875	55.2745626	50.9999861	20.5436478
Gabarap	159.670149	-0.348111	0.3146312	-1.1064098	0.26854919	0.99938111		Gabarap	158.602934	167.433776	146.316356	258.517501	103.218327	76.4435441	138.566	228.262753
Syngr1	431.816117	0.35673113	0.32256382	1.10592417	0.26875934	0.99938111		Syngr1	236.560308	389.170398	333.346828	508.762443	665.924688	449.252828	223.245222	648.266219
Map7	37.182714	-0.3329619	0.301198	-1.1054585	0.26896096	0.99938111		Map7	43.010965	56.1129411	43.5768277	22.7495401	33.2962344	25.8731995	31.7547083	41.0872956
Ttll3	10.3384766	0.42353787	0.38325432	1.10510921	0.26911227	0.99938111		Ttll3	8.06455594	5.88280835	9.86045007	8.27256004	21.6425524	4.7042181	10.5849028	13.6957652
Tagln3	143.988468	-0.3509834	0.31769537	-1.1047799	0.26925499	0.99938111		Tagln3	164.65135	115.846072	132.320878	248.176801	139.844185	61.1548353	116.433931	173.479692
Fam188b	2.40044867	0.45294534	0.41002329	1.10468197	0.26929742	NA		Fam188b	2.68818531	0.45252372	2.22655324	0	4.99443516	2.35210905	1.92452778	4.56525506
Dnal4	41.3717336	-0.4334277	0.39236949	-1.1046417	0.2693149	0.99938111		Dnal4	36.9625481	30.3190892	32.1259825	103.407001	46.6147282	8.23238167	23.0943333	50.2178057
Dak	8.26069488	0.4391129	0.39752612	1.10461395	0.2693269	0.99938111		Dak	8.06455594	6.33533207	5.40734359	4.13628002	16.6481172	4.7042181	4.81131944	15.9783927
Zfp507	19.2256514	0.43625576	0.39518385	1.10393114	0.26962301	0.99938111		Zfp507	11.4247876	15.8383302	20.6751372	12.4088401	9.98887032	42.3379629	36.5660278	4.56525506
Gng13	9.64725545	-0.4585659	0.41541401	-1.1038768	0.26964659	0.99938111		Gng13	20.1613899	12.2181404	12.0870033	6.20442003	1.66481172	2.35210905	20.2075417	2.28262753
Slk	40.8054342	0.39906667	0.36155644	1.10374655	0.2697031	0.99938111		Slk	37.6345944	38.9170398	41.6683535	14.4769801	34.9610461	88.2040893	50.0377222	20.5436478
Ormdl2	7.40384168	-0.4480214	0.40595092	-1.1036344	0.26975175	0.99938111		Ormdl2	12.0968339	13.5757116	8.58813393	2.06814001	8.3240586	9.40843619	2.88679167	2.28262753
Kcna4	25.5734799	-0.4017025	0.36400626	-1.1035593	0.26978434	0.99938111		Kcna4	25.5377605	39.3695635	41.6683535	14.4769801	13.3184938	37.6337448	21.1698055	11.4131377
Tsc22d2	47.1962911	0.28417116	0.25759658	1.10316359	0.26995613	0.99938111		Tsc22d2	47.043243	43.8948007	34.9886938	41.3628002	46.6147282	56.4506172	43.301875	63.9135709
Csrp2	0.84701828	0.37815304	0.34284517	1.10298487	0.27003373	NA		Csrp2	0.67204633	0	0.63615807	0	3.32962344	1.17605452	0.96226389	0
Jmjd7	1.70527465	0.42366169	0.38410909	1.10297228	0.2700392	NA		Jmjd7	0.67204633	1.35757116	0	2.06814001	1.66481172	2.35210905	0.96226389	4.56525506
Gm14023	0.74129125	0.31652686	0.28705889	1.10265479	0.27017711	NA		Gm14023	0	0	0.31807903	0	3.32962344	0	0	2.28262753
Arfgap1	51.0072432	0.37004296	0.33560183	1.10262498	0.27019006	0.99938111		Arfgap1	45.6991503	25.3413283	37.215247	64.1123403	78.2461509	32.9295267	42.3396111	82.1745911
Arfrp1	16.2450601	0.37943553	0.34417106	1.10246203	0.27026086	0.99938111		Arfrp1	12.7688802	9.95552182	11.7689243	20.6814001	18.3129289	11.7605452	17.32075	27.3915304
Ppard	15.9086653	-0.4346332	0.39428344	-1.1023369	0.27031522	0.99938111		Ppard	12.0968339	18.1009488	16.2220308	33.0902402	23.3073641	3.52816357	16.3584861	4.56525506
Pcolce	2.00264958	-0.4362579	0.39579271	-1.1022383	0.27035808	NA		Pcolce	3.36023164	5.88280835	2.22655324	0	1.66481172	0	2.88679167	0
Olfr286	0.57493386	-0.3470593	0.31488	-1.1021954	0.27037675	NA		Olfr286	0.67204633	0.90504744	0.9542371	2.06814001	0	0	0	0
Six3os1	5.44600496	-0.4499687	0.40830478	-1.1020413	0.27044372	0.99938111		Six3os1	4.7043243	8.14542694	6.04350166	8.27256004	4.99443516	5.88027262	0.96226389	4.56525506
Gm5643	27.2104541	0.38861244	0.35264419	1.10199585	0.27046348	0.99938111		Gm5643	28.2259458	19.0059962	28.6271131	14.4769801	23.3073641	36.4576902	53.8867778	13.6957652
Adora2b	2.60952985	-0.4465489	0.40528315	-1.1018195	0.27054015	NA		Adora2b	1.34409266	2.71514231	2.86271131	8.27256004	3.32962344	2.35210905	0	0
Ubr2	50.4800003	0.31765167	0.28834526	1.10163654	0.27061972	0.99938111		Ubr2	53.0916599	38.4645161	46.12146	37.2265202	88.2350212	51.7463991	43.301875	45.6525506
Igfbp6	5.54797295	-0.4583935	0.41617119	-1.1014541	0.27069907	0.99938111		Igfbp6	9.4086486	11.313093	4.45310648	2.06814001	1.66481172	1.17605452	2.88679167	11.4131377
Myom1	0.73175948	0.37453839	0.34012338	1.10118389	0.27081663	NA		Myom1	0	0.45252372	0.63615807	0	1.66481172	1.17605452	1.92452778	0
Mlst8	20.692679	0.40882668	0.37140143	1.10076764	0.2709978	0.99938111		Mlst8	18.8172972	15.3858064	21.6293743	8.27256004	43.2851047	11.7605452	14.4339583	31.9567854
Igf2	27.5792629	-0.4435149	0.40295956	-1.1006437	0.27105176	0.99938111		Igf2	14.1129729	66.068463	34.3525357	20.6814001	6.65924688	9.40843619	25.981125	43.3699231
Alkbh8	21.0126194	0.44225456	0.40185237	1.10053987	0.27109697	0.99938111		Alkbh8	18.8172972	19.4585199	21.3112953	4.13628002	8.3240586	38.8097993	48.1131944	9.13051012
Ndufb5	76.383654	-0.3565846	0.32401405	-1.100522	0.27110474	0.99938111		Ndufb5	84.0057911	72.403795	81.4282328	117.883981	34.9610461	70.5632714	108.735819	41.0872956
Zfp866	13.5213295	0.43625988	0.39645853	1.10039223	0.27116126	0.99938111		Zfp866	12.0968339	13.1231878	12.0870033	4.13628002	4.99443516	35.2816357	17.32075	9.13051012
Cmklr1	0.84946318	-0.3567092	0.32429655	-1.0999475	0.271355	NA		Cmklr1	0.67204633	4.52523719	0.63615807	0	0	0	0.96226389	0
2610318N02Rik	0.56320975	0.27892799	0.25368211	1.09951776	0.27154229	NA		2610318N02R	0	0	0	0	3.32962344	1.17605452	0	0
A930013F10Rik	0.56320975	0.27892799	0.25368211	1.09951776	0.27154229	NA		A930013F10Ri	0	0	0	0	3.32962344	1.17605452	0	0
Tsku	0.91430523	-0.3836362	0.34897459	-1.0993241	0.27162673	NA		Tsku	1.34409266	2.71514231	1.59039517	0	1.66481172	0	0	0
Ppp1r8	27.951911	-0.3824561	0.34793447	-1.0992189	0.2716726	0.99938111		Ppp1r8	30.9141311	20.8160911	24.4920857	55.8397803	16.6481172	15.2887088	23.0943333	36.5220405
Ywhag	1840.23431	0.41344008	0.37614375	1.09915447	0.27170069	0.99938111		Ywhag	1084.01073	879.253586	999.086247	2942.96323	2662.03394	1060.80118	1183.58458	3910.14096
Ngly1	9.24889123	-0.4250227	0.38670294	-1.0990934	0.27172731	0.99938111		Ngly1	14.1129729	12.2181404	14.3135566	4.13628002	8.3240586	7.05632714	11.5471667	2.28262753
Chrna7	14.8501715	0.37954102	0.34535084	1.0990013	0.2717675	0.99938111		Chrna7	8.73660227	14.480759	14.3135566	10.3407001	13.3184938	19.9929269	12.5094306	25.1089028
Akap12	10.7625667	0.43229099	0.39335421	1.09898658	0.27177392	0.99938111		Akap12	7.39250961	8.59795066	6.36158069	12.4088401	14.9833055	24.697145	4.81131944	6.84788259
4933404O12Rik	14.7917205	0.40244309	0.3661984	1.09897556	0.27177873	0.99938111		4933404O12R	6.72046328	12.2181404	17.1762679	10.3407001	26.6369875	14.1126543	10.5849028	20.5436478
Zfp286	7.82177711	-0.4413672	0.40162058	-1.0989655	0.27178309	0.99938111		Zfp286	16.1291119	10.8605693	6.04350166	6.20442003	1.66481172	9.40843619	7.69811111	4.56525506
Mr1	1.60046443	-0.421055	0.38317378	-1.0988616	0.27182842	NA		Mr1	2.01613899	5.88280835	1.59039517	0	0	2.35210905	0.96226389	0

GeneID	Base mean	log2(FC)	StdErr	Wald-Stats	P-value	P-adj		GeneID	Normalised expression for Chow#1	Normalised expression for Chow#2	Normalised expression for Chow#3	Normalised expression for Chow#4	Normalised expression for HFD#1	Normalised expression for HFD#2	Normalised expression for HFD#3	Normalised expression for HFD#4
Rbm38	2.87706257	-0.441044	0.40146486	-1.0985867	0.27194837	NA		Rbm38	2.01613899	2.26261859	2.22655324	10.3407001	4.99443516	1.17605452	0	0
Postn	0.49674749	-0.3246354	0.29553858	-1.0984535	0.2720065	NA		Postn	1.34409266	1.35757116	1.27231614	0	0	0	0	0
Tlr13	0.49674749	-0.3246354	0.29553858	-1.0984535	0.2720065	NA		Tlr13	1.34409266	1.35757116	1.27231614	0	0	0	0	0
Dpp4	0.78224084	-0.3668811	0.33401092	-1.0984105	0.27202527	NA		Dpp4	0.67204633	2.71514231	1.90847421	0	0	0	0.96226389	0
Rpl35a	5.07259423	0.44429757	0.40461543	1.09807374	0.27217229	0.99938111		Rpl35a	4.7043243	3.16766603	5.08926455	2.06814001	6.65924688	4.7042181	9.62263889	4.56525506
Plaa	39.7217373	0.33437265	0.30454241	1.09795102	0.27222588	0.99938111		Plaa	36.2905017	35.7493738	44.8491439	18.6132601	36.6258578	62.3308898	49.0754583	34.239413
Tspan3	182.393209	-0.3495975	0.31842	-1.0979131	0.27224245	0.99938111		Tspan3	129.704941	324.006983	224.563798	157.178641	98.2238915	231.682741	138.566	155.218672
H3f3a	21.7995547	-0.429416	0.39116229	-1.097795	0.27229403	0.99938111		H3f3a	46.3711967	25.3413283	28.3090341	6.20442003	8.3240586	17.6408179	30.7924444	11.4131377
Slu7	37.0176689	0.39633222	0.36102919	1.09778442	0.27229864	0.99938111		Slu7	44.3550577	23.9837571	27.354797	26.8858201	19.9777406	78.7956531	51.96225	22.8262753
Mgea5	143.345497	0.29244713	0.26640981	1.09773408	0.27232062	0.99938111		Mgea5	151.88247	141.639924	133.911274	78.5893204	149.833055	214.041923	155.88675	120.979259
Nrm	1.00833779	-0.3839024	0.34981697	-1.097438	0.27244998	NA		Nrm	1.34409266	3.16766603	1.27231614	0	0	0	0	2.28262753
Pnmal2	524.525275	0.42089607	0.38365366	1.097073	0.27260948	0.99938111		Pnmal2	290.324014	204.088197	279.90955	891.368344	933.959375	376.337448	275.207472	945.007798
Hars	48.2909006	0.30200697	0.27529568	1.09702762	0.27262932	0.99938111		Hars	57.1239379	32.129184	43.8949068	37.2265202	49.9443516	49.39429	66.3962083	50.2178057
Zfp583	3.26428131	0.45608764	0.4158318	1.09680799	0.27272533	NA		Zfp583	1.34409266	4.97776091	1.90847421	0	1.66481172	5.88027262	5.77358333	4.56525506
Cluh	72.8668899	0.36578225	0.33352414	1.09671896	0.27276427	0.99938111		Cluh	48.3873356	51.587704	48.3480132	99.2707205	141.508996	56.4506172	52.9245139	84.4572186
2410127L17Rik	1.86637659	-0.4270847	0.38942871	-1.0966956	0.2727745	NA		2410127L17Ri	4.7043243	0.45252372	3.49886938	2.06814001	0	0	1.92452778	2.28262753
Asxl1	37.7323808	0.31396342	0.28631536	1.09656508	0.27283156	0.99938111		Asxl1	36.9625481	33.0342315	34.0344567	26.8858201	31.6314227	59.9787807	33.6792361	45.6525506
Foxc1	1.97824015	-0.4441467	0.40528161	-1.0958964	0.27312411	NA		Foxc1	2.68818531	4.97776091	3.18079035	0	1.66481172	2.35210905	0.96226389	0
Adamtsl1	4.80594267	0.45545935	0.41561924	1.09585721	0.27314127	0.99938111		Adamtsl1	2.68818531	3.16766603	3.18079035	4.13628002	11.653682	3.52816357	0.96226389	9.13051012
Prr5	10.9485864	-0.4436046	0.40483015	-1.0957797	0.27317521	0.99938111		Prr5	9.4086486	9.95552182	5.40734359	31.0221002	6.65924688	4.7042181	6.73584722	13.6957652
Scarb1	12.5147976	-0.4495387	0.41025985	-1.0957414	0.27319195	0.99938111		Scarb1	10.0806949	18.1009488	11.4508452	22.7495401	19.9777406	1.17605452	2.88679167	13.6957652
Rarg	5.71704324	-0.4557221	0.4160243	-1.0954217	0.27333192	0.99938111		Rarg	7.39250961	10.8605693	2.86271131	8.27256004	8.3240586	1.17605452	0	6.84788259
Tsn	54.6169254	0.29652879	0.27072525	1.09531262	0.2733797	0.99938111		Tsn	38.3066407	44.7998482	48.3480132	64.1123403	39.9554813	62.3308898	68.3207361	70.7614535
Eral1	10.3668095	-0.4096098	0.37399659	-1.0952234	0.27341876	0.99938111		Eral1	12.0968339	9.05047438	11.1327662	18.6132601	6.65924688	4.7042181	11.5471667	9.13051012
Trpc4ap	134.690425	0.27438297	0.25058522	1.09496869	0.27353035	0.99938111		Trpc4ap	129.704941	100.007742	114.826531	136.497241	141.508996	144.654706	104.886764	205.436478
Tmem176a	11.5259191	-0.4129427	0.37713223	-1.0949548	0.27353644	0.99938111		Tmem176a	8.06455594	16.2908539	13.6773985	16.5451201	8.3240586	11.7605452	3.84905555	13.6957652
Rabep1	113.087727	0.37684497	0.34420735	1.09481967	0.27359565	0.99938111		Rabep1	114.247876	105.438027	104.011844	53.7716403	73.2517157	201.105324	186.679194	66.1961984
Rnf168	30.8985463	0.37275501	0.34051217	1.09468924	0.2736528	0.99938111		Rnf168	30.9141311	23.0787097	34.6706148	14.4769801	31.6314227	54.0985081	42.3396111	15.9783927
Itpa	10.3736421	-0.4054989	0.37045003	-1.0946116	0.27368685	0.99938111		Itpa	12.7688802	13.1231878	15.9039517	6.20442003	8.3240586	11.7605452	5.77358333	9.13051012
Chst3	1.40733868	-0.3287462	0.30050868	-1.0939657	0.27397	NA		Chst3	0	1.81009488	0	8.27256004	0	1.17605452	0	0
Adcy6	29.8739768	0.39293425	0.35928833	1.093646	0.27411027	0.99938111		Adcy6	14.1129729	27.6039469	21.3112953	35.1583802	41.620293	36.4576902	12.5094306	50.2178057
Rab11fip4	112.641331	0.27850522	0.254672	1.093584	0.27413747	0.99938111		Rab11fip4	104.839227	78.2866034	84.9271022	136.497241	141.508996	114.077289	97.1886527	143.805534
Sat2	3.13619797	-0.4541976	0.41536373	-1.0934937	0.27417711	NA		Sat2	5.37637063	3.16766603	2.54463228	6.20442003	1.66481172	0	3.84905555	2.28262753
Mcc	22.5606572	-0.38249	0.34994683	-1.0929947	0.27439612	0.99938111		Mcc	22.8495752	36.2018975	24.1740066	22.7495401	31.6314227	12.9365998	23.0943333	6.84788259
Tnfsf13	0.96303718	-0.3764792	0.34455586	-1.0926508	0.27454715	NA		Tnfsf13	2.68818531	2.71514231	0.63615807	0	1.66481172	0	0	0
Slit1	53.9515649	0.36089812	0.33031758	1.09257923	0.27457859	0.99938111		Slit1	32.2582238	61.9957495	52.4830407	33.0902402	89.8998329	65.8590533	29.8301805	66.1961984
Cdc14a	2.74727856	-0.4521786	0.41401595	-1.0921767	0.27475545	NA		Cdc14a	2.01613899	3.16766603	3.81694841	6.20442003	0	3.52816357	0.96226389	2.28262753
Ing3	9.15327388	-0.4406216	0.40343611	-1.0921719	0.27475755	0.99938111		Ing3	13.4409266	12.6706641	13.6773985	6.20442003	4.99443516	16.4647633	5.77358333	0
Impa1	41.4647077	0.28440963	0.26040887	1.09216566	0.27476029	0.99938111		Impa1	38.978687	36.2018975	41.9864326	28.9539601	36.6258578	52.9224536	48.1131944	47.9351782
Wscd2	31.0630381	-0.3335989	0.30558195	-1.091684	0.274972	0.99938111		Wscd2	31.5861774	47.9675142	36.897168	22.7495401	26.6369875	34.1055812	21.1698055	27.3915304
Asb7	23.6018644	0.32651167	0.2991174	1.09158369	0.27501613	0.99938111		Asb7	22.1775288	21.7211385	20.0389792	16.5451201	23.3073641	19.9929269	30.7924444	34.239413
Calml4	0.52589441	0.27591107	0.25285267	1.091193	0.27518797	NA		Calml4	0	0	0	0	0	0	1.92452778	2.28262753
Rab6a	385.166665	0.25989791	0.23819219	1.0911269	0.27521705	0.99938111		Rab6a	361.560925	328.984744	410.958113	285.403321	299.66611	389.274047	539.830041	465.656016
Lmo1	5.67825829	-0.4528822	0.41514993	-1.0908884	0.27532199	0.99938111		Lmo1	6.04841696	5.43028463	6.67965972	10.3407001	1.66481172	0	3.84905555	11.4131377
Plin2	1.7108843	-0.4339252	0.39780438	-1.0908005	0.27536067	NA		Plin2	2.01613899	1.35757116	2.86271131	4.13628002	0	2.35210905	0.96226389	0
Ptrhd1	33.4269814	-0.3400422	0.31178065	-1.0906455	0.27542891	0.99938111		Ptrhd1	57.1239379	34.3918026	31.1717454	31.0221002	28.3017992	30.5774176	36.5660278	18.2610202
Park7	41.3113941	0.3030385	0.27792725	1.09035188	0.27555817	0.99938111		Park7	40.3227797	31.6766603	36.579089	37.2265202	34.9610461	35.2816357	59.6603611	54.7830607
Klk8	1.33763776	-0.416522	0.3820856	-1.0901274	0.27565703	NA		Klk8	1.34409266	1.81009488	1.27231614	4.13628002	0	1.17605452	0.96226389	0
Ubn2	41.5373997	0.33790523	0.30997897	1.09009082	0.27567314	0.99938111		Ubn2	40.994826	38.9170398	35.3067728	28.9539601	44.9499164	55.2745626	67.3584722	20.5436478
Igfbpl1	0.61910195	0.31058575	0.28492111	1.09007628	0.27567954	NA		Igfbpl1	0	0	0.31807903	0	0	2.35210905	0	2.28262753
Trem2	0.92449691	-0.3370007	0.30915715	-1.0900629	0.27568546	NA		Trem2	0	4.52523719	1.90847421	0	0	0	0.96226389	0
Kif3c	297.871516	0.25624285	0.23512894	1.08979715	0.27580251	0.99938111		Kif3c	235.216215	236.217381	244.920857	361.924502	351.275273	315.182612	250.188611	388.04668
Sypl2	1.24684571	-0.4099729	0.37629567	-1.0894967	0.27593493	NA		Sypl2	3.36023164	2.71514231	1.27231614	0	1.66481172	0	0.96226389	0
Slc26a6	2.30892594	0.44381265	0.4074489	1.08924739	0.27604481	NA		Slc26a6	1.34409266	1.81009488	1.90847421	0	4.99443516	0	3.84905555	4.56525506
Cdc42ep5	3.75242101	0.44237815	0.40614411	1.08921473	0.27605921	NA		Cdc42ep5	2.68818531	0.90504744	1.59039517	4.13628002	8.3240586	0	0.96226389	11.4131377
Rbm17	11.0263811	-0.4026444	0.36969906	-1.0891139	0.27610365	0.99938111		Rbm17	17.4732045	10.8605693	9.54237104	14.4769801	6.65924688	5.88027262	9.62263889	13.6957652
Irak3	1.04059344	-0.3940415	0.36186281	-1.0889252	0.27618686	NA		Irak3	1.34409266	1.35757116	1.27231614	2.06814001	0	0	0	2.28262753
Ptpn21	4.12201845	-0.453384	0.41636386	-1.088913	0.27619227	NA		Ptpn21	1.34409266	5.88280835	4.77118552	10.3407001	3.32962344	1.17605452	3.84905555	2.28262753
Pgm3	10.6950094	0.38402589	0.35272134	1.08875148	0.27626349	0.99938111		Pgm3	9.4086486	7.2403795	9.224292	10.3407001	14.9833055	11.7605452	13.4716944	9.13051012
G2e3	4.22194156	0.45222863	0.41543527	1.08856582	0.2763454	NA		G2e3	2.68818531	3.16766603	4.45310648	2.06814001	3.32962344	9.40843619	8.660375	0
Rnf20	31.3173204	0.35080251	0.32226814	1.08854231	0.27635577	0.99938111		Rnf20	36.9625481	21.2686148	26.4005599	22.7495401	21.6425524	55.2745626	36.5660278	29.6741579
Scnm1	10.4899618	-0.4359715	0.40052979	-1.088487	0.27638016	0.99938111		Scnm1	17.4732045	15.3858064	5.40734359	12.4088401	3.32962344	5.88027262	5.77358333	18.2610202
Wfs1	132.458214	-0.391729	0.35993572	-1.0883303	0.2764493	0.99938111		Wfs1	100.806949	234.85981	158.721438	128.224681	179.799666	111.72518	33.6792361	111.848749
Nphp4	9.4444106	0.42130131	0.387119	1.08829923	0.27646303	0.99938111		Nphp4	5.37637063	4.52523719	8.2700549	12.4088401	8.3240586	5.88027262	12.5094306	18.2610202
Nhlh2	1.07382988	-0.2794341	0.2568049	-1.0881183	0.27654288	NA		Nhlh2	0	0	0.31807903	8.27256004	0	0	0	0
Sh2d5	56.0623509	0.38244273	0.35153645	1.08791772	0.27663143	0.99938111		Sh2d5	45.6991503	32.5817078	39.7598793	68.2486203	101.553515	44.6900719	26.9433889	89.0224737
Scg3	109.803871	-0.4401148	0.40460244	-1.0877711	0.27669618	0.99938111		Scg3	149.194285	226.261859	141.227091	28.9539601	38.2906696	191.696887	73.1320555	29.6741579
Ndrg3	362.193925	-0.2466944	0.22683016	-1.087573	0.27678364	0.99938111		Ndrg3	446.910808	402.74611	459.942284	270.926341	301.330921	315.182612	387.792347	312.719972
Shb	6.81652427	0.43589136	0.40079978	1.0875539	0.27679209	0.99938111		Shb	3.36023164	2.71514231	6.99773876	8.27256004	11.653682	4.7042181	7.69811111	9.13051012
Abca8b	14.7190928	0.41523542	0.38181315	1.08753569	0.27680014	0.99938111		Abca8b	13.4409266	18.5534725	9.54237104	4.13628002	21.6425524	29.4013631	9.62263889	11.4131377
Tada2b	34.900317	0.30245123	0.27822231	1.08708473	0.27699936	0.99938111		Tada2b	32.2582238	31.6766603	37.8514051	18.6132601	39.9554813	39.9858538	42.3396111	36.5220405
Hist1h3b	2.60953898	-0.4502304	0.41416641	-1.0870762	0.27700314	NA		Hist1h3b	2.01613899	4.07271347	3.81694841	4.13628002	1.66481172	0	2.88679167	2.28262753
Pacsin1	421.307995	0.33082469	0.30435623	1.08696539	0.2770521	0.99938111		Pacsin1	314.517682	300.475749	279.273392	564.602223	634.293265	346.936085	311.7735	618.592061
Bend4	23.970138	0.38145388	0.35109767	1.08646086	0.27727515	0.99938111		Bend4	20.1613899	13.1231878	25.7644018	20.6814001	23.3073641	48.2182355	15.3962222	25.1089028
Soga3	76.2251656	0.23644732	0.21773699	1.08593087	0.27750958	0.99938111		Soga3	69.2207718	74.6664136	66.7965972	66.1804803	91.5646446	87.0280348	72.1697916	82.1745911
Lipo1	2.31237802	-0.4375633	0.40294738	-1.0859068	0.27752023	NA		Lipo1	5.37637063	2.26261859	5.40734359	0	0	3.52816357	1.92452778	0
Bsdc1	43.5953301	-0.2835281	0.26117688	-1.085579	0.2776653	0.99938111		Bsdc1	41.6668724	52.0402277	43.5768277	57.9079203	33.2962344	45.8661264	35.6037639	38.804668
Asphd1	7.70793313	0.43290838	0.39880324	1.08551872	0.27769198	0.99938111		Asphd1	6.04841696	6.33533207	4.45310648	6.20442003	14.9833055	7.05632714	2.88679167	13.6957652
Dctn1	287.267773	0.37248403	0.34318819	1.08536378	0.27776057	0.99938111		Dctn1	193.549343	155.668159	187.984709	426.036842	419.532554	231.682741	183.792403	499.895429
Proz	5.97239472	0.45100267	0.41558905	1.08521307	0.2778273	0.99938111		Proz	2.01613899	4.07271347	3.18079035	8.27256004	4.99443516	10.5844907	0.96226389	13.6957652
Krtcap3	1.44132413	0.39617245	0.36514098	1.0849849	0.27792834	NA		Krtcap3	0	0.90504744	0.31807903	2.06814001	4.99443516	0	0.96226389	2.28262753
Mob3a	13.80344	-0.4043991	0.37277997	-1.0848199	0.27800142	0.99938111		Mob3a	18.8172972	10.8605693	9.224292	28.9539601	9.98887032	10.5844907	10.5849028	11.4131377
Zbtb4	223.362176	0.3451824	0.31820651	1.0847748	0.27802141	0.99938111		Zbtb4	176.076138	139.377305	158.403359	291.607741	321.308662	166.999742	158.773542	374.350915
Pdxk	287.124594	0.38414141	0.35420393	1.08452043	0.27813411	0.99938111		Pdxk	174.732045	152.04797	184.803919	440.513822	466.147282	235.210905	166.471653	477.069154
Rasl12	0.90844266	-0.3763051	0.34699144	-1.0844794	0.27815229	NA		Rasl12	0.67204633	2.71514231	0.63615807	2.06814001	0	1.17605452	0	0
Arpc2	107.094675	-0.3097128	0.2855922	-1.084458	0.27816177	0.99938111		Arpc2	163.307258	126.706641	115.780769	74.4530404	66.5924688	87.0280348	127.018833	95.8703563
Tmcc2	119.612794	0.3908017	0.36048572	1.08409757	0.27832154	0.99938111		Tmcc2	74.5971425	85.5269829	64.888123	169.587481	248.056946	72.9153805	76.9811111	164.349182
Lrp2	0.73069975	0.31139373	0.28725358	1.0840378	0.27834804	NA		Lrp2	0	0	0.31807903	0	0	0	0.96226389	4.56525506
Ssh1	17.2690288	0.38469869	0.35492587	1.08388462	0.27841596	0.99938111		Ssh1	17.4732045	11.313093	9.86045007	20.6814001	31.6314227	18.8168724	19.2452778	9.13051012
Hmgxb3	47.6226636	0.31379331	0.2896648	1.08329803	0.27867616	0.99938111		Hmgxb3	40.3227797	36.2018975	33.0802196	57.9079203	51.6091633	39.9858538	44.2641389	77.6093361
Ngdn	6.25151007	0.42870468	0.39578736	1.0831692	0.27873333	0.99938111		Ngdn	5.37637063	5.88280835	5.40734359	2.06814001	3.32962344	8.23238167	10.5849028	9.13051012
Cav2	18.1373035	-0.412246	0.38060321	-1.0831384	0.27874699	0.99938111		Cav2	24.8657142	31.2241366	24.4920857	6.20442003	11.653682	10.5844907	26.9433889	9.13051012
Srsf9	11.8464984	0.38132143	0.35207331	1.08307396	0.2787756	0.99938111		Srsf9	12.0968339	9.5029981	8.2700549	10.3407001	13.3184938	10.5844907	19.2452778	11.4131377
Taok1	315.192746	0.33695273	0.31117174	1.08285132	0.27887442	0.99938111		Taok1	305.109033	309.526224	328.257564	140.633521	226.414394	488.062627	454.188555	269.350049
Cxxc5	64.6929419	-0.3342202	0.30867394	-1.0827614	0.27891433	0.99938111		Cxxc5	67.2046328	65.1634155	52.8011197	109.611421	69.9220923	39.9858538	37.5282917	75.3267085
Minos1	48.8928691	-0.3516788	0.32484122	-1.0826177	0.27897815	0.99938111		Minos1	61.1562159	50.6826565	55.663831	59.9760603	24.9721758	35.2816357	76.0188472	27.3915304
A430035B10Rik	0.77440732	-0.3664458	0.33851044	-1.0825243	0.27901961	NA		A430035B10R	2.01613899	2.26261859	0.9542371	0	0	0	0.96226389	0
Eif2b3	4.04071635	0.44781612	0.41392201	1.08188526	0.27930351	NA		Eif2b3	3.36023164	4.52523719	3.18079035	0	3.32962344	4.7042181	8.660375	4.56525506
Lnx1	14.9703082	0.40029362	0.37011194	1.08154745	0.27945366	0.99938111		Lnx1	12.0968339	12.6706641	18.130505	4.13628002	14.9833055	30.5774176	13.4716944	13.6957652
Zfp36l2	21.0343364	-0.4065102	0.37598551	-1.0811859	0.27961442	0.99938111		Zfp36l2	25.5377605	27.1514231	12.0870033	35.1583802	23.3073641	7.05632714	10.5849028	27.3915304
Naa38	12.5352109	-0.4060342	0.37564908	-1.0808869	0.27974741	0.99938111		Naa38	18.8172972	19.9110436	13.3593194	6.20442003	6.65924688	5.88027262	13.4716944	15.9783927
Cog7	37.8200858	0.32105533	0.29708822	1.0806734	0.27984242	0.99938111		Cog7	35.6184554	19.0059962	33.7163777	45.4990802	44.9499164	39.9858538	40.4150833	43.3699231
H2-Ke2	14.9967397	0.41955066	0.38825998	1.08059208	0.27987861	0.99938111		H2-Ke2	16.8011582	3.62018975	9.86045007	18.6132601	28.3017992	11.7605452	17.32075	13.6957652
Arf3	2124.45037	0.43861144	0.40593663	1.08049238	0.27992298	0.99938111		Arf3	1122.98941	887.851537	1014.67212	3400.02218	3898.98905	624.484952	926.660125	5119.93355
Zscan25	7.98622098	0.4489486	0.41553251	1.08041752	0.2799563	0.99938111		Zscan25	4.03227797	2.26261859	5.08926455	12.4088401	18.3129289	9.40843619	0.96226389	11.4131377
Ankfy1	39.1587831	0.28994561	0.26838993	1.08031477	0.28000204	0.99938111		Ankfy1	33.6023164	36.6544212	38.8056422	28.9539601	53.2739751	50.5703445	39.4528194	31.9567854
Gadd45gip1	38.4049985	-0.3414838	0.31617418	-1.0800497	0.28012005	0.99938111		Gadd45gip1	41.6668724	29.4140417	37.5333261	68.2486203	38.2906696	21.1689814	29.8301805	41.0872956
Mrto4	5.14594275	0.44637207	0.41329553	1.08003122	0.28012828	0.99938111		Mrto4	3.36023164	2.26261859	2.22655324	8.27256004	8.3240586	4.7042181	2.88679167	9.13051012
Map3k10	107.611101	0.39650424	0.36717556	1.07987647	0.28019719	0.99938111		Map3k10	81.3176057	84.6219354	66.7965972	117.883981	226.414394	59.9787807	48.1131944	175.76232
Tmem141	7.04694037	-0.4383822	0.40604803	-1.0796313	0.28030638	0.99938111		Tmem141	7.39250961	4.52523719	8.90621297	14.4769801	4.99443516	1.17605452	5.77358333	9.13051012
Fam184b	3.53612188	0.44918417	0.41606907	1.07959038	0.28032463	NA		Fam184b	3.36023164	3.16766603	1.90847421	0	6.65924688	1.17605452	2.88679167	9.13051012
2610005L07Rik	7.7553452	0.4267681	0.39534928	1.07947104	0.2803778	0.99938111		2610005L07Ri	8.06455594	7.2403795	3.81694841	6.20442003	4.99443516	11.7605452	15.3962222	4.56525506

GeneID	Base mean	log2(FC)	StdErr	Wald-Stats	P-value	P-adj	GeneID	Normalised expression for Chow#1	Normalised expression for Chow#2	Normalised expression for Chow#3	Normalised expression for Chow#4	Normalised expression for HFD#1	Normalised expression for HFD#2	Normalised expression for HFD#3	Normalised expression for HFD#4
Abcb8	27.5158328	0.38371988	0.35547841	1.07944637	0.28038879	0.99938111	Abcb8	18.8172972	20.8160911	18.130505	33.0902402	39.9554813	15.2887088	19.2452778	54.7830607
Hn1l	4.86091333	-0.4481946	0.41531037	-1.0791798	0.28050756	0.99938111	Hn1l	5.37637063	4.97776091	3.18079035	12.4088401	4.99443516	4.7042181	0.96226389	2.28262753
Bola3	5.70070185	-0.4403024	0.40801124	-1.0791428	0.28052409	0.99938111	Bola3	10.0806949	5.88280835	5.08926455	8.27256004	1.66481172	2.35210905	7.69811111	4.56525506
Fam107b	6.00684852	-0.4362278	0.40424623	-1.079114	0.28053691	0.99938111	Fam107b	10.7527413	6.78785578	6.99773876	6.20442003	1.66481172	4.7042181	8.660375	2.28262753
Sod2	183.605516	0.24539619	0.22745372	1.07888406	0.28063942	0.99938111	Sod2	162.635211	148.880304	178.760417	179.928181	191.453348	192.872942	263.660305	150.653417
Bphl	10.4265703	-0.4156494	0.38541536	-1.0784453	0.28083508	0.99938111	Bphl	11.4247876	7.69290322	14.6316356	18.6132601	4.99443516	7.05632714	14.4339583	4.56525506
Stat6	2.87925676	-0.4489009	0.41625582	-1.0784256	0.28084387	NA	Stat6	4.7043243	3.62018975	3.18079035	4.13628002	3.32962344	1.17605452	2.88679167	0
Ddx56	18.3657309	0.35587153	0.33002706	1.07831016	0.28089536	0.99938111	Ddx56	16.1291119	14.480759	15.5858727	14.4769801	14.9833055	16.4647633	18.2830139	36.5220405
Car7	6.00452494	-0.4422076	0.41012835	-1.0782177	0.28093661	0.99938111	Car7	9.4086486	5.43028463	5.40734359	10.3407001	8.3240586	5.88027262	0.96226389	2.28262753
Anapc5	71.715192	-0.262757	0.24369859	-1.0782048	0.28094239	0.99938111	Anapc5	82.6616984	71.9512713	81.7463119	80.6574604	43.2851047	74.091435	73.1320555	66.1961984
Hspa1b	8.28293251	0.4472832	0.41487229	1.07812262	0.28097904	0.99938111	Hspa1b	4.7043243	7.2403795	6.67965972	4.13628002	26.6369875	3.52816357	1.92452778	11.4131377
Ksr1	31.0529837	0.34833054	0.32313175	1.07798302	0.28104133	0.99938111	Ksr1	22.1775288	25.793852	24.1740066	33.0902402	48.2795399	23.5210905	21.1698055	50.2178057
Gli3	5.38987082	-0.4488344	0.41637077	-1.0779682	0.28104797	0.99938111	Gli3	10.0806949	9.05047438	7.95197586	0	6.65924688	0	4.81131944	4.56525506
Man2b1	14.7147547	-0.3969614	0.36835111	-1.0776712	0.28118053	0.99938111	Man2b1	14.7850192	22.6261859	20.0389792	12.4088401	18.3129289	17.6408179	9.62263889	2.28262753
Vash2	3.02901696	0.43644732	0.40502987	1.07756822	0.28122649	NA	Vash2	3.36023164	0	2.22655324	2.06814001	0	5.88027262	3.84905555	6.84788259
Sema4b	10.9506889	-0.438935	0.40740191	-1.0774004	0.28130143	0.99938111	Sema4b	14.7850192	20.8160911	10.4966081	8.27256004	19.9777406	9.40843619	3.84905555	0
Gm6251	12.5006176	-0.446154	0.41410805	-1.0773856	0.28130804	0.99938111	Gm6251	28.8979921	9.05047438	18.448584	8.27256004	3.32962344	4.7042181	25.0188611	2.28262753
Chrm1	122.910509	-0.3496492	0.32457249	-1.0772608	0.28136379	0.99938111	Chrm1	125.000617	214.948766	150.769462	70.3167603	146.503431	108.197016	62.5471528	105.000866
Gm1604b	7.65451564	0.44813771	0.4160313	1.07717307	0.28140295	0.99938111	Gm1604b	6.72046328	3.62018975	7.63389683	2.06814001	14.9833055	4.7042181	0.96226389	20.5436478
Fxyd2	4.63902455	-0.4478645	0.41584126	-1.0770083	0.28147657	NA	Fxyd2	6.04841696	4.97776091	1.59039517	12.4088401	0	2.35210905	2.88679167	6.84788259
Icam4	1.10213191	0.3517327	0.32665998	1.07675478	0.28158983	NA	Icam4	0.67204633	0.45252372	0.63615807	0	0	7.05632714	0	0
Ggta1	0.95736945	-0.3380011	0.31391867	-1.0767155	0.28160739	NA	Ggta1	0	3.62018975	2.86271131	0	0	1.17605452	0	0
Kirrel2	2.59517922	-0.4258741	0.39559786	-1.0765328	0.28168902	NA	Kirrel2	2.01613899	7.69290322	4.13502745	0	0	2.35210905	0	4.56525506
Tpm4	8.18490813	-0.4189586	0.38937376	-1.0759806	0.28193592	0.99938111	Tpm4	11.4247876	10.4080455	12.7231614	4.13628002	3.32962344	4.7042181	9.62263889	9.13051012
Son	151.203277	0.23480122	0.2183736	1.07522713	0.28227305	0.99938111	Son	143.817914	133.947021	122.778507	153.042361	169.810795	201.105324	129.905625	155.218672
Map3k13	28.9108297	0.33975481	0.31606348	1.07495753	0.28239374	0.99938111	Map3k13	24.1936678	26.6988994	29.5813502	18.6132601	18.3129289	47.042181	39.4528194	27.3915304
Isca1	118.926927	-0.2843902	0.26456486	-1.0749357	0.28240353	0.99938111	Isca1	118.9522	129.421784	150.133304	132.360961	73.2517157	104.668853	155.88675	86.7398462
Zfp597	22.4733883	0.33907539	0.31545916	1.07486305	0.28243605	0.99938111	Zfp597	20.8334362	24.4362808	19.7209001	10.3407001	26.6369875	19.9929269	32.7169722	25.1089028
Efhc1	0.64117481	0.27695123	0.25775582	1.07447128	0.28261151	NA	Efhc1	0	0	0.31807903	0	0	0	4.81131944	0
Maats1	1.09035044	0.39876964	0.37113674	1.07445476	0.28261891	NA	Maats1	0.67204633	0.45252372	0.9542371	0	3.32962344	2.35210905	0.96226389	0
Abca4	0.82577026	-0.3604231	0.33545911	-1.0744174	0.28263563	NA	Abca4	0.67204633	3.16766603	1.59039517	0	0	1.17605452	0	0
Gm2694	1.1159265	-0.3607712	0.33582024	-1.0742986	0.28268885	NA	Gm2694	0.67204633	0.45252372	0.63615807	6.20442003	0	0	0.96226389	0
Acaca	22.5894901	0.34470752	0.32096057	1.07398713	0.28282845	0.99938111	Acaca	18.8172972	25.793852	19.0847421	12.4088401	34.9610461	30.5774176	23.0943333	15.9783927
Reps2	262.911158	0.34284183	0.31927999	1.0737968	0.28291376	0.99938111	Reps2	241.936678	257.033472	286.58921	113.747701	209.766277	398.682484	406.075361	189.458085
Dynlt1c	2.70111815	-0.4432652	0.41280572	-1.0737866	0.28291834	NA	Dynlt1c	4.7043243	1.81009488	2.54463228	6.20442003	0	1.17605452	2.88679167	2.28262753
Camsap1	80.7361239	0.29438824	0.27419839	1.07363227	0.28298753	0.99938111	Camsap1	70.5648645	58.3755597	70.9316247	88.9300204	113.207197	110.549125	80.8301666	52.5004332
Pcm1	94.7787769	0.4104179	0.3823072	1.07352909	0.2830338	0.99938111	Pcm1	100.806949	81.4542694	91.2886829	28.9539601	49.9443516	216.394032	141.452792	47.9351782
Hspe1	16.7750638	-0.4202891	0.39154013	-1.0734253	0.28308034	0.99938111	Hspe1	26.8818531	20.8160911	22.5836114	12.4088401	9.98887032	9.40843619	29.8301805	2.28262753
Naa30	35.9409147	0.3089446	0.28784204	1.07331299	0.28313071	0.99938111	Naa30	32.9302701	31.6766603	33.3982986	28.9539601	24.9721758	49.39429	51.96225	34.239413
Mrps27	12.568955	0.36785341	0.34280342	1.07307395	0.28323794	0.99938111	Mrps27	10.7527413	7.69290322	11.7689243	12.4088401	16.6481172	14.1126543	13.4716944	13.6957652
Zbtb20	22.6140356	-0.4090628	0.38121305	-1.0730556	0.28324618	0.99938111	Zbtb20	18.8172972	28.0564706	22.2655324	41.3628002	31.6314227	8.23238167	25.981125	4.56525506
Sqle	32.0381025	-0.3588016	0.3343798	-1.073036	0.28325497	0.99938111	Sqle	25.5377605	56.5654649	42.3045116	22.7495401	33.2962344	32.9295267	26.9433889	15.9783927
Dbn1	103.195903	0.42770307	0.39861819	1.07296424	0.28328716	0.99938111	Dbn1	72.5810035	58.8280835	58.2084633	130.292821	278.023557	31.7534722	45.2264028	150.653417
Arsa	17.4841964	-0.4052483	0.3777109	-1.0729061	0.28331323	0.99938111	Arsa	8.73660227	31.2241366	20.3570582	22.7495401	24.9721758	11.7605452	8.660375	11.4131377
Diras1	408.669596	0.44022712	0.41033211	1.07285564	0.28333589	0.99938111	Diras1	142.473822	140.734877	194.664369	740.394124	502.77314	162.295524	187.641458	1198.37945
Grik2	52.8877153	0.36273871	0.33816568	1.07266564	0.28342116	0.99938111	Grik2	33.6023164	65.6159392	54.7095939	22.7495401	43.2851047	98.78858	58.6980972	45.6525506
Reck	2.47229945	-0.4446838	0.4145855	-1.0725986	0.28345126	NA	Reck	3.36023164	4.52523719	3.18079035	2.06814001	3.32962344	2.35210905	0.96226389	0
Adprm	6.82258614	-0.4183015	0.39021675	-1.0719721	0.28373257	0.99938111	Adprm	6.72046328	8.14542694	8.58813393	10.3407001	4.99443516	3.52816357	7.69811111	4.56525506
Nutf2-ps1	6.52432528	-0.4378826	0.40849926	-1.0719299	0.28375153	0.99938111	Nutf2-ps1	4.7043243	9.5029981	11.7689243	6.20442003	3.32962344	1.17605452	8.660375	6.84788259
Dnah1	10.0300051	-0.3984464	0.37171782	-1.0719055	0.28376247	0.99938111	Dnah1	10.0806949	9.5029981	10.1785291	18.6132601	6.65924688	7.05632714	6.73584722	11.4131377
1810014B01Rik	1.16854557	-0.3732942	0.34830632	-1.071741	0.28383639	NA	1810014B01Ri	0.67204633	4.52523719	2.22655324	0	0	0	1.92452778	0
4930412C18Rik	0.60686022	-0.3270785	0.30525058	-1.0715081	0.28394102	NA	4930412C18Ri	2.01613899	0.45252372	0.31807903	2.06814001	0	0	0	0
Tmem131	55.8854538	-0.4144799	0.38682199	-1.0715004	0.2839445	0.99938111	Tmem131	51.7475673	118.561214	66.4785182	33.0902402	34.9610461	96.436471	29.8301805	15.9783927
Neu3	1.78616247	-0.4306522	0.40192434	-1.0714758	0.28395554	NA	Neu3	3.36023164	3.16766603	1.27231614	2.06814001	0	1.17605452	0.96226389	2.28262753
Gins4	5.74887635	-0.4309234	0.40221828	-1.071367	0.28400443	0.99938111	Gins4	4.7043243	7.2403795	6.99773876	10.3407001	1.66481172	4.7042181	5.77358333	4.56525506
Ipo5	56.2709905	0.30629173	0.28598823	1.07099417	0.28417205	0.99938111	Ipo5	65.1884939	44.7998482	48.3480132	39.2946602	79.9109626	76.4435441	59.6603611	36.5220405
Cript	22.3402005	-0.4183467	0.39061706	-1.0709893	0.28417426	0.99938111	Cript	37.6345944	35.7493738	28.9451921	6.20442003	9.98887032	23.5210905	29.8301805	6.84788259
Fam32a	61.8457209	0.31826607	0.29720401	1.07086735	0.28422908	0.99938111	Fam32a	43.010965	40.7271347	48.9841713	86.8618804	48.2795399	62.3308898	84.6792222	79.8919636
Hcrtr2	3.51298933	0.4428305	0.41354198	1.07082356	0.28424877	NA	Hcrtr2	2.68818531	4.07271347	1.59039517	0	0	3.52816357	4.81131944	11.4131377
Lemd3	26.229689	0.38700868	0.36142068	1.07079839	0.28426009	0.99938111	Lemd3	18.8172972	29.8665654	24.4920857	12.4088401	28.3017992	58.8027262	21.1698055	15.9783927
Cep250	9.98086329	0.40582448	0.37900974	1.07074946	0.2842821	0.99938111	Cep250	9.4086486	5.43028463	5.40734359	14.4769801	11.653682	14.1126543	12.5094306	6.84788259
Hic1	2.46059605	-0.4406823	0.41163185	-1.0705738	0.28436112	NA	Hic1	4.03227797	2.71514231	2.22655324	4.13628002	3.32962344	0	0.96226389	2.28262753
Hcn3	21.7650845	0.37383723	0.34933029	1.07015406	0.28454997	0.99938111	Hcn3	14.7850192	19.0059962	15.2677937	22.7495401	34.9610461	14.1126543	14.4339583	38.804668
B3gnt2	15.2689932	-0.4061418	0.37954571	-1.0700735	0.28458621	0.99938111	B3gnt2	17.4732045	23.9837571	20.9932163	10.3407001	6.65924688	25.8731995	7.69811111	9.13051012
Ube2t	0.50738223	-0.3086813	0.28848898	-1.0699935	0.28462225	NA	Ube2t	2.01613899	0.45252372	1.59039517	0	0	0	0	0
Klf10	16.2685395	-0.4249754	0.3971932	-1.0699464	0.28464342	0.99938111	Klf10	36.9625481	14.9332827	24.1740066	2.06814001	16.6481172	15.2887088	8.660375	11.4131377
Dpf3	2.48638216	0.4410666	0.41225256	1.06989415	0.28466696	NA	Dpf3	0.67204633	1.81009488	1.90847421	2.06814001	6.65924688	3.52816357	0.96226389	2.28262753
Zfp606	9.42777666	0.40264117	0.37648651	1.06947039	0.28485776	0.99938111	Zfp606	8.06455594	8.14542694	9.86045007	4.13628002	8.3240586	11.7605452	18.2830139	6.84788259
Flcn	29.1501201	0.36746441	0.34380995	1.06880096	0.28515937	0.99938111	Flcn	23.5216215	17.1959013	23.5378486	33.0902402	41.620293	21.1689814	18.2830139	54.7830607
9330151L19Rik	17.0935698	0.39408709	0.36872811	1.0687742	0.28517143	0.99938111	9330151L19Ri	16.8011582	12.2181404	9.54237104	16.5451201	28.3017992	11.7605452	9.62263889	31.9567854
Tada1	24.0247199	0.32509493	0.30427405	1.06842804	0.28532747	0.99938111	Tada1	18.8172972	25.3413283	24.1740066	12.4088401	24.9721758	24.697145	29.8301805	31.9567854
Irgq	151.368834	0.38799776	0.36315782	1.06839985	0.28534018	0.99938111	Irgq	96.102625	89.5996963	87.7898135	225.427261	274.693934	82.3238167	103.9245	251.089028
Chtf8	69.969726	-0.27481	0.25724126	-1.0682968	0.28538665	0.99938111	Chtf8	70.5648645	78.2866034	67.4327553	93.0663005	66.5924688	42.3379629	61.5848889	79.8919636
Mum1	15.5386456	0.41583607	0.38925151	1.06829661	0.28538674	0.99938111	Mum1	13.4409266	8.59795066	10.8146872	16.5451201	31.6314227	21.1689814	3.84905555	18.2610202
Rspry1	24.8009434	0.35415133	0.33153353	1.06822177	0.28542049	0.99938111	Rspry1	20.8334362	27.1514231	21.9474534	14.4769801	26.6369875	29.4013631	44.2641389	13.6957652
Pomt2	12.8585672	-0.3807833	0.35657845	-1.0678808	0.28557429	0.99938111	Pomt2	10.0806949	14.480759	16.2220308	20.6814001	8.3240586	15.2887088	8.660375	9.13051012
Tanc1	21.3418216	0.38918365	0.3644675	1.06781442	0.28560423	0.99938111	Tanc1	19.4893435	26.6988994	17.4943469	4.13628002	38.2906696	29.4013631	19.2452778	15.9783927
Zfp651	58.6794528	0.39479491	0.369743	1.06775493	0.28563107	0.99938111	Zfp651	48.3873356	34.8443264	28.6271131	80.6574604	119.866444	44.6900719	27.9056528	84.4572186
Tmc7	7.02431807	-0.4218484	0.39510066	-1.0676986	0.2856565	0.99938111	Tmc7	6.04841696	12.6706641	9.224292	6.20442003	8.3240586	4.7042181	6.73584722	2.28262753
Tjp1	50.192097	-0.4049804	0.37933652	-1.0676021	0.28570006	0.99938111	Tjp1	75.9412351	87.3370778	62.3434908	14.4769801	43.2851047	50.5703445	53.8867778	13.6957652
Commd9	12.3785292	-0.4369212	0.40926644	-1.0675716	0.28571382	0.99938111	Commd9	14.7850192	9.5029981	11.1327662	26.8858201	16.6481172	0	8.660375	11.4131377
Trappc10	117.509596	0.26855675	0.25165874	1.06714652	0.28590568	0.99938111	Trappc10	79.9735131	99.5552182	95.7417894	148.906081	141.508996	121.133616	104.886764	148.37079
Angptl6	4.57228508	0.43963182	0.41198632	1.06710297	0.28592534	NA	Angptl6	2.01613899	2.71514231	4.77118552	4.13628002	8.3240586	2.35210905	7.69811111	4.56525506
Mapk7	15.0072976	-0.4180076	0.39177671	-1.0669536	0.28599279	0.99938111	Mapk7	17.4732045	13.1231878	14.6316356	26.8858201	18.3129289	2.35210905	6.73584722	20.5436478
Plgrkt	2.99114142	-0.4442391	0.41637022	-1.0669329	0.28600213	NA	Plgrkt	4.03227797	4.97776091	4.77118552	2.06814001	1.66481172	3.52816357	2.88679167	0
Alkbh3	3.21887964	0.44400959	0.41622246	1.06676028	0.2860801	NA	Alkbh3	2.01613899	2.26261859	1.59039517	4.13628002	1.66481172	4.7042181	4.81131944	4.56525506
Capn1	10.5020477	0.43100632	0.40413649	1.06648702	0.28620355	0.99938111	Capn1	12.7688802	8.14542694	7.31581779	2.06814001	18.3129289	2.35210905	12.5094306	20.5436478
Rbm42	41.9114172	0.4106415	0.38505853	1.06643918	0.28622516	0.99938111	Rbm42	36.2905017	18.1009488	20.0389792	59.9760603	59.9332219	22.345036	25.0188611	93.5877288
Nfatc2	20.7375	-0.4439042	0.41629378	-1.0663244	0.28627702	0.99938111	Nfatc2	14.1129729	12.6706641	15.5858727	66.1804803	19.9777406	0	7.69811111	29.6741579
Dnmt3a	43.8949284	0.38214974	0.3585177	1.06591597	0.28646163	0.99938111	Dnmt3a	26.8818531	26.2463757	24.8101647	70.3167603	89.8998329	37.6337448	36.5660278	38.804668
Rragd	126.328973	-0.2955934	0.27732788	-1.0658627	0.28648573	0.99938111	Rragd	165.323397	149.332827	151.087541	99.2707205	108.212762	149.358925	124.132042	63.9135709
Bcl7b	35.7119073	-0.3615888	0.33938629	-1.0654195	0.28668611	0.99938111	Bcl7b	42.3389187	36.6544212	37.215247	47.5672202	29.966611	10.5844907	28.8679167	52.5004332
Hdc	0.77073719	-0.3049496	0.28622924	-1.0654032	0.28669349	NA	Hdc	0.67204633	1.35757116	0	4.13628002	0	0	0	0
Lama1	2.17783403	-0.4350877	0.40852647	-1.0650171	0.28686817	NA	Lama1	2.68818531	3.16766603	3.81694841	2.06814001	3.32962344	2.35210905	0	0
Obfc1	4.48885856	-0.4432096	0.41624076	-1.0647913	0.28697037	NA	Obfc1	9.4086486	8.14542694	5.72542262	0	3.32962344	3.52816357	5.77358333	0
Snord104	1.29258751	-0.2470024	0.23199672	-1.0646806	0.2870205	NA	Snord104	0	0	0	10.3407001	0	0	0	0
Ccdc60	0.76456634	-0.3085223	0.28990055	-1.0642349	0.28722229	NA	Ccdc60	1.34409266	0	0.63615807	4.13628002	0	0	0	0
Tmie	7.76086152	-0.4264527	0.40082004	-1.0639506	0.28735109	0.99938111	Tmie	8.73660227	6.78785578	6.36158069	16.5451201	8.3240586	2.35210905	3.84905555	9.13051012
B3gat1	87.3827162	-0.2914893	0.2740294	-1.0637155	0.28745761	0.99938111	B3gat1	86.02193	137.114687	98.9225797	66.1804803	91.5646446	82.3238167	79.8679027	57.0656883
Ttll12	17.4396506	0.38031634	0.35757884	1.06358735	0.28751568	0.99938111	Ttll12	18.1452509	14.0282353	13.0412404	10.3407001	16.6481172	21.1689814	9.62263889	36.5220405
Pomk	22.1085902	-0.3528537	0.33184628	-1.0633046	0.28764382	0.99938111	Pomk	22.1775288	36.2018975	25.4463228	16.5451201	11.653682	24.697145	17.32075	22.8262753
Hpcal4	810.435885	0.24365164	0.22916687	1.06320624	0.28768843	0.99938111	Hpcal4	819.896521	750.284326	756.391944	614.237583	1188.67557	653.886315	830.433736	869.681089
Pdcl3	12.1960027	0.40228715	0.37837821	1.06318792	0.28769674	0.99938111	Pdcl3	4.03227797	10.4080455	12.0870033	14.4769801	11.653682	18.8168724	19.2452778	6.84788259
Tigd5	5.90480141	-0.4423985	0.41612367	-1.0631419	0.28771761	0.99938111	Tigd5	4.7043243	3.16766603	3.81694841	20.6814001	4.99443516	4.7042181	2.88679167	2.28262753
Cox6b1	131.08941	-0.3442369	0.32396434	-1.0625764	0.2879741	0.99938111	Cox6b1	153.226563	126.706641	127.867772	192.337021	71.586904	57.6266717	129.905625	189.458085
Fyb	2.92436984	-0.4191986	0.39451208	-1.0625749	0.28797479	NA	Fyb	1.34409266	3.62018975	3.49886938	8.27256004	6.65924688	0	0	0
Mettl21a	6.75601023	0.43452502	0.4089686	1.06248992	0.28801333	0.99938111	Mettl21a	10.7527413	3.62018975	4.77118552	0	4.99443516	11.7605452	6.73584722	11.4131377
Stox1	2.51198155	0.4351523	0.4095854	1.06242143	0.28804441	NA	Stox1	1.34409266	2.71514231	0.63615807	2.06814001	0	5.88027262	2.88679167	4.56525506
Usp27x	18.0755354	0.3360538	0.31635272	1.06227565	0.28811057	0.99938111	Usp27x	15.4570656	18.1009488	13.3593194	14.4769801	21.6425524	15.2887088	21.1698055	25.1089028
Zfp811	7.22859185	0.43064945	0.40548638	1.06205652	0.28821003	0.99938111	Zfp811	8.06455594	7.2403795	6.04350166	0	8.3240586	15.2887088	10.5849028	2.28262753
Snx30	35.2276306	0.28068115	0.26432698	1.06187098	0.28829426	0.99938111	Snx30	26.8818531	31.2241366	33.7163777	35.1583802	34.9610461	42.3379629	43.301875	34.239413
Nras	31.8889971	-0.3952761	0.37227279	-1.0617915	0.28833037	0.99938111	Nras	36.2905017	61.0907021	43.2587487	10.3407001	19.9777406	32.9295267	37.5282917	13.6957652

GeneID	Base mean	log2(FC)	StdErr	Wald-Stats	P-value	P-adj		GeneID	Normalised expression for Chow#1	Normalised expression for Chow#2	Normalised expression for Chow#3	Normalised expression for Chow#4	Normalised expression for HFD#1	Normalised expression for HFD#2	Normalised expression for HFD#3	Normalised expression for HFD#4
Nos3	2.06687553	0.37709819	0.35531628	1.06130289	0.28855227	NA		Nos3	0.67204633	2.71514231	0.31807903	0	11.653682	1.17605452	0	0
Arhgef7	113.912577	0.21268501	0.2004258	1.06116579	0.28861457	0.99938111		Arhgef7	102.823088	100.912789	111.327662	105.475141	111.542385	116.429398	139.528264	123.261887
Ccl27a	17.4352832	-0.3936659	0.3711287	-1.0607262	0.28881434	0.99938111		Ccl27a	22.1775288	20.3635674	16.2220308	22.7495401	11.653682	3.52816357	15.3962222	27.3915304
Nfatc3	10.8825797	-0.3952831	0.3727095	-1.0605662	0.28888711	0.99938111		Nfatc3	13.4409266	17.1959013	13.3593194	8.27256004	4.99443516	11.7605452	13.4716944	4.56525506
Gm16793	1.20791022	0.33922909	0.31987494	1.06050536	0.28891475	NA		Gm16793	0	0	0.31807903	2.06814001	4.99443516	0	0	2.28262753
Gm11128	0.47994191	-0.3088833	0.29157609	-1.0593576	0.28943695	NA		Gm11128	1.34409266	0.90504744	1.59039517	0	0	0	0	0
Prrc1	29.0287501	0.42349589	0.3998463	1.0591467	0.28953297	0.99938111		Prrc1	13.4409266	18.1009488	15.5858727	43.4309402	53.2739751	12.9365998	11.5471667	63.9135709
Zdhhc16	10.3449694	-0.4197132	0.39633916	-1.0589749	0.28961119	0.99938111		Zdhhc16	14.7850192	16.2908539	13.0412404	6.20442003	0	11.7605452	11.5471667	9.13051012
Atg9b	6.74233634	-0.4354945	0.41134505	-1.0587086	0.28973253	0.99938111		Atg9b	4.7043243	9.95552182	5.40734359	14.4769801	0	3.52816357	6.73584722	9.13051012
Pcsk5	7.3971852	0.43179459	0.40786644	1.05866661	0.28975164	0.99938111		Pcsk5	6.04841696	9.5029981	5.72542262	0	14.9833055	12.9365998	7.69811111	2.28262753
Met	3.76806419	0.43929291	0.41497811	1.05859299	0.28978518	NA		Met	4.03227797	3.62018975	2.54463228	0	1.66481172	10.5844907	7.69811111	0
Rnd2	22.3385041	0.43189052	0.40798907	1.05858356	0.28978948	0.99938111		Rnd2	12.0968339	6.78785578	8.90621297	41.3628002	29.966611	9.40843619	15.3962222	54.7830607
Itpripl2	4.0022453	-0.4396314	0.41533372	-1.0585016	0.28982681	NA		Itpripl2	4.03227797	8.14542694	4.13502745	4.13628002	8.3240586	0	0.96226389	2.28262753
Zfp760	5.39325224	-0.4312453	0.40748139	-1.058319	0.28991003	0.99938111		Zfp760	5.37637063	8.59795066	5.72542262	8.27256004	1.66481172	3.52816357	7.69811111	2.28262753
Meis1	7.87586914	-0.4313772	0.40760958	-1.0583098	0.28991423	0.99938111		Meis1	4.7043243	16.2908539	8.58813393	8.27256004	3.32962344	2.35210905	5.77358333	13.6957652
Orc2	19.1487089	-0.4001527	0.37813954	-1.0582144	0.2899577	0.99938111		Orc2	25.5377605	21.2686148	28.9451921	16.5451201	3.32962344	28.2253086	20.2075417	9.13051012
Grip2	34.997579	0.38870153	0.36731866	1.0582134	0.28995816	0.99938111		Grip2	18.8172972	18.1009488	15.5858727	66.1804803	41.620293	39.9858538	31.7547083	47.9351782
Gpr157	1.97140452	0.40794068	0.38551788	1.0581628	0.28998123	NA		Gpr157	0.67204633	0.45252372	2.54463228	0	3.32962344	0	1.92452778	6.84788259
Lhfpl2	3.84715394	-0.4405878	0.41637068	-1.0581623	0.28998146	NA		Lhfpl2	7.39250961	7.69290322	4.77118552	0	3.32962344	4.7042181	2.88679167	0
Nim1k	24.7391786	-0.3548302	0.33533021	-1.0581516	0.28998633	0.99938111		Nim1k	36.2905017	29.8665654	21.6293743	24.8176801	28.3017992	9.40843619	20.2075417	27.3915304
Gnaq	300.937775	0.21669908	0.20479109	1.05814699	0.28998843	0.99938111		Gnaq	237.9044	273.77685	269.412942	326.766122	299.66611	292.837576	303.113125	404.025073
Lrp5	4.80532517	-0.4347237	0.41085392	-1.058098	0.29001075	0.99938111		Lrp5	3.36023164	9.05047438	6.99773876	6.20442003	11.653682	1.17605452	0	0
Cirbp	55.4527582	0.39063585	0.36919358	1.05807865	0.29001959	0.99938111		Cirbp	38.3066407	21.7211385	32.4440615	90.9981605	78.2461509	35.2816357	46.1886666	100.435611
Fnta	40.1206882	-0.3576285	0.33811235	-1.0577209	0.29018271	0.99938111		Fnta	55.1077989	43.8948007	46.7576181	41.3628002	11.653682	47.042181	50.0377222	25.1089028
Flna	6.94964957	0.43821648	0.41442138	1.05741764	0.29032102	0.99938111		Flna	2.68818531	6.78785578	4.45310648	6.20442003	23.3073641	4.7042181	2.88679167	4.56525506
Ears2	3.9830472	0.43513832	0.41163288	1.05710294	0.29046461	NA		Ears2	2.68818531	3.16766603	3.49886938	2.06814001	3.32962344	3.52816357	6.73584722	6.84788259
Olfm1	528.749596	-0.4045153	0.38278688	-1.0567638	0.29061938	0.99938111		Olfm1	656.589263	1035.82679	713.769353	128.224681	396.225189	636.245498	459.962139	203.15385
Tenm4	102.165613	0.32456746	0.30714771	1.05671458	0.29064187	0.99938111		Tenm4	86.02193	108.153169	88.1078926	70.3167603	134.849749	171.703961	57.7358333	100.435611
Pomp	29.4149669	-0.3646179	0.34509817	-1.0565628	0.29071116	0.99938111		Pomp	34.9464091	27.6039469	25.1282437	49.6353602	16.6481172	11.7605452	30.7924444	38.804668
Sugp2	48.896836	0.32656919	0.30909685	1.05652709	0.29072747	0.99938111		Sugp2	36.2905017	35.2968501	33.7163777	66.1804803	74.9165274	51.7463991	33.6792361	59.3483158
Mrps18b	8.23835115	-0.4195291	0.3970856	-1.0565206	0.29073044	0.99938111		Mrps18b	14.7850192	5.88280835	10.1785291	10.3407001	8.3240586	3.52816357	10.5849028	2.28262753
Tyms	0.5762458	0.29838923	0.28250767	1.05621639	0.29086937	NA		Tyms	0	0	0.31807903	0	3.32962344	0	0.96226389	0
Csrnp1	6.67400903	0.42718271	0.40446606	1.05616452	0.29089306	0.99938111		Csrnp1	4.03227797	4.97776091	4.13502745	8.27256004	16.6481172	8.23238167	4.81131944	2.28262753
Nbas	23.1346758	0.38240148	0.36230043	1.0554817	0.29120507	0.99938111		Nbas	15.4570656	17.1959013	17.1762679	28.9539601	38.2906696	39.9858538	21.1698055	6.84788259
Gm16702	7.58358129	0.40582239	0.38451417	1.05541596	0.29123513	0.99938111		Gm16702	5.37637063	8.14542694	6.99773876	2.06814001	9.98887032	7.05632714	9.62263889	11.4131377
Nek9	32.1404502	-0.3261897	0.3090817	-1.0553511	0.29126476	0.99938111		Nek9	29.5700384	31.2241366	29.2632712	57.9079203	33.2962344	21.1689814	25.0188611	29.6741579
Chkb	7.59986866	0.40638578	0.3850825	1.05532134	0.29127838	0.99938111		Chkb	4.03227797	5.88280835	5.08926455	10.3407001	8.3240586	7.05632714	8.660375	11.4131377
AI597479	31.26787	0.31797561	0.30131127	1.05530605	0.29128538	0.99938111		AI597479	30.2420848	26.6988994	31.1717454	20.6814001	31.6314227	29.4013631	52.9245139	27.3915304
Mfap1a	26.5410786	0.3295012	0.31224045	1.05528032	0.29129714	0.99938111		Mfap1a	26.8818531	21.7211385	18.766663	26.8858201	19.9777406	32.9295267	42.3396111	22.8262753
Ccdc90b	9.00697775	-0.4164887	0.39470312	-1.0551947	0.29133628	0.99938111		Ccdc90b	10.0806949	11.7656167	13.0412404	10.3407001	1.66481172	9.40843619	13.4716944	2.28262753
Hadha	26.1940287	-0.3068738	0.29088526	-1.0549652	0.29144122	0.99938111		Hadha	36.2905017	26.2463757	27.0367179	28.9539601	24.9721758	24.697145	23.0943333	18.2610202
Cbln1	10.6830157	-0.4292922	0.40707358	-1.0545812	0.29161689	0.99938111		Cbln1	6.72046328	9.05047438	6.04350166	33.0902402	9.98887032	4.7042181	6.73584722	9.13051012
Ank3	111.030275	0.32954246	0.31264527	1.05404591	0.2918619	0.99938111		Ank3	109.543552	104.532979	104.648002	64.1123403	144.83862	194.048996	104.886764	61.6309433
Gtpbp2	27.2602406	0.40133957	0.38077169	1.05401629	0.29187546	0.99938111		Gtpbp2	11.4247876	19.9110436	16.8581888	41.3628002	59.9332219	12.9365998	25.981125	29.6741579
Taf1b	7.48976673	-0.4351863	0.41322462	-1.053147	0.29227362	0.99938111		Taf1b	16.8011582	10.4080455	8.90621297	2.06814001	6.65924688	3.52816357	11.5471667	0
Abhd11	12.3764457	-0.373318	0.35456943	-1.0528769	0.29239742	0.99938111		Abhd11	14.7850192	9.95552182	14.9497146	18.6132601	11.653682	7.05632714	10.5849028	11.4131377
Zfp784	8.49364703	0.40635149	0.38595526	1.0528461	0.29241153	0.99938111		Zfp784	6.04841696	4.97776091	5.08926455	12.4088401	11.653682	10.5844907	5.77358333	11.4131377
Trove2	64.3518753	0.37917623	0.36020742	1.05266081	0.29249648	0.99938111		Trove2	52.4196136	52.4927514	63.6158069	45.4990802	28.3017992	117.605452	118.358458	36.5220405
Sri	46.2792032	-0.2737817	0.2601146	-1.0525425	0.29255074	0.99938111		Sri	51.7475673	59.2806072	46.7576181	47.5672202	31.6314227	41.1619083	50.9999861	41.0872956
Wdr61	20.6892337	0.3303021	0.31383247	1.05247906	0.29257982	0.99938111		Wdr61	16.1291119	15.8383302	20.3570582	20.6814001	26.6369875	22.345036	29.8301805	13.6957652
Wnt4	4.73824823	0.43643466	0.41468358	1.05245225	0.29259211	0.99938111		Wnt4	2.01613899	6.33533207	4.45310648	0	9.98887032	7.05632714	5.77358333	2.28262753
4933439C10Rik	2.38684364	-0.416492	0.39578268	-1.052325	0.29265047	NA		4933439C10Ri	7.39250961	1.35757116	0.9542371	4.13628002	3.32962344	0	1.92452778	0
Deptor	20.9437994	-0.3300162	0.31362098	-1.0522772	0.29267238	0.99938111		Deptor	19.4893435	30.3190892	24.1740066	20.6814001	13.3184938	18.8168724	20.2075417	20.5436478
Ankib1	65.7598385	0.29606239	0.28135825	1.05226125	0.2926797	0.99938111		Ankib1	56.4518916	69.236129	55.9819101	51.7035003	68.2572805	103.492798	79.8679027	41.0872956
Htr1f	1.50891957	-0.3866424	0.36764383	-1.0516766	0.29294796	NA		Htr1f	1.34409266	4.97776091	2.86271131	0	0	0	2.88679167	0
Unc13b	13.6096966	-0.3652288	0.34735637	-1.0514527	0.29305073	0.99938111		Unc13b	19.4893435	13.5757116	17.4943469	12.4088401	11.653682	16.4647633	8.660375	9.13051012
Samd12	10.8320135	0.43000817	0.40900517	1.05135142	0.29309722	0.99938111		Samd12	9.4086486	11.7656167	9.224292	2.06814001	3.32962344	27.0492541	19.2452778	4.56525506
Rsbn1l	20.483872	0.34459689	0.32783116	1.05114134	0.29319368	0.99938111		Rsbn1l	24.1936678	19.0059962	17.4943469	8.27256004	19.9777406	25.8731995	30.7924444	18.2610202
Zhx3	53.4821101	-0.3412684	0.32471821	-1.0509679	0.29327335	0.99938111		Zhx3	57.1239379	89.1471726	64.888123	33.0902402	28.3017992	65.8590533	52.9245139	36.5220405
Prtg	2.37002048	0.42932764	0.40851961	1.05093519	0.29328835	NA		Prtg	0.67204633	2.26261859	0.9542371	2.06814001	1.66481172	3.52816357	0.96226389	6.84788259
Rnf128	11.6952932	0.42325213	0.40275475	1.05089295	0.29330776	0.99938111		Rnf128	6.04841696	12.6706641	12.7231614	4.13628002	6.65924688	32.9295267	11.5471667	6.84788259
Cdk8	7.16158958	-0.4146882	0.39464225	-1.0507953	0.29335262	0.99938111		Cdk8	11.4247876	8.59795066	9.224292	6.20442003	6.65924688	9.40843619	5.77358333	0
St8sia3os	0.80113115	-0.3575774	0.34033611	-1.0506597	0.29341492	NA		St8sia3os	2.01613899	2.26261859	0.9542371	0	0	1.17605452	0	0
Btd	4.54602676	-0.433374	0.41248418	-1.050644	0.29342211	NA		Btd	6.72046328	7.2403795	3.81694841	4.13628002	3.32962344	2.35210905	1.92452778	6.84788259
Oaz3	2.5793684	0.43639635	0.41555022	1.05016513	0.2936422	NA		Oaz3	1.34409266	2.26261859	2.86271131	0	3.32962344	4.7042181	3.84905555	2.28262753
Pde4a	160.091058	0.32704207	0.31148313	1.04995114	0.29374058	0.99938111		Pde4a	116.936061	117.203643	131.048562	188.200741	206.436653	97.6125255	135.679208	287.611069
Ido1	0.66307426	-0.2738917	0.26086332	-1.0499433	0.29374418	NA		Ido1	4.03227797	0	1.27231614	0	0	0	0	0
Pwwp2a	16.7966911	0.37997272	0.36201124	1.04961582	0.29389478	0.99938111		Pwwp2a	19.4893435	13.1231878	13.6773985	8.27256004	6.65924688	18.8168724	26.9433889	27.3915304
Zmym5	18.6575693	0.39871251	0.38000024	1.04924279	0.29406639	0.99938111		Zmym5	13.4409266	14.480759	23.8559276	8.27256004	11.653682	37.6337448	30.7924444	9.13051012
Rpa2	6.04991098	-0.4327035	0.4125843	-1.0487639	0.29428679	0.99938111		Rpa2	6.04841696	4.52523719	4.77118552	16.5451201	4.99443516	1.17605452	5.77358333	4.56525506
Hecw2	41.7773123	0.2965846	0.28279824	1.04874979	0.29429329	0.99938111		Hecw2	48.3873356	26.6988994	42.3045116	28.9539601	48.2795399	52.9224536	43.301875	43.3699231
Pax6	6.16054287	-0.4287861	0.40888328	-1.048676	0.29432724	0.99938111		Pax6	10.0806949	8.59795066	10.1785291	2.06814001	4.99443516	4.7042181	8.660375	0
Dock9	84.0553162	0.36104952	0.34437326	1.04842494	0.29444287	0.99938111		Dock9	77.2853278	93.6724098	81.4282328	28.9539601	98.2238915	156.415252	88.5282777	47.9351782
Siglech	1.75670273	-0.3726256	0.35557141	-1.0479627	0.29465579	NA		Siglech	0	7.2403795	3.49886938	0	0	2.35210905	0.96226389	0
Foxred1	16.5966376	0.37540101	0.35854633	1.04700837	0.29509572	0.99938111		Foxred1	12.0968339	10.8605693	14.9497146	16.5451201	23.3073641	7.05632714	18.2830139	29.6741579
Wfikkn2	0.89931817	-0.2933101	0.28014215	-1.0470044	0.29509754	NA		Wfikkn2	0.67204633	0	0.31807903	6.20442003	0	0	0	0
Hic2	5.20307387	0.43531944	0.41578579	1.04698009	0.29510876	0.99938111		Hic2	6.04841696	2.71514231	2.22655324	4.13628002	14.9833055	1.17605452	5.77358333	4.56525506
Tead3	1.56607065	-0.3993695	0.38155172	-1.0466983	0.29523874	NA		Tead3	1.34409266	1.81009488	1.90847421	4.13628002	3.32962344	0	0	0
Polr2j	18.4124835	-0.382952	0.36593825	-1.0464936	0.29533319	0.99938111		Polr2j	28.2259458	24.8888045	20.6751372	10.3407001	6.65924688	15.2887088	11.5471667	29.6741579
Rexo4	12.5090682	0.3880808	0.3708509	1.04646045	0.29534849	0.99938111		Rexo4	11.4247876	9.05047438	10.1785291	8.27256004	16.6481172	9.40843619	7.69811111	27.3915304
Lrrc8a	55.7773274	-0.3772259	0.36061249	-1.04607	0.2955287	0.99938111		Lrrc8a	35.6184554	107.248121	66.7965972	51.7035003	39.9554813	84.6759257	25.981125	34.239413
Trhde	21.4445128	0.41806066	0.39976667	1.04576168	0.29567108	0.99938111		Trhde	11.4247876	30.3190892	19.7209001	4.13628002	13.3184938	56.4506172	20.2075417	15.9783927
Taldo1	18.9346641	-0.3866452	0.36973388	-1.0457392	0.29568144	0.99938111		Taldo1	32.2582238	16.7433776	14.6316356	24.8176801	23.3073641	8.23238167	8.660375	22.8262753
Tgif2	1.39873241	-0.4009657	0.38353526	-1.0454467	0.29581654	NA		Tgif2	1.34409266	1.81009488	1.27231614	4.13628002	1.66481172	0	0.96226389	0
Fam150b	2.3541669	0.42876314	0.41026874	1.04507876	0.29598657	NA		Fam150b	2.68818531	2.26261859	0.9542371	0	1.66481172	7.05632714	1.92452778	2.28262753
1700009P17Rik	0.87931752	-0.3670782	0.35127311	-1.0449937	0.2960259	NA		1700009P17Ri	1.34409266	1.81009488	0.63615807	2.06814001	0	1.17605452	0	0
Haghl	54.1209072	0.41588403	0.39806197	1.04477208	0.29612832	0.99938111		Haghl	32.2582238	17.1959013	24.1740066	97.2025805	71.586904	29.4013631	35.6037639	125.544514
Foxn3	16.4199624	0.37003748	0.3542069	1.04469305	0.29616486	0.99938111		Foxn3	18.8172972	14.9332827	12.4050823	6.20442003	11.653682	19.9929269	15.3962222	31.9567854
Limd2	35.5458959	-0.3551756	0.3399889	-1.0446681	0.29617638	0.99938111		Limd2	57.1239379	42.9897533	38.1694841	22.7495401	36.6258578	14.1126543	26.9433889	45.6525506
BC027072	0.64082319	0.30155609	0.2886763	1.04461673	0.29620014	NA		BC027072	0	0	0.63615807	0	0	3.52816357	0.96226389	0
Ppl	0.64082319	0.30155609	0.2886763	1.04461673	0.29620014	NA		Ppl	0	0	0.63615807	0	0	3.52816357	0.96226389	0
Ccdc148	9.85314278	0.41398691	0.3963956	1.04437819	0.29631045	0.99938111		Ccdc148	11.4247876	4.97776091	10.4966081	4.13628002	6.65924688	21.1689814	15.3962222	4.56525506
Slc25a23	310.115175	-0.2688709	0.25744949	-1.0443637	0.29631715	0.99938111		Slc25a23	403.899843	388.265351	363.882415	208.882141	301.330921	205.809542	291.565958	317.285227
Glud1	100.111846	-0.2395437	0.22942575	-1.0441011	0.29643862	0.99938111		Glud1	135.081312	92.7673624	93.8333152	115.815841	94.8942681	83.4998712	91.4150694	93.5877288
Cerk	68.444283	0.3135084	0.30027145	1.04408328	0.29644686	0.99938111		Cerk	64.5164475	46.6099431	54.3915149	72.3849004	64.9276571	58.8027262	55.8113055	130.109769
4933433G19Rik	1.50121576	0.39276917	0.37624198	1.04392702	0.29651915	NA		4933433G19R	0	0.45252372	2.22655324	0	1.66481172	1.17605452	1.92452778	4.56525506
Fbxl3	54.5683223	-0.3059343	0.29306104	-1.0439267	0.29651929	0.99938111		Fbxl3	74.5971425	69.236129	65.8423601	35.1583802	48.2795399	54.0985081	59.6603611	29.6741579
Msantd1	1.38938828	0.40583679	0.38878374	1.04386254	0.29654899	NA		Msantd1	2.01613899	0.45252372	0.63615807	0	1.66481172	1.17605452	2.88679167	2.28262753
Acsl1	28.4701702	0.31390344	0.3007314	1.04380002	0.29657792	0.99938111		Acsl1	24.1936678	28.0564706	27.672876	16.5451201	28.3017992	38.8097993	23.0943333	41.0872956
Ssr3	72.6581117	-0.3867133	0.370549	-1.0436226	0.29666004	0.99938111		Ssr3	65.8605402	136.209639	94.1513942	47.5672202	19.9777406	102.316744	74.0943194	41.0872956
Dennd2c	0.44866744	0.25615873	0.24548721	1.04347074	0.29673032	NA		Dennd2c	0	0	0	0	1.66481172	0	1.92452778	0
D16Ertd472e	4.28191132	-0.4339716	0.41602336	-1.0431423	0.29688238	NA		D16Ertd472e	6.72046328	7.69290322	6.04350166	2.06814001	4.99443516	0	6.73584722	0
Terf2ip	42.8612981	0.30122708	0.28887257	1.04276804	0.29705574	0.99938111		Terf2ip	38.978687	30.7716129	40.3960374	43.4309402	33.2962344	61.1548353	60.622625	34.239413
Trak2	111.38223	0.28045276	0.26895744	1.04274031	0.29706858	0.99938111		Trak2	115.591968	110.415787	109.101109	59.9760603	106.54795	139.950488	155.88675	93.5877288
Usp36	24.4863873	0.35345056	0.33896333	1.04273981	0.29706882	0.99938111		Usp36	22.8495752	14.0282353	19.0847421	26.8858201	39.9554813	21.1689814	15.3962222	36.5220405
Hinfp	5.72634477	0.41564736	0.39865722	1.04261842	0.29712505	0.99938111		Hinfp	5.37637063	2.71514231	5.72542262	4.13628002	8.3240586	8.23238167	6.73584722	4.56525506
Pappa	2.19212561	0.42023411	0.40311899	1.04245675	0.29719997	NA		Pappa	0	2.26261859	2.54463228	0	4.99443516	3.52816357	1.92452778	2.28262753
1110054M08Rik	2.1035168	-0.4279793	0.41055599	-1.0424384	0.29720849	NA		1110054M08R	4.03227797	2.71514231	2.54463228	2.06814001	3.32962344	1.17605452	0.96226389	0
Gm5113	17.8337094	0.34948552	0.33534786	1.0421582	0.29733834	0.99938111		Gm5113	14.1129729	17.1959013	16.2220308	12.4088401	18.3129289	11.7605452	29.8301805	22.8262753
Lrrc23	0.81449648	-0.3615315	0.34690978	-1.0421483	0.29734292	NA		Lrrc23	0.67204633	0.90504744	1.90847421	2.06814001	0	0	0.96226389	0
Dnah2	1.17154689	0.37152483	0.35652256	1.04207945	0.29737485	NA		Dnah2	2.01613899	0.45252372	0	0	1.66481172	2.35210905	2.88679167	0
Hcls1	1.53381537	0.40042637	0.38428032	1.04201634	0.2974041	NA		Hcls1	1.34409266	0.45252372	1.27231614	0	4.99443516	0	1.92452778	2.28262753

GeneID	Base mean	log2(FC)	StdErr	Wald-Stats	P-value	P-adj		GeneID	Normalised expression for Chow#1	Normalised expression for Chow#2	Normalised expression for Chow#3	Normalised expression for Chow#4	Normalised expression for HFD#1	Normalised expression for HFD#2	Normalised expression for HFD#3	Normalised expression for HFD#4
Ddx20	6.97656546	0.41476018	0.39803852	1.04201015	0.29740697	0.99938111		Ddx20	8.73660227	4.97776091	4.45310648	4.13628002	4.99443516	15.2887088	8.660375	4.56525506
Pcdh17	112.641806	0.35709395	0.34274531	1.04186386	0.2974748	0.99938111		Pcdh17	71.9089571	152.500493	92.560999	62.0442003	113.207197	235.210905	84.6792222	89.0224737
Ppfia2	93.5832682	0.38730559	0.37176133	1.04181247	0.29749863	0.99938111		Ppfia2	98.7908103	89.1471726	88.4259716	28.9539601	63.2628454	188.168724	146.264111	45.6525506
G3bp2	258.511369	0.2638646	0.25335518	1.04148099	0.29765237	0.99938111		G3bp2	253.361466	226.714383	253.508991	196.473301	219.755147	378.689557	338.716889	200.871223
Ubxn10	1.78710865	-0.4171009	0.40052681	-1.0413808	0.29769886	NA		Ubxn10	2.01613899	2.71514231	2.86271131	2.06814001	0	2.35210905	0	2.28262753
Slc40a1	1.56792118	-0.3986888	0.38300026	-1.0409623	0.29789304	NA		Slc40a1	0.67204633	5.43028463	1.27231614	2.06814001	0	1.17605452	1.92452778	0
Pnkp	9.08489997	0.43234814	0.41545955	1.0406504	0.29803783	0.99938111		Pnkp	6.72046328	2.26261859	3.81694841	14.4769801	19.9777406	2.35210905	4.81131944	18.2610202
BC053749	3.01797384	0.41897072	0.40262053	1.04060944	0.29805685	NA		BC053749	0	3.16766603	0.63615807	4.13628002	8.3240586	2.35210905	0.96226389	4.56525506
Adck1	18.8052218	0.34350085	0.33010665	1.0405754	0.29807266	0.99938111		Adck1	12.0968339	16.2908539	15.2677937	20.6814001	31.6314227	15.2887088	16.3584861	22.8262753
Fez2	18.5142347	-0.3303203	0.31744779	-1.0405499	0.2980845	0.99938111		Fez2	22.1775288	20.3635674	20.3570582	22.7495401	19.9777406	14.1126543	19.2452778	9.13051012
Ap2b1	249.41238	0.274051	0.26339523	1.04045543	0.29812836	0.99938111		Ap2b1	229.167798	191.417533	227.42651	241.972381	396.225189	215.217978	185.71693	308.154717
Bpgm	39.1858182	-0.3078593	0.29597567	-1.0401506	0.29826992	0.99938111		Bpgm	57.1239379	41.1796584	38.8056422	39.2946602	19.9777406	31.7534722	44.2641389	41.0872956
Pgls	14.9103958	-0.4316918	0.4150466	-1.0401045	0.29829134	0.99938111		Pgls	12.0968339	9.05047438	9.54237104	45.4990802	13.3184938	1.17605452	5.77358333	22.8262753
Strc	1.94926612	0.36251261	0.34860916	1.03988263	0.29839443	NA		Strc	0	0	0.31807903	4.13628002	3.32962344	0	0.96226389	6.84788259
Dcbld1	2.84458659	-0.4316773	0.41518854	-1.0397139	0.29847286	NA		Dcbld1	4.7043243	4.52523719	5.08926455	0	1.66481172	3.52816357	0.96226389	2.28262753
Zfyve16	6.77323055	-0.4154965	0.3996668	-1.0396072	0.29852245	0.99938111		Zfyve16	9.4086486	9.5029981	6.36158069	8.27256004	0	7.05632714	6.73584722	6.84788259
Poc1a	3.27923531	-0.43277	0.4163529	-1.0394308	0.29860441	NA		Poc1a	4.03227797	3.16766603	3.49886938	6.20442003	1.66481172	1.17605452	1.92452778	4.56525506
Sox2ot	25.1143657	0.30057156	0.28919744	1.03932995	0.29865131	0.99938111		Sox2ot	20.8334362	23.0787097	24.8101647	18.6132601	21.6425524	28.2253086	31.7547083	31.9567854
Ei24	52.8613666	-0.2834486	0.2727532	-1.0392128	0.29870579	0.99938111		Ei24	42.3389187	66.5209867	58.5265423	70.3167603	38.2906696	49.39429	58.6980972	38.804668
Ugp2	51.6214774	-0.407042	0.39172569	-1.0390995	0.29875848	0.99938111		Ugp2	74.5971425	73.7613662	73.1581779	28.9539601	14.9833055	55.2745626	80.8301666	11.4131377
Lman2l	22.2406827	-0.3821042	0.36772678	-1.0390981	0.29875911	0.99938111		Lman2l	12.0968339	40.7271347	22.9016905	28.9539601	18.3129289	27.0492541	9.62263889	18.2610202
Ugcg	88.2501428	0.32459978	0.31250022	1.03871857	0.29893564	0.99938111		Ugcg	67.8766792	112.225882	85.2451812	37.2265202	76.5813391	141.126543	87.5660139	98.1529838
F8a	25.5057553	0.41965713	0.404047	1.03863445	0.29897478	0.99938111		F8a	13.4409266	12.6706641	17.4943469	35.1583802	29.966611	4.7042181	22.1320694	68.4788259
Edem2	6.42956025	-0.4125798	0.39729779	-1.038465	0.29905364	0.99938111		Edem2	6.04841696	9.95552182	6.67965972	8.27256004	8.3240586	4.7042181	2.88679167	4.56525506
Gm12522	1.16560463	-0.3357272	0.32334107	-1.0383067	0.29912731	NA		Gm12522	0.67204633	0	1.27231614	6.20442003	0	1.17605452	0	0
Abcb6	6.26618639	-0.4184129	0.40302839	-1.0381722	0.2991899	0.99938111		Abcb6	4.03227797	11.313093	8.58813393	6.20442003	6.65924688	5.88027262	2.88679167	4.56525506
Capns1	137.331034	-0.3097744	0.29840787	-1.0380905	0.29922791	0.99938111		Capns1	147.850192	123.086452	131.68472	215.086561	123.196067	78.7956531	89.4905416	189.458085
Fam168a	259.018935	0.20121977	0.19384105	1.03806587	0.29923939	0.99938111		Fam168a	254.033512	247.077951	259.870571	196.473301	293.006863	308.126285	276.169736	237.393263
Rfc2	17.8054834	0.38083886	0.36688144	1.03804342	0.29924984	0.99938111		Rfc2	19.4893435	10.4080455	10.8146872	18.6132601	24.9721758	7.05632714	25.981125	25.1089028
Hist1h2bk	1.66915348	-0.4133093	0.39820238	-1.0379378	0.29929903	NA		Hist1h2bk	4.03227797	0.90504744	2.54463228	2.06814001	1.66481172	1.17605452	0.96226389	0
Hes5	11.6144087	-0.4290116	0.41335437	-1.0378785	0.2993266	0.99938111		Hes5	10.0806949	10.4080455	10.8146872	26.8858201	11.653682	0	4.81131944	18.2610202
Snx25	28.8251681	0.40830201	0.39344136	1.03777094	0.29937671	0.99938111		Snx25	21.5054825	29.4140417	33.0802196	6.20442003	16.6481172	74.091435	33.6792361	15.9783927
Nuak1	53.5975122	0.26372071	0.25420962	1.03741437	0.29954278	0.99938111		Nuak1	56.4518916	45.2523719	40.7141164	49.6353602	69.9220923	50.5703445	50.0377222	66.1961984
Chac2	5.70349065	0.42426989	0.40897372	1.03740136	0.29954884	0.99938111		Chac2	5.37637063	4.97776091	2.22655324	6.20442003	4.99443516	7.05632714	12.5094306	2.28262753
Mycbp	4.47339214	-0.4315254	0.4162655	-1.0366592	0.29989473	NA		Mycbp	4.7043243	3.62018975	3.18079035	12.4088401	0	1.17605452	3.84905555	6.84788259
D5Ertd579e	86.3814187	0.2619293	0.2527049	1.03650265	0.29996771	0.99938111		D5Ertd579e	80.6455594	76.0239848	84.9271022	70.3167603	81.5757743	129.365998	101.999972	66.1961984
Tmem184b	74.8443565	-0.3592221	0.34657142	-1.0365023	0.29996789	0.99938111		Tmem184b	55.1077989	114.488501	76.0208892	99.2707205	79.9109626	51.7463991	24.0565972	98.1529838
Mpp2	208.719377	0.37943567	0.36608312	1.0364741	0.29998101	0.99938111		Mpp2	160.619072	119.918786	122.460428	285.403321	351.275273	109.373071	121.24525	399.459818
A930005H10Rik	6.33129346	-0.4219843	0.40722178	-1.0362517	0.30008474	0.99938111		A930005H10R	10.0806949	5.88280835	10.1785291	4.13628002	3.32962344	1.17605452	6.73584722	9.13051012
Celf2	346.104355	0.25074047	0.24198944	1.03616286	0.30012617	0.99938111		Celf2	317.205867	348.895787	374.697103	208.882141	377.912261	375.161393	464.773458	301.306834
A4galt	1.03842232	-0.3472945	0.33519856	-1.036086	0.30016201	NA		A4galt	0	3.62018975	0.9542371	2.06814001	1.66481172	0	0	0
Prr5l	3.4307925	-0.4259731	0.41123662	-1.0358346	0.30027929	NA		Prr5l	1.34409266	5.88280835	2.22655324	10.3407001	1.66481172	1.17605452	4.81131944	0
Nsun2	39.8804099	0.34538397	0.33349609	1.03564622	0.30036722	0.99938111		Nsun2	43.010965	35.2968501	40.3960374	16.5451201	31.6314227	56.4506172	68.3207361	27.3915304
Dhrs7	5.80886097	-0.4246591	0.41017842	-1.0353033	0.30052731	0.99938111		Dhrs7	4.7043243	12.6706641	3.81694841	8.27256004	4.99443516	5.88027262	3.84905555	2.28262753
Rnase1	0.47545588	-0.2947361	0.28481012	-1.0348513	0.30073834	NA		Rnase1	0.67204633	0.90504744	2.22655324	0	0	0	0	0
Alox12b	1.27354306	0.33665678	0.32533373	1.0348044	0.30076027	NA		Alox12b	0	1.35757116	0.31807903	0	1.66481172	0	0	6.84788259
Slc20a1	44.5191164	-0.3969739	0.38373999	-1.0344866	0.30090873	0.99938111		Slc20a1	37.6345944	89.1471726	55.345752	31.0221002	38.2906696	70.5632714	25.0188611	9.13051012
Puf60	81.523855	0.31916567	0.30871411	1.03385513	0.3012039	0.99938111		Puf60	71.9089571	56.1129411	56.6180681	99.2707205	116.53682	51.7463991	72.1697916	127.827142
Gda	220.332774	0.33315381	0.32224457	1.03385392	0.30120446	0.99938111		Gda	235.216215	187.34482	227.108431	107.543281	228.079206	311.654449	349.301792	116.414004
Gstp2	18.5713164	-0.4054407	0.39220136	-1.0337566	0.30124998	0.99938111		Gstp2	14.7850192	16.7433776	17.4943469	41.3628002	16.6481172	2.35210905	16.3584861	22.8262753
Pfdn2	20.5403135	0.34335696	0.33225424	1.03341636	0.30140909	0.99938111		Pfdn2	22.1775288	14.480759	18.130505	16.5451201	18.3129289	21.1689814	37.5282917	15.9783927
Gnl3l	287.929848	0.24968214	0.24162318	1.03335343	0.30143853	0.99938111		Gnl3l	310.485404	224.451765	288.497684	219.222841	238.068076	384.569829	373.358389	264.784794
Ido2	0.86251193	-0.3652866	0.35352501	-1.0332696	0.30147776	NA		Ido2	1.34409266	1.35757116	0.9542371	2.06814001	0	1.17605452	0	0
Txlna	22.1883103	0.32035301	0.31005996	1.03319699	0.30151172	0.99938111		Txlna	18.8172972	20.3635674	19.0847421	16.5451201	29.966611	21.1689814	17.32075	34.239413
0610010B08Rik	63.6178873	0.42331438	0.40974244	1.03312312	0.30154629	0.99938111		0610010B08Ri	49.7314283	55.6604174	68.3869924	18.6132601	18.3129289	112.901234	169.358444	15.9783927
Bambi-ps1	1.53645966	0.40861779	0.39553019	1.03308877	0.30156236	NA		Bambi-ps1	0.67204633	0.90504744	0.63615807	2.06814001	1.66481172	1.17605452	2.88679167	2.28262753
Yars2	8.45038358	0.38535298	0.37311046	1.03281204	0.30169187	0.99938111		Yars2	6.04841696	8.14542694	5.72542262	8.27256004	13.3184938	10.5844907	8.660375	6.84788259
3110021N24Rik	9.92759828	0.38539253	0.37316235	1.03277442	0.30170948	0.99938111		3110021N24R	10.0806949	6.33533207	9.86045007	6.20442003	6.65924688	10.5844907	18.2830139	11.4131377
Hist4h4	7.13832183	-0.4288663	0.41531055	-1.0326401	0.30177237	0.99938111		Hist4h4	12.7688802	6.78785578	6.99773876	8.27256004	6.65924688	0	1.92452778	13.6957652
Lum	0.74248096	-0.3418301	0.33103683	-1.0326045	0.30178903	NA		Lum	0.67204633	2.71514231	1.59039517	0	0	0	0.96226389	0
Herc6	3.41634595	-0.4294874	0.41598725	-1.0324532	0.30185985	NA		Herc6	2.01613899	7.2403795	4.45310648	4.13628002	4.99443516	3.52816357	0.96226389	0
Col11a1	0.58725341	-0.3005602	0.29112022	-1.0324264	0.30187243	NA		Col11a1	0	1.35757116	1.27231614	2.06814001	0	0	0	0
Eef1d	28.2287111	0.37767578	0.36586952	1.03226903	0.30194611	0.99938111		Eef1d	19.4893435	19.0059962	16.2220308	39.2946602	48.2795399	21.1689814	14.4339583	47.9351782
St3gal5	53.5299824	-0.382705	0.37075936	-1.0322193	0.30196941	0.99938111		St3gal5	58.4680306	113.13093	65.2062021	14.4769801	48.2795399	57.6266717	41.3773472	29.6741579
Il6st	40.0551887	-0.3964819	0.38412948	-1.0321569	0.3019986	0.99938111		Il6st	28.8979921	87.3370778	54.3915149	20.6814001	28.3017992	61.1548353	25.981125	13.6957652
Ptgfr	0.9660539	-0.3427934	0.33212036	-1.0321361	0.30200834	NA		Ptgfr	0	1.35757116	1.27231614	4.13628002	0	0	0.96226389	0
Ush1g	0.91975923	0.34993524	0.33903985	1.03213603	0.3020084	NA		Ush1g	0	0	1.27231614	0	1.66481172	1.17605452	0.96226389	2.28262753
Shc2	34.6569873	0.41348419	0.40072543	1.03183915	0.30214748	0.99938111		Shc2	22.1775288	10.8605693	14.6316356	62.0442003	54.9387868	16.4647633	23.0943333	73.044081
Ap4m1	7.06160075	-0.4221465	0.40914359	-1.0317808	0.30217481	0.99938111		Ap4m1	7.39250961	4.07271347	6.36158069	18.6132601	8.3240586	2.35210905	4.81131944	4.56525506
Rsad1	9.21098904	0.38580665	0.37398691	1.03160468	0.30225735	0.99938111		Rsad1	6.72046328	5.43028463	7.63389683	10.3407001	11.653682	8.23238167	7.69811111	15.9783927
Zfand4	3.28906751	0.42937135	0.41637046	1.03122433	0.30243563	NA		Zfand4	2.01613899	1.35757116	2.54463228	4.13628002	4.99443516	7.05632714	1.92452778	2.28262753
Gpr75	10.5588902	-0.3879335	0.37625425	-1.0310409	0.30252164	0.99938111		Gpr75	13.4409266	17.648425	13.9954775	4.13628002	11.653682	9.40843619	9.62263889	4.56525506
Cbx6	344.082697	0.35149144	0.34097373	1.03084611	0.30261299	0.99938111		Cbx6	262.098068	221.736622	231.243458	450.854522	635.958077	229.330632	207.849	513.591194
Gm13889	36.888754	0.42753315	0.41477827	1.03075107	0.30265757	0.99938111		Gm13889	20.8334362	11.7656167	16.2220308	59.9760603	44.9499164	8.23238167	14.4339583	118.696632
Dzip3	61.0788886	0.35166394	0.3412302	1.03057685	0.3027393	0.99938111		Dzip3	55.7798453	66.5209867	56.9361472	26.8858201	34.9610461	108.197016	91.4150694	47.9351782
Rnf181	34.6135624	-0.3010731	0.29218939	-1.0304041	0.30282035	0.99938111		Rnf181	32.9302701	38.0119924	33.3982986	53.7716403	24.9721758	24.697145	39.4528194	29.6741579
Lgmn	36.2383789	-0.3710585	0.36017823	-1.030208	0.3029124	0.99938111		Lgmn	37.6345944	68.7836053	39.1237212	22.7495401	34.9610461	49.39429	14.4339583	22.8262753
Gas7	602.053561	0.20885783	0.20273702	1.0301909	0.3029204	0.99938111		Gas7	615.594437	592.806072	608.167114	401.219162	702.550546	619.780734	666.848875	609.461551
Ccny	137.485627	-0.2683323	0.26048977	-1.0301069	0.30295982	0.99938111		Ccny	165.323397	176.936774	164.76494	99.2707205	118.201632	111.72518	165.509389	98.1529838
Dhrs1	44.1077329	-0.3418261	0.33184132	-1.0300892	0.30296815	0.99938111		Dhrs1	49.059382	38.9170398	36.2610099	78.5893204	31.6314227	21.1689814	35.6037639	61.6309433
Frat2	7.60418403	0.42883961	0.41633282	1.03004037	0.30299105	0.99938111		Frat2	3.36023164	2.26261859	2.86271131	14.4769801	4.99443516	2.35210905	7.69811111	22.8262753
Doc2g	0.87454535	-0.3553846	0.34503645	-1.0299915	0.303014	NA		Doc2g	1.34409266	2.71514231	1.27231614	0	1.66481172	0	0	0
Cox7c	12.1561001	0.36340009	0.35281989	1.02998753	0.30301586	0.99938111		Cox7c	8.73660227	9.05047438	8.90621297	14.4769801	16.6481172	10.5844907	10.5849028	18.2610202
Zfp85	3.26452386	0.42056758	0.40832425	1.02998434	0.30301736	NA		Zfp85	2.01613899	2.26261859	4.13502745	0	1.66481172	3.52816357	12.5094306	0
Pik3cb	31.5086234	0.29660905	0.2880063	1.02987	0.30307103	0.99938111		Pik3cb	28.8979921	29.4140417	31.1717454	18.6132601	46.6147282	32.9295267	27.9056528	36.5220405
Snip1	8.06991696	-0.4068635	0.39508991	-1.0297998	0.30310401	0.99938111		Snip1	6.72046328	8.59795066	7.63389683	18.6132601	4.99443516	7.05632714	8.660375	2.28262753
Actg1	348.760176	0.29763842	0.28902918	1.02978674	0.30311013	0.99938111		Actg1	295.028338	240.290095	251.918595	442.581962	419.532554	251.675668	325.245194	563.809
6030419C18Rik	144.814889	0.35292234	0.34278024	1.02958777	0.30320356	0.99938111		6030419C18Ri	118.9522	109.51074	99.2406588	161.314921	248.056946	88.2040893	79.8679027	253.371656
Irgm1	2.25317622	-0.420703	0.40881187	-1.029087	0.30343879	NA		Irgm1	2.68818531	3.62018975	4.13502745	2.06814001	1.66481172	0	3.84905555	0
Lrrc47	111.312149	0.42595329	0.41404546	1.02875972	0.3035926	0.99938111		Lrrc47	37.6345944	34.8443264	40.7141164	217.154701	179.799666	47.042181	36.5660278	296.741579
Ttll5	29.9416279	0.32823142	0.3190562	1.02875738	0.3035937	0.99938111		Ttll5	24.8657142	32.5817078	25.1282437	20.6814001	16.6481172	48.2182355	39.4528194	31.9567854
C77370	62.6567319	0.33711043	0.32768737	1.02875627	0.30359422	0.99938111		C77370	51.7475673	54.75537	63.9338859	45.4990802	56.6035985	107.020962	94.3018611	27.3915304
Shroom1	4.10987912	0.42545899	0.41358083	1.02872028	0.30361114	NA		Shroom1	3.36023164	4.07271347	1.27231614	4.13628002	4.99443516	4.7042181	5.77358333	4.56525506
2010320M18Rik	1.82065539	0.41390208	0.40236229	1.0286801	0.30363002	NA		2010320M18R	1.34409266	2.26261859	0.9542371	0	1.66481172	3.52816357	4.81131944	0
Atp13a5	2.37710807	-0.4016037	0.39040959	-1.0286727	0.3036335	NA		Atp13a5	2.68818531	8.14542694	2.22655324	0	4.99443516	0	0.96226389	0
BC048507	1.7099902	-0.4126928	0.40120753	-1.0286267	0.30365513	NA		BC048507	1.34409266	3.62018975	2.22655324	2.06814001	0	1.17605452	0.96226389	2.28262753
Csmd1	59.1893151	0.34075302	0.33130132	1.02852901	0.30370105	0.99938111		Csmd1	40.3227797	69.236129	63.2977279	26.8858201	104.883138	76.4435441	49.0754583	43.3699231
2310057M21Rik	16.7899057	-0.3347916	0.3255076	-1.0285216	0.30370452	0.99938111		2310057M21R	21.5054825	17.648425	19.4028211	18.6132601	9.98887032	19.9929269	13.4716944	13.6957652
Manbal	19.1319533	-0.3708461	0.36057538	-1.0284842	0.30372211	0.99938111		Manbal	20.8334362	18.5534725	18.130505	33.0902402	18.3129289	4.7042181	21.1698055	18.2610202
2410015M20Rik	17.5882291	-0.3728079	0.36251151	-1.0284028	0.30376037	0.99938111		2410015M20R	17.4732045	20.8160911	21.3112953	24.8176801	4.99443516	12.9365998	26.9433889	11.4131377
B230217C12Rik	44.6883477	0.2665964	0.25923581	1.02839342	0.3037648	0.99938111		B230217C12Ri	32.2582238	41.1796584	39.7598793	47.5672202	39.9554813	47.042181	48.1131944	61.6309433
Pole3	7.09689367	0.41048736	0.39942236	1.0277025	0.30408979	0.99938111		Pole3	2.01613899	5.88280835	5.08926455	10.3407001	9.98887032	4.7042181	9.62263889	9.13051012
Dhx38	21.9234928	0.34601318	0.33670646	1.02764046	0.30411899	0.99938111		Dhx38	14.7850192	13.1231878	16.8581888	33.0902402	33.2962344	19.9929269	25.981125	18.2610202
Chmp6	13.2261523	0.40372946	0.3929039	1.0275527	0.30416029	0.99938111		Chmp6	7.39250961	5.88280835	6.36158069	24.8176801	16.6481172	16.4647633	7.69811111	20.5436478
Ranbp3l	2.04761147	-0.4161202	0.4050123	-1.027426	0.30421992	NA		Ranbp3l	4.7043243	2.26261859	3.81694841	0	0	2.35210905	0.96226389	2.28262753
H2-Aa	0.96411366	0.33589572	0.32694142	1.0273881	0.30423775	NA		H2-Aa	0	0.90504744	0.31807903	0	0	0	1.92452778	4.56525506
Hist2h3b	7.78774247	0.42548306	0.41430629	1.02697708	0.30443126	0.99938111		Hist2h3b	5.37637063	4.07271347	4.77118552	8.27256004	3.32962344	2.35210905	6.73584722	27.3915304
Wwp2	27.2978382	0.3232528	0.31477956	1.02691802	0.30445907	0.99938111		Wwp2	28.2259458	22.6261859	17.8124259	24.8176801	36.6258578	24.697145	20.2075417	43.3699231
Dfna5	6.71303971	0.41903004	0.40805642	1.0268924	0.30447114	0.99938111		Dfna5	8.06455594	1.81009488	7.31581779	2.06814001	9.98887032	8.23238167	4.81131944	11.4131377
Camkv	315.113115	0.34842899	0.33932976	1.0268153	0.30450744	0.99938111		Camkv	269.490578	201.373055	206.115214	392.946602	589.343349	195.225051	203.03768	463.373389
Nap1l3	55.3533105	0.34264289	0.33370606	1.02678056	0.30452381	0.99938111		Nap1l3	52.4196136	35.2968501	49.6203294	53.7716403	28.3017992	101.140689	78.9056389	43.3699231
Sirt4	2.84675654	-0.4232593	0.41225673	-1.0266886	0.30456712	NA		Sirt4	2.01613899	3.16766603	2.54463228	8.27256004	0	3.52816357	0.96226389	2.28262753

GeneID	Base mean	log2(FC)	StdErr	Wald-Stats	P-value	P-adj		GeneID	Normalised expression for Chow#1	Normalised expression for Chow#2	Normalised expression for Chow#3	Normalised expression for Chow#4	Normalised expression for HFD#1	Normalised expression for HFD#2	Normalised expression for HFD#3	Normalised expression for HFD#4
Dhx33	21.7807624	0.31347607	0.30537468	1.02652937	0.30464213	0.99938111		Dhx33	20.8334362	17.648425	22.9016905	12.4088401	28.3017992	25.8731995	21.1698055	25.1089028
Slx1b	5.34433346	-0.4243712	0.41342211	-1.026484	0.30466351	0.99938111		Slx1b	4.7043243	8.14542694	1.27231614	14.4769801	0	1.17605452	3.84905555	9.13051012
Nr6a1	4.23433954	-0.4218664	0.41103039	-1.0263631	0.30472047	NA		Nr6a1	7.39250961	6.33533207	5.08926455	2.06814001	3.32962344	3.52816357	3.84905555	2.28262753
Mtap	7.75442621	-0.3997753	0.38950772	-1.0263603	0.30472178	0.99938111		Mtap	8.73660227	7.69290322	9.86045007	12.4088401	1.66481172	9.40843619	7.69811111	4.56525506
Zfp800	13.3390757	-0.4142605	0.40372143	-1.0261049	0.30484216	0.99938111		Zfp800	18.1452509	17.648425	18.448584	12.4088401	0	15.2887088	20.2075417	4.56525506
Mum1l1	4.27611352	-0.4197448	0.40911753	-1.025976	0.30490291	NA		Mum1l1	4.7043243	6.33533207	6.04350166	4.13628002	3.32962344	3.52816357	3.84905555	2.28262753
Klhl26	46.0240859	0.35388285	0.34508462	1.02549587	0.30512927	0.99938111		Klhl26	29.5700384	30.7716129	31.8079035	64.1123403	64.9276571	31.7534722	30.7924444	84.4572186
Ctdspl	21.3884985	-0.3185151	0.3106224	-1.0254091	0.30517018	0.99938111		Ctdspl	30.9141311	20.8160911	26.0824808	18.6132601	16.6481172	17.6408179	22.1320694	18.2610202
Smad2	37.4596054	-0.2930503	0.28579991	-1.0253687	0.30518923	0.99938111		Smad2	46.3711967	33.9392789	38.4875632	51.7035003	23.3073641	39.9858538	38.4905555	27.3915304
Zfp382	7.90892536	0.41301177	0.40281432	1.02531553	0.30521433	0.99938111		Zfp382	4.7043243	6.78785578	10.4966081	2.06814001	9.98887032	10.5844907	16.3584861	2.28262753
Srd5a1	6.45220445	-0.4220311	0.41162325	-1.0252849	0.30522879	0.99938111		Srd5a1	2.68818531	7.69290322	6.99773876	16.5451201	3.32962344	8.23238167	3.84905555	2.28262753
Cd164	52.4057731	-0.3481634	0.33958888	-1.0252498	0.30524532	0.99938111		Cd164	35.6184554	95.9350284	68.7050715	41.3628002	43.2851047	57.6266717	53.8867778	22.8262753
Gm20939	1.45987104	-0.3981129	0.38831448	-1.0252332	0.30525316	NA		Gm20939	2.01613899	3.16766603	3.18079035	0	0	2.35210905	0.96226389	0
Naa15	50.044776	0.37856008	0.36954237	1.02440238	0.30564526	0.99938111		Naa15	54.4357526	37.5594687	44.5310648	28.9539601	23.3073641	94.0843619	92.3773333	25.1089028
Mir1900	0.96849108	0.30329895	0.29611265	1.02426879	0.30570834	NA		Mir1900	0	0.45252372	0.63615807	0	6.65924688	0	0	0
Cttnbp2	47.2029203	0.34587697	0.33770355	1.02420294	0.30573943	0.99938111		Cttnbp2	54.4357526	52.0402277	38.1694841	14.4769801	44.9499164	72.9153805	66.3962083	34.239413
Gm11627	1.89398254	-0.4071958	0.39765022	-1.0240049	0.30583295	NA		Gm11627	4.7043243	2.71514231	3.18079035	0	1.66481172	0	2.88679167	0
Fxyd5	1.59225201	-0.3869003	0.37786592	-1.0239089	0.30587831	NA		Fxyd5	0.67204633	4.97776091	3.49886938	0	1.66481172	0	1.92452778	0
Gt(ROSA)26Sor	4.69745358	0.42589127	0.41597975	1.02382693	0.30591703	0.99938111		t(ROSA)26Sc	1.34409266	3.62018975	2.22655324	8.27256004	8.3240586	7.05632714	6.73584722	0
Shank2	130.844318	0.36434245	0.35590699	1.02370131	0.30597638	0.99938111		Shank2	104.839227	121.72888	99.5587378	109.611421	301.330921	77.6195986	65.4339444	166.63181
Cttnbp2nl	21.0644717	-0.3725395	0.36392434	-1.0236728	0.30598983	0.99938111		Cttnbp2nl	30.9141311	29.8665654	22.5836114	16.5451201	6.65924688	25.8731995	26.9433889	9.13051012
Stoml1	29.2112593	0.34462003	0.33665108	1.02367124	0.30599058	0.99938111		Stoml1	18.1452509	27.1514231	20.6751372	33.0902402	43.2851047	27.0492541	16.3584861	47.9351782
Rasa1	52.789032	0.34834061	0.34028893	1.0236613	0.30599528	0.99938111		Rasa1	55.7798453	56.1129411	48.9841713	16.5451201	43.2851047	97.6125255	60.622625	43.3699231
Xdh	0.59475765	-0.2672515	0.26108439	-1.0236212	0.30601422	NA		Xdh	0	3.16766603	1.59039517	0	0	0	0	0
9130023H24Rik	4.1668299	-0.424418	0.41465089	-1.0235549	0.30604554	NA		9130023H24Ri	4.03227797	6.33533207	3.49886938	8.27256004	4.99443516	2.35210905	3.84905555	0
Gm14308	64.1445544	0.41764219	0.40808433	1.02342127	0.30610871	0.99938111		Gm14308	50.4034746	56.1129411	68.7050715	20.6814001	19.9777406	112.901234	168.39618	15.9783927
Gm14430	64.1445544	0.41764219	0.40808433	1.02342127	0.30610871	0.99938111		Gm14430	50.4034746	56.1129411	68.7050715	20.6814001	19.9777406	112.901234	168.39618	15.9783927
Gm14434	64.1445544	0.41764219	0.40808433	1.02342127	0.30610871	0.99938111		Gm14434	50.4034746	56.1129411	68.7050715	20.6814001	19.9777406	112.901234	168.39618	15.9783927
Gm4724	64.1445544	0.41764219	0.40808433	1.02342127	0.30610871	0.99938111		Gm4724	50.4034746	56.1129411	68.7050715	20.6814001	19.9777406	112.901234	168.39618	15.9783927
Cygb	133.84686	0.38274904	0.37399879	1.02339648	0.30612042	0.99938111		Cygb	90.054208	54.3028463	82.0643909	213.018421	191.453348	75.2674895	95.264125	269.350049
Med16	77.6445626	0.39671016	0.38765454	1.02336002	0.30613766	0.99938111		Med16	51.075521	43.442277	47.0756971	107.543281	123.196067	29.4013631	41.3773472	178.044947
Dnajc7	71.3914595	0.31366931	0.30655016	1.02322344	0.30620221	0.99938111		Dnajc7	81.9896521	45.7048956	59.4807795	64.1123403	54.9387868	111.72518	102.962236	50.2178057
Bbox1	1.28178879	-0.3771924	0.36865612	-1.0231552	0.30623449	NA		Bbox1	2.01613899	0.90504744	1.27231614	4.13628002	0	0	1.92452778	0
C130083M11Rik	2.57476047	0.41359272	0.40423871	1.0231398	0.30624175	NA		130083M11R	0	2.26261859	2.54463228	2.06814001	0	4.7042181	6.73584722	2.28262753
Efcc1	6.02920214	-0.4187123	0.40931317	-1.0229631	0.30632532	0.99938111		Efcc1	5.37637063	7.2403795	7.31581779	10.3407001	6.65924688	0	6.73584722	4.56525506
Apoa2	0.41429662	0.2475072	0.2419936	1.02278408	0.30640995	NA		Apoa2	0	0	0	0	0	2.35210905	0.96226389	0
Ccdc36	0.41429662	0.2475072	0.2419936	1.02278408	0.30640995	NA		Ccdc36	0	0	0	0	0	2.35210905	0.96226389	0
Murc	0.41429662	0.2475072	0.2419936	1.02278408	0.30640995	NA		Murc	0	0	0	0	0	2.35210905	0.96226389	0
Kif11	0.63162144	0.32187043	0.31470977	1.02275324	0.30642453	NA		Kif11	0.67204633	0	0.31807903	0	0	1.17605452	2.88679167	0
Htr6	2.2712497	-0.4222024	0.41284729	-1.0226599	0.30646866	NA		Htr6	2.68818531	2.71514231	2.54463228	4.13628002	1.66481172	1.17605452	0.96226389	2.28262753
Gabarapl1	579.623662	0.33505009	0.32769631	1.02244083	0.3065723	0.99938111		Gabarapl1	445.566716	315.861556	366.427048	862.414384	620.974772	418.675411	582.169653	1024.89976
Sh2d7	5.11938277	-0.4249973	0.41568748	-1.0223962	0.30659342	0.99938111		Sh2d7	4.7043243	4.97776091	4.13502745	12.4088401	4.99443516	0	2.88679167	6.84788259
Ncf1	1.60779483	-0.3989223	0.39023232	-1.0222688	0.30665372	NA		Ncf1	4.03227797	4.07271347	0.9542371	0	1.66481172	1.17605452	0.96226389	0
Slc1a6	7.92021045	0.42179016	0.41260436	1.02226296	0.30665646	0.99938111		Slc1a6	2.01613899	4.97776091	3.81694841	14.4769801	16.6481172	9.40843619	2.88679167	9.13051012
Slc13a4	7.08464129	-0.4253258	0.41608078	-1.0222193	0.30667711	0.99938111		Slc13a4	10.0806949	16.7433776	6.67965972	2.06814001	0	2.35210905	9.62263889	9.13051012
Eif4enif1	36.8118905	-0.2920898	0.28580524	-1.021989	0.30678612	0.99938111		Eif4enif1	51.7475673	40.274611	40.3960374	33.0902402	29.966611	39.9858538	38.4905555	20.5436478
Ildr2	129.328586	0.34377666	0.33639054	1.02195698	0.30680126	0.99938111		Ildr2	84.6778374	171.959013	122.142349	59.9760603	134.849749	249.323559	113.547139	98.1529838
Vgf	249.639002	0.37497759	0.36699363	1.02175504	0.30689685	0.99938111		Vgf	118.9522	223.546717	173.671153	308.152862	329.632721	321.062885	76.9811111	445.112369
Ajap1	35.5482185	-0.2878412	0.28172845	-1.0216973	0.30692421	0.99938111		Ajap1	40.3227797	52.0402277	36.2610099	28.9539601	31.6314227	34.1055812	33.6792361	27.3915304
Abtb1	12.4592809	0.42024756	0.41148764	1.02128841	0.30711781	0.99938111		Abtb1	11.4247876	4.97776091	9.86045007	10.3407001	28.3017992	3.52816357	3.84905555	27.3915304
Agpat5	24.687108	-0.3457522	0.33862572	-1.0210453	0.307233	0.99938111		Agpat5	24.1936678	45.7048956	27.672876	14.4769801	16.6481172	19.9929269	25.981125	22.8262753
Krt222	36.6244818	0.33537785	0.32847176	1.02102492	0.30724263	0.99938111		Krt222	37.6345944	30.7716129	36.2610099	20.6814001	21.6425524	41.1619083	68.3207361	36.5220405
Ccdc32	19.9879352	0.3257613	0.31911502	1.02082723	0.3073363	0.99938111		Ccdc32	20.1613899	19.4585199	16.8581888	12.4088401	14.9833055	24.697145	30.7924444	20.5436478
Nucb2	4.64601367	0.42143944	0.41291897	1.02063473	0.30742753	NA		Nucb2	2.68818531	4.52523719	5.08926455	0	1.66481172	16.4647633	6.73584722	0
Slco3a1	36.2289583	0.34089306	0.33400236	1.02063069	0.30742944	0.99938111		Slco3a1	20.8334362	44.7998482	25.7644018	31.0221002	49.9443516	47.042181	20.2075417	50.2178057
Abhd5	9.50073152	-0.3955492	0.38760422	-1.0204976	0.30749253	0.99938111		Abhd5	14.1129729	16.2908539	12.0870033	2.06814001	9.98887032	8.23238167	8.660375	4.56525506
Fam168b	311.338469	0.25972701	0.25454468	1.02035921	0.30755813	0.99938111		Fam168b	284.947643	250.69814	266.86831	316.425422	391.230754	244.619341	258.848986	477.069154
Asna1	66.3024414	-0.2936966	0.28786869	-1.0202452	0.30761218	0.99938111		Asna1	67.2046328	64.7108918	61.0711746	107.543281	49.9443516	44.6900719	82.7546944	52.5004332
Eed	6.56642824	0.4133786	0.40527697	1.01999035	0.30773304	0.99938111		Eed	4.7043243	5.88280835	8.58813393	0	6.65924688	10.5844907	11.5471667	4.56525506
Gm11549	8.84279757	-0.4206293	0.41241118	-1.019927	0.30776309	0.99938111		Gm11549	10.7527413	15.8383302	15.5858727	0	9.98887032	2.35210905	4.81131944	11.4131377
Syp	760.455283	0.33109287	0.32465732	1.01982259	0.30781261	0.99938111		Syp	469.760384	875.180872	594.171636	674.213643	943.948245	896.153547	347.377264	1282.83667
Trub2	18.9174267	-0.3300434	0.32363498	-1.0198012	0.30782273	0.99938111		Trub2	22.8495752	19.4585199	26.0824808	16.5451201	18.3129289	10.5844907	19.2452778	18.2610202
Acot5	0.90647171	-0.3484179	0.34182048	-1.0193007	0.30806022	NA		Acot5	2.68818531	2.26261859	0.63615807	0	1.66481172	0	0	0
Ankrd10	11.9145345	0.37787267	0.37085895	1.01891209	0.3082447	0.99938111		Ankrd10	8.06455594	5.43028463	9.86045007	18.6132601	13.3184938	16.4647633	14.4339583	9.13051012
Rinl	1.40590589	-0.3640931	0.35734505	-1.0188839	0.3082581	NA		Rinl	0.67204633	1.81009488	0.63615807	6.20442003	0	0	1.92452778	0
Pagr1a	14.7209262	0.38111349	0.37409575	1.0187592	0.3083173	0.99938111		Pagr1a	17.4732045	6.33533207	11.1327662	12.4088401	21.6425524	11.7605452	9.62263889	27.3915304
Foxp4	53.8875281	-0.3735864	0.36670964	-1.0187525	0.30832049	0.99938111		Foxp4	63.1723549	44.7998482	41.3502745	103.407001	61.5980337	23.5210905	20.2075417	73.044081
Hmgxb4	11.5231649	0.37519728	0.36829518	1.01874068	0.3083261	0.99938111		Hmgxb4	11.4247876	8.59795066	11.7689243	6.20442003	14.9833055	21.1689814	13.4716944	4.56525506
Syngap1	218.678246	0.33057659	0.32450339	1.01871537	0.30833812	0.99938111		Syngap1	211.694593	188.702391	170.172283	179.928181	439.510294	155.239197	127.981097	276.197931
Uncx	1.44097087	-0.3110789	0.30540164	-1.0185894	0.30839796	NA		Uncx	0	0	1.59039517	8.27256004	1.66481172	0	0	0
Msantd4	50.702523	0.38277322	0.37579818	1.01856061	0.30841161	0.99938111		Msantd4	53.7637063	44.3473245	47.0756971	20.6814001	29.966611	88.2040893	101.037708	20.5436478
Cpsf6	88.2539706	0.24291771	0.23856731	1.01823552	0.30856605	0.99938111		Cpsf6	69.8928182	81.4542694	78.8836006	93.0663005	73.2517157	122.309671	98.1509166	89.0224737
Gm5803	2.44455109	0.40306457	0.39596312	1.01793462	0.30870903	NA		Gm5803	3.36023164	0	0.63615807	2.06814001	3.32962344	2.35210905	0.96226389	6.84788259
Rpain	3.41398808	-0.4234775	0.41602675	-1.0179093	0.30872106	NA		Rpain	5.37637063	4.52523719	5.40734359	2.06814001	1.66481172	1.17605452	4.81131944	2.28262753
Ercc5	8.81399972	0.39484815	0.38790557	1.01789759	0.30872663	0.99938111		Ercc5	7.39250961	9.95552182	8.2700549	2.06814001	8.3240586	16.4647633	13.4716944	4.56525506
Shq1	3.20178792	0.42282839	0.41539759	1.01788839	0.308731	NA		Shq1	2.68818531	2.71514231	1.90847421	2.06814001	3.32962344	3.52816357	4.81131944	4.56525506
Timmdc1	20.1065384	-0.3324153	0.32664123	-1.017677	0.30883147	0.99938111		Timmdc1	25.5377605	19.4585199	20.6751372	24.8176801	13.3184938	12.9365998	14.4339583	29.6741579
Aktip	47.9653539	-0.3695189	0.36311369	-1.0176397	0.30884922	0.99938111		Aktip	69.2207718	71.0462239	58.2084633	26.8858201	33.2962344	48.2182355	65.4339444	11.4131377
Pcca	11.8602005	0.38469734	0.3780467	1.01759211	0.30887184	0.99938111		Pcca	15.4570656	9.05047438	7.95197586	6.20442003	4.99443516	21.1689814	16.3584861	13.6957652
4930520O04Rik	0.4540565	0.2804985	0.27569129	1.01743692	0.30894563	NA		4930520O04R	0	0	0.31807903	0	0	2.35210905	0.96226389	0
Gm3002	0.4540565	0.2804985	0.27569129	1.01743692	0.30894563	NA		Gm3002	0	0	0.31807903	0	0	2.35210905	0.96226389	0
Pram1	0.4540565	0.2804985	0.27569129	1.01743692	0.30894563	NA		Pram1	0	0	0.31807903	0	0	2.35210905	0.96226389	0
Snord116	0.4540565	0.2804985	0.27569129	1.01743692	0.30894563	NA		Snord116	0	0	0.31807903	0	0	2.35210905	0.96226389	0
Snord116l1	0.4540565	0.2804985	0.27569129	1.01743692	0.30894563	NA		Snord116l1	0	0	0.31807903	0	0	2.35210905	0.96226389	0
Snord116l2	0.4540565	0.2804985	0.27569129	1.01743692	0.30894563	NA		Snord116l2	0	0	0.31807903	0	0	2.35210905	0.96226389	0
Ifit1	1.27115405	-0.3752754	0.36885072	-1.0174182	0.30895454	NA		Ifit1	1.34409266	1.81009488	0.9542371	4.13628002	0	0	1.92452778	0
Ccdc73	3.91519957	0.42271718	0.41556635	1.01720742	0.30905477	NA		Ccdc73	2.01613899	1.35757116	4.45310648	4.13628002	4.99443516	8.23238167	3.84905555	2.28262753
Prr13	63.9703248	-0.366256	0.36009496	-1.0171095	0.30910134	0.99938111		Prr13	64.5164475	70.1411764	59.1627004	103.407001	49.9443516	18.8168724	38.4905555	107.283494
Zwilch	1.19627239	0.36198333	0.3559073	1.01707193	0.30911921	NA		Zwilch	0.67204633	0	1.59039517	0	0	1.17605452	3.84905555	2.28262753
Wdr12	8.79304634	0.38003784	0.37370158	1.0169554	0.30917465	0.99938111		Wdr12	8.06455594	8.59795066	6.99773876	4.13628002	8.3240586	7.05632714	13.4716944	13.6957652
Ern1	3.52295542	-0.4234235	0.41637046	-1.0169395	0.30918223	NA		Ern1	6.04841696	6.78785578	4.77118552	0	1.66481172	4.7042181	1.92452778	2.28262753
Rexo2	21.5727312	-0.3478301	0.34215419	-1.0165886	0.30934917	0.99938111		Rexo2	30.9141311	30.7716129	26.7186389	10.3407001	19.9777406	16.4647633	25.981125	11.4131377
Txnip	4.36118871	-0.4224625	0.41561998	-1.0164633	0.30940881	NA		Txnip	6.04841696	9.95552182	5.40734359	0	4.99443516	2.35210905	3.84905555	2.28262753
Pde4c	0.48842732	0.28252022	0.27800209	1.01625213	0.30950934	NA		Pde4c	0	0	0.31807903	0	1.66481172	0	1.92452778	0
Efna5	31.3941079	0.33775433	0.33242298	1.01603787	0.30961135	0.99938111		Efna5	18.1452509	27.1514231	19.0847421	45.4990802	23.3073641	41.1619083	28.8679167	47.9351782
Abhd1	1.05571421	-0.3569292	0.35130321	-1.0160147	0.30962237	NA		Abhd1	2.68818531	0.45252372	0.9542371	2.06814001	0	0	0	2.28262753
Pak1	340.405732	-0.2808753	0.27645173	-1.0160014	0.30962872	0.99938111		Pak1	411.964399	443.925768	418.910088	237.836101	243.062511	326.943158	421.471583	219.132243
Csl	26.7380246	0.30376615	0.2989923	1.01596648	0.30964535	0.99938111		Csl	26.2098068	23.5312334	27.672876	14.4769801	39.9554813	29.4013631	29.8301805	22.8262753
AI197445	1.54702947	-0.4029773	0.39670626	-1.0158077	0.30972098	NA		AI197445	2.68818531	2.71514231	1.59039517	2.06814001	0	2.35210905	0.96226389	0
Gpalpp1	37.8589021	0.38579731	0.37980708	1.01577174	0.30973809	0.99938111		Gpalpp1	37.6345944	35.2968501	42.6225906	6.20442003	21.6425524	68.2111624	61.5848889	29.6741579
Rnf182	9.69772109	-0.4007649	0.39458899	-1.0156515	0.30979536	0.99938111		Rnf182	10.0806949	9.05047438	12.4050823	16.5451201	13.3184938	2.35210905	11.5471667	2.28262753
Lrrc57	16.898733	-0.3770305	0.3713179	-1.0153847	0.30992247	0.99938111		Lrrc57	21.5054825	22.6261859	23.5378486	12.4088401	4.99443516	11.7605452	26.9433889	11.4131377
Garem	35.2455347	0.31221046	0.30756312	1.01511018	0.31005331	0.99938111		Garem	38.3066407	32.129184	36.2610099	14.4769801	34.9610461	39.9858538	53.8867778	31.9567854
Man1a	19.6427673	0.40092215	0.39497301	1.01506214	0.31007621	0.99938111		Man1a	10.7527413	20.8160911	23.2197695	6.20442003	19.9777406	50.5703445	9.62263889	15.9783927
St6galnac2	0.92047608	-0.339466	0.33445144	-1.0149933	0.31010904	NA		St6galnac2	0.67204633	4.07271347	0.9542371	0	1.66481172	0	0	0
Cd38	1.61321287	-0.383789	0.37827203	-1.0145847	0.31030384	NA		Cd38	0.67204633	3.62018975	1.59039517	4.13628002	0	0	2.88679167	0
Pcnx	120.689791	0.3387898	0.33393611	1.01453478	0.31032764	0.99938111		Pcnx	67.8766792	166.528729	111.96382	64.1123403	146.503431	214.041923	89.4905416	105.000866
1110037F02Rik	30.1630361	0.32501707	0.32038837	1.01444717	0.31036943	0.99938111		1110037F02Ri	29.5700384	30.3190892	31.4898244	10.3407001	39.9554813	44.6900719	29.8301805	25.1089028
Prdx1	20.9473412	-0.3947163	0.38913861	-1.0143334	0.31042369	0.99938111		Prdx1	25.5377605	23.5312334	28.6271131	24.8176801	8.3240586	11.7605452	40.4150833	4.56525506
Mal2	87.4942113	-0.3192513	0.31474267	-1.0143248	0.31042779	0.99938111		Mal2	63.1723549	148.880304	117.689243	66.1804803	54.9387868	87.0280348	98.1509166	63.9135709
1700120C14Rik	1.37825669	-0.3255805	0.32098555	-1.014315	0.31043248	NA		1700120C14Ri	0	0.45252372	0.63615807	8.27256004	1.66481172	0	0	0
Bod1l	69.9128832	0.39150843	0.38599454	1.01428489	0.31044683	0.99938111		Bod1l	59.8121232	70.1411764	68.0689134	26.8858201	28.3017992	178.760288	88.5282777	38.804668

GeneID	Base mean	log2(FC)	StdErr	Wald-Stats	P-value	P-adj		GeneID	Normalised expression for Chow#1	Normalised expression for Chow#2	Normalised expression for Chow#3	Normalised expression for Chow#4	Normalised expression for HFD#1	Normalised expression for HFD#2	Normalised expression for HFD#3	Normalised expression for HFD#4
Oxnad1	11.4154175	-0.3641551	0.35903884	-1.0142498	0.31046357	0.99938111		Oxnad1	16.1291119	13.1231878	16.5401098	6.20442003	9.98887032	10.5844907	9.62263889	9.13051012
Neu4	3.26311191	-0.4222681	0.41635974	-1.0141905	0.31049184	NA		Neu4	2.68818531	5.43028463	3.81694841	4.13628002	3.32962344	1.17605452	0.96226389	4.56525506
Arhgap27	11.0942951	-0.4197488	0.41390119	-1.0141281	0.31052163	0.99938111		Arhgap27	13.4409266	7.69290322	6.67965972	28.9539601	9.98887032	0	10.5849028	11.4131377
Scarb2	44.8021335	-0.3504509	0.34562828	-1.0139533	0.31060503	0.99938111		Scarb2	36.9625481	83.2643643	54.7095939	31.0221002	33.2962344	63.5069443	25.981125	29.6741579
Dok7	4.19163266	-0.422084	0.41631854	-1.0138486	0.310655	NA		Dok7	2.01613899	3.16766603	5.08926455	12.4088401	3.32962344	2.35210905	2.88679167	2.28262753
Dpy19l1	48.4456398	-0.3527095	0.34789713	-1.0138327	0.31066258	0.99938111		Dpy19l1	43.6830113	82.3593168	64.570044	33.0902402	38.2906696	72.9153805	29.8301805	22.8262753
Rab3il1	4.29274455	-0.4209742	0.41532799	-1.0135946	0.31077623	NA		Rab3il1	6.72046328	1.81009488	5.72542262	8.27256004	3.32962344	2.35210905	3.84905555	2.28262753
Fam101b	5.5885742	-0.4080985	0.40267306	-1.0134736	0.31083399	0.99938111		Fam101b	8.73660227	6.33533207	6.99773876	6.20442003	4.99443516	4.7042181	6.73584722	0
Snrpe	4.16570532	0.42187615	0.41630208	1.01338948	0.31087416	NA		Snrpe	2.01613899	1.35757116	5.08926455	4.13628002	8.3240586	4.7042181	7.69811111	0
Anapc11	43.6987258	-0.3879057	0.38288387	-1.0131158	0.31100485	0.99938111		Anapc11	37.6345944	41.6321821	35.9429309	93.0663005	41.620293	8.23238167	29.8301805	61.6309433
Thap2	10.6238702	-0.4041163	0.39891632	-1.0130354	0.31104326	0.99938111		Thap2	8.73660227	20.8160911	14.3135566	8.27256004	1.66481172	14.1126543	12.5094306	4.56525506
Rela	12.7518087	-0.3610922	0.35646458	-1.012982	0.31106877	0.99938111		Rela	15.4570656	14.480759	13.6773985	14.4769801	16.6481172	5.88027262	7.69811111	13.6957652
Apitd1	2.38731213	0.4086011	0.403376	1.01295343	0.3110824	NA		Apitd1	1.34409266	1.35757116	0.31807903	4.13628002	0	3.52816357	3.84905555	4.56525506
Rpia	2.95339279	-0.4123546	0.40708556	-1.0129432	0.31108729	NA		Rpia	10.0806949	0.90504744	3.18079035	2.06814001	3.32962344	1.17605452	2.88679167	0
Ube2f	16.5131021	-0.3654511	0.36078653	-1.012929	0.31109409	0.99938111		Ube2f	22.8495752	21.7211385	18.130505	14.4769801	6.65924688	8.23238167	24.0565972	15.9783927
Tma7	15.142537	-0.3971973	0.39212849	-1.0129264	0.31109532	0.99938111		Tma7	20.1613899	14.9332827	22.9016905	16.5451201	3.32962344	11.7605452	26.9433889	4.56525506
Ston1	2.18042207	-0.4128219	0.40755772	-1.0129164	0.31110009	NA		Ston1	2.68818531	3.62018975	5.40734359	0	1.66481172	1.17605452	2.88679167	0
Pdpk1	168.174474	0.20682285	0.20420152	1.01283696	0.31113804	0.99938111		Pdpk1	137.097451	147.070209	156.176806	184.064461	188.123724	154.063143	203.03768	175.76232
Aph1b	20.7642622	-0.3954102	0.39050439	-1.0125629	0.311269	0.99938111		Aph1b	14.1129729	35.7493738	28.3090341	22.7495401	4.99443516	34.1055812	19.2452778	6.84788259
Mterf1a	1.12743016	-0.3697966	0.36543367	-1.0119391	0.31156718	NA		Mterf1a	0.67204633	1.81009488	2.54463228	2.06814001	0	0	1.92452778	0
Trappc9	69.9834831	0.3463412	0.34226448	1.01191101	0.31158061	0.99938111		Trappc9	49.7314283	38.4645161	53.4372778	97.2025805	123.196067	54.0985081	43.301875	100.435611
Htt	164.804214	0.26937235	0.26620352	1.01190379	0.31158405	0.99938111		Htt	125.672663	128.96926	141.227091	196.473301	248.056946	196.401106	124.132042	157.5013
Slc25a30	3.98103282	0.41995743	0.41502382	1.01188755	0.31159182	NA		Slc25a30	2.01613899	4.52523719	2.54463228	2.06814001	8.3240586	5.88027262	1.92452778	4.56525506
Zfp219	21.4135225	0.37864946	0.37427348	1.01169194	0.31168537	0.99938111		Zfp219	15.4570656	12.2181404	13.9954775	28.9539601	28.3017992	9.40843619	17.32075	45.6525506
Coro1c	75.3881768	0.32431134	0.32065069	1.01141632	0.31181721	0.99938111		Coro1c	67.8766792	44.7998482	61.0711746	86.8618804	123.196067	45.8661264	61.5848889	111.848749
1500009C09Rik	110.035928	-0.335297	0.33168736	-1.0108826	0.31207265	0.99938111		1500009C09Ri	102.151042	102.27036	94.4694732	204.745861	108.212762	51.7463991	68.3207361	148.37079
Kif5b	126.114916	0.3876565	0.38356843	1.010658	0.31218015	0.99938111		Kif5b	124.328571	110.415787	113.872294	59.9760603	69.9220923	258.731995	232.867861	38.804668
Mrps16	8.76898227	-0.4026197	0.39841966	-1.0105417	0.31223583	0.99938111		Mrps16	10.7527413	8.59795066	6.36158069	18.6132601	8.3240586	2.35210905	10.5849028	4.56525506
Nhp2l1	55.8379193	-0.3198367	0.3165066	-1.0105213	0.31224561	0.99938111		Nhp2l1	56.4518916	58.8280835	63.9338859	74.4530404	23.3073641	32.9295267	68.3207361	68.4788259
Tpcn2	0.72399056	-0.3382228	0.33474268	-1.0103963	0.31230544	NA		Tpcn2	2.01613899	0.90504744	1.90847421	0	0	0	0.96226389	0
Mdga1	23.8418993	-0.3354084	0.33199323	-1.0102869	0.31235787	0.99938111		Mdga1	20.1613899	39.3695635	27.0367179	20.6814001	28.3017992	21.1689814	13.4716944	20.5436478
Arhgap28	1.24552089	0.36498349	0.36130769	1.0101736	0.31241213	NA		Arhgap28	1.34409266	0	0.9542371	0	0	1.17605452	1.92452778	4.56525506
Slc8a1	244.153541	0.38931965	0.38543044	1.01009058	0.31245189	0.99938111		Slc8a1	175.404092	316.31408	218.838376	76.5211804	194.782971	609.196243	261.735778	100.435611
Cend1	218.034409	0.22914072	0.22689995	1.00987558	0.3125549	0.99938111		Cend1	237.232354	174.221632	195.618606	190.268881	181.464478	214.041923	293.490486	257.936911
Myo5b	50.8669944	0.3647218	0.36129406	1.0094874	0.31274094	0.99938111		Myo5b	55.7798453	44.7998482	56.6180681	10.3407001	33.2962344	84.6759257	71.2075278	50.2178057
9430091E24Rik	3.73277542	0.42023461	0.416359	1.00930836	0.31282677	NA		9430091E24Ri	2.68818531	0.90504744	2.22655324	6.20442003	3.32962344	7.05632714	2.88679167	4.56525506
Anapc2	57.7459582	0.28456016	0.28193786	1.00930098	0.31283031	0.99938111		Anapc2	44.3550577	39.8220873	46.7576181	76.5211804	81.5757743	50.5703445	53.8867778	68.4788259
Grin1os	48.908569	0.32072051	0.31779144	1.00921695	0.3128706	0.99938111		Grin1os	32.9302701	52.0402277	38.1694841	45.4990802	66.5924688	69.3872169	25.0188611	61.6309433
Ssb	60.2135529	0.39928748	0.3957879	1.00884207	0.31305038	0.99938111		Ssb	59.8121232	45.7048956	62.6615698	22.7495401	19.9777406	112.901234	132.792417	25.1089028
Arid1b	73.2837828	0.24512376	0.24310739	1.00829419	0.31331325	0.99938111		Arid1b	69.8928182	75.1189373	70.2954666	47.5672202	99.8887032	79.9717076	75.0565833	68.4788259
Itga1	0.87005932	-0.3427852	0.33997552	-1.0082643	0.31332759	NA		Itga1	0.67204633	2.71514231	1.90847421	0	1.66481172	0	0	0
Zfp91Cntf	21.6515757	0.35788613	0.35495811	1.00824891	0.31333498	0.99938111		Zfp91Cntf	26.2098068	18.1009488	19.7209001	8.27256004	16.6481172	38.8097993	31.7547083	13.6957652
Ube2a	39.4584282	-0.2779408	0.27568137	-1.0081958	0.31336046	0.99938111		Ube2a	39.6507334	47.0624668	40.7141164	49.6353602	26.6369875	30.5774176	47.1509305	34.239413
Dpagt1	9.62843829	-0.3901301	0.38695962	-1.0081933	0.31336169	0.99938111		Dpagt1	9.4086486	14.0282353	11.1327662	10.3407001	11.653682	9.40843619	1.92452778	9.13051012
Lrrc14b	10.6137497	0.39389334	0.39075331	1.00803585	0.31343725	0.99938111		Lrrc14b	6.72046328	12.6706641	10.1785291	4.13628002	13.3184938	21.1689814	14.4339583	2.28262753
Cox6c	52.6814931	-0.3148316	0.31266958	-1.0069146	0.31397579	0.99938111		Cox6c	54.4357526	41.6321821	59.7988585	86.8618804	33.2962344	56.4506172	61.5848889	27.3915304
Isy1	10.1667445	0.3758093	0.37328156	1.00677165	0.31404452	0.99938111		Isy1	11.4247876	6.78785578	7.63389683	8.27256004	8.3240586	8.23238167	19.2452778	11.4131377
Isg20l2	6.91218883	-0.4019112	0.39923575	-1.0067013	0.31407832	0.99938111		Isg20l2	4.03227797	9.05047438	6.99773876	14.4769801	6.65924688	4.7042181	4.81131944	4.56525506
Txlng	13.0173815	0.35224617	0.3500119	1.0063834	0.31423118	0.99938111		Txlng	12.7688802	14.480759	9.54237104	6.20442003	9.98887032	18.8168724	16.3584861	15.9783927
Rragc	63.5231088	-0.23499	0.23351401	-1.0063207	0.31426132	0.99938111		Rragc	62.5003085	69.236129	73.794336	72.3849004	46.6147282	61.1548353	65.4339444	57.0656883
Zmat1	15.3643859	0.39207499	0.38961679	1.00630929	0.31426681	0.99938111		Zmat1	15.4570656	9.5029981	19.7209001	4.13628002	11.653682	30.5774176	25.0188611	6.84788259
Vhl	32.8449579	-0.2956863	0.29386186	-1.0062085	0.31431526	0.99938111		Vhl	34.2743627	34.3918026	35.9429309	43.4309402	38.2906696	17.6408179	33.6792361	25.1089028
Arhgap36	9.65993176	-0.4080725	0.40568838	-1.0058767	0.31447486	0.99938111		Arhgap36	4.03227797	11.313093	10.8146872	22.7495401	11.653682	10.5844907	3.84905555	2.28262753
Zfp60	25.5854844	0.35382074	0.35182821	1.00566335	0.31457754	0.99938111		Zfp60	18.1452509	24.4362808	30.8536663	12.4088401	21.6425524	41.1619083	42.3396111	13.6957652
Ppp1r13b	55.060743	-0.2653038	0.26382101	-1.0056205	0.31459817	0.99938111		Ppp1r13b	71.2369108	60.6381783	56.9361472	53.7716403	68.2572805	51.7463991	41.3773472	36.5220405
Slc16a8	0.96400514	0.35855983	0.35660076	1.00549374	0.31465916	NA		Slc16a8	0.67204633	0	0.9542371	0	1.66481172	1.17605452	0.96226389	2.28262753
1700018L02Rik	4.02834191	-0.4121355	0.40995027	-1.0053305	0.31473773	NA		1700018L02Ri	4.7043243	1.35757116	2.22655324	14.4769801	3.32962344	0	3.84905555	2.28262753
Ppp4c	33.6804791	-0.4080158	0.40591728	-1.0051698	0.31481508	0.99938111		Ppp4c	18.8172972	22.6261859	18.130505	107.543281	21.6425524	9.40843619	27.9056528	43.3699231
Eif4g2	572.586494	-0.3123323	0.31083633	-1.0048129	0.31498697	0.99938111		Eif4g2	703.632506	760.239848	722.039408	401.219162	298.001298	603.315971	754.414889	337.828875
Thoc1	22.9257119	0.35091734	0.34931017	1.00460099	0.31508902	0.99938111		Thoc1	25.5377605	17.648425	22.2655324	12.4088401	21.6425524	28.2253086	44.2641389	11.4131377
Anxa7	52.6890358	0.24706951	0.24598762	1.00439813	0.31518675	0.99938111		Anxa7	50.4034746	54.75537	46.12146	39.2946602	58.2684102	63.5069443	63.5094166	45.6525506
9330188P03Rik	0.90932442	0.34842984	0.34700584	1.00410365	0.31532865	NA		9330188P03Ri	0.67204633	0.45252372	0.63615807	0	1.66481172	0	3.84905555	0
Plxna4	120.172459	0.34958838	0.3481953	1.00400086	0.3153782	0.99938111		Plxna4	73.2530498	162.456015	116.735006	51.7035003	153.162678	225.802469	84.6792222	93.5877288
Ppap2c	0.6236658	-0.2840158	0.28290384	-1.0039304	0.31541216	NA		Ppap2c	2.01613899	0.90504744	0	2.06814001	0	0	0	0
Rfx1	7.94430206	-0.410449	0.4089656	-1.0036272	0.31555833	0.99938111		Rfx1	8.73660227	7.69290322	4.77118552	18.6132601	11.653682	2.35210905	2.88679167	6.84788259
Thoc3	22.9522889	0.30918554	0.3081503	1.00335952	0.31568743	0.99938111		Thoc3	16.8011582	18.5534725	20.0389792	24.8176801	29.966611	16.4647633	25.0188611	31.9567854
Mettl5	4.80964622	0.41116642	0.40980433	1.00332375	0.31570468	0.99938111		Mettl5	3.36023164	1.81009488	4.45310648	6.20442003	3.32962344	7.05632714	7.69811111	4.56525506
Fam160a2	117.597804	0.26613481	0.26531724	1.00308145	0.31582156	0.99938111		Fam160a2	94.086486	93.2198861	87.4717345	148.906081	161.486737	105.844907	99.1131805	150.653417
Paip2	85.8108462	0.37423013	0.37310672	1.00301097	0.31585557	0.99938111		Paip2	82.6616984	74.6664136	83.9728651	41.3628002	39.9554813	150.534979	172.245236	41.0872956
Styx	4.25721615	0.41391367	0.4126824	1.00298357	0.31586879	NA		Styx	4.03227797	4.07271347	4.13502745	0	4.99443516	5.88027262	8.660375	2.28262753
Lcor	4.16809005	-0.4171114	0.41597879	-1.0027227	0.31599467	NA		Lcor	9.4086486	2.71514231	7.31581779	2.06814001	4.99443516	5.88027262	0.96226389	0
Zdhhc6	10.0675911	-0.3961368	0.39510702	-1.0026062	0.31605088	0.99938111		Zdhhc6	6.72046328	19.9110436	15.5858727	6.20442003	4.99443516	12.9365998	9.62263889	4.56525506
Traf7	19.1924983	-0.3320284	0.33121967	-1.0024418	0.31613026	0.99938111		Traf7	23.5216215	19.4585199	20.6751372	22.7495401	21.6425524	8.23238167	14.4339583	22.8262753
Kcns2	17.0037824	-0.3762513	0.37538399	-1.0023106	0.31619361	0.99938111		Kcns2	12.0968339	30.3190892	26.7186389	10.3407001	13.3184938	23.5210905	10.5849028	9.13051012
Gse1	52.603369	-0.3107166	0.31003448	-1.0022001	0.31624695	0.99938111		Gse1	47.7152893	54.3028463	48.0299342	88.9300204	71.586904	36.4576902	32.7169722	41.0872956
Slc35f4	10.0556029	0.38167425	0.38086694	1.00211967	0.3162858	0.99938111		Slc35f4	6.72046328	12.6706641	6.36158069	6.20442003	4.99443516	11.7605452	13.4716944	18.2610202
Aldh3a1	0.38757279	0.2393373	0.23884844	1.00204671	0.31632103	NA		Aldh3a1	0	0	0	0	0	1.17605452	1.92452778	0
Olfr539	0.38757279	0.2393373	0.23884844	1.00204671	0.31632103	NA		Olfr539	0	0	0	0	0	1.17605452	1.92452778	0
Otogl	0.38757279	0.2393373	0.23884844	1.00204671	0.31632103	NA		Otogl	0	0	0	0	0	1.17605452	1.92452778	0
Lat2	1.00970543	0.34744113	0.34673469	1.00203741	0.31632552	NA		Lat2	0	0.45252372	0.31807903	2.06814001	0	2.35210905	2.88679167	0
Mllt6	134.370882	0.37757634	0.37682751	1.00198721	0.31634977	0.99938111		Mllt6	84.0057911	78.7391271	79.8378377	198.541441	274.693934	95.2604165	53.8867778	210.001733
C230052I12Rik	2.93963491	-0.4169225	0.41619802	-1.0017408	0.31646879	NA		C230052I12Ri	4.03227797	4.07271347	3.18079035	4.13628002	4.99443516	1.17605452	1.92452778	0
Fam169a	60.7754354	0.3266492	0.32609678	1.00169404	0.31649138	0.99938111		Fam169a	55.7798453	54.75537	60.4350166	39.2946602	46.6147282	101.140689	96.2263889	31.9567854
Ptgfrn	16.1359209	-0.3838805	0.38326225	-1.0016132	0.31653045	0.99938111		Ptgfrn	12.7688802	28.0564706	18.766663	16.5451201	19.9777406	19.9929269	3.84905555	9.13051012
D030056L22Rik	15.1044861	0.32488307	0.32440653	1.00146896	0.31660014	0.99938111		D030056L22Ri	14.7850192	11.313093	13.3593194	12.4088401	18.3129289	14.1126543	18.2830139	18.2610202
Ehd1	36.1735526	0.35553233	0.35506716	1.00131008	0.31667692	0.99938111		Ehd1	32.9302701	19.0059962	24.8101647	45.4990802	59.9332219	16.4647633	33.6792361	57.0656883
Speg	64.3961306	0.3697831	0.3693502	1.00117205	0.31674363	0.99938111		Speg	63.1723549	47.0624668	53.4372778	47.5672202	149.833055	42.3379629	25.0188611	86.7398462
Cntnap1	153.095975	0.33263614	0.33229257	1.00103393	0.3168104	0.99938111		Cntnap1	113.575829	161.550968	132.320878	115.815841	271.36431	202.281378	63.5094166	164.349182
Myl6	41.5453231	0.38531797	0.38497738	1.00088469	0.31688256	0.99938111		Myl6	28.8979921	14.9332827	25.7644018	66.1804803	51.6091633	19.9929269	33.6792361	91.3051012
Ctu2	9.12678758	0.41250363	0.412262	1.00058609	0.31702696	0.99938111		Ctu2	6.72046328	2.71514231	3.18079035	16.5451201	16.6481172	3.52816357	7.69811111	15.9783927
Nucks1	211.393516	0.29315483	0.29300502	1.0005113	0.31706313	0.99938111		Nucks1	200.941852	165.171157	208.023689	173.723761	168.145984	276.372813	361.811222	136.957652
Ppp1r36	0.45250158	-0.2852114	0.28507656	-1.0004729	0.3170817	NA		Ppp1r36	0.67204633	1.35757116	1.59039517	0	0	0	0	0
Vav1	0.45250158	-0.2852114	0.28507656	-1.0004729	0.3170817	NA		Vav1	0.67204633	1.35757116	1.59039517	0	0	0	0	0
Itsn1	199.622836	0.28349737	0.28342905	1.00024106	0.31719386	0.99938111		Itsn1	126.34471	161.098444	161.26607	262.653781	221.419959	283.42914	143.377319	237.393263
Neurl1a	150.211825	0.37237292	0.37253335	0.99956935	0.31751896	0.99938111		Neurl1a	105.511274	121.72888	97.6502636	169.587481	331.297532	70.5632714	70.2452639	235.110636
Tgfb3	9.96062932	0.37989234	0.38010592	0.99943811	0.31758251	0.99938111		Tgfb3	8.73660227	12.2181404	8.2700549	2.06814001	13.3184938	17.6408179	10.5849028	6.84788259
Zfp385a	169.407114	0.37485961	0.37508822	0.9993905	0.31760556	0.99938111		Zfp385a	130.376988	78.2866034	94.1513942	254.381221	204.771842	88.2040893	119.320722	385.764053
Tgfb1i1	10.8603635	-0.3888857	0.3891254	-0.9993839	0.31760877	0.99938111		Tgfb1i1	15.4570656	14.480759	13.9954775	6.20442003	18.3129289	3.52816357	5.77358333	9.13051012
Nudcd1	14.8151459	0.33868063	0.33891626	0.99930474	0.31764709	0.99938111		Nudcd1	14.7850192	12.6706641	12.4050823	10.3407001	8.3240586	21.1689814	18.2830139	20.5436478
Uhrf2	23.7499276	-0.3678157	0.36826499	-0.99878	0.31790126	0.99938111		Uhrf2	22.8495752	33.9392789	35.3067728	20.6814001	13.3184938	24.697145	34.6415	4.56525506
Erbb2	0.57171661	0.32314261	0.32354177	0.99876629	0.31790792	NA		Erbb2	0	0.45252372	0.31807903	0	1.66481172	1.17605452	0.96226389	0
Tmem29	12.1489246	-0.4003159	0.40081566	-0.9987532	0.31791428	0.99938111		Tmem29	12.0968339	15.3858064	14.6316356	18.6132601	0	9.40843619	20.2075417	6.84788259
Ext2	20.1693431	-0.345221	0.34580589	-0.9983087	0.31812968	0.99938111		Ext2	20.8334362	37.106945	19.4028211	14.4769801	13.3184938	23.5210905	14.4339583	18.2610202
Tob2	24.8220305	-0.3624369	0.36308365	-0.9982189	0.31817324	0.99938111		Tob2	26.8818531	28.961518	19.7209001	39.2946602	21.6425524	18.8168724	6.73584722	36.5220405
Apobec1	0.55197957	-0.3039757	0.30462475	-0.9978692	0.31834277	NA		Apobec1	0.67204633	1.35757116	0.31807903	2.06814001	0	0	0	0
Stab2	0.55197957	-0.3039757	0.30462475	-0.9978692	0.31834277	NA		Stab2	0.67204633	1.35757116	0.31807903	2.06814001	0	0	0	0
Ube3a	116.917644	0.28546317	0.28614942	0.99760176	0.31847251	0.99938111		Ube3a	79.3014668	95.4825047	97.0141055	146.837941	76.5813391	162.295524	156.849014	120.979259
Clmn	64.1492861	0.2810299	0.28171986	0.99755092	0.31849717	0.99938111		Clmn	49.059382	61.9957495	43.8949068	74.4530404	101.553515	54.0985081	59.6603611	68.4788259
R74862	7.86466894	-0.3863841	0.38738461	-0.9974173	0.31856198	0.99938111		R74862	7.39250961	7.2403795	8.90621297	14.4769801	8.3240586	5.88027262	3.84905555	6.84788259
Zfp72	2.49539732	-0.4079028	0.40911257	-0.9970429	0.31874368	NA		Zfp72	4.7043243	4.07271347	4.77118552	0	0	3.52816357	2.88679167	0
Snapc1	24.262645	0.34120178	0.3422228	0.99701651	0.3187565	0.99938111		Snapc1	16.8011582	23.9837571	21.9474534	20.6814001	9.98887032	39.9858538	35.6037639	25.1089028
Gsta4	11.5987159	-0.3798076	0.38099902	-0.9968728	0.31882623	0.99938111		Gsta4	18.1452509	18.1009488	13.9954775	4.13628002	13.3184938	7.05632714	13.4716944	4.56525506

GeneID	Base mean	log2(FC)	StdErr	Wald-Stats	P-value	P-adj		GeneID	Normalised expression for Chow#1	Normalised expression for Chow#2	Normalised expression for Chow#3	Normalised expression for Chow#4	Normalised expression for HFD#1	Normalised expression for HFD#2	Normalised expression for HFD#3	Normalised expression for HFD#4
Scd4	2.13460042	-0.3906767	0.39191174	-0.9968487	0.31883795	NA		Scd4	2.68818531	4.97776091	3.18079035	0	1.66481172	0	0	4.56525506
Ppip5k2	22.4591361	0.3818821	0.38314656	0.9966998	0.31891025	0.99938111		Ppip5k2	19.4893435	18.1009488	25.1282437	10.3407001	9.98887032	50.5703445	34.6415	11.4131377
Nsmf	173.684449	0.2708079	0.27171626	0.99665693	0.31893106	0.99938111		Nsmf	184.81274	149.332827	139.954775	144.769801	278.023557	119.957561	162.622597	210.001733
Rsl24d1	26.1753282	-0.3397542	0.34089459	-0.9966548	0.31893209	0.99938111		Rsl24d1	22.8495752	22.1736622	25.4463228	53.7716403	21.6425524	19.9929269	29.8301805	13.6957652
Krtcap2	30.914566	-0.3897065	0.39107169	-0.996509	0.31900288	0.99938111		Krtcap2	18.8172972	28.961518	24.4920857	76.5211804	36.6258578	10.5844907	12.5094306	38.804668
Uba5	29.6586901	0.27853874	0.27955546	0.99636305	0.31907378	0.99938111		Uba5	29.5700384	22.6261859	30.2175083	22.7495401	28.3017992	35.2816357	36.5660278	31.9567854
Iqsec1	334.122481	0.27046089	0.27148395	0.99623161	0.31913762	0.99938111		Iqsec1	285.61969	235.764858	253.190911	421.900562	474.47134	301.069958	262.698042	438.264486
Psip1	134.801923	0.29548153	0.29664413	0.99608085	0.31921087	0.99938111		Psip1	134.409266	104.080455	129.140088	107.543281	68.2572805	183.464506	196.301833	155.218672
Rimklb	27.6798644	0.34473895	0.34617246	0.99585897	0.31931867	0.99938111		Rimklb	11.4247876	22.6261859	23.2197695	39.2946602	16.6481172	36.4576902	37.5282917	34.239413
Arsg	4.02182491	-0.4145226	0.41637006	-0.9955628	0.3194626	NA		Arsg	2.68818531	7.69290322	8.2700549	2.06814001	3.32962344	2.35210905	5.77358333	0
Mcph1	11.4668022	0.39122771	0.39298203	0.99553586	0.31947571	0.99938111		Mcph1	5.37637063	11.7656167	10.4966081	10.3407001	9.98887032	16.4647633	25.0188611	2.28262753
Mir6516	0.58737553	0.28457154	0.28585572	0.99550761	0.31948944	NA		Mir6516	0	0	0.63615807	0	0	1.17605452	2.88679167	0
Traf3ip3	0.58737553	0.28457154	0.28585572	0.99550761	0.31948944	NA		Traf3ip3	0	0	0.63615807	0	0	1.17605452	2.88679167	0
Gpr55	2.0190467	0.38078135	0.38252123	0.99545156	0.31951669	NA		Gpr55	0	2.26261859	1.90847421	0	4.99443516	4.7042181	0	2.28262753
Ppt1	45.5524018	-0.3425546	0.34421587	-0.9951737	0.31965179	0.99938111		Ppt1	45.6991503	80.0966983	58.5265423	24.8176801	31.6314227	62.3308898	38.4905555	22.8262753
4632415L05Rik	20.8360327	0.3529578	0.35468969	0.99511716	0.31967928	0.99938111		4632415L05Ri	13.4409266	19.9110436	13.0412404	26.8858201	13.3184938	31.7534722	34.6415	13.6957652
Spink10	1.34631062	0.37904249	0.38117281	0.99441117	0.32002273	NA		Spink10	0.67204633	1.81009488	0.63615807	0	1.66481172	1.17605452	4.81131944	0
Igfbp3	10.8361792	0.39704423	0.39931846	0.99430471	0.32007454	0.99938111		Igfbp3	4.03227797	11.313093	10.8146872	8.27256004	16.6481172	4.7042181	24.0565972	6.84788259
Usp40	4.60408923	-0.4136207	0.41611849	-0.9939974	0.32022412	NA		Usp40	4.7043243	6.78785578	8.58813393	4.13628002	0	5.88027262	6.73584722	0
Lingo4	0.92360658	-0.3539496	0.35609915	-0.9939636	0.32024057	NA		Lingo4	1.34409266	1.35757116	0.9542371	2.06814001	1.66481172	0	0	0
Dbnl	36.7626999	0.30305481	0.30494866	0.9937896	0.32032531	0.99938111		Dbnl	33.6023164	24.8888045	27.990955	43.4309402	41.620293	23.5210905	44.2641389	54.7830607
Fam185a	14.1069643	0.34441867	0.34660526	0.99369141	0.32037313	0.99938111		Fam185a	10.0806949	9.95552182	12.0870033	16.5451201	8.3240586	17.6408179	15.3962222	22.8262753
Mark1	61.8558892	0.2659078	0.26766017	0.993453	0.32048924	0.99938111		Mark1	57.1239379	50.2301328	65.8423601	49.6353602	54.9387868	83.4998712	85.6414861	47.9351782
Reps1	24.157098	-0.3361804	0.33843157	-0.9933482	0.32054029	0.99938111		Reps1	36.2905017	32.5817078	26.0824808	16.5451201	18.3129289	23.5210905	30.7924444	9.13051012
Dap3	44.6969221	0.26429146	0.26610897	0.99317007	0.32062708	0.99938111		Dap3	47.043243	29.8665654	36.2610099	49.6353602	51.6091633	49.39429	48.1131944	45.6525506
Nckap5l	14.413035	0.39556373	0.39830963	0.99310614	0.32065823	0.99938111		Nckap5l	11.4247876	11.7656167	6.67965972	16.5451201	41.620293	11.7605452	8.660375	6.84788259
Daam2	39.8053719	-0.3823099	0.38496684	-0.9930983	0.32066204	0.99938111		Daam2	51.075521	55.6604174	31.4898244	49.6353602	63.2628454	15.2887088	8.660375	43.3699231
Larp1	128.150387	0.26084632	0.26266998	0.99305721	0.32068208	0.99938111		Larp1	116.936061	118.561214	99.5587378	124.088401	188.123724	117.605452	91.4150694	168.914437
Sec22c	83.8482744	0.39762664	0.40054559	0.99271257	0.32085005	0.99938111		Sec22c	32.2582238	52.9452751	41.0321955	138.565381	114.872009	56.4506172	26.9433889	207.719105
Ube2l3	130.328528	-0.2270326	0.22878061	-0.9923595	0.3210222	0.99938111		Ube2l3	140.457683	146.617685	134.865511	142.701661	111.542385	84.6759257	135.679208	146.088162
Drd5	1.70287318	-0.393555	0.39673028	-0.9919963	0.32119932	NA		Drd5	3.36023164	2.71514231	1.27231614	2.06814001	0	0	1.92452778	2.28262753
Fam120c	85.1873115	0.21945716	0.22127928	0.99176552	0.32131192	0.99938111		Fam120c	68.5487255	77.8340797	79.5197586	86.8618804	108.212762	79.9717076	84.6792222	95.8703563
C2cd5	42.7109955	0.28264321	0.28502433	0.99164588	0.3213703	0.99938111		C2cd5	37.6345944	37.5594687	29.8994292	47.5672202	31.6314227	41.1619083	50.0377222	66.1961984
Flot1	51.5712275	0.31313894	0.31585706	0.99139446	0.321493	0.99938111		Flot1	45.6991503	36.2018975	41.6683535	55.8397803	93.2294563	31.7534722	44.2641389	63.9135709
Zbtb39	14.5872578	0.35666814	0.35986621	0.99111317	0.32163032	0.99938111		Zbtb39	11.4247876	8.59795066	10.8146872	18.6132601	9.98887032	14.1126543	13.4716944	29.6741579
Gba	14.4870861	-0.3496509	0.35288666	-0.9908306	0.32176829	0.99938111		Gba	13.4409266	23.9837571	17.1762679	10.3407001	14.9833055	9.40843619	10.5849028	15.9783927
Khdrbs1	95.1058882	0.26600085	0.26861445	0.99027005	0.32204214	0.99938111		Khdrbs1	86.02193	64.7108918	72.5220199	119.952121	83.240586	97.6125255	95.264125	141.522907
Taf13	8.18031852	-0.4077233	0.41178486	-0.9901368	0.32210728	0.99938111		Taf13	14.1129729	9.95552182	9.54237104	8.27256004	0	5.88027262	15.3962222	2.28262753
Tead2	1.08942572	-0.3635261	0.36714752	-0.9901362	0.32210755	NA		Tead2	3.36023164	2.26261859	0.9542371	0	0	1.17605452	0.96226389	0
Piezo1	3.64075915	0.41201919	0.41620769	0.9899365	0.32220516	NA		Piezo1	2.01613899	4.97776091	2.54463228	0	8.3240586	7.05632714	1.92452778	2.28262753
Cyp26a1	0.42733267	0.27043834	0.27323826	0.98975281	0.32229496	NA		Cyp26a1	0	0	0.31807903	0	0	1.17605452	1.92452778	0
Nrg3	41.2212876	-0.3267864	0.33022098	-0.9895992	0.32237005	0.99938111		Nrg3	53.7637063	63.8058444	48.6660923	20.6814001	29.966611	54.0985081	33.6792361	25.1089028
Pdha1	137.794303	0.29085181	0.29395074	0.98945764	0.32243928	0.99938111		Pdha1	156.586795	132.58945	125.959298	70.3167603	108.212762	194.048996	198.226361	116.414004
Cox6b2	0.7284766	-0.3295027	0.33317518	-0.9889774	0.32267421	NA		Cox6b2	2.68818531	0.90504744	1.27231614	0	0	0	0.96226389	0
Mcm6	3.97770137	0.41161666	0.41631035	0.98872551	0.32279746	NA		Mcm6	3.36023164	2.26261859	3.49886938	2.06814001	1.66481172	1.17605452	8.660375	9.13051012
Mrgbp	2.41272507	0.4094864	0.41432324	0.98832591	0.32299306	NA		Mrgbp	2.68818531	1.35757116	1.90847421	0	3.32962344	3.52816357	1.92452778	4.56525506
Fbxl22	1.66587976	0.37456241	0.3791958	0.98778101	0.32325991	NA		Fbxl22	2.01613899	0.45252372	0.9542371	0	6.65924688	0	0.96226389	2.28262753
Cdc45	0.87903139	-0.3356619	0.3398456	-0.9876895	0.32330472	NA		Cdc45	2.01613899	2.71514231	0.63615807	0	1.66481172	0	0	0
Lyrm4	26.0602491	-0.3080228	0.31194569	-0.9874245	0.32343456	0.99938111		Lyrm4	32.9302701	32.5817078	27.990955	24.8176801	14.9833055	19.9929269	34.6415	20.5436478
Tmem70	27.0398542	-0.2957819	0.29959349	-0.9872775	0.3235066	0.99938111		Tmem70	28.2259458	28.5089943	29.8994292	35.1583802	13.3184938	29.4013631	22.1320694	29.6741579
Slc38a1	232.097212	0.31314863	0.31727502	0.98699428	0.32364544	0.99938111		Slc38a1	149.866331	249.340569	209.932163	198.541441	201.442218	481.0063	188.603722	178.044947
Ttc9	52.1683728	0.30068966	0.30468594	0.98688394	0.32369954	0.99938111		Ttc9	34.9464091	35.7493738	41.0321955	74.4530404	53.2739751	38.8097993	68.3207361	70.7614535
Mapk14	55.2548828	0.23300887	0.23624797	0.9862894	0.32399112	0.99938111		Mapk14	53.7637063	45.7048956	48.9841713	53.7716403	64.9276571	65.8590533	51.96225	57.0656883
Ticam1	7.71510808	-0.4073275	0.41302923	-0.9861953	0.3240373	0.99938111		Ticam1	6.04841696	6.33533207	6.99773876	20.6814001	13.3184938	3.52816357	4.81131944	0
Fam192a	29.7607496	0.33848163	0.34325563	0.98609201	0.32408796	0.99938111		Fam192a	23.5216215	19.9110436	16.2220308	43.4309402	29.966611	22.345036	27.9056528	54.7830607
Pthlh	5.48454072	0.39699917	0.40261222	0.98605842	0.32410445	0.99938111		Pthlh	3.36023164	3.16766603	6.04350166	4.13628002	4.99443516	8.23238167	4.81131944	9.13051012
Gpr173	15.0124495	-0.3551869	0.36047927	-0.9853184	0.32446768	0.99938111		Gpr173	10.7527413	24.8888045	14.6316356	18.6132601	8.3240586	11.7605452	10.5849028	20.5436478
Tspan2	44.3695553	-0.3543247	0.35962824	-0.9852527	0.32449997	0.99938111		Tspan2	36.2905017	85.5269829	51.5288036	33.0902402	18.3129289	64.6829988	40.4150833	25.1089028
Snrpd3	20.223811	0.35941761	0.36479965	0.98524657	0.32450297	0.99938111		Snrpd3	20.8334362	15.3858064	19.7209001	12.4088401	13.3184938	30.5774176	40.4150833	9.13051012
Commd10	12.1597752	-0.3771948	0.38305573	-0.9846994	0.32477174	0.99938111		Commd10	20.1613899	14.480759	19.7209001	2.06814001	11.653682	11.7605452	10.5849028	6.84788259
Plxna1	94.6865986	0.32588304	0.33095795	0.98466601	0.32478815	0.99938111		Plxna1	50.4034746	100.912789	65.5242811	109.611421	138.179373	127.013889	47.1509305	118.696632
Dlx6	11.6861776	0.34997476	0.35544011	0.98462371	0.32480894	0.99938111		Dlx6	10.7527413	11.313093	9.224292	8.27256004	6.65924688	15.2887088	18.2830139	13.6957652
Abcf3	30.9404726	0.31094201	0.31597234	0.98407985	0.32507626	0.99938111		Abcf3	38.3066407	17.1959013	22.5836114	31.0221002	44.9499164	35.2816357	30.7924444	27.3915304
Ndufb10	62.1151832	-0.2859298	0.29060365	-0.9839166	0.3251565	0.99938111		Ndufb10	88.7101153	60.6381783	55.027673	72.3849004	46.6147282	34.1055812	66.3962083	73.044081
Otulin	7.69927724	-0.3972786	0.40381203	-0.9838206	0.32520375	0.99938111		Otulin	14.1129729	4.52523719	6.04350166	14.4769801	3.32962344	5.88027262	8.660375	4.56525506
Proca1	0.48891397	-0.2751704	0.27972749	-0.9837088	0.32525873	NA		Proca1	2.68818531	0.90504744	0.31807903	0	0	0	0	0
Gimap6	0.88138954	-0.2928476	0.29770289	-0.9836909	0.3252675	NA		Gimap6	2.01613899	4.07271347	0	0	0	0	0.96226389	0
Rfng	46.1409384	-0.3123482	0.3176483	-0.9833146	0.32545262	0.99938111		Rfng	54.4357526	60.6381783	50.8926455	41.3628002	68.2572805	21.1689814	40.4150833	31.9567854
Zfp963	5.29244067	0.40526157	0.41219235	0.98318558	0.32551611	0.99938111		Zfp963	2.68818531	1.35757116	5.08926455	8.27256004	3.32962344	7.05632714	7.69811111	6.84788259
Tcea1	28.1654402	0.32654411	0.33228529	0.98272214	0.32574421	0.99938111		Tcea1	23.5216215	19.0059962	30.2175083	24.8176801	16.6481172	25.8731995	50.9999861	34.239413
Ngb	18.2351897	-0.4047175	0.41189939	-0.9825639	0.3258221	0.99938111		Ngb	12.0968339	5.88280835	6.67965972	72.3849004	18.3129289	0	7.69811111	22.8262753
Cnnm1	87.2956585	0.26064425	0.26527763	0.98253385	0.32583692	0.99938111		Cnnm1	65.8605402	95.029981	88.1078926	62.0442003	109.877574	118.781507	67.3584722	91.3051012
Prps2	23.7996795	-0.3235892	0.32940249	-0.9823521	0.32592644	0.99938111		Prps2	26.2098068	26.6988994	21.6293743	37.2265202	16.6481172	18.8168724	31.7547083	11.4131377
Tex10	12.4665933	-0.3667107	0.37354287	-0.9817097	0.32624289	0.99938111		Tex10	16.8011582	13.5757116	13.0412404	16.5451201	1.66481172	16.4647633	12.5094306	9.13051012
Pth2r	2.11036113	0.37250546	0.37946068	0.98167077	0.32626207	NA		Pth2r	0.67204633	2.26261859	0.31807903	2.06814001	3.32962344	8.23238167	0	0
Car8	6.9412415	-0.3847666	0.39196181	-0.9816431	0.32627569	0.99938111		Car8	9.4086486	8.14542694	8.58813393	8.27256004	3.32962344	5.88027262	9.62263889	2.28262753
Arhgef28	27.5698298	0.3374967	0.34383711	0.98155983	0.32631674	0.99938111		Arhgef28	26.2098068	34.3918026	19.7209001	12.4088401	49.9443516	28.2253086	33.6792361	15.9783927
Mrpl22	3.40599777	-0.4083028	0.4159935	-0.9815125	0.3263401	NA		Mrpl22	4.7043243	4.07271347	5.40734359	2.06814001	3.32962344	1.17605452	1.92452778	4.56525506
Rap1a	8.9997886	-0.4020806	0.40970169	-0.9813984	0.32639629	0.99938111		Rap1a	16.1291119	11.7656167	11.1327662	6.20442003	0	12.9365998	11.5471667	2.28262753
Stag2	38.5247628	-0.3749772	0.382132	-0.9812768	0.32645628	0.99938111		Stag2	53.7637063	63.3533207	50.8926455	14.4769801	11.653682	50.5703445	45.2264028	18.2610202
Cish	6.42625371	-0.3995105	0.40718382	-0.9811552	0.32651621	0.99938111		Cish	5.37637063	4.52523719	6.36158069	16.5451201	6.65924688	3.52816357	3.84905555	4.56525506
Itga11	1.55163251	0.37921155	0.38653346	0.98105751	0.32656438	NA		Itga11	0	1.81009488	1.27231614	0	1.66481172	1.17605452	1.92452778	4.56525506
Rbm28	12.1720668	0.37635279	0.38368364	0.9808935	0.32664526	0.99938111		Rbm28	15.4570656	12.6706641	8.90621297	2.06814001	11.653682	17.6408179	22.1320694	6.84788259
Fbxw4	13.8773502	0.36232232	0.36948868	0.98060465	0.32678774	0.99938111		Fbxw4	8.06455594	11.7656167	9.86045007	16.5451201	21.6425524	5.88027262	14.4339583	22.8262753
Mon1a	16.6459124	-0.3460801	0.35293265	-0.980584	0.32679792	0.99938111		Mon1a	18.1452509	22.1736622	16.8581888	18.6132601	19.9777406	4.7042181	14.4339583	18.2610202
Rpl7l1	20.8279075	-0.3191784	0.3255365	-0.980469	0.32685466	0.99938111		Rpl7l1	26.2098068	25.3413283	19.7209001	22.7495401	8.3240586	17.6408179	19.2452778	27.3915304
Eif3l	42.358015	-0.2621513	0.26747174	-0.9801084	0.32703263	0.99938111		Eif3l	50.4034746	49.7776091	41.0321955	47.5672202	31.6314227	36.4576902	50.0377222	31.9567854
Efnb1	15.0038053	0.34520149	0.35230653	0.9798328	0.32716866	0.99938111		Efnb1	12.0968339	13.1231878	11.7689243	12.4088401	26.6369875	10.5844907	10.5849028	22.8262753
Wtip	3.07284457	-0.4014666	0.40991238	-0.9793962	0.32738425	NA		Wtip	4.03227797	1.35757116	1.59039517	10.3407001	1.66481172	2.35210905	0.96226389	2.28262753
Phf21b	4.39050341	-0.404031	0.41256645	-0.9793113	0.32742619	NA		Phf21b	6.04841696	3.62018975	4.45310648	8.27256004	4.99443516	3.52816357	1.92452778	2.28262753
Vamp7	16.1076211	0.33268864	0.33983974	0.97895746	0.327601	0.99938111		Vamp7	16.1291119	11.313093	15.2677937	12.4088401	13.3184938	12.9365998	26.9433889	20.5436478
Myh7	7.56182161	0.38893851	0.39736896	0.97878433	0.32768655	0.99938111		Myh7	10.0806949	4.52523719	7.95197586	0	9.98887032	8.23238167	10.5849028	9.13051012
Bend5	3.93455489	-0.4066251	0.41552295	-0.9785863	0.32778443	NA		Bend5	9.4086486	3.16766603	5.40734359	2.06814001	8.3240586	1.17605452	1.92452778	0
Ankrd13d	38.4571131	0.35034231	0.3580263	0.97853792	0.32780834	0.99938111		Ankrd13d	30.9141311	23.9837571	24.8101647	49.6353602	43.2851047	29.4013631	21.1698055	84.4572186
Emx2os	3.11242778	-0.4066306	0.41556724	-0.9784952	0.32782944	NA		Emx2os	6.04841696	4.52523719	3.49886938	2.06814001	1.66481172	0	4.81131944	2.28262753
Arhgap19	3.01616532	-0.3939989	0.40267251	-0.97846	0.32784687	NA		Arhgap19	0.67204633	2.26261859	2.22655324	12.4088401	0	2.35210905	1.92452778	2.28262753
Mib1	94.3766201	0.22951857	0.2346474	0.9781424	0.3280039	0.99938111		Mib1	88.038069	85.5269829	93.1971571	78.5893204	119.866444	116.429398	104.886764	68.4788259
Galt	14.373828	-0.3481383	0.35593599	-0.9780924	0.32802864	0.99938111		Galt	16.8011582	15.3858064	15.5858727	18.6132601	19.9777406	4.7042181	12.5094306	11.4131377
Clec3b	0.74161205	-0.2748584	0.28105756	-0.9779436	0.32810224	NA		Clec3b	1.34409266	0.45252372	0	4.13628002	0	0	0	0
Lyve1	0.57180336	-0.250108	0.25574978	-0.97794	0.32810398	NA		Lyve1	0	3.62018975	0.9542371	0	0	0	0	0
AW495222	1.1227718	0.35576234	0.36382539	0.97783812	0.3281544	NA		AW495222	1.34409266	0.90504744	0.31807903	0	0	3.52816357	2.88679167	0
Acy3	1.3970476	-0.3664456	0.37477571	-0.977773	0.32818659	NA		Acy3	2.68818531	0.45252372	1.27231614	4.13628002	1.66481172	0	0.96226389	0
Fam114a1	4.82448162	0.40706433	0.4163229	0.97776108	0.32819251	0.99938111		Fam114a1	4.7043243	4.52523719	3.18079035	2.06814001	1.66481172	7.05632714	15.3962222	0
Cd164l2	1.28132451	0.34498264	0.35293728	0.97746159	0.32834069	NA		Cd164l2	0.67204633	0	0.31807903	2.06814001	1.66481172	0	0.96226389	4.56525506
Amigo2	4.92708572	-0.4047739	0.41411428	-0.9774449	0.32834895	0.99938111		Amigo2	4.7043243	10.4080455	4.45310648	4.13628002	6.65924688	3.52816357	0.96226389	4.56525506
Gps1	17.8171022	0.34723415	0.35534136	0.97718474	0.3284777	0.99938111		Gps1	14.7850192	10.4080455	15.9039517	18.6132601	26.6369875	8.23238167	18.2830139	29.6741579
Rangap1	149.072984	0.33813734	0.34605811	0.97711146	0.32851397	0.99938111		Rangap1	114.247876	94.1249335	95.1056313	204.745861	258.045817	102.316744	86.60375	237.393263
Timm8b	32.9113077	-0.3747359	0.38351866	-0.9770996	0.32851984	0.99938111		Timm8b	50.4034746	43.8948007	43.8949068	18.6132601	8.3240586	30.5774176	53.8867778	13.6957652
Nsg2	518.322532	0.21631441	0.22138594	0.97709192	0.32852365	0.99938111		Nsg2	430.781697	572.442504	522.285775	376.401482	489.454646	691.52006	490.754583	572.93951
Dctd	3.27168866	-0.4066089	0.41620291	-0.9769486	0.3285946	NA		Dctd	4.7043243	4.07271347	4.13502745	4.13628002	0	5.88027262	0.96226389	2.28262753
Trim65	3.1145856	0.40543793	0.41502522	0.9768995	0.32861891	NA		Trim65	2.68818531	1.35757116	1.27231614	4.13628002	1.66481172	1.17605452	5.77358333	6.84788259
Stac2	64.4265007	0.26588926	0.27240488	0.97608112	0.32902427	0.99938111		Stac2	47.043243	61.5432258	49.6203294	72.3849004	76.5813391	62.3308898	50.0377222	95.8703563

GeneID	Base mean	log2(FC)	StdErr	Wald-Stats	P-value	P-adj	GeneID	Normalised expression for Chow#1	Normalised expression for Chow#2	Normalised expression for Chow#3	Normalised expression for Chow#4	Normalised expression for HFD#1	Normalised expression for HFD#2	Normalised expression for HFD#3	Normalised expression for HFD#4
Glis2	6.33900151	-0.3994864	0.40932314	-0.9759682	0.32908022	0.99938111	Glis2	9.4086486	5.88280835	4.13502745	12.4088401	8.3240586	1.17605452	4.81131944	4.56525506
Dmkn	0.44018203	-0.2794601	0.28634188	-0.9759665	0.32908109	NA	Dmkn	1.34409266	0.90504744	1.27231614	0	0	0	0	0
Gpr27	15.9886934	0.40139287	0.41131416	0.97587905	0.3291244	0.99938111	Gpr27	8.73660227	14.9332827	4.77118552	20.6814001	39.9554813	4.7042181	6.73584722	27.3915304
Kif6	1.35686661	0.32269074	0.33070265	0.97577308	0.32917693	NA	Kif6	0	0.45252372	0.63615807	2.06814001	0	0	7.69811111	0
Stk32c	92.9042903	0.27507237	0.2819155	0.97572632	0.3292001	0.99938111	Stk32c	92.7423933	68.3310816	70.6135457	99.2707205	93.2294563	63.5069443	104.886764	150.653417
Rttn	1.87752838	-0.3949468	0.40499349	-0.9751929	0.32946458	NA	Rttn	2.68818531	3.16766603	2.54463228	2.06814001	1.66481172	0	2.88679167	0
Egr3	104.03632	-0.2883343	0.29571835	-0.9750301	0.32954532	0.99938111	Egr3	155.242702	137.567211	105.920318	62.0442003	109.877574	67.0351079	82.7546944	111.848749
Cers4	15.4430486	0.34150707	0.35035953	0.97473321	0.32969261	0.99938111	Cers4	7.39250961	15.3858064	14.6316356	14.4769801	21.6425524	21.1689814	10.5849028	18.2610202
Shisa6	43.3482327	0.35024962	0.35943578	0.97444283	0.32983671	0.99938111	Shisa6	30.2420848	53.3977988	49.3022503	10.3407001	66.5924688	65.8590533	41.3773472	29.6741579
Il3ra	1.20374498	-0.3306644	0.33938526	-0.9743039	0.32990565	NA	Il3ra	0.67204633	0.45252372	0.63615807	6.20442003	1.66481172	0	0	0
Hapln4	76.000081	0.29802642	0.30598783	0.9739813	0.33006582	0.99938111	Hapln4	50.4034746	74.2138899	68.0689134	72.3849004	104.883138	83.4998712	40.4150833	114.131377
Poc5	23.5185508	-0.3282974	0.33713977	-0.9737724	0.33016958	0.99938111	Poc5	30.9141311	23.0787097	23.2197695	33.0902402	11.653682	18.8168724	33.6792361	13.6957652
Stk32a	3.29035686	-0.4053079	0.41625242	-0.9737069	0.33020208	NA	Stk32a	5.37637063	3.16766603	5.08926455	4.13628002	0	4.7042181	3.84905555	0
Efcab6	2.80125727	0.40120429	0.41218226	0.97336621	0.33037133	NA	Efcab6	4.03227797	0.45252372	1.27231614	2.06814001	4.99443516	1.17605452	3.84905555	4.56525506
Lrrc39	1.18081321	0.35434748	0.36420671	0.97292958	0.3305883	NA	Lrrc39	0	0.90504744	0.31807903	2.06814001	1.66481172	3.52816357	0.96226389	0
Dzank1	253.887538	0.22895964	0.23534427	0.97287109	0.33061738	0.99938111	Dzank1	211.022547	186.892296	232.197695	301.948441	271.36431	351.640303	238.641444	237.393263
2510049J12Rik	1.20246479	0.32134621	0.33038891	0.97263014	0.33073716	NA	2510049J12Ri	0	0.45252372	1.27231614	0	3.32962344	0	0	4.56525506
Hist1h3g	2.47239905	-0.4005366	0.41187241	-0.9724774	0.3308131	NA	Hist1h3g	4.03227797	3.16766603	2.22655324	4.13628002	3.32962344	0	2.88679167	0
Abca8a	3.88616229	-0.3936455	0.40481447	-0.9724096	0.33084682	NA	Abca8a	4.7043243	9.95552182	6.04350166	0	3.32962344	7.05632714	0	0
Mgat4a	35.7216392	-0.3395345	0.34918616	-0.9723595	0.33087172	0.99938111	Mgat4a	36.2905017	53.3977988	49.9384084	24.8176801	28.3017992	55.2745626	24.0565972	13.6957652
Cstf2t	75.4129124	0.24468786	0.25172273	0.9720531	0.33102413	0.99938111	Cstf2t	66.5325865	58.3755597	67.7508343	80.6574604	64.9276571	85.8519803	67.3584722	111.848749
Mfsd7b	5.05821123	0.40470406	0.41637079	0.97197996	0.33106052	0.99938111	Mfsd7b	0.67204633	4.07271347	5.40734359	4.13628002	14.9833055	4.7042181	1.92452778	4.56525506
Fam65b	69.7163247	0.23466519	0.2414381	0.97194763	0.3310766	0.99938111	Fam65b	71.9089571	61.9957495	68.3869924	51.7035003	73.2517157	85.8519803	87.5660139	57.0656883
Eya1	11.6107093	0.34892231	0.35915945	0.97149695	0.33130087	0.99938111	Eya1	9.4086486	12.6706641	11.1327662	4.13628002	11.653682	10.5844907	17.32075	15.9783927
Khsrp	156.463154	0.39203194	0.40357471	0.97139868	0.33134979	0.99938111	Khsrp	83.3337447	79.6441745	78.8836006	250.244941	253.051381	51.7463991	64.4716805	390.329308
3110062M04Rik	0.8241286	-0.3328505	0.34268256	-0.9713085	0.33139466	NA	3110062M04R	1.34409266	1.35757116	2.22655324	0	1.66481172	0	0	0
Cdkn2c	0.69736614	-0.2926808	0.30134647	-0.9712434	0.33142709	NA	Cdkn2c	0.67204633	0.45252372	0.31807903	4.13628002	0	0	0	0
Fblim1	0.69736614	-0.2926808	0.30134647	-0.9712434	0.33142709	NA	Fblim1	0.67204633	0.45252372	0.31807903	4.13628002	0	0	0	0
Grrp1	0.69736614	-0.2926808	0.30134647	-0.9712434	0.33142709	NA	Grrp1	0.67204633	0.45252372	0.31807903	4.13628002	0	0	0	0
Cct3	30.5385542	0.29623949	0.30518929	0.97067461	0.33171034	0.99938111	Cct3	31.5861774	18.1009488	21.3112953	39.2946602	36.6258578	31.7534722	33.6792361	31.9567854
Lrrc40	18.1899334	0.35368236	0.36437274	0.97066085	0.33171719	0.99938111	Lrrc40	14.1129729	15.8383302	20.6751372	10.3407001	21.6425524	15.2887088	38.4905555	9.13051012
Sep-11	83.1710764	0.21763996	0.22434933	0.97009412	0.33199958	0.99938111	Sep-11	83.3337447	76.9290322	77.9293635	64.1123403	86.5702095	96.436471	75.0565833	105.000866
Als2cl	3.67766319	-0.4025138	0.41493114	-0.9700737	0.33200976	NA	Als2cl	2.01613899	2.71514231	3.49886938	12.4088401	3.32962344	3.52816357	1.92452778	0
Retsat	4.58323311	-0.3988621	0.41117358	-0.9700576	0.33201776	NA	Retsat	8.73660227	4.07271347	6.04350166	4.13628002	1.66481172	5.88027262	3.84905555	2.28262753
Nup210	15.2741471	-0.3413038	0.35205319	-0.9694666	0.33231242	0.99938111	Nup210	14.7850192	23.9837571	12.4050823	18.6132601	16.6481172	8.23238167	11.5471667	15.9783927
Rfc1	24.0407821	0.33029081	0.34069496	0.96946196	0.33231475	0.99938111	Rfc1	22.8495752	28.961518	18.766663	10.3407001	36.6258578	37.6337448	21.1698055	15.9783927
Rnasel	9.75725862	0.35183203	0.36293498	0.96940787	0.33234172	0.99938111	Rnasel	5.37637063	9.05047438	9.54237104	8.27256004	9.98887032	10.5844907	11.5471667	13.6957652
C2cd4c	22.3217225	0.39283957	0.40529857	0.9692597	0.33241563	0.99938111	C2cd4c	15.4570656	8.59795066	11.7689243	35.1583802	56.6035985	9.40843619	9.62263889	31.9567854
Frem3	6.95170552	0.3999225	0.41266843	0.96911337	0.33248863	0.99938111	Frem3	5.37637063	7.2403795	6.04350166	0	6.65924688	27.0492541	0.96226389	2.28262753
Tmem81	2.53628236	0.39784074	0.41056605	0.96900546	0.33254246	NA	Tmem81	1.34409266	3.16766603	0.63615807	2.06814001	6.65924688	3.52816357	2.88679167	0
Mipol1	3.29913826	0.40310406	0.41612302	0.96871367	0.33268807	NA	Mipol1	4.03227797	1.81009488	1.27231614	2.06814001	1.66481172	3.52816357	2.88679167	9.13051012
Brms1l	45.3692758	0.32290694	0.33336936	0.96861611	0.33273676	0.99938111	Brms1l	39.6507334	34.8443264	42.6225906	41.3628002	24.9721758	59.9787807	87.5660139	31.9567854
Ercc6	19.8517512	0.32651326	0.33716259	0.96841485	0.33283722	0.99938111	Ercc6	20.8334362	20.8160911	19.7209001	4.13628002	24.9721758	24.697145	23.0943333	20.5436478
Gm6642	1.47332913	-0.3719877	0.38412178	-0.9684109	0.33283922	NA	Gm6642	4.03227797	3.16766603	1.27231614	0	0	2.35210905	0.96226389	0
Serpina3n	4.39993127	-0.400125	0.41317887	-0.9684062	0.33284152	NA	Serpina3n	3.36023164	8.14542694	7.31581779	2.06814001	3.32962344	3.52816357	2.88679167	4.56525506
Stil	0.47086208	0.26938685	0.27817972	0.96839137	0.33284895	NA	Stil	0	0.45252372	0	0	0	2.35210905	0.96226389	0
OTTMUSG00000016609	49.6497385	0.40312051	0.41628938	0.96836608	0.33286157	0.99938111	TTMUSG0000001	37.6345944	44.3473245	53.1191988	10.3407001	11.653682	94.0843619	141.452792	4.56525506
Zak	15.9664916	0.36988043	0.38198589	0.96830914	0.33289	0.99938111	Zak	14.1129729	16.7433776	15.2677937	6.20442003	8.3240586	22.345036	35.6037639	9.13051012
Rps6kl1	17.9373432	0.35635104	0.36810365	0.96807256	0.33300813	0.99938111	Rps6kl1	20.1613899	7.69290322	11.1327662	22.7495401	29.966611	11.7605452	24.0565972	15.9783927
Hdx	1.53470992	-0.3813597	0.39402198	-0.9678641	0.33311227	NA	Hdx	3.36023164	2.26261859	1.27231614	2.06814001	0	2.35210905	0.96226389	0
Acot4	0.70503565	0.30867245	0.31893239	0.96783038	0.33312909	NA	Acot4	0	0.45252372	0.63615807	0	1.66481172	0	2.88679167	0
Gltscr1l	66.9745696	0.25225918	0.26073064	0.96750874	0.33328977	0.99938111	Gltscr1l	53.7637063	61.9957495	62.6615698	62.0442003	69.9220923	90.5561984	48.1131944	86.7398462
Cdc14b	8.20222113	-0.3692187	0.3816455	-0.9674389	0.33332467	0.99938111	Cdc14b	8.06455594	9.95552182	12.0870033	8.27256004	4.99443516	10.5844907	4.81131944	6.84788259
Cpne6	122.935619	0.29375718	0.30381717	0.96688803	0.33360001	0.99938111	Cpne6	139.11359	90.9572675	91.924841	109.611421	219.755147	81.1477622	104.886764	146.088162
Dclk3	24.4311181	-0.3283589	0.33964308	-0.9667765	0.33365578	0.99938111	Dclk3	45.6991503	18.1009488	27.0367179	20.6814001	24.9721758	24.697145	18.2830139	15.9783927
Emc8	43.9612927	0.27125785	0.2806325	0.96659458	0.33374675	0.99938111	Emc8	40.994826	34.3918026	30.8536663	51.7035003	46.6147282	37.6337448	43.301875	66.1961984
Amdhd2	7.78343108	0.39820295	0.41198956	0.9665365	0.33377579	0.99938111	Amdhd2	4.7043243	5.88280835	1.90847421	12.4088401	16.6481172	3.52816357	5.77358333	11.4131377
Epn2	70.4818561	-0.2686767	0.27808305	-0.9661743	0.33395697	0.99938111	Epn2	71.9089571	81.9067931	66.1604392	93.0663005	84.9053977	37.6337448	71.2075278	57.0656883
Zfp59	3.81414588	0.40221255	0.41629603	0.96616955	0.33395935	NA	Zfp59	2.01613899	4.07271347	3.18079035	2.06814001	0	8.23238167	8.660375	2.28262753
Lsg1	7.59336061	0.37427086	0.38748374	0.96590081	0.33409382	0.99938111	Lsg1	8.73660227	4.97776091	6.99773876	4.13628002	4.99443516	10.5844907	13.4716944	6.84788259
Cyp4f15	3.63483926	-0.4008594	0.41517934	-0.9655091	0.33428989	NA	Cyp4f15	4.03227797	6.33533207	5.08926455	2.06814001	4.99443516	2.35210905	1.92452778	2.28262753
1810055G02Rik	9.11674171	-0.3827079	0.3964109	-0.9654323	0.33432835	0.99938111	1810055G02R	13.4409266	16.7433776	10.8146872	2.06814001	11.653682	8.23238167	7.69811111	2.28262753
Pisd-ps3	21.9920681	-0.3259395	0.33763989	-0.9653466	0.33437124	0.99938111	Pisd-ps3	17.4732045	24.8888045	20.9932163	39.2946602	28.3017992	15.2887088	18.2830139	11.4131377
Cand1	149.048302	0.20265483	0.21004482	0.96481709	0.33463643	0.99938111	Cand1	134.409266	138.924782	155.222569	122.020261	141.508996	196.401106	157.811278	146.088162
G630025P09Rik	1.6993467	0.33651499	0.34880939	0.96475323	0.33466843	NA	G630025P09R	0	0	0.31807903	4.13628002	3.32962344	3.52816357	0	2.28262753
Mgrn1	212.748909	0.29306825	0.30379986	0.9646754	0.33470742	0.99938111	Mgrn1	159.27498	152.500493	160.947991	277.130761	306.325357	156.415252	160.698069	328.698364
Dnajb3	1.42031016	-0.3511523	0.3642039	-0.964164	0.3349637	NA	Dnajb3	1.34409266	1.35757116	0.31807903	6.20442003	0	1.17605452	0.96226389	0
Nox1	1.64714611	0.35013215	0.36318117	0.96407022	0.33501072	NA	Nox1	0	1.81009488	0.31807903	2.06814001	0	7.05632714	1.92452778	0
Osbpl3	15.2751439	0.36756126	0.38125997	0.96406992	0.33501087	0.99938111	Osbpl3	14.7850192	14.0282353	17.8124259	2.06814001	9.98887032	30.5774176	19.2452778	13.6957652
Dctn5	41.8504691	-0.2830901	0.29365215	-0.9640322	0.33502979	0.99938111	Dctn5	55.1077989	36.2018975	50.5745665	43.4309402	26.6369875	27.0492541	43.301875	52.5004332
Tert	1.27291789	0.29603277	0.30710133	0.96395797	0.335067	NA	Tert	0	0.90504744	0.9542371	0	8.3240586	0	0	0
Sumo2	41.716137	-0.3147262	0.32660522	-0.963629	0.33523198	0.99938111	Sumo2	42.3389187	51.1351802	47.0756971	49.6353602	18.3129289	23.5210905	60.622625	41.0872956
Ccndbp1	34.4823156	-0.2824766	0.29317308	-0.9635148	0.33528922	0.99938111	Ccndbp1	38.978687	33.9392789	38.8056422	43.4309402	16.6481172	29.4013631	40.4150833	34.239413
Btbd10	32.8294962	0.25872928	0.2685641	0.96337998	0.33535687	0.99938111	Btbd10	34.2743627	31.2241366	25.4463228	26.8858201	38.2906696	37.6337448	34.6415	34.239413
St8sia2	2.13206902	-0.3901979	0.40505481	-0.9633211	0.3353864	NA	St8sia2	4.03227797	4.07271347	3.49886938	0	0	3.52816357	1.92452778	0
Cdk7	37.0787607	0.26393236	0.27410904	0.96287361	0.33561095	0.99938111	Cdk7	30.2420848	31.6766603	41.3502745	28.9539601	33.2962344	39.9858538	50.0377222	41.0872956
Samd4b	77.2404912	-0.3141042	0.32625029	-0.9627707	0.3356626	0.99938111	Samd4b	74.5971425	103.175408	69.0231505	101.338861	93.2294563	37.6337448	38.4905555	100.435611
Stard5	4.18454739	0.40014615	0.41584585	0.96224635	0.33592587	NA	Stard5	2.68818531	1.35757116	4.13502745	4.13628002	8.3240586	1.17605452	4.81131944	6.84788259
Chac1	15.8283086	0.39678889	0.41236235	0.96223354	0.3359323	0.99938111	Chac1	5.37637063	9.5029981	7.31581779	26.8858201	23.3073641	3.52816357	9.62263889	41.0872956
Dgkq	30.0330871	0.35587312	0.36990152	0.96207532	0.33601177	0.99938111	Dgkq	17.4732045	13.5757116	19.7209001	51.7035003	49.9443516	22.345036	22.1320694	43.3699231
Mrps7	21.5749063	-0.319128	0.33171199	-0.9620636	0.33601768	0.99938111	Mrps7	23.5216215	20.8160911	19.7209001	35.1583802	19.9777406	9.40843619	21.1698055	22.8262753
Plekhg2	1.45043969	0.35134226	0.3652469	0.96193084	0.33608435	NA	Plekhg2	1.34409266	1.35757116	0.31807903	0	6.65924688	0	1.92452778	0
4930452B06Rik	10.3345838	0.38663121	0.40193911	0.96191489	0.33609236	0.99938111	4930452B06Ri	12.0968339	8.14542694	10.1785291	2.06814001	4.99443516	22.345036	18.2830139	4.56525506
Sdk2	10.5331004	0.38919142	0.40460903	0.96189503	0.33610233	0.99938111	Sdk2	6.72046328	12.2181404	10.1785291	2.06814001	28.3017992	4.7042181	8.660375	11.4131377
Klhl17	11.134327	0.39037665	0.40588074	0.96180137	0.33614939	0.99938111	Klhl17	8.06455594	8.14542694	2.86271131	16.5451201	14.9833055	4.7042181	8.660375	25.1089028
Atp11b	18.8593101	-0.3833493	0.3986032	-0.9617315	0.33618447	0.99938111	Atp11b	21.5054825	36.6544212	27.990955	4.13628002	6.65924688	29.4013631	15.3962222	9.13051012
Kcna6	82.4415692	0.28404197	0.29537103	0.96164463	0.33622815	0.99938111	Kcna6	61.8282622	78.2866034	63.2977279	88.9300204	109.877574	115.253343	46.1886666	95.8703563
Snora78	0.5052329	0.26947191	0.28029982	0.96137027	0.33636603	NA	Snora78	0	0.45252372	0	0	1.66481172	0	1.92452778	0
Smarcc2	246.042898	0.2000976	0.20813905	0.96136503	0.33636866	0.99938111	Smarcc2	223.791427	208.613434	193.392053	289.539601	251.38657	289.309413	233.830125	278.480559
Cnst	36.0886106	0.3071686	0.31951445	0.96136058	0.3363709	0.99938111	Cnst	36.2905017	36.2018975	37.5333261	14.4769801	34.9610461	54.0985081	50.0377222	25.1089028
Tpp2	38.0558964	0.33723445	0.35091605	0.96101175	0.33654626	0.99938111	Tpp2	28.2259458	38.9170398	48.3480132	12.4088401	33.2962344	62.3308898	55.8113055	25.1089028
Gm166	12.23083	-0.3388144	0.35264003	-0.9607941	0.33665572	0.99938111	Gm166	14.1129729	14.0282353	14.3135566	14.4769801	6.65924688	16.4647633	8.660375	9.13051012
Crhr1	5.9375432	-0.4000348	0.41637034	-0.9607667	0.33666947	0.99938111	Crhr1	8.06455594	12.2181404	6.99773876	2.06814001	9.98887032	5.88027262	0	2.28262753
Top3b	11.8283328	0.35399512	0.36862423	0.96031429	0.33689706	0.99938111	Top3b	10.0806949	7.69290322	10.4966081	10.3407001	9.98887032	16.4647633	6.73584722	22.8262753
Gtf2f2	7.81488031	0.38090599	0.39666433	0.96027284	0.33691791	0.99938111	Gtf2f2	8.73660227	5.88280835	7.31581779	2.06814001	3.32962344	7.05632714	14.4339583	13.6957652
Zfp865	37.736018	0.3695699	0.38490648	0.96015507	0.33697718	0.99938111	Zfp865	22.8495752	20.8160911	22.9016905	57.9079203	76.5813391	27.0492541	14.4339583	59.3483158
Cklf	1.74273246	-0.3451419	0.35948697	-0.9600956	0.33700708	NA	Cklf	2.01613899	2.26261859	0	6.20442003	0	1.17605452	0	2.28262753
Asah1	21.0506363	-0.3600282	0.37506113	-0.9599186	0.33709617	0.99938111	Asah1	22.1775288	38.0119924	24.8101647	14.4769801	11.653682	30.5774176	22.1320694	4.56525506
Irak2	7.26071085	-0.3895383	0.4059732	-0.9595172	0.33729824	0.99938111	Irak2	10.0806949	7.69290322	6.36158069	10.3407001	9.98887032	3.52816357	0.96226389	9.13051012
Tbc1d2b	17.4567876	0.34430237	0.35883657	0.95949631	0.33730878	0.99938111	Tbc1d2b	10.7527413	13.1231878	8.90621297	28.9539601	18.3129289	22.345036	14.4339583	22.8262753
Trmt61b	9.30561518	0.39050961	0.40701457	0.95944873	0.33733274	0.99938111	Trmt61b	3.36023164	4.52523719	4.45310648	18.6132601	11.653682	5.88027262	7.69811111	18.2610202
Arih1	85.8998872	0.27500474	0.28684292	0.95872941	0.33769508	0.99938111	Arih1	83.3337447	75.5714611	94.4694732	51.7035003	61.5980337	118.781507	124.132042	77.6093361
Dhfr	4.00466884	0.39911463	0.41631669	0.95868035	0.3377198	NA	Dhfr	4.03227797	3.16766603	1.90847421	2.06814001	4.99443516	0	6.73584722	9.13051012
Stk33	1.1187798	-0.3364668	0.35103211	-0.9585073	0.33780702	NA	Stk33	0.67204633	0.90504744	0.9542371	4.13628002	0	0	0	2.28262753
Apba1	291.56663	0.28033727	0.29255997	0.95822154	0.33795106	0.99938111	Apba1	217.070964	233.502239	206.751372	378.469622	414.538118	261.084104	201.113153	420.003466
Uqcrc1	116.027949	0.29396142	0.30680955	0.95812341	0.33800053	0.99938111	Uqcrc1	94.086486	81.4542694	80.7920748	153.042361	174.805231	84.6759257	90.4528055	168.914437
Gnpnat1	3.90117406	-0.3988567	0.41630072	-0.9580976	0.33801353	NA	Gnpnat1	8.06455594	4.97776091	5.40734359	2.06814001	0	5.88027262	4.81131944	0
2900008C10Rik	4.54003198	-0.3929828	0.41019696	-0.9580343	0.33804547	NA	2900008C10Ri	6.04841696	0.90504744	4.77118552	12.4088401	6.65924688	0	0.96226389	4.56525506
Ddx5	205.302059	0.19181353	0.20025344	0.95785387	0.33813645	0.99938111	Ddx5	223.119381	168.791347	186.394314	186.132601	186.458913	236.386959	240.565972	214.566988
Nova2	286.084301	0.32099653	0.33518236	0.95767726	0.33822553	0.99938111	Nova2	220.431196	211.7811	197.52708	355.720082	412.873307	188.168724	170.320708	531.852215
Cep57	10.4458807	0.36451757	0.38067227	0.95756271	0.33828331	0.99938111	Cep57	12.7688802	5.43028463	11.4508452	4.13628002	8.3240586	11.7605452	18.2830139	11.4131377
Aldh18a1	18.3033165	0.32912581	0.34396598	0.9568557	0.33864009	0.99938111	Aldh18a1	20.1613899	11.7656167	16.5401098	12.4088401	13.3184938	16.4647633	19.2452778	36.5220405

GeneID	Base mean	log2(FC)	StdErr	Wald-Stats	P-value	P-adj		GeneID	Normalised expression for Chow#1	Normalised expression for Chow#2	Normalised expression for Chow#3	Normalised expression for Chow#4	Normalised expression for HFD#1	Normalised expression for HFD#2	Normalised expression for HFD#3	Normalised expression for HFD#4
Commd3	36.4995109	-0.2933676	0.30662588	-0.9567607	0.33868805	0.99938111		Commd3	41.6668724	45.7048956	39.4418003	39.2946602	23.3073641	42.3379629	44.2641389	15.9783927
Vdac3	30.7910312	0.32531504	0.3400403	0.95669554	0.33872095	0.99938111		Vdac3	20.8334362	16.2908539	32.4440615	37.2265202	33.2962344	17.6408179	45.2264028	43.3699231
Pcdha4-g	34.2862169	-0.3355822	0.35091929	-0.9562947	0.33892339	0.99938111		Pcdha4-g	47.043243	56.5654649	37.8514051	14.4769801	21.6425524	45.8661264	21.1698055	29.6741579
Plin3	1.71514949	-0.3770362	0.39427685	-0.9562728	0.33893445	NA		Plin3	2.01613899	4.07271347	1.27231614	2.06814001	3.32962344	0	0.96226389	0
Gstt1	5.83857865	-0.3950706	0.41315446	-0.9562298	0.33895612	0.99938111		Gstt1	2.68818531	6.78785578	4.45310648	16.5451201	3.32962344	3.52816357	4.81131944	4.56525506
Tmem102	0.97724093	0.33753033	0.35304096	0.95606566	0.33903906	NA		Tmem102	0	0.45252372	0.31807903	2.06814001	1.66481172	2.35210905	0.96226389	0
H13	34.4728024	-0.3588588	0.37539234	-0.9559566	0.33909414	0.99938111		H13	28.8979921	68.7836053	44.5310648	18.6132601	16.6481172	55.2745626	20.2075417	22.8262753
Trap1	33.2264378	0.28623979	0.29949874	0.95572953	0.3392089	0.99938111		Trap1	40.994826	30.3190892	28.6271131	14.4769801	39.9554813	37.6337448	32.7169722	41.0872956
Map2k7	72.227364	0.34027453	0.35614603	0.9554354	0.33935756	0.99938111		Map2k7	51.075521	37.106945	49.9384084	107.543281	98.2238915	41.1619083	55.8113055	136.957652
Usp25	24.9953626	0.31926496	0.33420845	0.95528693	0.33943262	0.99938111		Usp25	27.5538995	23.5312334	24.1740066	10.3407001	19.9777406	37.6337448	38.4905555	18.2610202
A730017L22Rik	7.65194801	0.37937704	0.39714478	0.95526129	0.33944558	0.99938111		A730017L22Ri	2.01613899	6.78785578	6.04350166	10.3407001	13.3184938	5.88027262	7.69811111	9.13051012
Ndufa7	20.1684491	-0.3593982	0.37624592	-0.9552216	0.33946564	0.99938111		Ndufa7	16.1291119	17.1959013	23.2197695	39.2946602	8.3240586	7.05632714	25.0188611	25.1089028
A330033J07Rik	1.23926366	0.35368174	0.3703234	0.95506182	0.33954644	NA		A330033J07Ri	2.01613899	0	0.63615807	0	1.66481172	2.35210905	0.96226389	2.28262753
Rbak	4.01576797	0.39501606	0.41373277	0.95476134	0.3396984	NA		Rbak	2.68818531	2.26261859	4.77118552	2.06814001	8.3240586	5.88027262	3.84905555	2.28262753
Lrrc73	27.1218183	0.37153719	0.38927553	0.95443243	0.3398648	0.99938111		Lrrc73	29.5700384	17.1959013	15.2677937	24.8176801	36.6258578	10.5844907	14.4339583	68.4788259
Fam228b	1.21522727	0.3477799	0.36450513	0.95411524	0.34002532	NA		Fam228b	0.67204633	0	0.63615807	2.06814001	0	1.17605452	2.88679167	2.28262753
Srp54c	34.6630754	0.29000556	0.30396102	0.954088	0.3400391	0.99938111		Srp54c	30.9141311	23.9837571	26.7186389	43.4309402	26.6369875	54.0985081	32.7169722	38.804668
Cstad	1.20764576	0.33944295	0.35594861	0.9536291	0.34027142	NA		Cstad	1.34409266	0.90504744	0.31807903	0	0	0	4.81131944	2.28262753
Rbm15	14.7403815	0.33230209	0.34851707	0.95347435	0.34034978	0.99938111		Rbm15	14.1129729	12.6706641	9.86045007	14.4769801	16.6481172	25.8731995	10.5849028	13.6957652
Fgfr1op	9.43737392	0.38468318	0.40349555	0.9533765	0.34039934	0.99938111		Fgfr1op	6.72046328	9.95552182	12.0870033	0	9.98887032	12.9365998	19.2452778	4.56525506
Dgkk	20.1546504	-0.392827	0.4120755	-0.953289	0.34044368	0.99938111		Dgkk	8.73660227	12.2181404	10.4966081	70.3167603	9.98887032	23.5210905	7.69811111	18.2610202
Gpr158	111.321395	-0.3562844	0.37381081	-0.9531143	0.34053215	0.99938111		Gpr158	112.903783	201.373055	144.407882	62.0442003	71.586904	195.225051	59.6603611	43.3699231
Nol6	153.107812	0.35588733	0.37341651	0.95305729	0.34056105	0.99938111		Nol6	99.4628566	78.7391271	94.1513942	237.836101	291.342051	84.6759257	87.5660139	251.089028
Cped1	2.64962156	0.36992557	0.38829658	0.9526882	0.34074807	NA		Cped1	0	3.16766603	2.86271131	0	8.3240586	5.88027262	0.96226389	0
Ciart	7.60425344	-0.3656007	0.38383958	-0.952483	0.34085211	0.99938111		Ciart	6.72046328	7.69290322	11.1327662	10.3407001	6.65924688	4.7042181	6.73584722	6.84788259
Aars2	13.5346267	0.3673506	0.3856823	0.95246945	0.34085895	0.99938111		Aars2	10.7527413	4.97776091	7.95197586	22.7495401	23.3073641	12.9365998	9.62263889	15.9783927
Ociad1	229.978702	0.20783994	0.21823251	0.95237843	0.34090509	0.99938111		Ociad1	180.108416	213.591195	203.570582	254.381221	196.447783	302.246013	233.830125	255.654283
Wrnip1	40.8967296	0.27952892	0.29359368	0.95209448	0.34104907	0.99938111		Wrnip1	30.2420848	28.5089943	35.6248519	51.7035003	51.6091633	36.4576902	33.6792361	59.3483158
Fam188a	28.7203644	-0.3055896	0.32102334	-0.9519234	0.34113584	0.99938111		Fam188a	32.2582238	35.7493738	34.9886938	26.8858201	11.653682	22.345036	38.4905555	27.3915304
Hsbp1	178.026716	-0.3188227	0.33498022	-0.9517657	0.34121581	0.99938111		Hsbp1	245.29691	204.993245	226.790352	134.429101	81.5757743	155.239197	280.018792	95.8703563
Eif6	19.5406922	0.36915483	0.38789492	0.95168771	0.34125539	0.99938111		Eif6	12.7688802	9.95552182	7.95197586	35.1583802	21.6425524	11.7605452	18.2830139	38.804668
Cacna1g	47.5455292	0.34496993	0.36251078	0.95161288	0.34129335	0.99938111		Cacna1g	21.5054825	52.9452751	37.215247	47.5672202	84.9053977	51.7463991	18.2830139	66.1961984
Zfp580	33.1204782	-0.3545635	0.37263349	-0.9515072	0.34134695	0.99938111		Zfp580	41.6668724	38.9170398	23.2197695	49.6353602	28.3017992	8.23238167	20.2075417	54.7830607
Lif	0.93244273	-0.3061813	0.32186405	-0.9512751	0.34146475	NA		Lif	0	0.45252372	1.90847421	4.13628002	0	0	0.96226389	0
Nrg1	6.03050728	-0.3834306	0.40309122	-0.9512255	0.34148993	0.99938111		Nrg1	9.4086486	9.5029981	6.99773876	2.06814001	8.3240586	3.52816357	3.84905555	4.56525506
Cemip	8.864259	0.37857009	0.39800073	0.95117937	0.34151333	0.99938111		Cemip	5.37637063	8.59795066	9.224292	4.13628002	8.3240586	19.9929269	3.84905555	11.4131377
Dennd4c	9.33584896	-0.3465942	0.36441069	-0.9511087	0.3415492	0.99938111		Dennd4c	11.4247876	10.4080455	11.7689243	10.3407001	8.3240586	8.23238167	9.62263889	4.56525506
Cbr1	21.788273	0.32002729	0.3366823	0.95053197	0.34184202	0.99938111		Cbr1	18.8172972	14.480759	27.990955	12.4088401	31.6314227	17.6408179	30.7924444	20.5436478
Ddhd1	40.4376263	-0.2678747	0.28195852	-0.9500502	0.34208676	0.99938111		Ddhd1	52.4196136	44.3473245	52.1649617	28.9539601	28.3017992	38.8097993	44.2641389	34.239413
G6pdx	21.3707521	-0.3146112	0.33124259	-0.9497909	0.34221852	0.99938111		G6pdx	24.1936678	17.648425	20.6751372	35.1583802	23.3073641	11.7605452	15.3962222	22.8262753
Stox2	185.771834	0.19708925	0.2075145	0.94976135	0.34223353	0.99938111		Stox2	161.291119	178.294345	176.533864	171.655621	174.805231	239.915123	164.547125	219.132243
Oas1c	0.44466806	-0.268808	0.28304182	-0.9497111	0.34225908	NA		Oas1c	2.01613899	0.90504744	0.63615807	0	0	0	0	0
Ppp3r2	0.44466806	-0.268808	0.28304182	-0.9497111	0.34225908	NA		Ppp3r2	2.01613899	0.90504744	0.63615807	0	0	0	0	0
Tmem179	93.9733942	0.34408075	0.36233509	0.94962028	0.34230523	0.99938111		Tmem179	46.3711967	73.7613662	62.6615698	134.429101	161.486737	69.3872169	46.1886666	157.5013
Fstl1	10.2401446	-0.3628312	0.38209261	-0.9495896	0.34232084	0.99938111		Fstl1	9.4086486	15.8383302	16.5401098	6.20442003	11.653682	9.40843619	10.5849028	2.28262753
Pcdhb16	11.16063	0.39001676	0.41074115	0.94954392	0.34234404	0.99938111		Pcdhb16	5.37637063	7.2403795	10.4966081	12.4088401	19.9777406	27.0492541	6.73584722	0
Ttll11	27.9778159	0.27928699	0.29431301	0.94894546	0.34264835	0.99938111		Ttll11	25.5377605	24.8888045	24.1740066	22.7495401	24.9721758	24.697145	28.8679167	47.9351782
Ssbp3	114.702041	-0.2844288	0.29973994	-0.9489186	0.34266203	0.99938111		Ssbp3	127.688802	118.561214	82.700549	184.064461	121.531256	59.9787807	95.264125	127.827142
E2f6	22.7022584	-0.3194283	0.33667302	-0.948779	0.34273304	0.99938111		E2f6	24.1936678	21.7211385	32.1259825	26.8858201	8.3240586	23.5210905	28.8679167	15.9783927
Poglut1	28.1604303	-0.2943727	0.31031851	-0.9486148	0.34281654	0.99938111		Poglut1	25.5377605	25.793852	31.4898244	45.4990802	16.6481172	31.7534722	21.1698055	27.3915304
Tusc3	69.4499218	-0.3452458	0.36398286	-0.9485221	0.34286374	0.99938111		Tusc3	61.8282622	137.114687	95.1056313	26.8858201	48.2795399	95.2604165	50.0377222	41.0872956
Erc1	72.6937786	0.21363126	0.22527971	0.94829339	0.34298011	0.99938111		Erc1	72.5810035	67.8785578	68.7050715	57.9079203	83.240586	89.3801438	77.943375	63.9135709
Rpl14	20.0309014	-0.3899236	0.41128197	-0.9480687	0.34309447	0.99938111		Rpl14	49.059382	17.1959013	19.0847421	14.4769801	8.3240586	9.40843619	40.4150833	2.28262753
Ddx3x	134.907648	0.30744026	0.32431608	0.94796489	0.34314733	0.99938111		Ddx3x	152.554517	116.298596	116.416927	84.7937404	88.2350212	248.147505	183.792403	89.0224737
Tmem206	24.0969922	-0.2934644	0.30960211	-0.9478758	0.34319267	0.99938111		Tmem206	20.1613899	27.6039469	22.9016905	39.2946602	21.6425524	22.345036	18.2830139	20.5436478
Scg2	165.559328	0.38739918	0.40872444	0.94782485	0.34321863	0.99938111		Scg2	100.806949	202.730626	165.719177	45.4990802	69.9220923	553.921681	117.396194	68.4788259
Dis3l2	20.2827289	0.29537012	0.31163992	0.94779296	0.34323486	0.99938111		Dis3l2	17.4732045	19.0059962	18.448584	16.5451201	28.3017992	17.6408179	28.8679167	15.9783927
Hdlbp	144.176842	0.26016342	0.27450975	0.94773835	0.34326267	0.99938111		Hdlbp	163.307258	132.136926	128.50393	93.0663005	114.872009	231.682741	159.735805	130.109769
Slc25a44	99.0241909	0.24446494	0.25795102	0.94771844	0.34327281	0.99938111		Slc25a44	75.2691888	95.9350284	82.700549	103.407001	119.866444	92.9083074	76.0188472	146.088162
Rasgrp3	4.1942148	-0.3945377	0.41636567	-0.9475749	0.34334589	NA		Rasgrp3	6.72046328	7.69290322	6.99773876	0	1.66481172	4.7042181	5.77358333	0
Sssca1	11.9597883	-0.3512861	0.3708015	-0.9473698	0.34345039	0.99938111		Sssca1	11.4247876	18.1009488	9.54237104	16.5451201	6.65924688	5.88027262	11.5471667	15.9783927
Narg2	2.57932525	0.39352416	0.41541806	0.94729673	0.3434876	NA		Narg2	2.01613899	2.71514231	2.22655324	0	1.66481172	5.88027262	3.84905555	2.28262753
Ttc3	841.720125	0.35152839	0.37110411	0.94725005	0.34351138	0.99938111		Ttc3	759.412351	906.405009	828.913964	312.289142	529.410127	1981.65187	972.848791	442.829741
Zcchc7	3.13105369	0.39382182	0.41578258	0.94718212	0.34354599	NA		Zcchc7	3.36023164	3.62018975	1.90847421	0	3.32962344	7.05632714	5.77358333	0
2810032G03Rik	3.35092249	-0.393905	0.41589002	-0.9471375	0.34356874	NA		2810032G03R	7.39250961	4.07271347	5.40734359	0	1.66481172	1.17605452	4.81131944	2.28262753
Zfp652	18.280583	0.37010341	0.39076873	0.94711625	0.34357955	0.99938111		Zfp652	18.1452509	22.1736622	15.5858727	2.06814001	36.6258578	18.8168724	25.981125	6.84788259
Zfp239	34.0883204	-0.3175554	0.33531528	-0.9470354	0.34362076	0.99938111		Zfp239	44.3550577	42.0847059	41.0321955	28.9539601	14.9833055	45.8661264	39.4528194	15.9783927
Ube2j1	108.70257	0.26145364	0.27617269	0.94670344	0.34378992	0.99938111		Ube2j1	106.855366	91.8623149	118.64348	72.3849004	81.5757743	151.711034	152.999958	93.5877288
Actr3b	46.5126771	0.29568109	0.31238184	0.94653739	0.34387456	0.99938111		Actr3b	56.4518916	33.9392789	45.1672229	24.8176801	66.5924688	30.5774176	52.9245139	61.6309433
Taf6	38.5499237	-0.2482288	0.26226691	-0.9464741	0.34390685	0.99938111		Taf6	39.6507334	37.106945	40.3960374	53.7716403	36.6258578	32.9295267	33.6792361	34.239413
Rabgap1	74.9953888	0.26702116	0.28226909	0.94598086	0.34415835	0.99938111		Rabgap1	70.5648645	73.7613662	72.5220199	51.7035003	58.2684102	114.077289	101.999972	57.0656883
Actr10	88.5373238	0.26893006	0.2844273	0.94551424	0.3443964	0.99938111		Actr10	83.3337447	74.6664136	99.2406588	57.9079203	61.5980337	92.9083074	140.490528	98.1529838
Hist1h3h	3.84474688	-0.393526	0.41631388	-0.9452628	0.34452471	NA		Hist1h3h	6.04841696	4.52523719	3.81694841	4.13628002	0	1.17605452	1.92452778	9.13051012
Tppp	407.577864	0.18771694	0.19859458	0.9452269	0.34454305	0.99938111		Tppp	428.765558	376.499734	395.054161	316.425422	369.588202	462.189428	508.075333	404.025073
Eva1b	0.88868343	-0.2413743	0.25538901	-0.9451242	0.34459547	NA		Eva1b	0	0.90504744	0	6.20442003	0	0	0	0
Fgf22	0.88868343	-0.2413743	0.25538901	-0.9451242	0.34459547	NA		Fgf22	0	0.90504744	0	6.20442003	0	0	0	0
Nop14	16.0061479	0.33459438	0.354035	0.94508844	0.34461373	0.99938111		Nop14	20.1613899	13.1231878	11.4508452	10.3407001	9.98887032	22.345036	26.9433889	13.6957652
Gpr124	6.89021749	-0.3932374	0.41616038	-0.9449178	0.34470083	0.99938111		Gpr124	5.37637063	14.480759	4.77118552	10.3407001	14.9833055	0	2.88679167	2.28262753
Tbc1d31	1.95247614	0.37292691	0.39476165	0.94468881	0.34481777	NA		Tbc1d31	1.34409266	1.35757116	2.22655324	0	0	5.88027262	4.81131944	0
Vrk3	10.0114597	-0.3425496	0.36263902	-0.9446021	0.34486206	0.99938111		Vrk3	10.0806949	13.5757116	12.7231614	10.3407001	9.98887032	8.23238167	10.5849028	4.56525506
Smpd1	81.8658028	-0.2653568	0.28099718	-0.9443399	0.34499599	0.99938111		Smpd1	69.2207718	127.159165	88.4259716	74.4530404	73.2517157	84.6759257	50.9999861	86.7398462
Rsph1	2.82750758	0.3857479	0.40854012	0.94421059	0.34506205	NA		Rsph1	3.36023164	0.45252372	0.63615807	4.13628002	1.66481172	5.88027262	1.92452778	4.56525506
Glra1	3.74165511	-0.3929338	0.41616398	-0.9441803	0.34507752	NA		Glra1	2.68818531	4.52523719	5.72542262	6.20442003	1.66481172	5.88027262	0.96226389	2.28262753
Lrba	32.1133015	0.29193646	0.30920041	0.94416583	0.34508491	0.99938111		Lrba	32.9302701	30.7716129	30.2175083	18.6132601	31.6314227	55.2745626	34.6415	22.8262753
Proser2	1.44747284	0.33334871	0.35306258	0.94416323	0.34508624	NA		Proser2	0	0	1.27231614	2.06814001	4.99443516	0	0.96226389	2.28262753
Slc30a1	21.5811035	0.34285491	0.36315597	0.94409823	0.34511945	0.99938111		Slc30a1	13.4409266	23.9837571	24.1740066	10.3407001	19.9777406	45.8661264	21.1698055	13.6957652
Enho	31.5515043	-0.3857758	0.40864719	-0.9440314	0.34515361	0.99938111		Enho	29.5700384	28.0564706	19.7209001	76.5211804	34.9610461	4.7042181	8.660375	50.2178057
Fam216b	0.52089182	0.23407824	0.24800784	0.94383403	0.34525447	NA		Fam216b	0	0	0.31807903	0	0	0	3.84905555	0
Zfp711	7.39974235	0.39232146	0.4156797	0.94380714	0.34526821	0.99938111		Zfp711	10.0806949	4.97776091	5.40734359	2.06814001	1.66481172	10.5844907	22.1320694	2.28262753
Nxt1	4.15856153	0.39188768	0.4152734	0.94368596	0.34533015	NA		Nxt1	2.01613899	1.81009488	3.49886938	6.20442003	1.66481172	3.52816357	7.69811111	6.84788259
Zfp113	7.50778629	0.38259833	0.40551057	0.94349781	0.34542634	0.99938111		Zfp113	3.36023164	7.69290322	3.81694841	10.3407001	1.66481172	10.5844907	13.4716944	9.13051012
Kcnk10	4.35487522	0.39069974	0.41414501	0.94338874	0.3454821	NA		Kcnk10	2.68818531	6.78785578	0.31807903	2.06814001	8.3240586	5.88027262	1.92452778	6.84788259
Fip1l1	49.723493	0.22985518	0.2437325	0.94306334	0.34564851	0.99938111		Fip1l1	50.4034746	49.7776091	43.2587487	37.2265202	56.6035985	52.9224536	59.6603611	47.9351782
Gbp2	0.42954729	-0.2647935	0.28086387	-0.9427823	0.34579226	NA		Gbp2	0.67204633	1.81009488	0.9542371	0	0	0	0	0
Tmem140	0.42954729	-0.2647935	0.28086387	-0.9427823	0.34579226	NA		Tmem140	0.67204633	1.81009488	0.9542371	0	0	0	0	0
Plscr3	9.45530848	-0.3923084	0.41614201	-0.9427271	0.34582049	0.99938111		Plscr3	10.0806949	6.33533207	9.86045007	20.6814001	3.32962344	0	4.81131944	20.5436478
AI854517	23.1118034	0.32852935	0.34851287	0.9426606	0.34585453	0.99938111		AI854517	26.8818531	11.313093	15.2677937	26.8858201	29.966611	14.1126543	30.7924444	29.6741579
Rnf166	20.3686059	-0.3219873	0.34173788	-0.9422053	0.34608753	0.99938111		Rnf166	20.8334362	16.7433776	19.0847421	39.2946602	14.9833055	12.9365998	23.0943333	15.9783927
Ccnd1	41.7373568	0.3076834	0.32661249	0.94204419	0.34617002	0.99938111		Ccnd1	33.6023164	29.8665654	32.1259825	49.6353602	66.5924688	23.5210905	34.6415	63.9135709
Srgap1	32.7019541	-0.2987816	0.31719608	-0.9419459	0.34622031	0.99938111		Srgap1	28.8979921	35.2968501	28.6271131	57.9079203	18.3129289	37.6337448	29.8301805	25.1089028
Mar-03	2.77097257	0.39203574	0.416251	0.94182533	0.34628207	NA		Mar-03	2.01613899	2.71514231	1.27231614	2.06814001	3.32962344	2.35210905	3.84905555	4.56525506
Eif1b	85.5055416	-0.3114374	0.33068134	-0.9418052	0.34629236	0.99938111		Eif1b	94.086486	76.4765085	68.7050715	148.906081	56.6035985	39.9858538	76.0188472	123.261887
Cap2	141.481728	-0.2365125	0.25117634	-0.9416195	0.34638751	0.99938111		Cap2	189.517065	166.076205	161.26607	99.2707205	113.207197	139.950488	152.999958	109.566121
Parp2	6.6298338	-0.392007	0.41632253	-0.9415944	0.34640031	0.99938111		Parp2	16.1291119	7.69290322	8.2700549	2.06814001	1.66481172	4.7042181	12.5094306	0
Birc2	20.8309132	0.31335286	0.33283183	0.94147503	0.34646148	0.99938111		Birc2	20.8334362	14.9332827	25.4463228	8.27256004	24.9721758	18.8168724	25.981125	27.3915304
Cul7	32.9765743	0.37668656	0.40010327	0.94147332	0.34646236	0.99938111		Cul7	18.1452509	15.3858064	17.1762679	55.8397803	61.5980337	21.1689814	10.5849028	63.9135709
Slc25a4	392.330518	0.28348185	0.30111636	0.94143622	0.34648136	0.99938111		Slc25a4	381.050268	247.530474	256.371702	504.626163	329.632721	319.886831	433.01875	666.527239
Ahcyl1	263.641684	-0.254778	0.27065601	-0.9413351	0.34653315	0.99938111		Ahcyl1	305.781079	310.883795	335.255302	208.882141	174.805231	282.253086	322.358403	168.914437
Mccc2	10.9468895	-0.3534795	0.37557228	-0.9411757	0.34661484	0.99938111		Mccc2	12.0968339	9.5029981	10.4966081	20.6814001	4.99443516	5.88027262	12.5094306	11.4131377
Aff1	13.151998	-0.3537266	0.37583937	-0.9411644	0.34662062	0.99938111		Aff1	13.4409266	25.3413283	14.3135566	8.27256004	4.99443516	15.2887088	14.4339583	9.13051012
Ddx21	12.1770607	0.37479108	0.39822146	0.94116242	0.34662163	0.99938111		Ddx21	16.8011582	9.5029981	11.7689243	0	11.653682	23.5210905	17.32075	6.84788259
Fam189b	28.8727187	-0.3519556	0.37396616	-0.9411429	0.34663165	0.99938111		Fam189b	21.5054825	40.7271347	27.672876	43.4309402	34.9610461	12.9365998	8.660375	41.0872956

GeneID	Base mean	log2(FC)	StdErr	Wald-Stats	P-value	P-adj	GeneID	Normalised expression for Chow#1	Normalised expression for Chow#2	Normalised expression for Chow#3	Normalised expression for Chow#4	Normalised expression for HFD#1	Normalised expression for HFD#2	Normalised expression for HFD#3	Normalised expression for HFD#4
Ppip5k1	101.334866	0.2521285	0.26794666	0.9409653	0.34672264	0.99938111	Ppip5k1	81.9896521	71.4987476	104.011844	107.543281	153.162678	85.8519803	85.6414861	120.979259
Zfp169	14.5792806	0.31873406	0.33873612	0.94095089	0.34673003	0.99938111	Zfp169	10.0806949	10.8605693	11.7689243	18.6132601	16.6481172	11.7605452	16.3584861	20.5436478
Hexim1	39.8042683	0.25690204	0.27304527	0.94087707	0.34676786	0.99938111	Hexim1	40.3227797	28.5089943	30.5355873	45.4990802	39.9554813	45.8661264	37.5282917	50.2178057
Mtmr3	66.5567199	0.24574982	0.2612	0.94084923	0.34678213	0.99938111	Mtmr3	57.7959842	50.2301328	56.9361472	78.5893204	86.5702095	85.8519803	54.8490416	61.6309433
Map2k1	177.289568	0.24062089	0.25578626	0.94071077	0.3468531	0.99938111	Map2k1	182.796601	123.991499	175.261548	159.246781	234.738453	128.189943	178.981083	235.110636
D17Wsu92e	183.091864	0.32451621	0.34497552	0.94069347	0.34686197	0.99938111	D17Wsu92e	137.097451	114.035977	114.826531	262.653781	246.392135	109.373071	135.679208	344.676757
Trim9	226.130099	-0.2245044	0.2387015	-0.9405238	0.34694897	0.99938111	Trim9	285.61969	268.346565	242.694303	179.928181	244.727323	186.992669	158.773542	241.958518
Prrg2	2.37144418	-0.3827205	0.4069661	-0.9404235	0.34700036	NA	Prrg2	3.36023164	2.26261859	1.27231614	6.20442003	1.66481172	0	1.92452778	2.28262753
Kcnj8	0.71491074	0.31861458	0.33884138	0.94030598	0.34706063	NA	Kcnj8	0.67204633	0.45252372	0.31807903	0	0	2.35210905	1.92452778	0
G3bp1	26.2863452	0.26771868	0.28472791	0.94026147	0.34708346	0.99938111	G3bp1	21.5054825	21.2686148	23.5378486	28.9539601	34.9610461	27.0492541	27.9056528	25.1089028
Avl9	65.8211442	0.24870827	0.26451868	0.94022951	0.34709985	0.99938111	Avl9	68.5487255	60.6381783	62.9796488	45.4990802	51.6091633	82.3238167	93.3395972	61.6309433
Thbs3	2.1605674	-0.3391443	0.36077537	-0.9400429	0.34719556	NA	Thbs3	2.68818531	0.45252372	0	10.3407001	1.66481172	1.17605452	0.96226389	0
Adamts15	10.1991067	0.383065	0.40750225	0.94003161	0.34720135	0.99938111	Adamts15	4.03227797	3.62018975	5.72542262	20.6814001	14.9833055	11.7605452	4.81131944	15.9783927
3110009E18Rik	0.54749353	-0.2690677	0.28626084	-0.9399389	0.34724889	NA	3110009E18Ri	0	1.35757116	0.9542371	2.06814001	0	0	0	0
Rbbp5	27.4742792	0.29848704	0.31764675	0.93968234	0.34738053	0.99938111	Rbbp5	17.4732045	24.4362808	23.2197695	31.0221002	43.2851047	18.8168724	25.0188611	36.5220405
Pias1	49.5522138	-0.2432584	0.25895002	-0.939403	0.34752389	0.99938111	Pias1	57.1239379	47.9675142	66.1604392	45.4990802	43.2851047	51.7463991	48.1131944	36.5220405
Ipo4	54.184398	0.31025269	0.33027233	0.93938444	0.3475334	0.99938111	Ipo4	47.7152893	42.0847059	39.4418003	57.9079203	88.2350212	30.5774176	38.4905555	89.0224737
Gemin7	10.8872373	0.3697071	0.39356663	0.93937613	0.34753767	0.99938111	Gemin7	7.39250961	4.97776091	7.95197586	16.5451201	16.6481172	3.52816357	16.3584861	13.6957652
Vps33a	89.7662939	0.21947321	0.23363877	0.93936981	0.34754091	0.99938111	Vps33a	81.3176057	73.7613662	84.2909441	88.9300204	121.531256	81.1477622	79.8679027	107.283494
Ndufa3	54.3734792	-0.2896788	0.30839238	-0.939319	0.34756697	0.99938111	Ndufa3	65.8605402	50.2301328	54.0734359	76.5211804	24.9721758	45.8661264	74.0943194	43.3699231
Nr2c2	129.193891	0.2308468	0.24582512	0.93906919	0.34769522	0.99938111	Nr2c2	113.575829	114.035977	139.318617	105.475141	131.520126	159.943415	176.094292	93.5877288
Ablim3	42.8010541	-0.2883766	0.30716753	-0.938825	0.34782059	0.99938111	Ablim3	47.7152893	36.2018975	33.0802196	78.5893204	34.9610461	35.2816357	42.3396111	34.239413
Leprel2	3.31107477	0.3463656	0.36901839	0.93861338	0.34792928	NA	Leprel2	0	0	0.63615807	8.27256004	9.98887032	4.7042181	2.88679167	0
Lipt1	2.37530084	-0.3880006	0.41341243	-0.9385316	0.34797128	NA	Lipt1	3.36023164	3.62018975	3.18079035	2.06814001	0	3.52816357	0.96226389	2.28262753
Tram1l1	22.8025152	0.32846579	0.35002673	0.938402	0.34803786	0.99938111	Tram1l1	14.1129729	27.6039469	19.4028211	16.5451201	23.3073641	45.8661264	17.32075	18.2610202
Pet117	0.89227575	0.32639059	0.3478324	0.93835591	0.34806154	NA	Pet117	0	0.90504744	0.63615807	0	0	2.35210905	0.96226389	2.28262753
Socs6	9.92016728	0.33853579	0.36078001	0.93834409	0.34806761	0.99938111	Socs6	6.04841696	8.14542694	9.224292	10.3407001	9.98887032	9.40843619	12.5094306	13.6957652
4933428G20Rik	0.85827811	0.27036409	0.28813524	0.93832358	0.34807814	NA	4933428G20R	0	0	0.63615807	0	1.66481172	0	0	4.56525506
Mcam	1.35481727	-0.3402685	0.36280813	-0.9378744	0.34830897	NA	Mcam	1.34409266	3.62018975	2.54463228	0	3.32962344	0	0	0
Morf4l2	163.616438	0.26444479	0.28202703	0.93765759	0.3484204	0.99938111	Morf4l2	143.145868	141.1874	169.854204	132.360961	98.2238915	202.281378	259.81125	162.066555
Pafah2	4.65651373	0.39014176	0.41611198	0.9375884	0.34845597	0.99938111	Pafah2	5.37637063	4.07271347	1.59039517	2.06814001	9.98887032	1.17605452	3.84905555	9.13051012
Trmt10c	10.6810268	0.35994894	0.38399153	0.93738771	0.34855916	0.99938111	Trmt10c	5.37637063	13.5757116	10.4966081	4.13628002	6.65924688	10.5844907	16.3584861	18.2610202
Gemin2	1.32948344	0.36218717	0.3864336	0.9372559	0.34862694	NA	Gemin2	1.34409266	0.90504744	0.63615807	0	3.32962344	1.17605452	0.96226389	2.28262753
Polb	16.4974201	-0.3833744	0.4090657	-0.9371951	0.34865819	0.99938111	Polb	29.5700384	26.6988994	20.6751372	4.13628002	4.99443516	17.6408179	25.981125	2.28262753
Trpv6	0.55364224	-0.2626313	0.28024981	-0.9371328	0.34869025	NA	Trpv6	0	0.45252372	1.90847421	2.06814001	0	0	0	0
Srrm2	348.868854	-0.17427	0.18599778	-0.9369465	0.34878606	0.99938111	Srrm2	370.297527	399.578444	375.333261	337.106822	382.906696	342.231867	286.754639	296.741579
Wdr20	12.1059546	0.32610192	0.34807656	0.93686838	0.34882627	0.99938111	Wdr20	14.1129729	8.59795066	9.86045007	8.27256004	16.6481172	14.1126543	11.5471667	13.6957652
Stip1	90.6540052	0.24190449	0.25822821	0.93678566	0.34886882	0.99938111	Stip1	96.7746713	71.4987476	69.0231505	90.9981605	76.5813391	96.436471	84.6792222	139.240279
Fcf1	0.69288011	-0.2669298	0.28494328	-0.9367822	0.34887062	NA	Fcf1	0	0.45252372	0.9542371	4.13628002	0	0	0	0
Sfpq	71.7437457	0.26608566	0.28404765	0.93676417	0.34887988	0.99938111	Sfpq	71.9089571	61.0907021	71.5677828	49.6353602	43.2851047	87.0280348	79.8679027	109.566121
Fbxo34	24.0907323	0.30048745	0.32077491	0.93675484	0.34888468	0.99938111	Fbxo34	28.2259458	21.2686148	22.5836114	10.3407001	36.6258578	22.345036	30.7924444	20.5436478
St3gal1	15.2115461	0.31466172	0.33592402	0.93670504	0.3489103	0.99938111	St3gal1	10.0806949	15.3858064	13.6773985	14.4769801	18.3129289	22.345036	18.2830139	9.13051012
Tmem43	21.9328956	0.35746788	0.3817907	0.93629279	0.34912246	0.99938111	Tmem43	16.8011582	26.6988994	13.0412404	14.4769801	34.9610461	31.7534722	5.77358333	31.9567854
Bbs1	31.9624303	0.31885395	0.34056866	0.93623986	0.3491497	0.99938111	Bbs1	38.978687	27.6039469	23.8559276	16.5451201	48.2795399	23.5210905	22.1320694	54.7830607
Prkca	142.558878	0.32963058	0.35210253	0.9361778	0.34918165	0.99938111	Prkca	145.834053	158.383302	139.000538	41.3628002	133.184938	245.795396	192.452778	84.4572186
Phlpp2	77.2541443	0.25483084	0.27228111	0.93591083	0.3493191	0.99938111	Phlpp2	60.4841696	56.5654649	67.1146763	95.1344405	101.553515	79.9717076	56.7735694	100.435611
Hist1h4j	6.00360271	-0.3882541	0.41493849	-0.9356908	0.34943243	0.99938111	Hist1h4j	5.37637063	5.43028463	4.77118552	14.4769801	4.99443516	0	3.84905555	9.13051012
Hdac11	208.598274	0.34767451	0.37163541	0.93552579	0.3495174	0.99938111	Hdac11	139.11359	101.817837	106.556477	351.583802	291.342051	122.309671	152.037694	404.025073
A930024E05Rik	1.09728102	0.34659664	0.37062906	0.93515774	0.34970701	NA	A930024E05Ri	1.34409266	0.45252372	0.63615807	0	0	1.17605452	2.88679167	2.28262753
Cdc23	25.0973444	0.31725338	0.33932858	0.93494446	0.34981692	0.99938111	Cdc23	20.1613899	26.6988994	23.5378486	16.5451201	13.3184938	37.6337448	42.3396111	20.5436478
Ecel1	32.980421	0.3511355	0.37561653	0.93482439	0.34987881	0.99938111	Ecel1	18.1452509	30.7716129	23.2197695	37.2265202	49.9443516	38.8097993	8.660375	57.0656883
Dusp8	200.689217	0.3204692	0.34295062	0.93444705	0.35007334	0.99938111	Dusp8	151.88247	146.165161	135.501669	256.449361	339.621591	125.837834	114.509403	335.546247
Otud3	3.17309295	0.38904397	0.41635787	0.93439803	0.35009861	NA	Otud3	2.68818531	3.16766603	1.27231614	2.06814001	4.99443516	4.7042181	1.92452778	4.56525506
Actn3	0.63281126	0.2880929	0.30832495	0.93438077	0.35010751	NA	Actn3	0	0.45252372	0.31807903	0	3.32962344	0	0.96226389	0
Fstl3	0.71417172	-0.2620657	0.28048126	-0.9343429	0.35012702	NA	Fstl3	0.67204633	0.90504744	0	4.13628002	0	0	0	0
Gm14436	7.96806247	0.38479701	0.41190962	0.93417824	0.35021196	0.99938111	Gm14436	5.37637063	6.33533207	8.58813393	2.06814001	0	21.1689814	20.2075417	0
Rpusd4	3.2805474	0.38886413	0.41633764	0.93401145	0.35029798	NA	Rpusd4	1.34409266	2.26261859	4.13502745	2.06814001	4.99443516	4.7042181	6.73584722	0
Tfip11	35.328456	0.3044796	0.3260066	0.9339676	0.3503206	0.99938111	Tfip11	38.978687	19.0059962	25.4463228	41.3628002	58.2684102	25.8731995	39.4528194	34.239413
Gm12359	0.97908267	0.30023073	0.32146575	0.93394315	0.35033322	NA	Gm12359	1.34409266	0	0.31807903	0	4.99443516	1.17605452	0	0
Prkdc	11.9023028	0.34752214	0.3721549	0.93381046	0.35040168	0.99938111	Prkdc	7.39250961	8.59795066	13.9954775	10.3407001	16.6481172	21.1689814	12.5094306	4.56525506
Wif1	1.87781467	0.35107993	0.37611147	0.9334465	0.35058948	NA	Wif1	0	0	1.27231614	4.13628002	1.66481172	4.7042181	0.96226389	2.28262753
Mas1	10.4248806	-0.3815374	0.40875882	-0.9334046	0.3506111	0.99938111	Mas1	11.4247876	22.1736622	12.7231614	2.06814001	11.653682	3.52816357	3.84905555	15.9783927
Ficd	5.77191071	-0.3886329	0.41637067	-0.9333819	0.35062281	0.99938111	Ficd	5.37637063	12.6706641	7.63389683	4.13628002	0	10.5844907	5.77358333	0
Ctnna2	76.3449424	0.24341644	0.26082048	0.93327196	0.35067957	0.99938111	Ctnna2	81.9896521	81.4542694	67.4327553	43.4309402	69.9220923	102.316744	86.60375	77.6093361
Lbr	4.84271358	-0.3868383	0.41450379	-0.9332563	0.35068766	0.99938111	Lbr	1.34409266	8.59795066	6.36158069	8.27256004	3.32962344	4.7042181	3.84905555	2.28262753
Zdhhc7	20.2747459	-0.3217988	0.34483315	-0.9332014	0.35071597	0.99938111	Zdhhc7	23.5216215	22.1736622	13.3593194	35.1583802	21.6425524	18.8168724	11.5471667	15.9783927
Zbtb7b	6.89581268	-0.3852663	0.41288558	-0.9331067	0.3507649	0.99938111	Zbtb7b	3.36023164	7.69290322	5.08926455	18.6132601	8.3240586	2.35210905	2.88679167	6.84788259
Cldn5	3.57637667	-0.3756876	0.40265302	-0.9330307	0.3508041	NA	Cldn5	1.34409266	12.2181404	2.86271131	2.06814001	6.65924688	1.17605452	0	2.28262753
Urb1	7.97215351	0.3714921	0.39824899	0.93281368	0.35091618	0.99938111	Urb1	9.4086486	5.43028463	7.63389683	2.06814001	18.3129289	10.5844907	5.77358333	4.56525506
Pign	8.7664772	-0.3762508	0.40336176	-0.9327875	0.3509297	0.99938111	Pign	7.39250961	13.1231878	15.9039517	6.20442003	6.65924688	14.1126543	6.73584722	0
Zfp12	20.5340048	0.30525203	0.32732064	0.93257801	0.35103789	0.99938111	Zfp12	25.5377605	13.5757116	18.448584	14.4769801	24.9721758	24.697145	28.8679167	13.6957652
Galk1	4.89138145	0.3873076	0.41557141	0.93198809	0.35134268	0.99938111	Galk1	4.7043243	2.26261859	3.18079035	4.13628002	11.653682	1.17605452	2.88679167	9.13051012
Dtx3l	6.89652403	0.37174348	0.39906033	0.93154707	0.35157065	0.99938111	Dtx3l	8.06455594	6.33533207	4.13502745	4.13628002	6.65924688	5.88027262	15.3962222	4.56525506
Tbk1	16.7868544	0.32613368	0.35018088	0.9313292	0.3516833	0.99938111	Tbk1	16.8011582	10.8605693	20.6751372	8.27256004	19.9777406	27.0492541	19.2452778	11.4131377
Slfn5	2.34386138	0.38290717	0.41131265	0.93093945	0.35188488	NA	Slfn5	1.34409266	1.81009488	1.27231614	2.06814001	6.65924688	2.35210905	0.96226389	2.28262753
Eps8	38.6209588	0.25953949	0.27881622	0.93086225	0.35192483	0.99938111	Eps8	38.978687	31.2241366	40.7141164	26.8858201	29.966611	45.8661264	51.96225	43.3699231
Utp15	19.2010747	0.30474104	0.32740043	0.93078999	0.35196221	0.99938111	Utp15	13.4409266	13.1231878	16.5401098	26.8858201	23.3073641	23.5210905	23.0943333	13.6957652
Unc45a	9.78956807	0.35056612	0.37664491	0.93076028	0.35197758	0.99938111	Unc45a	8.06455594	8.59795066	6.99773876	10.3407001	14.9833055	16.4647633	10.5849028	2.28262753
Klhl35	0.93728131	0.32104245	0.34493144	0.93074278	0.35198664	NA	Klhl35	0.67204633	0	0.9542371	0	1.66481172	0	1.92452778	2.28262753
Lyst	52.1793725	0.32930058	0.35381506	0.93071384	0.35200161	0.99938111	Lyst	49.7314283	51.1351802	58.5265423	16.5451201	83.240586	84.6759257	46.1886666	27.3915304
Rfxap	18.0729797	0.30725832	0.33019273	0.93054233	0.35209036	0.99938111	Rfxap	13.4409266	11.7656167	18.766663	20.6814001	14.9833055	25.8731995	23.0943333	15.9783927
Mrps18a	25.4966864	-0.3116523	0.33500984	-0.9302781	0.3522271	0.99938111	Mrps18a	34.2743627	23.9837571	23.5378486	33.0902402	23.3073641	9.40843619	22.1320694	34.239413
Med24	56.4750013	0.2688064	0.289028	0.93003583	0.35235253	0.99938111	Med24	55.1077989	50.2301328	56.9361472	35.1583802	66.5924688	63.5069443	37.5282917	86.7398462
Pak6	77.7492707	0.28480346	0.30627993	0.92987961	0.35243342	0.99938111	Pak6	71.9089571	52.4927514	48.3480132	103.407001	96.5590798	70.5632714	57.7358333	120.979259
Atxn7l3	206.120382	0.3421622	0.36796476	0.92987763	0.35243445	0.99938111	Atxn7l3	129.032895	111.773359	130.412404	322.629842	337.956779	112.901234	129.905625	374.350915
Fbxl15	12.7397914	0.38589824	0.41501265	0.92984695	0.35245034	0.99938111	Fbxl15	6.04841696	4.07271347	6.67965972	22.7495401	26.6369875	3.52816357	4.81131944	27.3915304
Nox4	0.71179293	0.26831352	0.28857482	0.92978843	0.35248064	NA	Nox4	0.67204633	0	0.31807903	0	0	4.7042181	0	0
Dab1	71.0734962	-0.2943185	0.31655261	-0.9297617	0.35249446	0.99938111	Dab1	81.3176057	112.678406	85.2451812	39.2946602	44.9499164	70.5632714	86.60375	47.9351782
Tstd2	14.550651	-0.3184895	0.34258161	-0.9296748	0.3525395	0.99938111	Tstd2	18.1452509	16.2908539	17.8124259	12.4088401	8.3240586	9.40843619	13.4716944	20.5436478
BC005764	41.4951272	0.35508284	0.38208367	0.92933267	0.35271671	0.99938111	BC005764	16.1291119	38.9170398	37.8514051	43.4309402	81.5757743	37.6337448	12.5094306	63.9135709
Alas2	0.77151936	0.31837598	0.3426458	0.92916937	0.35280132	NA	Alas2	0	0.45252372	0.9542371	0	1.66481172	1.17605452	1.92452778	0
St8sia6	0.72944491	-0.3059662	0.32945287	-0.9287101	0.35303934	NA	St8sia6	0.67204633	2.71514231	1.27231614	0	0	1.17605452	0	0
1500011B03Rik	92.058109	-0.2499149	0.26911946	-0.9286394	0.35307602	0.99938111	1500011B03Ri	90.7262543	87.7896015	82.3824699	144.769801	78.2461509	64.6829988	76.0188472	111.848749
Nol4	91.3737435	0.2432402	0.26193209	0.92863838	0.35307652	0.99938111	Nol4	90.7262543	88.6946489	83.9728651	68.2486203	109.877574	94.0843619	133.754681	61.6309433
Slc31a2	2.51474785	0.38568932	0.41547438	0.92831072	0.35324642	NA	Slc31a2	2.01613899	2.71514231	1.90847421	0	4.99443516	2.35210905	3.84905555	2.28262753
Ift122	18.2681299	0.30243925	0.3258034	0.92828759	0.35325841	0.99938111	Ift122	16.1291119	19.0059962	16.2220308	10.3407001	23.3073641	12.9365998	23.0943333	25.1089028
Vps18	55.7933997	0.37607168	0.40518538	0.92814721	0.35333121	0.99938111	Vps18	24.1936678	25.3413283	33.0802196	95.1344405	124.860879	21.1689814	22.1320694	100.435611
Map3k9	104.962426	0.26060936	0.28079515	0.92811204	0.35334946	0.99938111	Map3k9	68.5487255	80.549222	89.3802087	140.633521	138.179373	123.485725	77.943375	120.979259
Ints4	18.9378925	-0.3221253	0.34708071	-0.9280992	0.35335612	0.99938111	Ints4	16.1291119	25.793852	20.3570582	26.8858201	11.653682	18.8168724	25.0188611	6.84788259
Nisch	411.554043	0.32415359	0.34944833	0.92761523	0.35360719	0.99938111	Nisch	269.490578	283.279848	279.591471	577.011063	749.165274	295.189686	215.547111	623.157316
Wdr26	124.767092	0.28358594	0.30583484	0.92725192	0.35379575	0.99938111	Wdr26	116.264015	120.823833	125.641219	78.5893204	86.5702095	188.168724	195.339569	86.7398462
Mbd2	22.204787	-0.3244443	0.35013262	-0.9266327	0.35411728	0.99938111	Mbd2	37.6345944	24.4362808	28.6271131	10.3407001	11.653682	25.8731995	23.0943333	15.9783927
Inpp1	15.6260541	0.34896466	0.37673269	0.92629249	0.354294	0.99938111	Inpp1	14.7850192	4.52523719	13.3593194	20.6814001	21.6425524	21.1689814	10.5849028	18.2610202
Entpd4	107.207031	-0.2951161	0.31875968	-0.9258262	0.35453633	0.99938111	Entpd4	97.4467176	167.433776	116.098848	99.2707205	94.8942681	138.774434	43.301875	100.435611
Sh3glb2	75.1491812	-0.2550322	0.27548507	-0.925757	0.35457225	0.99938111	Sh3glb2	88.7101153	67.8785578	79.2016796	95.1344405	103.218327	50.5703445	54.8490416	61.6309433
Atl3	21.6686699	0.30789865	0.33259837	0.92573711	0.35458262	0.99938111	Atl3	18.1452509	22.1736622	21.6293743	12.4088401	38.2906696	22.345036	26.9433889	11.4131377
Prpf6	27.8437235	0.26416928	0.2854357	0.92549488	0.35470854	0.99938111	Prpf6	25.5377605	22.6261859	23.8559276	28.9539601	38.2906696	24.697145	33.6792361	25.1089028
Ankrd28	28.0501685	-0.3348116	0.36180624	-0.9253892	0.3547635	0.99938111	Ankrd28	43.6830113	36.6544212	33.3982986	16.5451201	13.3184938	27.0492541	42.3396111	11.4131377
C030046E11Rik	26.7062058	0.25588896	0.27654107	0.92531993	0.35479951	0.99938111	C030046E11Ri	24.8657142	24.4362808	23.8559276	22.7495401	28.3017992	27.0492541	32.7169722	29.6741579
Aff2	26.7446868	0.33225735	0.35908982	0.92527642	0.35482214	0.99938111	Aff2	20.1613899	28.0564706	19.4028211	24.8176801	14.9833055	58.8027262	31.7547083	15.9783927
Zfp41	4.99876155	-0.382614	0.41362909	-0.9250172	0.35495698	0.99938111	Zfp41	5.37637063	4.52523719	3.49886938	12.4088401	6.65924688	2.35210905	2.88679167	2.28262753
Rbmx2	2.09559146	0.372009	0.40218204	0.92497666	0.35497805	NA	Rbmx2	1.34409266	0.90504744	1.90847421	2.06814001	1.66481172	1.17605452	7.69811111	0

GeneID	Base mean	log2(FC)	StdErr	Wald-Stats	P-value	P-adj		GeneID	Normalised expression for Chow#1	Normalised expression for Chow#2	Normalised expression for Chow#3	Normalised expression for Chow#4	Normalised expression for HFD#1	Normalised expression for HFD#2	Normalised expression for HFD#3	Normalised expression for HFD#4
Romo1	17.7798264	-0.3843924	0.41566972	-0.9247543	0.35509374	0.99938111		Romo1	7.39250961	8.14542694	10.1785291	64.1123403	11.653682	4.7042181	8.660375	27.3915304
Tjap1	4.77655129	-0.3848679	0.41619474	-0.9247304	0.35510619	0.99938111		Tjap1	6.72046328	3.16766603	5.40734359	8.27256004	1.66481172	0	3.84905555	9.13051012
Zfp281	35.3953628	0.31205654	0.33746349	0.92471202	0.35511572	0.99938111		Zfp281	28.2259458	39.3695635	39.1237212	14.4769801	23.3073641	47.042181	59.6603611	31.9567854
Ttll4	5.35011868	0.37020485	0.40037703	0.92464059	0.35515289	0.99938111		Ttll4	2.68818531	4.97776091	5.08926455	4.13628002	6.65924688	4.7042181	7.69811111	6.84788259
Med12l	54.3312657	0.27300821	0.29530838	0.92448514	0.35523379	0.99938111		Med12l	46.3711967	52.0402277	46.7576181	49.6353602	68.2572805	90.5561984	49.0754583	31.9567854
Itsn2	23.8854963	-0.3252521	0.3518206	-0.9244828	0.35523501	0.99938111		Itsn2	21.5054825	32.129184	35.9429309	20.6814001	13.3184938	35.2816357	23.0943333	9.13051012
Sesn1	23.5912282	-0.284578	0.30783523	-0.9244492	0.35525249	0.99938111		Sesn1	32.9302701	22.1736622	29.8994292	20.6814001	18.3129289	24.697145	24.0565972	15.9783927
Sult1a1	1.83840753	-0.3660314	0.3959491	-0.9244405	0.35525702	NA		Sult1a1	2.01613899	6.33533207	1.59039517	0	1.66481172	1.17605452	1.92452778	0
Arl5b	11.7725196	0.34626879	0.37459814	0.92437403	0.35529161	0.99938111		Arl5b	12.0968339	9.95552182	11.1327662	6.20442003	4.99443516	15.2887088	23.0943333	11.4131377
Polr1a	21.5232767	0.31261189	0.33825638	0.92418623	0.35538936	0.99938111		Polr1a	20.8334362	10.8605693	19.4028211	24.8176801	34.9610461	27.0492541	18.2830139	15.9783927
Etv6	15.0232692	0.32453575	0.35129888	0.92381664	0.35558179	0.99938111		Etv6	16.1291119	11.7656167	17.1762679	4.13628002	21.6425524	14.1126543	19.2452778	15.9783927
Tradd	1.46907205	-0.3523485	0.38142487	-0.923769	0.3556066	NA		Tradd	1.34409266	0.90504744	1.90847421	4.13628002	0	1.17605452	0	2.28262753
Shpk	1.32448085	0.35883926	0.38858219	0.92345781	0.35576868	NA		Shpk	1.34409266	0.90504744	0.9542371	0	3.32962344	1.17605452	2.88679167	0
Arf4	88.2188159	-0.2242638	0.24286641	-0.9234038	0.35579679	0.99938111		Arf4	98.7908103	101.365313	110.373425	70.3167603	76.5813391	67.0351079	99.1131805	82.1745911
Gm16677	0.93723807	0.30287752	0.32812657	0.92305089	0.35598068	NA		Gm16677	0	0.90504744	0.63615807	0	4.99443516	0	0.96226389	0
Ccnjl	3.6346304	-0.3842238	0.41634355	-0.9228528	0.3560839	NA		Ccnjl	4.03227797	4.52523719	5.08926455	4.13628002	6.65924688	2.35210905	0	2.28262753
Xkr8	4.43322617	-0.383585	0.41568052	-0.922788	0.35611771	NA		Xkr8	3.36023164	11.313093	3.49886938	4.13628002	4.99443516	5.88027262	0	2.28262753
Mmp19	1.19133495	-0.3447917	0.37367084	-0.9227152	0.35615567	NA		Mmp19	1.34409266	2.71514231	2.22655324	0	0	0	0.96226389	2.28262753
Pygb	103.91507	-0.2831949	0.30703982	-0.9223392	0.35635165	0.99938111		Pygb	103.495135	92.7673624	79.8378377	190.268881	121.531256	64.6829988	76.0188472	102.718239
Kcnq4	2.25450056	-0.3672106	0.3981537	-0.9222835	0.35638073	NA		Kcnq4	4.03227797	1.35757116	1.27231614	6.20442003	0	0	2.88679167	2.28262753
Rala	48.6803309	0.32933655	0.35715534	0.92211012	0.35647114	0.99938111		Rala	47.7152893	50.2301328	52.1649617	14.4769801	31.6314227	75.2674895	83.7169583	34.239413
Fance	7.57080918	0.35941678	0.38982462	0.92199611	0.35653061	0.99938111		Fance	6.04841696	4.97776091	8.2700549	4.13628002	13.3184938	4.7042181	7.69811111	11.4131377
Erdr1	8.15079608	0.38380666	0.41637068	0.92179079	0.35663771	0.99938111		Erdr1	2.01613899	3.16766603	3.49886938	16.5451201	9.98887032	2.35210905	4.81131944	22.8262753
Cdk15	0.643941	0.29171411	0.31646597	0.92178668	0.35663986	NA		Cdk15	0	0.45252372	0.63615807	0	0	1.17605452	2.88679167	0
Gpatch3	4.08686691	0.38374797	0.41634239	0.92171246	0.35667858	NA		Gpatch3	2.01613899	1.81009488	1.59039517	8.27256004	4.99443516	2.35210905	4.81131944	6.84788259
Ufc1	21.5468836	-0.3125926	0.33932502	-0.921219	0.35693612	0.99938111		Ufc1	34.2743627	22.1736622	20.3570582	22.7495401	11.653682	17.6408179	29.8301805	13.6957652
Fam110a	7.90743755	-0.3833753	0.41624035	-0.9210432	0.3570279	0.99938111		Fam110a	5.37637063	8.14542694	2.22655324	24.8176801	8.3240586	2.35210905	2.88679167	9.13051012
Cul2	32.223123	0.30511213	0.33130618	0.92093701	0.35708332	0.99938111		Cul2	29.5700384	33.9392789	30.2175083	18.6132601	24.9721758	54.0985081	48.1131944	18.2610202
Dazap1	40.8345642	0.35844507	0.3892441	0.92087476	0.35711582	0.99938111		Dazap1	24.1936678	20.8160911	21.6293743	68.2486203	49.9443516	19.9929269	25.981125	95.8703563
Spon1	49.63361	0.352747	0.38311443	0.92073537	0.35718861	0.99938111		Spon1	35.6184554	63.8058444	37.215247	26.8858201	49.9443516	128.189943	21.1698055	34.239413
Sdccag8	10.4027955	0.35723207	0.38811934	0.92041812	0.35735431	0.99938111		Sdccag8	12.0968339	8.59795066	10.8146872	2.06814001	6.65924688	18.8168724	17.32075	6.84788259
Cep70	4.16116936	0.3811618	0.41412078	0.92041215	0.35735742	NA		Cep70	4.03227797	4.52523719	3.18079035	0	1.66481172	14.1126543	5.77358333	0
Zfp385b	42.290196	0.24756292	0.26905875	0.92010733	0.35751668	0.99938111		Zfp385b	38.978687	43.8948007	36.2610099	31.0221002	38.2906696	38.8097993	47.1509305	63.9135709
Aebp2	38.9470763	0.26606864	0.28923129	0.91991651	0.35761639	0.99938111		Aebp2	42.3389187	38.9170398	32.7621406	24.8176801	33.2962344	59.9787807	45.2264028	34.239413
Ndufa12	36.4385752	-0.2964817	0.32233026	-0.9198072	0.3576735	0.99938111		Ndufa12	43.010965	42.9897533	51.8468826	24.8176801	14.9833055	31.7534722	43.301875	38.804668
Lypd1	98.5438715	-0.2794235	0.30383841	-0.9196452	0.35775822	0.99938111		Lypd1	108.871505	159.288349	114.190373	53.7716403	118.201632	82.3238167	74.0943194	77.6093361
Tmem135	42.9287649	-0.241316	0.26241676	-0.9195906	0.35778674	0.99938111		Tmem135	38.3066407	54.75537	47.7118552	45.4990802	36.6258578	34.1055812	38.4905555	47.9351782
Pgam5	26.1950941	0.27975426	0.30429136	0.91936315	0.35790566	0.99938111		Pgam5	18.8172972	17.648425	25.4463228	33.0902402	29.966611	23.5210905	33.6792361	27.3915304
Ndufaf1	13.532352	0.31399933	0.34155444	0.91932442	0.35792591	0.99938111		Ndufaf1	9.4086486	11.7656167	13.6773985	12.4088401	9.98887032	19.9929269	17.32075	13.6957652
Zfp82	2.1375999	-0.3555711	0.38678096	-0.9193087	0.35793415	NA		Zfp82	2.01613899	0.90504744	1.27231614	8.27256004	0	2.35210905	0	2.28262753
Kcnh2	29.1735922	0.30328934	0.32992383	0.9192708	0.35795395	0.99938111		Kcnh2	17.4732045	28.961518	20.0389792	35.1583802	34.9610461	30.5774176	18.2830139	47.9351782
0610039K10Rik	3.22674793	0.38075997	0.41424829	0.91915881	0.35801251	NA		0610039K10Ri	1.34409266	1.81009488	0.9542371	6.20442003	6.65924688	2.35210905	1.92452778	4.56525506
Coil	11.8524339	0.32276298	0.35117535	0.9190935	0.35804667	0.99938111		Coil	10.7527413	9.05047438	11.4508452	8.27256004	16.6481172	8.23238167	14.4339583	15.9783927
Myo7a	15.7645335	0.33098492	0.36017786	0.91894855	0.35812248	0.99938111		Myo7a	15.4570656	9.95552182	8.90621297	20.6814001	28.3017992	15.2887088	11.5471667	15.9783927
Cd63	5.14598524	-0.3803109	0.41390446	-0.9188373	0.35818067	0.99938111		Cd63	4.03227797	6.78785578	1.27231614	14.4769801	3.32962344	1.17605452	0.96226389	9.13051012
Gapvd1	45.1953046	0.2518738	0.27420069	0.91857464	0.3583181	0.99938111		Gapvd1	45.027104	40.274611	49.9384084	24.8176801	51.6091633	59.9787807	44.2641389	45.6525506
Gm3264	0.6099002	0.26476276	0.28826465	0.91847112	0.35837227	NA		Gm3264	0.67204633	0	0	0	0	0	1.92452778	2.28262753
Rgmb	79.3904844	0.30711921	0.33438582	0.91845762	0.35837934	0.99938111		Rgmb	43.010965	73.7613662	66.7965972	90.9981605	83.240586	78.7956531	43.301875	155.218672
Map3k15	15.6640888	0.31947983	0.34801835	0.91799708	0.35862039	0.99938111		Map3k15	17.4732045	13.5757116	13.0412404	10.3407001	13.3184938	28.2253086	20.2075417	9.13051012
D130040H23Rik	3.46673285	0.37806872	0.41186927	0.91793379	0.35865353	NA		D130040H23R	2.68818531	2.26261859	3.18079035	2.06814001	0	11.7605452	5.77358333	0
Mogs	12.0011895	-0.3448824	0.37572148	-0.9179203	0.35866059	0.99938111		Mogs	11.4247876	15.8383302	14.6316356	12.4088401	6.65924688	12.9365998	3.84905555	18.2610202
Dmbx1	0.66406427	0.23224843	0.25312157	0.91753711	0.35886126	NA		Dmbx1	0	0	0.31807903	0	4.99443516	0	0	0
Pfn4	5.5667532	-0.3759419	0.40979664	-0.9173865	0.35894013	0.99938111		Pfn4	7.39250961	8.59795066	4.13502745	8.27256004	1.66481172	3.52816357	8.660375	2.28262753
Abca6	0.71712536	-0.3060957	0.33367978	-0.9173338	0.35896775	NA		Abca6	1.34409266	2.26261859	0.9542371	0	0	1.17605452	0	0
Gls2	10.8860079	-0.3580034	0.39030257	-0.9172459	0.35901379	0.99938111		Gls2	12.0968339	8.59795066	7.63389683	24.8176801	4.99443516	11.7605452	5.77358333	11.4131377
Cd34	5.10691754	-0.3818288	0.41635075	-0.9170843	0.35909845	0.99938111		Cd34	4.03227797	13.5757116	6.99773876	0	6.65924688	1.17605452	3.84905555	4.56525506
Fam210a	54.3234091	0.2700273	0.29458175	0.9166464	0.35932796	0.99938111		Fam210a	49.7314283	38.4645161	55.345752	51.7035003	31.6314227	71.739326	78.9056389	57.0656883
H2-T23	2.78517691	0.37634673	0.41062082	0.91653105	0.35938843	NA		H2-T23	1.34409266	2.71514231	1.27231614	2.06814001	3.32962344	4.7042181	0	6.84788259
Tmem64	45.0239691	0.27684316	0.30208815	0.91643172	0.3594405	0.99938111		Tmem64	24.8657142	55.2078937	44.2129858	33.0902402	59.9332219	50.5703445	37.5282917	54.7830607
1700001J11Rik	1.724243	-0.357977	0.39069177	-0.9162645	0.35952816	NA		1700001J11Ri	5.37637063	1.81009488	2.54463228	0	0	1.17605452	2.88679167	0
Cox8a	337.511902	-0.3574443	0.39023104	-0.9159813	0.35967669	0.99938111		Cox8a	277.555134	265.631423	262.097124	790.029484	273.029122	85.8519803	168.39618	577.504765
Epha3	3.28965761	-0.378657	0.41345805	-0.9158292	0.35975648	NA		Epha3	2.68818531	6.33533207	7.63389683	0	0	3.52816357	3.84905555	2.28262753
Acads	2.01499002	-0.3636991	0.39717305	-0.9157194	0.35981407	NA		Acads	2.01613899	3.16766603	1.27231614	4.13628002	0	0	0.96226389	4.56525506
Neto1	111.023316	0.29682681	0.32422	0.91551049	0.3599237	0.99938111		Neto1	94.7585323	111.773359	119.597717	62.0442003	78.2461509	189.344778	161.660333	70.7614535
Dym	51.3797282	-0.2393632	0.26149112	-0.9153779	0.35999326	0.99938111		Dym	69.2207718	51.1351802	57.8903843	45.4990802	44.9499164	37.6337448	56.7735694	47.9351782
Mafa	1.08615128	0.32859904	0.35900401	0.91530741	0.36003027	NA		Mafa	1.34409266	0.45252372	0.31807903	0	3.32962344	0	0.96226389	2.28262753
Tgfb1	2.19362281	-0.3549552	0.38780245	-0.915299	0.36003468	NA		Tgfb1	0	4.97776091	3.18079035	4.13628002	3.32962344	0	1.92452778	0
Sdf2l1	2.9543076	-0.3730639	0.40759094	-0.9152899	0.36003944	NA		Sdf2l1	1.34409266	5.88280835	3.49886938	4.13628002	0	0	1.92452778	6.84788259
Lmcd1	0.80566034	-0.3119978	0.34090741	-0.915198	0.36008768	NA		Lmcd1	2.01613899	1.81009488	0.9542371	0	1.66481172	0	0	0
Kirrel	7.794205	0.37097942	0.40536522	0.91517327	0.36010068	0.99938111		Kirrel	4.03227797	7.69290322	4.45310648	8.27256004	4.99443516	11.7605452	2.88679167	18.2610202
Sh2d3c	28.9102959	0.36081503	0.3942904	0.9150997	0.36013929	0.99938111		Sh2d3c	18.1452509	15.3858064	19.7209001	41.3628002	64.9276571	16.4647633	9.62263889	45.6525506
Bag6	169.04173	0.32863609	0.35919278	0.91492954	0.36022862	0.99938111		Bag6	113.575829	117.203643	98.9225797	246.108661	302.995733	108.197016	91.4150694	273.915304
AI606473	0.73390881	-0.2996694	0.32754647	-0.9148912	0.36024876	NA		AI606473	2.01613899	0.45252372	2.22655324	0	0	1.17605452	0	0
Nudt19	43.0425331	-0.3038793	0.33230544	-0.9144578	0.36047632	0.99938111		Nudt19	42.3389187	38.9170398	44.5310648	70.3167603	21.6425524	21.1689814	52.9245139	52.5004332
Layn	1.23473438	0.33221662	0.36333425	0.91435536	0.36053015	NA		Layn	0.67204633	0.90504744	0.9542371	0	4.99443516	2.35210905	0	0
Csnk1a1	150.139074	0.22030037	0.24098044	0.9141836	0.36062038	0.99938111		Csnk1a1	133.737219	145.260114	147.906751	124.088401	111.542385	184.64056	207.849	146.088162
Zkscan1	74.2111171	0.30327134	0.33184605	0.91389168	0.36077376	0.99938111		Zkscan1	78.6294204	61.5432258	74.7485731	43.4309402	39.9554813	130.542052	107.773556	57.0656883
Acot2	8.24024461	-0.3528643	0.38618226	-0.9137249	0.36086142	0.99938111		Acot2	11.4247876	9.05047438	6.99773876	12.4088401	8.3240586	3.52816357	9.62263889	4.56525506
Kazald1	1.12326584	-0.3149227	0.34468794	-0.9136459	0.36090295	NA		Kazald1	1.34409266	0.90504744	0.31807903	4.13628002	0	0	0	2.28262753
Gstcd	4.36186132	0.38020605	0.41615107	0.91362508	0.36091388	NA		Gstcd	5.37637063	1.35757116	5.72542262	0	3.32962344	5.88027262	8.660375	4.56525506
Fli1	1.86169111	-0.3672407	0.40220178	-0.9130757	0.36120271	NA		Fli1	0.67204633	4.52523719	2.86271131	2.06814001	1.66481172	1.17605452	1.92452778	0
Lair1	1.73314443	-0.3585601	0.3929472	-0.9124894	0.36151116	NA		Lair1	2.68818531	2.71514231	2.54463228	2.06814001	0	0	3.84905555	0
N4bp1	96.5023688	0.21314691	0.23365625	0.9122243	0.36165065	0.99938111		N4bp1	84.6778374	81.9067931	81.1101538	107.543281	111.542385	84.6759257	90.4528055	130.109769
Tmem178b	92.423101	0.26557879	0.29115008	0.91217143	0.36167848	0.99938111		Tmem178b	59.8121232	106.343074	66.4785182	97.2025805	104.883138	115.253343	61.5848889	127.827142
Mapk1ip1	12.9505261	-0.334836	0.36708353	-0.9121521	0.36168866	0.99938111		Mapk1ip1	17.4732045	14.480759	11.7689243	18.6132601	6.65924688	11.7605452	18.2830139	4.56525506
Prpf40b	29.7259242	0.32683722	0.35832991	0.9121126	0.36170945	0.99938111		Prpf40b	25.5377605	19.4585199	15.9039517	41.3628002	44.9499164	28.2253086	14.4339583	47.9351782
Rpp21	6.21284387	-0.3775043	0.41388205	-0.912106	0.36171291	0.99938111		Rpp21	12.0968339	6.33533207	7.95197586	4.13628002	4.99443516	0	9.62263889	4.56525506
Doc2b	40.1129136	-0.3061804	0.33571673	-0.9120202	0.36175807	0.99938111		Doc2b	45.027104	42.9897533	35.3067728	62.0442003	41.620293	37.6337448	47.1509305	9.13051012
Ceacam1	0.51523789	-0.2289267	0.25101527	-0.912003	0.36176712	NA		Ceacam1	0	3.16766603	0.9542371	0	0	0	0	0
Grcc10	99.4512655	-0.3467216	0.38020498	-0.9119334	0.36180376	0.99938111		Grcc10	74.5971425	76.4765085	67.1146763	248.176801	84.9053977	32.9295267	72.1697916	139.240279
Slc35g1	0.41722774	-0.2562952	0.28108132	-0.9118185	0.36186426	NA		Slc35g1	1.34409266	1.35757116	0.63615807	0	0	0	0	0
Ap2a1	261.625265	0.31654174	0.34717407	0.91176667	0.36189156	0.99938111		Ap2a1	235.216215	160.193397	187.66663	316.425422	461.152847	164.647633	138.566	429.133976
Slc7a4	33.9199266	0.36430231	0.39959453	0.91167991	0.36193724	0.99938111		Slc7a4	16.8011582	47.9675142	26.4005599	16.5451201	86.5702095	36.4576902	8.660375	31.9567854
Vsig10l	3.06143708	-0.3794841	0.4163442	-0.9114672	0.36204925	NA		Vsig10l	3.36023164	2.71514231	3.49886938	6.20442003	3.32962344	1.17605452	1.92452778	2.28262753
Aspg	0.76905238	-0.2298693	0.2522279	-0.9113556	0.36210806	NA		Aspg	2.01613899	0	0	4.13628002	0	0	0	0
Ngef	107.565207	-0.2945881	0.32324416	-0.9113486	0.36211174	0.99938111		Ngef	151.210424	104.080455	116.098848	109.611421	143.173808	62.3308898	46.1886666	127.827142
P2ry14	1.42117907	-0.352211	0.38648029	-0.9113297	0.3621217	NA		P2ry14	0.67204633	3.62018975	1.90847421	2.06814001	0	1.17605452	1.92452778	0
Cdh12	17.4905326	0.36185516	0.39709086	0.91126538	0.36215556	0.99938111		Cdh12	11.4247876	21.7211385	20.3570582	2.06814001	13.3184938	38.8097993	23.0943333	9.13051012
Cdh9	15.0273455	0.37794889	0.41475481	0.91125861	0.36215913	0.99938111		Cdh9	11.4247876	17.1959013	14.3135566	0	8.3240586	45.8661264	23.0943333	0
Inf2	46.0905693	-0.331112	0.3633999	-0.9111506	0.36221605	0.99938111		Inf2	69.8928182	45.7048956	56.9361472	37.2265202	68.2572805	17.6408179	18.2830139	54.7830607
Il1rap	7.10547211	-0.3738256	0.41035682	-0.910977	0.36230748	0.99938111		Il1rap	8.06455594	15.3858064	9.224292	2.06814001	4.99443516	9.40843619	7.69811111	0
Ino80e	32.3986734	0.32169404	0.35315686	0.91090979	0.36234291	0.99938111		Ino80e	28.8979921	25.3413283	25.7644018	28.9539601	46.6147282	15.2887088	22.1320694	66.1961984
Trip13	0.68697542	0.28381712	0.31185595	0.91009042	0.36277483	NA		Trip13	0.67204633	0	0.31807903	0	3.32962344	1.17605452	0	0
Abcd1	6.12109104	-0.3626481	0.39851838	-0.9099909	0.3628273	0.99938111		Abcd1	6.72046328	9.95552182	5.08926455	8.27256004	4.99443516	5.88027262	5.77358333	2.28262753
Ski	163.342919	0.22452009	0.24675801	0.90987964	0.36288599	0.99938111		Ski	146.5061	159.288349	159.039517	128.224681	216.425524	119.957561	155.88675	221.414871
4930427A07Rik	0.75428415	0.29815073	0.3277299	0.90974529	0.36295685	NA		4930427A07Ri	0.67204633	0.45252372	0	0	1.66481172	0	0.96226389	2.28262753
Vdr	0.75428415	0.29815073	0.3277299	0.90974529	0.36295685	NA		Vdr	0.67204633	0.45252372	0	0	1.66481172	0	0.96226389	2.28262753
Ibtk	19.9399624	0.28591835	0.31436915	0.90949877	0.36308691	0.99938111		Ibtk	15.4570656	21.7211385	17.1762679	16.5451201	21.6425524	21.1689814	29.8301805	15.9783927
Prpsap1	19.5556622	-0.2981505	0.32784808	-0.9094166	0.36313027	0.99938111		Prpsap1	22.8495752	23.0787097	18.448584	24.8176801	8.3240586	15.2887088	23.0943333	20.5436478
Nipsnap3b	9.88731733	0.3547522	0.39009611	0.90939693	0.36314064	0.99938111		Nipsnap3b	8.73660227	9.5029981	7.95197586	6.20442003	4.99443516	5.88027262	22.1320694	13.6957652
Ccdc88c	6.91102759	0.36934961	0.40614804	0.90939651	0.36314086	0.99938111		Ccdc88c	4.7043243	5.88280835	5.40734359	6.20442003	14.9833055	12.9365998	2.88679167	2.28262753
Smdt1	45.7760635	-0.2814642	0.30951974	-0.9093578	0.36316128	0.99938111		Smdt1	55.7798453	39.8220873	39.7598793	70.3167603	28.3017992	27.0492541	48.1131944	57.0656883

GeneID	Base mean	log2(FC)	StdErr	Wald-Stats	P-value	P-adj	GeneID	Normalised expression for Chow#1	Normalised expression for Chow#2	Normalised expression for Chow#3	Normalised expression for Chow#4	Normalised expression for HFD#1	Normalised expression for HFD#2	Normalised expression for HFD#3	Normalised expression for HFD#4
Sdcbp2	0.81629508	-0.3081052	0.33883543	-0.9093063	0.36318845	NA	Sdcbp2	2.68818531	0.90504744	1.27231614	0	1.66481172	0	0	0
Atg4d	16.9457832	0.33990665	0.37400241	0.90883544	0.363437	0.99938111	Atg4d	14.7850192	8.14542694	16.2220308	16.5451201	21.6425524	8.23238167	13.4716944	36.5220405
Cyb5r4	11.9315739	0.35573632	0.39145604	0.90875165	0.36348123	0.99938111	Cyb5r4	7.39250961	5.88280835	9.86045007	16.5451201	14.9833055	3.52816357	14.4339583	22.8262753
Msrb2	10.149983	-0.3660918	0.4028572	-0.9087383	0.36348829	0.99938111	Msrb2	8.06455594	10.4080455	13.6773985	16.5451201	1.66481172	2.35210905	12.5094306	15.9783927
Gm11747	1.07412679	0.2982722	0.32826119	0.90864291	0.36353865	NA	Gm11747	0	1.35757116	0.31807903	0	0	2.35210905	0	4.56525506
Aldh3b1	1.56936687	-0.3556004	0.39145964	-0.908396	0.36366906	NA	Aldh3b1	2.68818531	1.35757116	1.27231614	4.13628002	0	1.17605452	1.92452778	0
Mxd4	18.9495372	-0.3035134	0.33416469	-0.9082749	0.36373301	0.99938111	Mxd4	28.2259458	18.1009488	23.2197695	14.4769801	24.9721758	14.1126543	12.5094306	15.9783927
Hax1	9.40008022	-0.3761211	0.41420963	-0.9080454	0.36385425	0.99938111	Hax1	6.72046328	6.78785578	5.08926455	28.9539601	8.3240586	1.17605452	6.73584722	11.4131377
Kdm5a	41.2781693	0.29011056	0.31950183	0.90800907	0.36387343	0.99938111	Kdm5a	26.8818531	36.6544212	33.3982986	51.7035003	36.6258578	71.739326	48.1131944	25.1089028
Rab6b	983.560711	0.15074345	0.16602229	0.90797116	0.36389346	0.99938111	Rab6b	969.762852	916.813055	958.69021	870.686944	1067.14431	889.09722	1034.43368	1161.85741
Mks1	1.57095134	-0.3368511	0.37104067	-0.9078548	0.36395492	NA	Mks1	0.67204633	1.81009488	0.63615807	6.20442003	0	0	0.96226389	2.28262753
Gm16386	3.45050914	0.376787	0.4150917	0.90771992	0.36402622	NA	Gm16386	2.68818531	3.16766603	2.54463228	2.06814001	1.66481172	7.05632714	3.84905555	4.56525506
N6amt1	11.0325103	0.33438058	0.36837903	0.90770796	0.36403253	0.99938111	N6amt1	13.4409266	9.05047438	10.1785291	4.13628002	8.3240586	17.6408179	16.3584861	9.13051012
Mlh1	2.98841893	-0.3774179	0.41579501	-0.907702	0.36403571	NA	Mlh1	3.36023164	5.88280835	3.18079035	2.06814001	4.99443516	1.17605452	0.96226389	2.28262753
Pdcd2l	5.89394249	0.36491065	0.40211676	0.90747436	0.364156	0.99938111	Pdcd2l	3.36023164	2.71514231	6.67965972	6.20442003	6.65924688	4.7042181	7.69811111	9.13051012
Rps6ka6	7.26937138	0.36425598	0.40140654	0.90744906	0.36416937	0.99938111	Rps6ka6	6.04841696	9.5029981	5.40734359	2.06814001	8.3240586	5.88027262	16.3584861	4.56525506
Mrps25	84.3687483	0.22895701	0.25234508	0.90731711	0.36423913	0.99938111	Mrps25	69.2207718	66.9735104	78.5655215	97.2025805	99.8887032	84.6759257	114.509403	63.9135709
Tctn3	7.67104682	-0.3581024	0.39476168	-0.9071357	0.36433504	0.99938111	Tctn3	6.04841696	12.6706641	10.4966081	6.20442003	11.653682	5.88027262	3.84905555	4.56525506
Fbxw2	42.0544436	0.24332204	0.26826239	0.90703	0.36439093	0.99938111	Fbxw2	37.6345944	37.5594687	42.3045116	33.0902402	43.2851047	32.9295267	54.8490416	54.7830607
Gm9855	7.17269439	0.35644755	0.39302615	0.90693087	0.36444335	0.99938111	Gm9855	6.72046328	7.69290322	5.40734359	2.06814001	13.3184938	8.23238167	4.81131944	9.13051012
Tdg	7.17269439	0.35644755	0.39302615	0.90693087	0.36444335	0.99938111	Tdg	6.72046328	7.69290322	5.40734359	2.06814001	13.3184938	8.23238167	4.81131944	9.13051012
Sfrp2	2.96751378	-0.3775212	0.41627548	-0.9069023	0.36445847	NA	Sfrp2	2.68818531	5.43028463	4.77118552	2.06814001	3.32962344	3.52816357	1.92452778	0
Dlg5	49.8699112	-0.2751745	0.30353261	-0.9065733	0.36463251	0.99938111	Dlg5	61.8282622	42.9897533	41.6683535	76.5211804	63.2628454	35.2816357	31.7547083	45.6525506
Ralgapa1	123.451265	0.28384818	0.3131209	0.90651305	0.36466436	0.99938111	Ralgapa1	125.000617	118.561214	129.458167	62.0442003	113.207197	183.464506	182.830139	73.044081
Opa3	63.6636376	0.31604346	0.34872457	0.90628388	0.36478562	0.99938111	Opa3	41.6668724	53.8503226	48.0299342	74.4530404	91.5646446	37.6337448	36.5660278	125.544514
Spint1	0.42786248	-0.2516061	0.27768465	-0.9060856	0.36489055	NA	Spint1	2.01613899	0.45252372	0.9542371	0	0	0	0	0
Klc4	8.29267269	0.35851462	0.39568184	0.90606791	0.36489991	0.99938111	Klc4	8.73660227	5.88280835	6.36158069	4.13628002	9.98887032	5.88027262	4.81131944	20.5436478
Stxbp1	995.854731	0.20833783	0.22993778	0.90606176	0.36490316	0.99938111	Stxbp1	1012.77382	738.066186	870.582317	1048.54699	1283.56984	932.611238	809.26393	1271.42353
Relt	6.07509005	0.37281536	0.41149889	0.9059936	0.36493924	0.99938111	Relt	1.34409266	4.52523719	3.81694841	10.3407001	8.3240586	8.23238167	2.88679167	9.13051012
Tmem55b	84.4804761	0.26127166	0.28844202	0.90580303	0.36504012	0.99938111	Tmem55b	59.1400769	64.2583681	74.112415	105.475141	86.5702095	65.8590533	78.9056389	141.522907
Myh7b	4.49130256	0.37567424	0.41486697	0.90552942	0.36518498	NA	Myh7b	4.7043243	1.81009488	2.22655324	4.13628002	13.3184938	0	2.88679167	6.84788259
Ipw	2.76254391	0.37597989	0.4152273	0.90547969	0.36521131	NA	Ipw	3.36023164	0.90504744	3.18079035	0	4.99443516	3.52816357	3.84905555	2.28262753
Ap1s2	83.7945858	0.30653084	0.33861088	0.90525986	0.36532774	0.99938111	Ap1s2	57.7959842	70.5937002	87.4717345	76.5211804	43.2851047	152.887088	127.018833	54.7830607
Nell2	194.294427	-0.3353512	0.37065796	-0.9047457	0.36560011	0.99938111	Nell2	202.957991	373.784592	257.962097	64.1123403	186.458913	259.90805	108.735819	100.435611
Rtn4ip1	12.8754091	0.34111252	0.37704773	0.90469321	0.36562794	0.99938111	Rtn4ip1	9.4086486	6.78785578	10.8146872	16.5451201	24.9721758	11.7605452	6.73584722	15.9783927
Lhx1	2.12470548	-0.1775241	0.19624837	-0.904589	0.36568314	NA	Lhx1	0	0.45252372	0	16.5451201	0	0	0	0
Ythdf2	28.1170008	0.29470466	0.32579902	0.9045597	0.3656987	0.99938111	Ythdf2	34.9464091	23.5312334	29.2632712	8.27256004	29.966611	31.7534722	37.5282917	29.6741579
Acly	105.47621	0.25268796	0.27936278	0.90451547	0.36572214	0.99938111	Acly	120.296293	76.9290322	92.560999	88.9300204	179.799666	90.5561984	94.3018611	100.435611
Ctdsp2	51.7997629	-0.3118588	0.3448509	-0.9043295	0.36582071	0.99938111	Ctdsp2	46.3711967	53.3977988	36.2610099	101.338861	73.2517157	30.5774176	29.8301805	43.3699231
Gpx4	39.0656093	-0.3710275	0.4103145	-0.9042514	0.36586211	0.99938111	Gpx4	39.6507334	21.2686148	26.0824808	103.407001	44.9499164	4.7042181	15.3962222	57.0656883
Gpr25	24.1593847	0.36425338	0.40287717	0.90413011	0.36592642	0.99938111	Gpr25	14.1129729	11.7656167	14.6316356	37.2265202	31.6314227	9.40843619	10.5849028	63.9135709
Osbpl9	33.0426835	0.30543174	0.33792073	0.90385617	0.36607167	0.99938111	Osbpl9	36.9625481	28.0564706	31.4898244	35.1583802	36.6258578	41.1619083	57.7358333	13.6957652
Ppp3cc	12.9395629	-0.3483257	0.38548676	-0.9035997	0.3662077	0.99938111	Ppp3cc	24.8657142	12.6706641	18.766663	4.13628002	8.3240586	10.5844907	17.32075	6.84788259
Trpc5	15.378429	0.31378375	0.34732903	0.90341928	0.36630341	0.99938111	Trpc5	9.4086486	18.5534725	15.9039517	8.27256004	18.3129289	19.9929269	21.1698055	11.4131377
Acer2	3.84680485	-0.3662373	0.40540423	-0.9033879	0.36632006	NA	Acer2	2.68818531	5.89795066	9.224292	0	0	3.52816357	6.73584722	0
Sik2	27.8060634	0.28062277	0.31082773	0.90282411	0.36661925	0.99938111	Sik2	28.2259458	23.5312334	28.6271131	16.5451201	43.2851047	37.6337448	24.0565972	20.5436478
Bcat2	2.24035336	-0.3644297	0.40371364	-0.9026935	0.36668861	NA	Bcat2	4.7043243	1.81009488	1.59039517	4.13628002	3.32962344	2.35210905	0	0
Pphln1	28.7790058	0.29967474	0.33205066	0.90249705	0.36679289	0.99938111	Pphln1	28.8979921	27.6039469	26.0824808	18.6132601	21.6425524	42.3379629	49.0754583	15.9783927
Kcnj11	25.9926934	0.33818053	0.37476207	0.90238729	0.36685117	0.99938111	Kcnj11	20.1613899	21.7211385	19.4028211	24.8176801	53.2739751	18.8168724	8.660375	41.0872956
Pdzd4	82.2896243	0.2224356	0.24651394	0.90232463	0.36688445	0.99938111	Pdzd4	88.038069	61.0907021	59.7988585	95.1344405	86.5702095	82.3238167	89.4905416	95.8703563
Ddx26b	4.3145195	0.37531972	0.41600038	0.90221005	0.3669453	NA	Ddx26b	2.01613899	5.43028463	3.81694841	2.06814001	3.32962344	8.23238167	9.62263889	0
Gramd2	1.24658062	0.3465143	0.38422322	0.90185675	0.36713297	NA	Gramd2	1.34409266	0.45252372	1.27231614	0	1.66481172	2.35210905	2.88679167	0
BC037034	75.3955802	0.36027269	0.39949935	0.90181045	0.36715757	0.99938111	BC037034	36.2905017	35.7493738	42.3045116	130.292821	119.866444	37.6337448	29.8301805	171.197065
Ghitm	316.770219	0.23827213	0.264259	0.90166138	0.36723678	0.99938111	Ghitm	256.049651	216.758861	261.779045	417.764282	274.693934	306.950231	336.792361	463.373389
Pdlim1	2.38366441	0.36627058	0.4062908	0.90149859	0.36732328	NA	Pdlim1	0.67204633	1.35757116	2.22655324	2.06814001	8.3240586	1.17605452	0.96226389	2.28262753
Inadl	7.57751311	-0.3470531	0.38503694	-0.9013502	0.36740213	0.99938111	Inadl	8.73660227	7.2403795	10.1785291	10.3407001	6.65924688	9.40843619	5.77358333	2.28262753
Rrp36	7.14560873	0.36681267	0.4069854	0.90129195	0.36743311	0.99938111	Rrp36	8.06455594	1.35757116	5.72542262	8.27256004	13.3184938	5.88027262	7.69811111	6.84788259
Scyl1	46.6927258	0.23499351	0.26076476	0.90117051	0.36749767	0.99938111	Scyl1	43.010965	34.8443264	47.3937761	43.4309402	63.2628454	43.5140174	43.301875	54.7830607
Taf1	64.2066229	0.31785184	0.3527225	0.90113853	0.36751467	0.99938111	Taf1	65.1884939	52.0402277	64.251965	39.2946602	49.9443516	137.598379	77.943375	27.3915304
Timm17b	19.5521281	-0.3719382	0.41274573	-0.9011316	0.36751837	0.99938111	Timm17b	12.0968339	9.05047438	9.86045007	66.1804803	9.98887032	7.05632714	12.5094306	29.6741579
Cln6	12.0170108	0.36379807	0.40372124	0.90111203	0.36752876	0.99938111	Cln6	6.04841696	14.9332827	9.224292	6.20442003	21.6425524	5.88027262	4.81131944	27.3915304
Tra2b	15.1594094	0.36396111	0.40394664	0.90101284	0.36758149	0.99938111	Tra2b	20.1613899	14.0282353	12.0870033	2.06814001	9.98887032	24.697145	33.6792361	4.56525506
E4f1	6.80397566	0.36495791	0.40514837	0.90080062	0.36769434	0.99938111	E4f1	7.39250961	3.62018975	2.86271131	8.27256004	8.3240586	3.52816357	6.73584722	13.6957652
Spry2	39.0766164	-0.2525765	0.28046	-0.9005793	0.36781202	0.99938111	Spry2	54.4357526	37.106945	43.5768277	33.0902402	29.966611	28.2253086	38.4905555	45.6525506
Cpd	67.2770101	-0.3360057	0.37321631	-0.9002975	0.36796196	0.99938111	Cpd	54.4357526	132.136926	99.5587378	24.8176801	56.6035985	94.0843619	42.3396111	34.239413
Lrp4	21.1666146	-0.3509	0.38977911	-0.9002534	0.36798541	0.99938111	Lrp4	16.1291119	45.1796584	22.5836114	18.6132601	39.9554813	11.7605452	7.69811111	11.4131377
Pcmtd2	39.1138321	0.27539137	0.30595947	0.90009101	0.36807182	0.99938111	Pcmtd2	45.6991503	36.6544212	37.5333261	16.5451201	29.966611	44.6900719	53.8867778	47.9351782
Krr1	28.6143449	0.34717209	0.38572674	0.90004674	0.36809538	0.99938111	Krr1	26.8818531	33.0342315	29.5813502	4.13628002	19.9777406	54.0985081	45.2264028	15.9783927
Phactr3	79.4234522	-0.2534705	0.28163809	-0.8999865	0.36812745	0.99938111	Phactr3	116.264015	95.4825047	88.4259716	47.5672202	59.9332219	69.3872169	87.5660139	70.7614535
Mef2d	174.658643	-0.2707779	0.30087648	-0.8999637	0.36813959	0.99938111	Mef2d	191.533204	186.892296	172.716916	223.359121	251.38657	109.373071	88.5282777	173.479692
Atg3	22.4254833	0.33075953	0.36765106	0.89965613	0.36830327	0.99938111	Atg3	28.2259458	18.5534725	21.9474534	6.20442003	11.653682	37.6337448	34.6415	20.5436478
Cep76	6.76417177	0.36333408	0.40388311	0.89960206	0.36833206	0.99938111	Cep76	4.7043243	4.97776091	3.49886938	10.3407001	9.98887032	3.52816357	12.5094306	4.56525506
Tnks	84.8658726	0.27414995	0.3048561	0.89927656	0.36850537	0.99938111	Tnks	70.5648645	95.029981	85.2451812	49.6353602	76.5813391	151.711034	88.5282777	61.6309433
Impa2	1.0200972	0.30063011	0.33434058	0.89917326	0.36856038	NA	Impa2	0.67204633	0	1.27231614	0	3.32962344	0	2.88679167	0
Elp2	37.2021298	0.29615495	0.32944484	0.89895158	0.36867845	0.99938111	Elp2	31.5861774	20.8160911	22.9016905	57.9079203	51.6091633	30.5774176	36.5660278	45.6525506
Taf2	27.5506391	0.27520858	0.30615467	0.89892007	0.36869524	0.99938111	Taf2	18.1452509	29.4140417	27.990955	22.7495401	24.9721758	32.9295267	41.3773472	22.8262753
Foxb1	1.03407001	-0.200406	0.22295395	-0.8988673	0.36872333	NA	Foxb1	0	0	0	8.27256004	0	0	0	0
Sucla2	91.1203496	0.31728103	0.35302556	0.89874803	0.36878689	0.99938111	Sucla2	94.7585323	81.4542694	85.2451812	51.7035003	46.6147282	143.478652	173.2075	52.5004332
Brsk1	202.898513	0.27860972	0.31004492	0.89861082	0.36885999	0.99938111	Brsk1	203.630038	155.668159	142.817486	215.086561	321.308662	137.598379	136.641472	310.437344
Prss23	4.0658722	-0.3713532	0.41342919	-0.8982269	0.36906462	NA	Prss23	3.36023164	12.2181404	5.08926455	0	1.66481172	1.17605452	6.73584722	2.28262753
Prpf31	14.8255642	0.30814328	0.34312314	0.8980545	0.36915649	0.99938111	Prpf31	16.1291119	13.1231878	13.3593194	8.27256004	23.3073641	14.1126543	21.1698055	9.13051012
Sppl2b	9.81033232	-0.3411633	0.37990777	-0.8980161	0.36917694	0.99938111	Sppl2b	8.06455594	14.9332827	7.95197586	14.4769801	9.98887032	5.88027262	5.77358333	11.4131377
Cdv3	47.2642278	0.25490481	0.283867	0.89797268	0.36920012	0.99938111	Cdv3	45.027104	41.6321821	46.7576181	37.2265202	31.6314227	67.0351079	65.4339444	43.3699231
2310010J17Rik	0.65886183	0.255624	0.28470323	0.89786126	0.36925952	NA	2310010J17Ri	0	0	0.63615807	0	0	2.35210905	0	2.28262753
Eapp	15.4876242	0.35394333	0.39422089	0.89782997	0.36927621	0.99938111	Eapp	17.4732045	17.1959013	14.6316356	0	11.653682	29.4013631	22.1320694	11.4131377
Gatad2b	53.2628501	-0.2797461	0.31162475	-0.8977018	0.36934456	0.99938111	Gatad2b	71.2369108	72.403795	62.6615698	31.0221002	33.2962344	63.5069443	57.7358333	34.239413
Nr5a1	1.84938239	-0.175479	0.19548296	-0.8976689	0.36936209	NA	Nr5a1	0	0	0.31807903	14.4769801	0	0	0	0
Gm15760	1.62387846	0.3083935	0.34356268	0.89763388	0.36938077	NA	Gm15760	0	0.90504744	1.90847421	0	3.32962344	0	0	6.84788259
Alyref	8.88032857	0.36336526	0.40485875	0.8975112	0.3694462	0.99938111	Alyref	6.04841696	4.97776091	8.58813393	8.27256004	3.32962344	3.52816357	13.4716944	22.8262753
Dhtkd1	1.9987153	0.36502233	0.40674036	0.89743326	0.36948777	NA	Dhtkd1	2.01613899	1.81009488	0.9542371	0	3.32962344	2.35210905	0.96226389	4.56525506
Ubl5	50.4280119	-0.2832345	0.31565749	-0.8972844	0.3695672	0.99938111	Ubl5	45.6991503	42.9897533	43.2587487	97.2025805	51.6091633	30.5774176	50.9999861	41.0872956
Emx1	5.38614969	0.37255169	0.41521866	0.89724216	0.36958971	0.99938111	Emx1	6.04841696	3.16766603	3.81694841	2.06814001	21.6425524	1.17605452	2.88679167	2.28262753
Zfp84	17.9497488	0.30791253	0.34333334	0.8968326	0.36980825	0.99938111	Zfp84	12.0968339	14.480759	23.5378486	10.3407001	14.9833055	27.0492541	18.2830139	22.8262753
Tcte1	14.8336993	0.3229085	0.36006469	0.89680692	0.36982195	0.99938111	Tcte1	6.72046328	13.5757116	14.9497146	16.5451201	9.98887032	25.8731995	17.32075	13.6957652
Ap1g1	94.7669713	0.21919614	0.24447452	0.89660116	0.36993178	0.99938111	Ap1g1	76.6132814	100.460266	102.421449	66.1804803	106.54795	121.133616	104.886764	79.8919636
Mrpl23	3.33029581	-0.3732333	0.41628922	-0.8965721	0.36994732	NA	Mrpl23	7.39250961	3.62018975	3.81694841	2.06814001	3.32962344	3.52816357	2.88679167	0
Gm10012	4.81056382	-0.3732555	0.41634758	-0.8964998	0.36998587	NA	Gm10012	9.4086486	7.69290322	7.31581779	0	1.66481172	4.7042181	7.69811111	0
1700030K09Rik	4.74561999	0.37320874	0.41631088	0.89646645	0.37000369	0.99938111	1700030K09Ri	0.67204633	3.16766603	2.22655324	10.3407001	6.65924688	5.88027262	6.73584722	2.28262753
C130021I20Rik	1.80962251	-0.1751025	0.19534116	-0.8963933	0.37004272	NA	C130021I20Ri	0	0	0	14.4769801	0	0	0	0
Pitx2	1.80962251	-0.1751025	0.19534116	-0.8963933	0.37004272	NA	Pitx2	0	0	0	14.4769801	0	0	0	0
Rhno1	9.05326609	-0.3340887	0.37273213	-0.8963238	0.37007985	0.99938111	Rhno1	13.4409266	10.8605693	11.4508452	6.20442003	6.65924688	10.5844907	8.660375	4.56525506
Zfp488	2.23092522	-0.3565979	0.39785341	-0.8963047	0.37009007	NA	Zfp488	2.68818531	1.81009488	5.40734359	0	0	0	3.84905555	2.28262753
Fos	14.2271132	-0.357707	0.39920911	-0.8960393	0.3702318	0.99938111	Fos	16.1291119	4.97776091	19.7209001	26.8858201	4.99443516	10.5844907	7.69811111	22.8262753
Prosc	32.2633607	-0.2646167	0.29531846	-0.8960386	0.37023215	0.99938111	Prosc	39.6507334	36.6544212	36.579089	31.0221002	18.3129289	29.4013631	41.3773472	25.1089028
Eno2	769.132865	0.31885664	0.35587031	0.89599113	0.3702575	0.99938111	Eno2	678.094745	395.958254	512.107246	1046.47885	1205.32369	437.492283	471.509305	1406.09856
Cd83	8.88217409	-0.369391	0.41228028	-0.8959706	0.37026849	0.99938111	Cd83	4.7043243	15.3858064	9.54237104	14.4769801	0	15.2887088	4.81131944	6.84788259
1700034P13Rik	8.48211525	0.35323226	0.39433133	0.89577528	0.37037279	0.99938111	1700034P13Ri	10.0806949	5.88280835	5.40734359	6.20442003	16.6481172	12.9365998	3.84905555	6.84788259
Scaf11	41.6414435	0.24601607	0.27472077	0.89551315	0.37051284	0.99938111	Scaf11	41.6668724	34.3918026	39.7598793	35.1583802	33.2962344	63.5069443	44.2641389	41.0872956
Ttc19	54.4879651	0.27333212	0.30530924	0.89526317	0.37064642	0.99938111	Ttc19	49.7314283	54.75537	62.3434908	24.8176801	48.2795399	85.8519803	64.4716805	45.6525506
Pnrc2	25.1197595	-0.3231945	0.36102639	-0.8952103	0.37067471	0.99938111	Pnrc2	30.2420848	37.5594687	31.8079035	16.5451201	9.98887032	25.8731995	37.5282917	11.4131377
Psmb5	23.8972566	0.36857301	0.4117276	0.89518654	0.37068738	0.99938111	Psmb5	6.72046328	13.5757116	12.0870033	43.4309402	24.9721758	4.7042181	24.0565972	61.6309433
Nop2	11.0035697	-0.3247714	0.3630077	-0.8946681	0.37096456	0.99938111	Nop2	16.1291119	9.05047438	12.4050823	12.4088401	11.653682	8.23238167	6.73584722	11.4131377

GeneID	Base mean	log2(FC)	StdErr	Wald-Stats	P-value	P-adj	GeneID	Normalised expression for Chow#1	Normalised expression for Chow#2	Normalised expression for Chow#3	Normalised expression for Chow#4	Normalised expression for HFD#1	Normalised expression for HFD#2	Normalised expression for HFD#3	Normalised expression for HFD#4
Parp9	0.73224613	-0.2955803	0.33042232	-0.894553	0.3710261	NA	Parp9	2.68818531	1.35757116	0.63615807	0	0	1.17605452	0	0
Afap1l2	3.10823672	-0.3723011	0.41625234	-0.894412	0.37110147	NA	Afap1l2	2.01613899	6.33533207	4.77118552	2.06814001	3.32962344	1.17605452	2.88679167	2.28262753
Klhdc1	16.406129	0.34088102	0.38119216	0.89424982	0.37118824	0.99938111	Klhdc1	10.0806949	14.9332827	22.9016905	6.20442003	11.653682	29.4013631	26.9433889	9.13051012
Gpr155	32.975842	-0.3590944	0.40157273	-0.89422	0.37120417	0.99938111	Gpr155	28.8979921	65.6159392	50.5745665	12.4088401	18.3129289	59.9787807	21.1698055	6.84788259
Tcirg1	3.36086284	0.35621755	0.39835906	0.89421223	0.37120835	NA	Tcirg1	2.01613899	3.62018975	0.9542371	2.06814001	14.9833055	0	0.96226389	2.28262753
Zmat3	224.89548	0.22614812	0.2529056	0.89419974	0.37121503	0.99938111	Zmat3	149.866331	201.825579	198.163238	272.994481	307.990168	190.520833	210.735792	267.067421
Rif1	27.7368175	0.32678248	0.36546352	0.89415896	0.37123685	0.99938111	Rif1	20.1613899	29.4140417	31.8079035	12.4088401	16.6481172	54.0985081	41.3773472	15.9783927
Fam184a	12.0488688	0.36412401	0.40728909	0.89401858	0.37131195	0.99938111	Fam184a	15.4570656	8.59795066	13.0412404	0	3.32962344	18.8168724	21.1698055	15.9783927
Elp6	4.60185285	-0.3722206	0.41636671	-0.8939729	0.3713364	NA	Elp6	6.72046328	3.62018975	3.81694841	8.27256004	3.32962344	0	1.92452778	9.13051012
Rasgrf2	134.327121	0.19209492	0.21494361	0.89369915	0.37148288	0.99938111	Rasgrf2	136.425405	108.605693	120.870033	136.497241	141.508996	169.351851	147.226375	114.131377
Zbtb7a	134.413695	0.34517005	0.38625259	0.89363814	0.37151553	0.99938111	Zbtb7a	84.0057911	71.9512713	67.1146763	223.359121	256.381005	72.9153805	64.4716805	235.110636
A330040F15Rik	4.79947138	0.34990681	0.39175162	0.89318535	0.37175792	0.99938111	A330040F15Ri	0.67204633	2.26261859	0	10.3407001	8.3240586	1.17605452	1.92452778	13.6957652
Ccbe1	4.66023362	0.37161531	0.41616484	0.89295219	0.37188278	0.99938111	Ccbe1	2.01613899	2.71514231	3.18079035	6.20442003	1.66481172	5.88027262	1.92452778	13.6957652
Zfp275	39.3096889	0.27000277	0.30243252	0.89277031	0.37198019	0.99938111	Zfp275	29.5700384	28.0564706	32.1259825	51.7035003	31.6314227	47.042181	32.7169722	61.6309433
Dpm2	33.5406274	-0.2840515	0.3182407	-0.892568	0.37208857	0.99938111	Dpm2	32.9302701	39.3695635	26.7186389	51.7035003	39.9554813	16.4647633	26.9433889	34.239413
Hdgfrp3	126.529589	0.33648043	0.37699456	0.89253392	0.37210682	0.99938111	Hdgfrp3	117.608107	118.108691	124.686982	64.1123403	53.2739751	228.154578	249.226347	57.0656883
Bche	2.017592	-0.3526434	0.39512904	-0.8924764	0.37213762	NA	Bche	2.68818531	5.88280835	2.54463228	0	0	1.17605452	3.84905555	0
Zcchc6	25.709297	0.34211213	0.38341136	0.8922848	0.3722403	0.99938111	Zcchc6	30.9141311	22.1736622	24.1740066	8.27256004	18.3129289	38.8097993	53.8867778	9.13051012
Cd93	2.5748739	0.34855476	0.39064202	0.89226131	0.37225289	NA	Cd93	0.67204633	2.26261859	1.59039517	2.06814001	11.653682	2.35210905	0	0
Slc26a4	1.50013949	0.33979804	0.38084885	0.89221234	0.37227913	NA	Slc26a4	2.01613899	0.90504744	0.9542371	0	0	2.35210905	5.77358333	0
Capn5	55.8055209	0.31626604	0.35448843	0.89217591	0.37229865	0.99938111	Capn5	41.6668724	36.6544212	30.2175083	84.7937404	101.553515	32.9295267	43.301875	75.3267085
Mtr	7.00163309	-0.3472271	0.38926371	-0.89201	0.37238758	0.99938111	Mtr	11.4247876	6.33533207	7.31581779	8.27256004	6.65924688	4.7042181	6.73584722	4.56525506
Tph1	0.53267323	0.25263793	0.28340988	0.89142246	0.37270257	NA	Tph1	0.67204633	0	0	0	1.66481172	0	1.92452778	0
Bbs12	2.89300944	0.37031684	0.41543029	0.89140551	0.37271166	NA	Bbs12	1.34409266	1.35757116	2.54463228	4.13628002	4.99443516	5.88027262	2.88679167	0
Akap9	69.1389055	0.36298455	0.40725325	0.89129934	0.3727686	0.99938111	Akap9	69.2207718	63.8058444	71.8858618	14.4769801	36.6258578	199.929269	78.9056389	18.2610202
Slc7a6	8.26100109	-0.3516513	0.39457884	-0.8912067	0.37281832	0.99938111	Slc7a6	5.37637063	12.2181404	10.8146872	10.3407001	11.653682	8.23238167	2.88679167	4.56525506
Soat1	25.5769224	-0.3283814	0.36851988	-0.8910819	0.37288526	0.99938111	Soat1	23.5216215	49.3250854	31.8079035	12.4088401	26.6369875	34.1055812	15.3962222	11.4131377
A930015D03Rik	3.23082485	-0.3660559	0.41084248	-0.8909883	0.37293543	NA	A930015D03R	2.01613899	4.52523719	0.9542371	10.3407001	1.66481172	1.17605452	2.88679167	2.28262753
Hspg2	7.1294286	0.37075933	0.41636859	0.89045941	0.37321925	0.99938111	Hspg2	4.03227797	4.52523719	4.45310648	8.27256004	23.3073641	2.35210905	0.96226389	9.13051012
Slc5a2	3.19141772	0.37022074	0.41578807	0.89040732	0.37324721	NA	Slc5a2	2.01613899	2.26261859	1.27231614	4.13628002	3.32962344	4.7042181	0.96226389	6.84788259
Gpr180	6.24028001	-0.3644543	0.40936315	-0.8902959	0.37330702	0.99938111	Gpr180	4.7043243	13.5757116	7.31581779	4.13628002	3.32962344	9.40843619	2.88679167	4.56525506
Ube2k	114.084876	0.28220308	0.31701274	0.89019476	0.37336132	0.99938111	Ube2k	106.18332	106.795598	126.913535	62.0442003	69.9220923	143.478652	199.188625	98.1529838
Smc6	32.9548568	0.33999525	0.38207786	0.88985854	0.37354185	0.99938111	Smc6	37.6345944	22.6261859	35.3067728	14.4769801	14.9833055	65.8590533	56.7735694	15.9783927
Micall1	42.3998626	0.31293859	0.3517427	0.88968041	0.37363752	0.99938111	Micall1	33.6023164	32.5817078	31.1717454	47.5672202	66.5924688	24.697145	23.0943333	79.8919636
Trp73	0.6243044	0.19469038	0.21885132	0.88960109	0.37368012	NA	Trp73	0	0	0	0	4.99443516	0	0	0
Ppp1r11	52.0920244	0.22868925	0.2570956	0.88951056	0.37372875	0.99938111	Ppp1r11	51.075521	42.0847059	56.6180681	37.2265202	59.9332219	45.8661264	57.7358333	66.1961984
Usp5	136.389279	0.29792594	0.33493846	0.88949456	0.37373734	0.99938111	Usp5	106.18332	69.6886527	89.0621297	213.018421	204.771842	115.253343	99.1131805	194.02334
Ablim2	97.2712266	-0.2374761	0.26705751	-0.8892321	0.37387834	0.99938111	Ablim2	125.000617	107.248121	92.8790781	97.2025805	119.866444	69.3872169	61.5848889	105.000866
Nbl1	14.4151259	-0.3506393	0.39436145	-0.8891318	0.37393224	0.99938111	Nbl1	10.7527413	25.3413283	16.2220308	14.4769801	26.6369875	4.7042181	5.77358333	11.4131377
Tnip2	3.10322617	0.36946093	0.41554927	0.88909056	0.37395441	NA	Tnip2	2.68818531	2.71514231	2.22655324	2.06814001	3.32962344	4.7042181	4.81131944	2.28262753
Tbc1d7	14.6919646	-0.3180278	0.35778853	-0.8888708	0.37407253	0.99938111	Tbc1d7	20.8334362	14.480759	15.9039517	16.5451201	9.98887032	5.88027262	20.2075417	13.6957652
Neb	0.96420488	0.28062469	0.31578638	0.88865356	0.37418929	NA	Neb	0	0.45252372	0	2.06814001	1.66481172	3.52816357	0	0
Ankrd39	4.8609528	0.36764306	0.41373805	0.88858895	0.37422403	0.99938111	Ankrd39	4.03227797	1.81009488	3.49886938	6.20442003	1.66481172	3.52816357	6.73584722	11.4131377
Farsa	10.3022656	0.32127879	0.3616164	0.88845194	0.3742977	0.99938111	Farsa	10.0806949	9.05047438	9.86045007	4.13628002	13.3184938	9.40843619	10.5849028	15.9783927
Mapk9	276.332386	0.23063087	0.25967989	0.88813525	0.374468	0.99938111	Mapk9	296.372431	236.217381	273.866049	200.609581	214.760712	352.816357	403.188569	232.828008
Meaf6	61.8672635	-0.289426	0.32595626	-0.8879289	0.37457902	0.99938111	Meaf6	86.02193	75.5714611	50.2564875	66.1804803	24.9721758	39.9858538	78.9056389	73.044081
Runx2	7.10018994	0.35698715	0.40207443	0.88786336	0.37461426	0.99938111	Runx2	5.37637063	3.62018975	4.13502745	10.3407001	6.65924688	5.88027262	4.81131944	15.9783927
Amot	15.4730624	-0.3262029	0.3674692	-0.8877014	0.37470138	0.99938111	Amot	15.4570656	15.8383302	15.9039517	24.8176801	26.6369875	10.5844907	7.69811111	6.84788259
Gm11974	0.48113194	0.18670151	0.21037091	0.88748729	0.37481661	NA	Gm11974	0	0	0	0	0	0	3.84905555	0
Pa2g4	26.5708917	0.28302548	0.31893126	0.88741846	0.37485365	0.99938111	Pa2g4	36.2905017	18.5534725	23.2197695	14.4769801	33.2962344	24.697145	34.6415	27.3915304
Tbc1d5	29.4669837	0.2560176	0.28854404	0.88727392	0.37493145	0.99938111	Tbc1d5	33.6023164	23.9837571	28.6271131	18.6132601	36.6258578	36.4576902	32.7169722	25.1089028
Hat1	9.54063907	0.34279261	0.3863509	0.88725719	0.37494045	0.99938111	Hat1	10.7527413	7.2403795	8.2700549	6.20442003	8.3240586	18.8168724	14.4339583	2.28262753
Qrsl1	10.683501	0.32053695	0.36136649	0.88701348	0.37507164	0.99938111	Qrsl1	9.4086486	5.88280835	11.1327662	10.3407001	9.98887032	10.5844907	14.4339583	13.6957652
Klhl34	37.3642801	0.30880192	0.34816219	0.88694844	0.37510666	0.99938111	Klhl34	33.6023164	36.2018975	26.7186389	31.0221002	78.2461509	28.2253086	19.2452778	45.6525506
Rgag1	3.48059367	0.36881031	0.41582406	0.88693838	0.37511208	NA	Rgag1	1.34409266	3.62018975	2.22655324	4.13628002	3.32962344	7.05632714	3.84905555	2.28262753
Mar-11	3.37465689	-0.3692311	0.41635451	-0.8868189	0.37517641	NA	Mar-11	2.68818531	2.71514231	4.13502745	8.27256004	1.66481172	2.35210905	2.88679167	2.28262753
Cnbp	138.992944	0.25497985	0.28756566	0.88668395	0.37524908	0.99938111	Cnbp	168.683628	105.89055	135.819748	88.9300204	114.872009	162.295524	221.320694	114.131377
Skap2	3.47912947	-0.3688828	0.41609799	-0.8865286	0.37533276	NA	Skap2	6.04841696	1.81009488	4.45310648	6.20442003	3.32962344	1.17605452	4.81131944	0
Enpp2	55.4835869	-0.340302	0.38397362	-0.8862641	0.37547525	0.99938111	Enpp2	58.4680306	104.532979	80.7920748	14.4769801	18.3129289	62.3308898	61.5848889	43.3699231
Vps9d1	33.5179939	0.30550354	0.34476957	0.88610935	0.3755586	0.99938111	Vps9d1	38.3066407	18.1009488	22.5836114	37.2265202	58.2684102	25.8731995	22.1320694	45.6525506
Numa1	44.390078	0.3021757	0.34111931	0.88583581	0.37570601	0.99938111	Numa1	28.8979921	29.8665654	31.8079035	66.1804803	79.9109626	51.7463991	27.9056528	38.804668
Inpp5a	45.1177091	-0.2490877	0.28119359	-0.8858229	0.37571294	0.99938111	Inpp5a	53.7637063	50.2301328	51.2107246	43.4309402	21.6425524	48.2182355	49.0754583	43.3699231
D830030K20Rik	2.01633587	-0.3551044	0.40087738	-0.8858181	0.37571557	NA	D830030K20Ri	1.34409266	2.71514231	1.59039517	6.20442003	0	2.35210905	1.92452778	0
Zfp526	9.29820449	-0.3284887	0.37091219	-0.8856239	0.37582024	0.99938111	Zfp526	13.4409266	9.95552182	12.0870033	6.20442003	9.98887032	5.88027262	7.69811111	9.13051012
Fsd1l	20.829268	0.29756783	0.33613272	0.88526885	0.37601164	0.99938111	Fsd1l	21.5054825	16.2908539	22.5836114	12.4088401	23.3073641	23.5210905	35.6037639	11.4131377
Exd1	0.40042215	-0.2477807	0.27989538	-0.8852619	0.37601538	NA	Exd1	1.34409266	0.90504744	0.9542371	0	0	0	0	0
LOC101056043	15.5331766	-0.3220337	0.36379391	-0.8852091	0.37604387	0.99938111	LOC10105604	16.8011582	18.1009488	10.1785291	26.8858201	16.6481172	12.9365998	6.73584722	15.9783927
Gm10033	2.34133825	-0.3585295	0.40505598	-0.8851356	0.37608348	NA	Gm10033	5.37637063	1.81009488	4.77118552	0	0	3.52816357	0.96226389	2.28262753
Rnf217	21.8496156	0.26297976	0.29711241	0.88511873	0.3760926	0.99938111	Rnf217	17.4732045	18.5534725	18.766663	24.8176801	26.6369875	25.8731995	22.1320694	20.5436478
Rasl11a	2.75194366	-0.3652128	0.41268386	-0.8849699	0.37617289	NA	Rasl11a	4.7043243	3.62018975	1.59039517	4.13628002	3.32962344	2.35210905	0	2.28262753
B230118H07Rik	13.4676857	-0.3302267	0.37320295	-0.8848449	0.3762403	0.99938111	B230118H07R	23.5216215	16.7433776	15.9039517	6.20442003	6.65924688	16.4647633	15.3962222	6.84788259
Mto1	12.7839582	-0.3180045	0.35939543	-0.8848317	0.37624741	0.99938111	Mto1	20.1613899	16.7433776	13.0412404	8.27256004	6.65924688	10.5844907	15.3962222	11.4131377
Epb4.1l5	7.21258694	-0.3496001	0.39515918	-0.884707	0.37631467	0.99938111	Epb4.1l5	9.4086486	6.33533207	7.95197586	10.3407001	6.65924688	8.23238167	1.92452778	6.84788259
Intu	7.63737616	-0.3639334	0.41139432	-0.884634	0.37635405	0.99938111	Intu	11.4247876	10.8605693	8.90621297	6.20442003	0	15.2887088	3.84905555	4.56525506
Thrap3	52.8282506	0.23868785	0.26988551	0.88440411	0.3764781	0.99938111	Thrap3	48.3873356	35.2968501	44.5310648	66.1804803	64.9276571	62.3308898	46.1886666	54.7830607
Mpv17	33.2659354	-0.2543867	0.28764695	-0.8843712	0.37649584	0.99938111	Mpv17	38.3066407	38.4645161	34.0344567	37.2265202	28.3017992	22.345036	42.3396111	25.1089028
Taf1a	3.63370604	0.3678935	0.41600837	0.88434157	0.37651185	NA	Taf1a	2.01613899	3.16766603	2.54463228	4.13628002	1.66481172	9.40843619	3.84905555	2.28262753
Mrpl42	10.3594729	-0.3532471	0.39945947	-0.8843129	0.37652734	0.99938111	Mrpl42	16.8011582	12.2181404	14.6316356	6.20442003	3.32962344	5.88027262	19.2452778	4.56525506
Icosl	0.8386414	-0.3025352	0.34213298	-0.884262	0.37655479	NA	Icosl	1.34409266	1.81009488	1.27231614	0	0	0	0	2.28262753
I7Rn6	17.7183058	0.31867265	0.36040835	0.88419885	0.37658888	0.99938111	I7Rn6	10.7527413	15.3858064	21.9474534	12.4088401	19.9777406	12.9365998	34.6415	13.6957652
Egfem1	4.06868498	0.36757625	0.41577421	0.88407661	0.37665486	NA	Egfem1	2.01613899	1.81009488	3.81694841	6.20442003	1.66481172	7.05632714	7.69811111	2.28262753
4933406I18Rik	0.54499278	0.26970144	0.30511243	0.88394115	0.37672798	NA	4933406I18Ri	0	0.45252372	0.31807903	0	1.66481172	0	1.92452778	0
Pigt	90.2320402	-0.3035829	0.34354254	-0.8836835	0.3768671	0.99938111	Pigt	78.6294204	128.064212	80.4739957	119.952121	89.8998329	65.8590533	28.8679167	130.109769
4932438A13Rik	100.236953	0.35907888	0.40641639	0.88352461	0.37695289	0.99938111	4932438A13Ri	86.6939764	116.751119	95.4237104	20.6814001	64.9276571	292.837576	97.1886527	27.3915304
Hnrnpr	36.3979843	0.28713058	0.32499316	0.88349731	0.37696764	0.99938111	Hnrnpr	36.9625481	27.1514231	31.1717454	35.1583802	24.9721758	42.3379629	68.3207361	25.1089028
Npas1	9.74219788	0.35529402	0.40216459	0.88345428	0.37699087	0.99938111	Npas1	7.39250961	3.16766603	4.45310648	18.6132601	11.653682	7.05632714	9.62263889	15.9783927
Enpp4	15.2755795	-0.347773	0.39372972	-0.8832784	0.37708586	0.99938111	Enpp4	11.4247876	25.3413283	21.6293743	14.4769801	4.99443516	28.2253086	11.5471667	4.56525506
Sh3rf1	72.4683316	0.21779582	0.24661296	0.8831483	0.37715615	0.99938111	Sh3rf1	77.2853278	58.8280835	52.4830407	78.5893204	84.9053977	65.8590533	75.0565833	86.7398462
B930018H19Rik	0.62588077	0.27389795	0.31016077	0.88308379	0.377191	NA	B930018H19R	0.67204633	0	0.31807903	0	1.66481172	2.35210905	0	0
Nrbp1	54.9363386	0.25143284	0.28473169	0.88305184	0.37720826	0.99938111	Nrbp1	47.7152893	41.1796584	48.3480132	59.9760603	63.2628454	36.4576902	55.8113055	86.7398462
Cys1	5.2569879	-0.3676306	0.41634647	-0.882992	0.3772406	0.99938111	Cys1	4.03227797	5.43028463	4.13502745	12.4088401	4.99443516	0	1.92452778	9.13051012
Pla2g4e	4.03681669	0.36447757	0.4128498	0.88283336	0.37732632	NA	Pla2g4e	4.7043243	2.71514231	4.13502745	0	6.65924688	4.7042181	4.81131944	4.56525506
Anxa2	5.20768336	0.36713307	0.4159308	0.88267826	0.37741013	0.99938111	Anxa2	1.34409266	2.26261859	3.18079035	10.3407001	4.99443516	2.35210905	5.77358333	11.4131377
Gpr146	7.72684438	-0.3661039	0.41487451	-0.8824449	0.37753627	0.99938111	Gpr146	8.06455594	14.0282353	11.1327662	4.13628002	16.6481172	5.88027262	1.92452778	0
Cd82	4.91447855	-0.3637632	0.41225777	-0.8823684	0.3775776	0.99938111	Cd82	4.03227797	7.69290322	3.81694841	8.27256004	6.65924688	2.35210905	1.92452778	4.56525506
Slc26a8	1.59114037	0.33882773	0.38401875	0.88232079	0.37760336	NA	Slc26a8	0.67204633	0.45252372	0.9542371	2.06814001	1.66481172	2.35210905	0	4.56525506
Stk11	35.1792694	0.29422335	0.33347449	0.88229642	0.37761653	0.99938111	Stk11	31.5861774	31.6766603	23.2197695	35.1583802	44.9499164	18.8168724	29.8301805	66.1961984
Eif2s3x	44.6615124	0.25316416	0.28694255	0.8822817	0.37762449	0.99938111	Eif2s3x	33.6023164	30.7716129	46.439539	51.7035003	39.9554813	38.8097993	63.5094166	52.5004332
Chrm3	17.5602769	-0.3376645	0.38283594	-0.8820084	0.37777226	0.99938111	Chrm3	18.1452509	33.9392789	22.5836114	6.20442003	14.9833055	25.8731995	9.62263889	9.13051012
Ccdc88a	48.3852084	0.34507009	0.39125357	0.88196023	0.37779831	0.99938111	Ccdc88a	42.3389187	53.3977988	45.1672229	18.6132601	23.3073641	109.373071	78.9056389	15.9783927
Ubfd1	103.93864	0.28634726	0.3247571	0.88172747	0.3779242	0.99938111	Ubfd1	61.1562159	64.7108918	71.2497037	171.655621	106.54795	101.140689	95.264125	159.783927
Sos2	42.784654	-0.295154	0.33479076	-0.8816073	0.37798919	0.99938111	Sos2	53.0916599	54.3028463	57.2542262	28.9539601	21.6425524	56.4506172	50.0377222	20.5436478
Dip2a	71.6959401	0.27775326	0.31518998	0.88122491	0.3781961	0.99938111	Dip2a	51.7475673	44.3473245	54.7095939	105.475141	109.877574	72.9153805	50.0377222	84.4572186
Vps51	41.3602075	0.35124781	0.39871699	0.88094518	0.37834749	0.99938111	Vps51	17.4732045	25.3413283	22.9016905	70.3167603	98.2238915	16.4647633	23.0943333	57.0656883
Spns2	36.5364868	0.28041515	0.31834853	0.88084322	0.37840268	0.99938111	Spns2	21.5054825	38.9170398	30.2175083	37.2265202	54.9387868	32.9295267	24.0565972	52.5004332
Psen1	30.3934056	-0.3012456	0.34201076	-0.8808073	0.37842211	0.99938111	Psen1	24.8657142	49.7776091	38.4875632	24.8176801	14.9833055	41.1619083	30.7924444	18.2610202
Trim34a	0.78370867	-0.2965026	0.33662941	-0.8807984	0.37842694	NA	Trim34a	2.01613899	0.90504744	0.31807903	2.06814001	0	0	0.96226389	0
Ssfa2	15.1615768	-0.3078616	0.34956274	-0.880705	0.37847753	0.99938111	Ssfa2	13.4409266	25.3413283	18.448584	10.3407001	16.6481172	14.1126543	11.5471667	11.4131377
Fundc1	37.8737914	0.24805247	0.28166689	0.88065896	0.37850243	0.99938111	Fundc1	32.2582238	34.3918026	42.6225906	26.8858201	43.2851047	39.9858538	53.8867778	29.6741579
Rcan3	69.9574716	0.25691106	0.29172657	0.88065704	0.37850347	0.99938111	Rcan3	49.059382	51.1351802	55.663831	97.2025805	76.5813391	77.6195986	51.96225	100.435611
Ppp2r2a	69.8076177	0.21356887	0.24258432	0.88039025	0.37864793	0.99938111	Ppp2r2a	72.5810035	55.2078937	68.7050715	57.9079203	71.586904	59.9787807	78.9056389	93.5877288
P2rx4	3.59003271	-0.3664912	0.41630141	-0.8803506	0.37866942	NA	P2rx4	2.68818531	5.43028463	2.22655324	8.27256004	3.32962344	3.52816357	0.96226389	2.28262753

GeneID	Base mean	log2(FC)	StdErr	Wald-Stats	P-value	P-adj	GeneID	Normalised expression for Chow#1	Normalised expression for Chow#2	Normalised expression for Chow#3	Normalised expression for Chow#4	Normalised expression for HFD#1	Normalised expression for HFD#2	Normalised expression for HFD#3	Normalised expression for HFD#4
Cyp1b1	0.79053956	-0.2934627	0.33337235	-0.8802852	0.37870485	NA	Cyp1b1	0.67204633	2.71514231	1.27231614	0	1.66481172	0	0	0
Galnt4	0.48162672	-0.220231	0.25021319	-0.8801733	0.37876544	NA	Galnt4	0	2.26261859	1.59039517	0	0	0	0	0
Mospd3	56.9070466	-0.3659358	0.41583579	-0.8800008	0.37885888	0.99938111	Mospd3	29.5700384	30.3190892	23.8559276	198.541441	44.9499164	14.1126543	13.4716944	100.435611
Otub2	19.2286733	0.28642534	0.32552175	0.87989615	0.37891557	0.99938111	Otub2	16.8011582	18.5534725	20.3570582	10.3407001	14.9833055	18.8168724	28.8679167	25.1089028
Tmem234	17.6326206	-0.3009842	0.34207289	-0.8798833	0.37892255	0.99938111	Tmem234	21.5054825	21.2686148	15.2677937	20.6814001	8.3240586	14.1126543	12.5094306	27.3915304
Invs	5.86671479	-0.3512368	0.39923837	-0.8797671	0.37898548	0.99938111	Invs	4.7043243	7.2403795	6.67965972	10.3407001	4.99443516	5.88027262	4.81131944	2.28262753
Grpel1	23.8566377	0.28135788	0.31981377	0.87975535	0.37899185	0.99938111	Grpel1	22.8495752	18.5534725	23.2197695	20.6814001	16.6481172	24.697145	41.3773472	22.8262753
Lta4h	24.4246846	0.3054979	0.34733703	0.87954313	0.37910686	0.99938111	Lta4h	22.8495752	15.3858064	17.1762679	28.9539601	31.6314227	23.5210905	12.5094306	43.3699231
Fam72a	0.39593612	-0.242431	0.2757389	-0.8792051	0.37929008	NA	Fam72a	0.67204633	0.90504744	1.59039517	0	0	0	0	0
Gbp3	0.39593612	-0.242431	0.2757389	-0.8792051	0.37929008	NA	Gbp3	0.67204633	0.90504744	1.59039517	0	0	0	0	0
Sike1	21.6874722	0.27641347	0.31440314	0.87916893	0.37930969	0.99938111	Sike1	16.8011582	24.4362808	21.6293743	12.4088401	19.9777406	22.345036	30.7924444	25.1089028
Per3	22.5886579	-0.311414	0.3542322	-0.8791238	0.37933414	0.99938111	Per3	18.1452509	22.1736622	20.9932163	43.4309402	33.2962344	16.4647633	12.5094306	13.6957652
Axin2	19.7778051	0.31339587	0.35651714	0.87904853	0.37937496	0.99938111	Axin2	19.4893435	16.7433776	13.9954775	16.5451201	38.2906696	9.40843619	16.3584861	27.3915304
Skiv2l	41.8737048	0.34453282	0.39201134	0.87888484	0.37946372	0.99938111	Skiv2l	26.8818531	21.2686148	20.6751372	70.3167603	66.5924688	29.4013631	15.3962222	84.4572186
Snx1	39.4647889	-0.2654141	0.30200382	-0.8788437	0.37948605	0.99938111	Snx1	52.4196136	42.9897533	37.5333261	43.4309402	16.6481172	39.9858538	46.1886666	36.5220405
Vip	5.02229545	-0.3605022	0.41027747	-0.878679	0.37957534	0.99938111	Vip	2.68818531	16.7433776	6.67965972	0	1.66481172	4.7042181	7.69811111	0
1700034G24Rik	0.66025159	0.27315084	0.31100774	0.87827667	0.37979359	NA	1700034G24R	0.67204633	0	0.31807903	0	3.32962344	0	0.96226389	0
Fam193a	52.0391722	-0.2544733	0.28976797	-0.8781966	0.37983701	0.99938111	Fam193a	58.4680306	44.7998482	44.5310648	84.7937404	56.6035985	51.7463991	36.5660278	38.804668
Chd7	5.39900607	-0.3578667	0.40752564	-0.8781452	0.37986493	0.99938111	Chd7	4.7043243	7.69290322	4.13502745	10.3407001	6.65924688	3.52816357	3.84905555	2.28262753
Lingo3	22.0748164	-0.2745598	0.31267038	-0.8781125	0.37988268	0.99938111	Lingo3	20.1613899	26.6988994	28.9451921	22.7495401	19.9777406	27.0492541	17.32075	13.6957652
Trim46	57.0981227	0.32083977	0.3653807	0.8780972	0.37989097	0.99938111	Trim46	43.010965	39.8220873	44.5310648	66.1804803	93.2294563	34.1055812	24.0565972	111.848749
Clpx	15.4399016	0.30004433	0.34175546	0.87795035	0.37997066	0.99938111	Clpx	15.4570656	13.1231878	16.2220308	8.27256004	9.98887032	22.345036	22.1320694	15.9783927
Plin5	0.68086986	0.2239714	0.25510954	0.87794209	0.37997514	NA	Plin5	0	0.45252372	0	0	4.99443516	0	0	0
Nudt16l1	31.2104536	-0.3473414	0.39564184	-0.8779188	0.37998779	0.99938111	Nudt16l1	19.4893435	19.9110436	22.5836114	86.8618804	31.6314227	11.7605452	16.3584861	41.0872956
1700037H04Rik	133.888919	-0.360759	0.41099152	-0.8777773	0.3800646	0.99938111	1700037H04Ri	105.511274	75.1189373	72.2039408	397.082882	118.201632	19.9929269	61.5848889	221.414871
Fam178a	31.7417929	0.28314813	0.32270191	0.87742938	0.38025346	0.99938111	Fam178a	23.5216215	33.0342315	33.0802196	22.7495401	21.6425524	47.042181	50.0377222	22.8262753
Plxdc1	8.03488301	-0.3628471	0.41367863	-0.8771231	0.3804198	0.99938111	Plxdc1	4.03227797	14.9332827	8.2700549	12.4088401	4.99443516	14.1126543	0.96226389	4.56525506
Tctex1d2	5.87923385	0.36501056	0.41617175	0.87706713	0.38045018	0.99938111	Tctex1d2	1.34409266	1.81009488	2.54463228	14.4769801	8.3240586	4.7042181	11.5471667	2.28262753
Itpr2	7.97871422	-0.3454828	0.39401439	-0.8768278	0.38058018	0.99938111	Itpr2	9.4086486	14.0282353	10.1785291	2.06814001	8.3240586	5.88027262	4.81131944	9.13051012
Asb6	18.8917105	0.34421454	0.39259084	0.87677681	0.38060788	0.99938111	Asb6	14.7850192	8.59795066	7.63389683	33.0902402	33.2962344	10.5844907	13.4716944	29.6741579
Dlat	97.7812557	0.22233585	0.25358703	0.87676349	0.38061512	0.99938111	Dlat	92.070347	85.5269829	107.828793	70.3167603	69.9220923	117.605452	120.282986	118.696632
Nufip1	10.4872912	-0.3499069	0.39912005	-0.8766958	0.38065186	0.99938111	Nufip1	21.5054825	9.05047438	12.0870033	8.27256004	3.32962344	12.9365998	14.4339583	2.28262753
Cdk13	59.1497658	0.27654232	0.31546551	0.87661665	0.38069489	0.99938111	Cdk13	65.8605402	53.8503226	56.6180681	33.0902402	48.2795399	95.2604165	83.7169583	36.5220405
Nucb1	36.8669356	-0.2926675	0.33391635	-0.8764696	0.38077481	0.99938111	Nucb1	29.5700384	64.7108918	39.1237212	31.0221002	33.2962344	44.6900719	18.2830139	34.239413
Comt	59.2768462	-0.2601841	0.29694925	-0.8761905	0.38092647	0.99938111	Comt	62.5003085	81.0017457	57.2542262	57.9079203	49.9443516	49.39429	31.7547083	84.4572186
Klf2	2.9512601	-0.3612287	0.41228162	-0.8761697	0.3809378	NA	Klf2	2.01613899	7.2403795	1.90847421	4.13628002	4.99443516	2.35210905	0.96226389	0
Ggt5	0.72062974	0.24884545	0.28404882	0.87606578	0.38099428	NA	Ggt5	0	0.45252372	0.31807903	0	4.99443516	0	0	0
Kri1	11.309099	0.33310458	0.38026502	0.87598007	0.38104087	0.99938111	Kri1	10.0806949	4.07271347	9.54237104	16.5451201	9.98887032	17.6408179	13.4716944	9.13051012
Cep41	5.43059906	-0.3574362	0.40808039	-0.8758966	0.38108627	0.99938111	Cep41	7.39250961	9.95552182	5.72542262	2.06814001	3.32962344	2.35210905	5.77358333	6.84788259
Vps26b	102.084799	0.20695953	0.23639512	0.87548138	0.38131204	0.99938111	Vps26b	103.495135	76.0239848	104.966081	90.9981605	126.525691	83.4998712	119.320722	111.848749
Cdh2	83.6029956	-0.3432187	0.39210536	-0.8753227	0.38139837	0.99938111	Cdh2	75.9412351	174.674156	113.236136	28.9539601	39.9554813	148.18287	49.0754583	38.804668
Rad23a	52.4669379	-0.2508131	0.28661036	-0.8751011	0.38151887	0.99938111	Rad23a	53.7637063	53.8503226	63.2977279	62.0442003	56.6035985	34.1055812	66.3962083	29.6741579
Tunar	54.2352037	-0.3381174	0.38639546	-0.8750553	0.38154383	0.99938111	Tunar	43.010965	40.7271347	38.1694841	132.360961	51.6091633	31.7534722	16.3584861	79.8919636
A630089N07Rik	1.61320565	0.32426886	0.37062916	0.87491458	0.38162038	NA	A630089N07R	1.34409266	2.26261859	0.31807903	0	0	7.05632714	1.92452778	0
Nt5dc3	198.02671	0.32272248	0.36886173	0.8749145	0.38162043	0.99938111	Nt5dc3	118.280154	111.320835	109.419188	335.038682	324.638285	115.253343	134.716944	335.546247
Naif1	6.08407715	0.3634568	0.41558659	0.87456335	0.38181154	0.99938111	Naif1	8.06455594	4.97776091	2.54463228	2.06814001	16.6481172	2.35210905	2.88679167	9.13051012
Col9a3	1.58122213	0.34182629	0.39099379	0.87424991	0.38198217	NA	Col9a3	0.67204633	0.90504744	0.63615807	2.06814001	1.66481172	1.17605452	0.96226389	4.56525506
Ubxn7	67.1317081	0.26264204	0.30051123	0.87398411	0.38212691	0.99938111	Ubxn7	64.5164475	67.8785578	68.0689134	39.2946602	58.2684102	91.7322529	103.9245	43.3699231
Sypl	16.1302795	-0.3090821	0.35366487	-0.8739405	0.38215066	0.99938111	Sypl	14.7850192	21.7211385	17.8124259	18.6132601	16.6481172	19.9929269	5.77358333	13.6957652
Gm6402	10.5434155	-0.3577976	0.40941714	-0.8739193	0.38216219	0.99938111	Gm6402	12.7688802	12.2181404	6.04350166	20.6814001	3.32962344	1.17605452	14.4339583	13.6957652
Fundc2	49.7812074	0.28361256	0.32453054	0.8739164	0.38216378	0.99938111	Fundc2	43.010965	32.5817078	50.5745665	51.7035003	29.966611	61.1548353	90.4528055	38.804668
Nanos1	36.0249304	0.26538665	0.30370058	0.87384307	0.38220372	0.99938111	Nanos1	24.8657142	33.0342315	40.3960374	31.0221002	26.6369875	43.5140174	56.7735694	31.9567854
Podn	0.64615562	-0.2837049	0.32474481	-0.873624	0.38232305	NA	Podn	0.67204633	2.26261859	1.27231614	0	0	0	0.96226389	0
Lrrc71	18.7117747	0.33289037	0.38105889	0.87359298	0.38233995	0.99938111	Lrrc71	12.0968339	11.7656167	11.1327662	28.9539601	38.2906696	11.7605452	10.5849028	25.1089028
Pard6g	4.41748595	-0.3562438	0.40814858	-0.8728287	0.38275646	NA	Pard6g	2.01613899	4.07271347	6.04350166	12.4088401	0	1.17605452	9.62263889	0
Galm	1.30935183	-0.3200681	0.36684627	-0.8724858	0.38294344	NA	Galm	4.7043243	1.35757116	0.9542371	0	0	1.17605452	0	2.28262753
Wdr91	17.0590805	0.33338342	0.38222116	0.8722265	0.38308482	0.99938111	Wdr91	16.8011582	11.7656167	10.8146872	16.5451201	16.6481172	7.05632714	13.4716944	43.3699231
Coa6	11.1314382	0.33092521	0.37942781	0.8721691	0.38311613	0.99938111	Coa6	6.04841696	9.5029981	8.90621297	14.4769801	11.653682	5.88027262	21.1698055	11.4131377
Ano10	7.72356977	-0.3463488	0.39717254	-0.872036	0.38318872	0.99938111	Ano10	6.04841696	14.0282353	8.2700549	8.27256004	4.99443516	11.7605452	3.84905555	4.56525506
Serpinb1b	0.56710034	-0.238228	0.27319378	-0.8720112	0.38320226	NA	Serpinb1b	2.01613899	0.45252372	0	2.06814001	0	0	0	0
Fbxl5	46.8737175	-0.2710649	0.31085112	-0.8720087	0.38320361	0.99938111	Fbxl5	53.7637063	60.6381783	47.7118552	47.5672202	19.9777406	38.8097993	65.4339444	41.0872956
Vangl2	5.49762047	0.35681955	0.40924117	0.87190533	0.38326003	0.99938111	Vangl2	2.68818531	4.52523719	5.08926455	4.13628002	8.3240586	5.88027262	1.92452778	11.4131377
Kctd10	49.3919659	0.31631866	0.36288049	0.87168825	0.38337847	0.99938111	Kctd10	36.2905017	28.961518	32.1259825	72.3849004	86.5702095	32.9295267	25.981125	79.8919636
1700001O22Rik	1.3227393	-0.3238369	0.3715319	-0.8716262	0.38341233	NA	1700001O22R	2.68818531	2.26261859	0.31807903	2.06814001	0	0	0.96226389	2.28262753
Cd68	3.48568316	-0.3627725	0.41631587	-0.8713876	0.38354255	NA	Cd68	4.03227797	6.78785578	5.72542262	0	4.99443516	1.17605452	2.88679167	2.28262753
Rbbp9	19.5844944	-0.3136805	0.35998508	-0.8713709	0.38355165	0.99938111	Rbbp9	24.1936678	30.7716129	22.9016905	12.4088401	11.653682	19.9929269	27.9056528	6.84788259
Stk17b	3.02022361	-0.351276	0.40325834	-0.8710943	0.38370269	NA	Stk17b	6.04841696	5.43028463	4.77118552	0	0	1.17605452	6.73584722	0
Armc9	18.1791567	0.30532591	0.35064894	0.87074529	0.38389324	0.99938111	Armc9	20.1613899	14.480759	18.766663	6.20442003	26.6369875	14.1126543	15.3962222	29.6741579
Ccdc112	6.12319797	0.36231853	0.41614411	0.8706564	0.38394179	0.99938111	Ccdc112	4.7043243	4.97776091	5.40734359	4.13628002	0	18.8168724	8.660375	2.28262753
Cckar	2.87183871	-0.3564103	0.4094123	-0.8705413	0.38400464	NA	Cckar	2.68818531	1.35757116	3.18079035	8.27256004	1.66481172	3.52816357	0	2.28262753
Gm5901	2.97066679	0.36219495	0.41610756	0.87043589	0.38406224	NA	Gm5901	2.68818531	1.81009488	2.54463228	2.06814001	4.99443516	3.52816357	3.84905555	2.28262753
Gusb	5.33892371	-0.360213	0.41384667	-0.870402	0.38408076	0.99938111	Gusb	3.36023164	10.8605693	6.04350166	6.20442003	1.66481172	9.40843619	2.88679167	2.28262753
Dusp15	8.60032497	0.35029789	0.40258007	0.8701322	0.38422816	0.99938111	Dusp15	4.7043243	8.59795066	7.63389683	6.20442003	13.3184938	1.17605452	13.4716944	13.6957652
3110007F17Rik	0.96283669	-0.3153887	0.36247327	-0.8701019	0.38424474	NA	3110007F17Ri	1.34409266	1.35757116	2.86271131	0	0	1.17605452	0.96226389	0
Hyi	5.03335227	-0.3528788	0.40558606	-0.8700466	0.38427496	0.99938111	Hyi	4.03227797	0.45252372	2.22655324	20.6814001	4.99443516	2.35210905	0.96226389	4.56525506
Ppapdc2	33.5728201	-0.2625054	0.30173645	-0.8699824	0.38431001	0.99938111	Ppapdc2	30.2420848	47.5149905	42.3045116	26.8858201	21.6425524	38.8097993	26.9433889	34.239413
Yeats4	22.6915255	0.27949366	0.32128477	0.86992502	0.38434138	0.99938111	Yeats4	18.1452509	16.2908539	20.9932163	26.8858201	14.9833055	23.5210905	35.6037639	25.1089028
B930059L03Rik	0.49342991	0.21166243	0.24335539	0.86976678	0.38442787	NA	B930059L03Ri	0	0	0	0	1.66481172	0	0	2.28262753
Clic3	0.49342991	0.21166243	0.24335539	0.86976678	0.38442787	NA	Clic3	0	0	0	0	1.66481172	0	0	2.28262753
E330033B04Rik	0.49342991	0.21166243	0.24335539	0.86976678	0.38442787	NA	E330033B04Ri	0	0	0	0	1.66481172	0	0	2.28262753
Fam129c	0.49342991	0.21166243	0.24335539	0.86976678	0.38442787	NA	Fam129c	0	0	0	0	1.66481172	0	0	2.28262753
Gm14378	0.49342991	0.21166243	0.24335539	0.86976678	0.38442787	NA	Gm14378	0	0	0	0	1.66481172	0	0	2.28262753
Vax2os	0.49342991	0.21166243	0.24335539	0.86976678	0.38442787	NA	Vax2os	0	0	0	0	1.66481172	0	0	2.28262753
Fbxl19	63.3995311	0.34934195	0.40165484	0.86975659	0.38443344	0.99938111	Fbxl19	47.7152893	37.106945	35.9429309	84.7937404	123.196067	16.4647633	25.0188611	136.957652
Coro2b	190.952247	0.22660088	0.26055327	0.86969116	0.38446921	0.99938111	Coro2b	182.796601	151.142922	164.76494	196.473301	251.38657	139.950488	162.622597	278.480559
Coq9	29.179027	0.26982686	0.31027901	0.86962653	0.38450454	0.99938111	Coq9	21.5054825	20.3635674	24.1740066	39.2946602	38.2906696	21.1689814	29.8301805	38.804668
Mrps5	10.5848617	0.32788059	0.37707959	0.86952622	0.38455937	0.99938111	Mrps5	6.72046328	8.59795066	11.1327662	8.27256004	14.9833055	16.4647633	4.81131944	13.6957652
Aagab	28.6093547	-0.2888844	0.33226129	-0.8694495	0.38460129	0.99938111	Aagab	41.6668724	29.4140417	20.0389792	39.2946602	16.6481172	18.8168724	35.6037639	27.3915304
1810043G02Rik	16.8795757	-0.2896068	0.3331736	-0.8692368	0.38471762	0.99938111	1810043G02R	14.7850192	16.7433776	20.0389792	24.8176801	9.98887032	11.7605452	16.3584861	20.5436478
B3gntl1	3.52306655	-0.3557297	0.40926079	-0.8692004	0.38473752	NA	B3gntl1	3.36023164	3.16766603	0.9542371	12.4088401	1.66481172	4.7042181	1.92452778	0
1700001G11Rik	0.66574917	0.26848291	0.30888963	0.8691872	0.38474475	NA	1700001G11R	0	0.90504744	0	0	0	1.17605452	0.96226389	2.28262753
Sox21	8.24705362	-0.3566389	0.4103213	-0.8691699	0.38475419	0.99938111	Sox21	14.7850192	5.88280835	9.86045007	8.27256004	9.98887032	0	5.77358333	11.4131377
Nme3	11.2902436	0.33349359	0.3837082	0.86913335	0.3847742	0.99938111	Nme3	6.04841696	9.95552182	7.31581779	14.4769801	9.98887032	5.88027262	11.5471667	25.1089028
Morc2b	1.58125716	0.31883916	0.36684886	0.86912947	0.38477632	NA	Morc2b	0.67204633	0.45252372	2.54463228	0	0	7.05632714	1.92452778	0
Actl6a	2.89885026	-0.360079	0.41430019	-0.8691258	0.38477832	NA	Actl6a	4.7043243	3.16766603	4.45310648	2.06814001	6.65924688	1.17605452	0.96226389	0
Upf1	58.1730424	0.30824886	0.35470141	0.8690376	0.38482656	0.99938111	Upf1	39.6507334	37.5594687	43.2587487	80.6574604	109.877574	35.2816357	34.6415	84.4572186
Brap	39.6541685	0.22948167	0.26406864	0.86902281	0.38483466	0.99938111	Brap	45.027104	34.8443264	35.9429309	26.8858201	49.9443516	41.1619083	42.3396111	41.0872956
Mrpl55	13.3867048	-0.309547	0.35624743	-0.8689102	0.38489624	0.99938111	Mrpl55	19.4893435	14.480759	12.7231614	14.4769801	9.98887032	5.88027262	16.3584861	13.6957652
Zfp58	3.91051856	-0.3609747	0.41545564	-0.8688645	0.38492123	NA	Zfp58	7.39250961	3.16766603	5.40734359	4.13628002	1.66481172	4.7042181	4.81131944	0
Rps6ka2	80.8763451	0.20596135	0.23706219	0.86880726	0.38495256	0.99938111	Rps6ka2	76.6132814	76.4765085	71.2497037	72.3849004	116.53682	72.9153805	74.0943194	86.7398462
Tsc2	91.9028431	0.29191259	0.33604666	0.86866683	0.38502939	0.99938111	Tsc2	83.3337447	69.6886527	62.6615698	105.475141	169.810795	79.9717076	43.301875	120.979259
Mef2a	158.38134	0.24795436	0.28548813	0.86852775	0.38510549	0.99938111	Mef2a	170.027721	154.310588	165.719177	78.5893204	119.866444	198.753215	222.282958	157.5013
Crym	15.8829944	0.34660225	0.39924592	0.86814224	0.38531647	0.99938111	Crym	18.8172972	12.6706641	18.448584	0	29.966611	9.40843619	24.0565972	13.6957652
1700034H15Rik	3.17165433	0.35469799	0.40871616	0.86783451	0.38548493	NA	1700034H15Ri	0	2.26261859	3.81694841	2.06814001	4.99443516	1.17605452	1.92452778	9.13051012
Ankrd11	146.458832	0.21999158	0.25359407	0.86749495	0.38567088	0.99938111	Ankrd11	133.065173	164.26611	128.185851	109.611421	144.83862	225.802469	128.943361	136.957652
Slc3a1	38.5330925	0.27376864	0.31561154	0.86742279	0.3857104	0.99938111	Slc3a1	29.5700384	23.5312334	31.4898244	53.7716403	56.6035985	29.4013631	33.6792361	50.2178057
Mast4	46.218855	-0.2540321	0.29296479	-0.867108	0.38588286	0.99938111	Mast4	36.9625481	54.75537	38.8056422	76.5211804	48.2795399	38.8097993	41.3773472	34.239413
Serpinb6a	10.8805836	-0.3433911	0.39602166	-0.8671017	0.38588629	0.99938111	Serpinb6a	14.1129729	6.78785578	11.4508452	20.6814001	4.99443516	3.52816357	16.3584861	9.13051012
Omg	83.1450012	-0.3608217	0.41636883	-0.8665916	0.38616583	0.99938111	Omg	89.3821617	179.651916	136.773985	8.27256004	23.3073641	159.943415	58.6980972	9.13051012
Thap1	8.22826424	-0.3270634	0.37742438	-0.8665666	0.38617952	0.99938111	Thap1	11.4247876	9.5029981	9.86045007	6.20442003	6.65924688	8.23238167	4.81131944	9.13051012
Adamts12	0.48078033	0.21036445	0.24277343	0.86650526	0.38621314	NA	Adamts12	0	0	0.31807903	0	0	3.52816357	0	0
Hsph1	164.767144	0.25192584	0.29074448	0.86648537	0.38622405	0.99938111	Hsph1	176.076138	166.981252	162.856466	84.7937404	143.173808	265.788322	183.792403	134.675024

GeneID	Base mean	log2(FC)	StdErr	Wald-Stats	P-value	P-adj
S1pr2	1.16170138	-0.2970978	0.34299385	-0.8661899	0.38638606	NA
Rusc1	183.434242	0.31824455	0.36747072	0.86604055	0.38646792	0.99938111
Fam206a	22.4919088	-0.3072929	0.35490103	-0.8658553	0.38656951	0.99938111
Patz1	29.5941138	0.29016136	0.33515722	0.86574702	0.38662891	0.99938111
A330021E22Rik	8.76316016	0.35112401	0.40561446	0.86565949	0.38667692	0.99938111
Tmem256	19.891944	-0.3251641	0.37566307	-0.8655739	0.3867239	0.99938111
Tom1l2	283.518966	0.27700155	0.32017716	0.86515088	0.38695598	0.99938111
Gm11992	0.73497673	-0.295658	0.34174168	-0.8651507	0.38695609	NA
Rad9a	17.2452134	-0.3146559	0.36376296	-0.8650025	0.3870374	0.99938111
Mfsd4	43.036681	-0.2715346	0.31392579	-0.8649643	0.3870584	0.99938111
Zfp37	24.3067241	0.32528581	0.37619666	0.86466958	0.38722017	0.99938111
Hcn1	109.544232	0.32429806	0.37509792	0.86456905	0.38727536	0.99938111
Eml5	33.2164637	0.30255547	0.35017498	0.86401226	0.38758115	0.99938111
Tmem132d	13.6085941	0.32545718	0.37674428	0.86386762	0.38766061	0.99938111
Lipa	9.77987482	-0.3476204	0.40244196	-0.8637777	0.38771002	0.99938111
Scn8a	230.630832	0.30161851	0.34927813	0.86354824	0.38783611	0.99938111
Zdhhc22	32.9321074	-0.3044599	0.35261464	-0.8634351	0.38789828	0.99938111
Gm4925	0.67191113	-0.2781591	0.32216262	-0.8634122	0.38791087	NA
Adprh	40.2034834	0.26705255	0.30939001	0.86315829	0.38805044	0.99938111
Mir124a-2	0.48389813	0.26042616	0.30178654	0.86294825	0.38816592	NA
Tagln	0.48389813	0.26042616	0.30178654	0.86294825	0.38816592	NA
Snx7	15.9930943	0.32273816	0.37402057	0.8628888	0.38819861	0.99938111
Oscar	0.83761659	0.27230488	0.31572039	0.86248746	0.38841933	NA
Kat5	37.6497417	-0.2348729	0.27235174	-0.862388	0.38847406	0.99938111
Ak4	30.8243582	-0.2710344	0.31428942	-0.8623721	0.3884828	0.99938111
Wdr41	27.531016	-0.2948341	0.34192926	-0.8622663	0.388541	0.99938111
Usp14	132.815236	0.24808893	0.28772534	0.86224219	0.38855426	0.99938111
Kcnk4	2.77213988	-0.3558935	0.41283498	-0.862072	0.38864791	NA
I830012O16Rik	1.29777848	-0.3176733	0.36854303	-0.8619708	0.38870359	NA
Tbr1	79.7261756	-0.3036578	0.35240565	-0.8616711	0.38886855	0.99938111
Ccdc58	2.00822471	0.35119185	0.4078154	0.86115396	0.38915325	NA
Eif2d	12.5938936	0.31919111	0.37069509	0.86106106	0.38920441	0.99938111
Calcr	4.81357232	0.35680223	0.41452645	0.8607466	0.38937762	0.99938111
Aif1l	3.23590555	-0.3556286	0.41336214	-0.8603318	0.38960618	NA
Ddx18	3.76136246	-0.3580826	0.41621669	-0.8603274	0.38960858	NA
Crhr2	2.40120797	0.35109589	0.40812893	0.86025728	0.38964723	NA
Zscan21	5.07041395	-0.3546822	0.41236479	-0.8601176	0.38972424	0.99938111
BC089491	1.21431103	0.28889582	0.33595219	0.85993133	0.3898269	NA
A230065H16Rik	1.07901992	-0.297029	0.34545459	-0.8598207	0.38988789	NA
Btaf1	21.5540853	0.30648867	0.3564649	0.85980044	0.38989906	0.99938111
Atp7b	0.90333973	-0.2716833	0.3160882	-0.8595173	0.39005519	NA
4732491K20Rik	2.69153101	0.35730001	0.41583845	0.85922794	0.39021477	NA
Ttbk2	177.867657	0.26102969	0.30412756	0.85829015	0.39073227	0.99938111
Lonrf2	313.38199	0.25308293	0.29488505	0.85824266	0.39075848	0.99938111
Zfp516	19.9318242	0.29872796	0.34807586	0.85822659	0.39076736	0.99938111
Ptbp3	27.1626385	-0.2496441	0.29093426	-0.8580772	0.39084982	0.99938111
Kpnb1	129.822604	0.1951672	0.22748469	0.85793553	0.39092806	0.99938111
Chst7	0.50571956	-0.2108662	0.24578588	-0.8579266	0.39093301	NA
Inpp5f	111.587775	0.30853342	0.35966805	0.85782827	0.3909873	0.99938111
Ergic1	115.00082	-0.2082386	0.24284175	-0.8575073	0.39116459	0.99938111
Cep290	15.4039475	0.35690472	0.41632599	0.85727225	0.39129444	0.99938111
Vopp1	50.5818701	0.23489182	0.27408542	0.85700226	0.39144364	0.99938111
Llgl1	37.6998495	-0.347535	0.40564119	-0.8567547	0.39158046	0.99938111
Sprn	82.9133	-0.2687671	0.31372424	-0.8566984	0.39161157	0.99938111
Plekhh1	9.77823708	-0.3411014	0.39816226	-0.8566895	0.39161652	0.99938111
Tmsb10	35.1521131	-0.2873667	0.33546027	-0.8566341	0.39164715	0.99938111
Ptplad1	71.6544341	-0.2965284	0.34617439	-0.8565869	0.39167324	0.99938111
Syne2	9.90476897	0.32699577	0.38178253	0.85649747	0.39172267	0.99938111
Slc37a1	3.79936403	0.34576063	0.40370698	0.85646433	0.39174099	NA
Ddx10	9.19603114	0.34048884	0.39764627	0.85626061	0.39185364	0.99938111
Erich6	2.04657742	-0.3363166	0.39279091	-0.856223	0.39187446	NA
Nov	52.2133192	-0.3435147	0.40121751	-0.8561806	0.39189788	0.99938111
Tomm40	49.614686	0.33060457	0.38629937	0.85582477	0.39209471	0.99938111
Npas3	26.7903283	-0.3320594	0.38801266	-0.8557953	0.39211101	0.99938111
Htr3a	2.764889	-0.3477172	0.40631972	-0.8557725	0.39212364	NA
Cldn14	0.85955829	-0.2123541	0.24815214	-0.8557417	0.39214066	NA
Idh3g	77.3558083	-0.2096491	0.24499976	-0.8557114	0.39215744	0.99938111
Kcnk1	41.9223888	-0.2663291	0.31128676	-0.8555748	0.39223303	0.99938111
Hecw1	52.3145737	0.2717464	0.31762365	0.85556098	0.39224066	0.99938111
Ccdc158	0.5885222	0.26169647	0.30589447	0.85551224	0.39226763	NA
Lrrc4	81.3018968	0.26228518	0.30658711	0.85549971	0.39227457	0.99938111
Nlk	133.815151	0.23886183	0.27930744	0.85519322	0.39244419	0.99938111
Haus6	3.78139504	0.35533632	0.41553967	0.85512008	0.39248467	NA
Cela1	0.66992581	-0.2389644	0.27946563	-0.8550762	0.39250896	NA
Ninj2	0.66992581	-0.2389644	0.27946563	-0.8550762	0.39250896	NA
Prickle3	0.66992581	-0.2389644	0.27946563	-0.8550762	0.39250896	NA
Cdk20	7.57128173	0.34410303	0.40257214	0.85476117	0.39268338	0.99938111
Mier3	16.9001037	-0.3022365	0.35360767	-0.8547226	0.39270472	0.99938111
Bcs1l	15.5169877	-0.2820437	0.33002881	-0.8546033	0.39277077	0.99938111
2510009E07Rik	67.4532649	0.19515456	0.22836016	0.85455123	0.39279963	0.99938111
Clint1	39.5854443	0.22295891	0.26098069	0.8543119	0.3929322	0.99938111
Abhd14b	3.78838107	-0.3556577	0.41637065	-0.8541853	0.39300232	NA
Gm7120	16.3978746	-0.3350781	0.39229812	-0.8541414	0.39302666	0.99938111
Rab11b	198.142558	-0.3041797	0.35619879	-0.8539605	0.39312686	0.99938111
Atp9a	216.255534	0.28573793	0.33466934	0.85379177	0.39322038	0.99938111
1600023N17Rik	0.43233526	0.20488682	0.24002275	0.85361418	0.3933188	NA
Pif1	0.43233526	0.20488682	0.24002275	0.85361418	0.3933188	NA
Eif3m	2.87910114	0.35459697	0.41548737	0.85344826	0.39341077	NA
Dctn2	135.667063	0.23995912	0.2812409	0.85321557	0.39353977	0.99938111
Pcp4l1	96.9365805	-0.252487	0.29599473	-0.8530119	0.39365268	0.99938111
Hhatl	3.46036986	-0.354432	0.41562253	-0.8527737	0.39378479	NA
Smc1a	37.8731708	0.32480698	0.38107048	0.85235408	0.3940176	0.99938111
Rnf39	2.23979702	0.3286598	0.38567171	0.85217502	0.39411696	NA
Tie1	1.1538464	0.29293219	0.34388048	0.85184302	0.39430123	NA
Actr8	27.4776142	0.26638601	0.31280785	0.85159629	0.3944382	0.99938111
E530011L22Rik	8.08203866	0.33364773	0.39180933	0.85155638	0.39446036	0.99938111
Wdr34	28.1959615	0.32316597	0.37951017	0.85153443	0.39447255	0.99938111
A330093E20Rik	1.30771886	-0.3219081	0.37806021	-0.8514731	0.39450658	NA
Mastl	1.38622732	0.29850108	0.35061414	0.85136637	0.39456587	NA
Nckap5	2.71733025	0.34873586	0.4096197	0.85136495	0.39456666	NA
Gm7444	1.48612984	0.29258528	0.34367889	0.8513333	0.39458423	NA
Tomm20	225.477954	0.18397235	0.21609942	0.85133201	0.39458495	0.99938111
BB031773	0.51388236	-0.2357461	0.27696351	-0.851181	0.39466882	NA
Pdgfd	0.51388236	-0.2357461	0.27696351	-0.851181	0.39466882	NA
Hmces	3.511082	-0.3520079	0.41371085	-0.8508548	0.39485003	NA
Ccdc23	2.49151904	-0.3500469	0.41152739	-0.8506042	0.39498924	NA
Supt7l	11.4446324	-0.3170185	0.37273685	-0.8505157	0.39503846	0.99938111
Ppp5c	71.1800053	0.203901	0.23974712	0.85048365	0.39505625	0.99938111
Tnnt1	1.21305638	0.29838222	0.35084815	0.85045973	0.39506954	NA
Zc3h15	42.8156885	0.28420839	0.33420061	0.85041252	0.39509578	0.99938111
Vamp4	45.0697508	0.26048718	0.30631559	0.85038824	0.39510927	0.99938111
Crybb1	0.74729628	-0.2760697	0.32467777	-0.8502882	0.39516487	NA

GeneID	Normalised expression for Chow#1	Normalised expression for Chow#2	Normalised expression for Chow#3	Normalised expression for Chow#4	Normalised expression for HFD#1	Normalised expression for HFD#2	Normalised expression for HFD#3	Normalised expression for HFD#4
S1pr2	0	3.16766603	3.49886938	0	1.66481172	0	0.96226389	0
Rusc1	130.376988	85.0744592	114.826531	295.744021	271.36431	127.013889	102.962236	340.111502
Fam206a	37.6345944	26.6988994	25.1282437	12.4088401	11.653682	14.1126543	31.7547083	20.5436478
Patz1	22.1775288	19.0059962	25.1282437	37.2265202	41.620293	21.1689814	20.2075417	50.2178057
A330021E22Ri	8.06455594	4.97776091	5.72542262	10.3407001	0	10.5844907	14.4339583	15.9783927
Tmem256	28.2259458	15.3858064	22.9016905	24.8176801	6.65924688	7.05632714	22.1320694	31.9567854
Tom1l2	219.087103	209.065958	206.433293	368.128922	482.795399	218.746141	171.282972	392.611935
Gm11992	0.67204633	0.90504744	1.27231614	2.06814001	0	0	0.96226389	0
Rad9a	16.1291119	11.7656167	15.5858727	37.2265202	16.6481172	11.7605452	10.5849028	18.2610202
Mfsd4	28.8979921	68.3310816	45.4853019	47.5672202	43.2851047	42.3379629	25.0188611	43.3699231
Zfp37	24.1936678	22.6261859	28.3090341	6.20442003	18.3129289	45.8661264	37.5282917	11.4131377
Hcn1	71.2369108	141.639924	111.009583	45.4990802	64.9276571	265.788322	107.773556	68.4788259
Eml5	28.8979921	28.0564706	32.7621406	26.8858201	33.2962344	47.042181	59.6603611	9.13051012
Tmem132d	5.37637063	18.1009488	14.6316356	6.20442003	11.653682	21.1689814	13.4716944	18.2610202
Lipa	9.4086486	16.7433776	14.3135566	6.20442003	1.66481172	17.6408179	7.69811111	4.56525506
Scn8a	160.619072	282.374801	228.062668	126.156541	283.017992	475.126028	129.905625	159.783927
Zdhhc22	27.5538995	42.0847059	31.1717454	47.5672202	39.9554813	21.1689814	10.5849028	43.3699231
Gm4925	2.68818531	0.45252372	1.27231614	0	0	0	0.96226389	0
Adprh	28.2259458	28.961518	29.8994292	57.9079203	41.620293	36.4576902	34.6415	63.9135709
Mir124a-2	0	0.45252372	0.31807903	0	0	1.17605452	1.92452778	0
Tagln	0	0.45252372	0.31807903	0	0	1.17605452	1.92452778	0
Snx7	19.4893435	15.3858064	12.4050823	6.20442003	6.65924688	16.4647633	30.7924444	20.5436478
Oscar	0	0.45252372	0.63615807	0	3.32962344	0	0	2.28262753
Kat5	41.6668724	39.3695635	44.5310648	39.2946602	24.9721758	30.5774176	44.2641389	36.5220405
Ak4	36.9625481	27.6039469	25.4463228	49.6353602	38.2906696	21.1689814	26.9433889	20.5436478
Wdr41	31.5861774	20.3635674	26.7186389	49.6353602	24.9721758	27.0492541	30.7924444	9.13051012
Usp14	124.328571	114.035977	127.867772	113.747701	74.9165274	192.872942	191.490514	123.261887
Kcnk4	2.01613899	4.07271347	3.49886938	4.13628002	4.99443516	1.17605452	0	2.28262753
I830012O16Ri	2.68818531	2.26261859	2.54463228	0	0	0	2.88679167	0
Tbr1	104.839227	119.013738	100.831054	35.1583802	114.872009	34.1055812	67.3584722	61.6309433
Ccdc58	0.67204633	1.35757116	1.59039517	2.06814001	4.99443516	1.17605452	1.92452778	2.28262753
Eif2d	12.0968339	5.43028463	11.1327662	14.4769801	21.6425524	9.40843619	10.5849028	15.9783927
Calcr	1.34409266	3.62018975	2.54463228	8.27256004	1.66481172	15.2887088	5.77358333	0
Aif1l	2.01613899	3.62018975	3.18079035	8.27256004	6.65924688	1.17605452	0.96226389	0
Ddx18	7.39250961	3.62018975	6.04350166	2.06814001	1.66481172	3.52816357	5.77358333	0
Crhr2	0	2.26261859	1.27231614	4.13628002	1.66481172	4.7042181	2.88679167	2.28262753
Zscan21	8.06455594	6.33533207	6.99773876	4.13628002	1.66481172	4.7042181	8.660375	0
BC089491	0.67204633	0	1.59039517	0	0	0	2.88679167	4.56525506
A230065H16R	0.67204633	0.90504744	0.63615807	4.13628002	0	0	0	2.28262753
Btaf1	24.1936678	18.1009488	24.1740066	6.20442003	13.3184938	37.6337448	25.981125	22.8262753
Atp7b	0	1.81009488	0.31807903	4.13628002	0	0	0.96226389	0
4732491K20Ri	3.36023164	1.35757116	2.86271131	0	3.32962344	3.52816357	4.81131944	2.28262753
Ttbk2	156.586795	173.316584	189.893184	115.815841	158.157113	302.246013	224.207486	102.718239
Lonrf2	217.070964	219.926527	229.971142	461.195222	336.291968	351.640303	236.716917	454.242879
Zfp516	14.7850192	18.1009488	11.1327662	26.8858201	33.2962344	23.5210905	13.4716944	18.2610202
Ptbp3	24.8657142	30.7716129	27.672876	37.2265202	19.9777406	28.2253086	21.1698055	27.3915304
Kpnb1	127.016756	127.611689	120.233875	107.543281	113.207197	164.647633	166.471653	111.848749
Chst7	2.68818531	1.35757116	0	0	0	0	0	0
Inpp5f	108.199459	105.438027	110.055346	59.9760603	48.2795399	204.633487	187.641458	68.4788259
Ergic1	115.591968	159.740873	114.190373	103.407001	111.542385	108.197016	81.7924305	125.544514
Cep290	8.06455594	19.0059962	15.2677937	4.13628002	1.66481172	45.8661264	26.9433889	2.28262753
Vopp1	36.9625481	61.9957495	44.2129858	39.2946602	44.9499164	68.2111624	51.96225	57.0656883
Llgl1	42.3389187	28.0564706	29.5813502	80.6574604	66.5924688	3.52816357	21.1698055	29.6741579
Sprn	100.134903	125.801594	103.693765	37.2265202	69.9220923	90.5561984	78.9056389	57.0656883
Plekhh1	9.4086486	13.1231878	9.86045007	14.4769801	18.3129289	8.23238167	4.81131944	0
Tmsb10	59.8121232	33.4867552	29.8994292	33.0902402	21.6425524	19.9929269	30.7924444	52.5004332
Ptplad1	59.1400769	128.96926	95.7417894	39.2946602	49.9443516	103.492798	50.9999861	45.6525506
Syne2	7.39250961	5.43028463	7.95197586	14.4769801	16.6481172	14.1126543	8.660375	4.56525506
Slc37a1	0.67204633	5.43028463	3.18079035	0	4.99443516	4.7042181	0	11.4131377
Ddx10	10.0806949	7.69290322	8.58813393	4.13628002	3.32962344	18.8168724	16.3584861	4.56525506
Erich6	4.03227797	0.90504744	0.9542371	6.20442003	0	2.35210905	1.92452778	0
Nov	62.5003085	110.868311	67.4327553	6.20442003	49.9443516	47.042181	57.7358333	15.9783927
Tomm40	33.6023164	20.8160911	27.990955	84.7937404	83.240586	28.2253086	26.9433889	91.3051012
Npas3	18.1452509	19.4585199	19.4028211	70.3167603	36.6258578	10.5844907	19.2452778	20.5436478
Htr3a	2.68818531	8.59795066	3.18079035	0	1.66481172	1.17605452	4.81131944	0
Cldn14	0.67204633	0	0	6.20442003	0	0	0	0
Idh3g	98.7908103	69.6886527	82.0643909	84.7937404	66.5924688	57.6266717	88.5282777	70.7614535
Kcnk1	33.6023164	69.6886527	43.5768277	37.2265202	34.9610461	44.6900719	25.981125	45.6525506
Hecw1	50.4034746	44.7998482	46.12146	45.4990802	71.586904	88.2040893	49.0754583	22.8262753
Ccdc158	0	0.90504744	0	0	1.66481172	1.17605452	0.96226389	0
Lrrc4	73.9250961	100.912789	79.8378377	37.2265202	79.9109626	119.957561	94.3018611	68.4788259
Nlk	113.575829	119.918786	139.318617	107.543281	158.157113	101.140689	104.886764	225.980126
Haus6	0.67204633	5.88280835	2.54463228	2.06814001	3.32962344	10.5844907	2.88679167	2.28262753
Cela1	0	0.90504744	0.31807903	4.13628002	0	0	0	0
Ninj2	0	0.90504744	0.31807903	4.13628002	0	0	0	0
Prickle3	0	0.90504744	0.31807903	4.13628002	0	0	0	0
Cdk20	5.37637063	3.62018975	5.40734359	10.3407001	6.65924688	7.05632714	3.84905555	18.2610202
Mier3	26.8818531	20.8160911	15.9039517	14.4769801	13.3184938	19.9929269	19.2452778	4.56525506
Bcs1l	17.4732045	16.7433776	17.8124259	18.6132601	18.3129289	12.9365998	15.3962222	6.84788259
2510009E07Rik	60.4841696	61.5432258	60.7530956	70.3167603	66.5924688	78.7956531	81.7924305	59.3483158
Clint1	33.6023164	34.8443264	42.6225906	33.0902402	34.9610461	47.042181	47.1509305	43.3699231
Abhd14b	4.03227797	4.97776091	3.18079035	6.20442003	4.99443516	2.35210905	0	4.56525506
Gm7120	12.7688802	33.9392789	20.3570582	14.4769801	6.65924688	22.345036	20.2075417	4.56525506
Rab11b	178.092277	154.763112	172.398837	397.082882	198.112595	71.739326	129.905625	283.045814
Atp9a	132.393127	225.809336	184.167761	215.086561	372.917825	258.731995	87.5660139	253.371656
1600023N17R	0	0	0	0	0	1.17605452	0	2.28262753
Pif1	0	0	0	0	0	1.17605452	0	2.28262753
Eif3m	4.03227797	1.35757116	2.22655324	0	3.32962344	2.35210905	2.88679167	6.84788259
Dctn2	131.049034	80.0966983	117.371164	163.383061	161.486737	99.9646345	135.679208	196.305968
Pcp4l1	145.162007	81.4542694	128.185851	70.3167603	61.5980337	75.2674895	95.264125	118.696632
Hhatl	3.36023164	4.07271347	3.18079035	6.20442003	1.66481172	2.35210905	0	6.84788259
Smc1a	33.6023164	33.0342315	45.4853019	14.4769801	29.966611	97.6125255	25.981125	22.8262753
Rnf39	0	0.90504744	2.54463228	2.06814001	6.65924688	1.17605452	0	4.56525506
Tie1	0	1.35757116	0.9542371	0	4.99443516	0	1.92452778	0
Actr8	26.8818531	14.9332827	28.3090341	28.9539601	21.6425524	30.5774176	36.5660278	31.9567854
E530011L22Ri	6.04841696	5.88280835	3.81694841	12.4088401	6.65924688	9.40843619	6.73584722	13.6957652
Wdr34	17.4732045	15.8383302	15.5858727	47.5672202	33.2962344	15.2887088	21.1698055	59.3483158
A330093E20Ri	2.01613899	4.07271347	1.27231614	0	0	1.17605452	1.92452778	0
Mastl	2.68818531	0.45252372	0	0	0	4.7042181	0.96226389	2.28262753
Nckap5	4.03227797	2.26261859	1.59039517	0	1.66481172	3.52816357	8.660375	0
Gm7444	0.67204633	0	0.63615807	2.06814001	1.66481172	0	0	6.84788259
Tomm20	174.059999	220.831575	226.154194	221.290981	191.453348	279.900977	273.282944	216.849615
BB031773	0	0.45252372	1.59039517	2.06814001	0	0	0	0
Pdgfd	0	0.45252372	1.59039517	2.06814001	0	0	0	0
Hmces	4.03227797	6.33533207	6.67965972	0	1.66481172	0	4.81131944	4.56525506
Ccdc23	3.36023164	2.71514231	2.86271131	4.13628002	3.32962344	3.52816357	0	0
Supt7l	14.7850192	10.4080455	13.0412404	16.5451201	6.65924688	8.23238167	17.32075	4.56525506
Ppp5c	79.3014668	63.8058444	57.8903843	62.0442003	93.2294563	69.3872169	79.8679027	63.9135709
Tnnt1	1.34409266	0	0.9542371	0	1.66481172	1.17605452	0	4.56525506
Zc3h15	49.7314283	35.2968501	42.3045116	22.7495401	23.3073641	55.2745626	75.0565833	38.804668
Vamp4	30.9141311	42.0847059	44.8491439	45.4990802	34.9610461	72.9153805	59.6603611	29.6741579
Crybb1	0	1.35757116	1.59039517	2.06814001	0	0	0.96226389	0

GeneID	Base mean	log2(FC)	StdErr	Wald-Stats	P-value	P-adj		GeneID	Normalised expression for Chow#1	Normalised expression for Chow#2	Normalised expression for Chow#3	Normalised expression for Chow#4	Normalised expression for HFD#1	Normalised expression for HFD#2	Normalised expression for HFD#3	Normalised expression for HFD#4
Myadml2	2.99948427	0.35315956	0.41534766	0.85027459	0.39517244	NA		Myadml2	2.68818531	3.16766603	2.22655324	0	3.32962344	7.05632714	0.96226389	4.56525506
Gpr137b-ps	12.8758026	-0.2975787	0.3500022	-0.8502194	0.39520313	0.99938111		Gpr137b-ps	11.4247876	12.6706641	15.2677937	20.6814001	9.98887032	9.40843619	14.4339583	9.13051012
Glg1	86.2693859	0.31868877	0.37483308	0.85021517	0.39520547	0.99938111		Glg1	70.5648645	111.773359	87.4717345	20.6814001	93.2294563	183.464506	56.7735694	66.1961984
Champ1	33.5448023	-0.2418992	0.28474766	-0.8495214	0.39559125	0.99938111		Champ1	35.6184554	37.5594687	32.1259825	43.4309402	39.9554813	34.1055812	25.0188611	20.5436478
Bcam	5.60436293	-0.3488746	0.41085179	-0.8491494	0.39579815	0.99938111		Bcam	4.7043243	7.2403795	6.67965972	8.27256004	9.98887032	4.7042181	0.96226389	2.28262753
Oser1	4.99595323	-0.3522022	0.41486537	-0.8489553	0.39590616	0.99938111		Oser1	9.4086486	5.43028463	4.45310648	6.20442003	0	3.52816357	8.660375	2.28262753
Trmt12	8.42276112	0.34398566	0.40519334	0.84894205	0.39591354	0.99938111		Trmt12	10.0806949	4.07271347	2.22655324	12.4088401	13.3184938	9.40843619	6.73584722	9.13051012
Setx	66.7666251	0.28677314	0.33784661	0.84882645	0.39597787	0.99938111		Setx	53.0916599	65.6159392	71.8858618	43.4309402	68.2572805	132.894161	69.283	29.6741579
Gatc	30.6314149	-0.2764945	0.32573774	-0.8488256	0.39597834	0.99938111		Gatc	47.043243	29.8665654	41.9864326	16.5451201	23.3073641	22.345036	36.5660278	27.3915304
Art3	0.40659299	-0.2274689	0.26798259	-0.8488197	0.39598162	NA		Art3	0.67204633	2.26261859	0.31807903	0	0	0	0	0
Ntrk1	0.40659299	-0.2274689	0.26798259	-0.8488197	0.39598162	NA		Ntrk1	0.67204633	2.26261859	0.31807903	0	0	0	0	0
Nudt22	4.07659523	-0.3439351	0.40521245	-0.8487772	0.39600528	NA		Nudt22	2.01613899	1.81009488	1.59039517	16.5451201	1.66481172	1.17605452	0.96226389	6.84788259
Col4a2	10.4560507	0.34606062	0.40772955	0.8487504	0.3960202	0.99938111		Col4a2	6.04841696	10.4080455	6.36158069	10.3407001	26.6369875	8.23238167	1.92452778	13.6957652
Spns1	30.4313904	0.346136	0.40804281	0.84828353	0.39628009	0.99938111		Spns1	13.4409266	20.8160911	18.130505	45.4990802	58.2684102	8.23238167	10.5849028	68.4788259
Wdr53	7.7268246	0.34978695	0.41242289	0.84812691	0.3963673	0.99938111		Wdr53	4.03227797	0.45252372	4.13502745	14.4769801	6.65924688	1.17605452	5.77358333	25.1089028
Ube2v1	102.056886	-0.2725197	0.32132507	-0.8481122	0.39637551	0.99938111		Ube2v1	123.656524	85.5269829	96.3779475	148.906081	119.866444	41.1619083	73.1320555	127.827142
Rps8	11.0649976	-0.3444205	0.40619311	-0.8479231	0.39648078	0.99938111		Rps8	14.7850192	9.05047438	8.90621297	22.7495401	1.66481172	11.7605452	17.32075	2.28262753
Lims2	4.38468398	-0.3512943	0.41441081	-0.8476958	0.39660739	NA		Lims2	4.7043243	3.16766603	4.13502745	10.3407001	4.99443516	3.52816357	1.92452778	2.28262753
Gm10790	0.38978741	-0.2312657	0.27285591	-0.8475746	0.39667491	NA		Gm10790	0.67204633	1.81009488	0.63615807	0	0	0	0	0
Gm10785	1.07103051	0.29726068	0.35082832	0.84731097	0.39682181	NA		Gm10785	0	1.35757116	0.63615807	0	3.32962344	0	0.96226389	2.28262753
Samd15	1.81760235	0.33640549	0.39718101	0.8469828	0.39700471	NA		Samd15	2.01613899	1.35757116	1.59039517	0	1.66481172	1.17605452	6.73584722	0
Gm16701	3.1502249	-0.3519958	0.41559546	-0.8469673	0.39701334	NA		Gm16701	4.7043243	5.43028463	5.40734359	0	0	3.52816357	3.84905555	2.28262753
Sema4a	20.6381969	-0.3110199	0.36726964	-0.8468433	0.39708244	0.99938111		Sema4a	18.8172972	32.5817078	18.766663	22.7495401	28.3017992	15.2887088	5.77358333	22.8262753
Sh2b2	9.67591197	-0.3470388	0.41000439	-0.8464269	0.39731461	0.99938111		Sh2b2	8.73660227	4.97776091	6.99773876	26.8858201	4.99443516	8.23238167	2.88679167	13.6957652
Btrc	105.367327	0.20932089	0.24736245	0.84621127	0.3974349	0.99938111		Btrc	100.134903	74.2138899	93.5152361	122.020261	133.184938	124.66178	85.6414861	109.566121
Smc4	4.86884851	0.34862612	0.4120836	0.84600824	0.39754815	0.99938111		Smc4	4.03227797	4.07271347	4.13502745	4.13628002	3.32962344	10.5844907	8.660375	0
Naglu	3.39814195	0.34284972	0.40527493	0.84596825	0.39757046	NA		Naglu	1.34409266	0.90504744	1.90847421	6.20442003	11.653682	0	2.88679167	2.28262753
Ndufs6	17.8431413	-0.3382591	0.39991293	-0.845832	0.3976465	0.99938111		Ndufs6	20.1613899	9.5029981	8.58813393	47.5672202	18.3129289	9.40843619	8.660375	20.5436478
Ehbp1	37.1826951	0.31275614	0.3699463	0.84540957	0.3978822	0.99938111		Ehbp1	32.9302701	38.4645161	40.7141164	14.4769801	21.6425524	69.3872169	61.5848889	18.2610202
Osbpl8	118.364842	0.28258239	0.334263	0.84538937	0.39789348	0.99938111		Osbpl8	125.672663	102.722884	136.773985	49.6353602	81.5757743	182.288451	183.792403	84.4572186
5730408K05Rik	0.40561143	0.20145932	0.23830559	0.84538226	0.39789745	NA		5730408K05Ri	0	0	0	0	0	0	0.96226389	2.28262753
A930007I19Rik	0.40561143	0.20145932	0.23830559	0.84538226	0.39789745	NA		A930007I19Ri	0	0	0	0	0	0	0.96226389	2.28262753
Clca2	0.40561143	0.20145932	0.23830559	0.84538226	0.39789745	NA		Clca2	0	0	0	0	0	0	0.96226389	2.28262753
Gm5936	0.40561143	0.20145932	0.23830559	0.84538226	0.39789745	NA		Gm5936	0	0	0	0	0	0	0.96226389	2.28262753
Kcne2	0.40561143	0.20145932	0.23830559	0.84538226	0.39789745	NA		Kcne2	0	0	0	0	0	0	0.96226389	2.28262753
Tjp3	0.40561143	0.20145932	0.23830559	0.84538226	0.39789745	NA		Tjp3	0	0	0	0	0	0	0.96226389	2.28262753
Pclo	320.369669	0.34708385	0.41058226	0.84534547	0.39791798	0.99938111		Pclo	267.474439	410.439013	290.406159	51.7035003	168.145984	969.068928	309.848972	95.8703563
Cbll1	22.8873226	-0.3155338	0.37327595	-0.8453097	0.39793797	0.99938111		Cbll1	32.2582238	28.5089943	37.5333261	6.20442003	11.653682	28.2253086	25.0188611	13.6957652
Psme2b	12.9737843	0.29333948	0.34711852	0.8450701	0.3980717	0.99938111		Psme2b	10.7527413	8.59795066	10.8146872	16.5451201	9.98887032	17.6408179	13.4716944	15.9783927
Esr1	10.2658425	0.31807018	0.37643636	0.84495074	0.39813834	0.99938111		Esr1	4.7043243	6.78785578	8.90621297	16.5451201	8.3240586	12.9365998	12.5094306	11.4131377
Chchd7	10.7413188	0.33229159	0.3933091	0.84486119	0.39818835	0.99938111		Chchd7	8.06455594	14.0282353	9.86045007	2.06814001	4.99443516	9.40843619	19.2452778	18.2610202
Fam98c	8.76362746	0.34939428	0.41359854	0.84476671	0.39824111	0.99938111		Fam98c	3.36023164	4.52523719	4.13502745	16.5451201	4.99443516	4.7042181	6.73584722	25.1089028
Xrn2	24.5024753	-0.2732091	0.32346849	-0.8446235	0.39832111	0.99938111		Xrn2	28.8979921	25.793852	35.9429309	18.6132601	14.9833055	31.7534722	24.0565972	15.9783927
Ngrn	27.1718788	0.2939361	0.34811598	0.84436256	0.39846684	0.99938111		Ngrn	20.1613899	17.1959013	18.130505	41.3628002	18.3129289	19.9929269	36.5660278	45.6525506
Tmx4	211.372989	0.3086855	0.36559902	0.84432803	0.39848613	0.99938111		Tmx4	127.688802	259.748615	214.385269	122.020261	134.849749	513.935827	190.52825	127.827142
Lpin2	70.435825	0.21914313	0.25955152	0.84431457	0.39849365	0.99938111		Lpin2	65.1884939	66.068463	62.3434908	66.1804803	71.586904	109.373071	70.2452639	52.5004332
Map3k12	99.8254486	0.18472435	0.21879159	0.84429366	0.39850533	0.99938111		Map3k12	104.839227	73.7613662	96.6960265	97.2025805	99.8887032	104.668853	109.698083	111.848749
A230050P20Rik	34.0726594	0.32913671	0.38996068	0.84402539	0.39865522	0.99938111		A230050P20Ri	24.1936678	12.6706641	23.8559276	53.7716403	49.9443516	11.7605452	27.9056528	68.4788259
Fn3k	15.1725607	0.32006618	0.37922977	0.8439901	0.39867494	0.99938111		Fn3k	6.72046328	9.95552182	11.1327662	24.8176801	24.9721758	9.40843619	11.5471667	22.8262753
Avil	0.53966001	-0.2328271	0.2758654	-0.8439881	0.39867603	NA		Avil	1.34409266	0.90504744	0	2.06814001	0	0	0	0
Sntb1	2.33489759	0.32630249	0.38666238	0.84389511	0.39872802	NA		Sntb1	0	1.35757116	0.9542371	4.13628002	0	1.17605452	1.92452778	9.13051012
Ankrd63	35.1078907	-0.287414	0.34071154	-0.84357	0.39890976	0.99938111		Ankrd63	43.6830113	39.3695635	47.3937761	24.8176801	54.9387868	22.345036	16.3584861	31.9567854
Ndufs4	25.0151166	-0.3032416	0.3594756	-0.8435665	0.39891168	0.99938111		Ndufs4	32.9302701	26.2463757	35.3067728	20.6814001	8.3240586	30.5774176	34.6415	11.4131377
Snrpa	31.607245	-0.3147864	0.37322278	-0.8434276	0.39898932	0.99938111		Snrpa	34.2743627	34.8443264	29.2632712	45.4990802	41.620293	8.23238167	13.4716944	45.6525506
Mbtd1	10.4384862	0.32357644	0.38367516	0.84336043	0.3990269	0.99938111		Mbtd1	12.0968339	9.95552182	10.1785291	2.06814001	8.3240586	10.5844907	21.1698055	9.13051012
Cep95	2.43537147	0.34609034	0.41044434	0.84320895	0.3991116	NA		Cep95	1.34409266	3.62018975	1.59039517	0	1.66481172	7.05632714	1.92452778	2.28262753
Ttc30b	9.47906624	0.31254012	0.370821	0.84283285	0.39932193	0.99938111		Ttc30b	7.39250961	10.8605693	6.67965972	8.27256004	11.653682	12.9365998	13.4716944	4.56525506
Rpl22l1	5.12573365	0.35034474	0.41570038	0.84278185	0.39935046	0.99938111		Rpl22l1	5.37637063	3.16766603	6.67965972	0	3.32962344	7.05632714	15.3962222	0
4932441J04Rik	0.92746251	-0.2930317	0.34769807	-0.8427762	0.39935362	NA		4932441J04Ri	1.34409266	0.45252372	1.27231614	2.06814001	0	0	0	2.28262753
Klhl33	2.14887404	0.32572737	0.38653593	0.8426833	0.39940559	NA		Klhl33	2.01613899	0	0.31807903	4.13628002	1.66481172	3.52816357	0.96226389	4.56525506
Ube2l6	3.51342306	-0.3508197	0.41636021	-0.8425869	0.39945951	NA		Ube2l6	6.72046328	3.16766603	4.45310648	2.06814001	4.99443516	1.17605452	0.96226389	4.56525506
Prrc2b	506.094848	0.25458761	0.30220402	0.8424362	0.39954384	0.99938111		Prrc2b	391.130963	429.897533	357.202756	636.987123	840.729919	560.978008	295.415014	536.41747
Ubald2	11.0632635	-0.3463941	0.41121414	-0.8423692	0.39958135	0.99938111		Ubald2	11.4247876	6.33533207	7.31581779	28.9539601	16.6481172	3.52816357	2.88679167	11.4131377
Pcyox1l	18.6782293	0.34829929	0.41363222	0.84205069	0.39975958	0.99938111		Pcyox1l	8.73660227	16.2908539	12.4050823	20.6814001	28.3017992	14.1126543	0.96226389	47.9351782
Cyb5b	161.646886	0.18348026	0.21789702	0.84205034	0.39975978	0.99938111		Cyb5b	133.737219	145.260114	142.181328	184.064461	163.151549	138.774434	201.113153	184.89283
Cd2bp2	48.0934174	0.27675209	0.3287216	0.84190418	0.39984159	0.99938111		Cd2bp2	36.2905017	29.8665654	34.6706148	70.3167603	48.2795399	35.2816357	43.301875	86.7398462
Cnih2	158.760194	-0.2845313	0.33799662	-0.8418171	0.39989036	0.99938111		Cnih2	169.355675	160.64592	128.185851	254.381221	166.481172	77.6195986	76.0188472	237.393263
Gtf3c6	11.3991621	0.31355431	0.37248338	0.84179412	0.39990321	0.99938111		Gtf3c6	8.73660227	5.88280835	10.1785291	16.5451201	8.3240586	14.1126543	18.2830139	9.13051012
Tmem121	9.51626232	0.33683012	0.4001568	0.84174533	0.39993052	0.99938111		Tmem121	6.04841696	5.88280835	6.67965972	12.4088401	9.98887032	9.40843619	2.88679167	22.8262753
1700001L19Rik	7.98407108	-0.3390875	0.40286254	-0.8416954	0.39995848	0.99938111		1700001L19Ri	6.72046328	11.7656167	8.90621297	12.4088401	3.32962344	8.23238167	12.5094306	0
Sh3tc2	0.47547801	-0.2053691	0.24399777	-0.8416842	0.39996474	NA		Sh3tc2	0	3.16766603	0.63615807	0	0	0	0	0
Prkar2b	55.8268706	0.30545282	0.36293571	0.84161688	0.40000244	0.99938111		Prkar2b	61.1562159	47.5149905	62.0254117	20.6814001	44.9499164	108.197016	76.9811111	25.1089028
Strn3	69.933812	0.27901022	0.33172223	0.84109595	0.40029419	0.99938111		Strn3	75.9412351	73.3088425	63.6158069	33.0902402	46.6147282	103.492798	115.471667	47.9351782
Mpp1	13.8754823	-0.3032324	0.36056548	-0.8409913	0.40035284	0.99938111		Mpp1	17.4732045	13.1231878	17.4943469	16.5451201	6.65924688	9.40843619	21.1698055	9.13051012
Tomm5	8.23832374	-0.3311288	0.39382658	-0.8407985	0.40046085	0.99938111		Tomm5	9.4086486	6.33533207	12.0870033	12.4088401	4.99443516	5.88027262	12.5094306	2.28262753
Trim28	101.654285	0.27124655	0.32264363	0.84070017	0.40051592	0.99938111		Trim28	75.2691888	67.8785578	77.2932054	140.633521	151.497867	72.9153805	70.2452639	157.5013
Ggcx	7.05537103	-0.3490134	0.41524765	-0.8404945	0.40063117	0.99938111		Ggcx	4.03227797	13.1231878	8.58813393	8.27256004	4.99443516	10.5844907	0	6.84788259
Zfp36	3.7389533	-0.3346564	0.39834099	-0.8401254	0.4008381	NA		Zfp36	1.34409266	4.07271347	1.90847421	12.4088401	3.32962344	0	0	6.84788259
Gabbr1	622.099473	-0.2342712	0.27888854	-0.8400173	0.40089869	0.99938111		Gabbr1	498.658376	896.902011	652.3801	670.077363	665.924688	684.463733	319.471611	588.917903
1110007C09Rik	2.58393954	-0.3208195	0.38197284	-0.8399012	0.40096376	NA		1110007C09Ri	2.01613899	2.26261859	1.27231614	8.27256004	0	0	0	6.84788259
Mtfr1	2.41762766	-0.3422409	0.40761443	-0.8396193	0.40112189	NA		Mtfr1	2.01613899	7.2403795	3.18079035	0	1.66481172	2.35210905	2.88679167	0
Clic6	5.4180116	-0.3453427	0.41144792	-0.8393351	0.4012813	0.99938111		Clic6	2.01613899	7.2403795	3.49886938	16.5451201	0	1.17605452	10.5849028	2.28262753
Hs3st4	21.164204	-0.3102334	0.36962848	-0.8393115	0.40129456	0.99938111		Hs3st4	26.8818531	27.6039469	32.4440615	10.3407001	9.98887032	21.1689814	31.7547083	9.13051012
Mul1	32.1978769	0.31021334	0.3698034	0.83886016	0.40154779	0.99938111		Mul1	17.4732045	22.6261859	19.4028211	51.7035003	46.6147282	21.1689814	19.2452778	59.3483158
Pkm	505.004507	0.31296951	0.37341261	0.8381332	0.4019559	0.99938111		Pkm	405.915982	207.255863	310.445138	796.233904	844.059542	279.900977	305.999917	890.224737
Cebpzos	8.21757147	-0.3318605	0.39597474	-0.8380851	0.4019829	0.99938111		Cebpzos	8.06455594	9.5029981	10.8146872	10.3407001	11.653682	1.17605452	9.62263889	4.56525506
Rab14	241.836676	0.18881823	0.22529854	0.83808011	0.40198571	0.99938111		Rab14	253.361466	214.496243	226.472273	204.745861	179.799666	276.372813	314.660292	264.784794
Zdhhc9	47.0578471	-0.2448164	0.29215196	-0.8379761	0.4020441	0.99938111		Zdhhc9	34.9464091	72.8563187	49.9384084	47.5672202	38.2906696	47.042181	35.6037639	50.2178057
Itgb1bp1	14.0516502	-0.3273639	0.39071977	-0.8378483	0.4021159	0.99938111		Itgb1bp1	22.8495752	14.0282353	20.9932163	8.27256004	4.99443516	9.40843619	25.0188611	6.84788259
Opa1	134.434299	0.21786666	0.26014814	0.83747152	0.40232758	0.99938111		Opa1	125.672663	123.538975	140.590933	103.407001	106.54795	174.05607	194.377305	107.283494
Stat5b	39.2375715	0.30119044	0.35974449	0.83723434	0.40246086	0.99938111		Stat5b	28.8979921	20.8160911	26.7186389	59.9760603	48.2795399	31.7534722	22.1320694	75.3267085
Dnajb4	51.9219965	0.28257889	0.3375729	0.83708997	0.402542	0.99938111		Dnajb4	55.7798453	57.0179886	48.9841713	18.6132601	28.3017992	69.3872169	77.943375	59.3483158
0610037L13Rik	9.84240987	0.34184577	0.40842985	0.83697549	0.40260634	0.99938111		0610037L13Ri	16.8011582	7.69290322	5.72542262	0	16.6481172	4.7042181	13.4716944	13.6957652
Vrk1	6.47325538	0.32363847	0.3866889	0.83694791	0.40262185	0.99938111		Vrk1	6.72046328	4.97776091	5.40734359	4.13628002	6.65924688	7.05632714	7.69811111	9.13051012
Dnajc9	14.6834804	0.30162607	0.36049439	0.83670113	0.40276058	0.99938111		Dnajc9	11.4247876	13.5757116	13.0412404	14.4769801	9.98887032	21.1689814	26.9433889	6.84788259
Man2a1	9.33744987	0.34740742	0.41524076	0.83664093	0.40279443	0.99938111		Man2a1	4.03227797	14.0282353	8.2700549	2.06814001	16.6481172	23.5210905	3.84905555	2.28262753
Ubxn6	47.0550251	-0.2119261	0.2534682	-0.8361052	0.40309574	0.99938111		Ubxn6	55.1077989	50.2301328	45.4853019	53.7716403	46.6147282	34.1055812	50.0377222	41.0872956
Zfp26	17.8066988	0.27096222	0.32410509	0.83603198	0.40313691	0.99938111		Zfp26	14.7850192	16.2908539	19.7209001	10.3407001	16.6481172	16.4647633	23.0943333	25.1089028
Ucma	0.50773365	-0.2338578	0.27976279	-0.8359145	0.40320299	NA		Ucma	0	1.35757116	0.63615807	2.06814001	0	0	0	0
Klf6	36.9091263	0.30184419	0.36114313	0.83580209	0.40326625	0.99938111		Klf6	24.8657142	39.8220873	40.0779583	22.7495401	19.9777406	37.6337448	82.7546944	27.3915304
Hiat1	35.2249555	-0.2793073	0.33422378	-0.8356895	0.40332961	0.99938111		Hiat1	24.8657142	59.2806072	46.439539	26.8858201	28.3017992	44.6900719	30.7924444	20.5436478
Tatdn3	2.40588073	0.34537409	0.41328246	0.83568535	0.40333194	NA		Tatdn3	1.34409266	1.81009488	2.22655324	2.06814001	0	4.7042181	4.81131944	2.28262753
Rnaset2b	9.90040023	-0.3229547	0.38646157	-0.8356709	0.40334009	0.99938111		Rnaset2b	9.4086486	13.1231878	16.8581888	6.20442003	11.653682	12.9365998	6.73584722	2.28262753
Ptgr2	22.5131047	-0.3315218	0.39690861	-0.8352597	0.4035715	0.99938111		Ptgr2	32.2582238	21.2686148	28.6271131	24.8176801	1.66481172	14.1126543	41.3773472	15.9783927
Ppp1r3fos	1.83480256	0.31172157	0.37325395	0.83514607	0.40363547	NA		Ppp1r3fos	1.34409266	0.45252372	0.63615807	2.06814001	3.32962344	0	0	6.84788259
Scn10a	0.7232962	-0.2611816	0.31278723	-0.8350136	0.40371002	NA		Scn10a	0.67204633	3.62018975	0.31807903	0	0	1.17605452	0	0
Usp9x	289.269625	0.24837775	0.29746368	0.83498511	0.40372609	0.99938111		Usp9x	239.920539	275.586945	330.484117	194.405161	266.369875	491.590791	337.754625	178.044947
Glrx5	24.0809795	-0.2886181	0.3457267	-0.8348157	0.40382148	0.99938111		Glrx5	25.5377605	17.648425	24.4920857	43.4309402	14.9833055	12.9365998	30.7924444	22.8262753
Syt2	9.63525967	-0.3436438	0.41169206	-0.8347109	0.40388053	0.99938111		Syt2	8.06455594	13.1231878	15.2677937	10.3407001	1.66481172	21.1689814	2.88679167	4.56525506
Zfp7	3.00701352	-0.3470663	0.41593397	-0.8344264	0.40404074	NA		Zfp7	6.04841696	2.71514231	2.86271131	4.13628002	1.66481172	4.7042181	1.92452778	0
Wdr78	7.53332667	0.34724976	0.41635402	0.83402523	0.40426677	0.99938111		Wdr78	10.0806949	5.88280835	6.99773876	0	1.66481172	14.1126543	19.2452778	2.28262753
Nkpd1	0.99730691	-0.2759069	0.33085143	-0.8339299	0.4043205	NA		Nkpd1	0	0.90504744	1.27231614	4.13628002	1.66481172	0	0	0

GeneID	Base mean	log2(FC)	StdErr	Wald-Stats	P-value	P-adj	GeneID	Normalised expression for Chow#1	Normalised expression for Chow#2	Normalised expression for Chow#3	Normalised expression for Chow#4	Normalised expression for HFD#1	Normalised expression for HFD#2	Normalised expression for HFD#3	Normalised expression for HFD#4
1700010K23Rik	0.80513053	0.28528012	0.34209927	0.83391035	0.4043315	NA	1700010K23Ri	0	1.35757116	0.31807903	0	1.66481172	1.17605452	1.92452778	0
Kif9	0.76170055	-0.2862214	0.34333093	-0.8336605	0.40447232	NA	Kif9	0.67204633	0.90504744	1.27231614	2.06814001	0	1.17605452	0	0
Lrif1	12.1861163	0.32599833	0.39106318	0.83362062	0.4044948	0.99938111	Lrif1	9.4086486	11.7656167	14.6316356	4.13628002	3.32962344	16.4647633	24.0565972	13.6957652
Ovca2	14.4148095	0.29851619	0.35812152	0.83356114	0.40452833	0.99938111	Ovca2	12.0968339	12.6706641	7.31581779	18.6132601	13.3184938	11.7605452	14.4339583	25.1089028
Igsf3	23.2129851	0.2879509	0.3454695	0.83350598	0.40455943	0.99938111	Igsf3	18.1452509	23.5312334	19.4028211	18.6132601	34.9610461	31.7534722	9.62263889	29.6741579
Dopey1	34.5558696	0.32089008	0.3850057	0.83346839	0.40458062	0.99938111	Dopey1	36.2905017	33.9392789	38.8056422	6.20442003	23.3073641	70.5632714	49.0754583	18.2610202
Sae1	59.5851358	0.19305854	0.23167296	0.83332358	0.40466226	0.99938111	Sae1	62.5003085	54.3028463	54.3915149	49.6353602	59.9332219	61.1548353	73.1320555	61.6309433
Kin	4.00691034	0.34647172	0.4160103	0.8328441	0.40493266	NA	Kin	5.37637063	3.62018975	3.18079035	0	1.66481172	8.23238167	7.69811111	2.28262753
Lama5	2.30084852	0.33317242	0.4000623	0.83280133	0.40495679	NA	Lama5	0.67204633	1.35757116	1.90847421	2.06814001	6.65924688	1.17605452	0	4.56525506
Gpd1	39.0744395	0.27786666	0.33369383	0.83269944	0.40501426	0.99938111	Gpd1	37.6345944	22.1736622	33.7163777	43.4309402	58.2684102	29.4013631	24.0565972	63.9135709
Armcx2	79.4166724	0.27226342	0.32698136	0.83265732	0.40503802	0.99938111	Armcx2	72.5810035	99.1026944	81.1101538	24.8176801	61.5980337	111.72518	88.5282777	95.8703563
Tinagl1	1.61095587	0.25841209	0.3103489	0.83265025	0.40504201	NA	Tinagl1	0	2.26261859	0.63615807	0	9.98887032	0	0	0
Cgref1	11.5130958	0.32330897	0.38835447	0.83250999	0.40512114	0.99938111	Cgref1	15.4570656	9.05047438	9.86045007	4.13628002	6.65924688	24.697145	15.3962222	6.84788259
Gucy2g	3.74880804	-0.3456838	0.41531397	-0.8323433	0.40521522	NA	Gucy2g	5.37637063	4.97776091	7.95197586	0	6.65924688	1.17605452	3.84905555	0
Reep2	55.7283962	-0.2609339	0.31350016	-0.8323247	0.40522568	0.99938111	Reep2	48.3873356	82.3593168	64.888123	49.6353602	66.5924688	62.3308898	25.981125	45.6525506
Mllt10	19.0123296	0.27483235	0.33026463	0.83215798	0.40531978	0.99938111	Mllt10	16.1291119	16.2908539	23.5378486	8.27256004	23.3073641	21.1689814	18.2830139	25.1089028
Fam161b	9.42953945	0.32728581	0.39335533	0.83203605	0.4053886	0.99938111	Fam161b	8.06455594	9.05047438	8.58813393	6.20442003	9.98887032	5.88027262	23.0943333	4.56525506
Fam110b	32.9859057	0.22618978	0.2719002	0.8318853	0.40547369	0.99938111	Fam110b	31.5861774	26.6988994	27.672876	35.1583802	28.3017992	36.4576902	34.6415	43.3699231
Irf2bp1	53.4607191	0.33234154	0.39952603	0.83183953	0.40549953	0.99938111	Irf2bp1	41.6668724	21.7211385	28.3090341	84.7937404	88.2350212	21.1689814	23.0943333	118.696632
Cercam	5.5579756	0.34363661	0.41311613	0.83181601	0.40551281	0.99938111	Cercam	2.01613899	2.26261859	4.13502745	10.3407001	4.99443516	3.52816357	5.77358333	11.4131377
2310035C23Rik	58.5357201	0.20368577	0.24488874	0.83174821	0.40555108	0.99938111	2310035C23Ri	61.8282622	43.8948007	58.8446214	49.6353602	64.9276571	56.4506172	59.6603611	73.044081
Pcdhb12	6.3985818	0.34358587	0.41321122	0.83150179	0.40569022	0.99938111	Pcdhb12	2.01613899	9.05047438	2.86271131	6.20442003	4.99443516	11.7605452	2.88679167	11.4131377
Zic4	1.18026092	-0.3070584	0.36932585	-0.8314023	0.40574638	NA	Zic4	0.67204633	0.90504744	1.59039517	4.13628002	0	1.17605452	0.96226389	0
Mdm4	17.6929136	0.27896768	0.33557933	0.83130175	0.40580319	0.99938111	Mdm4	16.8011582	17.1959013	19.0847421	8.27256004	13.3184938	25.8731995	25.0188611	15.9783927
Cebpg	19.826952	0.30076122	0.36179898	0.83129373	0.40580772	0.99938111	Cebpg	13.4409266	20.8160911	25.7644018	6.20442003	18.3129289	15.2887088	33.6792361	25.1089028
Coa4	2.28782486	-0.3392517	0.40810257	-0.8312902	0.40580971	NA	Coa4	2.68818531	2.26261859	1.90847421	6.20442003	0	2.35210905	2.88679167	0
Sntb2	6.48197922	0.33097403	0.39820504	0.83116485	0.40588051	0.99938111	Sntb2	4.03227797	7.69290322	4.77118552	4.13628002	6.65924688	3.52816357	9.62263889	11.4131377
Sox4	15.6130789	-0.3272399	0.39372075	-0.8311471	0.40589054	0.99938111	Sox4	22.1775288	15.8383302	10.8146872	22.7495401	8.3240586	8.23238167	4.81131944	31.9567854
Gas2l3	2.40299335	0.3410666	0.41047697	0.83090313	0.40602836	NA	Gas2l3	1.34409266	2.26261859	2.54463228	0	1.66481172	5.88027262	0.96226389	4.56525506
Nup43	3.5479026	0.34555152	0.41594691	0.8307587	0.40610996	NA	Nup43	3.36023164	3.16766603	2.54463228	2.06814001	1.66481172	2.35210905	8.660375	4.56525506
Tle3	41.8546514	0.27346116	0.32918532	0.83072102	0.40613125	0.99938111	Tle3	37.6345944	38.0119924	27.990955	43.4309402	73.2517157	28.2253086	26.9433889	59.3483158
Cyth1	31.4084505	0.24445388	0.29434711	0.83049524	0.40625884	0.99938111	Cyth1	38.978687	20.3635674	26.7186389	26.8858201	38.2906696	31.7534722	31.7547083	36.5220405
Kif19a	2.12701007	0.31036763	0.37383068	0.83023583	0.40640546	NA	Kif19a	1.34409266	1.81009488	1.59039517	0	9.98887032	0	0	2.28262753
Ddb1	145.091628	0.215719	0.25987697	0.83008125	0.40649285	0.99938111	Ddb1	138.441544	109.058216	140.272854	142.701661	209.766277	144.654706	100.075444	175.76232
Spred1	176.168533	-0.2520691	0.30391937	-0.8293946	0.40688113	0.99938111	Spred1	170.027721	219.474004	196.572843	194.405161	89.8998329	206.985596	233.830125	98.1529838
Slc25a28	14.0575542	-0.3207611	0.38676054	-0.8293533	0.4069045	0.99938111	Slc25a28	16.8011582	12.2181404	9.54237104	26.8858201	13.3184938	3.52816357	9.62263889	20.5436478
Taf5l	17.0843914	0.27773026	0.33494614	0.82917885	0.40700321	0.99938111	Taf5l	11.4247876	13.5757116	16.2220308	18.6132601	14.9833055	12.9365998	19.2452778	29.6741579
Recql	9.51411747	0.31200433	0.37634006	0.82904893	0.40707672	0.99938111	Recql	6.04841696	7.2403795	11.4508452	8.27256004	9.98887032	14.1126543	14.4339583	4.56525506
Lin7b	14.2624011	-0.3055265	0.36857966	-0.8289293	0.40714443	0.99938111	Lin7b	19.4893435	18.5534725	18.448584	6.20442003	6.65924688	9.40843619	12.5094306	22.8262753
Rab10os	7.42386688	0.33260069	0.40127305	0.82886377	0.4071815	0.99938111	Rab10os	5.37637063	4.52523719	4.45310648	10.3407001	4.99443516	4.7042181	6.73584722	18.2610202
2900011O08Rik	228.653966	-0.154678	0.18662706	-0.8288082	0.40721295	0.99938111	2900011O08R	240.592586	229.882049	252.872832	244.040521	208.101465	189.344778	252.113139	212.28436
Nqo2	6.25028838	-0.3326311	0.40136012	-0.8287596	0.40724044	0.99938111	Nqo2	6.72046328	7.69290322	8.2700549	8.27256004	3.32962344	7.05632714	8.660375	0
Nbn	12.2711669	0.34365261	0.41468773	0.82870215	0.40727297	0.99938111	Nbn	11.4247876	14.480759	10.4966081	0	4.99443516	21.1689814	35.6037639	0
Fcho1	47.4738778	0.28403324	0.34274549	0.82870015	0.4072741	0.99938111	Fcho1	34.9464091	42.9897533	30.5355873	57.9079203	88.2350212	35.2816357	25.981125	63.9135709
Rev3l	50.8498292	0.3299414	0.39823883	0.82850131	0.40738665	0.99938111	Rev3l	44.3550577	54.3028463	60.1169375	8.27256004	29.966611	109.373071	79.8679027	20.5436478
Plvap	18.06354	-0.3322817	0.40141379	-0.8277785	0.40779596	0.99938111	Plvap	13.4409266	17.648425	11.1327662	43.4309402	18.3129289	3.52816357	9.62263889	27.3915304
Ermap	1.38436401	0.27619823	0.33366523	0.82777047	0.4078005	NA	Ermap	1.34409266	0.45252372	0.9542371	0	8.3240586	0	0	0
Crygn	0.70831019	0.2134581	0.25795878	0.82748917	0.40795986	NA	Crygn	0.67204633	0	0	0	4.99443516	0	0	0
Nrxn2	506.669616	0.30259999	0.36570726	0.82743776	0.40798899	0.99938111	Nrxn2	340.055442	505.01647	301.857004	593.556183	894.003894	376.337448	175.132028	867.398462
Macrod2	15.6325741	0.29590835	0.35770085	0.82725091	0.40809486	0.99938111	Macrod2	16.8011582	12.2181404	17.8124259	6.20442003	8.3240586	22.345036	23.0943333	18.2610202
Rdh13	11.4641189	0.30563827	0.36946893	0.82723673	0.4081029	0.99938111	Rdh13	11.4247876	8.14542694	6.99773876	14.4769801	19.9777406	9.40843619	14.4339583	6.84788259
Itga7	1.42998543	-0.3076757	0.3719339	-0.8272322	0.40810546	NA	Itga7	1.34409266	1.35757116	1.27231614	4.13628002	3.32962344	0	0	0
Fam78b	32.1649604	-0.2676538	0.32370039	-0.8268567	0.40831831	0.99938111	Fam78b	44.3550577	32.5817078	41.9864326	24.8176801	36.6258578	27.0492541	38.4905555	11.4131377
Ptdss2	21.7936279	-0.2993223	0.36201071	-0.8268328	0.40833186	0.99938111	Ptdss2	17.4732045	35.7493738	23.8559276	20.6814001	19.9777406	27.0492541	6.73584722	22.8262753
Irs2	81.8367377	0.29722344	0.35950934	0.82674746	0.40838021	0.99938111	Irs2	57.1239379	57.4705123	53.1191988	115.815841	146.503431	47.042181	45.2264028	132.392397
Atp6v0a4	0.37298182	-0.2257611	0.27308547	-0.8267048	0.40840438	NA	Atp6v0a4	0.67204633	1.35757116	0.9542371	0	0	0	0	0
Cd59b	0.37298182	-0.2257611	0.27308547	-0.8267048	0.40840438	NA	Cd59b	0.67204633	1.35757116	0.9542371	0	0	0	0	0
Irak4	0.37298182	-0.2257611	0.27308547	-0.8267048	0.40840438	NA	Irak4	0.67204633	1.35757116	0.9542371	0	0	0	0	0
Odf3b	0.37298182	-0.2257611	0.27308547	-0.8267048	0.40840438	NA	Odf3b	0.67204633	1.35757116	0.9542371	0	0	0	0	0
Trim21	0.37298182	-0.2257611	0.27308547	-0.8267048	0.40840438	NA	Trim21	0.67204633	1.35757116	0.9542371	0	0	0	0	0
3632451O06Rik	11.3893626	-0.3330612	0.40294006	-0.8265775	0.40847657	0.99938111	3632451O06R	8.73660227	22.6261859	14.3135566	8.27256004	1.66481172	19.9929269	8.660375	6.84788259
2010107E04Rik	27.4783842	-0.333634	0.40379272	-0.8262507	0.40866191	0.99938111	2010107E04Ri	38.978687	37.106945	42.3045116	12.4088401	6.65924688	16.4647633	56.7735694	9.13051012
Oaz2	108.262163	0.26249546	0.31771079	0.82620882	0.40868565	0.99938111	Oaz2	87.3660227	71.9512713	69.0231505	159.246781	128.190502	72.9153805	103.9245	173.479692
Per2	22.3368135	-0.2688688	0.32553332	-0.8259333	0.40884192	0.99938111	Per2	29.5700384	20.3635674	18.766663	31.0221002	26.6369875	14.1126543	15.3962222	22.8262753
Gm16796	0.85374892	0.2604959	0.31560531	0.82538503	0.40915303	NA	Gm16796	0	0.45252372	0.63615807	0	0	1.17605452	0	4.56525506
Hsf2	16.3213047	0.30958754	0.37514976	0.8252372	0.40923694	0.99938111	Hsf2	16.1291119	16.7433776	16.5401098	6.20442003	11.653682	24.697145	31.7547083	6.84788259
Map7d2	170.341625	0.21552247	0.26117126	0.82521509	0.40924949	0.99938111	Map7d2	184.140694	144.355066	179.714654	115.815841	136.514561	232.858796	223.245222	146.088162
Hk2	3.75824275	-0.3376079	0.40917701	-0.82509	0.40932048	NA	Hk2	3.36023164	1.81009488	1.59039517	14.4769801	1.66481172	2.35210905	4.81131944	0
Hist1h2ai	2.26345933	-0.3371675	0.40872766	-0.8249197	0.4094172	NA	Hist1h2ai	3.36023164	1.81009488	2.22655324	4.13628002	3.32962344	0	0.96226389	2.28262753
Polr3k	33.3839916	0.29298178	0.35523141	0.82476317	0.40950606	0.99938111	Polr3k	29.5700384	25.3413283	39.1237212	22.7495401	19.9777406	44.6900719	67.3584722	18.2610202
Map4k4	55.8412765	-0.2099509	0.25458467	-0.8246802	0.40955318	0.99938111	Map4k4	66.5325865	60.6381783	51.8468826	64.1123403	63.2628454	57.6266717	46.1886666	36.5220405
Bckdk	34.7001877	-0.2568179	0.31144697	-0.8245958	0.40960112	0.99938111	Bckdk	36.9625481	33.9392789	25.4463228	59.9760603	33.2962344	22.345036	33.6792361	31.9567854
Ywhae	717.418073	-0.2105746	0.25537282	-0.8245773	0.40961161	0.99938111	Ywhae	858.203161	761.597419	809.193064	676.281783	459.488035	691.52006	960.339361	522.721705
Alkbh2	1.11892329	-0.3080913	0.37364685	-0.824552	0.409626	NA	Alkbh2	0.67204633	1.35757116	2.22655324	2.06814001	1.66481172	0	0.96226389	0
Tob1	30.7642932	-0.2493705	0.30244004	-0.8245288	0.40963916	0.99938111	Tob1	36.2905017	44.7998482	29.5813502	22.7495401	26.6369875	32.9295267	21.1698055	31.9567854
Pld1	3.71963585	-0.3413965	0.41413283	-0.8243647	0.40973238	NA	Pld1	4.03227797	9.5029981	4.13502745	0	0	2.35210905	2.88679167	6.84788259
Nol10	13.2470081	0.28839718	0.34984909	0.82434738	0.4097422	0.99938111	Nol10	15.4570656	9.95552182	12.0870033	8.27256004	11.653682	22.345036	12.5094306	13.6957652
B4galnt4	41.6379396	0.23701382	0.28752034	0.82433758	0.40974777	0.99938111	B4galnt4	28.8979921	51.1351802	36.2610099	33.0902402	51.6091633	54.0985081	34.6415	43.3699231
Rmnd1	6.6367252	0.32817217	0.39815512	0.82423194	0.40980778	0.99938111	Rmnd1	6.04841696	7.2403795	6.67965972	0	9.98887032	8.23238167	5.77358333	9.13051012
Atg2b	73.1309154	0.23192541	0.281423	0.82411676	0.40987322	0.99938111	Atg2b	55.7798453	66.068463	61.7073327	82.7256004	123.196067	59.9787807	62.5471528	73.044081
Fzd1	11.6488136	0.32910813	0.3993538	0.82410165	0.4098818	0.99938111	Fzd1	4.7043243	12.6706641	9.54237104	10.3407001	23.3073641	8.23238167	3.84905555	20.5436478
Rybp	43.2319551	0.26862473	0.32596798	0.82408318	0.4098923	0.99938111	Rybp	32.2582238	51.587704	44.2129858	24.8176801	28.3017992	61.1548353	69.283	34.239413
Nr2c2ap	7.71922631	0.32727527	0.3971551	0.82404901	0.40991171	0.99938111	Nr2c2ap	5.37637063	4.07271347	4.77118552	12.4088401	9.98887032	4.7042181	6.73584722	13.6957652
Chaf1a	3.93709982	0.3427414	0.41597108	0.82395489	0.40996519	NA	Chaf1a	4.7043243	1.81009488	3.18079035	2.06814001	13.3184938	3.52816357	2.88679167	0
Lrrtm3	37.8995331	-0.313555	0.38055658	-0.8239378	0.40997488	0.99938111	Lrrtm3	40.3227797	66.5209867	47.7118552	20.6814001	21.6425524	63.5069443	33.6792361	9.13051012
Cep85l	10.4539856	0.30884469	0.37488601	0.82383624	0.41003261	0.99938111	Cep85l	13.4409266	10.8605693	6.67965972	4.13628002	16.6481172	10.5844907	14.4339583	6.84788259
Vezf1	39.1098003	-0.2678237	0.32511316	-0.823786	0.41006115	0.99938111	Vezf1	51.7475673	35.7493738	48.9841713	39.2946602	14.9833055	44.6900719	50.0377222	27.3915304
Zim1	9.6755298	0.32311138	0.3922324	0.82377533	0.41006723	0.99938111	Zim1	7.39250961	9.95552182	6.99773876	8.27256004	3.32962344	22.345036	7.69811111	11.4131377
Sema3e	12.5182379	0.33597217	0.407935	0.82359241	0.41017119	0.99938111	Sema3e	12.7688802	12.6706641	13.9954775	0	14.9833055	30.5774176	10.5849028	4.56525506
Rab11fip5	122.830008	0.3105013	0.37702994	0.82354548	0.41019787	0.99938111	Rab11fip5	82.6616984	59.2806072	72.5220199	204.745861	204.771842	63.5069443	76.0188472	219.132243
Entpd6	34.737033	0.26897528	0.3266158	0.82352195	0.41021124	0.99938111	Entpd6	22.8495752	38.0119924	34.3525357	24.8176801	49.9443516	32.9295267	20.2075417	54.7830607
Ccm2	25.5153531	-0.2866941	0.34816899	-0.8234338	0.41026137	0.99938111	Ccm2	30.9141311	21.7211385	24.4920857	39.2946602	21.6425524	8.23238167	32.7169722	25.1089028
Hook1	20.819679	0.29693328	0.36060853	0.82342278	0.41026761	0.99938111	Hook1	20.1613899	15.8383302	19.0847421	18.6132601	9.98887032	25.8731995	43.301875	13.6957652
4632428N05Rik	5.10586344	-0.3423117	0.41578491	-0.8232903	0.41034294	0.99938111	4632428N05R	5.37637063	14.9332827	4.13502745	0	4.99443516	5.88027262	0.96226389	4.56525506
Fut11	11.7621695	-0.2840463	0.34506245	-0.8231735	0.41040933	0.99938111	Fut11	12.0968339	13.5757116	13.0412404	14.4769801	8.3240586	10.5844907	10.5849028	11.4131377
Dpp9	68.6766129	0.27751397	0.3371562	0.8231021	0.41044994	0.99938111	Dpp9	64.5164475	52.4927514	50.8926455	72.3849004	103.218327	47.042181	35.6037639	123.261887
Atg16l1	31.1325475	0.25902388	0.31470026	0.82308124	0.4104618	0.99938111	Atg16l1	32.2582238	19.0059962	28.3090341	31.0221002	49.9443516	24.697145	25.0188611	38.804668
Zbtb25	7.21059945	0.3355151	0.40763412	0.82307903	0.41046306	0.99938111	Zbtb25	4.7043243	9.05047438	8.58813393	0	8.3240586	7.05632714	15.3962222	4.56525506
Fnbp1l	62.6759505	0.24471782	0.29733118	0.82304795	0.41048073	0.99938111	Fnbp1l	53.7637063	61.0907021	64.251965	47.5672202	41.620293	97.6125255	87.5660139	47.9351782
Zfp420	9.97907429	-0.340938	0.41424509	-0.8230346	0.41048832	0.99938111	Zfp420	14.1129729	14.9332827	15.9039517	4.13628002	1.66481172	11.7605452	17.32075	0
Hbs1l	19.6578146	-0.299384	0.36377864	-0.822984	0.41051707	0.99938111	Hbs1l	20.1613899	25.793852	26.4005599	18.6132601	11.653682	14.1126543	33.6792361	6.84788259
Tbcc	9.65689515	0.30150611	0.36640831	0.8228692	0.41058238	0.99938111	Tbcc	8.73660227	6.33533207	8.2700549	10.3407001	14.9833055	11.7605452	7.69811111	9.13051012
Jak3	3.95275928	0.34140344	0.41527134	0.82212137	0.41100783	NA	Jak3	4.7043243	3.16766603	1.59039517	2.06814001	13.3184938	3.52816357	0.96226389	2.28262753
Mtg1	7.88741716	0.31094673	0.378251	0.82206453	0.41104017	0.99938111	Mtg1	8.73660227	4.97776091	6.67965972	6.20442003	11.653682	7.05632714	8.660375	9.13051012
Tmed4	33.98812	-0.2521661	0.30676561	-0.8220155	0.41106805	0.99938111	Tmed4	25.5377605	54.3028463	38.4875632	31.0221002	33.2962344	35.2816357	28.8679167	25.1089028
Ica1	25.3135966	0.28000582	0.34066522	0.82193837	0.41111197	0.99938111	Ica1	30.2420848	16.2908539	27.990955	14.4769801	19.9777406	32.9295267	42.3396111	18.2610202
BC037032	14.8302472	0.33773258	0.41102575	0.8216823	0.41125773	0.99938111	BC037032	10.7527413	9.05047438	10.4966081	16.5451201	29.966611	4.7042181	2.88679167	34.239413
Wac	98.7930332	-0.2172947	0.26446225	-0.8216474	0.4112776	0.99938111	Wac	113.575829	120.371309	115.46269	78.5893204	56.6035985	107.020962	109.698083	89.0224737
1810062O18Rik	0.5281872	0.22847562	0.27811259	0.82152204	0.41134898	NA	1810062O18R	0	0	0.63615807	0	1.66481172	0	1.92452778	0
Gm17762	2.56919623	-0.3176985	0.38673254	-0.821494	0.41136493	NA	Gm17762	0.67204633	1.35757116	2.22655324	10.3407001	4.99443516	0	0.96226389	0
Rdm1	3.25684073	-0.336889	0.41010179	-0.8214766	0.41137485	NA	Rdm1	2.68818531	3.16766603	3.81694841	6.20442003	3.32962344	0	0	6.84788259
H3f3b	87.3232602	0.26309218	0.32033668	0.82129897	0.41147599	0.99938111	H3f3b	73.2530498	82.3593168	70.6135457	86.8618804	51.6091633	81.1477622	175.132028	77.6093361

GeneID	Base mean	log2(FC)	StdErr	Wald-Stats	P-value	P-adj		GeneID	Normalised expression for Chow#1	Normalised expression for Chow#2	Normalised expression for Chow#3	Normalised expression for Chow#4	Normalised expression for HFD#1	Normalised expression for HFD#2	Normalised expression for HFD#3	Normalised expression for HFD#4
Lmo4	105.668797	-0.2542083	0.30956057	-0.8211907	0.41153766	0.99938111		Lmo4	157.930887	126.254118	113.554215	68.2486203	99.8887032	56.4506172	138.566	84.4572186
Tmem200c	13.0497097	0.30328659	0.36934109	0.82115585	0.4115575	0.99938111		Tmem200c	8.06455594	13.5757116	11.4508452	10.3407001	21.6425524	15.2887088	5.77358333	18.2610202
Fh1	32.3348537	0.2463662	0.30005506	0.82106997	0.41160641	0.99938111		Fh1	39.6507334	21.7211385	32.1259825	22.7495401	29.966611	35.2816357	45.2264028	31.9567854
Slc22a4	3.17075604	-0.3417861	0.41629546	-0.8210181	0.41163594	NA		Slc22a4	4.03227797	4.97776091	2.54463228	4.13628002	3.32962344	1.17605452	2.88679167	2.28262753
Pgrmc2	47.561254	-0.2431355	0.29615639	-0.8209699	0.41166343	0.99938111		Pgrmc2	51.075521	63.8058444	54.0734359	39.2946602	23.3073641	59.9787807	43.301875	45.6525506
Ksr2	73.4601392	0.25305478	0.30829125	0.82083023	0.41174298	0.99938111		Ksr2	48.3873356	65.6159392	56.2999891	93.0663005	101.553515	57.6266717	50.9999861	114.131377
Ppifos	0.67705717	0.2540791	0.3095952	0.82068164	0.41182763	NA		Ppifos	0.67204633	0.45252372	0	0	3.32962344	0	0.96226389	0
Hipk3	112.536499	0.24326701	0.29657318	0.82025964	0.41206811	0.99938111		Hipk3	112.903783	115.846072	114.190373	62.0442003	113.207197	177.584233	133.754681	70.7614535
Dhx15	46.2940492	0.23827292	0.29050677	0.82019747	0.41210355	0.99938111		Dhx15	47.7152893	43.442277	49.6203294	24.8176801	33.2962344	63.5069443	57.7358333	50.2178057
4930432K21Rik	2.42070197	0.32691605	0.39880198	0.8197453	0.41236132	NA		4930432K21Ri	2.68818531	1.35757116	0.31807903	2.06814001	1.66481172	1.17605452	0.96226389	9.13051012
Bmp2k	15.8780572	-0.2749896	0.33550245	-0.8196352	0.41242413	0.99938111		Bmp2k	16.8011582	14.9332827	18.130505	22.7495401	19.9777406	14.1126543	13.4716944	6.84788259
Caly	92.8975321	-0.2067693	0.25230252	-0.8195294	0.41248442	0.99938111		Caly	96.7746713	128.064212	97.6502636	74.4530404	76.5813391	84.6759257	73.1320555	111.848749
Fdps	3.71184902	-0.3406003	0.41564231	-0.8194554	0.41252663	NA		Fdps	4.7043243	5.43028463	2.86271131	6.20442003	3.32962344	2.35210905	4.81131944	0
Prx	1.21321308	0.28987175	0.35379068	0.81933123	0.41259746	NA		Prx	2.01613899	0.45252372	0.31807903	0	4.99443516	0	1.92452778	0
Slc6a12	0.65312023	-0.2286646	0.279122	-0.819228	0.41265632	NA		Slc6a12	0	0.45252372	0.63615807	4.13628002	0	0	0	0
Hbb-b2	2.10641339	0.32234265	0.39354742	0.81906939	0.41274683	NA		Hbb-b2	1.34409266	1.81009488	2.22655324	0	6.65924688	0	4.81131944	0
Hbb-bt	2.10641339	0.32234265	0.39354742	0.81906939	0.41274683	NA		Hbb-bt	1.34409266	1.81009488	2.22655324	0	6.65924688	0	4.81131944	0
Med29	10.9056645	-0.3401902	0.41544088	-0.8188654	0.4128632	0.99938111		Med29	12.7688802	3.62018975	5.40734359	33.0902402	8.3240586	0	5.77358333	18.2610202
Hapln1	26.1854595	0.33573434	0.41006251	0.81873941	0.41293511	0.99938111		Hapln1	21.5054825	37.5594687	20.6751372	4.13628002	9.98887032	67.0351079	39.4528194	9.13051012
C130046K22Rik	3.14675493	-0.3400672	0.41537032	-0.8187084	0.41295278	NA		C130046K22Ri	6.72046328	1.81009488	4.77118552	2.06814001	0	2.35210905	2.88679167	4.56525506
Polr2e	22.2903449	-0.2867441	0.35025561	-0.8186711	0.4129741	0.99938111		Polr2e	34.9464091	18.5534725	18.766663	28.9539601	23.3073641	8.23238167	25.0188611	20.5436478
Wbscr17	33.4738496	-0.2494973	0.30479867	-0.8185644	0.413035	0.99938111		Wbscr17	26.2098068	48.8725616	35.6248519	35.1583802	38.2906696	28.2253086	21.1698055	34.239413
Fbll1	40.5671521	0.31229263	0.38159032	0.81839767	0.41313015	0.99938111		Fbll1	24.8657142	16.7433776	21.6293743	76.5211804	51.6091633	27.0492541	30.7924444	75.3267085
Rhox8	0.94945737	0.23387061	0.28582452	0.81823142	0.41322506	NA		Rhox8	0	0	0	2.06814001	0	0	0.96226389	4.56525506
Rusc2	132.066675	0.28452807	0.34774298	0.81821371	0.41323517	0.99938111		Rusc2	104.167181	104.532979	97.6502636	155.110501	279.688369	81.1477622	72.1697916	162.066555
Sep-10	1.66969163	-0.3261013	0.39857733	-0.8181631	0.41326405	NA		Sep-10	2.68818531	1.35757116	3.18079035	2.06814001	0	1.17605452	2.88679167	0
Zfand5	72.025985	0.29265481	0.35769754	0.81816276	0.41326426	0.99938111		Zfand5	67.8766792	74.2138899	83.336707	22.7495401	36.6258578	82.3238167	133.754681	75.3267085
2610015P09Rik	1.21669583	0.29847632	0.36482161	0.81814319	0.41327543	NA		2610015P09Ri	2.01613899	0.45252372	0.63615807	0	0	4.7042181	1.92452778	0
Camk1d	122.03204	0.23856009	0.29161864	0.81805504	0.41332576	0.99938111		Camk1d	118.280154	133.494497	125.641219	62.0442003	159.821925	154.063143	145.301847	77.6093361
Psma5	5.34585235	-0.3351887	0.4097485	-0.8180353	0.41333702	0.99938111		Psma5	3.36023164	4.07271347	8.2700549	10.3407001	4.99443516	2.35210905	4.81131944	4.56525506
Mybpc1	0.95500317	-0.2957947	0.3615947	-0.8180284	0.41334099	NA		Mybpc1	2.68818531	0.90504744	1.90847421	0	0	1.17605452	0.96226389	0
Selenbp1	1.66853658	-0.3010716	0.36809236	-0.817924	0.41340058	NA		Selenbp1	4.03227797	0	1.59039517	4.13628002	1.66481172	0	1.92452778	0
Dner	183.131824	0.28649811	0.35028915	0.81789033	0.41341981	0.99938111		Dner	100.806949	258.391044	182.259287	95.1344405	136.514561	349.288194	121.24525	221.414871
Cramp1l	41.1417852	0.25015469	0.30588505	0.8178062	0.41346786	0.99938111		Cramp1l	36.9625481	33.4867552	30.2175083	47.5672202	69.9220923	36.4576902	28.8679167	45.6525506
F730043M19Rik	5.42889262	-0.3400914	0.41586445	-0.8177938	0.41347496	0.99938111		F730043M19R	7.39250961	10.8605693	8.2700549	0	3.32962344	5.88027262	7.69811111	0
Kdsr	17.3588089	-0.3038958	0.37172058	-0.8175384	0.41362082	0.99938111		Kdsr	15.4570656	27.1514231	24.1740066	12.4088401	14.9833055	28.2253086	9.62263889	6.84788259
1700012D14Rik	1.93437586	0.27217309	0.33296521	0.817422	0.41368731	NA		1700012D14Ri	0	1.35757116	0.63615807	2.06814001	0	0	0	11.4131377
Man1c1	41.4721928	0.23028096	0.28175657	0.81730466	0.41375434	0.99938111		Man1c1	29.5700384	45.2523719	33.3982986	41.3628002	46.6147282	43.5140174	32.7169722	59.3483158
Mad1l1	6.36804942	0.3345998	0.40941679	0.81725959	0.41378009	0.99938111		Mad1l1	7.39250961	1.81009488	6.67965972	4.13628002	8.3240586	10.5844907	2.88679167	9.13051012
Rnf126	56.1877776	-0.2938993	0.35974608	-0.8169633	0.41394943	0.99938111		Rnf126	52.4196136	59.7331309	44.8491439	97.2025805	63.2628454	23.5210905	24.0565972	84.4572186
Pcyt1a	30.2768078	0.24337263	0.29790497	0.81694721	0.4139586	0.99938111		Pcyt1a	20.8334362	25.3413283	26.4005599	39.2946602	41.620293	27.0492541	36.5660278	25.1089028
Kif21a	176.609623	0.27886548	0.34135543	0.81693584	0.41396509	0.99938111		Kif21a	181.452509	158.835825	180.668892	99.2707205	148.168243	359.872684	200.150889	84.4572186
Slc26a5	0.61147649	0.26834683	0.32849862	0.81688877	0.413992	NA		Slc26a5	0	0.45252372	0.63615807	0	1.66481172	1.17605452	0.96226389	0
Hivep1	61.4769731	0.25376995	0.31066834	0.81685166	0.41401321	0.99938111		Hivep1	67.8766792	51.587704	54.0734359	47.5672202	51.6091633	116.429398	61.5848889	41.0872956
Dnm2	22.1637961	-0.3125799	0.38270425	-0.8167663	0.41406198	0.99938111		Dnm2	34.9464091	18.5534725	19.4028211	28.9539601	41.620293	8.23238167	9.62263889	15.9783927
Irf9	2.24878147	-0.3290896	0.4029488	-0.8167033	0.41409801	NA		Irf9	3.36023164	1.81009488	1.59039517	6.20442003	0	1.17605452	3.84905555	0
Cpt1c	50.4947838	0.28649922	0.35082237	0.81665036	0.41412826	0.99938111		Cpt1c	45.6991503	52.0402277	33.7163777	43.4309402	74.9165274	67.0351079	16.3584861	70.7614535
Klf16	74.8193075	0.32414304	0.39691908	0.81664768	0.4141298	0.99938111		Klf16	60.4841696	40.274611	42.3045116	105.475141	149.833055	17.6408179	43.301875	139.240279
Zfp560	3.46768776	-0.340005	0.41636071	-0.8166115	0.41415047	NA		Zfp560	4.7043243	5.88280835	6.04350166	0	1.66481172	2.35210905	4.81131944	2.28262753
Rraga	89.1152197	0.18916511	0.23166613	0.81654194	0.41419024	0.99938111		Rraga	79.9735131	70.1411764	78.5655215	105.475141	83.240586	84.6759257	105.849028	105.000866
Gm10408	1.02682784	0.26917457	0.32968766	0.81645329	0.41424093	NA		Gm10408	0	0.45252372	1.27231614	0	0	0	1.92452778	4.56525506
Pitpnb	52.0923655	0.20050027	0.24557962	0.81643693	0.41425028	0.99938111		Pitpnb	44.3550577	45.2523719	50.5745665	53.7716403	46.6147282	51.7463991	67.3584722	57.0656883
Polr3h	24.6227373	-0.2878176	0.35260714	-0.8162556	0.41435396	0.99938111		Polr3h	26.8818531	16.2908539	18.766663	51.7035003	18.3129289	16.4647633	21.1698055	27.3915304
2900092D14Rik	16.0814946	0.32083148	0.39323306	0.81588124	0.41456806	0.99938111		2900092D14Ri	10.0806949	7.2403795	9.224292	28.9539601	28.3017992	15.2887088	6.73584722	22.8262753
Mrpl39	23.8196453	-0.286707	0.3514384	-0.8158102	0.41460869	0.99938111		Mrpl39	36.9625481	23.9837571	27.0367179	20.6814001	8.3240586	32.9295267	26.9433889	13.6957652
B130034C11Rik	1.30940332	0.31111376	0.38138454	0.8157482	0.41464416	NA		B130034C11Ri	0.67204633	0.90504744	1.59039517	0	0	1.17605452	3.84905555	2.28262753
Mrpl52	12.7288716	0.30090408	0.36887738	0.8157293	0.41465498	0.99938111		Mrpl52	11.4247876	8.59795066	9.224292	14.4769801	6.65924688	10.5844907	13.4716944	27.3915304
Rtfdc1	28.3903425	0.26623431	0.32639477	0.81568192	0.41468208	0.99938111		Rtfdc1	24.8657142	16.2908539	23.2197695	37.2265202	39.9554813	22.345036	22.1320694	41.0872956
Mcrs1	9.09460877	-0.3151606	0.38638936	-0.8156554	0.41469723	0.99938111		Mcrs1	9.4086486	7.69290322	7.31581779	18.6132601	6.65924688	5.88027262	5.77358333	11.4131377
Cep128	2.76383378	-0.3376078	0.41405849	-0.8153625	0.41486485	NA		Cep128	4.7043243	4.97776091	4.13502745	0	1.66481172	4.7042181	1.92452778	0
Grin2a	58.86655	0.29778967	0.36523393	0.81533957	0.41487797	0.99938111		Grin2a	47.043243	76.4765085	62.6615698	14.4769801	76.5813391	107.020962	43.301875	43.3699231
Usp30	24.5549395	-0.2556622	0.31360171	-0.815245	0.41493208	0.99938111		Usp30	22.1775288	25.3413283	21.6293743	41.3628002	19.9777406	23.5210905	17.32075	25.1089028
Taf4a	10.6310364	0.29034619	0.35635172	0.81477419	0.41520158	0.99938111		Taf4a	10.0806949	9.5029981	9.54237104	6.20442003	13.3184938	11.7605452	8.660375	15.9783927
Atp8b2	39.0818235	0.25195043	0.30923227	0.81476114	0.41520905	0.99938111		Atp8b2	26.2098068	40.274611	30.2175083	43.4309402	53.2739751	48.2182355	23.0943333	47.9351782
Rbl2	50.7127826	-0.2500061	0.3068553	-0.8147361	0.41522336	0.99938111		Rbl2	62.5003085	62.4482732	62.3434908	37.2265202	38.2906696	62.3308898	57.7358333	22.8262753
Mcat	11.1245725	0.3090785	0.37936334	0.8147295	0.41522716	0.99938111		Mcat	8.06455594	5.88280835	9.54237104	14.4769801	14.9833055	5.88027262	9.62263889	20.5436478
Adrb1	13.4036532	-0.3038226	0.37292108	-0.8147102	0.4152382	0.99938111		Adrb1	14.1129729	17.1959013	9.224292	20.6814001	18.3129289	8.23238167	5.77358333	13.6957652
B3galnt1	26.5216068	-0.3114225	0.38231282	-0.814575	0.41531564	0.99938111		B3galnt1	27.5538995	46.1574193	37.8514051	10.3407001	8.3240586	39.9858538	25.981125	15.9783927
Kdm2b	17.7670395	0.27255146	0.33472082	0.81426503	0.41549314	0.99938111		Kdm2b	19.4893435	11.7656167	17.1762679	14.4769801	29.966611	17.6408179	20.2075417	11.4131377
B4galt6	229.776332	0.21938192	0.26947883	0.81409705	0.41558935	0.99938111		B4galt6	159.27498	278.754611	229.016905	171.655621	183.129289	345.76003	230.943333	239.675891
Twf1	22.2540678	-0.2804972	0.34456038	-0.8140727	0.4156033	0.99938111		Twf1	24.1936678	32.129184	26.0824808	18.6132601	13.3184938	17.6408179	34.6415	11.4131377
Senp8	9.60075006	-0.31906	0.39193787	-0.8140576	0.41561198	0.99938111		Senp8	14.1129729	6.33533207	10.4966081	14.4769801	3.32962344	14.1126543	4.81131944	9.13051012
Il17d	10.1693486	0.3355054	0.4122778	0.81378479	0.41576825	0.99938111		Il17d	8.06455594	5.43028463	6.04350166	12.4088401	13.3184938	3.52816357	2.88679167	29.6741579
Pabpc4	8.5850201	-0.3204884	0.39385277	-0.8137264	0.41580169	0.99938111		Pabpc4	8.06455594	6.78785578	8.58813393	16.5451201	6.65924688	3.52816357	4.81131944	13.6957652
Grin2b	257.043285	0.26016321	0.31975386	0.81363586	0.41585359	0.99938111		Grin2b	196.909574	343.918026	234.106169	138.565381	319.64385	432.788065	171.282972	219.132243
Ghdc	1.07978031	-0.2946253	0.36211989	-0.8136127	0.41586687	NA		Ghdc	0.67204633	2.26261859	2.86271131	0	1.66481172	1.17605452	0	0
Nhlrc2	5.21111601	-0.3307316	0.40653124	-0.8135455	0.41590539	0.99938111		Nhlrc2	8.06455594	8.14542694	5.72542262	2.06814001	4.99443516	2.35210905	5.77358333	4.56525506
Ptchd4	8.87325346	0.33869509	0.41636914	0.81344908	0.41596063	0.99938111		Ptchd4	5.37637063	11.313093	9.86045007	0	1.66481172	28.2253086	7.69811111	6.84788259
4930404I05Rik	0.86505692	0.25760532	0.31670774	0.81338497	0.41599737	NA		4930404I05Ri	0.67204633	0	0.63615807	0	3.32962344	0	0	2.28262753
Upf3a	12.9274083	0.29849069	0.36698446	0.81336056	0.41601137	0.99938111		Upf3a	12.7688802	10.8605693	11.7689243	10.3407001	14.9833055	21.1689814	19.2452778	2.28262753
Clcn2	9.83036925	0.3364761	0.41372069	0.8132929	0.41605015	0.99938111		Clcn2	5.37637063	7.2403795	7.95197586	10.3407001	28.3017992	7.05632714	0.96226389	11.4131377
2610100L16Rik	10.2136953	-0.3164129	0.38908256	-0.8132283	0.41608719	0.99938111		2610100L16Ri	13.4409266	14.0282353	13.0412404	4.13628002	8.3240586	4.7042181	5.77358333	18.2610202
Zfp655	28.3218572	0.30755218	0.37834701	0.81288386	0.41628465	0.99938111		Zfp655	24.1936678	28.961518	30.5355873	12.4088401	8.3240586	54.0985081	45.2264028	22.8262753
Gfod1	95.9648915	0.18569962	0.22844707	0.81287808	0.41628797	0.99938111		Gfod1	90.054208	100.007742	94.7875523	68.2486203	123.196067	101.140689	87.5660139	102.718239
5730559C18Rik	2.74972908	-0.337471	0.41523661	-0.8127198	0.41637872	NA		5730559C18Ri	6.04841696	2.26261859	3.18079035	2.06814001	1.66481172	3.52816357	0.96226389	2.28262753
Slc6a20a	3.23954978	-0.3327993	0.40950742	-0.8126819	0.41640048	NA		Slc6a20a	2.01613899	7.69290322	6.04350166	0	4.99443516	0	2.88679167	2.28262753
Midn	74.3277941	-0.2826648	0.34792129	-0.8124388	0.41653988	0.99938111		Midn	92.070347	61.9957495	63.6158069	115.815841	78.2461509	34.1055812	34.6415	114.131377
Mcemp1	0.44102045	0.16604736	0.2044057	0.81234213	0.41659534	NA		Mcemp1	0	0	0	0	0	3.52816357	0	0
Ptgdr2	0.44102045	0.16604736	0.2044057	0.81234213	0.41659534	NA		Ptgdr2	0	0	0	0	0	3.52816357	0	0
Acox3	17.7668906	0.28869055	0.35541223	0.8122696	0.41663695	0.99938111		Acox3	16.1291119	12.2181404	8.58813393	26.8858201	18.3129289	15.2887088	17.32075	27.3915304
AI462493	5.12404712	-0.3363741	0.41425376	-0.8120001	0.41679155	0.99938111		AI462493	5.37637063	4.52523719	3.18079035	12.4088401	6.65924688	2.35210905	1.92452778	4.56525506
Lonp2	42.5881451	0.22289559	0.27462364	0.81164019	0.41699812	0.99938111		Lonp2	49.059382	34.3918026	32.1259825	41.3628002	38.2906696	58.8027262	43.301875	43.3699231
Hip1	25.3256629	-0.3015745	0.37164072	-0.8114679	0.41709702	0.99938111		Hip1	30.2420848	47.0624668	24.1740066	12.4088401	34.9610461	25.8731995	9.62263889	18.2610202
Adam12	1.3759084	-0.3107018	0.38289369	-0.8114571	0.41710322	NA		Adam12	0.67204633	2.71514231	3.81694841	0	1.66481172	1.17605452	0.96226389	0
Tmem136	15.751041	-0.3086821	0.3804636	-0.8113315	0.41717533	0.99938111		Tmem136	7.39250961	19.0059962	12.4050823	35.1583802	11.653682	10.5844907	11.5471667	18.2610202
Abcc10	4.51841604	0.33772552	0.41629212	0.81127051	0.41721034	NA		Abcc10	1.34409266	2.71514231	4.77118552	4.13628002	4.99443516	3.52816357	0.96226389	13.6957652
Adamts16	0.84331412	0.25924845	0.31963552	0.81107522	0.41732248	NA		Adamts16	0.67204633	0.90504744	0	0	0	0	2.88679167	2.28262753
Dek	40.8973514	0.3257032	0.40159621	0.8110216	0.41735327	0.99938111		Dek	34.9464091	46.1574193	44.8491439	8.27256004	13.3184938	81.1477622	77.943375	20.5436478
AW551984	38.194677	0.26592012	0.32792666	0.81091339	0.41741541	0.99938111		AW551984	26.2098068	23.5312334	26.0824808	64.1123403	38.2906696	52.9224536	35.6037639	38.804668
4930515G01Rik	0.47860852	-0.2423731	0.2990871	-0.8103762	0.41772402	NA		4930515G01R	0.67204633	0.45252372	0.63615807	2.06814001	0	0	0	0
Tmem37	0.47860852	-0.2423731	0.2990871	-0.8103762	0.41772402	NA		Tmem37	0.67204633	0.45252372	0.63615807	2.06814001	0	0	0	0
Astn2	31.7373157	0.2684015	0.33120971	0.81036726	0.41772913	0.99938111		Astn2	24.8657142	29.8665654	32.7621406	22.7495401	29.966611	49.39429	16.3584861	47.9351782
Ctps	15.5706896	0.31092764	0.38370902	0.81032142	0.41775547	0.99938111		Ctps	9.4086486	7.69290322	10.4966081	26.8858201	18.3129289	7.05632714	17.32075	27.3915304
Katnal1	95.9167423	0.17024572	0.2101308	0.81018929	0.41783139	0.99938111		Katnal1	94.086486	87.3370778	94.7875523	82.7256004	86.5702095	102.316744	114.509403	105.000866
Ddx46	42.3887057	0.28198161	0.34808086	0.81010376	0.41788054	0.99938111		Ddx46	40.994826	32.129184	34.6706148	43.4309402	31.6314227	62.3308898	77.943375	15.9783927
Rpf2	5.04983106	-0.335471	0.41415352	-0.810016	0.41793101	0.99938111		Rpf2	10.7527413	5.43028463	0.9542371	8.27256004	3.32962344	0	4.81131944	6.84788259
Terf1	4.36022006	0.3334546	0.41178717	0.80977414	0.41807	NA		Terf1	5.37637063	3.16766603	3.49886938	2.06814001	1.66481172	5.88027262	8.660375	4.56525506
Dnajc16	45.8925661	0.26504916	0.32734864	0.8096846	0.41812147	0.99938111		Dnajc16	29.5700384	50.6826565	40.3960374	41.3628002	59.9332219	70.5632714	22.1320694	52.5004332
Ccdc13	1.38750329	0.30459826	0.3762195	0.80962913	0.41815336	NA		Ccdc13	0	1.81009488	0.31807903	2.06814001	1.66481172	2.35210905	2.88679167	0
Ash1l	193.345241	0.27079076	0.33451521	0.80950207	0.41822641	0.99938111		Ash1l	190.861157	212.233624	204.20674	74.4530404	168.145984	297.541795	282.905583	116.414004
Metap2	39.2631349	0.21790744	0.26919941	0.80946478	0.41824785	0.99938111		Metap2	33.6023164	33.9392789	30.2175083	47.5672202	36.6258578	41.1619083	38.4905555	52.5004332
Nup50	33.457221	0.21430021	0.26475754	0.80942062	0.41827324	0.99938111		Nup50	30.9141311	33.4867552	30.5355873	26.8858201	33.2962344	42.3379629	33.6792361	36.5220405

GeneID	Base mean	log2(FC)	StdErr	Wald-Stats	P-value	P-adj	GeneID	Normalised expression for Chow#1	Normalised expression for Chow#2	Normalised expression for Chow#3	Normalised expression for Chow#4	Normalised expression for HFD#1	Normalised expression for HFD#2	Normalised expression for HFD#3	Normalised expression for HFD#4
Wtap	26.9592948	-0.2679478	0.33105473	-0.8093761	0.41829885	0.99938111	Wtap	39.6507334	26.2463757	37.5333261	16.5451201	18.3129289	23.5210905	35.6037639	18.2610202
Mthfsd	10.5417103	0.31828293	0.39331411	0.80923345	0.41838088	0.99938111	Mthfsd	8.73660227	6.78785578	7.31581779	12.4088401	23.3073641	8.23238167	3.84905555	13.6957652
Cml1	3.27878404	-0.336547	0.4158909	-0.8092194	0.41838897	NA	Cml1	3.36023164	4.97776091	2.22655324	6.20442003	3.32962344	0	3.84905555	2.28262753
Agbl5	12.467413	0.29097658	0.35962217	0.80911746	0.41844758	0.99938111	Agbl5	10.0806949	6.78785578	11.1327662	16.5451201	9.98887032	10.5844907	16.3584861	18.2610202
Cox19	13.0283628	-0.302078	0.3734318	-0.808924	0.41855885	0.99938111	Cox19	12.7688802	10.8605693	15.2677937	22.7495401	8.3240586	5.88027262	19.2452778	9.13051012
Lurap1l	16.2299906	0.30511151	0.37720757	0.808869	0.4185905	0.99938111	Lurap1l	10.0806949	11.7656167	10.1785291	24.8176801	8.3240586	10.5844907	22.1320694	31.9567854
Rnaseh1	2.18594556	-0.3297709	0.40783167	-0.8085955	0.41874785	NA	Rnaseh1	3.36023164	1.35757116	3.18079035	4.13628002	0	3.52816357	1.92452778	0
Col9a2	2.26739414	0.33125737	0.40967431	0.80858711	0.41875268	NA	Col9a2	2.68818531	0.45252372	1.59039517	2.06814001	4.99443516	1.17605452	2.88679167	2.28262753
E130309D14Rik	27.0226417	-0.2644725	0.32710041	-0.808536	0.41878207	0.99938111	E130309D14Ri	27.5538995	32.5817078	27.990955	31.0221002	39.9554813	11.7605452	20.2075417	25.1089028
Itgav	46.2604972	-0.3002095	0.37130825	-0.8085183	0.4187923	0.99938111	Itgav	47.043243	86.4320303	55.9819101	20.6814001	54.9387868	62.3308898	22.1320694	20.5436478
Tm4sf1	0.8227952	-0.2798604	0.3461472	-0.8085012	0.41880214	NA	Tm4sf1	0.67204633	0.90504744	1.27231614	2.06814001	1.66481172	0	0	0
Medag	5.92573844	-0.3254273	0.40262722	-0.8082597	0.4189411	0.99938111	Medag	7.39250961	5.88280835	9.224292	6.20442003	1.66481172	7.05632714	7.69811111	2.28262753
Polr2h	4.47449617	0.33553785	0.41514751	0.80823766	0.41895378	NA	Polr2h	3.36023164	1.81009488	2.54463228	8.27256004	1.66481172	5.88027262	7.69811111	4.56525506
Pelp1	37.0257788	0.30610103	0.37875126	0.80818485	0.41898418	0.99938111	Pelp1	22.1775288	24.4362808	22.2655324	57.9079203	51.6091633	19.9929269	20.2075417	77.6093361
Fam174a	20.5599412	0.26344316	0.32597679	0.80816538	0.41899538	0.99938111	Fam174a	14.7850192	23.0787097	16.8581888	16.5451201	29.966611	15.2887088	18.2830139	29.6741579
Mthfd2	5.02454792	0.33022729	0.40861962	0.80815329	0.41900234	0.99938111	Mthfd2	3.36023164	2.71514231	3.81694841	8.27256004	4.99443516	7.05632714	7.69811111	2.28262753
Prr14	18.7831475	-0.3001462	0.37143294	-0.8080765	0.41904653	0.99938111	Prr14	19.4893435	21.2686148	15.2677937	28.9539601	21.6425524	4.7042181	11.5471667	27.3915304
Mrpl48	6.54422494	-0.3341707	0.41361226	-0.8079324	0.4191295	0.99938111	Mrpl48	12.0968339	7.69290322	8.58813393	2.06814001	8.3240586	0	6.73584722	6.84788259
Jmy	60.8106051	0.26177267	0.32408058	0.80773944	0.41924059	0.99938111	Jmy	49.059382	66.068463	65.8423601	35.1583802	61.5980337	101.140689	77.943375	29.6741579
Psmf1	21.17661	0.24287499	0.30073708	0.8075991	0.41932141	0.99938111	Psmf1	18.1452509	17.648425	18.130505	22.7495401	19.9777406	19.9929269	23.0943333	29.6741579
Anapc13	8.22240594	-0.3105331	0.38461737	-0.8073819	0.41944651	0.99938111	Anapc13	12.7688802	7.2403795	6.67965972	12.4088401	8.3240586	7.05632714	6.73584722	4.56525506
Apc	261.990177	0.31796029	0.39384833	0.80731659	0.41948411	0.99938111	Apc	241.936678	311.788842	244.602778	76.5211804	179.799666	593.907535	374.320653	73.044081
Psg16	1.29017464	-0.3078472	0.3813894	-0.807173	0.41956683	NA	Psg16	2.01613899	1.81009488	3.18079035	0	0	2.35210905	0.96226389	0
Gdi1	533.840277	0.20834102	0.25814383	0.80707341	0.41962419	0.99938111	Gdi1	481.857217	367.44926	444.992569	670.077363	604.326654	465.717592	464.773458	771.528105
Hirip3	8.59748453	0.31705982	0.392879	0.80701646	0.419657	0.99938111	Hirip3	6.04841696	5.43028463	9.54237104	8.27256004	1.66481172	12.9365998	13.4716944	11.4131377
Arl6ip5	73.0996559	-0.2483701	0.30794564	-0.8065388	0.41993225	0.99938111	Arl6ip5	73.2530498	116.751119	80.1559167	51.7035003	48.2795399	99.9646345	64.4716805	50.2178057
Gpr62	6.9946062	0.33365946	0.41369912	0.80652689	0.41993911	0.99938111	Gpr62	2.68818531	4.97776091	4.45310648	10.3407001	8.3240586	8.23238167	0.96226389	15.9783927
Ccnt1	18.2679039	-0.2845498	0.35283025	-0.8064779	0.41996736	0.99938111	Ccnt1	23.5216215	26.2463757	20.6751372	12.4088401	13.3184938	12.9365998	27.9056528	9.13051012
Prom1	2.79218075	-0.305877	0.37927612	-0.8064757	0.4199686	NA	Prom1	0	9.05047438	5.40734359	0	0	2.35210905	0.96226389	4.56525506
Nek8	1.46191179	-0.2936771	0.36427412	-0.806198	0.42012866	NA	Nek8	2.68818531	0.90504744	0.63615807	4.13628002	3.32962344	0	0	0
Rps20	23.2313019	-0.3218388	0.39929355	-0.8060206	0.42023094	0.99938111	Rps20	36.2905017	16.7433776	26.4005599	31.0221002	4.99443516	8.23238167	46.1886666	15.9783927
Ctbp1	158.064755	0.23135506	0.28711084	0.80580398	0.42035588	0.99938111	Ctbp1	127.688802	105.438027	124.050823	219.222841	221.419959	116.429398	153.962222	196.305968
Ppp1r12a	46.8739129	0.29805583	0.37000466	0.80554617	0.42050457	0.99938111	Ppp1r12a	57.1239379	38.9170398	44.2129858	20.6814001	19.9777406	101.140689	58.6980972	34.239413
Cox7b2	0.35510828	0.18508359	0.22979227	0.80543871	0.42056656	NA	Cox7b2	0	0	0	0	1.66481172	1.17605452	0	0
Gm12504	0.35510828	0.18508359	0.22979227	0.80543871	0.42056656	NA	Gm12504	0	0	0	0	1.66481172	1.17605452	0	0
Gm5176	0.35510828	0.18508359	0.22979227	0.80543871	0.42056656	NA	Gm5176	0	0	0	0	1.66481172	1.17605452	0	0
Neurog2	0.35510828	0.18508359	0.22979227	0.80543871	0.42056656	NA	Neurog2	0	0	0	0	1.66481172	1.17605452	0	0
Camsap2	276.203611	0.22727567	0.28222003	0.80531376	0.42063864	0.99938111	Camsap2	260.753975	262.463757	277.682997	204.745861	199.777406	450.428883	348.339528	205.436478
Sugt1	31.2019128	0.26911979	0.33425071	0.80514352	0.42073687	0.99938111	Sugt1	30.2420848	28.5089943	31.8079035	20.6814001	26.6369875	47.042181	50.9999861	13.6957652
Mrps2	32.2418837	-0.2290787	0.28454308	-0.8050755	0.42077609	0.99938111	Mrps2	37.6345944	39.8220873	34.3525357	28.9539601	33.2962344	23.5210905	37.5282917	22.8262753
Kcnk13	4.350618	-0.3334756	0.41427698	-0.8049581	0.42084386	NA	Kcnk13	4.03227797	7.2403795	4.45310648	4.13628002	4.99443516	1.17605452	1.92452778	6.84788259
Fdx1l	18.5802898	0.3217117	0.3997524	0.80477742	0.42094814	0.99938111	Fdx1l	11.4247876	4.52523719	10.8146872	37.2265202	19.9777406	10.5844907	22.1320694	31.9567854
Adat3	1.6241778	0.2585454	0.32129919	0.80468737	0.42100011	NA	Adat3	0	0	0	4.13628002	3.32962344	0	0.96226389	4.56525506
Pabpn1	79.305588	0.32968193	0.40973076	0.80463066	0.42103284	0.99938111	Pabpn1	42.3389187	20.8160911	29.5813502	165.451201	118.201632	41.1619083	36.5660278	180.327575
Mapk1ip1l	46.0570688	0.226291	0.28124052	0.80461735	0.42104052	0.99938111	Mapk1ip1l	37.6345944	39.3695635	42.3045116	47.5672202	48.2795399	30.5774176	51.96225	70.7614535
Cyyr1	1.99916669	-0.3095783	0.38479514	-0.8045274	0.42109242	NA	Cyyr1	0.67204633	6.78785578	1.27231614	2.06814001	1.66481172	3.52816357	0	0
Ankrd44	6.26862814	0.32401668	0.40283128	0.80434835	0.42119582	0.99938111	Ankrd44	4.7043243	6.78785578	4.13502775	6.20442003	8.3240586	9.40843619	10.5849028	0
Zfp746	17.7694794	0.29121146	0.36205374	0.80433216	0.42120517	0.99938111	Zfp746	18.1452509	12.2181404	8.90621297	22.7495401	18.3129289	14.1126543	13.4716944	34.239413
Aatk	215.498191	0.3150386	0.3917801	0.804121	0.4213271	0.99938111	Aatk	121.640385	129.874307	110.373425	361.924502	401.219625	145.830761	67.3584722	385.764053
Tmem147	33.3842336	-0.2923232	0.36356802	-0.80404	0.42137385	0.99938111	Tmem147	18.1452509	47.9675142	28.9451921	55.8397803	24.9721758	22.345036	16.3584861	52.5004332
Cyb561a3	11.6031232	-0.3134657	0.38995552	-0.8038498	0.42148372	0.99938111	Cyb561a3	8.73660227	10.8605693	11.1327662	22.7495401	8.3240586	4.7042181	5.77358333	20.5436478
Epha5	98.9634368	0.29899659	0.37195948	0.80384183	0.42148833	0.99938111	Epha5	74.5971425	129.421784	95.4237104	39.2946602	76.5813391	238.739068	76.0188472	61.6309433
Rplp1	163.108646	-0.3331952	0.41451431	-0.8038206	0.42150058	0.99938111	Rplp1	145.834053	72.8563187	96.3779475	473.604062	183.129289	17.6408179	52.9245139	262.502166
Amotl2	9.35431283	0.3201821	0.3983561	0.8037585	0.42153646	0.99938111	Amotl2	8.06455594	8.59795066	6.04350166	14.4769801	11.653682	10.5844907	4.81131944	15.9783927
Srsf6	35.0433261	0.28479595	0.35438476	0.80363487	0.42160788	0.99938111	Srsf6	27.5538995	26.6988994	36.897168	33.0902402	18.3129289	54.0985081	65.4339444	18.2610202
D15Ertd621e	77.019392	-0.2151524	0.26781192	-0.8033711	0.42176028	0.99938111	D15Ertd621e	87.3660227	85.0744592	87.4717345	76.5211804	53.2739751	89.3801438	91.4150694	45.6525506
Otud4	43.0995117	0.23945619	0.29821847	0.8029556	0.4220004	0.99938111	Otud4	49.7314283	42.0847059	39.7598793	22.7495401	31.6314227	63.5069443	51.96225	43.3699231
Lenep	2.61324394	-0.3341786	0.4161856	-0.8029556	0.42200041	NA	Lenep	3.36023164	4.07271347	3.18079035	2.06814001	1.66481172	2.35210905	1.92452778	2.28262753
Ss18	7.44849438	-0.3094608	0.38552399	-0.8027017	0.42214716	0.99938111	Ss18	12.0968339	7.69290322	6.99773876	8.27256004	4.99443516	8.23238167	6.73584722	4.56525506
Mef2c	296.689758	0.28482248	0.35491856	0.80250095	0.42226324	0.99938111	Mef2c	335.351118	285.99499	324.440615	86.8618804	256.381005	364.576902	535.018722	184.89283
Mrrf	17.32659	0.27727678	0.34553788	0.80244974	0.42229285	0.99938111	Mrrf	12.7688802	11.7656167	15.2677937	20.6814001	19.9777406	11.7605452	14.4339583	31.9567854
Zfp868	11.6274253	0.31804955	0.3963759	0.80239378	0.42232521	0.99938111	Zfp868	8.06455594	5.88280835	15.2677937	10.3407001	11.653682	7.05632714	27.9056528	6.84788259
Bub1b	0.46709255	0.21971769	0.27387027	0.80226921	0.42239725	NA	Bub1b	0	0	0.63615807	0	0	1.17605452	1.92452778	0
Cml2	0.46709255	0.21971769	0.27387027	0.80226921	0.42239725	NA	Cml2	0	0	0.63615807	0	0	1.17605452	1.92452778	0
Gramd1c	0.46709255	0.21971769	0.27387027	0.80226921	0.42239725	NA	Gramd1c	0	0	0.63615807	0	0	1.17605452	1.92452778	0
Prdm9	0.46709255	0.21971769	0.27387027	0.80226921	0.42239725	NA	Prdm9	0	0	0.63615807	0	0	1.17605452	1.92452778	0
Glb1	12.0505995	-0.2895141	0.36086935	-0.8022683	0.42239775	0.99938111	Glb1	12.0968339	17.648425	9.54237104	16.5451201	9.98887032	14.1126543	9.62263889	6.84788259
Gjd2	7.00832162	0.31073998	0.38742403	0.80206688	0.42251427	0.99938111	Gjd2	7.39250961	6.33533207	5.72542262	4.13628002	4.99443516	12.9365998	7.69811111	6.84788259
Tollip	120.68751	0.25012418	0.31186595	0.80202466	0.4225387	0.99938111	Tollip	92.7423933	86.4320303	81.4282328	173.723761	149.833055	88.2040893	99.1131805	194.02334
Ube2s	17.1621384	0.31874017	0.39758012	0.80170047	0.42272625	0.99938111	Ube2s	11.4247876	7.2403795	10.4966081	28.9539601	23.3073641	5.88027262	13.4716944	36.5220405
Mettl9	36.6480831	-0.2505029	0.31254485	-0.8014942	0.42284558	0.99938111	Mettl9	40.3227797	41.1796584	46.7576181	35.1583802	24.9721758	29.4013631	54.8490416	20.5436478
Angptl2	1.51373579	0.29802943	0.3718615	0.80145279	0.42286957	NA	Angptl2	0.67204633	2.26261859	0.31807903	0	3.32962344	0	0.96226389	4.56525506
Nenf	22.9045443	-0.2724425	0.33997679	-0.8013562	0.42292548	0.99938111	Nenf	15.4570656	27.1514231	23.5378486	37.2265202	9.98887032	19.9929269	20.2075417	29.6741579
Msra	11.5673049	-0.2948764	0.36797933	-0.8013397	0.42293501	0.99938111	Msra	16.1291119	12.2181404	12.7231614	10.3407001	13.3184938	3.52816357	10.5849028	13.6957652
Inmt	0.83211797	-0.1980862	0.24719782	-0.8013266	0.42294257	NA	Inmt	0	0.45252372	0	6.20442003	0	0	0	0
Aard	1.72414259	0.30071988	0.37531239	0.80125221	0.42298565	NA	Aard	0.67204633	0.90504744	0	4.13628002	1.66481172	3.52816357	2.88679167	0
Surf2	20.518671	-0.2745706	0.34268303	-0.8012378	0.42299399	0.99938111	Surf2	32.2582238	23.5312334	24.8101647	10.3407001	18.3129289	12.9365998	25.981125	15.9783927
Grm5	219.225944	0.29273814	0.36540648	0.80113012	0.42305632	0.99938111	Grm5	176.748184	274.229374	222.655324	82.7256004	168.145984	502.175282	192.452778	134.675024
Gss	5.48166815	-0.3311029	0.41331743	-0.8010863	0.42308169	0.99938111	Gss	5.37637063	1.35757116	2.22655324	20.6814001	1.66481172	3.52816357	6.73584722	2.28262753
Snapc4	10.0458876	0.30956149	0.38649065	0.80095466	0.42315789	0.99938111	Snapc4	8.73660227	5.88280835	10.4966081	8.27256004	23.3073641	5.88027262	8.660375	9.13051012
Tmem164	23.38485	-0.2612566	0.32628166	-0.8007089	0.42330021	0.99938111	Tmem164	20.8334362	31.6766603	24.4920857	26.8858201	34.9610461	15.2887088	19.2452778	13.6957652
5530601H04Rik	3.99647026	0.33022227	0.41241827	0.80069747	0.42330681	NA	5530601H04Ri	2.68818531	4.97776091	2.54463228	2.06814001	3.32962344	4.7042181	4.81131944	6.84788259
Hfm1	0.95895939	0.28625547	0.35764259	0.80039535	0.42348177	NA	Hfm1	1.34409266	0.45252372	0.63615807	0	0	2.35210905	2.88679167	0
Wdr82	151.177448	0.15976069	0.1996321	0.80027556	0.42355116	0.99938111	Wdr82	144.489961	131.684402	152.359858	138.565381	178.134854	132.894161	157.811278	173.479692
Cx3cl1	284.21644	-0.2591915	0.32390184	-0.8001842	0.42360408	0.99938111	Cx3cl1	287.635829	476.507476	316.806718	177.860041	337.956779	303.422067	127.018833	246.523773
Apip	2.87870631	0.33104383	0.41382022	0.79997017	0.42372808	NA	Apip	2.68818531	3.62018975	2.22655324	0	1.66481172	7.05632714	5.77358333	0
Arhgap6	28.3835436	-0.2699066	0.33745267	-0.7998354	0.42380618	0.99938111	Arhgap6	31.5861774	33.0342315	29.8994292	35.1583802	19.9777406	38.8097993	31.7547083	6.84788259
Casp9	33.2103046	0.26832621	0.33551447	0.79974557	0.42385822	0.99938111	Casp9	23.5216215	19.0059962	28.3090341	47.5672202	33.2962344	30.5774176	24.0565972	59.3483158
Ncor2	178.925454	0.30629169	0.38299081	0.79973641	0.42386353	0.99938111	Ncor2	110.215598	110.868311	100.512975	287.471461	419.532554	122.309671	75.0565833	205.436478
Nae1	21.948337	0.3121356	0.39031142	0.79970911	0.42387935	0.99938111	Nae1	19.4893435	23.9837235	22.2655324	8.27256004	11.653682	38.8097993	44.2641389	6.84788259
Tusc2	77.7234895	-0.2124941	0.26577167	-0.7995362	0.42397955	0.99938111	Tusc2	84.0057911	81.0017457	75.7028102	95.1344405	68.2572805	43.5140174	76.0188472	98.1529838
Scx	1.06128987	0.27650013	0.34588585	0.79939706	0.42406021	NA	Scx	0	0	0.9542371	2.06814001	3.32962344	1.17605452	0.96226389	0
Klhl9	111.118701	0.18796768	0.23514279	0.79937673	0.424072	0.99938111	Klhl9	90.7262543	102.722884	124.686982	95.1344405	98.2238915	142.302597	134.716944	100.435611
Gm11710	0.53734579	0.22127162	0.27684289	0.79926783	0.42413513	NA	Gm11710	0	0.45252372	0.31807903	0	0	3.52816357	0	0
Gm11711	0.53734579	0.22127162	0.27684289	0.79926783	0.42413513	NA	Gm11711	0	0.45252372	0.31807903	0	0	3.52816357	0	0
Vim	6.77255623	-0.3307292	0.41382573	-0.7991992	0.42417494	0.99938111	Vim	4.03227797	9.5029981	8.90621297	10.3407001	14.9833055	3.52816357	2.88679167	0
Casp6	0.37746786	-0.2149504	0.26896145	-0.7991866	0.4241822	NA	Casp6	1.34409266	1.35757116	0.31807903	0	0	0	0	0
Pstpip1	0.37746786	-0.2149504	0.26896145	-0.7991866	0.4241822	NA	Pstpip1	1.34409266	1.35757116	0.31807903	0	0	0	0	0
Tmed10	107.916259	-0.248068	0.31041042	-0.7991612	0.42419692	0.99938111	Tmed10	67.2046328	142.544971	113.236136	153.042361	71.586904	121.133616	64.4716805	130.109769
Setd1a	55.4071846	0.23837336	0.29840678	0.79882017	0.42439469	0.99938111	Setd1a	42.3389187	46.6099431	38.8056422	74.4530404	84.9053977	54.0985081	40.4150833	61.6309433
Psme2	1.11094628	-0.25418	0.31824081	-0.7987034	0.42446244	NA	Psme2	2.01613899	0.45252372	0	4.13628002	0	0	0	2.28262753
Pold1	3.22339533	0.28781315	0.36035141	0.79870131	0.42446363	NA	Pold1	0	1.81009488	0.31807903	6.20442003	8.3240586	0	0	9.13051012
Cacybp	48.282653	0.27276472	0.34162428	0.7984348	0.42461821	0.99938111	Cacybp	53.0916599	49.7776091	41.9864326	24.8176801	21.6425524	62.3308898	84.6792222	47.9351782
Tceanc2	14.2693748	0.27409829	0.34330561	0.79840898	0.42463319	0.99938111	Tceanc2	14.7850192	8.14542694	12.4050823	16.5451201	16.6481172	18.8168724	15.3962222	11.4131377
Mrpl1	13.4419161	0.30434297	0.38130132	0.79816919	0.42477232	0.99938111	Mrpl1	12.7688802	10.8605693	14.6316356	8.27256004	8.3240586	21.1689814	26.9433889	4.56525506
Mob3b	4.01296641	-0.331071	0.41484298	-0.7980633	0.42483375	NA	Mob3b	2.68818531	6.33533207	5.72542262	4.13628002	6.65924688	2.35210905	1.92452778	2.28262753
Hey1	46.9048592	-0.2462257	0.3085398	-0.7980355	0.42484989	0.99938111	Hey1	45.6991503	37.5594687	40.7141164	84.7937404	46.6147282	31.7534722	35.6037639	52.5004332
Ltbp1	3.08559594	-0.3316819	0.41565776	-0.7979687	0.42488864	NA	Ltbp1	4.7043243	5.88280835	2.54463228	2.06814001	4.99443516	3.52816357	0.96226389	0
Emilin1	0.75155281	0.232	0.29075915	0.79791125	0.424922	NA	Emilin1	0.67204633	0	0.63615807	0	0	4.7042181	0	0
Slmo1	32.5588613	-0.281278	0.3525542	-0.797829	0.42496974	0.99938111	Slmo1	36.9625481	14.9332827	32.7621406	64.1123403	31.6314227	21.1689814	26.9433889	31.9567854
Rrp8	12.1952004	-0.3022136	0.37879725	-0.7978242	0.42497252	0.99938111	Rrp8	17.4732045	13.1231878	11.1327662	12.4088401	11.653682	3.52816357	7.69811111	20.5436478
Mff	153.240558	-0.2275337	0.28519329	-0.7978227	0.42497341	0.99938111	Mff	173.387953	148.42778	175.897706	173.723761	81.5757743	132.894161	221.320694	118.696632

GeneID	Base mean	log2(FC)	StdErr	Wald-Stats	P-value	P-adj
Palm3	0.62151651	-0.2583896	0.32390099	-0.7977427	0.42501983	NA
Apba2	93.0546036	0.22505844	0.28216271	0.79761938	0.4250914	0.99938111
Rpl5	20.0714004	0.3199916	0.40121871	0.79754906	0.42513222	0.99938111
D8Ertd82e	31.190365	0.25797198	0.32346095	0.7975367	0.4251394	0.99938111
Ndfip2	51.5042343	-0.2395198	0.30033266	-0.797515	0.42515198	0.99938111
F420014N23Rik	3.10387702	-0.3318695	0.41616444	-0.7974479	0.42519093	NA
Asic2	61.9593288	-0.2691341	0.33750604	-0.7974202	0.42520706	0.99938111
Tal1	0.92379331	-0.2743688	0.34410544	-0.7973393	0.42525399	NA
Cant1	31.7140758	0.26923257	0.33767969	0.79730164	0.42527587	0.99938111
Zfp395	23.1730797	0.31887459	0.40005412	0.79707863	0.42540536	0.99938111
Prss12	7.54084701	0.31024054	0.38926759	0.79698529	0.42545958	0.99938111
Gltpd1	29.1937952	-0.2478003	0.31101651	-0.7967431	0.42560026	0.99938111
Rpap1	18.024815	0.32964142	0.41390014	0.79642741	0.42578365	0.99938111
Fto	166.617663	0.21831578	0.27414455	0.79635281	0.425827	0.99938111
Kmt2c	93.1524213	0.22005785	0.27633262	0.79635134	0.42582785	0.99938111
Lurap1	11.1361069	0.30522595	0.38332674	0.79625531	0.42588366	0.99938111
Pold4	10.2967048	0.33097193	0.41566831	0.79624048	0.42589227	0.99938111
Nfkb1	8.78429217	0.3146838	0.39532618	0.79601052	0.42602592	0.99938111
Gcc1	15.512798	0.257319	0.32330458	0.79590273	0.42608858	0.99938111
Morc2a	33.192853	0.24921568	0.31321869	0.7956603	0.42622951	0.99938111
Smc3	37.4944098	0.28511363	0.35834785	0.79563371	0.42624496	0.99938111
Dusp10	13.3979642	0.29138831	0.3662358	0.79563033	0.42624693	0.99938111
Tle4	44.3511943	0.20774374	0.26115551	0.79547906	0.42633489	0.99938111
Dusp7	60.9191943	-0.2375414	0.29866816	-0.7953356	0.42641832	0.99938111
Slc25a24	1.24833004	-0.2952716	0.37138173	-0.7950622	0.42657735	NA
Kctd21	15.770951	-0.2640843	0.33215861	-0.7950547	0.42658167	0.99938111
Enpp6	1.60193182	0.30083827	0.37846324	0.79489429	0.426675	NA
Mn1	84.3274918	0.28979181	0.36461117	0.79479686	0.42673168	0.99938111
Sema6b	111.137202	0.30071185	0.37839138	0.79471115	0.42678154	0.99938111
Coa3	36.5150677	-0.2381383	0.29971289	-0.7945547	0.42687256	0.99938111
Sec16a	61.1739271	0.22456259	0.28278425	0.79411279	0.42712977	0.99938111
Gal3st1	6.04146638	0.31997792	0.40307908	0.7938341	0.42729202	0.99938111
Smoc2	3.1405793	-0.3243601	0.40860028	-0.7938322	0.4272931	NA
4931428F04Rik	69.141395	0.32684743	0.41186219	0.79358444	0.4274374	0.99938111
Igdcc3	1.11277459	-0.2959202	0.3728937	-0.7935778	0.42744126	NA
Hnrnph1	55.903301	0.21912419	0.27615215	0.79349082	0.42749192	0.99938111
Mttp	13.8715718	0.2820586	0.35547158	0.79347721	0.42749984	0.99938111
Igbp1b	0.86148713	0.24691408	0.31122443	0.79336343	0.42756611	NA
Itgb3	0.7154273	0.24937953	0.314429	0.79311875	0.42770864	NA
AA415398	3.00702245	-0.3290248	0.41489415	-0.7930332	0.42775848	NA
Rcsd1	1.45667523	-0.3054104	0.3852632	-0.7927319	0.42793403	NA
2610037D02Rik	0.32838445	0.17987142	0.22696856	0.79249488	0.42807217	NA
BC035044	0.32838445	0.17987142	0.22696856	0.79249488	0.42807217	NA
Ccr2	0.32838445	0.17987142	0.22696856	0.79249488	0.42807217	NA
Fap	0.32838445	0.17987142	0.22696856	0.79249488	0.42807217	NA
Gm20172	0.32838445	0.17987142	0.22696856	0.79249488	0.42807217	NA
Il7r	0.32838445	0.17987142	0.22696856	0.79249488	0.42807217	NA
Itgal	0.32838445	0.17987142	0.22696856	0.79249488	0.42807217	NA
Snhg9	0.32838445	0.17987142	0.22696856	0.79249488	0.42807217	NA
Car10	96.9178486	-0.2075894	0.26203999	-0.7922051	0.42824108	0.99938111
Aurka	2.50552267	-0.3188759	0.40251915	-0.7922005	0.42824379	NA
AF529169	8.38820402	0.32571109	0.41115431	0.79218698	0.42825166	0.99938111
Mfap1b	20.1507813	0.28272545	0.35693716	0.79208747	0.42830968	0.99938111
Slamf1	0.70382415	0.20045239	0.25308419	0.7920384	0.42833829	NA
Calm3	1993.10228	0.31282645	0.39499635	0.79197302	0.42837641	0.99938111
Dram2	11.0014375	-0.3289817	0.41544151	-0.7918845	0.428428	0.99938111
Gm14306	3.3306128	0.32137972	0.40586376	0.7918414	0.42845316	NA
Zfp51	3.4578265	-0.3229711	0.40801781	-0.7915612	0.42861657	NA
Mapt	439.31598	0.21676812	0.27391663	0.79136531	0.42873085	0.99938111
Dusp14	31.2041748	-0.2439943	0.30833172	-0.791337	0.42874735	0.99938111
Tmem260	12.5900219	-0.28887	0.36511354	-0.7911786	0.42883978	0.99938111
Slc39a10	130.041962	-0.309371	0.39107173	-0.791085	0.42889438	0.99938111
Nbeal1	16.1833374	-0.3010408	0.380582	-0.7910011	0.42894334	0.99938111
Pde11a	4.22080384	0.32924456	0.41627758	0.79092552	0.42898746	NA
Larp4	20.0141132	0.27086235	0.34248599	0.79087133	0.42901908	0.99938111
Plek	6.63700342	-0.3256026	0.41174138	-0.7907939	0.42906426	0.99938111
Ubap2	51.2604079	-0.2088291	0.26411313	-0.7906804	0.4291305	0.99938111
Dkkl1	3.67732229	-0.3226347	0.4080647	-0.7906458	0.4291507	NA
Tyro3	54.3762074	-0.2795622	0.35359811	-0.7906212	0.42916505	0.99938111
Coro7	44.1077445	0.28342725	0.35850104	0.79058976	0.42918342	0.99938111
Sgms1	18.4317977	-0.3071829	0.38855897	-0.7905696	0.42919521	0.99938111
Slc50a1	7.65420707	-0.3103335	0.39267051	-0.7903154	0.42934362	0.99938111
Celsr2	246.085449	0.28246868	0.35754225	0.79002881	0.42951094	0.99938111
Phrf1	30.8595988	0.24011068	0.30404628	0.78971754	0.42969274	0.99938111
Slc12a9	12.4384493	0.3182394	0.40301901	0.78963869	0.42973881	0.99938111
Aebp1	7.94757467	-0.3197074	0.40500177	-0.7893975	0.42987972	0.99938111
L2hgdh	11.9517321	0.28734154	0.3640062	0.78938638	0.42988622	0.99938111
Nmnat2	277.415317	0.2265056	0.28699666	0.78922731	0.42997916	0.99938111
Bccip	25.2312476	0.29782857	0.37737418	0.78921289	0.42998759	0.99938111
Rhot2	64.9512571	0.31073041	0.39377411	0.78910829	0.43004872	0.99938111
Cdc25a	3.30223859	-0.3285084	0.41635498	-0.7890104	0.4301059	NA
Ylpm1	145.695272	0.18051716	0.22879122	0.78900387	0.43010975	0.99938111
Shmt1	1.86110169	-0.2915616	0.36969594	-0.7886526	0.4303151	NA
Zyg11b	210.691261	0.20762918	0.26331068	0.78853309	0.43038495	0.99938111
Card6	1.46230718	-0.2994058	0.37971402	-0.7885035	0.43040225	NA
Smcr8	46.5536998	0.19469827	0.24694074	0.78844127	0.43043864	0.99938111
Slc25a1	38.5623413	-0.3221355	0.40857872	-0.7884294	0.4304456	0.99938111
Ldhd	5.05429912	0.32536	0.41268244	0.78840283	0.43046112	0.99938111
Ercc8	5.24457402	-0.3183893	0.40397358	-0.7881439	0.43061253	0.99938111
Thoc2	24.3577705	0.27521988	0.34920051	0.78814284	0.43061316	0.99938111
Scly	8.17928152	0.30297952	0.38442354	0.78813988	0.43061489	0.99938111
Zfa-ps	1.71894919	0.30096527	0.38190946	0.78805398	0.43066513	NA
4932418E24Rik	0.42506126	-0.191525	0.24311988	-0.78778	0.43082538	NA
Aplf	3.37002918	0.32705589	0.41518941	0.78772695	0.43085644	NA
Ppp6r3	47.3037847	0.21343872	0.27097895	0.78765793	0.43089682	0.99938111
Gm10509	4.66934119	0.32021647	0.40658522	0.78757528	0.43094518	0.99938111
A230020J21Rik	1.70369756	0.27597362	0.35042688	0.78753553	0.43096844	NA
Zfp248	12.9631325	0.29079779	0.36928382	0.78746422	0.43101017	0.99938111
Sord	6.82425545	-0.3161139	0.40149384	-0.7873443	0.43108034	0.99938111
Ntn5	1.08163535	-0.2820142	0.35826684	-0.7871624	0.43118682	NA
Akap1	14.0583544	0.29502592	0.37480801	0.78713877	0.43120064	0.99938111
Ankrd53	0.53318979	0.21532828	0.27355824	0.78713871	0.43120067	NA
Fam46c	1.07717922	0.27234882	0.34606945	0.78697737	0.43129512	NA
Ccdc74a	5.23571123	-0.3253854	0.41349132	-0.7869219	0.43132756	0.99938111
Ddx50	13.7548601	-0.2826004	0.35923443	-0.786674	0.43147271	0.99938111
Atf5	26.7121055	0.32308083	0.41071645	0.78662744	0.43149999	0.99938111
0610012G03Rik	21.6320311	-0.2393096	0.30422427	-0.7866224	0.43150296	0.99938111
Naa40	10.0356049	0.30008102	0.38160713	0.78636115	0.43165594	0.99938111
Tmco4	1.51504592	-0.2880458	0.36633338	-0.7862942	0.43169512	NA
Tsacc	0.40060884	0.18436457	0.23447618	0.78628274	0.43170186	NA
Uox	0.40060884	0.18436457	0.23447618	0.78628274	0.43170186	NA
Arhgap23	82.2146499	0.31833172	0.40489141	0.78621504	0.43174152	0.99938111

GeneID	Normalised expression for Chow#1	Normalised expression for Chow#2	Normalised expression for Chow#3	Normalised expression for Chow#4	Normalised expression for HFD#1	Normalised expression for HFD#2	Normalised expression for HFD#3	Normalised expression for HFD#4
Palm3	2.01613899	1.35757116	0.63615807	0	0	0	0.96226389	0
Apba2	106.18332	65.1634155	76.3389683	90.9981605	128.190502	112.901234	59.6603611	105.000866
Rpl5	27.5538995	15.3858064	19.0847421	4.13628002	6.65924688	38.8097993	37.5282917	11.4131377
D8Ertd82e	30.9141311	27.1514231	22.5836114	28.9539601	46.6147282	19.9929269	23.0943333	50.2178057
Ndfip2	47.043243	70.5937002	65.2062021	43.4309402	26.6369875	56.4506172	61.5848889	41.0872956
F420014N23R	4.7043243	3.62018975	3.81694841	4.13628002	0	4.7042181	3.84905555	0
Asic2	51.7475673	73.7613662	41.9864326	109.611421	63.2628454	42.3379629	30.7924444	82.1745911
Tal1	2.01613899	0.90504744	2.54463228	0	0	0	1.92452778	0
Cant1	22.1775288	37.5594687	21.9474534	28.9539601	43.2851047	36.4576902	15.3962222	47.9351782
Zfp395	10.0806949	9.95552182	13.0412404	45.4990802	34.9610461	11.7605452	14.4339583	45.6525506
Prss12	6.72046328	8.14542694	6.67965972	2.06814001	6.65924688	10.5844907	5.77358333	13.6957652
Gltpd1	28.8979921	28.5089943	27.672876	43.4309402	31.6314227	17.6408179	19.2452778	36.5220405
Rpap1	7.39250961	8.14542694	7.95197586	35.1583802	36.6258578	4.7042181	7.69811111	36.5220405
Fto	162.635211	123.991499	128.50393	194.405161	234.738453	154.063143	115.471667	219.132243
Kmt2c	84.6778374	98.6501707	86.8355764	70.3167603	113.207197	141.126543	93.3395972	57.0656883
Lurap1	9.4086486	4.97776091	8.2700549	16.5451201	19.9777406	11.7605452	6.73584722	11.4131377
Pold4	6.72046328	4.52523719	4.77118552	16.5451201	11.653682	2.35210905	3.84905555	31.9567854
Nfkb1	8.06455594	8.14542694	3.81694841	10.3407001	8.3240586	17.6408179	4.81131944	9.13051012
Gcc1	12.7688802	13.1231878	13.3593194	16.5451201	21.6425524	15.2887088	15.3962222	15.9783927
Morc2a	38.978687	32.129184	29.8994292	16.5451201	29.966611	55.2745626	30.7924444	31.9567854
Smc3	45.6991503	28.961518	35.6248519	20.6814001	21.6425524	72.9153805	53.8867778	20.5436478
Dusp10	12.0968339	8.14542694	8.90621297	18.6132601	6.65924688	16.4647633	13.4716944	22.8262753
Tle4	43.6830113	40.274611	44.2129858	35.1583802	46.6147282	43.5140174	62.5471528	38.804668
Dusp7	73.9250961	56.1129411	55.027673	82.7256004	78.2461509	29.4013631	54.8490416	57.0656883
Slc25a24	0.67204633	2.71514231	3.49886938	0	0	1.17605452	1.92452778	0
Kctd21	18.8172972	18.5534725	15.5858727	16.5451201	8.3240586	15.2887088	12.5094306	20.5436478
Enpp6	2.01613899	0	0.63615807	2.06814001	4.99443516	1.17605452	1.92452778	0
Mn1	73.9250961	51.587704	62.9796488	103.407001	161.486737	65.8590533	29.8301805	125.544514
Sema6b	73.2530498	110.415787	76.9751264	117.883981	193.11816	77.6195986	29.8301805	210.001733
Coa3	38.978687	38.9170398	30.2175083	55.8397803	24.9721758	30.5774176	45.2264028	27.3915304
Sec16a	50.4034746	63.3533207	55.663831	51.7035003	98.2238915	67.0351079	41.3773472	61.6309433
Gal3st1	2.68818531	5.88280835	5.08926455	6.20442003	8.3240586	2.35210905	8.660375	9.13051012
Smoc2	2.01613899	4.97776091	3.81694841	4.13628002	3.32962344	0	0	6.84788259
4931428F04Ri	26.8818531	22.1736622	30.2175083	144.769801	64.9276571	25.8731995	44.2641389	194.02334
Igdcc3	0.67204633	2.26261859	1.27231614	2.06814001	1.66481172	0	0.96226389	0
Hnrnph1	63.1723549	35.2968501	46.7576181	62.0442003	58.2684102	74.091435	59.6603611	47.9351782
Mttp	11.4247876	11.7656167	14.3135566	10.3407001	11.653682	25.8731995	9.62263889	15.9783927
Igbp1b	0	0	0.31807903	2.06814001	3.32962344	1.17605452	0	0
Itgb3	0	0.45252372	0.63615807	0	0	2.35210905	0	2.28262753
AA415398	4.7043243	4.97776091	3.18079035	2.06814001	0	5.88027262	0.96226389	2.28262753
Rcsd1	2.68818531	3.16766603	1.59039517	0	0	0	1.92452778	2.28262753
2610037D02Ri	0	0	0	0	1.66481172	0	0.96226389	0
BC035044	0	0	0	0	1.66481172	0	0.96226389	0
Ccr2	0	0	0	0	1.66481172	0	0.96226389	0
Fap	0	0	0	0	1.66481172	0	0.96226389	0
Gm20172	0	0	0	0	1.66481172	0	0.96226389	0
Il7r	0	0	0	0	1.66481172	0	0.96226389	0
Itgal	0	0	0	0	1.66481172	0	0.96226389	0
Snhg9	0	0	0	0	1.66481172	0	0.96226389	0
Car10	102.151042	132.58945	108.146872	74.4530404	83.240586	121.133616	76.0188472	77.6093361
Aurka	1.34409266	2.26261859	1.59039517	8.27256004	3.32962344	0	0.96226389	2.28262753
AF529169	8.73660227	5.43028463	7.63389683	6.20442003	4.99443516	23.5210905	10.5849028	0
Mfap1b	21.5054825	10.4080455	13.9954775	26.8858201	9.98887032	30.5774176	25.0188611	22.8262753
Slamf1	0	0	0.63615807	0	4.99443516	0	0	0
Calm3	1299.06555	1044.42474	1049.66081	3278.00192	2881.78909	666.822915	1084.4714	4640.58177
Dram2	11.4247876	17.1959013	22.5836114	2.06814001	1.66481172	21.1689814	9.62263889	2.28262753
Gm14306	2.68818531	2.71514231	4.13502745	0	0	9.40843619	7.69811111	0
Zfp51	4.03227797	8.59795066	5.08926455	0	0	7.05632714	2.88679167	0
Mapt	384.4105	333.962505	337.481856	552.193383	584.348914	396.330375	311.7735	614.026806
Dusp14	47.7152893	30.7716129	30.5355873	26.8858201	38.2906696	18.8168724	26.9433889	29.6741579
Tmem260	16.8011582	16.7433776	10.1785291	14.4769801	11.653682	17.6408179	8.660375	4.56525506
Slc39a10	125.672663	262.011233	177.170022	37.2265202	96.5590798	199.929269	102.962236	38.804668
Nbeal1	22.1775288	19.4585199	19.7209001	14.4769801	6.65924688	23.5210905	21.1698055	2.28262753
Pde11a	3.36023164	4.07271347	5.08926455	0	0	7.05632714	9.62263889	4.56525506
Larp4	20.8334362	22.1736622	18.448584	8.27256004	18.3129289	35.2816357	23.0943333	13.6957652
Plek	10.0806949	12.6706641	8.58813393	0	3.32962344	9.40843619	6.73584722	2.28262753
Ubap2	53.7637063	57.923036	48.6660923	59.9760603	51.6091633	47.042181	31.7547083	59.3483158
Dkkl1	5.37637063	6.78785578	6.04350166	0	8.3240586	0	2.88679167	0
Tyro3	59.8121232	88.6946489	55.345752	39.2946602	88.2350212	48.2182355	21.1698055	34.239413
Coro7	30.2420848	28.5089943	29.8994292	66.1804803	79.9109626	28.2253086	25.981125	63.9135709
Sgms1	14.1129729	33.9392789	27.0367179	10.3407001	9.98887032	28.2253086	19.2452778	4.56525506
Slc50a1	6.72046328	13.5757116	7.63389683	6.20442003	4.99443516	5.88027262	4.81131944	11.4131377
Celsr2	170.699767	299.118178	201.980187	184.064461	492.784269	261.084104	82.7546944	276.197931
Phrf1	19.4893435	29.8665654	27.354797	35.1583802	43.2851047	37.6337448	22.1320694	31.9567854
Slc12a9	4.03227797	13.5757116	8.58813393	14.4769801	26.6369875	5.88027262	5.77358333	20.5436478
Aebp1	9.4086486	15.8383302	9.224292	2.06814001	13.3184938	4.7042181	6.73584722	2.28262753
L2hgdh	8.06455594	7.2403795	12.7231614	14.4769801	6.65924688	14.1126543	16.3584861	15.9783927
Nmnat2	234.544169	211.7811	219.474534	343.311242	362.928955	219.922196	198.226361	429.133976
Bccip	26.8818531	25.793852	21.9474534	12.4088401	19.9777406	34.1055812	53.8867778	6.84788259
Rhot2	34.9464091	38.0119924	38.1694841	107.543281	93.2294563	24.697145	34.6415	148.37079
Cdc25a	6.72046328	3.16766603	2.86271131	4.13628002	3.32962344	2.35210905	3.84905555	0
Ylpm1	131.049034	147.070209	149.815225	111.679561	161.486737	184.64056	115.471667	164.349182
Shmt1	5.37637063	3.16766603	0	2.06814001	0	2.35210905	1.92452778	0
Zyg11b	210.350501	172.411537	219.792613	173.723761	186.458913	314.006558	262.698042	146.088162
Card6	1.34409266	3.16766603	1.59039517	2.06814001	0	3.52816357	0	0
Smcr8	49.7314283	41.6321821	40.3960374	41.3628002	54.9387868	49.39429	53.8867778	41.0872956
Slc25a1	26.8818531	20.8160911	20.3570582	115.815841	36.6258578	9.40843619	19.2452778	59.3483158
Ldhd	5.37637063	3.16766603	2.86271131	4.13628002	4.99443516	2.35210905	3.84905555	13.6957652
Ercc8	7.39250961	5.43028463	4.77118552	8.27256004	3.32962344	4.7042181	5.77358333	2.28262753
Thoc2	23.5216215	26.2463757	24.8101647	10.3407001	24.9721758	44.6900719	28.8679167	11.4131377
Scly	10.7527413	4.97776091	6.99773876	4.13628002	11.653682	5.88027262	9.62263889	11.4131377
Zfa-ps	0	2.71514231	1.59039517	0	0	2.35210905	4.81131944	2.28262753
4932418E24Ri	0	1.81009488	1.59039517	0	0	0	0	0
Aplf	2.01613899	2.71514231	3.49886938	2.06814001	3.32962344	5.88027262	2.88679167	4.56525506
Ppp6r3	53.7637063	39.3695635	45.4853019	35.1583802	43.2851047	52.9224536	67.3584722	41.0872956
Gm10509	4.03227797	4.07271347	5.40734359	0	0	9.40843619	14.4339583	0
A230020J21Ri	0	0	0.63615807	4.13628002	3.32962344	0	0.96226389	4.56525506
Zfp248	14.1129729	5.88280835	8.58813393	18.6132601	9.98887032	16.4647633	16.3584861	13.6957652
Sord	10.7527413	9.05047438	6.99773876	6.20442003	1.66481172	7.05632714	10.5849028	2.28262753
Ntn5	4.03227797	1.35757116	0.63615807	0	1.66481172	0	0.96226389	0
Akap1	13.4409266	19.4585199	10.1785291	4.13628002	13.3184938	27.0492541	13.4716944	11.4131377
Ankrd53	0	0	0.31807903	0	1.66481172	0	0	2.28262753
Fam46c	0	0.45252372	1.59039517	0	3.32962344	0	0.96226389	2.28262753
Ccdc74a	7.39250961	7.2403795	4.13502745	6.20442003	3.32962344	0	6.73584722	6.84788259
Ddx50	16.1291119	11.313093	14.6316356	22.7495401	8.3240586	11.7605452	18.2830139	6.84788259
Atf5	14.1129729	10.4080455	11.4508452	51.7035003	31.6314227	8.23238167	15.3962222	70.7614535
0610012G03R	30.2420848	22.1736622	21.9474534	20.6814001	19.9777406	17.6408179	22.1320694	18.2610202
Naa40	7.39250961	9.05047438	6.99773876	10.3407001	19.9777406	7.05632714	5.77358333	13.6957652
Tmco4	0	1.81009488	2.22655324	4.13628002	1.66481172	0	0	2.28262753
Tsacc	0	0	0.31807903	0	0	0	2.88679167	0
Uox	0	0	0.31807903	0	0	0	2.88679167	0
Arhgap23	46.3711967	44.7998482	35.9429309	144.769801	189.788536	30.5774176	30.7924444	134.675024

GeneID	Base mean	log2(FC)	StdErr	Wald-Stats	P-value	P-adj
Phactr1	272.225126	-0.1863042	0.23701286	-0.786051	0.43183761	0.99938111
Tmem116	1.45343472	-0.3035569	0.38628119	-0.7858444	0.43195868	NA
Mettl3	8.93355018	-0.2958042	0.3764398	-0.7857941	0.43198812	0.99938111
Amz1	16.1049886	0.30758926	0.39179653	0.78507398	0.4324102	0.99938111
Mus81	5.10125742	-0.3236732	0.41241807	-0.7848182	0.43256019	0.99938111
Mar-10	1.5191109	0.23650989	0.30137313	0.78477431	0.4325859	NA
B4galt5	48.8062284	-0.2546436	0.32458406	-0.7845227	0.43273346	0.99938111
Nacc1	129.762502	0.25033944	0.31910803	0.78449746	0.43274827	0.99938111
Lgi2	46.2901476	0.29947018	0.38182333	0.7843161	0.43285466	0.99938111
Ccdc108	1.12789536	-0.2943406	0.3752881	-0.7843057	0.43286078	NA
Ankrd29	14.7851823	0.27628587	0.35236602	0.78408773	0.43298864	0.99938111
Rab40c	25.4689553	0.25635869	0.32695253	0.78408537	0.43299002	0.99938111
Rac3	10.7616055	-0.3240423	0.41328722	-0.7840607	0.43300449	0.99938111
Alg9	14.1334024	-0.2767977	0.35305154	-0.7840149	0.43303138	0.99938111
Vdac1	253.047854	0.16318265	0.20817527	0.78387145	0.43311555	0.99938111
Sdsl	1.45324799	-0.2709819	0.34581017	-0.7836147	0.43326623	NA
Scmh1	25.0157771	-0.2665911	0.34022108	-0.783582	0.43328543	0.99938111
Nid1	3.97622473	-0.3262067	0.41635744	-0.7834776	0.43334668	NA
Agap2	1108.1585	0.28435201	0.36301088	0.78331541	0.43344192	0.99938111
Lrriq3	1.0774222	0.27100436	0.34599735	0.78325558	0.43347704	NA
Mtrf1	5.42054235	0.32242299	0.41168241	0.78318379	0.43351919	0.99938111
Havcr2	0.45083891	-0.1876022	0.23956606	-0.7830917	0.43357325	NA
Cyr61	0.72331722	-0.2556435	0.32649637	-0.7829904	0.43363277	NA
Twf2	20.4388025	0.29724066	0.37969685	0.78283679	0.43372296	0.99938111
Clstn2	39.7553331	0.29441552	0.37609023	0.78283214	0.43372569	0.99938111
Syne1	153.652682	0.23863055	0.30488676	0.78268584	0.43381162	0.99938111
Mgat5b	59.8834692	-0.2582447	0.32995247	-0.7826726	0.43381942	0.99938111
Tsen15	6.855165	-0.318028	0.4063418	-0.7826613	0.43382605	0.99938111
Nqo1	0.99583097	-0.2831664	0.36192403	-0.7823918	0.43398433	NA
Nkiras2	7.66579073	-0.3220871	0.4117537	-0.7822325	0.43407797	0.99938111
Snhg7	0.99027698	0.28072479	0.35908804	0.78177148	0.43434888	NA
Pim2	44.7505218	-0.2727927	0.34902754	-0.7815792	0.43446192	0.99938111
Sco2	11.9459817	-0.3011979	0.38537126	-0.7815785	0.43446232	0.99938111
Fam135a	20.1912689	0.28606406	0.36603255	0.78152628	0.43449303	0.99938111
Commd5	9.56209886	0.29194924	0.37362337	0.78139982	0.43456737	0.99938111
Fam196a	19.0724689	-0.272165	0.34833832	-0.7813239	0.43461204	0.99938111
Nf1	253.740445	0.20880273	0.26724278	0.78132223	0.434613	0.99938111
Zfp106	211.335238	0.21171533	0.27097393	0.78131252	0.43461871	0.99938111
Arg2	2.54919659	-0.3183397	0.4074477	-0.7813021	0.43462484	NA
Sgcz	2.52013617	0.31211629	0.3994996	0.78126809	0.43464484	NA
Rxfp1	3.03807549	-0.3251771	0.4162869	-0.781137	0.43472195	NA
Chrnb2	43.6359632	0.26906319	0.34446162	0.78111225	0.43473648	0.99938111
Cacul1	91.7662383	-0.2152005	0.27550793	-0.7811045	0.43474106	0.99938111
Zfp810	9.58907394	0.29245773	0.37441925	0.78109694	0.43474548	0.99938111
Atp2b3	82.5525502	0.27150462	0.34761227	0.78105591	0.43476961	0.99938111
Ptges3	80.3305042	0.19667474	0.2518468	0.78093008	0.43484362	0.99938111
Impad1	95.7009816	0.28370186	0.36332202	0.78085511	0.43488772	0.99938111
Efcab4b	0.47209514	0.21236775	0.27199858	0.78076784	0.43493905	NA
Pcgf3	34.6720981	-0.2416608	0.30952233	-0.780754	0.43494721	0.99938111
Ece1	32.248934	-0.2609593	0.33435648	-0.7804822	0.43510712	0.99938111
Ninl	1.16324817	0.28451395	0.36459923	0.7803471	0.43518659	NA
Kcnk12	7.1068907	0.32393912	0.4151769	0.7802436	0.4352475	0.99938111
Dmpk	2.56157157	-0.3143783	0.40293397	-0.780223	0.43525965	NA
Nr3c1	54.1530399	-0.2582354	0.3310135	-0.7801356	0.43531109	0.99938111
Fxn	1.77387286	-0.3024322	0.38766661	-0.7801347	0.4353116	NA
Ufsp2	34.6141135	0.27564691	0.35338821	0.7800116	0.43538405	0.99938111
Gm1976	16.5213995	-0.2556972	0.3278535	-0.7799131	0.43544202	0.99938111
Elmsan1	29.6104808	0.25986885	0.33325948	0.77977932	0.43552078	0.99938111
4632427E13Rik	0.64961685	0.22162383	0.28431811	0.77949247	0.43568967	NA
Tfb1m	3.10837225	0.3242901	0.4161178	0.77932281	0.43578958	NA
Zfp930	9.59161034	0.31856844	0.4088966	0.77909289	0.435925	0.99938111
Tubgcp5	12.1561783	-0.2897639	0.371963	-0.7790127	0.43597223	0.99938111
Eif4e3	14.4878369	0.27378197	0.3514991	0.77889807	0.43603976	0.99938111
Krt25	0.857409	-0.2581436	0.33149076	-0.7787356	0.43613551	NA
Ldoc1l	100.568007	0.28253489	0.36291203	0.7785217	0.43626152	0.99938111
Wbscr27	3.04509189	-0.3180784	0.40863924	-0.7783843	0.43634248	NA
Fig4	19.7175135	-0.262955	0.33787326	-0.7782651	0.43641273	0.99938111
Gm13446	2.1119377	0.30706072	0.39461933	0.7781188	0.43649898	NA
Bola2	12.4945603	-0.2912557	0.37436828	-0.7779924	0.43657348	0.99938111
Fbxl14	39.3059078	0.28630933	0.36811948	0.77776195	0.43670936	0.99938111
Dsc2	0.44537131	0.21091576	0.27122758	0.7776339	0.43678487	NA
Cul4b	59.6716379	0.27147276	0.34927195	0.77725325	0.43700937	0.99938111
Ngf	3.66490289	0.32314485	0.41582178	0.77712343	0.43708595	NA
Nup37	2.02624179	0.31715282	0.40811232	0.7771214	0.43708715	NA
Kcnt2	10.8236658	0.3109881	0.40023191	0.77701975	0.43714712	0.99938111
Asb16	1.69051896	0.31133431	0.40068423	0.77700665	0.43715485	NA
B3galt4	2.76754626	0.32325322	0.41602512	0.77700408	0.43715636	NA
1700086O06Rik	3.71672743	-0.307541	0.39582172	-0.7769685	0.43717737	NA
Pgbd1	1.94027738	-0.3162092	0.40705799	-0.7768161	0.43726725	NA
Agk	11.215752	0.28176031	0.36275824	0.77671649	0.43732606	0.99938111
Bex1	63.3234623	0.27193111	0.35011882	0.77668235	0.4373462	0.99938111
Ccdc47	22.3819266	0.31527509	0.40592623	0.77668076	0.43734714	0.99938111
Sp7	2.06708376	0.31888028	0.41066961	0.77648862	0.43746054	NA
Irf2bpl	109.531643	0.27770357	0.35768746	0.77638609	0.43752106	0.99938111
Rbfox3	233.560166	-0.2229557	0.2872579	-0.7761516	0.43765947	0.99938111
Triqk	12.9762566	0.27377936	0.35274491	0.77613979	0.43766645	0.99938111
Alkbh6	24.9055429	-0.2937005	0.37845957	-0.776042	0.43772421	0.99938111
Ppp1r2-ps3	4.33199752	-0.3166989	0.40812479	-0.7759855	0.43775753	NA
Ing4	16.3288776	0.2928174	0.37735318	0.77597703	0.43776255	0.99938111
Slc22a23	61.9396351	0.23641445	0.30475132	0.77576185	0.43788961	0.99938111
Cr2	0.81127923	0.26651182	0.3436087	0.77562592	0.43796989	NA
Amn	5.89004239	0.32266947	0.4160316	0.77558883	0.4379918	0.99938111
Cnnm3	17.3678706	-0.2833911	0.36546505	-0.7754259	0.43808805	0.99938111
Nrap	0.60471093	-0.2510657	0.32380314	-0.7753652	0.43812387	NA
1700008J07Rik	7.78033336	0.31884671	0.41131095	0.77519626	0.4382237	0.99938111
Rad50	6.97275346	0.32152512	0.41482882	0.77507904	0.43829296	0.99938111
U2surp	53.1512286	0.29576305	0.38167192	0.77491435	0.43839027	0.99938111
Satb2	18.232748	-0.2990305	0.38593126	-0.7748285	0.43844102	0.99938111
Ggct	6.22022576	0.31544158	0.40716313	0.77473021	0.4384991	0.99938111
Nat14	38.6564215	0.28636277	0.36971066	0.77455915	0.4386002	0.99938111
Fn3krp	35.7102625	0.21303035	0.27505569	0.77449897	0.43863578	0.99938111
Dpy19l4	14.3216349	0.29445784	0.38019974	0.77448194	0.43864584	0.99938111
Dnajc8	45.2553423	-0.2086489	0.26940797	-0.774472	0.43865172	0.99938111
Glipr1	1.61993604	-0.2987341	0.38581916	-0.7742852	0.43876213	NA
Suox	9.46828617	-0.2932913	0.37882454	-0.7742142	0.43880415	0.99938111
Cacna2d3	40.0987718	0.31232087	0.40353134	0.7739693	0.43894894	0.99938111
Bicd1	52.434308	0.26283215	0.33960946	0.77392471	0.43897532	0.99938111
Elf4	0.58959015	-0.2482265	0.32078435	-0.7738112	0.43904245	NA
Fzd7	3.96285866	-0.3221625	0.41636084	-0.773758	0.4390739	NA
Eif4g1	179.50611	0.18090709	0.23382029	0.77370141	0.43910738	0.99938111
Picalm	105.011786	-0.2422611	0.31312678	-0.7736837	0.43911789	0.99938111
Fbxo31	95.7339267	0.28803032	0.37230323	0.77364443	0.43914109	0.99938111

GeneID	Normalised expression for Chow#1	Normalised expression for Chow#2	Normalised expression for Chow#3	Normalised expression for Chow#4	Normalised expression for HFD#1	Normalised expression for HFD#2	Normalised expression for HFD#3	Normalised expression for HFD#4
Phactr1	379.034129	258.391044	287.861526	237.836101	314.649415	251.675668	197.264097	251.089028
Tmem116	0.67204633	1.81009488	1.90847421	4.13628002	0	1.17605452	1.92452778	0
Mettl3	7.39250961	9.95552182	12.7231614	10.3407001	9.98887032	9.40843619	4.81131944	6.84788259
Amz1	12.0968339	9.95552182	11.7689243	20.6814001	38.2906696	5.88027262	9.62263889	20.5436478
Mus81	6.72046328	2.26261859	5.72542262	10.3407001	4.99443516	2.35210905	3.84905555	4.56525506
Mar-10	0	0	0.9542371	2.06814001	0	0	0	9.13051012
B4galt5	38.3066407	64.2583681	38.4875632	76.5211804	58.2684102	51.7463991	24.0565972	38.804668
Nacc1	112.903783	86.4320303	101.785291	163.383061	179.799666	78.7956531	98.1509166	216.849615
Lgi2	36.2905017	47.5149905	54.7095939	18.6132601	26.6369875	119.957561	34.6415	31.9567854
Ccdc108	2.01613899	1.35757116	0.9542371	2.06814001	1.66481172	0	0.96226389	0
Ankrd29	12.7688802	14.9332827	14.3135566	10.3407001	8.3240586	21.1689814	25.0188611	11.4131377
Rab40c	18.1452509	20.3635674	22.9016905	28.9539601	41.620293	21.1689814	16.3584861	34.239413
Rac3	7.39250961	5.88280835	8.58813393	31.0221002	3.32962344	2.35210905	11.5471667	15.9783927
Alg9	11.4247876	23.5312334	15.5858727	12.4088401	11.653682	16.4647633	10.5849028	11.4131377
Vdac1	276.883087	195.037723	236.332723	241.972381	248.056946	231.682741	277.132	317.285227
Sdsl	0	2.26261859	0.31807903	6.20442003	1.66481172	1.17605452	0	0
Scmh1	43.010965	28.5089943	27.990955	10.3407001	19.9777406	28.2253086	19.2452778	22.8262753
Nid1	2.01613899	8.14542694	6.36158069	2.06814001	6.65924688	2.35210905	1.92452778	2.28262753
Agap2	982.531732	774.720607	772.295896	1319.47333	2017.75181	522.168209	529.245139	1947.08128
Lrriq3	0	0	0.9542371	2.06814001	0	2.35210905	0.96226389	2.28262753
Mtrf1	7.39250961	3.62018975	6.04350166	0	4.99443516	9.40843619	9.62263889	2.28262753
Havcr2	1.34409266	2.26261859	0	0	0	0	0	0
Cyr61	0.67204633	0.90504744	2.54463228	0	1.66481172	0	0	0
Twf2	23.5216215	11.313093	13.3593194	20.6814001	26.6369875	11.7605452	10.5849028	45.6525506
Clstn2	32.2582238	42.5372296	48.0299342	12.4088401	34.9610461	92.9083074	29.8301805	25.1089028
Syne1	172.715906	142.544971	147.588672	90.9981605	221.419959	196.401106	179.943347	77.6093361
Mgat5b	46.3711967	88.6946489	69.0231505	59.9760603	83.240586	52.9224536	24.0565972	54.7830607
Tsen15	9.4086486	9.95552182	6.04350166	8.27256004	1.66481172	4.7042181	12.5094306	2.28262753
Nqo1	1.34409266	1.35757116	1.27231614	2.06814001	0	0	1.92452778	0
Nkiras2	6.72046328	6.78785578	6.99773876	16.5451201	11.653682	0	5.77358333	6.84788259
Snhg7	0	0.45252372	0.63615807	2.06814001	1.66481172	1.17605452	1.92452778	0
Pim2	47.7152893	35.7493738	44.8491439	72.3849004	43.2851047	12.9365998	39.4528194	61.6309433
Sco2	12.0968339	9.95552182	10.8146872	22.7495401	13.3184938	2.35210905	10.5849028	13.6957652
Fam135a	20.1613899	19.9110436	21.6293743	8.27256004	18.3129289	31.7534722	34.6415	6.84788259
Commd5	10.7527413	6.78785578	7.95197586	6.20442003	13.3184938	5.88027262	9.62263889	15.9783927
Fam196a	29.5700384	17.1959013	20.0389792	20.6814001	9.98887032	19.9929269	25.981125	9.13051012
Nf1	211.694593	234.85981	271.321416	215.086561	276.358746	406.914865	242.4905	171.197065
Zfp106	222.447335	212.686148	199.117476	140.633521	173.140419	328.119212	250.188611	164.349182
Arg2	4.7043243	4.07271347	4.45310648	0	0	2.35210905	4.81131944	0
Sgcz	0	1.81009488	3.49886938	2.06814001	1.66481172	8.23238167	2.88679167	0
Rxfp1	3.36023164	6.33533207	3.49886938	2.06814001	1.66481172	3.52816357	3.84905555	0
Chrnb2	26.2098068	47.9675142	47.7118552	31.0221002	53.2739751	82.3238167	24.0565972	36.5220405
Cacul1	96.102625	96.8400759	94.4694732	113.747701	44.9499164	109.373071	101.037708	77.6093361
Zfp810	8.06455594	8.59795066	9.86045007	6.20442003	11.653682	5.88027262	17.32075	9.13051012
Atp2b3	65.8605402	99.1026944	86.5174974	37.2265202	83.240586	169.351851	52.9245139	66.1961984
Ptges3	69.2207718	70.5937002	73.794336	84.7937404	58.2684102	76.4435441	106.811292	102.718239
Impad1	65.1884939	119.013738	108.146872	39.2946602	66.5924688	206.985596	101.037708	59.3483158
Efcab4b	0	0	0.31807903	0	0	1.17605452	0	2.28262753
Pcgf3	45.027104	48.8725616	36.897168	20.6814001	23.3073641	30.5774176	42.3396111	29.6741579
Ece1	37.6345944	46.6099431	36.579089	22.7495401	33.2962344	44.6900719	25.0188611	11.4131377
Ninl	2.01613899	0.45252372	0.63615807	0	0	2.35210905	3.84905555	0
Kcnk12	5.37637063	3.16766603	2.86271131	10.3407001	13.3184938	3.52816357	0	18.2610202
Dmpk	2.01613899	2.71514231	1.27231614	8.27256004	3.32962344	0	2.88679167	0
Nr3c1	70.5648645	66.9735104	53.4372778	51.7035003	23.3073641	70.5632714	69.283	27.3915304
Fxn	2.68818531	2.26261859	3.49886938	0	0	1.17605452	0	4.56525506
Ufsp2	40.3227797	30.7716129	34.9886938	14.4769801	14.9833055	49.39429	57.7358333	34.239413
Gm1976	19.4893435	17.1959013	18.448584	18.6132601	11.653682	10.5844907	20.2075417	15.9783927
Elmsan1	26.8818531	28.0564706	18.766663	31.0221002	49.9443516	27.0492541	16.3584861	38.804668
4632427E13Ri	0	0.90504744	0	0	3.32962344	0	0.96226389	0
Tfb1m	2.68818531	3.16766603	3.18079035	0	4.99443516	4.7042181	3.84905555	2.28262753
Zfp930	6.72046328	6.78785578	12.7231614	2.06814001	0	17.6408179	30.7924444	0
Tubgcp5	17.4732045	11.313093	19.0847421	6.20442003	4.99443516	12.9365998	11.5471667	13.6957652
Eif4e3	9.4086486	14.480759	15.2677937	12.4088401	11.653682	15.2887088	25.981125	11.4131377
Krt25	0.67204633	0.45252372	0.63615807	4.13628002	0	0	0.96226389	0
Ldoc1l	65.8605402	71.0462239	74.4304941	138.565381	168.145984	49.39429	56.7735694	180.327575
Wbscr27	4.7043243	1.81009488	6.04350166	2.06814001	0	0	2.88679167	6.84788259
Fig4	25.5377605	26.6988994	20.6751372	14.4769801	8.3240586	16.4647633	25.0188611	20.5436478
Gm13446	3.36023164	1.35757116	1.27231614	0	0	7.05632714	3.84905555	0
Bola2	20.8334362	13.1231878	14.9497146	6.20442003	11.653682	4.7042181	12.5094306	15.9783927
Fbxl14	20.1613899	26.2463757	26.7186389	64.1123403	48.2795399	28.2253086	23.0943333	77.6093361
Dsc2	0	0	0.31807903	0	0	0	0.96226389	2.28262753
Cul4b	63.8444012	54.75537	63.6158069	26.8858201	26.6369875	103.492798	85.6414861	52.5004332
Ngf	4.03227797	2.26261859	4.45310648	0	0	8.23238167	5.77358333	4.56525506
Nup37	0.67204633	2.26261859	1.27231614	2.06814001	1.66481172	1.17605452	4.81131944	2.28262753
Kcnt2	10.0806949	11.7656167	11.7689243	2.06814001	9.98887032	9.40843619	26.9433889	4.56525506
Asb16	2.01613899	0.90504744	1.27231614	0	1.66481172	1.17605452	1.92452778	4.56525506
B3galt4	1.34409266	3.16766603	2.22655324	2.06814001	4.99443516	3.52816357	4.81131944	0
1700086O06R	0.67204633	1.35757116	2.54463228	16.5451201	1.66481172	1.17605452	5.77358333	0
Pgbd1	2.68818531	1.81009488	1.90847421	4.13628002	1.66481172	2.35210905	0.96226389	0
Agk	8.06455594	6.33533207	12.4050823	12.4088401	11.653682	9.40843619	13.4716944	15.9783927
Bex1	45.027104	26.6988994	41.3502745	113.747701	56.6035985	57.6266717	85.6414861	79.8919636
Ccdc47	12.7688802	25.3413283	27.0367179	8.27256004	6.65924688	63.5069443	24.0565972	11.4131377
Sp7	2.01613899	2.71514231	0.9542371	0	3.32962344	2.35210905	2.88679167	2.28262753
Irf2bpl	86.6939764	69.236129	66.4785182	161.314921	189.788536	70.5632714	58.6980972	173.479692
Rbfox3	279.571273	266.083947	222.337245	246.108661	253.051381	150.534979	128.943361	321.850482
Triqk	14.7850192	8.59795066	13.9954775	8.27256004	13.3184938	16.4647633	19.2452778	9.13051012
Alkbh6	20.8334362	27.1514231	16.5401098	49.6353602	19.9777406	7.05632714	19.2452778	38.804668
Ppp1r2-ps3	6.04841696	5.43028463	5.08926455	4.13628002	3.32962344	3.52816357	4.81131944	2.28262753
Ing4	10.0806949	9.05047438	8.90621297	31.0221002	13.3184938	12.9365998	20.2075417	25.1089028
Slc22a23	43.6830113	82.3593168	58.5265423	37.2265202	68.2572805	96.436471	51.96225	57.0656883
Cr2	0	0.45252372	1.27231614	0	1.66481172	1.17605452	1.92452778	0
Amn	0.67204633	3.16766603	3.81694841	12.4088401	11.653682	4.7042181	3.84905555	6.84788259
Cnnm3	10.7527413	29.4140417	18.448584	18.6132601	21.6425524	14.1126543	7.69811111	18.2610202
Nrap	2.01613899	0.90504744	0.9542371	0	0	0	0.96226389	0
1700008J07Ri	2.68818531	1.35757116	1.90847421	18.6132601	18.3129289	4.7042181	0.96226389	13.6957652
Rad50	9.4086486	7.69290322	3.49886938	2.06814001	4.99443516	11.7605452	16.3584861	0
U2surp	73.2530498	45.2523719	48.3480132	14.4769801	26.6369875	92.9083074	92.3773333	31.9567854
Satb2	30.2420848	24.8888045	22.5836114	4.13628002	8.3240586	9.40843619	21.1698055	25.1089028
Ggct	5.37637063	4.07271347	6.04350166	6.20442003	3.32962344	7.05632714	15.3962222	2.28262753
Nat14	21.5054825	21.2686148	24.8101647	68.2486203	59.9332219	25.8731995	25.981125	61.6309433
Fn3krp	37.6345944	31.6766603	31.4898244	28.9539601	28.3017992	34.1055812	43.301875	50.2178057
Dpy19l4	16.1291119	13.5757116	11.7689243	8.27256004	6.65924688	28.2253086	23.0943333	6.84788259
Dnajc8	42.3389187	51.1351802	41.3502745	62.0442003	31.6314227	43.5140174	37.5282917	52.5004332
Glipr1	1.34409266	5.43028463	1.90847421	0	0	2.35210905	1.92452778	0
Suox	9.4086486	11.313093	7.31581779	16.5451201	9.98887032	4.7042181	9.62263889	6.84788259
Cacna2d3	33.6023164	46.1574193	38.4875632	14.4769801	13.3184938	116.429398	42.3396111	15.9783927
Bicd1	55.1077989	41.6321821	41.9864326	49.6353602	33.2962344	69.3872169	101.037708	27.3915304
Elf4	0.67204633	1.81009488	1.27231614	0	0	0	0.96226389	0
Fzd7	3.36023164	7.2403795	3.81694841	4.13628002	6.65924688	0	1.92452778	4.56525506
Eif4g1	185.484787	176.936774	159.357596	144.769801	244.727323	208.161651	138.566	178.044947
Picalm	129.704941	132.58945	125.959298	74.4530404	48.2795399	132.894161	123.169778	73.044081
Fbxo31	49.7314283	61.5432258	55.663831	165.451201	163.151549	52.9224536	64.4716805	152.936045

GeneID	Base mean	log2(FC)	StdErr	Wald-Stats	P-value	P-adj		GeneID	Normalised expression for Chow#1	Normalised expression for Chow#2	Normalised expression for Chow#3	Normalised expression for Chow#4	Normalised expression for HFD#1	Normalised expression for HFD#2	Normalised expression for HFD#3	Normalised expression for HFD#4
Sdhaf1	8.07891771	0.31326643	0.40497988	0.77353578	0.43920536	0.99938111		Sdhaf1	10.0806949	6.78785578	6.04350166	2.06814001	4.99443516	3.52816357	10.5849028	20.5436478
Stx6	47.4555228	-0.2274404	0.29403753	-0.773508	0.4392218	0.99938111		Stx6	63.8444012	48.8725616	47.7118552	43.4309402	46.6147282	34.1055812	28.8679167	66.1961984
Pml	8.42477003	-0.2971578	0.38422377	-0.7733977	0.43928705	0.99938111		Pml	10.0806949	7.69290322	9.54237104	10.3407001	9.98887032	3.52816357	4.81131944	11.4131377
Hypk	20.34298	0.27641829	0.35767787	0.77281351	0.43963276	0.99938111		Hypk	29.5700384	15.3858064	11.1327662	16.5451201	16.6481172	27.0492541	32.7169722	13.6957652
Gm12992	44.8016307	0.30471383	0.39437816	0.77264377	0.43973323	0.99938111		Gm12992	31.5861774	23.5312334	21.9474534	74.4530404	41.620293	19.9929269	28.8679167	116.414004
Ubxn2b	36.6621204	-0.2765433	0.35801838	-0.7724277	0.43986115	0.99938111		Ubxn2b	40.3227797	47.0624668	49.9384084	28.9539601	18.3129289	58.8027262	38.4905555	11.4131377
Sh3gl2	185.405504	0.20194613	0.26146021	0.77237806	0.43989054	0.99938111		Sh3gl2	190.861157	172.411537	203.570582	113.747701	221.419959	142.302597	254.037667	184.89283
2900041M22Rik	1.12234137	0.28263819	0.36606682	0.77209453	0.44005844	NA		2900041M22Ri	0.67204633	0.45252372	0.31807903	2.06814001	3.32962344	1.17605452	0.96226389	0
Cspp1	3.15034643	0.30818618	0.39915738	0.77209189	0.44006001	NA		Cspp1	2.68818531	1.81009488	4.45310648	0	0	4.7042181	11.5471667	0
Kif1b	771.685345	0.22246392	0.28818725	0.77194227	0.44014862	0.99938111		Kif1b	506.050885	762.049943	633.931516	909.981605	1083.79243	969.068928	450.3395	858.267952
Man2a2	149.370655	0.22871929	0.2963056	0.77190337	0.44017166	0.99938111		Man2a2	105.511274	171.959013	140.909012	122.020261	206.436653	210.51376	84.6792222	152.936045
Arhgap29	10.0841301	-0.310717	0.40254761	-0.7718763	0.44018767	0.99938111		Arhgap29	11.4247876	21.7211385	6.99773876	6.20442003	11.653682	12.9365998	2.88679167	6.84788259
Gale	5.60437161	0.32072524	0.41556629	0.77177876	0.44024547	0.99938111		Gale	4.03227797	2.71514231	1.90847421	10.3407001	1.66481172	4.7042181	5.77358333	13.6957652
Tnfaip8l3	21.5746058	0.24851145	0.32203885	0.77168158	0.44030304	0.99938111		Tnfaip8l3	18.1452509	23.0787097	20.9932163	14.4769801	14.9833055	21.1689814	34.6415	25.1089028
Ppp3ca	415.372667	-0.2559502	0.33167903	-0.7716805	0.4403037	0.99938111		Ppp3ca	678.094745	496.41852	487.297081	181.996321	316.314227	432.788065	492.679111	237.393263
Ccnd2	60.4939988	0.2051646	0.26590524	0.77157034	0.44036894	0.99938111		Ccnd2	63.8444012	53.3977988	51.8468826	51.7035003	68.2572805	41.1619083	69.283	84.4572186
Ctr9	29.835633	0.23868263	0.30940166	0.77143292	0.44045037	0.99938111		Ctr9	23.5216215	25.793852	38.4875632	18.6132601	29.966611	43.5140174	33.6792361	25.1089028
Ptcd3	22.8705419	0.28982214	0.37577429	0.77126654	0.44054896	0.99938111		Ptcd3	21.5054825	28.0564706	22.2655324	6.20442003	11.653682	38.8097993	38.4905555	15.9783927
Ptpn23	56.3965658	0.28417018	0.36858738	0.770971	0.44072412	0.99938111		Ptpn23	36.2905017	34.3918026	42.6225906	82.7256004	93.2294563	25.8731995	35.6037639	100.435611
Tfap2d	0.90012252	-0.2790523	0.36199596	-0.7708713	0.44078325	NA		Tfap2d	1.34409266	1.81009488	1.90847421	0	0	1.17605452	0.96226389	0
Dcdc2b	1.85637268	-0.3015731	0.39136715	-0.7705632	0.44096585	NA		Dcdc2b	4.7043243	3.62018975	1.27231614	0	3.32962344	0	1.92452778	0
0610040F04Rik	1.09352454	-0.2807006	0.36457738	-0.7699342	0.4413389	NA		0610040F04Ri	2.01613899	1.35757116	0.9542371	2.06814001	0	2.35210905	0	0
Frmd7	0.9360051	0.25647683	0.33316715	0.76981429	0.44141006	NA		Frmd7	1.34409266	0.90504744	0	0	0	2.35210905	2.88679167	0
Acsl3	75.0701545	-0.3130557	0.40668921	-0.7697664	0.44143848	0.99938111		Acsl3	73.2530498	157.930778	101.467212	20.6814001	41.620293	139.950488	51.96225	13.6957652
Gtl3	24.3788879	-0.2442835	0.31741549	-0.7696016	0.44153623	0.99938111		Gtl3	24.8657142	31.2241366	30.2175083	18.6132601	13.3184938	22.345036	20.2075417	34.239413
Ggnbp2	49.5387703	0.26717052	0.3472458	0.76939886	0.44165657	0.99938111		Ggnbp2	45.6991503	41.6321821	51.5288036	37.2265202	29.966611	84.6759257	82.7546944	22.8262753
Snx22	11.1648253	-0.2958275	0.38451824	-0.7693457	0.44168811	0.99938111		Snx22	14.1129729	14.9332827	15.2677937	6.20442003	8.3240586	18.8168724	4.81131944	6.84788259
Gmeb2	14.5275734	0.27885272	0.36247251	0.76930722	0.44171095	0.99938111		Gmeb2	8.73660227	9.05047438	11.4508452	22.7495401	18.3129289	10.5844907	12.5094306	22.8262753
Riok2	10.4429156	0.30995328	0.40314431	0.76883952	0.44198858	0.99938111		Riok2	8.73660227	13.1231878	10.4966081	2.06814001	4.99443516	16.4647633	23.0943333	4.56525506
Ireb2	57.8970731	0.2674794	0.3479143	0.7688083	0.44200712	0.99938111		Ireb2	43.010965	52.4927514	60.7530956	49.6353602	36.6258578	96.436471	99.1131805	25.1089028
Vcan	8.04626318	0.31502833	0.40982237	0.76869482	0.4420745	0.99938111		Vcan	3.36023164	10.4080455	7.95197586	2.06814001	1.66481172	34.1055812	4.81131944	0
Wdr6	354.639351	0.31272451	0.40689446	0.76856419	0.44215207	0.99938111		Wdr6	169.355675	107.248121	146.316356	746.598544	700.885734	189.344778	158.773542	618.592061
Lamtor4	12.6582297	-0.3104907	0.40430448	-0.7679626	0.44250938	0.99938111		Lamtor4	12.7688802	8.14542694	5.08926455	35.1583802	11.653682	7.05632714	7.69811111	13.6957652
Spata1	0.83831153	0.24581151	0.32017008	0.76775292	0.44263399	NA		Spata1	0.67204633	0.90504744	0.31807903	0	0	0	4.81131944	0
Mapre3	201.518988	0.22923074	0.29862944	0.76760932	0.44271932	0.99938111		Mapre3	192.877296	144.80759	160.629912	231.631681	229.744017	148.18287	148.188639	356.089895
Cstf2	46.4047033	0.21772821	0.28366798	0.76754597	0.44275697	0.99938111		Cstf2	43.6830113	42.0847059	50.5745665	33.0902402	36.6258578	70.5632714	55.8113055	38.804668
Cdhr1	6.39899017	-0.3152072	0.41069258	-0.7675016	0.44278334	0.99938111		Cdhr1	4.7043243	16.2908539	10.8146872	0	6.65924688	11.7605452	0.96226389	0
Etfa	3.1011754	-0.3184762	0.41499704	-0.767418	0.44283302	NA		Etfa	3.36023164	3.62018975	3.49886938	6.20442003	0	2.35210905	5.77358333	0
Trmt10a	2.83216756	0.31915921	0.41616551	0.76690452	0.44313829	NA		Trmt10a	2.68818531	3.62018975	1.90847421	0	4.99443516	2.35210905	4.81131944	2.28262753
Napg	106.074988	0.22760402	0.29679628	0.76686953	0.44315909	0.99938111		Napg	111.559691	98.6501707	112.599978	62.0442003	93.2294563	163.471579	138.566	68.4788259
Gstm2	0.88833273	-0.2466011	0.32167145	-0.7666241	0.44330506	NA		Gstm2	2.01613899	1.35757116	0	2.06814001	1.66481172	0	0	0
Xrn1	20.5852742	0.31496129	0.41092944	0.76646076	0.44340219	0.99938111		Xrn1	22.1775288	16.7433776	23.5378486	4.13628002	33.2962344	38.8097993	25.981125	0
Snx5	35.1769832	-0.2753342	0.35923207	-0.766452	0.44340737	0.99938111		Snx5	55.7798453	41.6321821	48.0299342	12.4088401	24.9721758	27.0492541	50.9999861	20.5436478
Pde7a	9.90454055	-0.3171361	0.41381972	-0.7663629	0.44346042	0.99938111		Pde7a	24.1936678	8.59795066	16.5401098	0	0	7.05632714	18.2830139	4.56525506
Lipt2	3.77583109	-0.3186303	0.41581802	-0.7662734	0.44351366	NA		Lipt2	4.7043243	3.62018975	2.54463228	8.27256004	3.32962344	3.52816357	1.92452778	2.28262753
Ccar2	73.4406576	0.24106943	0.31461426	0.76623808	0.44353465	0.99938111		Ccar2	62.5003085	41.6321821	55.027673	107.543281	109.877574	59.9787807	59.6603611	91.3051012
4932702P03Rik	9.4086969	0.3185704	0.41591338	0.76595373	0.44370383	0.99938111		4932702P03Ri	1.34409266	3.16766603	3.49886938	22.7495401	19.9777406	4.7042181	3.84905555	15.9783927
Dennd3	2.09612109	-0.3114563	0.40667287	-0.7658646	0.4437569	NA		Dennd3	3.36023164	1.81009488	1.59039517	4.13628002	1.66481172	0	1.92452778	2.28262753
Mrpl18	12.524916	0.31224283	0.40775716	0.76575684	0.443821	0.99938111		Mrpl18	18.1452509	9.05047438	10.1785291	4.13628002	13.3184938	9.40843619	33.6792361	2.28262753
Tbc1d12	13.0209983	0.28219322	0.36854852	0.76568811	0.44386191	0.99938111		Tbc1d12	16.1291119	10.4080455	12.4050823	6.20442003	6.65924688	18.8168724	22.1320694	11.4131377
C1qtnf5	5.28178976	-0.3169758	0.41402028	-0.7656046	0.44391159	0.99938111		C1qtnf5	8.73660227	5.43028463	2.86271131	8.27256004	8.3240586	1.17605452	2.88679167	4.56525506
Mfrp	5.28178976	-0.3169758	0.41402028	-0.7656046	0.44391159	0.99938111		Mfrp	8.73660227	5.43028463	2.86271131	8.27256004	8.3240586	1.17605452	2.88679167	4.56525506
Slc11a2	18.1933544	-0.2622106	0.34254299	-0.7654823	0.44398441	0.99938111		Slc11a2	17.4732045	25.793852	25.7644018	10.3407001	14.9833055	11.7605452	21.1698055	18.2610202
Nup62-il4i1	1.52233318	-0.284419	0.37158436	-0.7654225	0.44402003	NA		Nup62-il4i1	2.68818531	0.45252372	0.9542371	4.13628002	1.66481172	0	0	2.28262753
Mkks	24.3502099	-0.2896631	0.37843966	-0.7654143	0.44402487	0.99938111		Mkks	36.9625481	35.2968501	30.5355873	8.27256004	9.98887032	30.5774176	31.7547083	11.4131377
Lin37	8.22875522	0.30089702	0.39313468	0.76537898	0.44404591	0.99938111		Lin37	6.72046328	9.5029981	6.67965972	4.13628002	4.99443516	4.7042181	15.3962222	13.6957652
Hs1bp3	7.32357761	-0.2973924	0.3885868	-0.7653179	0.44408226	0.99938111		Hs1bp3	8.73660227	9.5029981	9.86045007	4.13628002	9.98887032	4.7042181	4.81131944	6.84788259
Cdkl5	72.8422969	-0.3016219	0.39425147	-0.7650496	0.44424203	0.99938111		Cdkl5	104.167181	107.700645	106.556477	18.6132601	41.620293	111.72518	74.0943194	18.2610202
Gm17066	7.80209984	0.30624859	0.4003537	0.76494509	0.44430424	0.99938111		Gm17066	8.06455594	4.97776091	9.224292	4.13628002	3.32962344	11.7605452	16.3584861	4.56525506
Apool	1.42496963	-0.2979186	0.38960558	-0.7646673	0.4444697	NA		Apool	0.67204633	1.35757116	3.49886938	2.06814001	1.66481172	1.17605452	0.96226389	0
Il16	1.04749437	0.27040765	0.353766	0.76436867	0.44464757	NA		Il16	2.01613899	0	0.63615807	0	1.66481172	1.17605452	2.88679167	0
4930507D05Rik	6.82719682	0.29355362	0.38407324	0.76431677	0.44467849	0.99938111		4930507D05Ri	0.67204633	0.45252372	1.59039517	16.5451201	8.3240586	0	1.92452778	25.1089028
Fam98b	15.4190473	0.27192461	0.35586724	0.76411814	0.44479684	0.99938111		Fam98b	10.7527413	15.3858064	13.0412404	16.5451201	9.98887032	14.1126543	29.8301805	13.6957652
Rassf5	27.5788304	0.23327233	0.30533484	0.76398858	0.44487405	0.99938111		Rassf5	23.5216215	17.19590	18.766663	33.0902402	28.3017992	19.9929269	34.6415	36.5220405
Tufm	8.74551507	0.31745869	0.41553227	0.76398082	0.44487867	0.99938111		Tufm	4.7043243	2.71514231	3.18079035	18.6132601	11.653682	4.7042181	3.84905555	20.5436478
Zfp763	12.9695441	0.30837787	0.40367849	0.76391869	0.4449157	0.99938111		Zfp763	13.4409266	11.7656167	15.2677937	2.06814001	3.32962344	24.697145	24.0565972	9.13051012
Sall2	91.800934	0.2355464	0.3083692	0.76384543	0.44495936	0.99938111		Sall2	63.8444012	74.6664136	66.1604392	128.224681	114.872009	92.9083074	56.7735694	136.957652
Tesc	19.4358909	-0.263062	0.34442027	-0.763782	0.44499715	0.99938111		Tesc	28.2259458	12.6706641	19.7209001	26.8858201	16.6481172	10.5844907	20.2075417	20.5436478
Abcd2	16.5569761	-0.3016036	0.39488772	-0.7637706	0.44500398	0.99938111		Abcd2	19.4893435	28.5089943	20.6751372	8.27256004	3.32962344	27.0492541	18.2830139	6.84788259
Pik3r4	26.1579567	0.24258911	0.31765772	0.76368082	0.44505748	0.99938111		Pik3r4	26.8818531	20.3635674	22.9016905	22.7495401	43.2851047	27.0492541	16.3584861	29.6741579
Nemf	22.5775793	0.30966067	0.40554007	0.763576	0.44511996	0.99938111		Nemf	24.8657142	15.8383302	22.2655324	22.7495401	11.653682	57.6266717	33.6792361	2.28262753
Npy2r	15.76853	0.30466122	0.39900642	0.76354967	0.44513565	0.99938111		Npy2r	8.06455594	19.9110436	18.130505	6.20442003	13.3184938	41.1619083	12.5094306	6.84788259
Alg11	31.1840806	-0.2646495	0.34672768	-0.7632777	0.44529779	0.99938111		Alg11	27.5538995	50.6826565	36.897168	24.8176801	13.3184938	42.3379629	35.6037639	18.2610202
Map4	439.442967	0.15023694	0.19688052	0.76308688	0.44541158	0.99938111		Map4	409.94826	428.992486	447.537202	372.265202	379.577072	421.02752	549.45268	506.743312
Eaf1	12.9050558	-0.2812946	0.36879802	-0.7627335	0.44562234	0.99938111		Eaf1	12.0968339	17.1959013	11.7689243	18.6132601	9.98887032	19.9929269	6.73584722	6.84788259
Anp32b	10.9786806	-0.2851257	0.37384818	-0.7626778	0.4456556	0.99938111		Anp32b	9.4086486	9.95552182	11.4508452	20.6814001	9.98887032	4.7042181	12.5094306	9.13051012
Gm12657	3.28993553	-0.3174773	0.41636908	-0.7624901	0.44576752	NA		Gm12657	3.36023164	4.52523719	6.36158069	2.06814001	1.66481172	3.52816357	4.81131944	0
Hist2h2bb	13.6156909	-0.2978211	0.39064108	-0.7623907	0.44582686	0.99938111		Hist2h2bb	23.5216215	17.648425	15.2677937	6.20442003	3.32962344	9.40843619	22.1320694	11.4131377
Pde3a	6.24799726	0.31221718	0.40960562	0.76223853	0.44591766	0.99938111		Pde3a	4.03227797	8.14542694	4.45310648	4.13628002	1.66481172	15.2887088	7.69811111	4.56525506
Cog1	23.8847343	0.22766607	0.29868311	0.76223281	0.44592106	0.99938111		Cog1	23.5216215	18.5534725	20.6751372	24.8176801	34.9610461	25.8731995	22.1320694	20.5436478
Ghrl	0.69521685	-0.2573387	0.3377789	-0.7618554	0.44614629	NA		Ghrl	0.67204633	0.90504744	0.9542371	2.06814001	0	0	0.96226389	0
Apoo	4.78232067	-0.317159	0.41631563	-0.7618234	0.44616538	0.99938111		Apoo	9.4086486	8.59795066	3.49886938	2.06814001	0	4.7042181	7.69811111	2.28262753
Atg4b	34.7150639	0.27285699	0.35825433	0.76162927	0.4462813	0.99938111		Atg4b	32.9302701	23.5312334	21.9474534	43.4309402	64.9276571	17.6408179	23.0943333	50.2178057
Ppfibp1	12.3239942	-0.2797663	0.36736393	-0.7615509	0.44632811	0.99938111		Ppfibp1	17.4732045	11.313093	17.4943469	10.3407001	8.3240586	11.7605452	17.32075	4.56525506
Rnf41	85.4897966	0.25853057	0.33951299	0.76147475	0.44637356	0.99938111		Rnf41	62.5003085	48.8725616	64.988123	128.224681	86.5702095	61.1548353	67.3584722	164.349182
Fam35a	4.89667022	0.30806436	0.40466358	0.76128511	0.44648679	0.99938111		Fam35a	3.36023164	3.62018975	5.40734359	4.13628002	3.32962344	7.05632714	7.69811111	4.56525506
Rnf138	5.45383149	-0.3163231	0.41552607	-0.7612593	0.44650223	0.99938111		Rnf138	10.0806949	7.69290322	8.2700549	0	3.32962344	2.35210905	9.62263889	2.28262753
Ppm1d	12.0019631	0.26500985	0.3481465	0.7612021	0.44653636	0.99938111		Ppm1d	9.4086486	10.4080455	12.4050823	10.3407001	8.3240586	14.1126543	17.32075	13.6957652
Atp1b1	966.230637	0.26591139	0.34939512	0.76106212	0.44661996	0.99938111		Atp1b1	728.49822	1178.37176	1020.07946	477.740342	581.01929	2094.55311	868.924291	780.658616
4930563E22Rik	2.76651955	-0.2905414	0.38183676	-0.7609047	0.446714	NA		4930563E22Ri	1.34409266	1.35757116	0.31807903	12.4088401	0	1.17605452	0.96226389	4.56525506
Exoc7	39.403206	0.21664832	0.28474515	0.76084991	0.44674672	0.99938111		Exoc7	45.027104	35.2968501	37.215247	24.8176801	48.2795399	30.5774176	52.9245139	41.0872956
Kdelc2	3.79116146	-0.3166841	0.41627726	-0.7607529	0.44680469	NA		Kdelc2	6.04841696	6.78785578	3.81694841	2.06814001	1.66481172	7.05632714	2.88679167	0
Crabp2	0.80598962	-0.2585437	0.33988246	-0.7606857	0.44684481	NA		Crabp2	0.67204633	0.45252372	1.59039517	2.06814001	1.66481172	0	0	0
Zfp119a	2.12836439	0.30412297	0.39980154	0.76068485	0.44684532	NA		Zfp119a	0.67204633	1.35757116	3.81694841	0	1.66481172	4.7042181	4.81131944	0
A630007B06Rik	23.5336322	0.30942204	0.40676973	0.76068107	0.44684758	0.99938111		A630007B06Ri	22.1775288	18.5534725	27.0367179	24.8176801	11.653682	52.9224536	43.301875	2.28262753
Ctu1	5.6596375	-0.3150803	0.41421991	-0.7606595	0.44686047	0.99938111		Ctu1	4.7043243	4.07271347	4.45310648	14.4769801	6.65924688	1.17605452	2.88679167	6.84788259
Edn3	2.40191155	-0.2972687	0.39081471	-0.7606384	0.44687306	NA		Edn3	0.67204633	6.33533207	2.86271131	2.06814001	4.99443516	0	0	2.28262753
Mterfd1	8.41895235	-0.3144448	0.41354292	-0.760368	0.44703466	0.99938111		Mterfd1	12.0968339	12.6706641	15.2677937	0	3.32962344	8.23238167	13.4716944	2.28262753
Ano3	49.0161615	-0.3092946	0.40679005	-0.7603298	0.4470575	0.99938111		Ano3	58.4680306	85.9795066	79.2016796	6.20442003	23.3073641	87.0280348	33.6792361	18.2610202
Dnaja2	118.614893	0.25203873	0.3315254	0.76023958	0.44711139	0.99938111		Dnaja2	143.145868	100.460266	113.554215	66.1804803	71.586904	189.344778	184.754667	79.8919636
Mrps18c	1.33380366	-0.2935926	0.38658265	-0.7594563	0.44757967	NA		Mrps18c	2.68818531	0.90504744	1.90847421	2.06814001	0	1.17605452	1.92452778	0
Rsrc1	26.9278148	0.25872642	0.34068617	0.75942744	0.4475969	0.99938111		Rsrc1	24.8657142	19.4585199	22.2655324	31.0221002	11.653682	27.0492541	47.1509305	31.9567854
Cers1	89.3166176	-0.2760438	0.36349145	-0.759423	0.44759957	0.99938111		Cers1	79.9735131	115.846072	75.3847312	130.292821	116.53682	48.2182355	25.0188611	123.261887
Gdf1	89.3166176	-0.2760438	0.36349145	-0.759423	0.44759957	0.99938111		Gdf1	79.9735131	115.846072	75.3847312	130.292821	116.53682	48.2182355	25.0188611	123.261887
Dpyd	4.36192663	0.3061048	0.40309729	0.75938193	0.44762412	NA		Dpyd	3.36023164	4.52523719	4.77118552	0	0	5.88027262	16.3584861	0
Prima1	1.13853902	-0.2800112	0.36888758	-0.7590693	0.44781108	NA		Prima1	1.34409266	2.71514231	1.59039517	0	0	1.17605452	0	2.28262753
Polr2d	5.51904718	-0.3145896	0.41451894	-0.7589269	0.44789626	0.99938111		Polr2d	9.4086486	8.59795066	6.36158069	2.06814001	0	3.52816357	9.62263889	4.56525506
Mkl2	267.104384	-0.2193465	0.28903864	-0.7588831	0.44792249	0.99938111		Mkl2	310.485404	350.253358	342.889199	157.178641	283.017992	305.774176	225.16975	162.066555
Slc5a3	66.5148408	0.27362992	0.36058473	0.75885056	0.44794196	0.99938111		Slc5a3	37.6345944	95.9350284	56.2999891	41.3628002	63.2628454	130.542052	31.7547083	75.3267085
D17H6S53E	16.7806857	0.29034861	0.38278548	0.75851521	0.44814261	0.99938111		D17H6S53E	12.0968339	10.8605693	8.58813393	26.8858201	19.9777406	12.9365998	8.660375	34.239413
M6pr	35.6786119	-0.2608498	0.34403522	-0.7582066	0.44832732	0.99938111		M6pr	33.6023164	63.8058444	41.6683535	18.6132601	24.9721758	49.39429	25.981125	27.3915304
Ddx4	1.51217271	0.28043316	0.36989793	0.7581366	0.44836921	NA		Ddx4	0	3.16766603	0.63615807	0	1.66481172	4.7042181	1.92452778	0

GeneID	Base mean	log2(FC)	StdErr	Wald-Stats	P-value	P-adj		GeneID	Normalised expression for Chow#1	Normalised expression for Chow#2	Normalised expression for Chow#3	Normalised expression for Chow#4	Normalised expression for HFD#1	Normalised expression for HFD#2	Normalised expression for HFD#3	Normalised expression for HFD#4
Nubp2	12.101114	-0.2929159	0.38644008	-0.7579853	0.44845977	0.99938111		Nubp2	14.1129729	9.05047438	9.86045007	22.7495401	16.6481172	5.88027262	4.81131944	13.6957652
D430036J16Rik	3.49076379	-0.314947	0.41556915	-0.757869	0.44852941	NA		D430036J16Ri	4.7043243	6.33533207	4.13502745	2.06814001	1.66481172	0	6.73584722	2.28262753
Hes1	3.617329	-0.3151841	0.415958	-0.7577305	0.44861235	NA		Hes1	3.36023164	2.71514231	2.86271131	10.3407001	0	3.52816357	3.84905555	2.28262753
Rerg	15.3685097	0.2732255	0.36059172	0.75771428	0.44862205	0.99938111		Rerg	16.8011582	9.5029981	12.0870033	14.4769801	13.3184938	9.40843619	15.3962222	31.9567854
Epb4.1l3	145.07345	0.26251871	0.34652666	0.75757148	0.44870756	0.99938111		Epb4.1l3	170.027721	116.751119	149.179067	76.5211804	84.9053977	304.598122	155.88675	102.718239
Suv420h1	38.4002416	0.21951653	0.28982355	0.75741441	0.44880163	0.99938111		Suv420h1	39.6507334	40.274611	36.579089	22.7495401	46.6147282	52.9224536	43.301875	25.1089028
Foxm1	1.2722793	-0.2800645	0.3697859	-0.7573694	0.44882861	NA		Foxm1	2.01613899	1.81009488	0.9542371	2.06814001	3.32962344	0	0	0
Tub	136.75547	0.18893357	0.24947674	0.7573194	0.44885853	0.99938111		Tub	108.871505	112.678406	104.329923	186.132601	171.475607	144.654706	128.943361	136.957652
Dnph1	1.91423538	0.30142705	0.39821041	0.75695422	0.44907729	NA		Dnph1	1.34409266	0	2.22655324	2.06814001	3.32962344	1.17605452	2.88679167	2.28262753
Zfp827	55.1042967	-0.2030275	0.26825718	-0.7568389	0.44914637	0.99938111		Zfp827	67.2046328	53.8503226	48.6660923	70.3167603	61.5980337	59.9787807	40.4150833	38.804668
Clk4	7.38447859	0.30281295	0.40012941	0.75678756	0.44917715	0.99938111		Clk4	9.4086486	8.14542694	5.08926455	2.06814001	4.99443516	9.40843619	15.3962222	4.56525506
Parp1	36.9324705	0.21483951	0.2839181	0.75669536	0.44923239	0.99938111		Parp1	47.7152893	27.1514231	28.9451921	31.0221002	39.9554813	38.8097993	38.4905555	43.3699231
Tyw5	4.15732922	0.31401918	0.41502575	0.75662579	0.44927409	NA		Tyw5	2.01613899	4.52523719	4.77118552	2.06814001	1.66481172	8.23238167	7.69811111	2.28262753
Myh11	0.78172447	0.2341553	0.30953902	0.75646455	0.44937072	NA		Myh11	1.34409266	0	0	0	1.66481172	0	0.96226389	2.28262753
Immp2l	2.24992422	0.30724955	0.40629099	0.75623028	0.44951114	NA		Immp2l	1.34409266	1.81009488	2.54463228	0	0	3.52816357	1.92452778	6.84788259
Sep-06	37.0283221	0.22263178	0.29450076	0.75596335	0.44967117	0.99938111		Sep-06	45.6991503	25.793852	37.8514051	24.8176801	36.6258578	51.7463991	39.4528194	34.239413
Tc2n	0.36084896	0.14700353	0.19447265	0.75590853	0.44970404	NA		Tc2n	0	0	0	0	0	0	2.88679167	0
Tectb	0.36084896	0.14700353	0.19447265	0.75590853	0.44970404	NA		Tectb	0	0	0	0	0	0	2.88679167	0
Wdr73	20.3182875	0.24275799	0.32116261	0.75587252	0.44972563	0.99938111		Wdr73	18.1452509	12.6706641	18.448584	24.8176801	24.9721758	15.2887088	23.0943333	25.1089028
Baiap2l2	4.09285228	0.31466672	0.41630622	0.75585399	0.44973674	NA		Baiap2l2	6.04841696	2.26261859	1.90847421	2.06814001	1.66481172	3.52816357	3.84905555	11.4131377
Ap4b1	9.96663729	0.31014071	0.41043706	0.75563524	0.44986792	0.99938111		Ap4b1	10.7527413	6.33533207	9.54237104	4.13628002	14.9833055	1.17605452	7.69811111	25.1089028
Gsk3b	450.978744	0.18657972	0.24693681	0.75557678	0.44990298	0.99938111		Gsk3b	348.792044	365.639165	377.559814	585.283623	494.449081	377.513502	444.565916	614.026806
Npy5r	1.94634883	-0.3075133	0.40699753	-0.7555654	0.4499098	NA		Npy5r	3.36023164	3.62018975	2.86271131	0	1.66481172	1.17605452	2.88679167	0
Srrm4	53.5568424	0.24340671	0.32218216	0.75549406	0.4499526	0.99938111		Srrm4	42.3389187	37.5594687	38.8056422	74.4530404	61.5980337	52.9224536	31.7547083	89.0224737
Gpr89	20.0636348	-0.2724915	0.36068007	-0.7554936	0.44995289	0.99938111		Gpr89	14.7850192	29.4140417	22.5836114	22.7495401	16.6481172	27.0492541	6.73584722	20.5436478
Txn2	116.66928	-0.2347882	0.31078393	-0.7554709	0.44996647	0.99938111		Txn2	106.855366	106.343074	108.78303	190.268881	129.855314	58.8027262	81.7924305	150.653417
Exosc4	14.4260075	0.2821082	0.37344132	0.75542848	0.44999193	0.99938111		Exosc4	9.4086486	9.5029981	11.7689243	20.6814001	29.966611	8.23238167	14.4339583	11.4131377
Rfwd2	11.6340789	0.27389654	0.36257862	0.75541282	0.45000132	0.99938111		Rfwd2	6.04841696	12.2181404	11.7689243	10.3407001	9.98887032	9.40843619	17.32075	15.9783927
Dcun1d1	27.8787871	0.27126117	0.35915985	0.75526584	0.45008949	0.99938111		Dcun1d1	31.5861774	19.4585199	32.4440615	14.4769801	23.3073641	47.042181	43.301875	11.4131377
Mcm5	2.53552234	0.29594128	0.39187528	0.75519251	0.45013348	NA		Mcm5	4.03227797	0	2.54463228	0	8.3240586	1.17605452	1.92452778	2.28262753
Ppil4	21.8149179	0.28752118	0.38082632	0.75499293	0.45025322	0.99938111		Ppil4	22.8495752	21.2686148	24.1740066	6.20442003	9.98887032	38.8097993	37.5282917	13.6957652
Lamtor5	15.7510456	-0.2642994	0.35007435	-0.7549808	0.45026052	0.99938111		Lamtor5	16.8011582	23.9837571	20.0389792	8.27256004	8.3240586	15.2887088	17.32075	15.9783927
Ttc34	2.61760809	0.29598405	0.39216067	0.75475201	0.45039779	NA		Ttc34	0.67204633	2.26261859	0.63615807	4.13628002	9.98887032	0	0.96226389	2.28262753
Cxx1c	181.868044	-0.2684335	0.35567335	-0.7547193	0.45041743	0.99938111		Cxx1c	161.291119	139.829829	139.318617	376.401482	179.799666	79.9717076	113.547139	264.784794
Gxylt2	0.41891255	-0.1794254	0.23784607	-0.7543761	0.45062342	NA		Gxylt2	0	2.71514231	0.63615807	0	0	0	0	0
Mpzl2	0.41891255	-0.1794254	0.23784607	-0.7543761	0.45062342	NA		Mpzl2	0	2.71514231	0.63615807	0	0	0	0	0
Smim4	2.58027955	0.3011274	0.39929381	0.75414993	0.4507592	NA		Smim4	0	2.26261859	2.54463228	2.06814001	4.99443516	0	1.92452778	6.84788259
Vax1	1.03477401	-0.2478961	0.32874578	-0.7540663	0.45080939	NA		Vax1	0	0.90504744	0.9542371	4.13628002	0	0	0	2.28262753
Orai1	6.08389313	-0.313956	0.41636802	-0.7540348	0.4508283	0.99938111		Orai1	5.37637063	11.7656167	4.77118552	6.20442003	3.32962344	3.52816357	0	13.6957652
2310069G16Rik	5.26746594	-0.3108487	0.41229212	-0.7539526	0.4508777	0.99938111		2310069G16R	4.03227797	4.07271347	5.08926455	12.4088401	1.66481172	1.17605452	0	13.6957652
Phip	40.5027332	0.26944729	0.35740479	0.7538995	0.45090957	0.99938111		Phip	40.994826	48.8725616	29.8994292	22.7495401	31.6314227	83.4998712	48.1131944	18.2610202
Scn9a	4.36536519	0.31386809	0.41636126	0.75383595	0.45094774	NA		Scn9a	1.34409266	3.62018975	5.08926455	4.13628002	1.66481172	12.9365998	3.84905555	2.28262753
Lcorl	16.2490614	0.30269143	0.40159714	0.7537191	0.45101791	0.99938111		Lcorl	13.4409266	16.7433776	18.130505	6.20442003	8.3240586	14.1126543	46.1886666	6.84788259
Hnrnpll	33.9500773	0.21582763	0.28641355	0.75355244	0.45111801	0.99938111		Hnrnpll	33.6023164	33.4867552	30.5355873	26.8858201	31.6314227	30.5774176	52.9245139	31.9567854
Cdk14	196.693937	-0.2464376	0.32703707	-0.7535463	0.45112171	0.99938111		Cdk14	233.872122	271.514231	250.646279	113.747701	126.525691	205.809542	273.282944	98.1529838
Wasl	189.885035	0.16246844	0.21576596	0.75298456	0.45145919	0.99938111		Wasl	178.764323	201.825579	195.618606	134.429101	168.145984	215.217978	224.207486	200.871223
9130019P16Rik	9.44309962	0.29502805	0.39181289	0.752982	0.45146073	0.99938111		9130019P16Ri	8.06455594	4.52523719	8.58813393	10.3407001	6.65924688	5.88027262	8.660375	22.8262753
Mgat3	168.60285	0.21811456	0.28980698	0.75262011	0.45167823	0.99938111		Mgat3	122.984478	164.26611	123.732744	204.745861	224.749582	182.288451	100.075444	225.980126
Slc30a6	5.21697765	-0.3078748	0.40925887	-0.752274	0.45188633	0.99938111		Slc30a6	4.7043243	8.14542694	7.31581779	4.13628002	4.99443516	8.23238167	1.92452778	2.28262753
Stx17	17.1487532	0.24505357	0.32577042	0.75222782	0.45191407	0.99938111		Stx17	14.1129729	16.2908539	18.130505	12.4088401	11.653682	19.9929269	24.0565972	20.5436478
Rdx	45.4409426	0.21303168	0.28320637	0.7522136	0.45192261	0.99938111		Rdx	38.3066407	48.4200379	44.2129858	35.1583802	46.6147282	72.9153805	41.3773472	36.5220405
Iba57	6.89452602	0.30714179	0.4083357	0.75217961	0.45194305	0.99938111		Iba57	5.37637063	7.2403795	3.18079035	6.20442003	11.653682	5.88027262	1.92452778	13.6957652
Gfra4	26.7319299	-0.2585813	0.34378874	-0.7521518	0.45195977	0.99938111		Gfra4	36.9625481	28.5089943	24.8101647	28.9539601	26.6369875	8.23238167	34.6415	25.1089028
Fuk	10.1260537	0.31253813	0.41577453	0.75170101	0.45223089	0.99938111		Fuk	6.04841696	4.97776091	4.13502745	16.5451201	18.3129289	5.88027262	0	25.1089028
Nptx2	23.0514904	-0.2757636	0.36688784	-0.7516292	0.45227406	0.99938111		Nptx2	18.1452509	39.3695635	26.0824808	18.6132601	23.3073641	27.0492541	6.73584722	25.1089028
Dennd6a	29.0754909	0.22868787	0.30435911	0.75137514	0.45242692	0.99938111		Dennd6a	23.5216215	27.6039469	30.5355873	22.7495401	16.6481172	32.9295267	37.5282917	41.0872956
Aldh3a2	32.4385176	-0.2208476	0.29396322	-0.7512763	0.45248637	0.99938111		Aldh3a2	40.994826	38.4645161	38.8056422	22.7495401	26.6369875	37.6337448	33.6792361	20.5436478
Gigyf1	31.1663262	0.30317709	0.40366024	0.75107	0.45261053	0.99938111		Gigyf1	27.5538995	10.4080455	19.0847421	47.5672202	71.586904	18.8168724	8.660375	45.6525506
Fam219a	174.099917	0.28522945	0.37981345	0.75097249	0.45266921	0.99938111		Fam219a	116.264015	81.4542694	95.1056313	308.152862	224.749582	99.9646345	108.735819	358.372522
Pnma1	3.56729937	-0.3125928	0.41636148	-0.7507726	0.4527895	NA		Pnma1	4.03227797	2.71514231	3.49886938	8.27256004	4.99443516	1.17605452	3.84905555	0
2810428I15Rik	16.1619264	0.28754214	0.38307985	0.75060626	0.45288965	0.99938111		2810428I15Ri	11.4247876	9.05047438	7.95197586	28.9539601	21.6425524	9.40843619	13.4716944	27.3915304
Nadk2	26.0593943	-0.2244436	0.2990472	-0.7505291	0.4529361	0.99938111		Nadk2	30.9141311	31.6766603	27.672876	24.8176801	21.6425524	28.2253086	29.8301805	13.6957652
Pus3	6.47912563	0.31201577	0.41572981	0.75052536	0.45293835	0.99938111		Pus3	5.37637063	4.52523719	6.99773876	4.13628002	0	4.7042181	19.2452778	6.84788259
Cdca7	0.871271	0.24915649	0.33199514	0.75048234	0.45296426	NA		Cdca7	0.67204633	0.45252372	0.31807903	0	0	0	0.96226389	4.56525506
Nudcd2	6.85710678	0.30215197	0.4026916	0.75033095	0.45305541	0.99938111		Nudcd2	8.73660227	4.07271347	4.77118552	6.20442003	1.66481172	12.9365998	9.62263889	6.84788259
Cfb	2.15928702	-0.3086644	0.41139698	-0.7502837	0.45308388	NA		Cfb	2.01613899	3.16766603	2.22655324	4.13628002	1.66481172	1.17605452	2.88679167	0
Mir682	25.1783086	-0.2865616	0.38199142	-0.750178	0.45314752	0.99938111		Mir682	49.059382	27.1514231	17.4943469	22.7495401	11.653682	17.6408179	44.2641389	11.4131377
Selo	11.4738811	0.27691599	0.36913697	0.7501714	0.45315148	0.99938111		Selo	12.0968339	8.59795066	5.72542262	14.4769801	13.3184938	12.9365998	8.660375	15.9783927
Idua	6.81299425	-0.3091242	0.41209541	-0.7501278	0.45317775	0.99938111		Idua	4.7043243	12.2181404	7.95197586	6.20442003	0	5.88027262	3.84905555	13.6957652
Pcsk7	10.734026	0.26892884	0.35853187	0.7500835	0.45320441	0.99938111		Pcsk7	7.39250961	9.05047438	8.90621297	12.4088401	13.3184938	8.23238167	10.5849028	15.9783927
Prom2	0.39486816	0.19826304	0.26433279	0.75005087	0.45322407	NA		Prom2	0	0	0.31807903	0	1.66481172	1.17605452	0	0
Slc18a1	0.39486816	0.19826304	0.26433279	0.75005087	0.45322407	NA		Slc18a1	0	0	0.31807903	0	1.66481172	1.17605452	0	0
Kremen2	2.10854005	-0.286274	0.38179405	-0.7498125	0.45336762	NA		Kremen2	1.34409266	1.81009488	0.31807903	8.27256004	1.66481172	1.17605452	0	2.28262753
Klhl22	53.8416466	0.21901357	0.29210139	0.74978612	0.45338353	0.99938111		Klhl22	44.3550577	42.9897533	41.6683535	68.2486203	78.2461509	41.1619083	43.301875	70.7614535
Susd5	2.0263419	-0.2933371	0.39131241	-0.7496238	0.45348134	NA		Susd5	2.01613899	4.52523719	3.49886938	0	4.99443516	1.17605452	0	0
A930004D18Rik	2.95662554	-0.3025238	0.40360401	-0.7495559	0.45352219	NA		A930004D18R	0.67204633	1.81009488	3.49886938	10.3407001	1.66481172	4.7042181	0.96226389	0
A830018L16Rik	61.3545097	0.26294413	0.35080441	0.74954627	0.45352802	0.99938111		A830018L16Ri	70.5648645	58.8280835	56.2999891	31.0221002	53.2739751	50.5703445	133.754681	36.5220405
B3gnt5	1.95725717	0.3056878	0.40784368	0.74952198	0.45354265	NA		B3gnt5	0.67204633	2.71514231	1.90847421	0	1.66481172	3.52816357	2.88679167	2.28262753
Lypd6	29.7058334	0.26050741	0.34766038	0.7493158	0.45366689	0.99938111		Lypd6	29.5700384	25.3413283	34.6706148	14.4769801	13.3184938	51.7463991	36.5660278	31.9567854
Ust	19.1424441	-0.260274	0.34737073	-0.7492687	0.45369524	0.99938111		Ust	14.7850192	33.0342315	16.5401098	20.6814001	14.9833055	22.345036	12.5094306	18.2610202
Arhgdig	30.0532088	-0.2871926	0.38330456	-0.7492544	0.45370388	0.99938111		Arhgdig	45.027104	24.4362808	25.1282437	41.3628002	38.2906696	7.05632714	13.4716944	45.6525506
Zc4h2	14.1239251	0.27024518	0.36072334	0.74917577	0.45375127	0.99938111		Zc4h2	17.4732045	13.1231878	12.7231614	6.20442003	13.3184938	21.1689814	22.1320694	6.84788259
Myo1f	0.88216189	-0.2398244	0.32012136	-0.7491672	0.45375646	NA		Myo1f	2.68818531	0	0.63615807	2.06814001	1.66481172	0	0	0
Cacna1a	96.518083	0.24953553	0.33322384	0.74885259	0.45394606	0.99938111		Cacna1a	67.8766792	125.34907	90.9706039	57.9079203	153.162678	132.894161	48.1131944	95.8703563
Nrip1	28.0389373	0.27840704	0.37183091	0.74874636	0.4540101	0.99938111		Nrip1	30.9141311	28.961518	23.2197695	14.4769801	39.9554813	44.6900719	37.5282917	4.56525506
Lypd6b	9.21257884	0.29469881	0.39359549	0.74873522	0.45401681	0.99938111		Lypd6b	7.39250961	7.2403795	9.54237104	8.27256004	3.32962344	14.1126543	19.2452778	4.56525506
Fbxl17	109.843485	0.19749575	0.26381653	0.74861019	0.45409219	0.99938111		Fbxl17	81.3176057	76.0239848	97.6502636	155.110501	114.872009	103.492798	127.018833	123.261887
Ubald1	123.548161	-0.2377622	0.31761637	-0.7485829	0.45410867	0.99938111		Ubald1	133.065173	124.896546	114.190373	169.587481	129.855314	55.2745626	78.9056389	182.610202
Slc6a6	71.5482097	0.24839071	0.33182771	0.74855324	0.45412652	0.99938111		Slc6a6	43.6830113	93.2198861	61.0711746	55.8397803	114.872009	88.2040893	35.6037639	79.8919636
Pak7	30.3281893	-0.2515074	0.33602737	-0.748473	0.45417491	0.99938111		Pak7	49.059382	33.0342315	37.5333261	14.4769801	24.9721758	32.9295267	34.6415	15.9783927
Uros	9.59332949	-0.2873939	0.38400703	-0.7484079	0.45421417	0.99938111		Uros	14.7850192	9.5029981	14.6316356	4.13628002	4.99443516	7.05632714	12.5094306	9.13051012
Lpar4	1.94803364	-0.3021871	0.40379794	-0.7483621	0.45424179	NA		Lpar4	2.01613899	4.97776091	2.86271131	0	1.66481172	1.17605452	2.88679167	0
Pex26	19.336638	0.28085809	0.37548245	0.7479926	0.45446462	0.99938111		Pex26	12.0968339	11.313093	10.4966081	35.1583802	23.3073641	18.8168724	11.5471667	31.9567854
Kcnk2	38.8925593	-0.2435395	0.32564311	-0.7478724	0.45453712	0.99938111		Kcnk2	46.3711967	46.6099431	49.6203294	24.8176801	43.2851047	23.5210905	22.1320694	54.7830607
Pgd	19.4190697	0.26580884	0.3554549	0.74779906	0.45458137	0.99938111		Pgd	16.8011582	15.8383302	12.7231614	22.7495401	33.2962344	11.7605452	12.5094306	29.6741579
Tuba1a	458.579538	-0.1785832	0.23883342	-0.7477311	0.45462239	0.99938111		Tuba1a	478.496986	454.333814	510.834929	512.898723	547.723056	284.605195	375.282917	504.460684
Mrpl20	23.1071441	-0.2593693	0.34688631	-0.7477069	0.45463694	0.99938111		Mrpl20	20.8334362	22.1736622	16.5401098	45.4990802	18.3129289	12.9365998	21.1698055	27.3915304
Srsf4	48.1081563	0.30127368	0.40298092	0.74761277	0.45469376	0.99938111		Srsf4	27.5538995	21.2686148	23.8559276	88.9300204	48.2795399	21.1689814	25.981125	127.827142
Osr1	0.87737647	0.23782192	0.31812368	0.7475769	0.4547154	NA		Osr1	0	0.45252372	0.9542371	0	3.32962344	0	0	2.28262753
Sgsh	4.09227811	-0.3090355	0.41340237	-0.7475416	0.45473668	NA		Sgsh	4.03227797	5.43028463	5.08926455	4.13628002	4.99443516	3.52816357	0.96226389	4.56525506
Syde2	2.19322029	0.30457551	0.40744923	0.74751769	0.45475113	NA		Syde2	3.36023164	0.90504744	0.63615807	2.06814001	1.66481172	4.7042181	1.92452778	2.28262753
Hibch	4.74958563	0.31093892	0.41598522	0.74747587	0.45477636	0.99938111		Hibch	6.04841696	1.81009488	4.45310648	2.06814001	3.32962344	1.17605452	7.69811111	11.4131377
Aldh3b2	4.62909763	-0.3111481	0.41634727	-0.7473282	0.45486546	NA		Aldh3b2	6.72046328	2.26261859	5.72542262	8.27256004	9.98887032	1.17605452	2.88679167	0
Ypel3	157.525263	-0.2810681	0.37611487	-0.7472934	0.4548865	0.99938111		Ypel3	161.963165	119.013738	107.828793	326.766122	143.173808	51.7463991	89.4905416	260.219539
Sstr4	6.57215436	0.31066492	0.41573236	0.74727145	0.45489972	0.99938111		Sstr4	0	5.88280835	7.95197586	6.20442003	18.3129289	3.52816357	3.84905555	6.84788259
AA465934	0.67586736	0.21457805	0.28715915	0.74724436	0.45491607	NA		AA465934	1.34409266	0	0	0	0	1.17605452	2.88679167	0
Csgalnact2	5.46914776	0.30622589	0.40997301	0.74694158	0.45509882	0.99938111		Csgalnact2	3.36023164	7.2403795	6.04350166	0	8.3240586	9.40843619	4.81131944	4.56525506
Lrch1	12.4201896	0.27631838	0.37000739	0.74679152	0.45518942	0.99938111		Lrch1	13.4409266	5.88280835	9.54237104	16.5451201	8.3240586	18.8168724	15.3962222	11.4131377
Ipo8	50.4514467	0.18163496	0.24322936	0.74676411	0.45520597	0.99938111		Ipo8	45.027104	52.9452751	47.7118552	41.3628002	59.9332219	59.9787807	50.9999861	45.6525506
Brox	21.1000916	0.23328929	0.31240477	0.74675328	0.4552125	0.99938111		Brox	19.4893435	17.648425	22.9016905	16.5451201	16.6481172	29.4013631	27.9056528	18.2610202
Mrpl51	27.7371956	-0.2350223	0.31477866	-0.7466272	0.45528862	0.99938111		Mrpl51	31.5861774	27.1514231	22.9016905	43.4309402	14.9833055	28.2253086	30.7924444	22.8262753
Creld1	51.7609669	-0.2526092	0.33835334	-0.7465841	0.45531462	0.99938111		Creld1	33.6023164	85.0744592	51.2107246	57.9079203	53.2739751	44.6900719	22.1320694	66.1961984

GeneID	Base mean	log2(FC)	StdErr	Wald-Stats	P-value	P-adj		GeneID	Normalised expression for Chow#1	Normalised expression for Chow#2	Normalised expression for Chow#3	Normalised expression for Chow#4	Normalised expression for HFD#1	Normalised expression for HFD#2	Normalised expression for HFD#3	Normalised expression for HFD#4
C920006O11Rik	1.37402441	0.2553378	0.34205287	0.74648637	0.45537366	NA		C920006O11Ri	0	2.26261859	0.63615807	0	0	3.52816357	0	4.56525506
Zfp61	4.4595763	0.30802716	0.41268752	0.7463932	0.45542992	NA		Zfp61	5.37637063	2.26261859	4.77118552	2.06814001	1.66481172	8.23238167	6.73584722	4.56525506
Fuca1	10.0329599	-0.28606	0.38327378	-0.7463595	0.4554503	0.99938111		Fuca1	8.06455594	16.2908539	7.63389683	14.4769801	4.99443516	12.9365998	6.73584722	9.13051012
Ebp	8.88367997	-0.3040087	0.4073743	-0.7462638	0.45550807	0.99938111		Ebp	4.03227797	12.2181404	6.67965972	18.6132601	4.99443516	4.7042181	3.84905555	15.9783927
Hp1bp3	155.27823	0.16253647	0.21793824	0.74579141	0.45579343	0.99938111		Hp1bp3	161.963165	113.583453	160.629912	148.906081	146.503431	185.816615	174.169764	150.653417
Synrg	81.4186214	0.1694997	0.22728829	0.7457476	0.4558199	0.99938111		Synrg	80.6455594	84.6219354	75.0666521	64.1123403	96.5590798	84.6759257	97.1886527	68.4788259
Btg3	3.38229649	-0.3103792	0.41624385	-0.7456667	0.45586876	NA		Btg3	5.37637063	3.62018975	4.13502745	4.13628002	1.66481172	2.35210905	5.77358333	0
Gm7334	3.38229649	-0.3103792	0.41624385	-0.7456667	0.45586876	NA		Gm7334	5.37637063	3.62018975	4.13502745	4.13628002	1.66481172	2.35210905	5.77358333	0
Matn4	1.75584743	-0.3022468	0.4054844	-0.7453968	0.45603191	NA		Matn4	3.36023164	2.26261859	1.59039517	2.06814001	1.66481172	1.17605452	1.92452778	0
Gpn1	15.6905886	0.26135389	0.35064922	0.74534284	0.45606449	0.99938111		Gpn1	14.1129729	14.0282353	14.6316356	12.4088401	9.98887032	10.5844907	26.9433889	22.8262753
Dnajc2	5.75294737	0.29785135	0.3998383	0.74492952	0.45631433	0.99938111		Dnajc2	5.37637063	4.97776091	6.36158069	2.06814001	9.98887032	8.23238167	6.73584722	2.28262753
C030034I22Rik	1.46942459	0.28874565	0.38762403	0.74491164	0.45632514	NA		C030034I22Ri	1.34409266	0.45252372	1.59039517	0	1.66481172	1.17605452	0.96226389	4.56525506
Gm16523	1.94513755	0.30111056	0.40424576	0.74487006	0.45635028	NA		Gm16523	2.68818531	1.81009488	1.27231614	0	1.66481172	2.35210905	5.77358333	0
Gtf2e1	9.62681751	-0.2818545	0.37844224	-0.7447755	0.45640746	0.99938111		Gtf2e1	8.73660227	11.7656167	10.4966081	14.4769801	6.65924688	5.88027262	14.4339583	4.56525506
Mar-07	24.1023692	0.26525449	0.35627097	0.74453018	0.45655579	0.99938111		Mar-07	27.5538995	19.4585199	27.354797	10.3407001	21.6425524	31.7534722	43.301875	11.4131377
Usb1	5.64719678	-0.301552	0.40503253	-0.744513	0.45656615	0.99938111		Usb1	4.7043243	5.88280835	4.77118552	12.4088401	3.32962344	4.7042181	4.81131944	4.56525506
Gng11	1.28581035	-0.2786902	0.37433386	-0.7444964	0.45657619	NA		Gng11	1.34409266	2.71514231	1.27231614	2.06814001	0	0	2.88679167	0
Tspyl3	32.5459463	0.25694169	0.34522765	0.74426741	0.45671471	0.99938111		Tspyl3	22.8495752	23.5312334	22.9016905	47.5672202	46.6147282	15.2887088	33.6792361	47.9351782
Aspa	3.21824172	-0.3094621	0.4159262	-0.7440313	0.45685754	NA		Aspa	4.7043243	5.43028463	3.18079035	2.06814001	1.66481172	3.52816357	2.88679167	2.28262753
Lrwd1	5.36023684	0.30674995	0.41229554	0.74400503	0.45687343	0.99938111		Lrwd1	4.7043243	2.71514231	5.08926455	4.13628002	11.653682	3.52816357	1.92452778	9.13051012
Ppp2r1a	322.701982	0.23194667	0.31177557	0.74395394	0.45690434	0.99938111		Ppp2r1a	294.356292	220.379051	242.376224	409.491722	436.180671	205.809542	243.452764	529.569587
Kcnt1	28.5000062	0.2613605	0.35132241	0.74393346	0.45691673	0.99938111		Kcnt1	14.1129729	34.8443264	33.0802196	16.5451201	36.6258578	43.5140174	17.32075	31.9567854
Wdr11	35.068114	0.20327078	0.27326433	0.74386138	0.45696034	0.99938111		Wdr11	36.9625481	24.8888045	34.3525357	33.0902402	44.9499164	36.4576902	35.6037639	34.239413
C1ql2	2.20443522	-0.2893801	0.38906853	-0.7437766	0.45701166	NA		C1ql2	2.01613899	0.90504744	1.27231614	8.27256004	0	0	2.88679167	2.28262753
Grap	0.98884418	0.26081296	0.35067371	0.74374827	0.45702878	NA		Grap	0.67204633	0.45252372	1.27231614	0	1.66481172	0	3.84905555	0
Rlim	93.8649351	0.2017303	0.27123758	0.74374023	0.45703364	0.99938111		Rlim	86.02193	92.7673624	93.5152361	74.4530404	69.9220923	139.950488	121.24525	73.044081
Memo1	8.96889892	0.28731638	0.38637789	0.74361497	0.45710945	0.99938111		Memo1	6.72046328	7.69290322	7.63389683	8.27256004	4.99443516	4.7042181	13.4716944	18.2610202
Scaper	32.4089708	0.28574967	0.38428023	0.74359713	0.45712024	0.99938111		Scaper	36.2905017	31.2241366	29.5813502	14.4769801	11.653682	62.3308898	57.7358333	15.9783927
A330032B11Rik	0.36814433	0.19532583	0.26268517	0.74357388	0.45713431	NA		A330032B11Ri	0	0	0.31807903	0	1.66481172	0	0.96226389	0
Dnajc25	0.36814433	0.19532583	0.26268517	0.74357388	0.45713431	NA		Dnajc25	0	0	0.31807903	0	1.66481172	0	0.96226389	0
Slc16a5	0.36814433	0.19532583	0.26268517	0.74357388	0.45713431	NA		Slc16a5	0	0	0.31807903	0	1.66481172	0	0.96226389	0
Sprr2a1	0.36814433	0.19532583	0.26268517	0.74357388	0.45713431	NA		Sprr2a1	0	0	0.31807903	0	1.66481172	0	0.96226389	0
Sprr2a2	0.36814433	0.19532583	0.26268517	0.74357388	0.45713431	NA		Sprr2a2	0	0	0.31807903	0	1.66481172	0	0.96226389	0
Zfp446	5.08092257	0.29996258	0.4035443	0.74332007	0.45728792	0.99938111		Zfp446	2.68818531	4.97776091	5.08926455	4.13628002	3.32962344	5.88027262	7.69811111	6.84788259
Cyp19a1	0.5772706	-0.2402284	0.32324148	-0.7431854	0.45736943	NA		Cyp19a1	1.34409266	1.35757116	0.9542371	0	0	0	0.96226389	0
Tgtp2	0.54134483	-0.1787466	0.24051801	-0.7431736	0.45737661	NA		Tgtp2	0	2.26261859	0	2.06814001	0	0	0	0
Fbxl18	27.1978049	0.29129797	0.39196905	0.74316574	0.45738134	0.99938111		Fbxl18	23.5216215	13.1231878	18.766663	37.2265202	53.2739751	14.1126543	9.62263889	47.9351782
Aamdc	9.76717683	-0.2843051	0.3825972	-0.7430924	0.45742572	0.99938111		Aamdc	14.1129729	7.69290322	8.2700549	16.5451201	9.98887032	10.5844907	8.660375	2.28262753
Rlf	23.3074455	0.30659208	0.41264008	0.74300121	0.45748095	0.99938111		Rlf	23.5216215	21.7211385	28.3090341	2.06814001	11.653682	49.39429	45.2264028	4.56525506
Frmd4a	35.1274546	0.23383529	0.3147572	0.7429069	0.45753805	0.99938111		Frmd4a	43.6830113	29.4140417	30.8536663	20.6814001	44.9499164	48.2182355	22.1320694	41.0872956
Pcf11	26.9354176	0.25519193	0.34350643	0.74290293	0.45754045	0.99938111		Pcf11	31.5861774	20.8160911	30.5355873	12.4088401	19.9777406	37.6337448	44.2641389	18.2610202
Tmem8b	100.978942	0.26015757	0.35021845	0.7428437	0.45757631	0.99938111		Tmem8b	59.1400769	95.4825047	85.5632603	115.815841	176.470042	69.3872169	46.1886666	159.783927
Cdk6	0.68937673	0.2326357	0.31331503	0.74249776	0.45778581	NA		Cdk6	0	0.90504744	0.31807903	0	3.32962344	0	0.96226389	0
Wbp4	39.5332441	0.22973372	0.30941669	0.74247358	0.45780046	0.99938111		Wbp4	41.6668724	27.1514231	42.6225906	33.0902402	28.3017992	54.0985081	59.6603611	29.6741579
Pacrg	10.3358528	-0.2774771	0.37372576	-0.7424618	0.45780759	0.99938111		Pacrg	12.7688802	9.95552182	15.5858727	8.27256004	6.65924688	5.88027262	14.4339583	9.13051012
C630043F03Rik	4.37793582	-0.3082811	0.41523375	-0.7424278	0.45782818	NA		C630043F03Ri	2.68818531	6.33533207	8.2700549	4.13628002	3.32962344	3.52816357	6.73584722	0
Ddx54	32.0546338	0.2701242	0.36407115	0.74195441	0.45811496	0.99938111		Ddx54	19.4893435	16.2908539	22.9016905	55.8397803	51.6091633	19.9929269	26.9433889	43.3699231
Mta3	41.4632699	0.21601295	0.29118181	0.74184903	0.45817881	0.99938111		Mta3	44.3550577	43.8948007	37.5333261	24.8176801	51.6091633	58.8027262	43.301875	27.3915304
Pfdn5	13.2314453	-0.2893398	0.39002987	-0.7418401	0.45818423	0.99938111		Pfdn5	18.1452509	8.59795066	11.4508452	24.8176801	6.65924688	5.88027262	21.1698055	9.13051012
Parl	5.96711605	-0.3062646	0.41290893	-0.7417243	0.45825436	0.99938111		Parl	4.03227797	3.62018975	6.99773876	16.5451201	4.99443516	0	11.5471667	0
Rps5	60.3498672	-0.2404947	0.32428947	-0.7416049	0.45832675	0.99938111		Rps5	57.7959842	45.7048956	52.1649617	113.747701	56.6035985	31.7534722	70.2452639	54.7830607
Nhp2	10.4627989	-0.2709259	0.36539751	-0.7414554	0.45841738	0.99938111		Nhp2	14.1129729	13.5757116	10.1785291	10.3407001	6.65924688	11.7605452	12.5094306	4.56525506
Tdrd7	17.6534324	0.24777857	0.33423573	0.74132879	0.45849411	0.99938111		Tdrd7	20.1613899	15.8383302	18.766663	6.20442003	19.9777406	24.697145	17.32075	18.2610202
Arfgap2	33.0834197	0.2903772	0.39170837	0.74130967	0.4585057	0.99938111		Arfgap2	28.8979921	19.4585199	13.3593194	51.7035003	54.9387868	14.1126543	18.2830139	63.9135709
Fam71e1	1.74172928	-0.2961717	0.39958162	-0.7412045	0.45856944	NA		Fam71e1	2.01613899	1.81009488	1.90847421	4.13628002	0	1.17605452	2.88679167	0
Pced1a	11.8612696	0.27835101	0.37562472	0.74103485	0.45867231	0.99938111		Pced1a	7.39250961	9.95552182	8.2700549	16.5451201	16.6481172	4.7042181	15.3962222	15.9783927
Yae1d1	18.0428494	0.27988169	0.37804688	0.74033595	0.45909617	0.99938111		Yae1d1	16.8011582	18.1009488	19.0847421	8.27256004	6.65924688	19.9929269	39.4528194	15.9783927
Adcy3	36.5189745	0.25547676	0.34519062	0.74010341	0.45923725	0.99938111		Adcy3	28.2259458	46.1574193	31.1717454	22.7495401	69.9220923	47.042181	24.0565972	22.8262753
Klhl13	71.2193108	-0.2115708	0.28593142	-0.7399355	0.45933911	0.99938111		Klhl13	86.6939764	74.2138899	91.924841	55.8397803	38.2906696	69.3872169	89.4905416	63.9135709
Serac1	14.3967511	0.25724412	0.34768522	0.73987649	0.45937494	0.99938111		Serac1	13.4409266	16.7433776	14.6316356	4.13628002	18.3129289	18.8168724	15.3962222	13.6957652
1110019D14Rik	0.92160082	0.25667042	0.34699455	0.73969582	0.45948459	NA		1110019D14Ri	0.67204633	1.35757116	0.31807903	0	0	1.17605452	3.84905555	0
Ankrd52	82.5451523	0.27892319	0.37718065	0.73949496	0.4596065	0.99938111		Ankrd52	43.6830113	60.6381783	51.8468826	130.292821	138.179373	51.7463991	35.6037639	148.37079
Specc1l	27.8873162	0.2293777	0.31019304	0.73946761	0.4596231	0.99938111		Specc1l	20.8334362	20.8160911	28.3090341	33.0902402	23.3073641	44.6900719	26.9433889	25.1089028
Plcxd1	18.8637177	0.29437043	0.39809145	0.73945429	0.45963119	0.99938111		Plcxd1	10.0806949	10.4080455	9.86045007	35.1583802	41.620293	9.40843619	11.5471667	22.8262753
Tex30	2.26126689	-0.3059795	0.41379531	-0.7394465	0.45963591	NA		Tex30	4.03227797	2.71514231	2.22655324	2.06814001	1.66481172	1.17605452	1.92452778	2.28262753
5830416P10Rik	0.2672898	0.15900932	0.21505063	0.73940408	0.45966166	NA		5830416P10Ri	0	0	0	0	0	1.17605452	0.96226389	0
9330175M20Rik	0.2672898	0.15900932	0.21505063	0.73940408	0.45966166	NA		9330175M20R	0	0	0	0	0	1.17605452	0.96226389	0
Gm12060	0.2672898	0.15900932	0.21505063	0.73940408	0.45966166	NA		Gm12060	0	0	0	0	0	1.17605452	0.96226389	0
Gm19619	0.2672898	0.15900932	0.21505063	0.73940408	0.45966166	NA		Gm19619	0	0	0	0	0	1.17605452	0.96226389	0
Snora16a	0.2672898	0.15900932	0.21505063	0.73940408	0.45966166	NA		Snora16a	0	0	0	0	0	1.17605452	0.96226389	0
Ssu2	0.2672898	0.15900932	0.21505063	0.73940408	0.45966166	NA		Ssu2	0	0	0	0	0	1.17605452	0.96226389	0
Tdo2	0.2672898	0.15900932	0.21505063	0.73940408	0.45966166	NA		Tdo2	0	0	0	0	0	1.17605452	0.96226389	0
Uba1y	0.2672898	0.15900932	0.21505063	0.73940408	0.45966166	NA		Uba1y	0	0	0	0	0	1.17605452	0.96226389	0
Tpd52l1	12.3728093	-0.2626701	0.35526345	-0.7393671	0.45968412	0.99938111		Tpd52l1	16.8011582	14.9332827	13.3593194	8.27256004	8.3240586	9.40843619	9.62263889	18.2610202
Abl2	71.3696976	0.20569557	0.27826691	0.73920243	0.45978409	0.99938111		Abl2	65.8605402	66.068463	57.8903843	74.4530404	96.5590798	103.492798	58.6980972	47.9351782
Chchd4	28.7752375	0.27552845	0.3727742	0.73912961	0.4598283	0.99938111		Chchd4	33.6023164	15.8383302	18.448584	33.0902402	18.3129289	12.9365998	50.0377222	47.9351782
Fancf	1.19448759	-0.2753857	0.37260508	-0.7390821	0.45985714	NA		Fancf	1.34409266	2.26261859	0.63615807	2.06814001	0	0	0.96226389	2.28262753
Folr2	0.41741442	0.17519381	0.23712316	0.73883044	0.46000996	NA		Folr2	0	0.45252372	0	0	0	0	2.88679167	0
Lefty1	0.41741442	0.17519381	0.23712316	0.73883044	0.46000996	NA		Lefty1	0	0.45252372	0	0	0	0	2.88679167	0
B930003M22Rik	0.85446542	0.23369203	0.31630586	0.73881662	0.46001836	NA		B930003M22R	0.67204633	0	0.63615807	0	0	0	0.96226389	4.56525506
Srp54b	101.285063	0.23513375	0.31834516	0.73861261	0.46014227	0.99938111		Srp54b	81.9896521	53.8503226	64.251965	169.587481	103.218327	104.668853	104.886764	127.827142
D930015E06Rik	6.58323419	0.29540846	0.39995905	0.73859676	0.46015189	0.99938111		D930015E06Ri	4.7043243	8.59795066	5.40734359	2.06814001	8.3240586	10.5844907	3.84905555	9.13051012
3000002C10Rik	1.40848664	0.28891408	0.39123569	0.73846556	0.46023159	NA		3000002C10Ri	2.01613899	0.90504744	0.9542371	0	3.32962344	1.17605452	2.88679167	0
Rnf19b	25.2002234	0.22022056	0.29821486	0.73846271	0.46023332	0.99938111		Rnf19b	30.2420848	21.7211385	22.9016905	16.5451201	26.6369875	27.0492541	33.6792361	22.8262753
6330403A02Rik	232.780976	0.2283575	0.30923569	0.73845777	0.46023632	0.99938111		6330403A02Ri	218.415057	290.520228	188.938946	142.701661	419.532554	157.591306	229.981069	214.566988
Slc22a3	1.61212353	-0.2958784	0.40071088	-0.7383837	0.46028132	NA		Slc22a3	2.68818531	2.26261859	3.18079035	0	1.66481172	1.17605452	1.92452778	0
Tmem87a	19.1802939	-0.2786453	0.37744002	-0.7382506	0.4603622	0.99938111		Tmem87a	12.7688802	39.3695635	19.7209001	14.4769801	8.3240586	27.0492541	13.4716944	18.2610202
Tigd3	3.2414035	0.30705739	0.41611181	0.7379204	0.46056282	NA		Tigd3	1.34409266	1.35757116	2.22655324	6.20442003	4.99443516	2.35210905	2.88679167	4.56525506
4930414N06Rik	0.65500591	0.23039495	0.31233908	0.73764368	0.46073101	NA		4930414N06R	0	0.90504744	0.31807903	0	1.66481172	2.35210905	0	0
Plcb4	31.0913359	0.26918817	0.36501862	0.7374642	0.46084011	0.99938111		Plcb4	29.5700384	25.3413283	28.6271131	26.8858201	19.9777406	69.3872169	37.5282917	11.4131377
Golim4	10.4221179	0.30159605	0.4090217	0.73735954	0.46090373	0.99938111		Golim4	3.36023164	13.1231878	8.58813393	10.3407001	1.66481172	21.1689814	18.2830139	6.84788259
Unk	14.4386558	-0.2790116	0.37841125	-0.7373236	0.46092557	0.99938111		Unk	14.1129729	11.7656167	12.7231614	26.8858201	16.6481172	7.05632714	5.77358333	20.5436478
2610002J02Rik	23.6655196	-0.3003055	0.4073083	-0.7372929	0.46094423	0.99938111		2610002J02Ri	14.7850192	14.0282353	16.8581888	66.1804803	28.3017992	4.7042181	12.5094306	31.9567854
Gm15787	0.70933499	-0.243477	0.33026173	-0.7372243	0.46098597	NA		Gm15787	2.01613899	1.35757116	0.63615807	0	1.66481172	0	0	0
Msantd2	1.83000829	0.27063026	0.36717665	0.73705739	0.46108745	NA		Msantd2	1.34409266	0.45252372	2.86271131	0	0	0	7.69811111	2.28262753
Gm996	114.020457	0.28153863	0.38207637	0.7368648	0.46120457	0.99938111		Gm996	88.7101153	77.8340797	67.1146763	159.246781	201.442218	50.5703445	48.1131944	219.132243
Mir8094	0.33937065	-0.1931737	0.26217085	-0.7368238	0.46122951	NA		Mir8094	0.67204633	0.45252372	1.59039517	0	0	0	0	0
Camk2g	129.941256	0.1849137	0.25096262	0.73681768	0.46123323	0.99938111		Camk2g	116.936061	86.884554	110.691504	171.655621	136.514561	125.837834	128.943361	162.066555
Proser1	31.0704729	-0.255068	0.3461864	-0.7367938	0.46124776	0.99938111		Proser1	30.2420848	35.2968501	20.9932163	51.7035003	24.9721758	15.2887088	22.1320694	47.9351782
Fam173b	2.37639045	0.29900275	0.40591269	0.73661839	0.46135445	NA		Fam173b	0	2.26261859	1.27231614	4.13628002	4.99443516	1.17605452	2.88679167	2.28262753
2700060E02Rik	12.6452463	-0.2873529	0.39016788	-0.7364852	0.46143546	0.99938111		2700060E02Ri	16.8011582	6.78785578	10.1785291	24.8176801	9.98887032	4.7042181	9.62263889	18.2610202
Gadd45g	5.65464515	0.30427685	0.4131546	0.73647214	0.46144342	0.99938111		Gadd45g	5.37637063	2.26261859	4.13502745	6.20442003	8.3240586	2.35210905	2.88679167	13.6957652
Galnt15	0.35002753	-0.1923942	0.26126095	-0.7364063	0.46148346	NA		Galnt15	0.67204633	1.81009488	0.31807903	0	0	0	0	0
Glp2r	0.35002753	-0.1923942	0.26126095	-0.7364063	0.46148346	NA		Glp2r	0.67204633	1.81009488	0.31807903	0	0	0	0	0
Prss57	0.35002753	-0.1923942	0.26126095	-0.7364063	0.46148346	NA		Prss57	0.67204633	1.81009488	0.31807903	0	0	0	0	0
Lrrc56	2.60517417	-0.3019994	0.41013271	-0.7363455	0.46152049	NA		Lrrc56	1.34409266	1.81009488	3.81694841	6.20442003	0	1.17605452	1.92452778	4.56525506
Rapgef6	35.9847861	0.23578055	0.32020789	0.73633587	0.46152632	0.99938111		Rapgef6	30.9141311	30.7716129	41.6683535	26.8858201	24.9721758	62.3308898	45.2264028	25.1089028
Jpx	3.46790913	-0.3065441	0.41636786	-0.7362339	0.46158839	NA		Jpx	5.37637063	4.07271347	2.54463228	4.13628002	1.66481172	1.17605452	1.92452778	6.84788259
Fbxo27	5.1818906	0.30562637	0.41540177	0.73573679	0.4618909	0.99938111		Fbxo27	4.7043243	3.62018975	1.59039517	6.20442003	13.3184938	0	2.88679167	9.13051012
Nkx2-1	18.9179152	-0.2970312	0.4037301	-0.7357173	0.46190274	0.99938111		Nkx2-1	3.36023164	3.16766603	3.49886938	86.8618804	9.98887032	10.5844907	1.92452778	31.9567854
Tmem14c	15.3892932	-0.2788966	0.37908842	-0.7357033	0.46191129	0.99938111		Tmem14c	12.0968339	12.2181404	12.7231614	35.1583802	14.9833055	5.88027262	16.3584861	13.6957652
Klhl4	5.71221869	-0.3002628	0.40823356	-0.7355171	0.4620246	0.99938111		Klhl4	8.73660227	7.69290322	5.40734359	6.20442003	3.32962344	4.7042181	9.62263889	0

GeneID	Base mean	log2(FC)	StdErr	Wald-Stats	P-value	P-adj
Pum1	101.414434	0.17068737	0.23209205	0.7354296	0.4620779	0.99938111
Cep85	7.24012884	0.29207069	0.39785564	0.73411223	0.46288034	0.99938111
Wnt2	1.72789966	0.27978701	0.38120524	0.73395373	0.46297694	NA
Atmin	98.7710454	0.18090171	0.24650124	0.73387749	0.46302341	0.99938111
Acox1	135.375234	-0.1708933	0.2328886	-0.7337987	0.46307146	0.99938111
Akna	1.39031267	-0.278536	0.37968664	-0.7335944	0.463196	NA
Akap17b	15.8816047	0.27899421	0.38032986	0.73355851	0.46321786	0.99938111
U2af1l4	16.0640591	-0.2865141	0.39066782	-0.7333957	0.46331709	0.99938111
Gm1995	0.74286762	0.23136294	0.31548395	0.73335883	0.4633396	NA
Fam126a	11.0923605	-0.269444	0.36746756	-0.7332457	0.46340857	0.99938111
Mrpl54	10.9441599	-0.2718358	0.37097748	-0.7327555	0.46370756	0.99938111
Snrk	77.6940362	0.18960113	0.25881972	0.73256058	0.46382648	0.99938111
Prkar1b	713.924469	0.23019207	0.31427083	0.732464	0.46388541	0.99938111
Inhba	0.92830148	-0.241795	0.33011812	-0.73245	0.46389398	NA
Ctnnb1	242.342563	0.16201359	0.2212227	0.73235519	0.4639518	0.99938111
Topors	18.5484383	0.24039438	0.32830696	0.73222446	0.46403158	0.99938111
Diras2	151.983798	0.17263039	0.23576681	0.73220822	0.46404149	0.99938111
Mcm4	5.81664252	-0.3011914	0.41148618	-0.7319598	0.46419308	0.99938111
Ube2r2	69.1018349	-0.2368817	0.32362857	-0.7319553	0.46419584	0.99938111
Hmgn2	15.1344151	-0.2968104	0.40551282	-0.7319384	0.46420615	0.99938111
Tab3	21.8838766	0.28007139	0.38265963	0.73190734	0.46422513	0.99938111
Gltscr2	12.9029099	0.29483705	0.40293447	0.73172455	0.46433671	0.99938111
Rhot1	56.0217893	-0.2233947	0.30542414	-0.7314244	0.46451997	0.99938111
Dlgap3	249.394595	0.28081814	0.38400595	0.73128589	0.46460455	0.99938111
Tfb2m	10.0912489	0.28664643	0.39198526	0.73126839	0.46461524	0.99938111
Bfsp2	0.41105689	-0.1738847	0.23778768	-0.7312603	0.4646202	NA
Mvk	8.72796298	-0.2849488	0.38968473	-0.7312289	0.46463933	0.99938111
Rbm3os	0.82417179	0.2274079	0.31103479	0.73113334	0.46469771	NA
Mrap2	8.20236481	0.27702013	0.37893657	0.73104616	0.46475096	0.99938111
Tm2d2	28.5326576	-0.2511433	0.34355199	-0.7310198	0.46476704	0.99938111
Agpat1	131.26942	-0.2201297	0.30116863	-0.7309186	0.46482889	0.99938111
Pinx1	1.84621079	-0.2896513	0.39634692	-0.7308024	0.46489988	NA
Gm14446	2.65367733	0.30288041	0.41444959	0.73080157	0.46490037	NA
4931431C16Rik	0.54999537	0.20138053	0.27564703	0.73057393	0.46503944	NA
Gnat1	0.54999537	0.20138053	0.27564703	0.73057393	0.46503944	NA
Nradd	0.54999537	0.20138053	0.27564703	0.73057393	0.46503944	NA
Zfp473	0.54999537	0.20138053	0.27564703	0.73057393	0.46503944	NA
Ofd1	4.26690399	0.30258505	0.41420761	0.73051541	0.4650752	NA
Dand5	7.37065287	-0.292095	0.39986605	-0.7304822	0.46509549	0.99938111
Tmem201	56.8687783	0.26120375	0.35757769	0.73048111	0.46509616	0.99938111
Erbb3	2.51892525	-0.3008873	0.41198056	-0.7303433	0.46518034	NA
Caskin1	149.908102	0.26128039	0.3577599	0.7303233	0.46519259	0.99938111
Slc39a8	0.7110198	-0.2357064	0.32275513	-0.7302949	0.46520993	NA
Rims3	54.5047884	0.28369243	0.38847022	0.73028101	0.46521843	0.99938111
Saal1	6.19322299	0.29900385	0.40979028	0.72965091	0.46560359	0.99938111
Mex3b	17.5438379	0.27191139	0.37275786	0.72945851	0.46572124	0.99938111
Hcar1	0.42339858	-0.1694007	0.23223469	-0.7294374	0.46573412	NA
Pfkfb2	19.9440094	0.24191306	0.3316648	0.72939023	0.46576299	0.99938111
Cox18	7.17819541	-0.2904927	0.398295	-0.7293406	0.46579334	0.99938111
Agtpbp1	190.676502	0.21272066	0.29173337	0.72916122	0.46590305	0.99938111
Cc2d1a	31.3843941	0.24878052	0.34119456	0.72914561	0.4659126	0.99938111
Fermt3	1.03809268	0.26608814	0.36497407	0.72906037	0.46596473	NA
Egr2	4.69648198	0.30288827	0.41554394	0.7288959	0.46606534	0.99938111
Rnf6	55.7225776	0.19017597	0.26093174	0.72883419	0.46610309	0.99938111
Mir541	0.57065688	0.15489226	0.2125721	0.72865753	0.46621118	NA
Fancd2	0.96469173	-0.2362328	0.32429463	-0.7284513	0.46633738	NA
Gm16039	34.8740863	0.19842441	0.27241324	0.72839488	0.46637189	0.99938111
Uevld	7.23599486	0.28309027	0.38872211	0.72825873	0.46645522	0.99938111
Cntnap2	110.497497	0.25846401	0.35494398	0.72818254	0.46650185	0.99938111
Ung	1.73018742	0.24888564	0.34179359	0.72817528	0.4665063	NA
Iqub	0.98351141	-0.2598415	0.35703069	-0.7277848	0.46674533	NA
Nme2	27.3220652	-0.2972708	0.40849934	-0.7277144	0.46678844	0.99938111
Morn4	60.9222403	-0.2604468	0.35792112	-0.7276654	0.46681847	0.99938111
Ccdc25	30.6368085	0.22649888	0.31130612	0.72757605	0.46687314	0.99938111
Hmg20b	7.15016677	-0.293936	0.40409895	-0.7273862	0.46698943	0.99938111
Lmo2	7.54949495	-0.2816192	0.38728114	-0.72717	0.46712182	0.99938111
Chm	39.9060264	0.27888283	0.38354232	0.72712401	0.46714999	0.99938111
Zfp933	13.2959796	0.27739414	0.3816049	0.72691451	0.46727833	0.99938111
Slc35f3	21.5638986	0.24774099	0.3408475	0.72683822	0.46732507	0.99938111
Dgkb	147.835696	-0.2653829	0.36520103	-0.7266763	0.46742428	0.99938111
Med11	2.1199443	-0.2954308	0.40660005	-0.7265883	0.46747822	NA
Kcng2	4.11553174	-0.3005475	0.41365135	-0.726572	0.46748817	NA
Nfkbiz	1.80465243	0.28830967	0.39687342	0.72645245	0.46756145	NA
Ebpl	5.32300658	-0.2994333	0.41222649	-0.7263806	0.46760549	0.99938111
Adra1b	7.58940357	-0.2923723	0.40258553	-0.7262364	0.46769387	0.99938111
Lpcat4	60.1483218	-0.2583917	0.35585469	-0.7261157	0.46776787	0.99938111
4930538K18Rik	1.4107445	-0.2787411	0.383903	-0.7260717	0.46779481	NA
Hif1an	32.2566798	0.24911072	0.34314191	0.72596996	0.46785718	0.99938111
Gcnt4	2.79302421	-0.2989024	0.41183682	-0.7257788	0.46797439	NA
Cdt1	2.1154071	0.28288425	0.38982223	0.72567501	0.46803802	NA
Amfr	71.5308508	-0.2217449	0.30565228	-0.7254811	0.46815696	0.99938111
H2afv	14.510087	-0.2480102	0.34189997	-0.7253881	0.46821395	0.99938111
Tctn1	7.45161555	0.29773369	0.41046436	0.7253582	0.46823231	0.99938111
1600002K03Rik	0.62080002	-0.2347026	0.32366614	-0.7251381	0.46836734	NA
Rac2	0.62080002	-0.2347026	0.32366614	-0.7251381	0.46836734	NA
Snx16	20.4373409	0.2550118	0.35169134	0.72510118	0.46838996	0.99938111
Txndc15	34.1838381	0.21756214	0.30007196	0.72503323	0.46843165	0.99938111
Thbs1	1.45447366	-0.2703134	0.37285806	-0.7249768	0.46846626	NA
Dnajc28	7.28057937	0.28952038	0.39944731	0.72480243	0.46857325	0.99938111
Luc7l2	56.1238171	0.21516937	0.29694363	0.72461353	0.46868915	0.99938111
Taco1	6.82875854	0.2875892	0.39691628	0.72455885	0.46872271	0.99938111
Srpk2	187.862947	0.24567192	0.33908746	0.72450901	0.4687533	0.99938111
Dnah7b	0.58475266	0.22473865	0.31022498	0.72443762	0.46879711	NA
Olfml2a	0.84577163	-0.2289562	0.31606333	-0.7243999	0.46882026	NA
Ntn1	7.82560452	-0.2827905	0.39043932	-0.7242878	0.46888905	0.99938111
Mmp15	17.2666764	-0.2908813	0.40161332	-0.7242819	0.46889268	0.99938111
Dapk1	85.9585364	0.15762996	0.21764435	0.72425476	0.46890934	0.99938111
Map3k14	2.50446906	0.29802061	0.41153428	0.72416958	0.46896163	NA
Mthfd1l	6.71133179	-0.2833803	0.39132856	-0.7241493	0.46897405	0.99938111
Ephb3	6.08116568	-0.2942735	0.40644676	-0.7240148	0.46905663	0.99938111
Rbm8a	23.8811017	0.21892173	0.30241497	0.72391167	0.46911996	0.99938111
Mllt1	51.7183937	-0.232442	0.32113357	-0.7238173	0.46917792	0.99938111
Fbln1	2.39523307	-0.2900414	0.40071988	-0.723801	0.46918794	NA
Far1	34.5711538	0.2725859	0.37662132	0.72376653	0.46920908	0.99938111
Creg1	15.657031	-0.2893561	0.39981557	-0.723724	0.46923521	0.99938111
4931406C07Rik	8.62613489	-0.2868588	0.39650345	-0.723471	0.46939054	0.99938111
Zcrb1	9.81316214	0.2926613	0.40458516	0.72336143	0.46945786	0.99938111
Esyt2	29.0215144	0.28986003	0.40073799	0.72331558	0.46948602	0.99938111
Gm7367	10.6854663	-0.297445	0.41135357	-0.7230883	0.46962563	0.99938111
Fam65a	92.1791865	-0.1966594	0.2719939	-0.7230289	0.46966215	0.99938111
Kank1	10.9984362	-0.2920464	0.40394663	-0.7229827	0.46969052	0.99938111
Idi1	13.2270884	-0.2833593	0.3920226	-0.7228138	0.46979432	0.99938111

GeneID	Normalised expression for Chow#1	Normalised expression for Chow#2	Normalised expression for Chow#3	Normalised expression for Chow#4	Normalised expression for HFD#1	Normalised expression for HFD#2	Normalised expression for HFD#3	Normalised expression for HFD#4
Pum1	90.7262543	102.722884	98.6045007	84.7937404	141.508996	102.316744	85.6414861	105.000866
Cep85	8.73660227	4.97776091	8.2700549	2.06814001	9.98887032	12.9365998	8.660375	2.28262753
Wnt2	2.01613899	2.26261859	0	0	1.66481172	2.35210905	0.96226389	4.56525506
Atmin	85.3498837	99.1026944	90.9706039	88.9300204	99.8887032	83.4998712	89.4905416	152.936045
Acox1	172.715906	126.254118	141.863249	134.429101	166.481172	119.957561	104.886764	116.414004
Akna	1.34409266	2.26261859	3.49886938	0	1.66481172	2.35210905	0	0
Akap17b	18.8172972	10.8605693	15.5858727	10.3407001	13.3184938	35.2816357	18.2830139	4.56525506
U2af1l4	28.8979921	16.7433776	9.86045007	16.5451201	18.3129289	8.23238167	4.81131944	25.1089028
Gm1995	0.67204633	0	0.63615807	0	0	2.35210905	0	2.28262753
Fam126a	10.7527413	14.9332827	16.8581888	6.20442003	6.65924688	9.40843619	12.5094306	11.4131377
Mrpl54	16.1291119	9.05047438	9.54237104	14.4769801	6.65924688	7.05632714	8.660375	15.9783927
Snrk	61.8282622	64.7108918	59.4807795	105.475141	96.5590798	78.7956531	70.2452639	84.4572186
Prkar1b	592.744862	507.731613	530.237751	951.344405	990.562974	435.140174	560.037583	1143.59639
Inhba	2.01613899	3.16766603	0.31807903	0	0	0	1.92452778	0
Ctnnb1	239.248493	214.948766	213.749111	244.040521	344.616026	252.851723	226.132014	203.15385
Topors	15.4570656	20.3635674	18.448584	12.4088401	14.9833055	27.0492541	25.981125	13.6957652
Diras2	167.339536	116.298596	158.403359	126.156541	178.134854	137.598379	197.264097	134.675024
Mcm4	9.4086486	5.43028463	10.4966081	2.06814001	1.66481172	9.40843619	5.77358333	2.28262753
Ube2r2	76.6132814	71.4987476	89.3802087	68.2486203	24.9721758	84.6759257	89.4905416	47.9351782
Hmgn2	21.5054825	23.0787097	20.0389792	6.20442003	1.66481172	10.5844907	28.8679167	9.13051012
Tab3	14.1129729	29.8665654	22.9016905	8.27256004	14.9833055	41.1619083	34.6415	9.13051012
Gltscr2	12.7688802	5.43028463	6.67965972	18.6132601	9.98887032	5.88027262	9.62263889	34.239413
Rhot1	67.8766792	62.4482732	66.4785182	49.6353602	51.6091633	56.4506172	73.1320555	20.5436478
Dlgap3	206.990269	168.338823	152.359858	330.902402	541.063809	96.436471	108.735819	390.329308
Tfb2m	8.06455594	7.2403795	13.3593194	4.13628002	3.32962344	10.5844907	13.4716944	20.5436478
Bfsp2	2.01613899	0	1.27231614	0	0	0	0	0
Mvk	8.73660227	7.69290322	10.8146872	12.4088401	14.9833055	3.52816357	4.81131944	6.84788259
Rbm3os	0	0	0.31807903	2.06814001	0	0	1.92452778	2.28262753
Mrap2	5.37637063	6.33533207	6.99773876	10.3407001	6.65924688	11.7605452	6.73584722	11.4131377
Tm2d2	26.8818531	45.2523719	27.0367179	24.8176801	18.3129289	25.8731995	14.4339583	45.6525506
Agpat1	127.016756	166.528729	105.602239	171.655621	158.157113	84.6759257	72.1697916	164.349182
Pinx1	4.03227797	3.16766603	2.54463228	0	0	1.17605452	3.84905555	0
Gm14446	2.68818531	1.81009488	1.59039517	2.06814001	1.66481172	5.88027262	0.96226389	4.56525506
4931431C16Ri	0	0.45252372	0	0	1.66481172	0	0	2.28262753
Gnat1	0	0.45252372	0	0	1.66481172	0	0	2.28262753
Nradd	0	0.45252372	0	0	1.66481172	0	0	2.28262753
Zfp473	0	0.45252372	0	0	1.66481172	0	0	2.28262753
Ofd1	6.04841696	2.26261859	3.81694841	2.06814001	3.32962344	4.7042181	9.62263889	2.28262753
Dand5	7.39250961	5.88280835	6.67965972	14.4769801	4.99443516	2.35210905	5.77358333	11.4131377
Tmem201	40.994826	47.5149905	35.3067728	76.5211804	81.5757743	38.8097993	26.9433889	107.283494
Erbb3	4.03227797	4.52523719	3.49886938	0	4.99443516	1.17605452	1.92452778	0
Caskin1	114.919922	94.5774573	86.5174974	233.699821	264.705064	105.844907	79.8679027	219.132243
Slc39a8	0.67204633	2.71514231	0.63615807	0	1.66481172	0	0	0
Rims3	32.9302701	23.0787097	27.354797	105.475141	74.9165274	45.8661264	25.981125	100.435611
Saal1	1.34409266	4.97776091	5.40734359	10.3407001	6.65924688	4.7042181	11.5471667	4.56525506
Mex3b	12.0968339	17.648425	10.4966081	20.6814001	23.3073641	5.88027262	18.2830139	31.9567854
Hcar1	0.67204633	2.71514231	0	0	0	0	0	0
Pfkfb2	16.1291119	19.0059962	22.2655324	14.4769801	18.3129289	32.9295267	25.0188611	11.4131377
Cox18	10.0806949	9.05047438	10.1785291	4.13628002	6.65924688	10.5844907	6.73584722	0
Agtpbp1	213.710732	177.389298	198.163238	107.543281	166.481172	269.316486	262.698042	130.109769
Cc2d1a	26.2098068	22.6261859	24.1740066	39.2946602	58.2684102	17.6408179	24.0565972	38.804668
Fermt3	1.34409266	0.45252372	0.63615807	0	1.66481172	0	1.92452778	2.28262753
Egr2	7.39250961	2.26261859	3.49886938	0	6.65924688	1.17605452	2.88679167	13.6957652
Rnf6	61.8282622	51.1351802	52.4830407	41.3628002	49.9443516	74.091435	69.283	45.6525506
Mir541	0	0	0	0	0	0	0	4.56525506
Fancd2	4.7043243	0.45252372	0.63615807	0	0	0	1.92452778	0
Gm16039	32.2582238	31.2241366	34.0344567	31.0221002	24.9721758	38.8097993	43.301875	43.3699231
Uevld	7.39250961	7.2403795	5.72542262	4.13628002	6.65924688	14.1126543	5.77358333	6.84788259
Cntnap2	94.086486	124.444023	115.46269	55.8397803	74.9165274	248.147505	104.886764	66.1961984
Ung	0	2.26261859	1.27231614	0	0	1.17605452	0	9.13051012
Iqub	2.01613899	0.90504744	0.9542371	2.06814001	0	0	1.92452778	0
Nme2	21.5054825	11.7656167	19.4028211	76.5211804	11.653682	7.05632714	25.0188611	45.6525506
Morn4	79.3014668	48.4200379	47.7118552	97.2025805	59.9332219	16.4647633	53.8867778	84.4572186
Ccdc25	31.5861774	23.5312334	29.2632712	28.9539601	24.9721758	42.3379629	46.1886666	18.2610202
Hmg20b	4.03227797	8.59795066	5.40734359	16.5451201	8.3240586	5.88027262	3.84905555	4.56525506
Lmo2	7.39250961	7.69290322	12.7231614	6.20442003	4.99443516	5.88027262	8.660375	6.84788259
Chm	37.6345944	41.6321821	48.9841713	8.27256004	19.9777406	77.6195986	57.7358333	27.3915304
Zfp933	16.8011582	13.5757116	12.4050823	2.06814001	6.65924688	19.9929269	21.1698055	13.6957652
Slc35f3	14.7850192	28.0564706	22.5836114	10.3407001	21.6425524	34.1055812	25.0188611	15.9783927
Dgkb	212.36664	192.322581	205.797135	53.7716403	84.9053977	196.401106	173.2075	63.9135709
Med11	2.68818531	3.62018975	3.81694841	0	1.66481172	0	2.88679167	2.28262753
Kcng2	4.03227797	4.07271347	3.81694841	8.27256004	4.99443516	3.52816357	1.92452778	2.28262753
Nfkbiz	0.67204633	0.90504744	1.59039517	2.06814001	4.99443516	0	1.92452778	2.28262753
Ebpl	2.68818531	5.43028463	6.99773876	10.3407001	3.32962344	1.17605452	5.77358333	6.84788259
Adra1b	8.73660227	9.05047438	4.77118552	12.4088401	9.98887032	4.7042181	1.92452778	9.13051012
Lpcat4	57.1239379	95.9350284	56.9361472	55.8397803	76.5813391	34.1055812	20.2075417	84.4572186
4930538K18Ri	3.36023164	1.81009488	1.90847421	0	0	0	1.92452778	2.28262753
Hif1an	21.5054825	22.6261859	21.3112953	51.7035003	41.620293	37.6337448	18.2830139	43.3699231
Gcnt4	2.68818531	7.69290322	3.18079035	0	3.32962344	3.52816357	1.92452778	0
Cdt1	4.03227797	0.45252372	0.9542371	0	4.99443516	0	1.92452778	4.56525506
Amfr	54.4357526	108.605693	94.1513942	53.7716403	56.6035985	94.0843619	55.8113055	54.7830607
H2afv	16.1291119	14.9332827	20.6751372	12.4088401	11.653682	10.5844907	18.2830139	11.4131377
Tctn1	4.03227797	4.97776091	6.67965972	8.27256004	11.653682	7.05632714	0.96226389	15.9783927
1600002K03Ri	1.34409266	1.81009488	0.63615807	0	0	1.17605452	0	0
Rac2	1.34409266	1.81009488	0.63615807	0	0	1.17605452	0	0
Snx16	17.4732045	24.8888045	16.2220308	14.4769801	9.98887032	31.7534722	32.7169722	15.9783927
Txndc15	19.4893435	32.5817078	31.8079035	41.3628002	28.3017992	34.1055812	35.6037639	50.2178057
Thbs1	2.68818531	2.71514231	0.63615807	2.06814001	0	3.52816357	0	0
Dnajc28	9.4086486	6.33533207	7.63389683	0	4.99443516	8.23238167	12.5094306	9.13051012
Luc7l2	48.3873356	53.8503226	53.4372778	51.7035003	53.2739751	68.2111624	90.4528055	29.6741579
Taco1	7.39250961	4.97776091	7.95197586	2.06814001	3.32962344	8.23238167	11.5471667	9.13051012
Srpk2	180.780462	177.841822	208.977926	103.407001	109.877574	312.830503	297.339542	111.848749
Dnah7b	0	0.45252372	0.63615807	0	1.66481172	0	1.92452778	0
Olfml2a	0	2.71514231	0.31807903	2.06814001	1.66481172	0	0	0
Ntn1	6.04841696	9.95552182	6.67965972	14.4769801	6.65924688	9.40843619	4.81131944	4.56525506
Mmp15	12.0968339	24.8888045	19.4028211	22.7495401	23.3073641	16.4647633	0.96226389	18.2610202
Dapk1	87.3660227	82.8118406	76.6570473	74.4530404	103.218327	79.9717076	82.7546944	100.435611
Map3k14	3.36023164	0.90504744	1.59039517	2.06814001	6.65924688	3.52816357	1.92452778	0
Mthfd1l	8.06455594	5.88280835	6.99773876	10.3407001	8.3240586	4.7042181	4.81131944	4.56525506
Ephb3	8.06455594	5.43028463	6.04350166	8.27256004	8.3240586	4.7042181	0.96226389	6.84788259
Rbm8a	20.1613899	16.7433776	21.6293743	31.0221002	21.6425524	25.8731995	28.8679167	25.1089028
Mllt1	49.7314283	45.7048956	46.7576181	86.8618804	78.2461509	35.2816357	34.6415	36.5220405
Fbln1	0.67204633	7.2403795	3.49886938	0	3.32962344	1.17605452	0.96226389	2.28262753
Far1	36.9625481	32.129184	38.4875632	12.4088401	21.6425524	63.5069443	57.7358333	13.6957652
Creg1	16.1291119	25.793852	18.766663	12.4088401	3.32962344	31.7534722	12.5094306	4.56525506
4931406C07Ri	16.1291119	12.6706641	8.58813393	2.06814001	4.99443516	9.40843619	10.5849028	4.56525506
Zcrb1	14.1129729	8.14542694	8.58813393	2.06814001	4.99443516	12.9365998	23.0943333	4.56525506
Esyt2	18.8172972	40.7271347	34.9886938	2.06814001	24.9721758	59.9787807	34.6415	15.9783927
Gm7367	11.4247876	6.33533207	6.36158069	26.8858201	16.6481172	3.52816357	2.88679167	11.4131377
Fam65a	104.839227	97.2925996	95.4237104	97.2025805	121.531256	55.2745626	65.4339444	100.435611
Kank1	10.0806949	12.6706641	8.90621297	20.6814001	21.6425524	8.23238167	5.77358333	0
Idi1	22.1775288	13.1231878	10.4966081	16.5451201	1.66481172	17.6408179	17.32075	6.84788259

GeneID	Base mean	log2(FC)	StdErr	Wald-Stats	P-value	P-adj	GeneID	Normalised expression for Chow#1	Normalised expression for Chow#2	Normalised expression for Chow#3	Normalised expression for Chow#4	Normalised expression for HFD#1	Normalised expression for HFD#2	Normalised expression for HFD#3	Normalised expression for HFD#4
Gstt2	0.56179837	0.20256368	0.28026079	0.72276855	0.46982209	NA	Gstt2	0	0.90504744	0	0	1.66481172	0	1.92452778	0
Gm8363	0.84820818	0.23962103	0.33157695	0.72267095	0.46988207	NA	Gm8363	1.34409266	0	0.31807903	0	1.66481172	1.17605452	0	2.28262753
Dirc2	55.5353441	-0.2154095	0.29807742	-0.7226628	0.46988705	0.99938111	Dirc2	43.010965	81.9067931	58.8446214	55.8397803	36.6258578	58.8027262	38.4905555	70.7614535
Ccdc59	10.3058108	0.29898434	0.41381587	0.72250574	0.4699836	0.99938111	Ccdc59	14.1129729	9.95552182	7.63389683	2.06814001	1.66481172	16.4647633	25.981125	4.56525506
Apbb3	13.7902303	0.26914287	0.37256525	0.72240465	0.47004573	0.99938111	Apbb3	10.7527413	6.78785578	10.8146872	20.6814001	16.6481172	14.1126543	7.69811111	22.8262753
Chrm4	11.8814635	-0.2646931	0.36647051	-0.7222767	0.47012439	0.99938111	Chrm4	14.7850192	14.0282353	17.8124259	6.20442003	8.3240586	16.4647633	10.5849028	6.84788259
Actn2	5.88970512	0.29240407	0.40489376	0.7221748	0.47018701	0.99938111	Actn2	5.37637063	3.62018975	5.08926455	6.20442003	13.3184938	3.52816357	7.69811111	2.28262753
Dapl1	0.33322194	-0.1912865	0.26487712	-0.7221706	0.47018962	NA	Dapl1	0.67204633	1.35757116	0.63615807	0	0	0	0	0
Popdc3	0.33322194	-0.1912865	0.26487712	-0.7221706	0.47018962	NA	Popdc3	0.67204633	1.35757116	0.63615807	0	0	0	0	0
Zc3hav1	1.25938683	-0.2740625	0.37962541	-0.7219289	0.47033822	NA	Zc3hav1	3.36023164	1.81009488	1.59039517	0	0	2.35210905	0.96226389	0
Cops6	54.494252	0.22091685	0.30601169	0.72192291	0.47034188	0.99938111	Cops6	42.3389187	38.9170398	45.1672229	74.4530404	59.9332219	32.9295267	76.0188472	66.1961984
Gpr34	2.0137577	-0.282127	0.39081254	-0.7218986	0.47035684	NA	Gpr34	2.01613899	5.88280835	2.54463228	0	0	4.7042181	0.96226389	0
Tsc1	54.5518476	0.22101296	0.30636346	0.7214077	0.47065871	0.99938111	Tsc1	42.3389187	45.2523719	47.0756971	66.1804803	74.9165274	84.6759257	39.4528194	36.5220405
Ptprn	348.011103	0.22989765	0.3186974	0.72136655	0.47068402	0.99938111	Ptprn	265.4583	409.533966	272.593733	308.152862	522.75088	408.09092	150.113167	447.394996
Ocel1	2.64993005	0.29509197	0.40911195	0.72129882	0.47072568	NA	Ocel1	2.68818531	3.16766603	1.59039517	0	6.65924688	0	4.81131944	2.28262753
Gatad1	63.9814102	-0.212088	0.29405506	-0.7212528	0.47075396	0.99938111	Gatad1	76.6132814	66.5209867	76.9751264	57.9079203	29.966611	55.2745626	84.6792222	63.9135709
Rpp14	20.5667589	-0.2578738	0.35758625	-0.7211514	0.47081639	0.99938111	Rpp14	30.2420848	23.0787097	27.990955	10.3407001	13.3184938	14.1126543	31.7547083	13.6957652
Piezo2	0.52054021	0.17546084	0.24337063	0.72096146	0.47093323	NA	Piezo2	0	0	0.63615807	0	0	3.52816357	0	0
Prrg4	0.52054021	0.17546084	0.24337063	0.72096146	0.47093323	NA	Prrg4	0	0	0.63615807	0	0	3.52816357	0	0
Crem	6.76932705	0.2915887	0.40451954	0.72082724	0.47101581	0.99938111	Crem	4.7043243	7.69290322	7.95197586	2.06814001	3.32962344	9.40843619	14.4339583	4.56525506
Hnrnpab	97.6800195	0.17757261	0.24635368	0.72080357	0.47103038	0.99938111	Hnrnpab	91.3983007	78.7391271	92.8790781	103.407001	71.586904	98.78858	132.792417	111.848749
Ddx39b	62.6446197	0.24274573	0.33687252	0.72058631	0.47116408	0.99938111	Ddx39b	47.043243	42.9897533	53.4372778	80.6574604	98.2238915	34.1055812	44.2641389	100.435611
Mdp1	18.9659599	0.22875655	0.31751881	0.7204504	0.47124773	0.99938111	Mdp1	18.1452509	15.8383302	17.8124259	16.5451201	21.6425524	12.9365998	25.981125	22.8262753
Supt5	89.5707698	0.18806485	0.26105979	0.72038994	0.47128494	0.99938111	Supt5	101.478996	71.4987476	65.8423601	93.0663005	108.212762	91.7322529	68.3207361	116.414004
Cnpy4	2.35257643	-0.2962525	0.41128085	-0.7203168	0.47132994	NA	Cnpy4	3.36023164	3.16766603	4.77118552	0	0	2.35210905	2.88679167	2.28262753
Fastkd1	10.4754153	0.28611268	0.3972429	0.72024616	0.47137345	0.99938111	Fastkd1	14.7850192	9.05047438	8.90621297	2.06814001	3.32962344	11.7605452	20.2075417	13.6957652
Slc35c1	18.6911885	-0.2622679	0.36415368	-0.7202121	0.47139439	0.99938111	Slc35c1	9.4086486	23.0787097	20.0389792	31.0221002	18.3129289	12.9365998	9.62263889	25.1089028
Fbxl6	11.3630942	0.26329315	0.36557929	0.72020804	0.47139691	0.99938111	Fbxl6	8.73660227	8.59795066	8.58813393	14.4769801	18.3129289	11.7605452	6.73584722	13.6957652
Dnajb12	20.1417155	-0.2419409	0.33598978	-0.7200842	0.47147315	0.99938111	Dnajb12	24.1936678	29.8665654	21.3112953	12.4088401	18.3129289	11.7605452	25.0188611	18.2610202
Tgds	2.33588346	-0.2956254	0.41059412	-0.7199941	0.4715286	NA	Tgds	2.68818531	2.26261859	1.59039517	6.20442003	1.66481172	2.35210905	1.92452778	0
Setmar	2.94234491	-0.2986628	0.41490526	-0.7198337	0.4716274	NA	Setmar	4.03227797	4.52523719	5.72542262	0	1.66481172	4.7042181	2.88679167	0
Usp20	67.3724456	0.26465021	0.36780268	0.71954398	0.47180581	0.99938111	Usp20	43.6830113	38.0119924	47.7118552	107.543281	93.2294563	41.1619083	37.5282917	130.109769
Pi4k2b	1.04927863	-0.2617507	0.36378846	-0.7195136	0.47182452	NA	Pi4k2b	1.34409266	1.35757116	1.27231614	2.06814001	0	2.35210905	0	0
Ptcd2	27.2728236	-0.2341793	0.32547489	-0.7195004	0.47183265	0.99938111	Ptcd2	29.5700384	19.9110436	26.7186389	47.5672202	18.3129289	27.0492541	30.7924444	18.2610202
Hey2	3.42669389	-0.2991496	0.41578463	-0.7194821	0.47184392	NA	Hey2	2.68818531	4.97776091	3.18079035	6.20442003	1.66481172	3.52816357	2.88679167	2.28262753
Irf3	5.51117719	-0.2990719	0.41572703	-0.7193949	0.47189766	0.99938111	Irf3	8.73660227	4.07271347	3.18079035	10.3407001	0	1.17605452	2.88679167	13.6957652
Ccser2	133.442172	0.23166542	0.32203329	0.71938345	0.47190469	0.99938111	Ccser2	120.968339	135.757116	148.22483	76.5211804	83.240586	204.633487	206.886736	91.3051012
2310003H01Rik	13.1818326	0.29871646	0.41525591	0.7193551	0.47192216	0.99938111	2310003H01Ri	8.06455594	4.07271347	6.99773876	24.8176801	38.2906696	4.7042181	4.81131944	13.6957652
Dzip1l	10.012828	-0.2596025	0.36092854	-0.7192628	0.471979	0.99938111	Dzip1l	12.0968339	11.7656167	9.224292	12.4088401	6.65924688	8.23238167	10.5849028	9.13051012
Atp4a	23.4280488	-0.2468941	0.34330743	-0.7191632	0.47204038	0.99938111	Atp4a	28.8979921	34.3918026	23.8559276	16.5451201	21.6425524	35.2816357	15.3962222	11.4131377
AA474331	1.18128681	0.2681975	0.372931	0.71916119	0.47204161	NA	AA474331	2.01613899	0.45252372	0.63615807	0	0	1.17605452	2.88679167	2.28262753
Esyt1	1.52029234	-0.2689748	0.37424127	-0.7187202	0.47231332	NA	Esyt1	0.67204633	3.16766603	3.81694841	0	3.32962344	1.17605452	0	0
Maml2	8.49957461	-0.2909375	0.40488603	-0.7185665	0.47240808	0.99938111	Maml2	8.73660227	13.5757116	10.1785291	8.27256004	4.99443516	5.88027262	16.3584861	0
Nexn	0.85991088	-0.2186451	0.30439165	-0.718302	0.47257112	NA	Nexn	2.68818531	0	1.90847421	0	0	0	0	2.28262753
2610027K06Rik	0.71996973	-0.2327628	0.32407496	-0.7182376	0.47261081	NA	2610027K06Ri	2.68818531	0.45252372	0.9542371	0	1.66481172	0	0	0
Trim35	175.180516	0.25840091	0.35982667	0.71812608	0.47267955	0.99938111	Trim35	110.215598	95.9350284	125.641219	287.471461	244.727323	88.2040893	143.377319	305.872089
Zfp804b	2.89310422	0.27831808	0.38756821	0.7181138	0.47268712	NA	Zfp804b	0	0.45252372	5.72542262	2.06814001	0	5.88027262	6.73584722	2.28262753
Lhfpl5	3.07362243	0.29814895	0.41519294	0.71809734	0.47269727	NA	Lhfpl5	1.34409266	1.81009488	2.22655324	4.13628002	1.66481172	2.35210905	1.92452778	9.13051012
Mpi	23.5045376	-0.2202775	0.30675721	-0.7180841	0.47270543	0.99938111	Mpi	26.8818531	23.5312334	22.9016905	28.9539601	23.3073641	12.9365998	22.1320694	27.3915304
Ccdc12	7.44758887	-0.2986992	0.41598472	-0.7180532	0.47272448	0.99938111	Ccdc12	9.4086486	3.16766603	3.49886938	20.6814001	9.98887032	1.17605452	4.81131944	6.84788259
Acadsb	35.2521096	0.20621677	0.28720621	0.71800945	0.47275146	0.99938111	Acadsb	29.5700384	33.4867552	31.8079035	37.2265202	33.2962344	37.6337448	53.8867778	25.1089028
Ccdc141	5.51334883	-0.290683	0.40485137	-0.7179993	0.47275773	0.99938111	Ccdc141	5.37637063	7.69290322	8.58813393	4.13628002	6.65924688	5.88027262	5.77358333	0
Zfp207	61.5336691	-0.2533901	0.35297363	-0.7178727	0.47283581	0.99938111	Zfp207	70.5648645	87.3370778	69.9773876	47.5672202	21.6425524	84.6759257	80.8301666	29.6741579
Cct2	85.5609574	0.18109681	0.25245091	0.71735456	0.47315535	0.99938111	Cct2	81.3176057	82.3593168	74.112415	82.7256004	58.2684102	103.492798	115.471667	86.7398462
Htr2c	77.7977021	0.26346032	0.36740268	0.71708874	0.47331935	0.99938111	Htr2c	45.027104	99.1026944	86.1994184	41.3628002	64.9276571	177.584233	44.2641389	63.9135709
Akap11	199.634163	0.26858883	0.37463368	0.71693723	0.47341283	0.99938111	Akap11	178.764323	239.385047	204.524819	72.3849004	149.833055	452.780992	212.660319	86.7398462
Rplp0	30.0642778	-0.2631005	0.36706655	-0.7167651	0.47351902	0.99938111	Rplp0	35.6184554	17.1959013	27.354797	55.8397803	31.6314227	9.40843619	26.9433889	36.5220405
Nod2	0.67740863	-0.234736	0.3275673	-0.7166039	0.47361852	NA	Nod2	0.67204633	1.81009488	1.27231614	0	1.66481172	0	0	0
Cacna1b	88.0231258	0.24260438	0.33859846	0.71649582	0.47368525	0.99938111	Cacna1b	67.8766792	112.225882	65.5242811	68.2486203	171.475607	105.844907	49.0754583	63.9135709
Fam198a	2.63568168	-0.2979917	0.41597339	-0.7163721	0.47376161	NA	Fam198a	4.03227797	3.62018975	3.49886938	2.06814001	1.66481172	2.35210905	3.84905555	0
Slc25a13	1.23714903	-0.2671019	0.37299341	-0.7161036	0.47392737	NA	Slc25a13	4.03227797	1.81009488	0.9542371	0	0	1.17605452	1.92452778	0
Fat3	91.6440004	0.25225919	0.35227508	0.71608582	0.47393836	0.99938111	Fat3	80.6455594	102.722884	99.5587378	41.3628002	106.54795	170.527906	95.264125	36.5220405
Smim8	7.95292032	-0.2968179	0.41452598	-0.7160416	0.47396565	0.99938111	Smim8	13.4409266	13.1231878	11.1327662	0	3.32962344	5.88027262	14.4339583	2.28262753
Scaf8	49.6794909	0.20708968	0.28933759	0.71573721	0.47415363	0.99938111	Scaf8	53.7637063	42.9897533	48.3480132	37.2265202	46.6147282	83.4998712	46.1886666	38.804668
Dtymk	7.037008	-0.2797685	0.39088235	-0.7157358	0.47415451	0.99938111	Dtymk	11.4247876	8.59795066	7.95197586	4.13628002	8.3240586	5.88027262	7.69811111	2.28262753
Slc16a10	0.63402275	0.20427271	0.28545723	0.71559831	0.47423942	NA	Slc16a10	0	0.90504744	0.31807903	0	0	0	3.84905555	0
Nup214	38.6334985	0.23216088	0.32449027	0.71546329	0.47432281	0.99938111	Nup214	25.5377605	39.3695635	27.672876	47.5672202	69.9220923	29.4013631	30.7924444	38.804668
Cldn12	30.4945951	-0.2491544	0.34827576	-0.7153939	0.47436566	0.99938111	Cldn12	24.8657142	52.4927514	42.3045116	14.4769801	18.3129289	31.7534722	34.6415	25.1089028
Hs6st3	10.9119624	0.27411022	0.38323401	0.71525547	0.4744512	0.99938111	Hs6st3	11.4247876	10.4080455	10.1785291	4.13628002	23.3073641	12.9365998	5.77358333	9.13051012
Fgf5	0.87716823	-0.2569057	0.35920337	-0.7152095	0.4744796	NA	Fgf5	1.34409266	2.26261859	1.27231614	0	0	1.17605452	0.96226389	0
Nol3	68.7505742	0.29498017	0.41244539	0.71519813	0.47448662	0.99938111	Nol3	29.5700384	21.7211385	29.2632712	146.837941	63.2628454	25.8731995	39.4528194	194.02334
Hmgcll1	13.7702497	0.26955062	0.37690544	0.71516775	0.47450539	0.99938111	Hmgcll1	15.4570656	13.1231878	13.3593194	6.20442003	16.6481172	19.9929269	23.0943333	2.28262753
9830147E19Rik	1.82661665	0.28323627	0.39609372	0.71507387	0.4745634	NA	9830147E19Ri	0.67204633	0.45252372	1.27231614	4.13628002	1.66481172	3.52816357	2.88679167	0
2900056M20Rik	79.9930774	0.21866314	0.30580851	0.71503288	0.47458873	0.99938111	2900056M20R	43.010965	71.4987476	67.1146763	111.679561	58.2684102	91.7322529	77.943375	118.696632
Tbc1d19	18.3081491	-0.234339	0.32773496	-0.7150261	0.47459293	0.99938111	Tbc1d19	22.1775288	21.7211385	19.4028211	18.6132601	11.653682	16.4647633	25.0188611	11.4131377
Stab1	5.10920239	0.28590475	0.40002954	0.71470909	0.47478882	0.99938111	Stab1	2.01613899	8.14542694	3.81694841	0	19.9777406	2.35210905	0	4.56525506
Lrrc18	0.69970288	-0.2369243	0.33164194	-0.714398	0.47498113	NA	Lrrc18	1.34409266	0.90504744	0.31807903	2.06814001	0	0	0.96226389	0
Gm3219	3.00995335	0.29703371	0.41579125	0.71438181	0.47499111	NA	Gm3219	4.03227797	0.90504744	2.86271131	2.06814001	1.66481172	3.52816357	6.73584722	2.28262753
Slc25a33	20.517957	-0.2601865	0.36421677	-0.7143726	0.4749968	0.99938111	Slc25a33	19.4893435	14.480759	18.766663	41.3628002	6.65924688	19.9929269	18.2830139	25.1089028
6330403K07Rik	243.25704	-0.2599757	0.36395339	-0.7143105	0.47503521	0.99938111	6330403K07Ri	160.619072	165.171157	159.675675	608.033163	219.755147	138.774434	215.547111	278.480559
Dcc	31.6432779	0.27791212	0.38910281	0.71423827	0.47507986	0.99938111	Dcc	22.8495752	39.8220873	34.6706148	10.3407001	11.653682	75.2674895	28.8679167	29.6741579
Csmd2	31.0377975	0.26112242	0.36562022	0.7141903	0.47510951	0.99938111	Csmd2	25.5377605	36.2018975	24.8101647	20.6814001	71.586904	21.1689814	16.3584861	31.9567854
Cwf19l1	6.63166422	-0.281263	0.39387564	-0.7140907	0.47517107	0.99938111	Cwf19l1	6.04841696	9.05047438	7.31581779	8.27256004	3.32962344	3.52816357	8.660375	6.84788259
Fam109a	6.09914206	-0.2885026	0.40405266	-0.7140224	0.47521334	0.99938111	Fam109a	6.04841696	2.71514231	2.22655324	20.6814001	14.9833055	1.17605452	0.96226389	0
Dbt	11.790189	-0.2851833	0.39940421	-0.7140219	0.47521365	0.99938111	Dbt	18.8172972	13.5757116	16.5401098	6.20442003	3.32962344	15.2887088	18.2830139	2.28262753
Optc	1.42314274	0.24631276	0.34498258	0.71398608	0.47523579	NA	Optc	0	2.71514231	0.63615807	0	3.32962344	4.7042181	0	0
Snhg18	0.90981108	-0.240247	0.33653792	-0.7138779	0.47530271	NA	Snhg18	3.36023164	1.35757116	0.63615807	0	0	0	1.92452778	0
Tmem117	2.7585881	-0.285217	0.39956623	-0.7138166	0.47534059	NA	Tmem117	4.03227797	5.88280835	4.13502745	0	0	7.05632714	0.96226389	0
Tapt1	27.3255772	-0.2778827	0.38932117	-0.7137622	0.47537424	0.99938111	Tapt1	31.5861774	56.5654649	29.8994292	6.20442003	16.6481172	36.4576902	29.8301805	11.4131377
Zfpm2	18.6586987	0.23326727	0.32681752	0.71375388	0.47537938	0.99938111	Zfpm2	19.4893435	16.7433776	12.7231614	20.6814001	16.6481172	22.345036	26.9433889	13.6957652
Rfc3	3.18868207	0.2938539	0.41176946	0.71363696	0.4754517	NA	Rfc3	2.01613899	4.07271347	1.27231614	2.06814001	0	1.17605452	5.77358333	9.13051012
Crabp1	7.87429938	-0.2970574	0.41633585	-0.7135043	0.47553378	0.99938111	Crabp1	6.04841696	4.07271347	5.40734359	22.7495401	9.98887032	2.35210905	0.96226389	11.4131377
Bnc2	0.5995084	-0.2295935	0.32178814	-0.7134929	0.47554083	NA	Bnc2	0.67204633	1.35757116	1.59039517	0	0	1.17605452	0	0
Mei4	0.5995084	-0.2295935	0.32178814	-0.7134929	0.47554083	NA	Mei4	0.67204633	1.35757116	1.59039517	0	0	1.17605452	0	0
Rtcb	48.2132119	0.2107788	0.29542888	0.71346714	0.47555674	0.99938111	Rtcb	40.3227797	32.5817078	37.8514051	68.2486203	59.9332219	54.0985081	35.6037639	57.0656883
Irgm2	1.05347853	-0.257264	0.3606976	-0.7132401	0.47569721	NA	Irgm2	2.68818531	2.26261859	0.63615807	0	1.66481172	1.17605452	0	0
Katna1	1.05347853	-0.257264	0.3606976	-0.7132401	0.47569721	NA	Katna1	2.68818531	2.26261859	0.63615807	0	1.66481172	1.17605452	0	0
Myl12a	7.32027699	-0.2839729	0.39816888	-0.7131972	0.47572376	0.99938111	Myl12a	12.7688802	4.52523719	7.31581779	10.3407001	3.32962344	7.05632714	8.660375	4.56525506
Bdp1	30.561467	0.29288726	0.41069067	0.71315781	0.47574811	0.99938111	Bdp1	30.9141311	33.0342315	34.3525357	2.06814001	8.3240586	72.9153805	42.3396111	20.5436478
Egln3	6.84915869	-0.2801423	0.3929408	-0.7129377	0.47588433	0.99938111	Egln3	10.0806949	4.97776091	10.1785291	6.20442003	4.99443516	7.05632714	6.73584722	4.56525506
Pbx1	184.778753	-0.1428656	0.20043514	-0.712777	0.47598378	0.99938111	Pbx1	176.076138	199.56296	185.440077	219.222841	198.112595	148.18287	189.565986	162.066555
Rprd1a	78.4779571	0.23801869	0.33394114	0.71275642	0.4759965	0.99938111	Rprd1a	75.2691888	76.9290322	88.1078926	41.3628002	53.2739751	103.492798	141.452792	47.9351782
Pgm5	2.23368328	0.26689377	0.37447567	0.71271326	0.47602321	NA	Pgm5	0.67204633	1.35757116	3.49886938	0	9.98887032	2.35210905	0	0
2900005J15Rik	2.43296751	-0.2521435	0.35380351	-0.7126654	0.47605281	NA	2900005J15Ri	0.67204633	0.45252372	0.31807903	12.4088401	3.32962344	0	0	2.28262753
Acbd4	16.1064333	-0.255543	0.35858058	-0.7126516	0.47606136	0.99938111	Acbd4	14.7850192	16.2908539	13.3593194	28.9539601	23.3073641	8.23238167	12.5094306	11.4131377
Camk1g	41.854754	0.23401904	0.32839232	0.71262032	0.47608073	0.99938111	Camk1g	37.6345944	17.1959013	35.3067728	64.1123403	44.9499164	47.042181	45.2264028	43.3699231
Eif4a3	11.580055	0.24403132	0.34246868	0.71256535	0.47611476	0.99938111	Eif4a3	10.0806949	10.8605693	9.86045007	10.3407001	13.3184938	12.9365998	11.5471667	13.6957652
Ctns	7.43160464	-0.2860254	0.40141387	-0.7125449	0.47612739	0.99938111	Ctns	4.03227797	10.8605693	10.1785291	10.3407001	8.3240586	7.05632714	8.660375	0
Fbxo25	43.6797741	0.20262802	0.28441277	0.71244348	0.4761902	0.99938111	Fbxo25	31.5861774	38.4645161	40.3960374	49.6353602	53.2739751	31.7534722	40.4150833	63.9135709
1110012L19Rik	2.99630835	-0.2935113	0.41199396	-0.7124165	0.47620688	NA	1110012L19Ri	3.36023164	3.62018975	4.45310648	4.13628002	1.66481172	0	6.73584722	0
Samd9l	2.13951553	-0.2811526	0.39471027	-0.7123011	0.47627834	NA	Samd9l	2.68818531	2.71514231	5.72542262	0	0	1.17605452	4.81131944	0
Prepl	325.488368	0.17756037	0.24934197	0.71211584	0.47639305	0.99938111	Prepl	322.582238	277.39704	369.289759	244.040521	264.705064	432.788065	421.471583	271.632676

GeneID	Base mean	log2(FC)	StdErr	Wald-Stats	P-value	P-adj		GeneID	Normalised expression for Chow#1	Normalised expression for Chow#2	Normalised expression for Chow#3	Normalised expression for Chow#4	Normalised expression for HFD#1	Normalised expression for HFD#2	Normalised expression for HFD#3	Normalised expression for HFD#4
Smchd1	27.740075	0.23404526	0.32867378	0.71208985	0.47640914	0.99938111		Smchd1	30.2420848	25.3413283	31.1717454	12.4088401	28.3017992	39.9858538	38.4905555	15.9783927
Kbtbd3	8.58509273	0.27652321	0.3884137	0.7119296	0.47650837	0.99938111		Kbtbd3	9.4086486	7.69290322	8.58813393	4.13628002	4.99443516	12.9365998	16.3584861	4.56525506
2510002D24Rik	6.67300915	0.28145081	0.39534053	0.71191995	0.47651435	0.99938111		2510002D24Ri	4.03227797	4.52523719	7.31581779	6.20442003	4.99443516	5.88027262	6.73584722	13.6957652
Elmod1	113.946261	-0.2828426	0.39742621	-0.7116859	0.47665928	0.99938111		Elmod1	143.145868	210.423529	143.453645	26.8858201	48.2795399	190.520833	107.773556	41.0872956
Ell3	1.07100895	0.23325601	0.3277558	0.71167623	0.47666529	NA		Ell3	0	2.26261859	0.31807903	0	0	1.17605452	4.81131944	0
Gpr108	7.44617853	-0.2961321	0.41616029	-0.7115818	0.47672379	0.99938111		Gpr108	4.7043243	13.1231878	9.86045007	6.20442003	4.99443516	4.7042181	0	15.9783927
Atxn7l2	6.14315535	0.28384066	0.39897362	0.71142715	0.47681958	0.99938111		Atxn7l2	6.04841696	3.62018975	4.45310648	8.27256004	8.3240586	9.40843619	6.73584722	2.28262753
Mau2	41.8579007	-0.2006954	0.28224398	-0.7110707	0.47704044	0.99938111		Mau2	31.5861774	45.2523719	47.7118552	57.9079203	48.2795399	31.7534722	40.4150833	31.9567854
Parp11	24.7925416	0.20865639	0.29344081	0.71106804	0.47704207	0.99938111		Parp11	20.8334362	21.2686148	19.4028211	31.0221002	26.6369875	23.5210905	25.981125	29.6741579
Exoc6b	92.6631851	0.1834517	0.25800859	0.71102943	0.477066	0.99938111		Exoc6b	72.5810035	91.8623149	75.0666521	107.543281	63.2628454	111.72518	109.698083	109.566121
Pik3ca	78.3483119	0.23125296	0.32533842	0.7108074	0.47720359	0.99938111		Pik3ca	71.2369108	77.8340797	90.0163668	43.4309402	58.2684102	125.837834	114.509403	45.6525506
Pabpc1	43.246247	-0.1915583	0.26949646	-0.7108007	0.47720778	0.99938111		Pabpc1	47.7152893	47.5149905	45.1672229	47.5672202	46.6147282	35.2816357	50.9999861	25.1089028
Gpr125	10.9094424	-0.2720749	0.3827907	-0.7107669	0.47722871	0.99938111		Gpr125	8.06455594	20.3635674	13.9954775	6.20442003	9.98887032	14.1126543	7.69811111	6.84788259
Gosr2	71.8039788	-0.2318002	0.32612823	-0.710764	0.47723049	0.99938111		Gosr2	71.2369108	70.1411764	58.5265423	115.815841	79.9109626	29.4013631	55.8113055	93.5877288
Rbbp6	100.030132	0.21415205	0.30133527	0.71067701	0.47728441	0.99938111		Rbbp6	74.5971425	65.6159392	76.9751264	150.974221	83.240586	112.901234	87.5660139	148.37079
Lcmt2	7.38748918	0.29571204	0.41618231	0.71053487	0.47737251	0.99938111		Lcmt2	2.68818531	4.52523719	4.45310648	12.4088401	14.9833055	1.17605452	2.88679167	15.9783927
Rce1	8.32296008	-0.2943239	0.41426509	-0.7104724	0.47741121	0.99938111		Rce1	10.0806949	7.2403795	5.72542262	16.5451201	4.99443516	10.5844907	0	11.4131377
Stradb	26.4065554	-0.2162269	0.30441314	-0.7103073	0.47751358	0.99938111		Stradb	31.5861774	31.6766603	27.990955	24.8176801	26.6369875	21.1689814	33.6792361	13.6957652
Cdc25c	0.7111412	0.22654729	0.31905967	0.71004677	0.47767513	NA		Cdc25c	0.67204633	0	0.9542371	0	0	1.17605452	2.88679167	0
Zdhhc8	82.6970511	-0.2365952	0.33335011	-0.70975	0.47785917	0.99938111		Zdhhc8	75.9412351	110.868311	68.7050715	107.543281	109.877574	56.4506172	31.7547083	100.435611
Hnrnpa3	74.7694535	0.26196076	0.36910739	0.70971422	0.47788137	0.99938111		Hnrnpa3	78.6294204	62.4482732	88.1078926	33.0902402	41.620293	128.189943	131.830153	34.239413
Bag5	25.6277355	0.26033067	0.36684135	0.70965467	0.47791831	0.99938111		Bag5	28.2259458	13.1231878	32.1259825	16.5451201	9.98887032	35.2816357	42.3396111	27.3915304
Mis12	16.2058514	-0.2733042	0.38516331	-0.7095802	0.47796452	0.99938111		Mis12	17.4732045	19.0059962	25.1282437	12.4088401	1.66481172	22.345036	20.2075417	11.4131377
Cdh24	4.12421768	-0.2944881	0.41508924	-0.7094573	0.47804076	NA		Cdh24	6.04841696	4.97776091	2.54463228	6.20442003	6.65924688	2.35210905	1.92452778	2.28262753
Ephb4	1.42975146	-0.2536646	0.35780633	-0.7089438	0.47835935	NA		Ephb4	1.34409266	3.62018975	1.90847421	0	0	0	0	4.56525506
Colgalt2	3.4295615	0.29473279	0.41578335	0.70886146	0.47841045	NA		Colgalt2	3.36023164	4.52523719	2.54463228	0	4.99443516	5.88027262	3.84905555	2.28262753
Prdx4	4.2058042	-0.292696	0.41302982	-0.708656	0.478538	NA		Prdx4	0.67204633	10.8605693	5.40734359	4.13628002	1.66481172	7.05632714	3.84905555	0
Taf11	26.3490856	0.22097744	0.31183118	0.70864445	0.47854514	0.99938111		Taf11	27.5538995	17.1959013	22.5836114	31.0221002	26.6369875	23.5210905	39.4528194	22.8262753
Ap2a2	205.704596	0.21704614	0.30629764	0.70861184	0.47856539	0.99938111		Ap2a2	184.140694	154.310588	157.449122	254.381221	369.588202	172.880015	133.754681	219.132243
Siah1b	4.26960016	-0.2925091	0.41290232	-0.7084221	0.47868315	NA		Siah1b	6.04841696	5.43028463	5.40734359	2.06814001	3.32962344	1.17605452	3.84905555	6.84788259
Pak1ip1	19.2058497	0.24029186	0.3392183	0.70836939	0.4787159	0.99938111		Pak1ip1	22.8495752	14.480759	19.4028211	12.4088401	18.3129289	18.8168724	33.6792361	13.6957652
Polr3e	18.3057448	0.25574989	0.3610594	0.70833189	0.47873917	0.99938111		Polr3e	22.1775288	13.5757116	14.3135566	12.4088401	26.6369875	10.5844907	12.5094306	34.239413
Oma1	3.34320576	0.29371101	0.41465622	0.70832413	0.47874399	NA		Oma1	3.36023164	2.71514231	1.59039517	2.06814001	4.99443516	0	2.88679167	9.13051012
Ldlrad4	13.8587589	-0.2582604	0.3646804	-0.7081827	0.4788318	0.99938111		Ldlrad4	20.8334362	20.8160911	14.6316356	4.13628002	14.9833055	10.5844907	13.4716944	11.4131377
Ddx3y	31.8886328	0.28614664	0.40421213	0.70791204	0.47899988	0.99938111		Ddx3y	38.3066407	35.2968501	29.2632712	4.13628002	19.9777406	56.4506172	62.5471528	9.13051012
G730013B05Rik	0.30704968	0.17937552	0.25340299	0.70786663	0.47902809	NA		G730013B05R	0	0	0.31807903	0	0	1.17605452	0.96226389	0
Gm16287	0.30704968	0.17937552	0.25340299	0.70786663	0.47902809	NA		Gm16287	0	0	0.31807903	0	0	1.17605452	0.96226389	0
Loxl4	0.30704968	0.17937552	0.25340299	0.70786663	0.47902809	NA		Loxl4	0	0	0.31807903	0	0	1.17605452	0.96226389	0
Nav2	44.8273969	0.21364751	0.30184049	0.70781595	0.47905956	0.99938111		Nav2	38.3066407	42.9897533	35.9429309	45.4990802	74.9165274	30.5774176	35.6037639	54.7830607
Crlf2	8.31028045	0.29453953	0.41630969	0.70750102	0.47925518	0.99938111		Crlf2	4.03227797	7.69290322	2.54463228	12.4088401	14.9833055	2.35210905	1.92452778	20.5436478
Pigg	10.8161765	0.28532994	0.40337653	0.70735385	0.47934661	0.99938111		Pigg	8.06455594	16.2908539	9.224292	2.06814001	8.3240586	27.0492541	8.660375	6.84788259
Rhoa	82.5836674	-0.1794584	0.25378762	-0.7071203	0.47949173	0.99938111		Rhoa	87.3660227	77.3815559	109.419188	78.5893204	63.2628454	64.6829988	100.075444	79.8919636
Tnpo1	20.7135884	-0.2445371	0.34582378	-0.7071147	0.47949522	0.99938111		Tnpo1	20.1613899	19.9110436	15.9039517	39.2946602	16.6481172	14.1126543	25.981125	13.6957652
Reep1	77.5458491	0.22697313	0.32101459	0.70704924	0.47953588	0.99938111		Reep1	57.7959842	93.6724098	81.4282328	45.4990802	76.5813391	136.422325	49.0754583	79.8919636
Cdyl2	25.7963245	0.20758313	0.29368057	0.70683303	0.47967024	0.99938111		Cdyl2	20.8334362	25.3413283	20.9932163	26.8858201	24.9721758	23.5210905	25.0188611	38.804668
Gpr1	0.46797378	-0.1910022	0.27026831	-0.7067133	0.47974469	NA		Gpr1	0	1.35757116	0.31807903	2.06814001	0	0	0	0
Rab39	0.46797378	-0.1910022	0.27026831	-0.7067133	0.47974469	NA		Rab39	0	1.35757116	0.31807903	2.06814001	0	0	0	0
Slc22a21	0.46797378	-0.1910022	0.27026831	-0.7067133	0.47974469	NA		Slc22a21	0	1.35757116	0.31807903	2.06814001	0	0	0	0
Sv2c	44.4315502	0.24880932	0.35207361	0.70669687	0.47975488	0.99938111		Sv2c	30.9141311	51.587704	45.1672229	28.9539601	34.9610461	90.5561984	23.0943333	50.2178057
Crtc1	216.234891	0.27127839	0.38393912	0.70656616	0.47983613	0.99938111		Crtc1	145.834053	171.506489	121.188112	310.221002	434.515859	116.429398	74.0943194	356.089895
Copa	146.835759	0.15190417	0.21505062	0.70636472	0.47996136	0.99938111		Copa	154.570656	119.013738	145.680198	134.429101	176.470042	174.05607	128.943361	141.522907
Ccp110	35.6948506	0.25100733	0.35535992	0.70634676	0.47997252	0.99938111		Ccp110	39.6507334	35.2968501	36.897168	14.4769801	24.9721758	61.1548353	54.8490416	18.2610202
Cyp20a1	2.71597001	-0.2925337	0.41420565	-0.7062524	0.48003119	NA		Cyp20a1	2.68818531	4.97776091	5.08926455	0	1.66481172	1.17605452	3.84905555	2.28262753
Pyroxd1	4.1452096	0.29387601	0.41623293	0.70603739	0.48016489	NA		Pyroxd1	4.03227797	3.62018975	4.13502745	2.06814001	1.66481172	7.05632714	10.5849028	0
Vegfa	19.0121092	0.25255488	0.35784776	0.70576067	0.48033699	0.99938111		Vegfa	13.4409266	23.5312334	23.5378486	4.13628002	26.6369875	22.345036	20.2075417	18.2610202
Slc17a5	11.8448067	-0.2640096	0.37416721	-0.7055926	0.48044155	0.99938111		Slc17a5	7.39250961	15.8383302	13.6773985	16.5451201	18.3129289	9.40843619	6.73584722	6.84788259
Lrrc7	97.7881708	0.23162039	0.32827176	0.70557513	0.48045239	0.99938111		Lrrc7	105.511274	96.3875521	111.96382	37.2265202	96.5590798	143.478652	131.830153	59.3483158
Ly96	0.65158783	-0.2091108	0.29638513	-0.7055375	0.48047579	NA		Ly96	0	1.81009488	2.22655324	0	0	1.17605452	0	0
F11r	1.47893516	-0.2649276	0.3755631	-0.7054142	0.48055249	NA		F11r	1.34409266	5.43028463	0.63615807	0	0	1.17605452	0.96226389	2.28262753
BC052040	8.15801231	-0.2809512	0.39856951	-0.7048989	0.48087318	0.99938111		BC052040	10.7527413	14.480759	9.86045007	2.06814001	3.32962344	10.5844907	9.62263889	4.56525506
A430005L14Rik	6.22208094	-0.2847841	0.4040352	-0.7048498	0.48090373	0.99938111		A430005L14Ri	9.4086486	9.05047438	5.08926455	6.20442003	6.65924688	4.7042181	8.660375	0
Zer1	202.315377	0.25335548	0.3594869	0.70476973	0.48095355	0.99938111		Zer1	149.866331	120.823833	141.54517	304.016582	352.940085	104.668853	125.094306	319.567854
Gns	58.3145774	-0.2567558	0.36431329	-0.7047665	0.48095558	0.99938111		Gns	53.7637063	104.532979	77.9293635	22.7495401	33.2962344	83.4998712	33.6792361	57.0656883
Heg1	14.43314	-0.274936	0.39024244	-0.7045262	0.48110516	0.99938111		Heg1	12.0968339	24.8888045	16.5401098	12.4088401	13.3184938	25.8731995	5.77358333	4.56525506
Vwa5b2	14.1061002	0.28063136	0.39859831	0.70404553	0.48140442	0.99938111		Vwa5b2	12.7688802	5.88280835	7.63389683	22.7495401	28.3017992	8.23238167	6.73584722	20.5436478
Dhdh	8.6650551	0.26899091	0.38220092	0.70379452	0.48156075	0.99938111		Dhdh	10.0806949	5.43028463	5.08926455	10.3407001	13.3184938	8.23238167	7.69811111	9.13051012
Ets2	62.2843501	0.20652142	0.29344364	0.70378565	0.48156627	0.99938111		Ets2	49.059382	48.4200379	52.4830407	78.5893204	79.9109626	52.9224536	43.301875	93.5877288
Bms1	9.08703016	0.28550442	0.4057328	0.70367596	0.48163459	0.99938111		Bms1	8.06455594	10.8605693	10.8146872	0	9.98887032	15.2887088	15.3962222	2.28262753
Hist1h3a	3.56553207	-0.2928468	0.41633561	-0.7033912	0.481812	NA		Hist1h3a	2.01613899	4.97776091	3.49886938	6.20442003	1.66481172	2.35210905	0.96226389	6.84788259
Rasgrp2	17.6649226	0.23684106	0.33679776	0.70321448	0.4819221	0.99938111		Rasgrp2	10.7527413	13.5757116	19.7209001	18.6132601	14.9833055	17.6408179	16.3584861	29.6741579
Ubtd1	8.0380307	0.29202882	0.41530192	0.70317234	0.48194835	0.99938111		Ubtd1	3.36023164	4.07271347	5.40734359	12.4088401	14.9833055	3.52816357	0	20.5436478
Gprasp1	404.631773	0.17262846	0.24555349	0.70301774	0.48204469	0.99938111		Gprasp1	411.292353	302.285844	385.829869	415.696142	387.901131	602.139916	421.471583	310.437344
Cdkl4	11.8285819	-0.269532	0.38346917	-0.702878	0.48213177	0.99938111		Cdkl4	16.8011582	17.648425	14.9497146	4.13628002	11.653682	11.7605452	15.3962222	2.28262753
Smurf2	22.8925527	-0.2364325	0.33639135	-0.7028494	0.48214962	0.99938111		Smurf2	31.5861774	30.3190892	24.4920857	14.4769801	11.653682	30.5774176	24.0565972	15.9783927
Dbh	0.68189467	-0.230497	0.32797085	-0.7027972	0.48218213	NA		Dbh	1.34409266	1.81009488	0.63615807	0	1.66481172	0	0	0
Wfdc1	0.68189467	-0.230497	0.32797085	-0.7027972	0.48218213	NA		Wfdc1	1.34409266	1.81009488	0.63615807	0	1.66481172	0	0	0
Cbx8	4.63483088	0.29154072	0.41489018	0.70269371	0.48224665	NA		Cbx8	5.37637063	0.90504744	1.27231614	8.27256004	9.98887032	7.05632714	1.92452778	2.28262753
D7Ertd443e	2.43077601	-0.2907993	0.41401188	-0.7023936	0.48243371	NA		D7Ertd443e	3.36023164	2.71514231	2.54463228	4.13628002	1.66481172	1.17605452	3.84905555	0
Bco2	1.92368066	-0.2860511	0.40761246	-0.7017723	0.48282118	NA		Bco2	2.68818531	3.16766603	2.22655324	2.06814001	0	2.35210905	2.88679167	0
Ammecr1l	25.2109785	-0.2123442	0.30272569	-0.701441	0.48302786	0.99938111		Ammecr1l	19.4893435	28.5089943	26.4005599	37.2265202	24.9721758	24.697145	22.1320694	18.2610202
Snx10	75.4469878	0.25332332	0.36119901	0.70134001	0.48309085	0.99938111		Snx10	90.054208	76.4765085	78.2474425	20.6814001	46.6147282	89.3801438	140.490528	61.6309433
Xpot	57.3220093	0.17649455	0.25175424	0.70105889	0.48326627	0.99938111		Xpot	52.4196136	43.442277	54.3915149	66.1804803	46.6147282	62.3308898	69.283	63.9135709
Mchr1	7.62426931	-0.2897205	0.41333063	-0.7009412	0.4833397	0.99938111		Mchr1	4.7043243	12.6706641	10.4966081	8.27256004	9.98887032	12.9365998	1.92452778	0
Gpr19	9.82252307	-0.278052	0.39675372	-0.7008176	0.48341684	0.99938111		Gpr19	13.4409266	12.6706641	11.1327662	6.20442003	3.32962344	12.9365998	2.88679167	15.9783927
Hspa13	24.6469002	0.27456235	0.39178076	0.7008061	0.48342403	0.99938111		Hspa13	20.1613899	33.9392789	23.5378486	6.20442003	13.3184938	59.9787807	24.0565972	15.9783927
Fam171b	172.722433	-0.2894566	0.41313636	-0.700632	0.48353268	0.99938111		Fam171b	173.387953	355.683643	242.694303	41.3628002	54.9387868	337.527648	151.07543	25.1089028
Abcd4	1.26183129	-0.2672899	0.38154762	-0.7005414	0.48358928	NA		Abcd4	2.01613899	2.26261859	2.22655324	0	1.66481172	0	1.92452778	0
Inca1	1.68915486	-0.2675876	0.38198359	-0.7005211	0.48360192	NA		Inca1	3.36023164	0.45252372	1.27231614	4.13628002	3.32962344	0	0.96226389	0
Vangl1	0.56663586	-0.2194216	0.31323126	-0.7005099	0.48360891	NA		Vangl1	0.67204633	2.26261859	0.63615807	0	0	0	0.96226389	0
Lhx9	3.41450401	0.29000383	0.41401566	0.70046584	0.48363643	NA		Lhx9	0.67204633	1.81009488	3.18079035	6.20442003	3.32962344	1.17605452	8.660375	2.28262753
Tmem127	95.4977815	0.20632481	0.29459379	0.70037054	0.48369593	0.99938111		Tmem127	86.02193	116.751119	91.2886829	51.7035003	114.872009	115.253343	62.5471528	125.544514
Dusp28	20.3906168	-0.2733757	0.39040707	-0.7002324	0.48378218	0.99938111		Dusp28	20.8334362	11.313093	22.9016905	37.2265202	21.6425524	8.23238167	6.73584722	34.239413
Tmem150c	18.0320232	-0.2635833	0.37646512	-0.7001532	0.48383163	0.99938111		Tmem150c	16.8011582	31.2241366	25.1282437	6.20442003	8.3240586	23.5210905	12.5094306	20.5436478
Sep-09	51.7496265	0.17432756	0.24899446	0.70012628	0.48384844	0.99938111		Sep-09	45.6991503	49.7776091	42.6225906	55.8397803	61.5980337	42.3379629	56.7735694	59.3483158
Rad51c	2.63464254	-0.2824983	0.40366401	-0.6998353	0.48403019	NA		Rad51c	0	3.62018975	4.13502745	6.20442003	1.66481172	3.52816357	1.92452778	0
Thtpa	22.5285691	0.21457817	0.30671846	0.69959327	0.48418135	0.99938111		Thtpa	17.4732045	19.0059962	21.9474534	22.7495401	19.9777406	22.345036	20.2075417	36.5220405
Asph	112.729775	0.2094813	0.29946147	0.69952671	0.48422293	0.99938111		Asph	95.4305786	131.231878	106.556477	78.5893204	61.5980337	174.05607	135.679208	118.696632
Krt12	9.49152817	-0.2832571	0.40535346	-0.6987905	0.48468298	0.99938111		Krt12	16.8011582	8.14542694	14.6316356	4.13628002	6.65924688	2.35210905	16.3584861	6.84788259
Icmt	39.9994933	0.21048138	0.30131645	0.69853928	0.48484	0.99938111		Icmt	25.5377605	40.7271347	34.0344567	47.5672202	51.6091633	56.4506172	29.8301805	34.239413
Eif3f	85.4364285	-0.2513691	0.35992307	-0.6983966	0.4849292	0.99938111		Eif3f	80.6455594	59.2806072	76.3389683	165.451201	98.2238915	29.4013631	57.7358333	116.414004
Zswim4	21.7655002	0.26504861	0.37953319	0.69835424	0.48495569	0.99938111		Zswim4	16.8011582	13.1231878	12.0870033	35.1583802	41.620293	14.1126543	11.5471667	29.6741579
Zdhhc18	21.5361509	-0.2449534	0.35083274	-0.6982055	0.48504866	0.99938111		Zdhhc18	20.8334362	24.4362808	18.766663	31.0221002	31.6314227	14.1126543	8.660375	22.8262753
Abt1	8.77430085	-0.277494	0.39748525	-0.6981241	0.4850996	0.99938111		Abt1	16.8011582	11.313093	4.45310648	8.27256004	8.3240586	5.88027262	10.5849028	4.56525506
Pgap3	4.58069181	-0.2904043	0.41605341	-0.6979975	0.48517874	NA		Pgap3	1.34409266	8.14542694	5.72542262	6.20442003	4.99443516	4.7042181	0.96226389	4.56525506
Pamr1	5.04655206	-0.2883355	0.41329448	-0.6976515	0.48539519	0.99938111		Pamr1	3.36023164	10.4080455	7.31581779	2.06814001	4.99443516	7.05632714	2.88679167	2.28262753
Sbsn	1.94158932	0.283523	0.40657454	0.69734568	0.48558648	NA		Sbsn	2.01613899	0.90504744	1.27231614	2.06814001	4.99443516	2.35210905	1.92452778	0
Nol7	16.5822098	0.26905295	0.38583033	0.69733487	0.48559325	0.99938111		Nol7	18.1452509	14.0282353	17.1762679	8.27256004	4.99443516	18.8168724	37.5282917	13.6957652
Slc25a47	0.9048168	0.22188275	0.31821787	0.69726679	0.48563585	NA		Slc25a47	0.67204633	0	0.9542371	0	3.32962344	0	0	2.28262753
Nit1	18.8615008	0.23458976	0.33652299	0.69709875	0.485741	0.99938111		Nit1	17.4732045	15.8383302	16.2220308	18.6132601	21.6425524	9.40843619	28.8679167	22.8262753
Spg11	14.4582581	0.23344838	0.33489975	0.69706945	0.48575933	0.99938111		Spg11	16.8011582	13.1231878	13.0412404	8.27256004	19.9777406	17.6408179	15.3962222	11.4131377
Caskin2	15.9746713	-0.2779825	0.39885315	-0.6969545	0.48583125	0.99938111		Caskin2	10.0806949	16.7433776	13.9954775	33.0902402	24.9721758	2.35210905	10.5849028	15.9783927
Cadps2	52.9016444	0.21708663	0.31156173	0.69676926	0.48594721	0.99938111		Cadps2	61.1562159	41.6321821	55.345752	35.1583802	53.2739751	69.3872169	79.8679027	27.3915304

GeneID	Base mean	log2(FC)	StdErr	Wald-Stats	P-value	P-adj
Gm20187	0.52365801	0.21393206	0.30705459	0.69672323	0.48597602	NA
Nceh1	81.3890736	0.25967731	0.37273938	0.69667258	0.48600772	0.99938111
Usp46	146.08556	0.15577781	0.22361663	0.69662891	0.48603506	0.99938111
St6galnac4	19.1979508	0.25817883	0.37068982	0.69648213	0.48612695	0.99938111
Gpaa1	17.4982766	-0.2461456	0.35341695	-0.6964737	0.48613219	0.99938111
Mgll	87.9763269	-0.219585	0.31528204	-0.6964717	0.48613348	0.99938111
Eepd1	9.18461374	0.26692522	0.38326331	0.69645388	0.48614463	0.99938111
Pde4d	94.9255374	0.18570467	0.26680027	0.69604377	0.48640142	0.99938111
Aco1	17.2735515	-0.2399349	0.34474649	-0.695975	0.48644449	0.99938111
Sash3	0.59239137	-0.216752	0.31152553	-0.6957762	0.48656901	NA
Sdf4	131.210916	0.1928585	0.27737061	0.69530979	0.48686118	0.99938111
Il6ra	0.66224471	-0.2036273	0.29286481	-0.6952945	0.48687078	NA
Mctp1	36.7650807	-0.2386369	0.34323254	-0.695263	0.4868905	0.99938111
Gm10409	0.61462917	-0.2220906	0.31943543	-0.6952597	0.48689258	NA
Rbbp7	33.7930246	0.18697205	0.26893279	0.69523707	0.48690674	0.99938111
Tmem181a	38.4977257	0.22410133	0.32236719	0.69517412	0.48694619	0.99938111
Nwd2	56.4564632	0.22708988	0.32668772	0.6951283	0.4869749	0.99938111
Sec14l2	17.4697132	-0.245166	0.35271871	-0.6950752	0.48700817	0.99938111
Pard6a	18.9096388	0.28395219	0.40855111	0.69502244	0.48704124	0.99938111
Mfsd9	5.29737913	0.28018531	0.40320037	0.6949034	0.48711584	0.99938111
Cutc	3.1043507	0.28923668	0.41625572	0.69485335	0.48714721	NA
Cyfip2	794.576706	0.15401795	0.22174358	0.69457682	0.48732054	0.99938111
Xk	22.3204366	0.22308527	0.32131517	0.694288	0.48750161	0.99938111
Jsrp1	0.94365193	-0.2416291	0.34804285	-0.694251	0.48752481	NA
Gfod2	22.9483788	0.2777241	0.40004853	0.69422602	0.48754048	0.99938111
4933426M11Rik	22.6442061	-0.2331244	0.33585022	-0.6941321	0.48759938	0.99938111
Apaf1	16.6639646	-0.2383104	0.34337094	-0.6940319	0.48766219	0.99938111
Tmem255a	52.8234386	-0.2508105	0.36139247	-0.6940114	0.48767504	0.99938111
Vwa5b1	9.52747108	-0.2887517	0.41611373	-0.6939249	0.48772931	0.99938111
Ppp1r37	117.176868	0.2485233	0.35817748	0.69385517	0.48777304	0.99938111
Rb1	25.3353218	-0.2765595	0.39861282	-0.6938049	0.48780454	0.99938111
Dtd1	31.7517389	0.21320896	0.30743624	0.69350627	0.48799189	0.99938111
Frmd4b	7.01913817	-0.2759444	0.39789764	-0.6935059	0.48799213	0.99938111
Cth	0.90795604	-0.2305435	0.33249185	-0.6933809	0.48807057	NA
Bbs5	7.27596815	0.2826211	0.40766384	0.69326996	0.48814015	0.99938111
Ptges	1.94573173	-0.274744	0.39630264	-0.6932682	0.48814123	NA
Nup98	40.4722936	0.18841288	0.27180707	0.6931861	0.48819277	0.99938111
Spsb1	10.2535319	-0.2855367	0.41207496	-0.6929242	0.48835711	0.99938111
Fendrr	0.8935088	0.22019804	0.31780738	0.69286635	0.48839343	NA
Vps8	29.321981	0.19821505	0.28613015	0.69274438	0.48846999	0.99938111
Elmo3	0.82148435	0.22953574	0.33141685	0.69258921	0.48856739	NA
BC025920	1.08812227	-0.2531651	0.36553799	-0.6925822	0.48857176	NA
Xndc1	9.28769654	-0.2862605	0.41338493	-0.6924793	0.48863641	0.99938111
Gm15441	0.66508908	-0.2285738	0.33008761	-0.6924641	0.48864594	NA
Psmc3	46.1654424	0.21128546	0.3051854	0.69231838	0.48873741	0.99938111
Slc37a3	26.9739855	-0.2338511	0.33779643	-0.6922842	0.48875884	0.99938111
Lig4	12.2343155	0.25244488	0.36486785	0.69188031	0.4890125	0.99938111
Gmppa	19.7939893	0.25229668	0.36471652	0.69176103	0.48908742	0.99938111
Neu2	1.24046681	-0.2403061	0.34742518	-0.6916773	0.48914003	NA
Fkbp1a	475.507011	-0.2245305	0.32464269	-0.6916236	0.48917374	0.99938111
Smarcc1	68.2249857	0.15277711	0.22098098	0.69135863	0.48934019	0.99938111
Kdelc1	2.80598702	0.28644311	0.41433292	0.69133562	0.48935465	NA
Gpr111	0.38494992	0.18356066	0.26554228	0.69126719	0.48939765	NA
Gm14057	2.0168882	-0.2800751	0.40516514	-0.6912616	0.48940117	NA
Rrn3	59.3650023	-0.192859	0.27900609	-0.6912357	0.48941745	0.99938111
Arhgef2	52.080916	0.18508326	0.26781232	0.69109315	0.489507	0.99938111
Zfp426	18.1021414	0.25887954	0.37461452	0.69105581	0.48953047	0.99938111
Khnyn	8.21421142	0.27393377	0.39641016	0.69103618	0.4895428	0.99938111
Rpa3	1.79899795	-0.2724004	0.39425801	-0.690919	0.48961643	NA
Wdfy1	9.12873867	0.25024722	0.36226251	0.69078973	0.48969769	0.99938111
9530026P05Rik	0.39427344	-0.1607166	0.23266143	-0.6907746	0.4897072	NA
Cyp39a1	0.39427344	-0.1607166	0.23266143	-0.6907746	0.4897072	NA
Ppp1r10	46.5024342	0.18930736	0.27405462	0.69076506	0.4897132	0.99938111
Pnpla3	1.45542	0.27007072	0.39097524	0.6907617	0.48971531	NA
Tbrg1	21.5169358	-0.2245514	0.32509726	-0.6907208	0.48974102	0.99938111
Amz2	36.5702539	-0.1877688	0.27185217	-0.6907018	0.48975296	0.99938111
Triap1	6.32488525	-0.2810245	0.40689174	-0.6906616	0.48977821	0.99938111
Fbxw9	10.5943827	0.27352847	0.39606516	0.6906148	0.48980764	0.99938111
Mettl20	1.64668165	0.26847326	0.38875569	0.69059635	0.48981924	NA
Ubox5	15.5263337	0.25873431	0.37476934	0.6903828	0.48995349	0.99938111
Efnb2	41.3189994	0.25349096	0.3672112	0.6903138	0.48999687	0.99938111
Cntln	10.3258895	0.28713409	0.41599587	0.69023303	0.49004765	0.99938111
Lzic	7.10486226	-0.2737968	0.39671565	-0.6901588	0.49009435	0.99938111
Mrpl21	2.48504879	0.27406236	0.39719953	0.68998661	0.49020261	NA
Lrguk	0.97382285	-0.2427846	0.3519163	-0.6898929	0.49026152	NA
Pla2g4a	0.73905485	0.2183874	0.31655516	0.68988737	0.49026502	NA
Xrcc4	2.75385875	0.27241157	0.39488	0.68985912	0.49028278	NA
Klhdc7a	3.80524457	-0.2777709	0.40265988	-0.6898401	0.49029472	NA
Casp8ap2	13.2251215	0.27266337	0.39540903	0.68957295	0.49046278	0.99938111
Jmjd4	32.7944008	-0.1850247	0.2683277	-0.6895476	0.49047875	0.99938111
Cdan1	9.75671129	0.26158147	0.37939486	0.68947024	0.4905274	0.99938111
Ddx31	1.97577354	-0.2796596	0.40562453	-0.6894543	0.49053741	NA
Gm10421	0.48309455	-0.1837403	0.26652085	-0.6894031	0.49056963	NA
Rpl28	11.6125842	0.26463285	0.38391224	0.6893056	0.49063097	0.99938111
Stard3nl	14.3422547	-0.2589898	0.37573441	-0.6892896	0.49064105	0.99938111
A230056P14Rik	21.6249289	0.24549573	0.35619743	0.68921254	0.49068953	0.99938111
Rdh11	7.54566679	-0.2744787	0.39827612	-0.6891669	0.49071825	0.99938111
Gemin6	1.79218919	-0.2742251	0.39791465	-0.6891556	0.49072536	NA
Ccl21a	1.19644961	-0.2286414	0.33178149	-0.6891325	0.49073987	NA
Gm1987	1.19644961	-0.2286414	0.33178149	-0.6891325	0.49073987	NA
Rbpms2	1.97533039	-0.2730869	0.3962814	-0.6891237	0.49074544	NA
Uba2	44.255791	0.18711986	0.27159171	0.68897487	0.49083908	0.99938111
Nfatc2ip	3.52655508	0.28682215	0.41632443	0.68893903	0.49086163	NA
Tacr3	3.4960962	-0.2817267	0.40893752	-0.6889235	0.4908714	NA
Rps6ka3	35.0715892	-0.2261229	0.32823236	-0.6889112	0.49087915	0.99938111
Fam111a	0.31641636	-0.1812386	0.26317701	-0.6886565	0.49103944	NA
Fbxo5	0.31641636	-0.1812386	0.26317701	-0.6886565	0.49103944	NA
Glp1r	0.31641636	-0.1812386	0.26317701	-0.6886565	0.49103944	NA
Vsig10	0.31641636	-0.1812386	0.26317701	-0.6886565	0.49103944	NA
Naa10	5.80224727	0.27896326	0.40517035	0.68850858	0.49113257	0.99938111
Tubb3	261.67889	0.25803576	0.37478032	0.6884987	0.49113879	0.99938111
Clvs2	31.7881735	0.18894142	0.27447131	0.68838313	0.49121155	0.99938111
Capn7	31.9452741	0.23493122	0.34135043	0.68824059	0.49130129	0.99938111
Brcc3	14.4605728	0.26763099	0.38891961	0.68813961	0.49136487	0.99938111
Pno1	14.0121957	0.26818375	0.3897838	0.68803206	0.49143259	0.99938111
Zcchc10	5.97582434	-0.2773413	0.40309694	-0.6880263	0.49143621	0.99938111
Fam195b	58.5949034	-0.2736974	0.39805573	-0.6875857	0.49171371	0.99938111
Lars	18.7470451	0.25057029	0.36445878	0.68751339	0.49175926	0.99938111
Arl5a	92.5264892	0.22829859	0.3321354	0.68736602	0.49185211	0.99938111
Bmp5	0.36849579	-0.1612257	0.23456599	-0.6873364	0.49187077	NA
Tnik	103.319415	0.19379958	0.2820499	0.68711099	0.49201279	0.99938111
Hspbap1	2.24585998	-0.2801689	0.40775013	-0.6871094	0.49201382	NA

GeneID	Normalised expression for Chow#1	Normalised expression for Chow#2	Normalised expression for Chow#3	Normalised expression for Chow#4	Normalised expression for HFD#1	Normalised expression for HFD#2	Normalised expression for HFD#3	Normalised expression for HFD#4
Gm20187	0	0.45252372	0.63615807	0	0	1.17605452	1.92452778	0
Nceh1	51.075521	123.086452	78.5655215	31.0221002	44.9499164	172.880015	67.3584722	82.1745911
Usp46	122.312432	154.763112	153.314095	119.952121	141.508996	171.703961	181.867875	123.261887
St6galnac4	16.1291119	21.2686148	11.7689243	16.5451201	29.966611	11.7605452	9.62263889	36.5220405
Gpaa1	18.1452509	21.7211385	17.4943469	18.6132601	24.9721758	11.7605452	6.73584722	20.5436478
Mgll	115.591968	98.197647	87.7898135	80.6574604	131.520126	37.6337448	70.2452639	82.1745911
Eepd1	9.4086486	6.33533207	10.4966081	6.20442003	9.98887032	15.2887088	13.4716944	2.28262753
Pde4d	100.134903	78.2866034	104.648002	68.2486203	66.5924688	131.718107	111.622611	98.1529838
Aco1	16.8011582	19.9110436	19.0847421	22.7495401	14.9833055	24.697145	15.3962222	4.56525506
Sash3	2.68818531	0.45252372	0.63615807	0	0	0	0.96226389	0
Sdf4	106.855366	152.500493	131.366641	93.0663005	98.2238915	209.337705	132.792417	125.544514
Il6ra	0	3.16766603	0.9542371	0	0	1.17605452	0	0
Mctp1	48.3873356	53.3977988	47.0756971	12.4088401	33.2962344	37.6337448	41.3773472	20.5436478
Gm10409	2.01613899	0.45252372	1.27231614	0	0	1.17605452	0	0
Rbbp7	30.9141311	28.5089943	29.2632712	39.2946602	34.9610461	41.1619083	36.5660278	29.6741579
Tmem181a	30.2420848	50.6826565	36.2610099	18.6132601	39.9554813	48.2182355	26.9433889	57.0656883
Nwd2	64.5164475	51.1351802	59.7988585	26.8858201	93.2294563	74.091435	50.0377222	31.9567854
Sec14l2	22.8495752	21.2686148	18.130505	16.5451201	19.9777406	22.345036	16.3584861	2.28262753
Pard6a	12.0968339	8.14542694	10.4966081	33.0902402	24.9721758	5.88027262	8.660375	47.9351782
Mfsd9	2.68818531	5.43028463	5.72542262	4.13628002	8.3240586	7.05632714	6.73584722	2.28262753
Cutc	3.36023164	1.35757116	3.18079035	2.06814001	4.99443516	4.7042181	2.88679167	2.28262753
Cyfip2	864.923625	716.345047	805.058036	605.965023	1087.12205	856.167694	729.396028	691.636142
Xk	19.4893435	25.3413283	19.0847421	16.5451201	21.6425524	37.6337448	18.2830139	20.5436478
Jsrp1	1.34409266	2.26261859	1.59039517	0	0	2.35210905	0	0
Gfod2	20.1613899	6.78785578	11.1327662	41.3628002	28.3017992	10.5844907	17.32075	47.9351782
4933426M11R	16.1291119	33.9392789	22.5836114	26.8858201	24.9721758	25.8731995	12.5094306	18.2610202
Apaf1	16.8011582	22.6261859	19.7209001	14.4769801	19.9777406	9.40843619	21.1698055	9.13051012
Tmem255a	32.2582238	84.6219354	65.5242811	53.7716403	34.9610461	92.9083074	28.8679167	29.6741579
Vwa5b1	2.01613899	6.78785578	5.40734359	33.0902402	9.98887032	8.23238167	3.84905555	6.84788259
Ppp1r37	94.086486	86.4320303	83.336707	150.974221	144.83862	74.091435	54.8490416	248.806401
Rb1	32.2582238	37.5594687	40.3960374	6.20442003	14.9833055	28.2253086	38.4905555	4.56525506
Dtd1	31.5861774	20.8160911	29.8994292	33.0902402	31.6314227	21.1689814	35.6037639	50.2178057
Frmd4b	7.39250961	12.2181404	9.54237104	2.06814001	3.32962344	7.05632714	7.69811111	6.84788259
Cth	0	2.26261859	2.86271131	0	0	1.17605452	0.96226389	0
Bbs5	10.7527413	5.88280835	4.45310648	4.13628002	3.32962344	12.9365998	14.4339583	2.28262753
Ptges	1.34409266	3.62018975	1.27231614	4.13628002	1.66481172	3.52816357	0	0
Nup98	39.6507334	33.9392789	38.4875632	39.2946602	46.6147282	57.6266717	38.4905555	29.6741579
Spsb1	10.0806949	12.6706641	5.72542262	18.6132601	6.65924688	3.52816357	1.92452778	22.8262753
Fendrr	0	0.45252372	0.9542371	0	0	1.17605452	0	4.56525506
Vps8	28.8979921	25.793852	32.7621406	18.6132601	28.3017992	29.4013631	36.5660278	34.239413
Elmo3	1.34409266	0	0.31807903	0	1.66481172	0	0.96226389	2.28262753
BC025920	1.34409266	1.35757116	2.54463228	0	0	1.17605452	0	2.28262753
Xndc1	7.39250961	5.88280835	6.36158069	24.8176801	4.99443516	1.17605452	7.69811111	15.9783927
Gm15441	1.34409266	1.35757116	0.9542371	0	1.66481172	0	0	0
Psmc3	61.8282622	34.8443264	41.6683535	28.9539601	43.2851047	35.2816357	66.3962083	57.0656883
Slc37a3	20.8334362	43.8948007	33.0802196	20.6814001	18.3129289	37.6337448	23.0943333	18.2610202
Lig4	14.1129729	12.2181404	9.54237104	6.20442003	9.98887032	21.1689814	8.660375	15.9783927
Gmppa	16.1291119	20.3635674	16.8581888	14.4769801	38.2906696	12.9365998	9.62263889	29.6741579
Neu2	5.37637063	0.45252372	0.63615807	0	0	1.17605452	0	2.28262753
Fkbp1a	471.776523	437.137912	441.811779	732.121564	454.4936	189.344778	374.320653	703.04928
Smarcc1	67.2046328	63.3533207	66.4785182	59.9760603	76.5813391	79.9717076	68.3207361	63.9135709
Kdelc1	4.7043243	2.26261859	1.59039517	0	1.66481172	7.05632714	2.88679167	2.28262753
Gpr111	0	0.45252372	0	0	1.66481172	0	0.96226389	0
Gm14057	2.68818531	3.16766603	2.54463228	2.06814001	0	4.7042181	0.96226389	0
Rrn3	65.8605402	71.9512713	72.5220199	41.3628002	58.2684102	36.4576902	57.7358333	70.7614535
Arhgef2	60.4841696	43.8948007	49.6203294	37.2265202	74.9165274	54.0985081	46.1886666	50.2178057
Zfp426	14.1129729	16.7433776	20.9932163	12.4088401	9.98887032	32.9295267	30.7924444	6.84788259
Khnyn	3.36023164	8.59795066	6.04350166	10.3407001	14.9833055	9.40843619	3.84905555	9.13051012
Rpa3	0.67204633	2.26261859	4.13502745	2.06814001	3.32962344	0	1.92452778	0
Wdfy1	8.73660227	8.14542694	8.90621297	6.20442003	9.98887032	9.40843619	12.5094306	9.13051012
9530026P05Ri	1.34409266	1.81009488	0	0	0	0	0	0
Cyp39a1	1.34409266	1.81009488	0	0	0	0	0	0
Ppp1r10	38.3066407	48.4200379	41.3502745	41.3628002	48.2795399	44.6900719	36.5660278	73.044081
Pnpla3	1.34409266	0.90504744	0.63615807	2.06814001	1.66481172	1.17605452	3.84905555	0
Tbrg1	28.2259458	19.4585199	19.4028211	28.9539601	13.3184938	15.2887088	26.9433889	20.5436478
Amz2	41.6668724	39.3695635	38.1694841	39.2946602	23.3073641	36.4576902	42.3396111	31.9567854
Triap1	12.0968339	5.88280835	6.36158069	4.13628002	8.3240586	1.17605452	5.77358333	6.84788259
Fbxw9	5.37637063	5.43028463	5.72542262	22.7495401	13.3184938	8.23238167	12.5094306	11.4131377
Mettl20	0.67204633	3.16766603	0.63615807	0	0	3.52816357	2.88679167	2.28262753
Ubox5	12.0968339	15.3858064	14.3135566	10.3407001	24.9721758	5.88027262	11.5471667	29.6741579
Efnb2	28.2259458	47.0624668	53.4372778	14.4769801	24.9721758	76.4435441	28.8679167	57.0656883
Cntln	10.7527413	8.14542694	9.224292	6.20442003	1.66481172	23.5210905	23.0943333	0
Lzic	7.39250961	8.59795066	8.2700549	8.27256004	6.65924688	1.17605452	9.62263889	6.84788259
Mrpl21	2.01613899	0.90504744	0.63615807	4.13628002	6.65924688	0	0.96226389	4.56525506
Lrguk	0	1.35757116	2.22655324	2.06814001	0	1.17605452	0.96226389	0
Pla2g4a	0	0.45252372	0.9542371	0	3.32962344	1.17605452	0	0
Xrcc4	2.68818531	2.26261859	3.18079035	0	0	2.35210905	11.5471667	0
Klhdc7a	5.37637063	9.05047438	4.45310648	0	3.32962344	8.23238167	0	0
Casp8ap2	9.4086486	14.9332827	10.1785291	12.4088401	6.65924688	25.8731995	24.0565972	2.28262753
Jmjd4	36.2905017	35.7493738	31.1717454	37.2265202	28.3017992	28.2253086	28.8679167	36.5220405
Cdan1	6.72046328	7.2403795	7.95197586	12.4088401	18.3129289	8.23238167	5.77358333	11.4131377
Ddx31	4.7043243	1.35757116	1.59039517	2.06814001	1.66481172	1.17605452	0.96226389	2.28262753
Gm10421	1.34409266	0.45252372	0	2.06814001	0	0	0	0
Rpl28	11.4247876	8.14542694	7.95197586	12.4088401	18.3129289	3.52816357	10.5849028	20.5436478
Stard3nl	12.0968339	22.6261859	18.448584	12.4088401	9.98887032	17.6408179	19.2452778	2.28262753
A230056P14Ri	9.4086486	21.7211385	26.4005599	18.6132601	16.6481172	36.4576902	16.3584861	27.3915304
Rdh11	8.06455594	12.6706641	8.2700549	6.20442003	6.65924688	11.7605452	6.73584722	0
Gemin6	1.34409266	2.71514231	1.59039517	4.13628002	1.66481172	0	2.88679167	0
Ccl21a	0.67204633	0.45252372	0.31807903	6.20442003	0	0	1.92452778	0
Gm1987	0.67204633	0.45252372	0.31807903	6.20442003	0	0	1.92452778	0
Rbpms2	1.34409266	4.52523719	1.90847421	2.06814001	4.99443516	0	0.96226389	0
Uba2	47.7152893	40.7271347	39.7598793	37.2265202	36.6258578	52.9224536	62.5471528	36.5220405
Nfatc2ip	1.34409266	5.43028463	3.18079035	0	4.99443516	3.52816357	2.88679167	6.84788259
Tacr3	1.34409266	8.14542694	5.40734359	2.06814001	1.66481172	7.05632714	0	2.28262753
Rps6ka3	38.3066407	49.7776091	46.439539	18.6132601	21.6425524	43.5140174	39.4528194	22.8262753
Fam111a	0.67204633	0.90504744	0.9542371	0	0	0	0	0
Fbxo5	0.67204633	0.90504744	0.9542371	0	0	0	0	0
Glp1r	0.67204633	0.90504744	0.9542371	0	0	0	0	0
Vsig10	0.67204633	0.90504744	0.9542371	0	0	0	0	0
Naa10	5.37637063	5.43028463	4.77118552	4.13628002	4.99443516	2.35210905	12.5094306	6.84788259
Tubb3	206.318223	132.58945	161.26607	417.764282	396.225189	123.485725	155.88675	499.895429
Clvs2	25.5377605	30.7716129	27.0367179	35.1583802	36.6258578	27.0492541	35.6037639	36.5220405
Capn7	30.9141311	25.793852	37.8514051	20.6814001	23.3073641	52.9224536	48.1131944	15.9783927
Brcc3	18.1452509	12.6706641	11.7689243	8.27256004	6.65924688	17.6408179	33.6792361	6.84788259
Pno1	15.4570656	12.6706641	17.1762679	2.06814001	6.65924688	16.4647633	27.9056528	13.6957652
Zcchc10	5.37637063	5.88280835	7.63389683	10.3407001	4.99443516	5.88027262	7.69811111	0
Fam195b	47.7152893	33.0342315	33.7163777	155.110501	58.2684102	18.8168724	30.7924444	91.3051012
Lars	25.5377605	14.480759	14.6316356	12.4088401	14.9833055	37.6337448	21.1698055	9.13051012
Arl5a	78.6294204	87.7896015	101.785291	66.1804803	43.2851047	111.72518	173.2075	77.6093361
Bmp5	0	1.35757116	1.59039517	0	0	0	0	0
Tnik	123.656524	86.884554	100.512975	70.3167603	93.2294563	164.647633	109.698083	77.6093361
Hspbap1	3.36023164	4.52523719	2.54463228	0	3.32962344	0	1.92452778	2.28262753

GeneID	Base mean	log2(FC)	StdErr	Wald-Stats	P-value	P-adj	GeneID	Normalised expression for Chow#1	Normalised expression for Chow#2	Normalised expression for Chow#3	Normalised expression for Chow#4	Normalised expression for HFD#1	Normalised expression for HFD#2	Normalised expression for HFD#3	Normalised expression for HFD#4
Maz	121.57097	0.25201639	0.36678477	0.68709611	0.49202216	0.99938111	Maz	83.3337447	76.4765085	73.794336	196.473301	184.794101	63.5069443	75.0565833	219.132243
Trim66	59.2771138	0.25514993	0.37145246	0.68689794	0.49214705	0.99938111	Trim66	31.5861774	31.2241366	36.2610099	111.679561	78.2461509	48.2182355	36.5660278	100.435611
Icam5	79.8688276	0.25221765	0.36722209	0.68682592	0.49219244	0.99938111	Icam5	70.5648645	98.6501707	62.6615698	47.5672202	159.821925	78.7956531	25.0188611	95.8703563
Mgat1	46.1757755	0.27015785	0.3933646	0.6867874	0.49221671	0.99938111	Mgat1	25.5377605	29.8665654	25.4463228	78.5893204	51.6091633	28.2253086	18.2830139	111.848749
Hnrnpf	17.1539923	-0.2436686	0.35483799	-0.6867039	0.49226933	0.99938111	Hnrnpf	30.2420848	16.7433776	17.4943469	12.4088401	11.653682	16.4647633	23.0943333	9.13051012
Mapkap1	33.4559668	-0.2245311	0.32700022	-0.686639	0.49231024	0.99938111	Mapkap1	44.3550577	43.8948007	39.1237212	18.6132601	16.6481172	35.2816357	42.3396111	27.3915304
Ccdc153	0.93477947	-0.2417155	0.35209948	-0.6864978	0.49239923	NA	Ccdc153	0.67204633	0.90504744	1.90847421	2.06814001	0	0	1.92452778	0
Slc35e4	13.1545885	0.2576444	0.37530536	0.68649273	0.49240245	0.99938111	Slc35e4	8.73660227	9.95552182	7.95197586	20.6814001	19.9777406	12.9365998	6.73584722	18.2610202
Cox15	25.5117502	0.2198102	0.32019611	0.68648614	0.4924066	0.99938111	Cox15	19.4893435	18.5534725	20.9932163	35.1583802	39.9554813	22.345036	20.2075417	27.3915304
Spata17	0.63631464	-0.1696159	0.24712144	-0.6863664	0.49248209	NA	Spata17	0	0	0.9542371	4.13628002	0	0	0	0
Kpna2	2.06732597	-0.2809884	0.40957111	-0.6860553	0.49267821	NA	Kpna2	2.68818531	1.81009488	3.81694841	2.06814001	1.66481172	3.52816357	0.96226389	0
Mir692-2a	0.94455512	0.24367452	0.35519214	0.68603579	0.49269054	NA	Mir692-2a	0.67204633	0.90504744	0.9542371	0	0	1.17605452	3.84905555	0
Mir692-2b	0.94455512	0.24367452	0.35519214	0.68603579	0.49269054	NA	Mir692-2b	0.67204633	0.90504744	0.9542371	0	0	1.17605452	3.84905555	0
Chchd3	28.7341817	-0.2602264	0.37933176	-0.6860127	0.4927051	0.99938111	Chchd3	40.994826	39.8220873	34.3525357	14.4769801	6.65924688	25.8731995	47.1509305	20.5436478
Zfp661	2.6514625	-0.2843204	0.41459765	-0.6857741	0.49285554	NA	Zfp661	4.7043243	3.16766603	4.77118552	0	3.32962344	2.35210905	2.88679167	0
Chd9	49.3957623	0.20988651	0.30607574	0.6857339	0.49288093	0.99938111	Chd9	40.3227797	52.4927514	58.2084633	28.9539601	49.9443516	68.2111624	67.3584722	29.6741579
Lrrc10b	9.03105637	-0.2790091	0.40696935	-0.6855777	0.49297945	0.99938111	Lrrc10b	10.7527413	6.33533207	6.04350166	18.6132601	8.3240586	2.35210905	3.84905555	15.9783927
Wdr74	11.471353	0.25227466	0.36798031	0.68556566	0.49298704	0.99938111	Wdr74	12.0968339	7.69290322	8.58813393	12.4088401	4.99443516	12.9365998	12.5094306	20.5436478
Lias	14.3852803	0.23152949	0.33787714	0.68524759	0.4931877	0.99938111	Lias	13.4409266	9.95552182	14.9497146	14.4769801	13.3184938	21.1689814	16.3584861	11.4131377
Rin1	36.8088106	0.25412551	0.37089394	0.6851703	0.49323646	0.99938111	Rin1	44.3550577	37.106945	30.2175083	14.4769801	56.6035985	18.8168724	22.1320694	70.7614535
Phlpp1	79.0817584	-0.2143737	0.31290799	-0.6851014	0.49327992	0.99938111	Phlpp1	86.6939764	70.1411764	74.112415	113.747701	119.866444	51.7463991	43.301875	73.044081
Gm10754	1.19290312	-0.257532	0.37593817	-0.6850382	0.49331981	NA	Gm10754	3.36023164	1.81009488	1.27231614	0	0	1.17605452	1.92452778	0
Sdccag3	31.2668693	0.19680246	0.28729046	0.68502958	0.49332525	0.99938111	Sdccag3	30.2420848	23.5312334	23.8559276	39.2946602	34.9610461	30.5774176	28.8679167	38.804668
BC029722	103.735134	-0.2521511	0.36824064	-0.6847454	0.49350461	0.99938111	BC029722	88.7101153	68.7836053	78.2474425	229.563541	101.553515	41.1619083	71.2075278	150.653417
Plcd4	5.69834004	-0.275756	0.40272088	-0.6847324	0.49351282	0.99938111	Plcd4	6.72046328	8.59795066	7.31581779	2.06814001	6.65924688	3.52816357	3.84905555	6.84788259
Hist1h1e	36.8570823	0.25454943	0.37183346	0.68457914	0.49360953	0.99938111	Hist1h1e	20.1613899	22.1736622	34.0344567	53.7716403	28.3017992	16.4647633	42.3396111	77.6093361
Zdhhc15	11.798513	0.24675065	0.36047343	0.68451829	0.49364794	0.99938111	Zdhhc15	6.04841696	14.480759	11.1327662	10.3407001	13.3184938	16.4647633	13.4716944	9.13051012
Ftsj2	7.3102417	0.28497826	0.41636256	0.68444738	0.49369271	0.99938111	Ftsj2	8.06455594	3.62018975	3.18079035	8.27256004	9.98887032	0	4.81131944	20.5436478
Zbtb18	139.952732	0.21497127	0.31415803	0.68427748	0.49379996	0.99938111	Zbtb18	143.817914	164.26611	136.137827	64.1123403	144.83862	127.013889	236.716917	102.718239
Ypel1	10.8235644	-0.2580776	0.37717863	-0.6842318	0.49382878	0.99938111	Ypel1	14.1129729	12.2181404	17.1762679	4.13628002	8.3240586	7.05632714	14.4339583	9.13051012
Cyc1	127.676181	0.2570423	0.37569551	0.68417719	0.49386329	0.99938111	Cyc1	92.7423933	74.2138899	92.560999	188.200741	159.821925	44.6900719	95.264125	273.915304
Kcnj2	8.28475973	-0.2728041	0.39877167	-0.684111	0.49390505	0.99938111	Kcnj2	6.04841696	13.5757116	13.0412404	4.13628002	11.653682	9.40843619	3.84905555	4.56525506
Aven	2.55338248	0.28421495	0.41547275	0.68407603	0.49392716	NA	Aven	1.34409266	2.71514231	2.22655324	2.06814001	1.66481172	2.35210905	5.77358333	2.28262753
Fscn1	184.717672	0.2434093	0.3558599	0.68400318	0.49397316	0.99938111	Fscn1	161.291119	125.801594	140.272854	229.563541	341.286403	87.0280348	104.886764	287.611069
Zfp804a	57.3039816	0.26531576	0.3879926	0.68381655	0.49409102	0.99938111	Zfp804a	57.1239379	52.0402277	60.7530956	28.9539601	24.9721758	139.950488	74.0943194	20.5436478
Phkg1	4.89598097	-0.281564	0.41176952	-0.6837904	0.49410756	0.99938111	Phkg1	10.0806949	5.88280835	4.77118552	2.06814001	4.99443516	2.35210905	6.73584722	2.28262753
Zfp317	16.9808808	-0.2609854	0.38184383	-0.6834872	0.49429905	0.99938111	Zfp317	21.5054825	20.8160911	24.8101647	10.3407001	6.65924688	21.1689814	25.981125	4.56525506
Pnp	6.85062201	-0.2711522	0.39672622	-0.6834743	0.49430719	0.99938111	Pnp	9.4086486	6.33533207	7.31581779	8.27256004	3.32962344	2.35210905	8.660375	9.13051012
Pld5	12.6882946	-0.2448371	0.35824843	-0.6834281	0.49433635	0.99938111	Pld5	13.4409266	13.1231878	17.1762679	10.3407001	9.98887032	8.23238167	8.660375	20.5436478
Smad4	35.3775076	-0.2201073	0.32222973	-0.6830756	0.49455909	0.99938111	Smad4	38.978687	43.8948007	51.8468826	18.6132601	21.6425524	34.1055812	44.2641389	29.6741579
Chd4	109.991657	0.16760692	0.24542963	0.68291233	0.49466225	0.99938111	Chd4	115.591968	98.197647	93.5152361	105.475141	111.542385	161.11947	89.4905416	105.000866
Rnf2	19.5344459	-0.2665968	0.39042449	-0.6828382	0.49470908	0.99938111	Rnf2	29.5700384	20.3635674	20.0389792	20.6814001	4.99443516	15.2887088	38.4905555	6.84788259
Trmt6	15.0406521	0.25275298	0.37020664	0.68273486	0.4947744	0.99938111	Trmt6	16.8011582	11.7656167	14.9497146	10.3407001	6.65924688	24.697145	25.981125	9.13051012
Hspb11	3.76393738	0.27901218	0.40870316	0.68267683	0.49481108	NA	Hspb11	2.68818531	2.71514231	6.04350166	0	1.66481172	3.52816357	13.4716944	0
Hspd1	36.8369132	0.20781819	0.30451727	0.68245123	0.49495367	0.99938111	Hspd1	41.6668724	31.6766603	37.215247	24.8176801	29.966611	49.39429	54.8490416	25.1089028
Acot12	0.45116819	-0.1867563	0.27370927	-0.6823162	0.49503901	NA	Acot12	0	0.90504744	0.63615807	2.06814001	0	0	0	0
Dnaic2	0.45116819	-0.1867563	0.27370927	-0.6823162	0.49503901	NA	Dnaic2	0	0.90504744	0.63615807	2.06814001	0	0	0	0
Trim26	16.2624256	0.24701399	0.36203415	0.68229471	0.49505262	0.99938111	Trim26	12.7688802	8.59795066	13.9954775	24.8176801	24.9721758	22.345036	13.4716944	9.13051012
Txnl4b	10.0970975	-0.2607058	0.38216726	-0.6821771	0.49512695	0.99938111	Txnl4b	11.4247876	8.59795066	7.63389683	18.6132601	11.653682	4.7042181	6.73584722	11.4131377
Emilin2	0.87858797	0.22491742	0.32974301	0.68209915	0.49517626	NA	Emilin2	0	0.90504744	0.9542371	0	0	0	2.88679167	2.28262753
Qtrtd1	5.9690803	-0.2756334	0.40422393	-0.6818828	0.49531305	0.99938111	Qtrtd1	5.37637063	4.97776091	6.04350166	12.4088401	8.3240586	3.52816357	4.81131944	2.28262753
Glt25d1	17.6747781	-0.2477016	0.36327711	-0.681853	0.49533193	0.99938111	Glt25d1	15.4570656	28.5089943	23.8559276	8.27256004	23.3073641	14.1126543	9.62263889	18.2610202
Il17rc	1.08908166	-0.2512368	0.36854284	-0.681703	0.49542677	NA	Il17rc	0.67204633	0.90504744	2.22655324	2.06814001	1.66481172	1.17605452	0	0
Scarf1	1.27029511	0.24299883	0.35647422	0.68167295	0.49544578	NA	Scarf1	1.34409266	0.90504744	0.63615807	0	4.99443516	0	0	2.28262753
L3mbtl1	4.66081068	-0.2821917	0.41410352	-0.681452	0.4955855	0.99938111	L3mbtl1	5.37637063	3.16766603	3.81694841	10.3407001	4.99443516	1.17605452	3.84905555	4.56525506
BC003965	27.9364892	-0.2001414	0.2937116	-0.6814216	0.49560478	0.99938111	BC003965	26.8818531	27.6039469	31.8079035	33.0902402	26.6369875	18.8168724	22.1320694	36.5220405
Ppm1a	121.494145	0.19042337	0.27952173	0.6812471	0.49571515	0.99938111	Ppm1a	130.376988	95.9350284	113.554215	111.679561	81.5757743	135.24627	196.301833	107.283494
Daxx	20.907975	-0.2320192	0.34063277	-0.6811416	0.4957819	0.99938111	Daxx	20.1613899	15.3858064	21.9474534	35.1583802	23.3073641	11.7605452	14.4339583	25.1089028
Cck	71.4167361	-0.2401093	0.35253543	-0.6810927	0.49581284	0.99938111	Cck	88.038069	121.72888	84.2909441	20.6814001	59.9332219	55.2745626	86.60375	54.7830607
Cic	154.397648	0.24384279	0.35805167	0.68102683	0.49585452	0.99938111	Cic	140.457683	142.544971	107.510714	157.178641	314.649415	90.5561984	65.4339444	216.849615
Psd2	77.6774953	-0.2567142	0.37705928	-0.6808326	0.49597742	0.99938111	Psd2	59.1400769	69.236129	48.0299342	173.723761	88.2350212	31.7534722	39.4528194	111.848749
S100pbp	17.9410743	0.23354186	0.34305887	0.68076323	0.49602132	0.99938111	S100pbp	16.8011582	17.1959013	20.0389792	10.3407001	13.3184938	29.4013631	25.0188611	11.4131377
Fuca2	20.569414	-0.2665023	0.39151472	-0.6806955	0.49606419	0.99938111	Fuca2	20.1613899	39.8220873	25.4463228	8.27256004	11.653682	30.5774176	24.0565972	4.56525506
Zfp637	12.9732467	-0.2450679	0.36012949	-0.6804993	0.49618837	0.99938111	Zfp637	17.4732045	14.480759	13.9954775	12.4088401	3.32962344	15.2887088	15.3962222	11.4131377
Ctps2	18.4473994	0.24425208	0.35900979	0.68034937	0.49628327	0.99938111	Ctps2	12.7688802	16.7433776	26.7186389	8.27256004	13.3184938	25.8731995	27.9056528	15.9783927
Nup160	10.5061666	0.25815375	0.37945165	0.68033372	0.49629318	0.99938111	Nup160	4.03227797	13.1231878	8.2700549	12.4088401	8.3240586	15.2887088	13.4716944	9.13051012
9330117O12Rik	0.56495105	-0.2157298	0.31710036	-0.6803204	0.49630163	NA	9330117O12Ri	2.01613899	0.90504744	0.63615807	0	0	0	0.96226389	0
Hpdl	0.56495105	-0.2157298	0.31710036	-0.6803204	0.49630163	NA	Hpdl	2.01613899	0.90504744	0.63615807	0	0	0	0.96226389	0
Pot1a	11.8337609	0.2694997	0.3961882	0.68023152	0.49635788	0.99938111	Pot1a	7.39250961	12.6706641	17.1762679	2.06814001	4.99443516	16.4647633	20.2075417	13.6957652
Alkbh7	8.44758858	0.28282636	0.41588927	0.68005206	0.4964715	0.99938111	Alkbh7	4.7043243	1.35757116	3.49886938	18.6132601	9.98887032	1.17605452	7.69811111	20.5436478
Otof	27.6332056	0.26645667	0.39185249	0.67999228	0.49650935	0.99938111	Otof	30.9141311	19.4585199	23.2197695	20.6814001	74.9165274	12.9365998	11.5471667	27.3915304
Fer1l5	4.92259176	-0.2827369	0.41583967	-0.679918	0.49655637	0.99938111	Fer1l5	6.72046328	9.5029981	6.99773876	0	3.32962344	7.05632714	5.77358333	0
Zranb1	22.7221769	0.2548036	0.37476897	0.67989512	0.49657087	0.99938111	Zranb1	25.5377605	26.2463757	17.8124259	10.3407001	9.98887032	43.5140174	34.6415	13.6957652
Slc9a7	28.9937582	0.21855258	0.32152119	0.67974548	0.49666563	0.99938111	Slc9a7	20.8334362	31.6766603	28.3090341	24.8176801	29.966611	51.7463991	24.0565972	20.5436478
Raver2	8.55197788	0.27725787	0.40792501	0.67967853	0.49670803	0.99938111	Raver2	10.0806949	10.4080455	7.31581779	0	14.9833055	4.7042181	16.3584861	4.56525506
Glra2	32.4512445	0.26917727	0.39604802	0.67965818	0.49672092	0.99938111	Glra2	23.5216215	42.5372296	36.579089	8.27256004	16.6481172	74.091435	44.2641389	13.6957652
D6Wsu163e	15.4998142	-0.2593164	0.38172781	-0.6793228	0.49693333	0.99938111	D6Wsu163e	20.1613899	19.9110436	25.1282437	4.13628002	8.3240586	14.1126543	23.0943333	9.13051012
Ppid	15.7258134	0.26869141	0.39562138	0.67916302	0.49703457	0.99938111	Ppid	20.8334362	11.313093	14.3135566	8.27256004	4.99443516	29.4013631	29.8301805	6.84788259
Rhbdl1	40.5835567	0.27664993	0.407401	0.6790605	0.49709953	0.99938111	Rhbdl1	32.9302701	22.6261859	19.4028211	62.0442003	59.9332219	5.88027262	25.981125	95.8703563
Col14a1	0.58718884	-0.2196329	0.32344634	-0.6790397	0.49711269	NA	Col14a1	1.34409266	0.90504744	1.27231614	0	0	1.17605452	0	0
Gm7694	1.45451681	-0.2649602	0.39040733	-0.6786764	0.49734294	NA	Gm7694	2.01613899	2.26261859	1.27231614	2.06814001	1.66481172	2.35210905	0	0
Mbd3	38.8575084	0.20579396	0.30327307	0.67857643	0.49740628	0.99938111	Mbd3	26.2098068	37.5594687	37.215247	41.3628002	44.9499164	22.345036	50.9999861	50.2178057
Rab10	163.307016	-0.1378705	0.20319076	-0.6785274	0.49743735	0.99938111	Rab10	163.979304	161.098444	188.302788	173.723761	121.531256	166.999742	166.471653	164.349182
Zfp612	42.7580442	0.19959226	0.29415663	0.67852376	0.49743967	0.99938111	Zfp612	33.6023164	34.8443264	44.2129858	47.5672202	31.6314227	57.6266717	60.622625	31.9567854
Slc35f6	12.2355293	-0.262572	0.38700053	-0.6784796	0.49746767	0.99938111	Slc35f6	10.0806949	20.3635674	13.0412404	10.3407001	6.65924688	15.2887088	3.84905555	18.2610202
Wbp2	443.089253	-0.2283591	0.33657989	-0.6784692	0.49747427	0.99938111	Wbp2	518.147719	429.897533	378.514051	620.442003	527.745315	159.943415	280.018792	630.005199
8430427H17Rik	45.7684578	-0.2306216	0.33995276	-0.678393	0.49752253	0.99938111	8430427H17Ri	57.1239379	72.8563187	52.8011197	16.5451201	39.9554813	57.6266717	32.7169722	36.5220405
Fam175b	21.540845	-0.217446	0.32055287	-0.6783466	0.49755195	0.99938111	Fam175b	22.1775288	23.9837571	25.1282437	24.8176801	14.9833055	19.9929269	29.8301805	11.4131377
Naprt1	1.31954194	-0.2335722	0.34433567	-0.6783272	0.49756426	NA	Naprt1	0	0.45252372	1.27231614	6.20442003	1.66481172	0	0.96226389	0
H2-T24	4.40699581	0.28047954	0.41348866	0.67832461	0.4975659	NA	H2-T24	1.34409266	2.26261859	5.40734359	6.20442003	4.99443516	4.7042181	5.77358333	4.56525506
Zfp940	7.03557607	0.27408839	0.40414381	0.6781952	0.49764794	0.99938111	Zfp940	8.06455594	8.14542694	6.67965972	0	11.653682	11.7605452	7.69811111	2.28262753
Rab39b	47.4819926	0.23186854	0.34201731	0.67794387	0.49780728	0.99938111	Rab39b	40.994826	48.4200379	53.7553568	26.8858201	23.3073641	90.5561984	54.8490416	41.0872956
Adamts1	3.34748312	0.28224826	0.41637075	0.67787725	0.49784953	NA	Adamts1	4.03227797	1.35757116	4.45310648	0	4.99443516	3.52816357	3.84905555	4.56525506
4930414L22Rik	24.9204642	0.2632046	0.3883664	0.67772238	0.49794774	0.99938111	4930414L22Ri	14.7850192	13.1231878	16.2220308	43.4309402	39.9554813	11.7605452	14.4339583	45.6525506
Rab33b	28.6003153	0.20844856	0.30761513	0.67762779	0.49800772	0.99938111	Rab33b	16.1291119	29.8665654	27.0367179	31.0221002	26.6369875	25.8731995	28.8679167	43.3699231
Ccdc82	7.1011289	0.27309414	0.40310772	0.67747189	0.4981066	0.99938111	Ccdc82	6.72046328	5.88280835	9.224292	2.06814001	3.32962344	10.5844907	14.4339583	4.56525506
Lancl1	117.824184	0.18082758	0.26693551	0.67742046	0.49813922	0.99938111	Lancl1	118.9522	101.365313	119.915796	95.1344405	106.54795	79.9717076	147.226375	173.479692
Zfp644	61.5137089	0.25852991	0.38168884	0.67733159	0.49819559	0.99938111	Zfp644	65.8605402	68.3310816	61.7073327	18.6132601	31.6314227	94.0843619	122.207514	29.6741579
Pxdc1	4.68468229	0.28193452	0.41637078	0.67712368	0.49832748	0.99938111	Pxdc1	5.37637063	0.90504744	1.90847421	8.27256004	8.3240586	2.35210905	5.77358333	4.56525506
Trim17	1.46433561	0.26815657	0.39602311	0.67712353	0.49832758	NA	Trim17	1.34409266	1.81009488	0.9542371	0	3.32962344	2.35210905	1.92452778	0
Casr	3.27059473	-0.2808817	0.414825	-0.6771088	0.49833693	NA	Casr	3.36023164	6.33533207	4.77118552	0	4.99443516	1.17605452	0.96226389	4.56525506
6330418K02Rik	0.56794708	0.19054141	0.2814386	0.67702656	0.49838911	NA	6330418K02Ri	0	0	0.9542371	0	1.66481172	0	1.92452778	0
C4b	8.87739766	-0.2224158	0.32869083	-0.6766717	0.4986143	0.99938111	C4b	10.0806949	14.480759	8.2700549	16.5451201	21.6425524	0	0	0
Mybpc2	0.60155825	0.21003897	0.31045738	0.67654686	0.49869351	NA	Mybpc2	0	0.90504744	0.31807903	0	1.66481172	0	1.92452778	0
Zfp958	5.23718795	0.27301154	0.40356215	0.67650434	0.49872049	0.99938111	Zfp958	3.36023164	4.07271347	8.2700549	0	0	11.7605452	14.4339583	0
Rasl11b	26.5570426	0.23687491	0.35022296	0.67635459	0.49881554	0.99938111	Rasl11b	23.5216215	18.1009488	27.672876	24.8176801	41.620293	9.40843619	30.7924444	36.5220405
Ndst2	9.71091196	0.2550913	0.37718494	0.676303	0.49884829	0.99938111	Ndst2	6.04841696	11.313093	7.63389683	8.27256004	16.6481172	10.5844907	5.77358333	11.4131377
Wdr76	2.69767178	-0.2782099	0.41144906	-0.676171	0.49893206	NA	Wdr76	6.04841696	4.07271347	0.9542371	2.06814001	1.66481172	3.52816357	0.96226389	2.28262753
Fgl2	1.14364137	0.2367632	0.35026672	0.67595117	0.49907165	NA	Fgl2	0	1.35757116	1.59039517	0	0	2.35210905	3.84905555	0
Aurkaip1	44.1344019	-0.2223266	0.32895059	-0.6758661	0.49912566	0.99938111	Aurkaip1	38.978687	31.6766603	39.1237212	86.8618804	43.2851047	25.8731995	46.1886666	41.0872956
Coq3	12.9886693	0.24138256	0.35719395	0.67577449	0.49918384	0.99938111	Coq3	8.73660227	9.05047438	10.1785291	20.6814001	9.98887032	12.9365998	16.3584861	15.9783927
Cdpf1	5.17402042	-0.2785525	0.41224284	-0.6757001	0.49923107	0.99938111	Cdpf1	4.03227797	4.97776091	5.08926455	10.3407001	8.3240586	1.17605452	2.88679167	4.56525506

GeneID	Base mean	log2(FC)	StdErr	Wald-Stats	P-value	P-adj		GeneID	Normalised expression for Chow#1	Normalised expression for Chow#2	Normalised expression for Chow#3	Normalised expression for Chow#4	Normalised expression for HFD#1	Normalised expression for HFD#2	Normalised expression for HFD#3	Normalised expression for HFD#4
Myo9a	134.43886	0.22262806	0.32949061	0.67567346	0.49924799	0.99938111		Myo9a	127.688802	142.092448	138.36438	78.5893204	103.218327	235.210905	181.867875	68.4788259
Ssc5d	0.83445471	-0.2073202	0.30692633	-0.6754723	0.49937576	NA		Ssc5d	0.67204633	0.90504744	0	4.13628002	0	0	0.96226389	0
Fam131c	5.50139986	-0.275347	0.40771278	-0.6753455	0.4994563	0.99938111		Fam131c	2.01613899	2.26261859	4.77118552	18.6132601	8.3240586	1.17605452	0	6.84788259
Sf3b3	57.7498751	0.19256373	0.28519384	0.67520298	0.49954681	0.99938111		Sf3b3	61.8282622	41.6321821	50.5745665	59.9760603	91.5646446	42.3379629	61.5848889	52.5004332
Tbx18	1.32568401	-0.2504245	0.37100986	-0.6749807	0.499688	NA		Tbx18	4.7043243	1.35757116	0.9542371	0	1.66481172	0	1.92452778	0
Stard10	16.2384637	-0.2224477	0.32965162	-0.6747962	0.49980522	0.99938111		Stard10	20.1613899	14.0282353	19.0847421	18.6132601	13.3184938	11.7605452	19.2452778	13.6957652
Nhlrc1	14.9878142	0.24518002	0.36345879	0.67457447	0.49994616	0.99938111		Nhlrc1	11.4247876	9.05047438	12.4050823	20.6814001	23.3073641	10.5844907	9.62263889	22.8262753
Kctd6	20.7292527	-0.2298694	0.34093366	-0.674235	0.5001619	0.99938111		Kctd6	27.5538995	30.3190892	19.4028211	14.4769801	14.9833055	18.8168724	28.8679167	11.4131377
Ptpn9	38.9965976	-0.1740071	0.25808933	-0.6742128	0.50017604	0.99938111		Ptpn9	45.027104	44.3473245	41.0321955	35.1583802	33.2962344	32.9295267	41.3773472	38.804668
Bysl	23.8500538	-0.2163651	0.32093503	-0.6741711	0.50020254	0.99938111		Bysl	31.5861774	19.9110436	19.7209001	33.0902402	21.6425524	18.8168724	16.3584861	29.6741579
Ctsb	211.834272	-0.2066848	0.30665834	-0.6739904	0.50031742	0.99938111		Ctsb	191.533204	321.744364	257.962097	146.837941	149.833055	309.30234	146.264111	171.197065
Cd24a	11.2748985	0.24779212	0.36773229	0.67383836	0.50041408	0.99938111		Cd24a	8.06455594	12.2181404	11.4508452	8.27256004	4.99443516	16.4647633	17.32075	11.4131377
Nosip	23.2302128	0.24951189	0.37034794	0.67372291	0.50048749	0.99938111		Nosip	28.8979921	17.1959013	22.9016905	10.3407001	36.6258578	24.697145	8.660375	36.5220405
Banp	11.9421215	-0.2487901	0.36931389	-0.6736548	0.50053083	0.99938111		Banp	17.4732045	18.1009488	11.1327662	6.20442003	6.65924688	15.2887088	11.5471667	9.13051012
5330426P16Rik	3.18272363	-0.2793002	0.41466141	-0.6735621	0.50058975	NA		5330426P16Ri	5.37637063	2.71514231	5.72542262	2.06814001	1.66481172	1.17605452	6.73584722	0
Zfp445	62.9908262	0.22776219	0.33819551	0.6734631	0.50065272	0.99938111		Zfp445	63.8444012	50.6826565	69.9773876	43.4309402	38.2906696	108.197016	95.264125	34.239413
Frs3os	1.44646025	-0.207533	0.30816683	-0.6734438	0.50066502	NA		Frs3os	0.67204633	0	0	8.27256004	1.66481172	0	0.96226389	0
Ndufab1	28.534137	0.2750883	0.40849582	0.67341767	0.50068161	0.99938111		Ndufab1	6.04841696	7.69290322	8.2700549	66.1804803	16.6481172	3.52816357	5.77358333	114.131377
Pomgnt1	26.234533	0.22101557	0.32821049	0.6733958	0.50069552	0.99938111		Pomgnt1	20.1613899	32.5817078	28.9451921	10.3407001	23.3073641	35.2816357	25.0188611	34.239413
Trmt11	2.83000868	0.27041527	0.4016245	0.67330371	0.50075409	NA		Trmt11	3.36023164	0.45252372	3.18079035	2.06814001	0	5.88027262	7.69811111	0
Pias2	59.3994093	-0.1839644	0.27336572	-0.6729609	0.50097217	0.99938111		Pias2	57.1239379	68.7836053	70.2954666	59.9760603	38.2906696	52.9224536	79.8679027	47.9351782
Trim33	66.551479	0.23597831	0.35065734	0.67295986	0.50097283	0.99938111		Trim33	69.2207718	70.5937002	76.3389683	20.6814001	53.2739751	111.72518	89.4905416	41.0872956
Mc4r	2.18110413	-0.2631984	0.39111259	-0.6729479	0.50098045	NA		Mc4r	0.67204633	5.88280835	1.90847421	2.06814001	0	2.35210905	0	4.56525506
2810049E08Rik	0.44485475	0.16209857	0.24089864	0.67289118	0.50101652	NA		2810049E08Ri	0.67204633	0	0	0	0	0	2.88679167	0
Sorbs2	109.096485	0.20087106	0.29864591	0.67260609	0.50119793	0.99938111		Sorbs2	132.393127	107.248121	103.693765	55.8397803	154.82749	122.309671	127.981097	68.4788259
Gpm6a	595.540402	-0.2563607	0.38120162	-0.6725069	0.50126104	0.99938111		Gpm6a	553.766175	1156.1981	757.028102	215.086561	334.627156	984.357637	516.735708	246.523773
Dnajc6	277.115715	0.132955	0.19770268	0.67249974	0.50126561	0.99938111		Dnajc6	264.786253	279.659658	281.818025	227.495401	281.353181	345.76003	296.377278	239.675891
Rpp30	8.15615673	-0.2661013	0.39572648	-0.6724373	0.50130531	0.99938111		Rpp30	9.4086486	12.2181404	11.1327662	4.13628002	3.32962344	4.7042181	13.4716944	6.84788259
Gnb1l	4.38894778	-0.2794146	0.415641	-0.6722499	0.50142465	NA		Gnb1l	4.03227797	2.26261859	1.90847421	14.4769801	1.66481172	2.35210905	3.84905555	4.56525506
Nhlrc3	2.71518817	0.27687164	0.41185854	0.67224937	0.50142496	NA		Nhlrc3	1.34409266	2.26261859	1.27231614	4.13628002	3.32962344	0	4.81131944	4.56525506
1110038B12Rik	3.10989034	-0.2788649	0.41505759	-0.6718705	0.50166616	NA		1110038B12Ri	2.01613899	2.71514231	2.54463228	8.27256004	1.66481172	1.17605452	1.92452778	4.56525506
Tefm	2.07247282	0.27561473	0.41022351	0.67186478	0.50166978	NA		Tefm	2.01613899	2.71514231	1.27231614	0	1.66481172	4.7042181	1.92452778	2.28262753
Tsnax	113.553659	0.16823288	0.25046246	0.67168902	0.50178169	0.99938111		Tsnax	118.9522	83.716888	101.467212	119.952121	123.196067	89.3801438	109.698083	162.066555
Col28a1	0.41620293	0.1336796	0.19904158	0.67161647	0.50182789	NA		Col28a1	0	0	0	0	3.32962344	0	0	0
Defb1	0.41620293	0.1336796	0.19904158	0.67161647	0.50182789	NA		Defb1	0	0	0	0	3.32962344	0	0	0
Gm16325	0.41620293	0.1336796	0.19904158	0.67161647	0.50182789	NA		Gm16325	0	0	0	0	3.32962344	0	0	0
Mir8097	0.41620293	0.1336796	0.19904158	0.67161647	0.50182789	NA		Mir8097	0	0	0	0	3.32962344	0	0	0
Nlrp4f	0.41620293	0.1336796	0.19904158	0.67161647	0.50182789	NA		Nlrp4f	0	0	0	0	3.32962344	0	0	0
Prr19	0.41620293	0.1336796	0.19904158	0.67161647	0.50182789	NA		Prr19	0	0	0	0	3.32962344	0	0	0
Rhod	0.41620293	0.1336796	0.19904158	0.67161647	0.50182789	NA		Rhod	0	0	0	0	3.32962344	0	0	0
Slc22a29	0.41620293	0.1336796	0.19904158	0.67161647	0.50182789	NA		Slc22a29	0	0	0	0	3.32962344	0	0	0
Slc4a9	0.41620293	0.1336796	0.19904158	0.67161647	0.50182789	NA		Slc4a9	0	0	0	0	3.32962344	0	0	0
Zfp69	0.41620293	0.1336796	0.19904158	0.67161647	0.50182789	NA		Zfp69	0	0	0	0	3.32962344	0	0	0
Lyn	1.56172891	-0.2681317	0.39923553	-0.6716129	0.50183017	NA		Lyn	2.01613899	3.16766603	2.54463228	0	1.66481172	1.17605452	1.92452778	0
Gsk3a	161.200134	-0.2130157	0.31720214	-0.6715457	0.50187293	0.99938111		Gsk3a	141.129729	163.813586	162.538387	233.699821	159.821925	70.5632714	118.358458	239.675891
Emc7	33.8673239	0.22785564	0.33931784	0.67151093	0.5018951	0.99938111		Emc7	27.5538995	41.1796584	36.579089	14.4769801	21.6425524	57.6266717	30.7924444	41.0872956
Galnt2	34.7309362	-0.2202112	0.32795489	-0.671468	0.50192243	0.99938111		Galnt2	26.8818531	55.2078937	38.8056422	31.0221002	23.3073641	50.5703445	26.9433889	25.1089028
D830015G02Rik	1.4609823	0.23710982	0.35317912	0.67135854	0.50199215	NA		D830015G02R	0	0.45252372	1.27231614	2.06814001	3.32962344	0	0	4.56525506
Mettl1	5.29989249	-0.2744032	0.40877958	-0.6712741	0.50204592	0.99938111		Mettl1	6.72046328	4.07271347	4.77118552	10.3407001	1.66481172	3.52816357	6.73584722	4.56525506
Spin2c	2.2100777	-0.2656848	0.39579577	-0.6712673	0.50205027	NA		Spin2c	2.01613899	5.88280835	2.86271131	0	4.99443516	0	1.92452778	0
Tbcd	22.7323097	0.21854363	0.32559821	0.67120648	0.502089	0.99938111		Tbcd	16.1291119	17.1959013	19.4028211	31.0221002	33.2962344	15.2887088	22.1320694	27.3915304
Trp53inp1	9.19531273	-0.2496595	0.37200569	-0.6711175	0.50214567	0.99938111		Trp53inp1	12.7688802	8.14542694	10.8146872	10.3407001	8.3240586	7.05632714	11.5471667	4.56525506
Pmm2	5.7106774	-0.270545	0.40316759	-0.6710484	0.50218968	0.99938111		Pmm2	10.0806949	5.43028463	6.67965972	4.13628002	4.99443516	8.23238167	3.84905555	2.28262753
Raly	27.0716428	0.21338188	0.31800746	0.67099644	0.5022228	0.99938111		Raly	24.1936678	21.2686148	23.8559276	28.9539601	14.9833055	29.4013631	25.981125	47.9351782
Eif3g	28.4819019	0.21611831	0.32208671	0.67099418	0.50222424	0.99938111		Eif3g	29.5700384	22.1736622	25.1282437	28.9539601	19.9777406	28.2253086	50.9999861	22.8262753
Sap30bp	21.7546966	0.22707614	0.3384823	0.67086561	0.50230615	0.99938111		Sap30bp	24.1936678	23.9837571	20.9932163	8.27256004	28.3017992	27.0492541	29.8301805	11.4131377
Soga1	82.2959363	-0.2489179	0.37113857	-0.6706873	0.50241972	0.99938111		Soga1	61.8282622	79.1916508	56.9361472	171.655621	139.844185	49.39429	35.6037639	63.9135709
Hus1	13.8612655	-0.2440073	0.36387212	-0.6705854	0.50248468	0.99938111		Hus1	11.4247876	19.9110436	15.5858727	16.5451201	8.3240586	15.2887088	19.2452778	4.56525506
Ccdc184	52.1619296	0.27662442	0.41255706	0.67051192	0.50253151	0.99938111		Ccdc184	21.5054825	14.9332827	17.1762679	122.020261	46.6147282	27.0492541	35.6037639	132.392397
Scpep1	5.20254424	-0.2760727	0.41174981	-0.6704864	0.50254774	0.99938111		Scpep1	6.72046328	9.95552182	3.81694841	4.13628002	1.66481172	8.23238167	4.81131944	2.28262753
Ostm1	27.481817	-0.2605713	0.38863119	-0.6704849	0.50254873	0.99938111		Ostm1	26.8818531	50.6826565	39.4418003	6.20442003	9.98887032	38.8097993	25.0188611	22.8262753
Pdcd5	5.00994282	-0.2766269	0.41258023	-0.6704804	0.50255163	0.99938111		Pdcd5	3.36023164	4.52523719	6.67965972	10.3407001	1.66481172	3.52816357	7.69811111	2.28262753
Trim47	1.07132989	-0.2483238	0.37039911	-0.6704224	0.50258859	NA		Trim47	2.01613899	0.90504744	0.9542371	2.06814001	1.66481172	0	0.96226389	0
Calhm2	2.32973077	0.26768208	0.39942948	0.67016103	0.50275514	NA		Calhm2	1.34409266	2.26261859	2.22655324	0	4.99443516	0	0.96226389	6.84788259
Klhl20	17.801477	0.21877548	0.32646538	0.67013378	0.50277251	0.99938111		Klhl20	12.0968339	13.5757116	15.9039517	24.8176801	19.9777406	15.2887088	20.2075417	20.5436478
Irf1	2.9673962	-0.27158	0.40528836	-0.6700907	0.50279998	NA		Irf1	4.03227797	1.81009488	1.59039517	8.27256004	3.32962344	4.7042181	0	0
Mmp16	23.0413421	0.27037721	0.40349776	0.67008356	0.50280453	0.99938111		Mmp16	14.1129729	35.7493738	21.9474534	6.20442003	11.653682	57.6266717	27.9056528	9.13051012
Maneal	40.2849922	-0.2579043	0.38494955	-0.6699692	0.50287745	0.99938111		Maneal	40.3227797	73.3088425	51.2107246	16.5451201	23.3073641	75.2674895	24.0565972	18.2610202
2310033P09Rik	6.9438327	-0.278633	0.41591943	-0.6699207	0.50290836	0.99938111		2310033P09Ri	7.39250961	3.62018975	2.54463228	20.6814001	6.65924688	5.88027262	1.92452778	6.84788259
Gtf2h5	27.2123762	-0.218846	0.32671893	-0.6698296	0.50296644	0.99938111		Gtf2h5	37.6345944	29.8665654	28.3090341	22.7495401	16.6481172	15.2887088	37.5282917	29.6741579
Riiad1	1.38006367	-0.2542721	0.37966045	-0.6697355	0.50302644	NA		Riiad1	0.67204633	0.90504744	2.22655324	4.13628002	0	1.17605452	1.92452778	0
Nacad	30.7824081	0.24442927	0.36496754	0.6697288	0.50303069	0.99938111		Nacad	20.8334362	16.2908539	17.1762679	57.9079203	43.2851047	31.7534722	20.2075417	38.804668
Gnptab	52.9720598	-0.2626098	0.39224666	-0.6695018	0.50317545	0.99938111		Gnptab	45.6991503	106.343074	73.476257	14.4769801	38.2906696	85.8519803	41.3773472	18.2610202
Zfp719	9.40642032	0.25975945	0.3880804	0.66934443	0.50327579	0.99938111		Zfp719	8.73660227	8.59795066	12.4050823	2.06814001	6.65924688	16.4647633	13.4716944	6.84788259
Arhgap32	371.72572	0.18091836	0.27037239	0.66914509	0.50340293	0.99938111		Arhgap32	385.082546	408.628918	383.285237	200.609581	519.421257	426.907792	334.867833	315.002599
Itga4	8.58677319	-0.2704986	0.40428264	-0.669083	0.50344255	0.99938111		Itga4	6.72046328	19.0059962	11.1327662	2.06814001	4.99443516	10.5844907	9.62263889	4.56525506
Dync1i2	64.2433948	-0.2091787	0.31265101	-0.6690486	0.50346449	0.99938111		Dync1i2	89.3821617	67.4260341	69.9773876	53.7716403	29.966611	81.1477622	78.9056389	43.3699231
Clpb	20.624989	0.23140473	0.34592925	0.66893658	0.50353593	0.99938111		Clpb	24.1936678	14.480759	19.4028211	16.5451201	24.9721758	37.6337448	16.3584861	11.4131377
G530011O06Rik	1.11648792	0.23420521	0.35020618	0.66876378	0.50364618	NA		G530011O06R	0.67204633	1.81009488	0.31807903	0	0	0	3.84905555	2.28262753
Gpr162	104.141697	0.25073687	0.37498142	0.66866477	0.50370934	0.99938111		Gpr162	47.043243	83.716888	67.1146763	169.587481	169.810795	82.3238167	42.3396111	171.197065
Mc5r	0.37915267	-0.1522912	0.22777152	-0.6686138	0.50374186	NA		Mc5r	0	2.71514231	0.31807903	0	0	0	0	0
Slc19a3	0.37915267	-0.1522912	0.22777152	-0.6686138	0.50374186	NA		Slc19a3	0	2.71514231	0.31807903	0	0	0	0	0
Hid1	82.0803368	0.23347657	0.34922214	0.66856176	0.50377507	0.99938111		Hid1	74.5971425	53.8503226	54.3915149	111.679561	138.179373	47.042181	49.0754583	127.827142
Smg8	9.98155454	-0.2632259	0.39373548	-0.6685348	0.50379228	0.99938111		Smg8	14.7850192	13.5757116	14.6316356	2.06814001	4.99443516	11.7605452	13.4716944	4.56525506
Rnd1	12.4186713	-0.2579749	0.38598338	-0.6683574	0.50390545	0.99938111		Rnd1	11.4247876	9.95552182	9.224292	26.8858201	4.99443516	7.05632714	11.5471667	18.2610202
Tex15	1.34440409	-0.2534409	0.37921113	-0.6683371	0.50391845	NA		Tex15	1.34409266	3.16766603	2.22655324	0	1.66481172	2.35210905	0	0
Arpc5l	39.8110928	0.20453872	0.30604859	0.66832106	0.50392867	0.99938111		Arpc5l	47.043243	33.4867552	29.2632712	37.2265202	21.6425524	47.042181	54.8490416	47.9351782
Commd4	14.7336817	-0.2401496	0.35933886	-0.6683097	0.50393593	0.99938111		Commd4	17.4732045	11.7656167	21.3112953	14.4769801	14.9833055	5.88027262	18.2830139	13.6957652
Gin1	4.16422928	0.27614874	0.413244	0.66824621	0.50397644	NA		Gin1	4.7043243	1.81009488	3.81694841	4.13628002	1.66481172	5.88027262	6.73584722	4.56525506
Iqcb1	12.9550582	-0.2652209	0.39691259	-0.6682098	0.5039997	0.99938111		Iqcb1	14.7850192	13.1231878	20.0389792	12.4088401	3.32962344	9.40843619	25.981125	4.56525506
Mlf2	437.21459	-0.2347085	0.35133316	-0.668051	0.50410105	0.99938111		Mlf2	454.975364	337.130171	392.19145	748.666684	526.080504	161.11947	252.113139	625.439944
Ubp1	48.3669413	-0.1771823	0.26523304	-0.6680252	0.50411754	0.99938111		Ubp1	57.1239379	43.8948007	43.2587487	64.1123403	36.6258578	39.9858538	47.1509305	54.7830607
Flot2	103.3009	-0.2283157	0.34178055	-0.6680184	0.50412182	0.99938111		Flot2	72.5810035	89.5996963	85.2451812	208.882141	121.531256	54.0985081	71.2075278	123.261887
Gm6654	3.31721163	0.27705979	0.41477387	0.66797794	0.50414767	NA		Gm6654	2.68818531	3.62018975	2.54463228	2.06814001	4.99443516	3.52816357	4.81131944	2.28262753
Camk2b	508.76497	0.22313909	0.33406683	0.66794746	0.50416713	0.99938111		Camk2b	481.185171	336.225123	386.147948	634.918983	839.065107	294.013631	315.622555	782.941243
Pkd1l3	1.03111895	0.23545708	0.352527	0.6679122	0.50418964	NA		Pkd1l3	1.34409266	0.90504744	0.31807903	0	3.32962344	2.35210905	0	0
Slx4	20.6821036	0.20870014	0.31249344	0.66785446	0.5042265	0.99938111		Slx4	21.5054825	13.5757116	23.2197695	16.5451201	23.3073641	22.345036	22.1320694	22.8262753
Plcb3	5.1157627	-0.2779879	0.41635464	-0.667671	0.5043436	0.99938111		Plcb3	8.73660227	4.07271347	5.08926455	6.20442003	11.653682	0	2.88679167	2.28262753
Anks1b	313.427934	-0.1802428	0.26999308	-0.667583	0.50439979	0.99938111		Anks1b	401.883704	368.354307	380.104446	190.268881	236.403264	291.661522	376.24518	262.502166
Klf4	1.37352923	-0.2478915	0.37133142	-0.6675749	0.50440498	NA		Klf4	0.67204633	4.07271347	2.22655324	0	1.66481172	2.35210905	0	0
Csnk2a2	24.5294076	-0.2126288	0.31853035	-0.6675307	0.50443319	0.99938111		Csnk2a2	26.2098068	28.961518	26.7186389	26.8858201	13.3184938	23.5210905	34.6415	15.9783927
Mphosph9	12.1012523	0.25653526	0.38430736	0.66752627	0.50443603	0.99938111		Mphosph9	14.1129729	10.4080455	13.3593194	4.13628002	14.9833055	21.1689814	16.3584861	2.28262753
2810006K23Rik	8.73252639	-0.2595418	0.38890116	-0.6673722	0.50453444	0.99938111		2810006K23Ri	9.4086486	5.88280835	9.86045007	16.5451201	4.99443516	7.05632714	11.5471667	4.56525506
Mtcl1	61.6164368	0.22587937	0.33853725	0.66722159	0.5046306	0.99938111		Mtcl1	51.7475673	33.0342315	45.803381	93.0663005	88.2350212	47.042181	40.4150833	93.5877288
Ctnnd2	276.80591	0.14905608	0.22341724	0.66716461	0.50466699	0.99938111		Ctnnd2	301.748801	255.223377	237.923118	248.176801	374.582637	272.84465	220.35843	303.589462
Zfp455	1.70647796	0.26259694	0.39377257	0.66687465	0.5048522	NA		Zfp455	2.01613899	1.81009488	1.27231614	0	0	4.7042181	3.84905555	0
Uxs1	2.64788062	-0.2651512	0.39763495	-0.6668205	0.50488677	NA		Uxs1	2.68818531	8.14542694	2.54463228	0	0	5.88027262	1.92452778	0
Aloxe3	7.7422675	0.25655907	0.38483585	0.66667145	0.50498202	0.99938111		Aloxe3	3.36023164	6.78785578	7.31581779	10.3407001	8.3240586	7.05632714	9.62263889	9.13051012
Hmg20a	79.0706781	0.19522692	0.29284696	0.66665167	0.50499465	0.99938111		Hmg20a	60.4841696	49.3250854	63.2977279	122.020261	91.5646446	78.7956531	71.2075278	95.8703563
Ptplad2	0.82115433	0.22967072	0.34454108	0.66659896	0.50502834	NA		Ptplad2	0.67204633	0.45252372	0.9542371	0	0	3.52816357	0.96226389	0
Rslcan18	0.82115433	0.22967072	0.34454108	0.66659896	0.50502834	NA		Rslcan18	0.67204633	0.45252372	0.9542371	0	0	3.52816357	0.96226389	0
Iqcg	3.95116117	-0.2768233	0.41528153	-0.666592	0.5050328	NA		Iqcg	6.72046328	7.2403795	3.49886938	2.06814001	0	8.23238167	3.84905555	0

GeneID	Base mean	log2(FC)	StdErr	Wald-Stats	P-value	P-adj
Prkg1	17.0753161	0.22778351	0.34171611	0.66658699	0.50503598	0.99938111
Api5	45.1294394	0.20347053	0.30527925	0.66650628	0.50508755	0.99938111
Ncdn	1265.11306	0.25234274	0.37866265	0.66640516	0.50515217	0.99938111
Fam175a	3.19818352	0.27729536	0.41614302	0.66634629	0.50518978	NA
Spsb3	26.3096126	0.25852597	0.38801152	0.66628427	0.50522942	0.99938111
Susd4	64.0952902	-0.1933694	0.29022294	-0.6662787	0.50523296	0.99938111
Tusc1	2.93900561	-0.2772237	0.41623005	-0.6660348	0.50538885	NA
Zfp791	4.54386056	0.27719807	0.41629175	0.66587452	0.50549131	NA
Srrm1	52.9143221	-0.1935555	0.29069806	-0.6658301	0.50551972	0.99938111
Glrx3	6.58485231	-0.2666269	0.40050782	-0.6657221	0.50558874	0.99938111
H2afy	39.5294821	0.20128106	0.3023912	0.66563135	0.50564677	0.99938111
Ppef1	0.59335969	-0.2100805	0.31561839	-0.6656156	0.50565683	NA
Bora	0.93366836	0.22441705	0.33719543	0.66554001	0.50570516	NA
Prdm4	19.1620962	-0.2451835	0.36848706	-0.6653788	0.50580824	0.99938111
Robo2	74.3467416	-0.2680068	0.40285397	-0.6652705	0.50587752	0.99938111
Atg101	20.6356041	0.27514576	0.41366563	0.66514049	0.50596064	0.99938111
Gprin2	2.52890749	0.24802849	0.37303263	0.66489758	0.50611601	NA
Ftsj1	14.9229524	0.22477215	0.33812951	0.66475166	0.50620935	0.99938111
Rapgefl1	175.132128	0.23587026	0.35490133	0.66460799	0.50630126	0.99938111
Mroh2a	1.4427229	0.23456869	0.35301273	0.66447658	0.50638534	NA
Pcdhb13	3.5108434	-0.2762959	0.41598432	-0.6641979	0.50656367	NA
Tfeb	2.88015977	-0.274541	0.41339743	-0.6641091	0.50662046	NA
Acad8	14.5715703	0.218906	0.32976588	0.66382248	0.50680393	0.99938111
Hvcn1	1.36674149	-0.2616294	0.39418679	-0.6637194	0.50686992	NA
Rprd2	69.2063114	0.20789764	0.31324802	0.66368381	0.5068927	0.99938111
Acan	1.70649952	0.26145725	0.3940333	0.663541	0.50698413	NA
Zpr1	17.2176556	-0.2283475	0.34417793	-0.6634577	0.50703748	0.99938111
Pnn	17.7860228	0.25012048	0.37718134	0.66313057	0.50724693	0.99938111
Zfp788	38.7681723	0.2500259	0.37709516	0.66303132	0.50731049	0.99938111
Lamp5	36.9408803	-0.2531512	0.38181597	-0.6630189	0.50731843	0.99938111
Armcx3	32.0303723	0.23849945	0.35971856	0.66301682	0.50731978	0.99938111
Crbn	60.2478103	0.23042503	0.34769063	0.66273006	0.50750345	0.99938111
Dnajc10	46.287986	-0.2575849	0.38874855	-0.6626003	0.50758655	0.99938111
Magi1	85.8971476	0.17061068	0.25761201	0.66227768	0.50779328	0.99938111
2700081O15Rik	27.7686289	0.2548334	0.38484228	0.66217621	0.5078583	0.99938111
Csnk2a1	56.1585298	-0.1685989	0.2547006	-0.6619495	0.50800359	0.99938111
Ltk	2.12389963	-0.268843	0.40616717	-0.6619023	0.50803382	NA
Ivd	28.5929126	-0.2344401	0.35421697	-0.6618545	0.50806449	0.99938111
Dock7	23.4454578	0.2163471	0.32691152	0.66179101	0.50810517	0.99938111
Disp2	362.673137	0.20729094	0.31338467	0.66145846	0.50831834	0.99938111
Gbf1	85.0124241	0.18953345	0.28657986	0.66136346	0.50837926	0.99938111
Cdc7	3.38595261	0.27524782	0.41620658	0.66132501	0.50840391	NA
Calb2	81.9453379	-0.2750265	0.41597236	-0.6611653	0.50850629	0.99938111
Acvr2b	4.40457415	0.27386042	0.41426248	0.66107947	0.50856135	NA
Zfp114	1.16564948	0.24158672	0.36544459	0.6610762	0.50856345	NA
Tecpr1	51.2416923	0.26073317	0.39440957	0.66107211	0.50856607	0.99938111
Zic3	1.21571384	-0.2420794	0.36626349	-0.6609432	0.50864876	NA
Cdh7	8.05454271	-0.2682169	0.40594183	-0.6607275	0.50878708	0.99938111
St6galnac3	3.39722617	-0.2750196	0.41633216	-0.6605775	0.50888333	NA
Cyp4f17	0.75586043	0.21169853	0.32051885	0.66048699	0.50894136	NA
Lhfpl3	34.6469691	-0.2403825	0.36415206	-0.6601158	0.50917953	0.99938111
Rasa4	0.93842797	0.22292065	0.33771629	0.6600826	0.50920082	NA
Mrps30	15.249219	-0.2263285	0.34288289	-0.6600754	0.50920546	0.99938111
Adra2c	11.9301672	0.26737619	0.40511826	0.65999539	0.50925679	0.99938111
Katnal2	1.52322423	-0.2508854	0.38013596	-0.6599886	0.50926114	NA
Mcl1	37.0480485	0.17252415	0.26147694	0.65980637	0.5093781	0.99938111
Snapc3	14.4504281	0.23815718	0.36096855	0.65977268	0.50939972	0.99938111
Cacnb4	213.978105	0.24250714	0.36760249	0.65969941	0.50944675	0.99938111
Rfxank	13.2217546	-0.2227812	0.33778852	-0.6595285	0.50955642	0.99938111
Git1	187.815385	0.16918165	0.25664608	0.65920218	0.50976595	0.99938111
Zfp266	35.2141253	-0.1874791	0.28441067	-0.6591844	0.50977734	0.99938111
Camkmt	7.55183673	0.25668818	0.38940669	0.65917763	0.50978171	0.99938111
Ptprj	85.1246845	-0.2485627	0.37711085	-0.6591238	0.50981625	0.99938111
Ankmy1	0.43884864	-0.1911563	0.29015781	-0.658801	0.51002354	NA
Meox1	0.43884864	-0.1911563	0.29015781	-0.658801	0.51002354	NA
Rnf24	39.0839215	0.2113419	0.32080796	0.65878011	0.51003698	0.99938111
Tcf25	548.053703	-0.1177104	0.17869845	-0.6587095	0.51008234	0.99938111
4933432I03Rik	0.32385527	0.16906395	0.25671561	0.65856513	0.51017506	NA
C330013E15Rik	0.32385527	0.16906395	0.25671561	0.65856513	0.51017506	NA
Hsf5	0.32385527	0.16906395	0.25671561	0.65856513	0.51017506	NA
Mis18bp1	0.32385527	0.16906395	0.25671561	0.65856513	0.51017506	NA
Napsa	0.32385527	0.16906395	0.25671561	0.65856513	0.51017506	NA
Strn4	159.961108	0.211484	0.32113797	0.65854561	0.5101876	0.99938111
Arid1a	177.295248	0.14471613	0.21977339	0.65847887	0.51023047	0.99938111
Vstm5	7.5306064	0.2609178	0.39633125	0.65833265	0.5103244	0.99938111
Siae	7.44294476	-0.2669615	0.40565654	-0.6580973	0.5104756	0.99938111
Glcci1	34.0169032	-0.2100236	0.31934708	-0.6576657	0.51075299	0.99938111
E130201H02Rik	1.03536135	0.21904521	0.33313672	0.65752345	0.5108444	NA
Arl4a	30.408718	0.23989068	0.3648955	0.65742297	0.51090899	0.99938111
Pkd2	15.6978355	-0.2336904	0.35547248	-0.657408	0.51091861	0.99938111
Gtf3a	9.77122385	-0.2457831	0.37389688	-0.6573554	0.51095242	0.99938111
2610203C20Rik	10.5425261	-0.2485431	0.37820142	-0.6571712	0.51107084	0.99938111
Zc3h11a	26.6096378	0.19642042	0.29891674	0.65710747	0.51111181	0.99938111
Zfp575	19.4586757	-0.2554619	0.38887271	-0.6569293	0.51122639	0.99938111
Lgr6	0.72224926	0.18900467	0.2877383	0.65686308	0.51126896	NA
Nop58	21.6489342	0.24012171	0.36556124	0.65685769	0.51127243	0.99938111
Dus4l	3.96668067	0.2713627	0.41316219	0.65679462	0.51131299	NA
Chrna3	0.71130593	-0.2082544	0.31738942	-0.656148	0.51172888	NA
Sys1	21.4023808	-0.2613117	0.39829922	-0.6560689	0.51177982	0.99938111
Arhgap31	15.302427	-0.2586849	0.39432384	-0.6560214	0.51181038	0.99938111
Creb5	2.79751799	0.2689643	0.41004219	0.65594299	0.5118608	NA
2810002D19Rik	2.23839079	-0.2680125	0.40863105	-0.6558789	0.51190205	NA
Smyd5	25.6980938	-0.1953764	0.29796592	-0.6557006	0.51201681	0.99938111
Sra1	12.0182463	-0.2679783	0.40877914	-0.6555576	0.51210881	0.99938111
Copg2	75.5909418	0.20503693	0.31279187	0.65550594	0.51214206	0.99938111
Lamb1	11.518049	0.24310784	0.370886	0.65547861	0.51215965	0.99938111
Rnf31	22.0141274	0.26468845	0.40384963	0.65541337	0.51220164	0.99938111
Ppia	109.252929	-0.1986575	0.30311992	-0.6553761	0.51222566	0.99938111
Eif1a	36.310522	0.21995972	0.33568739	0.65525168	0.51230573	0.99938111
Fam84b	4.9745618	-0.2684627	0.40971225	-0.6552469	0.51230882	0.99938111
Mccc1	5.23448687	0.26389143	0.40289616	0.65498621	0.51247664	0.99938111
BC051142	0.48961722	0.18140668	0.27698335	0.65493712	0.51250824	NA
Pbld2	0.48961722	0.18140668	0.27698335	0.65493712	0.51250824	NA
Znhit3	9.64638822	-0.2538483	0.38768675	-0.6547768	0.51261148	0.99938111
Rps29	5.19822023	-0.2723543	0.41596296	-0.6547561	0.51262477	0.99938111
Gm13251	1.31230468	0.24240028	0.37022123	0.65474441	0.51263233	NA
Sh2b3	11.053248	-0.2547813	0.389149	-0.6547139	0.51265197	0.99938111
Cthrc1	4.26634756	-0.2725123	0.41624462	-0.6546926	0.51266569	NA
Parvg	4.00670105	-0.2707531	0.41367318	-0.6545097	0.51278345	NA
Chmp4b	120.601408	0.17816855	0.27230848	0.65428939	0.51292538	0.99938111
Lrch3	17.2141714	0.25146899	0.38435163	0.65426805	0.51293913	0.99938111
Tox2	88.2996955	0.25584905	0.39109025	0.65419441	0.51298656	0.99938111

GeneID	Normalised expression for Chow#1	Normalised expression for Chow#2	Normalised expression for Chow#3	Normalised expression for Chow#4	Normalised expression for HFD#1	Normalised expression for HFD#2	Normalised expression for HFD#3	Normalised expression for HFD#4
Prkg1	11.4247876	15.8383302	11.1327662	24.8176801	13.3184938	17.6408179	17.32075	25.1089028
Api5	39.6507334	44.7998482	48.3480132	33.0902402	29.966611	49.39429	76.9811111	38.804668
Ncdn	1092.07528	869.298064	875.989661	1600.74037	2547.16193	518.640045	521.547028	2095.45207
Fam175a	4.03227797	1.35757116	3.81694841	0	3.32962344	2.35210905	3.84905555	6.84788259
Spsb3	12.7688802	14.0282353	15.2677937	51.7035003	46.6147282	15.2887088	18.2830139	36.5220405
Susd4	50.4034746	99.1026944	58.5265423	66.1804803	69.9220923	49.39429	46.1886666	73.044081
Tusc1	4.7043243	4.07271347	2.86271131	2.06814001	0	2.35210905	2.88679167	4.56525506
Zfp791	4.7043243	3.62018975	4.45310648	2.06814001	3.32962344	15.2887088	2.88679167	0
Srrm1	79.9735131	45.2523719	56.6180681	45.4990802	31.6314227	48.2182355	56.7735694	59.3483158
Glrx3	8.73660227	6.78785578	10.1785291	4.13628002	1.66481172	4.7042181	9.62263889	6.84788259
H2afy	30.9141311	37.106945	40.0779583	37.2265202	19.9777406	56.4506172	44.2641389	50.2178057
Ppef1	0.67204633	2.26261859	0.63615807	0	0	1.17605452	0	0
Bora	0.67204633	0.45252372	0	2.06814001	0	2.35210905	1.92452778	0
Prdm4	28.2259458	24.8888045	22.2655324	10.3407001	11.653682	31.7534722	17.32075	6.84788259
Robo2	96.102625	135.757116	88.4259716	20.6814001	28.3017992	152.887088	45.2264028	27.3915304
Atg101	9.4086486	5.88280835	7.63389683	47.5672202	38.2906696	8.23238167	11.5471667	36.5220405
Gprin2	0.67204633	0	0.9542371	6.20442003	6.65924688	1.17605452	0	4.56525506
Ftsj1	13.4409266	14.9332827	14.6316356	10.3407001	8.3240586	21.1689814	18.2830139	18.2610202
Rapgefl1	145.162007	144.80759	120.233875	215.086561	316.314227	76.4435441	106.811292	276.197931
Mroh2a	0.67204633	2.71514231	0	0	1.66481172	0	1.92452778	4.56525506
Pcdhb13	5.37637063	3.16766603	5.08926455	2.06814001	3.32962344	3.52816357	0.96226389	4.56525506
Tfeb	4.03227797	2.71514231	1.59039517	6.20442003	3.32962344	0	2.88679167	2.28262753
Acad8	13.4409266	15.3858064	11.1327662	12.4088401	16.6481172	12.9365998	16.3584861	18.2610202
Hvcn1	1.34409266	1.81009488	1.90847421	2.06814001	1.66481172	1.17605452	0.96226389	0
Rprd2	66.5325865	79.1916508	63.6158069	43.4309402	88.2350212	104.668853	76.0188472	31.9567854
Acan	2.01613899	0.90504744	1.59039517	0	3.32962344	3.52816357	0	2.28262753
Zpr1	23.5216215	16.7433776	20.0389792	14.4769801	21.6425524	7.05632714	18.2830139	15.9783927
Pnn	22.1775288	14.9332827	15.9039517	10.3407001	13.3184938	34.1055812	26.9433889	4.56525506
Zfp788	36.9625481	38.4645161	44.5310648	16.5451201	23.3073641	63.5069443	73.1320555	13.6957652
Lamp5	54.4357526	54.75537	48.0299342	8.27256004	21.6425524	56.4506172	33.6792361	18.2610202
Armcx3	38.978687	23.0787097	34.0344567	18.6132601	33.2962344	52.9224536	46.1886666	9.13051012
Crbn	59.8121232	68.7836053	67.7508343	18.6132601	43.2851047	87.0280348	93.3395972	43.3699231
Dnajc10	47.043243	74.2138899	69.6593086	18.6132601	23.3073641	74.091435	51.96225	11.4131377
Magi1	96.102625	73.3088425	78.2474425	74.4530404	59.9332219	112.901234	107.773556	84.4572186
2700081O15R	16.1291119	14.480759	14.9497146	53.7716403	49.9443516	19.9929269	16.3584861	36.5220405
Csnk2a1	59.8121232	52.0402277	63.9338859	64.1123403	59.9332219	37.6337448	61.5848889	50.2178057
Ltk	1.34409266	2.26261859	2.54463228	4.13628002	0	1.17605452	0.96226389	4.56525506
Ivd	30.2420848	18.1009488	23.5378486	55.8397803	29.966611	15.2887088	19.2452778	36.5220405
Dock7	24.8657142	23.0787097	25.4463228	10.3407001	28.3017992	36.4576902	23.0943333	15.9783927
Disp2	236.560308	420.394535	326.349089	341.243102	466.147282	544.513245	171.282972	394.894563
Gbf1	84.0057911	79.6441745	61.7073327	88.9300204	141.508996	81.1477622	58.6980972	84.4572186
Cdc7	2.01613899	2.26261859	3.49886938	4.13628002	1.66481172	3.52816357	7.69811111	2.28262753
Calb2	20.1613899	18.5534725	22.2655324	332.970542	74.9165274	35.2816357	32.7169722	118.696632
Acvr2b	2.68818531	5.43028463	2.22655324	4.13628002	9.98887032	2.35210905	3.84905555	4.56525506
Zfp114	1.34409266	1.35757116	0.63615807	0	0	1.17605452	4.81131944	0
Tecpr1	40.994826	19.0059962	23.8559276	95.1344405	101.553515	29.4013631	26.9433889	73.044081
Zic3	2.01613899	1.35757116	0.9542371	2.06814001	3.32962344	0	0	0
Cdh7	10.0806949	12.2181404	13.6773985	0	3.32962344	10.5844907	7.69811111	6.84788259
St6galnac3	2.68818531	5.88280835	4.77118552	2.06814001	4.99443516	3.52816357	0.96226389	2.28262753
Cyp4f17	0	0.90504744	0.63615807	0	3.32962344	1.17605452	0	0
Lhfpl3	28.8979921	55.2078937	48.6660923	20.6814001	11.653682	52.9224536	31.7547083	27.3915304
Rasa4	0.67204633	0.90504744	0.31807903	0	3.32962344	0	0	2.28262753
Mrps30	14.7850192	14.480759	20.6751372	18.6132601	9.98887032	14.1126543	20.2075417	9.13051012
Adra2c	6.72046328	6.78785578	8.90621297	18.6132601	26.6369875	4.7042181	4.81131944	18.2610202
Katnal2	1.34409266	4.97776091	0.9542371	0	1.66481172	0	0.96226389	2.28262753
Mcl1	35.6184554	37.106945	33.3982986	33.0902402	38.2906696	38.8097993	48.1131944	31.9567854
Snapc3	17.4732045	12.2181404	15.5858727	4.13628002	18.3129289	9.40843619	20.2075417	18.2610202
Cacnb4	227.823705	197.300341	226.154194	107.543281	96.5590798	389.274047	359.886694	107.283494
Rfxank	14.7850192	14.9332827	14.6316356	12.4088401	11.653682	11.7605452	9.62263889	15.9783927
Git1	197.581621	176.48425	158.403359	167.519341	289.677239	164.647633	140.490528	207.719105
Zfp266	37.6345944	39.8220873	38.4875632	37.2265202	21.6425524	42.3379629	39.4528194	25.1089028
Camkmt	5.37637063	5.88280835	7.63389683	8.27256004	6.65924688	15.2887088	6.73584722	4.56525506
Ptprj	73.9250961	172.864061	98.9225797	35.1583802	54.9387868	143.478652	60.622625	41.0872956
Ankmy1	0.67204633	0.45252372	0.31807903	2.06814001	0	0	0	0
Meox1	0.67204633	0.45252372	0.31807903	2.06814001	0	0	0	0
Rnf24	22.1775288	49.3250854	42.9406697	26.8858201	34.9610461	62.3308898	37.5282917	36.5220405
Tcf25	618.282622	472.887286	577.631527	618.373863	514.426822	486.886573	532.13193	563.809
4933432I03Ri	0	0.45252372	0	0	0	1.17605452	0.96226389	0
C330013E15Ri	0	0.45252372	0	0	0	1.17605452	0.96226389	0
Hsf5	0	0.45252372	0	0	0	1.17605452	0.96226389	0
Mis18bp1	0	0.45252372	0	0	0	1.17605452	0.96226389	0
Napsa	0	0.45252372	0	0	0	1.17605452	0.96226389	0
Strn4	153.226563	116.298596	109.101109	204.745861	248.056946	84.6759257	137.603736	225.980126
Arid1a	150.538378	180.10444	160.311833	179.928181	243.062511	183.464506	152.037694	168.914437
Vstm5	6.72046328	6.78785578	7.31581779	4.13628002	1.66481172	7.05632714	10.5849028	15.9783927
Siae	6.04841696	14.480759	10.8146872	2.06814001	4.99443516	11.7605452	4.81131944	4.56525506
Glcci1	40.994826	40.274611	45.803381	20.6814001	19.9777406	41.1619083	40.4150833	22.8262753
E130201H02Ri	1.34409266	0	1.27231614	0	0	4.7042181	0.96226389	0
Arl4a	37.6345944	30.3190892	27.672876	12.4088401	16.6481172	36.4576902	61.5848889	20.5436478
Pkd2	10.0806949	25.3413283	20.0389792	12.4088401	11.653682	15.2887088	12.5094306	18.2610202
Gtf3a	10.0806949	10.8605693	9.54237104	12.4088401	8.3240586	9.40843619	3.84905555	13.6957652
2610203C20Ri	12.0968339	12.2181404	10.4966081	14.4769801	3.32962344	11.7605452	15.3962222	4.56525506
Zc3h11a	20.8334362	30.7716129	27.0367179	18.6132601	29.966611	35.2816357	29.8301805	20.5436478
Zfp575	20.8334362	22.1736622	21.6293743	20.6814001	16.6481172	5.88027262	6.73584722	41.0872956
Lgr6	0	0	1.27231614	0	3.32962344	1.17605452	0	0
Nop58	21.5054825	23.5312334	23.2197695	8.27256004	11.653682	37.6337448	33.6792361	13.6957652
Dus4l	0.67204633	7.69290322	3.49886938	0	3.32962344	2.35210905	9.62263889	4.56525506
Chrna3	0	1.81009488	0.63615807	2.06814001	0	1.17605452	0	0
Sys1	16.1291119	19.4585199	14.3135566	47.5672202	19.9777406	8.23238167	6.73584722	38.804668
Arhgap31	11.4247876	30.7716129	16.5401098	10.3407001	24.9721758	16.4647633	9.62263889	2.28262753
Creb5	0.67204633	3.62018975	1.90847421	2.06814001	6.65924688	0	2.88679167	4.56525506
2810002D19Ri	5.37637063	1.81009488	3.81694841	0	1.66481172	2.35210905	2.88679167	0
Smyd5	35.6184554	25.3413283	23.2197695	26.8858201	19.9777406	21.1689814	25.981125	27.3915304
Sra1	14.7850192	10.4080455	12.0870033	20.6814001	3.32962344	11.7605452	23.0943333	0
Copg2	65.1884939	84.1694117	79.5197586	47.5672202	61.5980337	134.070216	84.6792222	47.9351782
Lamb1	10.0806949	12.6706641	10.4966081	8.27256004	14.9833055	14.1126543	19.2452778	2.28262753
Rnf31	10.7527413	13.5757116	10.4966081	41.3628002	24.9721758	7.05632714	15.3962222	52.5004332
Ppia	131.049034	92.7673624	144.407882	105.475141	64.9276571	77.6195986	166.471653	91.3051012
Eif1a	32.9302701	34.8443264	41.3502745	22.7495401	23.3073641	47.042181	65.4339444	22.8262753
Fam84b	6.04841696	6.33533207	3.18079035	8.27256004	1.66481172	5.88027262	3.84905555	4.56525506
Mccc1	4.7043243	4.07271347	5.72542262	2.06814001	6.65924688	4.7042181	4.81131944	9.13051012
BC051142	0.67204633	0	0	0	0	0	0.96226389	2.28262753
Pbld2	0.67204633	0	0	0	0	0	0.96226389	2.28262753
Znhit3	8.73660227	7.69290322	14.3135566	14.4769801	3.32962344	10.5844907	13.4716944	4.56525506
Rps29	2.68818531	3.16766603	5.40734359	14.4769801	8.3240586	2.35210905	2.88679167	2.28262753
Gm13251	1.34409266	1.35757116	0.9542371	0	0	5.88027262	0.96226389	0
Sh2b3	12.7688802	12.2181404	8.2700549	16.5451201	19.9777406	4.7042181	4.81131944	9.13051012
Cthrc1	2.68818531	9.05047438	3.81694841	4.13628002	0	4.7042181	2.88679167	6.84788259
Parvg	6.04841696	4.97776091	3.81694841	4.13628002	6.65924688	3.52816357	2.88679167	0
Chmp4b	110.215598	77.8340797	98.2864217	167.519341	113.207197	116.429398	162.622597	118.696632
Lrch3	8.73660227	14.480759	13.3593194	26.8858201	9.98887032	24.697145	32.7169722	6.84788259
Tox2	49.7314283	41.6321821	43.5768277	173.723761	119.866444	58.8027262	43.301875	175.76232

GeneID	Base mean	log2(FC)	StdErr	Wald-Stats	P-value	P-adj	GeneID	Normalised expression for Chow#1	Normalised expression for Chow#2	Normalised expression for Chow#3	Normalised expression for Chow#4	Normalised expression for HFD#1	Normalised expression for HFD#2	Normalised expression for HFD#3	Normalised expression for HFD#4
9530077C05Rik	3.53273052	0.27222448	0.41620574	0.65406229	0.51307168	NA	9530077C05Ri	4.03227797	3.16766603	3.49886938	0	1.66481172	9.40843619	1.92452778	4.56525506
Pgr	34.6285283	0.1882549	0.28787829	0.6539392	0.51315098	0.99938111	Pgr	24.1936678	30.3190892	33.3982986	43.4309402	33.2962344	38.8097993	46.1886666	27.3915304
Rab15	185.158031	-0.1901907	0.29088302	-0.653839	0.51321554	0.99938111	Rab15	200.269806	179.651916	187.66663	227.495401	213.0959	102.316744	117.396194	253.371656
Qtrt1	3.39685963	-0.2647904	0.40513964	-0.6535781	0.51338363	NA	Qtrt1	0	3.62018975	3.18079035	10.3407001	3.32962344	1.17605452	0.96226389	4.56525506
Bag4	94.7714126	0.14057327	0.21508417	0.65357329	0.51338675	0.99938111	Bag4	88.038069	85.0744592	98.2864217	86.8618804	88.2350212	88.2040893	111.622611	111.848749
Lor	11.0728037	0.27102913	0.41502911	0.65303645	0.51373278	0.99938111	Lor	2.68818531	5.88280835	7.95197586	20.6814001	11.653682	2.35210905	7.69811111	29.6741579
Aldh7a1	32.0671287	-0.1984334	0.30387439	-0.6530112	0.51374909	0.99938111	Aldh7a1	43.6830113	29.4140417	30.2175083	35.1583802	44.9499164	25.8731995	22.1320694	25.1089028
Tmem120b	1.5160934	0.24266414	0.37162836	0.65297531	0.5137722	NA	Tmem120b	0.67204633	1.81009488	1.59039517	0	0	0	5.77358333	2.28262753
Slc7a14	135.870417	0.21761766	0.33327564	0.65296599	0.51377821	0.99938111	Slc7a14	78.6294204	181.462011	139.954775	90.9981605	153.162678	242.267232	81.7924305	118.696632
Prkar2a	72.5372829	0.1492532	0.22858369	0.65294771	0.51378999	0.99938111	Prkar2a	78.6294204	65.1634155	62.9796488	66.1804803	74.9165274	83.4998712	64.4716805	84.4572186
Rpe	19.3070996	0.21937879	0.33605522	0.65280579	0.51388149	0.99938111	Rpe	20.8334362	17.1959013	19.7209001	12.4088401	11.653682	25.8731995	30.7924444	15.9783927
Gdap1l1	72.3389448	0.20581313	0.31532815	0.65269509	0.51395287	0.99938111	Gdap1l1	73.2530498	41.1796584	52.1649617	99.2707205	88.2350212	50.5703445	64.4716805	109.566121
Kifc2	205.493634	0.17857515	0.27359836	0.65269087	0.51395559	0.99938111	Kifc2	200.941852	221.736622	167.627651	171.655621	339.621591	189.344778	145.301847	207.719105
Nav1	125.058386	0.1571596	0.24080209	0.6526505	0.51398162	0.99938111	Nav1	116.264015	119.918786	114.190373	119.952121	184.794101	136.422325	103.9245	105.000866
Abcc3	0.72333936	-0.1942553	0.29770929	-0.6525	0.51407869	NA	Abcc3	0	3.16766603	0.9542371	0	1.66481172	0	0	0
Sidt2	36.8104773	-0.2266454	0.34735458	-0.6524901	0.51408504	0.99938111	Sidt2	32.9302701	54.3028463	47.0756971	24.8176801	56.6035985	25.8731995	16.3584861	36.5220405
D3Ertd751e	8.91853594	-0.2669104	0.40912383	-0.6523952	0.51414629	0.99938111	D3Ertd751e	14.7850192	10.8605693	11.7689243	4.13628002	0	11.7605452	13.4716944	4.56525506
Leprel1	0.55431631	-0.2023964	0.31042365	-0.6520005	0.51440082	NA	Leprel1	1.34409266	1.81009488	0.31807903	0	0	0	0.96226389	0
Synj1	433.371812	0.15940208	0.2445651	0.65177768	0.51454461	0.99938111	Synj1	467.744245	414.511727	452.944545	291.607741	464.48247	569.21039	486.905528	319.567854
Unc119	33.0942045	-0.2252969	0.34567921	-0.6517513	0.51456165	0.99938111	Unc119	28.8979921	23.0787097	28.6271131	68.2486203	26.6369875	18.8168724	38.4905555	31.9567854
Rbmx	18.3808537	0.22872272	0.3510329	0.65157061	0.51467821	0.99938111	Rbmx	24.1936678	14.9332827	18.448584	8.27256004	16.6481172	22.345036	30.7924444	11.4131377
Epha10	16.6300353	0.25576858	0.39269666	0.65131336	0.51484423	0.99938111	Epha10	12.0968339	23.5312334	10.1785291	10.3407001	23.3073641	22.345036	3.84905555	27.3915304
Chl1	138.704454	0.26624569	0.40892763	0.65108268	0.51499312	0.99938111	Chl1	111.559691	177.841822	154.586411	24.8176801	83.240586	375.161393	148.188639	34.239413
Yipf1	14.4252568	0.21891117	0.33626715	0.65100373	0.51504408	0.99938111	Yipf1	9.4086486	13.1231878	13.6773985	16.5451201	19.9777406	16.4647633	12.5094306	13.6957652
Ttc25	0.90573304	0.20057801	0.30820524	0.65079365	0.5151797	NA	Ttc25	0.67204633	0	0	2.06814001	3.32962344	1.17605452	0	0
Txnrd1	46.090688	0.19006204	0.29205832	0.65076742	0.51519664	0.99938111	Txnrd1	41.6668724	30.3190892	33.7163777	68.2486203	51.6091633	43.5140174	47.1509305	52.5004332
Smarcd2	4.27098005	-0.2700928	0.4150678	-0.6507197	0.51522746	NA	Smarcd2	4.03227797	3.16766603	5.40734359	8.27256004	6.65924688	4.7042181	1.92452778	0
Cryz	9.02660891	0.24912902	0.38293665	0.65057501	0.51532087	0.99938111	Cryz	13.4409266	7.69290322	6.99773876	2.06814001	8.3240586	9.40843619	10.5849028	13.6957652
Serf1	5.94105561	-0.2705946	0.41594673	-0.6505511	0.51533627	0.99938111	Serf1	10.0806949	7.69290322	9.54237104	0	0	4.7042181	8.660375	6.84788259
Dact2	25.6396545	0.26413455	0.40602622	0.6505357	0.51534625	0.99938111	Dact2	22.8495752	13.5757116	15.5858727	35.1583802	49.9443516	5.88027262	9.62263889	52.5004332
Snhg1	1.61831652	-0.2508107	0.38556025	-0.6505096	0.51536308	NA	Snhg1	1.34409266	5.88280835	0.9542371	0	1.66481172	1.17605452	1.92452778	0
Golga7b	73.8675294	0.21839481	0.33579301	0.65038522	0.51544342	0.99938111	Golga7b	67.8766792	52.9452751	48.0299342	99.2707205	123.196067	49.39429	45.2264028	105.000866
Med7	10.6402738	0.25654604	0.39446245	0.65036874	0.51545406	0.99938111	Med7	10.0806949	13.1231878	9.54237104	4.13628002	3.32962344	18.8168724	19.2452778	6.84788259
Elk1	53.8118328	-0.2039121	0.31355398	-0.6503255	0.51548199	0.99938111	Elk1	72.5810035	51.1351802	51.5288036	55.8397803	59.9332219	24.697145	39.4528194	75.3267085
Gak	82.5116196	0.1697997	0.2611852	0.65011228	0.5156197	0.99938111	Gak	72.5810035	75.5714611	63.9338859	97.2025805	119.866444	69.3872169	70.2452639	91.3051012
Orc3	52.7781532	0.21912027	0.33710672	0.65000268	0.51569049	0.99938111	Orc3	53.7637063	54.3028463	61.3892537	20.6814001	43.2851047	74.091435	82.7546944	31.9567854
Tmem230	8.66793845	-0.2530267	0.38931241	-0.6499323	0.51573594	0.99938111	Tmem230	9.4086486	14.0282353	7.95197586	8.27256004	1.66481172	10.5844907	10.5849028	6.84788259
Cep68	24.395736	-0.203925	0.31381348	-0.6498287	0.51580286	0.99938111	Cep68	34.2743627	28.5089943	21.3112953	20.6814001	29.966611	23.5210905	16.3584861	20.5436478
Ccs	4.23827382	0.27016228	0.41580966	0.64972584	0.51586933	NA	Ccs	6.72046328	0.90504744	3.81694841	2.06814001	4.99443516	4.7042181	3.84905555	6.84788259
Wrap53	5.02724409	-0.2658839	0.40925729	-0.6496742	0.5159027	0.99938111	Wrap53	3.36023164	5.88280835	5.40734359	8.27256004	4.99443516	3.52816357	1.92452778	6.84788259
Stat2	8.54859412	0.24651595	0.37945059	0.64966547	0.51590833	0.99938111	Stat2	10.0806949	7.2403795	9.54237104	2.06814001	11.653682	9.40843619	11.5471667	6.84788259
Zfp74	11.0848108	0.26920731	0.41443555	0.64957582	0.51596625	0.99938111	Zfp74	11.4247876	7.69290322	15.5858727	2.06814001	3.32962344	27.0492541	19.2452778	2.28262753
Gm13308	0.92145729	-0.2353798	0.36239454	-0.6495126	0.51600714	NA	Gm13308	1.34409266	1.81009488	1.59039517	0	1.66481172	0	0.96226389	0
Gm20878	0.92145729	-0.2353798	0.36239454	-0.6495126	0.51600714	NA	Gm20878	1.34409266	1.81009488	1.59039517	0	1.66481172	0	0.96226389	0
Gm21586	0.92145729	-0.2353798	0.36239454	-0.6495126	0.51600714	NA	Gm21586	1.34409266	1.81009488	1.59039517	0	1.66481172	0	0.96226389	0
Prob1	4.53682442	0.27039944	0.41633077	0.64948223	0.51602673	NA	Prob1	3.36023164	3.16766603	0.9542371	8.27256004	4.99443516	3.52816357	2.88679167	9.13051012
Gm10677	3.82839821	0.27035489	0.41630137	0.64942108	0.51606624	NA	Gm10677	2.68818531	4.07271347	2.22655324	4.13628002	4.99443516	10.5844907	1.92452778	0
5830417I10Rik	35.8131986	0.18132735	0.2792235	0.64939859	0.51608078	0.99938111	5830417I10Ri	25.5377605	35.7493738	36.897168	33.0902402	31.6314227	36.4576902	34.6415	52.5004332
Calm1	1858.52299	0.18289032	0.28164466	0.64936547	0.51610218	0.99938111	Calm1	2004.7142	1796.06664	2027.75384	1063.02397	1621.52662	2163.94032	2807.88603	1383.27228
Usf2	46.1006585	0.25289741	0.38945885	0.64935593	0.51610834	0.99938111	Usf2	28.8979921	25.3413283	24.8101647	82.7256004	58.2684102	22.345036	25.981125	100.435611
Sfxn3	137.478264	0.22971265	0.35380011	0.6492724	0.51616232	0.99938111	Sfxn3	104.839227	80.0966983	93.8333152	215.086561	176.470042	77.6195986	96.2263889	255.654283
Slc38a11	0.7484869	-0.2051362	0.31600094	-0.6491632	0.5162329	NA	Slc38a11	0.67204633	2.71514231	0.31807903	0	0	0	0	2.28262753
Itpripl1	1.04910102	-0.2317827	0.35715174	-0.6489754	0.51635427	NA	Itpripl1	1.34409266	3.16766603	0.63615807	0	0	0	0.96226389	2.28262753
Aste1	1.83674375	0.25880188	0.39880934	0.64893636	0.51637951	NA	Aste1	0.67204633	1.81009488	1.59039517	2.06814001	0	4.7042181	3.84905555	0
Zbtb6	15.6093332	-0.2451241	0.37778824	-0.64884	0.51644181	0.99938111	Zbtb6	24.1936678	16.2908539	16.5401098	14.4769801	8.3240586	23.5210905	19.2452778	2.28262753
Kif3b	62.760766	0.15575138	0.24007655	0.64875716	0.51649535	0.99938111	Kif3b	58.4680306	56.1129411	58.5265423	66.1804803	69.9220923	74.091435	73.1320555	45.6525506
Pepd	10.8111569	0.25339279	0.39073116	0.6485093	0.5166556	0.99938111	Pepd	8.73660227	5.88280835	6.04350166	18.6132601	14.9833055	4.7042181	11.5471667	15.9783927
Ptpra	88.6074867	-0.2534668	0.39092476	-0.6483775	0.51674083	0.99938111	Ptpra	90.7262543	150.237875	104.329923	55.8397803	44.9499164	175.232124	69.283	18.2610202
C2cd2	5.12000095	-0.2699406	0.41633908	-0.6483673	0.51674743	0.99938111	C2cd2	2.01613899	11.7656167	5.08926455	6.20442003	1.66481172	9.40843619	4.81131944	0
Ophn1	15.4415491	0.259923	0.40101556	0.6481619	0.51688024	0.99938111	Ophn1	15.4570656	17.1959013	17.8124259	2.06814001	16.6481172	27.0492541	25.0188611	2.28262753
Timm10b	15.2829945	-0.2223471	0.34305882	-0.6481313	0.51690006	0.99938111	Timm10b	14.7850192	12.6706641	20.0389792	20.6814001	14.9833055	9.40843619	18.2830139	11.4131377
Yipf2	30.0751217	-0.2167716	0.33447427	-0.6480964	0.51692258	0.99938111	Yipf2	35.6184554	33.0342315	22.5836114	39.2946602	31.6314227	12.9365998	22.1320694	43.3699231
Hsp90ab1	611.226064	0.13977504	0.21578717	0.64774491	0.51714995	0.99938111	Hsp90ab1	704.976598	547.101176	593.217399	471.535922	610.985901	793.836804	640.86775	527.28696
Zzef1	29.7542659	0.215721	0.33308923	0.64763728	0.51721958	0.99938111	Zzef1	20.1613899	37.5594687	30.2175083	18.6132601	41.620293	45.8661264	21.1698055	22.8262753
Csnk1g2	63.530572	0.20355901	0.31431692	0.64762344	0.51722853	0.99938111	Csnk1g2	63.8444012	52.4927514	38.4875632	78.5893204	104.883138	49.39429	45.2264028	75.3267085
Twist2	1.45836023	0.21343928	0.32960383	0.64756313	0.51726755	NA	Twist2	1.34409266	1.81009488	0	0	1.66481172	0	0	6.84788259
Gm7030	0.45596281	0.15342495	0.23693629	0.64753675	0.51728462	NA	Gm7030	0	0	0.31807903	0	3.32962344	0	0	0
Hspb7	0.45596281	0.15342495	0.23693629	0.64753675	0.51728462	NA	Hspb7	0	0	0.31807903	0	3.32962344	0	0	0
1700025F24Rik	0.56790392	0.21110574	0.32613974	0.64728617	0.51744675	NA	1700025F24Ri	0.67204633	0.45252372	0.31807903	0	0	1.17605452	1.92452778	0
Fert2	9.14632249	0.2665108	0.4117698	0.64723251	0.51748147	0.99938111	Fert2	10.0806949	6.78785578	10.1785291	4.13628002	4.99443516	23.5210905	13.4716944	0
Mettl22	10.2683308	-0.2433901	0.37606684	-0.6471989	0.51750321	0.99938111	Mettl22	10.0806949	10.8605693	14.6316356	10.3407001	3.32962344	7.05632714	14.4339583	11.4131377
Efr3b	145.075073	0.18035062	0.27868839	0.6471408	0.51754082	0.99938111	Efr3b	114.247876	110.415787	114.826531	202.677721	204.771842	161.11947	108.735819	143.805534
Fzd6	2.2474584	-0.2561768	0.39586037	-0.6471394	0.51754174	NA	Fzd6	2.01613899	7.2403795	1.59039517	0	4.99443516	1.17605452	0.96226389	0
Sgcb	17.9276474	-0.2596467	0.40134151	-0.6469471	0.51766617	0.99938111	Sgcb	18.8172972	30.7716129	27.672876	4.13628002	4.99443516	30.5774176	17.32075	9.13051012
Mmaa	5.72199433	-0.2626694	0.40606474	-0.6468659	0.51771871	0.99938111	Mmaa	9.4086486	7.2403795	6.99773876	2.06814001	6.65924688	8.23238167	2.88679167	2.28262753
Lyrm5	12.915348	0.24391418	0.37712403	0.64677443	0.51777794	0.99938111	Lyrm5	9.4086486	12.6706641	11.1327662	14.4769801	11.653682	17.6408179	24.0565972	2.28262753
Snupn	5.32719816	-0.2630783	0.40679717	-0.6467063	0.51782205	0.99938111	Snupn	4.7043243	5.88280835	4.13502745	10.3407001	3.32962344	3.52816357	3.84905555	6.84788259
Mast1	127.062944	0.23516419	0.3636893	0.64660738	0.51788608	0.99938111	Mast1	93.4144396	70.1411764	105.602239	184.064461	221.419959	72.9153805	63.5094166	205.436478
Tmem115	20.0647374	0.247278	0.38245433	0.64655561	0.51791959	0.99938111	Tmem115	8.06455594	19.9110436	14.3135566	28.9539601	43.2851047	12.9365998	12.5094306	20.5436478
Fbxo2	35.4105378	-0.2535879	0.39227713	-0.6464509	0.51798742	0.99938111	Fbxo2	48.3873356	22.6261859	28.9451921	59.9760603	51.6091633	5.88027262	20.2075417	45.6525506
9430041J12Rik	2.48785794	-0.2608416	0.40359434	-0.6462965	0.51808739	NA	9430041J12Ri	1.34409266	3.62018975	3.18079035	4.13628002	6.65924688	0	0.96226389	0
Ptgs2	4.28231488	-0.2684945	0.41567613	-0.6459224	0.51832958	NA	Ptgs2	2.01613899	10.4080455	6.04350166	2.06814001	0	4.7042181	6.73584722	2.28262753
Atp9b	21.4710902	0.20280116	0.3140606	0.64573893	0.51844844	0.99938111	Atp9b	18.1452509	24.4362808	22.5836114	12.4088401	24.9721758	28.2253086	25.0188611	15.9783927
Tbrg4	22.1878743	0.24757857	0.3834054	0.64573575	0.51845051	0.99938111	Tbrg4	20.1613899	17.648425	18.448584	20.6814001	29.966611	3.52816357	25.981125	41.0872956
Agpat2	0.63016593	-0.1571461	0.24336632	-0.6457185	0.51846169	NA	Agpat2	0	0.90504744	0	4.13628002	0	0	0	0
Usp38	26.6875145	0.1806315	0.27975818	0.64567012	0.51849301	0.99938111	Usp38	24.8657142	23.5312334	24.4920857	26.8858201	26.6369875	24.697145	32.7169722	29.6741579
Lin7a	79.9230683	0.24559163	0.38048458	0.64547065	0.51862223	0.99938111	Lin7a	79.3014668	86.4320303	92.24292	22.7495401	41.620293	125.837834	150.113167	41.0872956
Zfp687	26.7246691	0.2466491	0.38213766	0.64544568	0.51863841	0.99938111	Zfp687	27.5538995	14.480759	16.8581888	35.1583802	51.6091633	16.4647633	10.5849028	41.0872956
Gm12191	1.29154303	0.23391001	0.36244697	0.6453634	0.51869171	NA	Gm12191	2.68818531	0	0.9542371	0	1.66481172	1.17605452	3.84905555	0
Arhgap11a	0.81683322	-0.2194899	0.34011249	-0.645345	0.51870363	NA	Arhgap11a	1.34409266	1.35757116	1.90847421	0	0	0	1.92452778	0
Mitd1	3.03850548	0.26816782	0.41559355	0.64526464	0.51875571	NA	Mitd1	2.68818531	2.71514231	4.13502745	0	3.32962344	4.7042181	6.73584722	0
Mrpl49	26.6359269	-0.2280813	0.35358517	-0.6450532	0.5188927	0.99938111	Mrpl49	37.6345944	29.4140417	34.6706148	16.5451201	14.9833055	27.0492541	41.3773472	11.4131377
Ino80b	11.5347384	0.26773623	0.4150884	0.64501013	0.51892062	0.99938111	Ino80b	5.37637063	4.97776091	2.86271131	26.8858201	19.9777406	5.88027262	5.77358333	20.5436478
Cstf3	17.310588	0.25493778	0.39533133	0.64487119	0.51901067	0.99938111	Cstf3	12.7688802	8.59795066	20.0389792	20.6814001	8.3240586	31.7534722	31.7547083	4.56525506
Slc25a37	11.2542527	0.23829803	0.36958227	0.64477666	0.51907193	0.99938111	Slc25a37	6.72046328	9.5029981	10.8146872	12.4088401	11.653682	5.88027262	12.5094306	20.5436478
Cd59a	2.26568229	-0.2623442	0.40695156	-0.6446572	0.51914937	NA	Cd59a	0.67204633	3.62018975	2.86271131	4.13628002	1.66481172	0	2.88679167	2.28262753
Rxrb	58.2348591	0.25025102	0.38820614	0.64463437	0.51916415	0.99938111	Rxrb	39.6507334	33.9392789	37.215247	93.0663005	86.5702095	22.345036	29.8301805	123.261887
Rpl11	2.55741676	0.26639327	0.41350294	0.64423549	0.51942274	NA	Rpl11	3.36023164	0.90504744	1.90847421	2.06814001	1.66481172	1.17605452	4.81131944	4.56525506
Zfp182	3.41368053	0.26665777	0.41392151	0.64422303	0.51943082	NA	Zfp182	2.01613899	4.52523719	4.13502745	0	1.66481172	8.23238167	6.73584722	0
Cideb	2.16975753	-0.2420046	0.37568628	-0.6441668	0.51946728	NA	Cideb	4.03227797	3.16766603	3.49886938	0	6.65924688	0	0	0
Lzts1	29.3427586	-0.2074006	0.32204235	-0.6440166	0.51956466	0.99938111	Lzts1	40.3227797	37.5594687	25.4463228	22.7495401	43.2851047	22.345036	20.2075417	22.8262753
Mreg	5.77118765	0.26749592	0.41541705	0.64392138	0.51962642	0.99938111	Mreg	1.34409266	2.26261859	7.63389683	8.27256004	1.66481172	5.88027262	7.69811111	11.4131377
6330408A02Rik	9.83685195	0.26346988	0.40936467	0.6436068	0.51983044	0.99938111	6330408A02Ri	4.7043243	7.69290322	4.45310648	18.6132601	23.3073641	7.05632714	10.5849028	2.28262753
Map3k3	5.03950418	-0.2606854	0.40515561	-0.6434205	0.51995128	0.99938111	Map3k3	7.39250961	5.43028463	4.77118552	6.20442003	3.32962344	7.05632714	3.84905555	2.28262753
Nup54	6.08234274	0.25853388	0.40253573	0.6422632	0.52070231	0.99938111	Nup54	5.37637063	4.07271347	7.31581779	4.13628002	1.66481172	10.5844907	8.660375	6.84788259
Hist1h2bp	0.83292231	-0.2260463	0.35214166	-0.6419188	0.52092591	NA	Hist1h2bp	0.67204633	2.26261859	1.59039517	0	0	1.17605452	0.96226389	0
Irf5	2.02633379	-0.2606701	0.40614049	-0.6418224	0.5209885	NA	Irf5	4.7043243	3.16766603	1.90847421	0	3.32962344	1.17605452	1.92452778	0
Tln1	14.1891018	-0.2492598	0.38836739	-0.6418145	0.52099366	0.99938111	Tln1	12.0968339	14.9332827	12.7231614	24.8176801	28.3017992	7.05632714	6.73584722	6.84788259
Fam207a	22.8205078	0.21343666	0.33255783	0.64180314	0.52100101	0.99938111	Fam207a	20.8334362	18.5534725	15.2677937	28.9539601	34.9610461	14.1126543	20.2075417	29.6741579
Upf2	45.3766162	0.2078586	0.32406307	0.64141403	0.52125372	0.99938111	Upf2	51.7475673	47.0624668	35.6248519	31.0221002	21.6425524	74.091435	53.8867778	47.9351782
Rab8b	47.2573989	-0.1941185	0.30264763	-0.6414012	0.52126207	0.99938111	Rab8b	46.3711967	60.6381783	54.7095939	43.4309402	23.3073641	57.6266717	57.7358333	34.239413
Nfu1	8.35650268	-0.2496683	0.38934955	-0.6412446	0.5213638	0.99938111	Nfu1	12.0968339	9.95552182	8.90621297	6.20442003	6.65924688	2.35210905	11.5471667	9.13051012

GeneID	Base mean	log2(FC)	StdErr	Wald-Stats	P-value	P-adj		GeneID	Normalised expression for Chow#1	Normalised expression for Chow#2	Normalised expression for Chow#3	Normalised expression for Chow#4	Normalised expression for HFD#1	Normalised expression for HFD#2	Normalised expression for HFD#3	Normalised expression for HFD#4
Pqlc2	2.9053512	-0.2669448	0.41631945	-0.6412018	0.52139155	NA		Pqlc2	4.03227797	4.07271347	3.18079035	2.06814001	3.32962344	2.35210905	1.92452778	2.28262753
Gm7102	0.85103911	0.2206873	0.34418568	0.64118676	0.52140135	NA		Gm7102	0	0.45252372	1.59039517	0	1.66481172	1.17605452	1.92452778	0
Irs1	29.8205127	0.21409429	0.33395341	0.6410903	0.52146402	0.99938111		Irs1	16.8011582	19.0059962	27.672876	47.5672202	38.2906696	36.4576902	23.0943333	29.6741579
Gjc1	1.06912455	-0.1880007	0.29331274	-0.6409566	0.52155086	NA		Gjc1	0	5.88280835	0.31807903	0	0	2.35210905	0	0
Aifm2	7.07126374	0.26422107	0.41224759	0.64092811	0.52156939	0.99938111		Aifm2	4.03227797	2.26261859	4.45310648	14.4769801	9.98887032	7.05632714	2.88679167	11.4131377
Ckmt1	175.041354	0.24190714	0.37746578	0.64087172	0.52160603	0.99938111		Ckmt1	107.527413	85.0744592	110.691504	316.425422	263.040252	89.3801438	115.471667	312.719972
Fat1	58.0430301	0.22816747	0.3560718	0.6407906	0.52165874	0.99938111		Fat1	55.7798453	71.9512713	55.9819101	22.7495401	79.9109626	105.844907	35.6037639	36.5220405
Jph3	176.646266	0.21710491	0.33882329	0.64076146	0.52167767	0.99938111		Jph3	129.032895	121.276357	127.867772	262.653781	296.336486	122.309671	104.886764	248.806401
Gcfc2	4.46181847	0.26678138	0.4163631	0.64074214	0.52169023	NA		Gcfc2	9.4086486	2.71514231	2.22655324	0	6.65924688	4.7042181	7.69811111	2.28262753
Kat8	8.68419836	0.24662638	0.38511855	0.6403908	0.52191856	0.99938111		Kat8	12.7688802	7.69290322	6.99773876	2.06814001	13.3184938	8.23238167	11.5471667	6.84788259
Slc38a9	15.3338586	-0.2450804	0.38270882	-0.6403835	0.52192331	0.99938111		Slc38a9	14.7850192	28.5089943	15.2677937	10.3407001	8.3240586	27.0492541	11.5471667	6.84788259
Ushbp1	0.98179229	-0.2303257	0.3597137	-0.6403029	0.5219757	NA		Ushbp1	1.34409266	2.71514231	0.9542371	0	1.66481172	1.17605452	0	0
Eif3e	8.9629249	0.25089155	0.39189204	0.64020578	0.52203883	0.99938111		Eif3e	11.4247876	5.88280835	8.58813393	6.20442003	4.99443516	11.7605452	18.2830139	4.56525506
Papss1	39.7618	0.18950921	0.29604289	0.64014107	0.52208089	0.99938111		Papss1	35.6184554	28.0564706	33.0802196	51.7035003	53.2739751	27.0492541	41.3773472	47.9351782
Banf1	35.7609565	-0.227704	0.355713	-0.6401341	0.52208542	0.99938111		Banf1	43.6830113	31.6766603	36.897168	43.4309402	31.6314227	11.7605452	23.0943333	63.9135709
Tmem33	58.8308047	-0.2397742	0.37465148	-0.6399928	0.52217729	0.99938111		Tmem33	33.6023164	104.985503	80.7920748	43.4309402	33.2962344	98.78858	52.9245139	22.8262753
Dcaf11	47.4969517	0.16061605	0.25104088	0.63980041	0.52230237	0.99938111		Dcaf11	49.059382	46.6099431	38.8056422	45.4990802	46.6147282	49.39429	60.622625	43.3699231
Asap1	87.1376646	-0.1677997	0.26227627	-0.6397821	0.52231424	0.99938111		Asap1	113.575829	87.3370778	97.0141055	74.4530404	68.2572805	107.020962	92.3773333	57.0656883
Sun1	31.4104221	-0.2001372	0.31282925	-0.639765	0.52232538	0.99938111		Sun1	32.9302701	44.3473245	22.2655324	37.2265202	36.6258578	32.9295267	22.1320694	22.8262753
Ccdc151	1.83480237	-0.258731	0.40443138	-0.6397401	0.52234158	NA		Ccdc151	1.34409266	3.16766603	2.22655324	2.06814001	1.66481172	0	1.92452778	2.28262753
Hr	14.7645341	-0.2637613	0.41232568	-0.6396915	0.52237315	0.99938111		Hr	13.4409266	10.4080455	6.36158069	39.2946602	24.9721758	7.05632714	2.88679167	13.6957652
Nrn1l	1.5626312	-0.2474793	0.38689936	-0.6396478	0.52240163	NA		Nrn1l	1.34409266	0.90504744	1.90847421	4.13628002	0	0	1.92452778	2.28262753
Elf2	24.6984798	0.20268278	0.31687412	0.63963184	0.52241198	0.99938111		Elf2	28.2259458	17.1959013	19.0847421	26.8858201	28.3017992	16.4647633	31.7547083	29.6741579
Wnk3	78.4685111	0.20871553	0.326308	0.63962738	0.52241488	0.99938111		Wnk3	64.5164475	82.3593168	87.4717345	51.7035003	43.2851047	139.950488	99.1131805	59.3483158
Tmem74b	2.93742758	-0.2476261	0.38724558	-0.6394549	0.52252702	NA		Tmem74b	2.01613899	1.35757116	1.27231614	10.3407001	1.66481172	0	0	6.84788259
Ror1	0.69587689	-0.1909214	0.29865267	-0.6392758	0.5226435	NA		Ror1	0	1.35757116	2.54463228	0	1.66481172	0	0	0
Enc1	396.469531	0.19672922	0.30786299	0.63901548	0.52281286	0.99938111		Enc1	436.830113	418.58444	371.516312	227.495401	729.187534	310.478394	369.509333	308.154717
A730017C20Rik	50.2579189	-0.2480694	0.38831656	-0.6388328	0.52293169	0.99938111		A730017C20Ri	21.5054825	29.8665654	38.8056422	138.565381	46.6147282	31.7534722	35.6037639	59.3483158
Zfp408	8.18955101	0.24677188	0.38637986	0.63867687	0.52303316	0.99938111		Zfp408	8.73660227	5.88280835	5.40734359	8.27256004	11.653682	7.05632714	4.81131944	13.6957652
Ywhab	493.385398	0.17681116	0.27685358	0.63864504	0.52305388	0.99938111		Ywhab	539.653202	433.065199	504.473349	357.788222	291.342051	572.738553	720.735653	527.28696
Etf1	79.8368831	0.20902085	0.32734973	0.63852459	0.52313225	0.99938111		Etf1	88.7101153	70.5937002	84.2909441	47.5672202	44.9499164	104.668853	138.566	59.3483158
Pds5b	120.938619	0.21078515	0.33011608	0.63851827	0.52313636	0.99938111		Pds5b	142.473822	116.751119	124.368902	55.8397803	88.2350212	203.457433	158.773542	77.6093361
Ctnna1	21.510359	-0.1974101	0.30929931	-0.6382494	0.52331132	0.99938111		Ctnna1	26.2098068	25.3413283	24.4920857	16.5451201	21.6425524	25.8731995	18.2830139	13.6957652
Hccs	14.0444799	0.22872052	0.35839407	0.63818163	0.52335545	0.99938111		Hccs	8.73660227	11.7656167	18.766663	10.3407001	9.98887032	22.345036	14.4339583	15.9783927
Gpr107	17.900869	-0.240722	0.37723711	-0.6381185	0.52339655	0.99938111		Gpr107	9.4086486	30.7716129	23.5378486	14.4769801	13.3184938	21.1689814	7.69811111	22.8262753
Rbm34	10.521112	-0.24066	0.37731872	-0.6378163	0.5235933	0.99938111		Rbm34	11.4247876	8.59795066	12.4050823	16.5451201	3.32962344	10.5844907	14.4339583	6.84788259
2310036O22Rik	59.8180396	0.24942349	0.39106055	0.63781297	0.52359544	0.99938111		2310036O22R	32.9302701	27.1514231	36.579089	113.747701	79.9109626	21.1689814	52.9245139	114.131377
Gja4	0.3455415	-0.1467814	0.23013343	-0.6378102	0.52359726	NA		Gja4	0	1.81009488	0.9542371	0	0	0	0	0
Cyba	0.68020985	-0.2028045	0.31799564	-0.6377587	0.52363077	NA		Cyba	2.68818531	0.45252372	0.63615807	0	1.66481172	0	0	0
4933402D24Rik	0.44036872	0.15034394	0.23586448	0.63741659	0.52385352	NA		4933402D24Ri	0	0	0.63615807	0	0	0	2.88679167	0
Nuf2	0.44036872	0.15034394	0.23586448	0.63741659	0.52385352	NA		Nuf2	0	0	0.63615807	0	0	0	2.88679167	0
LOC100861615	0.29961077	-0.1632668	0.25615361	-0.6373786	0.52387825	NA		LOC10086161	0.67204633	0.45252372	1.27231614	0	0	0	0	0
Pidd1	0.29961077	-0.1632668	0.25615361	-0.6373786	0.52387825	NA		Pidd1	0.67204633	0.45252372	1.27231614	0	0	0	0	0
Rad51ap2	0.29961077	-0.1632668	0.25615361	-0.6373786	0.52387825	NA		Rad51ap2	0.67204633	0.45252372	1.27231614	0	0	0	0	0
Sec61g	0.29961077	-0.1632668	0.25615361	-0.6373786	0.52387825	NA		Sec61g	0.67204633	0.45252372	1.27231614	0	0	0	0	0
Upk1b	0.29961077	-0.1632668	0.25615361	-0.6373786	0.52387825	NA		Upk1b	0.67204633	0.45252372	1.27231614	0	0	0	0	0
Ccdc130	6.35970797	0.24966101	0.39171145	0.63735949	0.52389071	0.99938111		Ccdc130	6.72046328	5.88280835	5.08926455	4.13628002	11.653682	7.05632714	5.77358333	4.56525506
Arf6	49.9126513	0.17515047	0.27492553	0.63708335	0.52407055	0.99938111		Arf6	53.0916599	50.6826565	47.0756971	33.0902402	43.2851047	41.1619083	69.283	61.6309433
Pknox1	8.70022905	-0.2427389	0.3810207	-0.6370754	0.5240757	0.99938111		Pknox1	10.0806949	11.7656167	9.86045007	8.27256004	4.99443516	11.7605452	10.5849028	2.28262753
Rnpepl1	13.6653668	-0.2615297	0.41053297	-0.6370493	0.52409275	0.99938111		Rnpepl1	16.8011582	14.480759	12.4050823	18.6132601	28.3017992	1.17605452	3.84905555	13.6957652
C2cd4a	0.45565422	-0.1692401	0.26571411	-0.6369255	0.5241734	NA		C2cd4a	0.67204633	0.90504744	0	2.06814001	0	0	0	0
Sdad1	13.5848311	0.22752429	0.35727476	0.63683282	0.52423375	0.99938111		Sdad1	12.7688802	9.5029981	10.1785291	16.5451201	21.6425524	8.23238167	11.5471667	18.2610202
Serp1	27.6023235	-0.2024827	0.31803727	-0.6366636	0.524344	0.99938111		Serp1	30.9141311	34.8443264	34.6706148	18.6132601	21.6425524	18.8168724	38.4905555	22.8262753
Ap3m2	123.004396	0.16852659	0.26470396	0.63666062	0.52434593	0.99938111		Ap3m2	124.328571	98.6501707	92.560999	144.769801	168.145984	96.436471	103.9245	155.218672
Mfn2	222.924303	0.19237123	0.30223268	0.63650044	0.5244503	0.99938111		Mfn2	193.549343	141.1874	177.80618	310.221002	298.001298	178.760288	159.735805	324.133109
Gm13293	0.65097093	-0.2008727	0.31560406	-0.6364707	0.5244697	NA		Gm13293	0	0.90504744	1.27231614	2.06814001	0	0	0.96226389	0
Hras	62.5618557	-0.1817178	0.28551129	-0.6364645	0.52447373	0.99938111		Hras	74.5971425	45.2523719	62.0254117	86.8618804	56.6035985	42.3379629	52.9245139	79.8919636
Samd5	10.9240135	0.25444739	0.39987102	0.63632366	0.52456548	0.99938111		Samd5	6.04841696	4.52523719	6.67965972	22.7495401	14.9833055	12.9365998	5.77358333	13.6957652
Zfp39	7.95762638	-0.2431375	0.38215459	-0.636228	0.5246278	0.99938111		Zfp39	11.4247876	7.69290322	10.4966081	6.20442003	4.99443516	10.5844907	7.69811111	4.56525506
Trim16	0.77240058	-0.2042298	0.32106238	-0.6361061	0.52470724	NA		Trim16	0	1.81009488	0.63615807	2.06814001	1.66481172	0	0	0
Slc35f5	9.68698659	-0.2593734	0.40785378	-0.6359469	0.524811	0.99938111		Slc35f5	4.03227797	12.6706641	12.7231614	16.5451201	8.3240586	16.4647633	6.73584722	0
Ppp1r12b	32.025481	0.1819534	0.2861464	0.63587519	0.52485777	0.99938111		Ppp1r12b	32.2582238	31.2241366	27.990955	28.9539601	38.2906696	38.8097993	40.4150833	18.2610202
Pcdh19	78.1246138	0.20670637	0.32512638	0.63577237	0.5249248	0.99938111		Pcdh19	47.043243	110.415787	76.3389683	49.6353602	94.8942681	124.66178	55.8113055	66.1961984
Gm6682	57.3768359	0.19668565	0.30938917	0.63572247	0.52495733	0.99938111		Gm6682	50.4034746	52.0402277	40.7141164	68.2486203	91.5646446	31.7534722	55.8113055	68.4788259
Kctd17	112.426348	0.22755892	0.35796983	0.63569301	0.52497653	0.99938111		Kctd17	114.919922	82.8118406	69.9773876	134.429101	159.821925	70.5632714	50.0377222	216.849615
Adal	12.1411007	-0.2268789	0.3569369	-0.6356275	0.52501926	0.99938111		Adal	9.4086486	17.648425	14.6316356	12.4088401	9.98887032	11.7605452	14.4339583	6.84788259
Arhgef17	174.720509	0.23759914	0.37383919	0.63556508	0.52505993	0.99938111		Arhgef17	120.296293	106.343074	104.648002	289.539601	336.291968	122.309671	74.0943194	244.241146
Cd247	2.21244877	0.25369827	0.39929146	0.63537114	0.52518639	NA		Cd247	2.01613899	2.71514231	0.63615807	2.06814001	0	3.52816357	6.73584722	0
Kdm3a	14.6767891	-0.2419768	0.38084838	-0.6353627	0.52519191	0.99938111		Kdm3a	19.4893435	19.9110436	21.6293743	4.13628002	6.65924688	15.2887088	21.1698055	9.13051012
Zfp189	9.12980306	-0.2488968	0.39174502	-0.6353541	0.52519747	0.99938111		Zfp189	13.4409266	11.313093	7.63389683	10.3407001	3.32962344	14.1126543	10.5849028	2.28262753
Slc25a14	30.2483348	-0.2128124	0.33496516	-0.635327	0.52521515	0.99938111		Slc25a14	22.1775288	45.2523719	28.9451921	35.1583802	14.9833055	36.4576902	20.2075417	38.804668
Zfp952	9.42035011	0.24839074	0.39102465	0.63523038	0.52527817	0.99938111		Zfp952	6.72046328	8.14542694	9.224292	10.3407001	3.32962344	17.6408179	15.3962222	4.56525506
Mtmr1	27.6093183	0.23015621	0.36237222	0.63513757	0.52533869	0.99938111		Mtmr1	30.9141311	26.6988994	34.9886938	4.13628002	24.9721758	38.8097993	37.5282917	22.8262753
Mrpl34	17.2362912	-0.2330603	0.36724815	-0.6346125	0.52568115	0.99938111		Mrpl34	20.1613899	11.7656167	11.4508452	35.1583802	11.653682	11.7605452	15.3962222	20.5436478
Ston2	25.7967439	-0.2131529	0.33596896	-0.6344421	0.52579233	0.99938111		Ston2	26.2098068	23.5312334	22.9016905	41.3628002	38.2906696	22.345036	13.4716944	18.2610202
Mrps36	4.16967107	0.26225253	0.41338688	0.63439975	0.52581997	NA		Mrps36	3.36023164	4.97776091	4.13502745	0	6.65924688	3.52816357	3.84905555	6.84788259
Gnpda1	20.2832274	-0.2352187	0.37077979	-0.6343892	0.52582687	0.99938111		Gnpda1	16.1291119	17.1959013	16.2220308	41.3628002	19.9777406	8.23238167	13.4716944	29.6741579
Dock2	1.49253559	0.2380288	0.37528922	0.6342543	0.52591488	NA		Dock2	2.01613899	0.45252372	1.59039517	0	4.99443516	0	2.88679167	0
Gstp1	43.0624935	-0.2520821	0.3974517	-0.6342459	0.52592034	0.99938111		Gstp1	23.5216215	32.129184	33.0802196	107.543281	53.2739751	10.5844907	25.0188611	59.3483158
Rab23	28.5683014	0.18796887	0.29639803	0.63417718	0.5259652	0.99938111		Rab23	32.9302701	23.0787097	24.1740066	26.8858201	23.3073641	29.4013631	41.3773472	27.3915304
Zfp518a	9.25415511	0.26391841	0.41616278	0.63417111	0.52596916	0.99938111		Zfp518a	4.7043243	13.5757116	10.1785291	2.06814001	1.66481172	16.4647633	23.0943333	2.28262753
Ldb3	3.67578029	-0.2640092	0.41632271	-0.6341455	0.5259859	NA		Ldb3	6.04841696	3.62018975	5.72542262	2.06814001	4.99443516	1.17605452	5.77358333	0
Aff4	262.755487	0.18183204	0.28677178	0.63406533	0.52603819	0.99938111		Aff4	226.479613	263.368804	272.911812	213.018421	199.777406	431.61201	321.396139	173.479692
Lima1	3.76468891	-0.2638289	0.41614846	-0.6339778	0.52609533	NA		Lima1	5.37637063	5.88280835	2.86271131	4.13628002	1.66481172	1.17605452	6.73584722	2.28262753
Ankrd12	96.0476168	0.25416888	0.40093428	0.63394149	0.52611901	0.99938111		Ankrd12	100.806949	89.1471726	112.281899	28.9539601	36.6258578	214.041923	156.849014	29.6741579
Ccnh	20.2480678	0.26161292	0.4128386	0.63369299	0.5262812	0.99938111		Ccnh	16.8011582	20.3635674	30.2175083	0	9.98887032	34.1055812	41.3773472	9.13051012
Cmc1	7.0836553	-0.2627701	0.41467908	-0.6336711	0.52629551	0.99938111		Cmc1	10.7527413	10.4080455	10.1785291	2.06814001	1.66481172	12.9365998	8.660375	0
Nudt8	2.68499511	0.26285963	0.41500551	0.63338829	0.52648011	NA		Nudt8	3.36023164	0.90504744	2.22655324	2.06814001	3.32962344	1.17605452	3.84905555	4.56525506
Foxf1	0.4343626	-0.1706524	0.26943097	-0.6333806	0.52648511	NA		Foxf1	0	0.45252372	0.9542371	2.06814001	0	0	0	0
Gm4890	0.4343626	-0.1706524	0.26943097	-0.6333806	0.52648511	NA		Gm4890	0	0.45252372	0.9542371	2.06814001	0	0	0	0
Olfr1344	0.4343626	-0.1706524	0.26943097	-0.6333806	0.52648511	NA		Olfr1344	0	0.45252372	0.9542371	2.06814001	0	0	0	0
Tctex1d1	0.4343626	-0.1706524	0.26943097	-0.6333806	0.52648511	NA		Tctex1d1	0	0.45252372	0.9542371	2.06814001	0	0	0	0
A630072M18Rik	6.2097872	0.2633775	0.41597133	0.63316263	0.52662745	0.99938111		A630072M18R	6.72046328	5.88280835	2.86271131	4.13628002	8.3240586	0	5.77358333	15.9783927
Lzts2	29.8629776	0.25058618	0.39581012	0.63309695	0.52667034	0.99938111		Lzts2	13.4409266	19.0059962	18.766663	53.7716403	59.9332219	12.9365998	15.3962222	45.6525506
D630041G03Rik	5.83974902	-0.2527734	0.39929812	-0.6330443	0.52670474	0.99938111		D630041G03R	8.06455594	6.33533207	6.67965972	4.13628002	4.99443516	3.52816357	3.84905555	9.13051012
Nfib	103.229716	0.20817187	0.32905314	0.63263906	0.52696937	0.99938111		Nfib	107.527413	111.773359	103.057607	53.7716403	91.5646446	110.549125	190.52825	57.0656883
D330050G23Rik	0.82675147	-0.2235315	0.35334092	-0.6326226	0.52698012	NA		D330050G23R	1.34409266	0.90504744	2.22655324	0	0	1.17605452	0.96226389	0
Mbnl2	191.595282	-0.1986946	0.31419664	-0.6323892	0.52713256	0.99938111		Mbnl2	219.759149	248.888045	237.605039	124.088401	109.877574	230.506687	250.188611	111.848749
Prss35	0.82131926	-0.2154987	0.34077179	-0.6323842	0.52713585	NA		Prss35	2.01613899	1.35757116	1.27231614	0	0	0	1.92452778	0
Ptpla	0.82131926	-0.2154987	0.34077179	-0.6323842	0.52713585	NA		Ptpla	2.01613899	1.35757116	1.27231614	0	0	0	1.92452778	0
Dmtf1	38.5874522	-0.2334624	0.36921946	-0.6323132	0.52718221	0.99938111		Dmtf1	57.1239379	52.9452751	46.7576181	14.4769801	18.3129289	51.7463991	49.0754583	18.2610202
Fbxl4	10.09973	-0.2410158	0.38120372	-0.6322493	0.52722402	0.99938111		Fbxl4	8.06455594	15.3858064	12.4050823	10.3407001	6.65924688	14.1126543	11.5471667	2.28262753
Mphosph10	9.11258511	0.26180441	0.41413125	0.63217738	0.52727098	0.99938111		Mphosph10	9.4086486	8.14542694	9.224292	4.13628002	0	15.2887088	22.1320694	4.56525506
Sbno2	5.32177516	0.25870914	0.40929749	0.63208094	0.52733399	0.99938111		Sbno2	2.68818531	5.88280835	3.18079035	6.20442003	8.3240586	2.35210905	4.81131944	9.13051012
Fam102a	127.934123	-0.1972456	0.31210711	-0.6319804	0.52739969	0.99938111		Fam102a	132.393127	120.371309	98.6045007	202.677721	154.82749	68.2111624	86.60375	159.783927
Sec24a	23.2347815	0.1904813	0.30142101	0.63194432	0.52742326	0.99938111		Sec24a	20.1613899	17.648425	19.4028211	31.0221002	21.6425524	25.8731995	25.0188611	25.1089028
Ctsz	6.02288979	-0.2601451	0.41167655	-0.6319162	0.52744162	0.99938111		Ctsz	4.03227797	11.313093	5.72542262	6.20442003	8.3240586	7.05632714	0.96226389	4.56525506
Coq4	11.9337509	-0.2310268	0.36569358	-0.6317496	0.52755054	0.99938111		Coq4	15.4570656	12.2181404	10.4966081	14.4769801	4.99443516	7.05632714	12.5094306	18.2610202
Cacna2d1	103.442833	-0.2544522	0.40277851	-0.6317422	0.52755535	0.99938111		Cacna2d1	102.823088	204.540721	139.318617	24.8176801	53.2739751	177.584233	100.075444	25.1089028
1110034G24Rik	3.46907681	-0.2589765	0.409971	-0.6316947	0.52758642	NA		1110034G24R	5.37637063	2.26261859	4.13502745	4.13628002	4.99443516	0	0	6.84788259
Slc35a3	6.90884328	-0.2618987	0.41460936	-0.6316759	0.52759869	0.99938111		Slc35a3	9.4086486	11.313093	9.86045007	2.06814001	1.66481172	9.40843619	11.5471667	0
Med4	6.76883099	-0.2486956	0.39371728	-0.6316603	0.5276089	0.99938111		Med4	7.39250961	8.14542694	9.224292	6.20442003	3.32962344	4.7042181	10.5849028	4.56525506

GeneID	Base mean	log2(FC)	StdErr	Wald-Stats	P-value	P-adj	GeneID	Normalised expression for Chow#1	Normalised expression for Chow#2	Normalised expression for Chow#3	Normalised expression for Chow#4	Normalised expression for HFD#1	Normalised expression for HFD#2	Normalised expression for HFD#3	Normalised expression for HFD#4
Kctd16	15.1184952	-0.221567	0.35084712	-0.6315201	0.52770052	0.99938111	Kctd16	19.4893435	18.5534725	16.8581888	12.4088401	6.65924688	17.6408179	20.2075417	9.13051012
Tmem110	8.0155413	-0.2417575	0.3828939	-0.6313955	0.52778196	0.99938111	Tmem110	6.04841696	11.313093	8.58813393	10.3407001	6.65924688	4.7042181	9.62263889	6.84788259
Hist1h3d	10.1371405	-0.240005	0.3801729	-0.6313049	0.52784117	0.99938111	Hist1h3d	8.73660227	14.480759	7.31581779	14.4769801	9.98887032	4.7042181	7.69811111	13.6957652
Lamtor1	40.6069157	-0.2406622	0.38123227	-0.6312746	0.52786099	0.99938111	Lamtor1	30.2420848	28.5089943	32.7621406	90.9981605	36.6258578	12.9365998	28.8679167	63.9135709
Eif4h	312.98071	0.2187395	0.34655731	0.63117843	0.52792387	0.99938111	Eif4h	210.350501	190.059962	202.616345	529.443843	439.510294	189.344778	254.037667	488.482292
Gm5148	50.1083297	-0.2437268	0.38624421	-0.6310175	0.5280291	0.99938111	Gm5148	77.2853278	42.0847059	65.8423601	41.3628002	16.6481172	43.5140174	98.1509166	15.9783927
Manea	32.8420013	0.26225941	0.41567721	0.63092082	0.5280923	0.99938111	Manea	16.1291119	47.9675142	37.8514051	4.13628002	3.32962344	96.436471	50.0377222	6.84788259
Wdpcp	4.49373841	-0.2606845	0.41344205	-0.6305225	0.52835281	NA	Wdpcp	5.37637063	6.78785578	3.49886938	6.20442003	4.99443516	2.35210905	6.73584722	0
Vps53	49.3667823	0.16734354	0.2654276	0.63046776	0.52838859	0.99938111	Vps53	57.7959842	44.3473245	42.9406697	39.2946602	63.2628454	42.3379629	61.5848889	43.3699231
Smad3	52.4572256	-0.1671887	0.26522046	-0.6303763	0.5284484	0.99938111	Smad3	55.1077989	65.6159392	53.7553568	47.5672202	69.9220923	42.3379629	44.2641389	41.0872956
Dlx6as2	0.43911407	0.17060427	0.27067446	0.6302932	0.52850277	NA	Dlx6as2	0.67204633	0	0	0	1.66481172	1.17605452	0	0
Mocos	0.43911407	0.17060427	0.27067446	0.6302932	0.52850277	NA	Mocos	0.67204633	0	0	0	1.66481172	1.17605452	0	0
Uhrf1	0.43911407	0.17060427	0.27067446	0.6302932	0.52850277	NA	Uhrf1	0.67204633	0	0	0	1.66481172	1.17605452	0	0
Asic3	0.62722235	0.15678144	0.24875947	0.63025318	0.52852895	NA	Asic3	0	0.45252372	0	0	0	0	0	4.56525506
Snhg3	4.38945143	0.2602665	0.41297092	0.63022961	0.52854437	NA	Snhg3	0.67204633	0.90504744	3.81694841	10.3407001	1.66481172	3.52816357	9.62263889	4.56525506
Ecsit	25.6691427	0.19453164	0.30867364	0.63021786	0.52855206	0.99938111	Ecsit	30.2420848	18.5534725	24.1740066	20.6814001	36.6258578	18.8168724	28.8679167	27.3915304
A930003A15Rik	0.3651483	-0.1434352	0.22761624	-0.6301622	0.52858845	NA	930003A15R	2.01613899	0.90504744	0	0	0	0	0	0
Tnfaip8l2	0.3651483	-0.1434352	0.22761624	-0.6301622	0.52858845	NA	Tnfaip8l2	2.01613899	0.90504744	0	0	0	0	0	0
Trp53cor1	0.3651483	-0.1434352	0.22761624	-0.6301622	0.52858845	NA	Trp53cor1	2.01613899	0.90504744	0	0	0	0	0	0
Wfdc18	0.3651483	-0.1434352	0.22761624	-0.6301622	0.52858845	NA	Wfdc18	2.01613899	0.90504744	0	0	0	0	0	0
Ptchd2	26.8770659	-0.2394661	0.38006348	-0.6300688	0.52864957	0.99938111	Ptchd2	18.1452509	49.3250854	22.5836114	28.9539601	38.2906696	25.8731995	6.73584722	25.1089028
Fam118b	8.63839775	0.25554474	0.40565137	0.6299615	0.52871978	0.99938111	Fam118b	10.0806949	9.05047438	9.86045007	0	8.3240586	14.1126543	15.3962222	2.28262753
Mapk8	88.6373766	0.21889299	0.34747777	0.62994818	0.52872849	0.99938111	Mapk8	104.167181	85.9795066	96.0598684	33.0902402	56.6035985	117.605452	153.962222	61.6309433
Gm15663	8.34550066	-0.2495483	0.39616237	-0.6299143	0.52875066	0.99938111	Gm15663	6.72046328	7.69290322	8.2700549	16.5451201	3.32962344	3.52816357	11.5471667	9.13051012
Wdr45	17.5809398	0.21887578	0.34749915	0.62985991	0.52878624	0.99938111	Wdr45	17.4732045	10.4080455	18.766663	18.6132601	11.653682	21.1689814	28.8679167	13.6957652
Zbtb11	49.2435231	0.21849683	0.34691805	0.6298226	0.52881066	0.99938111	Zbtb11	53.7637063	47.0624668	46.7576181	31.0221002	29.966611	92.9083074	67.3584722	25.1089028
Arhgef3	46.9301896	0.19160307	0.3042999	0.62965211	0.52892222	0.99938111	Arhgef3	51.7475673	39.3695635	55.9819101	24.8176801	46.6147282	48.2182355	72.1697916	36.5220405
Tnk2	143.768402	0.22736464	0.36120295	0.62946508	0.52904463	0.99938111	Tnk2	83.3337447	108.153169	95.1056313	229.563541	261.37544	90.5561984	78.9056389	203.15385
Dhx29	12.8363004	0.25964361	0.41261691	0.62926072	0.52917838	0.99938111	Dhx29	16.1291119	12.2181404	12.7231614	2.06814001	11.653682	30.5774176	17.32075	0
Mc3r	0.83730801	-0.2108659	0.33511157	-0.6292409	0.52919137	NA	Mc3r	0.67204633	1.35757116	0.31807903	2.06814001	0	0	0	2.28262753
C2cd4d	1.68515971	0.12281514	0.19518403	0.62922743	0.52920018	NA	C2cd4d	0	0	0	2.06814001	0	0	0	11.4131377
Hist1h2ab	1.65407666	-0.2517707	0.40016369	-0.6291692	0.52923831	NA	Hist1h2ab	3.36023164	2.26261859	2.22655324	0	0	1.17605452	1.92452778	2.28262753
Phlda3	26.1458543	-0.2617824	0.41635198	-0.6287527	0.52951098	0.99938111	Phlda3	15.4570656	8.14542694	6.04350166	95.1344405	18.3129289	11.7605452	8.660375	45.6525506
Gm11110	0.30409681	-0.1622408	0.25804047	-0.6287415	0.52951828	NA	Gm11110	1.34409266	0.45252372	0.63615807	0	0	0	0	0
Plscr1	0.30409681	-0.1622408	0.25804047	-0.6287415	0.52951828	NA	Plscr1	1.34409266	0.45252372	0.63615807	0	0	0	0	0
BC005537	148.374352	0.13453609	0.21398582	0.62871497	0.52953568	0.99938111	BC005537	139.785636	130.326831	152.996016	140.633521	131.520126	132.894161	187.641458	171.197065
Slc38a4	0.55974852	-0.2000035	0.31813424	-0.6286764	0.52956095	NA	Slc38a4	0.67204633	1.35757116	1.27231614	0	0	1.17605452	0	0
Anxa11	5.73042974	-0.2551601	0.40590199	-0.6286249	0.52959462	0.99938111	Anxa11	4.03227797	6.78785578	7.31581779	8.27256004	9.98887032	2.35210905	4.81131944	2.28262753
Rgs3	9.94384576	-0.2401553	0.38209827	-0.628517	0.5296653	0.99938111	Rgs3	7.39250961	11.313093	10.4966081	14.4769801	4.99443516	5.88027262	6.73584722	18.2610202
Trappc1	28.2772642	-0.2077439	0.33053218	-0.6285135	0.52966762	0.99938111	Trappc1	34.2743627	28.961518	33.3398286	26.8858201	11.653682	19.9929269	41.3773472	29.6741579
Cib1	5.65776245	-0.2596396	0.41310633	-0.6285056	0.52967279	0.99938111	Cib1	4.7043243	7.69290322	3.81694841	10.3407001	1.66481172	1.17605452	6.73584722	9.13051012
2410131K14Rik	20.582682	-0.2265334	0.36046221	-0.6284526	0.52970746	0.99938111	410131K14Ri	13.4409266	20.8160911	16.5401098	41.3628002	13.3184938	14.1126543	15.3962222	29.6741579
Ccdc14	1.56106887	-0.2468983	0.39309763	-0.628084	0.52994891	NA	Ccdc14	2.68818531	2.71514231	0.9542371	2.06814001	0	1.17605452	2.88679167	0
Hook2	4.66016885	0.25993514	0.41390621	0.62800492	0.53000072	0.99938111	Hook2	3.36023164	4.07271347	2.22655324	6.20442003	1.66481172	3.52816357	4.81131944	11.4131377
Acsm3	0.57038326	-0.1981915	0.3155898	-0.6280035	0.53000162	NA	Acsm3	1.34409266	0.45252372	1.59039517	0	0	1.17605452	0	0
Fam181b	11.17613	-0.260504	0.41483405	-0.6279716	0.53002253	0.99938111	Fam181b	12.0968339	6.78785578	8.58813393	24.8176801	21.6425524	1.17605452	2.88679167	11.4131377
Ifitm10	11.4077484	-0.2581127	0.41111274	-0.6278393	0.53010924	0.99938111	Ifitm10	5.37637063	9.5029981	7.63389683	31.0221002	4.99443516	3.52816357	8.660375	20.5436478
Aptx	19.4601628	0.21100451	0.33613552	0.62773644	0.53017661	0.99938111	Aptx	19.4893435	16.7433776	18.766663	16.5451201	14.9833055	15.2887088	35.6037639	18.2610202
1110059E24Rik	8.7226703	-0.2578221	0.41096082	-0.6273642	0.53042052	0.99938111	110059E24R	10.0806949	11.7656167	16.2220308	2.06814001	1.66481172	7.05632714	16.3584861	4.56525506
Bcas3	45.0443265	-0.1686955	0.26896081	-0.6272121	0.53052022	0.99938111	Bcas3	58.4680306	42.5372296	41.0321955	49.6353602	51.6091633	32.9295267	38.4905555	45.6525506
Zscan2	3.93035163	0.26034904	0.41517159	0.62708782	0.53060167	NA	Zscan2	5.37637063	3.62018975	3.18079035	0	3.32962344	2.35210905	6.73584722	6.84788259
Slc39a6	45.7098633	0.22101644	0.35250105	0.62699513	0.53066243	0.99938111	Slc39a6	31.5861774	61.0907021	44.5310648	26.8858201	36.6258578	96.436471	36.5660278	31.9567854
Npl	1.71785875	-0.2505146	0.39962567	-0.6268731	0.53074242	NA	Npl	2.01613899	2.26261859	2.22655324	2.06814001	0	0	2.88679167	2.28262753
Nr2f6	19.1538624	0.25753855	0.4108386	0.62686066	0.53075058	0.99938111	Nr2f6	14.1129729	8.59795066	11.7689243	31.0221002	46.6147282	4.7042181	6.73584722	29.6741579
Kl	9.8676606	0.25009253	0.39929566	0.62633421	0.53109575	0.99938111	Kl	2.68818531	8.14542694	10.4966081	12.4088401	6.65924688	7.05632714	8.660375	22.8262753
Acn9	4.16697463	0.2607172	0.41626509	0.62632493	0.5311018	NA	Acn9	4.7043243	4.52523719	2.86271131	2.06814001	0	8.23238167	8.660375	2.28262753
Ap5b1	5.94970981	0.2607214	0.41637072	0.62617612	0.53119943	0.99938111	Ap5b1	3.36023164	4.97776091	1.90847421	10.3407001	18.3129289	3.52816357	2.88679167	2.28262753
Cct8	104.413846	0.21572079	0.34468595	0.62584735	0.53141507	0.99938111	Cct8	122.312432	100.912789	100.831054	53.7716403	51.6091633	174.05607	158.773542	73.044081
Atp5g3	158.731554	-0.1756098	0.2806073	-0.6258206	0.53143261	0.99938111	Atp5g3	135.753358	141.1874	162.538387	241.972381	121.531256	98.78858	180.905611	187.175458
Brd9	36.4078478	0.19647353	0.31395634	0.62579889	0.53144686	0.99938111	Brd9	31.5861774	30.7716129	30.8536663	43.4309402	29.966611	28.2253086	64.4716805	31.9567854
Ern2	0.41239024	0.16861207	0.26947031	0.6257167	0.53150078	NA	Ern2	0.67204633	0	0	0	1.66481172	0	0.96226389	0
Gm3417	0.41239024	0.16861207	0.26947031	0.6257167	0.53150078	NA	Gm3417	0.67204633	0	0	0	1.66481172	0	0.96226389	0
Gm3448	0.41239024	0.16861207	0.26947031	0.6257167	0.53150078	NA	Gm3448	0.67204633	0	0	0	1.66481172	0	0.96226389	0
Cacna2d4	1.53859574	0.23503194	0.37566051	0.62564984	0.53154464	NA	Cacna2d4	0	0.45252372	1.90847421	2.06814001	0	2.35210905	0.96226389	4.56525506
Evi2a-evi2b	0.60024703	-0.1727424	0.27611925	-0.625608	0.53157206	NA	Evi2a-evi2b	0.67204633	3.16766603	0	0	0	0	0.96226389	0
Syt17	41.9630378	-0.1670592	0.26709412	-0.6254692	0.53166313	0.99938111	Syt17	47.7152893	44.3473245	39.7598793	49.6353602	38.2906696	35.2816357	50.9999861	29.6741579
Rsph9	5.94140624	0.25727119	0.41134121	0.62544472	0.53167922	0.99938111	Rsph9	4.03227797	4.07271347	2.54463228	10.3407001	3.32962344	4.7042181	4.81131944	13.6957652
Rps17	9.14021731	-0.2523447	0.40354527	-0.6253194	0.53176146	0.99938111	Rps17	8.73660227	6.78785578	8.90621297	18.6132601	1.66481172	3.52816357	13.4716944	11.4131377
Raph1	82.2740085	-0.1833842	0.29332313	-0.625195	0.53184307	0.99938111	Raph1	94.7585323	96.8400759	99.8768168	62.0442003	43.2851047	107.020962	90.4528055	63.9135709
Gnb4	23.3180831	0.20883833	0.33405575	0.62516012	0.53186597	0.99938111	Gnb4	22.1775288	14.9332827	26.4005599	20.6814001	16.6481172	16.4647633	32.7169722	36.5220405
Lbh	146.645916	0.2551624	0.40816067	0.62515184	0.53187141	0.99938111	Lbh	65.8605402	53.8503226	76.0208892	306.084722	143.173808	52.9224536	89.4905416	385.764053
Svop	106.46616	0.1925954	0.3080844	0.62513844	0.5318802	0.99938111	Svop	69.8928182	111.773359	91.2886829	117.883981	156.492302	94.0843619	59.6603611	150.653417
Ubap2l	94.6048037	-0.1354817	0.21673333	-0.6251078	0.53190031	0.99938111	Ubap2l	104.167181	92.3148387	98.9225797	103.407001	108.212762	81.1477622	93.3395972	75.3267085
Palmd	6.21593653	0.25841092	0.41347057	0.62498021	0.53198405	0.99938111	Palmd	2.68818531	10.8605693	6.04350166	0	8.3240586	3.52816357	18.2830139	0
Pikfyve	51.9900829	0.21369096	0.34192107	0.62497162	0.53198969	0.99938111	Pikfyve	48.3873356	45.2523719	58.2084633	37.2265202	64.9276571	88.2040893	57.7358333	15.9783927
Zfp3	6.01563041	-0.2573636	0.41181159	-0.6249548	0.53200072	0.99938111	Zfp3	10.0806949	10.4080455	6.36158069	0	6.65924688	2.35210905	7.69811111	4.56525506
Gpd1l	104.015147	0.1465274	0.23446338	0.62494795	0.53200522	0.99938111	Gpd1l	123.656524	83.2643643	88.7440506	97.2025805	118.201632	88.2040893	116.433931	116.414004
Ntng1	26.6648181	-0.2419008	0.38707848	-0.6249399	0.53201051	0.99938111	Ntng1	29.5700384	40.274611	37.8514051	12.4088401	14.9833055	39.9858538	33.6792361	4.56525506
Snrpd2	9.39812643	0.23554977	0.37693234	0.62491261	0.53202841	0.99938111	Snrpd2	12.7688802	6.78785578	6.04350166	8.27256004	11.653682	7.05632714	13.4716944	9.13051012
Cadm3	322.982614	-0.1854721	0.29694775	-0.6245951	0.53223685	0.99938111	Cadm3	280.243319	504.111423	355.294282	246.108661	356.269708	331.647376	165.509389	344.676757
Rgs7	65.6189822	-0.2163567	0.34643273	-0.6245274	0.53228127	0.99938111	Rgs7	106.855366	67.8785578	66.7965972	47.5672202	33.2962344	77.6195986	95.264125	29.6741579
2410007B07Rik	0.29401363	0.1169827	0.18735887	0.62437767	0.53237959	NA	410007B07Ri	0	0	0	0	0	2.35210905	0	0
4930461G14Rik	0.29401363	0.1169827	0.18735887	0.62437767	0.53237959	NA	930461G14R	0	0	0	0	0	2.35210905	0	0
7630403G23Rik	0.29401363	0.1169827	0.18735887	0.62437767	0.53237959	NA	630403G23R	0	0	0	0	0	2.35210905	0	0
Abra	0.29401363	0.1169827	0.18735887	0.62437767	0.53237959	NA	Abra	0	0	0	0	0	2.35210905	0	0
Apob	0.29401363	0.1169827	0.18735887	0.62437767	0.53237959	NA	Apob	0	0	0	0	0	2.35210905	0	0
BC021785	0.29401363	0.1169827	0.18735887	0.62437767	0.53237959	NA	BC021785	0	0	0	0	0	2.35210905	0	0
Cd300lh	0.29401363	0.1169827	0.18735887	0.62437767	0.53237959	NA	Cd300lh	0	0	0	0	0	2.35210905	0	0
Clec2f	0.29401363	0.1169827	0.18735887	0.62437767	0.53237959	NA	Clec2f	0	0	0	0	0	2.35210905	0	0
Cyp4f41-ps	0.29401363	0.1169827	0.18735887	0.62437767	0.53237959	NA	Cyp4f41-ps	0	0	0	0	0	2.35210905	0	0
Fam26f	0.29401363	0.1169827	0.18735887	0.62437767	0.53237959	NA	Fam26f	0	0	0	0	0	2.35210905	0	0
Gm10640	0.29401363	0.1169827	0.18735887	0.62437767	0.53237959	NA	Gm10640	0	0	0	0	0	2.35210905	0	0
Gm11166	0.29401363	0.1169827	0.18735887	0.62437767	0.53237959	NA	Gm11166	0	0	0	0	0	2.35210905	0	0
Naip2	0.29401363	0.1169827	0.18735887	0.62437767	0.53237959	NA	Naip2	0	0	0	0	0	2.35210905	0	0
Pate4	0.29401363	0.1169827	0.18735887	0.62437767	0.53237959	NA	Pate4	0	0	0	0	0	2.35210905	0	0
Psg20	0.29401363	0.1169827	0.18735887	0.62437767	0.53237959	NA	Psg20	0	0	0	0	0	2.35210905	0	0
1700021F07Rik	0.24056597	0.1169827	0.18735887	0.62437766	0.53237959	NA	700021F07Ri	0	0	0	0	0	0	1.92452778	0
4930430F21Rik	0.24056597	0.1169827	0.18735887	0.62437766	0.53237959	NA	930430F21Ri	0	0	0	0	0	0	1.92452778	0
Aknad1	0.24056597	0.1169827	0.18735887	0.62437766	0.53237959	NA	Aknad1	0	0	0	0	0	0	1.92452778	0
Capn13	0.24056597	0.1169827	0.18735887	0.62437766	0.53237959	NA	Capn13	0	0	0	0	0	0	1.92452778	0
Ccdc175	0.24056597	0.1169827	0.18735887	0.62437766	0.53237959	NA	Ccdc175	0	0	0	0	0	0	1.92452778	0
Dmrt3	0.24056597	0.1169827	0.18735887	0.62437766	0.53237959	NA	Dmrt3	0	0	0	0	0	0	1.92452778	0
Glod5	0.24056597	0.1169827	0.18735887	0.62437766	0.53237959	NA	Glod5	0	0	0	0	0	0	1.92452778	0
Gm13807	0.24056597	0.1169827	0.18735887	0.62437766	0.53237959	NA	Gm13807	0	0	0	0	0	0	1.92452778	0
Gm19557	0.24056597	0.1169827	0.18735887	0.62437766	0.53237959	NA	Gm19557	0	0	0	0	0	0	1.92452778	0
Gm9958	0.24056597	0.1169827	0.18735887	0.62437766	0.53237959	NA	Gm9958	0	0	0	0	0	0	1.92452778	0
Gpr82	0.24056597	0.1169827	0.18735887	0.62437766	0.53237959	NA	Gpr82	0	0	0	0	0	0	1.92452778	0
Krtap7-1	0.24056597	0.1169827	0.18735887	0.62437766	0.53237959	NA	Krtap7-1	0	0	0	0	0	0	1.92452778	0
Lkaaear1	0.24056597	0.1169827	0.18735887	0.62437766	0.53237959	NA	Lkaaear1	0	0	0	0	0	0	1.92452778	0
Mfi2	0.24056597	0.1169827	0.18735887	0.62437766	0.53237959	NA	Mfi2	0	0	0	0	0	0	1.92452778	0
Mir1898	0.24056597	0.1169827	0.18735887	0.62437766	0.53237959	NA	Mir1898	0	0	0	0	0	0	1.92452778	0
Otx2	0.24056597	0.1169827	0.18735887	0.62437766	0.53237959	NA	Otx2	0	0	0	0	0	0	1.92452778	0

GeneID	Base mean	log2(FC)	StdErr	Wald-Stats	P-value	P-adj		GeneID	Normalised expression for Chow#1	Normalised expression for Chow#2	Normalised expression for Chow#3	Normalised expression for Chow#4	Normalised expression for HFD#1	Normalised expression for HFD#2	Normalised expression for HFD#3	Normalised expression for HFD#4
Pbp2	0.24056597	0.1169827	0.18735887	0.62437766	0.53237959	NA		Pbp2	0	0	0	0	0	0	1.92452778	0
Steap1	0.24056597	0.1169827	0.18735887	0.62437766	0.53237959	NA		Steap1	0	0	0	0	0	0	1.92452778	0
Jmjd1c	49.8477609	0.21594695	0.34588382	0.62433377	0.53240841	0.99938111		Jmjd1c	52.4196136	55.6604174	51.2107246	20.6814001	39.9554813	72.9153805	80.8301666	25.1089028
Patl1	28.3544866	-0.1840795	0.29494364	-0.6241175	0.53255044	0.99938111		Patl1	23.5216215	37.106945	29.8994292	31.0221002	19.9777406	23.5210905	29.8301805	31.9567854
2900026A02Rik	42.3509336	-0.2079398	0.33325137	-0.6239729	0.53264538	0.99938111		2900026A02Ri	38.978687	47.0624668	38.8056422	59.9760603	69.9220923	19.9929269	29.8301805	34.239413
Nrep	24.7702549	0.20716988	0.33207965	0.62385598	0.53272217	0.99938111		Nrep	24.8657142	14.9332827	23.8559276	26.8858201	23.3073641	14.1126543	33.6792361	36.5220405
Esam	1.7933658	-0.2318868	0.37172901	-0.6238061	0.53275496	NA		Esam	0	3.16766603	3.49886938	2.06814001	3.32962344	0	0	2.28262753
Polr3g	3.00564602	-0.2596798	0.41633621	-0.6237263	0.53280737	NA		Polr3g	5.37637063	4.52523719	2.54463228	2.06814001	3.32962344	2.35210905	3.84905555	0
Acin1	46.7596925	0.16283591	0.26108477	0.62368981	0.53283131	0.99938111		Acin1	46.3711967	37.106945	39.4418003	53.7716403	49.9443516	56.4506172	38.4905555	52.5004332
Socs2	4.97896125	-0.2573675	0.41268107	-0.6236474	0.5328592	0.99938111		Socs2	9.4086486	4.07271347	7.63389683	2.06814001	4.99443516	5.88027262	5.77358333	0
Ajuba	1.03675933	-0.2222082	0.35641825	-0.6234479	0.53299026	NA		Ajuba	2.68818531	0.45252372	1.90847421	0	0	0	0.96226389	2.28262753
Agpat3	132.400069	-0.1859021	0.29824073	-0.6233288	0.53306845	0.99938111		Agpat3	106.18332	171.053966	115.780769	175.791901	158.157113	112.901234	66.3962083	152.936045
Hsdl1	52.0312427	0.18834338	0.30219706	0.62324689	0.53312229	0.99938111		Hsdl1	39.6507334	39.8220873	35.3067728	80.6574604	66.5924688	43.5140174	49.0754583	61.6309433
Ept1	47.7034228	0.21791107	0.34965426	0.62321869	0.53314082	0.99938111		Ept1	34.2743627	63.3533207	55.9819101	16.5451201	46.6147282	84.6759257	41.3773472	38.804668
Gm16740	0.76008103	-0.2073822	0.33281749	-0.6231109	0.53321164	NA		Gm16740	0.67204633	1.35757116	0.31807903	2.06814001	1.66481172	0	0	0
Mphosph8	32.7416179	0.23751092	0.38131192	0.62287832	0.53336449	0.99938111		Mphosph8	32.2582238	31.2241366	32.1259825	20.6814001	9.98887032	74.091435	43.301875	18.2610202
Rassf4	5.56295015	0.25506441	0.40962228	0.62268196	0.53349354	0.99938111		Rassf4	7.39250961	4.07271347	5.72542262	0	4.99443516	7.05632714	3.84905555	11.4131377
Zfp938	14.4660044	-0.2206982	0.3544599	-0.6226324	0.5335261	0.99938111		Zfp938	12.0968339	12.6706641	17.8124259	22.7495401	6.65924688	11.7605452	18.2830139	13.6957652
4933408J17Rik	2.13055665	0.21427398	0.3442001	0.62252734	0.53359517	NA		4933408J17Ri	0	0	0.31807903	6.20442003	4.99443516	0	0.96226389	4.56525506
Lmnb2	9.57200713	0.25315523	0.40668895	0.62247876	0.53362711	0.99938111		Lmnb2	9.4086486	3.16766603	4.77118552	16.5451201	9.98887032	10.5844907	3.84905555	18.2610202
Derl1	27.7369575	0.22561191	0.36245313	0.62245815	0.53364065	0.99938111		Derl1	15.4570656	41.6321821	26.4005599	14.4769801	29.966611	50.5703445	18.2830139	25.1089028
Slc35b3	2.82286459	-0.2589491	0.41601935	-0.6224447	0.53364947	NA		Slc35b3	2.68818531	2.71514231	3.49886938	4.13628002	1.66481172	2.35210905	0.96226389	4.56525506
St6gal2	11.7347181	-0.2336611	0.37551359	-0.622244	0.53378143	0.99938111		St6gal2	12.7688802	19.9110436	14.3135566	4.13628002	9.98887032	8.23238167	15.3962222	9.13051012
Brsk2	129.197091	0.18329925	0.29475248	0.62187517	0.53402395	0.99938111		Brsk2	119.624246	98.6501707	99.2406588	161.314921	191.453348	89.3801438	98.1509166	175.76232
A330076H08Rik	1.57631988	0.24868153	0.39993694	0.62180186	0.53407217	NA		A330076H08R	1.34409266	1.81009488	1.59039517	0	1.66481172	2.35210905	3.84905555	0
Atp13a2	88.6136181	0.23642726	0.38033722	0.62162536	0.53418824	0.99938111		Atp13a2	43.6830113	77.8340797	54.0734359	138.565381	164.81636	63.5069443	31.7547083	134.675024
Nod1	1.51531208	0.22500735	0.36196764	0.62162285	0.53418989	NA		Nod1	0	2.71514231	0.9542371	0	4.99443516	1.17605452	0	2.28262753
Dnah17	0.97820855	-0.1984737	0.31929464	-0.6216005	0.53420456	NA		Dnah17	0	0.45252372	0.9542371	4.13628002	0	0	0	2.28262753
Rest	1.92800098	-0.2503759	0.40281768	-0.6215615	0.53423027	NA		Rest	2.68818531	0.90504744	2.22655324	4.13628002	3.32962344	1.17605452	0.96226389	0
Gxylt1	5.22172934	0.25320355	0.40747172	0.62140153	0.53433546	0.99938111		Gxylt1	4.7043243	6.33533207	4.77118552	2.06814001	3.32962344	10.5844907	7.69811111	2.28262753
Bcl11a	78.9287308	-0.2112036	0.33989439	-0.62138	0.53434964	0.99938111		Bcl11a	110.215598	105.89055	85.8813393	43.4309402	54.9387868	82.3238167	114.509403	34.239413
Amer1	8.36227744	0.23623669	0.38019494	0.62135675	0.53436492	0.99938111		Amer1	5.37637063	6.78785578	7.95197586	10.3407001	14.9833055	8.23238167	8.660375	4.56525506
Tmem242	35.8537266	-0.2378999	0.38307459	-0.6210277	0.53458137	0.99938111		Tmem242	28.2259458	18.1009488	23.8559276	93.0663005	24.9721758	19.9929269	37.5282917	41.0872956
6330549D23Rik	4.02100067	-0.2569021	0.4137033	-0.6209815	0.53461181	NA		6330549D23Ri	4.03227797	7.2403795	5.08926455	2.06814001	3.32962344	2.35210905	5.77358333	2.28262753
Dpy30	16.3177414	0.21769689	0.35063664	0.62086178	0.53469057	0.99938111		Dpy30	16.1291119	13.1231878	13.0412404	18.6132601	8.3240586	16.4647633	28.8679167	15.9783927
Nhlh1	1.04309527	0.20929564	0.33715882	0.62076277	0.53475572	NA		Nhlh1	1.34409266	0.45252372	0.31807903	0	1.66481172	0	0	4.56525506
Dcx	22.8415019	0.24582183	0.39616294	0.62050687	0.53492413	0.99938111		Dcx	22.8495752	19.4585199	16.5401098	22.7495401	13.3184938	36.4576902	49.0754583	2.28262753
Zfp879	1.67035244	0.2479524	0.399613	0.62048132	0.53494095	NA		Zfp879	1.34409266	2.26261859	1.27231614	0	0	2.35210905	3.84905555	2.28262753
Zfp799	14.1767747	0.2227653	0.35904473	0.62043884	0.53496891	0.99938111		Zfp799	10.7527413	14.0282353	15.9039517	10.3407001	9.98887032	11.7605452	26.9433889	13.6957652
Zfp932	11.3574554	0.25761908	0.41529075	0.62033425	0.53503775	0.99938111		Zfp932	11.4247876	10.4080455	15.5858727	0	3.32962344	28.2253086	17.32075	4.56525506
Rpl15	42.8015588	0.21662168	0.34925085	0.62024667	0.5350954	0.99938111		Rpl15	36.9625481	41.6321821	45.803381	31.0221002	16.6481172	55.2745626	80.8301666	34.239413
Zfp672	24.4758877	0.18639834	0.30057829	0.62013242	0.53517061	0.99938111		Zfp672	18.8172972	28.5089943	22.9016905	18.6132601	24.9721758	21.1689814	28.8679167	31.9567854
Mtmr7	61.0916132	0.15069358	0.24308429	0.61992318	0.53530836	0.99938111		Mtmr7	64.5164475	51.1351802	58.5265423	57.9079203	51.6091633	72.9153805	75.0565833	57.0656883
9930111J21Rik1	0.52070514	-0.1959565	0.3161348	-0.6198512	0.53535575	NA		9930111J21Rik	1.34409266	0.90504744	0.9542371	0	0	0	0.96226389	0
F13a1	1.1666826	0.22088318	0.35645485	0.61966663	0.53547729	NA		F13a1	0	1.35757116	1.27231614	0	0	1.17605452	0.96226389	4.56525506
Myof	1.68641939	0.24625081	0.39745001	0.61957681	0.53553644	NA		Myof	1.34409266	0.90504744	2.54463228	0	0	3.52816357	2.88679167	2.28262753
Zdhhc5	52.248818	-0.1952534	0.31528881	-0.6192842	0.53572912	0.99938111		Zdhhc5	43.6830113	89.1471726	55.345752	37.2265202	39.9554813	67.0351079	49.0754583	36.5220405
Pspc1	22.5144661	0.22500596	0.3633967	0.61917447	0.53580143	0.99938111		Pspc1	25.5377605	13.5757116	20.6751372	22.7495401	8.3240586	30.5774176	40.4150833	18.2610202
Skiv2l2	33.2448239	0.22352368	0.36100717	0.61916685	0.53580645	0.99938111		Skiv2l2	42.3389187	25.3413283	29.8994292	22.7495401	23.3073641	68.2111624	40.4150833	13.6957652
Atf6	41.8999111	0.2040769	0.32961363	0.61913973	0.53582431	0.99938111		Atf6	35.6184554	48.8725616	48.0299342	18.6132601	34.9610461	74.091435	38.4905555	36.5220405
Fam120b	59.8757292	-0.161517	0.26098059	-0.6188849	0.53599217	0.99938111		Fam120b	56.4518916	58.8280835	64.570044	76.5211804	58.2684102	74.091435	42.3396111	47.9351782
Btg1	18.7192847	-0.2297123	0.37117933	-0.6188715	0.53600103	0.99938111		Btg1	22.8495752	23.9837571	21.3112953	16.5451201	8.3240586	22.345036	29.8301805	4.56525506
Zfp110	6.68835713	-0.2530883	0.40897171	-0.6188406	0.53602139	0.99938111		Zfp110	6.72046328	11.7656167	6.04350166	6.20442003	11.653682	8.23238167	2.88679167	0
Znf41-ps	1.65503605	-0.250216	0.40434453	-0.6188189	0.53603569	NA		Znf41-ps	2.68818531	1.81009488	1.90847421	2.06814001	1.66481172	1.17605452	1.92452778	0
Zfp931	4.03686808	0.25477422	0.41180425	0.61867798	0.53612852	NA		Zfp931	2.01613899	4.52523719	6.04350166	0	0	5.88027262	11.5471667	2.28262753
Apbb1	215.550099	0.2177903	0.35203731	0.61865688	0.53614242	0.99938111		Apbb1	168.683628	124.896546	158.403359	326.766122	347.94565	108.197016	153.962222	335.546247
3110057O12Rik	4.72889417	0.25234715	0.40791158	0.61863199	0.53615882	0.99938111		3110057O12R	5.37637063	4.07271347	4.77118552	2.06814001	3.32962344	8.23238167	7.69811111	2.28262753
Prkab1	7.85929368	0.25246361	0.40815957	0.61854143	0.53621849	0.99938111		Prkab1	7.39250961	2.71514231	4.45310648	14.4769801	9.98887032	3.52816357	13.4716944	6.84788259
Sec24b	54.6985639	-0.1589797	0.25703535	-0.6185132	0.53623712	0.99938111		Sec24b	53.0916599	68.7836053	55.345752	53.7716403	53.2739751	62.3308898	38.4905555	52.5004332
Sox7	0.62084317	-0.1996789	0.32288512	-0.6184209	0.5362979	NA		Sox7	0.67204633	1.35757116	1.27231614	0	1.66481172	0	0	0
Rgs7bp	275.990148	0.17341662	0.28043315	0.61838845	0.53631931	0.99938111		Rgs7bp	309.813357	267.894042	300.902767	146.837941	196.447783	330.471321	365.660278	289.893696
Slc5a5	30.557691	0.21973708	0.35537126	0.61833104	0.53635715	0.99938111		Slc5a5	20.1613899	20.8160911	24.8101647	45.4990802	19.9777406	17.6408179	38.4905555	57.0656883
Nck1	10.5990887	0.2431717	0.39335376	0.61820102	0.53644283	0.99938111		Nck1	10.0806949	10.8605693	13.0412404	2.06814001	3.32962344	17.6408179	16.3584861	11.4131377
Rpl31	5.99305959	-0.2524386	0.40840733	-0.6181049	0.53650618	0.99938111		Rpl31	6.72046328	3.16766603	5.72542262	12.4088401	6.65924688	3.52816357	2.88679167	6.84788259
Tlr9	1.2389764	-0.2113665	0.34207512	-0.617895	0.53664458	NA		Tlr9	0.67204633	2.26261859	0	4.13628002	1.66481172	1.17605452	0	0
Rapgef3	15.6357732	-0.2505077	0.40545777	-0.6178391	0.5366814	0.99938111		Rapgef3	16.8011582	19.4585199	11.7689243	22.7495401	31.6314227	7.05632714	1.92452778	13.6957652
Ap1s3	1.7240296	-0.2441814	0.39527325	-0.6177535	0.53673784	NA		Ap1s3	1.34409266	3.62018975	1.59039517	2.06814001	0	0	2.88679167	2.28262753
Cnnm2	20.027405	0.23076589	0.37369677	0.61752176	0.53689063	0.99938111		Cnnm2	8.06455594	21.2686148	23.8559276	16.5451201	21.6425524	28.2253086	8.660375	31.9567854
Pycr1	1.84777906	0.21975007	0.35594417	0.61737229	0.53698919	NA		Pycr1	4.7043243	0	0.31807903	0	6.65924688	1.17605452	1.92452778	0
Gnao1	770.543925	-0.13644	0.22103829	-0.6172685	0.53705764	0.99938111		Gnao1	858.875208	800.514459	768.160868	808.642744	835.735484	543.33719	617.773416	931.312033
Tubgcp4	28.5745121	0.17215304	0.27892025	0.61721242	0.53709463	0.99938111		Tubgcp4	26.8818531	31.6766603	25.7644018	20.6814001	33.2962344	31.7534722	28.8679167	29.6741579
Cpne7	32.4616229	0.22357681	0.36230783	0.61709074	0.53717487	0.99938111		Cpne7	21.5054825	21.7211385	22.9016905	51.7035003	54.9387868	15.2887088	25.981125	45.6525506
Stmn4	62.0034053	0.15110764	0.24489144	0.61703929	0.53720881	0.99938111		Stmn4	50.4034746	60.6381783	69.9773876	51.7035003	64.9276571	58.8027262	77.943375	61.6309433
Vstm2b	31.8236639	0.19694338	0.31931774	0.61676304	0.53739103	0.99938111		Vstm2b	29.5700384	33.9392789	32.4440615	18.6132601	28.3017992	51.7463991	21.1698055	38.804668
Lrrc32	1.05453323	-0.2214461	0.3591648	-0.6165584	0.53752607	NA		Lrrc32	0.67204633	2.71514231	1.59039517	0	0	1.17605452	0	2.28262753
Zfr2	42.4274178	0.23825171	0.38642988	0.61654576	0.53753438	0.99938111		Zfr2	34.2743627	20.3635674	23.8559276	72.3849004	86.5702095	24.697145	20.2075417	57.0656883
Scube3	1.85428744	-0.2447183	0.39697291	-0.616461	0.53759032	NA		Scube3	3.36023164	1.35757116	3.81694841	0	1.66481172	2.35210905	0	2.28262753
Dnajc19	4.92970637	-0.2566604	0.41637073	-0.6164228	0.5376155	0.99938111		Dnajc19	2.68818531	4.52523719	2.54463228	14.4769801	3.32962344	1.17605452	3.84905555	6.84788259
Immp1l	1.8914468	0.24726621	0.40134877	0.61608813	0.53783636	NA		Immp1l	2.01613899	2.71514231	0.9542371	0	0	2.35210905	4.81131944	2.28262753
2610316D01Rik	2.8949687	0.24971846	0.40533826	0.61607423	0.53784553	NA		2610316D01Ri	2.01613899	4.97776091	1.90847421	0	0	2.35210905	9.62263889	2.28262753
Creb3l2	6.42226459	-0.2564456	0.41626769	-0.6160593	0.53785542	0.99938111		Creb3l2	2.68818531	11.313093	6.04350166	10.3407001	11.653682	7.05632714	0	2.28262753
Sfswap	14.018213	0.20701725	0.33605034	0.61603049	0.5378744	0.99938111		Sfswap	12.7688802	11.7656167	14.6316356	12.4088401	9.98887032	17.6408179	19.2452778	13.6957652
Uprt	4.28601867	0.25377147	0.41196139	0.61600789	0.53788931	NA		Uprt	2.01613899	4.07271347	4.45310648	4.13628002	4.99443516	2.35210905	7.69811111	4.56525506
Sc5d	24.8969177	-0.2488151	0.40393863	-0.6159725	0.53791267	0.99938111		Sc5d	20.1613899	54.75537	27.354797	10.3407001	3.32962344	41.1619083	19.2452778	22.8262753
Arf1	229.802056	0.11646846	0.18909062	0.61593991	0.53793418	0.99938111		Arf1	256.049651	205.445768	212.794874	204.745861	214.760712	231.682741	254.99993	257.936911
Cetn3	24.6128547	0.2562765	0.41609238	0.6159125	0.53795227	0.99938111		Cetn3	30.9141311	21.7211385	25.1282437	4.13628002	6.65924688	43.5140174	62.5471528	2.28262753
Pkib	12.4594116	-0.2329676	0.3782957	-0.6158348	0.53800358	0.99938111		Pkib	12.0968339	21.2686148	11.4508452	10.3407001	6.65924688	5.88027262	18.2830139	13.6957652
Mtx2	20.8472529	-0.233175	0.37867439	-0.6157665	0.53804861	0.99938111		Mtx2	31.5861774	17.1959013	30.5355873	14.4769801	13.3184938	15.2887088	37.5282917	6.84788259
Sep-01	0.77199276	0.19748471	0.32075031	0.61569608	0.53809513	NA		Sep-01	0	0.90504744	0.63615807	0	0	2.35210905	0	2.28262753
Sostdc1	1.27000832	0.23237252	0.37748797	0.61557597	0.53817442	NA		Sostdc1	1.34409266	0.45252372	1.59039517	0	0	3.52816357	0.96226389	2.28262753
Fiz1	19.3639841	-0.2034413	0.33051675	-0.6155248	0.53820818	0.99938111		Fiz1	20.8334362	19.4585199	17.1762679	26.8858201	26.6369875	14.1126543	11.5471667	18.2610202
Hps5	3.95178654	0.25470294	0.41393051	0.61532778	0.53833828	NA		Hps5	4.03227797	5.43028463	2.86271131	0	4.99443516	5.88027262	3.84905555	4.56525506
Efcab4a	1.35523783	-0.2060272	0.33482821	-0.6153222	0.53834193	NA		Efcab4a	1.34409266	0.45252372	0	6.20442003	1.66481172	1.17605452	0	0
Abcb1a	4.60511083	-0.2470988	0.40159247	-0.6152974	0.53835832	NA		Abcb1a	1.34409266	13.5757116	6.67965972	0	8.3240586	2.35210905	0	4.56525506
Asb11	0.63147791	-0.1969503	0.32020761	-0.6150706	0.53850807	NA		Asb11	1.34409266	0.45252372	1.59039517	0	1.66481172	0	0	0
Adamtsl2	3.09139994	-0.2537909	0.4127408	-0.6148917	0.53862621	NA		Adamtsl2	3.36023164	0.90504744	2.86271131	8.27256004	1.66481172	1.17605452	1.92452778	4.56525506
Slc25a17	15.970211	0.20137456	0.32755682	0.61477748	0.53870168	0.99938111		Slc25a17	12.7688802	16.2908539	14.9497146	14.4769801	14.9833055	12.9365998	23.0943333	18.2610202
Dcaf10	21.4043089	-0.2023213	0.32910657	-0.6147593	0.53871369	0.99938111		Dcaf10	15.4570656	17.648425	27.990955	33.0902402	14.9833055	19.9929269	19.2452778	22.8262753
Mtif2	14.8395281	0.23855809	0.38808247	0.6147098	0.53874639	0.99938111		Mtif2	12.0968339	15.3858064	16.5401098	8.27256004	3.32962344	17.6408179	31.7547083	13.6957652
Sytl4	1.5745336	-0.2099548	0.34157196	-0.6146723	0.53877114	NA		Sytl4	0	0.90504744	0.31807903	8.27256004	0	1.17605452	1.92452778	0
Ero1lb	9.29765458	-0.2447493	0.39818723	-0.614659	0.53877997	0.99938111		Ero1lb	4.7043243	9.95552182	14.6316356	12.4088401	1.66481172	10.5844907	6.73584722	13.6957652
Cdo1	31.9310366	-0.1928546	0.31388921	-0.6144034	0.53894882	0.99938111		Cdo1	38.3066407	31.2241366	28.6271131	39.2946602	18.3129289	19.9929269	31.7547083	47.9351782
Gabrb1	26.3246908	-0.2376055	0.38672735	-0.6144005	0.53895073	0.99938111		Gabrb1	16.8011582	48.4200379	34.3525357	18.6132601	18.3129289	47.042181	20.2075417	6.84788259
Pecam1	3.94734361	-0.2492963	0.40581353	-0.6143125	0.53900882	NA		Pecam1	0.67204633	12.2181404	2.22655324	4.13628002	6.65924688	4.7042181	0.96226389	0
Cebpa	4.14860059	-0.2557354	0.41632955	-0.614262	0.53904223	NA		Cebpa	4.03227797	6.33533207	5.72542262	2.06814001	8.3240586	1.17605452	0.96226389	4.56525506
Kif13b	5.05720843	-0.2528628	0.41169874	-0.6141938	0.53908726	0.99938111		Kif13b	5.37637063	7.2403795	5.08926455	4.13628002	4.99443516	3.52816357	0.96226389	9.13051012
Dph5	4.9721046	0.25115227	0.40893378	0.61416365	0.53910719	0.99938111		Dph5	7.39250961	2.26261859	4.77118552	2.06814001	4.99443516	4.7042181	6.73584722	6.84788259
Dnlz	34.1342074	-0.2103401	0.34252897	-0.6140797	0.53916264	0.99938111		Dnlz	32.2582238	33.9392789	29.2632712	53.7716403	51.6091633	16.4647633	19.2452778	36.5220405
D3Ertd254e	23.6950055	0.22823937	0.37168574	0.61406545	0.53917208	0.99938111		D3Ertd254e	25.5377605	28.961518	23.2197695	6.20442003	18.3129289	36.4576902	39.4528194	11.4131377
Fadd	2.53814484	-0.2544569	0.41444716	-0.613967	0.53923716	NA		Fadd	4.03227797	2.71514231	3.18079035	2.06814001	4.99443516	2.35210905	0.96226389	0
Mmadhc	15.2901079	-0.2221145	0.36209785	-0.6134102	0.53960517	0.99938111		Mmadhc	10.7527413	19.4585199	24.8101647	12.4088401	11.653682	12.9365998	21.1698055	9.13051012
Deaf1	19.5529131	-0.2116782	0.34526804	-0.6130836	0.53982107	0.99938111		Deaf1	20.1613899	22.1736622	18.448584	22.7495401	19.9777406	9.40843619	11.5471667	31.9567854

GeneID	Base mean	log2(FC)	StdErr	Wald-Stats	P-value	P-adj	GeneID	Normalised expression for Chow#1	Normalised expression for Chow#2	Normalised expression for Chow#3	Normalised expression for Chow#4	Normalised expression for HFD#1	Normalised expression for HFD#2	Normalised expression for HFD#3	Normalised expression for HFD#4
Dtnb	44.1907269	0.15877511	0.25900773	0.613013	0.53986774	0.99938111	Dtnb	43.6830113	45.7048956	41.9864326	31.0221002	48.2795399	41.1619083	42.3396111	59.3483158
Ccdc126	4.13137813	-0.254733	0.41554391	-0.6130111	0.53986901	NA	Ccdc126	4.7043243	5.43028463	7.31581779	2.06814001	1.66481172	7.05632714	4.81131944	0
Fam118a	16.0970267	0.20222257	0.32989766	0.61298577	0.53988575	0.99938111	Fam118a	18.8172972	14.0282353	12.0870033	14.4769801	11.653682	21.1689814	18.2830139	18.2610202
Ndufc2	56.5710479	-0.2052705	0.33490339	-0.6129244	0.53992635	0.99938111	Ndufc2	42.3389187	53.3977988	45.803381	107.543281	44.9499164	28.2253086	66.3962083	63.9135709
N28178	116.504859	0.14932451	0.24368229	0.6127836	0.54001943	0.99938111	N28178	117.608107	121.276357	120.233875	76.5211804	149.833055	135.24627	97.1886527	114.131377
Ces5a	0.58975525	0.18320973	0.29905189	0.61263525	0.54011755	NA	Ces5a	0	0.45252372	0.31807903	0	1.66481172	0	0	2.28262753
Fkbp11	0.58975525	0.18320973	0.29905189	0.61263525	0.54011755	NA	Fkbp11	0	0.45252372	0.31807903	0	1.66481172	0	0	2.28262753
Prss50	0.58975525	0.18320973	0.29905189	0.61263525	0.54011755	NA	Prss50	0	0.45252372	0.31807903	0	1.66481172	0	0	2.28262753
Mmp9	1.22655744	-0.2313156	0.37758133	-0.6126245	0.54012464	NA	Mmp9	2.68818531	2.26261859	1.27231614	0	1.66481172	0	1.92452778	0
Ndst4	23.611042	-0.2542687	0.4150651	-0.6125996	0.54014111	0.99938111	Ndst4	30.2420848	39.8220873	37.5333261	2.06814001	6.65924688	45.8661264	22.1320694	4.56525506
Pcdhb7	4.037842	0.25312625	0.41326073	0.61250981	0.54020051	NA	Pcdhb7	2.01613899	6.78785578	3.49886938	0	4.99443516	11.7605452	0.96226389	2.28262753
Lpgat1	194.605564	0.21681799	0.35408677	0.61233009	0.54031938	0.99938111	Lpgat1	154.570656	244.362808	201.662108	101.338861	143.173808	390.450102	229.981069	91.3051012
Rrp15	2.65126204	0.24124107	0.39407352	0.61217276	0.54042346	NA	Rrp15	2.68818531	0	1.90847421	4.13628002	0	1.17605452	6.73584722	4.56525506
Lsm7	3.74191984	-0.2534408	0.41409382	-0.612037	0.54051325	NA	Lsm7	4.7043243	1.35757116	3.49886938	8.27256004	3.32962344	0	1.92452778	6.84788259
Dnajc3	25.5255926	-0.2536011	0.41436046	-0.6120301	0.54051786	0.99938111	Dnajc3	22.1775288	61.9957495	29.8994292	4.13628002	6.65924688	49.39429	23.0943333	6.84788259
Pi4k2a	76.4583981	0.19935666	0.32587882	0.6117509	0.54070258	0.99938111	Pi4k2a	71.9089571	47.0624668	50.5745665	111.679561	96.5590798	59.9787807	52.9245139	120.979259
9130024F11Rik	6.60807276	0.24986908	0.40846704	0.61172397	0.5407204	0.99938111	9130024F11Ri	8.73660227	4.07271347	4.45310648	4.13628002	8.3240586	2.35210905	4.81131944	15.9783927
Fam3c	46.9963739	0.22508026	0.36799014	0.61164753	0.54077098	0.99938111	Fam3c	39.6507334	56.5654649	52.4830407	18.6132601	33.2962344	104.668853	43.301875	27.3915304
Tmcc3	31.1867498	-0.1712439	0.28003399	-0.6115111	0.54086127	0.99938111	Tmcc3	29.5700384	38.4645161	31.4898244	35.1583802	23.3073641	31.7534722	34.6415	25.1089028
Top3a	3.20297702	0.25446735	0.41620196	0.61140353	0.54093247	NA	Top3a	2.01613899	1.81009488	2.86271131	4.13628002	4.99443516	2.35210905	2.88679167	4.56525506
Zik1	13.550253	0.21839849	0.35750126	0.61090272	0.54126399	0.99938111	Zik1	12.7688802	13.1231878	13.0412404	10.3407001	6.65924688	24.697145	16.3584861	11.4131377
Tmem238	1.31206917	0.22309155	0.36547838	0.61040971	0.54159044	NA	Tmem238	0	0.45252372	1.27231614	2.06814001	0	1.17605452	0.96226389	4.56525506
Lsm14a	37.3525488	0.2184579	0.35789399	0.61039834	0.54159797	0.99938111	Lsm14a	41.6668724	42.0847059	34.3525357	16.5451201	28.3017992	43.5140174	74.0943194	18.2610202
Ambra1	59.93702	0.17144388	0.28088611	0.61036795	0.54161809	0.99938111	Ambra1	55.1077989	41.6321821	49.9384084	78.5893204	81.5757743	47.042181	54.8490416	70.7614535
Lsm8	9.69853442	0.23314983	0.38217749	0.6100564	0.54182445	0.99938111	Lsm8	4.03227797	9.95552182	7.31581779	14.4769801	6.65924688	14.1126543	9.62263889	11.4131377
Rc3h1	86.9588774	0.16110411	0.26411033	0.6099879	0.54186982	0.99938111	Rc3h1	65.1884939	100.007742	82.0643909	80.6574604	78.2461509	108.197016	117.396194	63.9135709
Zfp9	29.1627666	0.20872237	0.34223926	0.60987268	0.54194615	0.99938111	Zfp9	34.9464091	28.961518	25.7644018	16.5451201	14.9833055	41.1619083	48.1131944	22.8262753
Polr2b	48.7476018	0.21794897	0.35740995	0.60980107	0.54199359	0.99938111	Polr2b	58.4680306	47.0624668	51.2107246	18.6132601	41.620293	76.4435441	76.0188472	20.5436478
Clk1	10.5855834	0.22051794	0.36181974	0.60946906	0.54221357	0.99938111	Clk1	12.0968339	8.59795066	9.86045007	8.27256004	8.3240586	15.2887088	15.3962222	6.84788259
1700019D03Rik	2.53287779	0.25244707	0.41428522	0.60935573	0.54228868	NA	1700019D03Ri	3.36023164	1.81009488	1.27231614	2.06814001	1.66481172	5.88027262	1.92452778	2.28262753
Iglon5	22.1618471	-0.1999688	0.32818865	-0.6093106	0.5423186	0.99938111	Iglon5	17.4732045	22.1736622	23.2197695	33.0902402	24.9721758	16.4647633	12.5094306	27.3915304
2810008D09Rik	1.74885975	-0.2371956	0.3894012	-0.609129	0.54243893	NA	2810008D09Ri	2.68818531	0.45252372	1.59039517	4.13628002	1.66481172	1.17605452	0	2.28262753
Sirt7	4.57670548	-0.2522149	0.41418585	-0.6089413	0.54256335	NA	Sirt7	6.04841696	3.62018975	5.40734359	6.20442003	4.99443516	0	5.77358333	4.56525506
Snx21	19.5597553	-0.2062293	0.33868633	-0.6089094	0.54258447	0.99938111	Snx21	18.8172972	18.5534725	18.766663	28.9539601	14.9833055	9.40843619	17.32075	29.6741579
Reln	97.6343574	-0.2260806	0.37129257	-0.6089015	0.54258972	0.99938111	Reln	101.478996	178.746869	119.279638	31.0221002	128.190502	115.253343	50.0377222	57.0656883
Minpp1	22.6896901	-0.198362	0.32585821	-0.6087373	0.54269858	0.99938111	Minpp1	22.8495752	29.4140417	24.8101647	22.7495401	11.653682	29.4013631	26.9433889	13.6957652
Tbc1d15	9.82896302	-0.2276912	0.37404135	-0.6087327	0.5427016	0.99938111	Tbc1d15	13.4409266	10.8605693	12.7231614	6.20442003	9.98887032	14.1126543	6.73584722	4.56525506
Baiap2l1	0.50193677	0.18168084	0.29850953	0.60862658	0.54277198	NA	Baiap2l1	0	0.45252372	0.31807903	0	0	0	0.96226389	2.28262753
Cmtm7	0.33488462	-0.1359851	0.22345896	-0.6085463	0.54282522	NA	Cmtm7	0	0.45252372	2.22655324	0	0	0	0	0
Tsen54	2.46275463	0.24958682	0.41038725	0.60817391	0.54307214	NA	Tsen54	2.68818531	1.35757116	1.27231614	2.06814001	3.32962344	1.17605452	0.96226389	6.84788259
Ctcf	39.2997382	0.18358521	0.30198355	0.60793117	0.54323313	0.99938111	Ctcf	42.3389187	43.442277	37.8514051	20.6814001	36.6258578	37.6337448	61.5848889	34.239413
Nudt12	4.19015935	0.25168867	0.41402227	0.60791094	0.54324655	NA	Nudt12	4.7043243	4.97776091	3.81694841	0	1.66481172	7.05632714	6.73584722	4.56525506
Mtss1l	450.818137	-0.2200883	0.36204772	-0.6078985	0.54325477	0.99938111	Mtss1l	340.727488	400.483491	311.717454	934.799285	584.348914	252.851723	197.264097	584.352648
A830082N09Rik	27.2555956	0.2384447	0.39224674	0.60789467	0.54325734	0.99938111	A830082N09Ri	26.2098068	27.1514231	30.2175083	12.4088401	13.3184938	57.6266717	44.2641389	6.84788259
Cdkn3	0.77270926	0.2120454	0.34896078	0.60764825	0.54342079	NA	Cdkn3	0.67204633	0.45252372	0.63615807	0	0	1.17605452	0.96226389	2.28262753
Ifitm1	0.57486929	-0.1913048	0.31496026	-0.6073933	0.54358992	NA	Ifitm1	2.01613899	0.45252372	0.9542371	0	0	1.17605452	0	0
Slc26a7	0.32873591	-0.1384556	0.22799128	-0.6072848	0.54366191	NA	Slc26a7	0	1.35757116	1.27231614	0	0	0	0	0
Tmem203	7.84879726	0.24959199	0.41118479	0.60700686	0.54384636	0.99938111	Tmem203	4.7043243	3.16766603	5.08926455	14.4769801	8.3240586	5.88027262	2.88679167	18.2610202
Abcb9	22.4243692	-0.2358777	0.38873216	-0.6067871	0.54399221	0.99938111	Abcb9	17.4732045	24.8888045	20.6751372	37.2265202	29.966611	21.1689814	2.88679167	25.1089028
Fhl4	0.89501959	-0.21225	0.34983351	-0.6067171	0.54403866	NA	Fhl4	0.67204633	0.90504744	1.59039517	2.06814001	0	0	1.92452778	0
Unc5b	20.0649982	0.24810095	0.40910374	0.60644996	0.544216	0.99938111	Unc5b	8.06455594	20.3635674	7.63389683	33.0902402	29.966611	16.4647633	3.84905555	41.0872956
Zfp251	35.4659363	0.16095871	0.26544088	0.60638251	0.54426078	0.99938111	Zfp251	38.3066407	30.3190892	34.9886938	28.9539601	36.6258578	44.6900719	35.6037639	34.239413
Mfsd11	13.4636187	-0.2181361	0.35984352	-0.6061972	0.54438379	0.99938111	Mfsd11	11.4247876	18.1009488	15.9039517	12.4088401	11.653682	16.4647633	5.77358333	15.9783927
Galc	10.8143257	-0.2299002	0.37927242	-0.6061612	0.54440772	0.99938111	Galc	14.7850192	16.7433776	12.0870033	4.13628002	8.3240586	15.2887088	10.5849028	4.56525506
Fbxw11	136.518894	0.15475591	0.25536807	0.6060112	0.54450732	0.99938111	Fbxw11	113.575829	120.371309	115.780769	165.451201	184.794101	95.2604165	146.264111	150.653417
Zbtb8b	5.9538386	0.24864041	0.4102995	0.60599735	0.54451651	0.99938111	Zbtb8b	2.68818531	4.52523719	2.86271131	12.4088401	3.32962344	8.23238167	6.73584722	6.84788259
Get4	28.005761	0.2027578	0.33463505	0.60590723	0.54457636	0.99938111	Get4	21.5054825	20.3635674	20.0389792	41.3628002	29.966611	18.8168724	24.0565972	47.9351782
Gm4980	6.45202528	0.2506024	0.41359958	0.60590585	0.54457727	0.99938111	Gm4980	2.68818531	11.7656167	5.72542262	0	6.65924688	4.7042181	8.660375	11.4131377
Dapk3	19.7121896	0.2086064	0.34429425	0.60589569	0.54458402	0.99938111	Dapk3	21.5054825	17.1959013	14.6316356	16.5451201	26.6369875	10.5844907	16.3584861	34.239413
Zfp280c	9.8930851	0.24310036	0.40144506	0.60556321	0.54480484	0.99938111	Zfp280c	8.73660227	13.1231878	8.58813393	4.13628002	9.98887032	8.23238167	24.0565972	2.28262753
Serpine1	0.82043783	0.18073446	0.29849605	0.60548361	0.54485771	NA	Serpine1	0	0.90504744	0.9542371	0	0	4.7042181	0	0
Mrpl19	20.3825833	0.20715166	0.34220618	0.60534167	0.544952	0.99938111	Mrpl19	24.1936678	23.0787097	19.7209001	4.13628002	23.3073641	22.345036	21.1698055	25.1089028
Cxcl16	1.01671468	0.22244899	0.36751342	0.60528127	0.54499213	NA	Cxcl16	0.67204633	1.35757116	0.63615807	0	3.32962344	1.17605452	0.96226389	0
Relb	3.05657889	0.25178671	0.41606421	0.6051631	0.54507063	NA	Relb	3.36023164	1.81009488	2.54463228	2.06814001	8.3240586	1.17605452	2.88679167	2.28262753
Ehd2	3.18069598	0.25176417	0.41611772	0.60503112	0.54515832	NA	Ehd2	2.01613899	2.71514231	1.90847421	4.13628002	8.3240586	1.17605452	2.88679167	2.28262753
Snx8	25.5130801	0.23632275	0.3906911	0.60488387	0.54525616	0.99938111	Snx8	14.1129729	14.0282353	15.2677937	47.5672202	28.3017992	11.7605452	18.2830139	54.7830607
Fkbp3	87.5440626	0.24668182	0.40784766	0.60483814	0.54528655	0.99938111	Fkbp3	104.839227	76.0239848	101.149133	18.6132601	26.6369875	142.302597	201.113153	29.6741579
Pacrgl	3.99920579	-0.2508062	0.41471112	-0.6047732	0.54532968	NA	Pacrgl	6.04841696	6.33533207	3.49886938	4.13628002	0	2.35210905	9.62263889	0
Rpl36a	2.52423556	-0.2512057	0.41544804	-0.6046622	0.5454035	NA	Rpl36a	2.68818531	4.07271347	3.49886938	2.06814001	1.66481172	2.35210905	3.84905555	0
C2cd3	36.4454108	-0.2021291	0.33430241	-0.6046295	0.54542519	0.99938111	C2cd3	40.3227797	49.3250854	35.9429309	33.0902402	44.9499164	51.7463991	20.2075417	15.9783927
Rbm48	5.50065691	-0.246371	0.4075494	-0.6045182	0.54549914	0.99938111	Rbm48	9.4086486	7.69290322	4.13502745	4.13628002	1.66481172	4.7042181	7.69811111	4.56525506
Nyap2	37.6320783	0.16503888	0.27309988	0.60431693	0.54563295	0.99938111	Nyap2	45.6991503	35.2968501	32.7621406	24.8176801	39.9554813	36.4576902	40.4150833	45.6525506
Sec14l1	146.905535	0.12717881	0.21046912	0.60426352	0.54566845	0.99938111	Sec14l1	151.88247	124.896546	145.680198	134.429101	148.168243	137.598379	138.566	194.02334
Celf5	336.967163	0.1809636	0.29952664	0.6041653	0.54573375	0.99938111	Celf5	287.635829	283.279848	263.369441	413.628002	471.141717	226.978523	233.830125	515.873822
Eya2	1.83473704	0.24149546	0.39974464	0.60412433	0.54576098	NA	Eya2	0.67204633	0.90504744	0.9542371	4.13628002	1.66481172	1.17605452	2.88679167	2.28262753
Zscan26	47.4164421	0.20523878	0.33975779	0.60407381	0.54579457	0.99938111	Zscan26	34.9464091	41.1796584	49.3022503	49.6353602	23.3073641	75.2674895	76.0188472	29.6741579
Mfsd12	16.0097275	0.24389621	0.40382415	0.60396639	0.54586599	0.99938111	Mfsd12	11.4247876	13.5757116	9.224292	20.6814001	26.6369875	9.40843619	2.88679167	34.239413
Clmp	15.2801185	-0.2263416	0.37481489	-0.6038756	0.54592632	0.99938111	Clmp	12.0968339	25.3413283	19.0847421	10.3407001	8.3240586	24.697145	8.660375	13.6957652
Tnfaip1	41.6761448	0.18690957	0.30951977	0.60386958	0.54593035	0.99938111	Tnfaip1	43.010965	41.1796584	39.1237212	26.8858201	61.5980337	28.2253086	31.7547083	61.6309433
Limk1	89.4474907	0.21600888	0.35771166	0.60386313	0.54593464	0.99938111	Limk1	61.1562159	48.8725616	53.4372778	161.314921	103.218327	71.739326	60.622625	155.218672
Polr1c	13.6429136	0.234969	0.38915338	0.60379535	0.54597971	0.99938111	Polr1c	8.06455594	5.43028463	12.4050823	24.8176801	16.6481172	8.23238167	22.1320694	11.4131377
Fam53b	18.9556295	-0.2053672	0.34019526	-0.6036746	0.54606003	0.99938111	Fam53b	20.8334362	23.0787097	17.8124259	20.6814001	31.6314227	11.7605452	14.4339583	11.4131377
Grsf1	73.0062132	0.17654788	0.29257545	0.60342686	0.54622476	0.99938111	Grsf1	79.9735131	67.4260341	72.2039408	51.7035003	41.620293	96.436471	103.9245	70.7614535
Zbtb24	6.992661	-0.2330041	0.38613503	-0.6034265	0.54622503	0.99938111	Zbtb24	8.06455594	6.78785578	8.90621297	8.27256004	6.65924688	8.23238167	6.73584722	2.28262753
Hist1h2an	0.69548228	0.20964501	0.34748566	0.60331991	0.54629589	NA	Hist1h2an	0.67204633	0.45252372	0.63615807	0	1.66481172	1.17605452	0.96226389	0
Sde2	20.754915	0.21310579	0.35324937	0.60327294	0.54632713	0.99938111	Sde2	8.73660227	15.8383302	18.130505	35.1583802	24.9721758	17.6408179	25.0188611	20.5436478
Parva	54.193788	0.14850251	0.24622172	0.60312513	0.54642545	0.99938111	Parva	59.1400769	47.0624668	50.8926455	47.5672202	56.6035985	48.2182355	69.283	54.7830607
Mtf1	33.5394701	-0.1701633	0.28214622	-0.6031034	0.54643993	0.99938111	Mtf1	34.9464091	38.9170398	42.9406697	24.8176801	38.2906696	30.5774176	32.7169722	25.1089028
Itm2c	425.111743	0.22187352	0.3679771	0.60295469	0.54653883	0.99938111	Itm2c	233.872122	381.024971	308.218584	599.760603	625.969207	322.23894	149.150903	780.658616
Gnpda2	11.3020524	-0.2250816	0.37331152	-0.6029324	0.54655366	0.99938111	Gnpda2	13.4409266	15.8383302	13.9954775	6.20442003	3.32962344	11.7605452	14.4339583	11.4131377
Nup205	14.9881854	0.2042617	0.33881454	0.6028717	0.54659404	0.99938111	Nup205	12.7688802	15.3858064	11.7689243	16.5451201	18.3129289	19.9929269	18.2830139	6.84788259
Usp15	42.6453538	0.22885346	0.37961529	0.60285629	0.5466043	0.99938111	Usp15	53.7637063	43.8948007	46.439539	6.20442003	46.6147282	61.1548353	62.5471528	20.5436478
Ints10	12.7344918	-0.2339538	0.38814264	-0.6027521	0.54667364	0.99938111	Ints10	16.8011582	14.480759	16.5401098	10.3407001	8.3240586	19.9929269	15.3962222	0
Sema6c	22.0071612	0.25047067	0.41559048	0.60268626	0.54671742	0.99938111	Sema6c	9.4086486	10.4080455	7.31581779	47.5672202	24.9721758	7.05632714	7.69811111	61.6309433
Tmem79	0.66412893	0.16608338	0.27558514	0.60265724	0.54673674	NA	Tmem79	0	0	0	2.06814001	0	0	0.96226389	2.28262753
Ubtd2	19.0687881	-0.2068274	0.34320122	-0.6026419	0.54674694	0.99938111	Ubtd2	32.2582238	18.1009488	21.9474534	10.3407001	16.6481172	16.4647633	23.0943333	13.6957652
Tmem144	1.69099209	-0.2348247	0.38976744	-0.602474	0.54685869	NA	Tmem144	0.67204633	4.52523719	2.86271131	0	3.32962344	1.17605452	0.96226389	0
Hint1	69.0482031	-0.1868839	0.31025203	-0.6023617	0.5469334	0.99938111	Hint1	86.6939764	68.3310816	71.2497037	72.3849004	29.966611	52.9224536	100.075444	70.7614535
Uap1l1	9.29231795	0.25019328	0.41540576	0.60228649	0.54698345	0.99938111	Uap1l1	3.36023164	3.16766603	2.54463228	24.8176801	13.3184938	7.05632714	8.660375	11.4131377
Acad9	20.4397534	0.22455704	0.37289647	0.60219674	0.54704319	0.99938111	Acad9	10.7527413	13.1231878	14.6316356	37.2265202	19.9777406	10.5844907	29.8301805	27.3915304
Ppwd1	3.25844466	-0.2506221	0.41626046	-0.6020801	0.54712082	NA	Ppwd1	6.72046328	2.71514231	3.49886938	2.06814001	3.32962344	3.52816357	1.92452778	2.28262753
Pm20d1	0.29346206	-0.1523637	0.253069	-0.6020638	0.54713167	NA	Pm20d1	0.67204633	1.35757116	0.31807903	0	0	0	0	0
Tgtp1	0.29346206	-0.1523637	0.253069	-0.6020638	0.54713167	NA	Tgtp1	0.67204633	1.35757116	0.31807903	0	0	0	0	0
Col4a1	4.63029784	0.25066009	0.4163669	0.60201733	0.5471626	NA	Col4a1	2.01613899	7.2403795	1.90847421	4.13628002	11.653682	5.88027262	1.92452778	2.28262753
Prpf18	30.6632226	0.17843015	0.29639392	0.6020034	0.54717187	0.99938111	Prpf18	32.2582238	20.8160911	24.1740066	39.2946602	38.2906696	29.4013631	33.6792361	27.3915304
Spcs2	35.9768158	-0.1787822	0.29701261	-0.6019347	0.54721761	0.99938111	Spcs2	40.3227797	49.7776091	30.2175083	35.1583802	29.966611	45.8661264	33.6792361	22.8262753
Gm8994	1.12898507	0.21538695	0.35782583	0.60193236	0.54721917	NA	Gm8994	0	0.45252372	1.27231614	2.06814001	0	2.35210905	2.88679167	0
Dock4	98.3396516	0.16668138	0.27692201	0.6019073	0.54723585	0.99938111	Dock4	112.903783	99.1026944	99.8768168	53.7716403	114.872009	130.542052	104.886764	70.7614535
E030013I19Rik	0.64213479	-0.1909813	0.31732315	-0.6018513	0.54727314	NA	E030013I19Ri	1.34409266	1.81009488	0.31807903	0	1.66481172	0	0	0
Actr1a	141.829376	0.17292301	0.28734329	0.60179938	0.54730769	0.99938111	Actr1a	136.425405	103.175408	124.686982	163.383061	214.760712	97.6125255	109.698083	184.89283
Wdr3	7.187098	0.23156455	0.38480315	0.60177405	0.54732455	0.99938111	Wdr3	8.73660227	6.78785578	5.72542262	4.13628002	6.65924688	7.05632714	11.5471667	6.84788259
Rnu11	0.69085276	0.16572042	0.27538667	0.60177357	0.54732487	NA	Rnu11	0	0	0	2.06814001	0	1.17605452	0	2.28262753

GeneID	Base mean	log2(FC)	StdErr	Wald-Stats	P-value	P-adj	GeneID	Normalised expression for Chow#1	Normalised expression for Chow#2	Normalised expression for Chow#3	Normalised expression for Chow#4	Normalised expression for HFD#1	Normalised expression for HFD#2	Normalised expression for HFD#3	Normalised expression for HFD#4
Diap1	36.7456439	0.18901554	0.31416601	0.60164222	0.54741232	0.99938111	Diap1	25.5377605	38.4645161	29.8994292	41.3628002	51.6091633	43.5140174	20.2075417	43.3699231
Zkscan8	21.4459615	0.19563777	0.32517695	0.6016348	0.54741726	0.99938111	Zkscan8	28.2259458	19.4585199	18.448584	12.4088401	18.3129289	24.697145	31.7547083	18.2610202
Cep89	3.63723262	0.24902365	0.41391577	0.60162881	0.54742125	NA	Cep89	4.03227797	3.62018975	2.22655324	2.06814001	4.99443516	4.7042181	2.88679167	4.56525506
Dcps	7.47508966	-0.2417967	0.40220909	-0.6011715	0.54772574	0.99938111	Dcps	6.72046328	4.97776091	6.67965972	16.5451201	6.65924688	2.35210905	6.73584722	9.13051012
Tcf19	2.95106561	-0.2366668	0.39369335	-0.6011449	0.54774347	NA	Tcf19	6.72046328	4.52523719	2.86271131	0	8.3240586	1.17605452	0	0
Gm2382	2.14992111	-0.2468936	0.41078324	-0.6010312	0.54781919	NA	Gm2382	3.36023164	4.07271347	2.86271131	0	1.66481172	2.35210905	2.88679167	0
Coro1b	22.6661013	0.1938848	0.32258695	0.60103113	0.54781926	0.99938111	Coro1b	21.5054825	18.1009488	19.4028211	22.7495401	23.3073641	18.8168724	16.3584861	41.0872956
Pdcd6	33.3522109	-0.1829475	0.30439123	-0.6010274	0.54782173	0.99938111	Pdcd6	33.6023164	38.0119924	38.8056422	33.0902402	16.6481172	25.8731995	44.2641389	36.5220405
Ipmk	32.514841	-0.1849744	0.30777344	-0.6010082	0.54783451	0.99938111	Ipmk	35.6184554	42.5372296	34.3525357	28.9539601	28.3017992	35.2816357	41.3773472	13.6957652
Pfkp	131.796752	0.16067348	0.267421	0.60082598	0.5479559	0.99938111	Pfkp	150.538378	97.7451233	106.238398	140.633521	204.771842	116.429398	119.320722	118.696632
Mrps9	11.387059	-0.2328229	0.38754956	-0.6007563	0.54800229	0.99938111	Mrps9	10.7527413	12.6706641	10.4966081	18.6132601	6.65924688	3.52816357	19.2452778	9.13051012
Rmnd5a	116.391508	0.15621667	0.26006003	0.60069466	0.54804338	0.99938111	Rmnd5a	115.591968	119.466262	129.776246	70.3167603	96.5590798	122.309671	160.698069	116.414004
Tasp1	24.2452981	0.19773373	0.32918096	0.60068398	0.54805049	0.99938111	Tasp1	22.1775288	15.3858064	20.0389792	33.0902402	23.3073641	15.2887088	32.7169722	31.9567854
Gm14169	3.36195254	0.24988011	0.41604754	0.60060471	0.5481033	NA	Gm14169	2.68818531	4.52523719	2.22655324	2.06814001	1.66481172	4.7042181	6.73584722	2.28262753
Bdnf	12.1859214	-0.2399604	0.39965904	-0.6004128	0.54823114	0.99938111	Bdnf	12.7688802	19.9110436	19.4028211	2.06814001	6.65924688	21.1689814	8.660375	6.84788259
Aph1a	15.1630355	-0.2429693	0.40467494	-0.600406	0.54823566	0.99938111	Aph1a	7.39250961	15.3858064	11.1327662	35.1583802	16.6481172	4.7042181	5.77358333	25.1089028
1500004A13Rik	23.7258291	-0.180616	0.30088898	-0.6002746	0.54832326	0.99938111	1500004A13Ri	24.8657142	30.7716129	27.990955	16.5451201	19.9777406	24.697145	22.1320694	22.8262753
Pcyox1	56.4176163	-0.1947271	0.32440603	-0.6002572	0.54833484	0.99938111	Pcyox1	51.7475673	90.0522201	60.7530956	41.3628002	38.2906696	87.0280348	43.301875	38.804668
Tex9	5.53431011	0.24621717	0.4102696	0.60013507	0.54841622	0.99938111	Tex9	4.7043243	3.16766603	6.04350166	6.20442003	1.66481172	10.5844907	9.62263889	2.28262753
Rabggtb	28.2604395	-0.2044305	0.34068826	-0.6000514	0.54847195	0.99938111	Rabggtb	38.978687	30.3190892	28.9451921	26.8858201	14.9833055	27.0492541	45.2264028	13.6957652
Igf2bp3	3.69860565	-0.2494776	0.41578605	-0.6000143	0.54849673	NA	Igf2bp3	6.04841696	6.78785578	2.86271131	2.06814001	1.66481172	8.23238167	1.92452778	0
1810012K16Rik	0.33377351	0.13394205	0.2232422	0.59998534	0.548516	NA	1810012K16Ri	0	0	0.31807903	0	0	2.35210905	0	0
5031434O11Rik	0.33377351	0.13394205	0.2232422	0.59998534	0.548516	NA	5031434O11R	0	0	0.31807903	0	0	2.35210905	0	0
Arhgef33	0.33377351	0.13394205	0.2232422	0.59998534	0.548516	NA	Arhgef33	0	0	0.31807903	0	0	2.35210905	0	0
Bves	0.33377351	0.13394205	0.2232422	0.59998534	0.548516	NA	Bves	0	0	0.31807903	0	0	2.35210905	0	0
Card11	0.33377351	0.13394205	0.2232422	0.59998534	0.548516	NA	Card11	0	0	0.31807903	0	0	2.35210905	0	0
Sdc1	0.33377351	0.13394205	0.2232422	0.59998534	0.548516	NA	Sdc1	0	0	0.31807903	0	0	2.35210905	0	0
Tnfrsf13c	0.33377351	0.13394205	0.2232422	0.59998534	0.548516	NA	Tnfrsf13c	0	0	0.31807903	0	0	2.35210905	0	0
Them4	17.238738	-0.2016573	0.33636184	-0.5995249	0.54882293	0.99938111	Them4	18.8172972	19.9110436	17.8124259	20.6814001	13.3184938	16.4647633	24.0565972	6.84788259
Prkra	10.9148986	-0.2193699	0.3659695	-0.5994212	0.54889206	0.99938111	Prkra	10.0806949	9.95552182	11.4508452	18.6132601	4.99443516	10.5844907	12.5094306	9.13051012
Zbtb42	1.54659828	-0.1886006	0.31463877	-0.5994194	0.54889325	NA	Zbtb42	0	0.45252372	0.31807903	8.27256004	3.32962344	0	0	0
Pigm	11.1843633	-0.221001	0.36871761	-0.5993774	0.54892124	0.99938111	Pigm	7.39250961	11.313093	15.5858727	14.4769801	6.65924688	9.40843619	8.660375	15.9783927
Agtr2	1.1104818	-0.218808	0.36514698	-0.5992327	0.54901775	NA	Agtr2	0.67204633	2.26261859	0.63615807	2.06814001	0	0	0.96226389	2.28262753
Ogdhl	59.2453997	0.21107685	0.35225017	0.59922427	0.54902334	0.99938111	Ogdhl	48.3873356	31.6766603	46.7576181	88.9300204	101.553515	30.5774176	46.1886666	79.8919636
Gm17769	0.72220611	0.20133143	0.33600292	0.59919548	0.54904254	NA	Gm17769	0.67204633	0.45252372	0.63615807	0	1.66481172	2.35210905	0	0
1810013L24Rik	63.8041062	0.19273597	0.32171693	0.59908557	0.54911582	0.99938111	1810013L24Ri	65.1884939	67.8785578	71.2497037	28.9539601	46.6147282	104.668853	77.943375	47.9351782
Utp6	27.4876703	-0.2128611	0.35532445	-0.5990613	0.54913202	0.99938111	Utp6	34.9464091	34.8443264	36.2610099	14.4769801	11.653682	39.9858538	31.7547083	15.9783927
Cmtr2	7.90183994	-0.2490808	0.41613393	-0.5985591	0.54946691	0.99938111	Cmtr2	4.03227797	6.78785578	5.40734359	22.7495401	0	12.9365998	6.73584722	4.56525506
Zfyve9	31.9616757	0.22395634	0.37424819	0.59841661	0.54956198	0.99938111	Zfyve9	26.2098068	31.2241366	43.8949068	12.4088401	18.3129289	57.6266717	50.0377222	15.9783927
Prr12	168.354156	0.23977247	0.40079656	0.59823985	0.5496799	0.99938111	Prr12	88.7101153	99.5552182	80.4739957	318.493562	347.94565	89.3801438	52.9245139	269.350049
Bst1	0.47276839	0.14312863	0.23933895	0.59801645	0.54982896	NA	Bst1	0	0.45252372	0	0	3.32962344	0	0	0
Tbxa2r	0.47276839	0.14312863	0.23933895	0.59801645	0.54982896	NA	Tbxa2r	0	0.45252372	0	0	3.32962344	0	0	0
Vwce	0.47276839	0.14312863	0.23933895	0.59801645	0.54982896	NA	Vwce	0	0.45252372	0	0	3.32962344	0	0	0
Map2k5	33.5620561	-0.2045103	0.34212824	-0.5977591	0.55000066	0.99938111	Map2k5	36.2905017	28.961518	27.354797	53.7716403	26.6369875	14.1126543	28.8679167	52.5004332
Mkrn2	19.6836997	0.21306028	0.35646139	0.59770928	0.55003393	0.99938111	Mkrn2	16.1291119	12.2181404	14.6316356	28.9539601	26.6369875	10.5844907	16.3584861	31.9567854
1700037C18Rik	2.17069053	0.23920248	0.40022095	0.59767606	0.5500561	NA	1700037C18Ri	1.34409266	0.90504744	1.90847421	2.06814001	3.32962344	0	0.96226389	6.84788259
Dll1	5.90358764	0.24193506	0.40480151	0.59766344	0.55006452	0.99938111	Dll1	4.7043243	3.62018975	4.13502745	8.27256004	9.98887032	3.52816357	3.84905555	9.13051012
Carm1	77.107563	-0.1785398	0.29874679	-0.5976293	0.55008729	0.99938111	Carm1	82.6616984	77.3815559	63.2977279	107.543281	96.5590798	38.8097993	61.5848889	89.0224737
Gdi2	136.138464	0.12650947	0.21170995	0.59756035	0.55013333	0.99938111	Gdi2	132.393127	112.225882	123.732744	155.110501	123.196067	149.358925	160.698069	132.392397
Nudt21	8.30422237	-0.2325936	0.38925658	-0.5975329	0.55015162	0.99938111	Nudt21	9.4086486	8.59795066	6.36158069	12.4088401	6.65924688	3.52816357	5.77358333	13.6957652
Rhbdd1	3.79668794	-0.248289	0.4155365	-0.5975143	0.55016404	NA	Rhbdd1	0.67204633	7.69290322	4.13502745	6.20442003	3.32962344	3.52816357	4.81131944	0
Fpgt	15.1633671	-0.2409707	0.40335758	-0.5974121	0.55023227	0.99938111	Fpgt	19.4893435	23.0787097	23.8559276	2.06814001	6.65924688	22.345036	19.2452778	4.56525506
Mettl24	0.85767444	0.20415287	0.3418005	0.59728663	0.55031603	NA	Mettl24	0.67204633	0	0.31807903	2.06814001	1.66481172	1.17605452	0.96226389	0
Zfp692	2.28564144	0.24040797	0.40250243	0.59728328	0.55031827	NA	Zfp692	0.67204633	3.16766603	0.9542371	2.06814001	3.32962344	3.52816357	0	4.56525506
Sbf1	155.915598	0.2272525	0.38052939	0.59720091	0.55037325	0.99938111	Sbf1	114.919922	97.7451233	94.1513942	248.176801	306.325357	87.0280348	61.5848889	237.393263
Plekhb2	279.258375	0.18043934	0.30217242	0.59714033	0.55041369	0.99938111	Plekhb2	187.500926	189.607438	223.92764	436.377542	309.65498	215.217978	272.32068	399.459818
Gm14326	18.3797736	0.21116597	0.35366815	0.59707375	0.55045814	0.99938111	Gm14326	12.0968339	17.1959013	17.8124259	4.13628002	0	50.5703445	45.2264028	0
Peli3	19.7943331	0.23098675	0.38694247	0.59695372	0.55053828	0.99938111	Peli3	13.4409266	10.4080455	12.7231614	35.1583802	39.9554813	9.40843619	14.4339583	22.8262753
Manf	15.4494592	-0.2460239	0.41224722	-0.5967874	0.55064934	0.99938111	Manf	12.0968339	31.6766603	24.8101647	2.06814001	3.32962344	23.5210905	19.2452778	6.84788259
Grik5	293.089796	0.23475732	0.39347659	0.59662335	0.55075888	0.99938111	Grik5	181.452509	205.898292	162.538387	479.808482	462.817658	136.422325	97.1886527	618.592061
Glt8d2	2.13803172	-0.236522	0.39646304	-0.5965803	0.55078763	NA	Glt8d2	3.36023164	4.97776091	1.90847421	0	3.32962344	3.52816357	0	0
Tmem108	14.6954041	-0.2224614	0.37298444	-0.5964361	0.55088393	0.99938111	Tmem108	10.0806949	23.9837571	20.6751372	8.27256004	9.98887032	17.6408179	8.660375	18.2610202
Stat1	9.61246409	-0.2387291	0.40034953	-0.5963016	0.55097376	0.99938111	Stat1	15.4570656	12.6706641	13.9954775	0	13.3184938	8.23238167	8.660375	4.56525506
Abcf2	45.2129508	0.1864078	0.31264412	0.59623001	0.55102158	0.99938111	Abcf2	47.7152893	39.8220873	30.5355873	47.5672202	48.2795399	29.4013631	38.4905555	79.8919636
Ubxn2a	40.7327862	0.21151739	0.35488979	0.59600868	0.55116943	0.99938111	Ubxn2a	39.6507334	46.1574193	45.803381	14.4769801	19.9777406	69.3872169	53.8867778	36.5220405
Rsad2	0.45143362	0.17485587	0.2933917	0.59598095	0.55118795	NA	Rsad2	0	0.45252372	0.31807903	0	1.66481172	1.17605452	0	0
B130024G19Rik	0.35129559	0.1556983	0.26133477	0.59578104	0.55132151	NA	B130024G19R	0.67204633	0	0	0	0	1.17605452	0.96226389	0
Gm4926	0.35129559	0.1556983	0.26133477	0.59578104	0.55132151	NA	Gm4926	0.67204633	0	0	0	0	1.17605452	0.96226389	0
Hp	0.35129559	0.1556983	0.26133477	0.59578104	0.55132151	NA	Hp	0.67204633	0	0	0	0	1.17605452	0.96226389	0
Setd3	42.2879636	-0.1678727	0.28179268	-0.5957311	0.55135486	0.99938111	Setd3	59.8121232	38.0119924	39.1237212	45.4990802	31.6314227	47.042181	45.2264028	31.9567854
Eps8l1	5.1043418	0.24484481	0.41101016	0.59571475	0.5513658	0.99938111	Eps8l1	2.68818531	4.97776091	3.49886938	6.20442003	9.98887032	4.7042181	1.92452778	6.84788259
Sphk2	71.9948168	-0.2142894	0.35978296	-0.5956075	0.55143746	0.99938111	Sphk2	88.7101153	57.923036	47.3937761	122.020261	78.2461509	29.4013631	40.4150833	111.848749
4930581F22Rik	1.17892753	-0.1984862	0.33340745	-0.5953263	0.55162541	NA	4930581F22Ri	0	0.45252372	0.63615807	6.20442003	0	1.17605452	0.96226389	0
Nxpe3	13.6421652	0.22107209	0.37135182	0.5953171	0.55163153	0.99938111	Nxpe3	8.06455594	13.5757116	16.5401098	10.3407001	8.3240586	27.0492541	11.5471667	13.6957652
Khdrbs2	3.16371739	0.24706732	0.41504029	0.59528515	0.55165288	NA	Khdrbs2	5.37637063	0.45252372	2.54463228	2.06814001	4.99443516	4.7042181	2.88679167	2.28262753
Rictor	54.2749509	0.17701096	0.29735779	0.59527939	0.55165673	0.99938111	Rictor	63.1723549	52.4927514	49.9384084	35.1583802	73.2517157	78.7956531	47.1509305	34.239413
Cyp2u1	6.23308495	-0.2417811	0.40628145	-0.5951073	0.55177173	0.99938111	Cyp2u1	5.37637063	13.5757116	5.08926455	4.13628002	3.32962344	7.05632714	6.73584722	4.56525506
Gfm1	23.5653871	0.19708628	0.33122707	0.59501864	0.55183101	0.99938111	Gfm1	21.5054825	23.0787097	24.4920857	14.4769801	19.9777406	31.7534722	14.4339583	38.804668
Rims1	109.77597	0.18738497	0.31492411	0.59501629	0.55183258	0.99938111	Rims1	149.866331	108.605693	97.3321846	47.5672202	99.8887032	163.471579	127.018833	84.4572186
BC017158	11.6628281	0.2201952	0.37008499	0.5949855	0.55185317	0.99938111	BC017158	8.06455594	15.3858064	6.99773876	12.4088401	9.98887032	18.8168724	12.5094306	9.13051012
Rabgap1l	121.60365	0.22051198	0.37068839	0.59487156	0.55192933	0.99938111	Rabgap1l	121.640385	124.896546	139.318617	49.6353602	58.2684102	182.288451	232.867861	63.9135709
Rbm26	38.7241009	0.16721467	0.28110695	0.59484359	0.55194803	0.99938111	Rbm26	32.2582238	39.3695635	39.7598793	33.0902402	24.9721758	51.7463991	45.2264028	43.3699231
Nudt17	3.54028464	0.2445403	0.41119944	0.59469999	0.55204403	NA	Nudt17	2.01613899	0.90504744	2.54463228	6.20442003	0	2.35210905	2.88679167	11.4131377
Spred2	114.661909	0.206022	0.34644828	0.59466884	0.55206485	0.99938111	Spred2	85.3498837	93.6724098	94.1513942	142.701661	146.503431	48.2182355	87.5660139	219.132243
Sec22a	6.55379956	-0.2375096	0.39943178	-0.5946187	0.55209836	0.99938111	Sec22a	6.04841696	9.95552182	7.63389683	6.20442003	1.66481172	10.5844907	5.77358333	4.56525506
Txndc11	15.5424545	0.20765904	0.34924426	0.59459541	0.55211395	0.99938111	Txndc11	15.4570656	15.3858064	11.7689243	12.4088401	19.9777406	18.8168724	7.69811111	22.8262753
Tpd52	34.6989363	-0.1880298	0.31624295	-0.594574	0.55212828	0.99938111	Tpd52	46.3711967	33.4867552	45.4853019	24.8176801	33.2962344	32.9295267	45.2264028	15.9783927
Rnf123	39.2305438	0.19727616	0.33196533	0.59426735	0.55233331	0.99938111	Rnf123	36.2905017	26.2463757	31.8079035	49.6353602	68.2572805	30.5774176	23.0943333	47.9351782
Lig3	10.89376	-0.2127093	0.35796628	-0.5942161	0.55236756	0.99938111	Lig3	8.73660227	13.5757116	13.0412404	12.4088401	11.653682	7.05632714	11.5471667	9.13051012
Wasf3	158.391983	-0.1332853	0.22431307	-0.5941933	0.55238285	0.99938111	Wasf3	144.489961	157.02573	152.041778	213.018421	159.821925	127.013889	135.679208	178.044947
Gm20300	44.8160459	0.18158792	0.30561201	0.59417794	0.55239311	0.99938111	Gm20300	38.978687	43.442277	39.1237212	47.5672202	38.2906696	59.9787807	68.3207361	22.8262753
Dennd4b	44.3741601	0.22121931	0.37232906	0.59415	0.55241179	0.99938111	Dennd4b	31.5861774	30.7716129	35.9429309	59.9760603	64.9276571	23.5210905	19.2452778	89.0224737
Emd	39.3274111	-0.211097	0.35529749	-0.5941416	0.5524174	0.99938111	Emd	34.9464091	38.0119924	31.8079035	68.2486203	23.3073641	15.2887088	41.3773472	61.6309433
Tsr2	71.6561115	-0.1485931	0.25013203	-0.5940586	0.55247291	0.99938111	Tsr2	77.2853278	61.9957495	80.4739957	86.8618804	66.5924688	76.4435441	77.943375	45.6525506
Arfgef1	112.610399	0.21281194	0.35831505	0.59392408	0.55256289	0.99938111	Arfgef1	104.167181	125.801594	124.686982	51.7035003	59.9332219	212.865869	157.811278	63.9135709
Tial1	46.5237082	0.15655398	0.26359281	0.59392355	0.55256325	0.99938111	Tial1	45.027104	42.0847059	44.5310648	45.4990802	39.9554813	54.0985081	64.4716805	36.5220405
D930020B18Rik	0.7902088	-0.181979	0.30668118	-0.5933818	0.55292568	NA	D930020B18R	0	0.90504744	0.31807903	4.13628002	0	0	0.96226389	0
Snrpg	3.31458845	0.24060807	0.40549114	0.59337442	0.5529306	NA	Snrpg	4.7043243	0	5.08926455	0	4.99443516	2.35210905	4.81131944	4.56525506
Alg5	4.8534845	-0.2450603	0.41301226	-0.5933488	0.55294772	0.99938111	Alg5	6.04841696	7.69290322	5.08926455	4.13628002	0	5.88027262	7.69811111	2.28262753
Tnfrsf11b	0.98207842	-0.2101316	0.35421343	-0.5932343	0.55302437	NA	Tnfrsf11b	0.67204633	1.81009488	0.9542371	2.06814001	0	2.35210905	0	0
Chmp2b	51.0717494	0.2219629	0.37423239	0.59311515	0.55310409	0.99938111	Chmp2b	45.027104	49.3250854	66.1604392	20.6814001	13.3184938	67.0351079	80.8301666	66.1961984
Dtd2	7.32194252	-0.2386559	0.40239304	-0.5930915	0.55311994	0.99938111	Dtd2	5.37637063	6.33533207	7.95197586	14.4769801	3.32962344	2.35210905	9.62263889	9.13051012
Fancg	5.24122143	-0.2407345	0.4059752	-0.5929783	0.55319569	0.99938111	Fancg	6.04841696	5.43028463	4.13502745	8.27256004	4.99443516	2.35210905	3.84905555	6.84788259
Esd	6.47791301	-0.2416814	0.40766298	-0.5928462	0.55328409	0.99938111	Esd	6.04841696	6.78785578	11.1327662	6.20442003	3.32962344	3.52816357	12.5094306	2.28262753
Degs2	33.3660062	-0.1702271	0.28718566	-0.5927423	0.55335364	0.99938111	Degs2	34.2743627	29.8665654	37.215247	43.4309402	19.9777406	38.8097993	33.6792361	29.6741579
Samsn1	0.94303504	-0.2152974	0.36333881	-0.5925527	0.55348055	NA	Samsn1	1.34409266	1.35757116	0.63615807	2.06814001	0	1.17605452	0.96226389	0
Dok4	12.5504437	0.22431009	0.37855289	0.59254624	0.55348487	0.99938111	Dok4	16.1291119	6.33533207	9.224292	12.4088401	14.9833055	11.7605452	6.73584722	22.8262753
Pik3r2	81.4888315	0.18960495	0.32001995	0.59247853	0.55353019	0.99938111	Pik3r2	74.5971425	61.9957495	59.7988585	103.407001	114.872009	49.39429	57.7358333	130.109769
Dmd	45.5892389	0.19669407	0.33198805	0.59247335	0.55353366	0.99938111	Dmd	48.3873356	36.6544212	44.5310648	39.2946602	29.966611	77.6195986	65.4339444	22.8262753
Lin28b	0.97318526	0.21166491	0.35728564	0.5924249	0.5535661	NA	Lin28b	0.67204633	0.90504744	0.9542371	0	3.32962344	0	1.92452778	0
Smg7	150.548585	-0.1321667	0.22321932	-0.5920933	0.5537881	0.99938111	Smg7	158.602934	179.199393	175.897706	115.815841	151.497867	163.471579	136.641472	123.261887
1700028J19Rik	0.57294967	0.16189196	0.27350241	0.59192151	0.55390315	NA	1700028J19Ri	0	0	0.63615807	0	1.66481172	0	0	2.28262753

GeneID	Base mean	log2(FC)	StdErr	Wald-Stats	P-value	P-adj		GeneID	Normalised expression for Chow#1	Normalised expression for Chow#2	Normalised expression for Chow#3	Normalised expression for Chow#4	Normalised expression for HFD#1	Normalised expression for HFD#2	Normalised expression for HFD#3	Normalised expression for HFD#4
Ddx11	0.57294967	0.16189196	0.27350241	0.59192151	0.55390315	NA		Ddx11	0	0	0.63615807	0	1.66481172	0	0	2.28262753
Nrtn	0.57294967	0.16189196	0.27350241	0.59192151	0.55390315	NA		Nrtn	0	0	0.63615807	0	1.66481172	0	0	2.28262753
Gucy1b3	103.719406	0.16707453	0.28226598	0.59190459	0.55391448	0.99938111		Gucy1b3	129.032895	87.7896015	111.327662	57.9079203	114.872009	118.781507	134.716944	75.3267085
Dpm1	24.5387654	-0.2305667	0.38956276	-0.5918601	0.55394426	0.99938111		Dpm1	36.9625481	32.129184	25.1282437	16.5451201	4.99443516	32.9295267	38.4905555	9.13051012
Gpr85	31.8604632	-0.2138151	0.36135303	-0.5917068	0.55404692	0.99938111		Gpr85	30.9141311	55.6604174	33.0802196	20.6814001	18.3129289	51.7463991	30.7924444	13.6957652
Fbxo10	32.551843	0.19949455	0.33717273	0.59166871	0.55407245	0.99938111		Fbxo10	26.2098068	32.129184	25.1282437	35.1583802	64.9276571	24.697145	20.2075417	31.9567854
Psmb2	75.667408	-0.2073941	0.35056411	-0.5916011	0.55411777	0.99938111		Psmb2	75.9412351	62.9007969	65.8423601	126.156541	76.5813391	23.5210905	62.5471528	111.848749
Trh	1.56258797	-0.227895	0.3852758	-0.5915114	0.5541778	NA		Trh	0.67204633	1.81009488	1.59039517	4.13628002	3.32962344	0	0.96226389	0
Vps13a	41.3108443	0.22867789	0.38664583	0.59144021	0.5542255	0.99938111		Vps13a	35.6184554	52.4927514	48.9841713	8.27256004	34.9610461	76.4435441	57.7358333	15.9783927
Cnga4	0.4247098	0.17304323	0.29258989	0.59141903	0.55423969	NA		Cnga4	0	0.45252372	0.31807903	0	1.66481172	0	0.96226389	0
D330050I16Rik	2.52958075	0.21558203	0.364639	0.59122044	0.55437273	NA		D330050I16Ri	0.67204633	0.45252372	0.63615807	6.20442003	9.98887032	0	0	2.28262753
Cybrd1	2.48639092	-0.2421	0.40957207	-0.5911047	0.5544503	NA		Cybrd1	1.34409266	6.33533207	3.49886938	0	3.32962344	1.17605452	1.92452778	2.28262753
Ago2	128.829438	0.19197998	0.32479154	0.59108676	0.55446229	0.99938111		Ago2	69.2207718	111.320835	98.6045007	196.473301	189.788536	143.478652	77.943375	143.805534
Zfp768	11.6786291	0.23359251	0.39527103	0.59096795	0.55454189	0.99938111		Zfp768	11.4247876	7.2403795	6.04350166	16.5451201	19.9777406	5.88027262	5.77358333	20.5436478
Slc24a5	9.66773064	-0.245384	0.41522972	-0.5909597	0.55454743	0.99938111		Slc24a5	17.4732045	9.95552182	15.2677937	2.06814001	0	16.4647633	11.5471667	4.56525506
Dusp6	25.5148261	-0.1842452	0.3118532	-0.5908074	0.55464949	0.99938111		Dusp6	34.9464091	22.6261859	30.5355873	20.6814001	21.6425524	16.4647633	29.8301805	27.3915304
Zfp869	19.1212953	0.1861632	0.31512246	0.59076461	0.55467815	0.99938111		Zfp869	16.1291119	18.1009488	21.3112953	14.4769801	16.6481172	18.8168724	26.9433889	20.5436478
Klhl2	83.0627296	0.14613213	0.24736458	0.59075608	0.55468387	0.99938111		Klhl2	88.7101153	88.6946489	79.5197586	55.8397803	86.5702095	94.0843619	104.886764	66.1961984
Gm15417	0.50142021	0.16110203	0.2727635	0.59062899	0.55476903	NA		Gm15417	0.67204633	0.45252372	0	0	0	0	2.88679167	0
Schip1	107.590845	-0.2160534	0.36580309	-0.5906275	0.55477001	0.99938111		Schip1	148.522239	151.595446	134.229353	39.2946602	51.6091633	123.485725	154.924486	57.0656883
Tmem68	19.4987133	-0.2424781	0.41070685	-0.5903921	0.5549278	0.99938111		Tmem68	16.1291119	39.3695635	23.8559276	10.3407001	4.99443516	38.8097993	20.2075417	2.28262753
Tmem126a	8.90405209	-0.2338846	0.39624525	-0.5902522	0.55502155	0.99938111		Tmem126a	11.4247876	9.05047438	10.4966081	10.3407001	3.32962344	4.7042181	17.32075	4.56525506
Fgf2	0.63416535	-0.1821166	0.30860389	-0.5901304	0.5551032	NA		Fgf2	0	0.45252372	1.59039517	2.06814001	0	0	0.96226389	0
Mlycd	13.3620474	0.22835973	0.38699318	0.59008722	0.55513217	0.99938111		Mlycd	11.4247876	9.5029981	9.224292	16.5451201	19.9777406	7.05632714	5.77358333	27.3915304
Grm4	28.6320785	-0.2251307	0.38154951	-0.5900433	0.55516162	0.99938111		Grm4	24.1936678	25.793852	20.0389792	57.9079203	36.6258578	23.5210905	6.73584722	34.239413
Rnf32	4.21037771	0.24563206	0.41635155	0.58996312	0.55521538	NA		Rnf32	1.34409266	7.69290322	4.13502745	0	3.32962344	5.88027262	6.73584722	4.56525506
Rbm4	17.9215184	-0.2170707	0.36795381	-0.58994	0.5552309	0.99938111		Rbm4	14.1129729	11.7656167	15.9039517	39.2946602	19.9777406	10.5844907	13.4716944	18.2610202
Ptp4a2	96.7614547	-0.1759217	0.29826123	-0.5898241	0.55530859	0.99938111		Ptp4a2	104.167181	109.058216	104.648002	99.2707205	56.6035985	81.1477622	152.999958	66.1961984
Ankmy2	23.7115887	0.22192989	0.37636739	0.58966292	0.55541666	0.99938111		Ankmy2	35.6184554	13.5757116	26.7186389	8.27256004	33.2962344	29.4013631	33.6792361	9.13051012
Rbpms	1.38980431	-0.2268717	0.38485705	-0.5894961	0.55552854	NA		Rbpms	0.67204633	2.26261859	1.90847421	2.06814001	0	0	1.92452778	2.28262753
Spty2d1	21.2005418	0.20784701	0.35259037	0.58948578	0.55553545	0.99938111		Spty2d1	31.5861774	16.2908539	20.0389792	8.27256004	18.3129289	34.1055812	25.0188611	15.9783927
Cpeb4	151.113722	0.13464794	0.22844903	0.58940037	0.55559273	0.99938111		Cpeb4	151.88247	159.288349	140.909012	122.020261	163.151549	198.753215	158.773542	114.131377
Tnr	70.0392916	0.20973907	0.35586054	0.58938557	0.55560266	0.99938111		Tnr	54.4357526	95.029981	67.4327553	35.1583802	89.8998329	132.894161	37.5282917	47.9351782
Zc3hc1	13.3714824	0.21668084	0.3677785	0.58916126	0.5557531	0.99938111		Zc3hc1	10.0806949	7.2403795	9.86045007	22.7495401	16.6481172	10.5844907	11.5471667	18.2610202
Stxbp2	5.92439273	0.23574464	0.4001908	0.58908061	0.5558072	0.99938111		Stxbp2	6.04841696	3.16766603	5.08926455	6.20442003	4.99443516	4.7042181	5.77358333	11.4131377
Krba1	18.2643741	0.21978323	0.37311367	0.58905167	0.55582661	0.99938111		Krba1	17.4732045	17.1959013	12.4050823	18.6132601	43.2851047	16.4647633	11.5471667	9.13051012
Rbbp4	56.391083	0.16461245	0.27956524	0.58881587	0.5559848	0.99938111		Rbbp4	45.6991503	44.3473245	57.8903843	64.1123403	58.2684102	37.6337448	76.9811111	66.1961984
Zfp532	32.9550514	-0.1684254	0.28606121	-0.588774	0.55601291	0.99938111		Zfp532	44.3550577	39.8220873	30.8536663	24.8176801	34.9610461	32.9295267	30.7924444	25.1089028
Usp33	79.3475481	0.21661137	0.36791195	0.58875873	0.55602314	0.99938111		Usp33	77.2853278	74.6664136	103.693765	28.9539601	46.6147282	136.422325	126.056569	41.0872956
Adcy5	175.403943	0.20577876	0.34951862	0.58874907	0.55602962	0.99938111		Adcy5	125.672663	156.120683	127.867772	227.495401	321.308662	148.18287	68.3207361	228.262753
Dock10	33.1648825	-0.1984305	0.33706358	-0.5887034	0.55606025	0.99938111		Dock10	26.8818531	49.3250854	37.5333261	31.0221002	26.6369875	52.9224536	25.0188611	15.9783927
Dse	2.7071986	0.24167811	0.41067862	0.58848476	0.55620696	NA		Dse	0.67204633	4.07271347	2.86271131	0	4.99443516	3.52816357	0.96226389	4.56525506
Dnaja3	55.0312367	0.17582547	0.29879508	0.58844834	0.5562314	0.99938111		Dnaja3	40.994826	41.1796584	46.7576181	78.5893204	69.9220923	36.4576902	69.283	57.0656883
Zfp850	8.13896201	0.23445506	0.39850887	0.58833084	0.55631025	0.99938111		Zfp850	11.4247876	6.33533207	8.2700549	2.06814001	3.32962344	15.2887088	11.5471667	6.84788259
Stim1	51.1949753	0.17253286	0.29329066	0.58826579	0.5563539	0.99938111		Stim1	33.6023164	54.3028463	48.6660923	53.7716403	53.2739751	72.9153805	33.6792361	59.3483158
N6amt2	11.5416982	-0.2310399	0.39278839	-0.5882045	0.55639506	0.99938111		N6amt2	16.8011582	14.480759	16.5401098	4.13628002	6.65924688	14.1126543	17.32075	2.28262753
Sec61a2	27.1979612	0.19780615	0.33633084	0.5881297	0.55644524	0.99938111		Sec61a2	18.8172972	36.6544212	26.0824808	16.5451201	28.3017992	45.8661264	20.2075417	25.1089028
Mar-06	200.994048	0.18060174	0.30715119	0.58798969	0.55653921	0.99938111		Mar-06	120.968339	260.201138	228.698826	132.360961	209.766277	321.062885	156.849014	178.044947
Taf12	8.53874018	-0.2289647	0.38944806	-0.5879211	0.55658524	0.99938111		Taf12	10.0806949	11.7656167	12.0870033	4.13628002	3.32962344	11.7605452	10.5849028	4.56525506
Smg5	63.7632655	-0.1703825	0.28985819	-0.5878134	0.55665755	0.99938111		Smg5	61.1562159	58.3755597	51.5288036	103.407001	56.6035985	62.3308898	41.3773472	75.3267085
Jmjd8	89.5850169	-0.1830856	0.3114816	-0.5877896	0.55667356	0.99938111		Jmjd8	79.9735131	74.6664136	75.0666521	157.178641	96.5590798	62.3308898	56.7735694	114.131377
Ranbp9	53.8154894	-0.2217259	0.37725194	-0.5877396	0.55670707	0.99938111		Ranbp9	79.9735131	76.9290322	62.9796488	18.6132601	24.9721758	68.2111624	76.0188472	22.8262753
Tlk2	32.3027506	0.20944185	0.3563572	0.5877301	0.55671347	0.99938111		Tlk2	36.2905017	29.4140417	40.0779583	10.3407001	26.6369875	43.5140174	53.8867778	18.2610202
6530411M01Rik	0.70665517	0.17010114	0.28948116	0.58760693	0.55679616	NA		6530411M01R	0	0	1.59039517	0	0	1.17605452	2.88679167	0
Cited1	4.54914067	-0.2042583	0.34767076	-0.5875048	0.55686471	NA		Cited1	0.67204633	0	0.9542371	22.7495401	0	0	2.88679167	9.13051012
Atxn1l	42.9770065	0.16390644	0.27900517	0.5874674	0.55688984	0.99938111		Atxn1l	44.3550577	40.7271347	40.0779583	33.0902402	64.9276571	39.9858538	32.7169722	47.9351782
Wdr25	8.42106517	0.24363985	0.41489405	0.58723389	0.55704664	0.99938111		Wdr25	4.7043243	4.07271347	4.13502745	16.5451201	14.9833055	1.17605452	5.77358333	15.9783927
Sarnp	2.78487605	-0.238564	0.4062675	-0.5872092	0.55706325	NA		Sarnp	2.01613899	3.16766603	7.63389683	0	3.32962344	0	3.84905555	2.28262753
Rbm24	13.6838572	0.20083546	0.3420381	0.58717278	0.55708767	0.99938111		Rbm24	12.0968339	10.4080455	12.0870033	16.5451201	19.9777406	10.5844907	16.3584861	11.4131377
Frmd6	8.67109471	-0.2285357	0.38928451	-0.5870661	0.55715935	0.99938111		Frmd6	10.7527413	8.59795066	6.36158069	14.4769801	13.3184938	5.88027262	7.69811111	2.28262753
Poldip3	86.1102523	0.18800194	0.32024151	0.587063	0.5571614	0.99938111		Poldip3	73.2530498	54.3028463	68.3869924	122.020261	111.542385	64.6829988	57.7358333	136.957652
Lrp6	37.8962396	0.20575542	0.35049339	0.5870451	0.55717342	0.99938111		Lrp6	31.5861774	52.4927514	33.7163777	18.6132601	31.6314227	75.2674895	27.9056528	31.9567854
Scrib	23.115586	0.23971548	0.40834671	0.58703909	0.55717745	0.99938111		Scrib	12.7688802	10.8605693	8.2700549	49.6353602	44.9499164	9.40843619	12.5094306	36.5220405
Mettl4	10.8153268	-0.2158865	0.3678256	-0.5869262	0.55725328	0.99938111		Mettl4	12.7688802	12.2181404	11.7689243	12.4088401	4.99443516	15.2887088	12.5094306	4.56525506
A730098P11Rik	110.627617	0.20238313	0.34482754	0.58691116	0.55726337	0.99938111		A730098P11Ri	112.903783	93.6724098	120.870033	76.5211804	54.9387868	141.126543	216.509375	68.4788259
Mmp28	0.68357948	-0.1710591	0.29146406	-0.5868961	0.55727351	NA		Mmp28	0	3.16766603	0.63615807	0	1.66481172	0	0	0
Elmod3	13.4408949	-0.210798	0.35934102	-0.5866238	0.5574564	0.99938111		Elmod3	14.1129729	19.0059962	12.4050823	12.4088401	4.99443516	10.5844907	13.4716944	20.5436478
Ranbp3	43.3335946	0.17742457	0.30250682	0.58651428	0.55752997	0.99938111		Ranbp3	38.3066407	31.2241366	38.4875632	53.7716403	66.5924688	27.0492541	43.301875	47.9351782
Bscl2	65.5178484	-0.1589763	0.27119956	-0.5861966	0.5577434	0.99938111		Bscl2	51.075521	76.4765085	72.5220199	76.5211804	51.6091633	58.8027262	48.1131944	89.0224737
Wdr18	47.8687811	0.22281789	0.38018421	0.58607875	0.5578226	0.99938111		Wdr18	29.5700384	26.2463757	36.897168	78.5893204	76.5813391	22.345036	25.981125	86.7398462
Izumo4	5.65113524	-0.243597	0.41564882	-0.5860644	0.55783225	0.99938111		Izumo4	1.34409266	9.5029981	6.67965972	8.27256004	3.32962344	1.17605452	5.77358333	9.13051012
Nab1	15.4295163	-0.235437	0.40173032	-0.5860574	0.55783692	0.99938111		Nab1	22.8495752	25.793852	17.1762679	4.13628002	8.3240586	18.8168724	24.0565972	2.28262753
Ggh	5.41571989	-0.2432636	0.41511376	-0.5860166	0.55786436	0.99938111		Ggh	2.68818531	8.14542694	8.90621297	6.20442003	1.66481172	7.05632714	8.660375	0
Hist1h3c	0.54742897	-0.1867928	0.3187999	-0.5859248	0.55792607	NA		Hist1h3c	1.34409266	0.90504744	0.9542371	0	0	1.17605452	0	0
Erlin1	9.76107792	-0.2436324	0.41594999	-0.5857251	0.55806026	0.99938111		Erlin1	10.0806949	21.2686148	14.3135566	0	6.65924688	19.9929269	5.77358333	0
Arhgap20	77.3580419	-0.1952155	0.33343155	-0.585474	0.55822903	0.99938111		Arhgap20	106.855366	99.5552182	98.9225797	28.9539601	58.2684102	84.6759257	91.4150694	50.2178057
Elmo1	234.505711	-0.1482738	0.25326788	-0.5854424	0.55825027	0.99938111		Elmo1	223.119381	244.815332	278.637234	241.972381	158.157113	228.154578	177.056555	324.133109
Acsl6	56.2526314	-0.197728	0.33775283	-0.5854223	0.5582638	0.99938111		Acsl6	63.1723549	89.5996963	55.663831	35.1583802	54.9387868	85.8519803	33.6792361	31.9567854
Abhd17b	17.3619749	-0.2178884	0.37226106	-0.5853108	0.55833872	0.99938111		Abhd17b	22.8495752	20.8160911	21.6293743	12.4088401	6.65924688	18.8168724	28.8679167	6.84788259
Alox12e	0.33770798	-0.1313177	0.22442504	-0.5851295	0.55846063	NA		Alox12e	1.34409266	1.35757116	0	0	0	0	0	0
Cd52	0.33770798	-0.1313177	0.22442504	-0.5851295	0.55846063	NA		Cd52	1.34409266	1.35757116	0	0	0	0	0	0
Ltn1	41.1697416	0.2117872	0.36199412	0.58505701	0.55850938	0.99938111		Ltn1	41.6668724	39.3695635	48.6660923	18.6132601	48.2795399	69.3872169	51.96225	11.4131377
Rbms1	6.23652305	-0.238399	0.40751333	-0.5850091	0.55854158	0.99938111		Rbms1	9.4086486	4.97776091	11.1327662	2.06814001	1.66481172	7.05632714	6.73584722	6.84788259
Noa1	12.0235889	0.20577228	0.35177014	0.58496233	0.55857304	0.99938111		Noa1	11.4247876	9.5029981	11.4508452	12.4088401	16.6481172	10.5844907	17.32075	6.84788259
Lrrc61	39.999044	0.23777806	0.40659397	0.58480469	0.55867905	0.99938111		Lrrc61	26.2098068	13.5757116	13.6773985	86.8618804	59.9332219	19.9929269	22.1320694	77.6093361
Blvrb	3.28176367	-0.241184	0.41250168	-0.584686	0.55875887	NA		Blvrb	6.72046328	2.71514231	0.9542371	6.20442003	0	3.52816357	3.84905555	2.28262753
Rab2a	130.220459	0.14479328	0.24765435	0.58465874	0.5587772	0.99938111		Rab2a	136.425405	116.751119	133.275115	105.475141	98.2238915	127.013889	187.641458	136.957652
Tmem65	127.245638	0.17448106	0.29849508	0.58453579	0.5588599	0.99938111		Tmem65	120.968339	96.8400759	137.092064	117.883981	59.9332219	148.18287	163.584861	173.479692
2610044O15Rik8	5.70803332	0.23978168	0.41021936	0.58452062	0.5588701	0.99938111		2610044O15Ri	10.0806949	2.71514231	3.18079035	4.13628002	6.65924688	4.7042181	9.62263889	4.56525506
Ufm1	39.3143799	0.18991352	0.32490492	0.58452028	0.55887033	0.99938111		Ufm1	42.3389187	40.7271347	36.579089	24.8176801	24.9721758	34.1055812	72.1697916	38.804668
Hist1h1d	6.92099867	-0.239242	0.4093632	-0.5844248	0.55893457	0.99938111		Hist1h1d	11.4247876	11.313093	8.2700549	0	3.32962344	5.88027262	10.5849028	4.56525506
Bai1	225.232968	0.20431147	0.34964705	0.58433631	0.55899407	0.99938111		Bai1	148.522239	260.653662	160.311833	248.176801	381.241884	211.689814	80.8301666	310.437344
Whsc1l1	82.6651192	0.18744059	0.32083013	0.58423623	0.55906139	0.99938111		Whsc1l1	78.6294204	95.029981	90.3344458	39.2946602	69.9220923	143.478652	87.5660139	57.0656883
Etohd2	4.19265097	-0.2431517	0.4162693	-0.5841212	0.55913879	NA		Etohd2	6.04841696	5.43028463	3.18079035	6.20442003	1.66481172	2.35210905	8.660375	0
Ndufa5	24.3884994	0.23333801	0.39954078	0.58401551	0.55920988	0.99938111		Ndufa5	32.9302701	23.5312334	20.9932163	8.27256004	11.653682	34.1055812	56.7735694	6.84788259
Wdr43	14.0652826	0.2314588	0.39633704	0.58399486	0.55922377	0.99938111		Wdr43	12.7688802	12.6706641	19.4028211	4.13628002	6.65924688	31.7534722	18.2830139	6.84788259
Akirin2	57.7228652	0.15087624	0.25835313	0.58399228	0.5592255	0.99938111		Akirin2	57.1239379	57.4705123	58.8446214	41.3628002	54.9387868	45.8661264	73.1320555	73.044081
Rpf1	7.31170219	0.24089356	0.41255973	0.58389984	0.5592877	0.99938111		Rpf1	9.4086486	6.33533207	6.99773876	2.06814001	0	15.2887088	11.5471667	6.84788259
Pias3	25.0024116	0.21942757	0.37579744	0.58389853	0.55928858	0.99938111		Pias3	13.4409266	16.7433776	15.9039517	45.4990802	41.620293	11.7605452	23.0943333	31.9567854
Spns3	0.61362578	0.15838903	0.27128781	0.5838413	0.55932709	NA		Spns3	0	0	0	2.06814001	1.66481172	1.17605452	0	0
Stk36	4.40498144	0.24038531	0.41182123	0.58371277	0.55941358	NA		Stk36	2.68818531	2.26261859	1.59039517	8.27256004	11.653682	0	1.92452778	6.84788259
Mat2a	50.0438992	0.15006764	0.25709989	0.58369391	0.55942627	0.99938111		Mat2a	43.6830113	42.0847059	55.9819101	45.4990802	61.5980337	57.6266717	41.3773472	52.5004332
Pet112	6.88263467	-0.2272615	0.38948941	-0.5834856	0.55956643	0.99938111		Pet112	7.39250961	6.33533207	10.8146872	6.20442003	4.99443516	7.05632714	7.69811111	4.56525506
Naa35	19.2237757	0.2115306	0.36278402	0.58307585	0.55984224	0.99938111		Naa35	14.7850192	22.1736622	21.9474534	10.3407001	11.653682	36.4576902	25.0188611	11.4131377
Fam126b	143.152356	0.18514646	0.31759285	0.58296798	0.55991486	0.99938111		Fam126b	130.376988	158.835825	169.536125	68.2486203	111.542385	219.922196	188.603722	98.1529838
Kiss1	0.58690195	0.15803094	0.27108309	0.58296126	0.55991938	NA		Kiss1	0	0	0	2.06814001	1.66481172	0	0.96226389	0
3110056K07Rik	2.42545262	0.23880212	0.40967294	0.58290919	0.55995443	NA		3110056K07Ri	2.01613899	2.26261859	3.18079035	0	4.99443516	1.17605452	5.77358333	0
6820431F20Rik	9.82306564	-0.2243384	0.38494208	-0.5827848	0.56003816	0.99938111		6820431F20Ri	14.1129729	11.7656167	10.4966081	8.27256004	4.99443516	7.05632714	17.32075	4.56525506
Gldn	2.7183062	0.24081864	0.41326979	0.58271532	0.56008496	NA		Gldn	1.34409266	1.81009488	4.77118552	0	1.66481172	4.7042181	2.88679167	4.56525506
Zfp948	6.75905687	-0.242034	0.41557802	-0.5824032	0.56029511	0.99938111		Zfp948	6.72046328	11.7656167	10.8146872	2.06814001	0	11.7605452	8.660375	2.28262753
Ly6c2	0.66677389	-0.1715819	0.29471579	-0.5821944	0.56043577	NA		Ly6c2	0	2.71514231	0.9542371	0	1.66481172	0	0	0
Arl1	18.2378455	-0.1911606	0.32848	-0.5819552	0.56059687	0.99938111		Arl1	28.2259458	18.1009488	17.1762679	14.4769801	14.9833055	14.1126543	18.2830139	20.5436478

GeneID	Base mean	log2(FC)	StdErr	Wald-Stats	P-value	P-adj
Nphp1	9.20109571	-0.2275934	0.3910886	-0.5819483	0.56060148	0.99938111
Myb	0.62451254	0.18223956	0.3132273	0.58181249	0.56069299	NA
Ttc30a1	4.03802636	-0.2422182	0.41634458	-0.5817734	0.56071931	NA
Lpcat2	1.44239323	-0.1749112	0.30070149	-0.5816771	0.56078417	NA
Smc5	11.120537	0.23462649	0.4033911	0.58163528	0.56081237	0.99938111
Mak	1.11578467	-0.2022934	0.3478407	-0.5815691	0.56085694	NA
Snrnp27	16.6252073	-0.2328601	0.4004135	-0.5815491	0.56087047	0.99938111
Asb2	0.87304723	0.20245815	0.34828498	0.58130028	0.5610381	NA
Itgbl1	5.02607599	-0.2408732	0.4144043	-0.5812516	0.56107087	0.99938111
Cyth2	43.91632	-0.1705349	0.29342017	-0.5811968	0.5611078	0.99938111
Clcn5	6.51865533	0.23235728	0.39982597	0.58114603	0.56114204	0.99938111
Gnai3	20.0320698	-0.2086725	0.35914367	-0.581028	0.56122161	0.99938111
Slc23a2	108.812407	0.19152631	0.32964103	0.58101477	0.5612305	0.99938111
AI314180	68.3107902	0.17923868	0.30851267	0.58097674	0.56125613	0.99938111
Thap7	15.5264065	-0.1931707	0.33250146	-0.5809618	0.56126618	0.99938111
Snx33	6.92894266	-0.2320901	0.39974683	-0.5805927	0.56151496	0.99938111
Pex16	8.85139775	-0.233604	0.40240246	-0.5805233	0.56156176	0.99938111
Gnb2l1	77.1122225	-0.196855	0.33914495	-0.5804451	0.56161449	0.99938111
Sumo1	38.5422335	-0.190871	0.32892039	-0.5802956	0.56171531	0.99938111
Tmem19	20.6492702	-0.1849407	0.31873206	-0.5802387	0.56175369	0.99938111
Polr3gl	11.6121312	-0.2180266	0.37576062	-0.5802273	0.56176132	0.99938111
Grhl3	1.90230285	0.23127856	0.39867736	0.58011462	0.56183733	NA
Gm16381	3.95284619	-0.2379547	0.41020607	-0.5800858	0.56185677	NA
Dleu2	2.41963363	-0.2362198	0.40722566	-0.580071	0.56186677	NA
Gm7244	0.9707408	0.20844006	0.35940271	0.57996241	0.56193996	NA
Gm1821	312.764463	0.14441882	0.24902134	0.57994558	0.56195132	0.99938111
Ubb	415.355846	0.10303963	0.17769314	0.57987398	0.5619996	0.99938111
Fdxr	2.32213091	-0.2316741	0.39960484	-0.5797579	0.56207788	NA
Dsg1c	0.3270511	-0.1304481	0.22500972	-0.5797445	0.56208695	NA
Msx2	0.3270511	-0.1304481	0.22500972	-0.5797445	0.56208695	NA
Plscr2	0.3270511	-0.1304481	0.22500972	-0.5797445	0.56208695	NA
Xirp2	0.3270511	-0.1304481	0.22500972	-0.5797445	0.56208695	NA
Akap3	1.24045356	0.18864887	0.32541192	0.5797233	0.56210123	NA
Ntrk3	57.991171	0.17714799	0.30561613	0.57964216	0.56215596	0.99938111
1700023F06Rik	4.55153544	-0.2411434	0.41621022	-0.5793788	0.56233363	NA
Ppara	3.87613511	-0.2387068	0.41216062	-0.5791596	0.56248152	NA
Adamtsl4	2.90822547	0.24040675	0.41512311	0.57912159	0.56250714	NA
Scarna13	2.10831967	0.23644312	0.40830203	0.57908877	0.56252928	NA
Atad5	4.08128396	0.23921708	0.41324337	0.578877	0.56267217	NA
Kidins220	189.901321	0.20080763	0.34689654	0.57886895	0.5626776	0.99938111
Mtfmt	4.16648781	-0.2393206	0.41368061	-0.5785153	0.56291627	NA
Plekha4	0.96682769	0.16435578	0.28411531	0.57848266	0.5629383	NA
Ppp2r3c	15.9987154	-0.2154191	0.3724176	-0.5784344	0.5629709	0.99938111
Nvl	10.9572275	0.21951727	0.37959688	0.57829051	0.563068	0.99938111
Sccpdh	33.8124866	0.18677396	0.32297802	0.57828691	0.56307043	0.99938111
Tmem170b	103.780044	0.13153963	0.22747252	0.57826602	0.56308453	0.99938111
Pou3f3	29.1255796	0.16545813	0.28620183	0.57811696	0.56318516	0.99938111
Rab13	2.36164781	-0.2356469	0.40761895	-0.5781058	0.56319268	NA
Gem	0.52853866	-0.164558	0.28469224	-0.5780205	0.56325028	NA
Odc1	24.9152687	0.20606479	0.3566254	0.5778186	0.5633866	0.99938111
Snrpb	29.8576438	0.23766602	0.41134987	0.577771	0.56341874	0.99938111
Dmp1	0.80996802	-0.2003901	0.34684247	-0.5777553	0.56342935	NA
3110001I22Rik	0.49941352	-0.1755442	0.3038556	-0.5777224	0.56345153	NA
Trip6	0.77338252	0.19425655	0.33639643	0.57746319	0.5636266	NA
5730405O15Rik	3.37526464	-0.2322916	0.40252208	-0.5770903	0.56387846	NA
Hydin	2.47647641	0.23013585	0.39878859	0.57708737	0.56388044	NA
Dctn4	76.1147519	-0.1576654	0.27323746	-0.577027	0.56392122	0.99938111
Sec63	45.2899631	0.21869876	0.37902871	0.57699787	0.5639409	0.99938111
Rom1	1.80649313	-0.2223342	0.38553865	-0.5766846	0.56415251	NA
Eif4ebp3	0.27665648	-0.1466492	0.25450084	-0.5762229	0.5644645	NA
Pole	0.27665648	-0.1466492	0.25450084	-0.5762229	0.5644645	NA
Prss16	0.27665648	-0.1466492	0.25450084	-0.5762229	0.5644645	NA
Rad9b	0.27665648	-0.1466492	0.25450084	-0.5762229	0.5644645	NA
Tnfrsf18	0.27665648	-0.1466492	0.25450084	-0.5762229	0.5644645	NA
Zc3h12a	0.27665648	-0.1466492	0.25450084	-0.5762229	0.5644645	NA
Rprm	33.7540296	-0.2049067	0.35582046	-0.575871	0.56470238	0.99938111
Lsp1	0.97124394	-0.205743	0.35740384	-0.5756597	0.56484519	NA
Efcab5	1.34535042	0.20280707	0.35240899	0.57548778	0.56496145	NA
Bcdin3d	2.0143735	0.2269009	0.39430452	0.57544586	0.5649898	NA
Rab37	0.50389955	-0.1779868	0.3093745	-0.5753118	0.56508046	NA
Tmc3	0.80391912	0.17997171	0.3129608	0.57506149	0.56524971	NA
Tmem200a	11.1230334	-0.2294359	0.39926587	-0.5746444	0.56553183	0.99938111
1200014J11Rik	12.3592488	0.19870903	0.3459268	0.57442509	0.56568018	0.99938111
Fasn	276.306905	0.2254328	0.39250284	0.57434692	0.56573306	0.99938111
Gatsl2	185.401103	0.20255393	0.35270646	0.57428471	0.56577515	0.99938111
Vps72	20.5433945	-0.2231871	0.38867005	-0.5742328	0.5658103	0.99938111
Ifi27	6.25429185	0.238929	0.41636895	0.57383962	0.56607634	0.99938111
Tiparp	13.9755873	0.21224236	0.36989384	0.57379264	0.56610813	0.99938111
Tcfl5	0.52519117	-0.1758096	0.30644432	-0.5737082	0.56616527	NA
Tgfb2	10.0620652	-0.2203123	0.38419535	-0.5734381	0.56634807	0.99938111
Tspan11	0.32256507	-0.1268398	0.22120671	-0.5733994	0.56637429	NA
Fnbp1	67.3580403	-0.1986641	0.3465921	-0.5731928	0.56651415	0.99938111
Zfp574	30.7157797	0.20621738	0.35978753	0.57316434	0.56653343	0.99938111
Ufd1l	41.2017015	-0.1667607	0.29098845	-0.5730836	0.56658811	0.99938111
Large	105.54329	0.20014828	0.34931658	0.57297103	0.56666431	0.99938111
Rarres2	0.82756737	-0.1767441	0.30849346	-0.5729266	0.56669437	NA
Gcdh	11.924293	0.20746687	0.36215711	0.57286426	0.56673661	0.99938111
9130401M01Rik	21.1380705	-0.2159368	0.3769663	-0.5728279	0.5667612	0.99938111
9030612E09Rik	1.34018275	0.21548865	0.37632911	0.57260691	0.56691088	NA
Ecd	20.555308	0.18655638	0.32589097	0.5724503	0.56701695	0.99938111
Rimbp3	2.27036417	0.22572588	0.39432116	0.57244172	0.56702276	NA
Tbl1xr1	109.295487	0.14797248	0.25854493	0.57232789	0.56709986	0.99938111
Cacna1c	96.6507181	0.19842087	0.34671528	0.57228766	0.56712711	0.99938111
Timeless	1.56640074	-0.2243223	0.39198414	-0.5722738	0.56713647	NA
9330182L06Rik	34.1359214	0.2277516	0.39800797	0.57222873	0.56716702	0.99938111
Mettl10	12.8505798	-0.2165048	0.37839998	-0.5721585	0.56721459	0.99938111
Aamp	167.913661	0.23228485	0.40616017	0.57190455	0.56738664	0.99938111
Thbd	4.77764265	-0.235891	0.4126191	-0.571692	0.56753065	0.99938111
Tmem246	57.4352825	-0.1850684	0.32373272	-0.5716703	0.56754533	0.99938111
Pld3	304.79598	-0.1881676	0.32929785	-0.5714208	0.56771443	0.99938111
Cfl2	54.7949689	0.20454378	0.3580266	0.57130891	0.56779026	0.99938111
1600012H06Rik	11.0063624	-0.2186207	0.38269001	-0.5712736	0.56781419	0.99938111
Psmd4	45.742523	0.15796513	0.27657092	0.57115597	0.56789392	0.99938111
Cgn	3.39910963	0.23606452	0.41337492	0.57106638	0.56795464	NA
Tbca	7.11694287	0.23348265	0.40890455	0.57099549	0.5680027	0.99938111
Clec18a	0.31193033	-0.1279441	0.22410083	-0.5709219	0.56805262	NA
2810013P06Rik	7.72501658	-0.222559	0.39003689	-0.5706102	0.56826387	0.99938111
Hck	0.33153713	-0.127689	0.2237969	-0.5705577	0.56829949	NA
Pomgnt2	24.2132664	0.21266195	0.37275981	0.57050665	0.56833411	0.99938111
Btbd1	73.5555434	-0.1934361	0.33908401	-0.5704665	0.56836136	0.99938111
Sgsm1	133.170883	0.20813883	0.36491004	0.57038396	0.56841731	0.99938111
Ago4	7.03461487	-0.2253802	0.39515359	-0.5703609	0.56843294	0.99938111

GeneID	Normalised expression for Chow#1	Normalised expression for Chow#2	Normalised expression for Chow#3	Normalised expression for Chow#4	Normalised expression for HFD#1	Normalised expression for HFD#2	Normalised expression for HFD#3	Normalised expression for HFD#4
Nphp1	11.4247876	5.88280835	8.90621297	16.5451201	3.32962344	5.88027262	12.5094306	9.13051012
Myb	0	0.45252372	0.9542371	0	1.66481172	0	1.92452778	0
Ttc30a1	4.7043243	2.71514231	4.13502745	8.27256004	0	1.17605452	6.73584722	4.56525506
Lpcat2	0	7.69290322	0.31807903	0	0	3.52816357	0	0
Smc5	11.4247876	10.8605693	13.9954775	2.06814001	4.99443516	24.697145	16.3584861	4.56525506
Mak	2.68818531	2.71514231	0.63615807	0	0	0	2.88679167	0
Snrnp27	20.1613899	19.4585199	27.672876	8.27256004	8.3240586	14.1126543	32.7169722	2.28262753
Asb2	1.34409266	0.45252372	0.63615807	0	1.66481172	0	2.88679167	0
Itgbl1	4.03227797	6.33533207	9.224292	4.13628002	3.32962344	3.52816357	9.62263889	0
Cyth2	66.5325865	38.9170398	41.9864326	41.3628002	41.620293	34.1055812	54.8490416	31.9567854
Clcn5	2.68818531	6.33533207	7.31581779	6.20442003	8.3240586	10.5844907	3.84905555	6.84788259
Gnai3	24.1936678	22.1736622	24.4920857	18.6132601	11.653682	17.6408179	34.6415	6.84788259
Slc23a2	59.8121232	127.611689	111.327662	99.2707205	119.866444	158.767361	50.0377222	143.805534
AI314180	63.8444012	65.1634155	78.8836006	45.4990802	56.6035985	108.197016	89.4905416	38.804668
Thap7	15.4570656	17.648425	14.3135566	20.6814001	16.6481172	9.40843619	16.3584861	13.6957652
Snx33	6.72046328	5.43028463	7.63389683	12.4088401	11.653682	3.52816357	5.77358333	2.28262753
Pex16	8.06455594	5.43028463	6.67965972	20.6814001	9.98887032	4.7042181	3.84905555	11.4131377
Gnb2l1	73.2530498	60.1856546	60.4350166	142.701661	78.2461509	31.7534722	72.1697916	98.1529838
Sumo1	40.994826	40.274611	51.2107246	35.1583802	21.6425524	25.8731995	63.5094166	29.6741579
Tmem19	19.4893435	28.961518	21.3112953	18.6132601	11.653682	21.1689814	21.1698055	22.8262753
Polr3gl	12.0968339	12.2181404	11.4508452	16.5451201	8.3240586	3.52816357	17.32075	11.4131377
Grhl3	2.01613899	1.35757116	0.63615807	2.06814001	3.32962344	3.52816357	0	2.28262753
Gm16381	7.39250961	7.69290322	4.13502745	0	0	4.7042181	7.69811111	0
Dleu2	2.01613899	5.88280835	2.22655324	2.06814001	0	2.35210905	4.81131944	0
Gm7244	2.01613899	0.45252372	0.31807903	0	1.66481172	2.35210905	0.96226389	0
Gm1821	312.501543	238.027476	294.859265	335.038682	342.951214	224.626414	302.150861	451.960251
Ubb	434.141928	352.968501	392.509529	421.900562	417.867742	391.626157	484.981	426.851348
Fdxr	2.01613899	2.71514231	1.27231614	6.20442003	1.66481172	4.7042181	0	0
Dsg1c	1.34409266	0	1.27231614	0	0	0	0	0
Msx2	1.34409266	0	1.27231614	0	0	0	0	0
Plscr2	1.34409266	0	1.27231614	0	0	0	0	0
Xirp2	1.34409266	0	1.27231614	0	0	0	0	0
Akap3	2.68818531	0	0.31807903	0	0	2.35210905	0	4.56525506
Ntrk3	41.6668724	79.1916508	54.0734359	39.2946602	71.586904	83.4998712	55.8113055	38.804668
1700023F06Ri	3.36023164	3.62018975	3.49886938	12.4088401	3.32962344	1.17605452	6.73584722	2.28262753
Ppara	6.04841696	4.97776091	6.67965972	0	9.98887032	2.35210905	0.96226389	0
Adamtsl4	2.01613899	1.35757116	3.49886938	2.06814001	6.65924688	1.17605452	1.92452778	4.56525506
Scarna13	2.01613899	1.81009488	0.9542371	2.06814001	0	3.52816357	1.92452778	4.56525506
Atad5	6.04841696	3.16766603	2.86271131	2.06814001	4.99443516	3.52816357	7.69811111	2.28262753
Kidins220	166.667489	273.77685	173.353074	76.5211804	171.475607	359.872684	167.433917	130.109769
Mtfmt	6.72046328	4.97776091	6.36158069	0	3.32962344	3.52816357	3.84905555	4.56525506
Plekha4	0.67204633	0	0	2.06814001	4.99443516	0	0	0
Ppp2r3c	22.1775288	16.7433776	18.448584	14.4769801	4.99443516	14.1126543	27.9056528	9.13051012
Nvl	8.06455594	12.2181404	12.4050823	6.20442003	4.99443516	21.1689814	13.4716944	9.13051012
Sccpdh	25.5377605	31.6766603	37.8514051	31.0221002	19.9777406	43.5140174	55.8113055	25.1089028
Tmem170b	90.7262543	102.27036	116.416927	84.7937404	104.883138	127.013889	117.396194	86.7398462
Pou3f3	28.2259458	31.6766603	21.3112953	28.9539601	28.3017992	30.5774176	36.5660278	27.3915304
Rab13	2.01613899	3.16766603	2.22655324	4.13628002	4.99443516	2.35210905	0	0
Gem	0	1.35757116	1.90847421	0	0	0	0.96226389	0
Odc1	24.8657142	15.3858064	15.9039517	35.1583802	24.9721758	11.7605452	27.9056528	43.3699231
Snrpb	15.4570656	12.2181404	18.130505	57.9079203	63.2628454	4.7042181	19.2452778	47.9351782
Dmp1	0.67204633	2.71514231	0.9542371	0	0	1.17605452	0.96226389	0
3110001I22Ri	0.67204633	0.45252372	1.90847421	0	0	0	0.96226389	0
Trip6	0.67204633	0.90504744	0.31807903	0	3.32962344	0	0.96226389	0
5730405O15R	2.01613899	1.35757116	1.59039517	12.4088401	4.99443516	2.35210905	0	2.28262753
Hydin	1.34409266	0.90504744	0.63615807	6.20442003	6.65924688	1.17605452	2.88679167	0
Dctn4	92.7423933	85.9795066	83.336707	62.0442003	71.586904	62.3308898	102.962236	47.9351782
Sec63	34.9464091	64.2583681	45.4853019	16.5451201	29.966611	101.140689	47.1509305	22.8262753
Rom1	1.34409266	1.35757116	0.63615807	6.20442003	1.66481172	0	0.96226389	2.28262753
Eif4ebp3	0.67204633	0.90504744	0.63615807	0	0	0	0	0
Pole	0.67204633	0.90504744	0.63615807	0	0	0	0	0
Prss16	0.67204633	0.90504744	0.63615807	0	0	0	0	0
Rad9b	0.67204633	0.90504744	0.63615807	0	0	0	0	0
Tnfrsf18	0.67204633	0.90504744	0.63615807	0	0	0	0	0
Zc3h12a	0.67204633	0.90504744	0.63615807	0	0	0	0	0
Rprm	32.2582238	31.6766603	26.0824808	57.9079203	49.9443516	14.1126543	19.2452778	38.804668
Lsp1	0.67204633	2.26261859	1.59039517	0	0	0	0.96226389	2.28262753
Efcab5	0	1.35757116	2.22655324	0	3.32962344	0	3.84905555	0
Bcdin3d	2.01613899	0	2.22655324	2.06814001	0	2.35210905	2.88679167	4.56525506
Rab37	1.34409266	0.45252372	1.27231614	0	0	0	0.96226389	0
Tmc3	1.34409266	0.45252372	0	0	0	2.35210905	0	2.28262753
Tmem200a	11.4247876	20.8160911	11.7689243	6.20442003	1.66481172	12.9365998	17.32075	6.84788259
1200014J11Ri	14.7850192	11.7656167	11.4508452	6.20442003	13.3184938	16.4647633	13.4716944	11.4131377
Fasn	192.20525	166.076205	164.128782	454.990802	649.276571	143.478652	93.3395972	346.959385
Gatsl2	126.34471	144.355066	110.373425	293.675881	229.744017	128.189943	105.849028	344.676757
Vps72	17.4732045	14.480759	16.5401098	43.4309402	9.98887032	8.23238167	15.3962222	38.804668
Ifi27	3.36023164	4.97776091	4.13502745	8.27256004	3.32962344	0	7.69811111	18.2610202
Tiparp	10.0806949	14.0282353	17.8124259	8.27256004	8.3240586	25.8731995	18.2830139	9.13051012
Tcfl5	2.01613899	0.90504744	0.31807903	0	0	0	0.96226389	0
Tgfb2	10.7527413	19.0059962	9.54237104	4.13628002	13.3184938	8.23238167	8.660375	6.84788259
Tspan11	0.67204633	0	1.90847421	0	0	0	0	0
Fnbp1	45.027104	54.75537	54.7095939	140.633521	73.2517157	51.7463991	36.5660278	82.1745911
Zfp574	28.2259458	23.0787097	18.766663	41.3628002	51.6091633	16.4647633	18.2830139	47.9351782
Ufd1l	50.4034746	39.8220873	37.8514051	49.6353602	43.2851047	24.697145	51.96225	31.9567854
Large	92.070347	141.639924	109.737267	39.2946602	99.8887032	201.105324	87.5660139	73.044081
Rarres2	0.67204633	0	0.63615807	4.13628002	0	1.17605452	0	0
Gcdh	10.0806949	10.4080455	9.54237104	12.4088401	14.9833055	5.88027262	11.5471667	20.5436478
9130401M01R	14.7850192	19.4585199	14.9497146	45.4990802	26.6369875	8.23238167	14.4339583	25.1089028
9030612E09Ri	0.67204633	0.45252372	0.9542371	2.06814001	3.32962344	0	0.96226389	2.28262753
Ecd	24.1936678	15.8383302	23.8559276	10.3407001	18.3129289	21.1689814	27.9056528	22.8262753
Rimbp3	0.67204633	2.71514231	2.54463228	0	0	1.17605452	1.92452778	9.13051012
Tbl1xr1	112.231737	106.343074	118.325401	74.4530404	83.240586	141.126543	140.490528	98.1529838
Cacna1c	71.9089571	127.159165	92.24292	59.9760603	148.168243	161.11947	50.9999861	61.6309433
Timeless	1.34409266	1.35757116	1.27231614	4.13628002	0	1.17605452	0.96226389	2.28262753
9330182L06Ri	34.2743627	47.0624668	31.8079035	6.20442003	14.9833055	75.2674895	45.2264028	18.2610202
Mettl10	18.8172972	11.7656167	10.1785291	18.6132601	8.3240586	16.4647633	16.3584861	2.28262753
Aamp	72.5810035	62.9007969	67.4327553	382.605902	234.738453	84.6759257	91.4150694	346.959385
Thbd	2.01613899	11.313093	6.67965972	2.06814001	11.653682	3.52816357	0.96226389	0
Tmem246	36.9625481	67.8785578	55.663831	86.8618804	51.6091633	51.7463991	28.8679167	79.8919636
Pld3	260.753975	478.317571	311.081296	264.721921	431.186236	259.90805	117.396194	315.002599
Cfl2	51.7475673	52.9452751	55.9819101	39.2946602	26.6369875	64.6829988	117.396194	29.6741579
1600012H06Ri	9.4086486	17.648425	10.4966081	10.3407001	3.32962344	14.1126543	6.73584722	15.9783927
Psmd4	41.6668724	33.0342315	42.9406697	55.8397803	34.9610461	42.3379629	55.8113055	59.3483158
Cgn	0.67204633	0.90504744	2.54463228	8.27256004	4.99443516	2.35210905	2.88679167	4.56525506
Tbca	5.37637063	3.16766603	7.63389683	10.3407001	3.32962344	9.40843619	15.3962222	2.28262753
Clec18a	0	0.90504744	1.59039517	0	0	0	0	0
2810013P06Ri	12.0968339	9.5029981	8.58813393	4.13628002	6.65924688	4.7042181	11.5471667	4.56525506
Hck	2.01613899	0	0.63615807	0	0	0	0	0
Pomgnt2	13.4409266	32.129184	20.6751372	18.6132601	33.2962344	23.5210905	8.660375	43.3699231
Btbd1	106.18332	91.4097912	74.7485731	47.5672202	34.9610461	77.6195986	112.584875	43.3699231
Sgsm1	95.4305786	72.8563187	77.2932054	237.836101	234.738453	109.373071	71.2075278	166.63181
Ago4	8.73660227	9.05047438	9.54237104	4.13628002	4.99443516	11.7605452	5.77358333	2.28262753

GeneID	Base mean	log2(FC)	StdErr	Wald-Stats	P-value	P-adj	GeneID	Normalised expression for Chow#1	Normalised expression for Chow#2	Normalised expression for Chow#3	Normalised expression for Chow#4	Normalised expression for HFD#1	Normalised expression for HFD#2	Normalised expression for HFD#3	Normalised expression for HFD#4
Scn4a	0.57206807	-0.1653069	0.28988317	-0.5702536	0.56850572	NA	Scn4a	0	1.81009488	1.59039517	0	0	1.17605452	0	0
Echdc1	27.7241308	-0.2132595	0.37400188	-0.5702097	0.56853545	0.99938111	Echdc1	32.9302701	35.7493738	34.0344567	20.6814001	13.3184938	18.8168724	54.8490416	11.4131377
Ky	0.89233215	-0.2042168	0.35822841	-0.5700744	0.56862725	NA	Ky	2.01613899	0.90504744	1.59039517	0	1.66481172	0	0.96226389	0
1810020O05Rik	0.28032585	0.12214959	0.21428513	0.57003298	0.56865533	NA	1810020O05R	0	0	0.31807903	0	0	0	1.92452778	0
4933406C10Rik	0.28032585	0.12214959	0.21428513	0.57003298	0.56865533	NA	4933406C10Ri	0	0	0.31807903	0	0	0	1.92452778	0
Agbl1	0.28032585	0.12214959	0.21428513	0.57003298	0.56865533	NA	Agbl1	0	0	0.31807903	0	0	0	1.92452778	0
Ms4a6b	0.28032585	0.12214959	0.21428513	0.57003298	0.56865533	NA	Ms4a6b	0	0	0.31807903	0	0	0	1.92452778	0
Prdm1	0.28032585	0.12214959	0.21428513	0.57003298	0.56865533	NA	Prdm1	0	0	0.31807903	0	0	0	1.92452778	0
Skap1	0.28032585	0.12214959	0.21428513	0.57003298	0.56865533	NA	Skap1	0	0	0.31807903	0	0	0	1.92452778	0
Zfp820	0.28032585	0.12214959	0.21428513	0.57003298	0.56865533	NA	Zfp820	0	0	0.31807903	0	0	0	1.92452778	0
Ciita	0.49326481	-0.1770685	0.31064535	-0.570002	0.56867634	NA	Ciita	0.67204633	1.35757116	0.9542371	0	0	0	0.96226389	0
Drg2	17.5084206	0.19949551	0.35000159	0.56998458	0.56868816	0.99938111	Drg2	18.1452509	13.5757116	16.2220308	14.4769801	11.653682	10.5844907	21.1698055	34.239413
Taz	7.80619827	-0.2226744	0.39067588	-0.5699723	0.56869646	0.99938111	Taz	9.4086486	7.69290322	12.0870033	6.20442003	4.99443516	8.23238167	11.5471667	2.28262753
Mad2l1	4.29709701	0.23662702	0.41519759	0.56991424	0.56873586	NA	Mad2l1	2.68818531	3.62018975	5.72542262	2.06814001	6.65924688	9.40843619	1.92452778	2.28262753
Myt1l	243.673155	0.16862618	0.29591236	0.56985176	0.56877824	0.99938111	Myt1l	263.442161	249.793093	261.142887	132.360961	208.101465	396.330375	257.886722	180.327575
Sox2	48.845473	0.17258106	0.30288114	0.56979796	0.56881474	0.99938111	Sox2	49.7314283	29.8665654	33.3982986	72.3849004	53.2739751	57.6266717	44.2641389	50.2178057
Utp23	5.03953515	0.23652675	0.41520253	0.56966597	0.56890427	0.99938111	Utp23	6.72046328	3.16766603	5.72542262	0	11.653682	2.35210905	3.84905555	6.84788259
6030443J06Rik	0.43462804	0.1512414	0.26549381	0.56966074	0.56890783	NA	6030443J06Ri	0	0	0.63615807	0	1.66481172	1.17605452	0	0
Fam203a	5.19913755	0.23702256	0.41634539	0.5692931	0.56915725	0.99938111	Fam203a	2.68818531	1.35757116	2.86271131	12.4088401	11.653682	3.52816357	4.81131944	2.28262753
Ncapd2	9.92851482	0.20389261	0.35815208	0.56929059	0.56915895	0.99938111	Ncapd2	8.73660227	9.95552182	8.90621297	8.27256004	11.653682	14.1126543	8.660375	9.13051012
Gm16515	9.58331611	-0.2358303	0.41433533	-0.5691774	0.56923573	0.99938111	Gm16515	13.4409266	11.313093	8.2700549	10.3407001	21.6425524	0	4.81131944	6.84788259
Alg1	11.19811	0.22283745	0.39151536	0.56916655	0.56924312	0.99938111	Alg1	9.4086486	9.95552182	10.1785291	8.27256004	16.6481172	9.40843619	2.88679167	22.8262753
Glra3	14.7698676	0.21955527	0.38576758	0.56913873	0.56926199	0.99938111	Glra3	8.73660227	21.7211385	13.6773985	8.27256004	18.3129289	29.4013631	13.4716944	4.56525506
Fbxl20	39.0994614	0.17773569	0.31232358	0.56907548	0.56930492	0.99938111	Fbxl20	24.8657142	42.5372296	31.8079035	45.4990802	61.5980337	32.9295267	27.9056528	45.6525506
Stambpl1	19.1329965	-0.1942066	0.34127031	-0.5690698	0.56930878	0.99938111	Stambpl1	24.1936678	11.7656167	18.448584	31.0221002	14.9833055	16.4647633	20.2075417	15.9783927
Arpc1b	0.64580416	0.17650229	0.31031299	0.56878796	0.56950004	NA	Arpc1b	0.67204633	0.90504744	0	0	1.66481172	0	1.92452778	0
Dnaaf2	9.54896148	0.23611009	0.41523454	0.56861863	0.56961498	0.99938111	Dnaaf2	8.73660227	4.52523719	4.77118552	14.4769801	18.3129289	1.17605452	3.84905555	20.5436478
Xrcc3	5.57617102	-0.2325815	0.40907611	-0.5685532	0.56965939	0.99938111	Xrcc3	6.72046328	3.62018975	7.95197586	6.20442003	4.99443516	1.17605452	4.81131944	9.13051012
Prox2	0.87821401	-0.1946808	0.342444	-0.5685039	0.56969283	NA	Prox2	0.67204633	0.45252372	1.90847421	2.06814001	0	0	1.92452778	0
Scamp4	33.580517	-0.2218364	0.39025202	-0.568444	0.56973354	0.99938111	Scamp4	32.2582238	37.106945	19.7209001	59.9760603	44.9499164	10.5844907	11.5471667	52.5004332
Pparg	1.40350562	0.19270635	0.3390926	0.56830006	0.56983124	NA	Pparg	2.01613899	0	1.59039517	0	6.65924688	0	0.96226389	0
Kcnb1	172.680868	0.17646509	0.3105982	0.5681459	0.5699359	0.99938111	Kcnb1	124.328571	233.502239	183.849682	95.1344405	214.760712	237.563014	109.698083	182.610202
Chn1os3	13.2095689	-0.2191395	0.38576818	-0.5680602	0.56999412	0.99938111	Chn1os3	18.1452509	9.5029981	17.1762679	12.4088401	23.3073641	4.7042181	6.73584722	13.6957652
Zfp770	33.8955642	-0.2355168	0.41478904	-0.5677991	0.57017142	0.99938111	Zfp770	47.7152893	50.6826565	41.6683535	16.5451201	9.98887032	45.8661264	58.6980972	0
Pramef8	12.7895	-0.2032129	0.35790699	-0.5677814	0.57018339	0.99938111	Pramef8	11.4247876	12.2181404	18.448584	12.4088401	9.98887032	7.05632714	12.5094306	18.2610202
AI429214	2.67142965	-0.2356835	0.41514556	-0.567713	0.57022984	NA	AI429214	3.36023164	5.43028463	3.18079035	0	1.66481172	3.52816357	1.92452778	2.28262753
Inip	11.9114354	-0.2068938	0.36446707	-0.5676612	0.57026507	0.99938111	Inip	16.1291119	12.6706641	15.5858727	6.20442003	11.653682	5.88027262	13.4716944	13.6957652
Dlst	83.6130137	0.14293709	0.25200653	0.56719597	0.57058104	0.99938111	Dlst	90.054208	72.8563187	77.9293635	74.4530404	76.5813391	62.3308898	109.698083	105.000866
Rhov	7.93156997	0.22628951	0.3990033	0.56713695	0.57062114	0.99938111	Rhov	5.37637063	4.97776091	4.77118552	14.4769801	8.3240586	3.52816357	10.5849028	11.4131377
Sars2	7.08327568	0.23606177	0.41634596	0.56698467	0.5707246	0.99938111	Sars2	5.37637063	2.71514231	5.40734359	10.3407001	18.3129289	1.17605452	1.92452778	11.4131377
5430417L22Rik	41.3221593	-0.1464436	0.25829309	-0.5669669	0.57073667	0.99938111	5430417L22Ri	37.6345944	40.7271347	44.8491439	53.7716403	34.9610461	38.8097993	43.301875	36.5220405
Gba2	43.099704	0.17961462	0.31681577	0.56693713	0.5707569	0.99938111	Gba2	39.6507334	28.0564706	38.8056422	53.7716403	73.2517157	39.9858538	27.9056528	43.3699231
Acsf3	10.5837413	0.22395201	0.39508534	0.56684463	0.57081974	0.99938111	Acsf3	9.4086486	7.69290322	7.95197586	12.4088401	26.6369875	4.7042181	6.73584722	9.13051012
Ublcp1	11.2224197	-0.2317907	0.40900278	-0.5667216	0.57090334	0.99938111	Ublcp1	18.8172972	10.4080455	14.6316356	8.27256004	3.32962344	14.1126543	20.2075417	0
Lrrtm4	22.0520578	-0.2358114	0.41621848	-0.5665567	0.57101541	0.99938111	Lrrtm4	17.4732045	51.587704	30.8536663	2.06814001	3.32962344	41.1619083	23.0943333	6.84788259
BC030500	16.1461585	0.20278001	0.35801146	0.5664065	0.57111748	0.99938111	BC030500	13.4409266	8.59795066	12.4050823	26.8858201	16.6481172	11.7605452	21.1698055	18.2610202
Thrsp	6.76490374	-0.2273502	0.40143185	-0.5663481	0.57115716	0.99938111	Thrsp	3.36023164	6.33533207	8.58813393	12.4088401	4.99443516	3.52816357	5.77358333	9.13051012
Kat2b	9.10957644	0.22754753	0.40185203	0.56624706	0.57122585	0.99938111	Kat2b	10.0806949	10.8605693	10.1785291	0	8.3240586	10.5844907	18.2830139	4.56525506
Synpr	65.542671	-0.2140351	0.37800025	-0.56623	0.57123741	0.99938111	Synpr	56.4518916	117.656167	91.924841	22.7495401	24.9721758	83.4998712	83.7169583	43.3699231
Gm16861	5.46981681	-0.2326229	0.4109029	-0.5661262	0.57130801	0.99938111	Gm16861	1.34409266	2.26261859	2.86271131	20.6814001	6.65924688	1.17605452	1.92452778	6.84788259
Acss3	0.40790421	0.14957251	0.26420475	0.56612348	0.57130984	NA	Acss3	0	0	0.63615807	0	1.66481172	0	0.96226389	0
Srpx2	0.3225872	-0.1245589	0.22012689	-0.5658505	0.57149542	NA	Srpx2	0	2.26261859	0.31807903	0	0	0	0	0
Ube2e1	24.2764668	0.19357316	0.34211563	0.56581211	0.57152152	0.99938111	Ube2e1	24.1936678	21.2686148	27.354797	16.5451201	24.9721758	19.9929269	46.1886666	13.6957652
Hist1h4i	6.9715217	-0.2335973	0.41293217	-0.5657038	0.57159514	0.99938111	Hist1h4i	9.4086486	6.78785578	4.77118552	10.3407001	4.99443516	0	5.77358333	13.6957652
Bmyc	43.6710967	0.18750792	0.33148745	0.56565618	0.57162753	0.99938111	Bmyc	45.027104	37.5594687	36.897168	39.2946602	56.6035985	17.6408179	43.301875	73.044081
Nubp1	5.21815469	0.23061307	0.40769726	0.56564783	0.57163321	0.99938111	Nubp1	4.7043243	4.07271347	5.72542262	2.06814001	4.99443516	5.88027262	2.88679167	11.4131377
Prcc	41.2489956	-0.1736406	0.30701805	-0.5655714	0.57168517	0.99938111	Prcc	53.0916599	35.7493738	36.897168	49.6353602	44.9499164	24.697145	27.9056528	57.0656883
Mir22hg	9.80018967	0.21106926	0.37323507	0.56551295	0.57172492	0.99938111	Mir22hg	11.4247876	8.14542694	8.58813393	8.27256004	8.3240586	11.7605452	17.32075	4.56525506
Ptger1	6.0685467	0.23155131	0.40958821	0.56532708	0.57185131	0.99938111	Ptger1	4.7043243	4.97776091	3.18079035	8.27256004	11.653682	4.7042181	1.92452778	9.13051012
Cwc27	7.44749301	0.22723811	0.40198855	0.56528503	0.57187991	0.99938111	Cwc27	12.0968339	6.78785578	6.36158069	0	9.98887032	8.23238167	11.5471667	4.56525506
Zfp184	2.1879748	0.22653623	0.40079489	0.56521738	0.57192592	NA	Zfp184	4.7043243	0.90504744	1.27231614	0	0	3.52816357	4.81131944	2.28262753
Snrnp25	2.36470113	-0.2334661	0.41307666	-0.5651883	0.57194571	NA	Snrnp25	4.7043243	2.71514231	1.90847421	2.06814001	0	2.35210905	2.88679167	2.28262753
Pdzd7	3.22522957	0.23520846	0.41622714	0.56509641	0.57200819	NA	Pdzd7	3.36023164	4.97776091	1.90847421	0	3.32962344	7.05632714	2.88679167	2.28262753
Gm2518	5.5278553	0.23377211	0.41372549	0.56504159	0.57204548	0.99938111	Gm2518	1.34409266	1.35757116	3.81694841	12.4088401	3.32962344	1.17605452	4.81131944	15.9783927
Lphn2	62.7558511	0.21730939	0.38462839	0.56498531	0.57208376	0.99938111	Lphn2	49.7314283	80.0966983	70.6135457	22.7495401	46.6147282	152.887088	51.96225	27.3915304
Gm13363	30.291913	0.20253104	0.35854415	0.56487058	0.5721618	0.99938111	Gm13363	27.5538995	27.6039469	40.0779583	14.4769801	18.3129289	34.1055812	59.6603611	20.5436478
Cisd1	33.3244482	-0.160358	0.2838846	-0.5648704	0.57216191	0.99938111	Cisd1	30.2420848	33.4867552	37.215247	43.4309402	34.9610461	28.2253086	38.4905555	20.5436478
Phf7	1.7048227	-0.2283556	0.40445295	-0.5646037	0.57234334	NA	Phf7	2.01613899	2.26261859	1.90847421	2.06814001	0	1.17605452	1.92452778	2.28262753
Trim24	23.6188876	0.22519503	0.39908728	0.56427515	0.57256689	0.99938111	Trim24	24.8657142	19.9110436	30.2175083	8.27256004	9.98887032	38.8097993	50.0377222	6.84788259
9530080O11Rik	1.83845033	0.22520612	0.39911338	0.56426604	0.57257309	NA	9530080O11R	1.34409266	1.35757116	2.54463228	0	3.32962344	0	3.84905555	2.28262753
Aimp1	8.83399304	0.21952323	0.38909918	0.56418322	0.57262945	0.99938111	Aimp1	13.4409266	8.14542694	7.31581779	2.06814001	6.65924688	11.7605452	14.4339583	6.84788259
Nsun7	3.14166087	0.23419177	0.41527786	0.56393994	0.572795	NA	Nsun7	0.67204633	3.16766603	2.86271131	4.13628002	0	5.88027262	3.84905555	4.56525506
Ptprc	1.13281994	-0.2068283	0.36687241	-0.5637608	0.57291691	NA	Ptprc	0.67204633	3.16766603	1.90847421	0	0	2.35210905	0.96226389	0
Ncf2	0.56943708	-0.1567131	0.27800759	-0.5637007	0.57295781	NA	Ncf2	2.68818531	0.90504744	0	0	0	0	0.96226389	0
Cdc5l	13.7275529	0.20686747	0.36704787	0.56359806	0.57302771	0.99938111	Cdc5l	15.4570656	10.8605693	14.9497146	8.27256004	4.99443516	22.345036	19.2452778	13.6957652
4930526I15Rik	6.41913327	0.23348527	0.41438397	0.56345151	0.57312747	0.99938111	4930526I15Ri	3.36023164	4.07271347	2.86271131	12.4088401	8.3240586	4.7042181	1.92452778	13.6957652
Wdr48	57.527992	0.15745854	0.2796013	0.56315383	0.57333014	0.99938111	Wdr48	64.5164475	63.3533207	55.9819101	28.9539601	46.6147282	68.2111624	66.3962083	66.1961984
Oxsr1	13.8951765	-0.2027571	0.36012912	-0.5630121	0.57342664	0.99938111	Oxsr1	13.4409266	16.7433776	17.1762679	14.4769801	6.65924688	22.345036	13.4716944	6.84788259
Ndst3	18.805943	0.23406224	0.41575556	0.5629804	0.57344823	0.99938111	Ndst3	13.4409266	30.7716129	19.0847421	0	9.98887032	34.1055812	38.4905555	4.56525506
Kctd7	18.2584222	0.19291954	0.34267712	0.56297759	0.57345014	0.99938111	Kctd7	16.1291119	14.0282353	12.4050823	24.8176801	21.6425524	12.9365998	14.4339583	29.6741579
Prn	6.71585178	0.23400102	0.41571273	0.56289115	0.57350901	0.99938111	Prn	0	7.2403795	8.90621297	6.20442003	4.99443516	8.23238167	6.73584722	11.4131377
Ccna2	5.36141811	-0.2303765	0.40931346	-0.5628363	0.57354634	0.99938111	Ccna2	7.39250961	6.33533207	2.86271131	8.27256004	1.66481172	4.7042181	4.81131944	6.84788259
Wdr5	18.0331883	-0.2032682	0.36115618	-0.5628263	0.57355315	0.99938111	Wdr5	18.8172972	14.480759	15.2677937	31.0221002	26.6369875	8.23238167	11.5471667	18.2610202
H2-Q2	1.95020382	-0.2154919	0.38310785	-0.5624837	0.57378649	NA	H2-Q2	1.34409266	1.35757116	0.9542371	6.20442003	0	1.17605452	0	4.56525506
Gp1bb	147.740545	-0.1852441	0.32933465	-0.5624798	0.57378918	0.99938111	Gp1bb	188.172972	147.975256	136.137827	163.383061	201.442218	59.9787807	88.5282777	196.305968
Tax1bp1	75.3990253	0.22177563	0.39431903	0.5624269	0.5738252	0.99938111	Tax1bp1	78.6294204	75.5714611	79.5197586	33.0902402	18.3129289	170.527906	108.735819	38.804668
Col11a2	2.40310182	0.22462643	0.3994292	0.56236859	0.57386492	NA	Col11a2	1.34409266	3.16766603	1.90847421	0	4.99443516	0	0.96226389	6.84788259
Tox	26.0880253	0.18770786	0.33379151	0.56235063	0.57387715	0.99938111	Tox	24.8657142	25.3413283	24.4920857	22.7495401	14.9833055	44.6900719	35.6037639	15.9783927
Thoc6	4.7467967	0.23174576	0.41211129	0.5623378	0.57388589	0.99938111	Thoc6	2.68818531	2.71514231	3.49886938	8.27256004	3.32962344	3.52816357	4.81131944	9.13051012
Reep5	269.984015	-0.1984447	0.35309279	-0.5620185	0.57410344	0.99938111	Reep5	235.888261	459.764098	345.751911	134.429101	141.508996	436.316228	221.320694	184.89283
Pgpep1	12.3187823	-0.2269004	0.4037257	-0.5620162	0.57410499	0.99938111	Pgpep1	17.4732045	7.69290322	8.2700549	22.7495401	14.9833055	1.17605452	12.5094306	13.6957652
Trappc11	43.5601232	0.16338736	0.29071728	0.56201459	0.57410608	0.99938111	Trappc11	40.3227797	32.129184	44.5310648	47.5672202	61.5980337	56.4506172	38.4905555	27.3915304
Neurod6	25.2233046	0.21686416	0.38604993	0.56175157	0.57428529	0.99938111	Neurod6	30.2420848	27.1514231	25.7644018	6.20442003	16.6481172	18.8168724	58.6980972	18.2610202
Pms2	8.49140675	0.21667777	0.38576955	0.5616767	0.57433631	0.99938111	Pms2	8.06455594	6.78785578	11.7689243	2.06814001	6.65924688	10.5844907	10.5849028	11.4131377
Pcnxl4	36.2391263	0.18671425	0.33246915	0.56159873	0.57438944	0.99938111	Pcnxl4	23.5216215	38.4645161	34.9886938	37.2265202	31.6314227	69.3872169	25.0188611	29.6741579
Stard13	4.39639722	-0.2304147	0.41035053	-0.5615069	0.57445203	NA	Stard13	4.7043243	7.69290322	5.08926455	2.06814001	4.99443516	3.52816357	4.81131944	2.28262753
1110046J04Rik	1.17424249	-0.1932508	0.34417147	-0.5614955	0.57445976	NA	1110046J04Ri	0	2.26261859	3.81694841	0	0	2.35210905	0.96226389	0
Fam229b	4.26342097	-0.2334493	0.41590611	-0.561303	0.57459103	NA	Fam229b	7.39250961	4.97776091	3.81694841	4.13628002	1.66481172	1.17605452	8.660375	2.28262753
Zfp263	9.30306354	0.21063814	0.37530453	0.56124594	0.57462989	0.99938111	Zfp263	6.72046328	9.05047438	6.36158069	12.4088401	6.65924688	14.1126543	7.69811111	11.4131377
Bbx	36.9218403	0.18541917	0.33037331	0.56124137	0.57463301	0.99938111	Bbx	36.9625481	40.7271347	41.0321955	16.5451201	39.9554813	47.042181	54.8490416	18.2610202
Cep170	98.9924614	0.20231567	0.36064145	0.56098841	0.57480544	0.99938111	Cep170	105.511274	98.6501707	101.467212	53.7716403	48.2795399	199.929269	131.830153	52.5004332
BC039966	0.7755525	-0.1184698	0.21119967	-0.5609373	0.57484027	NA	BC039966	0	0	0	6.20442003	0	0	0	0
Btla	0.7755525	-0.1184698	0.21119967	-0.5609373	0.57484027	NA	Btla	0	0	0	6.20442003	0	0	0	0
Foxa1	0.7755525	-0.1184698	0.21119967	-0.5609373	0.57484027	NA	Foxa1	0	0	0	6.20442003	0	0	0	0
Hmx3	0.7755525	-0.1184698	0.21119967	-0.5609373	0.57484027	NA	Hmx3	0	0	0	6.20442003	0	0	0	0
Trim58	0.7755525	-0.1184698	0.21119967	-0.5609373	0.57484027	NA	Trim58	0	0	0	6.20442003	0	0	0	0
Uqcc2	10.3059578	-0.228026	0.40652926	-0.5609091	0.5748595	0.99938111	Uqcc2	10.0806949	6.78785578	9.54237104	20.6814001	18.3129289	1.17605452	6.73584722	9.13051012
Ecm2	0.92853116	-0.173179	0.30883258	-0.5607538	0.57496541	NA	Ecm2	0	3.16766603	1.90847421	0	0	2.35210905	0	0
Asic1	46.7431388	-0.1828065	0.32600445	-0.5607484	0.57496908	0.99938111	Asic1	38.3066407	67.4260341	40.7141164	53.7716403	54.9387868	47.042181	19.2452778	52.5004332
Mpc2	18.6925546	0.1991735	0.35519642	0.56074187	0.57497352	0.99938111	Mpc2	12.7688802	18.5534725	21.3112953	14.4769801	9.98887032	12.9365998	29.8301805	29.6741579
Trpm4	3.68280925	-0.2287669	0.40801888	-0.5606772	0.5750176	NA	Trpm4	2.01613899	2.26261859	1.90847421	12.4088401	6.65924688	0	1.92452778	2.28262753
Zfp85os	4.8790908	-0.2313245	0.41258317	-0.5606736	0.57502008	0.99938111	Zfp85os	3.36023164	5.43028463	9.54237104	4.13628002	1.66481172	5.88027262	6.73584722	2.28262753

GeneID	Base mean	log2(FC)	StdErr	Wald-Stats	P-value	P-adj		GeneID	Normalised expression for Chow#1	Normalised expression for Chow#2	Normalised expression for Chow#3	Normalised expression for Chow#4	Normalised expression for HFD#1	Normalised expression for HFD#2	Normalised expression for HFD#3	Normalised expression for HFD#4
Enkur	0.71228787	0.19465784	0.34721132	0.56063218	0.57504831	NA		Enkur	0.67204633	0.90504744	0.31807903	0	1.66481172	1.17605452	0.96226389	0
2310009B15Rik	1.59715974	0.2203046	0.39297208	0.56061132	0.57506253	NA		2310009B15Ri	3.36023164	0.45252372	0.9542371	0	1.66481172	1.17605452	2.88679167	2.28262753
Pacs1	103.593785	0.16645925	0.29693728	0.56058725	0.57507894	0.99938111		Pacs1	97.4467176	78.7391271	87.4717345	122.020261	139.844185	65.8590533	79.8679027	157.5013
Arr3	0.59655476	-0.1356639	0.24202866	-0.5605283	0.57511914	NA		Arr3	0	0	0.63615807	4.13628002	0	0	0	0
Gng8	0.59655476	-0.1356639	0.24202866	-0.5605283	0.57511914	NA		Gng8	0	0	0.63615807	4.13628002	0	0	0	0
Akip1	1.17013551	0.18764318	0.33480676	0.56045219	0.57517104	NA		Akip1	2.01613899	1.35757116	0	0	0	1.17605452	4.81131944	0
Surf4	38.3149901	-0.2102933	0.37524081	-0.5604223	0.57519146	0.99938111		Surf4	34.2743627	64.2583681	52.1649617	14.4769801	41.620293	28.2253086	14.4339583	57.0656883
Epb4.1l2	27.2508544	-0.2229589	0.39789005	-0.5603531	0.57523865	0.99938111		Epb4.1l2	39.6507334	41.1796584	38.1694841	2.06814001	29.966611	27.0492541	30.7924444	9.13051012
Bap1	99.9663322	0.11159822	0.19918211	0.56028234	0.57528687	0.99938111		Bap1	98.1187639	90.0522201	94.1513942	101.338861	103.218327	99.9646345	101.037708	111.848749
Ppp1r7	71.8438766	0.20044424	0.3578217	0.56017912	0.57535727	0.99938111		Ppp1r7	94.7585323	61.0907021	79.5197586	24.8176801	39.9554813	81.1477622	131.830153	61.6309433
Dcaf8	93.2828528	0.14155681	0.25273195	0.56010649	0.5754068	0.99938111		Dcaf8	90.054208	65.6159392	76.3389683	124.088401	96.5590798	89.3801438	92.3773333	111.848749
Rab11fip1	3.11809374	-0.2234627	0.3990034	-0.5600522	0.57544384	NA		Rab11fip1	3.36023164	1.81009488	0.63615807	10.3407001	6.65924688	1.17605452	0.96226389	0
Loxl1	0.81623867	0.18851829	0.33675929	0.55980133	0.57561496	NA		Loxl1	0.67204633	0.90504744	0.31807903	0	0	2.35210905	0	2.28262753
Rab35	68.3521526	-0.1625599	0.29044796	-0.5596867	0.57569316	0.99938111		Rab35	67.2046328	63.3533207	49.6203294	113.747701	73.2517157	51.7463991	54.8490416	73.044081
Rtp1	1.10626121	0.2054034	0.36704192	0.55961838	0.57573976	NA		Rtp1	0	1.81009488	0.9542371	0	1.66481172	1.17605452	0.96226389	2.28262753
Mblac1	4.04008935	0.23196274	0.41452236	0.55959043	0.57575883	NA		Mblac1	1.34409266	1.81009488	1.59039517	10.3407001	8.3240586	4.7042181	1.92452778	2.28262753
Cdh15	3.32939531	0.21529305	0.38481952	0.559465	0.57584441	NA		Cdh15	1.34409266	0.45252372	2.22655324	6.20442003	4.99443516	0	0	11.4131377
Mtmr2	22.077885	-0.1773757	0.31717681	-0.5592329	0.57600276	0.99938111		Mtmr2	29.5700384	24.8888045	26.7186389	12.4088401	19.9777406	18.8168724	25.981125	18.2610202
Top2b	85.6807535	0.18105113	0.32381658	0.55911632	0.57608234	0.99938111		Top2b	100.134903	82.3593168	88.4259716	45.4990802	71.586904	130.542052	121.24525	45.6525506
Impdh1	61.5100491	0.22087893	0.39521057	0.55888923	0.57623732	0.99938111		Impdh1	44.3550577	37.5594687	32.7621406	103.407001	101.553515	27.0492541	22.1320694	123.261887
Aspscr1	22.2070365	0.20801968	0.37229416	0.55875087	0.57633176	0.99938111		Aspscr1	19.4893435	12.2181404	12.7231614	37.2265202	34.9610461	11.7605452	17.32075	31.9567854
Wapal	44.4621274	0.2022979	0.3621912	0.55853897	0.5764764	0.99938111		Wapal	45.027104	44.3473245	43.8949068	28.9539601	26.6369875	83.4998712	67.3584722	15.9783927
Rhob	156.879898	-0.1334085	0.2388739	-0.5584893	0.5765103	0.99938111		Rhob	139.785636	145.260114	159.357596	217.154701	178.134854	112.901234	147.226375	155.218672
B930041F14Rik	32.5680793	-0.215201	0.38538465	-0.5584057	0.57656741	0.99938111		B930041F14Ri	22.8495752	26.6988994	22.9016905	72.3849004	31.6314227	12.9365998	16.3584861	54.7830607
Ak1	120.255402	0.20945194	0.37520628	0.55823144	0.57668635	0.99938111		Ak1	91.3983007	56.1129411	63.6158069	223.359121	171.475607	71.739326	78.9056389	205.436478
Hnrnpl	97.3104366	0.12583924	0.22543198	0.55821378	0.57669841	0.99938111		Hnrnpl	100.806949	83.716888	108.146872	76.5211804	91.5646446	97.6125255	117.396194	102.718239
Glyctk	5.01503241	0.22259866	0.39892134	0.55800139	0.57684344	0.99938111		Glyctk	0	2.26261859	1.90847421	12.4088401	9.98887032	1.17605452	0.96226389	11.4131377
C1d	16.9692176	-0.2193364	0.39308342	-0.5579893	0.57685167	0.99938111		C1d	20.8334362	24.8888045	23.5378486	6.20442003	3.32962344	17.6408179	27.9056528	11.4131377
Abce1	51.4619726	0.20445116	0.36643519	0.5579463	0.57688105	0.99938111		Abce1	55.1077989	57.4705123	50.8926455	22.7495401	23.3073641	68.2111624	101.999972	31.9567854
Map3k7	29.4842716	0.15493477	0.27776034	0.55780019	0.57698083	0.99938111		Map3k7	25.5377605	25.793852	31.1717454	28.9539601	26.6369875	30.5774176	37.5282917	29.6741579
Adamts18	0.97636008	-0.1813162	0.32505986	-0.5577932	0.57698561	NA		Adamts18	2.01613899	3.16766603	0	0	1.66481172	0	0.96226389	0
Fam63a	11.7536835	-0.2001382	0.35881864	-0.5577699	0.57700151	0.99938111		Fam63a	11.4247876	11.313093	11.4508452	18.6132601	8.3240586	7.05632714	14.4339583	11.4131377
Map4k3	56.4259434	0.18445171	0.33071503	0.55773609	0.57702461	0.99938111		Map4k3	58.4680306	64.7108918	55.345752	28.9539601	31.6314227	82.3238167	86.60375	43.3699231
9430037G07Rik	1.06000045	0.18711092	0.3354851	0.55773242	0.57702711	NA		9430037G07R	0	0.90504744	1.90847421	0	0	4.7042181	0.96226389	0
2010015L04Rik	5.21097289	0.23219333	0.4163612	0.55767283	0.57706782	0.99938111		2010015L04Ri	5.37637063	6.33533207	4.13502745	2.06814001	0	7.05632714	14.4339583	2.28262753
1810044D09Rik	0.42204305	-0.1476502	0.26476466	-0.5576659	0.57707253	NA		1810044D09Ri	0.67204633	0	0.63615807	2.06814001	0	0	0	0
Ifih1	1.80511752	-0.2226107	0.39920544	-0.5576344	0.57709405	NA		Ifih1	3.36023164	2.71514231	1.27231614	2.06814001	0	1.17605452	3.84905555	0
Phf3	74.2642105	0.146422	0.26259175	0.55760321	0.57711536	0.99938111		Phf3	82.6616984	70.1411764	74.112415	51.7035003	53.2739751	101.140689	78.9056389	82.1745911
Selm	58.6626018	0.19915214	0.35726534	0.55743482	0.57723038	0.99938111		Selm	37.6345944	32.5817078	39.7598793	105.475141	54.9387868	43.5140174	48.1131944	107.283494
Sowaha	79.0155385	0.17787513	0.31910605	0.55741697	0.57724257	0.99938111		Sowaha	80.6455594	69.236129	73.794336	66.1804803	136.514561	49.39429	49.0754583	107.283494
Rab34	4.9482164	-0.2316879	0.41564984	-0.5574112	0.57724652	0.99938111		Rab34	10.0806949	3.16766603	3.18079035	6.20442003	8.3240586	1.17605452	2.88679167	4.56525506
Adcyap1	6.49904038	-0.2313454	0.41514092	-0.5572696	0.57734327	0.99938111		Adcyap1	1.34409266	14.0282353	7.31581779	8.27256004	4.99443516	14.1126543	1.92452778	0
2810454H06Rik	0.7185451	0.1785824	0.32050669	0.55718773	0.57739917	NA		2810454H06Ri	0	0.90504744	0.63615807	0	0	0	1.92452778	2.28262753
Tmem80	10.0468772	-0.2058665	0.36948585	-0.5571702	0.57741113	0.99938111		Tmem80	12.0968339	9.95552182	9.224292	12.4088401	4.99443516	7.05632714	8.660375	15.9783927
Cenpv	18.2707604	-0.2091794	0.37547286	-0.5571094	0.57745272	0.99938111		Cenpv	13.4409266	15.3858064	14.9497146	37.2265202	18.3129289	11.7605452	7.69811111	27.3915304
Nme4	0.96955913	-0.2007496	0.36034272	-0.5571074	0.57745404	NA		Nme4	2.01613899	0.90504744	1.59039517	0	0	0	0.96226389	2.28262753
BC068281	6.31687376	-0.2248215	0.40355235	-0.5571063	0.57745482	0.99938111		BC068281	8.73660227	10.4080455	3.49886938	6.20442003	3.32962344	7.05632714	6.73584722	4.56525506
Sema7a	31.8917502	0.16547064	0.29710512	0.55694306	0.57756633	0.99938111		Sema7a	34.9464091	31.6766603	23.8559276	26.8858201	41.620293	32.9295267	22.1320694	41.0872956
Kdm1a	36.213001	-0.1603215	0.28794293	-0.5567822	0.57767627	0.99938111		Kdm1a	42.3389187	34.8443264	43.8949068	33.0902402	26.6369875	49.39429	29.8301805	29.6741579
Brca1	0.78699159	-0.1956509	0.35149864	-0.5566193	0.57778757	NA		Brca1	1.34409266	0.90504744	1.90847421	0	0	1.17605452	0.96226389	0
Ptpmt1	13.1344348	-0.1953293	0.35095886	-0.556559	0.57782875	0.99938111		Ptpmt1	16.1291119	11.7656167	14.3135566	16.5451201	8.3240586	10.5844907	18.2830139	9.13051012
Tspan18	2.9067499	0.22854376	0.41076081	0.55639134	0.57794336	NA		Tspan18	0.67204633	5.88280835	0.9542371	2.06814001	1.66481172	5.88027262	3.84905555	2.28262753
Eif2ak1	59.0108767	-0.16642	0.29918387	-0.5562466	0.57804228	0.99938111		Eif2ak1	63.8444012	71.0462239	81.1101538	35.1583802	44.9499164	71.739326	65.4339444	38.804668
Man2c1	18.2374632	0.22184163	0.39882731	0.55623479	0.57805036	0.99938111		Man2c1	10.0806949	9.5029981	11.4508452	35.1583802	39.9554813	7.05632714	14.4339583	18.2610202
Dr1	57.9220006	0.16243922	0.2920669	0.55617127	0.57809378	0.99938111		Dr1	53.7637063	49.3250854	54.3915149	57.9079203	33.2962344	58.8027262	57.7358333	98.1529838
Osgep	4.53360704	-0.2288758	0.41159266	-0.5560734	0.57816064	NA		Osgep	6.72046328	5.43028463	3.81694841	4.13628002	3.32962344	1.17605452	4.81131944	6.84788259
Mis18a	3.99699149	0.23009424	0.41386379	0.55596611	0.57823402	NA		Mis18a	6.04841696	2.26261859	4.77118552	0	4.99443516	2.35210905	11.5471667	0
Stt3b	52.8848866	-0.1809231	0.32542944	-0.555952	0.57824367	0.99938111		Stt3b	49.7314283	87.3370778	62.6615698	24.8176801	54.9387868	56.4506172	34.6415	52.5004332
Atp8a2	25.3578619	0.2044772	0.36788195	0.55582285	0.57833196	0.99938111		Atp8a2	25.5377605	26.2463757	23.8559276	16.5451201	26.6369875	54.0985081	23.0943333	6.84788259
Rragb	76.6724051	0.15753465	0.2835096	0.55565897	0.57844401	0.99938111		Rragb	70.5648645	70.5937002	81.4282328	66.1804803	46.6147282	101.140689	110.660347	66.1961984
Poll	4.61664423	-0.2302136	0.41440941	-0.5555222	0.5785375	NA		Poll	6.04841696	4.97776091	3.49886938	6.20442003	8.3240586	2.35210905	0.96226389	4.56525506
Maged1	426.176533	0.11419734	0.20557462	0.5555031	0.57855059	0.99938111		Maged1	407.260075	351.158406	385.193711	490.149182	417.867742	445.724665	373.358389	538.700097
Zfp40	7.525901	0.22961156	0.41355534	0.55521362	0.57874855	0.99938111		Zfp40	9.4086486	6.33533207	8.90621297	0	1.66481172	22.345036	11.5471667	0
Slc7a7	1.21203139	0.2042418	0.36793358	0.55510507	0.5788228	NA		Slc7a7	0	1.35757116	1.90847421	0	3.32962344	1.17605452	1.92452778	0
Zbtb22	31.4386574	0.227634	0.41026963	0.55484001	0.5790041	0.99938111		Zbtb22	16.1291119	14.0282353	12.0870033	68.2486203	54.9387868	9.40843619	17.32075	59.3483158
Abl1	51.2419427	0.17921249	0.32309234	0.55467885	0.57911435	0.99938111		Abl1	39.6507334	45.7048956	37.8514051	66.1804803	76.5813391	38.8097993	29.8301805	75.3267085
Syncrip	36.3692503	0.22524333	0.40615051	0.55458095	0.57918132	0.99938111		Syncrip	36.9625481	39.3695635	42.3045116	8.27256004	18.3129289	70.5632714	68.3207361	6.84788259
Cyb5r3	129.498979	0.22075726	0.39813658	0.55447621	0.57925298	0.99938111		Cyb5r3	75.9412351	66.068463	67.7508343	248.176801	149.833055	62.3308898	57.7358333	308.154717
Arid5b	34.496648	0.16973935	0.30615879	0.55441607	0.57929413	0.99938111		Arid5b	35.6184554	36.6544212	35.6248519	18.6132601	23.3073641	51.7463991	35.6037639	38.804668
Rap2b	64.8825105	-0.1325006	0.23905386	-0.5542708	0.57939354	0.99938111		Rap2b	70.5648645	65.6159392	69.6593086	66.1804803	78.2461509	48.2182355	63.5094166	57.0656883
Tmem160	18.8418319	0.22709723	0.40973611	0.55425242	0.57940611	0.99938111		Tmem160	4.7043243	1.35757116	4.77118552	51.7035003	29.966611	2.35210905	12.5094306	43.3699231
Mre11a	4.86911453	0.22965073	0.4143547	0.55423706	0.57941662	0.99938111		Mre11a	6.04841696	5.88280835	4.13502745	0	4.99443516	11.7605452	3.84905555	2.28262753
Spag1	3.15400801	0.23073931	0.41636736	0.55417243	0.57946084	NA		Spag1	5.37637063	2.71514231	2.22655324	0	3.32962344	3.52816357	5.77358333	2.28262753
Elfn2	93.9880159	0.16710316	0.30155844	0.55413193	0.57948856	0.99938111		Elfn2	81.9896521	104.985503	65.2062021	97.2025805	144.83862	92.9083074	52.9245139	111.848749
Gm12709	7.23393181	0.22599164	0.40786975	0.55407796	0.57952549	0.99938111		Gm12709	6.72046328	8.14542694	6.36158069	4.13628002	0	14.1126543	11.5471667	6.84788259
4933412O06Rik	1.73674906	-0.222797	0.40214954	-0.5540152	0.57956845	NA		4933412O06R	3.36023164	1.81009488	1.27231614	2.06814001	0	1.17605452	1.92452778	2.28262753
Eva1c	1.1779249	-0.1934884	0.34930434	-0.553925	0.57963017	NA		Eva1c	0	1.81009488	0.63615807	4.13628002	1.66481172	1.17605452	0	0
Spats1	1.27382008	-0.2004364	0.36188648	-0.5538653	0.57967101	NA		Spats1	0.67204633	0.90504744	1.59039517	4.13628002	0	0	2.88679167	0
Nipa1	30.3791684	0.17063426	0.30824073	0.55357468	0.57986996	0.99938111		Nipa1	22.1775288	38.4645161	33.7163777	16.5451201	29.966611	38.8097993	33.6792361	29.6741579
Luzp1	61.9959089	0.20032688	0.36194903	0.55346709	0.57994361	0.99938111		Luzp1	41.6668724	75.5714611	61.0711746	47.5672202	51.6091633	134.070216	61.5848889	22.8262753
Lysmd1	7.32545284	0.21715552	0.3924731	0.55330038	0.58005774	0.99938111		Lysmd1	6.04841696	4.07271347	6.67965972	10.3407001	13.3184938	5.88027262	7.69811111	4.56525506
Rpl14-ps1	56.6914649	-0.1873561	0.3386224	-0.5532891	0.58006546	0.99938111		Rpl14-ps1	73.9250961	44.3473245	43.5768277	86.8618804	28.3017992	42.3379629	88.5282777	45.6525506
Gm9962	2.56171625	0.22815308	0.41238392	0.55325407	0.58008944	NA		Gm9962	3.36023164	1.81009488	3.18079035	0	1.66481172	4.7042181	5.77358333	0
Cope	29.2121262	0.16983809	0.30700313	0.55321288	0.58011765	0.99938111		Cope	28.2259458	18.1009488	26.4005599	37.2265202	38.2906696	22.345036	28.8679167	34.239413
Fcho2	15.6904652	-0.2135075	0.38596045	-0.5531849	0.58013682	0.99938111		Fcho2	21.5054825	24.4362808	20.6751372	2.06814001	13.3184938	16.4647633	20.2075417	6.84788259
Ssh2	35.3357841	0.16463054	0.29779899	0.55282438	0.58038367	0.99938111		Ssh2	38.3066407	32.5817078	36.2610099	24.8176801	31.6314227	56.4506172	37.5282917	25.1089028
Abca2	139.317358	0.18834493	0.34080712	0.55264376	0.58050737	0.99938111		Abca2	74.5971425	190.512486	114.826531	130.292821	218.090335	177.584233	62.5471528	146.088162
Tbc1d25	14.8215062	0.21094893	0.38172724	0.55261691	0.58052576	0.99938111		Tbc1d25	16.1291119	7.69290322	14.6316356	14.4769801	31.6314227	5.88027262	14.4339583	13.6957652
Tank	13.9686072	-0.2270648	0.4109027	-0.5525999	0.58053744	0.99938111		Tank	22.1775288	23.0787097	16.2220308	2.06814001	3.32962344	15.2887088	25.0188611	4.56525506
L3mbtl4	1.12701413	0.1988841	0.35997747	0.55249042	0.58061239	NA		L3mbtl4	2.01613899	0	1.27231614	0	1.66481172	1.17605452	2.88679167	0
D8Ertd738e	21.8901295	-0.18029	0.32639492	-0.5523677	0.58069643	0.99938111		D8Ertd738e	26.8818531	15.3858064	22.9016905	31.0221002	26.6369875	16.4647633	22.1320694	13.6957652
Etaa1	6.78509862	-0.2275935	0.4120426	-0.5523543	0.5807056	0.99938111		Etaa1	8.73660227	8.59795066	11.7689243	2.06814001	3.32962344	8.23238167	11.5471667	0
Ntan1	28.7440728	-0.1838119	0.3328556	-0.5522273	0.58079263	0.99938111		Ntan1	35.6184554	21.2686148	33.3982986	35.1583802	18.3129289	16.4647633	42.3396111	27.3915304
Mansc1	5.31032342	-0.2297558	0.41614282	-0.5521081	0.58087427	0.99938111		Mansc1	0.67204633	10.8605693	8.90621297	4.13628002	3.32962344	9.40843619	2.88679167	2.28262753
Stam	45.6967955	0.14976202	0.27133861	0.55193773	0.58099101	0.99938111		Stam	31.5861774	39.8220873	48.6660923	53.7716403	36.6258578	52.9224536	51.96225	50.2178057
Tcta	17.7531058	0.18746199	0.33964713	0.55193162	0.5809952	0.99938111		Tcta	12.7688802	13.5757116	16.2220308	22.7495401	13.3184938	14.1126543	17.32075	31.9567854
Galnt7	6.03921281	-0.2183601	0.39575036	-0.5517621	0.58111134	0.99938111		Galnt7	8.73660227	4.52523719	7.63389683	6.20442003	4.99443516	5.88027262	5.77358333	4.56525506
Mob4	48.6608714	0.22088579	0.40040558	0.55165512	0.58118467	0.99938111		Mob4	40.994826	43.8948007	71.8858618	14.4769801	19.9777406	85.8519803	96.2263889	15.9783927
Zfp598	22.8299221	-0.2136781	0.3873521	-0.5516378	0.58119654	0.99938111		Zfp598	18.8172972	18.1009488	16.8581888	47.5672202	21.6425524	14.1126543	6.73584722	38.804668
Arl16	15.7573957	0.20712569	0.3754963	0.55160515	0.58121891	0.99938111		Arl16	20.8334362	14.0282353	11.7689243	8.27256004	6.65924688	12.9365998	17.32075	34.239413
Vwc2l	11.9692245	-0.2185645	0.39627794	-0.5515435	0.58126115	0.99938111		Vwc2l	10.0806949	15.3858064	16.2220308	12.4088401	1.66481172	23.5210905	9.62263889	6.84788259
Unc5c	38.679886	0.21778086	0.3948958	0.55148944	0.58129821	0.99938111		Unc5c	18.8172972	58.8280835	42.6225906	16.5451201	31.6314227	88.2040893	41.3773472	11.4131377
Ino80d	48.2105319	0.14476866	0.26250533	0.55148848	0.58129886	0.99938111		Ino80d	38.978687	52.4927514	49.3022503	41.3628002	59.9332219	56.4506172	52.9245139	34.239413
Snx14	24.6861177	-0.2187914	0.39676753	-0.5514347	0.58133573	0.99938111		Snx14	15.4570656	48.8725616	29.5813502	16.5451201	6.65924688	45.8661264	23.0943333	11.4131377
Acvr1	10.6223878	0.21917747	0.39748692	0.55140802	0.58135401	0.99938111		Acvr1	7.39250961	18.5534725	8.58813393	2.06814001	9.98887032	19.9929269	11.5471667	6.84788259
Sdcbp	80.6158925	0.17797771	0.32280554	0.55134652	0.58139615	0.99938111		Sdcbp	90.7262543	72.8563187	87.1536555	47.5672202	48.2795399	95.2604165	141.452792	61.6309433
Megf10	11.5983738	-0.2236443	0.40570814	-0.5512443	0.58146624	0.99938111		Megf10	12.7688802	23.0787097	13.3593194	2.06814001	13.3184938	18.8168724	4.81131944	4.56525506
Zfp433	2.8028202	0.22912209	0.41572218	0.55114232	0.58153612	NA		Zfp433	2.01613899	2.71514231	2.86271131	2.06814001	0	4.7042181	5.77358333	2.28262753
Psme3	117.824235	0.12021624	0.21813255	0.55111552	0.58155449	0.99938111		Psme3	113.575829	101.365313	109.101109	126.156541	121.531256	96.436471	126.056569	148.37079
Klhl7	47.7354225	0.20178835	0.36616847	0.55108064	0.5815784	0.99938111		Klhl7	54.4357526	39.8220873	52.1649617	26.8858201	18.3129289	89.3801438	71.2075278	29.6741579
Lsm14b	70.7263027	-0.1405346	0.25518935	-0.5507072	0.58183442	0.99938111		Lsm14b	92.070347	74.2138899	78.8836006	51.7035003	53.2739751	70.5632714	78.9056389	66.1961984
Hist1h2be	8.41021988	-0.2090356	0.3796656	-0.5505781	0.58192293	0.99938111		Hist1h2be	12.0968339	9.05047438	8.2700549	8.27256004	9.98887032	10.5844907	6.73584722	2.28262753

GeneID	Base mean	log2(FC)	StdErr	Wald-Stats	P-value	P-adj		GeneID	Normalised expression for Chow#1	Normalised expression for Chow#2	Normalised expression for Chow#3	Normalised expression for Chow#4	Normalised expression for HFD#1	Normalised expression for HFD#2	Normalised expression for HFD#3	Normalised expression for HFD#4
Mmrn2	0.61469446	-0.1706037	0.30987271	-0.5505605	0.58193502	NA		Mmrn2	0.67204633	2.26261859	0.31807903	0	1.66481172	0	0	0
Mir8099-1	0.66698223	0.15262946	0.27722867	0.55055438	0.58193919	NA		Mir8099-1	0	0.45252372	0.31807903	0	0	0	0	4.56525506
Mir8099-2	0.66698223	0.15262946	0.27722867	0.55055438	0.58193919	NA		Mir8099-2	0	0.45252372	0.31807903	0	0	0	0	4.56525506
Pcdhb18	4.89195516	0.22870682	0.41550001	0.55043759	0.58201927	0.99938111		Pcdhb18	3.36023164	6.33533207	6.36158069	0	8.3240586	7.05632714	7.69811111	0
Celf3	105.357769	-0.1911963	0.34736256	-0.550423	0.58202931	0.99938111		Celf3	116.264015	95.9350284	92.8790781	150.974221	121.531256	36.4576902	64.4716805	164.349182
Adamts9	2.35175209	0.22561436	0.4099097	0.55040017	0.58204494	NA		Adamts9	1.34409266	3.62018975	2.22655324	0	4.99443516	4.7042181	1.92452778	0
Wdr81	21.7302682	0.22501243	0.40891494	0.55026707	0.58213621	0.99938111		Wdr81	13.4409266	14.9332827	12.0870033	35.1583802	34.9610461	8.23238167	4.81131944	50.2178057
Osbpl11	13.0827385	0.19396148	0.35250575	0.55023637	0.58215726	0.99938111		Osbpl11	12.7688802	10.4080455	11.7689243	12.4088401	8.3240586	18.8168724	9.62263889	20.5436478
Cnot6	45.3457584	-0.1960322	0.35628523	-0.5502114	0.58217442	0.99938111		Cnot6	47.7152893	64.2583681	61.0711746	24.8176801	24.9721758	65.8590533	55.8113055	18.2610202
Fam171a1	51.2222189	-0.2042358	0.37148404	-0.5497835	0.58246785	0.99938111		Fam171a1	30.2420848	98.6501707	65.8423601	28.9539601	41.620293	82.3238167	27.9056528	34.239413
Arhgap35	316.447996	0.10259578	0.18662235	0.54975074	0.58249035	0.99938111		Arhgap35	276.211041	308.621176	305.673952	328.834262	321.308662	382.21772	280.018792	328.698364
Atrnl1	79.0166844	0.19070554	0.34689589	0.54974862	0.5824918	0.99938111		Atrnl1	65.8605402	105.438027	85.8813393	31.0221002	66.5924688	136.422325	95.264125	45.6525506
Coro6	5.42746684	0.22216654	0.40414946	0.54971381	0.58251568	0.99938111		Coro6	3.36023164	3.62018975	6.99773876	4.13628002	6.65924688	4.7042181	4.81131944	9.13051012
Slc25a46	91.6659243	0.15766715	0.28690717	0.54954066	0.58263447	0.99938111		Slc25a46	81.9896521	80.0966983	110.055346	72.3849004	74.9165274	104.668853	145.301847	63.9135709
Usf1	24.7707575	0.21938899	0.39923209	0.54952744	0.58264354	0.99938111		Usf1	18.1452509	11.7656167	9.86045007	49.6353602	46.6147282	10.5844907	17.32075	34.239413
Bri3	21.2832138	-0.2212302	0.40265174	-0.5494332	0.58270822	0.99938111		Bri3	20.1613899	13.1231878	16.5401098	45.4990802	23.3073641	7.05632714	5.77358333	38.804668
Trhr2	1.16042536	0.2043924	0.37203386	0.54939193	0.58273651	NA		Trhr2	0.67204633	1.35757116	0.9542371	0	1.66481172	2.35210905	0	2.28262753
Tmem134	12.7138112	-0.2066002	0.37606095	-0.5493796	0.58274499	0.99938111		Tmem134	14.1129729	10.8605693	15.2677937	14.4769801	21.6425524	5.88027262	5.77358333	13.6957652
Gemin5	16.3934625	0.1895581	0.34508834	0.5493031	0.58279746	0.99938111		Gemin5	20.1613899	11.313093	10.4966081	18.6132601	14.9833055	14.1126543	16.3584861	25.1089028
Usp10	51.325899	-0.1574916	0.28674115	-0.5492468	0.58283611	0.99938111		Usp10	47.043243	57.4705123	43.8949068	70.3167603	31.6314227	42.3379629	47.1509305	70.7614535
Zmiz1	205.609619	-0.1689846	0.30773924	-0.5491163	0.58292563	0.99938111		Zmiz1	205.646176	252.508235	192.119737	227.495401	304.660545	105.844907	126.056569	230.545381
Zgrf1	1.35033145	0.19111142	0.34806048	0.54907533	0.58295376	NA		Zgrf1	0	2.26261859	1.59039517	0	0	1.17605452	5.77358333	0
Spice1	4.67079609	0.22771837	0.41476648	0.54902788	0.58298631	0.99938111		Spice1	5.37637063	5.88280835	2.86271131	2.06814001	4.99443516	2.35210905	11.5471667	2.28262753
Ccdc97	62.0582137	0.20431686	0.37214513	0.54902468	0.58298852	0.99938111		Ccdc97	44.3550577	50.6826565	40.0779583	88.9300204	101.553515	28.2253086	30.7924444	111.848749
Cyp46a1	101.615598	-0.1809467	0.32969793	-0.5488258	0.58312503	0.99938111		Cyp46a1	84.0057911	160.193397	117.689243	72.3849004	133.184938	74.091435	48.1131944	123.261887
1110008F13Rik	22.4831774	-0.1742011	0.31745197	-0.548748	0.58317839	0.99938111		1110008F13Ri	21.5054825	28.961518	27.672876	16.5451201	19.9777406	14.1126543	25.981125	25.1089028
Bmp6	3.95800849	-0.2274586	0.41453905	-0.5487024	0.58320968	NA		Bmp6	2.01613899	9.95552182	5.72542262	0	6.65924688	1.17605452	3.84905555	2.28262753
Ggps1	36.1544324	-0.1976133	0.36019452	-0.5486293	0.5832599	0.99938111		Ggps1	46.3711967	47.9675142	41.6683535	22.7495401	18.3129289	38.8097993	59.6603611	13.6957652
Chrna5	0.57333613	0.15440148	0.28143663	0.54861898	0.58326696	NA		Chrna5	0	0	1.27231614	0	0	2.35210905	0.96226389	0
Dph2	7.19371844	0.22620433	0.4123812	0.54853212	0.58332658	0.99938111		Dph2	6.72046328	2.71514231	2.54463228	14.4769801	4.99443516	4.7042181	7.69811111	13.6957652
Rwdd2b	5.50217583	0.22606138	0.4121707	0.54846542	0.58337237	0.99938111		Rwdd2b	7.39250961	1.81009488	6.04350166	4.13628002	4.99443516	3.52816357	11.5471667	4.56525506
Thap6	2.59385886	0.22745312	0.41478187	0.54836804	0.58343922	NA		Thap6	2.01613899	2.26261859	2.54463228	2.06814001	1.66481172	1.17605452	6.73584722	2.28262753
Gdpd1	22.4773662	-0.206062	0.37584464	-0.5482639	0.58351071	0.99938111		Gdpd1	22.1775288	39.3695635	25.4463228	10.3407001	11.653682	36.4576902	11.5471667	22.8262753
Lman2	49.9571103	-0.1736878	0.31682329	-0.5482166	0.58354315	0.99938111		Lman2	40.3227797	69.236129	43.5768277	59.9760603	46.6147282	52.9224536	23.0943333	63.9135709
Top1	87.135608	0.16557933	0.30209131	0.54811019	0.58361624	0.99938111		Top1	96.7746713	72.8563187	71.2497037	84.7937404	44.9499164	136.422325	82.7546944	107.283494
A230073K19Rik	19.9362195	0.22563046	0.4116867	0.54806353	0.58364828	0.99938111		A230073K19Ri	22.1775288	25.793852	14.9497146	6.20442003	3.32962344	49.39429	30.7924444	6.84788259
Cnksr3	1.37476173	-0.2032669	0.37095257	-0.5479592	0.5837199	NA		Cnksr3	0.67204633	1.81009488	4.45310648	0	0	1.17605452	2.88679167	0
Car15	2.24840423	0.22394482	0.40871797	0.54792017	0.58374672	NA		Car15	0.67204633	2.26261859	3.49886938	0	4.99443516	2.35210905	1.92452778	2.28262753
Fam120aos	10.5680818	0.22283411	0.40689507	0.54764514	0.58393559	0.99938111		Fam120aos	6.72046328	12.2181404	13.0412404	4.13628002	8.3240586	2.35210905	24.0565972	13.6957652
Gdpd5	10.910998	-0.2159344	0.39445461	-0.5474251	0.58408668	0.99938111		Gdpd5	10.0806949	20.8160911	13.9954775	2.06814001	14.9833055	11.7605452	6.73584722	6.84788259
Kcng1	16.7564811	-0.2122867	0.38779607	-0.5474183	0.58409138	0.99938111		Kcng1	15.4570656	22.6261859	20.9932163	12.4088401	23.3073641	7.05632714	4.81131944	27.3915304
Nid2	0.92522682	-0.1958032	0.35779372	-0.5472516	0.5842059	NA		Nid2	1.34409266	2.26261859	0.9542371	0	1.66481172	1.17605452	0	0
Gm5468	1.11601435	-0.2009191	0.36719362	-0.5471748	0.58425863	NA		Gm5468	0.67204633	2.71514231	2.22655324	0	0	2.35210905	0.96226389	0
Nipal3	102.029486	0.18320099	0.33486683	0.5470861	0.58431958	0.99938111		Nipal3	67.8766792	143.450019	100.194896	62.0442003	128.190502	161.11947	52.9245139	100.435611
Aes	352.580439	-0.1671892	0.30560787	-0.5470709	0.58432999	0.99938111		Aes	395.163241	296.85556	359.11123	454.990802	399.554813	155.239197	278.094264	481.634409
Rtn3	743.897354	-0.1611613	0.29460418	-0.5470436	0.5843488	0.99938111		Rtn3	594.761001	1152.12539	861.039946	560.465943	544.393433	1040.80825	537.905514	659.679356
1700017B05Rik	13.286479	-0.2146145	0.39232315	-0.5470349	0.58435475	0.99938111		1700017B05Ri	13.4409266	14.9332827	6.67965972	24.8176801	21.6425524	4.7042181	8.660375	11.4131377
Ldoc1	1.8641626	0.20765241	0.37966079	0.54694193	0.58441863	NA		Ldoc1	2.68818531	0.45252372	2.22655324	0	6.65924688	0	2.88679167	0
Pck2	11.9336986	0.21571709	0.39443322	0.54690395	0.58444472	0.99938111		Pck2	8.06455594	9.5029981	7.31581779	18.6132601	28.3017992	5.88027262	8.660375	9.13051012
Isyna1	20.0746217	-0.2179914	0.39862448	-0.546859	0.58447561	0.99938111		Isyna1	16.1291119	14.0282353	12.7231614	47.5672202	24.9721758	5.88027262	9.62263889	29.6741579
Dnali1	0.30578162	-0.1213381	0.22188544	-0.5468502	0.58448164	NA		Dnali1	0	1.81009488	0.63615807	0	0	0	0	0
Dok2	0.30578162	-0.1213381	0.22188544	-0.5468502	0.58448164	NA		Dok2	0	1.81009488	0.63615807	0	0	0	0	0
Itih2	0.30578162	-0.1213381	0.22188544	-0.5468502	0.58448164	NA		Itih2	0	1.81009488	0.63615807	0	0	0	0	0
Msln	0.30578162	-0.1213381	0.22188544	-0.5468502	0.58448164	NA		Msln	0	1.81009488	0.63615807	0	0	0	0	0
Evi5l	37.8493569	0.19870445	0.36339746	0.54679647	0.58451857	0.99938111		Evi5l	35.6184554	19.0059962	27.354797	55.8397803	43.2851047	29.4013631	19.2452778	73.044081
Plcb2	3.30918564	0.22665349	0.41453852	0.54676098	0.58454295	NA		Plcb2	1.34409266	2.26261859	1.59039517	6.20442003	1.66481172	2.35210905	1.92452778	9.13051012
Rpl34	3.03550773	-0.2233962	0.40859346	-0.5467445	0.58455425	NA		Rpl34	3.36023164	0.90504744	2.54463228	8.27256004	4.99443516	0	1.92452778	2.28262753
Zfp92	4.25436938	-0.2265787	0.4145159	-0.5466104	0.58464645	NA		Zfp92	3.36023164	2.26261859	6.04350166	8.27256004	3.32962344	2.35210905	3.84905555	4.56525506
Oprd1	11.7531087	0.20388301	0.37304473	0.54653771	0.58469637	0.99938111		Oprd1	10.7527413	13.5757116	11.1327662	4.13628002	13.3184938	10.5844907	7.69811111	22.8262753
E030011O05Rik	0.56341789	0.16955477	0.31025279	0.54650521	0.58471871	NA		E030011O05R	0	0.45252372	0.9542371	0	0	1.17605452	1.92452778	0
Esco1	13.3906919	0.20643466	0.37773667	0.54650414	0.58471944	0.99938111		Esco1	10.7527413	15.3858064	15.9039517	6.20442003	11.653682	17.6408179	25.0188611	4.56525506
Cdkn2d	33.4555475	0.20948283	0.383317	0.54650024	0.58472212	0.99938111		Cdkn2d	24.8657142	19.9110436	14.9497146	62.0442003	41.620293	14.1126543	30.7924444	59.3483158
Gtpbp6	8.13914974	0.22689279	0.41524813	0.54640291	0.58478901	0.99938111		Gtpbp6	3.36023164	4.07271347	5.40734359	14.4769801	16.6481172	0	2.88679167	18.2610202
Srxn1	81.501105	0.12297594	0.22507493	0.54637779	0.58480628	0.99938111		Srxn1	73.9250961	77.8340797	78.5655215	80.6574604	64.9276571	82.3238167	93.3395972	100.435611
Smim13	204.277635	0.15736709	0.2880336	0.54634976	0.58482554	0.99938111		Smim13	187.500926	229.429526	235.060406	111.679561	144.83862	268.140431	270.396153	187.175458
Pgrmc1	158.435007	0.18585963	0.3401991	0.54632603	0.58484185	0.99938111		Pgrmc1	116.936061	213.138672	172.398837	78.5893204	104.883138	296.36574	166.471653	118.696632
Zbtb7c	12.0169309	0.21059947	0.38548451	0.54632409	0.58484318	0.99938111		Zbtb7c	9.4086486	7.69290322	6.67965972	20.6814001	19.9777406	7.05632714	8.660375	15.9783927
Spire1	114.942179	-0.1407536	0.25764272	-0.5463133	0.58485059	0.99938111		Spire1	124.328571	157.02573	116.098848	86.8618804	86.5702095	129.365998	127.981097	91.3051012
Tmem106c	25.0387055	-0.1750682	0.32051492	-0.5462091	0.58492221	0.99938111		Tmem106c	24.8657142	29.8665654	20.9932163	31.0221002	19.9777406	25.8731995	13.4716944	34.239413
Pfkfb4	7.05716029	0.22102302	0.40466811	0.54618344	0.58493985	0.99938111		Pfkfb4	5.37637063	4.97776091	3.18079035	12.4088401	11.653682	5.88027262	3.84905555	9.13051012
Qpctl	12.668693	-0.198383	0.36323208	-0.5461606	0.58495557	0.99938111		Qpctl	8.73660227	18.1009488	11.7689243	16.5451201	11.653682	14.1126543	6.73584722	13.6957652
Epb4.1l4b	6.80709448	0.22330564	0.40900831	0.54596848	0.5850876	0.99938111		Epb4.1l4b	8.73660227	6.33533207	7.63389683	0	1.66481172	9.40843619	11.5471667	9.13051012
Txnl4a	74.9129145	0.14904572	0.27302016	0.54591472	0.58512456	0.99938111		Txnl4a	67.2046328	62.9007969	66.7965972	84.7937404	73.2517157	49.39429	80.8301666	114.131377
Nusap1	1.14650715	0.20030545	0.3670757	0.54567887	0.5852867	NA		Nusap1	0.67204633	0.45252372	0.9542371	2.06814001	0	1.17605452	3.84905555	0
Acp2	42.3911974	0.19910209	0.36506553	0.54538726	0.5854872	0.99938111		Acp2	30.9141311	57.4705123	45.803381	18.6132601	36.6258578	90.5561984	31.7547083	27.3915304
Cspg4	15.1792779	-0.2247382	0.41218626	-0.5452345	0.58559227	0.99938111		Cspg4	10.0806949	31.6766603	13.9954775	12.4088401	36.6258578	8.23238167	3.84905555	4.56525506
Pik3ap1	1.19011534	-0.1927379	0.35351703	-0.545201	0.58561525	NA		Pik3ap1	4.03227797	0.90504744	0.63615807	0	1.66481172	0	0	2.28262753
Micu3	146.055823	0.19390204	0.35580293	0.54497032	0.58577393	0.99938111		Micu3	159.27498	154.763112	150.133304	68.2486203	58.2684102	203.457433	257.886722	116.414004
Jarid2	21.1802606	0.18383912	0.33736442	0.54492742	0.58580344	0.99938111		Jarid2	24.8657142	18.5534725	22.2655324	12.4088401	23.3073641	32.9295267	25.981125	9.13051012
1700018G05Rik	0.67154606	-0.1675657	0.30771045	-0.5445565	0.5860586	NA		1700018G05R	0	1.81009488	0.31807903	2.06814001	0	1.17605452	0	0
Rngtt	18.1113433	0.17764048	0.32626964	0.54445911	0.58612558	0.99938111		Rngtt	16.8011582	19.0059962	15.5858727	16.5451201	23.3073641	15.2887088	26.9433889	11.4131377
Tmem11	24.7211585	0.20080467	0.36883956	0.5444228	0.58615056	0.99938111		Tmem11	23.5216215	13.1231878	12.0870033	43.4309402	31.6314227	14.1126543	27.9056528	31.9567854
Sgsm3	28.3652753	0.19153355	0.35192266	0.54424899	0.58627015	0.99938111		Sgsm3	24.8657142	24.8888045	22.2655324	31.0221002	56.6035985	17.6408179	15.3962222	34.239413
Rsrc2	37.798117	0.21670117	0.3982249	0.54416781	0.586326	0.99938111		Rsrc2	51.7475673	34.8443264	34.9886938	12.4088401	13.3184938	50.5703445	88.5282777	15.9783927
Ssna1	10.8900468	-0.2139825	0.39328261	-0.5440934	0.5863772	0.99938111		Ssna1	9.4086486	11.313093	12.4050823	14.4769801	19.9777406	2.35210905	5.77358333	11.4131377
Tes	0.34680956	0.13930987	0.25605853	0.5440548	0.58640376	NA		Tes	0	0	0.63615807	0	0	1.17605452	0.96226389	0
Tmem132a	94.0262507	0.20040567	0.36835823	0.54405103	0.58640636	0.99938111		Tmem132a	57.1239379	109.51074	81.7463119	90.9981605	206.436653	63.5069443	35.6037639	107.283494
4930404N11Rik	4.97151776	0.22419055	0.41223508	0.54384151	0.58655054	0.99938111		4930404N11R	2.01613899	5.88280835	3.18079035	6.20442003	6.65924688	7.05632714	1.92452778	6.84788259
Cab39	131.684911	0.1260966	0.23195058	0.54363562	0.58669224	0.99938111		Cab39	137.769497	131.684402	137.410143	93.0663005	108.212762	149.358925	163.584861	132.392397
Mrpl46	13.0855861	-0.2029163	0.37332112	-0.5435437	0.58675552	0.99938111		Mrpl46	17.4732045	9.95552182	13.3593194	18.6132601	3.32962344	16.4647633	16.3584861	9.13051012
Rad17	14.2111988	-0.1960031	0.36062814	-0.5435047	0.58678234	0.99938111		Rad17	14.7850192	19.9110436	14.3135566	14.4769801	8.3240586	19.9929269	17.32075	4.56525506
Pcx	35.0504597	0.20057685	0.36907282	0.54346146	0.58681212	0.99938111		Pcx	26.8818531	24.4362808	19.4028211	57.9079203	64.9276571	29.4013631	16.3584861	41.0872956
Arhgef4	128.828361	0.12528914	0.23054853	0.54343934	0.58682734	0.99938111		Arhgef4	118.280154	109.51074	99.5587378	167.519341	139.844185	127.013889	125.094306	143.805534
Vps41	134.931003	0.18837719	0.34664572	0.54342856	0.58683476	0.99938111		Vps41	138.441544	135.304592	154.586411	66.1804803	84.9053977	217.570087	211.698055	70.7614535
Ralgps1	70.2844251	0.14607739	0.2688368	0.54336829	0.58687625	0.99938111		Ralgps1	68.5487255	81.4542694	73.476257	39.2946602	61.5980337	87.0280348	84.6792222	66.1961984
Slc9a3r2	22.1944235	0.21591528	0.39737743	0.54335063	0.58688841	0.99938111		Slc9a3r2	8.06455594	17.1959013	14.6316356	39.2946602	28.3017992	10.5844907	11.5471667	47.9351782
Nufip2	89.6396252	0.1651045	0.30387466	0.54333091	0.58690198	0.99938111		Nufip2	86.6939764	98.6501707	91.2886829	57.9079203	61.5980337	104.668853	150.113167	66.1961984
Taf6l	8.84322525	0.22470143	0.41358157	0.5433062	0.586919	0.99938111		Taf6l	4.7043243	3.62018975	6.04350166	16.5451201	4.99443516	5.88027262	3.84905555	25.1089028
Ppp1r35	4.71671633	-0.2249654	0.41419565	-0.543138	0.58703479	0.99938111		Ppp1r35	8.06455594	0.90504744	2.86271131	10.3407001	3.32962344	1.17605452	1.92452778	9.13051012
Gna11	109.57276	0.170276	0.31356282	0.54303632	0.5871048	0.99938111		Gna11	88.7101153	81.9067931	75.3847312	163.383061	166.481172	71.739326	94.3018611	134.675024
Gm11837	5.93034766	-0.2232426	0.41124166	-0.5428501	0.58723302	0.99938111		Gm11837	8.06455594	10.4080455	6.67965972	2.06814001	3.32962344	8.23238167	8.660375	0
Trim36	4.920698	0.22327874	0.41132574	0.54282706	0.58724888	0.99938111		Trim36	4.03227797	5.43028463	2.54463228	6.20442003	8.3240586	7.05632714	5.77358333	0
Clpp	39.7778707	-0.193691	0.35684608	-0.5427858	0.58727731	0.99938111		Clpp	38.978687	23.9837571	26.7186389	86.8618804	31.6314227	24.697145	44.2641389	41.0872956
Qpct	8.1749171	0.22150249	0.40809105	0.54277713	0.58728326	0.99938111		Qpct	5.37637063	9.05047438	9.86045007	4.13628002	3.32962344	22.345036	6.73584722	4.56525506
Aatf	5.62626375	0.21804127	0.40178189	0.54268564	0.58734626	0.99938111		Aatf	7.39250961	4.07271347	4.45310648	4.13628002	4.99443516	10.5844907	4.81131944	4.56525506
Nav3	67.2871268	0.14155216	0.26087688	0.54260142	0.58740426	0.99938111		Nav3	76.6132814	68.3310816	66.7965972	41.3628002	63.2628454	81.1477622	83.7169583	57.0656883
Gatad2a	42.2438048	-0.1789389	0.32979952	-0.5425686	0.58742683	0.99938111		Gatad2a	30.2420848	39.8220873	37.5333261	76.5211804	59.9332219	31.7534722	27.9056528	34.239413
Sh3pxd2a	36.841441	0.17904535	0.33008627	0.54241987	0.5875293	0.99938111		Sh3pxd2a	43.010965	37.106945	29.8994292	24.8176801	58.2684102	51.7463991	20.2075417	29.6741579
Kdm1b	9.044711	-0.2089571	0.38530767	-0.5423124	0.58760333	0.99938111		Kdm1b	10.0806949	12.6706641	11.4508452	6.20442003	3.32962344	10.5844907	13.4716944	4.56525506
Lag3	2.21239137	-0.2160772	0.39856146	-0.5421427	0.5877202	NA		Lag3	1.34409266	2.71514231	2.22655324	4.13628002	4.99443516	0	0	2.28262753
Chst10	39.1712277	-0.1949765	0.35966387	-0.5421076	0.58774442	0.99938111		Chst10	40.3227797	66.5209867	42.3045116	20.6814001	28.3017992	67.0351079	23.0943333	25.1089028
Xrcc6bp1	4.88681732	0.22257325	0.41057288	0.54210413	0.58774677	0.99938111		Xrcc6bp1	4.03227797	4.07271347	3.81694841	4.13628002	4.99443516	4.7042181	1.92452778	11.4131377

GeneID	Base mean	log2(FC)	StdErr	Wald-Stats	P-value	P-adj		GeneID	Normalised expression for Chow#1	Normalised expression for Chow#2	Normalised expression for Chow#3	Normalised expression for Chow#4	Normalised expression for HFD#1	Normalised expression for HFD#2	Normalised expression for HFD#3	Normalised expression for HFD#4
9430021M05Rik	19.1178336	0.20146603	0.3717677	0.54191376	0.58787792	0.99938111		9430021M05R	15.4570656	15.3858064	21.9474534	16.5451201	3.32962344	28.2253086	26.9433889	25.1089028
Tbl3	15.5967379	0.21411821	0.39524941	0.54172935	0.58800496	0.99938111		Tbl3	12.0968339	11.313093	7.95197586	24.8176801	33.2962344	7.05632714	7.69811111	20.5436478
Zbtb3	2.47970371	0.22401465	0.41367664	0.54152114	0.58814843	NA		Zbtb3	2.68818531	2.26261859	2.22655324	0	4.99443516	1.17605452	1.92452778	4.56525506
Wsb1	7.41021074	0.21927482	0.40494434	0.54149372	0.58816733	0.99938111		Wsb1	8.73660227	4.97776091	3.81694841	10.3407001	9.98887032	4.7042181	14.4339583	2.28262753
Efcab12	0.41140831	-0.1431159	0.26430995	-0.54147	0.58818367	NA		Efcab12	0	0.90504744	0.31807903	2.06814001	0	0	0	0
Fbxo17	0.41140831	-0.1431159	0.26430995	-0.54147	0.58818367	NA		Fbxo17	0	0.90504744	0.31807903	2.06814001	0	0	0	0
Naaladl1	0.41140831	-0.1431159	0.26430995	-0.54147	0.58818367	NA		Naaladl1	0	0.90504744	0.31807903	2.06814001	0	0	0	0
Gm15421	16.4096322	0.22454363	0.41470183	0.54145801	0.58819193	0.99938111		Gm15421	13.4409266	11.7656167	21.6293743	10.3407001	4.99443516	12.9365998	53.8867778	2.28262753
Fzd4	4.41812713	-0.2236537	0.41307402	-0.5414374	0.58820612	NA		Fzd4	2.01613899	10.8605693	5.08926455	2.06814001	8.3240586	4.7042181	0	2.28262753
Adamts6	2.64018875	0.21758192	0.40195311	0.54131169	0.58829277	NA		Adamts6	2.01613899	0.45252372	2.86271131	4.13628002	0	5.88027262	5.77358333	0
Sox3	1.87923408	-0.205861	0.38030184	-0.5413094	0.58829434	NA		Sox3	0.67204633	1.35757116	1.27231614	6.20442003	0	0	0.96226389	4.56525506
Cables2	22.0331505	0.16466578	0.30421266	0.54128509	0.5883111	0.99938111		Cables2	16.1291119	20.8160911	18.448584	28.9539601	24.9721758	22.345036	24.0565972	20.5436478
Ncapg2	3.11730036	0.22532475	0.41631815	0.54123211	0.5883476	NA		Ncapg2	3.36023164	2.71514231	2.54463228	2.06814001	6.65924688	4.7042181	2.88679167	0
Atl1	43.7550007	0.1985386	0.36689567	0.54113094	0.58841733	0.99938111		Atl1	38.978687	57.0179886	47.0756971	14.4769801	31.6314227	91.7322529	39.4528194	29.6741579
Zfp282	6.21718974	0.21729126	0.40180051	0.54079389	0.58864965	0.99938111		Zfp282	2.68818531	5.43028463	8.58813393	4.13628002	8.3240586	4.7042181	6.73584722	9.13051012
Slc7a1	37.4742761	0.18002591	0.33289472	0.54078932	0.5886528	0.99938111		Slc7a1	28.2259458	50.6826565	38.4875632	18.6132601	63.2628454	42.3379629	30.7924444	27.3915304
Ripk4	0.47887395	0.16043771	0.29668236	0.54077268	0.58866428	NA		Ripk4	0.67204633	0	0.31807903	0	1.66481172	1.17605452	0	0
Megf9	71.777145	-0.2158198	0.39917898	-0.5406591	0.58874255	0.99938111		Megf9	66.5325865	145.712637	83.336707	24.8176801	31.6314227	135.24627	66.3962083	20.5436478
Srp68	50.4985882	0.14995816	0.27742405	0.54053772	0.58882626	0.99938111		Srp68	55.1077989	43.8948007	42.9406697	47.5672202	31.6314227	58.8027262	50.9999861	73.044081
Eid2b	39.8649567	0.18427083	0.34094383	0.54047269	0.58887109	0.99938111		Eid2b	25.5377605	22.6261859	28.6271131	74.4530404	34.9610461	43.5140174	48.1131944	41.0872956
Scamp5	317.280198	0.17346328	0.3211163	0.54018832	0.58906717	0.99938111		Scamp5	177.420231	320.839317	268.140626	409.491722	432.851047	283.42914	171.282972	474.786526
Creb3l3	0.65912634	0.1504723	0.27863065	0.54004214	0.58916797	NA		Creb3l3	0	0	0.31807903	2.06814001	0	0	2.88679167	0
Ncoa7	87.4124661	0.20334602	0.376674	0.53984617	0.58930312	0.99938111		Ncoa7	98.1187639	100.460266	91.2886829	24.8176801	51.6091633	158.767361	130.867889	43.3699231
Exd2	21.8729689	0.19517569	0.36154823	0.53983306	0.58931217	0.99938111		Exd2	18.8172972	29.4140417	21.6293743	8.27256004	16.6481172	42.3379629	17.32075	20.5436478
Fam222a	14.8930982	-0.2096268	0.38833842	-0.5398044	0.58933195	0.99938111		Fam222a	14.7850192	8.59795066	15.5858727	26.8858201	18.3129289	10.5844907	3.84905555	20.5436478
Clasp1	106.754618	0.12749754	0.23619492	0.53979798	0.58933636	0.99938111		Clasp1	98.7908103	109.963264	108.464951	88.9300204	134.849749	130.542052	104.886764	77.6093361
Tsta3	8.86389784	0.20445348	0.3788914	0.5396097	0.58946623	0.99938111		Tsta3	9.4086486	5.43028463	10.8146872	6.20442003	13.3184938	10.5844907	10.5849028	4.56525506
BC027231	5.5257956	0.22099966	0.40959744	0.53955333	0.58950511	0.99938111		BC027231	6.04841696	4.52523719	5.08926455	4.13628002	6.65924688	12.9365998	4.81131944	0
Usp7	117.930668	0.17737442	0.32881313	0.53943839	0.5895844	0.99938111		Usp7	126.34471	129.874307	121.188112	57.9079203	96.5590798	209.337705	133.754681	68.4788259
Gpatch11	15.3335039	0.20593981	0.38190085	0.53924942	0.58971477	0.99938111		Gpatch11	22.1775288	13.5757116	14.9497146	4.13628002	16.6481172	23.5210905	23.0943333	4.56525506
Ppp2ca	307.171311	0.13559038	0.25147517	0.53917998	0.58976267	0.99938111		Ppp2ca	311.829496	273.77685	311.717454	268.858201	209.766277	362.224793	440.716861	278.480559
Fcgr2b	0.54368157	-0.1469369	0.2725228	-0.539173	0.5897675	NA		Fcgr2b	0.67204633	2.71514231	0	0	0	0	0.96226389	0
4930519F09Rik	17.7594183	0.22137202	0.41064804	0.53907971	0.58983185	0.99938111		4930519F09Ri	32.2582238	9.95552182	15.5858727	4.13628002	8.3240586	36.4576902	30.7924444	4.56525506
Mknk1	11.436126	-0.1989885	0.36929535	-0.5388328	0.59000221	0.99938111		Mknk1	11.4247876	11.313093	11.4508452	16.5451201	14.9833055	14.1126543	4.81131944	6.84788259
Il10ra	0.59171803	-0.1707024	0.31697348	-0.5385383	0.59020546	NA		Il10ra	1.34409266	0.45252372	1.27231614	0	1.66481172	0	0	0
Galnt6	1.44438539	-0.1752453	0.32542224	-0.5385166	0.59022047	NA		Galnt6	0	4.52523719	3.18079035	0	0	0	3.84905555	0
Tmem165	12.6763766	-0.1954288	0.36299436	-0.5383798	0.59031489	0.99938111		Tmem165	12.0968339	20.3635674	13.6773985	8.27256004	8.3240586	17.6408179	9.62263889	11.4131377
Malsu1	5.47543916	0.22194866	0.41230351	0.53831378	0.59036044	0.99938111		Malsu1	4.03227797	5.88280835	5.40734359	4.13628002	0	8.23238167	11.5471667	4.56525506
Hgs	72.9637989	0.18714813	0.34771605	0.53822115	0.59042438	0.99938111		Hgs	65.8605402	42.5372296	50.5745665	109.611421	116.53682	47.042181	44.2641389	107.283494
Enoph1	25.3058761	0.18235324	0.33882086	0.53819955	0.59043929	0.99938111		Enoph1	30.2420848	24.8888045	18.766663	18.6132601	23.3073641	12.9365998	39.4528194	34.239413
Entpd3	7.34081289	0.20965386	0.38961824	0.53810072	0.59050752	0.99938111		Entpd3	4.7043243	8.14542694	6.04350166	8.27256004	3.32962344	11.7605452	9.62263889	6.84788259
Parp12	3.0978799	-0.2237652	0.41603247	-0.5378551	0.59067712	NA		Parp12	2.01613899	5.43028463	3.18079035	4.13628002	4.99443516	1.17605452	3.84905555	0
Chml	12.6807015	0.21487855	0.39952705	0.5378323	0.59069283	0.99938111		Chml	11.4247876	9.95552182	17.4943469	6.20442003	8.3240586	29.4013631	16.3584861	2.28262753
Cd33	1.36695035	-0.2048673	0.38092343	-0.5378175	0.59070306	NA		Cd33	1.34409266	3.62018975	1.90847421	0	0	1.17605452	2.88679167	0
Nle1	4.32992041	0.21997494	0.40904548	0.53777623	0.59073154	NA		Nle1	1.34409266	1.81009488	1.59039517	10.3407001	3.32962344	0	4.81131944	11.4131377
Akr1b10	3.68539884	-0.22266	0.41412578	-0.5376627	0.59080993	NA		Akr1b10	3.36023164	5.88280835	3.81694841	4.13628002	1.66481172	3.52816357	4.81131944	2.28262753
Cyp4f16	0.47645922	-0.1656773	0.30825129	-0.5374749	0.59093963	NA		Cyp4f16	0.67204633	0.90504744	1.27231614	0	0	0	0.96226389	0
Vmn2r87	0.551915	-0.166084	0.30901875	-0.5374562	0.59095255	NA		Vmn2r87	2.01613899	0.90504744	0.31807903	0	0	1.17605452	0	0
Kif16b	11.0767766	0.2005111	0.37317032	0.53731793	0.59104802	0.99938111		Kif16b	10.0806949	11.313093	9.86045007	10.3407001	9.98887032	16.4647633	18.2830139	2.28262753
Extl2	47.6369079	-0.1962749	0.36528869	-0.5373146	0.59105035	0.99938111		Extl2	51.075521	70.1411764	60.1169375	26.8858201	26.6369875	84.6759257	43.301875	18.2610202
Mrps15	18.4492088	0.19135922	0.35618679	0.53724402	0.59109907	0.99938111		Mrps15	24.1936678	15.3858064	15.5858727	12.4088401	6.65924688	25.8731995	26.9433889	20.5436478
Enah	279.626617	0.14318072	0.26653625	0.53719043	0.59113608	0.99938111		Enah	197.581621	246.172903	222.337245	392.946602	279.688369	335.175539	218.433903	344.676757
Baz2a	49.3189812	0.13525426	0.25178363	0.53718451	0.59114017	0.99938111		Baz2a	42.3389187	51.587704	44.2129858	47.5672202	48.2795399	55.2745626	41.3773472	63.9135709
Leprel4	3.52580773	-0.2234458	0.41620414	-0.5368659	0.59136023	NA		Leprel4	2.01613899	7.69290322	3.18079035	4.13628002	1.66481172	4.7042181	4.81131944	0
Tmem259	71.3907752	-0.1935264	0.36052872	-0.5367849	0.59141618	0.99938111		Tmem259	51.075521	79.6441745	62.0254117	117.883981	96.5590798	48.2182355	22.1320694	93.5877288
Acad11	11.3026642	-0.2119787	0.39493837	-0.5367386	0.59144819	0.99938111		Acad11	16.1291119	9.95552182	18.130505	6.20442003	14.9833055	10.5844907	14.4339583	0
Cdkn2aip	10.574114	-0.2138462	0.39843857	-0.5367106	0.59146751	0.99938111		Cdkn2aip	12.0968339	12.2181404	8.58813393	16.5451201	4.99443516	17.6408179	12.5094306	0
Eno1	141.30674	-0.1920113	0.35789318	-0.5365044	0.59161	0.99938111		Eno1	221.103242	95.9350284	120.870033	175.791901	214.760712	61.1548353	67.3584722	173.479692
Gm14431	13.097166	0.22280421	0.4154055	0.53635355	0.59171423	0.99938111		Gm14431	10.0806949	16.2908539	16.2220308	2.06814001	1.66481172	31.7534722	22.1320694	4.56525506
Gm8898	13.097166	0.22280421	0.4154055	0.53635355	0.59171423	0.99938111		Gm8898	10.0806949	16.2908539	16.2220308	2.06814001	1.66481172	31.7534722	22.1320694	4.56525506
Kcnc3	75.6776465	0.17971043	0.33510153	0.53628651	0.59176055	0.99938111		Kcnc3	61.8282622	89.5996963	67.1146763	57.9079203	108.212762	82.3238167	28.8679167	109.566121
Dsn1	1.69727621	0.2076687	0.38729182	0.5362073	0.59181529	NA		Dsn1	2.68818531	1.81009488	0.9542371	0	0	2.35210905	5.77358333	0
Fam217b	46.2893397	0.15233183	0.28422008	0.53596433	0.5919832	0.99938111		Fam217b	44.3550577	41.6321821	42.6225906	47.5672202	31.6314227	71.739326	51.96225	38.804668
Rbck1	58.8983928	0.20921483	0.39054514	0.53569948	0.59216626	0.99938111		Rbck1	48.3873356	25.3413283	38.1694841	99.2707205	89.8998329	18.8168724	39.4528194	111.848749
5730455P16Rik	57.9522149	0.17584979	0.32827625	0.53567624	0.59218233	0.99938111		5730455P16Ri	57.7959842	52.4927514	61.7073327	43.4309402	28.3017992	76.4435441	100.075444	43.3699231
Fam69a	22.5759251	0.22053889	0.4117575	0.53560381	0.59223239	0.99938111		Fam69a	23.5216215	26.2463757	26.0824808	2.06814001	4.99443516	45.8661264	40.4150833	11.4131377
Dysf	2.27469844	0.21608185	0.40348117	0.53554382	0.59227386	NA		Dysf	1.34409266	2.71514231	2.22655324	0	4.99443516	2.35210905	0	4.56525506
Crebbp	132.149693	0.15067294	0.28136811	0.53550112	0.59230338	0.99938111		Crebbp	125.672663	161.098444	137.092064	70.3167603	153.162678	182.288451	120.282986	107.283494
Prdm10	10.0982079	0.20903688	0.3903996	0.53544337	0.59234331	0.99938111		Prdm10	12.0968339	12.2181404	7.63389683	2.06814001	6.65924688	7.05632714	12.5094306	20.5436478
Rbm45	12.0196868	-0.1962456	0.36654622	-0.535391	0.59237951	0.99938111		Rbm45	15.4570656	18.5534725	13.0412404	4.13628002	8.3240586	11.7605452	13.4716944	11.4131377
Dars2	12.7864575	0.19586485	0.36598455	0.53517247	0.5925306	0.99938111		Dars2	12.7688802	7.69290322	14.9497146	12.4088401	9.98887032	16.4647633	21.1698055	6.84788259
Mcoln1	31.5488533	-0.186274	0.34811417	-0.5350946	0.59258441	0.99938111		Mcoln1	19.4893435	50.2301328	32.1259825	33.0902402	29.966611	32.9295267	13.4716944	41.0872956
1600029O15Rik	1.06703059	-0.1817753	0.33971412	-0.5350831	0.59259242	NA		1600029O15Ri	2.01613899	0.45252372	3.18079035	0	0	0	2.88679167	0
H2-T10	0.89483286	-0.1536975	0.28729714	-0.5349776	0.59266533	NA		H2-T10	0	1.35757116	0	4.13628002	1.66481172	0	0	0
Gm16982	4.85618603	-0.2197029	0.41075149	-0.5348804	0.59273255	0.99938111		Gm16982	6.04841696	8.14542694	5.72542262	2.06814001	4.99443516	7.05632714	4.81131944	0
Aspdh	1.87339395	0.20195747	0.37758659	0.534864	0.5927439	NA		Aspdh	0	1.35757116	0.63615807	4.13628002	3.32962344	0	0.96226389	4.56525506
Rnf38	48.6463166	-0.1612687	0.3015201	-0.5348522	0.59275208	0.99938111		Rnf38	45.027104	44.3473245	54.0734359	64.1123403	64.9276571	23.5210905	45.2264028	47.9351782
Poc1b	2.91545617	-0.2224856	0.41615705	-0.5346193	0.59291311	NA		Poc1b	4.7043243	3.16766603	5.08926455	0	1.66481172	3.52816357	2.88679167	2.28262753
Atp6v0d1	193.994675	0.12024909	0.22497032	0.53451089	0.59298812	0.99938111		Atp6v0d1	184.140694	155.215636	173.989232	229.563541	196.447783	156.415252	216.509375	239.675891
1110058L19Rik	7.54897938	0.21911496	0.40996308	0.53447486	0.59301304	0.99938111		1110058L19Ri	6.72046328	6.78785578	7.31581779	6.20442003	0	8.23238167	18.2830139	6.84788259
Clock	85.7669663	0.17902959	0.33506462	0.53431363	0.59312457	0.99938111		Clock	81.3176057	96.8400759	98.2864217	39.2946602	53.2739751	131.718107	126.056569	59.3483158
Gpr139	0.90942387	-0.1919761	0.35932265	-0.5342723	0.59315318	NA		Gpr139	1.34409266	0.45252372	1.27231614	2.06814001	0	1.17605452	0.96226389	0
Gm9776	2.33849378	-0.2191417	0.4102126	-0.534215	0.59319281	NA		Gm9776	2.68818531	3.16766603	2.54463228	2.06814001	4.99443516	0	0.96226389	2.28262753
Copg1	206.976173	0.12438679	0.23288125	0.5341211	0.59325775	0.99938111		Copg1	206.990269	156.120683	193.392053	233.699821	279.688369	204.633487	171.282972	210.001733
Zfp143	4.70450623	-0.221347	0.41441611	-0.5341178	0.59326003	0.99938111		Zfp143	8.06455594	4.07271347	6.99773876	2.06814001	0	7.05632714	4.81131944	4.56525506
Traip	1.25601186	-0.2037159	0.38142331	-0.5340939	0.59327656	NA		Traip	0.67204633	1.81009488	1.90847421	2.06814001	1.66481172	0	1.92452778	0
Babam1	54.482131	-0.1537481	0.28789045	-0.5340509	0.59330634	0.99938111		Babam1	69.2207718	46.6099431	54.3915149	59.9760603	51.6091633	29.4013631	53.8867778	70.7614535
Dmtn	261.386953	-0.1445256	0.27064804	-0.5339984	0.59334263	0.99938111		Dmtn	283.603551	299.118178	244.284698	275.062621	327.967909	152.887088	188.603722	319.567854
Tmem17	3.19842686	-0.2222815	0.41628045	-0.5339705	0.59336194	NA		Tmem17	4.03227797	5.43028463	2.86271131	2.06814001	0	4.7042181	1.92452778	4.56525506
Eme1	1.21723392	0.19931633	0.37333821	0.53387604	0.5934273	NA		Eme1	1.34409266	0.90504744	1.27231614	0	3.32962344	0	2.88679167	0
Dll4	0.51252827	0.14469018	0.27102137	0.53387	0.59343148	NA		Dll4	0	0.45252372	0.31807903	0	3.32962344	0	0	0
Tmem236	0.51252827	0.14469018	0.27102137	0.53387	0.59343148	NA		Tmem236	0	0.45252372	0.31807903	0	3.32962344	0	0	0
Nip7	17.4331484	-0.1802303	0.33760162	-0.5338551	0.5934418	0.99938111		Nip7	22.1775288	20.3635674	19.4028211	12.4088401	8.3240586	14.1126543	22.1320694	20.5436478
Pptc7	66.3220205	0.13100626	0.24551545	0.53359681	0.59362052	0.99938111		Pptc7	49.7314283	59.7331309	64.570044	78.5893204	73.2517157	59.9787807	62.5471528	82.1745911
Armc5	20.6335294	-0.2194874	0.4114349	-0.533468	0.59370965	0.99938111		Armc5	10.0806949	11.7656167	10.8146872	62.0442003	24.9721758	7.05632714	8.660375	29.6741579
Pip5k1c	334.390885	0.17484176	0.32775605	0.53345091	0.59372149	0.99938111		Pip5k1c	326.614516	276.039469	251.282437	382.605902	536.069374	177.584233	213.622583	511.308567
Cited4	6.97442596	0.22199138	0.41634267	0.53319393	0.59389935	0.99938111		Cited4	5.37637063	2.71514231	5.08926455	10.3407001	4.99443516	0	6.73584722	20.5436478
Rab8a	34.2018971	-0.1630362	0.30577698	-0.5331868	0.59390429	0.99938111		Rab8a	44.3550577	29.4140417	26.0824808	49.6353602	26.6369875	32.9295267	39.4528194	25.1089028
Ssr1	110.209148	-0.1873481	0.35139963	-0.5331483	0.59393092	0.99938111		Ssr1	85.3498837	178.746869	135.18359	78.5893204	63.2628454	196.401106	77.943375	66.1961984
Zfp429	1.05473317	-0.1883909	0.3533882	-0.5330989	0.5939651	NA		Zfp429	2.01613899	2.26261859	1.27231614	0	0	0	2.88679167	0
Rps3a1	22.1660687	-0.2128066	0.39921983	-0.5330561	0.59399473	0.99938111		Rps3a1	40.994826	17.1959013	25.4463228	16.5451201	8.3240586	19.9929269	44.2641389	4.56525506
Tm2d3	23.4259871	-0.1722399	0.32311903	-0.5330539	0.59399628	0.99938111		Tm2d3	18.1452509	33.0342315	20.9932163	28.9539601	19.9777406	29.4013631	16.3584861	20.5436478
Fam19a1	24.2304996	-0.2140387	0.40155515	-0.5330245	0.59401664	0.99938111		Fam19a1	22.1775288	47.5149905	34.0344567	4.13628002	14.9833055	35.2816357	28.8679167	6.84788259
Ctnnbl1	12.6695135	0.20091634	0.37693804	0.5330222	0.59401821	0.99938111		Ctnnbl1	13.4409266	5.88280835	7.95197586	20.6814001	11.653682	9.40843619	16.3584861	15.9783927
Fkbp8	276.77577	0.20794353	0.39023557	0.53286666	0.59412588	0.99938111		Fkbp8	195.565482	131.684402	142.181328	521.171283	391.230754	112.901234	169.358444	550.113235
Rbm14	36.7757273	0.21166338	0.39739507	0.53262709	0.59429174	0.99938111		Rbm14	19.4893435	23.9837571	21.9474534	66.1804803	68.2572805	10.5844907	22.1320694	61.6309433
Gm16938	1.85500389	0.21533667	0.40430854	0.5326048	0.59430718	NA		Gm16938	2.01613899	0.45252372	1.59039517	2.06814001	3.32962344	1.17605452	1.92452778	2.28262753
Rufy3	160.709695	0.14998511	0.28188702	0.53207524	0.59467389	0.99938111		Rufy3	172.715906	135.757116	169.218046	128.224681	114.872009	224.626414	226.132014	114.131377
Ccdc114	2.31173937	0.18091479	0.34003669	0.53204492	0.59469488	NA		Ccdc114	0.67204633	0.45252372	0	6.20442003	9.98887032	1.17605452	0	0
Inpp5k	20.011381	-0.1820569	0.34218866	-0.5320365	0.59470072	0.99938111		Inpp5k	16.8011582	25.3413283	24.8101647	16.5451201	14.9833055	12.9365998	14.4339583	34.239413
Tmem74	4.76399837	-0.2214879	0.41633325	-0.5319968	0.59472823	0.99938111		Tmem74	4.03227797	9.5029981	7.95197586	0	3.32962344	2.35210905	8.660375	2.28262753
Denr	32.2350362	0.17060163	0.32073253	0.53191246	0.59478663	0.99938111		Denr	36.9625481	24.4362808	31.1717454	28.9539601	19.9777406	43.5140174	50.0377222	22.8262753

GeneID	Base mean	log2(FC)	StdErr	Wald-Stats	P-value	P-adj		GeneID	Normalised expression for Chow#1	Normalised expression for Chow#2	Normalised expression for Chow#3	Normalised expression for Chow#4	Normalised expression for HFD#1	Normalised expression for HFD#2	Normalised expression for HFD#3	Normalised expression for HFD#4
Cntn2	44.9063578	-0.181153	0.34059679	-0.5318695	0.59481637	0.99938111		Cntn2	33.6023164	72.403795	46.439539	41.3628002	48.2795399	67.0351079	25.0188611	25.1089028
Adam23	121.665026	-0.187561	0.35267541	-0.5318232	0.59484844	0.99938111		Adam23	135.753358	184.177154	145.680198	62.0442003	81.5757743	203.457433	105.849028	54.7830607
Foxo6	17.8593744	0.21994993	0.41370209	0.53166259	0.5949597	0.99938111		Foxo6	13.4409266	5.43028463	8.58813393	35.1583802	28.3017992	3.52816357	9.62263889	38.804668
Srp14	46.7341565	-0.1695814	0.31913041	-0.5313858	0.59515145	0.99938111		Srp14	52.4196136	29.8665654	41.0321955	80.6574604	33.2962344	36.4576902	56.7735694	43.3699231
Clasp2	220.470152	-0.1510579	0.2843223	-0.5312911	0.59521709	0.99938111		Clasp2	264.114207	223.546717	251.282437	198.541441	178.134854	229.330632	306.96218	111.848749
Gm19522	2.21544469	-0.2154842	0.40566504	-0.5311875	0.59528883	NA		Gm19522	4.03227797	2.26261859	1.90847421	2.06814001	0	0	2.88679167	4.56525506
Erbb2ip	49.8300472	-0.2155154	0.40572537	-0.5311854	0.59529033	0.99938111		Erbb2ip	61.8282622	74.6664136	66.7965972	20.6814001	23.3073641	92.9083074	53.8867778	4.56525506
Spats2l	15.1165545	0.19342326	0.36414816	0.53116638	0.59530349	0.99938111		Spats2l	15.4570656	14.480759	16.8581888	8.27256004	6.65924688	24.697145	23.0943333	11.4131377
Dok6	18.6732957	0.20494195	0.3859603	0.53099231	0.59542411	0.99938111		Dok6	20.8334362	17.648425	18.766663	10.3407001	16.6481172	38.8097993	24.0565972	2.28262753
Rarres1	0.50558436	-0.1500288	0.28261568	-0.5308579	0.59551725	NA		Rarres1	0	1.81009488	1.27231614	0	0	0	0.96226389	0
Dedd2	9.26940831	-0.2159853	0.40702062	-0.5306494	0.59566173	0.99938111		Dedd2	12.0968339	6.78785578	5.72542262	16.5451201	8.3240586	3.52816357	2.88679167	18.2610202
Znrf1	103.027591	0.18120501	0.34150974	0.53059984	0.59569611	0.99938111		Znrf1	71.9089571	78.2866034	82.700549	146.837941	169.810795	68.2111624	55.8113055	150.653417
Pqbp1	56.2996808	0.16620114	0.31327247	0.53053222	0.59574298	0.99938111		Pqbp1	43.6830113	41.1796584	45.4853019	80.6574604	56.6035985	34.1055812	59.6603611	89.0224737
Cab39l	25.3202747	-0.1825353	0.34412079	-0.5304395	0.59580721	0.99938111		Cab39l	36.2905017	26.2463757	27.354797	20.6814001	18.3129289	17.6408179	42.3396111	13.6957652
Appl1	86.4485262	0.16805856	0.31693569	0.53026077	0.59593114	0.99938111		Appl1	69.8928182	76.0239848	94.7875523	82.7256004	46.6147282	143.478652	116.433931	61.6309433
Ppp2r2d	34.3598396	0.17783988	0.33546292	0.53013276	0.59601989	0.99938111		Ppp2r2d	46.3711967	31.6766603	30.5355873	18.6132601	26.6369875	44.6900719	55.8113055	20.5436478
Msl1	102.619487	0.11003669	0.2076012	0.53003879	0.59608504	0.99938111		Msl1	100.134903	101.817837	99.2406588	90.9981605	118.201632	116.429398	91.4150694	102.718239
Ncaph	0.93847121	0.16993976	0.32066524	0.52996002	0.59613965	NA		Ncaph	1.34409266	0	0.63615807	0	0	0	0.96226389	4.56525506
Ammecr1	2.18951523	0.2135963	0.4031355	0.5298375	0.59622461	NA		Ammecr1	0.67204633	1.35757116	1.59039517	4.13628002	6.65924688	1.17605452	1.92452778	0
Tekt4	1.33405474	-0.1785398	0.33700878	-0.5297779	0.59626595	NA		Tekt4	0	0.90504744	0.31807903	6.20442003	0	0	0.96226389	2.28262753
Ric3	45.5606014	0.20197196	0.38126151	0.52974652	0.59628769	0.99938111		Ric3	43.6830113	56.1129411	47.3937761	16.5451201	31.6314227	104.668853	46.1886666	18.2610202
4930426L09Rik	0.25985089	-0.1317438	0.24874076	-0.529643	0.59635944	NA		4930426L09Ri	0.67204633	0.45252372	0.9542371	0	0	0	0	0
5430435G22Rik	0.25985089	-0.1317438	0.24874076	-0.529643	0.59635944	NA		5430435G22R	0.67204633	0.45252372	0.9542371	0	0	0	0	0
Parp10	0.25985089	-0.1317438	0.24874076	-0.529643	0.59635944	NA		Parp10	0.67204633	0.45252372	0.9542371	0	0	0	0	0
Ano6	16.0007891	-0.1984799	0.37483531	-0.5295122	0.59645018	0.99938111		Ano6	18.1452509	25.793852	19.0847421	6.20442003	16.6481172	24.697145	10.5849028	6.84788259
Dnah6	0.45215012	0.15671875	0.29597092	0.52950726	0.59645361	NA		Dnah6	0.67204633	0	0.31807903	0	1.66481172	0	0.96226389	0
Tnk1	1.49609105	-0.2002829	0.37831454	-0.5294085	0.59652209	NA		Tnk1	0	1.81009488	3.18079035	2.06814001	1.66481172	0	0.96226389	2.28262753
Zmym3	94.4682774	0.15020035	0.28372475	0.52938755	0.59653663	0.99938111		Zmym3	82.6616984	67.8785578	78.8836006	128.224681	143.173808	88.2040893	73.1320555	93.5877288
Smim24	0.98306035	0.19587823	0.37004625	0.52933445	0.59657346	NA		Smim24	1.34409266	0.90504744	0.63615807	0	1.66481172	2.35210905	0.96226389	0
Ccdc11	0.29713144	0.11530921	0.21790651	0.52916826	0.59668873	NA		Ccdc11	0	0.45252372	0	0	0	0	1.92452778	0
Kif18b	0.29713144	0.11530921	0.21790651	0.52916826	0.59668873	NA		Kif18b	0	0.45252372	0	0	0	0	1.92452778	0
Lrp2bp	0.29713144	0.11530921	0.21790651	0.52916826	0.59668873	NA		Lrp2bp	0	0.45252372	0	0	0	0	1.92452778	0
Mms22l	0.29713144	0.11530921	0.21790651	0.52916826	0.59668873	NA		Mms22l	0	0.45252372	0	0	0	0	1.92452778	0
Snora65	0.29713144	0.11530921	0.21790651	0.52916826	0.59668873	NA		Snora65	0	0.45252372	0	0	0	0	1.92452778	0
Tbrg3	0.29713144	0.11530921	0.21790651	0.52916826	0.59668873	NA		Tbrg3	0	0.45252372	0	0	0	0	1.92452778	0
Eif1ad	13.2324664	-0.1846025	0.34890557	-0.5290901	0.59674292	0.99938111		Eif1ad	14.1129729	12.2181404	11.4508452	20.6814001	8.3240586	10.5844907	12.5094306	15.9783927
Pex2	14.0669613	-0.1828376	0.34558699	-0.5290639	0.59676115	0.99938111		Pex2	10.7527413	20.3635674	15.2677937	14.4769801	13.3184938	10.5844907	16.3584861	11.4131377
Tubb4b	106.097786	0.15191794	0.2871951	0.5289712	0.59682543	0.99938111		Tubb4b	104.839227	78.7391271	89.6982877	126.156541	163.151549	65.8590533	103.9245	116.414004
Gucy2f	0.92238245	-0.1598709	0.30223811	-0.5289569	0.59683534	NA		Gucy2f	0	4.07271347	0.9542371	0	0	2.35210905	0	0
Slc6a15	36.0503335	-0.1884789	0.35638216	-0.5288675	0.59689738	0.99938111		Slc6a15	27.5538995	49.3250854	49.9384084	31.0221002	21.6425524	49.39429	48.1131944	11.4131377
Ypel4	42.3956455	0.17040911	0.32223417	0.52883625	0.59691905	0.99938111		Ypel4	40.3227797	31.6766603	34.6706148	49.6353602	44.9499164	35.2816357	25.0188611	77.6093361
C1qtnf7	0.31026765	-0.1149568	0.21741267	-0.5287491	0.59697952	NA		C1qtnf7	0.67204633	1.81009488	0	0	0	0	0	0
Rassf9	0.31026765	-0.1149568	0.21741267	-0.5287491	0.59697952	NA		Rassf9	0.67204633	1.81009488	0	0	0	0	0	0
Tshz3	21.5054361	-0.1833787	0.34682612	-0.5287339	0.59699004	0.99938111		Tshz3	30.9141311	27.1514231	24.4920857	10.3407001	19.9777406	21.1689814	28.8679167	9.13051012
Uimc1	4.89409641	0.2183791	0.41311697	0.52861324	0.59707378	0.99938111		Uimc1	7.39250961	4.07271347	3.81694841	2.06814001	1.66481172	8.23238167	9.62263889	2.28262753
Mest	57.8167359	0.17850048	0.33798189	0.52813624	0.59740478	0.99938111		Mest	38.3066407	78.7391271	63.6158069	31.0221002	43.2851047	104.668853	48.1131944	54.7830607
Has1	0.88973927	0.15311202	0.28991108	0.52813441	0.59740605	NA		Has1	0	0	1.59039517	0	0	0	0.96226389	4.56525506
Crlf3	4.93400697	0.21908798	0.41487933	0.5280764	0.59744631	0.99938111		Crlf3	4.7043243	3.16766603	1.90847421	8.27256004	6.65924688	1.17605452	6.73584722	6.84788259
Ccdc81	0.65474047	-0.1656409	0.31371725	-0.5279944	0.59750323	NA		Ccdc81	0	1.35757116	0.63615807	2.06814001	0	1.17605452	0	0
Plb1	0.74546887	0.16616738	0.31484596	0.52777359	0.59765649	NA		Plb1	1.34409266	0.45252372	0.31807903	0	0	0	3.84905555	0
Ak2	5.4091338	0.21470039	0.40692674	0.52761435	0.59776703	0.99938111		Ak2	6.04841696	4.97776091	4.45310648	4.13628002	1.66481172	5.88027262	11.5471667	4.56525506
4933417G07Rik	2.94127693	0.21962924	0.416301	0.52757316	0.59779562	NA		4933417G07R	3.36023164	2.71514231	1.90847421	2.06814001	4.99443516	2.35210905	3.84905555	2.28262753
Ska2	7.32445056	0.21360011	0.40495051	0.52747214	0.59786576	0.99938111		Ska2	6.72046328	5.43028463	9.86045007	4.13628002	3.32962344	15.2887088	11.5471667	2.28262753
Opn3	6.26662321	0.21579747	0.40923488	0.52731934	0.59797185	0.99938111		Opn3	0.67204633	1.81009488	6.04350166	12.4088401	11.653682	0	3.84905555	13.6957652
Rgs10	12.7203337	-0.1850539	0.3510289	-0.5271757	0.59807157	0.99938111		Rgs10	12.0968339	14.480759	16.8581888	12.4088401	9.98887032	11.7605452	17.32075	6.84788259
Fam53a	9.47694198	-0.1963984	0.3726781	-0.5269922	0.59819902	0.99938111		Fam53a	13.4409266	8.14542694	9.86045007	10.3407001	13.3184938	9.40843619	6.73584722	4.56525506
Dopey2	64.268174	0.14360784	0.27256853	0.52686875	0.59828474	0.99938111		Dopey2	53.0916599	63.3533207	66.1604392	57.9079203	84.9053977	75.2674895	40.4150833	73.044081
Glrx2	47.8586635	0.13398122	0.25434597	0.52676762	0.59835497	0.99938111		Glrx2	51.075521	43.8948007	46.7576181	39.2946602	43.2851047	43.5140174	62.5471528	52.5004332
Gas5	3.51117365	0.21910439	0.415976	0.52672364	0.59838552	NA		Gas5	5.37637063	1.35757116	2.86271131	2.06814001	1.66481172	1.17605452	6.73584722	6.84788259
Ahnak	7.18591579	-0.2123272	0.40311259	-0.5267194	0.59838849	0.99938111		Ahnak	6.04841696	11.313093	8.2700549	6.20442003	13.3184938	2.35210905	7.69811111	2.28262753
Trappc4	31.5905135	0.16880862	0.32057349	0.52658322	0.59848304	0.99938111		Trappc4	28.8979921	21.2686148	28.6271131	39.2946602	24.9721758	19.9929269	39.4528194	50.2178057
D030018L15Rik	2.92191315	-0.2191826	0.41626607	-0.5265445	0.59850994	NA		D030018L15Ri	3.36023164	3.62018975	4.45310648	2.06814001	0	4.7042181	2.88679167	2.28262753
Slc36a4	28.4206434	-0.1577469	0.29962152	-0.5264872	0.59854974	0.99938111		Slc36a4	28.2259458	38.4645161	25.4463228	28.9539601	31.6314227	32.9295267	21.1698055	20.5436478
Cmc2	9.06568355	-0.2021056	0.38388553	-0.5264735	0.59855923	0.99938111		Cmc2	9.4086486	6.78785578	7.31581779	18.6132601	6.65924688	8.23238167	8.660375	6.84788259
Alg6	3.00133344	0.21400795	0.40650108	0.52646343	0.59856626	NA		Alg6	0.67204633	4.07271347	4.77118552	0	1.66481172	7.05632714	5.77358333	0
Ccdc3	10.6852224	-0.2070152	0.39337745	-0.5262508	0.59871395	0.99938111		Ccdc3	13.4409266	15.3858064	14.3135566	2.06814001	18.3129289	7.05632714	5.77358333	9.13051012
Alcam	133.810782	0.20911294	0.39738693	0.52621998	0.59873537	0.99938111		Alcam	101.478996	188.702391	159.675675	24.8176801	78.2461509	318.710776	121.24525	77.6093361
Fbxo45	52.7172023	0.1357796	0.25803012	0.52621607	0.59873809	0.99938111		Fbxo45	46.3711967	56.1129411	51.8468826	45.4990802	38.2906696	68.2111624	60.622625	54.7830607
Nat6	13.8210827	-0.2046945	0.38918424	-0.5259577	0.59891758	0.99938111		Nat6	11.4247876	13.1231878	9.54237104	26.8858201	11.653682	7.05632714	5.77358333	25.1089028
Prim2	3.22172438	-0.2187698	0.41595688	-0.5259434	0.59892756	NA		Prim2	3.36023164	5.88280835	3.81694841	2.06814001	1.66481172	7.05632714	1.92452778	0
Jazf1	30.4271127	0.15908793	0.30249191	0.52592459	0.5989406	0.99938111		Jazf1	30.2420848	28.0564706	28.3090341	28.9539601	23.3073641	29.4013631	50.0377222	25.1089028
Thap4	11.2912214	-0.1875219	0.35658716	-0.5258796	0.59897186	0.99938111		Thap4	14.1129729	10.8605693	11.1327662	12.4088401	11.653682	5.88027262	10.5849028	13.6957652
Stau2	71.3223066	0.21489665	0.40864279	0.52587896	0.59897231	0.99938111		Stau2	77.9573741	79.1916508	80.1559167	12.4088401	29.966611	112.901234	159.735805	18.2610202
Tsga10	4.57203374	-0.2164184	0.41153881	-0.525876	0.59897434	NA		Tsga10	6.04841696	6.33533207	9.224292	0	0	8.23238167	6.73584722	0
C330027C09Rik	1.51676651	-0.2045512	0.38907347	-0.5257393	0.59906934	NA		C330027C09Ri	0.67204633	3.62018975	2.86271131	0	1.66481172	2.35210905	0.96226389	0
AW554918	13.6839717	0.18188754	0.34607639	0.52557048	0.59918667	0.99938111		AW554918	14.1129729	13.1231878	12.0870033	12.4088401	11.653682	19.9929269	19.2452778	6.84788259
Zfp738	13.4327101	0.21505304	0.40922479	0.52551324	0.59922645	0.99938111		Zfp738	11.4247876	14.0282353	19.0847421	2.06814001	3.32962344	24.697145	25.981125	6.84788259
Arhgef40	11.5807931	0.20202585	0.38449089	0.52543728	0.59927925	0.99938111		Arhgef40	14.1129729	7.69290322	6.99773876	12.4088401	13.3184938	4.7042181	10.5849028	22.8262753
Aga	1.31272087	0.18892729	0.35986991	0.52498772	0.59959173	NA		Aga	0	1.81009488	0.63615807	2.06814001	0	1.17605452	4.81131944	0
1810021B22Rik	1.60281313	-0.1919467	0.36583724	-0.5246777	0.59980723	NA		1810021B22Ri	3.36023164	0.90504744	0	4.13628002	0	1.17605452	0.96226389	2.28262753
Epha6	60.6376088	0.2089063	0.39820554	0.52461927	0.59984789	0.99938111		Epha6	50.4034746	88.2421252	59.4807795	16.5451201	38.2906696	162.295524	35.6037639	34.239413
Sec31a	115.962936	0.12717277	0.24244368	0.5245456	0.59989911	0.99938111		Sec31a	113.575829	92.3148387	98.9225797	138.565381	154.82749	118.781507	94.3018611	116.414004
B3gat3	55.5760412	0.17441972	0.33260386	0.52440677	0.59999565	0.99938111		B3gat3	38.3066407	56.1129411	51.5288036	57.9079203	89.8998329	31.7534722	34.6415	84.4572186
1110004F10Rik	48.9610568	0.16960355	0.32352347	0.52423879	0.60011246	0.99938111		1110004F10Ri	34.2743627	38.9170398	39.7598793	70.3167603	38.2906696	30.5774176	59.6603611	79.8919636
Mppe1	10.2057227	-0.1901342	0.36272576	-0.5241818	0.60015209	0.99938111		Mppe1	12.7688802	12.2181404	12.7231614	6.20442003	8.3240586	12.9365998	9.62263889	6.84788259
Kdr	2.19376721	0.19374105	0.36967269	0.52408808	0.60021728	NA		Kdr	0	4.07271347	1.90847421	0	8.3240586	0	0.96226389	2.28262753
Trappc12	35.0511966	0.14037664	0.26785929	0.52406858	0.60023084	0.99938111		Trappc12	36.9625481	30.7716129	32.4440615	31.0221002	41.620293	37.6337448	28.8679167	41.0872956
Sin3a	30.1038017	-0.1666422	0.31801486	-0.5240075	0.6002733	0.99938111		Sin3a	40.3227797	28.5089943	29.2632712	31.0221002	44.9499164	30.5774176	20.2075417	15.9783927
Lrrc16b	19.3026667	0.18887597	0.36060159	0.5237802	0.60043143	0.99938111		Lrrc16b	12.0968339	14.0282353	12.0870033	35.1583802	29.966611	16.4647633	16.3584861	18.2610202
Syt13	96.36365	0.16149765	0.30836179	0.52372785	0.60046785	0.99938111		Syt13	60.4841696	110.415787	93.5152361	95.1344405	96.5590798	157.591306	56.7735694	100.435611
Magi2	119.365287	0.14509472	0.27706974	0.52367581	0.60050404	0.99938111		Magi2	120.296293	135.304592	105.920318	88.9300204	86.5702095	183.464506	138.566	95.8703563
Tmem185b	8.37656903	-0.2107787	0.40254276	-0.5236183	0.60054406	0.99938111		Tmem185b	5.37637063	14.0282353	7.95197586	10.3407001	3.32962344	15.2887088	3.84905555	6.84788259
Gadd45b	5.02767416	0.21561922	0.41180125	0.5236002	0.60055664	0.99938111		Gadd45b	4.03227797	1.81009488	5.72542262	6.20442003	1.66481172	5.88027262	5.77358333	9.13051012
Pygo1	28.9368814	0.15703435	0.29996424	0.52351024	0.60061923	0.99938111		Pygo1	32.9302701	28.961518	27.354797	16.5451201	21.6425524	34.1055812	28.8679167	41.0872956
Vldlr	60.3754123	0.20802152	0.39751088	0.52331025	0.60075837	0.99938111		Vldlr	43.010965	78.2866034	72.2039408	20.6814001	38.2906696	164.647633	38.4905555	27.3915304
Sema4c	6.48148832	0.20692372	0.395485	0.52321508	0.60082459	0.99938111		Sema4c	4.7043243	6.33533207	4.45310648	8.27256004	11.653682	7.05632714	4.81131944	4.56525506
Pecr	1.73125148	-0.2057382	0.39332961	-0.5230682	0.60092681	NA		Pecr	4.03227797	0.90504744	1.59039517	2.06814001	3.32962344	0	1.92452778	0
Avpi1	10.2143274	-0.2137742	0.40873886	-0.5230092	0.60096786	0.99938111		Avpi1	12.7688802	6.78785578	4.45310648	22.7495401	3.32962344	4.7042181	8.660375	18.2610202
Cacng7	70.2443521	-0.1805741	0.34533282	-0.5228989	0.60104464	0.99938111		Cacng7	50.4034746	81.4542694	68.0689134	103.407001	119.866444	41.1619083	33.6792361	63.9135709
Usp22	284.466363	0.11381202	0.21770747	0.52277497	0.60113086	0.99938111		Usp22	243.952817	247.982998	257.962097	341.243102	354.604896	266.964377	243.452764	319.567854
C730002L08Rik	1.07082226	-0.1913938	0.36612096	-0.522761	0.60114058	NA		C730002L08Ri	1.34409266	3.16766603	0.9542371	0	0	1.17605452	1.92452778	0
Pnpla6	24.1683206	0.19784388	0.37854119	0.52264823	0.60121907	0.99938111		Pnpla6	9.4086486	31.2241366	21.3112953	24.8176801	39.9554813	30.5774176	8.660375	27.3915304
Rassf8	10.2078495	-0.192056	0.36755567	-0.5225223	0.60130675	0.99938111		Rassf8	10.7527413	11.313093	9.54237104	14.4769801	4.99443516	14.1126543	9.62263889	6.84788259
Nudt2	5.50811736	-0.2159076	0.41358438	-0.52204	0.60164249	0.99938111		Nudt2	8.73660227	8.59795066	6.04350166	2.06814001	3.32962344	4.7042181	10.5849028	0
Myh2	1.06247977	0.18253033	0.34966332	0.52201738	0.60165823	NA		Myh2	0.67204633	0	0.63615807	2.06814001	1.66481172	1.17605452	0	2.28262753
Mettl23	4.70788347	-0.2171003	0.41606164	-0.5217983	0.60181075	0.99938111		Mettl23	4.7043243	3.62018975	7.63389683	4.13628002	1.66481172	3.52816357	0.96226389	11.4131377
Cacna1i	83.4831808	0.19493915	0.37394974	0.52129773	0.60215938	0.99938111		Cacna1i	42.3389187	81.9067931	59.1627004	119.952121	136.514561	97.6125255	23.0943333	107.283494
Swap70	4.73437452	-0.2163308	0.41504266	-0.5212254	0.60220974	0.99938111		Swap70	11.4247876	2.26261859	3.81694841	4.13628002	3.32962344	3.52816357	4.81131944	4.56525506
Sema4g	49.4681737	0.16936544	0.32493705	0.5212254	0.60220976	0.99938111		Sema4g	29.5700384	55.2078937	45.4853019	51.7035003	48.2795399	54.0985081	26.9433889	84.4572186
Ttn	0.9403476	-0.18473	0.35443172	-0.5212005	0.60222714	NA		Ttn	2.68818531	1.35757116	0.63615807	0	1.66481172	1.17605452	0	0
Clec2d	1.05520649	-0.1769949	0.33960628	-0.5211767	0.60224371	NA		Clec2d	0.67204633	3.16766603	1.27231614	0	3.32962344	0	0	0
Adam22	194.810423	0.19804347	0.38000086	0.52116584	0.60225125	0.99938111		Adam22	153.226563	276.944516	208.341768	64.1123403	146.503431	446.900719	159.735805	102.718239

GeneID	Base mean	log2(FC)	StdErr	Wald-Stats	P-value	P-adj		GeneID	Normalised expression for Chow#1	Normalised expression for Chow#2	Normalised expression for Chow#3	Normalised expression for Chow#4	Normalised expression for HFD#1	Normalised expression for HFD#2	Normalised expression for HFD#3	Normalised expression for HFD#4
Fank1	2.98597415	-0.2142716	0.4111817	-0.5211117	0.60228894	NA		Fank1	6.72046328	1.35757116	5.08926455	0	1.66481172	3.52816357	0.96226389	4.56525506
2610507I01Rik	12.8767211	-0.1859826	0.35697266	-0.5209995	0.60236711	0.99938111		2610507I01Ri	14.1129729	15.8383302	10.8146872	16.5451201	4.99443516	12.9365998	16.3584861	11.4131377
Slc16a12	1.87973074	0.17500101	0.33601258	0.52081685	0.60249437	NA		Slc16a12	2.01613899	2.71514231	0	0	0	1.17605452	0	9.13051012
Fam105a	6.60245458	0.21355647	0.41012213	0.52071434	0.60256578	0.99938111		Fam105a	6.04841696	9.95552182	6.36158069	0	3.32962344	12.9365998	9.62263889	4.56525506
Zbtb12	3.18796992	-0.2167375	0.41636585	-0.5205458	0.60268323	NA		Zbtb12	5.37637063	3.62018975	3.81694841	2.06814001	0	3.52816357	4.81131944	2.28262753
Sarm1	41.5349319	0.19360442	0.37197357	0.52047897	0.60272978	0.99938111		Sarm1	28.8979921	23.9837571	25.1282437	74.4530404	63.2628454	28.2253086	22.1320694	66.1961984
Ict1	17.1966009	0.17500418	0.33633568	0.52032593	0.60283643	0.99938111		Ict1	19.4893435	18.1009488	11.7689243	14.4769801	24.9721758	12.9365998	22.1320694	13.6957652
Rbpj	22.7762155	0.1761875	0.33863964	0.52028022	0.60286828	0.99938111		Rbpj	27.5538995	16.7433776	17.4943469	24.8176801	16.6481172	23.5210905	39.4528194	15.9783927
Slc9a5	14.7106328	-0.2006446	0.38569936	-0.5202099	0.60291726	0.99938111		Slc9a5	6.04841696	26.6988994	16.2220308	14.4769801	19.9777406	10.5844907	7.69811111	15.9783927
Armc6	10.3670487	0.20343145	0.39110478	0.52014565	0.60296206	0.99938111		Armc6	8.06455594	5.88280835	8.2700549	16.5451201	4.99443516	5.88027262	17.32075	15.9783927
Gm13710	0.75411905	-0.173712	0.3339719	-0.5201396	0.60296625	NA		Gm13710	1.34409266	1.81009488	0.9542371	0	0	0	1.92452778	0
Ppp1r15a	7.01034798	-0.2047188	0.39361857	-0.5200945	0.60299772	0.99938111		Ppp1r15a	8.06455594	7.2403795	6.67965972	10.3407001	8.3240586	3.52816357	9.62263889	2.28262753
Gm13483	0.4801286	0.12494238	0.24024416	0.52006419	0.60301884	NA		Gm13483	0	0	0.9542371	0	0	0	2.88679167	0
Simc1	16.5269679	0.17422788	0.33517809	0.51980687	0.60319819	0.99938111		Simc1	13.4409266	16.2908539	17.8124259	14.4769801	14.9833055	25.8731995	20.2075417	9.13051012
Lman1	16.6206251	-0.2100205	0.40403598	-0.5198065	0.60319847	0.99938111		Lman1	16.1291119	33.9392789	21.6293743	2.06814001	11.653682	24.697145	18.2830139	4.56525506
Ifi30	0.6874354	-0.1539404	0.29616278	-0.519783	0.60321485	NA		Ifi30	0	2.26261859	0.9542371	0	0	0	0	2.28262753
Alpl	2.48785075	0.20981758	0.40393675	0.51943176	0.60345969	NA		Alpl	4.03227797	1.81009488	1.59039517	0	6.65924688	3.52816357	0	2.28262753
Snord91a	1.63319304	0.20165312	0.38840867	0.5191777	0.60363683	NA		Snord91a	0.67204633	0.45252372	1.90847421	2.06814001	3.32962344	2.35210905	0	2.28262753
Dscaml1	47.2953897	0.17805209	0.34295987	0.51916305	0.60364704	0.99938111		Dscaml1	36.2905017	67.8785578	42.9406697	24.8176801	61.5980337	70.5632714	24.0565972	50.2178057
Trmt10b	7.75705918	0.20050576	0.38649905	0.51877426	0.60391817	0.99938111		Trmt10b	6.04841696	7.69290322	9.86045007	4.13628002	6.65924688	10.5844907	12.5094306	4.56525506
Aplp2	275.119815	0.17114007	0.33001913	0.51857619	0.60405631	0.99938111		Aplp2	206.318223	338.035218	298.040055	175.791901	218.090335	535.104808	169.358444	260.219539
Slc30a9	123.098044	-0.1246499	0.24039799	-0.5185148	0.60409912	0.99938111		Slc30a9	144.489961	120.823833	131.048562	119.952121	79.9109626	112.901234	150.113167	125.544514
Firre	3.26818472	0.2158208	0.41627495	0.51845734	0.60413921	NA		Firre	4.7043243	1.81009488	2.86271131	2.06814001	3.32962344	2.35210905	6.73584722	2.28262753
Pdlim7	28.2916116	-0.2114364	0.4078336	-0.5184379	0.60415278	0.99938111		Pdlim7	31.5861774	14.9332827	14.3135566	66.1804803	19.9777406	9.40843619	10.5849028	59.3483158
Prps1	33.9542685	0.16130424	0.31124392	0.51825667	0.6042792	0.99938111		Prps1	34.2743627	22.6261859	42.6225906	26.8858201	28.3017992	29.4013631	50.9999861	36.5220405
Atp13a3	56.2744956	0.20258835	0.39093191	0.518219	0.60430548	0.99938111		Atp13a3	36.9625481	79.6441745	63.9338859	20.6814001	31.6314227	136.422325	55.8113055	25.1089028
Slc12a2	25.4866105	-0.1793693	0.34613727	-0.5182027	0.60431685	0.99938111		Slc12a2	18.1452509	46.1574193	26.4005599	18.6132601	26.6369875	31.7534722	20.2075417	15.9783927
Tpt1	49.0473743	-0.2045983	0.39493564	-0.5180549	0.60441998	0.99938111		Tpt1	39.6507334	33.0342315	35.3067728	109.611421	39.9554813	11.7605452	31.7547083	91.3051012
Prkaa2	44.2193281	0.14164849	0.27342842	0.51804595	0.60442621	0.99938111		Prkaa2	35.6184554	47.9675142	46.12146	37.2265202	39.9554813	39.9858538	63.5094166	43.3699231
Nup85	12.0843554	-0.193696	0.37392459	-0.5180083	0.60445248	0.99938111		Nup85	8.06455594	12.2181404	12.0870033	20.6814001	13.3184938	4.7042181	9.62263889	15.9783927
Fam49a	173.689984	0.17757275	0.34293823	0.51779807	0.60459916	0.99938111		Fam49a	200.941852	171.053966	201.344029	66.1804803	121.531256	237.563014	279.056528	111.848749
Nob1	7.99381242	-0.2081494	0.40205978	-0.5177076	0.60466232	0.99938111		Nob1	12.7688802	6.33533207	10.4966081	4.13628002	3.32962344	7.05632714	3.84905555	15.9783927
Rit2	100.025773	-0.1639575	0.31674676	-0.5176297	0.60471668	0.99938111		Rit2	81.9896521	84.6219354	84.9271022	179.928181	56.6035985	78.7956531	126.056569	107.283494
Fzd10	0.28897603	-0.1140205	0.22038074	-0.5173797	0.60489115	NA		Fzd10	0	1.35757116	0.9542371	0	0	0	0	0
Hist1h2bn	1.75293849	-0.204481	0.39525209	-0.5173433	0.60491653	NA		Hist1h2bn	3.36023164	3.62018975	1.59039517	0	0	3.52816357	1.92452778	0
Mier1	24.2385672	0.16147982	0.31214338	0.51732578	0.60492876	0.99938111		Mier1	25.5377605	23.5312334	23.5378486	18.6132601	28.3017992	34.1055812	28.8679167	11.4131377
2310009A05Rik	3.48212706	-0.2153257	0.41630296	-0.5172332	0.60499336	NA		2310009A05Ri	7.39250961	3.62018975	4.77118552	0	1.66481172	2.35210905	5.77358333	2.28262753
Rad23b	186.488548	-0.1087258	0.21034645	-0.5168892	0.60523348	0.99938111		Rad23b	239.248493	199.110436	165.719177	171.655621	164.81636	197.57716	178.018819	175.76232
Dnajb5	52.850374	-0.1631461	0.31578481	-0.5166368	0.60540969	0.99938111		Dnajb5	75.2691888	49.3250854	56.6180681	41.3628002	59.9332219	25.8731995	41.3773472	73.044081
Helz	48.1047505	-0.161778	0.31314241	-0.5166276	0.60541614	0.99938111		Helz	53.7637063	60.6381783	59.1627004	31.0221002	56.6035985	62.3308898	38.4905555	22.8262753
Cdk2ap1	16.2694612	-0.1899464	0.3677307	-0.5165367	0.60547958	0.99938111		Cdk2ap1	17.4732045	19.0059962	26.0824808	8.27256004	14.9833055	17.6408179	22.1320694	4.56525506
Smndc1	14.1012617	-0.202759	0.3926687	-0.5163614	0.60560201	0.99938111		Smndc1	15.4570656	12.2181404	14.3135566	22.7495401	1.66481172	16.4647633	23.0943333	6.84788259
Ncbp2	31.746793	-0.181435	0.3514553	-0.5162393	0.6056873	0.99938111		Ncbp2	38.978687	28.0564706	27.354797	45.4990802	8.3240586	34.1055812	44.2641389	27.3915304
Bre	24.6486267	-0.1811202	0.35087704	-0.5161929	0.6057197	0.99938111		Bre	40.994826	24.8888045	29.8994292	10.3407001	23.3073641	29.4013631	26.9433889	11.4131377
Sntg2	1.44578788	0.18615673	0.36065044	0.51616943	0.60573608	NA		Sntg2	0.67204633	0.45252372	0.31807903	4.13628002	0	1.17605452	4.81131944	0
Tmed5	16.9014801	0.19766301	0.38297644	0.51612314	0.6057684	0.99938111		Tmed5	11.4247876	23.5312334	19.0847421	6.20442003	9.98887032	35.2816357	18.2830139	11.4131377
Ostc	15.7233825	-0.2018616	0.3911637	-0.5160541	0.6058166	0.99938111		Ostc	12.0968339	23.9837571	27.0367179	6.20442003	9.98887032	18.8168724	23.0943333	4.56525506
Glis3	3.13519552	-0.213068	0.41295927	-0.5159541	0.60588644	NA		Glis3	5.37637063	4.07271347	3.49886938	2.06814001	3.32962344	0	6.73584722	0
Casp3	8.06013129	0.19861387	0.38498939	0.51589439	0.60592817	0.99938111		Casp3	9.4086486	5.88280835	9.224292	4.13628002	4.99443516	8.23238167	13.4716944	9.13051012
Dyx1c1	0.78947161	0.14786216	0.28664969	0.51582876	0.60597401	NA		Dyx1c1	0	1.81009488	0	0	3.32962344	1.17605452	0	0
Mir8114	2.22737792	0.21111502	0.40933981	0.51574515	0.60603242	NA		Mir8114	1.34409266	2.71514231	2.86271131	0	1.66481172	1.17605452	5.77358333	2.28262753
Polr3c	13.3245594	-0.197287	0.38254992	-0.5157157	0.60605296	0.99938111		Polr3c	16.1291119	14.0282353	9.86045007	20.6814001	18.3129289	14.1126543	13.4716944	0
Efcab10	1.3463099	0.19274245	0.37378604	0.51564913	0.60609949	NA		Efcab10	0.67204633	0.45252372	1.59039517	2.06814001	0	1.17605452	4.81131944	0
3830406C13Rik	12.4307986	-0.212448	0.41206439	-0.5155699	0.60615485	0.99938111		3830406C13Ri	14.7850192	17.648425	12.7231614	12.4088401	4.99443516	7.05632714	29.8301805	0
Pcsk2os1	7.08395006	-0.2023448	0.39251838	-0.5155041	0.6062008	0.99938111		Pcsk2os1	7.39250961	10.8605693	4.77118552	8.27256004	6.65924688	7.05632714	4.81131944	6.84788259
Zcchc11	27.3106493	0.18769328	0.36411727	0.51547482	0.60622126	0.99938111		Zcchc11	30.9141311	25.793852	25.1282437	18.6132601	8.3240586	45.8661264	43.301875	20.5436478
Larp7	6.33447384	0.21368727	0.41458871	0.5154199	0.60625963	0.99938111		Larp7	8.73660227	4.52523719	6.99773876	2.06814001	3.32962344	10.5844907	14.4339583	0
Ttc8	10.9567963	0.1893545	0.36742354	0.51535758	0.60630317	0.99938111		Ttc8	7.39250961	10.8605693	10.8146872	12.4088401	8.3240586	11.7605452	19.2452778	6.84788259
Ccdc38	1.33405568	-0.188279	0.36549832	-0.5151295	0.60646253	NA		Ccdc38	2.01613899	2.26261859	2.54463228	0	0	0	3.84905555	0
Adam11	53.7032052	0.16440566	0.31924609	0.51498096	0.60656633	0.99938111		Adam11	36.2905017	61.9957495	38.8056422	62.0442003	71.586904	48.2182355	30.7924444	79.8919636
Eif5	108.944768	0.18325509	0.35588568	0.51492685	0.60660413	0.99938111		Eif5	124.328571	113.13093	113.872294	47.5672202	43.2851047	165.823688	172.245236	91.3051012
Xpa	2.78132884	0.21066128	0.40921171	0.51479778	0.60669434	NA		Xpa	2.01613899	1.81009488	4.77118552	0	0	2.35210905	6.73584722	4.56525506
Zcwpw1	2.46262342	-0.2053622	0.39893445	-0.5147767	0.60670909	NA		Zcwpw1	1.34409266	2.71514231	0.9542371	8.27256004	0	3.52816357	2.88679167	0
Acaa1a	10.9652932	0.20679829	0.40172938	0.51477014	0.60671366	0.99938111		Acaa1a	6.04841696	5.88280835	6.36158069	22.7495401	19.9777406	4.7042181	10.5849028	11.4131377
Gtf3c4	44.0064841	0.14068939	0.27335165	0.51468278	0.60677471	0.99938111		Gtf3c4	48.3873356	39.8220873	38.4875632	41.3628002	51.6091633	58.8027262	46.1886666	27.3915304
Rps6	18.1392438	0.19116519	0.37150528	0.51456924	0.60685406	0.99938111		Rps6	22.1775288	14.0282353	17.4943469	12.4088401	14.9833055	8.23238167	37.5282917	18.2610202
Rab30	17.9459109	0.20211907	0.39279656	0.51456427	0.60685754	0.99938111		Rab30	18.8172972	13.1231878	22.5836114	10.3407001	4.99443516	27.0492541	37.5282917	9.13051012
Zfp54	0.75955126	-0.1786685	0.34723309	-0.5145492	0.60686806	NA		Zfp54	0.67204633	1.35757116	1.90847421	0	0	1.17605452	0.96226389	0
Klf11	8.36331958	0.20336762	0.39529491	0.51447063	0.60692299	0.99938111		Klf11	6.04841696	7.2403795	5.72542262	10.3407001	9.98887032	3.52816357	5.77358333	18.2610202
Crb1	3.73427392	0.21372585	0.4154603	0.51443146	0.60695037	NA		Crb1	4.7043243	1.81009488	4.45310648	2.06814001	3.32962344	3.52816357	7.69811111	2.28262753
Stx16	59.5963495	0.13891744	0.27021179	0.51410576	0.60717805	0.99938111		Stx16	40.994826	55.6604174	47.7118552	84.7937404	61.5980337	57.6266717	64.4716805	63.9135709
Nfyc	10.8400756	-0.1928394	0.37509859	-0.5141033	0.60717976	0.99938111		Nfyc	16.8011582	11.7656167	11.7689243	6.20442003	4.99443516	7.05632714	14.4339583	13.6957652
Lipg	4.31950578	0.21398896	0.41632025	0.51400084	0.60725141	NA		Lipg	8.73660227	2.26261859	2.86271131	0	8.3240586	5.88027262	1.92452778	4.56525506
Rab5a	65.7315243	0.19328782	0.37606435	0.51397539	0.6072692	0.99938111		Rab5a	65.8605402	68.7836053	74.7485731	28.9539601	21.6425524	96.436471	126.056569	43.3699231
Gnb5	100.481098	0.13629443	0.26522901	0.51387452	0.60733972	0.99938111		Gnb5	109.543552	68.7836053	99.5587378	101.338861	84.9053977	90.5561984	96.2263889	152.936045
Spats2	15.6923495	0.1830053	0.35614538	0.51384999	0.60735687	0.99938111		Spats2	14.1129729	19.4585199	16.8581888	6.20442003	21.6425524	21.1689814	19.2452778	6.84788259
Hist3h2bb-ps	2.33676523	0.20214819	0.39341655	0.51382738	0.60737268	NA		Hist3h2bb-ps	2.68818531	0	1.59039517	4.13628002	3.32962344	1.17605452	5.77358333	0
4930579G24Rik	0.86937786	-0.1833858	0.35692221	-0.5137978	0.60739336	NA		4930579G24Ri	2.01613899	1.35757116	0.9542371	0	1.66481172	0	0.96226389	0
Ethe1	14.8365122	0.21090558	0.41057819	0.51367944	0.60747613	0.99938111		Ethe1	7.39250961	13.1231878	6.67965972	24.8176801	16.6481172	3.52816357	7.69811111	38.804668
Pde3b	5.30925594	0.20956326	0.40824049	0.51333287	0.6077185	0.99938111		Pde3b	3.36023164	5.88280835	6.36158069	2.06814001	1.66481172	8.23238167	5.77358333	9.13051012
Ankrd37	2.3417247	-0.2097283	0.40880655	-0.5130258	0.60793331	NA		Ankrd37	5.37637063	0.90504744	2.86271131	2.06814001	0	2.35210905	2.88679167	2.28262753
Gm21949	96.723851	-0.1977888	0.38565526	-0.5128643	0.60804627	0.99938111		Gm21949	133.737219	138.924782	123.732744	28.9539601	44.9499164	118.781507	148.188639	36.5220405
Hps4	7.8451767	-0.2024221	0.3949323	-0.5125488	0.60826699	0.99938111		Hps4	10.0806949	6.78785578	5.40734359	12.4088401	8.3240586	3.52816357	4.81131944	11.4131377
Rbfox1	277.911906	-0.1144119	0.22326856	-0.5124407	0.60834261	0.99938111		Rbfox1	336.023164	303.643415	313.307849	204.745861	258.045817	291.661522	294.45275	221.414871
Trappc6a	6.67697242	-0.2108725	0.41151234	-0.512433	0.60834801	0.99938111		Trappc6a	5.37637063	2.71514231	1.59039517	22.7495401	13.3184938	1.17605452	1.92452778	4.56525506
Lrrc2	0.7886764	-0.1675174	0.32696697	-0.5123375	0.60841481	NA		Lrrc2	0	2.26261859	1.90847421	0	0	1.17605452	0.96226389	0
Gm13826	45.1033083	-0.1891268	0.36922188	-0.5122308	0.60848948	0.99938111		Gm13826	78.6294204	50.6826565	42.9406697	24.8176801	21.6425524	32.9295267	81.7924305	27.3915304
Dpy19l3	41.6510632	-0.172238	0.33634054	-0.5120941	0.60858518	0.99938111		Dpy19l3	36.2905017	68.7836053	49.6203294	22.7495401	34.9610461	59.9787807	28.8679167	31.9567854
2310015A10Rik	5.34254973	0.21040417	0.41093109	0.51201814	0.60863831	0.99938111		2310015A10Ri	6.04841696	2.71514231	3.49886938	6.20442003	6.65924688	2.35210905	3.84905555	11.4131377
Svip	37.1314942	-0.1735576	0.33899345	-0.5119793	0.60866553	0.99938111		Svip	46.3711967	45.7048956	38.1694841	31.0221002	19.9777406	31.7534722	63.5094166	20.5436478
Trp53	9.98483574	-0.1963831	0.38366615	-0.5118592	0.60874953	0.99938111		Trp53	12.7688802	7.69290322	9.86045007	12.4088401	11.653682	4.7042181	4.81131944	15.9783927
Trpc2	0.39105547	0.14836925	0.28992461	0.51175113	0.6088252	NA		Trpc2	0.67204633	0	0.31807903	0	0	1.17605452	0.96226389	0
Tipin	6.6331101	-0.2079583	0.40644101	-0.5116569	0.60889118	0.99938111		Tipin	8.06455594	5.43028463	10.8146872	6.20442003	1.66481172	7.05632714	11.5471667	2.28262753
Cnot6l	45.117253	0.14892199	0.29109688	0.5115891	0.60893862	0.99938111		Cnot6l	36.2905017	44.7998482	50.5745665	39.2946602	34.9610461	57.6266717	65.4339444	31.9567854
4833424O15Rik	32.4000178	0.19598109	0.38314841	0.51150178	0.60899975	0.99938111		4833424O15Ri	18.8172972	43.8948007	34.0344567	20.6814001	13.3184938	68.2111624	44.2641389	15.9783927
Uaca	1.37770695	0.19451097	0.38039237	0.51134297	0.60911092	NA		Uaca	0.67204633	2.26261859	0.9542371	0	4.99443516	1.17605452	0.96226389	0
Lsmem1	0.58022348	0.16078671	0.31445157	0.51132426	0.60912402	NA		Lsmem1	0	0.90504744	0.63615807	0	0	1.17605452	1.92452778	0
Rab11a	121.853511	0.12376203	0.24205906	0.51128859	0.60914899	0.99938111		Rab11a	118.9522	112.678406	116.735006	117.883981	83.240586	135.24627	164.547125	125.544514
Pitpnm1	160.089959	0.19474094	0.38121133	0.51084772	0.60945769	0.99938111		Pitpnm1	91.3983007	87.7896015	97.6502636	304.016582	291.342051	102.316744	77.943375	228.262753
Nelfe	15.1974206	0.20139225	0.39427339	0.51079341	0.60949573	0.99938111		Nelfe	8.73660227	8.14542694	8.58813393	31.0221002	13.3184938	7.05632714	17.32075	27.3915304
Stxbp5l	126.882315	0.17779482	0.3480933	0.51076771	0.60951372	0.99938111		Stxbp5l	133.737219	124.896546	158.08528	49.6353602	99.8887032	226.978523	151.07543	70.7614535
R3hdm1	164.729765	-0.1373611	0.26899909	-0.5106377	0.6096048	0.99938111		R3hdm1	153.898609	231.23962	196.254764	111.679561	139.844185	191.696887	172.245236	120.979259
H2-L	2.90529397	0.20593331	0.4034364	0.51044801	0.60973763	NA		H2-L	0.67204633	2.26261859	0.9542371	6.20442003	6.65924688	0	1.92452778	4.56525506
Arap3	2.03304216	0.20403253	0.39971836	0.51044073	0.60974273	NA		Arap3	1.34409266	1.35757116	1.59039517	2.06814001	6.65924688	0	0.96226389	2.28262753
Pogk	50.7322886	0.14209962	0.27839288	0.51042837	0.60975139	0.99938111		Pogk	43.010965	42.9897533	54.7095939	51.7035003	29.966611	63.5069443	60.622625	59.3483158
Faf1	24.6651721	0.18052125	0.35379299	0.51024542	0.60987953	0.99938111		Faf1	21.5054825	21.7211385	27.672876	20.6814001	8.3240586	36.4576902	40.4150833	20.5436478
Elovl4	38.2960986	-0.1689962	0.33128574	-0.510122	0.60996598	0.99938111		Elovl4	40.994826	55.2078937	41.3502745	26.8858201	23.3073641	61.1548353	34.6415	22.8262753
4930503E24Rik	15.2044474	0.18646275	0.36564847	0.50995087	0.61008588	0.99938111		4930503E24Ri	18.1452509	14.9332827	12.7231614	8.27256004	4.99443516	18.8168724	16.3584861	27.3915304
Atp1a3	2601.75458	0.1867785	0.36629421	0.50991386	0.61011181	0.99938111		Atp1a3	1635.76076	2502.00364	1870.94088	3486.88406	3381.2326	2086.32073	831.396	5019.49794
Fam81a	101.420758	0.19574371	0.38398381	0.50977075	0.61021208	0.99938111		Fam81a	123.656524	107.700645	103.693765	31.0221002	64.9276571	144.654706	199.188625	36.5220405
Tceb3	27.3122605	0.15698248	0.30801607	0.50965677	0.61029194	0.99938111		Tceb3	28.8979921	29.8665654	21.9474534	22.7495401	31.6314227	34.1055812	35.6037639	13.6957652
Mamstr	2.81599046	-0.2113951	0.41484996	-0.5095699	0.61035281	NA		Mamstr	3.36023164	1.81009488	2.22655324	6.20442003	3.32962344	2.35210905	0.96226389	2.28262753

GeneID	Base mean	log2(FC)	StdErr	Wald-Stats	P-value	P-adj		GeneID	Normalised expression for Chow#1	Normalised expression for Chow#2	Normalised expression for Chow#3	Normalised expression for Chow#4	Normalised expression for HFD#1	Normalised expression for HFD#2	Normalised expression for HFD#3	Normalised expression for HFD#4
Sncg	3.39135393	-0.2088883	0.4099636	-0.5095289	0.61038152	NA		Sncg	3.36023164	1.35757116	1.90847421	10.3407001	4.99443516	0	2.88679167	2.28262753
Mrpl13	14.6333391	-0.211994	0.41613787	-0.509432	0.61044942	0.99938111		Mrpl13	21.5054825	23.9837571	20.0389792	2.06814001	0	14.1126543	30.7924444	4.56525506
Cbx1	10.2676245	0.19070605	0.37444289	0.50930609	0.61053769	0.99938111		Cbx1	11.4247876	5.88280835	6.99773876	14.4769801	14.9833055	10.5844907	8.660375	9.13051012
Lrrc8c	11.3232955	-0.2054586	0.40350882	-0.50918	0.61062605	0.99938111		Lrrc8c	7.39250961	20.3635674	19.4028211	2.06814001	14.9833055	14.1126543	7.69811111	4.56525506
Pcyt2	24.1874695	0.16969558	0.33333433	0.5090852	0.6106925	0.99938111		Pcyt2	23.5216215	14.9332827	16.2220308	37.2265202	23.3073641	19.9929269	24.0565972	34.239413
Zfp119b	1.40262411	-0.196938	0.38686738	-0.5090582	0.61071145	NA		Zfp119b	3.36023164	1.35757116	2.22655324	0	0	2.35210905	1.92452778	0
Serhl	11.9272805	-0.1799215	0.35361465	-0.5088066	0.61088778	0.99938111		Serhl	12.0968339	14.480759	12.7231614	12.4088401	4.99443516	10.5844907	14.4339583	13.6957652
Dazl	1.24639393	-0.1914844	0.37654328	-0.5085323	0.61108011	NA		Dazl	2.68818531	1.35757116	1.90847421	0	1.66481172	2.35210905	0	0
Uchl3	4.41057964	0.20846932	0.40996274	0.50850796	0.61109716	NA		Uchl3	4.03227797	4.07271347	5.08926455	2.06814001	1.66481172	7.05632714	6.73584722	4.56525506
Spryd7	30.6724168	0.19214824	0.37793916	0.50841052	0.61116548	0.99938111		Spryd7	42.3389187	28.5089943	33.3982986	6.20442003	19.9777406	37.6337448	56.7735694	20.5436478
A730085K08Rik	0.26433693	-0.1252009	0.24628232	-0.5083632	0.61119864	NA		A730085K08Ri	1.34409266	0.45252372	0.31807903	0	0	0	0	0
Col3a1	0.26433693	-0.1252009	0.24628232	-0.5083632	0.61119864	NA		Col3a1	1.34409266	0.45252372	0.31807903	0	0	0	0	0
Gadl1	0.26433693	-0.1252009	0.24628232	-0.5083632	0.61119864	NA		Gadl1	1.34409266	0.45252372	0.31807903	0	0	0	0	0
Il20ra	0.26433693	-0.1252009	0.24628232	-0.5083632	0.61119864	NA		Il20ra	1.34409266	0.45252372	0.31807903	0	0	0	0	0
Irf4	0.26433693	-0.1252009	0.24628232	-0.5083632	0.61119864	NA		Irf4	1.34409266	0.45252372	0.31807903	0	0	0	0	0
Med9os	0.26433693	-0.1252009	0.24628232	-0.5083632	0.61119864	NA		Med9os	1.34409266	0.45252372	0.31807903	0	0	0	0	0
Qprt	0.26433693	-0.1252009	0.24628232	-0.5083632	0.61119864	NA		Qprt	1.34409266	0.45252372	0.31807903	0	0	0	0	0
Slc44a5	8.32447596	0.20110651	0.39589245	0.50798269	0.61146548	0.99938111		Slc44a5	3.36023164	9.95552182	7.31581779	10.3407001	3.32962344	12.9365998	12.5094306	6.84788259
Gpbp1l1	24.9441181	-0.1633217	0.32151814	-0.5079705	0.61147402	0.99938111		Gpbp1l1	22.1775288	23.0787097	24.8101647	39.2946602	21.6425524	15.2887088	32.7169722	20.5436478
Slc2a5	0.97939934	0.17933649	0.35306024	0.50794871	0.61148932	NA		Slc2a5	0.67204633	1.35757116	0.63615807	0	0	0	2.88679167	2.28262753
Tmem62	16.6199268	-0.181614	0.35762796	-0.5078294	0.61157296	0.99938111		Tmem62	12.7688802	22.6261859	20.3570582	16.5451201	6.65924688	25.8731995	14.4339583	13.6957652
Prpf4b	28.8067966	0.18555257	0.36550316	0.50766339	0.61168943	0.99938111		Prpf4b	37.6345944	31.2241366	25.7644018	10.3407001	18.3129289	51.7463991	39.4528194	15.9783927
Src	101.222588	0.1830456	0.36058989	0.5076282	0.61171411	0.99938111		Src	69.8928182	80.0966983	70.2954666	150.974221	178.134854	56.4506172	50.9999861	152.936045
Ctxn3	1.19546878	0.18225398	0.35903799	0.50761755	0.61172158	NA		Ctxn3	0	0.45252372	1.59039517	2.06814001	0	3.52816357	1.92452778	0
Slc25a3	169.940272	0.1236351	0.24358476	0.507565	0.61175844	0.99938111		Slc25a3	131.049034	132.136926	169.536125	217.154701	198.112595	138.774434	174.169764	198.588595
Pfdn1	39.3393316	-0.1815962	0.35781077	-0.5075202	0.61178986	0.99938111		Pfdn1	44.3550577	23.0787097	27.0367179	78.5893204	24.9721758	23.5210905	45.2264028	47.9351782
Evl	98.0926955	-0.1374187	0.27076893	-0.5075128	0.61179507	0.99938111		Evl	114.247876	85.9795066	90.9706039	122.020261	64.9276571	98.78858	73.1320555	134.675024
Dhx34	13.2150872	0.20467668	0.40330089	0.50750366	0.61180147	0.99938111		Dhx34	9.4086486	6.33533207	9.224292	22.7495401	28.3017992	4.7042181	6.73584722	18.2610202
Atp5k	5.06593719	0.20815491	0.41020817	0.50743727	0.61184804	0.99938111		Atp5k	6.04841696	4.52523719	4.77118552	2.06814001	3.32962344	2.35210905	10.5849028	6.84788259
Ubqln2	248.838374	0.10324194	0.20347792	0.50738645	0.61188369	0.99938111		Ubqln2	231.183937	231.23962	250.3282	244.040521	194.782971	283.42914	252.113139	303.589462
Nus1	69.2936548	0.17524721	0.34557589	0.50711643	0.61207313	0.99938111		Nus1	68.5487255	94.5774573	66.4785182	24.8176801	48.2795399	112.901234	88.5282777	50.2178057
Mycl	16.6869715	0.1744309	0.34414454	0.50685362	0.61225753	0.99938111		Mycl	15.4570656	16.2908539	12.7231614	16.5451201	16.6481172	8.23238167	20.2075417	27.3915304
Abi2	301.434041	0.11938295	0.23555924	0.50680649	0.6122906	0.99938111		Abi2	291.668107	331.247362	302.811241	225.427261	274.693934	377.513502	375.282917	232.828008
Wls	14.4972761	-0.2006126	0.39604216	-0.5065436	0.61247507	0.99938111		Wls	13.4409266	28.961518	15.2677937	6.20442003	6.65924688	27.0492541	11.5471667	6.84788259
Ercc3	25.4561417	-0.1519629	0.30001761	-0.5065133	0.61249632	0.99938111		Ercc3	32.2582238	22.6261859	24.1740066	31.0221002	28.3017992	22.345036	26.9433889	15.9783927
Gstz1	17.7943435	-0.1681109	0.33195892	-0.5064208	0.61256128	0.99938111		Gstz1	22.8495752	14.9332827	20.6751372	18.6132601	19.9777406	11.7605452	22.1320694	11.4131377
C1qtnf2	0.30858284	-0.1092518	0.21578022	-0.5063104	0.61263879	NA		C1qtnf2	2.01613899	0.45252372	0	0	0	0	0	0
Ccm2l	0.30858284	-0.1092518	0.21578022	-0.5063104	0.61263879	NA		Ccm2l	2.01613899	0.45252372	0	0	0	0	0	0
Slc31a1	9.08164984	-0.2072424	0.40934265	-0.506281	0.61265937	0.99938111		Slc31a1	6.72046328	21.2686148	10.1785291	2.06814001	8.3240586	14.1126543	7.69811111	2.28262753
Zfp954	7.68884731	0.19532466	0.38581963	0.506259	0.61267484	0.99938111		Zfp954	6.04841696	7.69290322	8.2700549	6.20442003	4.99443516	14.1126543	9.62263889	4.56525506
Rfwd3	10.7121894	0.1833268	0.36214616	0.50622324	0.61269994	0.99938111		Rfwd3	8.73660227	13.5757116	10.1785291	6.20442003	13.3184938	15.2887088	11.5471667	6.84788259
Renbp	1.2424816	0.18161311	0.35887055	0.50606859	0.6128085	NA		Renbp	0.67204633	2.26261859	0.63615807	0	1.66481172	4.7042181	0	0
Gabpb1	10.2026565	0.18663669	0.36882397	0.50603189	0.61283426	0.99938111		Gabpb1	8.06455594	10.4080455	10.8146872	8.27256004	4.99443516	16.4647633	13.4716944	9.13051012
Vat1	132.081035	-0.2094808	0.41399943	-0.505993	0.61286157	0.99938111		Vat1	63.1723549	51.587704	49.6203294	434.309402	143.173808	51.7463991	46.1886666	216.849615
Mettl6	6.40907783	-0.2062104	0.40758586	-0.5059312	0.61290496	0.99938111		Mettl6	12.7688802	5.43028463	7.95197586	2.06814001	1.66481172	5.88027262	8.660375	6.84788259
Gpx7	1.51946739	-0.1990993	0.39365126	-0.5057759	0.61301396	NA		Gpx7	2.01613899	1.35757116	1.59039517	2.06814001	1.66481172	1.17605452	0	2.28262753
Stat4	0.79514699	0.16475068	0.32584987	0.50560302	0.61313536	NA		Stat4	1.34409266	0	0.9542371	0	0	1.17605452	2.88679167	0
Slc25a15	5.45431757	-0.2075077	0.41064131	-0.5053258	0.61333	0.99938111		Slc25a15	8.73660227	5.43028463	8.2700549	2.06814001	1.66481172	9.40843619	5.77358333	2.28262753
Med21	11.0714477	-0.1955093	0.38715209	-0.5049936	0.61356331	0.99938111		Med21	10.7527413	16.7433776	15.2677937	6.20442003	4.99443516	11.7605452	18.2830139	4.56525506
Pkn1	16.1868608	0.20526906	0.4064941	0.50497428	0.6135769	0.99938111		Pkn1	12.7688802	8.59795066	9.224292	26.8858201	23.3073641	3.52816357	8.660375	36.5220405
Gabrb3	224.751375	-0.1887086	0.37370685	-0.5049642	0.613584	0.99938111		Gabrb3	209.006408	371.06945	306.946268	93.0663005	119.866444	383.393775	216.509375	98.1529838
Grasp	21.4251434	-0.197175	0.39055936	-0.504853	0.61366211	0.99938111		Grasp	38.3066407	22.6261859	15.2677937	16.5451201	21.6425524	3.52816357	19.2452778	34.239413
Synpo2	3.43596112	-0.2099531	0.416079	-0.5045992	0.61384035	NA		Synpo2	2.68818531	5.88280835	3.18079035	4.13628002	3.32962344	1.17605452	4.81131944	2.28262753
0610030E20Rik	7.1249198	-0.2066055	0.4095029	-0.5045276	0.61389064	0.99938111		0610030E20Ri	4.7043243	8.59795066	5.08926455	14.4769801	11.653682	1.17605452	6.73584722	4.56525506
Zfp623	9.49104911	0.19645403	0.38944831	0.50444185	0.61395091	0.99938111		Zfp623	4.03227797	8.59795066	6.67965972	16.5451201	4.99443516	11.7605452	9.62263889	13.6957652
Edn1	0.8124259	0.16301129	0.3231699	0.5044136	0.61397077	NA		Edn1	0	1.35757116	0.63615807	0	3.32962344	1.17605452	0	0
Fn1	12.1672679	-0.2092209	0.4147804	-0.5044135	0.6139708	0.99938111		Fn1	13.4409266	27.6039469	12.0870033	2.06814001	33.2962344	2.35210905	1.92452778	4.56525506
2010012O05Rik	22.4375166	0.15811096	0.31347921	0.50437464	0.61399813	0.99938111		2010012O05R	18.8172972	23.5312334	21.9474534	20.6814001	18.3129289	22.345036	35.6037639	18.2610202
Tmf1	41.8480477	0.19083821	0.37845172	0.50426037	0.61407842	0.99938111		Tmf1	51.7475673	41.6321821	43.5768277	14.4769801	16.6481172	83.4998712	55.8113055	27.3915304
Hrasls	19.7192298	-0.1671628	0.33161475	-0.5040874	0.61419994	0.99938111		Hrasls	20.8334362	17.1959013	21.3112953	26.8858201	8.3240586	17.6408179	25.0188611	20.5436478
Tacr1	19.0098866	0.19246829	0.38182459	0.50407516	0.61420856	0.99938111		Tacr1	14.1129729	25.3413283	22.9016905	4.13628002	28.3017992	29.4013631	9.62263889	18.2610202
Cntnap5b	15.9591344	-0.2030162	0.40284177	-0.5039602	0.61428932	0.99938111		Cntnap5b	14.1129729	28.0564706	25.4463228	2.06814001	6.65924688	27.0492541	10.5849028	13.6957652
Ubl3	147.150374	-0.1067951	0.21193541	-0.5039039	0.61432893	0.99938111		Ubl3	153.898609	154.763112	156.494885	146.837941	143.173808	110.549125	172.245236	139.240279
Zfp937	5.86552586	0.20234865	0.40168342	0.50375157	0.61443597	0.99938111		Zfp937	3.36023164	5.88280835	4.45310648	8.27256004	4.99443516	10.5844907	4.81131944	4.56525506
Gtf2e2	8.26501441	0.20644152	0.40989804	0.50364114	0.61451358	0.99938111		Gtf2e2	6.04841696	8.14542694	7.95197586	8.27256004	1.66481172	10.5844907	21.1698055	2.28262753
Nat8l	299.302288	0.18603917	0.36946102	0.50354208	0.6145832	0.99938111		Nat8l	178.764323	204.088197	203.570582	506.694303	511.097198	216.394032	124.132042	449.677624
Zfp951	0.81738479	0.1641125	0.32596923	0.50346008	0.61464084	NA		Zfp951	0.67204633	0	1.59039517	0	0	2.35210905	1.92452778	0
Dctpp1	2.54907484	-0.2081015	0.41340782	-0.5033807	0.61469663	NA		Dctpp1	4.7043243	2.26261859	4.45310648	0	1.66481172	1.17605452	3.84905555	2.28262753
Stk35	32.8003273	-0.1765753	0.35079896	-0.5033518	0.61471697	0.99938111		Stk35	22.1775288	33.4867552	26.7186389	59.9760603	38.2906696	15.2887088	23.0943333	43.3699231
Zfp934	2.01772682	0.20013294	0.39762915	0.50331557	0.61474242	NA		Zfp934	3.36023164	1.35757116	1.90847421	0	0	4.7042181	4.81131944	0
Tmem161b	10.8867584	0.194407	0.38640896	0.50311205	0.6148855	0.99938111		Tmem161b	9.4086486	14.480759	11.1327662	4.13628002	11.653682	17.6408179	16.3584861	2.28262753
Mogat1	0.63400116	0.15107893	0.30040085	0.50292444	0.6150174	NA		Mogat1	0.67204633	0.45252372	0	0	1.66481172	0	0	2.28262753
Gpr64	1.01673537	-0.1727771	0.3437752	-0.5025875	0.61525433	NA		Gpr64	0	0.90504744	0.9542371	4.13628002	0	1.17605452	0.96226389	0
Gramd4	22.949577	0.15088584	0.30028159	0.50248115	0.61532911	0.99938111		Gramd4	22.1775288	22.6261859	18.766663	22.7495401	23.3073641	30.5774176	18.2830139	25.1089028
Slc2a3	96.0032236	0.13153598	0.26181795	0.50239482	0.61538982	0.99938111		Slc2a3	73.2530498	109.058216	97.6502636	82.7256004	129.855314	115.253343	71.2075278	89.0224737
Rdh14	14.8845987	0.18557606	0.36960422	0.50209398	0.61560141	0.99938111		Rdh14	9.4086486	17.1959013	16.8581888	10.3407001	4.99443516	24.697145	17.32075	18.2610202
Rgs4	138.489247	-0.1443903	0.28769932	-0.5018792	0.61575248	0.99938111		Rgs4	200.269806	133.494497	159.357596	93.0663005	108.212762	159.943415	164.547125	89.0224737
Gm13629	1.90322766	0.19478143	0.38815732	0.50181053	0.61580081	NA		Gm13629	2.68818531	0.90504744	2.54463228	0	0	2.35210905	6.73584722	0
Lrfn4	24.3901715	-0.1981448	0.39510267	-0.501502	0.61601786	0.99938111		Lrfn4	14.7850192	32.129184	19.0847421	41.3628002	41.620293	5.88027262	10.5849028	29.6741579
Lhfpl4	189.660546	0.2008376	0.40047622	0.50149694	0.61602143	0.99938111		Lhfpl4	75.2691888	124.444023	90.9706039	386.742182	279.688369	122.309671	63.5094166	374.350915
Kdm5c	65.4599831	0.13489572	0.26916322	0.501167	0.6162536	0.99938111		Kdm5c	56.4518916	52.4927514	58.8446214	80.6574604	86.5702095	56.4506172	50.0377222	82.1745911
Fam131a	157.708094	0.19023852	0.37965457	0.50108317	0.61631259	0.99938111		Fam131a	136.425405	102.722884	99.5587378	233.699821	298.001298	56.4506172	83.7169583	251.089028
Prrc2c	223.391822	0.14701272	0.29345081	0.50097908	0.61638584	0.99938111		Prrc2c	213.038686	252.508235	219.156455	155.110501	221.419959	388.097993	178.018819	159.783927
Xab2	22.2323765	0.16793701	0.33524349	0.50094041	0.61641306	0.99938111		Xab2	21.5054825	16.2908539	17.4943469	28.9539601	38.2906696	15.2887088	24.0565972	15.9783927
Rps6kb1	34.6990271	0.16997792	0.33936003	0.50087784	0.6164571	0.99938111		Rps6kb1	37.6345944	39.3695635	31.4898244	20.6814001	28.3017992	43.5140174	60.622625	15.9783927
Ccdc64	8.50126802	0.19433206	0.38803923	0.5008052	0.61650823	0.99938111		Ccdc64	6.04841696	8.14542694	5.72542262	10.3407001	6.65924688	7.05632714	5.77358333	18.2610202
Kcnip2	81.0151228	0.15256914	0.3046718	0.50076555	0.61653613	0.99938111		Kcnip2	85.3498837	83.2643643	74.7485731	57.9079203	93.2294563	38.8097993	102.962236	111.848749
Tap1	0.58556932	-0.1567606	0.31307424	-0.5007138	0.61657253	NA		Tap1	1.34409266	1.35757116	0.31807903	0	1.66481172	0	0	0
9030617O03Rik	6.19536561	0.20071207	0.40085415	0.50071097	0.61657455	0.99938111		9030617O03R	4.7043243	6.33533207	6.36158069	4.13628002	9.98887032	10.5844907	2.88679167	4.56525506
Dhx35	8.3371782	-0.192055	0.38377079	-0.500442	0.61676388	0.99938111		Dhx35	12.0968339	6.33533207	8.90621297	10.3407001	4.99443516	11.7605452	7.69811111	4.56525506
Syt1	938.635494	0.19541649	0.39051037	0.50041307	0.61678425	0.99938111		Syt1	834.68154	1196.02019	1038.84613	308.152862	501.108328	2365.04565	842.943166	422.286093
Trim3	80.6064746	0.14283791	0.28544765	0.50039966	0.61679369	0.99938111		Trim3	79.3014668	60.6381783	58.5265423	107.543281	101.553515	54.0985081	82.7546944	100.435611
Gapdhs	7.161476	-0.1990496	0.39779844	-0.500378	0.61680896	0.99938111		Gapdhs	10.0806949	5.88280835	5.40734359	10.3407001	8.3240586	2.35210905	5.77358333	9.13051012
Rps18	3.31232291	0.20721819	0.41414685	0.50034954	0.61682898	NA		Rps18	7.39250961	1.81009488	1.90847421	0	1.66481172	4.7042181	6.73584722	2.28262753
2610035D17Rik	15.688746	-0.1805039	0.36081935	-0.5002612	0.61689119	0.99938111		2610035D17Ri	12.7688802	18.5534725	16.2220308	18.6132601	18.3129289	8.23238167	7.69811111	25.1089028
Gas6	38.2596659	-0.154954	0.30975316	-0.5002501	0.61689901	0.99938111		Gas6	38.3066407	42.9897533	36.897168	45.4990802	31.6314227	57.6266717	21.1698055	31.9567854
Mb21d2	29.450565	0.17270678	0.34527979	0.50019372	0.61693868	0.99938111		Mb21d2	18.8172972	16.2908539	26.0824808	49.6353602	33.2962344	21.1689814	26.9433889	43.3699231
Arrdc1	7.15810831	-0.2079142	0.41574376	-0.5001019	0.61700335	0.99938111		Arrdc1	4.03227797	5.43028463	4.77118552	18.6132601	6.65924688	1.17605452	2.88679167	13.6957652
Adamtsl3	0.52447467	-0.1541732	0.30830612	-0.5000653	0.61702909	NA		Adamtsl3	1.34409266	1.35757116	0.31807903	0	0	1.17605452	0	0
Amy1	0.52447467	-0.1541732	0.30830612	-0.5000653	0.61702909	NA		Amy1	1.34409266	1.35757116	0.31807903	0	0	1.17605452	0	0
Scube2	0.52447467	-0.1541732	0.30830612	-0.5000653	0.61702909	NA		Scube2	1.34409266	1.35757116	0.31807903	0	0	1.17605452	0	0
Zfp287	9.75214152	0.20791436	0.41587891	0.49993964	0.61711758	0.99938111		Zfp287	9.4086486	14.0282353	9.86045007	0	1.66481172	21.1689814	17.32075	4.56525506
Pkd1	62.0896768	0.19170904	0.38350492	0.49988679	0.61715479	0.99938111		Pkd1	33.6023164	44.3473245	44.5310648	103.407001	134.849749	41.1619083	24.0565972	70.7614535
Col22a1	0.6076638	0.16615135	0.3324645	0.49975667	0.61724642	NA		Col22a1	0.67204633	0.45252372	0.63615807	0	0	1.17605452	1.92452778	0
Utp11l	4.61311935	0.20593866	0.41215175	0.49966709	0.61730951	NA		Utp11l	5.37637063	6.33533207	2.54463228	2.06814001	3.32962344	8.23238167	6.73584722	2.28262753
Nmt1	57.2220234	0.12016387	0.24051182	0.49961734	0.61734455	0.99938111		Nmt1	61.1562159	50.2301328	59.4807795	45.4990802	66.5924688	63.5069443	51.96225	59.3483158
Ppan	5.2632288	-0.202496	0.40541429	-0.4994793	0.61744179	0.99938111		Ppan	5.37637063	4.52523719	4.45310648	10.3407001	3.32962344	4.7042181	4.81131944	4.56525506
Mrps31	10.1467211	0.18280925	0.36603379	0.4994327	0.61747459	0.99938111		Mrps31	8.73660227	10.8605693	9.86045007	8.27256004	6.65924688	16.4647633	13.4716944	6.84788259
Dzip1	29.6629053	0.15798623	0.31639388	0.49933402	0.61754409	0.99938111		Dzip1	24.8657142	24.4362808	27.354797	37.2265202	24.9721758	49.39429	30.7924444	18.2610202
Fbn2	1.34481228	0.18718345	0.37490014	0.49928881	0.61757594	NA		Fbn2	1.34409266	0.90504744	1.59039517	0	4.99443516	0	1.92452778	0
Ndufa2	31.4645929	0.17189629	0.34434198	0.49920224	0.61763692	0.99938111		Ndufa2	26.2098068	18.1009488	23.5378486	51.7035003	19.9777406	25.8731995	45.2264028	41.0872956
Rps14	10.4407252	0.19250304	0.38565009	0.49916503	0.61766313	0.99938111		Rps14	12.7688802	5.43028463	8.58813393	12.4088401	13.3184938	5.88027262	18.2830139	6.84788259

GeneID	Base mean	log2(FC)	StdErr	Wald-Stats	P-value	P-adj	GeneID	Normalised expression for Chow#1	Normalised expression for Chow#2	Normalised expression for Chow#3	Normalised expression for Chow#4	Normalised expression for HFD#1	Normalised expression for HFD#2	Normalised expression for HFD#3	Normalised expression for HFD#4
H2-Eb1	0.39460272	-0.1316318	0.2637534	-0.4990713	0.61772916	NA	H2-Eb1	0	0.45252372	0.63615807	2.06814001	0	0	0	0
Hgf	1.30641541	-0.1812568	0.36330828	-0.4989063	0.61784543	NA	Hgf	0	3.16766603	2.86271131	0	0	1.17605452	0.96226389	2.28262753
Clec11a	3.63665816	0.20771774	0.41634678	0.49890561	0.61784588	NA	Clec11a	3.36023164	0.90504744	2.86271131	6.20442003	4.99443516	2.35210905	3.84905555	4.56525506
Tmem214	22.9444751	-0.1841961	0.36940586	-0.498628	0.61804147	0.99938111	Tmem214	12.0968339	34.3918026	26.4005599	24.8176801	28.3017992	11.7605452	11.5471667	34.239413
Inpp5e	32.3896836	0.13824457	0.27738642	0.49838263	0.61821438	0.99938111	Inpp5e	32.2582238	23.9837571	31.8079035	35.1583802	41.620293	30.5774176	31.7547083	31.9567854
Myo18b	1.33968689	-0.1620067	0.325088	-0.4983471	0.61823943	NA	Myo18b	0.67204633	0	0.9542371	6.20442003	0	0	2.88679167	0
Mcee	10.5409014	-0.1810567	0.36351645	-0.4980701	0.61843461	0.99938111	Mcee	8.73660227	13.1231878	10.4966081	14.4769801	6.65924688	8.23238167	13.4716944	9.13051012
Notch4	2.20175662	-0.1951539	0.39186825	-0.4980089	0.61847775	NA	Notch4	0.67204633	3.62018975	1.90847421	4.13628002	4.99443516	0	0	2.28262753
Smpdl3b	1.83627094	-0.2008193	0.40325401	-0.4979969	0.61848621	NA	Smpdl3b	2.68818531	3.62018975	2.22655324	0	1.66481172	3.52816357	0.96226389	0
Hps3	7.46739178	0.20088928	0.40340568	0.49798327	0.61849583	0.99938111	Hps3	7.39250961	2.26261859	7.95197586	10.3407001	11.653682	8.23238167	9.62263889	2.28262753
Rhcg	0.79596365	-0.172222	0.34592033	-0.4978661	0.61857842	NA	Rhcg	2.68818531	0.90504744	0.63615807	0	0	1.17605452	0.96226389	0
Rgsl1	0.60656084	0.13704222	0.27528771	0.49781453	0.61861478	NA	Rgsl1	0	0.90504744	0	0	1.66481172	0	0	2.28262753
Etnk2	6.05417913	-0.1951173	0.39196565	-0.4977918	0.61863078	0.99938111	Etnk2	4.7043243	0.45252372	0.63615807	24.8176801	0	9.40843619	3.84905555	4.56525506
Cops8	48.7439717	-0.1458102	0.29295373	-0.4977244	0.61867828	0.99938111	Cops8	55.1077989	52.9452751	55.663831	43.4309402	26.6369875	43.5140174	69.283	43.3699231
Il1rapl2	5.75197974	0.20493511	0.41177318	0.49768931	0.61870304	0.99938111	Il1rapl2	6.04841696	5.88280835	6.99773876	0	0	19.9929269	4.81131944	2.28262753
C230091D08Rik	42.7157736	0.15244859	0.30637044	0.49759562	0.61876909	0.99938111	C230091D08Ri	36.2905017	32.129184	41.6683535	53.7716403	28.3017992	54.0985081	63.5094166	31.9567854
Peo1	8.90177363	0.18736625	0.37657311	0.4975561	0.61879695	0.99938111	Peo1	10.0806949	7.2403795	9.224292	6.20442003	4.99443516	14.1126543	12.5094306	6.84788259
Hsd17b4	25.5903252	0.16484501	0.33134715	0.49749942	0.61883691	0.99938111	Hsd17b4	23.5216215	22.1736622	20.3570582	28.9539601	46.6147282	16.4647633	19.2452778	27.3915304
Tm2d1	4.884754	0.20550106	0.41316189	0.49738628	0.61891668	0.99938111	Tm2d1	2.01613899	4.97776091	1.27231614	8.27256004	4.99443516	0	3.84905555	13.6957652
Phyhd1	2.01029673	0.19735596	0.39691486	0.49722492	0.61903045	NA	Phyhd1	1.34409266	3.62018975	0.9542371	0	4.99443516	0	2.88679167	2.28262753
Cox7a2	37.320226	-0.1635082	0.32885346	-0.4972069	0.61904314	0.99938111	Cox7a2	36.9625481	34.8443264	47.7118552	41.3628002	14.9833055	29.4013631	56.7735694	36.5220405
Bclaf1	108.750184	0.19211973	0.38640014	0.49720409	0.61904513	0.99938111	Bclaf1	135.753358	101.365313	118.64348	37.2265202	48.2795399	185.816615	197.264097	45.6525506
Rundc3a	150.712038	0.1180433	0.23750999	0.4970035	0.61918658	0.99938111	Rundc3a	173.387953	118.561214	120.233875	165.451201	193.11816	129.365998	154.924486	150.653417
Rcor3	11.9140257	-0.1847409	0.37186295	-0.4967985	0.61933117	0.99938111	Rcor3	9.4086486	11.7656167	12.7231614	20.6814001	6.65924688	14.1126543	15.3962222	4.56525506
9030624G23Rik	1.03575594	0.17366991	0.34958184	0.49679329	0.61933483	NA	9030624G23R	0.67204633	0	0.63615807	2.06814001	1.66481172	0	0.96226389	2.28262753
Wisp1	1.0668878	0.17223312	0.34674931	0.49670791	0.61939505	NA	Wisp1	0	1.81009488	1.27231614	0	0	3.52816357	1.92452778	0
Nrg4	2.36832161	0.19510998	0.39281175	0.49670098	0.61939993	NA	Nrg4	0	1.35757116	4.13502745	2.06814001	3.32962344	0	5.77358333	2.28262753
Bnip3l	83.1916807	-0.171576	0.34546042	-0.496659	0.61942957	0.99938111	Bnip3l	94.7585323	97.7451233	116.098848	49.6353602	33.2962344	87.0280348	129.905625	57.0656883
Cstb	12.529574	-0.1824655	0.36743466	-0.4965932	0.61947598	0.99938111	Cstb	20.8334362	10.8605693	13.9954775	8.27256004	4.99443516	14.1126543	13.4716944	13.6957652
Ptch2	0.60005996	0.12365958	0.24904267	0.49653974	0.61951365	NA	Ptch2	0	0	1.27231614	0	0	3.52816357	0	0
Cox11	9.58089052	0.19708428	0.39692379	0.49652927	0.61952104	0.99938111	Cox11	6.04841696	5.43028463	10.4966081	12.4088401	3.32962344	5.88027262	12.5094306	20.5436478
Knop1	11.6870849	-0.1825041	0.36760481	-0.4964681	0.6195642	0.99938111	Knop1	12.7688802	16.7433776	8.2700549	14.4769801	11.653682	10.5844907	14.4339583	4.56525506
Igflr1	0.49572269	0.11804608	0.23782321	0.49636064	0.61963999	NA	Igflr1	0	0	0.63615807	0	3.32962344	0	0	0
Map3k8	0.49572269	0.11804608	0.23782321	0.49636064	0.61963999	NA	Map3k8	0	0	0.63615807	0	3.32962344	0	0	0
Abca17	0.37801942	0.11457036	0.23086536	0.49626484	0.61970757	NA	Abca17	0.67204633	0	0	0	0	2.35210905	0	0
Areg	0.37801942	0.11457036	0.23086536	0.49626484	0.61970757	NA	Areg	0.67204633	0	0	0	0	2.35210905	0	0
Pvrl1	68.2940631	0.1592877	0.32126373	0.49581601	0.62002423	0.99938111	Pvrl1	53.0916599	79.6441745	60.4350166	59.9760603	118.201632	70.5632714	33.6792361	70.7614535
Serpini1	96.7409433	0.20432117	0.41217703	0.49571217	0.6200975	0.99938111	Serpini1	86.02193	134.852068	109.419188	8.27256004	44.9499164	242.267232	111.622611	36.5220405
Krcc1	5.12618489	-0.2053364	0.4143118	-0.4956083	0.6201708	0.99938111	Krcc1	6.04841696	8.14542694	5.72542262	4.13628002	1.66481172	4.7042181	10.5849028	0
Txnrd3	6.57051764	0.20196328	0.40752819	0.49558112	0.62018998	0.99938111	Txnrd3	6.04841696	4.07271347	2.54463228	12.4088401	4.99443516	4.7042181	8.660375	9.13051012
Erich5	0.41755702	-0.1150139	0.23209405	-0.4955485	0.62021297	NA	Erich5	0	0	1.27231614	2.06814001	0	0	0	0
Zfp688	6.74593119	-0.2058938	0.41557367	-0.4954448	0.6202862	0.99938111	Zfp688	3.36023164	5.43028463	2.86271131	20.6814001	6.65924688	2.35210905	5.77358333	6.84788259
Btbd2	100.305672	0.19045145	0.38443872	0.49540133	0.62031686	0.99938111	Btbd2	71.2369108	57.4705123	65.5242811	169.587481	164.81636	41.1619083	50.0377222	182.610202
Utp20	11.8944083	0.17541518	0.35410279	0.49537926	0.62033243	0.99938111	Utp20	10.0806949	11.313093	11.4508452	10.3407001	6.65924688	16.4647633	10.5849028	18.2610202
Psmc4	42.5714786	0.14765692	0.29807912	0.4953615	0.62034496	0.99938111	Psmc4	49.059382	43.8948007	42.6225906	22.7495401	36.6258578	36.4576902	63.5094166	45.6525506
Haus7	2.29844711	-0.2049988	0.41385021	-0.4953453	0.6203564	NA	Haus7	2.68818531	2.71514231	3.18079035	2.06814001	0	3.52816357	1.92452778	2.28262753
Smim7	56.1314161	0.13045744	0.26338146	0.49531749	0.62037603	0.99938111	Smim7	45.027104	53.3977988	56.2999891	57.9079203	51.6091633	41.1619083	68.3207361	75.3267085
Scn1b	194.37973	0.17275251	0.34877168	0.49531689	0.62037645	0.99938111	Scn1b	164.65135	157.478254	126.277377	268.858201	251.38657	144.654706	85.6414861	356.089895
Spata9	1.86398489	0.1983441	0.40049054	0.49525288	0.62042163	NA	Spata9	2.01613899	2.71514231	0.63615807	0	1.66481172	2.35210905	0.96226389	4.56525506
Pde12	16.2963105	0.16797257	0.33917432	0.49523964	0.62043097	0.99938111	Pde12	16.8011582	9.95552182	13.3593194	22.7495401	18.3129289	15.2887088	20.2075417	13.6957652
Zfp619	3.08088029	-0.2036417	0.41120346	-0.4952335	0.62043531	NA	Zfp619	5.37637063	5.88280835	1.59039517	2.06814001	0	5.88027262	3.84905555	0
Rai1	72.1156377	0.1785441	0.36059651	0.49513541	0.62050454	0.99938111	Rai1	57.1239379	61.0907021	49.6203294	97.2025805	148.168243	42.3379629	34.6415	86.7398462
Eif3h	22.72226	-0.1719874	0.34736973	-0.4951134	0.6205201	0.99938111	Eif3h	36.2905017	15.8383302	14.9497146	33.0902402	19.9777406	23.5210905	22.1320694	15.9783927
2210016L21Rik	50.7037617	0.11823731	0.23886079	0.49500508	0.62059654	0.99938111	2210016L21Ri	51.075521	46.1574193	49.9384084	47.5672202	54.9387868	52.9224536	59.6603611	43.3699231
Etfb	7.12515522	0.19764599	0.39940146	0.49485545	0.62070216	0.99938111	Etfb	7.39250961	4.07271347	5.72542262	10.3407001	6.65924688	7.05632714	13.4716944	2.28262753
Bag2	9.20415052	0.19116137	0.38632601	0.4948188	0.62072803	0.99938111	Bag2	8.73660227	9.05047438	8.58813393	8.27256004	6.65924688	11.7605452	18.2830139	2.28262753
4921525O09Rik	0.79657979	0.16227522	0.32820256	0.49443616	0.62099818	NA	4921525O09R	0.67204633	0	0.31807903	2.06814001	0	2.35210905	0.96226389	0
Gpr153	22.3118844	-0.1926572	0.38967013	-0.494411	0.62101595	0.99938111	Gpr153	11.4247876	30.3190892	20.0389792	35.1583802	14.9833055	16.4647633	6.73584722	43.3699231
Rab5c	93.7063698	-0.1392318	0.28163369	-0.4943718	0.6210436	0.99938111	Rab5c	111.559691	82.3593168	74.112415	128.224681	106.54795	56.4506172	80.8301666	109.566121
Tmem255b	1.00505485	-0.1431925	0.28970441	-0.4942709	0.62111487	NA	Tmem255b	0	3.62018975	0	2.06814001	0	2.35210905	0	0
Psd	213.295953	0.16129484	0.32636677	0.49421342	0.62115546	0.99938111	Psd	223.791427	171.959013	159.993754	237.836101	361.264143	115.253343	139.528264	296.741579
4933427D14Rik	9.72405701	0.19986141	0.40469248	0.49385996	0.62140508	0.99938111	4933427D14Ri	6.72046328	12.6706641	13.9954775	0	6.65924688	16.4647633	14.4339583	6.84788259
4930579G18Rik	0.79170729	0.14882824	0.30137866	0.49382477	0.62142994	NA	4930579G18R	0	0	0.31807903	2.06814001	1.66481172	0	0	2.28262753
Eng	6.23485649	-0.1997881	0.40459609	-0.4937965	0.62144993	0.99938111	Eng	2.01613899	17.1959013	7.31581779	2.06814001	16.6481172	2.35210905	0	2.28262753
Lmo7	32.3060262	0.19125157	0.3873497	0.49374395	0.62148702	0.99938111	Lmo7	39.6507334	30.3190892	40.3960374	4.13628002	66.5924688	24.697145	29.8301805	22.8262753
Plce1	13.3119357	-0.1840144	0.37273748	-0.4936837	0.62152956	0.99938111	Plce1	11.4247876	19.0059962	9.86045007	18.6132601	21.6425524	11.7605452	9.62263889	4.56525506
Tbc1d10a	11.3706441	-0.1853658	0.37549855	-0.4936525	0.62155159	0.99938111	Tbc1d10a	11.4247876	7.69290322	9.54237104	22.7495401	13.3184938	9.40843619	7.69811111	9.13051012
Ptcd1	16.0358481	0.1655911	0.3354952	0.49357217	0.62160836	0.99938111	Ptcd1	16.8011582	13.5757116	12.4050823	16.5451201	14.9833055	10.5844907	18.2830139	25.1089028
Dffa	37.6145699	0.1573331	0.31877002	0.49356305	0.6216148	0.99938111	Dffa	32.9302701	23.9837571	34.6706148	49.6353602	36.6258578	22.345036	41.3773472	59.3483158
Arhgef25	50.0190202	0.12656755	0.25645538	0.49352658	0.62164057	0.99938111	Arhgef25	58.4680306	42.0847059	39.1237212	51.7035003	53.2739751	45.8661264	54.8490416	54.7830607
Zfp654	11.6136212	0.20545311	0.41634998	0.49346251	0.62168583	0.99938111	Zfp654	16.1291119	11.313093	12.0870033	0	1.66481172	31.7534722	15.3962222	4.56525506
Zfp346	29.5439355	0.1537615	0.31162	0.49342628	0.62171142	0.99938111	Zfp346	20.1613899	22.6261859	23.8559276	47.5672202	31.6314227	32.9295267	27.9056528	29.6741579
D430020J02Rik	0.83666971	-0.1620421	0.32844573	-0.4933604	0.62175793	NA	D430020J02Ri	1.34409266	0.45252372	2.54463228	0	0	2.35210905	0	0
Zcchc8	12.7721405	-0.1704033	0.34539602	-0.4933563	0.62176086	0.99938111	Zcchc8	14.7850192	12.2181404	16.2220308	10.3407001	13.3184938	12.9365998	8.660375	13.6957652
Mbnl1	107.669692	-0.1520708	0.30832837	-0.4932104	0.62186393	0.99938111	Mbnl1	113.575829	136.662163	127.549693	80.6574604	63.2628454	156.415252	119.320722	63.9135709
Gpc1	43.1513976	0.17154697	0.34782272	0.4932023	0.62186965	0.99938111	Gpc1	39.6507334	50.2301328	37.5333261	28.9539601	73.2517157	28.2253086	21.1698055	66.1961984
Anapc10	5.18098712	0.2015095	0.40857499	0.49320078	0.62187073	0.99938111	Anapc10	4.7043243	3.62018975	7.63389683	2.06814001	4.99443516	9.40843619	6.73584722	2.28262753
Ralgps2	39.2395417	-0.1668456	0.33850726	-0.4928863	0.62209292	0.99938111	Ralgps2	43.010965	56.1129411	37.5333261	33.0902402	18.3129289	55.2745626	50.0377222	20.5436478
Bmp3	11.3458202	-0.1908487	0.38720714	-0.4928853	0.62209361	0.99938111	Bmp3	8.06455594	19.4585199	16.2220308	4.13628002	16.6481172	9.40843619	7.69811111	9.13051012
E130309D02Rik	21.9323319	0.17806197	0.36131418	0.49281757	0.6221415	0.99938111	E130309D02Ri	24.8657142	14.9332827	13.6773985	26.8858201	31.6314227	10.5844907	16.3584861	36.5220405
Tspan6	11.6988191	-0.195624	0.3969686	-0.4927947	0.62215769	0.99938111	Tspan6	13.4409266	19.4585199	12.7231614	6.20442003	1.66481172	18.8168724	14.4339583	6.84788259
Smg9	17.6944564	0.18975957	0.38514313	0.49269884	0.6222254	0.99938111	Smg9	15.4570656	9.5029981	9.54237104	31.0221002	29.966611	9.40843619	11.5471667	25.1089028
Ddx39	3.85824675	-0.2046931	0.41560101	-0.4925232	0.62234952	NA	Ddx39	4.03227797	1.81009488	6.04350166	6.20442003	3.32962344	2.35210905	4.81131944	2.28262753
Ldlrap1	0.78074254	-0.162577	0.33031607	-0.4921863	0.62258765	NA	Ldlrap1	0.67204633	0.90504744	0.31807903	2.06814001	0	0	0	2.28262753
Vat1l	127.116442	-0.1310826	0.26644676	-0.4919656	0.62274365	0.99938111	Vat1l	116.264015	98.197647	118.961559	204.745861	133.184938	119.957561	100.075444	125.544514
Gabre	3.22367226	-0.1888841	0.38395828	-0.491939	0.62276246	NA	Gabre	2.01613899	1.35757116	0.31807903	12.4088401	1.66481172	1.17605452	0	6.84788259
Nedd4	387.628387	0.12253322	0.24918499	0.49173597	0.62290601	0.99938111	Nedd4	375.001851	379.214876	427.498222	295.744021	381.241884	564.506172	399.339514	278.480559
Kcnh6	3.44765578	0.20430783	0.41549162	0.49172552	0.62291339	NA	Kcnh6	3.36023164	2.71514231	2.22655324	4.13628002	6.65924688	2.35210905	3.84905555	2.28262753
Aldh1l2	2.25270366	-0.1958831	0.39839017	-0.4916865	0.622941	NA	Aldh1l2	4.03227797	2.71514231	3.18079035	0	0	3.52816357	0	4.56525506
Gtf2h2	11.8414342	0.17081953	0.34757618	0.49145925	0.62310167	0.99938111	Gtf2h2	10.7527413	11.313093	12.0870033	8.27256004	9.98887032	10.5844907	13.4716944	18.2610202
Tnfaip2	1.1594345	-0.1696614	0.34525453	-0.4914097	0.62313668	NA	Tnfaip2	1.34409266	0	0.9542371	4.13628002	1.66481172	1.17605452	0	0
Slc25a5	81.4609708	0.14161667	0.28838215	0.49107294	0.62337486	0.99938111	Slc25a5	71.2369108	63.3533207	70.2954666	103.407001	76.5813391	50.5703445	95.264125	120.979259
Gmnc	0.45965364	-0.147583	0.30063305	-0.4909073	0.62349202	NA	Gmnc	0.67204633	0.45252372	1.59039517	0	0	0	0.96226389	0
Dpf1	39.4066858	0.18453477	0.37598644	0.49080166	0.62356674	0.99938111	Dpf1	34.2743627	21.7211385	26.0824808	62.0442003	56.6035985	16.4647633	25.0188611	73.044081
Chmp2a	24.0873835	-0.1801305	0.36707008	-0.490725	0.62362094	0.99938111	Chmp2a	35.6184554	28.961518	26.0824808	14.4769801	8.3240586	27.0492541	38.4905555	13.6957652
Hemk1	2.16056404	-0.1992115	0.40599426	-0.4906757	0.62365582	NA	Hemk1	2.68818531	3.16766603	1.90847421	2.06814001	0	0	2.88679167	4.56525506
Gm11762	0.28729122	-0.1068138	0.217692	-0.4906649	0.62366351	NA	Gm11762	1.34409266	0	0.9542371	0	0	0	0	0
Lpo	0.28729122	-0.1068138	0.217692	-0.4906649	0.62366351	NA	Lpo	1.34409266	0	0.9542371	0	0	0	0	0
1700008F21Rik	0.33689132	0.12517479	0.25512038	0.4906499	0.62367409	NA	1700008F21Ri	0	0.45252372	0.31807903	0	0	0	1.92452778	0
A530072M11Rik	0.33689132	0.12517479	0.25512038	0.4906499	0.62367409	NA	A530072M11R	0	0.45252372	0.31807903	0	0	0	1.92452778	0
Cldn26	0.33689132	0.12517479	0.25512038	0.4906499	0.62367409	NA	Cldn26	0	0.45252372	0.31807903	0	0	0	1.92452778	0
Pdia5	0.33689132	0.12517479	0.25512038	0.4906499	0.62367409	NA	Pdia5	0	0.45252372	0.31807903	0	0	0	1.92452778	0
Tgif1	0.33689132	0.12517479	0.25512038	0.4906499	0.62367409	NA	Tgif1	0	0.45252372	0.31807903	0	0	0	1.92452778	0
Nutf2	17.7023483	-0.1796513	0.36616815	-0.4906252	0.62369158	0.99938111	Nutf2	18.1452509	25.3413283	23.2197695	8.27256004	14.9833055	7.05632714	24.0565972	20.5436478
Rbp1	3.16248798	-0.2040417	0.41592412	-0.4905744	0.62372749	NA	Rbp1	2.68818531	5.88280835	4.77118552	0	3.32962344	1.17605452	2.88679167	4.56525506
Actr6	9.06338006	0.19383143	0.39513031	0.49055065	0.6237443	0.99938111	Actr6	6.72046328	11.313093	10.4966081	4.13628002	6.65924688	16.4647633	14.4339583	2.28262753
Wiz	46.434265	-0.1309332	0.26691435	-0.4905439	0.6237491	0.99938111	Wiz	47.7152893	52.4927514	41.9864326	51.7035003	54.9387868	36.4576902	33.6792361	52.5004332
Btbd17	5.77896346	0.20303684	0.41391355	0.49052959	0.6237592	0.99938111	Btbd17	5.37637063	6.78785578	2.86271131	4.13628002	9.98887032	4.7042181	0.96226389	11.4131377
Ppm1m	11.5110658	-0.1991676	0.40605928	-0.490489	0.6237879	0.99938111	Ppm1m	6.72046328	8.14542694	6.99773876	31.0221002	13.3184938	3.52816357	8.660375	13.6957652
Ak8	1.25265117	-0.1886862	0.38475355	-0.4904079	0.6238453	NA	Ak8	2.01613899	1.35757116	2.22655324	0	0	1.17605452	0.96226389	2.28262753
Plekha3	29.8779006	0.16522716	0.3369916	0.49030052	0.62392126	0.99938111	Plekha3	25.5377605	34.3918026	31.1717454	20.6814001	23.3073641	41.1619083	49.0754583	13.6957652
Rhbdl3	23.5833071	-0.1870527	0.38158441	-0.4902	0.6239924	0.99938111	Rhbdl3	15.4570656	20.8160911	16.5401098	51.7035003	36.6258578	14.1126543	10.5849028	22.8262753

GeneID	Base mean	log2(FC)	StdErr	Wald-Stats	P-value	P-adj		GeneID	Normalised expression for Chow#1	Normalised expression for Chow#2	Normalised expression for Chow#3	Normalised expression for Chow#4	Normalised expression for HFD#1	Normalised expression for HFD#2	Normalised expression for HFD#3	Normalised expression for HFD#4
Arl6	20.7600625	0.18210291	0.37151459	0.49016354	0.62401818	0.99938111		Arl6	23.5216215	21.7211385	20.6751372	10.3407001	16.6481172	18.8168724	45.2264028	9.13051012
Thumpd2	1.26697742	0.18729893	0.38214796	0.49012151	0.62404792	NA		Thumpd2	1.34409266	1.81009488	0.63615807	0	0	1.17605452	2.88679167	2.28262753
Ndufs5	11.0523167	0.18258709	0.37257732	0.49006498	0.62408792	0.99938111		Ndufs5	16.8011582	8.59795066	6.99773876	8.27256004	8.3240586	16.4647633	11.5471667	11.4131377
Plcxd2	109.152157	-0.1052884	0.2149145	-0.4899082	0.62419886	0.99938111		Plcxd2	98.7908103	104.985503	129.776246	119.952121	101.553515	115.253343	93.3395972	109.566121
Ctdp1	9.69669041	0.18756222	0.38302379	0.48968819	0.62435456	0.99938111		Ctdp1	10.7527413	8.14542694	4.77118552	12.4088401	16.6481172	7.05632714	8.660375	9.13051012
Kif26a	4.36136564	0.1998759	0.40825445	0.48958659	0.62442647	NA		Kif26a	2.68818531	3.16766603	0.9542371	8.27256004	13.3184938	0	1.92452778	4.56525506
Ranbp2	86.0016358	0.18101579	0.36981845	0.48947203	0.62450755	0.99938111		Ranbp2	106.855366	90.0522201	91.924841	24.8176801	61.5980337	170.527906	94.3018611	47.9351782
4930481A15Rik	12.6057554	-0.2005885	0.41000012	-0.4892401	0.62467172	0.99938111		4930481A15Ri	23.5216215	14.0282353	16.5401098	2.06814001	1.66481172	11.7605452	22.1320694	9.13051012
Aasdhppt	19.9947067	0.18867816	0.3857525	0.48911713	0.62475878	0.99938111		Aasdhppt	24.8657142	22.1736622	19.0847421	6.20442003	8.3240586	29.4013631	38.4905555	11.4131377
E130307A14Rik	5.86634216	0.19917954	0.40723799	0.48909862	0.62477188	0.99938111		E130307A14Ri	6.72046328	3.16766603	4.13502745	8.27256004	4.99443516	3.52816357	11.5471667	4.56525506
Fam26e	1.61064755	0.19297426	0.39458928	0.48905096	0.62480562	NA		Fam26e	2.01613899	2.26261859	0.9542371	0	1.66481172	1.17605452	4.81131944	0
A530054K11Rik	8.91481788	-0.1946035	0.39793088	-0.4890385	0.62481443	0.99938111		A530054K11Ri	11.4247876	9.5029981	15.5858727	2.06814001	3.32962344	12.9365998	9.62263889	6.84788259
Rab18	89.1897813	0.14898709	0.3047126	0.48894298	0.62488207	0.99938111		Rab18	90.7262543	79.1916508	96.0598684	70.3167603	56.6035985	104.668853	152.037694	63.9135709
Nr1h3	0.56876374	-0.1551374	0.31730752	-0.4889181	0.62489971	NA		Nr1h3	1.34409266	0.90504744	0.63615807	0	1.66481172	0	0	0
Lmbr1l	3.69599053	0.20287705	0.41503704	0.48881674	0.62497144	NA		Lmbr1l	3.36023164	3.16766603	2.22655324	4.13628002	6.65924688	3.52816357	1.92452778	4.56525506
Irf6	0.77187082	-0.1591357	0.32566656	-0.4886461	0.62509228	NA		Irf6	0	1.81009488	2.22655324	0	0	1.17605452	0.96226389	0
Nupl1	38.1286685	0.14766836	0.30230825	0.48846948	0.62521734	0.99938111		Nupl1	41.6668724	38.9170398	41.0321955	20.6814001	43.2851047	35.2816357	56.7735694	27.3915304
Gk2	0.28280519	-0.1048636	0.21469682	-0.4884263	0.62524794	NA		Gk2	0.67204633	0	1.59039517	0	0	0	0	0
Rnaset2a	4.79163792	-0.2019251	0.41356172	-0.4882588	0.62536657	0.99938111		Rnaset2a	4.03227797	6.33533207	8.90621297	2.06814001	1.66481172	8.23238167	4.81131944	2.28262753
Galnt9	26.7417292	0.15440585	0.31625042	0.4882392	0.62538042	0.99938111		Galnt9	26.2098068	30.3190892	21.6293743	20.6814001	46.6147282	23.5210905	22.1320694	22.8262753
Dnmbp	7.51618036	-0.1960208	0.40154477	-0.4881668	0.62543168	0.99938111		Dnmbp	10.0806949	6.33533207	4.13502745	14.4769801	9.98887032	7.05632714	5.77358333	2.28262753
1700094J05Rik	0.54618268	0.146545	0.3002077	0.48814539	0.62544686	NA		1700094J05Ri	0.67204633	0.45252372	0	0	0	0	0.96226389	2.28262753
Sf3a1	60.6081629	-0.1147091	0.2349916	-0.4881413	0.62544974	0.99938111		Sf3a1	61.8282622	60.1856546	62.0254117	68.2486203	49.9443516	57.6266717	51.96225	73.044081
Mief1	41.4895938	0.15251796	0.31258754	0.48792077	0.62560596	0.99938111		Mief1	32.9302701	32.129184	33.0802196	57.9079203	49.9443516	34.1055812	27.9056528	63.9135709
Cct6a	92.5397894	-0.1544843	0.31670005	-0.4877937	0.62569599	0.99938111		Cct6a	119.624246	95.4825047	104.329923	76.5211804	54.9387868	112.901234	130.867889	45.6525506
Gpr21	1.86194331	0.18534933	0.38002472	0.48772965	0.62574135	NA		Gpr21	0	3.16766603	1.90847421	0	3.32962344	0	1.92452778	4.56525506
Uty	9.41153727	0.19619681	0.40234004	0.48763929	0.62580536	0.99938111		Uty	8.73660227	12.2181404	9.86045007	0	0	22.345036	22.1320694	0
Coq10a	22.7826972	0.1524812	0.31273718	0.48756978	0.62585461	0.99938111		Coq10a	23.5216215	15.3858064	22.9016905	22.7495401	29.966611	18.8168724	19.2452778	29.6741579
Rnf34	45.4601206	-0.1538835	0.31567863	-0.487469	0.62592604	0.99938111		Rnf34	49.059382	54.3028463	55.663831	35.1583802	21.6425524	55.2745626	60.622625	31.9567854
Tbc1d13	69.5927837	0.19550571	0.40116594	0.48734375	0.62601475	0.99938111		Tbc1d13	49.7314283	38.0119924	39.4418003	122.020261	109.877574	22.345036	26.9433889	148.37079
Gm10516	1.92757974	-0.1987286	0.40791694	-0.487179	0.62613149	NA		Gm10516	2.01613899	1.81009488	3.18079035	2.06814001	0	1.17605452	2.88679167	2.28262753
Peg10	156.716405	-0.1767677	0.36293714	-0.4870476	0.62622461	0.99938111		Peg10	72.5810035	148.880304	121.188112	339.174962	106.54795	231.682741	105.849028	127.827142
Oprm1	10.1848815	-0.1949278	0.40035712	-0.4868847	0.62634001	0.99938111		Oprm1	4.7043243	15.3858064	9.86045007	16.5451201	8.3240586	17.6408179	6.73584722	2.28262753
Smyd4	8.56896078	0.18246084	0.37476548	0.48686671	0.62635279	0.99938111		Smyd4	9.4086486	9.95552182	5.72542262	6.20442003	9.98887032	11.7605452	8.660375	6.84788259
Srsf10	25.9724333	0.1490246	0.30613055	0.48680083	0.62639948	0.99938111		Srsf10	24.8657142	31.2241366	24.1740066	16.5451201	21.6425524	37.6337448	28.8679167	22.8262753
Gnl2	14.6692298	-0.1942411	0.39926038	-0.4865024	0.62661097	0.99938111		Gnl2	25.5377605	13.5757116	18.130505	8.27256004	3.32962344	24.697145	19.2452778	4.56525506
Kcnj14	2.70663735	0.19648982	0.40391329	0.48646534	0.62663727	NA		Kcnj14	0.67204633	1.35757116	1.59039517	6.20442003	6.65924688	0	2.88679167	2.28262753
Asap3	1.81655487	-0.1953267	0.4017359	-0.4862068	0.62682055	NA		Asap3	1.34409266	1.35757116	2.22655324	4.13628002	3.32962344	1.17605452	0.96226389	0
Asun	9.34802797	-0.1980367	0.40731023	-0.4862062	0.62682099	0.99938111		Asun	5.37637063	10.4080455	6.99773876	18.6132601	3.32962344	4.7042181	4.81131944	20.5436478
Rfesd	12.211617	0.19976668	0.41091632	0.4861493	0.62686131	0.99938111		Rfesd	12.7688802	12.6706641	12.0870033	6.20442003	4.99443516	25.8731995	23.0943333	0
Hist1h2ae	2.10547452	-0.1962761	0.40376775	-0.4861113	0.62688827	NA		Hist1h2ae	1.34409266	2.26261859	1.90847421	4.13628002	1.66481172	0	0.96226389	4.56525506
Stk38l	10.1234106	0.18999234	0.39110437	0.48578424	0.62712015	0.99938111		Stk38l	10.0806949	9.05047438	15.2677937	0	11.653682	12.9365998	10.5849028	11.4131377
Eif4ebp2	30.8338279	0.16560319	0.34097106	0.48568106	0.62719331	0.99938111		Eif4ebp2	24.8657142	33.9392789	20.3570582	35.1583802	58.2684102	19.9929269	22.1320694	31.9567854
Acap3	94.1880112	0.17416223	0.35869721	0.48554108	0.62729258	0.99938111		Acap3	82.6616984	64.7108918	65.2062021	134.429101	163.151549	44.6900719	54.8490416	143.805534
Kpna6	110.853472	0.13581	0.2797152	0.48552957	0.62730074	0.99938111		Kpna6	92.7423933	78.2866034	93.8333152	157.178641	151.497867	101.140689	86.60375	125.544514
Sigmar1	16.6530357	-0.1916931	0.39485189	-0.485481	0.62733516	0.99938111		Sigmar1	16.1291119	28.0564706	17.8124259	8.27256004	13.3184938	10.5844907	4.81131944	34.239413
Cep83os	23.1948426	0.1705389	0.35128389	0.48547318	0.62734074	0.99938111		Cep83os	18.1452509	18.5534725	19.0847421	33.0902402	19.9777406	16.4647633	44.2641389	15.9783927
Mcfd2	33.1826449	0.14306044	0.2947184	0.48541402	0.62738269	0.99938111		Mcfd2	31.5861774	36.6544212	36.2610099	18.6132601	26.6369875	45.8661264	35.6037639	34.239413
Cmtm4	135.375669	0.12222253	0.25195473	0.48509716	0.62760743	0.99938111		Cmtm4	92.070347	168.338823	125.641219	130.292821	136.514561	177.584233	127.018833	125.544514
F930015N05Rik	0.64242092	-0.157522	0.32494172	-0.4847699	0.62783954	NA		F930015N05R	0.67204633	0.90504744	0.31807903	2.06814001	0	1.17605452	0	0
Clta	96.3076609	0.14388982	0.29696204	0.48453945	0.62800307	0.99938111		Clta	88.7101153	59.7331309	81.7463119	134.429101	94.8942681	65.8590533	105.849028	139.240279
Chsy1	10.7143958	-0.1955419	0.40363989	-0.4844465	0.62806903	0.99938111		Chsy1	13.4409266	17.1959013	13.0412404	4.13628002	3.32962344	18.8168724	13.4716944	2.28262753
Grik4	10.4293019	-0.1955071	0.4039115	-0.4840346	0.62836132	0.99938111		Grik4	5.37637063	9.5029981	9.54237104	22.7495401	9.98887032	12.9365998	1.92452778	11.4131377
Rad18	4.91798851	0.19794381	0.40899628	0.48397461	0.62840389	0.99938111		Rad18	7.39250961	2.71514231	3.81694841	4.13628002	4.99443516	8.23238167	5.77358333	2.28262753
Tgm2	2.19203925	0.19599592	0.40503846	0.48389459	0.62846068	NA		Tgm2	2.01613899	3.16766603	1.27231614	0	6.65924688	1.17605452	0.96226389	2.28262753
Rfx5	23.9270998	0.16491523	0.340946	0.48369896	0.62859953	0.99938111		Rfx5	17.4732045	12.6706641	22.5836114	39.2946602	31.6314227	23.5210905	25.981125	18.2610202
Dalrd3	40.9455897	0.14293555	0.29550553	0.48369839	0.62859994	0.99938111		Dalrd3	41.6668724	33.0342315	28.6271131	51.7035003	41.620293	31.7534722	37.5282917	61.6309433
Gpkow	22.8207655	0.17357904	0.35890004	0.48364173	0.62864015	0.99938111		Gpkow	21.5054825	22.6261859	24.4920857	16.5451201	13.3184938	36.4576902	38.4905555	9.13051012
Hiatl1	29.0067157	0.14888823	0.30786518	0.48361503	0.62865911	0.99938111		Hiatl1	20.1613899	37.5594687	29.8994292	18.6132601	36.6258578	27.0492541	27.9056528	34.239413
She	0.97461806	0.16342294	0.33799609	0.48350541	0.62873692	NA		She	0	0.90504744	0.31807903	2.06814001	3.32962344	1.17605452	0	0
Sphk1	0.91467847	-0.1714108	0.35453755	-0.483477	0.62875708	NA		Sphk1	0.67204633	1.81009488	1.59039517	0	0	0	0.96226389	2.28262753
Coprs	21.9457454	0.17937993	0.37105831	0.48342788	0.62879196	0.99938111		Coprs	12.7688802	11.7656167	15.5858727	43.4309402	21.6425524	14.1126543	28.8679167	27.3915304
Neil1	1.52920727	0.16455981	0.34066291	0.48305762	0.62905483	NA		Neil1	0	0.90504744	0	4.13628002	1.66481172	0	0.96226389	4.56525506
Zkscan7	0.99986594	0.17681611	0.36621653	0.48281849	0.62922462	NA		Zkscan7	1.34409266	1.35757116	0.31807903	0	1.66481172	2.35210905	0.96226389	0
Slc7a8	60.7679518	-0.1499225	0.31063158	-0.4826376	0.62935304	0.99938111		Slc7a8	51.075521	98.197647	62.9796488	43.4309402	73.2517157	65.8590533	36.5660278	54.7830607
Eif2ak2	2.42869734	-0.1959793	0.40608711	-0.4826041	0.62937685	NA		Eif2ak2	7.39250961	2.26261859	1.90847421	0	1.66481172	2.35210905	3.84905555	0
Faf2	30.2225331	0.16767575	0.34757536	0.48241553	0.62951079	0.99938111		Faf2	27.5538995	39.3695635	24.8101647	18.6132601	33.2962344	51.7463991	14.4339583	31.9567854
Adamts20	1.7991985	-0.1936794	0.40149817	-0.4823918	0.62952767	NA		Adamts20	1.34409266	3.62018975	1.90847421	2.06814001	0	3.52816357	1.92452778	0
Pou6f2	1.21800743	-0.1786521	0.37045061	-0.4822561	0.62962403	NA		Pou6f2	3.36023164	2.26261859	0.31807903	0	1.66481172	1.17605452	0.96226389	0
Tnfrsf8	1.21800743	-0.1786521	0.37045061	-0.4822561	0.62962403	NA		Tnfrsf8	3.36023164	2.26261859	0.31807903	0	1.66481172	1.17605452	0.96226389	0
Pcmt1	59.965105	-0.1528355	0.31693041	-0.4822369	0.62963769	0.99938111		Pcmt1	73.9250961	66.9735104	71.8858618	43.4309402	41.620293	82.3238167	72.1697916	27.3915304
Usp11	180.495554	0.14583687	0.30243354	0.4822113	0.62965585	0.99938111		Usp11	142.473822	116.751119	148.542909	272.994481	213.0959	152.887088	134.716944	262.502166
Hist1h2bq	4.43825535	-0.2002654	0.41539766	-0.4821052	0.6297312	NA		Hist1h2bq	10.0806949	4.07271347	3.18079035	2.06814001	1.66481172	4.7042181	2.88679167	6.84788259
Hist1h2br	4.43825535	-0.2002654	0.41539766	-0.4821052	0.6297312	NA		Hist1h2br	10.0806949	4.07271347	3.18079035	2.06814001	1.66481172	4.7042181	2.88679167	6.84788259
Btg2	9.92527226	-0.1929805	0.40028764	-0.4821047	0.62973158	0.99938111		Btg2	6.72046328	9.05047438	5.72542262	24.8176801	9.98887032	4.7042181	11.5471667	6.84788259
Nbr1	61.6669607	0.14579698	0.3024814	0.48200315	0.62980371	0.99938111		Nbr1	55.7798453	75.1189373	59.7988585	41.3628002	66.5924688	90.5561984	72.1697916	31.9567854
Txnl1	60.0397052	0.12773127	0.26508127	0.48185704	0.6299075	0.99938111		Txnl1	52.4196136	56.5654649	56.2999891	66.1804803	43.2851047	76.4435441	78.9056389	50.2178057
Paf1	27.884715	0.14271434	0.29620122	0.4818155	0.62993702	0.99938111		Paf1	22.1775288	24.4362808	21.9474534	39.2946602	26.6369875	32.9295267	25.981125	29.6741579
Trpv4	0.9429347	-0.1468002	0.30475886	-0.4816929	0.63002415	NA		Trpv4	0.67204633	0.45252372	0	4.13628002	0	0	0	2.28262753
St6galnac6	50.7983859	0.16232279	0.3369959	0.48167585	0.63003624	0.99938111		St6galnac6	34.2743627	33.4867552	40.3960374	82.7256004	78.2461509	32.9295267	40.4150833	63.9135709
A930011G23Rik	2.35770095	0.18671867	0.3877759	0.4815118	0.6301528	NA		A930011G23R	3.36023164	1.35757116	0.31807903	2.06814001	1.66481172	0	0.96226389	9.13051012
Nmral1	4.62857515	-0.199642	0.41466626	-0.4814522	0.63019512	NA		Nmral1	2.68818531	2.26261859	3.81694841	14.4769801	1.66481172	1.17605452	8.660375	2.28262753
4931417E11Rik	0.27217045	-0.1043394	0.21672063	-0.4814464	0.6301993	NA		4931417E11Ri	0	0.90504744	1.27231614	0	0	0	0	0
Mpzl3	0.27217045	-0.1043394	0.21672063	-0.4814464	0.6301993	NA		Mpzl3	0	0.90504744	1.27231614	0	0	0	0	0
Plp2	0.27217045	-0.1043394	0.21672063	-0.4814464	0.6301993	NA		Plp2	0	0.90504744	1.27231614	0	0	0	0	0
Haus1	2.83613631	0.19999022	0.4154827	0.48134429	0.63027183	NA		Haus1	4.03227797	2.26261859	1.90847421	2.06814001	3.32962344	2.35210905	6.73584722	0
Trnp1	87.0150557	0.1448975	0.3011557	0.48113815	0.63041832	0.99938111		Trnp1	86.6939764	71.9512713	80.1559167	86.8618804	78.2461509	48.2182355	93.3395972	150.653417
Tmod2	639.181403	0.12415292	0.25804425	0.48113036	0.63042385	0.99938111		Tmod2	594.088954	649.82406	678.144502	512.898723	506.102763	806.773404	904.528055	461.090761
Ighmbp2	4.24913856	0.19921732	0.41406994	0.48111998	0.63043123	NA		Ighmbp2	3.36023164	4.07271347	3.18079035	4.13628002	6.65924688	7.05632714	0.96226389	4.56525506
2610001J05Rik	16.6461454	-0.1866676	0.38804289	-0.4810488	0.6304818	0.99938111		2610001J05Ri	16.1291119	19.0059962	24.4920857	12.4088401	6.65924688	4.7042181	26.9433889	22.8262753
Nedd1	1.14777578	0.16286767	0.33863515	0.48095325	0.63054973	NA		Nedd1	0.67204633	1.35757116	1.27231614	0	0	5.88027262	0	0
Trdmt1	2.43295596	-0.1996149	0.41511959	-0.4808612	0.63061518	NA		Trdmt1	2.01613899	3.16766603	3.49886938	2.06814001	3.32962344	1.17605452	1.92452778	2.28262753
Mark4	63.9286302	0.13551827	0.28191677	0.48070312	0.63072752	0.99938111		Mark4	75.2691888	41.6321821	46.7576181	80.6574604	78.2461509	58.8027262	61.5848889	68.4788259
Trappc8	43.3518289	-0.1771033	0.36844175	-0.4806819	0.63074262	0.99938111		Trappc8	65.8605402	50.2301328	51.5288036	20.6814001	31.6314227	63.5069443	51.96225	11.4131377
Tnfsf13b	0.5988915	-0.1555269	0.32359039	-0.4806288	0.63078036	NA		Tnfsf13b	0.67204633	0.45252372	0.63615807	2.06814001	0	0	0.96226389	0
Zdhhc4	15.1366703	-0.1855755	0.38626227	-0.4804392	0.63091511	0.99938111		Zdhhc4	14.7850192	14.0282353	11.7689243	24.8176801	16.6481172	5.88027262	5.77358333	27.3915304
Dear1	2.33169352	0.19569603	0.40753127	0.48019881	0.63108603	NA		Dear1	0.67204633	2.71514231	3.49886938	0	4.99443516	3.52816357	0.96226389	2.28262753
Apoc3	0.67472043	0.11744058	0.24457847	0.48017547	0.63110263	NA		Apoc3	0	0	0	2.06814001	3.32962344	0	0	0
Stmn1-rs1	0.67472043	0.11744058	0.24457847	0.48017547	0.63110263	NA		Stmn1-rs1	0	0	0	2.06814001	3.32962344	0	0	0
Slc25a21	0.46823921	0.1287793	0.26819703	0.48016674	0.63110883	NA		Slc25a21	0	0.90504744	0	0	1.66481172	1.17605452	0	0
Sgpp1	32.6840213	0.15530156	0.32343959	0.48015629	0.63111626	0.99938111		Sgpp1	20.1613899	46.1574193	32.4440615	22.7495401	36.6258578	45.8661264	34.6415	22.8262753
Rpp38	2.85964273	0.19836836	0.41331686	0.47994259	0.63126822	NA		Rpp38	3.36023164	3.16766603	2.86271131	0	3.32962344	8.23238167	1.92452778	0
Rcan1	41.3628	-0.120176	0.25041171	-0.4799135	0.63128887	0.99938111		Rcan1	47.7152893	39.8220873	40.7141164	45.4990802	36.6258578	39.9858538	39.4528194	41.0872956
Mthfd1	16.7773419	-0.1607244	0.33494254	-0.4798568	0.63132924	0.99938111		Mthfd1	20.1613899	11.7656167	17.8124259	22.7495401	13.3184938	17.6408179	12.5094306	18.2610202
Fam219aos	46.4973003	0.18911488	0.39417358	0.47977565	0.63138693	0.99938111		Fam219aos	37.6345944	25.793852	26.0824808	78.5893204	71.586904	22.345036	16.3584861	93.5877288
Hes6	9.35628905	0.19915125	0.41511372	0.47975107	0.63140441	0.99938111		Hes6	8.73660227	4.97776091	2.86271131	16.5451201	14.9833055	2.35210905	3.84905555	20.5436478
Phospho1	18.0219429	0.18338768	0.38264067	0.47926866	0.63174752	0.99938111		Phospho1	18.8172972	12.6706641	14.6316356	18.6132601	26.6369875	3.52816357	17.32075	31.9567854
Zbtb38	45.7704432	0.16203891	0.33810131	0.47926142	0.63175267	0.99938111		Zbtb38	55.1077989	49.3250854	46.439539	18.6132601	39.9554813	72.9153805	58.6980972	25.1089028
Unc50	14.4804143	-0.1661849	0.34691078	-0.4790422	0.63190861	0.99938111		Unc50	13.4409266	14.0282353	16.2220308	20.6814001	16.6481172	12.9365998	17.32075	4.56525506
Scarf2	4.30355319	0.19944681	0.41636985	0.47901358	0.63192897	NA		Scarf2	2.01613899	1.81009488	3.49886938	8.27256004	9.98887032	2.35210905	1.92452778	4.56525506
Bfar	14.5331436	-0.1827784	0.38160831	-0.4789685	0.63196105	0.99938111		Bfar	15.4570656	21.2686148	19.7209001	6.20442003	4.99443516	24.697145	12.5094306	11.4131377
Rps27l	3.30581583	-0.1993838	0.41634716	-0.4788883	0.63201808	NA		Rps27l	4.03227797	2.26261859	3.18079035	6.20442003	0	2.35210905	3.84905555	4.56525506

GeneID	Base mean	log2(FC)	StdErr	Wald-Stats	P-value	P-adj		GeneID	Normalised expression for Chow#1	Normalised expression for Chow#2	Normalised expression for Chow#3	Normalised expression for Chow#4	Normalised expression for HFD#1	Normalised expression for HFD#2	Normalised expression for HFD#3	Normalised expression for HFD#4
Mrvi1	0.84193753	-0.1700631	0.35514557	-0.4788546	0.63204205	NA		Mrvi1	1.34409266	1.81009488	0.9542371	0	1.66481172	0	0.96226389	0
Pdzd9	0.5399686	0.13129545	0.27426158	0.47872347	0.63213536	NA		Pdzd9	0.67204633	0	0.31807903	0	3.32962344	0	0	0
Plekhj1	14.5648691	-0.1709078	0.35714806	-0.4785349	0.63226957	0.99938111		Plekhj1	16.8011582	17.1959013	12.7231614	14.4769801	18.3129289	5.88027262	10.5849028	20.5436478
Klhl24	61.9678875	0.14070175	0.29404457	0.47850485	0.63229092	0.99938111		Klhl24	58.4680306	60.6381783	69.9773876	45.4990802	56.6035985	84.6759257	85.6414861	34.239413
Serpina9	0.89327833	-0.1628532	0.34035785	-0.4784763	0.63231121	NA		Serpina9	0.67204633	0.45252372	3.18079035	0	1.66481172	1.17605452	0	0
Cers6	41.6977343	0.18521129	0.38715756	0.47838737	0.63237452	0.99938111		Cers6	32.2582238	52.9452751	50.8926455	14.4769801	24.9721758	99.9646345	37.5282917	20.5436478
Setd4	1.07518582	-0.1666994	0.34846191	-0.4783863	0.63237526	NA		Setd4	2.01613899	0	1.27231614	2.06814001	0	0	0.96226389	2.28262753
Tsg101	21.6223647	0.17321647	0.36213355	0.47832208	0.63242099	0.99938111		Tsg101	23.5216215	20.8160911	23.5378486	10.3407001	9.98887032	15.2887088	37.5282917	31.9567854
Ccdc43	14.3707629	0.16648354	0.34814036	0.47820809	0.6325021	0.99938111		Ccdc43	11.4247876	14.0282353	14.6316356	14.4769801	13.3184938	12.9365998	25.0188611	9.13051012
Wdr65	0.97857432	0.17672035	0.36959779	0.47814234	0.6325489	NA		Wdr65	0.67204633	0.90504744	1.27231614	0	1.66481172	2.35210905	0.96226389	0
1700007K09Rik	0.20810147	0.08919133	0.18656246	0.47807757	0.632595	NA		1700007K09Ri	0	0	0	0	1.66481172	0	0	0
4930455H04Rik	0.20810147	0.08919133	0.18656246	0.47807757	0.632595	NA		4930455H04Ri	0	0	0	0	1.66481172	0	0	0
4930486L24Rik	0.20810147	0.08919133	0.18656246	0.47807757	0.632595	NA		4930486L24Ri	0	0	0	0	1.66481172	0	0	0
4930502E09Rik	0.20810147	0.08919133	0.18656246	0.47807757	0.632595	NA		4930502E09Ri	0	0	0	0	1.66481172	0	0	0
4930540M03Rik	0.20810147	0.08919133	0.18656246	0.47807757	0.632595	NA		4930540M03R	0	0	0	0	1.66481172	0	0	0
4930567H17Rik	0.20810147	0.08919133	0.18656246	0.47807757	0.632595	NA		4930567H17Ri	0	0	0	0	1.66481172	0	0	0
4930578C19Rik	0.20810147	0.08919133	0.18656246	0.47807757	0.632595	NA		4930578C19Ri	0	0	0	0	1.66481172	0	0	0
4931419H13Rik	0.20810147	0.08919133	0.18656246	0.47807757	0.632595	NA		4931419H13Ri	0	0	0	0	1.66481172	0	0	0
5730422E09Rik	0.20810147	0.08919133	0.18656246	0.47807757	0.632595	NA		5730422E09Ri	0	0	0	0	1.66481172	0	0	0
9530091C08Rik	0.20810147	0.08919133	0.18656246	0.47807757	0.632595	NA		9530091C08Ri	0	0	0	0	1.66481172	0	0	0
A530016L24Rik	0.20810147	0.08919133	0.18656246	0.47807757	0.632595	NA		A530016L24Ri	0	0	0	0	1.66481172	0	0	0
AB041803	0.20810147	0.08919133	0.18656246	0.47807757	0.632595	NA		AB041803	0	0	0	0	1.66481172	0	0	0
Adcy10	0.20810147	0.08919133	0.18656246	0.47807757	0.632595	NA		Adcy10	0	0	0	0	1.66481172	0	0	0
AU040972	0.20810147	0.08919133	0.18656246	0.47807757	0.632595	NA		AU040972	0	0	0	0	1.66481172	0	0	0
AW822252	0.20810147	0.08919133	0.18656246	0.47807757	0.632595	NA		AW822252	0	0	0	0	1.66481172	0	0	0
B230208H11Rik	0.20810147	0.08919133	0.18656246	0.47807757	0.632595	NA		B230208H11R	0	0	0	0	1.66481172	0	0	0
Bach2os	0.20810147	0.08919133	0.18656246	0.47807757	0.632595	NA		Bach2os	0	0	0	0	1.66481172	0	0	0
Bhlhe23	0.20810147	0.08919133	0.18656246	0.47807757	0.632595	NA		Bhlhe23	0	0	0	0	1.66481172	0	0	0
Bpifc	0.20810147	0.08919133	0.18656246	0.47807757	0.632595	NA		Bpifc	0	0	0	0	1.66481172	0	0	0
Btc	0.20810147	0.08919133	0.18656246	0.47807757	0.632595	NA		Btc	0	0	0	0	1.66481172	0	0	0
C030013G03Rik	0.20810147	0.08919133	0.18656246	0.47807757	0.632595	NA		C030013G03R	0	0	0	0	1.66481172	0	0	0
Cabp4	0.20810147	0.08919133	0.18656246	0.47807757	0.632595	NA		Cabp4	0	0	0	0	1.66481172	0	0	0
Cacng6	0.20810147	0.08919133	0.18656246	0.47807757	0.632595	NA		Cacng6	0	0	0	0	1.66481172	0	0	0
Cdkn2a	0.20810147	0.08919133	0.18656246	0.47807757	0.632595	NA		Cdkn2a	0	0	0	0	1.66481172	0	0	0
Cfc1	0.20810147	0.08919133	0.18656246	0.47807757	0.632595	NA		Cfc1	0	0	0	0	1.66481172	0	0	0
Cpn1	0.20810147	0.08919133	0.18656246	0.47807757	0.632595	NA		Cpn1	0	0	0	0	1.66481172	0	0	0
Cpsf4l	0.20810147	0.08919133	0.18656246	0.47807757	0.632595	NA		Cpsf4l	0	0	0	0	1.66481172	0	0	0
Cryaa	0.20810147	0.08919133	0.18656246	0.47807757	0.632595	NA		Cryaa	0	0	0	0	1.66481172	0	0	0
Crybb3	0.20810147	0.08919133	0.18656246	0.47807757	0.632595	NA		Crybb3	0	0	0	0	1.66481172	0	0	0
Cyp2e1	0.20810147	0.08919133	0.18656246	0.47807757	0.632595	NA		Cyp2e1	0	0	0	0	1.66481172	0	0	0
Dgat2l6	0.20810147	0.08919133	0.18656246	0.47807757	0.632595	NA		Dgat2l6	0	0	0	0	1.66481172	0	0	0
Dpy19l2	0.20810147	0.08919133	0.18656246	0.47807757	0.632595	NA		Dpy19l2	0	0	0	0	1.66481172	0	0	0
Dupd1	0.20810147	0.08919133	0.18656246	0.47807757	0.632595	NA		Dupd1	0	0	0	0	1.66481172	0	0	0
E130304I02Rik	0.20810147	0.08919133	0.18656246	0.47807757	0.632595	NA		E130304I02Ri	0	0	0	0	1.66481172	0	0	0
Erich4	0.20810147	0.08919133	0.18656246	0.47807757	0.632595	NA		Erich4	0	0	0	0	1.66481172	0	0	0
Espnl	0.20810147	0.08919133	0.18656246	0.47807757	0.632595	NA		Espnl	0	0	0	0	1.66481172	0	0	0
Fcgbp	0.20810147	0.08919133	0.18656246	0.47807757	0.632595	NA		Fcgbp	0	0	0	0	1.66481172	0	0	0
Fgf17	0.20810147	0.08919133	0.18656246	0.47807757	0.632595	NA		Fgf17	0	0	0	0	1.66481172	0	0	0
Foxl2	0.20810147	0.08919133	0.18656246	0.47807757	0.632595	NA		Foxl2	0	0	0	0	1.66481172	0	0	0
Foxl2os	0.20810147	0.08919133	0.18656246	0.47807757	0.632595	NA		Foxl2os	0	0	0	0	1.66481172	0	0	0
Galnt5	0.20810147	0.08919133	0.18656246	0.47807757	0.632595	NA		Galnt5	0	0	0	0	1.66481172	0	0	0
Gdpd3	0.20810147	0.08919133	0.18656246	0.47807757	0.632595	NA		Gdpd3	0	0	0	0	1.66481172	0	0	0
Gjb3	0.20810147	0.08919133	0.18656246	0.47807757	0.632595	NA		Gjb3	0	0	0	0	1.66481172	0	0	0
Gm10635	0.20810147	0.08919133	0.18656246	0.47807757	0.632595	NA		Gm10635	0	0	0	0	1.66481172	0	0	0
Gm14207	0.20810147	0.08919133	0.18656246	0.47807757	0.632595	NA		Gm14207	0	0	0	0	1.66481172	0	0	0
Gm15881	0.20810147	0.08919133	0.18656246	0.47807757	0.632595	NA		Gm15881	0	0	0	0	1.66481172	0	0	0
Gm20735	0.20810147	0.08919133	0.18656246	0.47807757	0.632595	NA		Gm20735	0	0	0	0	1.66481172	0	0	0
Gpr20	0.20810147	0.08919133	0.18656246	0.47807757	0.632595	NA		Gpr20	0	0	0	0	1.66481172	0	0	0
Gpr97	0.20810147	0.08919133	0.18656246	0.47807757	0.632595	NA		Gpr97	0	0	0	0	1.66481172	0	0	0
Grxcr2	0.20810147	0.08919133	0.18656246	0.47807757	0.632595	NA		Grxcr2	0	0	0	0	1.66481172	0	0	0
Gypa	0.20810147	0.08919133	0.18656246	0.47807757	0.632595	NA		Gypa	0	0	0	0	1.66481172	0	0	0
H2-Q6	0.20810147	0.08919133	0.18656246	0.47807757	0.632595	NA		H2-Q6	0	0	0	0	1.66481172	0	0	0
H2-Q8	0.20810147	0.08919133	0.18656246	0.47807757	0.632595	NA		H2-Q8	0	0	0	0	1.66481172	0	0	0
Hhipl2	0.20810147	0.08919133	0.18656246	0.47807757	0.632595	NA		Hhipl2	0	0	0	0	1.66481172	0	0	0
Kcnk15	0.20810147	0.08919133	0.18656246	0.47807757	0.632595	NA		Kcnk15	0	0	0	0	1.66481172	0	0	0
Krt83	0.20810147	0.08919133	0.18656246	0.47807757	0.632595	NA		Krt83	0	0	0	0	1.66481172	0	0	0
Krt85	0.20810147	0.08919133	0.18656246	0.47807757	0.632595	NA		Krt85	0	0	0	0	1.66481172	0	0	0
Lrrc10	0.20810147	0.08919133	0.18656246	0.47807757	0.632595	NA		Lrrc10	0	0	0	0	1.66481172	0	0	0
Ltb4r2	0.20810147	0.08919133	0.18656246	0.47807757	0.632595	NA		Ltb4r2	0	0	0	0	1.66481172	0	0	0
Marcksl1-ps4	0.20810147	0.08919133	0.18656246	0.47807757	0.632595	NA		Marcksl1-ps4	0	0	0	0	1.66481172	0	0	0
Mir1249	0.20810147	0.08919133	0.18656246	0.47807757	0.632595	NA		Mir1249	0	0	0	0	1.66481172	0	0	0
Mir1928	0.20810147	0.08919133	0.18656246	0.47807757	0.632595	NA		Mir1928	0	0	0	0	1.66481172	0	0	0
Mir1966	0.20810147	0.08919133	0.18656246	0.47807757	0.632595	NA		Mir1966	0	0	0	0	1.66481172	0	0	0
Mir3083	0.20810147	0.08919133	0.18656246	0.47807757	0.632595	NA		Mir3083	0	0	0	0	1.66481172	0	0	0
Mir466d	0.20810147	0.08919133	0.18656246	0.47807757	0.632595	NA		Mir466d	0	0	0	0	1.66481172	0	0	0
Mir6390	0.20810147	0.08919133	0.18656246	0.47807757	0.632595	NA		Mir6390	0	0	0	0	1.66481172	0	0	0
Mir7045	0.20810147	0.08919133	0.18656246	0.47807757	0.632595	NA		Mir7045	0	0	0	0	1.66481172	0	0	0
Mir7682	0.20810147	0.08919133	0.18656246	0.47807757	0.632595	NA		Mir7682	0	0	0	0	1.66481172	0	0	0
Mir8115	0.20810147	0.08919133	0.18656246	0.47807757	0.632595	NA		Mir8115	0	0	0	0	1.66481172	0	0	0
Mmrn1	0.20810147	0.08919133	0.18656246	0.47807757	0.632595	NA		Mmrn1	0	0	0	0	1.66481172	0	0	0
Mybl2	0.20810147	0.08919133	0.18656246	0.47807757	0.632595	NA		Mybl2	0	0	0	0	1.66481172	0	0	0
Nxnl1	0.20810147	0.08919133	0.18656246	0.47807757	0.632595	NA		Nxnl1	0	0	0	0	1.66481172	0	0	0
Obp2a	0.20810147	0.08919133	0.18656246	0.47807757	0.632595	NA		Obp2a	0	0	0	0	1.66481172	0	0	0
Olfr1395	0.20810147	0.08919133	0.18656246	0.47807757	0.632595	NA		Olfr1395	0	0	0	0	1.66481172	0	0	0
Rhd	0.20810147	0.08919133	0.18656246	0.47807757	0.632595	NA		Rhd	0	0	0	0	1.66481172	0	0	0
Rprl3	0.20810147	0.08919133	0.18656246	0.47807757	0.632595	NA		Rprl3	0	0	0	0	1.66481172	0	0	0
Scnn1g	0.20810147	0.08919133	0.18656246	0.47807757	0.632595	NA		Scnn1g	0	0	0	0	1.66481172	0	0	0
Sgol1	0.20810147	0.08919133	0.18656246	0.47807757	0.632595	NA		Sgol1	0	0	0	0	1.66481172	0	0	0
Sigirr	0.20810147	0.08919133	0.18656246	0.47807757	0.632595	NA		Sigirr	0	0	0	0	1.66481172	0	0	0
Slc11a1	0.20810147	0.08919133	0.18656246	0.47807757	0.632595	NA		Slc11a1	0	0	0	0	1.66481172	0	0	0
Slc2a4rg-ps	0.20810147	0.08919133	0.18656246	0.47807757	0.632595	NA		Slc2a4rg-ps	0	0	0	0	1.66481172	0	0	0
Smim9	0.20810147	0.08919133	0.18656246	0.47807757	0.632595	NA		Smim9	0	0	0	0	1.66481172	0	0	0
Snora47	0.20810147	0.08919133	0.18656246	0.47807757	0.632595	NA		Snora47	0	0	0	0	1.66481172	0	0	0
Snord33	0.20810147	0.08919133	0.18656246	0.47807757	0.632595	NA		Snord33	0	0	0	0	1.66481172	0	0	0
Snord49b	0.20810147	0.08919133	0.18656246	0.47807757	0.632595	NA		Snord49b	0	0	0	0	1.66481172	0	0	0
Snord83b	0.20810147	0.08919133	0.18656246	0.47807757	0.632595	NA		Snord83b	0	0	0	0	1.66481172	0	0	0
Sox15	0.20810147	0.08919133	0.18656246	0.47807757	0.632595	NA		Sox15	0	0	0	0	1.66481172	0	0	0
Tmem106a	0.20810147	0.08919133	0.18656246	0.47807757	0.632595	NA		Tmem106a	0	0	0	0	1.66481172	0	0	0
Tmem184a	0.20810147	0.08919133	0.18656246	0.47807757	0.632595	NA		Tmem184a	0	0	0	0	1.66481172	0	0	0
Tnfrsf25	0.20810147	0.08919133	0.18656246	0.47807757	0.632595	NA		Tnfrsf25	0	0	0	0	1.66481172	0	0	0
Tnp2	0.20810147	0.08919133	0.18656246	0.47807757	0.632595	NA		Tnp2	0	0	0	0	1.66481172	0	0	0
Uts2r	0.20810147	0.08919133	0.18656246	0.47807757	0.632595	NA		Uts2r	0	0	0	0	1.66481172	0	0	0
Xpnpep2	0.20810147	0.08919133	0.18656246	0.47807757	0.632595	NA		Xpnpep2	0	0	0	0	1.66481172	0	0	0
1700007P06Rik	0.12028299	0.08919132	0.18656246	0.47807756	0.632595	NA		1700007P06Ri	0	0	0	0	0	0	0.96226389	0
1700009C05Rik	0.12028299	0.08919132	0.18656246	0.47807756	0.632595	NA		1700009C05Ri	0	0	0	0	0	0	0.96226389	0
1700015F17Rik	0.14700682	0.08919132	0.18656246	0.47807756	0.632595	NA		1700015F17Ri	0	0	0	0	0	1.17605452	0	0
1700016D06Rik	0.12028299	0.08919132	0.18656246	0.47807756	0.632595	NA		1700016D06Ri	0	0	0	0	0	0	0.96226389	0
1700024G13Rik	0.14700682	0.08919132	0.18656246	0.47807756	0.632595	NA		1700024G13R	0	0	0	0	0	1.17605452	0	0
1700034F02Rik	0.12028299	0.08919132	0.18656246	0.47807756	0.632595	NA		1700034F02Ri	0	0	0	0	0	0	0.96226389	0
1700063O14Rik	0.12028299	0.08919132	0.18656246	0.47807756	0.632595	NA		1700063O14R	0	0	0	0	0	0	0.96226389	0
1700065J18Rik	0.12028299	0.08919132	0.18656246	0.47807756	0.632595	NA		1700065J18Ri	0	0	0	0	0	0	0.96226389	0
2010005H15Rik	0.12028299	0.08919132	0.18656246	0.47807756	0.632595	NA		2010005H15Ri	0	0	0	0	0	0	0.96226389	0

GeneID	Base mean	log2(FC)	StdErr	Wald-Stats	P-value	P-adj		GeneID	Normalised expression for Chow#1	Normalised expression for Chow#2	Normalised expression for Chow#3	Normalised expression for Chow#4	Normalised expression for HFD#1	Normalised expression for HFD#2	Normalised expression for HFD#3	Normalised expression for HFD#4
2010106C02Rik	0.14700682	0.08919132	0.18656246	0.47807756	0.632595	NA		2010106C02Ri	0	0	0	0	0	1.17605452	0	0
2010109A12Rik	0.12028299	0.08919132	0.18656246	0.47807756	0.632595	NA		2010109A12Ri	0	0	0	0	0	0	0.96226389	0
2010308F09Rik	0.12028299	0.08919132	0.18656246	0.47807756	0.632595	NA		2010308F09Ri	0	0	0	0	0	0	0.96226389	0
2310043O21Rik	0.14700682	0.08919132	0.18656246	0.47807756	0.632595	NA		2310043O21R	0	0	0	0	0	1.17605452	0	0
2410012E07Rik	0.12028299	0.08919132	0.18656246	0.47807756	0.632595	NA		2410012E07Ri	0	0	0	0	0	0	0.96226389	0
2610207O16Rik	0.14700682	0.08919132	0.18656246	0.47807756	0.632595	NA		2610207O16R	0	0	0	0	0	1.17605452	0	0
2610528A11Rik	0.12028299	0.08919132	0.18656246	0.47807756	0.632595	NA		2610528A11Ri	0	0	0	0	0	0	0.96226389	0
2700038G22Rik	0.12028299	0.08919132	0.18656246	0.47807756	0.632595	NA		2700038G22R	0	0	0	0	0	0	0.96226389	0
2810404M03Rik	0.14700682	0.08919132	0.18656246	0.47807756	0.632595	NA		2810404M03R	0	0	0	0	0	1.17605452	0	0
3100003L05Rik	0.12028299	0.08919132	0.18656246	0.47807756	0.632595	NA		3100003L05Ri	0	0	0	0	0	0	0.96226389	0
3110015C05Rik	0.12028299	0.08919132	0.18656246	0.47807756	0.632595	NA		3110015C05Ri	0	0	0	0	0	0	0.96226389	0
3110021A11Rik	0.14700682	0.08919132	0.18656246	0.47807756	0.632595	NA		3110021A11Ri	0	0	0	0	0	1.17605452	0	0
4833427G06Rik	0.12028299	0.08919132	0.18656246	0.47807756	0.632595	NA		4833427G06R	0	0	0	0	0	0	0.96226389	0
4921501E09Rik	0.12028299	0.08919132	0.18656246	0.47807756	0.632595	NA		4921501E09Ri	0	0	0	0	0	0	0.96226389	0
4921509O07Rik	0.14700682	0.08919132	0.18656246	0.47807756	0.632595	NA		4921509O07R	0	0	0	0	0	1.17605452	0	0
4930467D21Rik	0.12028299	0.08919132	0.18656246	0.47807756	0.632595	NA		4930467D21Ri	0	0	0	0	0	0	0.96226389	0
4930467E23Rik	0.14700682	0.08919132	0.18656246	0.47807756	0.632595	NA		4930467E23Ri	0	0	0	0	0	1.17605452	0	0
4930487H11Rik	0.14700682	0.08919132	0.18656246	0.47807756	0.632595	NA		4930487H11Ri	0	0	0	0	0	1.17605452	0	0
4930515G16Rik	0.12028299	0.08919132	0.18656246	0.47807756	0.632595	NA		4930515G16R	0	0	0	0	0	0	0.96226389	0
4930515L03Rik	0.12028299	0.08919132	0.18656246	0.47807756	0.632595	NA		4930515L03Ri	0	0	0	0	0	0	0.96226389	0
4930556M19Rik	0.12028299	0.08919132	0.18656246	0.47807756	0.632595	NA		4930556M19R	0	0	0	0	0	0	0.96226389	0
4930567K20Rik	0.12028299	0.08919132	0.18656246	0.47807756	0.632595	NA		4930567K20Ri	0	0	0	0	0	0	0.96226389	0
4930592A05Rik	0.12028299	0.08919132	0.18656246	0.47807756	0.632595	NA		4930592A05Ri	0	0	0	0	0	0	0.96226389	0
4931408D14Rik	0.12028299	0.08919132	0.18656246	0.47807756	0.632595	NA		4931408D14Ri	0	0	0	0	0	0	0.96226389	0
4932443I19Rik	0.14700682	0.08919132	0.18656246	0.47807756	0.632595	NA		4932443I19Ri	0	0	0	0	0	1.17605452	0	0
4933433G15Rik	0.12028299	0.08919132	0.18656246	0.47807756	0.632595	NA		4933433G15R	0	0	0	0	0	0	0.96226389	0
9430076C15Rik	0.12028299	0.08919132	0.18656246	0.47807756	0.632595	NA		9430076C15Ri	0	0	0	0	0	0	0.96226389	0
A230009B12Rik	0.12028299	0.08919132	0.18656246	0.47807756	0.632595	NA		A230009B12R	0	0	0	0	0	0	0.96226389	0
Actl10	0.14700682	0.08919132	0.18656246	0.47807756	0.632595	NA		Actl10	0	0	0	0	0	1.17605452	0	0
AI427809	0.12028299	0.08919132	0.18656246	0.47807756	0.632595	NA		AI427809	0	0	0	0	0	0	0.96226389	0
Akr1b8	0.14700682	0.08919132	0.18656246	0.47807756	0.632595	NA		Akr1b8	0	0	0	0	0	1.17605452	0	0
Aldob	0.12028299	0.08919132	0.18656246	0.47807756	0.632595	NA		Aldob	0	0	0	0	0	0	0.96226389	0
Alox5	0.12028299	0.08919132	0.18656246	0.47807756	0.632595	NA		Alox5	0	0	0	0	0	0	0.96226389	0
Aqp1	0.14700682	0.08919132	0.18656246	0.47807756	0.632595	NA		Aqp1	0	0	0	0	0	1.17605452	0	0
Aqp3	0.14700682	0.08919132	0.18656246	0.47807756	0.632595	NA		Aqp3	0	0	0	0	0	1.17605452	0	0
Asprv1	0.12028299	0.08919132	0.18656246	0.47807756	0.632595	NA		Asprv1	0	0	0	0	0	0	0.96226389	0
Atp6v1e2	0.14700682	0.08919132	0.18656246	0.47807756	0.632595	NA		Atp6v1e2	0	0	0	0	0	1.17605452	0	0
Atp8b4	0.14700682	0.08919132	0.18656246	0.47807756	0.632595	NA		Atp8b4	0	0	0	0	0	1.17605452	0	0
Atp8b5	0.14700682	0.08919132	0.18656246	0.47807756	0.632595	NA		Atp8b5	0	0	0	0	0	1.17605452	0	0
AV051173	0.12028299	0.08919132	0.18656246	0.47807756	0.632595	NA		AV051173	0	0	0	0	0	0	0.96226389	0
Avpr1a	0.14700682	0.08919132	0.18656246	0.47807756	0.632595	NA		Avpr1a	0	0	0	0	0	1.17605452	0	0
BC051537	0.12028299	0.08919132	0.18656246	0.47807756	0.632595	NA		BC051537	0	0	0	0	0	0	0.96226389	0
BC055402	0.14700682	0.08919132	0.18656246	0.47807756	0.632595	NA		BC055402	0	0	0	0	0	1.17605452	0	0
Bnipl	0.14700682	0.08919132	0.18656246	0.47807756	0.632595	NA		Bnipl	0	0	0	0	0	1.17605452	0	0
C1ra	0.12028299	0.08919132	0.18656246	0.47807756	0.632595	NA		C1ra	0	0	0	0	0	0	0.96226389	0
C1rl	0.14700682	0.08919132	0.18656246	0.47807756	0.632595	NA		C1rl	0	0	0	0	0	1.17605452	0	0
C230029M16	0.12028299	0.08919132	0.18656246	0.47807756	0.632595	NA		C230029M16	0	0	0	0	0	0	0.96226389	0
C630031E19Rik	0.12028299	0.08919132	0.18656246	0.47807756	0.632595	NA		C630031E19Ri	0	0	0	0	0	0	0.96226389	0
Ccdc170	0.14700682	0.08919132	0.18656246	0.47807756	0.632595	NA		Ccdc170	0	0	0	0	0	1.17605452	0	0
Ccdc68	0.12028299	0.08919132	0.18656246	0.47807756	0.632595	NA		Ccdc68	0	0	0	0	0	0	0.96226389	0
Cdcp2	0.12028299	0.08919132	0.18656246	0.47807756	0.632595	NA		Cdcp2	0	0	0	0	0	0	0.96226389	0
Clec3a	0.12028299	0.08919132	0.18656246	0.47807756	0.632595	NA		Clec3a	0	0	0	0	0	0	0.96226389	0
Clec7a	0.14700682	0.08919132	0.18656246	0.47807756	0.632595	NA		Clec7a	0	0	0	0	0	1.17605452	0	0
Cndp1	0.12028299	0.08919132	0.18656246	0.47807756	0.632595	NA		Cndp1	0	0	0	0	0	0	0.96226389	0
Col9a1	0.12028299	0.08919132	0.18656246	0.47807756	0.632595	NA		Col9a1	0	0	0	0	0	0	0.96226389	0
Cyp2j8	0.12028299	0.08919132	0.18656246	0.47807756	0.632595	NA		Cyp2j8	0	0	0	0	0	0	0.96226389	0
D5Ertd605e	0.14700682	0.08919132	0.18656246	0.47807756	0.632595	NA		D5Ertd605e	0	0	0	0	0	1.17605452	0	0
D630010B17Rik	0.12028299	0.08919132	0.18656246	0.47807756	0.632595	NA		D630010B17R	0	0	0	0	0	0	0.96226389	0
D630032N06Rik	0.12028299	0.08919132	0.18656246	0.47807756	0.632595	NA		D630032N06R	0	0	0	0	0	0	0.96226389	0
Dsc1	0.12028299	0.08919132	0.18656246	0.47807756	0.632595	NA		Dsc1	0	0	0	0	0	0	0.96226389	0
Duox1	0.12028299	0.08919132	0.18656246	0.47807756	0.632595	NA		Duox1	0	0	0	0	0	0	0.96226389	0
Efhb	0.12028299	0.08919132	0.18656246	0.47807756	0.632595	NA		Efhb	0	0	0	0	0	0	0.96226389	0
Epb4.2	0.12028299	0.08919132	0.18656246	0.47807756	0.632595	NA		Epb4.2	0	0	0	0	0	0	0.96226389	0
Esrp1	0.14700682	0.08919132	0.18656246	0.47807756	0.632595	NA		Esrp1	0	0	0	0	0	1.17605452	0	0
Fbxw10	0.14700682	0.08919132	0.18656246	0.47807756	0.632595	NA		Fbxw10	0	0	0	0	0	1.17605452	0	0
Fcnb	0.14700682	0.08919132	0.18656246	0.47807756	0.632595	NA		Fcnb	0	0	0	0	0	1.17605452	0	0
Fgb	0.14700682	0.08919132	0.18656246	0.47807756	0.632595	NA		Fgb	0	0	0	0	0	1.17605452	0	0
Fgl1	0.12028299	0.08919132	0.18656246	0.47807756	0.632595	NA		Fgl1	0	0	0	0	0	0	0.96226389	0
Gldnos	0.14700682	0.08919132	0.18656246	0.47807756	0.632595	NA		Gldnos	0	0	0	0	0	1.17605452	0	0
Gm10536	0.12028299	0.08919132	0.18656246	0.47807756	0.632595	NA		Gm10536	0	0	0	0	0	0	0.96226389	0
Gm13238	0.14700682	0.08919132	0.18656246	0.47807756	0.632595	NA		Gm13238	0	0	0	0	0	1.17605452	0	0
Gm15645	0.12028299	0.08919132	0.18656246	0.47807756	0.632595	NA		Gm15645	0	0	0	0	0	0	0.96226389	0
Gm21119	0.14700682	0.08919132	0.18656246	0.47807756	0.632595	NA		Gm21119	0	0	0	0	0	1.17605452	0	0
Gm4971	0.12028299	0.08919132	0.18656246	0.47807756	0.632595	NA		Gm4971	0	0	0	0	0	0	0.96226389	0
Gm5535	0.14700682	0.08919132	0.18656246	0.47807756	0.632595	NA		Gm5535	0	0	0	0	0	1.17605452	0	0
Gm5941	0.12028299	0.08919132	0.18656246	0.47807756	0.632595	NA		Gm5941	0	0	0	0	0	0	0.96226389	0
Gm6588	0.14700682	0.08919132	0.18656246	0.47807756	0.632595	NA		Gm6588	0	0	0	0	0	1.17605452	0	0
Gm7361	0.14700682	0.08919132	0.18656246	0.47807756	0.632595	NA		Gm7361	0	0	0	0	0	1.17605452	0	0
Gnb3	0.12028299	0.08919132	0.18656246	0.47807756	0.632595	NA		Gnb3	0	0	0	0	0	0	0.96226389	0
Gpr87	0.12028299	0.08919132	0.18656246	0.47807756	0.632595	NA		Gpr87	0	0	0	0	0	0	0.96226389	0
Gucy1b2	0.12028299	0.08919132	0.18656246	0.47807756	0.632595	NA		Gucy1b2	0	0	0	0	0	0	0.96226389	0
H60b	0.12028299	0.08919132	0.18656246	0.47807756	0.632595	NA		H60b	0	0	0	0	0	0	0.96226389	0
Idi2	0.12028299	0.08919132	0.18656246	0.47807756	0.632595	NA		Idi2	0	0	0	0	0	0	0.96226389	0
Il1a	0.12028299	0.08919132	0.18656246	0.47807756	0.632595	NA		Il1a	0	0	0	0	0	0	0.96226389	0
Il1r2	0.12028299	0.08919132	0.18656246	0.47807756	0.632595	NA		Il1r2	0	0	0	0	0	0	0.96226389	0
Il31ra	0.12028299	0.08919132	0.18656246	0.47807756	0.632595	NA		Il31ra	0	0	0	0	0	0	0.96226389	0
Iqcf5	0.12028299	0.08919132	0.18656246	0.47807756	0.632595	NA		Iqcf5	0	0	0	0	0	0	0.96226389	0
Iqch	0.14700682	0.08919132	0.18656246	0.47807756	0.632595	NA		Iqch	0	0	0	0	0	1.17605452	0	0
Kbtbd12	0.14700682	0.08919132	0.18656246	0.47807756	0.632595	NA		Kbtbd12	0	0	0	0	0	1.17605452	0	0
Kbtbd13	0.12028299	0.08919132	0.18656246	0.47807756	0.632595	NA		Kbtbd13	0	0	0	0	0	0	0.96226389	0
Klb	0.14700682	0.08919132	0.18656246	0.47807756	0.632595	NA		Klb	0	0	0	0	0	1.17605452	0	0
Macc1	0.12028299	0.08919132	0.18656246	0.47807756	0.632595	NA		Macc1	0	0	0	0	0	0	0.96226389	0
Mgarp	0.14700682	0.08919132	0.18656246	0.47807756	0.632595	NA		Mgarp	0	0	0	0	0	1.17605452	0	0
Mir200c	0.12028299	0.08919132	0.18656246	0.47807756	0.632595	NA		Mir200c	0	0	0	0	0	0	0.96226389	0
Mir31	0.14700682	0.08919132	0.18656246	0.47807756	0.632595	NA		Mir31	0	0	0	0	0	1.17605452	0	0
Mir381	0.12028299	0.08919132	0.18656246	0.47807756	0.632595	NA		Mir381	0	0	0	0	0	0	0.96226389	0
Mir466b-3	0.12028299	0.08919132	0.18656246	0.47807756	0.632595	NA		Mir466b-3	0	0	0	0	0	0	0.96226389	0
Mir686	0.14700682	0.08919132	0.18656246	0.47807756	0.632595	NA		Mir686	0	0	0	0	0	1.17605452	0	0
Mir7221	0.12028299	0.08919132	0.18656246	0.47807756	0.632595	NA		Mir7221	0	0	0	0	0	0	0.96226389	0
Mirlet7f-1	0.14700682	0.08919132	0.18656246	0.47807756	0.632595	NA		Mirlet7f-1	0	0	0	0	0	1.17605452	0	0
Mlkl	0.12028299	0.08919132	0.18656246	0.47807756	0.632595	NA		Mlkl	0	0	0	0	0	0	0.96226389	0
Mtfr2	0.14700682	0.08919132	0.18656246	0.47807756	0.632595	NA		Mtfr2	0	0	0	0	0	1.17605452	0	0
Myh4	0.12028299	0.08919132	0.18656246	0.47807756	0.632595	NA		Myh4	0	0	0	0	0	0	0.96226389	0
Naip6	0.12028299	0.08919132	0.18656246	0.47807756	0.632595	NA		Naip6	0	0	0	0	0	0	0.96226389	0
Ndc80	0.14700682	0.08919132	0.18656246	0.47807756	0.632595	NA		Ndc80	0	0	0	0	0	1.17605452	0	0
Npb	0.12028299	0.08919132	0.18656246	0.47807756	0.632595	NA		Npb	0	0	0	0	0	0	0.96226389	0
Nr1h4	0.12028299	0.08919132	0.18656246	0.47807756	0.632595	NA		Nr1h4	0	0	0	0	0	0	0.96226389	0
Nxnl2	0.12028299	0.08919132	0.18656246	0.47807756	0.632595	NA		Nxnl2	0	0	0	0	0	0	0.96226389	0
Ostn	0.14700682	0.08919132	0.18656246	0.47807756	0.632595	NA		Ostn	0	0	0	0	0	1.17605452	0	0
Ovol1	0.12028299	0.08919132	0.18656246	0.47807756	0.632595	NA		Ovol1	0	0	0	0	0	0	0.96226389	0
P2rx2	0.12028299	0.08919132	0.18656246	0.47807756	0.632595	NA		P2rx2	0	0	0	0	0	0	0.96226389	0
Pdha2	0.14700682	0.08919132	0.18656246	0.47807756	0.632595	NA		Pdha2	0	0	0	0	0	1.17605452	0	0

GeneID	Base mean	log2(FC)	StdErr	Wald-Stats	P-value	P-adj	GeneID	Normalised expression for Chow#1	Normalised expression for Chow#2	Normalised expression for Chow#3	Normalised expression for Chow#4	Normalised expression for HFD#1	Normalised expression for HFD#2	Normalised expression for HFD#3	Normalised expression for HFD#4
Ptprq	0.12028299	0.08919132	0.18656246	0.47807756	0.632595	NA	Ptprq	0	0	0	0	0	0	0.96226389	0
Rdh18-ps	0.14700682	0.08919132	0.18656246	0.47807756	0.632595	NA	Rdh18-ps	0	0	0	0	0	1.17605452	0	0
Rnf151	0.14700682	0.08919132	0.18656246	0.47807756	0.632595	NA	Rnf151	0	0	0	0	0	1.17605452	0	0
Rnf17	0.12028299	0.08919132	0.18656246	0.47807756	0.632595	NA	Rnf17	0	0	0	0	0	0	0.96226389	0
Rp1	0.12028299	0.08919132	0.18656246	0.47807756	0.632595	NA	Rp1	0	0	0	0	0	0	0.96226389	0
Rpl39l	0.14700682	0.08919132	0.18656246	0.47807756	0.632595	NA	Rpl39l	0	0	0	0	0	1.17605452	0	0
Rtp2	0.14700682	0.08919132	0.18656246	0.47807756	0.632595	NA	Rtp2	0	0	0	0	0	1.17605452	0	0
Scarna6	0.12028299	0.08919132	0.18656246	0.47807756	0.632595	NA	Scarna6	0	0	0	0	0	0	0.96226389	0
Serpina10	0.12028299	0.08919132	0.18656246	0.47807756	0.632595	NA	Serpina10	0	0	0	0	0	0	0.96226389	0
Serpinc1	0.12028299	0.08919132	0.18656246	0.47807756	0.632595	NA	Serpinc1	0	0	0	0	0	0	0.96226389	0
Slc25a2	0.14700682	0.08919132	0.18656246	0.47807756	0.632595	NA	Slc25a2	0	0	0	0	0	1.17605452	0	0
Slc34a1	0.12028299	0.08919132	0.18656246	0.47807756	0.632595	NA	Slc34a1	0	0	0	0	0	0	0.96226389	0
Slc9a3	0.12028299	0.08919132	0.18656246	0.47807756	0.632595	NA	Slc9a3	0	0	0	0	0	0	0.96226389	0
Smok4a	0.14700682	0.08919132	0.18656246	0.47807756	0.632595	NA	Smok4a	0	0	0	0	0	1.17605452	0	0
Smpx	0.12028299	0.08919132	0.18656246	0.47807756	0.632595	NA	Smpx	0	0	0	0	0	0	0.96226389	0
Snora21	0.14700682	0.08919132	0.18656246	0.47807756	0.632595	NA	Snora21	0	0	0	0	0	1.17605452	0	0
Snora30	0.12028299	0.08919132	0.18656246	0.47807756	0.632595	NA	Snora30	0	0	0	0	0	0	0.96226389	0
Snora43	0.14700682	0.08919132	0.18656246	0.47807756	0.632595	NA	Snora43	0	0	0	0	0	1.17605452	0	0
Snora74a	0.12028299	0.08919132	0.18656246	0.47807756	0.632595	NA	Snora74a	0	0	0	0	0	0	0.96226389	0
Snora7a	0.12028299	0.08919132	0.18656246	0.47807756	0.632595	NA	Snora7a	0	0	0	0	0	0	0.96226389	0
Snord111	0.12028299	0.08919132	0.18656246	0.47807756	0.632595	NA	Snord111	0	0	0	0	0	0	0.96226389	0
Sorbs2os	0.14700682	0.08919132	0.18656246	0.47807756	0.632595	NA	Sorbs2os	0	0	0	0	0	1.17605452	0	0
Speer4b	0.12028299	0.08919132	0.18656246	0.47807756	0.632595	NA	Speer4b	0	0	0	0	0	0	0.96226389	0
Ssmem1	0.12028299	0.08919132	0.18656246	0.47807756	0.632595	NA	Ssmem1	0	0	0	0	0	0	0.96226389	0
St6galnac1	0.12028299	0.08919132	0.18656246	0.47807756	0.632595	NA	St6galnac1	0	0	0	0	0	0	0.96226389	0
Stpg2	0.12028299	0.08919132	0.18656246	0.47807756	0.632595	NA	Stpg2	0	0	0	0	0	0	0.96226389	0
Sult5a1	0.14700682	0.08919132	0.18656246	0.47807756	0.632595	NA	Sult5a1	0	0	0	0	0	1.17605452	0	0
Svopl	0.12028299	0.08919132	0.18656246	0.47807756	0.632595	NA	Svopl	0	0	0	0	0	0	0.96226389	0
Tas1r3	0.14700682	0.08919132	0.18656246	0.47807756	0.632595	NA	Tas1r3	0	0	0	0	0	1.17605452	0	0
Tinag	0.14700682	0.08919132	0.18656246	0.47807756	0.632595	NA	Tinag	0	0	0	0	0	1.17605452	0	0
Tnnt3	0.12028299	0.08919132	0.18656246	0.47807756	0.632595	NA	Tnnt3	0	0	0	0	0	0	0.96226389	0
Tpo	0.14700682	0.08919132	0.18656246	0.47807756	0.632595	NA	Tpo	0	0	0	0	0	1.17605452	0	0
Trim54	0.14700682	0.08919132	0.18656246	0.47807756	0.632595	NA	Trim54	0	0	0	0	0	1.17605452	0	0
Troap	0.14700682	0.08919132	0.18656246	0.47807756	0.632595	NA	Troap	0	0	0	0	0	1.17605452	0	0
Tsks	0.12028299	0.08919132	0.18656246	0.47807756	0.632595	NA	Tsks	0	0	0	0	0	0	0.96226389	0
Tsnaxip1	0.12028299	0.08919132	0.18656246	0.47807756	0.632595	NA	Tsnaxip1	0	0	0	0	0	0	0.96226389	0
Tspan2os	0.12028299	0.08919132	0.18656246	0.47807756	0.632595	NA	Tspan2os	0	0	0	0	0	0	0.96226389	0
Tyr	0.12028299	0.08919132	0.18656246	0.47807756	0.632595	NA	Tyr	0	0	0	0	0	0	0.96226389	0
Ucn	0.14700682	0.08919132	0.18656246	0.47807756	0.632595	NA	Ucn	0	0	0	0	0	1.17605452	0	0
Umodl1	0.14700682	0.08919132	0.18656246	0.47807756	0.632595	NA	Umodl1	0	0	0	0	0	1.17605452	0	0
Unc5cl	0.14700682	0.08919132	0.18656246	0.47807756	0.632595	NA	Unc5cl	0	0	0	0	0	1.17605452	0	0
Vmn2r84	0.14700682	0.08919132	0.18656246	0.47807756	0.632595	NA	Vmn2r84	0	0	0	0	0	1.17605452	0	0
Vmn2r86	0.14700682	0.08919132	0.18656246	0.47807756	0.632595	NA	Vmn2r86	0	0	0	0	0	1.17605452	0	0
Wdr72	0.12028299	0.08919132	0.18656246	0.47807756	0.632595	NA	Wdr72	0	0	0	0	0	0	0.96226389	0
Wnt9b	0.12028299	0.08919132	0.18656246	0.47807756	0.632595	NA	Wnt9b	0	0	0	0	0	0	0.96226389	0
Zc3h12d	0.14700682	0.08919132	0.18656246	0.47807756	0.632595	NA	Zc3h12d	0	0	0	0	0	1.17605452	0	0
Zfp474	0.12028299	0.08919132	0.18656246	0.47807756	0.632595	NA	Zfp474	0	0	0	0	0	0	0.96226389	0
Zp3r	0.12028299	0.08919132	0.18656246	0.47807756	0.632595	NA	Zp3r	0	0	0	0	0	0	0.96226389	0
1700064M15Rik	0.28532844	0.08919132	0.18656246	0.47807753	0.63259503	NA	1700064M15R	0	0	0	0	0	0	0	2.28262753
Crygs	0.28532844	0.08919132	0.18656246	0.47807753	0.63259503	NA	Crygs	0	0	0	0	0	0	0	2.28262753
Dgkeos	0.28532844	0.08919132	0.18656246	0.47807753	0.63259503	NA	Dgkeos	0	0	0	0	0	0	0	2.28262753
Dusp2	0.28532844	0.08919132	0.18656246	0.47807753	0.63259503	NA	Dusp2	0	0	0	0	0	0	0	2.28262753
Gchfr	0.28532844	0.08919132	0.18656246	0.47807753	0.63259503	NA	Gchfr	0	0	0	0	0	0	0	2.28262753
Gm11978	0.28532844	0.08919132	0.18656246	0.47807753	0.63259503	NA	Gm11978	0	0	0	0	0	0	0	2.28262753
Gm20199	0.28532844	0.08919132	0.18656246	0.47807753	0.63259503	NA	Gm20199	0	0	0	0	0	0	0	2.28262753
Gsdmd	0.28532844	0.08919132	0.18656246	0.47807753	0.63259503	NA	Gsdmd	0	0	0	0	0	0	0	2.28262753
Hes3	0.28532844	0.08919132	0.18656246	0.47807753	0.63259503	NA	Hes3	0	0	0	0	0	0	0	2.28262753
Hfe2	0.28532844	0.08919132	0.18656246	0.47807753	0.63259503	NA	Hfe2	0	0	0	0	0	0	0	2.28262753
Hs3st6	0.28532844	0.08919132	0.18656246	0.47807753	0.63259503	NA	Hs3st6	0	0	0	0	0	0	0	2.28262753
Ism2	0.28532844	0.08919132	0.18656246	0.47807753	0.63259503	NA	Ism2	0	0	0	0	0	0	0	2.28262753
Kifc1	0.28532844	0.08919132	0.18656246	0.47807753	0.63259503	NA	Kifc1	0	0	0	0	0	0	0	2.28262753
Krt23	0.28532844	0.08919132	0.18656246	0.47807753	0.63259503	NA	Krt23	0	0	0	0	0	0	0	2.28262753
Lep	0.28532844	0.08919132	0.18656246	0.47807753	0.63259503	NA	Lep	0	0	0	0	0	0	0	2.28262753
Mir128-1	0.28532844	0.08919132	0.18656246	0.47807753	0.63259503	NA	Mir128-1	0	0	0	0	0	0	0	2.28262753
Mir337	0.28532844	0.08919132	0.18656246	0.47807753	0.63259503	NA	Mir337	0	0	0	0	0	0	0	2.28262753
Mslnl	0.28532844	0.08919132	0.18656246	0.47807753	0.63259503	NA	Mslnl	0	0	0	0	0	0	0	2.28262753
Muc19	0.28532844	0.08919132	0.18656246	0.47807753	0.63259503	NA	Muc19	0	0	0	0	0	0	0	2.28262753
Oca2	0.28532844	0.08919132	0.18656246	0.47807753	0.63259503	NA	Oca2	0	0	0	0	0	0	0	2.28262753
Olfr95	0.28532844	0.08919132	0.18656246	0.47807753	0.63259503	NA	Olfr95	0	0	0	0	0	0	0	2.28262753
Osgin1	0.28532844	0.08919132	0.18656246	0.47807753	0.63259503	NA	Osgin1	0	0	0	0	0	0	0	2.28262753
Pdxk-ps	0.28532844	0.08919132	0.18656246	0.47807753	0.63259503	NA	Pdxk-ps	0	0	0	0	0	0	0	2.28262753
Prnd	0.28532844	0.08919132	0.18656246	0.47807753	0.63259503	NA	Prnd	0	0	0	0	0	0	0	2.28262753
Psors1c2	0.28532844	0.08919132	0.18656246	0.47807753	0.63259503	NA	Psors1c2	0	0	0	0	0	0	0	2.28262753
Ptx4	0.28532844	0.08919132	0.18656246	0.47807753	0.63259503	NA	Ptx4	0	0	0	0	0	0	0	2.28262753
Pyroxd2	0.28532844	0.08919132	0.18656246	0.47807753	0.63259503	NA	Pyroxd2	0	0	0	0	0	0	0	2.28262753
S100a14	0.28532844	0.08919132	0.18656246	0.47807753	0.63259503	NA	S100a14	0	0	0	0	0	0	0	2.28262753
Scarna17	0.28532844	0.08919132	0.18656246	0.47807753	0.63259503	NA	Scarna17	0	0	0	0	0	0	0	2.28262753
Shox2	0.28532844	0.08919132	0.18656246	0.47807753	0.63259503	NA	Shox2	0	0	0	0	0	0	0	2.28262753
Slc5a4a	0.28532844	0.08919132	0.18656246	0.47807753	0.63259503	NA	Slc5a4a	0	0	0	0	0	0	0	2.28262753
Spp2	0.28532844	0.08919132	0.18656246	0.47807753	0.63259503	NA	Spp2	0	0	0	0	0	0	0	2.28262753
Srrm4os	0.28532844	0.08919132	0.18656246	0.47807753	0.63259503	NA	Srrm4os	0	0	0	0	0	0	0	2.28262753
Tpsg1	0.28532844	0.08919132	0.18656246	0.47807753	0.63259503	NA	Tpsg1	0	0	0	0	0	0	0	2.28262753
Zp1	0.28532844	0.08919132	0.18656246	0.47807753	0.63259503	NA	Zp1	0	0	0	0	0	0	0	2.28262753
Ints7	18.2987705	-0.1771304	0.37050624	-0.4780768	0.63259553	0.99938111	Ints7	24.8657142	21.7211385	18.448584	12.4088401	28.3017992	4.7042181	15.3962222	20.5436478
9830166K06Rik	0.46413967	-0.1457003	0.30480122	-0.4780175	0.63263772	NA	9830166K06Ri	1.34409266	0.45252372	0.9542371	0	0	0	0.96226389	0
Man2c1os	2.35064766	0.18467445	0.38633463	0.47801682	0.63263823	NA	Man2c1os	0.67204633	0.45252372	0.9542371	6.20442003	4.99443516	0	0.96226389	4.56525506
Npr1	1.63124423	0.18041935	0.37747649	0.47796183	0.63267737	NA	Npr1	2.01613899	2.26261859	0.31807903	0	4.99443516	1.17605452	0	2.28262753
Actr1b	159.978857	0.14732652	0.30825725	0.47793368	0.63269741	0.99938111	Actr1b	135.753358	106.795598	120.233875	239.904241	191.453348	109.373071	136.641472	239.675891
Scml2	0.92772794	0.16281125	0.34080567	0.47772461	0.63284623	NA	Scml2	1.34409266	0	0.9542371	0	1.66481172	1.17605452	0	2.28262753
Cep44	1.33838492	-0.1813124	0.37954151	-0.4777143	0.63285357	NA	Cep44	0.67204633	2.26261859	2.86271131	0	1.66481172	0	0.96226389	2.28262753
Sgsm2	69.4337896	0.15623847	0.32714925	0.47757553	0.63295235	0.99938111	Sgsm2	63.8444012	46.6099431	50.8926455	99.2707205	118.201632	50.5703445	46.1886666	79.8919636
Prpf40a	21.4636111	0.19627667	0.41120515	0.47732055	0.63313388	0.99938111	Prpf40a	28.2259458	19.4585199	25.7644018	2.06814001	11.653682	48.2182355	31.7547083	4.56525506
Blzf1	9.38619793	0.19862977	0.41620299	0.47724255	0.63318942	0.99938111	Blzf1	6.04841696	14.0282353	12.0870033	0	3.32962344	19.9929269	17.32075	2.28262753
Rps6ka1	9.72588845	0.19511891	0.40884695	0.47724194	0.63318985	0.99938111	Rps6ka1	8.06455594	7.69290322	4.45310648	14.4769801	19.9777406	2.35210905	4.81131944	15.9783927
Map3k6	1.30851334	-0.1816653	0.38066063	-0.4772369	0.63319341	NA	Map3k6	2.01613899	2.26261859	0.31807903	2.06814001	1.66481172	1.17605452	0.96226389	0
Baiap2	125.995345	-0.1481184	0.31045345	-0.4771034	0.63328848	0.99938111	Baiap2	130.376988	153.405541	128.185851	119.952121	184.794101	69.3872169	71.2075278	150.653417
Ddah2	4.75181582	-0.1909866	0.40047231	-0.4769034	0.63343091	0.99938111	Ddah2	4.03227797	0.90504744	1.90847421	16.5451201	9.98887032	2.35210905	0	2.28262753
Btbd7	14.6847177	0.17313189	0.36304117	0.47689326	0.63343813	0.99938111	Btbd7	7.39250961	19.4585199	17.8124259	8.27256004	13.3184938	21.1689814	16.3584861	13.6957652
Pxk	29.5576278	0.1450984	0.30426878	0.47687575	0.6334506	0.99938111	Pxk	27.5538995	33.9392789	29.5813502	16.5451201	33.2962344	22.345036	29.8301805	43.3699231
Trit1	4.61976248	0.19483088	0.40859198	0.47683482	0.63347975	NA	Trit1	5.37637063	3.16766603	5.40734359	2.06814001	3.32962344	8.23238167	4.81131944	4.56525506
2900055J20Rik	14.1729613	0.17383093	0.36460099	0.4767703	0.63352569	0.99938111	2900055J20Ri	14.1129729	8.59795066	13.9954775	14.4769801	13.3184938	7.05632714	14.4339583	27.3915304
Pdp2	6.01926846	-0.1982343	0.4158138	-0.4767381	0.6335486	0.99938111	Pdp2	10.0806949	5.88280835	6.67965972	4.13628002	13.3184938	0	5.77358333	2.28262753
Xist	0.89583549	-0.1166467	0.24470447	-0.4766841	0.6335871	NA	Xist	0	0	0	6.20442003	0	0	0.96226389	0
Rnps1	54.1229339	-0.1276878	0.26791728	-0.4765942	0.63365109	0.99938111	Rnps1	58.4680306	44.7998482	52.1649617	74.4530404	43.2851047	41.1619083	61.5848889	57.0656883
Rcbtb2	13.2098639	0.17185451	0.36070689	0.4764381	0.6337623	0.99938111	Rcbtb2	18.8172972	14.0282353	8.90621297	6.20442003	14.9833055	18.8168724	12.5094306	11.4131377
Capn2	34.081761	-0.1520237	0.31911477	-0.476392	0.63379511	0.99938111	Capn2	42.3389187	43.8948007	35.3067728	22.7495401	16.6481172	47.042181	32.7169722	31.9567854
Rnd3	10.1855704	0.19311556	0.40544272	0.47630787	0.63385506	0.99938111	Rnd3	17.4732045	5.88280835	8.58813393	4.13628002	1.66481172	11.7605452	18.2830139	13.6957652
Acta1	6.43469336	-0.1874578	0.39362532	-0.4762341	0.63390762	0.99938111	Acta1	6.72046328	8.59795066	7.63389683	4.13628002	4.99443516	3.52816357	6.73584722	9.13051012
Marcks	195.025566	0.12134333	0.25483718	0.47616024	0.63396022	0.99938111	Marcks	145.834053	159.740873	180.986971	258.517501	208.101465	162.295524	179.943347	264.784794
Gmps	78.1954876	0.15570709	0.32707764	0.47605544	0.63403488	0.99938111	Gmps	84.6778374	70.5937002	80.1559167	57.9079203	61.5980337	130.542052	105.849028	34.239413
Gm14391	13.7796111	0.19782184	0.41575866	0.47580929	0.63421025	0.99938111	Gm14391	12.7688802	13.1231878	17.4943469	4.13628002	1.66481172	23.5210905	37.5282917	0
Hdac6	32.0159824	0.17460819	0.3670084	0.47576074	0.63424484	0.99938111	Hdac6	25.5377605	17.1959013	19.0847421	57.9079203	49.9443516	19.9929269	23.0943333	43.3699231

GeneID	Base mean	log2(FC)	StdErr	Wald-Stats	P-value	P-adj		GeneID	Normalised expression for Chow#1	Normalised expression for Chow#2	Normalised expression for Chow#3	Normalised expression for Chow#4	Normalised expression for HFD#1	Normalised expression for HFD#2	Normalised expression for HFD#3	Normalised expression for HFD#4
Phtf1	20.3119377	-0.1816055	0.38180797	-0.4756462	0.63432647	0.99938111		Phtf1	20.1613899	31.6766603	21.3112953	16.5451201	13.3184938	34.1055812	23.0943333	2.28262753
Ptdss1	50.9826264	-0.1410127	0.29653541	-0.475534	0.63440641	0.99938111		Ptdss1	33.6023164	61.5432258	55.345752	64.1123403	44.9499164	49.39429	32.7169722	66.1961984
Wdr45b	37.4227783	0.16127834	0.33919243	0.47547742	0.63444672	0.99938111		Wdr45b	36.9625481	32.129184	36.2610099	31.0221002	49.9443516	17.6408179	26.9433889	68.4788259
Cept1	21.6584452	-0.1754299	0.36906411	-0.4753371	0.63454671	0.99938111		Cept1	18.1452509	31.2241366	30.5355873	14.4769801	9.98887032	27.0492541	32.7169722	9.13051012
Ccdc9	12.7532985	0.17907784	0.37674796	0.47532531	0.63455512	0.99938111		Ccdc9	14.7850192	8.14542694	6.67965972	18.6132601	9.98887032	8.23238167	17.32075	18.2610202
Agpat4	23.0794082	0.18142259	0.38175211	0.47523664	0.63461831	0.99938111		Agpat4	13.4409266	29.8665654	20.0389792	18.6132601	23.3073641	24.697145	6.73584722	47.9351782
Mamld1	55.0350613	-0.1332263	0.28039615	-0.4751358	0.63469016	0.99938111		Mamld1	52.4196136	78.2866034	62.3434908	37.2265202	43.2851047	55.2745626	63.5094166	47.9351782
D19Bwg1357e	11.5719114	0.18572153	0.39092813	0.47507844	0.63473107	0.99938111		D19Bwg1357	16.8011582	13.1231878	7.95197586	4.13628002	13.3184938	17.6408179	17.32075	2.28262753
Pgm2l1	347.934178	0.16073857	0.33849411	0.47486371	0.63488412	0.99938111		Pgm2l1	378.362083	373.784592	396.326477	144.769801	312.984603	496.295009	505.188541	175.76232
Brd2	137.520883	0.11545293	0.24318089	0.47476153	0.63495696	0.99938111		Brd2	149.194285	118.108691	111.009583	146.837941	174.805231	116.429398	112.584875	171.197065
Plau	0.29177725	-0.1013653	0.21352966	-0.4747127	0.63499173	NA		Plau	2.01613899	0	0.31807903	0	0	0	0	0
Tmsb15b2	3.90838652	0.19764103	0.41636409	0.47468318	0.63501281	NA		Tmsb15b2	2.68818531	4.97776091	3.81694841	2.06814001	0	3.52816357	9.62263889	4.56525506
Rora	90.7097194	0.13947538	0.2938704	0.47461529	0.63506121	0.99938111		Rora	80.6455594	95.4825047	98.2864217	68.2486203	54.9387868	141.126543	111.622611	75.3267085
Cadm2	129.27377	-0.1838487	0.38745715	-0.4745007	0.63514288	0.99938111		Cadm2	151.88247	221.736622	159.993754	31.0221002	84.9053977	209.337705	125.094306	50.2178057
Rcor2	4.28537788	-0.1970408	0.41528602	-0.4744702	0.63516465	NA		Rcor2	11.4247876	3.62018975	4.45310648	0	6.65924688	2.35210905	5.77358333	0
Prkg2	7.141151	-0.1971654	0.41556121	-0.4744557	0.635175	0.99938111		Prkg2	12.7688802	10.8605693	3.49886938	6.20442003	1.66481172	10.5844907	11.5471667	0
Cenpu	0.96082256	0.16437392	0.34645668	0.47444293	0.63518408	NA		Cenpu	2.01613899	0.90504744	0	0	1.66481172	1.17605452	1.92452778	0
Abhd16a	27.1439618	-0.1516579	0.31987213	-0.4741205	0.63541397	0.99938111		Abhd16a	21.5054825	33.9392789	27.0367179	31.0221002	26.6369875	17.6408179	18.2830139	41.0872956
Twistnb	8.0823389	0.19158731	0.40436091	0.47380276	0.63564057	0.99938111		Twistnb	6.04841696	9.05047438	6.99773876	8.27256004	4.99443516	12.9365998	16.3584861	0
Rwdd3	3.66161991	0.19647086	0.41473914	0.47372154	0.63569849	NA		Rwdd3	5.37637063	2.26261859	2.86271131	2.06814001	4.99443516	2.35210905	4.81131944	4.56525506
4930549G23Rik	1.46611913	-0.1589491	0.33556112	-0.4736814	0.63572712	NA		4930549G23R	0.67204633	0.90504744	0	6.20442003	1.66481172	0	0	2.28262753
Arhgap20os	0.81371542	-0.1592301	0.33615847	-0.4736756	0.63573126	NA		Arhgap20os	1.34409266	0.90504744	1.90847421	0	0	2.35210905	0	0
Zfp213	7.69025753	0.19718986	0.41632159	0.47364794	0.63575099	0.99938111		Zfp213	4.03227797	4.97776091	3.81694841	14.4769801	23.3073641	1.17605452	2.88679167	6.84788259
Prps1l3	33.754655	0.15688195	0.33122973	0.47363486	0.63576031	0.99938111		Prps1l3	32.9302701	32.5817078	39.4418003	20.6814001	16.6481172	39.9858538	55.8113055	31.9567854
BC055111	0.32457176	0.10565501	0.22312312	0.47352785	0.63583664	NA		BC055111	0.67204633	0	0	0	0	0	1.92452778	0
3200001D21Rik	0.99181013	-0.1689195	0.35687985	-0.4733232	0.63598259	NA		3200001D21Ri	2.01613899	0.90504744	0.31807903	2.06814001	1.66481172	0	0.96226389	0
Ttl	40.1568622	0.14564494	0.30772484	0.47329601	0.63600201	0.99938111		Ttl	38.978687	36.6544212	30.2175083	45.4990802	66.5924688	24.697145	37.5282917	41.0872956
Dusp26	61.5604956	0.14860515	0.31400082	0.47326356	0.63602516	0.99938111		Dusp26	49.059382	37.5594687	43.5768277	105.475141	71.586904	55.2745626	68.3207361	61.6309433
Sytl1	0.33602316	-0.0892963	0.18870803	-0.4731983	0.63607173	NA		Sytl1	2.68818531	0	0	0	0	0	0	0
Kcnj3	48.2953478	-0.1419009	0.29988029	-0.4731917	0.63607639	0.99938111		Kcnj3	43.010965	76.4765085	49.3022503	33.0902402	54.9387868	50.5703445	35.6037639	43.3699231
Mylip	10.4631523	-0.1734826	0.36663509	-0.4731751	0.63608827	0.99938111		Mylip	8.06455594	9.95552182	12.7231614	14.4769801	8.3240586	5.88027262	10.5849028	13.6957652
Lrrtm1	35.431	0.16344846	0.34548133	0.47310361	0.63613926	0.99938111		Lrrtm1	31.5861774	47.9675142	36.897168	12.4088401	46.6147282	52.9224536	23.0943333	31.9567854
Kcnv1	35.4689242	-0.1750843	0.37016821	-0.4729858	0.63622331	0.99938111		Kcnv1	30.2420848	57.923036	50.2564875	14.4769801	38.2906696	49.39429	31.7547083	11.4131377
Foxc2	1.13638081	-0.1752008	0.37052616	-0.4728433	0.63632496	NA		Foxc2	2.68818531	0.90504744	1.90847421	0	1.66481172	0	1.92452778	0
Tbata	1.13638081	-0.1752008	0.37052616	-0.4728433	0.63632496	NA		Tbata	2.68818531	0.90504744	1.90847421	0	1.66481172	0	1.92452778	0
Ccdc33	0.46895571	0.13945377	0.29494405	0.47281431	0.63634566	NA		Ccdc33	0.67204633	0.45252372	0	0	1.66481172	0	0.96226389	0
Cfp	0.46895571	0.13945377	0.29494405	0.47281431	0.63634566	NA		Cfp	0.67204633	0.45252372	0	0	1.66481172	0	0.96226389	0
Acacb	2.10138817	-0.1909042	0.40381341	-0.4727535	0.63638903	NA		Acacb	1.34409266	2.26261859	1.27231614	6.20442003	1.66481172	1.17605452	2.88679167	0
2700089E24Rik	123.85745	0.14182124	0.30011579	0.47255509	0.63653063	0.99938111		2700089E24Ri	120.968339	129.874307	117.689243	99.2707205	68.2572805	136.422325	208.811264	109.566121
Timm10	10.6049634	0.17130524	0.36253767	0.47251707	0.63655776	0.99938111		Timm10	10.7527413	9.5029981	9.86045007	8.27256004	16.6481172	5.88027262	12.5094306	11.4131377
AA388235	3.79116902	-0.1957292	0.41426512	-0.4724732	0.63658907	NA		AA388235	3.36023164	5.43028463	4.77118552	4.13628002	3.32962344	3.52816357	5.77358333	0
C1galt1	7.15024326	0.19660512	0.41613861	0.47245105	0.63660487	0.99938111		C1galt1	2.68818531	12.6706641	8.90621297	0	4.99443516	14.1126543	11.5471667	2.28262753
Fopnl	22.2579218	-0.1752685	0.37101131	-0.4724073	0.63663609	0.99938111		Fopnl	22.1775288	24.8888045	26.4005599	24.8176801	9.98887032	14.1126543	44.2641389	11.4131377
Eif3k	42.6850415	0.15361022	0.32523039	0.47231201	0.6367041	0.99938111		Eif3k	35.6184554	38.9170398	43.5768277	41.3628002	36.6258578	19.9929269	68.3207361	57.0656883
Naa11	0.62666183	0.14112902	0.29893386	0.47210785	0.63684981	NA		Naa11	0	0	0.31807903	2.06814001	1.66481172	0	0.96226389	0
Card10	6.78400816	0.19543894	0.41420236	0.4718441	0.63703807	0.99938111		Card10	6.04841696	2.71514231	4.13502745	10.3407001	19.9777406	0	1.92452778	9.13051012
Uchl1	437.789159	-0.1544873	0.32749875	-0.4717188	0.63712752	0.99938111		Uchl1	419.356909	311.788842	326.985247	810.710884	476.136152	230.506687	367.584805	559.243745
Mlxip	22.7380091	-0.1410836	0.29918213	-0.4715644	0.63723775	0.99938111		Mlxip	23.5216215	27.6039469	21.9474534	22.7495401	18.3129289	17.6408179	25.0188611	25.1089028
Zfp423	24.4283329	-0.168171	0.35680144	-0.4713293	0.6374056	0.99938111		Zfp423	16.1291119	23.5312334	22.2655324	45.4990802	34.9610461	25.8731995	13.4716944	13.6957652
Dgki	50.3943193	0.12565536	0.2666129	0.47130264	0.63742463	0.99938111		Dgki	64.5164475	43.8948007	47.7118552	33.0902402	53.2739751	56.4506172	47.1509305	57.0656883
Dohh	47.3920991	0.19486103	0.41356237	0.47117687	0.63751443	0.99938111		Dohh	25.5377605	19.4585199	11.7689243	111.679561	81.5757743	14.1126543	25.981125	89.0224737
Med17	10.3056446	-0.1861375	0.39513005	-0.4710791	0.63758423	0.99938111		Med17	16.1291119	12.6706641	12.7231614	4.13628002	4.99443516	14.1126543	15.3962222	2.28262753
Arhgap1	57.6432305	-0.1466708	0.31142014	-0.4709739	0.63765937	0.99938111		Arhgap1	50.4034746	44.7998482	62.6615698	86.8618804	74.9165274	36.4576902	36.5660278	68.4788259
Ago1	96.3618968	0.13980162	0.29702291	0.47067621	0.63787198	0.99938111		Ago1	67.2046328	71.9512713	76.3389683	150.974221	114.872009	76.4435441	87.5660139	125.544514
Cntd1	12.5112368	-0.1690461	0.35925222	-0.4705499	0.63796216	0.99938111		Cntd1	7.39250961	14.9332827	13.6773985	18.6132601	13.3184938	8.23238167	12.5094306	11.4131377
Sgtb	79.6058439	0.17151611	0.36499802	0.46990969	0.63841954	0.99938111		Sgtb	106.855366	76.0239848	78.8836006	31.0221002	39.9554813	128.189943	127.981097	47.9351782
Msl2	66.4269305	0.15629685	0.33261651	0.46990105	0.63842572	0.99938111		Msl2	66.5325865	76.0239848	75.3847312	28.9539601	59.9332219	108.197016	79.8679027	36.5220405
Supt3	8.80182932	0.18091331	0.38501423	0.46988733	0.63843552	0.99938111		Supt3	6.72046328	5.88280835	6.36158069	14.4769801	4.99443516	10.5844907	7.69811111	13.6957652
Fam49b	104.931814	0.15761806	0.3354622	0.46985342	0.63845974	0.99938111		Fam49b	129.704941	91.4097912	121.188112	47.5672202	53.2739751	132.894161	160.698069	102.718239
Mki67	0.44151538	0.12546358	0.26707853	0.46976287	0.63852445	NA		Mki67	0	0.90504744	0	0	1.66481172	0	0.96226389	0
Tfap2c	0.44151538	0.12546358	0.26707853	0.46976287	0.63852445	NA		Tfap2c	0	0.90504744	0	0	1.66481172	0	0.96226389	0
Wdr52	0.47197319	-0.1300486	0.27688457	-0.4696851	0.63857999	NA		Wdr52	0	0.90504744	1.90847421	0	0	0	0.96226389	0
Hddc3	5.5981718	0.19291923	0.41099593	0.4693945	0.63878768	0.99938111		Hddc3	7.39250961	4.07271347	6.04350166	2.06814001	9.98887032	2.35210905	10.5849028	2.28262753
Gabrg1	25.8449719	-0.1902218	0.40526268	-0.4693791	0.63879869	0.99938111		Gabrg1	18.1452509	50.2301328	35.6248519	10.3407001	16.6481172	52.9224536	18.2830139	4.56525506
Chordc1	36.9341997	0.17879257	0.38097514	0.46930246	0.63885345	0.99938111		Chordc1	34.2743627	41.1796584	37.215247	22.7495401	13.3184938	78.7956531	51.96225	15.9783927
Polr2f	14.8816991	-0.1605024	0.34202302	-0.4692735	0.63887413	0.99938111		Polr2f	22.1775288	15.3858064	15.2677937	10.3407001	13.3184938	10.5844907	18.2830139	13.6957652
Zdhhc1	13.695664	0.1733471	0.36940133	0.46926496	0.63888026	0.99938111		Zdhhc1	8.73660227	12.2181404	12.0870033	16.5451201	14.9833055	14.1126543	5.77358333	25.1089028
Nthl1	0.76985596	0.15352682	0.32736701	0.46897463	0.63908777	NA		Nthl1	0.67204633	0	0.31807903	2.06814001	0	1.17605452	1.92452778	0
Mtmr4	79.001209	0.10976741	0.23410479	0.4688815	0.63915434	0.99938111		Mtmr4	81.9896521	72.403795	68.3869924	80.6574604	104.883138	82.3238167	68.3207361	73.044081
Pvr	2.69026254	-0.1933748	0.41250291	-0.468784	0.63922402	NA		Pvr	1.34409266	5.88280835	1.59039517	4.13628002	3.32962344	2.35210905	2.88679167	0
Rnf187	337.817318	0.1772468	0.37823772	0.46861216	0.63934688	0.99938111		Rnf187	256.049651	183.72463	204.20674	591.488043	389.565943	142.302597	236.716917	698.484025
B9d2	7.59926046	0.19330722	0.41275028	0.46833941	0.63954189	0.99938111		B9d2	3.36023164	7.69290322	3.49886938	12.4088401	11.653682	2.35210905	3.84905555	15.9783927
Chchd5	4.17082524	0.19466973	0.41581641	0.46816271	0.63966824	NA		Chchd5	1.34409266	5.88280835	3.18079035	4.13628002	0	2.35210905	9.62263889	6.84788259
Ttc33	30.3918073	0.15755847	0.33663914	0.46803372	0.63976047	0.99938111		Ttc33	32.2582238	21.7211385	30.5355873	31.0221002	18.3129289	32.9295267	55.8113055	20.5436478
Mnat1	4.56772641	-0.1947386	0.41634946	-0.4677287	0.63997861	NA		Mnat1	4.7043243	8.59795066	4.13502745	4.13628002	0	8.23238167	6.73584722	0
Ccdc39	4.074835	-0.1944179	0.415669	-0.4677228	0.6399828	NA		Ccdc39	6.04841696	6.33533207	6.36158069	0	1.66481172	3.52816357	8.660375	0
Ncoa5	32.1169599	0.14458672	0.30915887	0.46767773	0.64001507	0.99938111		Ncoa5	34.9464091	31.6766603	20.3570582	33.0902402	33.2962344	23.5210905	29.8301805	50.2178057
Ttc30a2	0.72050788	-0.1546052	0.33067779	-0.4675403	0.64011338	NA		Ttc30a2	1.34409266	0.90504744	1.59039517	0	0	0	1.92452778	0
Naa20	16.7469831	-0.1817393	0.38875577	-0.4674895	0.64014967	0.99938111		Naa20	24.1936678	24.4362808	21.6293743	2.06814001	8.3240586	18.8168724	23.0943333	11.4131377
Psmg1	8.397881	0.1916765	0.41003026	0.46746917	0.64016425	0.99938111		Psmg1	7.39250961	3.62018975	9.54237104	10.3407001	0	11.7605452	15.3962222	9.13051012
Sncb	620.467335	0.16806797	0.35957959	0.4674013	0.64021279	0.99938111		Sncb	534.276831	434.42277	415.411219	905.845324	680.907994	284.605195	418.584792	1289.68456
Ank2	199.012045	0.14541041	0.31119228	0.46726868	0.64030766	0.99938111		Ank2	194.893435	201.825579	183.849682	167.519341	341.286403	245.795396	158.773542	98.1529838
Kdm4c	15.2749528	0.18390015	0.3936407	0.46717768	0.64037276	0.99938111		Kdm4c	22.8495752	10.4080455	16.2220308	6.20442003	9.98887032	21.1689814	30.7924444	4.56525506
Rapgef1	103.535568	0.14569872	0.31188936	0.46714874	0.64039346	0.99938111		Rapgef1	90.054208	78.7391271	76.0208892	144.769801	121.531256	87.0280348	63.5094166	166.63181
Arid4b	33.9024307	0.17269073	0.36970012	0.46711029	0.64042097	0.99938111		Arid4b	40.994826	28.961518	30.8536663	24.8176801	23.3073641	75.2674895	35.6037639	11.4131377
Myh6	0.99279207	0.13737285	0.29411363	0.46707405	0.6404469	NA		Myh6	2.68818531	0	0	0	3.32962344	0	1.92452778	0
Tecta	0.62364439	-0.1329718	0.28482652	-0.4668518	0.64060589	NA		Tecta	2.68818531	0	0.63615807	0	1.66481172	0	0	0
Fgf23	0.37353339	0.10551436	0.22605843	0.46675705	0.64067371	NA		Fgf23	0	0	0.63615807	0	0	2.35210905	0	0
Plk1	0.37353339	0.10551436	0.22605843	0.46675705	0.64067371	NA		Plk1	0	0	0.63615807	0	0	2.35210905	0	0
Deb1	13.7285987	-0.1630552	0.34951934	-0.4665127	0.64084853	0.99938111		Deb1	16.8011582	15.3858064	13.0412404	14.4769801	4.99443516	14.1126543	17.32075	13.6957652
Frat1	14.9943239	-0.1861648	0.39917583	-0.4663729	0.64094862	0.99938111		Frat1	7.39250961	8.59795066	10.8146872	41.3628002	13.3184938	10.5844907	9.62263889	18.2610202
Letm2	4.25062352	-0.1934043	0.41470071	-0.4663707	0.64095018	NA		Letm2	4.03227797	7.2403795	5.40734359	2.06814001	0	5.88027262	4.81131944	4.56525506
Vegfc	1.07823903	0.17041411	0.36547052	0.46628689	0.64101016	NA		Vegfc	1.34409266	0.45252372	1.59039517	0	0	2.35210905	2.88679167	0
Agtrap	6.02256003	-0.1867916	0.40062294	-0.4662529	0.6410345	0.99938111		Agtrap	6.72046328	9.5029981	4.13502745	6.20442003	3.32962344	4.7042181	6.73584722	6.84788259
Dhx32	20.8775788	0.14533542	0.31188982	0.4659832	0.64122753	0.99938111		Dhx32	23.5216215	21.2686148	17.4943469	16.5451201	16.6481172	21.1689814	29.8301805	20.5436478
Ogdh	248.529631	0.11617656	0.24947394	0.46568616	0.64144016	0.99938111		Ogdh	247.313049	202.278102	208.659847	291.607741	327.967909	204.633487	195.339569	310.437344
Kcnh5	17.6531112	-0.1910892	0.41040186	-0.4656148	0.64149126	0.99938111		Kcnh5	20.1613899	28.961518	26.4005599	2.06814001	9.98887032	36.4576902	5.77358333	11.4131377
Dgat1	9.73512008	0.17854351	0.38345929	0.46561269	0.64149276	0.99938111		Dgat1	6.72046328	8.59795066	6.04350166	14.4769801	9.98887032	7.05632714	6.73584722	18.2610202
Guk1	28.5776393	0.15729177	0.33784656	0.46557163	0.64152215	0.99938111		Guk1	41.6668724	19.9110436	19.7209001	24.8176801	31.6314227	16.4647633	35.6037639	38.804668
Magee2	4.88569714	-0.1883553	0.40464856	-0.4654786	0.64158875	0.99938111		Magee2	7.39250961	4.52523719	5.40734359	4.13628002	3.32962344	5.88027262	3.84905555	4.56525506
Rnf152	34.6379882	0.15973801	0.34325881	0.46535734	0.64167558	0.99938111		Rnf152	25.5377605	18.5534725	24.4920857	64.1123403	44.9499164	30.5774176	34.6415	34.239413
Ist1	39.571035	-0.1186298	0.25494328	-0.4653183	0.6417035	0.99938111		Ist1	41.6668724	39.3695635	39.4418003	45.4990802	31.6314227	39.9858538	35.6037639	43.3699231
Paip1	61.7319803	-0.1621961	0.34864676	-0.4652161	0.64177669	0.99938111		Paip1	73.9250961	71.0462239	73.476257	47.5672202	21.6425524	70.5632714	99.1131805	36.5220405
Gm10125	1.17186258	-0.1616291	0.34751076	-0.4651052	0.6418561	NA		Gm10125	0	1.35757116	0.63615807	4.13628002	0	0	0.96226389	2.28262753
Emcn	1.22377836	-0.1498753	0.32226508	-0.4650684	0.64188246	NA		Emcn	0	5.88280835	0.31807903	0	1.66481172	0	1.92452778	0
Fam186b	1.12326673	0.16582067	0.35657122	0.46504222	0.64190122	NA		Fam186b	1.34409266	1.81009488	0.31807903	0	1.66481172	0	3.84905555	0
Gde1	90.6172506	-0.1462329	0.31448934	-0.4649852	0.64194206	0.99938111		Gde1	86.6939764	146.165161	104.011844	45.4990802	66.5924688	115.253343	80.8301666	79.8919636
4930479D17Rik	1.48871462	-0.1702748	0.36620554	-0.4649704	0.64195262	NA		4930479D17Ri	5.37637063	0.45252372	1.59039517	0	0	3.52816357	0.96226389	0
Lrrn3	34.7656377	-0.192807	0.41472589	-0.4649022	0.6420015	0.99938111		Lrrn3	32.2582238	67.8785578	47.7118552	8.27256004	9.98887032	77.6195986	29.8301805	4.56525506
Arl6ip6	9.30230329	0.16980071	0.36533329	0.46478302	0.64208685	0.99938111		Arl6ip6	7.39250961	8.14542694	7.63389683	12.4088401	8.3240586	11.7605452	9.62263889	9.13051012
Psmb4	60.7594186	0.1609767	0.34641232	0.46469681	0.64214859	0.99938111		Psmb4	43.010965	37.5594687	47.3937761	99.2707205	76.5813391	30.5774176	55.8113055	95.8703563
Tmem86a	7.33670893	0.19111577	0.41132962	0.46462924	0.64219699	0.99938111		Tmem86a	3.36023164	8.14542694	6.99773876	6.20442003	9.98887032	7.05632714	0.96226389	15.9783927

GeneID	Base mean	log2(FC)	StdErr	Wald-Stats	P-value	P-adj		GeneID	Normalised expression for Chow#1	Normalised expression for Chow#2	Normalised expression for Chow#3	Normalised expression for Chow#4	Normalised expression for HFD#1	Normalised expression for HFD#2	Normalised expression for HFD#3	Normalised expression for HFD#4
Zbed4	4.47919098	-0.1915497	0.41227466	-0.4646167	0.64220599	NA		Zbed4	5.37637063	5.43028463	6.99773876	2.06814001	6.65924688	3.52816357	5.77358333	0
Wasf1	247.719847	-0.1292013	0.27811298	-0.4645639	0.64224378	0.99938111		Wasf1	313.845635	277.39704	292.314633	155.110501	302.995733	159.943415	265.584833	214.566988
Chchd6	28.3322462	-0.1659815	0.35736764	-0.4644559	0.64232118	0.99938111		Chchd6	28.2259458	23.5312334	25.7644018	43.4309402	18.3129289	15.2887088	17.32075	54.7830607
Gad1os	1.11415117	0.16058916	0.34580994	0.46438561	0.6423715	NA		Gad1os	0	1.35757116	0.31807903	2.06814001	0	0	2.88679167	2.28262753
Sqrdl	1.54330381	-0.1808228	0.38938686	-0.4643784	0.64237669	NA		Sqrdl	2.01613899	3.16766603	1.90847421	0	3.32962344	0	1.92452778	0
Cpt2	3.05872822	-0.1925518	0.41468895	-0.4643283	0.64241256	NA		Cpt2	5.37637063	1.81009488	4.13502745	2.06814001	6.65924688	1.17605452	0.96226389	2.28262753
Fam83d	0.98951733	0.14859576	0.32015866	0.46413162	0.64255345	NA		Fam83d	0	1.35757116	0	2.06814001	0	3.52816357	0.96226389	0
Fbxo46	8.88885584	-0.1920519	0.413811	-0.4641053	0.64257229	0.99938111		Fbxo46	8.06455594	3.62018975	6.36158069	22.7495401	14.9833055	2.35210905	3.84905555	9.13051012
Slc25a20	6.45740986	0.1843431	0.3973494	0.463932	0.64269646	0.99938111		Slc25a20	6.72046328	6.33533207	6.99773876	2.06814001	13.3184938	5.88027262	5.77358333	4.56525506
Mta2	24.68229	0.15756144	0.33971074	0.4638106	0.64278345	0.99938111		Mta2	25.5377605	19.0059962	24.1740066	20.6814001	23.3073641	15.2887088	19.2452778	50.2178057
Cops3	33.0255143	0.14627094	0.31537426	0.46380113	0.64279023	0.99938111		Cops3	34.2743627	29.4140417	34.6706148	26.8858201	19.9777406	39.9858538	53.8867778	25.1089028
Slc25a53	6.62636733	-0.1802614	0.38879394	-0.4636425	0.64290393	0.99938111		Slc25a53	8.06455594	7.69290322	6.36158069	6.20442003	8.3240586	4.7042181	4.81131944	6.84788259
Nr1h2	42.5921578	-0.1646626	0.35523496	-0.4635315	0.64298345	0.99938111		Nr1h2	43.6830113	35.2968501	31.8079035	72.3849004	53.2739751	17.6408179	25.0188611	61.6309433
Gm14325	15.5785894	0.1632022	0.35214202	0.46345563	0.64303781	0.99938111		Gm14325	12.7688802	15.8383302	18.766663	0	1.66481172	29.4013631	46.1886666	0
Ccng1	39.3807203	-0.1820444	0.39283016	-0.4634177	0.64306499	0.99938111		Ccng1	50.4034746	55.2078937	52.4830407	14.4769801	9.98887032	38.8097993	73.1320555	20.5436478
Dok1	0.28114251	-0.0992618	0.2142243	-0.4633546	0.64311024	NA		Dok1	1.34409266	0.90504744	0	0	0	0	0	0
Trpc3	7.32387693	-0.1784266	0.38518907	-0.4632183	0.64320789	0.99938111		Trpc3	6.72046328	9.05047438	8.2700549	8.27256004	9.98887032	8.23238167	5.77358333	2.28262753
Cabp1	25.127207	-0.1624006	0.35065955	-0.463129	0.64327192	0.99938111		Cabp1	48.3873356	24.4362808	23.5378486	10.3407001	23.3073641	23.5210905	26.9433889	20.5436478
Sycp3	2.08934243	0.18544982	0.40047606	0.46307343	0.64331173	NA		Sycp3	0.67204633	1.81009488	2.86271131	2.06814001	0	3.52816357	5.77358333	0
Mrpl12	20.0478714	0.15200859	0.32828156	0.46304335	0.64333329	0.99938111		Mrpl12	20.1613899	13.1231878	14.9497146	28.9539601	18.3129289	17.6408179	22.1320694	25.1089028
Chchd1	9.17269841	-0.1916091	0.41390518	-0.4629299	0.64341465	0.99938111		Chchd1	13.4409266	12.6706641	14.6316356	0	1.66481172	12.9365998	13.4716944	4.56525506
Bloc1s4	7.67031733	0.18584824	0.40148156	0.46290604	0.64343172	0.99938111		Bloc1s4	4.03227797	8.59795066	5.72542262	8.27256004	9.98887032	5.88027262	2.88679167	15.9783927
Parp8	18.0501117	-0.1456595	0.31473628	-0.4627985	0.6435088	0.99938111		Parp8	16.1291119	19.0059962	21.6293743	20.6814001	14.9833055	19.9929269	18.2830139	13.6957652
Hist2h2be	4.32366447	-0.1915081	0.41389324	-0.4626993	0.64357989	NA		Hist2h2be	4.03227797	7.69290322	3.18079035	4.13628002	4.99443516	1.17605452	4.81131944	4.56525506
Ube2w	51.3120868	-0.1862796	0.40260588	-0.4626847	0.64359042	0.99938111		Ube2w	61.8282622	67.8785578	70.6135457	26.8858201	18.3129289	59.9787807	98.1509166	6.84788259
Gm11149	2.50749527	-0.1906682	0.41213741	-0.4626326	0.64362771	NA		Gm11149	1.34409266	5.43028463	2.86271131	2.06814001	3.32962344	1.17605452	3.84905555	0
Nnt	13.9070324	0.15519956	0.33559671	0.46245853	0.64375254	0.99938111		Nnt	11.4247876	15.8383302	10.4966081	14.4769801	13.3184938	15.2887088	14.4339583	15.9783927
Adar	220.734091	0.12668127	0.27395749	0.46241214	0.64378579	0.99938111		Adar	170.699767	162.003491	186.712393	322.629842	253.051381	246.97145	163.584861	260.219539
0610038B21Rik	0.57360047	-0.1086945	0.23506153	-0.4624088	0.64378819	NA		0610038B21Ri	0	0.45252372	0	4.13628002	0	0	0	0
Fcer2a	0.57360047	-0.1086945	0.23506153	-0.4624088	0.64378819	NA		Fcer2a	0	0.45252372	0	4.13628002	0	0	0	0
Gm20748	0.57360047	-0.1086945	0.23506153	-0.4624088	0.64378819	NA		Gm20748	0	0.45252372	0	4.13628002	0	0	0	0
Gm6756	0.57360047	-0.1086945	0.23506153	-0.4624088	0.64378819	NA		Gm6756	0	0.45252372	0	4.13628002	0	0	0	0
Hyal3	0.57360047	-0.1086945	0.23506153	-0.4624088	0.64378819	NA		Hyal3	0	0.45252372	0	4.13628002	0	0	0	0
Pcp2	0.57360047	-0.1086945	0.23506153	-0.4624088	0.64378819	NA		Pcp2	0	0.45252372	0	4.13628002	0	0	0	0
Rspo4	0.57360047	-0.1086945	0.23506153	-0.4624088	0.64378819	NA		Rspo4	0	0.45252372	0	4.13628002	0	0	0	0
Kdm6b	37.2655052	-0.1663849	0.35985851	-0.4623619	0.64382183	0.99938111		Kdm6b	27.5538995	37.106945	28.3090341	68.2486203	54.9387868	16.4647633	22.1320694	43.3699231
Ran	70.5409886	0.12930406	0.27966619	0.46235143	0.64382932	0.99938111		Ran	59.8121232	61.9957495	67.1146763	82.7256004	56.6035985	72.9153805	110.660347	52.5004332
Synj2bp	64.4065909	0.13332531	0.28860759	0.4619605	0.64410965	0.99938111		Synj2bp	47.7152893	52.9452751	53.1191988	95.1344405	51.6091633	85.8519803	74.0943194	54.7830607
Gm6710	15.6992927	0.19171498	0.41517176	0.46177268	0.64424434	0.99938111		Gm6710	15.4570656	14.480759	20.0389792	4.13628002	1.66481172	29.4013631	40.4150833	0
Dmxl1	74.1591262	0.1634071	0.35400176	0.46159967	0.64436843	0.99938111		Dmxl1	58.4680306	72.8563187	98.2864217	45.4990802	53.2739751	129.365998	105.849028	29.6741579
Gm5820	1.06591948	0.15821325	0.34291776	0.46137375	0.64453048	NA		Gm5820	2.01613899	0	1.27231614	0	0	2.35210905	2.88679167	0
Hsd11b1	1.79554274	-0.1856185	0.40233687	-0.461351	0.64454683	NA		Hsd11b1	4.7043243	1.81009488	1.90847421	0	1.66481172	2.35210905	1.92452778	0
Nsmce2	12.1205309	0.16660054	0.3611645	0.46128716	0.6445926	0.99938111		Nsmce2	10.0806949	12.2181404	8.2700549	16.5451201	8.3240586	14.1126543	18.2830139	9.13051012
Prkrip1	6.32214735	0.18859368	0.40886865	0.46125737	0.64461397	0.99938111		Prkrip1	4.03227797	2.26261859	5.40734359	12.4088401	3.32962344	8.23238167	5.77358333	9.13051012
Zfp831	28.6028764	0.14789507	0.32081174	0.46100266	0.6447967	0.99938111		Zfp831	28.2259458	24.4362808	24.4920857	31.0221002	41.620293	41.1619083	17.32075	20.5436478
Ube2n	123.827909	0.13724264	0.29783811	0.46079609	0.64494491	0.99938111		Ube2n	122.312432	109.963264	132.002799	105.475141	94.8942681	147.006816	205.924472	73.044081
Erich1	3.883765	-0.1916776	0.416022	-0.4607392	0.64498574	NA		Erich1	8.73660227	1.81009488	3.81694841	4.13628002	1.66481172	7.05632714	3.84905555	0
Rps2	27.5775559	0.17998482	0.39065137	0.46073004	0.6449923	0.99938111		Rps2	16.1291119	9.5029981	17.1762679	59.9760603	26.6369875	17.6408179	27.9056528	45.6525506
Lrrn1	49.426092	-0.1716017	0.37253383	-0.4606339	0.64506131	0.99938111		Lrrn1	43.6830113	96.3875521	50.2564875	22.7495401	26.6369875	85.8519803	35.6037639	34.239413
Atp10d	1.55730764	0.16632438	0.36126259	0.46039746	0.64523096	NA		Atp10d	0	0.90504744	1.59039517	2.06814001	3.32962344	0	0	4.56525506
Phc2	57.465645	-0.1662308	0.36123848	-0.4601692	0.64539481	0.99938111		Phc2	60.4841696	37.5594687	50.2564875	99.2707205	79.9109626	30.5774176	24.0565972	77.6093361
Rbm14-rbm4	17.6851925	0.16153588	0.35105461	0.46014458	0.64541245	0.99938111		Rbm14-rbm4	12.0968339	11.313093	14.9497146	28.9539601	19.9777406	11.7605452	17.32075	25.1089028
Erc2	123.098323	0.17703606	0.38486243	0.45999828	0.64551745	0.99938111		Erc2	165.323397	121.276357	130.730483	31.0221002	98.2238915	218.746141	176.094292	43.3699231
Vcpip1	92.1449964	0.15159557	0.32961693	0.45991439	0.64557767	0.99938111		Vcpip1	104.839227	102.27036	95.4237104	41.3628002	83.240586	148.18287	111.622611	50.2178057
Ache	54.5678496	-0.1601996	0.34847697	-0.4597136	0.64572181	0.99938111		Ache	36.9625481	67.8785578	40.7141164	88.9300204	73.2517157	50.5703445	21.1698055	57.0656883
Rpn1	65.7691917	-0.1579323	0.34381176	-0.4593567	0.645978	0.99938111		Rpn1	48.3873356	119.918786	66.7965972	45.4990802	46.6147282	103.492798	45.2264028	50.2178057
Sf3b4	40.4015728	0.17107236	0.37259616	0.45913614	0.64613641	0.99938111		Sf3b4	21.5054825	30.7716129	23.2197695	74.4530404	51.6091633	25.8731995	25.0188611	70.7614535
Dnttip2	13.5225912	-0.1870789	0.4074845	-0.4591067	0.64615751	0.99938111		Dnttip2	16.1291119	19.0059962	22.5836114	2.06814001	6.65924688	21.1689814	18.2830139	2.28262753
2210404O09Rik	1.84500764	0.18330537	0.39927982	0.45909001	0.64616953	NA		2210404O09R	0.67204633	2.71514231	2.54463228	0	1.66481172	2.35210905	4.81131944	0
Cacnb3	142.712349	0.13485831	0.29387135	0.45890256	0.64630414	0.99938111		Cacnb3	153.226563	145.260114	119.915796	117.883981	198.112595	88.2040893	106.811292	212.28436
Adck5	4.74489446	-0.1909698	0.41637075	-0.4586534	0.64648312	0.99938111		Adck5	5.37637063	1.81009488	2.86271131	12.4088401	6.65924688	2.35210905	1.92452778	4.56525506
Stx7	106.654414	0.13394201	0.29215287	0.45846548	0.64661805	0.99938111		Stx7	122.984478	103.175408	105.28416	72.3849004	74.9165274	109.373071	176.094292	89.0224737
N4bp2l2	20.9541626	0.16513845	0.36021955	0.45843835	0.64663754	0.99938111		N4bp2l2	20.1613899	22.6261859	24.1740066	10.3407001	9.98887032	25.8731995	38.4905555	15.9783927
Gm12216	0.56842048	0.13928926	0.30386567	0.45839091	0.64667162	NA		Gm12216	0	0.45252372	0.63615807	0	0	1.17605452	0	2.28262753
Hivep3	49.9045077	0.13403782	0.29246675	0.45830106	0.64673617	0.99938111		Hivep3	43.6830113	54.75537	41.0321955	47.5672202	76.5813391	36.4576902	37.5282917	61.6309433
Dnajb14	23.6780791	0.18045005	0.39375181	0.45828373	0.64674861	0.99938111		Dnajb14	22.1775288	33.4867552	23.2197695	6.20442003	9.98887032	55.2745626	23.0943333	15.9783927
Zfp68	31.7725829	0.15877953	0.34650842	0.45822705	0.64678933	0.99938111		Zfp68	35.6184554	29.4140417	33.0802196	20.6814001	14.9833055	51.7463991	48.1131944	20.5436478
Wdr55	7.59076255	-0.1897631	0.41416915	-0.4581779	0.64682465	0.99938111		Wdr55	12.0968339	4.97776091	6.36158069	12.4088401	4.99443516	3.52816357	16.3584861	0
Fbln7	0.73524216	0.16063488	0.35068822	0.45805609	0.64691214	NA		Fbln7	0.67204633	0.45252372	0.9542371	0	1.66481172	1.17605452	0.96226389	0
Crtap	2.27433777	-0.1871686	0.40865019	-0.4580167	0.64694043	NA		Crtap	4.03227797	1.81009488	0.9542371	4.13628002	1.66481172	2.35210905	0.96226389	2.28262753
Cad	17.4956635	0.19041108	0.41573094	0.45801517	0.64694154	0.99938111		Cad	8.06455594	7.2403795	9.86045007	37.2265202	39.9554813	10.5844907	1.92452778	25.1089028
Ints2	10.9799503	0.1626435	0.35525631	0.45782015	0.64708166	0.99938111		Ints2	7.39250961	9.5029981	10.4966081	14.4769801	13.3184938	12.9365998	10.5849028	9.13051012
Sbf2	47.0450123	-0.1343704	0.29361492	-0.4576416	0.64720992	0.99938111		Sbf2	51.7475673	53.8503226	62.9796488	28.9539601	39.9554813	48.2182355	58.6980972	31.9567854
Ppp1r17	2.25600722	0.17524563	0.38294618	0.45762469	0.64722211	NA		Ppp1r17	0.67204633	0.90504744	0.31807903	6.20442003	0	1.17605452	1.92452778	6.84788259
Zswim1	32.9256778	-0.1366981	0.29872676	-0.4576024	0.64723815	0.99938111		Zswim1	36.9625481	31.2241366	32.7621406	37.2265202	34.9610461	16.4647633	32.7169722	41.0872956
Mar-01	30.9710721	0.17077479	0.37320447	0.45759042	0.64724673	0.99938111		Mar-01	34.9464091	38.4645161	33.0802196	6.20442003	19.9777406	36.4576902	55.8113055	22.8262753
Ccnf	2.60901012	0.18914282	0.41343672	0.45748915	0.6473195	NA		Ccnf	2.01613899	2.71514231	2.86271131	0	3.32962344	1.17605452	1.92452778	6.84788259
Klhl14	3.5235064	0.19040741	0.41629029	0.45739096	0.64739006	NA		Klhl14	3.36023164	3.16766603	4.13502745	2.06814001	1.66481172	7.05632714	6.73584722	0
Lmtk2	131.486879	0.16339584	0.35725618	0.45736324	0.64740998	0.99938111		Lmtk2	102.823088	184.177154	132.002799	66.1804803	131.520126	263.436213	64.4716805	107.283494
Pold3	11.2965041	-0.1696198	0.37089103	-0.4573305	0.64743351	0.99938111		Pold3	16.8011582	14.480759	11.4508452	4.13628002	14.9833055	9.40843619	7.69811111	11.4131377
Snap91	409.259948	0.07427342	0.16241244	0.45731362	0.64744564	0.99938111		Snap91	388.442778	406.818823	385.829869	411.559862	461.152847	410.443029	382.981028	426.851348
Ptprv	0.4177793	0.12153582	0.26577178	0.45729393	0.64745979	NA		Ptprv	0.67204633	0	0.31807903	0	0	2.35210905	0	0
Trp63	0.4177793	0.12153582	0.26577178	0.45729393	0.64745979	NA		Trp63	0.67204633	0	0.31807903	0	0	2.35210905	0	0
Pilra	0.92255932	-0.1127007	0.24645704	-0.4572835	0.64746727	NA		Pilra	0	0	0	6.20442003	0	1.17605452	0	0
1700010I14Rik	0.82513195	-0.1624694	0.35541036	-0.4571318	0.64757631	NA		1700010I14Ri	1.34409266	1.35757116	1.27231614	0	1.66481172	0	0.96226389	0
Trp53inp2	169.453071	-0.1494908	0.32702775	-0.4571197	0.64758503	0.99938111		Trp53inp2	161.291119	195.94277	146.634435	215.086561	171.475607	77.6195986	106.811292	280.763186
Ing2	13.9322363	0.15510235	0.33936122	0.45704207	0.64764081	0.99938111		Ing2	16.1291119	10.8605693	14.3135566	10.3407001	11.653682	12.9365998	19.2452778	15.9783927
Cmas	56.3390598	-0.1502834	0.32891164	-0.4569113	0.64773482	0.99938111		Cmas	60.4841696	62.4482732	59.7988585	59.9760603	34.9610461	58.8027262	91.4150694	22.8262753
Rps6kb2	3.82610856	-0.1901258	0.41626286	-0.4567446	0.64785466	NA		Rps6kb2	2.01613899	6.78785578	5.72542262	2.06814001	0	2.35210905	4.81131944	6.84788259
Sema3c	11.7549117	0.19000174	0.41601879	0.45671433	0.64787639	0.99938111		Sema3c	8.06455594	15.3858064	16.8581888	0	8.3240586	34.1055812	6.73584722	4.56525506
Slc25a10	17.9490149	-0.1800626	0.39437283	-0.4565796	0.64797326	0.99938111		Slc25a10	12.7688802	10.8605693	11.4508452	45.4990802	24.9721758	8.23238167	11.5471667	18.2610202
Nkain2	44.7178078	-0.1709031	0.3743148	-0.4565759	0.6479759	0.99938111		Nkain2	49.059382	66.9735104	55.345752	22.7495401	19.9777406	56.4506172	71.2075278	15.9783927
Amigo1	81.2628498	-0.142579	0.31230598	-0.4565364	0.64800431	0.99938111		Amigo1	63.8444012	112.225882	79.8378377	86.8618804	78.2461509	97.6125255	35.6037639	95.8703563
Nr2e1	14.0697752	-0.1701074	0.37263352	-0.4565005	0.64803013	0.99938111		Nr2e1	25.5377605	17.648425	9.86045007	6.20442003	14.9833055	11.7605452	10.5849028	15.9783927
Shisa7	113.971653	0.15740648	0.34495377	0.45631183	0.64816576	0.99938111		Shisa7	67.2046328	158.835825	118.961559	76.5211804	161.486737	144.654706	47.1509305	136.957652
Idh1	21.7821092	-0.1583951	0.34716116	-0.4562581	0.64820439	0.99938111		Idh1	30.2420848	27.1514231	23.2197695	12.4088401	11.653682	17.6408179	33.6792361	18.2610202
1110004E09Rik	13.5125198	0.17378642	0.38094645	0.45619644	0.64824872	0.99938111		1110004E09Ri	17.4732045	5.88280835	16.2220308	10.3407001	9.98887032	11.7605452	25.0188611	11.4131377
Cenpo	5.69392307	-0.1838099	0.40297929	-0.4561274	0.6482984	0.99938111		Cenpo	6.72046328	4.97776091	6.67965972	6.20442003	9.98887032	3.52816357	2.88679167	4.56525506
Slc14a2	0.39908876	-0.1169808	0.25660002	-0.4558878	0.64847069	NA		Slc14a2	0.67204633	0.45252372	0	2.06814001	0	0	0	0
Wnt16	0.39908876	-0.1169808	0.25660002	-0.4558878	0.64847069	NA		Wnt16	0.67204633	0.45252372	0	2.06814001	0	0	0	0
Tuba1b	215.155187	0.13863304	0.30409739	0.45588368	0.64847363	0.99938111		Tuba1b	169.355675	157.930778	185.121998	299.880301	269.699499	132.894161	179.943347	326.415737
Ttc27	12.4694418	0.17294879	0.379461	0.45577487	0.64855188	0.99938111		Ttc27	9.4086486	9.95552182	11.7689243	14.4769801	6.65924688	21.1689814	5.77358333	20.5436478
Taf9	31.8274995	-0.1896143	0.41604537	-0.455754	0.6485669	0.99938111		Taf9	43.6830113	34.8443264	56.2999891	10.3407001	4.99443516	39.9858538	64.4716805	0
Ormdl1	4.25239931	0.18902973	0.41477757	0.4557376	0.64857868	NA		Ormdl1	3.36023164	3.16766603	6.36158069	2.06814001	6.65924688	4.7042181	7.69811111	0
Smarca5	21.8164012	0.1394492	0.30606994	0.4556122	0.64866887	0.99938111		Smarca5	20.8334362	23.9837571	19.0847421	18.6132601	14.9833055	28.2253086	25.981125	22.8262753
Trip10	0.86417533	-0.1605277	0.35236226	-0.4555757	0.64869515	NA		Trip10	0.67204633	1.81009488	1.59039517	0	1.66481172	1.17605452	0	0
Pank1	28.5560769	0.12858137	0.28229916	0.45547911	0.6487646	0.99938111		Pank1	29.5700384	25.3413283	29.8994292	22.7495401	26.6369875	37.6337448	26.9433889	29.6741579
Zbed5	28.4393947	0.18654927	0.40981872	0.45519947	0.64896574	0.99938111		Zbed5	14.7850192	13.1231878	14.3135566	59.9760603	24.9721758	10.5844907	14.4339583	75.3267085
C920021L13Rik	0.48637747	-0.1381786	0.30357712	-0.455168	0.64898841	NA		C920021L13Ri	0.67204633	0.45252372	1.59039517	0	0	1.17605452	0	0
Ccdc116	0.48637747	-0.1381786	0.30357712	-0.455168	0.64898841	NA		Ccdc116	0.67204633	0.45252372	1.59039517	0	0	1.17605452	0	0
AI414108	9.78382928	-0.1767603	0.38839213	-0.4551077	0.64903174	0.99938111		AI414108	4.7043243	13.1231878	9.54237104	16.5451201	6.65924688	14.1126543	6.73584722	6.84788259
R3hcc1	15.2953505	0.16687086	0.36671731	0.45503949	0.64908083	0.99938111		R3hcc1	14.1129729	10.4080455	8.90621297	24.8176801	8.3240586	15.2887088	15.3962222	25.1089028

GeneID	Base mean	log2(FC)	StdErr	Wald-Stats	P-value	P-adj	GeneID	Normalised expression for Chow#1	Normalised expression for Chow#2	Normalised expression for Chow#3	Normalised expression for Chow#4	Normalised expression for HFD#1	Normalised expression for HFD#2	Normalised expression for HFD#3	Normalised expression for HFD#4
Mgat4b	28.6686141	-0.1409142	0.3096828	-0.4550274	0.64908954	0.99938111	Mgat4b	31.5861774	38.9170398	20.9932163	28.9539601	34.9610461	21.1689814	23.0943333	29.6741579
Sdhc	83.4586925	-0.1662861	0.36550046	-0.4549547	0.64914186	0.99938111	Sdhc	61.1562159	73.3088425	68.7050715	157.178641	68.2572805	27.0492541	75.0565833	136.957652
Pex14	34.4727644	-0.1608933	0.35366423	-0.4549323	0.64915797	0.99938111	Pex14	23.5216215	33.4867552	27.0367179	66.1804803	54.9387868	24.697145	23.0943333	22.8262753
Rmdn2	3.21201465	0.18881292	0.41509532	0.45486642	0.64920535	NA	Rmdn2	3.36023164	3.62018975	3.81694841	0	0	5.88027262	6.73584722	2.28262753
C130074G19Rik	9.66849412	0.17270208	0.3796807	0.45486135	0.64920899	0.99938111	C130074G19R	9.4086486	9.5029981	11.1327662	4.13628002	11.653682	4.7042181	15.3962222	11.4131377
Polg	20.5161429	0.14473518	0.3182418	0.45479628	0.64925581	0.99938111	Polg	24.1936678	17.1959013	16.2220308	18.6132601	18.3129289	16.4647633	21.1698055	31.9567854
Neurl2	2.93883985	0.18777603	0.41301892	0.45464267	0.64936633	NA	Neurl2	2.01613899	1.35757116	1.27231614	6.20442003	4.99443516	1.17605452	1.92452778	4.56525506
B830017H08Rik	2.56739957	0.18791851	0.41338207	0.45458796	0.6494057	NA	B830017H08R	2.01613899	0.90504744	2.22655324	4.13628002	1.66481172	1.17605452	3.84905555	4.56525506
Tmem45a	0.62446939	0.15089408	0.33207227	0.45440132	0.64954	NA	Tmem45a	0.67204633	0.90504744	0.31807903	0	0	1.17605452	1.92452778	0
Itgad	1.05587836	-0.1272154	0.28003911	-0.4542772	0.64962936	NA	Itgad	0	0	0.31807903	6.20442003	0	0	1.92452778	0
Trim13	3.23592441	-0.1858683	0.40928617	-0.454128	0.64973671	NA	Trim13	2.68818531	8.14542694	3.18079035	0	0	1.17605452	3.84905555	6.84788259
Zfp418	3.00964543	-0.188785	0.41579231	-0.4540367	0.64980238	NA	Zfp418	5.37637063	4.07271347	4.13502745	0	3.32962344	2.35210905	4.81131944	0
Eif2b4	16.2502011	0.16736792	0.3687551	0.45387284	0.64992036	0.99938111	Eif2b4	12.0968339	13.5757116	18.448584	14.4769801	23.3073641	4.7042181	18.2830139	25.1089028
Nupl2	7.01744007	-0.1875178	0.41340712	-0.4535912	0.65012312	0.99938111	Nupl2	8.73660227	9.95552182	8.90621297	2.06814001	8.3240586	0	6.73584722	11.4131377
Col5a2	0.55195815	-0.1418689	0.3127752	-0.4535812	0.65013032	NA	Col5a2	1.34409266	0.45252372	0.9542371	0	1.66481172	0	0	0
Tbl2	11.3766256	-0.1710881	0.37720915	-0.453563	0.65014338	0.99938111	Tbl2	8.73660227	15.8383302	13.6773985	10.3407001	16.6481172	14.1126543	4.81131944	6.84788259
Cdh11	21.6643854	-0.1740737	0.38380663	-0.4535454	0.65015604	0.99938111	Cdh11	20.1613899	40.274611	21.6293743	12.4088401	16.6481172	36.4576902	21.1698055	4.56525506
Gm9866	23.9633809	0.14985109	0.33049064	0.45342006	0.6502463	0.99938111	Gm9866	26.8818531	16.2908539	29.8994292	16.5451201	16.6481172	34.1055812	30.7924444	20.5436478
Ppp1r21	43.3626193	-0.1366671	0.30149245	-0.4533019	0.65033137	0.99938111	Ppp1r21	53.0916599	46.6099431	49.3022503	35.1583802	24.9721758	49.39429	58.6980972	29.6741579
1700020L24Rik	0.55812899	-0.1389057	0.30647507	-0.4532365	0.65037847	NA	1700020L24Ri	0.67204633	1.81009488	0.31807903	0	1.66481172	0	0	0
Ccdc127	59.9022379	0.16258531	0.358741	0.45321085	0.65039692	0.99938111	Ccdc127	47.7152893	84.1694117	66.1604392	22.7495401	33.2962344	99.9646345	81.7924305	43.3699231
Zcchc4	3.43951286	-0.1881134	0.41508105	-0.4531968	0.65040701	NA	Zcchc4	4.03227797	2.26261859	2.22655324	8.27256004	6.65924688	1.17605452	2.88679167	0
Zfp772	7.38811106	-0.1775664	0.3918474	-0.453152	0.65043928	0.99938111	Zfp772	7.39250961	9.5029981	8.2700549	8.27256004	4.99443516	5.88027262	12.5094306	2.28262753
Cltc	512.92745	-0.1152504	0.25433542	-0.4531432	0.65044563	0.99938111	Cltc	600.137371	505.468994	652.698179	384.674042	456.158411	615.076516	551.377208	337.828875
Enox2	8.16180743	-0.1789228	0.39497161	-0.4530016	0.65054759	0.99938111	Enox2	14.1129729	7.69290322	9.86045007	4.13628002	3.32962344	12.9365998	8.660375	4.56525506
Dynlt1f	4.12855465	-0.1884611	0.41618288	-0.4528325	0.65066935	NA	Dynlt1f	4.7043243	2.71514231	3.18079035	8.27256004	0	1.17605452	3.84905555	9.13051012
Ccdc137	10.4060403	0.1756789	0.3880055	0.45277425	0.65071131	0.99938111	Ccdc137	4.03227797	9.05047438	8.2700549	18.6132601	9.98887032	16.4647633	7.69811111	9.13051012
Lnx2	5.34280294	0.18641205	0.41172871	0.45275455	0.65072549	0.99938111	Lnx2	4.03227797	8.59795066	4.13502745	0	13.3184938	3.52816357	0	9.13051012
S1pr3	5.16238405	-0.1839514	0.40637705	-0.4526618	0.65079228	0.99938111	S1pr3	4.7043243	8.59795066	6.99773876	2.06814001	4.99443516	5.88027262	5.77358333	2.28262753
Yif1a	12.4851688	0.18484886	0.4084407	0.45257208	0.65085691	0.99938111	Yif1a	4.03227797	12.6706641	7.31581779	20.6814001	13.3184938	3.52816357	8.660375	29.6741579
Tesk1	123.258576	0.1411363	0.31189938	0.45250588	0.65090458	0.99938111	Tesk1	103.495135	95.4825047	88.1078926	177.860041	149.833055	96.436471	80.8301666	194.02334
4833411C07Rik	0.2368966	-0.1095166	0.24205076	-0.4524528	0.6509428	NA	4833411C07Ri	0.67204633	0.90504744	0.31807903	0	0	0	0	0
4930529M08Rik	0.2368966	-0.1095166	0.24205076	-0.4524528	0.6509428	NA	4930529M08R	0.67204633	0.90504744	0.31807903	0	0	0	0	0
A730020M07Rik	0.2368966	-0.1095166	0.24205076	-0.4524528	0.6509428	NA	A730020M07R	0.67204633	0.90504744	0.31807903	0	0	0	0	0
Cbr2	0.2368966	-0.1095166	0.24205076	-0.4524528	0.6509428	NA	Cbr2	0.67204633	0.90504744	0.31807903	0	0	0	0	0
Cxcl10	0.2368966	-0.1095166	0.24205076	-0.4524528	0.6509428	NA	Cxcl10	0.67204633	0.90504744	0.31807903	0	0	0	0	0
Gm14393	0.2368966	-0.1095166	0.24205076	-0.4524528	0.6509428	NA	Gm14393	0.67204633	0.90504744	0.31807903	0	0	0	0	0
Slc44a4	0.2368966	-0.1095166	0.24205076	-0.4524528	0.6509428	NA	Slc44a4	0.67204633	0.90504744	0.31807903	0	0	0	0	0
Ttc22	0.2368966	-0.1095166	0.24205076	-0.4524528	0.6509428	NA	Ttc22	0.67204633	0.90504744	0.31807903	0	0	0	0	0
Neil2	0.38042073	0.11738524	0.25966504	0.45206409	0.65122281	NA	Neil2	0	0.90504744	0	0	0	1.17605452	0.96226389	0
Mbd6	27.7908952	0.17939506	0.39690726	0.45198232	0.65128172	0.99938111	Mbd6	16.1291119	27.6039469	13.6773985	43.4309402	53.2739751	12.9365998	9.62263889	45.6525506
Idh2	13.5647962	0.16173904	0.357844	0.45198197	0.65128197	0.99938111	Idh2	14.7850192	13.5757116	9.86045007	10.3407001	18.3129289	8.23238167	10.5849028	22.8262753
Ncapd3	12.2219603	0.15711395	0.34775078	0.45180043	0.65141276	0.99938111	Ncapd3	13.4409266	13.1231878	12.0870033	6.20442003	13.3184938	14.1126543	16.3584861	9.13051012
6430573F11Rik	7.15459551	-0.1847597	0.40898947	-0.4517469	0.65145131	0.99938111	6430573F11Ri	16.8011582	6.78785578	6.04350166	2.06814001	3.32962344	7.05632714	10.5849028	4.56525506
Ikbip	3.94363101	-0.1879798	0.4163587	-0.4514853	0.65163981	NA	Ikbip	5.37637063	7.69290322	2.54463228	2.06814001	0	3.52816357	5.77358333	4.56525506
Pik3r1	101.93174	-0.1293806	0.28658746	-0.4514526	0.65166341	0.99938111	Pik3r1	99.4628566	136.662163	118.325401	74.4530404	103.218327	121.133616	109.698083	52.5004332
Tmem132e	16.6529657	-0.1798838	0.39851794	-0.451382	0.65171427	0.99938111	Tmem132e	11.4247876	28.0564706	13.9954775	18.6132601	23.3073641	17.6408179	1.92452778	18.2610202
Gm17296	0.98827028	-0.1538738	0.34101099	-0.4512282	0.65182505	NA	Gm17296	0	0.45252372	2.54463228	2.06814001	1.66481172	1.17605452	0	0
Yipf4	40.5396021	-0.1390303	0.30825696	-0.4510209	0.65197446	0.99938111	Yipf4	27.5538995	55.6604174	53.7553568	33.0902402	26.6369875	39.9858538	44.2641389	43.3699231
Sema3f	3.30007506	-0.1870936	0.41486752	-0.4509719	0.65200977	NA	Sema3f	2.68818531	2.71514231	3.49886938	6.20442003	6.65924688	2.35210905	0	2.28262753
Atrn	152.906818	0.16313064	0.3619102	0.45074893	0.65217051	0.99938111	Atrn	128.360849	226.261859	153.632174	55.8397803	141.508996	303.422067	118.358458	95.8703563
Bod1	36.2762717	0.13873126	0.30778171	0.45074565	0.65217288	0.99938111	Bod1	30.2420848	25.3413283	32.7621406	49.6353602	33.2962344	24.697145	39.4528194	54.7830607
Erh	9.75906682	0.1759716	0.39060387	0.45051167	0.65234154	0.99938111	Erh	14.7850192	6.33533207	8.90621297	6.20442003	11.653682	10.5844907	17.32075	2.28262753
3110035E14Rik	109.103934	-0.17704	0.39304548	-0.4504313	0.65239947	0.99938111	3110035E14Ri	168.683628	138.924782	133.911274	35.1583802	98.2238915	62.3308898	205.924472	29.6741579
Farp2	10.6138251	-0.1622033	0.36011419	-0.4504218	0.65240635	0.99938111	Farp2	10.7527413	9.5029981	12.0870033	14.4769801	13.3184938	10.5844907	9.62263889	4.56525506
Arhgef6	15.7795752	0.15129288	0.33598791	0.45029262	0.65249946	0.99938111	Arhgef6	13.4409266	16.7433776	15.9039517	12.4088401	9.98887032	14.1126543	23.0943333	20.5436478
Arl13b	6.34922362	0.18452924	0.41001333	0.45005669	0.65266956	0.99938111	Arl13b	5.37637063	2.71514231	7.63389683	6.20442003	13.3184938	3.52816357	2.88679167	9.13051012
Sac3d1	12.7665631	-0.178154	0.39585656	-0.4500469	0.65267665	0.99938111	Sac3d1	14.1129729	8.59795066	10.1785291	22.7495401	16.6481172	3.52816357	5.77358333	20.5436478
Chrna2	2.41369795	0.09637058	0.21415628	0.45000117	0.6527096	NA	Chrna2	2.01613899	0	3.18079035	0	0	14.1126543	0	0
Espn	0.4911935	0.13452024	0.29904478	0.44983311	0.65283078	NA	Espn	0	0.45252372	0.63615807	0	1.66481172	1.17605452	0	0
Tada2a	11.8171949	0.16200865	0.36022291	0.44974555	0.65289393	0.99938111	Tada2a	12.7688802	10.8605693	13.6773985	6.20442003	8.3240586	15.2887088	18.2830139	9.13051012
Ncstn	37.9657706	-0.1430545	0.31822957	-0.4495323	0.65304769	0.99938111	Ncstn	34.2743627	52.9452751	50.2564875	20.6814001	41.620293	44.6900719	25.0188611	34.239413
Lbp	0.46582448	-0.1246501	0.27732701	-0.4494698	0.65309282	NA	Lbp	0	1.81009488	0.9542371	0	0	0	0.96226389	0
4930506C21Rik	0.53669406	0.12566941	0.27962079	0.449428	0.65312294	NA	4930506C21Ri	0	0.45252372	0.9542371	0	0	0	2.88679167	0
Prmt1	19.2054852	0.17117943	0.38090389	0.4494032	0.65314082	0.99938111	Prmt1	16.1291119	9.95552182	13.9954775	31.0221002	21.6425524	7.05632714	17.32075	36.5220405
Xkr6	3.25929038	0.18263044	0.40655851	0.44921071	0.65327966	NA	Xkr6	1.34409266	1.35757116	2.54463228	6.20442003	9.98887032	2.35210905	0	2.28262753
Acot6	1.92393207	0.17886725	0.39821384	0.44917387	0.65330624	NA	Acot6	0.67204633	0.90504744	2.54463228	2.06814001	4.99443516	0	1.92452778	2.28262753
2610020C07Rik	2.90231978	-0.1868569	0.41609147	-0.4490765	0.65337644	NA	2610020C07Ri	4.03227797	2.26261859	4.13502745	2.06814001	1.66481172	3.52816357	0.96226389	4.56525506
Gle1	26.1719731	-0.152114	0.33875842	-0.4490339	0.65340722	0.99938111	Gle1	34.2743627	23.9837571	19.4028211	33.0902402	31.6314227	14.1126543	16.3584861	36.5220405
Dcaf6	58.3352146	0.14573549	0.32456499	0.44901789	0.65341875	0.99938111	Dcaf6	65.8605402	61.5432258	70.2954666	18.6132601	46.6147282	76.4435441	70.2452639	57.0656883
Map2	458.093609	0.17097757	0.38089156	0.44888779	0.6535126	0.99938111	Map2	473.792662	535.788083	461.850758	206.814001	213.0959	996.118182	594.679083	182.610202
Phc3	75.3521626	0.11357887	0.25307478	0.44879568	0.65357905	0.99938111	Phc3	66.5325865	87.3370778	77.2932054	55.8397803	79.9109626	101.140689	73.1320555	61.6309433
Nsd1	165.712014	0.13720766	0.30572443	0.44879522	0.65357939	0.99938111	Nsd1	182.796601	176.48425	174.62539	90.9981605	158.157113	246.97145	202.075417	93.5877288
Stxbp3b	1.10371694	-0.1546313	0.34459996	-0.448727	0.65362862	NA	Stxbp3b	0.67204633	4.52523719	0.31807903	0	0	2.35210905	0.96226389	0
Arvcf	19.8886308	0.17493052	0.39002277	0.44851362	0.65378256	0.99938111	Arvcf	14.7850192	12.2181404	9.86045007	37.2265202	34.9610461	8.23238167	14.4339583	27.3915304
Foxj2	50.516255	0.12817684	0.28580639	0.44847435	0.65381089	0.99938111	Foxj2	45.027104	59.2806072	40.3960374	45.4990802	78.2461509	44.6900719	38.4905555	52.5004332
Ppp4r4	20.1268568	0.15147558	0.33777302	0.44845376	0.65382575	0.99938111	Ppp4r4	18.1452509	14.0282353	24.8101647	18.6132601	9.98887032	24.697145	27.9056528	22.8262753
Fam83h	2.57784758	0.18149728	0.40484011	0.44831843	0.6539234	NA	Fam83h	2.01613899	0.45252372	0.9542371	6.20442003	3.32962344	1.17605452	1.92452778	4.56525506
Echs1	18.9471597	-0.1441266	0.32154821	-0.4482271	0.65398933	0.99938111	Echs1	18.1452509	19.9110436	21.3112953	22.7495401	13.3184938	16.4647633	25.981125	13.6957652
Zfp202	2.09085399	-0.184506	0.41172589	-0.4481281	0.65406073	NA	Zfp202	3.36023164	1.81009488	2.22655324	2.06814001	1.66481172	2.35210905	0.96226389	2.28262753
Map2k3os	0.32008573	0.09786874	0.21843898	0.44803698	0.65412651	NA	Map2k3os	0	0	0.63615807	0	0	0	1.92452778	0
Tstd1	0.32008573	0.09786874	0.21843898	0.44803698	0.65412651	NA	Tstd1	0	0	0.63615807	0	0	0	1.92452778	0
Coro2a	24.5167585	-0.1460172	0.32593759	-0.4479914	0.65415939	0.99938111	Coro2a	28.8979921	24.8888045	17.1762679	33.0902402	23.3073641	15.2887088	19.2452778	34.239413
9330158H04Rik	1.11404265	0.16808713	0.37525172	0.44793167	0.65420251	NA	9330158H04Ri	0.67204633	0.45252372	0.9542371	2.06814001	1.66481172	1.17605452	1.92452778	0
Zfyve1	39.6934439	0.13333711	0.29769184	0.44790313	0.65422311	0.99938111	Zfyve1	41.6668724	31.6766603	38.8056422	37.2265202	58.2684102	47.042181	24.0565972	38.804668
Gpr98	1.12135944	-0.1549371	0.34594254	-0.4478696	0.65424733	NA	Gpr98	0	1.35757116	0.63615807	4.13628002	1.66481172	1.17605452	0	0
Stk25	166.294967	0.11384676	0.25421663	0.44783364	0.65427326	0.99938111	Stk25	182.124555	123.538975	155.858727	173.723761	138.179373	134.070216	180.905611	241.958518
Adm	0.26602174	-0.0944034	0.2108233	-0.4477845	0.65430873	NA	Adm	0	1.81009488	0.31807903	0	0	0	0	0
Ildr1	0.26602174	-0.0944034	0.2108233	-0.4477845	0.65430873	NA	Ildr1	0	1.81009488	0.31807903	0	0	0	0	0
Isg20	0.26602174	-0.0944034	0.2108233	-0.4477845	0.65430873	NA	Isg20	0	1.81009488	0.31807903	0	0	0	0	0
Eya3	23.2658634	0.14582142	0.32577691	0.44761128	0.65443376	0.99938111	Eya3	20.1613899	24.8888045	24.1740066	18.6132601	24.9721758	38.8097993	23.0943333	11.4131377
Ar	20.5817907	0.15386732	0.34378802	0.44756451	0.65446752	0.99938111	Ar	18.1452509	13.5757116	15.5858727	31.0221002	24.9721758	24.697145	11.5471667	25.1089028
Clec5a	0.49703435	-0.1348061	0.30153897	-0.4470605	0.65483141	NA	Clec5a	0.67204633	1.81009488	0.31807903	0	0	1.17605452	0	0
Ccsap	32.7286401	0.13155961	0.29430275	0.44702134	0.65485965	0.99938111	Ccsap	40.994826	25.3413283	32.4440615	24.8176801	34.9610461	27.0492541	44.2641389	31.9567854
Hpgds	0.72499391	-0.1473863	0.32974857	-0.4469656	0.65489989	NA	Hpgds	2.01613899	0.90504744	0.9542371	0	0	0	1.92452778	0
Bbip1	24.7988327	-0.1816377	0.40638745	-0.4469569	0.65490621	0.99938111	Bbip1	38.978687	27.6039469	32.7621406	10.3407001	1.66481172	32.9295267	40.4150833	13.6957652
Slc25a12	144.729773	0.12363062	0.27661941	0.44693402	0.6549227	0.99938111	Slc25a12	135.081312	96.3875521	122.460428	198.541441	129.855314	124.66178	138.566	212.28436
Ak6	2.52204312	-0.1819203	0.40714252	-0.4468221	0.6550035	NA	Ak6	3.36023164	4.97776091	3.49886938	0	0	3.52816357	4.81131944	0
Ube2i	69.581426	-0.1466401	0.32821296	-0.4467832	0.65503157	0.99938111	Ube2i	55.1077989	56.1129411	56.2999891	130.292821	86.5702095	42.3379629	50.0377222	79.8919636
Mtmr11	5.40903312	0.18364322	0.41112673	0.44668276	0.65510413	0.99938111	Mtmr11	2.68818531	5.43028463	3.49886938	8.27256004	11.653682	2.35210905	4.81131944	4.56525506
B3gnt3	0.75171774	-0.1554682	0.34809628	-0.4466241	0.65514648	NA	B3gnt3	2.01613899	0.90504744	0.9542371	0	0	1.17605452	0.96226389	0
Zeb1	40.4929173	-0.1600138	0.35830282	-0.4465881	0.65517252	0.99938111	Zeb1	43.6830113	56.5654649	47.7118552	26.8858201	21.6425524	54.0985081	59.6603611	13.6957652
Gpt	2.28755961	-0.1655953	0.37081893	-0.4465666	0.65518803	NA	Gpt	2.68818531	0	0.63615807	8.27256004	0	1.17605452	0.96226389	4.56525506
Tgm3	1.79631047	0.1677423	0.37583874	0.44631456	0.65537004	NA	Tgm3	0	1.81009488	1.90847421	2.06814001	6.65924688	0	1.92452778	0
Grina	321.827271	-0.1451802	0.32529896	-0.4462977	0.65538225	0.99938111	Grina	260.081929	436.685389	309.490901	357.788222	369.588202	208.161651	144.339583	488.482292
Gm6194	6.88144593	-0.1784574	0.39997354	-0.446173	0.65547225	0.99938111	Gm6194	8.06455594	8.59795066	4.45310648	8.27256004	6.65924688	4.7042181	2.88679167	11.4131377
Trim23	36.368534	0.17404739	0.39013099	0.44612553	0.65550657	0.99938111	Trim23	39.6507334	43.442277	35.3067728	14.4769801	16.6481172	58.8027262	71.2075278	11.4131377
Dgcr6	28.676344	-0.1649657	0.36988418	-0.4459927	0.6556025	0.99938111	Dgcr6	28.2259458	14.480759	22.9016905	59.9760603	31.6314227	12.9365998	25.0188611	34.239413
Rftn1	7.00088092	0.18222963	0.40862302	0.44596026	0.65562595	0.99938111	Rftn1	4.7043243	4.97776091	3.18079035	14.4769801	9.98887032	3.52816357	10.5849028	4.56525506
Acot13	24.7687107	0.14184	0.31814185	0.44583887	0.65571364	0.99938111	Acot13	24.1936678	21.2686148	25.1282437	24.8176801	19.9777406	30.5774176	38.4905555	13.6957652
Polrmt	14.5645643	0.16514907	0.37059208	0.44563571	0.65586041	0.99938111	Polrmt	12.0968339	10.8605693	9.224292	22.7495401	24.9721758	12.9365998	7.69811111	15.9783927
Htr7	10.0062154	0.16889975	0.37902092	0.44562118	0.65587091	0.99938111	Htr7	8.73660227	10.8605693	12.0870033	4.13628002	6.65924688	18.8168724	9.62263889	9.13051012

GeneID	Base mean	log2(FC)	StdErr	Wald-Stats	P-value	P-adj	GeneID	Normalised expression for Chow#1	Normalised expression for Chow#2	Normalised expression for Chow#3	Normalised expression for Chow#4	Normalised expression for HFD#1	Normalised expression for HFD#2	Normalised expression for HFD#3	Normalised expression for HFD#4
Zfr	292.0684	-0.1319187	0.29611362	-0.4455004	0.65595817	0.99938111	Zfr	341.399535	376.499734	322.85022	190.268881	194.782971	377.513502	348.339528	184.89283
Slc16a2	20.738088	-0.1660309	0.37268795	-0.4454958	0.65596151	0.99938111	Slc16a2	14.1129729	36.2018975	26.7186389	10.3407001	28.3017992	22.345036	9.62263889	18.2610202
Dtwd2	2.63179043	0.18027028	0.40467324	0.44547123	0.65597925	NA	Dtwd2	3.36023164	0.45252372	1.59039517	4.13628002	0	1.17605452	5.77358333	4.56525506
Psma7	36.657678	0.1516205	0.3405817	0.44518099	0.65618896	0.99938111	Psma7	28.8979921	26.2463757	31.4898244	53.7716403	21.6425524	30.5774176	66.3962083	34.239413
Zfp128	6.81697252	0.18259909	0.41017655	0.44517194	0.6561955	0.99938111	Zfp128	4.03227797	8.14542694	9.86045007	0	8.3240586	4.7042181	5.77358333	13.6957652
Cd44	3.30421859	0.18467066	0.41485056	0.44514984	0.65621147	NA	Cd44	1.34409266	4.97776091	1.90847421	4.13628002	1.66481172	4.7042181	7.69811111	0
Nabp1	7.14775484	0.17408084	0.39106296	0.44514786	0.65621291	0.99938111	Nabp1	5.37637063	10.4080455	6.04350166	4.13628002	6.65924688	9.40843619	10.5849028	4.56525506
Nxf1	23.6921508	-0.1350461	0.30361252	-0.4447977	0.65646597	0.99938111	Nxf1	22.8495752	25.3413283	23.5378486	28.9539601	24.9721758	14.1126543	26.9433889	22.8262753
Cd97	3.39320533	-0.1848784	0.41566885	-0.4447732	0.65648368	NA	Cd97	3.36023164	5.43028463	3.81694841	2.06814001	6.65924688	3.52816357	0	2.28262753
Cobll1	6.22219776	-0.1825313	0.4105219	-0.4446323	0.65658547	0.99938111	Cobll1	6.04841696	10.8605693	6.99773876	4.13628002	6.65924688	3.52816357	11.5471667	0
A930003O13Rik	10.1497406	-0.1808628	0.40682756	-0.4445688	0.65663141	0.99938111	A930003O13R	6.04841696	10.8605693	10.1785291	16.5451201	8.3240586	3.52816357	2.88679167	22.8262753
Pi15	0.49908347	0.10405448	0.23408276	0.44452004	0.65666664	NA	Pi15	0	0	0	2.06814001	0	0	1.92452778	0
Thsd1	0.54132341	-0.1387607	0.31221615	-0.444438	0.65672593	NA	Thsd1	0.67204633	1.35757116	0.63615807	0	1.66481172	0	0	0
Mfsd10	6.47293057	-0.1795452	0.40398543	-0.4444349	0.65672817	0.99938111	Mfsd10	0.67204633	5.43028463	5.72542262	18.6132601	13.3184938	1.17605452	0	6.84788259
Nipsnap1	44.7060818	0.12656484	0.28479546	0.44440608	0.65674902	0.99938111	Nipsnap1	44.3550577	31.6766603	34.3525357	62.0442003	46.6147282	37.6337448	46.1886666	54.7830607
Gas2	5.70546175	0.18335163	0.41264313	0.44433463	0.65680067	0.99938111	Gas2	6.04841696	5.43028463	5.40734359	4.13628002	6.65924688	3.52816357	14.4339583	0
Ndfip1	223.196877	0.11826819	0.26618391	0.44431006	0.65681843	0.99938111	Ndfip1	142.473822	230.334573	240.785829	237.836101	154.82749	275.196759	216.509375	287.611069
Leng8	18.5070079	0.17289271	0.38922341	0.44419915	0.6568986	0.99938111	Leng8	12.7688802	14.9332827	11.1327662	28.9539601	31.6314227	7.05632714	9.62263889	31.9567854
1700113A16Rik	13.9415066	0.15204126	0.34237146	0.44408276	0.65698275	0.99938111	1700113A16Ri	12.7688802	10.4080455	12.0870033	18.6132601	19.9777406	14.1126543	14.4339583	9.13051012
Snrpa1	8.59592824	0.18287573	0.41194568	0.44393166	0.65709199	0.99938111	Snrpa1	14.7850192	6.78785578	6.99773876	2.06814001	1.66481172	9.40843619	20.2075417	6.84788259
Nfe2l3	1.73158969	-0.1797643	0.40498354	-0.4438806	0.6571289	NA	Nfe2l3	1.34409266	1.81009488	2.54463228	2.06814001	1.66481172	1.17605452	0.96226389	2.28262753
Pin4	1.31165295	0.16967177	0.38232704	0.44378699	0.6571966	NA	Pin4	1.34409266	1.35757116	1.59039517	0	0	2.35210905	3.84905555	0
Elovl6	41.1066439	0.16719234	0.37679422	0.44372321	0.65724271	0.99938111	Elovl6	40.994826	52.9452751	42.3045116	14.4769801	34.9610461	82.3238167	47.1509305	13.6957652
Lztr1	76.8152287	0.13628718	0.30719595	0.44364902	0.65729636	0.99938111	Lztr1	72.5810035	64.2583681	68.0689134	84.7937404	136.514561	45.8661264	62.5471528	79.8919636
Txndc12	11.1155009	-0.1656145	0.37331158	-0.443636	0.65730578	0.99938111	Txndc12	5.37637063	16.2908539	12.0870033	14.4769801	8.3240586	9.40843619	11.5471667	11.4131377
Arid3a	4.95189281	-0.1846381	0.41633199	-0.4434877	0.65741305	0.99938111	Arid3a	4.03227797	4.07271347	1.27231614	14.4769801	4.99443516	2.35210905	3.84905555	4.56525506
Map2k6	12.5366441	0.16855735	0.38010415	0.44345044	0.65743996	0.99938111	Map2k6	11.4247876	16.7433776	12.7231614	4.13628002	19.9777406	8.23238167	20.2075417	6.84788259
Efhd2	255.955164	0.17172172	0.38724867	0.44344043	0.6574472	0.99938111	Efhd2	179.43637	159.288349	166.355335	430.173122	288.012428	82.3238167	164.547125	577.504765
Setd8	20.9252107	-0.1511121	0.34081788	-0.4433809	0.65749024	0.99938111	Setd8	22.8495752	25.793852	20.0389792	22.7495401	19.9777406	23.5210905	27.9056528	4.56525506
Nop9	9.23015227	-0.1774052	0.40028902	-0.4431929	0.65762625	0.99938111	Nop9	8.73660227	10.4080455	6.04350166	14.4769801	8.3240586	4.7042181	2.88679167	18.2610202
Tomm70a	178.342666	0.12117419	0.27347653	0.4430881	0.65770201	0.99938111	Tomm70a	194.893435	161.550968	201.662108	119.952121	113.207197	205.809542	242.4905	187.175458
Dnttip1	9.17926675	0.16965981	0.38307028	0.44289473	0.65784188	0.99938111	Dnttip1	8.73660227	4.52523719	7.31581779	14.4769801	13.3184938	8.23238167	7.69811111	9.13051012
Capza1	16.6547976	-0.1769787	0.39963827	-0.4428473	0.65787621	0.99938111	Capza1	19.4893435	19.4585199	22.5836114	12.4088401	1.66481172	19.9929269	30.7924444	6.84788259
Kti12	10.8555588	-0.1665195	0.37623667	-0.4425924	0.65806057	0.99938111	Kti12	13.4409266	6.33533207	9.54237104	18.6132601	8.3240586	8.23238167	8.660375	13.6957652
Plekhg3	4.37469606	-0.1842408	0.4162903	-0.4425778	0.65807114	NA	Plekhg3	2.68818531	3.62018975	4.77118552	8.27256004	6.65924688	1.17605452	0.96226389	6.84788259
Dock6	7.3669613	0.17876937	0.40397067	0.44253056	0.65810532	0.99938111	Dock6	2.01613899	1.81009488	3.18079035	18.6132601	19.9777406	0	1.92452778	11.4131377
Fstl4	9.41068418	-0.1773178	0.40069456	-0.442526	0.65810861	0.99938111	Fstl4	7.39250961	14.9332827	14.3135566	4.13628002	9.98887032	16.4647633	5.77358333	2.28262753
Dhrs7b	8.15973454	-0.1801922	0.40720046	-0.4425147	0.65811678	0.99938111	Dhrs7b	6.72046328	11.7656167	9.54237104	6.20442003	1.66481172	8.23238167	2.88679167	18.2610202
Zbtb44	53.2573765	0.14541751	0.32865754	0.44245906	0.65815705	0.99938111	Zbtb44	50.4034746	57.4705123	57.5723052	35.1583802	43.2851047	64.6829988	92.3773333	25.1089028
Cog2	9.89300477	-0.1626555	0.36772928	-0.442324	0.65825478	0.99938111	Cog2	10.0806949	10.4080455	13.9954775	8.27256004	6.65924688	9.40843619	13.4716944	6.84788259
Hdac1	2.44257491	0.18042783	0.40808495	0.44213301	0.65839296	NA	Hdac1	1.34409266	2.71514231	3.18079035	0	0	3.52816357	1.92452778	6.84788259
Crh	1.79242855	-0.1525762	0.34513841	-0.4420725	0.65843673	NA	Crh	0	5.88280835	2.22655324	0	1.66481172	0	0	4.56525506
Cyb5d1	6.49168805	-0.1818854	0.41164553	-0.4418496	0.65859807	0.99938111	Cyb5d1	4.03227797	9.05047438	4.77118552	10.3407001	6.65924688	4.7042181	0.96226389	11.4131377
Cactin	16.3914977	0.1670713	0.37814941	0.44181294	0.65862458	0.99938111	Cactin	13.4409266	10.8605693	11.7689243	24.8176801	31.6314227	9.40843619	8.660375	20.5436478
Fchsd1	42.7460965	0.11972304	0.27099474	0.44179102	0.65864044	0.99938111	Fchsd1	43.010965	40.7271347	35.9429309	41.3628002	44.9499164	36.4576902	35.6037639	63.9135709
Dpcd	12.822605	0.15567766	0.35242774	0.44172931	0.6586851	0.99938111	Dpcd	12.7688802	9.95552182	12.4050823	14.4769801	9.98887032	18.8168724	17.32075	6.84788259
Hexdc	10.163641	0.18141149	0.41085096	0.44155059	0.65881444	0.99938111	Hexdc	4.7043243	5.88280835	6.04350166	20.6814001	16.6481172	2.35210905	6.73584722	18.2610202
Pask	1.15757206	0.16662007	0.37735937	0.44154216	0.65882055	NA	Pask	0.67204633	0.90504744	0.63615807	2.06814001	1.66481172	2.35210905	0.96226389	0
Svil	2.1477188	-0.1744385	0.39506916	-0.4415392	0.65882272	NA	Svil	3.36023164	1.35757116	0.31807903	6.20442003	1.66481172	2.35210905	1.92452778	0
Ralgapb	124.847854	0.11795608	0.26715016	0.44153474	0.65882591	0.99938111	Ralgapb	121.640385	142.092448	132.320878	78.5893204	111.542385	183.464506	124.132042	105.000866
Extl1	34.5905244	-0.1680995	0.38072908	-0.44152	0.65883658	0.99938111	Extl1	33.6023164	44.7998482	37.8514051	31.0221002	51.6091633	17.6408179	7.69811111	52.5004332
Lipe	6.94479611	-0.1836171	0.41589468	-0.4414991	0.6588517	0.99938111	Lipe	4.03227797	4.97776091	4.13502745	18.6132601	8.3240586	1.17605452	2.88679167	11.4131377
Mia3	52.2508504	0.18091947	0.40996347	0.44130632	0.65899125	0.99938111	Mia3	45.027104	71.9512713	47.0756971	22.7495401	11.653682	161.11947	35.6037639	22.8262753
Panx2	46.6324291	0.14654427	0.33207713	0.44129588	0.65899881	0.99938111	Panx2	40.3227797	51.587704	37.8514051	43.4309402	81.5757743	42.3379629	21.1698055	54.7830607
Ttc4	18.3758637	-0.1516768	0.34371373	-0.4412881	0.65900442	0.99938111	Ttc4	18.8172972	15.8383302	19.4028211	26.8858201	14.9833055	11.7605452	27.9056528	11.4131377
Mvp	0.96269392	-0.15605	0.35369747	-0.4411964	0.65907084	NA	Mvp	1.34409266	2.26261859	0.63615807	0	0	1.17605452	0	2.28262753
Nub1	72.510214	0.12525603	0.28396187	0.44110159	0.65913945	0.99938111	Nub1	75.9412351	59.7331309	76.0208892	66.1804803	64.9276571	99.9646345	96.2263889	41.0872956
Hps1	2.06480342	-0.1785707	0.40495061	-0.4409691	0.6592354	NA	Hps1	3.36023164	2.26261859	1.90847421	2.06814001	4.99443516	0	1.92452778	0
Sgpp2	8.30345848	-0.1808679	0.41037649	-0.4407366	0.6594037	0.99938111	Sgpp2	4.7043243	14.0282353	14.6316356	2.06814001	8.3240586	12.9365998	2.88679167	6.84788259
Qrich1	76.5949258	-0.1212921	0.27528352	-0.440608	0.65949684	0.99938111	Qrich1	96.7746713	95.9350284	73.794336	53.7716403	54.9387868	97.6125255	76.0188472	63.9135709
Cdh1	0.98798415	-0.1487696	0.33770771	-0.4405277	0.65955497	NA	Cdh1	0.67204633	1.35757116	2.54463228	0	3.32962344	0	0	0
Tmem180	21.9174709	-0.1732417	0.39339496	-0.440376	0.65966479	0.99938111	Tmem180	8.73660227	25.3413283	18.130505	43.4309402	19.9777406	16.4647633	6.73584722	36.5220405
Vta1	19.0384046	-0.1431858	0.32528911	-0.4401803	0.65980656	0.99938111	Vta1	17.4732045	19.9110436	26.7186389	16.5451201	14.9833055	24.697145	18.2830139	13.6957652
St3gal3	28.6991672	0.14650595	0.33285704	0.44014676	0.65983081	0.99938111	St3gal3	24.1936678	31.6766603	28.6271131	20.6814001	44.9499164	31.7534722	13.4716944	34.239413
Abcg1	56.3468143	-0.12174	0.27668601	-0.4399933	0.65994194	0.99938111	Abcg1	54.4357526	57.4705123	50.5745665	74.4530404	58.2684102	63.5069443	32.7169722	59.3483158
Snx17	54.8901649	0.16446426	0.37382311	0.43995208	0.65997182	0.99938111	Snx17	36.2905017	38.9170398	27.354797	101.338861	74.9165274	28.2253086	38.4905555	93.5877288
9530082P21Rik	106.799751	0.15319107	0.34820828	0.43994092	0.6599799	0.99938111	9530082P21Ri	70.5648645	70.1411764	90.3344458	167.519341	136.514561	70.5632714	61.5848889	187.175458
Socs3	2.07387004	0.17530732	0.39848786	0.43993141	0.65998679	NA	Socs3	1.34409266	1.35757116	0.63615807	4.13628002	1.66481172	0	2.88679167	4.56525506
Bai2	237.278332	0.16407454	0.37303913	0.43983199	0.66005879	0.99938111	Bai2	161.291119	288.710133	165.083019	258.517501	506.102763	132.894161	86.60375	299.024207
Nsmce1	2.88521389	-0.182468	0.41489626	-0.4397919	0.66008783	NA	Nsmce1	2.01613899	2.71514231	4.13502745	4.13628002	1.66481172	0	3.84905555	4.56525506
Glmn	5.36303868	0.18279529	0.41583274	0.43958849	0.66023518	0.99938111	Glmn	5.37637063	6.78785578	5.08926455	2.06814001	1.66481172	9.40843619	12.5094306	0
Ccdc160	2.01895967	0.17219863	0.39204281	0.43923426	0.6604918	NA	Ccdc160	1.34409266	1.81009488	1.59039517	2.06814001	0	7.05632714	0	2.28262753
Adcy7	2.10346038	0.17125929	0.39001838	0.43910568	0.66058497	NA	Adcy7	0	3.16766603	1.90847421	2.06814001	1.66481172	7.05632714	0.96226389	0
Abracl	2.72420421	0.18167197	0.41398828	0.43883361	0.6607821	NA	Abracl	3.36023164	3.62018975	1.90847421	0	0	3.52816357	4.81131944	4.56525506
Bud13	13.1673092	-0.1546502	0.35247475	-0.4387553	0.66083884	0.99938111	Bud13	15.4570656	17.1959013	10.4966081	14.4769801	8.3240586	12.9365998	17.32075	9.13051012
Kif4	0.47438792	0.11829586	0.26965488	0.43869356	0.66088359	NA	Kif4	0	0	0.9542371	0	1.66481172	1.17605452	0	0
Nlrp5-ps	0.47438792	0.11829586	0.26965488	0.43869356	0.66088359	NA	Nlrp5-ps	0	0	0.9542371	0	1.66481172	1.17605452	0	0
Shisa3	0.47438792	0.11829586	0.26965488	0.43869356	0.66088359	NA	Shisa3	0	0	0.9542371	0	1.66481172	1.17605452	0	0
Pdia6	28.8863884	0.15152559	0.34548111	0.43859299	0.66095648	0.99938111	Pdia6	24.8657142	41.1796584	24.8101647	14.4769801	34.9610461	41.1619083	15.3962222	34.239413
Ubxn1	36.9512778	-0.139182	0.3174417	-0.4384491	0.66106078	0.99938111	Ubxn1	48.3873356	27.6039469	27.354797	53.7716403	34.9610461	30.5774176	25.0188611	47.9351782
Mkln1	67.8724302	0.1171085	0.26718939	0.43829773	0.66117047	0.99938111	Mkln1	61.1562159	70.1411764	63.6158069	66.1804803	44.9499164	92.9083074	84.6792222	59.3483158
Neurod1	6.84839468	-0.1824268	0.41637076	-0.4381355	0.66128803	0.99938111	Neurod1	8.06455594	9.95552182	9.224292	4.13628002	1.66481172	1.17605452	18.2830139	2.28262753
Gbp9	0.25536486	-0.0919567	0.21007974	-0.4377228	0.66158722	NA	Gbp9	0	0.45252372	1.59039517	0	0	0	0	0
Ptpn7	0.25536486	-0.0919567	0.21007974	-0.4377228	0.66158722	NA	Ptpn7	0	0.45252372	1.59039517	0	0	0	0	0
Pola2	2.34952318	-0.1797254	0.41059264	-0.4377219	0.66158788	NA	Pola2	2.01613899	2.26261859	2.22655324	4.13628002	1.66481172	0	1.92452778	4.56525506
Sez6l2	235.145605	-0.1429909	0.32681287	-0.4375313	0.66172605	0.99938111	Sez6l2	186.828879	366.544212	250.010121	192.337021	289.677239	272.84465	92.3773333	230.545381
Pdp1	80.1532585	-0.1313332	0.30018322	-0.4375101	0.66174148	0.99938111	Pdp1	103.495135	88.2421252	103.057607	41.3628002	54.9387868	83.4998712	98.1509166	68.4788259
Ap2s1	95.9112341	0.15662864	0.35806818	0.43742686	0.66180181	0.99938111	Ap2s1	67.8766792	61.5432258	66.4785182	161.314921	123.196067	54.0985081	61.5848889	171.197065
N4bp2l1	18.7571882	0.17119717	0.39148763	0.43729905	0.66189448	0.99938111	N4bp2l1	22.1775288	16.7433776	23.2197695	6.20442003	13.3184938	38.8097993	25.0188611	4.56525506
Med19	41.8854698	0.11962424	0.27358661	0.43724451	0.66193403	0.99938111	Med19	40.3227797	44.3473245	48.0299342	24.8176801	43.2851047	43.5140174	51.96225	38.804668
Mtag2	0.96537244	-0.1359906	0.31106003	-0.4371846	0.66197751	NA	Mtag2	1.34409266	0	0.31807903	4.13628002	0	0	1.92452778	0
Chek2	0.54169665	0.13272936	0.30365592	0.43710447	0.66203558	NA	Chek2	0	0.45252372	0.63615807	0	0	0	0.96226389	2.28262753
Gm10658	0.54169665	0.13272936	0.30365592	0.43710447	0.66203558	NA	Gm10658	0	0.45252372	0.63615807	0	0	0	0.96226389	2.28262753
Rrm2	0.54169665	0.13272936	0.30365592	0.43710447	0.66203558	NA	Rrm2	0	0.45252372	0.63615807	0	0	0	0.96226389	2.28262753
Tars2	14.0158251	0.16196493	0.37054536	0.43709879	0.66203971	0.99938111	Tars2	13.4409266	12.6706641	7.63389683	18.6132601	24.9721758	8.23238167	10.5849028	15.9783927
Wash	9.24738814	0.16438122	0.37618556	0.43696845	0.66213423	0.99938111	Wash	8.73660227	7.2403795	10.8146872	6.20442003	13.3184938	4.7042181	11.5471667	11.4131377
Lrrtm2	52.5468284	-0.1664363	0.38096487	-0.436881	0.66219765	0.99938111	Lrrtm2	55.1077989	84.1694117	69.3412295	18.6132601	23.3073641	83.4998712	63.5094166	22.8262753
Zfp740	40.2845582	0.14607161	0.33436949	0.43685687	0.66221515	0.99938111	Zfp740	36.9625481	23.9837571	26.0824808	66.1804803	48.2795399	32.9295267	30.7924444	57.0656883
3110070M22Rik	2.05438594	0.17509932	0.40083333	0.43683823	0.66222867	NA	3110070M22R	1.34409266	3.16766603	1.27231614	0	1.66481172	1.17605452	0.96226389	6.84788259
Ncln	26.3552503	0.17314436	0.39637133	0.43682362	0.66223927	0.99938111	Ncln	20.1613899	33.0342315	20.3570582	20.6814001	51.6091633	22.345036	3.84905555	38.804668
Zfp212	9.04444844	0.17682275	0.4048606	0.43674971	0.66229287	0.99938111	Zfp212	4.7043243	4.07271347	6.04350166	20.6814001	11.653682	12.9365998	7.69811111	4.56525506
Tm9sf1	20.337499	-0.1578524	0.36143881	-0.4367333	0.66230479	0.99938111	Tm9sf1	15.4570656	26.6988994	26.0824808	16.5451201	24.9721758	18.8168724	6.73584722	27.3915304
Ppm1k	61.061532	0.14599814	0.3343018	0.43672555	0.6623104	0.99938111	Ppm1k	61.8282622	62.9007969	72.8400989	31.0221002	46.6147282	97.6125255	83.7169583	31.9567854
Sepn1	17.9579221	-0.1386913	0.31759641	-0.4366903	0.66233594	0.99938111	Sepn1	16.8011582	23.0787097	18.130505	18.6132601	18.3129289	19.9929269	17.32075	11.4131377
Cc2d1b	8.58119956	-0.1738914	0.39823162	-0.4366591	0.66235862	0.99938111	Cc2d1b	5.37637063	10.4080455	5.08926455	18.6132601	4.99443516	10.5844907	6.73584722	6.84788259
Tfg	42.209914	-0.1369408	0.31365869	-0.4365918	0.66240743	0.99938111	Tfg	53.0916599	53.3977988	47.0756971	24.8176801	33.2962344	54.0985081	49.0754583	22.8262753
E030019B13Rik	1.99515297	0.15550138	0.35638245	0.43633288	0.66259523	NA	E030019B13Ri	0	0.45252372	0.31807903	6.20442003	0	1.17605452	0.96226389	6.84788259
Nudt3	258.677717	0.14455621	0.33138182	0.43622251	0.6626753	0.99938111	Nudt3	237.232354	188.702391	196.572843	347.447522	306.325357	134.070216	193.415042	465.656016
Prmt6	14.5630394	0.16838196	0.38605854	0.43615656	0.66272314	0.99938111	Prmt6	10.7527413	11.313093	7.95197586	24.8176801	23.3073641	4.7042181	15.3962222	18.2610202
Tpx2	1.85820051	0.1756431	0.40282919	0.43602377	0.66281949	NA	Tpx2	2.68818531	1.35757116	1.90847421	0	0	4.7042181	1.92452778	2.28262753
Lmln	8.64747134	0.16726354	0.38392929	0.43566235	0.66308173	0.99938111	Lmln	4.03227797	9.5029981	9.54237104	8.27256004	9.98887032	12.9365998	5.77358333	9.13051012
C77080	18.1418131	-0.1584335	0.36368498	-0.4356339	0.66310239	0.99938111	C77080	20.1613899	17.1959013	13.3593194	26.8858201	26.6369875	9.40843619	8.660375	22.8262753

GeneID	Base mean	log2(FC)	StdErr	Wald-Stats	P-value	P-adj	GeneID	Normalised expression for Chow#1	Normalised expression for Chow#2	Normalised expression for Chow#3	Normalised expression for Chow#4	Normalised expression for HFD#1	Normalised expression for HFD#2	Normalised expression for HFD#3	Normalised expression for HFD#4
Shisa9	46.9936091	0.1615481	0.3708694	0.43559298	0.66313207	0.99938111	Shisa9	22.8495752	71.9512713	53.1191988	24.8176801	33.2962344	95.2604165	40.4150833	34.239413
Scn2b	318.209243	0.11354594	0.26067223	0.43558895	0.66313499	0.99938111	Scn2b	212.36664	366.091689	292.632712	345.379382	321.308662	411.619083	224.207486	372.068288
4921504A21Rik	2.57445183	-0.1783076	0.40947802	-0.4354511	0.66323506	NA	4921504A21Ri	1.34409266	4.07271347	4.77118552	2.06814001	0	3.52816357	4.81131944	0
Pan3	30.204809	-0.1574837	0.36178887	-0.4352917	0.66335071	0.99938111	Pan3	40.3227797	37.5594687	37.215247	14.4769801	11.653682	31.7534722	48.1131944	20.5436478
Plekhg1	6.0081989	-0.1749577	0.40195197	-0.4352702	0.66336633	0.99938111	Plekhg1	5.37637063	9.5029981	5.08926455	6.20442003	4.99443516	2.35210905	7.69811111	6.84788259
Prmt5	45.5001597	0.126214	0.29004142	0.43515854	0.66344736	0.99938111	Prmt5	46.3711967	45.7048956	40.0779583	37.2265202	44.9499164	36.4576902	35.6037639	77.6093361
Lcmt1	51.2640297	0.13934912	0.32042069	0.43489426	0.66363918	0.99938111	Lcmt1	40.994826	38.0119924	38.8056422	76.5211804	36.6258578	45.8661264	44.2641389	89.0224737
Nlrp6	0.52489107	0.11964375	0.27529427	0.43460311	0.66385054	NA	Nlrp6	0	0	0.9542371	0	0	0	0.96226389	2.28262753
Cntnap5c	7.50294004	-0.1777162	0.40898225	-0.4345327	0.66390162	0.99938111	Cntnap5c	4.7043243	11.7656167	9.224292	8.27256004	4.99443516	15.2887088	5.77358333	0
Nckipsd	76.7901602	0.15955211	0.36724317	0.43445903	0.66395514	0.99938111	Nckipsd	67.2046328	53.3977988	46.12146	117.883981	123.196067	41.1619083	37.5282917	127.827142
Crim1	55.1200814	0.12794121	0.29458239	0.43431386	0.66406054	0.99938111	Crim1	43.010965	72.8563187	53.1191988	39.2946602	58.2684102	82.3238167	50.9999861	41.0872956
Nmnat3	0.43669934	-0.1318766	0.30372222	-0.4342015	0.66414213	NA	Nmnat3	0.67204633	0.90504744	0.9542371	0	0	0	0.96226389	0
2700062C07Rik	4.41026944	-0.1804834	0.41566839	-0.4342005	0.66414283	NA	2700062C07Ri	2.01613899	3.16766603	7.31581779	8.27256004	4.99443516	4.7042181	4.81131944	0
Syt11	379.773054	0.14474931	0.33343944	0.43410974	0.66420876	0.99938111	Syt11	293.012199	548.006224	396.962635	184.064461	401.219625	658.590533	273.282944	283.045814
Afap1l1	3.29067508	0.18072207	0.41633796	0.43407542	0.66423368	NA	Afap1l1	3.36023164	4.97776091	2.22655324	0	4.99443516	2.35210905	3.84905555	4.56525506
Wdr54	4.60976515	0.18024849	0.41543505	0.43387886	0.66437641	NA	Wdr54	1.34409266	4.52523719	4.45310648	6.20442003	11.653682	3.52816357	2.88679167	2.28262753
Best1	1.33232669	-0.1352048	0.31165605	-0.433827	0.66441407	NA	Best1	0.67204633	0.45252372	0	6.20442003	3.32962344	0	0	0
Tamm41	2.53497083	0.17996262	0.41492011	0.43372838	0.6644857	NA	Tamm41	4.03227797	1.35757116	2.86271131	0	3.32962344	3.52816357	2.88679167	2.28262753
Mcf2	4.75553831	-0.1772375	0.40867037	-0.433693	0.66451136	0.99938111	Mcf2	4.7043243	3.62018975	6.99773876	6.20442003	3.32962344	7.05632714	3.84905555	2.28262753
Ccdc122	0.71435917	-0.1419402	0.32737342	-0.4335728	0.66459867	NA	Ccdc122	1.34409266	1.81009488	0.63615807	0	0	0	1.92452778	0
Cdhr3	0.71435917	-0.1419402	0.32737342	-0.4335728	0.66459867	NA	Cdhr3	1.34409266	1.81009488	0.63615807	0	0	0	1.92452778	0
Ydjc	8.70946646	-0.1804965	0.41630643	-0.4335665	0.66460323	0.99938111	Ydjc	5.37637063	5.43028463	6.04350166	22.7495401	8.3240586	0	5.77358333	15.9783927
Cep104	18.2964951	0.14629791	0.33750484	0.43346909	0.66467402	0.99938111	Cep104	14.1129729	23.5312334	20.0389792	8.27256004	23.3073641	21.1689814	15.3962222	20.5436478
Frs3	31.2970735	0.17418888	0.4018661	0.43345005	0.66468784	0.99938111	Frs3	20.1613899	9.5029981	15.2677937	70.3167603	33.2962344	19.9929269	20.2075417	61.6309433
Cebpb	4.78626952	0.17932278	0.41380909	0.43334666	0.66476295	0.99938111	Cebpb	1.34409266	2.26261859	1.59039517	12.4088401	4.99443516	2.35210905	1.92452778	11.4131377
Fbxo3	56.6149587	0.11508998	0.26558925	0.43333825	0.66476906	0.99938111	Fbxo3	62.5003085	48.4200379	51.5288036	55.8397803	38.2906696	69.3872169	72.1697916	54.7830607
Rap1gap	165.377169	0.15265871	0.35231353	0.43330357	0.66479424	0.99938111	Rap1gap	167.339536	88.2421252	125.005061	235.767961	266.369875	105.844907	85.6414861	248.806401
Slc35e2	51.7992122	0.14938577	0.34477539	0.43328432	0.66480823	0.99938111	Slc35e2	43.6830113	73.3088425	55.663831	18.6132601	53.2739751	87.0280348	39.4528194	43.3699231
Dapp1	1.55490686	-0.167789	0.38732865	-0.4331955	0.66487273	NA	Dapp1	0.67204633	4.07271347	2.22655324	0	3.32962344	1.17605452	0.96226389	0
Pla2g6	20.4053449	0.16834555	0.38868761	0.43311274	0.66493287	0.99938111	Pla2g6	12.7688802	14.0282353	15.5858727	33.0902402	44.9499164	9.40843619	10.5849028	22.8262753
Hdac2	54.7025792	0.13939833	0.32188045	0.43307484	0.6649604	0.99938111	Hdac2	60.4841696	36.6544212	64.251965	45.4990802	28.3017992	71.739326	82.7546944	47.9351782
Dnaja1	63.4176136	0.14207637	0.32808733	0.43304437	0.66498254	0.99938111	Dnaja1	75.2691888	55.2078937	70.9316247	37.2265202	46.6147282	96.436471	91.4150694	34.239413
Fcgr1	0.49869702	-0.1213267	0.2801795	-0.4330319	0.66499161	NA	Fcgr1	0	0.90504744	1.90847421	0	0	1.17605452	0	0
Mdh2	190.531362	0.1392295	0.32153927	0.43300931	0.66500801	0.99938111	Mdh2	154.570656	109.963264	152.677937	301.948441	231.408829	130.542052	157.811278	285.328441
Hyal1	0.5093539	-0.1207586	0.27898817	-0.432845	0.66512736	NA	Hyal1	0	2.26261859	0.63615807	0	0	1.17605452	0	0
Trim30a	0.48022876	-0.13285	0.30702151	-0.4327057	0.66522857	NA	Trim30a	0.67204633	1.35757116	0.63615807	0	0	1.17605452	0	0
Dnase1	1.70654252	0.17124979	0.39581701	0.43264889	0.66526987	NA	Dnase1	0.67204633	2.26261859	1.27231614	2.06814001	0	3.52816357	3.84905555	0
1700101I11Rik	0.71979138	-0.149178	0.34483898	-0.4326019	0.66530398	NA	1700101I11Ri	0.67204633	1.35757116	1.59039517	0	0	1.17605452	0.96226389	0
Slc22a5	3.07492584	-0.1800346	0.41623226	-0.4325341	0.66535327	NA	Slc22a5	4.03227797	3.62018975	3.18079035	2.06814001	4.99443516	1.17605452	0.96226389	4.56525506
Ispd	7.02120437	-0.1741957	0.40286602	-0.4323912	0.66545708	0.99938111	Ispd	10.7527413	4.52523719	6.67965972	10.3407001	1.66481172	7.05632714	10.5849028	4.56525506
Vmp1	23.3174308	-0.1650501	0.38178335	-0.4323134	0.66551365	0.99938111	Vmp1	18.1452509	39.8220873	28.6271131	14.4769801	11.653682	43.5140174	21.1698055	9.13051012
Fam208a	35.1532236	0.15457476	0.35761044	0.43224341	0.66556451	0.99938111	Fam208a	40.3227797	42.0847059	34.9886938	12.4088401	16.6481172	54.0985081	50.9999861	29.6741579
Tcf3	11.8215157	0.17183545	0.39757563	0.4322082	0.6655901	0.99938111	Tcf3	14.1129729	8.14542694	9.224292	10.3407001	24.9721758	4.7042181	4.81131944	18.2610202
Zap70	1.69432159	-0.1447512	0.33501723	-0.432071	0.66568978	NA	Zap70	0.67204633	0	0.31807903	8.27256004	3.32962344	0	0.96226389	0
Cacna2d2	70.6277731	0.1443263	0.33406064	0.43203623	0.66571508	0.99938111	Cacna2d2	60.4841696	78.7391271	64.570044	59.9760603	84.9053977	114.077289	26.9433889	75.3267085
Cul4a	35.8023066	0.12248653	0.28354469	0.43198316	0.66575365	0.99938111	Cul4a	31.5861774	28.5089943	33.7163777	43.4309402	24.9721758	36.4576902	37.5282917	50.2178057
Cnep1r1	13.7361546	-0.1720032	0.39819857	-0.4319534	0.6657753	0.99938111	Cnep1r1	15.4570656	19.0059962	19.7209001	6.20442003	6.65924688	9.40843619	28.8679167	4.56525506
Sertad4	20.8318821	0.15862459	0.36735715	0.43179939	0.66588723	0.99938111	Sertad4	16.8011582	10.4080455	14.9497146	37.2265202	11.653682	21.1689814	20.2075417	34.239413
Ctsf	15.2347684	-0.1528694	0.35428986	-0.4314811	0.66611859	0.99938111	Ctsf	12.0968339	19.0059962	17.4943469	14.4769801	19.9777406	10.5844907	7.69811111	20.5436478
Unc13c	39.1043152	0.16606854	0.38488396	0.43147691	0.66612164	0.99938111	Unc13c	38.3066407	47.5149905	41.0321955	16.5451201	39.9554813	76.4435441	46.1886666	6.84788259
BC005561	11.0564887	0.16812365	0.3897305	0.43138438	0.6661889	0.99938111	BC005561	12.7688802	9.05047438	12.7231614	6.20442003	3.32962344	21.1689814	16.3584861	6.84788259
Bdh1	23.8720875	-0.1315865	0.30506145	-0.4313442	0.66621809	0.99938111	Bdh1	27.5538995	22.6261859	21.3112953	28.9539601	19.9777406	16.4647633	22.1320694	31.9567854
Jrk	10.4238717	0.17705194	0.41054554	0.43126018	0.6662792	0.99938111	Jrk	8.73660227	7.2403795	6.67965972	14.4769801	19.9777406	7.05632714	0.96226389	18.2610202
Mmachc	22.4541731	0.13803426	0.32009365	0.43123085	0.66630052	0.99938111	Mmachc	27.5538995	23.5312334	20.9932163	10.3407001	16.6481172	25.8731995	25.0188611	29.6741579
Atp6ap1	167.084952	-0.1457898	0.33810662	-0.4311946	0.66632685	0.99938111	Atp6ap1	136.425405	259.748615	210.8864	101.338861	136.514561	242.267232	76.0188472	173.479692
Gm9767	1.03856609	0.14806651	0.34355862	0.43097889	0.66648372	NA	Gm9767	1.34409266	0.90504744	0.31807903	0	0	1.17605452	0	4.56525506
Stxbp4	13.4941176	-0.1743478	0.40455453	-0.4309624	0.6664957	0.99938111	Stxbp4	16.1291119	19.0059962	18.448584	6.20442003	3.32962344	22.345036	20.2075417	2.28262753
Ncoa2	97.7008642	0.11055347	0.25654495	0.43093217	0.66651769	0.99938111	Ncoa2	104.839227	96.3875521	100.194896	72.3849004	76.5813391	139.950488	106.811292	84.4572186
1700026D08Rik	0.55677419	0.11754523	0.27280605	0.43087473	0.66655946	NA	1700026D08Ri	0.67204633	0.45252372	0	0	3.32962344	0	0	0
Actc1	0.55677419	0.11754523	0.27280605	0.43087473	0.66655946	NA	Actc1	0.67204633	0.45252372	0	0	3.32962344	0	0	0
Wars	77.9000895	0.12073971	0.28028798	0.43077022	0.66663546	0.99938111	Wars	71.2369108	55.6604174	63.9338859	107.543281	86.5702095	58.8027262	72.1697916	107.283494
Gm16576	0.51381779	-0.1204702	0.27967843	-0.4307453	0.66665358	NA	Gm16576	1.34409266	0	1.59039517	0	0	1.17605452	0	0
Alg8	3.85935856	-0.1791597	0.41598482	-0.4306881	0.66669515	NA	Alg8	2.01613899	4.07271347	7.31581779	4.13628002	4.99443516	3.52816357	4.81131944	0
Camk2n2	197.257726	-0.1619028	0.37592099	-0.430683	0.66669889	0.99938111	Camk2n2	171.371814	147.522732	153.314095	378.469622	196.447783	70.5632714	101.999972	358.372522
Rhobtb2	101.081378	-0.1467764	0.3409181	-0.4305327	0.66680819	0.99938111	Rhobtb2	115.591968	90.0522201	84.9271022	138.565381	134.849749	55.2745626	43.301875	146.088162
Il34	40.120995	-0.1362755	0.31663388	-0.4303884	0.66691314	0.99938111	Il34	37.6345944	59.7331309	32.7621406	37.2265202	39.9554813	39.9858538	21.1698055	52.5004332
Slc1a4	32.1191134	-0.1399815	0.32533251	-0.4302721	0.66699772	0.99938111	Slc1a4	25.5377605	50.6826565	33.0802196	24.8176801	34.9610461	37.6337448	18.2830139	31.9567854
Fsbp	1.15882671	0.15445926	0.35899895	0.4302499	0.66701387	NA	Fsbp	0	0.90504744	1.27231614	2.06814001	0	1.17605452	3.84905555	0
Atp2c2	0.6017448	-0.1363568	0.31698829	-0.4301636	0.66707662	NA	Atp2c2	0.67204633	0.90504744	0.9542371	0	0	0	0	2.28262753
Ift43	2.84959755	-0.1771917	0.41197561	-0.4301024	0.66712118	NA	Ift43	3.36023164	4.07271347	4.45310648	0	0	1.17605452	2.88679167	6.84788259
Ap5s1	16.7915696	-0.1660399	0.38613519	-0.4300047	0.66719224	0.99938111	Ap5s1	11.4247876	13.5757116	14.9497146	33.0902402	21.6425524	5.88027262	8.660375	25.1089028
Osbpl2	52.1132385	0.11174766	0.2599785	0.42983426	0.66731621	0.99938111	Osbpl2	55.7798453	45.2523719	48.9841713	49.6353602	36.6258578	67.0351079	51.96225	61.6309433
Oxa1l	35.8579274	-0.1390985	0.3236663	-0.4297589	0.66737106	0.99938111	Oxa1l	31.5861774	34.3918026	33.7163777	51.7035003	29.966611	17.6408179	30.7924444	57.0656883
Nars2	6.19277694	-0.1705341	0.39683754	-0.4297327	0.66739012	0.99938111	Nars2	8.06455594	5.88280835	7.63389683	6.20442003	3.32962344	9.40843619	6.73584722	2.28262753
Napa	130.393684	-0.112166	0.26103126	-0.4297033	0.66741146	0.99938111	Napa	164.65135	138.472258	123.732744	115.815841	128.190502	76.4435441	152.037694	143.805534
Rundc3b	35.4404757	0.12614854	0.29365553	0.42957998	0.6675012	0.99938111	Rundc3b	43.010965	28.0564706	34.0344567	28.9539601	23.3073641	35.2816357	45.2264028	45.6525506
Ubn1	43.564084	-0.1251154	0.29126042	-0.4295653	0.66751186	0.99938111	Ubn1	44.3550577	50.2301328	45.803381	39.2946602	46.6147282	38.8097993	24.0565972	59.3483158
Psmd3	112.940152	0.15307547	0.35637609	0.4295335	0.66753502	0.99938111	Psmd3	110.215598	69.6886527	90.6525248	148.906081	136.514561	59.9787807	61.5848889	225.980126
Ilf2	52.1805781	0.12670377	0.29499747	0.42950797	0.6675536	0.99938111	Ilf2	59.8121232	44.7998482	61.3892537	31.0221002	41.620293	52.9224536	77.943375	47.9351782
Ccdc51	1.94758207	0.15831378	0.3687212	0.42935904	0.66766195	NA	Ccdc51	3.36023164	0.45252372	1.59039517	0	3.32962344	0	0	6.84788259
Mcm7	3.28627956	-0.1785736	0.41599468	-0.4292689	0.66772754	NA	Mcm7	4.7043243	2.71514231	3.18079035	4.13628002	4.99443516	2.35210905	1.92452778	2.28262753
1810026J23Rik	74.2826094	0.10720186	0.24977681	0.4291906	0.66778452	0.99938111	1810026J23Ri	63.8444012	64.2583681	60.1169375	99.2707205	76.5813391	70.5632714	68.3207361	91.3051012
Bcl7c	2.60157045	0.17433751	0.40633418	0.42904957	0.66788715	NA	Bcl7c	0.67204633	1.35757116	2.54463228	4.13628002	3.32962344	0	1.92452778	6.84788259
Clvs1	95.7534308	0.11752294	0.27393126	0.42902347	0.66790615	0.99938111	Clvs1	92.7423933	101.365313	94.4694732	76.5211804	54.9387868	108.197016	132.792417	105.000866
Homez	17.7639224	-0.1537246	0.358325	-0.4290089	0.66791675	0.99938111	Homez	15.4570656	14.9332827	15.2677937	31.0221002	14.9833055	17.6408179	7.69811111	25.1089028
Dmwd	132.816162	0.15477304	0.36079305	0.42898011	0.6679377	0.99938111	Dmwd	115.591968	83.716888	81.7463119	213.018421	178.134854	71.739326	78.9056389	239.675891
Mzt1	59.6899069	0.14645884	0.34145748	0.42892262	0.66797954	0.99938111	Mzt1	51.075521	73.3088425	65.8423601	33.0902402	41.620293	54.0985081	117.396194	41.0872956
Clk3	26.6600485	0.15631994	0.3644683	0.4288986	0.66799702	0.99938111	Clk3	18.1452509	16.7433776	19.0847421	47.5672202	36.6258578	11.7605452	33.6792361	29.6741579
Lsm11	18.1432893	0.15000663	0.34979056	0.428847	0.66803457	0.99938111	Lsm11	17.4732045	11.7656167	20.3570582	18.6132601	31.6314227	11.7605452	22.1320694	11.4131377
Nat9	3.58617719	-0.1777085	0.41439084	-0.4288428	0.66803765	NA	Nat9	4.7043243	3.62018975	3.49886938	4.13628002	4.99443516	3.52816357	1.92452778	2.28262753
Isoc2a	9.97423927	-0.1683881	0.39268781	-0.4288092	0.66806209	0.99938111	Isoc2a	4.7043243	14.480759	7.63389683	16.5451201	6.65924688	7.05632714	6.73584722	15.9783927
Wnt9a	3.58166225	0.17698233	0.41284825	0.42868617	0.66815162	NA	Wnt9a	6.04841696	3.16766603	2.54463228	0	11.653682	2.35210905	2.88679167	0
Mettl7a1	8.0417834	-0.1637031	0.38199888	-0.4285434	0.66825556	0.99938111	Mettl7a1	6.72046328	10.4080455	6.67965972	12.4088401	6.65924688	8.23238167	8.660375	4.56525506
Ulk2	114.31774	-0.090969	0.21228806	-0.4285168	0.66827487	0.99938111	Ulk2	114.919922	122.181404	121.188112	113.747701	88.2350212	129.365998	103.9245	120.979259
Arpc1a	56.281191	0.12696147	0.29648396	0.42822374	0.66848823	0.99938111	Arpc1a	62.5003085	39.8220873	53.4372778	55.8397803	61.5980337	37.6337448	48.1131944	91.3051012
Thoc7	18.3366667	-0.1662422	0.38832825	-0.428097	0.66858052	0.99938111	Thoc7	21.5054825	23.5312334	22.9016905	12.4088401	3.32962344	21.1689814	32.7169722	9.13051012
Fbxo33	42.8042151	0.1335549	0.31200561	0.42805288	0.66861262	0.99938111	Fbxo33	47.7152893	41.6321821	33.7163777	41.3628002	33.2962344	56.4506172	65.4339444	22.8262753
Ythdf1	52.6847226	0.11175578	0.26108957	0.42803616	0.6686248	0.99938111	Ythdf1	63.1723549	45.2523719	53.7553568	39.2946602	56.6035985	58.8027262	63.5094166	41.0872956
Sh3bgrl2	27.4922929	0.1389195	0.32459829	0.4279736	0.66867034	0.99938111	Sh3bgrl2	22.1775288	26.2463757	36.2610099	18.6132601	31.6314227	37.6337448	33.6792361	13.6957652
1300002E11Rik	4.63313711	-0.1769647	0.41351506	-0.4279523	0.66868586	NA	1300002E11Ri	4.7043243	7.2403795	3.49886938	4.13628002	3.32962344	1.17605452	3.84905555	9.13051012
Rbm6	11.6379807	0.15836121	0.37011581	0.42786936	0.66874624	0.99938111	Rbm6	15.4570656	10.8605693	10.8146872	6.20442003	9.98887032	11.7605452	21.1698055	6.84788259
Fanci	1.82841528	-0.169146	0.39544169	-0.4277395	0.66884082	NA	Fanci	4.7043243	0.90504744	2.86271131	0	1.66481172	3.52816357	0.96226389	0
Glyr1	170.496881	0.11181712	0.26152444	0.42755897	0.66897224	0.99938111	Glyr1	177.420231	135.757116	140.909012	198.541441	239.732888	154.063143	121.24525	196.305968
Cxcr5	0.46446967	0.12752602	0.29827295	0.42754806	0.66898019	NA	Cxcr5	0	0.45252372	0.63615807	0	1.66481172	0	0.96226389	0
Zfx	17.1593758	0.16787268	0.39276594	0.42741149	0.66907964	0.99938111	Zfx	16.1291119	15.3858064	18.130505	14.4769801	8.3240586	31.7534722	30.7924444	2.28262753
Rin3	1.79967201	-0.1690016	0.39546977	-0.4273438	0.66912892	NA	Rin3	2.01613899	3.62018975	2.54463228	0	3.32962344	0	2.88679167	0
Paip2b	55.5974547	0.11377064	0.26624831	0.42731027	0.66915335	0.99938111	Paip2b	44.3550577	41.1796584	66.7965972	59.9760603	54.9387868	49.39429	59.6603611	68.4788259
Pkdcc	3.14398842	0.17725776	0.4149168	0.42721277	0.66922436	NA	Pkdcc	1.34409266	2.26261859	1.90847421	6.20442003	6.65924688	3.52816357	0.96226389	2.28262753
Zfp335	22.2826186	0.15052375	0.35234846	0.42720137	0.66923266	0.99938111	Zfp335	16.8011582	19.9110436	17.1762679	28.9539601	36.6258578	15.2887088	11.5471667	31.9567854
Ndel1	87.6171858	-0.1144904	0.26813676	-0.4269849	0.66939031	0.99938111	Ndel1	88.7101153	65.6159392	76.9751264	138.565381	83.240586	77.6195986	78.9056389	91.3051012
Tlr7	0.98733303	-0.1415674	0.33160741	-0.4269125	0.66944303	NA	Tlr7	0	3.16766603	1.27231614	0	0	1.17605452	0	2.28262753
Carkd	16.0676913	-0.1584614	0.37147318	-0.4265755	0.66968854	0.99938111	Carkd	14.7850192	22.1736622	10.4966081	20.6814001	9.98887032	8.23238167	12.5094306	29.6741579

GeneID	Base mean	log2(FC)	StdErr	Wald-Stats	P-value	P-adj		GeneID	Normalised expression for Chow#1	Normalised expression for Chow#2	Normalised expression for Chow#3	Normalised expression for Chow#4	Normalised expression for HFD#1	Normalised expression for HFD#2	Normalised expression for HFD#3	Normalised expression for HFD#4
Dgke	47.240757	0.15556591	0.36469249	0.42656735	0.66969448	0.99938111		Dgke	35.6184554	68.7836053	49.3022503	20.6814001	31.6314227	97.6125255	42.3396111	31.9567854
Rap2c	23.4751545	0.13839081	0.32443161	0.4265639	0.669697	0.99938111		Rap2c	28.2259458	20.3635674	20.3570582	20.6814001	21.6425524	18.8168724	39.4528194	18.2610202
Chd8	62.6697365	0.1180367	0.27673996	0.4265257	0.66972483	0.99938111		Chd8	64.5164475	57.4705123	66.4785182	51.7035003	58.2684102	92.9083074	71.2075278	38.804668
Fam96b	9.41484555	-0.1686521	0.39563146	-0.4262858	0.66989961	0.99938111		Fam96b	6.72046328	6.78785578	10.1785291	18.6132601	9.98887032	2.35210905	11.5471667	9.13051012
Agbl3	5.70388535	0.1758377	0.4125644	0.42620668	0.66995725	0.99938111		Agbl3	6.04841696	5.43028463	3.81694841	6.20442003	0	7.05632714	12.5094306	4.56525506
E330009J07Rik	13.3885318	0.1554288	0.36468703	0.42619777	0.66996374	0.99938111		E330009J07Ri	11.4247876	9.05047438	11.4508452	18.6132601	23.3073641	7.05632714	12.5094306	13.6957652
Nedd8	72.6793324	-0.1200639	0.28206144	-0.4256658	0.67035135	0.99938111		Nedd8	75.2691888	60.1856546	67.7508343	103.407001	66.5924688	42.3379629	83.7169583	82.1745911
Ap3b1	29.9665222	-0.1304915	0.30660138	-0.4256063	0.67039472	0.99938111		Ap3b1	43.010965	26.2463757	34.0344567	22.7495401	36.6258578	27.0492541	31.7547083	18.2610202
A230077H06Rik	5.51427421	-0.1703768	0.40031814	-0.4256034	0.67039686	0.99938111		A230077H06R	6.04841696	6.78785578	6.04350166	4.13628002	6.65924688	4.7042181	2.88679167	6.84788259
Adipor2	30.3845568	-0.1466176	0.34461479	-0.4254537	0.67050594	0.99938111		Adipor2	22.8495752	48.4200379	35.3067728	20.6814001	44.9499164	29.4013631	16.3584861	25.1089028
Artn	0.36433164	0.11041709	0.25962306	0.42529772	0.67061965	NA		Artn	0.67204633	0	0.31807903	0	0	0	1.92452778	0
Zfp959	2.2048101	0.16880602	0.39703045	0.42517146	0.67071169	NA		Zfp959	1.34409266	3.16766603	2.86271131	0	0	3.52816357	6.73584722	0
Mpp5	35.8845778	0.13984349	0.32893053	0.42514597	0.67073026	0.99938111		Mpp5	39.6507334	38.9170398	37.5333261	18.6132601	24.9721758	56.4506172	48.1131944	22.8262753
Smpd4	17.6021084	-0.1603277	0.37715868	-0.4250935	0.67076854	0.99938111		Smpd4	15.4570656	17.1959013	19.0847421	24.8176801	34.9610461	7.05632714	15.3962222	6.84788259
Trim12c	0.75204775	0.15039875	0.35385479	0.42502957	0.67081512	NA		Trim12c	0.67204633	0.90504744	0.63615807	0	1.66481172	1.17605452	0.96226389	0
Gabrd	11.0344167	-0.1682842	0.39597056	-0.4249918	0.67084266	0.99938111		Gabrd	14.7850192	15.8383302	13.9954775	2.06814001	18.3129289	12.9365998	5.77358333	4.56525506
Kcnh3	39.8565094	-0.1637542	0.38546812	-0.4248191	0.67096853	0.99938111		Kcnh3	33.6023164	64.7108918	51.2107246	20.6814001	79.9109626	22.345036	14.4339583	31.9567854
Gm3383	1.17394748	0.15038216	0.35414817	0.42463063	0.67110596	NA		Gm3383	1.34409266	0	0.9542371	2.06814001	0	1.17605452	3.84905555	0
Klc2	191.276461	0.1469244	0.34603135	0.42459853	0.67112937	0.99938111		Klc2	141.129729	131.684402	121.506191	322.629842	276.358746	108.197016	143.377319	285.328441
Cdkl3	6.83445502	0.17238072	0.40613906	0.42443768	0.67124664	0.99938111		Cdkl3	4.7043243	6.78785578	7.95197586	6.20442003	3.32962344	7.05632714	16.3584861	2.28262753
D11Wsu47e	3.73061345	0.17626099	0.4153443	0.42437319	0.67129367	NA		D11Wsu47e	4.03227797	4.07271347	2.54463228	2.06814001	3.32962344	1.17605452	5.77358333	6.84788259
Zfp97	2.17640146	0.16781845	0.39545422	0.42436885	0.67129684	NA		Zfp97	2.68818531	1.81009488	2.86271131	0	0	2.35210905	7.69811111	0
Ttc38	4.75190469	-0.1724872	0.40647337	-0.4243505	0.67131023	0.99938111		Ttc38	7.39250961	4.07271347	5.40734359	4.13628002	4.99443516	5.88027262	3.84905555	2.28262753
Atrx	140.395083	0.16204166	0.38191492	0.42428733	0.67135628	0.99938111		Atrx	156.586795	152.953017	155.222569	51.7035003	68.2572805	290.485467	188.603722	59.3483158
Rps24	13.7570263	-0.1642268	0.38712213	-0.4242249	0.67140181	0.99938111		Rps24	25.5377605	12.2181404	12.0870033	10.3407001	4.99443516	9.40843619	24.0565972	11.4131377
2610028E06Rik	0.85872098	-0.1382408	0.32590347	-0.4241772	0.67143657	NA		2610028E06Ri	2.01613899	0	2.22655324	0	1.66481172	0	0.96226389	0
Zfp90	7.18544749	0.17308491	0.40816699	0.42405416	0.67152631	0.99938111		Zfp90	10.0806949	3.62018975	8.58813393	4.13628002	3.32962344	12.9365998	12.5094306	2.28262753
Zbtb5	6.86461743	-0.1655491	0.39040712	-0.4240422	0.67153502	0.99938111		Zbtb5	6.72046328	6.33533207	7.63389683	10.3407001	4.99443516	4.7042181	9.62263889	4.56525506
Gldc	7.05203719	-0.1728118	0.4076473	-0.4239249	0.67162057	0.99938111		Gldc	8.06455594	9.95552182	7.95197586	4.13628002	16.6481172	3.52816357	3.84905555	2.28262753
Zfp458	10.0856053	0.17629129	0.41591742	0.42386128	0.67166699	0.99938111		Zfp458	8.73660227	10.4080455	13.9954775	2.06814001	0	25.8731995	17.32075	2.28262753
Zfp318	42.7417697	0.13225046	0.31202621	0.42384407	0.67167954	0.99938111		Zfp318	45.027104	43.442277	42.9406697	31.0221002	28.3017992	67.0351079	56.7735694	27.3915304
Ubr7	27.2411704	-0.1176309	0.27760789	-0.4237304	0.67176244	0.99938111		Ubr7	29.5700384	28.0564706	28.6271131	26.8858201	24.9721758	27.0492541	23.0943333	29.6741579
Golph3	117.545285	-0.1134359	0.26774386	-0.4236733	0.67180408	0.99938111		Golph3	106.18332	113.583453	119.279638	153.042361	103.218327	68.2111624	137.603736	139.240279
Ppp1r2	116.096806	0.10906452	0.25747437	0.42359369	0.67186216	0.99938111		Ppp1r2	105.511274	102.27036	101.467212	136.497241	93.2294563	88.2040893	139.528264	162.066555
Stk24	51.1447921	0.10841054	0.25593557	0.42358529	0.67186828	0.99938111		Stk24	55.7798453	47.5149905	50.5745665	41.3628002	38.2906696	64.6829988	53.8867778	57.0656883
Lclat1	30.0366468	-0.1575063	0.37202441	-0.4233764	0.67202069	0.99938111		Lclat1	25.5377605	56.1129411	34.0344567	12.4088401	13.3184938	45.8661264	27.9056528	25.1089028
Abi3	24.6014349	0.16769196	0.39610767	0.42334944	0.67204032	0.99938111		Abi3	20.8334362	15.8383302	19.4028211	33.0902402	38.2906696	4.7042181	14.4339583	50.2178057
Prss36	5.27901117	-0.1739799	0.41102053	-0.4232875	0.67208551	0.99938111		Prss36	9.4086486	5.88280835	3.18079035	4.13628002	3.32962344	2.35210905	4.81131944	9.13051012
Wdfy4	0.96865608	0.15692256	0.37081176	0.42318657	0.67215914	NA		Wdfy4	0.67204633	1.35757116	0.9542371	0	1.66481172	1.17605452	1.92452778	0
Plekhm3	53.3659803	0.11606053	0.27426762	0.42316524	0.6721747	0.99938111		Plekhm3	45.027104	58.8280835	37.8514051	62.0442003	59.9332219	48.2182355	44.2641389	70.7614535
Tug1	125.964993	-0.158054	0.37350674	-0.4231623	0.67217689	0.99938111		Tug1	133.065173	170.601442	170.490362	68.2486203	39.9554813	201.105324	162.622597	61.6309433
Prdx2	77.3740855	0.12309301	0.29099288	0.42301038	0.67228769	0.99938111		Prdx2	71.2369108	50.6826565	63.2977279	111.679561	83.240586	54.0985081	86.60375	98.1529838
Tcf12	32.1906603	-0.1562889	0.3695324	-0.422937	0.67234122	0.99938111		Tcf12	28.2259458	49.3250854	36.897168	24.8176801	11.653682	50.5703445	42.3396111	13.6957652
Psrc1	1.57897756	-0.1652676	0.39077061	-0.4229275	0.67234817	NA		Psrc1	4.03227797	0.90504744	2.22655324	0	3.32962344	1.17605452	0.96226389	0
Il12a	0.9131934	-0.147329	0.34838498	-0.4228915	0.67237443	NA		Il12a	1.34409266	0.90504744	0.63615807	2.06814001	0	2.35210905	0	0
Galnt14	10.5897787	0.15419464	0.36469854	0.42280027	0.67244099	0.99938111		Galnt14	10.0806949	12.6706641	10.8146872	4.13628002	11.653682	15.2887088	8.660375	11.4131377
Uba1	277.486762	0.08644889	0.20452043	0.42269074	0.67252092	0.99938111		Uba1	316.533821	259.748615	258.280176	235.767961	314.649415	250.499614	251.150875	333.26362
9330102E08Rik	12.9706961	-0.1557164	0.36840036	-0.4226827	0.67252681	0.99938111		9330102E08Ri	11.4247876	13.5757116	20.0389792	10.3407001	19.9777406	9.40843619	14.4339583	4.56525506
9330159M07Rik	1.40070448	0.16673412	0.39460901	0.42252993	0.67263826	NA		9330159M07R	1.34409266	0.90504744	1.90847421	0	1.66481172	1.17605452	1.92452778	2.28262753
Ehhadh	1.94875033	0.1695367	0.40125647	0.42251456	0.67264948	NA		Ehhadh	2.68818531	1.81009488	1.27231614	0	3.32962344	0	1.92452778	4.56525506
Ceacam16	0.86893426	0.11815276	0.27965755	0.42249086	0.67266677	NA		Ceacam16	0	0	0.31807903	2.06814001	0	0	0	4.56525506
Vps33b	8.75940889	0.16521188	0.39110832	0.42241976	0.67271865	0.99938111		Vps33b	10.7527413	4.97776091	10.1785291	6.20442003	16.6481172	9.40843619	9.62263889	2.28262753
Fam57b	99.0190066	-0.173091	0.40981625	-0.4223626	0.67276041	0.99938111		Fam57b	61.8282622	64.7108918	69.9773876	239.904241	108.212762	32.9295267	18.2830139	196.305968
Atp2a3	3.70798063	0.16393279	0.38825594	0.42222866	0.67285812	NA		Atp2a3	1.34409266	5.88280835	2.54463228	2.06814001	16.6481172	1.17605452	0	0
Cnot2	34.8037547	0.15036029	0.35632747	0.4219722	0.67304531	0.99938111		Cnot2	40.994826	35.2968501	41.6683535	10.3407001	18.3129289	51.7463991	48.1131944	31.9567854
Zfp292	42.3809703	0.17199043	0.40758991	0.4219693	0.67304743	0.99938111		Zfp292	32.2582238	55.6604174	52.4830407	12.4088401	19.9777406	88.2040893	71.2075278	6.84788259
Rere	263.400618	0.13059054	0.30957426	0.42183914	0.67314243	0.99938111		Rere	204.302084	231.692144	212.794874	347.447522	347.94565	170.527906	177.056555	415.438211
Dnajc27	156.722245	0.14960162	0.35464441	0.42183556	0.67314505	0.99938111		Dnajc27	98.1187639	87.3370778	105.602239	295.744021	144.83862	123.485725	127.018833	271.632676
Brwd3	8.28189053	0.16172059	0.38338412	0.42182391	0.67315355	0.99938111		Brwd3	9.4086486	7.69290322	9.224292	4.13628002	9.98887032	12.9365998	10.5849028	2.28262753
Prdm2	88.2231961	0.10529173	0.24965038	0.42175673	0.67320259	0.99938111		Prdm2	59.8121232	80.0966983	87.4717345	113.747701	98.2238915	95.2604165	79.8679027	91.3051012
8430408G22Rik	0.24921615	-0.0889872	0.21111054	-0.4215193	0.67337593	NA		8430408G22R	0	1.35757116	0.63615807	0	0	0	0	0
Birc3	0.24921615	-0.0889872	0.21111054	-0.4215193	0.67337593	NA		Birc3	0	1.35757116	0.63615807	0	0	0	0	0
Gm15545	0.24921615	-0.0889872	0.21111054	-0.4215193	0.67337593	NA		Gm15545	0	1.35757116	0.63615807	0	0	0	0	0
Iigp1	0.24921615	-0.0889872	0.21111054	-0.4215193	0.67337593	NA		Iigp1	0	1.35757116	0.63615807	0	0	0	0	0
Kntc1	0.24921615	-0.0889872	0.21111054	-0.4215193	0.67337593	NA		Kntc1	0	1.35757116	0.63615807	0	0	0	0	0
Serpina3g	0.24921615	-0.0889872	0.21111054	-0.4215193	0.67337593	NA		Serpina3g	0	1.35757116	0.63615807	0	0	0	0	0
Zar1l	0.24921615	-0.0889872	0.21111054	-0.4215193	0.67337593	NA		Zar1l	0	1.35757116	0.63615807	0	0	0	0	0
Nlgn3	135.412882	-0.1385226	0.32863432	-0.4215097	0.67338292	0.99938111		Nlgn3	109.543552	206.350816	145.998277	109.611421	196.447783	137.598379	56.7735694	120.979259
Fam220a	31.8649964	-0.1315352	0.31213882	-0.4213997	0.67346321	0.99938111		Fam220a	36.9625481	29.8665654	27.0367179	39.2946602	26.6369875	24.697145	20.2075417	50.2178057
Plcg2	1.15236956	-0.1546409	0.36708855	-0.4212632	0.6735629	NA		Plcg2	1.34409266	0.90504744	0.9542371	2.06814001	1.66481172	0	0	2.28262753
Selt	102.356106	-0.1515514	0.35991529	-0.4210752	0.67370018	0.99938111		Selt	103.495135	153.405541	125.641219	55.8397803	48.2795399	147.006816	139.528264	45.6525506
Myadm	55.6474505	-0.1306023	0.31021826	-0.4210013	0.67375417	0.99938111		Myadm	36.9625481	83.716888	55.9819101	55.8397803	68.2572805	45.8661264	34.6415	63.9135709
Adrb2	0.4490189	-0.1163497	0.27643814	-0.4208886	0.67383644	NA		Adrb2	0	1.35757116	1.27231614	0	0	0	0.96226389	0
AI115009	0.4490189	-0.1163497	0.27643814	-0.4208886	0.67383644	NA		AI115009	0	1.35757116	1.27231614	0	0	0	0.96226389	0
Fignl1	0.4490189	-0.1163497	0.27643814	-0.4208886	0.67383644	NA		Fignl1	0	1.35757116	1.27231614	0	0	0	0.96226389	0
Tmem101	8.3503404	0.16537399	0.39303452	0.420762	0.67392888	0.99938111		Tmem101	4.03227797	11.313093	5.08926455	10.3407001	13.3184938	5.88027262	7.69811111	9.13051012
Rprml	21.2367711	0.16521364	0.39267052	0.42074368	0.67394226	0.99938111		Rprml	18.8172972	20.8160911	17.4943469	18.6132601	29.966611	4.7042181	11.5471667	47.9351782
Suds3	56.9617649	0.11633824	0.27650826	0.42074056	0.67394454	0.99938111		Suds3	44.3550577	45.2523719	51.5288036	78.5893204	54.9387868	43.5140174	64.4716805	73.044081
Aoc2	0.92941275	0.14488929	0.34437395	0.42073244	0.67395047	NA		Aoc2	0	1.35757116	0.9542371	0	1.66481172	1.17605452	0	2.28262753
Nes	1.10072047	0.15735724	0.37415008	0.42057251	0.67406727	NA		Nes	1.34409266	1.35757116	0.63615807	0	3.32962344	1.17605452	0.96226389	0
Zzz3	29.6024518	0.14313599	0.34043842	0.42044604	0.67415964	0.99938111		Zzz3	31.5861774	29.4140417	30.2175083	20.6814001	34.9610461	31.7534722	49.0754583	9.13051012
Rsc1a1	18.2515187	-0.1384707	0.32936173	-0.4204212	0.67417775	0.99938111		Rsc1a1	18.1452509	22.6261859	19.4028211	18.6132601	16.6481172	23.5210905	20.2075417	6.84788259
Trmt2a	12.6214712	0.15859078	0.37727185	0.42036208	0.67422097	0.99938111		Trmt2a	8.06455594	7.2403795	11.4508452	20.6814001	13.3184938	12.9365998	6.73584722	20.5436478
Wbp1	13.7167589	-0.1653852	0.3935172	-0.4202744	0.67428501	0.99938111		Wbp1	13.4409266	12.2181404	7.63389683	26.8858201	11.653682	3.52816357	11.5471667	22.8262753
Lypla2	99.5364762	0.17435112	0.41504718	0.42007542	0.67443035	0.99938111		Lypla2	49.059382	45.7048956	40.7141164	219.222841	163.151549	15.2887088	39.4528194	223.697498
Papl	2.23175589	0.15700445	0.37376994	0.4200564	0.67444425	NA		Papl	1.34409266	4.07271347	1.27231614	0	9.98887032	1.17605452	0	0
Vps26a	40.7169061	0.1465255	0.34888033	0.41998784	0.67449433	0.99938111		Vps26a	31.5861774	49.7776091	38.8056422	33.0902402	18.3129289	42.3379629	79.8679027	31.9567854
Npas2	36.7983247	-0.141613	0.33729058	-0.4198545	0.67459174	0.99938111		Npas2	43.6830113	49.3250854	43.8949068	16.5451201	36.6258578	17.6408179	43.301875	43.3699231
Eif4e2	49.4325719	0.15054557	0.35911863	0.41920848	0.67506378	0.99938111		Eif4e2	35.6184554	27.1514231	39.7598793	82.7256004	61.5980337	24.697145	39.4528194	84.4572186
Ankzf1	6.3547783	0.16874088	0.40287008	0.41884689	0.67532803	0.99938111		Ankzf1	4.7043243	5.43028463	7.63389683	4.13628002	13.3184938	5.88027262	2.88679167	6.84788259
Phlda1	36.5878541	0.13470842	0.32166854	0.41878021	0.67537677	0.99938111		Phlda1	40.994826	28.5089943	34.3525357	33.0902402	28.3017992	19.9929269	48.1131944	59.3483158
1700021K19Rik	73.105022	0.10578884	0.25267382	0.4186775	0.67545184	0.99938111		1700021K19Ri	65.8605402	70.5937002	57.8903843	86.8618804	93.2294563	68.2111624	57.7358333	84.4572186
Snx4	19.8981868	-0.1469421	0.35108115	-0.4185417	0.67555107	0.99938111		Snx4	16.1291119	16.7433776	21.9474534	31.0221002	14.9833055	8.23238167	25.0188611	25.1089028
Slc4a11	0.55364296	-0.1200732	0.28694504	-0.4184536	0.6756155	NA		Slc4a11	0	1.81009488	0.9542371	0	1.66481172	0	0	0
Lsm10	7.31565201	-0.1634393	0.39062807	-0.4184012	0.67565379	0.99938111		Lsm10	7.39250961	8.14542694	6.99773876	8.27256004	3.32962344	5.88027262	4.81131944	13.6957652
Mrpl35	28.0012491	0.13771396	0.32915752	0.41838313	0.67566702	0.99938111		Mrpl35	24.1936678	33.0342315	28.9451921	18.6132601	13.3184938	43.5140174	32.7169722	29.6741579
Erlin2	28.0745814	0.15216558	0.36372864	0.4183492	0.67569182	0.99938111		Erlin2	21.5054825	33.4867552	32.7621406	16.5451201	16.6481172	58.8027262	28.8679167	15.9783927
Brdt	8.49486772	0.16976476	0.40579714	0.41834884	0.67569209	0.99938111		Brdt	8.06455594	8.59795066	8.2700549	6.20442003	0	9.40843619	18.2830139	9.13051012
Fam212b	28.1151344	-0.1656407	0.39611156	-0.4181667	0.6758252	0.99938111		Fam212b	42.3389187	38.4645161	34.3525357	6.20442003	48.2795399	17.6408179	30.7924444	6.84788259
4930528A17Rik	0.60337753	-0.1243371	0.29741949	-0.4180529	0.67590841	NA		4930528A17Ri	1.34409266	0.45252372	0	2.06814001	0	0	0.96226389	0
Gbp7	2.29838305	-0.1679659	0.40186062	-0.4179707	0.67596857	NA		Gbp7	4.03227797	5.43028463	1.27231614	0	1.66481172	1.17605452	4.81131944	0
Armc7	4.2017103	-0.1731488	0.41429958	-0.4179313	0.67599735	NA		Armc7	8.06455594	4.52523719	5.40734359	0	4.99443516	3.52816357	4.81131944	2.28262753
Atad3a	8.11286154	0.16128181	0.38592869	0.41790574	0.67601604	0.99938111		Atad3a	8.73660227	4.52523719	6.67965972	10.3407001	4.99443516	8.23238167	7.69811111	13.6957652
Cntrl	3.37520101	-0.1738686	0.41613484	-0.417818	0.67608017	NA		Cntrl	5.37637063	2.26261859	3.81694841	4.13628002	4.99443516	3.52816357	2.88679167	0
Slc25a19	30.110372	-0.1239667	0.29673461	-0.4177697	0.67611552	0.99938111		Slc25a19	29.5700384	28.0564706	26.4005599	43.4309402	29.966611	19.9929269	26.9433889	36.5220405
Ppp1r3e	9.47380075	0.16392455	0.39253266	0.41760742	0.67623417	0.99938111		Ppp1r3e	5.37637063	8.14542694	8.58813393	12.4088401	13.3184938	2.35210905	9.62263889	15.9783927
Cdh5	2.41585135	-0.1548273	0.37079481	-0.4175551	0.67627245	NA		Cdh5	0	7.69290322	3.18079035	0	4.99443516	1.17605452	0	2.28262753
Pex13	25.1008983	-0.1466454	0.3513151	-0.4174185	0.6763723	0.99938111		Pex13	31.5861774	36.6544212	27.672876	10.3407001	13.3184938	34.1055812	28.8679167	18.2610202
Dhrs11	2.55743809	0.17169179	0.41136769	0.4173682	0.67640911	NA		Dhrs11	1.34409266	2.26261859	1.59039517	4.13628002	4.99443516	0	3.84905555	2.28262753
Sub1	115.608936	0.15406577	0.36926213	0.41722603	0.67651309	0.99938111		Sub1	106.855366	110.868311	113.554215	99.2707205	39.9554813	163.471579	233.830125	57.0656883
Mkrn3	0.28282732	-0.0786355	0.18849925	-0.4171662	0.67655688	NA		Mkrn3	0	2.26261859	0	0	0	0	0	0
Psmb6	40.0497773	-0.143399	0.34382224	-0.4170732	0.67662489	0.99938111		Psmb6	37.6345944	25.3413283	27.672876	82.7256004	38.2906696	29.4013631	33.6792361	45.6525506

GeneID	Base mean	log2(FC)	StdErr	Wald-Stats	P-value	P-adj	GeneID	Normalised expression for Chow#1	Normalised expression for Chow#2	Normalised expression for Chow#3	Normalised expression for Chow#4	Normalised expression for HFD#1	Normalised expression for HFD#2	Normalised expression for HFD#3	Normalised expression for HFD#4
Senp3	28.0446712	-0.1378043	0.33048591	-0.4169748	0.67669684	0.99938111	Senp3	30.9141311	25.793852	20.6751372	41.3628002	31.6314227	18.8168724	16.3584861	38.804668
Lactb	6.0079824	0.17289973	0.41472899	0.41689812	0.67675293	0.99938111	Lactb	6.72046328	4.97776091	9.224292	0	8.3240586	8.23238167	10.5849028	0
Rpl21	14.7406178	0.17268442	0.41442356	0.41668582	0.67690823	0.99938111	Rpl21	23.5216215	12.6706641	10.8146872	6.20442003	1.66481172	19.9929269	38.4905555	4.56525506
Hebp1	7.38760349	0.1713399	0.41133292	0.41654799	0.67700906	0.99938111	Hebp1	6.04841696	7.2403795	4.45310648	8.27256004	16.6481172	1.17605452	3.84905555	11.4131377
Mlh3	22.0151561	-0.1469439	0.35289626	-0.416394	0.67712173	0.99938111	Mlh3	24.8657142	20.3635674	22.2655324	28.9539601	14.9833055	37.6337448	20.2075417	6.84788259
Acvr1c	11.3083067	-0.1640912	0.39409192	-0.416378	0.67713345	0.99938111	Acvr1c	12.0968339	19.4585199	10.1785291	8.27256004	9.98887032	14.1126543	16.3584861	0
2810021J22Rik	8.24052451	0.16576245	0.39812731	0.41635538	0.67714997	0.99938111	2810021J22Ri	12.7688802	5.88280835	6.67965972	4.13628002	18.3129289	5.88027262	7.69811111	4.56525506
Ddx24	124.128236	0.10014635	0.24053494	0.41634844	0.67715505	0.99938111	Ddx24	133.737219	97.2925996	118.961559	130.292821	94.8942681	163.471579	135.679208	118.696632
Fndc1	6.25050146	0.1726153	0.41469707	0.41624433	0.67723123	0.99938111	Fndc1	2.01613899	7.69290322	8.90621297	2.06814001	3.32962344	9.40843619	2.88679167	13.6957652
Hmgb2	0.58522607	0.12659574	0.30414576	0.41623379	0.67723893	NA	Hmgb2	0	0.90504744	0.31807903	0	0	1.17605452	0	2.28262753
C4a	0.64632072	0.12651899	0.30406324	0.41609434	0.67734097	NA	C4a	0	0.90504744	0.31807903	0	1.66481172	0	0	2.28262753
Zfp551	3.90028688	0.17283294	0.41540519	0.4160587	0.67736705	NA	Zfp551	1.34409266	2.71514231	5.72542262	4.13628002	6.65924688	3.52816357	4.81131944	2.28262753
Pex11a	3.52342837	0.17315767	0.41636379	0.4158807	0.6774973	NA	Pex11a	4.7043243	2.26261859	2.54463228	2.06814001	6.65924688	1.17605452	1.92452778	6.84788259
Phf2	56.7061094	-0.1204466	0.2897655	-0.4156691	0.67765218	0.99938111	Phf2	48.3873356	52.0402277	46.12146	95.1344405	54.9387868	63.5069443	43.301875	50.2178057
Dcaf13	7.48685252	0.17130883	0.41224346	0.41555256	0.67773744	0.99938111	Dcaf13	10.7527413	6.78785578	8.90621297	0	3.32962344	8.23238167	17.32075	4.56525506
AA986860	2.53972193	-0.1707697	0.41100106	-0.415497	0.67777812	NA	AA986860	4.7043243	4.07271347	2.54463228	0	3.32962344	4.7042181	0.96226389	0
Arfip2	61.0354529	0.13909424	0.33480907	0.41544346	0.6778173	0.99938111	Arfip2	51.7475673	42.9897533	41.0321955	95.1344405	59.9332219	34.1055812	60.622625	102.718239
Matn2	8.25297684	-0.1671512	0.40234473	-0.4154428	0.67781777	0.99938111	Matn2	4.7043243	13.5757116	12.4050823	4.13628002	14.9833055	5.88027262	5.77358333	4.56525506
Atad3aos	0.52933386	0.09977953	0.24020555	0.41539227	0.67785476	NA	Atad3aos	0	0.90504744	0	0	3.32962344	0	0	0
Tnxb	0.52933386	0.09977953	0.24020555	0.41539227	0.67785476	NA	Tnxb	0	0.90504744	0	0	3.32962344	0	0	0
Gucy2e	2.92455832	-0.1692423	0.40750627	-0.4153122	0.67791336	NA	Gucy2e	5.37637063	3.16766603	3.49886938	0	3.32962344	1.17605452	0	6.84788259
Clspn	0.81549983	-0.1426102	0.34344962	-0.415229	0.67797427	NA	Clspn	0.67204633	0.90504744	0.9542371	2.06814001	0	0	1.92452778	0
Ewsr1	35.4889953	0.13184835	0.31761217	0.41512374	0.67805132	0.99938111	Ewsr1	32.2582238	25.3413283	23.2197695	55.8397803	41.620293	28.2253086	31.7547083	45.6525506
Ubqln4	111.15926	-0.1073835	0.25871225	-0.4150692	0.67809128	0.99938111	Ubqln4	99.4628566	121.72888	100.512975	140.633521	129.855314	83.4998712	78.9056389	134.675024
Srsf7	29.0079334	0.11811514	0.28484244	0.41466834	0.67838471	0.99938111	Srsf7	24.8657142	25.3413283	30.8536663	31.0221002	23.3073641	31.7534722	37.5282917	27.3915304
Sertad3	0.67607524	-0.1277861	0.30823277	-0.4145766	0.67845188	NA	Sertad3	0	1.35757116	0.31807903	2.06814001	1.66481172	0	0	0
Slbp	6.72530946	0.16958292	0.40911262	0.41451402	0.6784977	0.99938111	Slbp	3.36023164	3.16766603	12.7231614	4.13628002	9.98887032	5.88027262	7.69811111	6.84788259
Dennd4a	79.2372089	-0.1422418	0.343156	-0.4145107	0.67850013	0.99938111	Dennd4a	89.3821617	96.8400759	95.7417894	55.8397803	59.9332219	123.485725	87.5660139	25.1089028
F2rl2	1.21010328	-0.1538805	0.37126225	-0.4144791	0.67852324	NA	F2rl2	0.67204633	2.71514231	0.63615807	2.06814001	1.66481172	0	1.92452778	0
Tubgcp3	24.2168888	-0.1315789	0.31752688	-0.4143865	0.67859106	0.99938111	Tubgcp3	33.6023164	23.0787097	23.2197695	20.6814001	33.2962344	16.4647633	18.2830139	25.1089028
Nipa2	21.9671793	-0.1557773	0.37596281	-0.4143423	0.67862342	0.99938111	Nipa2	15.4570656	32.129184	22.9016905	24.8176801	3.32962344	30.5774176	25.981125	20.5436478
Coq2	16.7802694	0.13484762	0.32548776	0.41429399	0.67865881	0.99938111	Coq2	14.1129729	14.9332827	13.9954775	20.6814001	21.6425524	12.9365998	15.3962222	20.5436478
Alad	17.9242895	-0.1490731	0.35991169	-0.4141935	0.67873237	0.99938111	Alad	12.0968339	16.2908539	16.8581888	33.0902402	11.653682	9.40843619	21.1698055	22.8262753
Ezr	14.1306814	-0.1537288	0.37117804	-0.4141645	0.67875363	0.99938111	Ezr	18.1452509	21.2686148	14.6316356	4.13628002	11.653682	8.23238167	14.4339583	20.5436478
Ntng2	13.7544921	-0.1526331	0.36859997	-0.4140888	0.67880909	0.99938111	Ntng2	12.7688802	15.3858064	11.4508452	18.6132601	19.9777406	5.88027262	7.69811111	18.2610202
Ltbp3	25.257916	0.15215719	0.36748573	0.41404924	0.67883805	0.99938111	Ltbp3	20.1613899	26.6988994	19.0847421	26.8858201	44.9499164	22.345036	7.69811111	34.239413
Slc2a13	117.479891	0.14992765	0.36232226	0.4137964	0.67902322	0.99938111	Slc2a13	98.1187639	157.02573	137.410143	43.4309402	69.9220923	235.210905	109.698083	89.0224737
Tmem35	50.0438511	0.14648874	0.35416953	0.41361193	0.67915834	0.99938111	Tmem35	28.2259458	75.1189373	62.6615698	18.6132601	44.9499164	77.6195986	45.2264028	47.9351782
Ccdc117	8.20835678	-0.1585548	0.38335049	-0.4136028	0.67916504	0.99938111	Ccdc117	7.39250961	7.69290322	13.3593194	6.20442003	4.99443516	8.23238167	8.660375	9.13051012
Mpg	11.114654	0.15825331	0.38281298	0.4133959	0.67931658	0.99938111	Mpg	7.39250961	8.59795066	7.31581779	18.6132601	9.98887032	5.88027262	10.5849028	20.5436478
Erlec1	30.661882	0.1677241	0.40593558	0.4131791	0.6794754	0.99938111	Erlec1	26.8818531	39.3695635	34.3525357	10.3407001	8.3240586	79.9717076	34.6415	11.4131377
Ccdc138	1.73812486	-0.162664	0.39370288	-0.4131645	0.67948611	NA	Ccdc138	1.34409266	0.90504744	4.13502745	2.06814001	0	3.52816357	1.92452778	0
Paxip1	31.5290484	-0.1318685	0.31917974	-0.4131481	0.67949813	0.99938111	Paxip1	48.3873356	35.2968501	28.9451921	20.6814001	26.6369875	29.4013631	42.3396111	20.5436478
Edrf1	11.7734267	0.15592793	0.37742533	0.41313584	0.67950709	0.99938111	Edrf1	12.0968339	9.05047438	14.3135566	8.27256004	8.3240586	24.697145	10.5849028	6.84788259
Foxk2	106.044576	0.09135288	0.22115882	0.41306459	0.67955929	0.99938111	Foxk2	118.280154	98.197647	99.5587378	90.9981605	129.855314	91.7322529	101.037708	118.696632
Msto1	13.6436844	-0.152737	0.36980961	-0.4130152	0.67959546	0.99938111	Msto1	17.4732045	9.05047438	11.1327662	22.7495401	19.9777406	9.40843619	12.5094306	6.84788259
Grm8	11.437488	0.17095536	0.41393177	0.41300372	0.67960389	0.99938111	Grm8	5.37637063	20.8160911	13.6773985	0	8.3240586	25.8731995	10.5849028	6.84788259
Ptbp2	76.1603056	0.15301028	0.37054683	0.41293102	0.67965715	0.99938111	Ptbp2	96.102625	82.8118406	79.8378377	22.7495401	29.966611	112.901234	116.433931	68.4788259
LOC106740	8.27566087	-0.1657959	0.40159474	-0.4128437	0.67972114	0.99938111	LOC106740	10.7527413	3.62018975	10.4966081	12.4088401	8.3240586	3.52816357	12.5094306	4.56525506
Agbl4	7.02889616	0.16062552	0.38912168	0.41278995	0.67976051	0.99938111	Agbl4	10.0806949	4.97776091	6.36158069	4.13628002	8.3240586	5.88027262	9.62263889	6.84788259
C3	0.43458489	0.10937426	0.26497109	0.41277809	0.67976921	NA	C3	0.67204633	0.45252372	0	0	0	2.35210905	0	0
Card14	0.43458489	0.10937426	0.26497109	0.41277809	0.67976921	NA	Card14	0.67204633	0.45252372	0	0	0	2.35210905	0	0
Ace2	0.22009101	-0.0987307	0.23923482	-0.4126937	0.67983101	NA	Ace2	0.67204633	0.45252372	0.63615807	0	0	0	0	0
Aqp9	0.22009101	-0.0987307	0.23923482	-0.4126937	0.67983101	NA	Aqp9	0.67204633	0.45252372	0.63615807	0	0	0	0	0
Pole2	0.22009101	-0.0987307	0.23923482	-0.4126937	0.67983101	NA	Pole2	0.67204633	0.45252372	0.63615807	0	0	0	0	0
Prss43	0.22009101	-0.0987307	0.23923482	-0.4126937	0.67983101	NA	Prss43	0.67204633	0.45252372	0.63615807	0	0	0	0	0
Rbm47	0.22009101	-0.0987307	0.23923482	-0.4126937	0.67983101	NA	Rbm47	0.67204633	0.45252372	0.63615807	0	0	0	0	0
Sftpc	0.22009101	-0.0987307	0.23923482	-0.4126937	0.67983101	NA	Sftpc	0.67204633	0.45252372	0.63615807	0	0	0	0	0
Tbc1d10c	0.22009101	-0.0987307	0.23923482	-0.4126937	0.67983101	NA	Tbc1d10c	0.67204633	0.45252372	0.63615807	0	0	0	0	0
Usp18	0.22009101	-0.0987307	0.23923482	-0.4126937	0.67983101	NA	Usp18	0.67204633	0.45252372	0.63615807	0	0	0	0	0
Itch	58.3780935	0.13635834	0.33041151	0.41269245	0.67983196	0.99938111	Itch	56.4518916	57.0179886	72.8400989	33.0902402	33.2962344	98.78858	72.1697916	43.3699231
Gaa	389.949657	0.13741928	0.33307779	0.41257412	0.67991866	0.99938111	Gaa	256.049651	484.652903	319.987509	405.355442	511.097198	496.295009	146.264111	499.895429
Mospd2	7.70487183	0.1717365	0.41636053	0.41247067	0.67999448	0.99938111	Mospd2	4.7043243	9.5029981	10.8146872	2.06814001	3.32962344	23.5210905	7.69811111	0
Khk	3.79987499	0.16975095	0.41175884	0.41225819	0.68015019	NA	Khk	1.34409266	1.81009488	1.27231614	10.3407001	8.3240586	1.17605452	3.84905555	2.28262753
Cul3	124.982834	0.16013378	0.38843902	0.41224947	0.68015658	0.99938111	Cul3	142.473822	142.997495	138.36438	35.1583802	56.6035985	218.746141	210.735792	54.7830607
Tnni3k	0.55644418	-0.1251873	0.30376562	-0.4121181	0.68025285	NA	Tnni3k	2.01613899	0.45252372	0.31807903	0	1.66481172	0	0	0
Hlcs	12.6767199	0.152423	0.3698551	0.41211545	0.68025481	0.99938111	Hlcs	14.7850192	11.313093	14.3135566	4.13628002	21.6425524	10.5844907	8.660375	15.9783927
1700028K03Rik	0.97929082	0.15219913	0.36934054	0.41208346	0.68027825	NA	1700028K03Ri	1.34409266	0.45252372	1.27231614	0	1.66481172	1.17605452	1.92452778	0
Map2k2	45.9792364	0.15277367	0.37074626	0.41207069	0.68028761	0.99938111	Map2k2	41.6668724	30.3190892	34.0344567	64.1123403	61.5980337	18.8168724	25.981125	91.3051012
Gpd2	74.7349938	0.10914435	0.26487105	0.41206597	0.68029107	0.99938111	Gpd2	81.9896521	71.9512713	76.9751264	55.8397803	81.5757743	90.5561984	93.3395972	45.6525506
Slc30a3	63.8044877	-0.1648289	0.40006477	-0.4120055	0.6803354	0.99938111	Slc30a3	57.1239379	119.466262	63.9338859	35.1583802	124.860879	24.697145	14.4339583	70.7614535
C330006A16Rik	73.0918718	-0.1643719	0.39901274	-0.4119465	0.68037863	0.99938111	C330006A16Ri	61.1562159	54.3028463	35.9429309	167.519341	98.2238915	18.8168724	34.6415	114.131377
Plekha5	22.4341571	-0.1377074	0.33431646	-0.4119074	0.68040726	0.99938111	Plekha5	32.9302701	21.2686148	23.5378486	18.6132601	16.6481172	22.345036	32.7169722	11.4131377
Zbtb2	6.98752565	-0.1714929	0.41635521	-0.4118908	0.68041945	0.99938111	Zbtb2	12.0968339	9.05047438	8.90621297	0	0	10.5844907	3.84905555	11.4131377
Fam103a1	28.8899857	0.13201416	0.3205813	0.4117962	0.6804888	0.99938111	Fam103a1	30.9141311	27.6039469	28.9451921	22.7495401	16.6481172	42.3379629	41.3773472	20.5436478
Galnt16	111.454026	-0.1265247	0.30726933	-0.4117712	0.68050715	0.99938111	Galnt16	94.086486	177.389298	104.648002	90.9981605	131.520126	132.894161	59.6603611	100.435611
Noc3l	7.68967693	-0.1648218	0.40041034	-0.4116323	0.68060893	0.99938111	Noc3l	14.1129729	4.52523719	10.4966081	4.13628002	3.32962344	9.40843619	8.660375	6.84788259
Ahctf1	29.1364645	0.15970306	0.38797734	0.41162988	0.68061073	0.99938111	Ahctf1	29.5700384	33.4867552	35.3067728	8.27256004	28.3017992	57.6266717	33.6792361	6.84788259
Ifrd1	10.3197656	-0.1538258	0.37407427	-0.4112174	0.68091316	0.99938111	Ifrd1	10.7527413	15.8383302	11.4508452	6.20442003	6.65924688	9.40843619	15.3962222	6.84788259
Triobp	6.39154305	0.16685373	0.405779	0.4111936	0.68093058	0.99938111	Triobp	6.72046328	7.2403795	7.63389683	0	13.3184938	5.88027262	5.77358333	4.56525506
Cacna1d	29.2288632	0.14100786	0.34297218	0.41113496	0.68097358	0.99938111	Cacna1d	29.5700384	31.6766603	32.4440615	14.4769801	31.6314227	52.9224536	18.2830139	22.8262753
Nlgn1	73.7196479	-0.1512339	0.36787715	-0.411099	0.68099992	0.99938111	Nlgn1	69.2207718	126.706641	88.1078926	31.0221002	68.2572805	123.485725	50.9999861	31.9567854
Scd3	3.2308829	-0.1684835	0.40995121	-0.4109843	0.68108405	NA	Scd3	4.03227797	5.43028463	0.9542371	4.13628002	6.65924688	2.35210905	0	2.28262753
Cdca4	3.50422124	0.17065738	0.41525235	0.41097271	0.68109255	NA	Cdca4	3.36023164	3.16766603	2.22655324	4.13628002	6.65924688	2.35210905	3.84905555	2.28262753
Canx	228.404196	0.16212077	0.39449287	0.41095994	0.68110191	0.99938111	Canx	161.291119	322.649412	260.824808	90.9981605	119.866444	585.675153	199.188625	86.7398462
Tiprl	29.2023238	0.13218018	0.32175357	0.41081185	0.6812105	0.99938111	Tiprl	23.5216215	21.7211385	24.4920857	43.4309402	28.3017992	18.8168724	41.3773472	31.9567854
Fdft1	29.2148139	-0.1165429	0.28386652	-0.4105552	0.68139874	0.99938111	Fdft1	26.8818531	28.0564706	35.3067728	33.0902402	29.966611	32.9295267	26.9433889	20.5436478
4732456N10Rik	0.55679488	-0.0961336	0.23415534	-0.4105548	0.68139898	NA	4732456N10R	0	0	0.31807903	4.13628002	0	0	0	0
5033406O09Rik	0.55679488	-0.0961336	0.23415534	-0.4105548	0.68139898	NA	5033406O09R	0	0	0.31807903	4.13628002	0	0	0	0
Chrnb4	0.55679488	-0.0961336	0.23415534	-0.4105548	0.68139898	NA	Chrnb4	0	0	0.31807903	4.13628002	0	0	0	0
Pipox	0.55679488	-0.0961336	0.23415534	-0.4105548	0.68139898	NA	Pipox	0	0	0.31807903	4.13628002	0	0	0	0
Tdgf1	0.55679488	-0.0961336	0.23415534	-0.4105548	0.68139898	NA	Tdgf1	0	0	0.31807903	4.13628002	0	0	0	0
Tmem82	0.55679488	-0.0961336	0.23415534	-0.4105548	0.68139898	NA	Tmem82	0	0	0.31807903	4.13628002	0	0	0	0
Ubap1l	0.55679488	-0.0961336	0.23415534	-0.4105548	0.68139898	NA	Ubap1l	0	0	0.31807903	4.13628002	0	0	0	0
Fam174b	21.9598318	-0.1269736	0.30952393	-0.4102221	0.68164304	0.99938111	Fam174b	18.1452509	26.6988994	20.3570582	26.8858201	23.3073641	18.8168724	16.3584861	25.1089028
Zfp955a	7.6623367	0.16957835	0.41338642	0.41021753	0.68164639	0.99938111	Zfp955a	7.39250961	7.2403795	6.04350166	8.27256004	1.66481172	15.2887088	15.3962222	0
Rock2	191.797481	0.16303563	0.39749159	0.41016121	0.68168769	0.99938111	Rock2	199.59776	222.189146	236.968881	41.3628002	96.5590798	386.921938	280.018792	70.7614535
5330434G04Rik	8.57591519	-0.1645991	0.40136206	-0.4101013	0.68173167	0.99938111	5330434G04R	6.72046328	11.313093	10.1785291	8.27256004	0	9.40843619	6.73584722	15.9783927
2610035F20Rik	0.55228815	0.11323438	0.27628171	0.40985117	0.68191513	NA	2610035F20Ri	0	0.45252372	0.63615807	0	3.32962344	0	0	0
Otop2	0.55228815	0.11323438	0.27628171	0.40985117	0.68191513	NA	Otop2	0	0.45252372	0.63615807	0	3.32962344	0	0	0
B3galt2	22.9412003	-0.1680862	0.41015201	-0.4098143	0.68194214	0.99938111	B3galt2	19.4893435	41.1796584	35.6248519	4.13628002	3.32962344	35.2816357	30.7924444	13.6957652
Gfer	15.0200355	0.14537556	0.35487443	0.40965352	0.68206013	0.99938111	Gfer	12.0968339	11.313093	10.8146872	22.7495401	14.9833055	10.5844907	12.5094306	25.1089028
E130311K13Rik	3.0976504	0.17056026	0.41637079	0.40963551	0.68207335	NA	E130311K13Ri	2.01613899	4.07271347	2.86271131	2.06814001	4.99443516	5.88027262	2.88679167	0
Grb10	77.9878994	0.1142303	0.2788604	0.40963254	0.68207552	0.99938111	Grb10	67.8766792	54.3028463	64.570044	115.815841	64.9276571	97.6125255	78.9056389	79.8919636
Irx6	0.38228317	-0.1047991	0.25592723	-0.4094879	0.68218163	NA	Irx6	0.67204633	0	0.31807903	2.06814001	0	0	0	0
Klhl41	0.38228317	-0.1047991	0.25592723	-0.4094879	0.68218163	NA	Klhl41	0.67204633	0	0.31807903	2.06814001	0	0	0	0
Polg2	0.38228317	-0.1047991	0.25592723	-0.4094879	0.68218163	NA	Polg2	0.67204633	0	0.31807903	2.06814001	0	0	0	0
Rtbdn	0.38228317	-0.1047991	0.25592723	-0.4094879	0.68218163	NA	Rtbdn	0.67204633	0	0.31807903	2.06814001	0	0	0	0
Slc2a9	0.38228317	-0.1047991	0.25592723	-0.4094879	0.68218163	NA	Slc2a9	0.67204633	0	0.31807903	2.06814001	0	0	0	0
Ccdc17	1.96591984	-0.1669322	0.40771886	-0.4094297	0.68222435	NA	Ccdc17	1.34409266	2.71514231	1.59039517	4.13628002	1.66481172	2.35210905	1.92452778	0
Wdr35	28.4659982	0.12212769	0.29836887	0.4093178	0.68230645	0.99938111	Wdr35	36.9625481	24.4362808	27.672876	18.6132601	29.966611	36.4576902	30.7924444	22.8262753
Exosc2	7.91321366	-0.1632955	0.39898036	-0.4092822	0.6823326	0.99938111	Exosc2	7.39250961	4.97776091	5.72542262	18.6132601	4.99443516	7.05632714	7.69811111	6.84788259

GeneID	Base mean	log2(FC)	StdErr	Wald-Stats	P-value	P-adj	GeneID	Normalised expression for Chow#1	Normalised expression for Chow#2	Normalised expression for Chow#3	Normalised expression for Chow#4	Normalised expression for HFD#1	Normalised expression for HFD#2	Normalised expression for HFD#3	Normalised expression for HFD#4
Vit	1.84162438	0.15825256	0.38667024	0.40927009	0.68234146	NA	Vit	0.67204633	1.81009488	0.31807903	4.13628002	1.66481172	0	3.84905555	2.28262753
Nme1	118.944656	0.11350042	0.27737464	0.40919539	0.68239628	0.99938111	Nme1	99.4628566	89.1471726	116.416927	150.974221	118.201632	75.2674895	149.150903	152.936045
Arsk	4.20653431	-0.169681	0.41469169	-0.4091738	0.68241213	NA	Arsk	4.03227797	7.69290322	5.08926455	2.06814001	3.32962344	4.7042181	6.73584722	0
Cdh22	36.7231009	0.14509843	0.35469956	0.40907416	0.68248524	0.99938111	Cdh22	20.1613899	43.8948007	27.354797	45.4990802	64.9276571	27.0492541	19.2452778	45.6525506
Skp2	1.82178812	0.1616819	0.39550666	0.40879691	0.68268871	NA	Skp2	0.67204633	1.81009488	3.18079035	0	0	4.7042181	1.92452778	2.28262753
Dkk3	38.8471158	-0.1407798	0.34443086	-0.4087316	0.68273665	0.99938111	Dkk3	25.5377605	66.5209867	49.6203294	20.6814001	48.2795399	37.6337448	25.981125	36.5220405
Kcnu1	2.04156234	-0.1670216	0.40879373	-0.4085717	0.682854	NA	Kcnu1	3.36023164	2.26261859	2.22655324	2.06814001	0	3.52816357	2.88679167	0
Txn1	17.1803821	0.15641962	0.38289676	0.40851643	0.68289457	0.99938111	Txn1	18.1452509	7.69290322	23.8559276	14.4769801	13.3184938	12.9365998	35.6037639	11.4131377
4831440E17Rik	0.50799909	0.12234621	0.29952606	0.40846599	0.68293159	NA	831440E17Ri	0	0.90504744	0.31807903	0	1.66481172	1.17605452	0	0
Oxgr1	0.50799909	0.12234621	0.29952606	0.40846599	0.68293159	NA	Oxgr1	0	0.90504744	0.31807903	0	1.66481172	1.17605452	0	0
Adam10	44.923838	0.15843337	0.38801387	0.40831882	0.68303962	0.99938111	Adam10	36.2905017	64.7108918	47.3937761	16.5451201	29.966611	102.316744	46.1886666	15.9783927
Ten1	10.1749761	-0.1657307	0.405902	-0.4083023	0.68305172	0.99938111	Ten1	15.4570656	4.97776091	10.1785291	14.4769801	8.3240586	1.17605452	15.3962222	11.4131377
1600002H07Rik	2.52378376	0.16914657	0.41465928	0.407917	0.68333461	NA	600002H07Ri	2.01613899	1.81009488	1.59039517	4.13628002	3.32962344	1.17605452	3.84905555	2.28262753
Slco1a4	3.79458302	-0.1511047	0.37054177	-0.4077941	0.68342488	NA	Slco1a4	0.67204633	11.7656167	5.08926455	0	11.653682	1.17605452	0	0
Phf20l1	21.2214684	0.14527575	0.35653308	0.40746781	0.68366444	0.99938111	Phf20l1	32.9302701	18.5534725	17.4943469	10.3407001	13.3184938	29.4013631	31.7547083	15.9783927
Cyp2j12	0.25370219	-0.0845197	0.20746234	-0.4073976	0.68371602	NA	Cyp2j12	0.67204633	1.35757116	0	0	0	0	0	0
Gm694	0.25370219	-0.0845197	0.20746234	-0.4073976	0.68371602	NA	Gm694	0.67204633	1.35757116	0	0	0	0	0	0
Pfas	18.0374742	0.1526049	0.37467999	0.40729396	0.6837921	0.99938111	Pfas	14.7850192	9.5029981	15.2677937	28.9539601	33.2962344	8.23238167	18.2830139	15.9783927
Snx9	5.27032288	-0.1692093	0.4154557	-0.4072861	0.68379786	0.99938111	Snx9	12.0968339	5.88280835	3.81694841	2.06814001	3.32962344	8.23238167	6.73584722	0
Ptrh2	10.6453953	-0.150125	0.36869132	-0.4071833	0.68387334	0.99938111	Ptrh2	12.0968339	8.59795066	16.5401098	8.27256004	8.3240586	12.9365998	11.5471667	6.84788259
Abca5	39.7042148	0.15357522	0.37732083	0.40701496	0.683997	0.99938111	Abca5	32.9302701	43.442277	55.345752	14.4769801	23.3073641	85.8519803	39.4528194	22.8262753
Zfp81	9.13874819	0.15684949	0.38537623	0.40700355	0.68400538	0.99938111	Zfp81	10.0806949	8.14542694	8.58813393	8.27256004	6.65924688	11.7605452	17.32075	2.28262753
Ptchd1	16.1100228	0.14922982	0.3667764	0.40686865	0.68410447	0.99938111	Ptchd1	13.4409266	16.2908539	20.3570582	8.27256004	9.98887032	29.4013631	10.5849028	20.5436478
Uchl1os	4.91447779	-0.1691789	0.41606613	-0.4066154	0.6842905	0.99938111	Uchl1os	2.01613899	5.88280835	2.54463228	12.4088401	6.65924688	2.35210905	2.88679167	4.56525506
Eri3	129.953244	-0.12706	0.31248421	-0.4066127	0.68429249	0.99938111	Eri3	131.049034	106.343074	105.920318	204.745861	138.179373	64.6829988	110.660347	178.044947
Srgn	1.10613926	-0.1395246	0.34315479	-0.4065937	0.68430639	NA	Srgn	0	2.71514231	2.54463228	0	1.66481172	0	1.92452778	0
Ankrd17	166.99317	0.11476527	0.28228265	0.40656154	0.68433005	0.99938111	Ankrd17	161.291119	173.316584	171.126521	132.360961	134.849749	281.077031	165.509389	116.414004
AK129341	9.78316325	-0.16803	0.41335241	-0.4065055	0.68437119	0.99938111	AK129341	14.1129729	13.5757116	16.5401098	0	0	21.1689814	10.5849028	2.28262753
Ube3c	85.164949	0.09471829	0.23300818	0.406502	0.68437379	0.99938111	Ube3c	84.0057911	74.6664136	90.9706039	80.6574604	81.5757743	107.020962	96.2263889	66.1961984
Ift57	21.192641	-0.1509484	0.37147309	-0.4063509	0.6844848	0.99938111	Ift57	28.2259458	27.6039469	28.3090341	6.20442003	11.653682	28.2253086	27.9056528	11.4131377
Ap2m1	188.837047	-0.1180872	0.29060905	-0.4063439	0.68448996	0.99938111	Ap2m1	209.678454	168.338823	187.984709	223.359121	201.442218	95.2604165	152.999958	271.632676
Whamm	8.98370176	-0.1570316	0.38654131	-0.4062479	0.68456049	0.99938111	Whamm	8.73660227	7.69290322	7.63389683	14.4769801	6.65924688	5.88027262	4.81131944	15.9783927
Scarna2	1.69850828	-0.1557997	0.38363205	-0.4061176	0.68465619	NA	Scarna2	1.34409266	0.90504744	1.59039517	4.13628002	3.32962344	0	0	2.28262753
Tmem38a	58.938441	0.10404104	0.25624396	0.40602341	0.6847254	0.99938111	Tmem38a	46.3711967	60.1856546	52.8011197	66.1804803	58.2684102	56.4506172	49.0754583	82.1745911
Rab3a	692.300894	0.13152768	0.32404079	0.40589854	0.68481715	0.99938111	Rab3a	712.369108	462.026717	503.201033	936.867425	744.170839	379.865611	594.679083	1205.22734
Atp2b1	212.29278	-0.1630999	0.40186754	-0.4058549	0.68484921	0.99938111	Atp2b1	249.329188	364.281594	269.731021	41.3628002	108.212762	402.210647	192.452778	70.7614535
Laptm4b	24.6978157	0.11450876	0.28215935	0.40583011	0.68486744	0.99938111	Laptm4b	23.5216215	25.3413283	22.5836114	22.7495401	26.6369875	24.697145	26.9433889	25.1089028
Gm12942	0.69171275	0.12769342	0.31493334	0.40546174	0.68513814	NA	Gm12942	0.67204633	0	1.27231614	0	1.66481172	0	1.92452778	0
Cnot7	82.7735861	-0.139815	0.3449613	-0.4053063	0.68525234	0.99938111	Cnot7	98.7908103	91.4097912	104.648002	57.9079203	43.2851047	78.7956531	146.264111	41.0872956
0610010F05Rik	21.9137886	0.16357479	0.40359033	0.40529909	0.68525768	0.99938111	610010F05Ri	25.5377605	26.6988994	23.2197695	4.13628002	8.3240586	28.2253086	50.0377222	9.13051012
Rrm2b	31.5716623	0.15259584	0.37672616	0.40505771	0.68543509	0.99938111	Rrm2b	26.8818531	35.2968501	43.8949068	10.3407001	16.6481172	43.5140174	57.7358333	18.2610202
Tnfaip3	1.62371847	-0.1591405	0.39293657	-0.4050029	0.68547537	NA	Tnfaip3	4.03227797	1.81009488	1.90847421	0	0	2.35210905	2.88679167	0
Zfp235	7.74820568	-0.1568886	0.38738931	-0.4049895	0.68548525	0.99938111	Zfp235	13.4409266	6.33533207	7.31581779	6.20442003	6.65924688	9.40843619	5.77358333	6.84788259
Cdc25b	4.58915515	0.16768085	0.41411334	0.40491536	0.68553972	NA	Cdc25b	2.01613899	3.62018975	3.18079035	8.27256004	3.32962344	2.35210905	4.81131944	9.13051012
Exph5	7.09277421	0.1616067	0.39926511	0.40476038	0.68565366	0.99938111	Exph5	6.04841696	8.59795066	9.54237104	0	9.98887032	7.05632714	8.660375	6.84788259
4921531C22Rik	0.46342317	-0.1241053	0.30668083	-0.4046726	0.68571818	NA	921531C22Ri	0.67204633	0.90504744	0.9542371	0	0	1.17605452	0	0
Celrr	0.46342317	-0.1241053	0.30668083	-0.4046726	0.68571818	NA	Celrr	0.67204633	0.90504744	0.9542371	0	0	1.17605452	0	0
Tnfrsf12a	0.46342317	-0.1241053	0.30668083	-0.4046726	0.68571818	NA	Tnfrsf12a	0.67204633	0.90504744	0.9542371	0	0	1.17605452	0	0
Dhx40	22.18227	-0.1310274	0.32390256	-0.4045273	0.685825	0.99938111	Dhx40	20.8334362	18.1009488	31.1717454	24.8176801	19.9777406	19.9929269	28.8679167	13.6957652
Gstm4	7.95422818	-0.1625106	0.40199802	-0.4042571	0.68602364	0.99938111	Gstm4	6.72046328	4.97776091	6.67965972	18.6132601	3.32962344	5.88027262	10.5849028	6.84788259
Tbc1d1	15.692136	0.14210688	0.35157744	0.40419796	0.68606715	0.99938111	Tbc1d1	12.0968339	19.9110436	14.6316356	10.3407001	21.6425524	19.9929269	8.660375	18.2610202
Col8a1	1.04098063	-0.1476317	0.36533563	-0.4040987	0.68614014	NA	Col8a1	1.34409266	2.71514231	0.9542371	0	0	2.35210905	0.96226389	0
Ube2d2a	104.440628	-0.1086064	0.26877206	-0.4040835	0.68615128	0.99938111	Ube2d2a	109.543552	103.627932	100.194896	124.088401	63.2628454	81.1477622	139.528264	114.131377
Zfp939	2.48551389	-0.1638458	0.40577851	-0.4037814	0.68637345	NA	Zfp939	4.7043243	2.71514231	0.31807903	4.13628002	1.66481172	1.17605452	2.88679167	2.28262753
Ybx2	0.95819896	-0.138497	0.34304475	-0.4037287	0.68641224	NA	Ybx2	2.01613899	0	0.9542371	2.06814001	1.66481172	0	0.96226389	0
1110008L16Rik	5.84048636	-0.1618381	0.40121763	-0.4033674	0.68667797	0.99938111	110008L16Ri	6.04841696	5.43028463	8.2700549	6.20442003	1.66481172	5.88027262	8.660375	4.56525506
Atp6v1c1	204.306821	0.1029998	0.2553504	0.40336649	0.68667863	0.99938111	Atp6v1c1	215.054825	209.518482	211.522558	148.906081	164.81636	235.210905	289.64143	159.783927
Tti1	13.7933381	0.1470985	0.36478027	0.40325234	0.6867626	0.99938111	Tti1	15.4570656	14.9332827	16.5401098	2.06814001	11.653682	14.1126543	17.32075	18.2610202
Fdx1	5.3361123	-0.1671787	0.41459233	-0.4032365	0.68677428	0.99938111	Fdx1	2.68818531	3.62018975	4.13502745	14.4769801	3.32962344	4.7042181	2.88679167	6.84788259
Has3	1.85592906	0.1626155	0.40330657	0.40320567	0.68679693	NA	Has3	0.67204633	2.71514231	0.9542371	2.06814001	1.66481172	3.52816357	0.96226389	2.28262753
Hilpda	1.85592906	0.1626155	0.40330657	0.40320567	0.68679693	NA	Hilpda	0.67204633	2.71514231	0.9542371	2.06814001	1.66481172	3.52816357	0.96226389	2.28262753
Cd160	0.49534953	-0.1205085	0.29891707	-0.4031504	0.68683759	NA	Cd160	2.01613899	0.45252372	0.31807903	0	0	1.17605452	0	0
Mkrn1	53.9479464	0.12056933	0.29910933	0.40309451	0.6868787	0.99938111	Mkrn1	59.1400769	31.2241366	49.6203294	66.1804803	76.5813391	42.3379629	47.1509305	59.3483158
Rpl27a	24.222808	-0.1374486	0.34103181	-0.4030586	0.68690514	0.99938111	Rpl27a	34.9464091	25.3413283	24.8101647	18.6132601	11.653682	25.8731995	36.5660278	15.9783927
Zfp62	24.7422399	0.16465723	0.40868032	0.40289982	0.68702192	0.99938111	Zfp62	31.5861774	28.0564706	23.8559276	6.20442003	6.65924688	44.6900719	50.0377222	6.84788259
Pcnp	79.1693889	-0.1434597	0.35608056	-0.4028855	0.68703246	0.99938111	Pcnp	81.9896521	100.077742	83.018628	74.4530404	31.6314227	102.316744	127.981097	31.9567854
Hectd2	15.5438167	0.15039227	0.37336893	0.40279805	0.6870968	0.99938111	Hectd2	14.1129729	15.3858064	21.6293743	6.20442003	9.98887032	19.9929269	27.9056528	9.13051012
Secisbp2	8.68536424	0.15335246	0.38114501	0.40234676	0.68742885	0.99938111	Secisbp2	10.0806949	4.97776091	6.36158069	12.4088401	9.98887032	8.23238167	10.5849028	6.84788259
Cd163	0.79524716	-0.1337002	0.33245238	-0.4021636	0.68756364	NA	Cd163	2.01613899	1.35757116	0.63615807	0	0	2.35210905	0	0
2610301B20Rik	19.5533477	-0.1282085	0.31889623	-0.4020383	0.68765587	0.99938111	610301B20Ri	20.8334362	19.9110436	25.4463228	16.5451201	14.9833055	19.9929269	25.0188611	13.6957652
Fzr1	74.2474852	0.16182241	0.40250976	0.4020335	0.68765937	0.99938111	Fzr1	48.3873356	34.3918026	41.9864326	124.088401	113.207197	18.8168724	44.2641389	146.088162
Brd1	38.7588094	0.10955028	0.27251465	0.40199775	0.68768568	0.99938111	Brd1	39.6507334	30.7716129	31.8079035	47.5672202	39.9554813	32.9295267	39.4528194	47.9351782
2810429I04Rik	0.70535737	0.12536504	0.31186591	0.40198378	0.68769597	NA	810429I04Ri	1.34409266	0.45252372	0.31807903	0	0	3.52816357	0	0
Ddb2	5.93639555	0.16484386	0.41047565	0.4015923	0.6879841	0.99938111	Ddb2	6.72046328	3.62018975	3.81694841	8.27256004	0	8.23238167	7.69811111	9.13051012
Ift46	30.8664945	-0.1182404	0.29460842	-0.4013477	0.68816415	0.99938111	Ift46	38.978687	24.8888045	27.990955	39.2946602	23.3073641	25.8731995	34.6415	31.9567854
Khdrbs3	81.5697215	0.14508638	0.36154785	0.40119248	0.68827842	0.99938111	Khdrbs3	100.134903	95.9350284	80.7920748	26.8858201	51.6091633	82.3238167	157.811278	57.0656883
Rn45s	3840534.57	-0.1218934	0.30397685	-0.4009958	0.68842321	0.99938111	Rn45s	4773355.55	2666296.9	3294563.72	5398314.89	4818582.76	2317346.05	2609042.85	4846773.8
Myo9b	9.33288281	0.15807654	0.39427245	0.40093224	0.68847001	0.99938111	Myo9b	5.37637063	8.14542694	8.58813393	12.4088401	21.6425524	5.88027262	5.77358333	6.84788259
Rnaseh2a	8.57458935	-0.1658903	0.41392601	-0.4007728	0.68858737	0.99938111	Rnaseh2a	6.72046328	5.43028463	2.86271131	24.8176801	11.653682	3.52816357	6.73584722	6.84788259
Sec14l3	1.21813694	0.1530385	0.38193179	0.40069591	0.68864403	NA	Sec14l3	0.67204633	0.90504744	2.22655324	0	1.66481172	2.35210905	1.92452778	0
BC018507	60.1446224	0.12214667	0.30507033	0.40038856	0.68887035	0.99938111	BC018507	60.4841696	66.9735104	60.1169375	41.3628002	66.5924688	87.0280348	71.2075278	27.3915304
Cnot11	26.8135219	0.11783115	0.29477722	0.3997295	0.68935576	0.99938111	Cnot11	30.9141311	22.6261859	26.4005599	22.7495401	23.3073641	25.8731995	37.5282917	25.1089028
Ryr3	16.4841885	0.1472547	0.3684707	0.39963746	0.68942356	0.99938111	Ryr3	26.2098068	11.7656167	15.9039517	6.20442003	28.3017992	17.6408179	14.4339583	11.4131377
Tfcp2l1	2.99958361	-0.1651962	0.41336976	-0.3996329	0.68942689	NA	Tfcp2l1	4.03227797	4.52523719	2.86271131	2.06814001	6.65924688	0	3.84905555	0
Thbs2	2.77224044	0.16592039	0.41533263	0.39948797	0.68953369	NA	Thbs2	2.01613899	3.62018975	2.54463228	2.06814001	1.66481172	3.52816357	6.73584722	0
H1fx	37.2760666	0.15860294	0.39713276	0.39937008	0.68962054	0.99938111	H1fx	29.5700384	22.1736622	19.4028211	66.1804803	43.2851047	8.23238167	31.7547083	77.6093361
Galnt12	0.44118538	-0.1194748	0.29933282	-0.3991371	0.68979219	NA	Galnt12	1.34409266	0.90504744	0.31807903	0	0	0	0.96226389	0
Slfn8	0.44118538	-0.1194748	0.29933282	-0.3991371	0.68979219	NA	Slfn8	1.34409266	0.90504744	0.31807903	0	0	0	0.96226389	0
Zbtb14	19.0991138	0.12375822	0.31008069	0.39911617	0.68980761	0.99938111	Zbtb14	17.4732045	17.1959013	21.9474534	14.4769801	23.3073641	17.6408179	20.2075417	20.5436478
Zfp617	19.7252137	0.16288144	0.40811317	0.39910852	0.68981325	0.99938111	Zfp617	24.8657142	14.9332827	23.8559276	8.27256004	3.32962344	35.2816357	40.4150833	6.84788259
Prdx3	42.3811789	0.12340587	0.30921335	0.39909618	0.68982233	0.99938111	Prdx3	38.978687	30.7716129	49.6203294	43.4309402	36.6258578	36.4576902	71.2075278	31.9567854
Tars	14.094464	0.13815827	0.34622035	0.39904721	0.68985842	0.99938111	Tars	16.1291119	13.1231878	13.9954775	10.3407001	11.653682	15.2887088	23.0943333	9.13051012
Yod1	13.9452155	-0.1451318	0.3637106	-0.3990309	0.68987041	0.99938111	Yod1	14.7850192	17.1959013	19.4028211	8.27256004	9.98887032	12.9365998	22.1320694	6.84788259
Zfp108	2.2960458	-0.1638767	0.41069618	-0.3990216	0.68987727	NA	Zfp108	3.36023164	1.81009488	3.18079035	2.06814001	0	4.7042181	0.96226389	2.28262753
Cnot4	39.6956498	-0.1448002	0.36290895	-0.3989988	0.6898941	0.99938111	Cnot4	43.6830113	50.6826565	47.0756971	28.9539601	11.653682	48.2182355	64.4716805	22.8262753
BC002163	38.091337	0.14175935	0.35529968	0.39898531	0.68990403	0.99938111	BC002163	57.1239379	35.7493738	27.672876	22.7495401	21.6425524	61.1548353	55.8113055	22.8262753
Ppp4r2	33.9252535	0.10891286	0.27309452	0.39881013	0.69003311	0.99938111	Ppp4r2	27.5538995	36.2018975	34.9886938	31.0221002	26.6369875	39.9858538	38.4905555	36.5220405
Pou2f1	12.5406555	-0.1422	0.35662118	-0.3987424	0.69008302	0.99938111	Pou2f1	12.7688802	17.648425	9.224292	14.4769801	9.98887032	9.40843619	15.3962222	11.4131377
Spata2l	22.8180273	-0.1548029	0.38823586	-0.3987342	0.69008904	0.99938111	Spata2l	41.6668724	17.1959013	20.3570582	18.6132601	48.2795399	10.5844907	14.4339583	11.4131377
Gkap1	4.86249189	0.16584455	0.41593147	0.39872889	0.69009298	0.99938111	Gkap1	8.06455594	4.07271347	5.08926455	0	4.99443516	7.05632714	9.62263889	0
Snrnp48	4.91437183	-0.1658716	0.41603471	-0.3986966	0.69011681	0.99938111	Snrnp48	7.39250961	9.05047438	5.40734359	0	0	9.40843619	5.77358333	2.28262753
Tbpl1	48.6837684	0.12022798	0.30168725	0.39851861	0.69024795	0.99938111	Tbpl1	38.3066407	59.2806072	47.7118552	39.2946602	26.6369875	50.5703445	68.3207361	59.3483158
Aimp2	16.696363	-0.1277801	0.32066247	-0.3984879	0.69027055	0.99938111	Aimp2	15.4570656	17.1959013	15.9039517	22.7495401	13.3184938	15.2887088	15.3962222	18.2610202
Ints8	23.9825524	-0.134841	0.33841673	-0.3984466	0.69030103	0.99938111	Ints8	32.9302701	31.6766603	25.7644018	10.3407001	21.6425524	31.7534722	24.0565972	13.6957652
Gpr123	109.338418	0.11318705	0.28424133	0.39820756	0.69047719	0.99938111	Gpr123	64.5164475	127.611689	107.828793	115.815841	121.531256	102.316744	79.8679027	155.218672
Zfp46	35.6282689	0.12278712	0.30842967	0.39810412	0.69055344	0.99938111	Zfp46	39.6507334	32.5817078	31.8079035	31.0221002	61.5980337	37.6337448	27.9056528	22.8262753
2410006H16Rik	7.39902615	0.165723	0.41629222	0.39809295	0.69056167	0.99938111	410006H16Ri	3.36023164	2.71514231	2.86271131	18.6132601	3.32962344	2.35210905	7.69811111	18.2610202
Cacng5	12.7870055	-0.1409607	0.35409656	-0.3980854	0.69056725	0.99938111	Cacng5	12.7688802	15.8383302	15.9039517	8.27256004	11.653682	16.4647633	7.69811111	13.6957652
Dstn	87.6897011	0.1392349	0.34978024	0.39806395	0.69058305	0.99938111	Dstn	96.102625	101.4097912	92.24292	49.6353602	43.2851047	124.66178	153.962222	50.2178057
Pcdh7	114.706547	0.14673883	0.36867662	0.39801502	0.69061911	0.99938111	Pcdh7	104.167181	165.623681	114.826531	41.3628002	94.8942681	241.091177	89.4905416	66.1961984
Cycs	52.1474121	0.16562976	0.41634963	0.39781412	0.69076721	0.99938111	Cycs	53.7637063	63.8058444	62.6615698	4.13628002	4.99443516	83.4998712	126.056569	18.2610202
1810032O08Rik	0.24304531	-0.082332	0.20699409	-0.3977506	0.690814	NA	810032O08R	0.67204633	0	1.27231614	0	0	0	0	0

GeneID	Base mean	log2(FC)	StdErr	Wald-Stats	P-value	P-adj	GeneID	Normalised expression for Chow#1	Normalised expression for Chow#2	Normalised expression for Chow#3	Normalised expression for Chow#4	Normalised expression for HFD#1	Normalised expression for HFD#2	Normalised expression for HFD#3	Normalised expression for HFD#4
Frrs1	0.24304531	-0.082332	0.20699409	-0.3977506	0.690814	NA	Frrs1	0.67204633	0	1.27231614	0	0	0	0	0
Map4k2	36.1785836	0.13704499	0.34460305	0.39768943	0.69085913	0.99938111	Map4k2	32.9302701	23.9837571	24.1740066	55.8397803	36.6258578	21.1689814	30.7924444	63.9135709
Zfp28	5.0752502	0.16552145	0.41634431	0.39755905	0.69095525	0.99938111	Zfp28	3.36023164	1.35757116	4.77118552	10.3407001	6.65924688	3.52816357	10.5849028	0
Ankrd55	9.61901546	0.15649049	0.3937177	0.39746876	0.69102182	0.99938111	Ankrd55	7.39250961	11.7656167	5.08926455	12.4088401	3.32962344	8.23238167	17.32075	11.4131377
L1cam	99.0376922	-0.1499496	0.37739919	-0.3973237	0.69112876	0.99938111	L1cam	96.102625	170.601442	125.32314	31.0221002	113.207197	145.830761	39.4528194	70.7614535
Clec2l	19.3441869	0.12492773	0.31469686	0.39697799	0.69138369	0.99938111	Clec2l	15.4570656	18.5534725	16.5401098	22.7495401	23.3073641	17.6408179	15.3962222	25.1089028
Dagla	72.4296858	0.1346576	0.33944934	0.39669426	0.69159293	0.99938111	Dagla	50.4034746	102.27036	63.9338859	53.7716403	131.520126	64.6829988	37.5282917	75.3267085
Emb	17.1133036	0.15143505	0.38176958	0.39666609	0.69161371	0.99938111	Emb	18.8172972	18.1009488	18.130505	8.27256004	8.3240586	38.8097993	17.32075	9.13051012
Farsb	41.2901156	-0.1342623	0.3384869	-0.3966544	0.69162233	0.99938111	Farsb	50.4034746	40.274611	54.3915149	31.0221002	33.2962344	44.6900719	62.5471528	13.6957652
Msantd3	4.56832067	-0.1644294	0.41456755	-0.3966286	0.69164134	NA	Msantd3	7.39250961	2.71514231	5.72542262	4.13628002	0	5.88027262	3.84905555	6.84788259
Synj2	67.8674602	0.11456417	0.28911531	0.39625771	0.69191492	0.99938111	Synj2	78.6294204	70.1411764	69.3412295	39.2946602	93.2294563	83.4998712	65.4339444	43.3699231
Snapc5	7.65296262	-0.1648836	0.41616517	-0.3961975	0.69195934	0.99938111	Snapc5	16.8011582	7.69290322	9.54237104	0	6.65924688	7.05632714	13.4716944	0
Aldh1b1	4.29139139	0.16495428	0.41636385	0.39617819	0.69197358	NA	Aldh1b1	6.72046328	1.35757116	4.45310648	2.06814001	8.3240586	5.88027262	0.96226389	4.56525506
Lphn1	476.530639	0.12419817	0.31351742	0.39614439	0.69199851	0.99938111	Lphn1	344.08772	610.454497	420.500484	430.173122	747.500462	486.886573	227.094278	545.54798
2210416O15Rik	0.32508832	0.08914542	0.22505117	0.39611176	0.69202258	NA	2210416O15R	0	0	0.31807903	0	0	0	0	2.28262753
4931440P22Rik	0.32508832	0.08914542	0.22505117	0.39611176	0.69202258	NA	4931440P22Ri	0	0	0.31807903	0	0	0	0	2.28262753
Car3	0.32508832	0.08914542	0.22505117	0.39611176	0.69202258	NA	Car3	0	0	0.31807903	0	0	0	0	2.28262753
Catip	0.32508832	0.08914542	0.22505117	0.39611176	0.69202258	NA	Catip	0	0	0.31807903	0	0	0	0	2.28262753
Gm15350	0.32508832	0.08914542	0.22505117	0.39611176	0.69202258	NA	Gm15350	0	0	0.31807903	0	0	0	0	2.28262753
Mir7666	0.32508832	0.08914542	0.22505117	0.39611176	0.69202258	NA	Mir7666	0	0	0.31807903	0	0	0	0	2.28262753
Plekhg4	0.32508832	0.08914542	0.22505117	0.39611176	0.69202258	NA	Plekhg4	0	0	0.31807903	0	0	0	0	2.28262753
Uvrag	21.8305736	0.14343863	0.36213125	0.3960957	0.69203443	0.99938111	Uvrag	23.5216215	16.2908539	26.0824808	16.5451201	23.3073641	16.4647633	43.301875	9.13051012
Fcrls	4.53675082	-0.1618186	0.40872441	-0.3959112	0.69217054	NA	Fcrls	1.34409266	11.7656167	6.04350166	0	1.66481172	1.17605452	2.88679167	11.4131377
Adrb3	0.98064563	-0.1455836	0.3677208	-0.395908	0.69217291	NA	Adrb3	1.34409266	1.81009488	1.59039517	0	0	1.17605452	1.92452778	0
Zbtbd6	2.87216871	-0.1611975	0.40725336	-0.3958162	0.69224062	NA	Zbtbd6	1.34409266	1.35757116	2.86271131	8.27256004	3.32962344	3.52816357	0	2.28262753
Yeats2	9.73602977	0.15143247	0.382611	0.39578702	0.69226215	0.99938111	Yeats2	8.06455594	12.6706641	10.4966081	4.13628002	11.653682	17.6408179	8.660375	4.56525506
Zfp947	0.43009885	0.10607353	0.26807797	0.39568165	0.6923399	NA	Zfp947	0	0.45252372	0.63615807	0	0	2.35210905	0	0
Ankk1	0.24753134	-0.0825905	0.20875837	-0.3956272	0.69238006	NA	Ankk1	1.34409266	0	0.63615807	0	0	0	0	0
Itk	0.24753134	-0.0825905	0.20875837	-0.3956272	0.69238006	NA	Itk	1.34409266	0	0.63615807	0	0	0	0	0
Ribc1	0.24753134	-0.0825905	0.20875837	-0.3956272	0.69238006	NA	Ribc1	1.34409266	0	0.63615807	0	0	0	0	0
Tcea3	0.24753134	-0.0825905	0.20875837	-0.3956272	0.69238006	NA	Tcea3	1.34409266	0	0.63615807	0	0	0	0	0
Ppp2r4	213.826969	0.15831998	0.4001866	0.39561538	0.69238879	0.99938111	Ppp2r4	122.984478	108.605693	115.14461	436.377542	314.649415	71.739326	109.698083	431.416603
Dnm1l	145.139399	0.14440751	0.36503849	0.39559531	0.6924036	0.99938111	Dnm1l	173.387953	147.522732	158.403359	62.0442003	73.2517157	257.555941	213.622583	75.3267085
[truncated: 1,819,657 more chars]
