# Supplementary material for: Stress and high fat diet reconfigure the active translatome of CeA-NPY neurons
Source: Mol Metab. 2025 Jun 4;98:102176. doi: 10.1016/j.molmet.2025.102176 (PMC12214123; doi:10.1016/j.molmet.2025.102176)

GO annotation	Genes	Qvalue	Treatment	Source
Regulation of synaptic plasticity	64	5.3345E-11	HFD	SYNGO
synaptic transmission, glutamatergic	28	0.014806	HFD	SYNGO
Synaptic vesicle transport	51	2.6443E-06	HFD	SYNGO
regulation of synapse structure or activity	65	0.000015504	HFD	SYNGO
Learning or memory	77	1.03E-09	HFD	SYNGO
Regulation of lipid metabolic process	71	0.0149	HFD	SYNGO
Cellular lipid metabolic process	179	3.57E-09	HFD	Webgestait
Response to lipid	164	0.00002	HFD	Webgestait
Homeostatic process	48	0.00044783	HFD	Webgestait
Sodium ion transport	56	0.00000279	HFD	Webgestait
glutathione metabolic process	52	0.002	HFD	Webgestait
Action potential	33	0.039	HFD	Webgestait

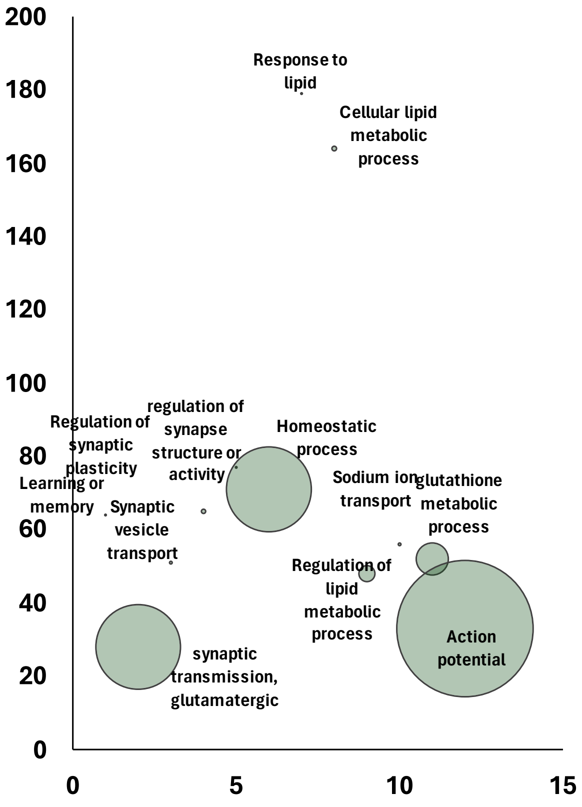

Supplement: Supplementary Table 2 — Functional ontology analysis of the HFD-induced differentially expressed genes. [file mmc2.pdf]
