# Supplementary material for: Stress and high fat diet reconfigure the active translatome of CeA-NPY neurons
Source: Mol Metab. 2025 Jun 4;98:102176. doi: 10.1016/j.molmet.2025.102176 (PMC12214123; doi:10.1016/j.molmet.2025.102176)
Supplement: Supplementary Table 3 — Functional ontology analysis of the HFDS-induced differentially expressed genes. [file mmc3.pdf]

GO annotation	Genes	Qvalue	Treatment	Source
Process in the synapses	56	0.000791	HFDS	SYNGO
Process in the pre-synapses	21	0.0413	HFDS	SYNGO
Post-synapses	41	0.0148	HFDS	SYNGO
Synaptic vesicle	12	0.0149	HFDS	SYNGO
synaptic vesicle membrane	11	0.0149	HFDS	SYNGO
integral component of presynaptic membrane	12	0.0149	HFDS	SYNGO
Positive regulation of synaptic transmission	23	0.0003204	HFDS	Webgestait
Regulation of neurotransmitter levels	34	0.00006682	HFDS	Webgestait
Anterograde trans-synaptic signalling	48	0.00044783	HFDS	Webgestait
chemical homeostasis	48	0.00044783	HFDS	Webgestait
L-amino acid transport	11	0.02	HFDS	Webgestait
Long-term synaptic potentiation	15	0.0012839	HFDS	Webgestait

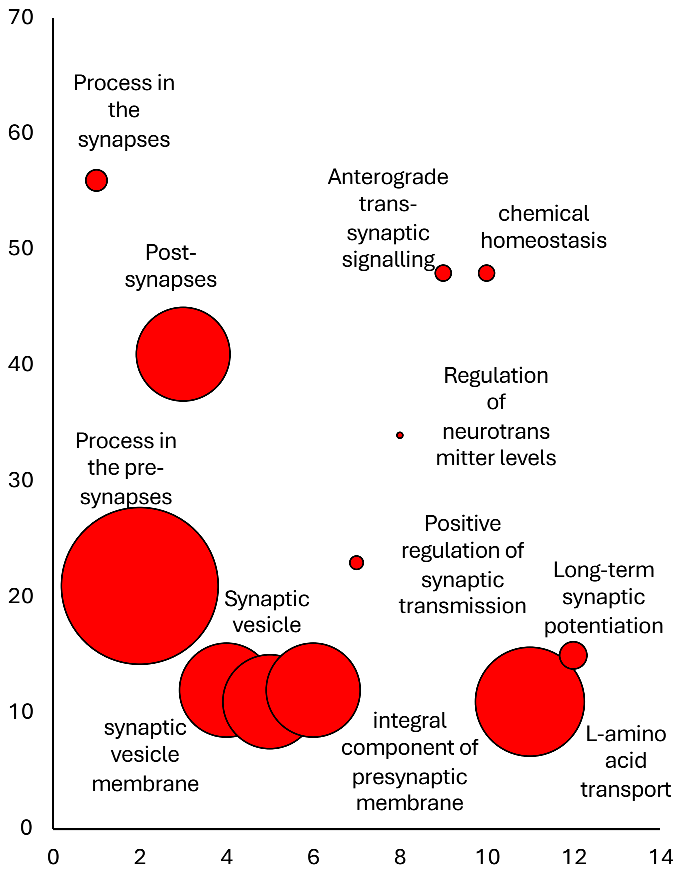

Supplementary Figure 3
