# Supplementary material for: Stress and high fat diet reconfigure the active translatome of CeA-NPY neurons
Source: Mol Metab. 2025 Jun 4;98:102176. doi: 10.1016/j.molmet.2025.102176 (PMC12214123; doi:10.1016/j.molmet.2025.102176)
Supplement: Supplementary Table 4 — Phenotypic ontology analysis of the HFDS-induced differentially expressed genes. [file mmc4.pdf]

GeneSet	description	link	size	overlap	expect	enrichmentR	pValue	FDR	overlapId	userId
MP:0003633	abnormal nervous system physiology	http://www.informatics.jax.org/searches/Phat.cgi?id=MP:0	1692	139	76.0797558	1.82703005	4.63E-14	2.18E-10	432530;1153	Ntsr2;Kcnj10;S1pr1;Ndrg2;Gpr37l1;Mobp;Cst3;Gstm1;Gfap;Avp;Hepacam;Scn4b;Apoe;Apod;Slc7a10;Agt;Lpar1;Mlc1;Tmem198b;Slc1a3;Gjb6;Nde1;Kcnj16;Slc38a3;Slc6a11;Fzd2;St3gal4;Mal;Drd2;Ucp2;F3;Pmp22;Tnfrsf1a;Slc17a6;Pcp4;Cd4;Dhrs3;Gpr17;S100a10;Plp1;Serpine2;Atp1a2;Mbp;Laptm4a;Csf1r;Pdyn;Panx1;Slc1a
MP:0002152	abnormal brain morphology	http://www.informatics.jax.org/searches/Phat.cgi?id=MP:0	1409	109	63.3548321	1.72046861	2.75E-09	1.30E-05	234734;4325	S1pr1;Slc9a3r1;Gpr37l1;Cst3;Gfap;Avp;Hepacam;Apoe;Ednrb;Agt;Lpar1;Gm2a;Mlc1;Slc1a3;Nde1;Bmp7;Drd2;Olig1;Tmem100;Ucp2;F3;Rxrg;Slc17a6;Rgma;B2m;Smo;Pcp4;Nfia;Zic1;Atp1b2;Plp1;Serpine2;Atp1a2;Mbp;Csf1r;Pdyn;Prex2;Slc1a2;Tlr3;Fa2h;Hexb;Trp53bp2;Mt3;Pou3f2;Cers2;Ptch1;Egr1;Olig2;Cables1;Fgfr2;Npc2
MP:0009956	abnormal cerebellar layer morphology	http://www.informatics.jax.org/searches/Phat.cgi?id=MP:0	208	29	9.3525941	3.100744	4.85E-08	2.29E-04	234734;7401	Gpr37l1;Gm2a;Slc1a3;Nfia;Atp1b2;Plp1;Serpine2;Pdyn;Prex2;Fa2h;Hexb;Cers2;Ptch1;Npc2;Qk;Nr1d1;Inpp4a;Myo5a;Aars;Herc1;Cit;Map1a;Bcl2l2;Ank1;Myh10;Trim2;Bicd2;Als2;Sptbn2
MP:0003635	abnormal synaptic transmission	http://www.informatics.jax.org/searches/Phat.cgi?id=MP:0	768	66	34.5326551	1.91123445	1.73E-07	8.13E-04	432530;1153	Ntsr2;Gstm1;Gfap;Scn4b;Slc7a10;Lpar1;Slc1a3;Gjb6;Slc38a3;Drd2;Ucp2;Tnfrsf1a;Slc17a6;S100a10;Serpine2;Atp1a2;Mbp;Laptm4a;Aqp4;Pdlim5;Cd81;Arpp21;Adora1;Cpe;Ptn;Th;Ly6e;Ptprz1;Igsf11;Efna3;Nrn1;Slc6a9;Gpr88;Epas1;Tspan7;Slc7a11;Arrdc3;Maf1;Kcnip3;Pde4b;Dlg2;Inpp4a;Myo5a;Cpeb3;Fez1;Adcy1;Mapk8ip3;
MP:0002882	abnormal neuron morphology	http://www.informatics.jax.org/searches/Phat.cgi?id=MP:0	1222	92	54.9464903	1.67435626	2.52E-07	0.00118395	234734;7401	Kcnj10;Gpr37l1;Cldn11;Cst3;Heyl;Hepacam;Apoe;Ednrb;Gm2a;Mlc1;Slc1a3;Gjb6;Nde1;Drd2;Olig1;Jam2;Pmp22;Slc17a6;Smo;Nfia;Atp1b2;Plp1;Mbp;Csf1r;Prex2;Panx1;Slc1a2;Tlr3;Fa2h;Hexb;Trp53bp2;Pou3f2;Rorb;Cers2;Ptch1;Olig2;Mertk;Nfe2l2;Fgfr2;Npc2;Qk;Ephb1;Lgi4;Phgdh;Rlbp1;Cd81;Hexa;Fgfr3;Gpm6b;Efna3;Nrn1;
MP:0000849	abnormal cerebellum morphology	http://www.informatics.jax.org/searches/Phat.cgi?id=MP:0	320	36	14.3886063	2.50197964	3.16E-07	0.00148509	234734;7401	Gpr37l1;Gm2a;Mlc1;Slc1a3;Olig1;Nfia;Zic1;Atp1b2;Plp1;Serpine2;Mbp;Pdyn;Prex2;Fa2h;Hexb;Cers2;Ptch1;Npc2;Qk;Phgdh;Nr1d1;Inpp4a;Myo5a;Aars;Herc1;Cit;Map1a;Bcl2l2;Ank1;Myh10;Trim2;Bicd2;Als2;Sptbn2;Sptbn4;Dnah5
MP:0004097	abnormal cerebellar cortex morphology	http://www.informatics.jax.org/searches/Phat.cgi?id=MP:0	242	30	10.8813835	2.75700236	4.00E-07	0.00188194	234734;7401	Gpr37l1;Gm2a;Slc1a3;Nfia;Zic1;Atp1b2;Plp1;Serpine2;Pdyn;Prex2;Fa2h;Hexb;Cers2;Ptch1;Npc2;Qk;Nr1d1;Inpp4a;Myo5a;Aars;Herc1;Cit;Map1a;Bcl2l2;Ank1;Myh10;Trim2;Bicd2;Als2;Sptbn2
MP:0011085	postnatal lethality, complete penetrance	http://www.informatics.jax.org/searches/Phat.cgi?id=MP:0	397	41	17.8508647	2.2968075	4.79E-07	0.00225356	11534;11733	Kcnj10;Avp;Ednrb;Gja1;Slc4a4;Slc38a3;Bmp7;F3;Slc17a6;Zic1;Lpcat3;Atp1b2;Plp1;Csf1r;Slc1a2;Trp53bp2;Pou3f2;Bag3;Ptch1;Fgfr2;Trf;Pdgfra;Th;Twsg1;Adk;Inpp4a;Myo5a;Camta1;Cit;Tcf4;Ryr2;Ank1;Myh10;Arx;Satb1;Sox6;Lhx6;Sp9;Unc13a;Plec;Maf
MP:0003871	abnormal myelin sheath morphology	http://www.informatics.jax.org/searches/Phat.cgi?id=MP:0	55	13	2.47304171	5.25668449	6.63E-07	0.00311864	11816;76893	Cldn11;Hepacam;Apoe;Mlc1;Pmp22;Plp1;Mbp;Fa2h;Cers2;Nfe2l2;Qk;Lgi4;Gpm6b
MP:0001392	abnormal locomotor behavior	http://www.informatics.jax.org/searches/Phat.cgi?id=MP:0	1310	95	58.9033571	1.6128113	8.65E-07	0.00406426	234734;7619	Sparc;Ntsr2;Slc9a3r1;Gpr37l1;Gstm1;Apoe;Apod;Slc7a10;Agt;Lpar1;Gatm;Clu;Fads1;Gjb6;Slc38a3;Drd2;Wwtr1;Ucp2;F3;Pmp22;Slc17a6;Zic1;Plp1;Atp1a2;Mbp;Csf1r;Pdyn;Prex2;Fa2h;Hexb;Trp53bp2;Rorb;Ctdsp1;Prg4;Chst8;Olig2;Timp3;Npc2;Qk;Lgi4;Pdlim5;Cd81;Htr1b;Pdgfra;Itpkb;Gprc5b;Cpe;Hexa;Fgfr3;Th;Slc6a9;Gpr88;
MP:0000847	abnormal metencephalon morphology	http://www.informatics.jax.org/searches/Phat.cgi?id=MP:0	349	37	15.6925738	2.35780316	9.44E-07	0.00443828	234734;7401	Gpr37l1;Gm2a;Mlc1;Slc1a3;Olig1;Nfia;Zic1;Atp1b2;Plp1;Serpine2;Mbp;Pdyn;Prex2;Fa2h;Hexb;Cers2;Ptch1;Npc2;Qk;Phgdh;Cntfr;Nr1d1;Inpp4a;Myo5a;Aars;Herc1;Cit;Map1a;Bcl2l2;Ank1;Myh10;Trim2;Bicd2;Als2;Sptbn2;Sptbn4;Dnah5
MP:0008415	abnormal neurite morphology	http://www.informatics.jax.org/searches/Phat.cgi?id=MP:0	322	35	14.4785351	2.41737163	1.05E-06	0.004935	74018;11816	Cldn11;Hepacam;Apoe;Mlc1;Jam2;Pmp22;Plp1;Mbp;Prex2;Tlr3;Fa2h;Cers2;Nfe2l2;Qk;Ephb1;Lgi4;Gpm6b;Nrn1;Tspan7;Nr1d1;Myo5a;Klc1;Cpeb3;Map1a;Bcl2l2;Gria1;Myh10;Trim2;Sptan1;Bicd2;Als2;Sptbn2;Elfn1;Sptbn4;Otud7a
MP:0002064	seizures	http://www.informatics.jax.org/searches/Phat.cgi?id=MP:0	311	34	13.9839268	2.43136285	1.32E-06	0.00619435	11816;11878	Gfap;Apoe;Slc7a10;Slc1a3;Slc6a11;St3gal4;Drd2;Pmp22;Plp1;Serpine2;Mbp;Pdyn;Panx1;Slc1a2;Hexb;Mt3;Bhlhe40;Qk;Pdgfra;Hexa;Myo5a;Cit;Ryr2;Gad2;Trim2;Arx;Cntnap4;Htr1a;Lhx6;Npy;Sptbn2;Elfn1;Otud7a;Kcnc2
MP:0003491	abnormal voluntary movement	http://www.informatics.jax.org/searches/Phat.cgi?id=MP:0	1405	99	63.1749746	1.56707621	1.80E-06	0.00845769	234734;7619	Sparc;Ntsr2;Kcnj10;Slc9a3r1;Gpr37l1;Gstm1;Apoe;Apod;Slc7a10;Agt;Lpar1;Gatm;Clu;Fads1;Slc1a3;Gjb6;Slc38a3;Drd2;Wwtr1;Ucp2;F3;Pmp22;Slc17a6;Zic1;Atp1b2;Plp1;Atp1a2;Mbp;Csf1r;Pdyn;Prex2;Fa2h;Hexb;Trp53bp2;Rorb;Ctdsp1;Prg4;Chst8;Olig2;Timp3;Npc2;Qk;Lgi4;Pdlim5;Cd81;Htr1b;Pdgfra;Itpkb;Gprc5b;Cpe;Hexa;F
MP:0009357	abnormal seizure response to inducing agent	http://www.informatics.jax.org/searches/Phat.cgi?id=MP:0	170	23	7.6439471	3.00891669	2.06E-06	0.00968268	11816;11878	Gfap;Apoe;Slc1a3;Slc6a11;St3gal4;Drd2;Plp1;Serpine2;Mbp;Pdyn;Panx1;Slc1a2;Hexb;Mt3;Bhlhe40;Cit;Ryr2;Arx;Htr1a;Lhx6;Npy;Elfn1;Kcnc2
MP:0001362	abnormal anxiety-related response	http://www.informatics.jax.org/searches/Phat.cgi?id=MP:0	308	33	13.8490336	2.38283775	2.96E-06	0.01389298	11539;11682	S100a1;Apoe;Gjb6;St3gal4;Drd2;S100a10;Atp1a2;Csf1r;Pdyn;Tlr3;Grm3;Htr1b;Adora1;Kcnip3;Pde4b;Chd6;Cpeb3;Crebrf;Tcf4;Gad2;Gria1;Als2;Arx;Ppp1r26;Htr1a;Npy;Lgals1;Trpc4;Elfn1;Rxfp3;Alk;Kcnc2;Crhbp
MP:0009745	abnormal behavioral response to xenobiotic	http://www.informatics.jax.org/searches/Phat.cgi?id=MP:0	266	30	11.960529	2.50825026	2.98E-06	0.0139689	11682;11829	Gstm1;Gfap;Scn4b;Slc1a3;Slc6a11;Drd2;S100a10;Serpine2;Pdyn;Panx1;Slc1a2;Mt3;Bhlhe40;Aqp4;Pdlim5;Cd81;Gpr88;Homer2;Cit;Ryr2;Gria1;Arx;Pnoc;Htr1a;Lhx6;Npy;Elfn1;Ppp1r9a;Alk;Kcnc2
MP:0002065	abnormal fear/anxiety-related behavior	http://www.informatics.jax.org/searches/Phat.cgi?id=MP:0	339	35	15.2429298	2.2961465	3.45E-06	0.01616671	11539;11682	S100a1;Apoe;Gm2a;Gjb6;St3gal4;Drd2;S100a10;Atp1a2;Csf1r;Pdyn;Tlr3;Grm3;Ptch1;Htr1b;Adora1;Kcnip3;Pde4b;Chd6;Cpeb3;Crebrf;Tcf4;Gad2;Gria1;Als2;Arx;Ppp1r26;Htr1a;Npy;Lgals1;Trpc4;Elfn1;Rxfp3;Alk;Kcnc2;Crhbp
MP:0005404	abnormal axon morphology	http://www.informatics.jax.org/searches/Phat.cgi?id=MP:0	155	21	6.96948118	3.01313677	5.62E-06	0.02631756	74018;11816	Cldn11;Hepacam;Apoe;Mlc1;Pmp22;Plp1;Mbp;Tlr3;Fa2h;Cers2;Nfe2l2;Qk;Ephb1;Lgi4;Gpm6b;Klc1;Map1a;Trim2;Sptan1;Als2;Sptbn4
MP:0002910	abnormal excitatory postsynaptic currents	http://www.informatics.jax.org/searches/Phat.cgi?id=MP:0	97	16	4.36154629	3.66842375	5.86E-06	0.02747893	432530;1153	Slc1a3;Drd2;Slc17a6;Serpine2;Adora1;Igsf11;Gpr88;Dlg2;Inpp4a;Adcy1;Cacng8;Sptbn2;Trpc4;Elfn1;Unc13a;Ppp1r9a
MP:0000877	abnormal Purkinje cell morphology	http://www.informatics.jax.org/searches/Phat.cgi?id=MP:0	157	21	7.05940997	2.97475286	6.89E-06	0.03228305	234734;7401	Gpr37l1;Gm2a;Slc1a3;Prex2;Fa2h;Hexb;Npc2;Qk;Nr1d1;Inpp4a;Myo5a;Aars;Herc1;Map1a;Bcl2l2;Ank1;Myh10;Trim2;Bicd2;Als2;Sptbn2
MP:0000875	abnormal cerebellar Purkinje cell layer	http://www.informatics.jax.org/searches/Phat.cgi?id=MP:0	172	22	7.73387589	2.84462801	8.71E-06	0.04076966	234734;7401	Gpr37l1;Gm2a;Slc1a3;Prex2;Fa2h;Hexb;Npc2;Qk;Nr1d1;Inpp4a;Myo5a;Aars;Herc1;Cit;Map1a;Bcl2l2;Ank1;Myh10;Trim2;Bicd2;Als2;Sptbn2
MP:0002906	increased susceptibility to pharmacologically induced seizures	http://www.informatics.jax.org/searches/Phat.cgi?id=MP:0	100	16	4.49643947	3.55837104	8.79E-06	0.04114894	11878;20893	Gfap;Slc1a3;Drd2;Serpine2;Pdyn;Slc1a2;Mt3;Bhlhe40;Cit;Ryr2;Arx;Htr1a;Lhx6;Npy;Elfn1;Kcnc2
MP:0000841	abnormal hindbrain morphology	http://www.informatics.jax.org/searches/Phat.cgi?id=MP:0	401	38	18.0307223	2.10751402	1.00E-05	0.04687502	234734;7401	Gpr37l1;Gm2a;Mlc1;Slc1a3;Olig1;Nfia;Zic1;Atp1b2;Plp1;Serpine2;Mbp;Pdyn;Prex2;Fa2h;Hexb;Cers2;Ptch1;Fgfr2;Npc2;Qk;Phgdh;Cntfr;Nr1d1;Inpp4a;Myo5a;Aars;Herc1;Cit;Map1a;Bcl2l2;Ank1;Myh10;Trim2;Bicd2;Als2;Sptbn2;Sptbn4;Dnah5
MP:0012349	increased susceptibility to induction of seizure by inducing agent	http://www.informatics.jax.org/searches/Phat.cgi?id=MP:0	101	16	4.54140387	3.52313964	1.00E-05	0.04689142	11878;20893	Gfap;Slc1a3;Drd2;Serpine2;Pdyn;Slc1a2;Mt3;Bhlhe40;Cit;Ryr2;Arx;Htr1a;Lhx6;Npy;Elfn1;Kcnc2

2;Tlr3;Fa2h;Hexb;Trp53bp2;Grm3;Mt3;Bhlhe40;Litaf;Gjc3;Egr1;Olig2;Nfe2l2;Aqp4;Fgfr2;Qk;Lgi4;Pdlim5;Cd81;Arpp21;Pdgfra;Adora1;Pbx3;Cpe;Hexa;Ptn;Th;Ly6e;Ptprz1;Igsf11;Efna3;Nrn1;Slc6a9;Gpr88;Epas1;Tspan7;Slc7a11;Arrdc3;Maf1;Ssr2;Kcnip3;Pde4b;Dlg2;Inpp4a;Myo5a;Klc1;Cpeb3;Arfgef2;Homer2;Fez1;Cit;Adcy1;Mapk8ip3;Cited2;Sst;Birc6;Tcf4;Cacng8;Cux2;Ryr2;Gad2;Gria1;Myh10;Trim2;Bicd2;Als2;Arx;St8sia1;Zfp536;Cntnap4;Grip1;Grik3;Rab27a;Htr1a;Lhx6;Npy;Sptbn2;Sp9;Trpc4;Gad1;Elfn1;Prokr2;Unc13a;Ppp1r9a;Prlr;Dpysl4;Sptbn4;Otud7a;Alk;Mafb;Kcnc2;Calb1

2;Qk;Ephb1;Phgdh;Cd81;Pdgfra;Hexa;Sall1;Lhx2;Efna3;Tspan7;Twsg1;Psat1;Cntfr;Id4;Ssr2;Nr1d1;Pde4b;Inpp4a;Myo5a;Klc1;Tardbp;Aars;Herc1;Cpeb3;Arfgef2;Cit;Adcy1;Mapk8ip3;Cited2;Exoc8;Map1a;Tcf4;Cacng8;Cux2;Dync2h1;Bcl2l2;Ank1;Myh10;Trim2;Sptan1;Bicd2;Als2;Arx;Grip1;Satb1;Lhx6;Sptbn2;Sp9;Sowahb;Lgals1;Gad1;Prokr2;Dpysl4;Sptbn4;Sptbn1;Dnah5;Alk;Mafb

;Sst;Tcf4;Cacng8;Gad2;Gria1;Zfp536;Cntnap4;Grik3;Rab27a;Htr1a;Lhx6;Sptbn2;Trpc4;Gad1;Elfn1;Unc13a;Ppp1r9a;Dpysl4;Otud7a

;Tspan7;Cntfr;Id4;Pde6d;Vegfb;Nr1d1;Pde4b;Inpp4a;Myo5a;Klc1;Tardbp;Aars;Herc1;Cpeb3;Arfgef2;Mapk8ip3;Map1a;Cacng8;Cux2;Kif5a;Bcl2l2;Gria1;Ank1;Myh10;Trim2;Sptan1;Bicd2;Als2;Arx;Cntnap4;Col25a1;Lhx6;Sptbn2;Sp9;Lgals1;Elfn1;Prokr2;Unc13a;Dpysl4;Sptbn4;Otud7a

;Twsg1;Arrdc3;Tmub1;Id4;Kcnip3;Nr1d1;Pde4b;Abhd12;Inpp4a;Myo5a;Camta1;Tardbp;Aars;Herc1;Cpeb3;Crebrf;Fez1;Cit;Map1a;Hook3;Tcf4;Nos1ap;Gad2;Gria1;Myh10;Trim2;Bicd2;Als2;Arx;Rab27a;Satb1;Ppp1r26;Htr1a;Npy;Sptbn2;Elfn1;Prokr2;Sptbn4;Rxfp3;Dnah5;Alk;Mafb;Calb1

Fgfr3;Th;Slc6a9;Gpr88;Twsg1;Arrdc3;Tmub1;Id4;Kcnip3;Nr1d1;Pde4b;Abhd12;Inpp4a;Myo5a;Camta1;Tardbp;Aars;Herc1;Cpeb3;Crebrf;Fez1;Cit;Map1a;Hook3;Tcf4;Nos1ap;Gad2;Gria1;Myh10;Trim2;Bicd2;Als2;Arx;Rab27a;Satb1;Ppp1r26;Htr1a;Npy;Sptbn2;Elfn1;Prokr2;Sptbn4;Rnf144b;Rxfp3;Dnah5;Alk;Mafb;Calb1
