# Supplementary material for: Stress and high fat diet reconfigure the active translatome of CeA-NPY neurons
Source: Mol Metab. 2025 Jun 4;98:102176. doi: 10.1016/j.molmet.2025.102176 (PMC12214123; doi:10.1016/j.molmet.2025.102176)
Supplement: Supplementary Figure 1 — (A) Bodyweight curve of female WT mice on a chow or a chow combined with stress paradigm for 25 days. Data means ± SEM, 7–9 mice per group. (B) Absolute Weight of dissected white adipose depots between male mice on a 14-day Chow or ChowS paradigm: inguinal fat (i), epididymal (e), mesenteric (m), perirenal fat (r) and summed total fat mass and BAT. Data are means ± SEM, 12 mice per group. (C) Fat mass weight of white adipose depots normalized to body weight of male mice on a 14-days Chow or ChowS paradigm: inguinal fat (i), epididymal (e), mesenteric (m), and perirenal fat (r), BAT and sum of total fat mass (Total). Data are means ± SEM, 12 mice per group. (D) Serum insulin levels of male mice on a 14-day Chow and ChowS paradigm at the end of treatment. Data are means ± SEM, 8 mice per group. (E) Quantification of Fos expression in the CeA of male mice 14 days on either a Chow or a ChowS paradigm. Data are means ± SEM, n = 5 mice per group. (F) Quantification of GFP positive neurons in the CeA in Npy GFP reporter mice under a 14day Chow or ChowS paradigm. Data are means ± SEM, 5 mice per group. [file mmc5.pptx]

## Slide 1
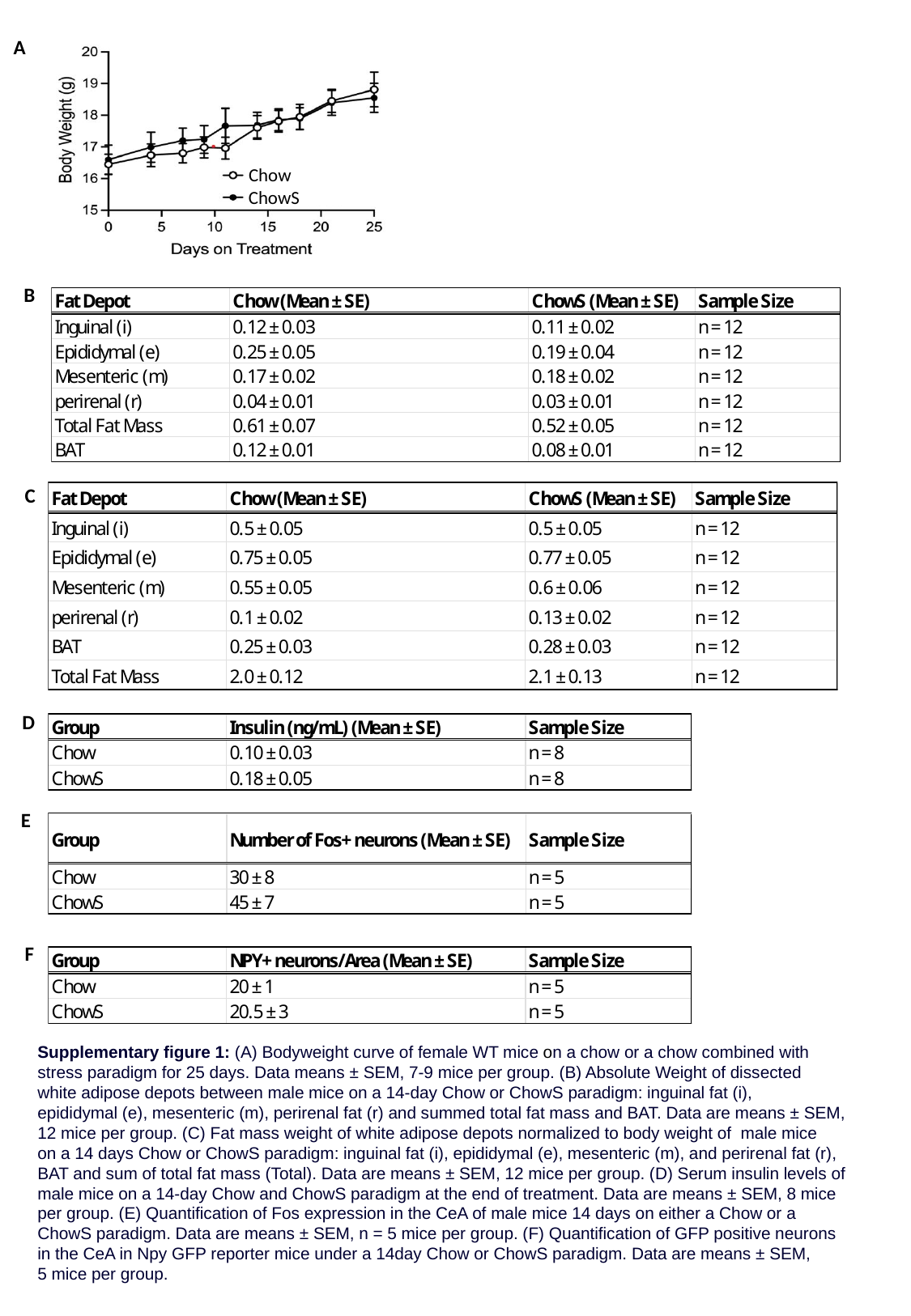

Chow
ChowS
A
B
C
D
E
F
Supplementary figure 1: (A) Bodyweight curve of female WT mice on a chow or a chow combined with
stress paradigm for 25 days. Data means ± SEM, 7-9 mice per group. (B) Absolute Weight of dissected
white adipose depots between male mice on a 14-day Chow or ChowS paradigm: inguinal fat (i),
epididymal (e), mesenteric (m), perirenal fat (r) and summed total fat mass and BAT. Data are means ± SEM,
12 mice per group. (C) Fat mass weight of white adipose depots normalized to body weight of male mice
on a 14 days Chow or ChowS paradigm: inguinal fat (i), epididymal (e), mesenteric (m), and perirenal fat (r),
BAT and sum of total fat mass (Total). Data are means ± SEM, 12 mice per group. (D) Serum insulin levels of
male mice on a 14-day Chow and ChowS paradigm at the end of treatment. Data are means ± SEM, 8 mice
per group. (E) Quantification of Fos expression in the CeA of male mice 14 days on either a Chow or a
ChowS paradigm. Data are means ± SEM, n = 5 mice per group. (F) Quantification of GFP positive neurons
in the CeA in Npy GFP reporter mice under a 14day Chow or ChowS paradigm. Data are means ± SEM,
5 mice per group.
